# Using weighted gene co-expression network analysis to identify key modules and hub genes in tongue squamous cell carcinoma

**DOI:** 10.1097/MD.0000000000017100

**Published:** 2019-09-13

**Authors:** Ke Yin, Ying Zhang, Suxin Zhang, Yang Bao, Jie Guo, Guanhua Zhang, Tianke Li

**Affiliations:** a Department of Stomatology, The Fourth Hospital of Hebei Medical University, Shijiazhuang; b Department of Stomatology, Xingtai People's Hospital of Hebei Medical University, Xingtai; c Department of Stomatology, The Third Hospital of Shijiazhuang City, Shijiazhuang, China.

**Keywords:** gene co-expression, GSEA, hub genes, tongue squamous cell carcinoma, WGCNA

## Abstract

**Background:**

Tongue squamous cell carcinoma (TSCC) is one of the most common malignant tumors in head and neck, but its molecular mechanism is not clear.

**Methods:**

Weighted gene co-expression network analysis (WGCNA) combining with gene differential expression analysis, survival analysis to screen key modules and hub genes related to the progress of TSCC. Gene Set Enrichment Analysis (GSEA) was used to identify biological pathways that might be involved.

**Results:**

Weighted gene co-expression network was constructed based on dataset GSE34105. The blue module and turquoise module most related to the progress of TSCC were identified by the network. Gene Ontology (GO) enrichment analysis showed that 2 key modules were significantly enriched in apoptosis and immunity related biological processes and pathway. Network topology analysis, gene difference analysis and survival analysis were used to screen 9 hub genes (*NOC2L, AIMP2, ANXA2, DIABLO, H2AFZ, MANBAL, PRDX6, SNX14, TIMM23*). The expression of hub genes was significantly correlated with the prognosis of TSCC. GSEA showed that the high expression group of hub genes was mainly enriched in olfactory transduction, neuroactive ligand receptor interaction, nicotinate and nicotinamide metabolism, and the low expression group was mainly enriched in base excision repair, cysteine and methionine metabolism, oxidative phosphorylation.

**Conclusion:**

Two key modules and 9 hub genes screened by WGCNA were closely related to the occurrence and prognosis of TSCC. Hub genes can be used as biomarkers and potential therapeutic targets for the accurate diagnosis and treatment of TSCC in the future.

## Abstract 2

Supplemental Digital Content is available in the text

## 1 Introduction

Tongue cancer is a common malignant tumor in oral and maxillofacial region, most of which are tongue squamous cell carcinoma (TSCC). It is one of the main subtypes of head and neck cancer (HNSC).^[1]^ TSCC patients are prone to early lymph node metastasis due to frequent tongue movement and abundant blood transport, and the prognosis is poor. However, the molecular pathogenesis of TSCC is not clear at present.^[2]^ According to the literature, the 5-year survival rate has not been significantly improved in recent years, which maintained unsatisfactory.^[3]^ Therefore, the early diagnosis and treatment of TSCC patients is still a difficult problem.

With the continuous discovery of molecular biology studies, a number of TSCC-related oncogenes, such as Bcl-2, c-Myc and EGFR have been found.^[4,5]^ In recent years, the research has shown that tumor is a multi-factor and multi-stage complex disease, which may be the result of the joint action of multiple genes. Systematic analysis of multiple genes can greatly help us understand the mechanisms of disease and develop effective diagnosis and treatment strategies.^[6]^ Weighted gene co-expression network analysis (WGCNA) is a widely used system biology method. It is based on the correlation between the changes of gene expression value, connecting with the complex changes of clinical phenotype, and constructing the corresponding function network.^[7]^ Compared with gene differential expression analysis, it can better analyze the changes of the whole biological process, making it possible to identify hundreds of pathogenic genes and treatment targets at the same time.^[8]^ WGCNA technology has been successfully used in the study of many biological problems, and some important discoveries have been made.^[9,10]^ Giulietti et al^[11]^ constructed lncRNA co-expression network, revealing Linc1133 and Linc0205 as new biomarkers of pancreatic ductal adenocarcinoma. Plasie et al^[12]^ identified the related pathways of family complex hyperlipidemia and genesis gene USF1 and FADS by combining WGCNA with genetic marker data. All in all, the WGCNA analysis method provides a solution for a comprehensive analysis of complex diseases.

**Figure 1:**
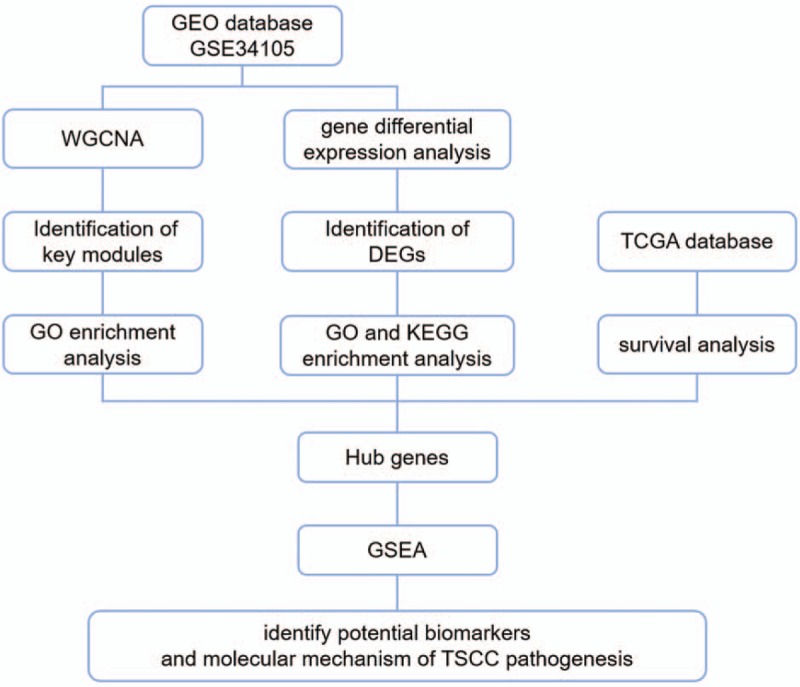
Whole framework of this study.

Therefore, in this study, we downloaded GSE34105 microarray data from Gene Expression Omnibus (GEO) database to build WGCNA networks. We identify key modules that are closely related to the occurrence of TSCC, conduct GO and Kyoto Encyclopedia of Genes and Genomes (KEGG) enrichment analysis of the key modules, identifying the biological functions and pathway in which they are involved. In these key modules, we screened and obtained hub genes based on network topology and gene difference analysis, and look for potential biomarkers for the diagnosis and treatment of TSCC (Fig. 1). These findings may help us to understand the molecular mechanism of TSCC pathogenesis.

## 2 Methods

### 2.1 Data resources

The GSE34105 data set for TSCC was obtained from the GEO database (https://www.ncbi.nlm.nih.gov/geo/). GSE34105 consists of 62 TSCC samples and 16 normal samples. Illumina HumanHT-12 WG-DASL v4.0 R2 expression beadchip platform annotation information is used to match probes and gene names, and relevant clinical information was used for WGCNA analysis. The study was approved by the local ethical review committee “Etik Provnings Namnden” (EPN), Permit number 08–003 M. For fresh frozen samples written consent was obtained from all patients. For the archival formalin fixed paraffin embedded samples written consent is available for samples from 2003 and onwards because of the establishment of the Swedish Act on Biobanks (SF 2002:297). The use of formalin fixed paraffin embedded samples dated before 2003 was approved by the local ethical review committee (EPN) according to their standard procedure. All samples came from Biobank VL (Vasterbottens Lan).

### 2.2 Construction of weighted gene co-expression network

The weighted gene co-expression network was constructed by WGCNA package in R. Gene ranked by SD from large to small (including normal, and TSCC samples), we chose the top 25% genes for WGCNA, calculated the power value by pickSoftThreshold function, and plotted the gene tree to present the results of hierarchical clustering. In the tree diagram, each vertical line corresponds to a gene, and each branch corresponds to a module. We used the dynamic tree cutting algorithm to segment the network module. In order to test the stability of each identified module, the GSE34105 expression data is randomly divided into training sets and test sets, and the stability of the module is tested using the module Preservation function in the WGCNA package.

### 2.3 Identification of clinical significance modules

The correlation between modules and clinical features was evaluated by Pearson correlation coefficient. We calculated the correlation between module eigengenes (MEs) and clinical features of the module to search for key modules. Gene significance (GS) was defined as the log10 transformation of the *P* value (GS = lgP) in the linear regression between gene expression and clinical information. Module significance was the average GS of all genes in a module.^[13]^ Generally, the module whose absolute value of module significance ranks first among all modules is considered to be the most relevant module to clinical traits and is considered to be the key module.

### 2.4 Identification of differential expression genes

The limma package in R was used to screen the differential expression gene (DEGs) between TSCC and normal samples. | logFC | > 1, *P* < .05 was defined as DEGs. Volcanic map was drawn by R package ggplot2. In order to further understand the biological function of differential expression genes, we used the cluster profiler function in R to conduct the GO enrichment analysis and KEGG pathway enrichment analysis of DEGs. *P* < .05 was considered to be a significant result of enrichment analysis.

### 2.5 Identification of hub genes in key modules

Genes that are highly connected to other genes in a module were considered to have important functions. We used Cytoscape 3.7.0 to draw the key module network and screened out the top 150 genes of degree in the module network, and intersected these genes with DEGs. We used the online tool jvenn to draw Venn diagrams (http://jvenn.toulouse.inra.fr/app/example.html).^[14]^ GEPIA is a website for analyzing RNA expression data from tumors and normal samples in TCGA databases(http://gepia.cancer-pku.cn/).^[15]^ We used GEPIA to conduct survival analysis of genes in the intersection region, and screened the significant results (logrank *P* < .05). These genes were considered to be hub genes.

### 2.6 Gene set enrichment analysis of hub genes

Gene Set Enrichment Analysis (GSEA) is a computational method that assesses whether an a priori defined set of genes shows statistically significant, concordant differences between 2 biological states. GSEA 3.0 software was used to enrich analyze the metabolic pathways associated with high or low expression of hub genes. The expression values of 9 hub genes in GSE34105 dataset were used as phenotypes. The c2.cp.kegg.v6.1.symbols.gmt dataset in the msigdb database of GSEA website was selected as the reference gene set. The number of random combinations is set to 1000.

## 3 Results

### 3.1 Weighted gene co-expression network construction and gene module recognition

**Figure 2:**
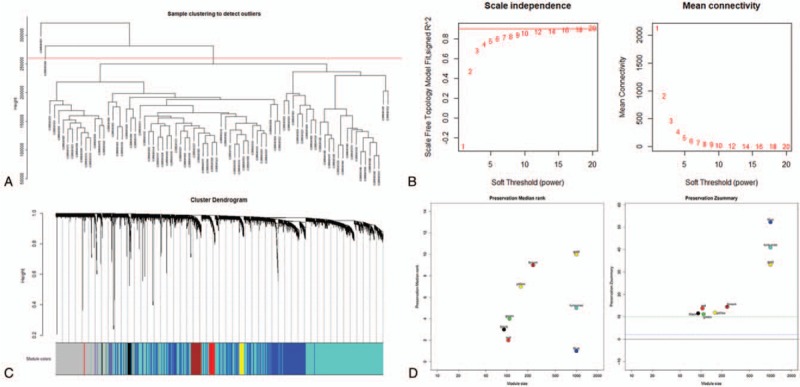
(A) The clustering was based on the expression data of GSE34105, which contained 62 TSCC, and 12 normal samples. The top 25% genes with the highest SD values were used for the analysis by WGCNA. (B) Analysis of the scale-free fit index for various soft-thresholding powers (β), 12 was the most fit power value. (C) The cluster dendrogram of genes in GSE34105. Each branch in the figure represents one gene, and every color below represents one co-expression module. (D)The medianrank and Zsummary statistics of module preservation. Module preservation was evaluated by medianrank and Zsummary statistics, which correlated to connectivity and density of networks. if Zsummary >10, there is a strong evidence that the module is preserved.

GSE34105 was downloaded from the GEO database. The Illumina HumanHT-12 WG-DASL v4.0 R2 expression beadchip platform annotation information was used to match probes and gene names (Table S1). Eventually, we obtained a total of 78 samples, including 16 normal samples and 62 TSCC samples, as well as their relevant clinical information (Table S2). We used the top 25% genes of the highest SD values in the GSE34105 microarray data for cluster analysis by WGCNA package. To ensure the reliability of the network structure, 2 outlier samples were deleted (Fig. 2A). The rest of the data was used to construct weighted co-expression networks. We analyzed the power values from 1 to 20. When the power value was set at 12, the connectivity between genes in the network satisfies the scale-free network distribution (Fig. 2B) and generates eight modules (Fig. 2C). The genes that could not be included in any modules were put into the gray module. To test the stability of each identified module, we randomly divided the dataset GSE34105 into the training set and the test set. Then, we used the module Preservation function (nPermutations = 200) in the WGCNA package to calculate module stability (Fig. 2D). As shown in the figure, the zSummy values of all modules are higher than 10, indicating that the modules have good stability.

### 3.2 Correlation between modules and identification of key modules

**Figure 3:**
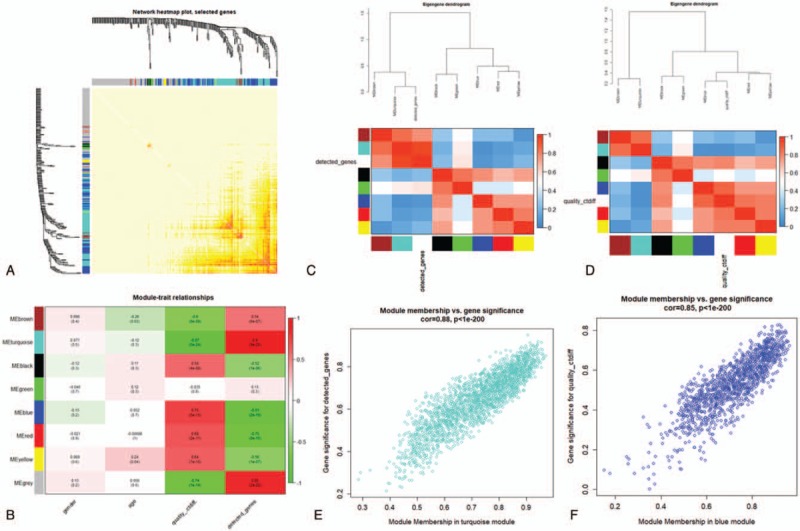
(A) Interaction relationship analysis of co-expression genes. Different colors of horizontal axis and vertical axis represent different modules. The brightness of yellow in the middle represents the degree of connectivity of different modules. There was no significant difference in interactions among different modules, indicating a high-scale independence degree among these modules. (B) Heatmap of the correlation between module eigengenes and the disease status of TSCC. Correlation coefficient along with *P* value in parenthesis underneath. Color-coded according to correlation coefficient (legend at right). (C) Hierarchical clustering of module genes that summarize the modules yielded in the clustering analysis. Blue module is the most closely related to quality ctdiff. (D) Turquoise module is the most closely related to detected genes. (E) Scatter plot of module eigengenes in the turquoise module. (F) Scatter plot of module eigengenes in the blue module.

After that, we analyzed the interaction between the eight modules, drew the network heat map, and demonstrated the relative independence of each module (Fig. 3A). Besides, we calculated the correlation between the modules and the clinical features, as shown in Figure 3B. It can be seen that detected genes and quality ctdiff are significantly correlated with most of the modules in the four clinical features. Compared with other modules, the black, blue, red, and yellow modules are positively correlated with quality ctdiff, while brown, turquoise modules are negatively correlated with quality ctdiff. Brown, turquoise modules are positively correlated with detected genes, while black, blue, red, yellow modules are negatively correlated with detected genes. In addition, we calculated the MEs and clustered them according to their correlation with detected genes. The turquoise module was most closely related to detected genes (Fig. 3C). Clustering was carried out according to the correlation between MEs and quality ctdiff, in which blue module is the most closely related to quality ctdiff (Fig. 3D). The heat map based on adjacencies also proves similar results. Therefore, we determined that the turquoise module and the blue module are the most relevant modules for TSCC. Figure 3E and F illustrates the relationship between the blue module, the turquoise module, and the Genetic significance, respectively.

**Figure 4:**
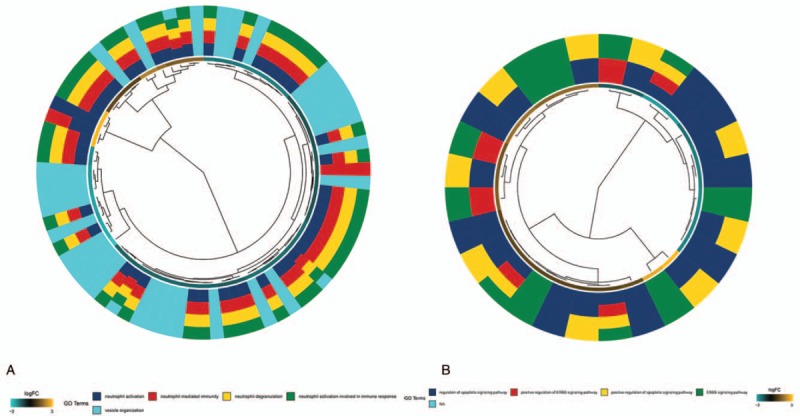
(A) Circos plot to indicate the relationship between genes of turquoise module and GO terms. (B) Circos plot to indicate the relationship between genes of blue module and GO terms.

The GO enrichment analysis of the nodes in the 2 modules was carried out by using R packet cluster Profiler. *P* < .05 is defined as a meaningful enrichment analysis (Fig. 4Aand B). The results of GO showed that most of the enriched biological processes (BP) in blue and turquoise modules are cancer-related. The top 3 of blue modules are regulation of apoptotic signaling pathway, positive regulation of ERBB signaling pathway, positive regulation of apoptotic signaling pathway. The top 3 of turquoise modules are neutrophil activation, neutrophil mediated immunity, neutrophil degranulation.

### 3.3 Identification of DEGs

**Figure 5:**
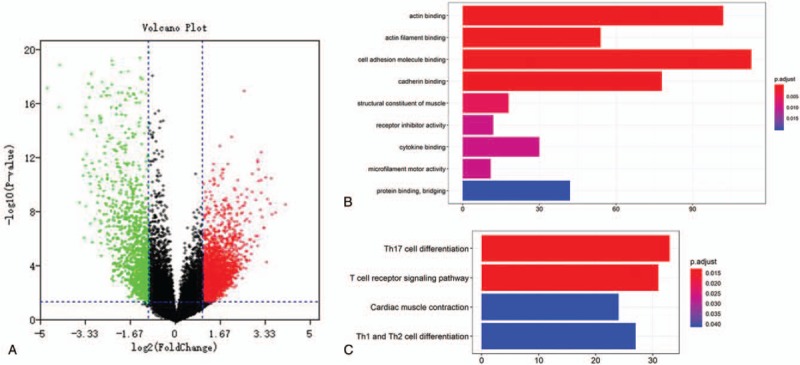
A volcanic map of gene expression between TSCC tissues and normal tissues identified in GSE34105, with red representing up-regulated genes and green representing down-regulated genes. (B) GO functional enrichment analyses for DEGs. The x-axis shows the number of genes and the y-axis shows the GO terms. The adjust *P* value of each term is colored according to the legend. (C) KEGG pathway enrichment analyses for DEGs. The x-axis shows the number of genes and the y-axis shows the KEGG pathway terms. The adjust *P* value of each term is colored according to the legend.

The microarray data of GSE34105 normal samples and TSCC samples were compared and analyzed by limma package. Using *P* < .05 and | logFC | > 1 as truncation values, a total of 1587 DEGs were obtained (Table S3). DEGs was shown in the volcanic map in Table 3 of Figure 5A. GO enrichment analysis of DEGs was carried out by using R-package cluster Profiler. *P* < .05 was defined as the result of meaningful enrichment analysis. The results of GO analysis show that the top 3 BP were actin binding, actin filament binding, cell adhesion molecule binding (Fig. 5B, Table S4). KEGG pathway enrichment analysis showed that the top 3 entries were Th17 cell differentiation, T-cell receptor signaling pathway, Cardiac muscle contraction (Fig. 5C, Table S5).

### 3.4 Identification of hub genes in the turquoise module and blue module

**Figure 6:**
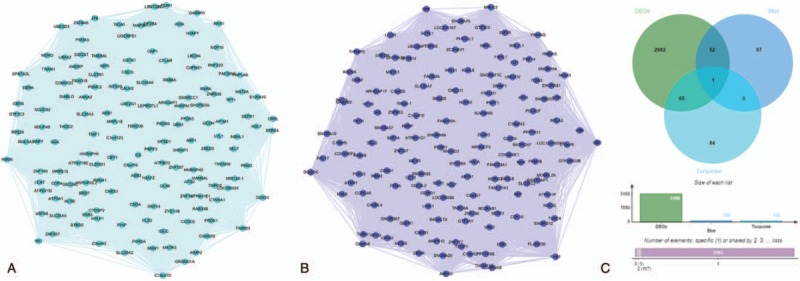
(A) The top 150 genes in the turquoise module. (B) The top 150 genes in the blue module. nodes represent genes. (C) Identification of genes between DEGs and the key modules by overlapping them.

**Figure 7:**
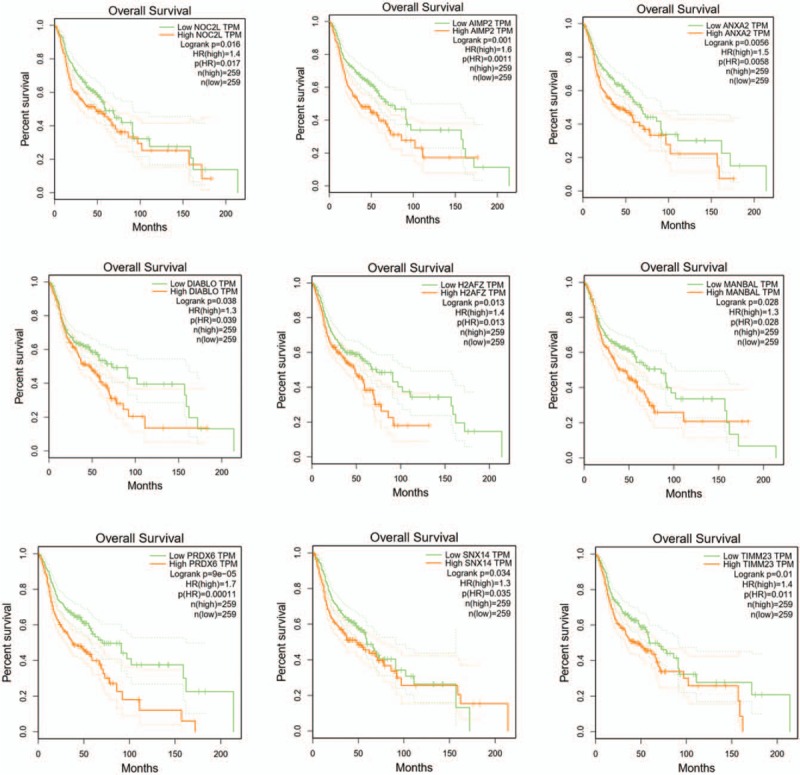
Survival analysis of hub genes. Notes: 9 hub genes in blue module and turquoise module with significant results of survival analysis (*P* < .05 was regarded as significant). They were *NOC2L, AIMP2, ANXA2, DIABLO, H2AFZ, MANBAL, PRDX6, SNX14,* and *TIMM23*, respectively.

The turquoise module and the blue module were visualized in Cytoscape. Then, we calculated the topology parameters of all nodes in the 2 module networks, screened out the top 150 nodes by sorting node degree for further analysis, and drew the network diagram as shown in Figure 6Aand B (Table S6, Table S7). These nodes were intersected with DEGs and 118 nodes were obtained for subsequent analysis as candidate nodes (Fig. 6C). We used GEPIA to analyze the survival rate. *P* < .05 was considered to be statistically significant. The 118 genes were further analyzed to evaluate their effect on the survival rate of TSCC. A total of 9 genes were significantly correlated with prognosis of patients (*P* < .05). They are NOC2L in the blue module and *AIMP2, ANXA2, DIABLO, H2AFZ, MANBAL, PRDX6, SNX14, TIMM23* in the turquoise module (Fig. 7). They were considered to be hub genes. With the increase of expression values of hub genes, the survival time was significantly shortened. These results suggest that 9 hub genes are adverse prognostic factors, and high expression is significantly associated with shortened survival.

### 3.5 GSEA analysis of hub genes

**Figure 8:**
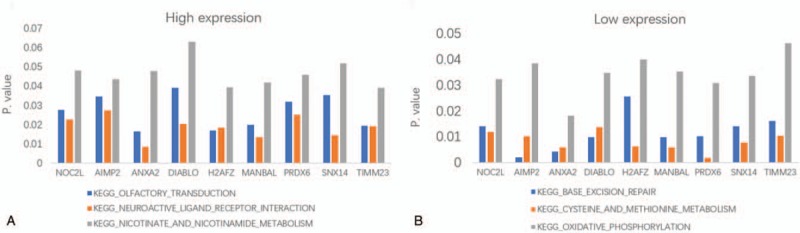
(A) Gene set enrichment analysis of high expression group. (B) Gene set enrichment analysis of low expression group.

In order to understand the possible biological functions of hub genes, GSEA analysis was carried out using the dataset GSE34105. The results showed that 3 genomes were enriched in all the high expression groups of hub genes, including olfactory transduction, neuroactive ligand receptor interaction, nicotinate and nicotinamide metabolism. There are 3 genomes enriched in all the low expression groups of hub genes, including base excision repair, cysteine and methionine metabolism, oxidative phosphorylation. (Fig. 8)

## 4 Discussion

TSCC is one of the most common malignant tumors in the head and neck. It is a complex molecular network disease. In order to further understand the molecular mechanism of the pathogenesis and progression of TSCC, we used WGCNA analysis and differential expression gene analysis to analyze the relevant risk modules in TSCC. First of all, we analyzed the modules of GSE34105 data set using WGCNA analysis method, and obtained a total of 8 modules, among which the turquoise module is the most relevant to the risk of cancer occurrence (cor = 0.88). The results of turquoise module enrichment analysis showed that neutrophil activation, neutrophil mediated immunity, neutrophil degranulation was closely related to the occurrence of TSCC. Associating with gene expression data and clinical features, we found that the blue module and quality ctdiff showed a strong correlation, the turquoise module and detected genes showed a strong correlation. Combined with network topology and gene difference analysis, 9 potential hub genes were obtained, including *NOC2L, AIMP2, ANXA2, DIABLO, H2AFZ, MANBAL, PRDX6, SNX14,* and *TIMM23*. Survival analysis showed that 9 hub genes are adverse prognostic factors, and high expression is significantly associated with shortened survival. GSEA analysis showed that 3 genomes were enriched in all the high expression groups of hub genes, including olfactory transduction, neuroactive ligand receptor interaction, nicotinate and nicotinamide metabolism. These findings may help us to understand the molecular mechanism of TSCC pathogenesis and identify potential biomarkers for the diagnosis and treatment of TSCC.

There are many genome-wide gene expressions of TSCC datasets available for researchers to carry out multiple analyses. However, there are some difficulties in these analyses. For example, data from different platforms cannot be compared together, but WGCNA methods avoid this limitation by focusing on a group of genes rather than on individual genes. Besides, the cutoff criterion is not necessary for WGCNA, and hence, important information that is omitted when using other methods can be retrieved. Until now, WGCNA has been applied in many cancers such as colon cancer,^[16]^ ovarian cancer,^[17]^ lung adenocarcinoma,^[18]^ etc. Although there were some reports on TSCC based on expression profiles.^[19]^ There were no studies in which the expression profile of TSCC was analyzed by WGCNA. In this study, we used WGCNA to analyze TSCC from a new perspective. At present, there are no reports in literature about the relationship between 9 hub genes identified in our research and TSCC. They may be potential therapeutic targets for TSCC in the future.

It is worth noting that in the differential expression genes, we obtained a number of genes encoding F-box family proteins, including *FBXL5, FBXO21, FBXO3,* and *FBXW7* etc. F-box family proteins can be divided into 3 subgroups according to their domains:

(1) The FBXW subfamily,

(2) The FBXW subfamily, and

(3) the FBXO subfamily.

At present, a large number of literatures have confirmed that F-box family protein is closely related to the occurrence and development of tumors.^[20,21]^ F-box protein has been studied in tumor-related fields, such as FBXW7 and FBXL1, which are considered to be star molecules in the process of tumorigenesis and development.^[22,23]^ FBXL5 can be abnormally expressed in a variety of cancer cell lines, but also can degrade and identify a variety of cancer proteins, such as p150Glued and HSSB1. Therefore, it mediates the participation in cell cycle, DNA damage repair, signal transduction, transcriptional regulation and other processes, affecting and participating in the occurrence and development of tumor.^[24]^ A large number of studies at home and abroad have found that FBXL5 is abnormally expressed in cervical cancer, gastric cancer, colon cancer and other cancer cell lines or tissues.^[25–27]^ At present, there are few reports on the correlation between F-box family proteins and TSCC, and the molecular mechanism of their participation in the occurrence and development of TSCC requires further exploration.

Compared with the traditional gene difference analysis, the gene module divided by WGCNA has obvious biological significance. Combined clinical information analysis, we can identify important modules and potential key genes related to the pathogenesis of the disease. This method can be used as a methodological tool to explore the molecular mechanism of TSCC and discover potential new targets for drug therapy. In order to further illustrate the relationship between the key genes or gene modules we screened and TSCC, we need to further verify the potential key genes in vivo and in vitro experiments, such as RT-PCR, western blot and animal experiments in the follow-up research.

However, our research has certain limitations. The current cost of gene sequencing is still insufficient to obtain large-scale multi-sample gene expression data, combined with social ethics and other factors, the number of cancer samples that can be obtained is relatively limited. With the development of bioinformatics, increasing the number of samples helps to improve the accuracy and reliability of our models. And, our research also requires validation of biological experiments. In the following studies, we will explore the biological functions of hub genes and analyze their roles in the development of TSCC. The correlation between the protein expression level of hub genes and the clinical features of the patients will be analyzed. The accuracy of hub genes will be verified from the overall level, which provides an effective criterion for the prognosis and diagnosis of TSCC.

## 5 Conclusion

In this study, we obtained 9 hub genes from 2 key modules based on DEGs analysis and WGCNA. Their high expression is all related to poor prognosis. GSEA analysis showed that 3 genomes were enriched in all the high expression groups of hub genes, including olfactory transduction, neuroactive ligand receptor interaction, nicotinate and nicotinamide metabolism. These genes and mechanisms may help early diagnosis and treatment of TSCC.

## Author contributions

**Data curation:** Ke Yin, Ying Zhang, Suxin Zhang, Yang Bao, Jie Guo, Guanhua Zhang.

**Formal analysis:** Ke Yin, Ying Zhang, Suxin Zhang, Yang Bao, Jie Guo.

**Funding acquisition:** Tianke Li.

**Investigation:** Jie Guo.

**Methodology:** Ying Zhang, Yang Bao, Tianke Li.

**Project administration:** Tianke Li.

**Resources:** Tianke Li.

**Software:** Suxin Zhang, Tianke Li.

**Supervision:** Tianke Li.

**Validation:** Tianke Li.

**Visualization:** Jie Guo, Guanhua Zhang, Tianke Li.

**Writing – original draft:** Ke Yin.

**Writing – review & editing:** Ke Yin.

## Supplementary Material

Supplemental Digital Content

## Supplementary Material

Supplemental Digital Content
