# Supplementary material for: Using weighted gene co-expression network analysis to identify key modules and hub genes in tongue squamous cell carcinoma
Source: Medicine (Baltimore). 2019 Sep 13;98(37):e17100. doi: 10.1097/MD.0000000000017100 (PMC6750333; doi:10.1097/MD.0000000000017100)
Supplement: Supplemental Digital Content [file medi-98-e17100-s001.doc]

| Sample | gender | age | quality_(ctdiff) | detected_genes |
| --- | --- | --- | --- | --- |
| GSM841875 | 1 | 78 | 7.8 | 7727 |
| GSM841876 | 1 | 66 | 6.9 | 9181 |
| GSM841877 | 2 | 29 | 3.6 | 11850 |
| GSM841878 | 1 | 83 | 7.4 | 8651 |
| GSM841879 | 2 | 38 | 3 | 12358 |
| GSM841880 | 2 | 40 | 6.3 | 10414 |
| GSM841881 | 1 | 55 | 5 | 13036 |
| GSM841882 | 1 | 57 | 3.2 | 11496 |
| GSM841883 | 1 | 60 | 7.9 | 6976 |
| GSM841884 | 2 | 66 | 3 | 14258 |
| GSM841885 | 2 | 31 | 2.8 | 12038 |
| GSM841886 | 1 | 79 | 5 | 12251 |
| GSM841887 | 1 | 31 | 10.7 | 6931 |
| GSM841888 | 2 | 19 | 2.9 | 12631 |
| GSM841889 | 1 | 41 | 5.3 | 10744 |
| GSM841890 | 1 | 77 | 6.7 | 7566 |
| GSM841891 | 1 | 81 | 5.8 | 11649 |
| GSM841892 | 2 | 61 | 4.9 | 12020 |
| GSM841893 | 2 | 84 | 8 | 7269 |
| GSM841894 | 2 | 81 | 4.4 | 9297 |
| GSM841895 | 2 | 52 | 4.2 | 11978 |
| GSM841896 | 2 | 33 | 4.7 | 10511 |
| GSM841897 | 2 | 86 | 3.3 | 12790 |
| GSM841898 | 2 | 40 | 6 | 8330 |
| GSM841899 | 2 | 60 | 5.4 | 10699 |
| GSM841900 | 2 | 74 | 4.1 | 12380 |
| GSM841901 | 1 | 64 | 5.2 | 13094 |
| GSM841902 | 2 | 75 | 4 | 10064 |
| GSM841903 | 1 | 74 | 2.7 | 11888 |
| GSM841904 | 2 | 37 | 7.2 | 8824 |
| GSM841905 | 2 | 48 | 6.1 | 10811 |
| GSM841906 | 1 | 30 | 6.8 | 9891 |
| GSM841907 | 1 | 52 | 7.2 | 6537 |
| GSM841908 | 1 | 34 | 1.4 | 13819 |
| GSM841909 | 2 | 68 | 0.9 | 14131 |
| GSM841910 | 1 | 57 | 5.1 | 10931 |
| GSM841911 | 2 | 77 | 6.3 | 7759 |
| GSM841912 | 1 | 63 | 5.5 | 10287 |
| GSM841913 | 2 | 52 | 4.2 | 11574 |
| GSM841914 | 1 | 56 | 2.2 | 12638 |
| GSM841915 | 1 | 26 | 5.2 | 9776 |
| GSM841916 | 1 | 62 | 2.6 | 12895 |
| GSM841917 | 1 | 61 | 4.6 | 9715 |
| GSM841918 | 1 | 34 | 4.8 | 9420 |
| GSM841919 | 2 | 75 | 4.8 | 12083 |
| GSM841920 | 1 | 65 | 7 | 9552 |
| GSM841921 | 1 | 69 | 4.7 | 9115 |
| GSM841922 | 1 | 64 | 7.4 | 9139 |
| GSM841923 | 2 | 64 | 6.8 | 9306 |
| GSM841924 | 2 | 69 | 5.8 | 10551 |
| GSM841925 | 1 | 52 | 3.2 | 13406 |
| GSM841926 | 1 | 47 | 5.8 | 8579 |
| GSM841927 | 1 | 64 | 5.2 | 7786 |
| GSM841928 | 2 | 81 | 9.1 | 7368 |
| GSM841929 | 1 | 73 | 0.6 | 13341 |
| GSM841930 | 1 | 37 | 6 | 10927 |
| GSM841931 | 1 | 36 | 8.1 | 5747 |
| GSM841932 | 2 | 88 | 8.2 | 9261 |
| GSM841933 | 2 | 45 | 6.8 | 8537 |
| GSM841934 | 1 | 68 | 1.3 | 13454 |
| GSM841935 | 1 | 61 | 2.7 | 14011 |
| GSM841936 | 2 | 67 | 3 | 13353 |
| GSM841937 | 1 | 44 | 3.8 | 13267 |
| GSM841938 | 1 | 40 | 3.3 | 12016 |
| GSM841939 | 1 | 60 | 2 | 12691 |
| GSM841940 | 2 | 39 | 2.9 | 12469 |
| GSM841941 | 2 | 70 | 2.5 | 13502 |
| GSM841942 | 2 | 40 | 3.2 | 11713 |
| GSM841943 | 2 | 49 | 2.2 | 13391 |
| GSM841944 | 1 | 38 | 9.8 | 5842 |
| GSM841945 | 2 | 79 | 4.2 | 10409 |
| GSM841946 | 2 | 35 | 3.7 | 10978 |
| GSM841947 | 2 | 62 | 1.7 | 12238 |
| GSM841948 | 1 | 16 | 1.6 | 12012 |
| GSM841949 | 1 | 45 | 1.3 | 13057 |
| GSM841950 | 2 | 72 | 3.3 | 11056 |
| GSM841951 | 1 | 41 | 3.3 | 11153 |
| GSM841952 | 1 | 57 | 2.9 | 13585 |

| ID | logFC |
| --- | --- |
| 43525 | 1.488729 |
| 43526 | -1.62959 |
| 43527 | -1.5062 |
| 43531 | -1.02927 |
| A2ML1 | -1.29599 |
| AACS | -1.63025 |
| AADACL2 | -1.04011 |
| AASDH | -1.63443 |
| ABAT | -1.27867 |
| ABCB10 | -1.03792 |
| ABCB8 | 1.349522 |
| ABCF2 | -1.23069 |
| ABCF3 | -1.11107 |
| ABHD12 | -1.15574 |
| ABHD14B | -2.04546 |
| ABHD4 | -1.1258 |
| ABHD5 | -2.11191 |
| ABHD8 | 1.014603 |
| ABHD9 | -1.44014 |
| ABI2 | 1.217298 |
| ABI3 | 1.992312 |
| ABI3BP | -1.09885 |
| ABL2 | 1.418635 |
| ABLIM1 | -1.25247 |
| ABLIM2 | -1.08982 |
| ABO | -1.701 |
| ABP1 | 1.065444 |
| ABR | -1.04891 |
| ABTB1 | 1.129133 |
| ACAA2 | -1.99145 |
| ACADM | -1.15366 |
| ACAP1 | 1.433398 |
| ACBD6 | -1.33735 |
| ACCN2 | 1.197172 |
| ACOX2 | -1.11269 |
| ACP6 | -1.3463 |
| ACPP | -2.62101 |
| ACSL4 | 1.181805 |
| ACSL5 | 2.839405 |
| ACSS1 | -1.46252 |
| ACTA1 | 1.563302 |
| ACTR1B | -1.06135 |
| ACVR1B | -1.05631 |
| ACVR2A | -1.06073 |
| ADAM10 | 1.034114 |
| ADAM12 | 1.578413 |
| ADAM17 | 1.536281 |
| ADAM22 | 1.426963 |
| ADAM8 | 1.163734 |
| ADAMDEC1 | 1.443227 |
| ADAMTS18 | 1.229646 |
| ADAMTSL2 | 1.346747 |
| ADH7 | -1.53932 |
| ADIPOR2 | -2.43919 |
| ADNP | -1.08295 |
| ADORA1 | 1.043887 |
| ADRBK2 | 1.684133 |
| ADSS | -1.21285 |
| ADSSL1 | -1.39359 |
| AFTPH | -1.11394 |
| AGMAT | 1.392838 |
| AGPAT1 | 1.257141 |
| AGR3 | -1.56218 |
| AGRN | 1.787476 |
| AHCYL2 | -1.3972 |
| AHDC1 | -1.91509 |
| AHNAK2 | -1.58688 |
| AHSP | 1.748301 |
| AIF1 | 1.726155 |
| AIF1L | -1.72322 |
| AIM2 | 2.911194 |
| AIMP2 | -1.21122 |
| AIRE | 1.327888 |
| AKAP10 | 1.024018 |
| AKAP11 | -2.06542 |
| AKAP7 | -1.90052 |
| AKAP8 | -1.69693 |
| AKR1A1 | -2.13378 |
| AKR1B10 | -1.97006 |
| AKTIP | -1.44919 |
| ALDH18A1 | -1.6642 |
| ALDH1A2 | -1.60188 |
| ALDH1A3 | -1.1673 |
| ALDH1B1 | 1.056652 |
| ALDH3A2 | -1.49372 |
| ALDH5A1 | 1.197065 |
| ALDOA | -1.3175 |
| ALG14 | -1.29292 |
| ALG1L | 1.897994 |
| ALG6 | -1.10015 |
| ALG8 | -1.4743 |
| ALKBH3 | -1.64287 |
| ALOX12 | -3.14749 |
| ALOXE3 | -1.12488 |
| ALPK2 | 2.439007 |
| ALS2CL | -2.36075 |
| ALS2CR4 | -1.25116 |
| AMHR2 | 1.151819 |
| AMPD1 | 1.67919 |
| AMPH | -1.08695 |
| AMTN | 1.319258 |
| AMY1A | -1.18081 |
| AMZ2 | -1.16148 |
| ANAPC1 | -1.40654 |
| ANG | -1.01059 |
| ANGPT2 | 1.055753 |
| ANGPTL1 | -1.60612 |
| ANGPTL5 | -1.2481 |
| ANK2 | -1.82454 |
| ANK3 | -1.15563 |
| ANKFN1 | -2.50577 |
| ANKFY1 | -1.4691 |
| ANKRD1 | 2.564708 |
| ANKRD10 | -1.77801 |
| ANKRD11 | -1.16566 |
| ANKRD12 | -1.02361 |
| ANKRD13A | -1.13897 |
| ANKRD16 | -1.41894 |
| ANKRD20A1 | -1.75703 |
| ANKRD37 | -1.254 |
| ANKRD38 | 1.732916 |
| ANKRD46 | -2.07618 |
| ANKRD52 | -1.51344 |
| ANKRD6 | -2.34284 |
| ANO10 | -1.39326 |
| ANO3 | -1.36634 |
| ANO8 | -1.28752 |
| ANXA1 | -1.27553 |
| ANXA2 | -1.32949 |
| ANXA5 | -1.30092 |
| AOX1 | -1.32389 |
| AP2M1 | -1.1057 |
| AP3B1 | -1.99091 |
| AP3D1 | 1.233033 |
| AP3S1 | -1.02903 |
| AP4B1 | -1.62272 |
| APBA2BP | 1.072239 |
| APCDD1 | -1.07968 |
| APCDD1L | 1.255051 |
| APEH | -1.57085 |
| API5 | -1.46254 |
| APITD1 | 1.061502 |
| APOBEC3A | 2.330819 |
| APOBEC3F | 1.4245 |
| APOC1 | 2.771876 |
| APOC2 | 1.270899 |
| APOD | -2.72773 |
| APOL1 | 1.470042 |
| APOM | -1.5103 |
| APOOL | -1.09847 |
| APP | 1.714183 |
| APPL2 | -1.58147 |
| AQP10 | 2.355705 |
| AQR | -1.15894 |
| ARAP2 | -1.17199 |
| ARCN1 | -1.01528 |
| ARF4 | -1.07325 |
| ARF5 | -1.69497 |
| ARG1 | -1.22026 |
| ARHGAP10 | -1.55586 |
| ARHGAP19 | -1.15281 |
| ARHGAP25 | 1.158589 |
| ARHGAP9 | 1.609099 |
| ARHGEF1 | 1.412184 |
| ARHGEF10 | -1.26561 |
| ARHGEF4 | -2.00414 |
| ARHGEF5 | -1.25308 |
| ARID4B | 1.044014 |
| ARID5A | 1.229822 |
| ARL1 | -2.04422 |
| ARL14 | 2.275571 |
| ARL2BP | -1.12099 |
| ARL4D | -1.16874 |
| ARL5A | -1.53161 |
| ARL8B | -1.3414 |
| ARL9 | -1.11728 |
| ARNTL | 1.093677 |
| ARPC1B | 1.385445 |
| ARPP-21 | 2.071589 |
| ARRDC4 | 1.279548 |
| ARRDC5 | 2.801647 |
| ARSD | -2.08802 |
| ASB10 | 1.887593 |
| ASB13 | -1.80385 |
| ASB3 | 1.277766 |
| ASB5 | 1.563895 |
| ASCC3 | 1.124665 |
| ASCL2 | 1.026805 |
| ASIP | 1.642297 |
| ASNSD1 | -1.2072 |
| ASPG | -1.67476 |
| ASPRV1 | -2.07891 |
| ATF4 | 1.564356 |
| ATG4A | -1.30719 |
| ATG9B | -1.95208 |
| ATHL1 | 1.666734 |
| ATL1 | -1.422 |
| ATOH8 | -1.04372 |
| ATP13A4 | -1.20021 |
| ATP1A1 | 1.047715 |
| ATP1B1 | -1.85071 |
| ATP1B3 | 1.100628 |
| ATP2A1 | 1.337357 |
| ATP2C1 | -1.00306 |
| ATP5A1 | -1.29325 |
| ATP5D | -1.27938 |
| ATP5F1 | -1.50914 |
| ATP5L | -1.2635 |
| ATP5S | -1.10824 |
| ATP6V0A4 | -1.72546 |
| ATP6V0D2 | 1.473969 |
| ATP6V1B2 | -1.53277 |
| ATP6V1C2 | -3.13748 |
| ATP6V1D | -1.168 |
| ATP7B | -2.20353 |
| AUH | 1.172666 |
| AURKC | -1.53244 |
| AVL9 | -1.04856 |
| AXIN1 | -1.57021 |
| AXIN2 | -2.19719 |
| AZIN1 | -1.65526 |
| B3GALT4 | -1.01894 |
| B4GALNT1 | 1.083131 |
| BAG1 | -1.24562 |
| BAG5 | -1.1308 |
| BAIAP2 | -1.50079 |
| BARD1 | -1.70962 |
| BARX2 | -1.66027 |
| BAT3 | -1.02853 |
| BATF | 2.399816 |
| BAZ1A | -1.05859 |
| BBOX1 | -1.38113 |
| BBS1 | -1.04225 |
| BBS10 | 1.179779 |
| BBS7 | -1.57662 |
| BCAP29 | -1.34703 |
| BCAS4 | 1.131374 |
| BCDIN3D | -1.24402 |
| BCL2L1 | 1.034956 |
| BCL2L13 | -1.01814 |
| BCLAF1 | -1.3211 |
| BCR | 1.523295 |
| BCS1L | -1.34149 |
| BDKRB1 | -1.2883 |
| BEND6 | -1.39424 |
| BEX2 | -2.10792 |
| BEX4 | -1.73686 |
| BEXL1 | -1.38606 |
| BIRC3 | 1.738257 |
| BIRC5 | 1.88915 |
| BLCAP | -1.31492 |
| BMP1 | 1.696273 |
| BMPR2 | -1.1403 |
| BNIP3L | -1.72541 |
| BNIPL | -1.04007 |
| BOC | -1.18323 |
| BOLA2 | 1.226904 |
| BOP1 | 1.287779 |
| BPGM | -1.36143 |
| BRDG1 | 2.76059 |
| BRE | -1.25543 |
| BRMS1 | 1.41291 |
| BSPRY | -2.06167 |
| BST2 | 1.667478 |
| BTBD11 | -1.18363 |
| BTBD16 | -1.31961 |
| BTN3A1 | 1.242869 |
| BYSL | -1.60341 |
| BZW2 | -1.4389 |
| C10orf10 | 1.003625 |
| C10orf113 | -1.2837 |
| C10orf137 | -1.82255 |
| C10orf33 | -1.16545 |
| C10orf61 | -1.48115 |
| C10orf76 | -1.61634 |
| C10orf84 | -1.86945 |
| C10orf99 | -1.2653 |
| C11orf1 | -1.4224 |
| C11orf41 | 1.092684 |
| C11orf45 | -1.3756 |
| C11orf75 | -1.16908 |
| C11orf87 | 1.003645 |
| C12orf29 | -1.07885 |
| C12orf34 | -1.27296 |
| C12orf49 | -1.17782 |
| C12orf76 | -1.17613 |
| C13orf1 | -1.37362 |
| C14orf133 | -1.04209 |
| C14orf138 | -1.14693 |
| C14orf139 | -1.81288 |
| C14orf147 | -1.12147 |
| C14orf156 | -1.62509 |
| C14orf166 | -1.58715 |
| C14orf167 | -1.22851 |
| C14orf2 | -1.00252 |
| C14orf32 | -1.61301 |
| C14orf73 | 2.092646 |
| C15orf24 | -1.66119 |
| C15orf48 | -2.34252 |
| C15orf58 | 1.661327 |
| C15orf59 | -1.33737 |
| C15orf62 | -1.00702 |
| C16orf5 | -1.25987 |
| C16orf56 | -1.44411 |
| C16orf63 | -1.13361 |
| C16orf72 | -1.32895 |
| C16orf73 | -1.33214 |
| C17orf106 | -1.02816 |
| C17orf48 | -1.08105 |
| C17orf56 | -1.27638 |
| C17orf59 | -1.14588 |
| C17orf63 | -1.06711 |
| C17orf65 | 1.446706 |
| C17orf69 | -1.02897 |
| C17orf82 | 1.232639 |
| C17orf87 | 2.089762 |
| C17orf97 | -1.45585 |
| C18orf19 | 1.389772 |
| C18orf25 | -1.42218 |
| C18orf26 | -1.43302 |
| C18orf55 | -1.81932 |
| C19orf21 | 1.592386 |
| C19orf50 | -1.1591 |
| C19orf6 | 1.248368 |
| C19orf73 | -1.6355 |
| C1orf106 | -1.68369 |
| C1orf115 | -1.37751 |
| C1orf116 | -2.30625 |
| C1orf124 | 1.009745 |
| C1orf128 | -2.38319 |
| C1orf131 | -1.96377 |
| C1orf190 | -1.05831 |
| C1orf198 | -1.0058 |
| C1orf21 | -1.39978 |
| C1orf210 | -1.05386 |
| C1orf25 | -1.1156 |
| C1orf51 | -2.07662 |
| C1orf66 | -1.37046 |
| C1orf68 | 1.332158 |
| C1QTNF3 | -2.05827 |
| C1QTNF5 | -1.51168 |
| C1QTNF6 | 2.164197 |
| C1R | 1.233552 |
| C20orf111 | -1.3098 |
| C20orf117 | -1.01431 |
| C20orf166 | 1.6591 |
| C20orf24 | -1.15585 |
| C20orf27 | 1.259041 |
| C20orf4 | -1.85601 |
| C20orf96 | -1.34768 |
| C21orf33 | -1.34319 |
| C21orf49 | 1.117589 |
| C21orf59 | -1.17343 |
| C21orf63 | -1.12576 |
| C21orf69 | 1.120769 |
| C22orf29 | -1.04126 |
| C22orf30 | -1.38301 |
| C2CD4A | 1.430894 |
| C2orf24 | -1.0756 |
| C2orf28 | -1.20047 |
| C2orf30 | -1.46814 |
| C2orf40 | -1.99022 |
| C2orf47 | -1.25419 |
| C2orf54 | -1.06372 |
| C2orf55 | -1.28309 |
| C2orf68 | -1.49904 |
| C2orf76 | -1.03184 |
| C3 | 2.199001 |
| C3orf14 | -1.09459 |
| C3orf31 | -1.86785 |
| C3orf32 | -1.32214 |
| C3orf35 | -1.02026 |
| C3orf39 | -1.8286 |
| C3orf51 | -1.14208 |
| C3orf54 | -1.16404 |
| C3orf58 | -1.96978 |
| C3orf64 | -1.04418 |
| C3orf70 | -1.12481 |
| C3orf74 | 1.58972 |
| C4orf14 | -1.2979 |
| C4orf19 | -1.11278 |
| C4orf28 | -1.13507 |
| C4orf31 | -1.46709 |
| C5orf21 | -1.42641 |
| C5orf39 | 1.275674 |
| C5orf51 | -1.20732 |
| C5orf56 | 1.478373 |
| C6orf106 | 1.578686 |
| C6orf124 | -1.03335 |
| C6orf145 | 1.183522 |
| C6orf150 | 1.387769 |
| C6orf205 | -3.36626 |
| C6orf48 | -1.22102 |
| C6orf70 | -1.55131 |
| C6orf85 | -2.17967 |
| C7orf26 | -1.13334 |
| C7orf30 | -1.34919 |
| C7orf36 | -1.02018 |
| C7orf44 | -1.10524 |
| C7orf55 | -1.51737 |
| C8orf40 | -1.81418 |
| C8orf41 | -1.11298 |
| C8orf58 | -1.02642 |
| C8orf76 | -1.42902 |
| C9orf103 | -1.4519 |
| C9orf125 | -1.95267 |
| C9orf140 | -1.15883 |
| C9orf152 | -2.62551 |
| C9orf16 | -1.43859 |
| C9orf164 | -1.08598 |
| C9orf167 | 1.624912 |
| C9orf24 | -1.76762 |
| C9orf30 | 1.354374 |
| C9orf61 | -2.72776 |
| C9orf66 | -1.23268 |
| C9orf9 | -1.86915 |
| CA2 | 1.429576 |
| CA3 | 1.045619 |
| CA5B | -1.10623 |
| CA8 | -1.36524 |
| CA9 | 1.593293 |
| CAB39L | -1.90815 |
| CABC1 | -1.73828 |
| CABLES2 | -1.41751 |
| CACNA1I | 2.269963 |
| CACNB1 | 1.208969 |
| CACNG1 | 1.901932 |
| CACNG6 | 2.100048 |
| CALM2 | -1.56872 |
| CALU | -1.12443 |
| CAMK2D | 1.19129 |
| CAMKK2 | 1.255077 |
| CAMKV | 1.381566 |
| CAMP | 2.766293 |
| CAMSAP1 | -1.24258 |
| CAP1 | -1.49739 |
| CAPN12 | 1.513374 |
| CAPNS2 | -1.48368 |
| CARD11 | 1.984445 |
| CARD16 | 1.639119 |
| CARD9 | 1.076839 |
| CARKD | -1.08561 |
| CARS | -1.16186 |
| CASC1 | -1.32991 |
| CASC2 | -1.72063 |
| CASC4 | 1.118268 |
| CASP14 | 1.48416 |
| CASP2 | -1.12694 |
| CASP3 | -1.06393 |
| CASP5 | 1.697908 |
| CASP9 | -1.04885 |
| CBFA2T3 | 1.221419 |
| CBFB | 1.191928 |
| CBR3 | -1.39044 |
| CBR4 | -1.18743 |
| CBS | 1.627574 |
| CBY1 | -2.04905 |
| CCDC101 | -1.02995 |
| CCDC115 | -1.08797 |
| CCDC127 | -1.11904 |
| CCDC144A | 1.27442 |
| CCDC17 | -1.17583 |
| CCDC34 | -1.23598 |
| CCDC49 | -1.17021 |
| CCDC53 | -1.43362 |
| CCDC6 | -1.71737 |
| CCDC64B | -1.47316 |
| CCDC80 | -1.2297 |
| CCDC90A | -1.85695 |
| CCL11 | 2.118813 |
| CCL14 | -1.47677 |
| CCL19 | 2.164098 |
| CCL2 | 1.327638 |
| CCL25 | 1.243772 |
| CCL3L3 | 2.096346 |
| CCL4L1 | 3.310251 |
| CCL5 | 3.039707 |
| CCL8 | 1.325228 |
| CCNB1 | 1.170118 |
| CCNB1IP1 | -1.38606 |
| CCNC | -1.67157 |
| CCNDBP1 | -1.58525 |
| CCNL1 | 1.290605 |
| CCR4 | 1.812489 |
| CCR6 | 2.048295 |
| CCR7 | 2.253769 |
| CCRN4L | 1.021342 |
| CCT5 | -1.05127 |
| CD163 | 1.075703 |
| CD164 | -1.3924 |
| CD164L2 | -1.73277 |
| CD177 | 1.536649 |
| CD180 | 1.129194 |
| CD19 | 2.798396 |
| CD207 | -1.18598 |
| CD226 | 1.976664 |
| CD24 | -1.48391 |
| CD247 | 1.727309 |
| CD27 | 1.977044 |
| CD276 | 1.142761 |
| CD28 | 1.185011 |
| CD300LF | 3.080125 |
| CD34 | -1.44463 |
| CD38 | 1.621379 |
| CD3D | 1.481978 |
| CD3G | 1.566288 |
| CD40 | 1.710886 |
| CD47 | -1.14985 |
| CD58 | -1.06436 |
| CD59 | -1.26774 |
| CD6 | 1.004652 |
| CD7 | 2.336181 |
| CD74 | 1.447349 |
| CD79A | 2.671387 |
| CD79B | 1.454023 |
| CD84 | 1.612656 |
| CD86 | 1.734878 |
| CD9 | -1.14291 |
| CD93 | -2.02075 |
| CD96 | 1.710247 |
| CDA | -1.14999 |
| CDC14A | 1.081847 |
| CDC14B | 1.103459 |
| CDC25C | 1.244587 |
| CDC2L2 | 1.27739 |
| CDC42BPB | -1.23651 |
| CDC42SE1 | 1.195271 |
| CDC42SE2 | 1.298749 |
| CDCA3 | 1.825493 |
| CDCA4 | -1.07301 |
| CDCA7 | -1.19572 |
| CDCP1 | -1.04071 |
| CDH13 | -1.95356 |
| CDH15 | 2.696898 |
| CDH16 | -2.22593 |
| CDH29 | -1.68655 |
| CDH6 | -1.14506 |
| CDK10 | 1.331344 |
| CDKN2A | 1.272998 |
| CDKN2B | -2.38084 |
| CDKN2D | -1.29782 |
| CDX1 | 2.484192 |
| CDYL | -1.34177 |
| CEACAM1 | -1.47342 |
| CEACAM7 | -2.24792 |
| CEBPG | -1.08388 |
| CELSR2 | -1.36585 |
| CELSR3 | 1.901567 |
| CENPBD1 | -1.34386 |
| CENPE | -1.01698 |
| CENPP | -1.20088 |
| CENPT | -1.79539 |
| CEP170 | 1.257758 |
| CEP290 | 1.213687 |
| CEP350 | -1.09985 |
| CEP63 | -1.26324 |
| CES2 | -1.14109 |
| CETP | 1.115586 |
| CGB1 | 2.812219 |
| CGNL1 | -1.71958 |
| CHCHD1 | -1.42949 |
| CHCHD10 | -1.86463 |
| CHCHD5 | -1.52425 |
| CHD1L | -1.32668 |
| CHDH | -1.1048 |
| CHES1 | -1.56627 |
| CHKB | 1.416181 |
| CHM | -1.58076 |
| CHMP2B | -1.38812 |
| CHMP4B | -1.36472 |
| CHMP4C | -1.20789 |
| CHPT1 | -1.13228 |
| CHRM2 | 1.322354 |
| CHRNA1 | 1.154831 |
| CHRNA10 | 1.084911 |
| CHST13 | 1.493115 |
| CHUK | -1.07258 |
| CIAPIN1 | -1.00328 |
| CIDEB | -1.44323 |
| CIDEC | 1.785205 |
| CIITA | 1.882188 |
| CILP | -1.08571 |
| CIRBP | -1.40414 |
| CISD2 | -1.71011 |
| CISH | -1.20481 |
| CITED2 | -1.80339 |
| CITED4 | -1.76963 |
| CKAP2 | 1.137949 |
| CKAP5 | -1.37707 |
| CKB | -1.0194 |
| CKMT1B | -2.09449 |
| CLASP1 | -1.37838 |
| CLCF1 | -1.08246 |
| CLCN3 | -1.23603 |
| CLCN6 | -1.03094 |
| CLCNKA | 1.13092 |
| CLDN17 | -2.09207 |
| CLDN23 | -1.05979 |
| CLDN7 | -1.55719 |
| CLDN8 | -1.08487 |
| CLDND1 | -1.87559 |
| CLEC12A | 1.055448 |
| CLEC4A | 1.277334 |
| CLEC4F | -1.5687 |
| CLEC4G | 1.280814 |
| CLEC4GP1 | -1.39484 |
| CLEC7A | 1.864478 |
| CLIC3 | -1.2395 |
| CLIC4 | 1.078419 |
| CLIC6 | -1.08679 |
| CLPP | -1.6524 |
| CLPTM1 | -1.12802 |
| CLPX | -1.81195 |
| CLSTN1 | 1.389035 |
| CLSTN3 | 1.229303 |
| CLTA | -1.44496 |
| CLU | -1.02264 |
| CLUAP1 | 1.186625 |
| CLYBL | -1.66604 |
| CMAS | -1.16391 |
| CMKLR1 | 1.531155 |
| CMTM1 | 1.561328 |
| CMYA3 | 1.475219 |
| CNBP | -1.57914 |
| CNGA1 | -1.61109 |
| CNGB1 | 1.148514 |
| CNKSR3 | -1.37976 |
| CNNM1 | -1.61103 |
| CNOT10 | -1.2865 |
| CNOT4 | -1.09044 |
| CNOT6L | -1.21711 |
| COASY | -1.11131 |
| COBL | -1.02473 |
| COCH | -1.39554 |
| COL11A1 | 1.057791 |
| COL19A1 | 1.035737 |
| COL22A1 | 2.827062 |
| COL4A2 | 1.585957 |
| COMMD3 | -1.3619 |
| COMMD6 | -1.27368 |
| COMP | -1.68645 |
| COMTD1 | -1.12061 |
| COPG2 | -1.14063 |
| COPS5 | -1.15195 |
| COPS6 | -1.77277 |
| COPS7B | -1.09706 |
| COQ5 | -1.27087 |
| CORO1A | 1.086799 |
| CORO1B | 1.068919 |
| CORO2B | -1.39315 |
| COX16 | -1.24024 |
| COX4NB | -1.07786 |
| COX5A | -1.04071 |
| COX6B2 | 2.517257 |
| COX7A1 | -1.32418 |
| COX7B | -1.07692 |
| COX7C | -2.14727 |
| COX8A | -1.37603 |
| CPA4 | -1.37595 |
| CPE | -1.14452 |
| CPEB1 | -1.51008 |
| CPLX1 | -1.00409 |
| CPLX3 | -1.06155 |
| CPN1 | 1.029235 |
| CPNE3 | -1.55137 |
| CPT2 | -1.14814 |
| CPVL | 1.209493 |
| CPXM2 | -1.2151 |
| CRB3 | -1.27147 |
| CREB3L3 | 1.549376 |
| CREG1 | -1.24037 |
| CRIM1 | 1.017474 |
| CRIP2 | -1.23014 |
| CRIPAK | -1.38997 |
| CRISP3 | -3.50259 |
| CRISPLD2 | -2.08022 |
| CRKRS | -1.36143 |
| CRLS1 | -1.09258 |
| CRNKL1 | -1.01512 |
| CRNN | -1.68 |
| CRTAC1 | -1.70347 |
| CRYZL1 | -1.08596 |
| CS | 1.036463 |
| CSAG1 | 1.748839 |
| CSAG3A | 1.420845 |
| CSDC2 | 2.364543 |
| CSF2 | 2.568197 |
| CSF2RB | 1.330129 |
| CSF3R | 2.062449 |
| CSMD2 | 2.057258 |
| CSNK2A2 | -1.02808 |
| CSNK2B | -1.25475 |
| CSRNP3 | -1.3545 |
| CSRP3 | 1.633953 |
| CST1 | 1.640433 |
| CST3 | -1.34563 |
| CST6 | -1.88218 |
| CSTB | -1.24497 |
| CSTF3 | -1.16675 |
| CTBP1 | 1.110632 |
| CTDSP1 | 1.011005 |
| CTDSP2 | -1.50618 |
| CTDSPL | -1.26273 |
| CTHRC1 | 1.03718 |
| CTLA4 | 2.859845 |
| CTNNA1 | -1.7118 |
| CTNNB1 | 1.206011 |
| CTNNBIP1 | -2.08433 |
| CTR9 | -1.2829 |
| CTRB2 | 1.412917 |
| CTSC | 1.047299 |
| CTSD | 1.259847 |
| CTSH | 1.106069 |
| CTSK | -1.70147 |
| CTSL1 | 1.032093 |
| CTSO | -1.028 |
| CTSW | 1.866664 |
| CUL2 | -1.52963 |
| CUL4A | -1.80228 |
| CUTA | -1.10969 |
| CUTC | -1.16875 |
| CWC15 | -1.03506 |
| CXADR | -1.16632 |
| CXCL1 | 2.79606 |
| CXCL10 | 3.149607 |
| CXCL11 | 1.656527 |
| CXCL13 | 2.396883 |
| CXCL6 | 3.210306 |
| CXCR3 | 1.353963 |
| CXCR4 | 3.331226 |
| CXCR5 | 1.338711 |
| CXorf21 | 2.221659 |
| CXorf40A | -1.44923 |
| CXorf40B | -1.08247 |
| CYB561D1 | -1.36937 |
| CYB5A | -2.12676 |
| CYB5D1 | -1.04967 |
| CYB5R1 | 1.046367 |
| CYBRD1 | -1.68559 |
| CYC1 | -1.32267 |
| CYFIP2 | 1.345391 |
| CYP26B1 | -1.59583 |
| CYP2C18 | -3.15247 |
| CYP2C19 | -1.50643 |
| CYP2E1 | -1.38163 |
| CYP2F1 | -2.41091 |
| CYP2J2 | -1.6834 |
| CYP2R1 | -1.65186 |
| CYP2S1 | -1.3905 |
| CYP3A4 | -1.13092 |
| CYP3A5 | -1.32244 |
| CYP4F12 | -3.00476 |
| CYP4F22 | -2.78147 |
| CYTH3 | -1.00147 |
| CYTSB | 1.003305 |
| CYYR1 | -1.4312 |
| D2HGDH | -1.16087 |
| D4S234E | -1.50489 |
| DAAM1 | -1.03525 |
| DACT2 | -1.43593 |
| DAD1 | -1.40566 |
| DAG1 | -1.18193 |
| DAGLA | -1.18161 |
| DAND5 | 1.326757 |
| DAP3 | -1.78274 |
| DAPL1 | -1.33665 |
| DARC | -1.39876 |
| DAZAP2 | -1.93721 |
| DBF4B | -1.10048 |
| DBI | -1.29328 |
| DBNDD1 | -3.00756 |
| DCAF15 | 1.03421 |
| DCBLD1 | 1.598478 |
| DCLRE1B | -1.08604 |
| DCTD | -1.61789 |
| DCTN1 | -1.00231 |
| DCTN3 | -1.02743 |
| DCTPP1 | -1.04485 |
| DDAH1 | -1.86633 |
| DDHD1 | 1.268929 |
| DDR2 | -1.41709 |
| DDX1 | -1.2608 |
| DDX10 | -1.46828 |
| DDX18 | -1.46191 |
| DDX23 | -1.22216 |
| DDX24 | -1.52933 |
| DDX41 | -1.77998 |
| DDX46 | -1.3787 |
| DDX5 | -1.34514 |
| DDX54 | -1.06367 |
| DDX60 | 3.242381 |
| DDX60L | 2.434084 |
| DEFB1 | -2.09002 |
| DEGS2 | -1.71923 |
| DENND1C | 1.834608 |
| DENND5A | -1.31454 |
| DERL3 | 1.125486 |
| DGCR10 | 1.025291 |
| DGKA | 1.161567 |
| DGKB | 1.021574 |
| DGKD | 1.340204 |
| DGKH | 1.056244 |
| DHRS4 | -1.85151 |
| DHRS7B | -1.13637 |
| DHRS9 | -1.73723 |
| DHX29 | -1.19418 |
| DHX30 | -1.28466 |
| DHX33 | -1.10822 |
| DHX40 | -1.58224 |
| DIABLO | -1.04692 |
| DIAPH1 | -1.75866 |
| DIAPH3 | 1.015902 |
| DIDO1 | -1.08535 |
| DIO2 | -1.55837 |
| DIP2B | -1.03901 |
| DIS3 | 1.052657 |
| DKK1 | 1.162678 |
| DKK3 | -1.06792 |
| DKK4 | -2.19138 |
| DLEU1 | -1.60084 |
| DLG2 | -2.02119 |
| DLGAP4 | -1.01232 |
| DLGAP5 | 1.012839 |
| DLX3 | -1.04229 |
| DMBT1 | 1.186483 |
| DMKN | -1.93948 |
| DNAH17 | 1.889246 |
| DNAH2 | 2.451359 |
| DNAJA4 | -2.16171 |
| DNAJB1 | -1.74518 |
| DNAJB12 | 1.193746 |
| DNAJB6 | -1.87323 |
| DNAJC13 | -1.46675 |
| DNAJC2 | 1.254298 |
| DNAJC25 | -1.27114 |
| DNAJC27 | -1.2852 |
| DNAL1 | 1.062781 |
| DNASE1 | -1.11891 |
| DNASE1L3 | -2.20595 |
| DOCK9 | -1.23491 |
| DOK3 | 2.003279 |
| DOPEY2 | -1.0279 |
| DPCR1 | -1.63081 |
| DPM1 | -1.09346 |
| DPT | -1.69972 |
| DPYD | 1.13022 |
| DPYSL4 | 1.431699 |
| DRD1IP | 1.015036 |
| DSCR3 | -1.38618 |
| DSE | 1.532605 |
| DSEL | 1.037695 |
| DSG3 | -1.16717 |
| DSP | -1.15941 |
| DSTN | -1.55977 |
| DTD1 | -2.2591 |
| DTNA | 1.012126 |
| DTNB | 1.038226 |
| DUS3L | -1.51907 |
| DUSP1 | -1.6368 |
| DUSP10 | 1.911738 |
| DUSP18 | -1.54198 |
| DUSP22 | -1.387 |
| DUT | -1.95725 |
| DVL2 | -1.10488 |
| DYM | -1.69831 |
| DYNLL1 | -1.15745 |
| DYNLT3 | -1.24041 |
| EAF1 | -1.33342 |
| EBF1 | -1.70512 |
| EBI2 | 1.434936 |
| ECE1 | 1.505363 |
| ECGF1 | 1.945327 |
| ECH1 | -1.13454 |
| ECHDC3 | -1.93796 |
| ECHS1 | -1.3117 |
| EDAR | -1.01079 |
| EDN3 | -1.32639 |
| EEF1E1 | -1.99211 |
| EEPD1 | -1.49686 |
| EFCAB1 | -1.18535 |
| EFHD1 | -2.14078 |
| EFNB3 | -2.37117 |
| EFS | -1.61175 |
| EGFL6 | 1.900604 |
| EHBP1 | -1.06806 |
| EIF1 | -1.34884 |
| EIF1AD | -1.04973 |
| EIF2B4 | -1.4957 |
| EIF2S2 | -1.31951 |
| EIF3H | -1.15276 |
| EIF3L | -1.15032 |
| EIF4E3 | -1.65891 |
| EIF4G2 | -1.74752 |
| EIF4H | -2.13029 |
| ELAC2 | -1.25888 |
| ELF2 | -1.86182 |
| ELF3 | -1.06512 |
| ELF5 | -2.40536 |
| ELK1 | -1.02086 |
| ELL2 | -1.14943 |
| ELL3 | -1.69403 |
| ELMO1 | -1.02053 |
| ELP4 | -1.53263 |
| EML1 | -1.30981 |
| EMP1 | -1.12429 |
| EMR2 | 1.229252 |
| EMR3 | 3.334447 |
| ENOPH1 | -2.57687 |
| ENPEP | 1.526403 |
| ENTPD2 | -1.2242 |
| ENTPD6 | 1.151103 |
| EOMES | 2.556815 |
| EPB41L1 | 1.04656 |
| EPHA1 | -1.068 |
| EPHA3 | -1.87102 |
| EPHB6 | -2.15282 |
| EPHX2 | -1.80387 |
| EPM2AIP1 | -1.09528 |
| EPN2 | -1.50698 |
| EPN3 | -1.1034 |
| EPS15 | -1.42103 |
| EPS15L1 | -1.07814 |
| EPS8L1 | -1.58476 |
| EPSTI1 | 2.126663 |
| ERBB3 | -1.65053 |
| ERCC3 | -1.52984 |
| ERGIC1 | 1.053064 |
| ERRFI1 | -1.95332 |
| ESF1 | 1.088758 |
| ESM1 | 2.098911 |
| ESPL1 | -1.59074 |
| ESPNL | 1.175627 |
| ETFDH | -1.22099 |
| ETNK1 | -1.23654 |
| ETNK2 | -1.09528 |
| ETV5 | -1.69555 |
| EVI2B | 1.390066 |
| EXD3 | -1.14582 |
| EXOSC7 | -1.36919 |
| EXPH5 | -1.22551 |
| EYA2 | -1.82368 |
| F10 | -1.47323 |
| F2RL2 | 1.126509 |
| FABP4 | 1.011217 |
| FABP5 | -1.01263 |
| FADS2 | 1.736366 |
| FAIM2 | -1.60454 |
| FAIM3 | 2.169256 |
| FAM101B | 1.07088 |
| FAM107A | -1.60806 |
| FAM127B | -2.00491 |
| FAM127C | -1.5815 |
| FAM129A | -1.54627 |
| FAM129B | -1.1788 |
| FAM149B1 | -1.41973 |
| FAM156A | -1.13427 |
| FAM156B | -1.37221 |
| FAM160A2 | -1.82526 |
| FAM162A | -1.82879 |
| FAM168B | -1.29523 |
| FAM175B | -1.13551 |
| FAM176A | 1.34057 |
| FAM179A | -1.11011 |
| FAM181A | 1.420775 |
| FAM25C | -1.18615 |
| FAM25G | -1.1307 |
| FAM26E | 1.006314 |
| FAM36A | -2.01579 |
| FAM3B | -1.9313 |
| FAM3D | -2.48655 |
| FAM43B | -1.57971 |
| FAM46B | -1.87431 |
| FAM46C | 1.874604 |
| FAM53B | 1.091496 |
| FAM54B | -1.00232 |
| FAM55C | 1.354838 |
| FAM57A | -1.68684 |
| FAM60A | -1.06006 |
| FAM70B | 1.076168 |
| FAM83A | 1.708748 |
| FAM83D | -1.77479 |
| FAM89B | -1.19689 |
| FAM96A | -1.40854 |
| FANCD2 | 1.467128 |
| FARSA | -1.14931 |
| FASTK | -1.15869 |
| FASTKD1 | -1.07714 |
| FAT1 | 1.186658 |
| FBN2 | 1.049757 |
| FBXL15 | -1.51826 |
| FBXL5 | -1.59681 |
| FBXO21 | 1.049232 |
| FBXO3 | -1.60361 |
| FBXO33 | -1.65023 |
| FBXO34 | -1.63562 |
| FBXO4 | -1.02689 |
| FBXO44 | -1.27422 |
| FBXO6 | 1.693631 |
| FBXW4 | -1.83263 |
| FBXW7 | -1.88377 |
| FCER1G | 1.258207 |
| FCGR2A | 1.649908 |
| FCGR3A | 2.965328 |
| FCHO2 | -1.40556 |
| FCN1 | 2.350232 |
| FCRLA | 1.807875 |
| FER | -1.01426 |
| FER1L4 | 1.514415 |
| FERMT3 | 1.268778 |
| FEZ1 | 2.358515 |
| FGD5 | -1.61257 |
| FGF12 | -1.74393 |
| FGFBP2 | -1.11702 |
| FHDC1 | -1.03057 |
| FHL1 | -1.4304 |
| FIG4 | -1.25859 |
| FIT1 | 1.097863 |
| FKBP9L | -1.58183 |
| FLCN | 1.278759 |
| FLG | -2.35926 |
| FLG2 | -2.02879 |
| FLJ10081 | -1.41976 |
| FLJ10996 | -1.31941 |
| FLJ12684 | -1.71632 |
| FLJ16165 | 1.102535 |
| FLJ20273 | -1.25426 |
| FLJ20674 | -1.9444 |
| FLJ22662 | -1.36894 |
| FLJ33590 | 2.60694 |
| FLJ33630 | -1.18787 |
| FLJ35776 | 1.462055 |
| FLJ38973 | -1.32842 |
| FLJ41423 | 1.580689 |
| FLJ41562 | -1.13811 |
| FLJ42627 | -1.34286 |
| FLJ44048 | 1.106458 |
| FLJ90650 | 1.11536 |
| FLNA | 1.092656 |
| FLYWCH2 | -1.34621 |
| FMNL3 | 1.486196 |
| FMO2 | -1.03395 |
| FMO3 | -1.06116 |
| FMO9P | -1.5384 |
| FMOD | -1.08559 |
| FN1 | 1.460751 |
| FNBP1L | -1.02656 |
| FNBP4 | -1.61253 |
| FNDC4 | -1.13894 |
| FOLR3 | 3.194423 |
| FOXA1 | -3.01614 |
| FOXRED2 | 1.05522 |
| FPGS | 1.02586 |
| FPR3 | 1.6046 |
| FRAS1 | -2.62201 |
| FRAT2 | -1.46444 |
| FRK | 1.107885 |
| FRYL | 1.036075 |
| FST | 1.210918 |
| FSTL1 | -2.14358 |
| FTL | 1.849893 |
| FTO | -1.03539 |
| FTSJ1 | -1.20503 |
| FUCA1 | -1.08549 |
| FURIN | 1.615865 |
| FUT3 | -1.91083 |
| FVT1 | -2.0927 |
| FXR1 | 1.942287 |
| FXYD2 | 1.053767 |
| FYN | 1.810678 |
| FZD4 | -1.54832 |
| FZD6 | -1.00966 |
| FZD7 | -1.85496 |
| G0S2 | 2.489477 |
| G6PC3 | -1.04731 |
| GAA | -1.53487 |
| GABARAPL2 | -1.11068 |
| GABRB3 | -1.55093 |
| GAD1 | 1.356119 |
| GALNT1 | -1.18841 |
| GALNT10 | 1.595757 |
| GALNT11 | -1.0564 |
| GALNTL1 | -1.9065 |
| GALNTL4 | -1.04878 |
| GAN | -1.03318 |
| GAR1 | -1.86456 |
| GART | 1.366062 |
| GAS1 | -1.07739 |
| GAS2L1 | -1.98224 |
| GAS8 | -1.77986 |
| GATA2 | 1.228675 |
| GATAD2B | 1.033783 |
| GBA | -1.422 |
| GBP1 | 1.178692 |
| GBP3 | 1.324473 |
| GBP5 | 2.047595 |
| GBP6 | -2.02593 |
| GCAT | -1.04223 |
| GCK | -1.15059 |
| GCNT1 | 1.337092 |
| GCNT3 | -1.43368 |
| GCOM1 | -1.50719 |
| GDE1 | -1.97621 |
| GDF10 | -1.43107 |
| GDPD3 | -1.94899 |
| GFI1 | 1.60274 |
| GFRA3 | -1.01568 |
| GGCX | -1.0141 |
| GGNBP2 | -1.04048 |
| GGPS1 | -1.09897 |
| GGT6 | -1.65045 |
| GINS3 | -1.28869 |
| GIPC2 | -1.70635 |
| GIYD1 | 1.071797 |
| GIYD2 | 1.313478 |
| GJA5 | 1.26755 |
| GK | 1.029308 |
| GLCE | -2.17854 |
| GLDN | -1.32523 |
| GLT8D1 | -1.68098 |
| GLTP | -1.18076 |
| GLTPD1 | -1.36577 |
| GLUL | -1.02003 |
| GMCL1 | -1.0559 |
| GMNN | -1.57115 |
| GNA11 | -1.46476 |
| GNA12 | 1.321278 |
| GNA15 | -1.46071 |
| GNAT1 | -1.03241 |
| GNLY | 2.54626 |
| GNRH1 | -1.04204 |
| GOLGA5 | -1.36786 |
| GOLGB1 | -1.06101 |
| GOLPH3L | -1.45575 |
| GOLPH4 | -1.31554 |
| GOLSYN | -1.09741 |
| GORASP1 | -1.6122 |
| GPATCH2 | -1.39673 |
| GPATCH4 | -1.22003 |
| GPATCH8 | -1.15012 |
| GPC4 | -1.18884 |
| GPHN | -1.2034 |
| GPM6B | -2.01284 |
| GPR114 | 1.425772 |
| GPR126 | -1.14586 |
| GPR153 | 1.040499 |
| GPR160 | 1.321741 |
| GPR172B | 1.461128 |
| GPR177 | -1.07543 |
| GPR183 | 1.368443 |
| GPR55 | 1.432197 |
| GPR56 | -1.34616 |
| GPR65 | 1.862326 |
| GPR98 | -1.47621 |
| GPRASP2 | -1.10415 |
| GPRC5A | -1.83094 |
| GPRIN3 | 1.358646 |
| GPSM2 | -1.227 |
| GPX2 | -1.26082 |
| GRAMD1A | 1.176451 |
| GRAMD1B | 1.002631 |
| GRAMD4 | -1.4239 |
| GRAP2 | 1.514091 |
| GRB7 | -1.38212 |
| GREM1 | 1.746513 |
| GREM2 | -1.93023 |
| GRHL1 | -1.07501 |
| GRHL3 | -1.10406 |
| GRINA | 1.439602 |
| GRM2 | 1.150088 |
| GRP | 3.42758 |
| GSN | -1.0384 |
| GSTA4 | -1.42578 |
| GSTM4 | -2.54365 |
| GSTM5 | -2.46134 |
| GSTT1 | -1.15163 |
| GTF2F1 | -1.0279 |
| GTF2IRD1 | -2.02283 |
| GTF3C1 | -1.28214 |
| GTF3C2 | -1.33734 |
| GUCY1A3 | 1.164202 |
| GUCY2C | -3.13241 |
| GZMA | 1.463086 |
| GZMB | 2.475432 |
| H19 | -1.63522 |
| H2AFJ | -1.06389 |
| H2AFZ | -2.08116 |
| H3F3A | -1.81654 |
| HADH2 | -2.37388 |
| HAMP | 1.009686 |
| HAS3 | 1.051205 |
| HAT1 | -1.06461 |
| HAUS5 | -1.17374 |
| HAVCR2 | 1.745397 |
| HBXIP | -2.02498 |
| HCLS1 | 1.0118 |
| HCST | 1.055151 |
| HDAC7A | 1.220081 |
| HDC | 1.052836 |
| HDGF | -1.09127 |
| HEATR5B | -1.53982 |
| HECTD1 | -1.52707 |
| HELZ | -1.0455 |
| HERC1 | -1.52265 |
| HERC5 | 1.87094 |
| HES2 | -1.0499 |
| HEYL | 1.060376 |
| HFE2 | 2.361771 |
| HHATL | 1.187012 |
| HIC2 | -1.23522 |
| HIF1A | 1.19538 |
| HINFP | -1.27908 |
| HIP1R | 1.136291 |
| HIST1H2AB | 1.107242 |
| HIST1H2AH | 1.493145 |
| HIST1H2AJ | 1.056808 |
| HIST1H2BD | 1.044833 |
| HIST1H2BF | 1.315736 |
| HIST1H2BH | 1.340217 |
| HIST1H2BJ | 1.017121 |
| HIST1H2BK | 1.185322 |
| HIST1H3H | 1.122927 |
| HIST1H4B | 1.025691 |
| HIST2H2AA4 | 1.199414 |
| HIST2H4B | 1.359588 |
| HIVEP1 | -1.17054 |
| HLA-A29.1 | 1.62705 |
| HLA-DMA | 1.664684 |
| HLA-DPA1 | 1.378991 |
| HLA-DQA1 | 1.928668 |
| HLA-DQA2 | 1.334023 |
| HLA-DQB2 | 1.060662 |
| HLA-DRA | 1.444507 |
| HLA-DRB6 | 1.406672 |
| HLA-F | 2.201067 |
| HLF | -2.43277 |
| HM13 | 1.252453 |
| HMCN1 | -1.26189 |
| HNMT | -1.23284 |
| HNRNPF | -1.47644 |
| HNRPH3 | -1.1513 |
| HNRPLL | -1.21858 |
| HOMER2 | -2.0096 |
| HOPX | -2.18642 |
| HOXA11AS | 1.326989 |
| HOXA3 | 2.009857 |
| HOXB5 | 1.25065 |
| HOXB7 | 1.033683 |
| HOXC4 | 2.227119 |
| HOXC6 | 1.378202 |
| HOXC9 | 3.341696 |
| HOXD10 | 1.218361 |
| HOXD13 | 1.12782 |
| HPCAL4 | 1.396544 |
| HPDL | -1.53545 |
| HPGD | -1.57113 |
| HRASLS | -2.74433 |
| HRCT1 | -1.24489 |
| HSBP1 | -1.06111 |
| HSD11B2 | -1.40256 |
| HSD17B1 | -1.60784 |
| HSD17B10 | -2.2736 |
| HSDL2 | -1.20418 |
| HSFX1 | 1.073349 |
| HSP90AB1 | -1.14987 |
| HSPA1B | -1.36927 |
| HSPA1L | -1.07206 |
| HSPB3 | 1.839662 |
| HSPC111 | -1.31829 |
| HSPC157 | -1.15888 |
| HTR3A | -2.25102 |
| HTRA4 | 1.214899 |
| HTT | -1.55089 |
| HYAL1 | -1.20639 |
| HYAL2 | 1.273081 |
| HYAL3 | -1.21866 |
| IARS2 | -1.11921 |
| ICA1 | -1.24205 |
| ICMT | -1.93963 |
| ICOS | 2.836664 |
| ICOSLG | -1.0007 |
| ID1 | -1.38434 |
| ID2 | -1.27202 |
| ID4 | -1.37759 |
| IDE | -1.82809 |
| IDH3A | -1.99253 |
| IDH3B | -1.47175 |
| IDH3G | -1.34419 |
| IDS | -2.09888 |
| IFFO2 | -1.50504 |
| IFI27 | 1.12373 |
| IFI35 | 1.339887 |
| IFI44 | 1.922845 |
| IFI44L | 2.102788 |
| IFI6 | 2.320002 |
| IFIT3 | 2.174704 |
| IFITM1 | 1.375557 |
| IFNAR2 | 1.451875 |
| IFNG | 1.09005 |
| IFRD1 | -1.53284 |
| IFT122 | 1.012484 |
| IGFBP2 | -1.55621 |
| IGFBP3 | -1.0457 |
| IGFBP5 | -1.26132 |
| IGFBP6 | -1.40303 |
| IGFL1 | -1.44729 |
| IGFN1 | 1.499529 |
| IGJ | 2.174635 |
| IGLL1 | 3.166936 |
| IGSF1 | -1.57915 |
| IHPK3 | 1.641742 |
| IKZF1 | 1.750957 |
| IKZF3 | 2.243885 |
| IL12RB1 | 2.098215 |
| IL13RA1 | -2.05287 |
| IL17C | 1.172093 |
| IL17D | -1.83421 |
| IL17RA | 1.401883 |
| IL18BP | 1.23668 |
| IL18R1 | 1.106878 |
| IL18RAP | 2.138036 |
| IL19 | 2.035881 |
| IL1F6 | -1.46017 |
| IL1RN | -1.84106 |
| IL21R | 3.111834 |
| IL24 | 4.787278 |
| IL2RA | 4.247185 |
| IL2RG | 1.560768 |
| IL32 | 2.049403 |
| IL7R | 2.253651 |
| IL8 | 2.788373 |
| ILDR1 | -1.27001 |
| IMMT | -1.07525 |
| IMPA2 | -1.65223 |
| IMPACT | -1.281 |
| IMPDH2 | -1.67862 |
| INDO | 1.687691 |
| ING1 | -1.05605 |
| INHBA | 2.959481 |
| INMT | -1.41293 |
| INS-IGF2 | 1.219127 |
| INSM2 | 1.17305 |
| INTS10 | -2.16233 |
| INTS12 | -1.02385 |
| INTS2 | -1.31716 |
| IQGAP1 | -1.60999 |
| IRF1 | 1.482625 |
| IRF2BP2 | 1.60213 |
| IRF6 | 1.122579 |
| IRX5 | 1.141527 |
| ISCA2 | 1.219856 |
| ISCU | -1.29244 |
| ISG15 | 2.103577 |
| ISL1 | -1.31267 |
| ISLR | 1.086287 |
| ISOC1 | -1.56006 |
| ITFG3 | -1.77675 |
| ITGA10 | -1.12772 |
| ITGA2 | 1.049987 |
| ITGA3 | 3.058668 |
| ITGA8 | 1.160678 |
| ITGAL | 2.230949 |
| ITGAX | 2.127343 |
| ITGB1BP1 | -1.25903 |
| ITGB4 | 1.519969 |
| ITGB4BP | -1.3766 |
| ITGB5 | -1.55703 |
| ITGB6 | 2.109164 |
| ITIH5 | -1.43287 |
| ITM2A | -1.74619 |
| ITM2C | 1.872486 |
| ITPR2 | -1.25578 |
| ITPRIP | -1.18581 |
| JAM2 | -1.4431 |
| JARID2 | -1.23517 |
| JAZF1 | -1.63004 |
| JMJD1A | -1.48506 |
| JMJD1C | -1.56487 |
| JPH2 | 1.748025 |
| JSRP1 | 3.178766 |
| JTB | -1.35502 |
| JUP | -1.98277 |
| KANK1 | -2.14643 |
| KANK4 | 2.27343 |
| KARS | -1.29534 |
| KAT2B | -2.23107 |
| KATNAL1 | -1.53936 |
| KATNAL2 | -1.05552 |
| KCNF1 | 2.188578 |
| KCNH3 | 1.697071 |
| KCNH4 | 1.320716 |
| KCNIP3 | 1.519906 |
| KCNK10 | -1.27018 |
| KCNQ1 | 1.784733 |
| KCNT1 | 1.451371 |
| KCTD12 | -1.10903 |
| KCTD18 | 1.251911 |
| KCTD21 | -1.2318 |
| KDELC2 | -1.32497 |
| KDELR3 | -1.13525 |
| KDM5B | -1.02936 |
| KEL | -1.2669 |
| KHDRBS3 | -1.16738 |
| KHNYN | -2.25219 |
| KIAA0020 | -1.12835 |
| KIAA0141 | -1.24274 |
| KIAA0196 | -1.18175 |
| KIAA0232 | -1.31605 |
| KIAA0247 | -1.0254 |
| KIAA0284 | -1.5492 |
| KIAA0319L | -1.372 |
| KIAA0355 | -1.51804 |
| KIAA0368 | -1.13035 |
| KIAA0460 | -1.63441 |
| KIAA0513 | -1.5413 |
| KIAA0564 | -1.09894 |
| KIAA0649 | -1.47049 |
| KIAA0895L | -1.82834 |
| KIAA0907 | -1.13799 |
| KIAA1147 | -1.28475 |
| KIAA1324L | 1.193451 |
| KIAA1429 | -1.08516 |
| KIAA1430 | -1.44689 |
| KIAA1524 | 1.024562 |
| KIAA1543 | -1.20681 |
| KIAA1618 | 1.778475 |
| KIAA1632 | -1.05723 |
| KIAA1737 | -1.19351 |
| KIAA1826 | -1.37824 |
| KIAA1875 | 1.365299 |
| KIAA1967 | -1.6578 |
| KIAA1984 | 1.166199 |
| KIDINS220 | -1.09412 |
| KIF16B | -1.00818 |
| KIF21A | -1.24129 |
| KIF23 | 1.880841 |
| KIFC1 | -1.12276 |
| KIR2DL1 | 2.072658 |
| KIR2DL3 | 1.70617 |
| KIR3DL2 | 1.042329 |
| KIRREL | 1.066667 |
| KITLG | 1.17077 |
| KLC1 | -1.15138 |
| KLC3 | -1.58919 |
| KLF10 | 1.470558 |
| KLF11 | -1.04195 |
| KLF4 | -2.05807 |
| KLF6 | -1.37154 |
| KLF8 | -1.83023 |
| KLF9 | -1.32179 |
| KLHDC9 | -1.54177 |
| KLHL21 | -1.12037 |
| KLHL5 | 1.281429 |
| KLHL7 | -1.2899 |
| KLK10 | -1.13271 |
| KLK12 | -1.16665 |
| KLK13 | -1.81155 |
| KLK5 | -1.25772 |
| KLRF1 | 1.978359 |
| KLRG1 | 1.286239 |
| KPNA2 | 1.054209 |
| KPNA6 | -1.76936 |
| KPRP | -2.5683 |
| KPTN | -1.40577 |
| KRAS | -1.14842 |
| KRT1 | -2.34489 |
| KRT13 | -2.21364 |
| KRT15 | -1.65087 |
| KRT2 | -1.88874 |
| KRT24 | 1.280633 |
| KRT3 | -2.7911 |
| KRT33B | -3.24513 |
| KRT34 | -1.72006 |
| KRT4 | -3.25811 |
| KRT76 | -3.03099 |
| KRT80 | -1.95538 |
| KRTAP13-2 | -1.37894 |
| KRTAP3-2 | -1.81709 |
| KRTAP4-7 | -1.74959 |
| KRTAP9-4 | -1.47848 |
| KTELC1 | -1.03992 |
| KTI12 | -1.45302 |
| LAD1 | -1.30212 |
| LAG3 | 1.060763 |
| LAGE3 | -2.09619 |
| LAIR2 | 2.431608 |
| LAMA3 | 1.842864 |
| LAMB1 | 1.233999 |
| LAMB2 | -1.18513 |
| LAMB3 | 2.168096 |
| LAMC2 | 2.542248 |
| LAMP2 | -1.40054 |
| LAMP2 | -1.40054 |
| LAMP3 | 1.090778 |
| LANCL2 | -1.20092 |
| LAPTM4B | 1.274203 |
| LARGE | 1.11487 |
| LARP1B | -1.09417 |
| LARP4B | -1.10602 |
| LAT | 1.479262 |
| LAT2 | 2.077156 |
| LATS2 | -1.13172 |
| LBX1 | 1.404548 |
| LCE1C | -1.07317 |
| LCE3A | -1.83876 |
| LCE3D | -1.67884 |
| LCE5A | -1.76121 |
| LCK | 1.287461 |
| LCMT1 | 1.381624 |
| LCN2 | -1.24431 |
| LCOR | -1.04333 |
| LCP2 | 2.21171 |
| LDB2 | -1.22306 |
| LDHA | -1.31394 |
| LDHD | -1.14979 |
| LDOC1 | -1.15128 |
| LEO1 | 1.167287 |
| LEPR | -1.13396 |
| LEPROTL1 | -1.24826 |
| LETMD1 | -1.36344 |
| LGALS3BP | 1.220068 |
| LGI1 | -1.15132 |
| LGSN | 1.080959 |
| LGTN | -1.75613 |
| LHCGR | -1.06041 |
| LHX1 | 1.328154 |
| LILRA3 | 2.004871 |
| LILRA5 | 1.917975 |
| LILRB1 | 1.299173 |
| LILRB2 | 1.450622 |
| LILRB3 | 1.71644 |
| LIMA1 | 1.007109 |
| LIMD2 | 1.46184 |
| LMBR1 | -1.34231 |
| LMNB1 | 1.046845 |
| LMX1A | -1.81651 |
| LOC100129387 | 1.128459 |
| LOC100131551 | -1.24795 |
| LOC100144604 | 1.308244 |
| LOC100216001 | 1.127057 |
| LOC144438 | -1.21963 |
| LOC201725 | -1.59558 |
| LOC283267 | -1.33891 |
| LOC284276 | -1.00151 |
| LOC389517 | 1.152855 |
| LOC400891 | -1.21232 |
| LOC401052 | -1.01325 |
| LOC401152 | -1.47127 |
| LOC401233 | -1.03317 |
| LOC401357 | -1.11694 |
| LOC401720 | 1.336695 |
| LOC440157 | 1.789078 |
| LOC440957 | -1.07374 |
| LOC645233 | -1.1948 |
| LOC645431 | 1.894346 |
| LOC645993 | -1.32554 |
| LOC647340 | -1.05852 |
| LOC653566 | 1.126685 |
| LOC654433 | -1.48064 |
| LOC728855 | 1.584872 |
| LOC728937 | -1.27218 |
| LOC729617 | -1.09963 |
| LOC730417 | -1.26067 |
| LOC730744 | -1.47345 |
| LOC730833 | -2.21612 |
| LOC92659 | -1.17053 |
| LOH3CR2A | -1.14603 |
| LONP1 | -1.33509 |
| LONRF1 | -1.1998 |
| LOR | -1.55222 |
| LPHN1 | -1.45324 |
| LPIN1 | -2.25825 |
| LRBA | -1.84344 |
| LRP4 | -1.21532 |
| LRPPRC | -1.36845 |
| LRRC25 | 1.618779 |
| LRRC37A4 | -1.06365 |
| LRRC49 | -1.03136 |
| LRRC57 | -1.34662 |
| LRRC8A | -1.19869 |
| LRRFIP2 | -1.38351 |
| LRRN4CL | -2.16921 |
| LSAMP | 1.204991 |
| LSM11 | 1.631683 |
| LSM12 | 1.640453 |
| LSM3 | -1.20751 |
| LSM6 | -1.01029 |
| LSS | -1.95087 |
| LST1 | 1.316646 |
| LTA | 2.592607 |
| LTB | 1.52159 |
| LTB4R2 | -1.16371 |
| LY6E | 1.023711 |
| LY6G6C | -2.15136 |
| LY6G6D | -1.03176 |
| LY6H | 2.131115 |
| LY75 | 1.092633 |
| LYAR | -1.21887 |
| LYPD1 | 1.434263 |
| LYPD3 | -1.28314 |
| LYPLAL1 | -1.73654 |
| LYRM4 | -1.49343 |
| LYRM5 | -1.02539 |
| LYSMD2 | -1.43809 |
| LYVE1 | -1.07872 |
| LYZ | 1.443038 |
| LZTFL1 | -1.24434 |
| LZTS2 | -1.11824 |
| MACC1 | -1.04455 |
| MACF1 | 1.10745 |
| MACROD1 | -1.43349 |
| MAF | -1.04894 |
| MAFB | -1.01897 |
| MAGED1 | -1.60986 |
| MAGED4 | 2.004966 |
| MAGOH | -1.56771 |
| MAL | -2.01694 |
| MAL2 | -1.0835 |
| MAMDC2 | -2.50141 |
| MANBA | -1.30785 |
| MANBAL | -1.08484 |
| MANSC1 | -1.46651 |
| MAOB | -1.26389 |
| MAP2 | -1.60346 |
| MAP2K1 | -1.35387 |
| MAP2K4 | -1.05237 |
| MAP3K4 | -1.69216 |
| MAP3K7IP3 | -1.51671 |
| MAP3K8 | -2.03498 |
| MAP4 | 1.096731 |
| MAP4K1 | 1.660714 |
| MAP4K5 | -1.38642 |
| MAP7 | -1.40479 |
| MAPK1 | -1.14882 |
| MAPK13 | -1.33984 |
| MAPK3 | -1.79237 |
| MAPK6 | -1.27622 |
| MAPK7 | -1.4997 |
| MAPT | -1.68115 |
| MARCKSL1 | 1.127451 |
| MARVELD2 | -1.50489 |
| MASP1 | -1.49311 |
| MAT2B | 1.250942 |
| MATK | 1.295832 |
| MATN2 | -1.25766 |
| MAX | 1.413442 |
| MAZ | 1.005906 |
| MBD4 | -1.05312 |
| MBIP | -1.04041 |
| MBLAC1 | -1.64667 |
| MBNL1 | -1.29751 |
| MBNL2 | -1.677 |
| MBP | -1.62044 |
| MCEE | -1.62396 |
| MCM2 | -1.8019 |
| MCTS1 | -1.27181 |
| MDH1 | -1.20641 |
| MDK | 1.204382 |
| ME1 | -1.00215 |
| ME3 | -1.76243 |
| MED24 | -2.05187 |
| MED31 | -1.25694 |
| MEFV | 2.788119 |
| MEGF6 | -1.75105 |
| MEGF9 | -2.82444 |
| MEIS1 | -1.15964 |
| MEIS2 | -1.12452 |
| MEOX2 | -1.56926 |
| METAP1 | -1.37814 |
| METTL7A | -1.0862 |
| MEX3D | 1.133823 |
| MFAP2 | 1.632589 |
| MFF | -1.20058 |
| MFSD5 | -1.82169 |
| MFSD8 | -1.83242 |
| MGAT1 | -1.17117 |
| MGAT2 | 1.00965 |
| MGC26718 | -1.95312 |
| MGC29506 | 2.578928 |
| MGC40069 | 1.025637 |
| MGC42105 | -1.26798 |
| MGC42367 | -2.88637 |
| MGC4677 | 1.064947 |
| MGLL | -1.61936 |
| MGP | -2.59575 |
| MGST1 | -1.31538 |
| MGST2 | -1.42291 |
| MICALL1 | -1.08874 |
| MICALL2 | -1.05155 |
| MICB | 1.130618 |
| MID1IP1 | -1.34668 |
| MID2 | -1.15945 |
| MIER1 | -1.05468 |
| MIMT1 | -1.1019 |
| MIOS | -1.27875 |
| MIPEP | -1.1173 |
| MIR1207 | 1.335478 |
| MIR205 | -1.00728 |
| MIR320B2 | 1.728949 |
| MIR567 | 1.055388 |
| MIR648 | 1.119428 |
| MKLN1 | -1.26594 |
| MKNK1 | 2.205275 |
| MKRN3 | -1.05859 |
| MLEC | -1.35137 |
| MLF1IP | -1.30709 |
| MLF2 | -1.19573 |
| MLH1 | -1.29293 |
| MLL2 | 1.083779 |
| MLL4 | -1.20582 |
| MLLT10 | -1.12633 |
| MLLT11 | -1.17797 |
| MLPH | -1.05449 |
| MMAB | -1.26152 |
| MMP1 | 3.440196 |
| MMP10 | 3.583705 |
| MMP11 | 2.839112 |
| MMP12 | 5.28905 |
| MMP13 | 3.333813 |
| MMP25 | 1.490714 |
| MMP3 | 2.598135 |
| MMP7 | 1.075981 |
| MMP9 | 3.106983 |
| MMRN1 | -1.36873 |
| MND1 | 1.120843 |
| MOBKL2A | 1.479654 |
| MOBKL3 | -1.26155 |
| MOCS1 | -1.20605 |
| MOSC1 | -1.38894 |
| MOSPD3 | -1.40602 |
| MOXD1 | -1.08879 |
| MPHOSPH10 | -1.09206 |
| MPP4 | -1.44288 |
| MPP7 | -2.03632 |
| MPRIP | -1.3246 |
| MPZL2 | -1.59914 |
| MRPL1 | -1.27755 |
| MRPL11 | -1.07457 |
| MRPL18 | -1.05253 |
| MRPL21 | -1.69817 |
| MRPL41 | -1.01182 |
| MRPL46 | -1.66582 |
| MRPL48 | -2.00751 |
| MRPL49 | -1.42198 |
| MRPL50 | -1.14985 |
| MRPL53 | -1.50262 |
| MRPS10 | -1.00448 |
| MRPS12 | -1.20445 |
| MRPS24 | -1.06411 |
| MS4A4A | 1.5463 |
| MSLN | 2.47913 |
| MSRA | -1.99773 |
| MSRB3 | -1.14932 |
| MT1H | -1.18186 |
| MTA3 | -1.16118 |
| MTERFD1 | -1.16956 |
| MTHFD2 | 1.247511 |
| MTHFS | -1.51395 |
| MTMR14 | 1.23388 |
| MUC15 | -1.9229 |
| MUC16 | 2.494813 |
| MUC20 | -1.26832 |
| MUC21 | -4.05826 |
| MUC4 | 1.699083 |
| MUCL1 | 1.790212 |
| MUDENG | -1.02349 |
| MVK | -1.15371 |
| MVP | 1.077695 |
| MXD1 | -1.08111 |
| MXI1 | -1.31376 |
| MYBPC1 | 2.240802 |
| MYBPC2 | 2.543892 |
| MYBPH | 3.021152 |
| MYC | -1.04919 |
| MYCBP2 | -1.55396 |
| MYH11 | -1.06833 |
| MYH13 | -1.4725 |
| MYH14 | -1.90306 |
| MYH2 | 2.391472 |
| MYH3 | 1.096086 |
| MYH6 | 2.799608 |
| MYH7 | 2.319227 |
| MYH8 | 1.317201 |
| MYL1 | 1.883217 |
| MYL2 | 2.330215 |
| MYL3 | 1.877003 |
| MYL9 | 1.399478 |
| MYLIP | -2.61118 |
| MYLK2 | 1.525928 |
| MYO1B | 1.153628 |
| MYO1D | -1.00918 |
| MYO5B | -1.92572 |
| MYO5C | -3.56169 |
| MYO6 | -1.48188 |
| MYOC | -1.05615 |
| MYPN | 2.234219 |
| MYRIP | -1.32352 |
| MYST3 | -1.59245 |
| N4BP2L1 | -1.15757 |
| NACC2 | 1.117691 |
| NADSYN1 | -1.52856 |
| NAGS | 1.258799 |
| NAP1L4 | -1.09624 |
| NAP1L5 | -1.07498 |
| NARG1 | -1.07099 |
| NARG1L | -1.20986 |
| NAT8B | 2.662776 |
| NBL1 | -1.68144 |
| NBN | 1.074041 |
| NBPF11 | 1.525689 |
| NCAPD2 | 1.473799 |
| NCCRP1 | -1.09663 |
| NCKAP1L | 1.604362 |
| NCOA1 | -1.99628 |
| NCOA5 | -1.17354 |
| NCRNA00152 | 1.108315 |
| NCRNA00219 | -1.80666 |
| NDRG3 | 1.068209 |
| NDUFA2 | -2.4005 |
| NDUFA6 | -1.60374 |
| NDUFA7 | -1.17772 |
| NDUFA9 | -1.61514 |
| NDUFB1 | 1.585377 |
| NDUFB11 | -1.05962 |
| NDUFB2 | -1.70393 |
| NDUFC1 | -2.2995 |
| NDUFV3 | -1.29491 |
| NEBL | -1.25186 |
| NECAP1 | -1.30874 |
| NECAP2 | -1.11884 |
| NEK11 | -1.03372 |
| NEK2 | 1.24318 |
| NEK6 | 1.836464 |
| NEK8 | 1.47568 |
| NEU4 | 1.949412 |
| NF1 | 1.061617 |
| NF2 | 1.09325 |
| NFATC2IP | -1.63788 |
| NFATC3 | 1.47332 |
| NFATC4 | -2.09464 |
| NFE2 | 3.23349 |
| NFE2L3 | 1.190089 |
| NFIA | -1.37817 |
| NFIB | -1.0146 |
| NFIX | -1.36891 |
| NFKB2 | 1.392754 |
| NFKBIE | 1.131197 |
| NGB | 1.631728 |
| NGDN | 1.012897 |
| NGEF | -2.25584 |
| NGLY1 | -1.69229 |
| NGRN | -1.4088 |
| NHLRC3 | -1.24155 |
| NHP2 | -1.08592 |
| NIPAL1 | -1.05632 |
| NKG7 | 1.385836 |
| NLF2 | 1.508753 |
| NLRC3 | 1.010298 |
| NLRP1 | 1.081673 |
| NMU | -2.0906 |
| NNAT | -1.33273 |
| NNMT | 1.121561 |
| NOB1 | -1.04034 |
| NOC2L | 1.092101 |
| NOL11 | -1.21347 |
| NOL3 | -1.00116 |
| NOMO2 | -1.04717 |
| NOMO3 | 1.001004 |
| NONO | -1.35221 |
| NOS1AP | 1.303531 |
| NOTCH2 | -1.59275 |
| NOV | -1.4197 |
| NOX4 | 1.712675 |
| NOXA1 | -1.64329 |
| NPL | 1.468711 |
| NPLOC4 | -1.19195 |
| NPR3 | -1.04393 |
| NPTN | -1.33621 |
| NPTX2 | -1.12099 |
| NPW | 1.152913 |
| NR3C1 | -1.35174 |
| NR4A3 | 1.038879 |
| NRD1 | -1.0227 |
| NRG1 | 2.709531 |
| NRIP3 | 1.44555 |
| NRP1 | -1.1094 |
| NRP2 | 1.058875 |
| NT5DC2 | -2.47796 |
| NTF3 | -1.58667 |
| NTHL1 | -1.38631 |
| NTM | -1.43697 |
| NTN5 | -1.41531 |
| NTSR1 | 2.015829 |
| NUAK2 | -1.93362 |
| NUCB2 | -1.60511 |
| NUDCD3 | -1.0643 |
| NUDT16 | -1.29323 |
| NUDT16P | 1.04544 |
| NUDT2 | -1.20116 |
| NUDT5 | -1.06268 |
| NUFIP1 | -1.13024 |
| NUMA1 | -1.95925 |
| NUP133 | -1.42087 |
| NUP62 | 1.381368 |
| NUP93 | -1.34679 |
| NXF1 | -1.66869 |
| NYNRIN | -1.52033 |
| OAF | -1.12463 |
| OAS1 | 1.768393 |
| OAS2 | 2.11635 |
| OAS3 | 2.36979 |
| OASL | 2.118528 |
| OAT | -1.04935 |
| OAZ2 | -1.07963 |
| OBFC1 | -1.78173 |
| OCA2 | -1.10981 |
| OCLN | 1.282792 |
| ODC1 | 1.370641 |
| ODF2 | 1.06585 |
| ODF2L | 1.019368 |
| ODZ3 | 1.266993 |
| ODZ4 | -1.57364 |
| OGFOD1 | -1.15797 |
| OLFM4 | 1.327016 |
| OLFML2A | -2.10446 |
| OLR1 | 1.067774 |
| OPN3 | 1.015248 |
| OPN4 | -1.07018 |
| OR7E37P | -1.49299 |
| OR7E91P | -1.79817 |
| ORAI1 | 1.35868 |
| ORC6L | 1.179569 |
| OSBPL10 | -1.64051 |
| OSBPL11 | -1.30813 |
| OSBPL5 | -1.85 |
| OSBPL6 | -1.67163 |
| OSBPL7 | 2.045174 |
| OSBPL8 | -1.11936 |
| OSTalpha | -1.77287 |
| OSTF1 | -1.08262 |
| OTOF | 2.563704 |
| OTX1 | -1.33222 |
| OXCT2 | 1.307193 |
| OXSR1 | -2.15239 |
| P11 | -1.05746 |
| P2RX1 | 1.547542 |
| P2RX2 | 1.707269 |
| P2RX6 | 1.87769 |
| P2RY10 | 1.882853 |
| P2RY6 | 1.337981 |
| PAAF1 | -1.51141 |
| PABPC4L | -1.27324 |
| PACS1 | -1.02276 |
| PACSIN1 | 1.975919 |
| PADI4 | 2.169962 |
| PAEP | 1.254266 |
| PAFAH1B1 | -1.30302 |
| PAG1 | 1.028703 |
| PAICS | -1.89177 |
| PAIP2 | -1.48099 |
| PAK2 | -1.18698 |
| PAK6 | -1.51366 |
| PALLD | -1.95374 |
| PALMD | -1.71896 |
| PAN2 | -1.44991 |
| PANK4 | -1.39278 |
| PANX2 | 1.092929 |
| PARD3 | -1.59015 |
| PARD6G | -1.6067 |
| PARM1 | -1.73679 |
| PARP10 | 1.140113 |
| PARP2 | -1.26174 |
| PART1 | -1.10721 |
| PARVA | -1.05017 |
| PARVG | 1.387249 |
| PAX9 | -1.79558 |
| PBX3 | -1.18208 |
| PC | -1.07843 |
| PCBD2 | -1.48134 |
| PCBP1 | -1.77736 |
| PCBP2 | -1.32809 |
| PCCA | -1.20778 |
| PCCB | -1.05729 |
| PCDH17 | 1.892941 |
| PCDH21 | -1.29515 |
| PCK2 | -1.27663 |
| PCNP | -1.49103 |
| PCOLCE2 | -3.31311 |
| PCSK1 | 2.335708 |
| PCYOX1 | -1.2611 |
| PDCD2L | -1.19931 |
| PDCL2 | -1.37942 |
| PDE12 | -1.4158 |
| PDE4B | 1.293834 |
| PDE4DIP | 1.510824 |
| PDE6A | -1.59026 |
| PDE6G | 1.447188 |
| PDE7A | 1.607621 |
| PDE7B | -1.18026 |
| PDE8B | -1.49326 |
| PDGFRL | -1.58175 |
| PDLIM7 | 2.19144 |
| PDPN | 1.026039 |
| PDSS2 | -1.31683 |
| PEAR1 | 1.156449 |
| PEF1 | -1.08024 |
| PEG3 | -2.25482 |
| PELI1 | -1.22039 |
| PENK | -1.88851 |
| PEPD | -1.61445 |
| PERP | -1.17649 |
| PEX13 | -1.05636 |
| PEX26 | 1.021344 |
| PFDN5 | -1.20705 |
| PFKFB2 | -1.00267 |
| PFKP | -2.2012 |
| PGAP3 | -1.46984 |
| PGD | -1.99893 |
| PGLYRP2 | 2.03321 |
| PGLYRP3 | -1.04054 |
| PGM1 | -1.11032 |
| PGM2 | -2.09653 |
| PGM3 | -1.08497 |
| PGM5 | -1.97773 |
| PHACTR2 | -1.2373 |
| PHACTR4 | -1.92698 |
| PHC2 | 1.991386 |
| PHF19 | -1.14409 |
| PHF20L1 | -1.28818 |
| PHF6 | 1.121445 |
| PHKB | -1.68689 |
| PHLDB2 | 1.265403 |
| PHLDB3 | -1.52917 |
| PHLPP1 | -2.21738 |
| PHRF1 | 1.566256 |
| PI15 | 1.395437 |
| PIGN | -1.40415 |
| PIGY | -1.70088 |
| PIGZ | -1.11388 |
| PIK3C2B | -1.15154 |
| PIK3R1 | -1.22417 |
| PILRA | 1.352061 |
| PIM1 | -1.26624 |
| PIM2 | 1.092688 |
| PITPNA | -2.28786 |
| PITX2 | -1.20102 |
| PKM2 | 1.334607 |
| PKN2 | -1.15118 |
| PKN3 | -1.23421 |
| PKP1 | -1.41471 |
| PLA2G3 | -1.24209 |
| PLA2G4A | -2.72327 |
| PLA2G7 | 1.653325 |
| PLAG1 | 1.060852 |
| PLAGL1 | -1.55385 |
| PLAGL2 | -1.1743 |
| PLAUR | 1.052159 |
| PLCXD1 | -1.27302 |
| PLD5 | -1.36281 |
| PLD6 | 1.272383 |
| PLEC1 | 1.149759 |
| PLEK | 1.018449 |
| PLEKHA1 | -1.08314 |
| PLEKHB2 | 1.421773 |
| PLEKHG4 | -1.7301 |
| PLEKHG6 | -2.36539 |
| PLEKHH2 | -1.40858 |
| PLEKHM1 | -1.45531 |
| PLLP | -1.07512 |
| PLS1 | -1.81076 |
| PLSCR1 | 1.376636 |
| PLTP | 1.011907 |
| PMF1 | -1.43638 |
| PML | 1.124741 |
| PMP2 | -2.43881 |
| PMP22 | 1.117982 |
| PNCK | 1.058746 |
| PNMA3 | 1.767293 |
| PODXL | -1.67081 |
| PODXL2 | -1.74223 |
| POFUT1 | 1.156321 |
| POFUT2 | -1.04798 |
| POLD2 | -2.32489 |
| POLR1D | -1.89477 |
| POLR2I | -2.00457 |
| POLR2L | -1.42719 |
| POLR3G | -1.36915 |
| POLR3H | -1.12957 |
| POMT2 | -1.22891 |
| POP4 | -1.07041 |
| POP5 | -1.40166 |
| POPDC3 | 1.682707 |
| POSTN | 2.665748 |
| POU2AF1 | 1.142677 |
| PPA1 | -1.10014 |
| PPA2 | -1.61308 |
| PPAP2A | -1.2898 |
| PPAP2B | -1.69848 |
| PPARG | -1.33299 |
| PPARGC1A | -1.5652 |
| PPIL6 | -1.78743 |
| PPL | -2.25652 |
| PPM1E | -1.03899 |
| PPOX | -1.20322 |
| PPP1CB | -1.85235 |
| PPP1CC | -1.14532 |
| PPP1R10 | -2.41906 |
| PPP1R12B | 1.217673 |
| PPP1R3C | -1.5569 |
| PPP1R9B | 1.300636 |
| PPP2CB | -1.65852 |
| PPP2R2C | -1.18238 |
| PPP2R5A | -2.14447 |
| PPP3CA | -1.02955 |
| PPP4R4 | 1.444156 |
| PPT2 | -1.1202 |
| PPWD1 | -1.0492 |
| PQLC3 | -1.38736 |
| PRDM2 | -1.0636 |
| PRDX1 | -1.24723 |
| PRDX6 | -1.19622 |
| PREPL | -1.17859 |
| PRIC285 | 1.011074 |
| PRKAB1 | -1.47065 |
| PRKACA | 1.004438 |
| PRKAR1A | 1.475133 |
| PRKCH | -1.447 |
| PRKCQ | 1.387395 |
| PRKD1 | -1.11672 |
| PRKRIR | -1.52106 |
| PRKX | -1.38144 |
| PRNP | 1.721843 |
| PROC | 1.78371 |
| PRODH | -1.70256 |
| PROM1 | 2.024343 |
| PROS1 | -1.09349 |
| ProSAPiP1 | -1.08856 |
| PROSC | -1.64125 |
| PRPF3 | -1.10398 |
| PRPF31 | -1.8319 |
| PRPF4 | -1.08682 |
| PRPS1 | -1.08011 |
| PRPS2 | -1.2055 |
| PRPSAP1 | -1.51259 |
| PRRC1 | -1.25401 |
| PRRG1 | -1.51969 |
| PRRX1 | -1.75005 |
| PRSS3 | -2.28863 |
| PSAT1 | -1.46947 |
| PSCD2 | -1.17273 |
| PSCD4 | 1.201896 |
| PSCDBP | 2.007232 |
| PSMA3 | -1.09542 |
| PSMB5 | -1.17946 |
| PSMB8 | 1.580024 |
| PSMB9 | 1.453542 |
| PSMC3IP | 1.151294 |
| PSMC4 | 1.03296 |
| PSMD12 | -1.01315 |
| PSMD9 | -1.47418 |
| PSME3 | -1.5417 |
| PSMG1 | -1.21751 |
| PTCD2 | -1.29576 |
| PTCH1 | -1.14442 |
| PTCRA | 2.354807 |
| PTDSS1 | -1.90438 |
| PTGES2 | 1.392494 |
| PTGES3 | -1.03661 |
| PTGIS | -1.42617 |
| PTGR1 | -1.37922 |
| PTGS1 | -1.15735 |
| PTHLH | 2.564133 |
| PTK2 | 1.637221 |
| PTK6 | -2.15308 |
| PTP4A3 | 1.029118 |
| PTPDC1 | 1.04661 |
| PTPN13 | -1.41438 |
| PTPN22 | 1.081815 |
| PTPRA | -1.705 |
| PTPRC | 1.46389 |
| PTPRCAP | 2.043352 |
| PTPRO | 1.774777 |
| PTTG3P | -1.03639 |
| PUM1 | -1.48674 |
| PUM2 | -1.60411 |
| PURA | -1.22394 |
| PURB | -1.16007 |
| PUS1 | 1.320094 |
| PUS7L | -1.53751 |
| PVRIG | 1.381681 |
| PVRL4 | -2.16078 |
| PWP1 | -1.41214 |
| PXDN | 1.490799 |
| PYHIN1 | 1.176717 |
| QPCTL | 1.764991 |
| QPRT | 1.610096 |
| QSOX2 | -1.16045 |
| RAB11A | -1.23002 |
| RAB11B | 1.597642 |
| RAB11FIP2 | -1.45657 |
| RAB11FIP3 | 1.227629 |
| RAB19 | 1.176311 |
| RAB1A | -1.01686 |
| RAB22A | -1.94106 |
| RAB23 | 1.01214 |
| RAB24 | -1.33898 |
| RAB25 | -1.01498 |
| RAB27A | 1.22667 |
| RAB36 | -1.28267 |
| RAB38 | -1.22848 |
| RAB3IP | 1.168077 |
| RAB43 | 1.323737 |
| RAB4A | -1.07767 |
| RAB4B | 1.454318 |
| RAB6B | -1.50562 |
| RAB7B | -1.03332 |
| RAB7L1 | 1.766952 |
| RAB8A | -1.8368 |
| RABGAP1 | -1.20527 |
| RAC2 | 1.465466 |
| RAD17 | 1.111974 |
| RAD51C | 1.027761 |
| RAE1 | -1.19466 |
| RAET1G | -2.19366 |
| RAF1 | -1.58446 |
| RAG1 | 1.024944 |
| RALGPS1 | -1.29669 |
| RALY | 1.133229 |
| RANBP2 | -1.04674 |
| RANGRF | 1.014085 |
| RAP2A | -2.3584 |
| RAPGEF2 | -1.69975 |
| RAPGEF5 | -2.31504 |
| RAPH1 | -1.97743 |
| RARRES1 | 1.134104 |
| RASAL2 | -1.85837 |
| RASD2 | 2.018365 |
| RASSF4 | 1.028013 |
| RASSF5 | -1.03825 |
| RASSF9 | -2.13527 |
| RAVER2 | -1.6716 |
| RBBP6 | -1.09405 |
| RBM15B | -1.11447 |
| RBM3 | 1.069407 |
| RBM4 | 1.448555 |
| RBM41 | 1.065552 |
| RBM47 | -1.5254 |
| RBM4B | -1.48152 |
| RBM5 | -1.63906 |
| RBMS1 | -1.75816 |
| RBMS2 | 1.398009 |
| RBP1 | 2.014767 |
| RBP7 | -2.07516 |
| RBPMS | -1.33381 |
| RBX1 | -1.09538 |
| RCCD1 | -1.24464 |
| RCOR3 | -1.09831 |
| RDH12 | -2.56491 |
| RDH13 | -1.39702 |
| REC8 | 1.164303 |
| REEP1 | -1.94675 |
| REEP6 | -1.38254 |
| RELL1 | -2.12287 |
| RERE | -2.2972 |
| RERG | -1.01514 |
| REV1 | -1.42154 |
| REXO2 | -1.29733 |
| RFC3 | -1.41101 |
| RFNG | -1.00828 |
| RFWD2 | 1.056332 |
| RGL2 | -1.23926 |
| RGS5 | -1.33245 |
| RHCG | -1.96247 |
| RHOD | -1.56864 |
| RHPN2 | 1.853758 |
| RICS | -1.17062 |
| RIT1 | -1.04256 |
| RLTPR | 1.661401 |
| RMND5A | -1.69335 |
| RNASE4 | -1.32138 |
| RNASEH1 | -1.38671 |
| RNASEK | -1.38048 |
| RND3 | -1.44753 |
| RNF160 | -1.13864 |
| RNF175 | -1.01715 |
| RNF185 | -1.83189 |
| RNF34 | 1.116495 |
| RNF38 | -1.20629 |
| RNF39 | -1.1394 |
| RNF43 | -1.36833 |
| RNH1 | -1.89022 |
| RNPS1 | 1.012421 |
| RNU6ATAC | 1.22407 |
| ROBO2 | -1.55437 |
| RORA | -1.30287 |
| RORC | -1.22184 |
| RPA1 | -1.21953 |
| RPAIN | -1.75989 |
| RPL17 | 1.167595 |
| RPL22 | -1.30106 |
| RPL23AP13 | -1.04037 |
| RPL26L1 | -1.00479 |
| RPL29 | -1.06032 |
| RPL34 | -1.394 |
| RPL39 | -1.34193 |
| RPL6 | 1.636603 |
| RPLP1 | -1.81137 |
| RPRD2 | -1.56071 |
| RPRM | -1.43841 |
| RPRML | -1.04188 |
| RPS12 | -1.01713 |
| RPS13 | -1.28216 |
| RPS23 | -1.18353 |
| RPS27 | -1.33522 |
| RPS27L | -1.75635 |
| RPS5 | -1.0513 |
| RPS6KA5 | -1.3203 |
| RRAD | -1.32363 |
| RRAGA | -1.80615 |
| RREB1 | -1.18506 |
| RRP12 | -2.09527 |
| RSAD1 | -1.96807 |
| RSAD2 | 2.280932 |
| RSC1A1 | -1.19625 |
| RSPO1 | -1.71422 |
| RTN3 | -1.8175 |
| RTN4 | -1.08414 |
| RTP3 | 1.095099 |
| RUFY1 | -1.75637 |
| RUFY4 | 2.140279 |
| RUNX1 | 3.463865 |
| RUNX3 | 1.368196 |
| RWDD1 | -1.035 |
| RXRG | 1.082257 |
| RYR1 | 1.081429 |
| S100A1 | 1.127018 |
| S100A7A | 2.849318 |
| S1PR3 | -1.14629 |
| SAA1 | 2.349609 |
| SAA2 | 4.334303 |
| SALL2 | -1.27955 |
| SAMD14 | 1.316494 |
| SAMD5 | -2.16138 |
| SAMM50 | -1.42565 |
| SAMSN1 | 1.246476 |
| SAP130 | -1.26399 |
| SAP18 | -1.05665 |
| SASH1 | -2.42168 |
| SAT2 | -1.54056 |
| SATB2 | -1.33626 |
| SATL1 | 1.226668 |
| SBNO1 | 1.172778 |
| SC4MOL | -1.69649 |
| SCAF1 | -1.3941 |
| SCAND2 | 1.062542 |
| SCARA3 | -1.94607 |
| SCARA5 | -3.6943 |
| SCARB2 | -1.01318 |
| SCARNA13 | 1.191629 |
| SCARNA2 | 1.132731 |
| SCARNA21 | 1.110204 |
| SCARNA5 | 1.187501 |
| SCARNA6 | 1.300574 |
| SCARNA7 | 1.066642 |
| SCARNA8 | 1.318696 |
| SCD | -2.15059 |
| SCD5 | 1.491299 |
| SCEL | -1.27298 |
| SCG5 | 1.875592 |
| SCGB2A1 | -3.1789 |
| SCIN | -1.30428 |
| SCLY | -1.2771 |
| SCN11A | -1.0166 |
| SCNN1B | -2.19501 |
| SCYL1 | -1.41981 |
| SDC3 | 1.156912 |
| SDCBP2 | -2.06848 |
| SDHAF1 | -1.39217 |
| SDHD | -1.30049 |
| SDK2 | 1.092209 |
| SDPR | -1.17202 |
| SDS | 3.257197 |
| SEC11C | -1.23027 |
| SEC13 | -1.56679 |
| SEC14L1 | -1.20898 |
| SEC16A | -1.09649 |
| SEC23B | -1.25543 |
| SEC61G | -1.46378 |
| SECISBP2L | -1.5944 |
| SEL1L | -1.1834 |
| SEL1L3 | 1.443418 |
| Selenoprotein 15 | -1.4 |
| SELL | 1.513115 |
| SELPLG | 1.470275 |
| SELS | -1.12694 |
| SELT | 1.066425 |
| SEMA4A | -1.22501 |
| SEMA4B | -1.05955 |
| SEMA6B | 2.019429 |
| SEMA7A | 1.570888 |
| SENP8 | -1.08539 |
| SERAC1 | -1.14302 |
| SERGEF | -1.68485 |
| SERINC1 | -1.14893 |
| SERINC5 | -1.26924 |
| SERP2 | -2.19342 |
| SERPINA1 | 2.105881 |
| SERPINA3 | 1.713264 |
| SERPINB4 | 1.567144 |
| SERPINB9 | 1.020672 |
| SERPINC1 | -1.2218 |
| SERPINI2 | -1.11912 |
| SERTAD2 | -1.0648 |
| SERTAD3 | 1.027779 |
| SERTAD4 | -1.06827 |
| SESN1 | -1.45339 |
| SET | -1.48385 |
| SETMAR | -1.53899 |
| SEZ6L2 | 1.925468 |
| SF1 | 1.108348 |
| SF3B5 | -1.8931 |
| SFMBT2 | 2.150099 |
| SFRP2 | -1.59867 |
| SFRP4 | -1.07589 |
| SFRS14 | 1.086464 |
| SFRS18 | -1.63285 |
| SFRS2 | -1.10022 |
| SFRS4 | -1.30621 |
| SFRS9 | -1.52836 |
| SFT2D1 | -1.29166 |
| SFT2D2 | 1.01516 |
| SFT2D3 | -1.36334 |
| SFTA2 | -2.18089 |
| SFTPD | -1.29138 |
| SFXN3 | 1.341308 |
| SFXN5 | -1.19025 |
| SGCG | -1.94678 |
| SGEF | -1.79724 |
| SGK | -1.44784 |
| SH2D3C | -1.24154 |
| SH3BGRL2 | -1.73314 |
| SH3BP1 | -1.19206 |
| SH3D19 | -1.3375 |
| SH3GL2 | -1.34921 |
| SH3GL3 | -3.39223 |
| SH3KBP1 | 1.509338 |
| SHANK3 | -1.51895 |
| SHBG | 1.002794 |
| SHC1 | 1.263499 |
| SHD | 4.326428 |
| SHE | -1.41224 |
| SHMT1 | -1.17063 |
| SHPRH | -1.95919 |
| SHRM | -1.43548 |
| SHROOM3 | -3.05404 |
| SIGLEC10 | 1.208887 |
| SIGLEC14 | 1.873753 |
| SIGLEC16 | 1.040674 |
| SIK3 | -1.03443 |
| SILV | -1.30515 |
| SIM2 | -1.97495 |
| SIN3A | -1.36263 |
| SIN3B | -1.1041 |
| SIRT1 | -1.80115 |
| SIRT6 | -1.49414 |
| SKAP1 | 2.450396 |
| SKAP2 | 2.222743 |
| SKIL | 1.442145 |
| SKP1A | 1.003216 |
| SLA | 1.562443 |
| SLA2 | 1.373606 |
| SLAMF6 | 1.163725 |
| SLAMF8 | 2.264897 |
| SLC10A3 | -1.40207 |
| SLC10A7 | -1.30132 |
| SLC11A1 | 1.740484 |
| SLC12A2 | -1.57841 |
| SLC12A3 | 1.043188 |
| SLC13A4 | -1.09448 |
| SLC13A5 | 1.752888 |
| SLC15A2 | 1.851172 |
| SLC15A3 | 1.634767 |
| SLC15A4 | -1.53054 |
| SLC16A10 | 2.200876 |
| SLC16A2 | -1.07169 |
| SLC16A3 | 1.357334 |
| SLC16A8 | -1.02938 |
| SLC17A7 | -1.05485 |
| SLC19A1 | 1.093532 |
| SLC19A3 | 1.197106 |
| SLC1A4 | -1.22544 |
| SLC22A17 | -1.08468 |
| SLC22A18 | -1.64474 |
| SLC22A23 | -1.26197 |
| SLC22A5 | -1.55378 |
| SLC24A3 | -1.30076 |
| SLC25A1 | 1.131206 |
| SLC25A12 | -1.55882 |
| SLC25A17 | -1.12348 |
| SLC25A25 | -1.88607 |
| SLC25A29 | -1.12146 |
| SLC25A3 | -1.19525 |
| SLC25A35 | -1.11408 |
| SLC25A43 | -1.31927 |
| SLC25A46 | -1.09888 |
| SLC25A5 | -1.4584 |
| SLC27A1 | -1.33682 |
| SLC29A4 | 1.270562 |
| SLC2A10 | -1.93307 |
| SLC2A12 | -1.18563 |
| SLC2A3 | 1.46811 |
| SLC2A6 | 1.386909 |
| SLC30A1 | 1.269569 |
| SLC31A1 | 1.058807 |
| SLC34A3 | -1.35122 |
| SLC35A3 | -1.26037 |
| SLC35A4 | -1.93423 |
| SLC35B1 | -1.36646 |
| SLC35C1 | -1.51483 |
| SLC35C2 | 1.015965 |
| SLC35E1 | -1.19878 |
| SLC35F2 | -1.49295 |
| SLC38A10 | 1.471368 |
| SLC38A3 | -1.47242 |
| SLC39A2 | -1.5364 |
| SLC39A3 | 1.05226 |
| SLC43A2 | 1.434788 |
| SLC4A8 | 2.021741 |
| SLC6A15 | -1.07558 |
| SLC6A2 | 1.400543 |
| SLC7A11 | -1.31088 |
| SLC7A2 | -1.56349 |
| SLC7A5P1 | 1.684505 |
| SLC7A6 | 1.28521 |
| SLC7A6OS | 1.443441 |
| SLC7A8 | 1.573046 |
| SLC8A2 | -1.22984 |
| SLC9A6 | -1.07353 |
| SLC9A8 | -1.27297 |
| SLCO1B3 | 1.177228 |
| SLFN5 | 1.347179 |
| SLIT2 | -1.40548 |
| SLIT3 | -1.85381 |
| SLITRK5 | -1.94987 |
| SLK | -1.55712 |
| SLMAP | -1.34754 |
| SLN | 1.084625 |
| SLTM | -1.21757 |
| SLURP1 | -1.8325 |
| SMAD5 | -1.3481 |
| SMAGP | -2.60657 |
| SMAP1 | -1.22574 |
| SMARCAL1 | -1.29191 |
| SMARCC1 | -1.95391 |
| SMARCD3 | -1.02847 |
| SMC3 | -1.19289 |
| SMPDL3A | -1.48741 |
| SMPDL3B | 2.36693 |
| SMTN | 2.625697 |
| SNAI2 | 1.120266 |
| SNAP25 | 1.57345 |
| SNAP29 | -1.54569 |
| SNCA | -1.26762 |
| SNHG5 | -1.50709 |
| SNHG9 | -1.78408 |
| SNORA10 | 1.048585 |
| SNORA11 | 1.368728 |
| SNORA11D | 1.240888 |
| SNORA11E | 1.497489 |
| SNORA14B | 1.149565 |
| SNORA21 | 1.020738 |
| SNORA34 | 1.241263 |
| SNORA38 | 1.41653 |
| SNORA39 | 1.416398 |
| SNORA48 | 1.021616 |
| SNORA54 | 1.244089 |
| SNORA55 | 1.177712 |
| SNORA71A | 1.653788 |
| SNORA71C | 2.68232 |
| SNORA74A | 1.653265 |
| SNORA77 | 1.022247 |
| SNORA79 | -1.08348 |
| SNORA81 | 1.129031 |
| SNORD11B | 1.103687 |
| SNORD12 | 1.153728 |
| SNORD1A | 1.285796 |
| SNORD30 | 1.042731 |
| SNORD33 | 1.002003 |
| SNORD35A | 1.253693 |
| SNORD36C | 1.1306 |
| SNORD62B | 1.331467 |
| SNORD66 | 1.158128 |
| SNORD67 | 1.24864 |
| SNORD83B | 1.108081 |
| SNORD89 | 1.196481 |
| SNORD93 | 1.482865 |
| SNORD95 | 1.349598 |
| SNRNP48 | -1.78502 |
| SNRPF | -1.62077 |
| SNTB2 | 1.224817 |
| SNX10 | 1.22946 |
| SNX14 | -1.56726 |
| SNX15 | -1.33749 |
| SNX16 | -1.18715 |
| SNX2 | -1.48 |
| SNX21 | -1.33359 |
| SNX22 | 1.01248 |
| SNX24 | -1.28709 |
| SNX3 | -1.02789 |
| SNX9 | 1.16368 |
| SOCS5 | 1.077483 |
| SOCS7 | 1.075993 |
| SOD2 | 1.567452 |
| SORBS2 | -1.27568 |
| SORBS3 | -1.13541 |
| SORT1 | -2.13551 |
| SOX11 | 2.477809 |
| SOX2 | -1.18187 |
| SOX7 | -1.16029 |
| SP100 | 1.46746 |
| SP110 | 1.355345 |
| SP140 | 2.157983 |
| SP140L | 1.228429 |
| SPANXB1 | 1.573431 |
| SPARC | -2.04911 |
| SPARCL1 | -1.51925 |
| SPATA18 | -3.04417 |
| SPATS2 | 1.373837 |
| SPEN | -1.17909 |
| SPG11 | -1.22296 |
| SPHAR | -1.38583 |
| SPI1 | 1.571133 |
| SPIB | 1.127026 |
| SPINK5 | -1.84024 |
| SPINK6 | -1.33208 |
| SPINK7 | -3.49136 |
| SPINK9 | -1.26437 |
| SPINT2 | -1.81079 |
| SPN | 1.605975 |
| SPNS2 | -1.36849 |
| SPOCD1 | 3.102213 |
| SPOCK2 | 1.571871 |
| SPON1 | -1.57254 |
| SPP1 | 2.800107 |
| SPPL2A | -1.16348 |
| SPRR2C | -2.51992 |
| SPRR2D | -1.80923 |
| SPRR2F | -1.85867 |
| SPRR2G | -1.29081 |
| SPSB1 | -1.14245 |
| SPTB | 2.30203 |
| SPTBN2 | -1.40852 |
| SPTLC1 | -1.39225 |
| SR140 | 1.211008 |
| SRD5A1 | -1.51135 |
| SREBF1 | -1.08599 |
| SRGAP1 | 1.071985 |
| SRGN | 2.03938 |
| SRP68 | -1.07105 |
| SRPK2 | 1.162533 |
| SRPX | -1.38662 |
| SRXN1 | -1.44571 |
| ST3GAL2 | 1.887115 |
| ST3GAL4 | -1.23295 |
| ST5 | 1.303254 |
| ST7 | -1.54326 |
| STAB2 | -1.22149 |
| STAM | -1.13939 |
| STAP2 | -2.11637 |
| STARD13 | 1.539033 |
| STARD3NL | 1.007918 |
| STAT1 | 1.513981 |
| STAT5B | -1.03402 |
| STATH | -2.14495 |
| STAU2 | -1.58512 |
| STK17B | 1.172574 |
| STK19 | -2.04583 |
| STK33 | 1.095282 |
| STK38 | -1.20416 |
| STK39 | -1.37724 |
| STOM | 1.204012 |
| STRADB | -1.54177 |
| STRBP | -1.17836 |
| STX17 | -1.058 |
| STX2 | -1.11355 |
| STXBP5 | -1.43229 |
| SULF2 | 1.428146 |
| SULT1A3 | 1.228673 |
| SUMF1 | -1.48146 |
| SUPT16H | -1.51213 |
| SUPT4H1 | -1.7307 |
| SUPT5H | -1.00841 |
| SURF1 | -1.008 |
| SUSD1 | 1.247489 |
| SUSD4 | -1.03527 |
| SVEP1 | -1.40718 |
| SVIL | 1.127095 |
| SYNC | 1.56673 |
| SYNGR1 | -2.33359 |
| SYNGR4 | 1.041032 |
| SYNPO2 | 1.440416 |
| SYP | -1.17107 |
| SYT7 | 1.156978 |
| TACC1 | -1.4963 |
| TADA1L | -1.24774 |
| TAF1 | 1.040224 |
| TAF1D | 1.088443 |
| TAF5 | -1.02609 |
| TAGAP | 1.858925 |
| TAGLN3 | 1.280623 |
| TALDO1 | -2.50508 |
| TAOK1 | -1.07974 |
| TAP1 | 1.17697 |
| TAP2 | 1.44193 |
| TARBP1 | -1.96738 |
| TARS | -1.20484 |
| TAS1R1 | 1.775795 |
| TBC1D10C | 1.466104 |
| TBC1D14 | -1.15267 |
| TBC1D15 | -1.33732 |
| TBC1D2 | -1.00997 |
| TBC1D22B | -1.61728 |
| TBC1D7 | -1.06803 |
| TBC1D9B | -2.06773 |
| TBRG4 | -1.16814 |
| TBX1 | 1.173048 |
| TCAP | 1.470155 |
| TCEAL3 | -1.08722 |
| TCEB1 | -1.02586 |
| TCIRG1 | 1.112869 |
| TCOF1 | 2.254053 |
| TCP11L2 | -2.15122 |
| TCTEX1D2 | -1.57447 |
| TCTN3 | -2.10975 |
| TDO2 | 1.874577 |
| TEAD4 | 2.120217 |
| TECR | -2.38606 |
| TEK | -1.39946 |
| TEP1 | 1.080109 |
| TERF2IP | -1.15776 |
| TES | 1.067416 |
| TEX101 | -1.92726 |
| TEX11 | 2.215194 |
| TEX2 | -1.48409 |
| TF | -1.98967 |
| TFCP2 | -1.32202 |
| TFDP1 | -1.00718 |
| TFPI | -1.46865 |
| TGFBR2 | -1.99251 |
| TGFBR3 | -1.79432 |
| TGM2 | 3.508707 |
| TGM3 | -1.02289 |
| TGM5 | -2.75751 |
| TH | 1.025119 |
| THBD | -1.53994 |
| THBS4 | -1.93817 |
| THEM2 | -1.13676 |
| THOC7 | -1.18245 |
| THUMPD2 | -1.12382 |
| TIFA | -1.10657 |
| TIGA1 | -1.65946 |
| TIMM23 | -1.30021 |
| TINAGL1 | 1.821019 |
| TJP3 | -1.1933 |
| TKTL1 | 1.043255 |
| TLCD1 | -1.7511 |
| TLN2 | 1.229957 |
| TLR1 | 1.316193 |
| TLR5 | -1.04953 |
| TLX1 | -2.27788 |
| TM2D3 | 1.31251 |
| TM4SF19 | 1.779388 |
| TM7SF2 | -1.02934 |
| TMBIM4 | -1.86887 |
| TMCO3 | -1.14397 |
| TMED10P | -1.78396 |
| TMED2 | -1.58018 |
| TMEFF2 | -1.86603 |
| TMEM106B | -1.22077 |
| TMEM111 | -1.54992 |
| TMEM119 | -1.5747 |
| TMEM120A | -1.14507 |
| TMEM125 | -1.3651 |
| TMEM126A | -1.23515 |
| TMEM128 | -1.25937 |
| TMEM134 | -2.20026 |
| TMEM139 | -1.12585 |
| TMEM143 | -1.6177 |
| TMEM145 | 1.438796 |
| TMEM147 | -1.85669 |
| TMEM156 | 1.505877 |
| TMEM159 | -1.43952 |
| TMEM165 | -1.2254 |
| TMEM168 | -1.4755 |
| TMEM17 | -1.04384 |
| TMEM176A | 1.620826 |
| TMEM176B | 1.530433 |
| TMEM178 | -1.674 |
| TMEM180 | 1.179737 |
| TMEM185A | -1.15182 |
| TMEM190 | 1.146059 |
| TMEM194B | 1.042024 |
| TMEM200C | 1.078164 |
| TMEM203 | -1.42832 |
| TMEM231 | -1.26899 |
| TMEM30B | -1.08114 |
| TMEM40 | -1.54151 |
| TMEM44 | 1.402297 |
| TMEM55B | -1.05984 |
| TMEM57 | -1.31894 |
| TMEM60 | -1.16629 |
| TMEM62 | -1.56009 |
| TMEM66 | -1.55422 |
| TMEM77 | -1.20552 |
| TMEM85 | -1.01984 |
| TMEM9 | -1.35642 |
| TMEM91 | 1.393189 |
| TMEM92 | 1.268192 |
| TMF1 | -1.74557 |
| TMOD2 | -1.16634 |
| TMOD3 | -1.14825 |
| TMPO | 1.062896 |
| TMPRSS11A | -1.40609 |
| TMPRSS11B | -1.56967 |
| TMPRSS11D | -1.56811 |
| TMPRSS2 | -2.82017 |
| TMX4 | -1.25323 |
| TNFRSF12A | 1.748879 |
| TNFRSF13B | 3.872891 |
| TNFRSF19 | -1.72704 |
| TNFRSF1A | -1.67177 |
| TNFRSF25 | -1.33951 |
| TNFRSF4 | 3.075017 |
| TNFRSF9 | 1.444234 |
| TNFSF10 | 1.472198 |
| TNFSF13B | 1.219763 |
| TNFSF14 | 1.793029 |
| TNFSF9 | 1.382797 |
| TNIP3 | 3.164591 |
| TNN | -1.41007 |
| TNNT3 | 2.285701 |
| TNRC6B | -1.0808 |
| TOMM20 | -1.29022 |
| TOMM6 | -1.3713 |
| TOR1AIP1 | -1.49326 |
| TOR1AIP2 | -1.2374 |
| TP53AIP1 | -1.47436 |
| TP53BP2 | -1.5296 |
| TP53I11 | 1.085126 |
| TP53INP2 | -1.91067 |
| TP73L | -1.40102 |
| TPM1 | 1.4831 |
| TPM2 | 2.341095 |
| TPPP3 | -1.5694 |
| TPR | -1.48048 |
| TPRG1 | -1.01928 |
| TPTE2 | 1.233504 |
| TRAF1 | 1.177161 |
| TRAK1 | -1.35727 |
| TRAM1 | -1.22239 |
| TRAPPC2 | -1.39949 |
| TRAPPC4 | -1.37318 |
| TRAPPC9 | -1.26961 |
| TREM1 | 1.238758 |
| TRIL | -1.21056 |
| TRIM17 | 1.754179 |
| TRIM2 | -1.29189 |
| TRIM22 | 1.379562 |
| TRIM29 | -1.48122 |
| TRIM33 | -1.26099 |
| TRIM35 | -1.4092 |
| TRIM56 | 1.087409 |
| TRIM7 | -1.58466 |
| TRIML2 | 1.072188 |
| TRIOBP | -1.16454 |
| TRIP10 | -1.75796 |
| TRIP12 | -1.58515 |
| TRIP6 | -1.06306 |
| TRNT1 | -1.0876 |
| TROVE2 | -1.78633 |
| TRPC1 | -2.20534 |
| TRPM2 | 2.068345 |
| TRPM8 | 1.232847 |
| TRRAP | -1.07363 |
| TRUB2 | 1.034196 |
| TSC22D3 | 1.176662 |
| TSEN54 | -1.37071 |
| TSPAN10 | 1.208971 |
| TSPAN32 | 1.410897 |
| TSPAN6 | -1.19118 |
| TSPAN7 | -1.53672 |
| TSPO | -1.00638 |
| TSPYL2 | 1.211714 |
| TSTD1 | -1.57479 |
| TTC1 | -1.38434 |
| TTC22 | -1.52092 |
| TTC3 | 1.046481 |
| TTC31 | -1.28072 |
| TTC39B | -1.05654 |
| TTF2 | -1.49924 |
| TTLL12 | -1.29404 |
| TTN | 1.456578 |
| TTPAL | 1.027095 |
| TUB | -1.03613 |
| TUBAL3 | -1.3668 |
| TUBG1 | 1.812624 |
| TUBGCP5 | -1.12636 |
| TULP4 | -1.77377 |
| TXN | -2.54403 |
| TXNDC15 | 1.166097 |
| TXNDC5 | 1.820384 |
| TXNL4A | -1.43285 |
| TYMP | 2.542519 |
| TYRO3 | -2.63318 |
| U2AF1 | -1.77812 |
| UBA1 | -1.15104 |
| UBA5 | -1.81291 |
| UBAP1 | -2.0286 |
| UBD | 2.897935 |
| UBE1 | -1.47038 |
| UBE2B | -1.07762 |
| UBE2CBP | -1.07147 |
| UBE2E1 | 1.002081 |
| UBE2G1 | -2.24361 |
| UBE2G2 | 1.544868 |
| UBE2L6 | 1.871933 |
| UBE4A | -1.32407 |
| UBL3 | -2.17264 |
| UBL4A | -1.18094 |
| UBQLN2 | -1.2654 |
| UBR7 | -1.99629 |
| UCHL3 | -1.33247 |
| UCKL1 | -1.39633 |
| UFSP1 | -1.2172 |
| UGT1A6 | -1.26564 |
| ULBP2 | -1.5599 |
| UNC13B | -1.99328 |
| UNC13D | 1.007518 |
| UNC45A | -1.1002 |
| UNC5A | 1.552462 |
| UNC93A | -1.80292 |
| UNG | -1.30997 |
| UPF3A | -1.02298 |
| UQCRC1 | -1.79542 |
| UQCRFS1 | -1.2255 |
| UQCRQ | -1.61181 |
| UROD | -1.4669 |
| USO1 | -1.88121 |
| USP3 | -1.38165 |
| USP39 | -1.89312 |
| USP42 | -1.85474 |
| USP5 | -1.4204 |
| USP7 | -1.06444 |
| USP9X | -1.65956 |
| UXT | -1.41138 |
| VAMP3 | -1.28416 |
| VAV3 | -1.34782 |
| VCAM1 | 1.194059 |
| VCAN | -1.33908 |
| VDR | 1.722619 |
| VIT | -1.21294 |
| VNN2 | 1.639949 |
| VPS18 | 1.084616 |
| VPS24 | -1.78115 |
| VPS26 | -1.66131 |
| VPS26A | -1.24281 |
| VPS28 | -1.24923 |
| VPS39 | -1.43496 |
| VPS45 | -1.08933 |
| VPS4A | -1.02204 |
| VPS8 | -1.35162 |
| VRK2 | 1.015164 |
| VTCN1 | 1.399793 |
| VTRNA1-1 | 1.306251 |
| VTRNA1-2 | 1.255677 |
| WAC | -1.42845 |
| WASF3 | -1.3018 |
| WASL | -2.45555 |
| WDR1 | -1.07681 |
| WDR26 | -1.16835 |
| WDR33 | -1.62988 |
| WDR36 | -1.54901 |
| WDR37 | -1.61487 |
| WDR4 | 1.165448 |
| WDR40A | -2.6247 |
| WDR45 | -1.33803 |
| WDR48 | -1.3896 |
| WDR54 | 1.062938 |
| WDR55 | -1.2849 |
| WDR61 | -1.594 |
| WDR70 | -1.0778 |
| WDR73 | -1.24955 |
| WDSUB1 | -1.00294 |
| WFDC12 | -1.06518 |
| WHSC1L1 | -1.67974 |
| WIPI2 | -1.09136 |
| WISP2 | -1.29892 |
| WNK4 | -1.61631 |
| WRNIP1 | -1.5772 |
| WWC3 | -1.00913 |
| WWTR1 | -1.59126 |
| XAF1 | 1.148693 |
| XBP1 | 1.026027 |
| XDH | 1.180922 |
| XIRP2 | 1.755834 |
| XK | -1.05089 |
| XKR4 | -1.20183 |
| XKR8 | -1.24421 |
| XPNPEP2 | -1.21059 |
| XPO1 | -1.47232 |
| XPO4 | -1.29921 |
| XPR1 | 1.114695 |
| XRCC1 | -1.12857 |
| XYLT1 | -1.06929 |
| YAP1 | -1.37889 |
| YEATS4 | -1.45063 |
| YIF1B | 1.086055 |
| YIPF6 | -1.18493 |
| YOD1 | -2.09807 |
| YPEL5 | -1.26671 |
| ZADH2 | -1.32706 |
| ZBED1 | -1.17714 |
| ZBED2 | -2.04935 |
| ZBED5 | -1.69504 |
| ZBP1 | 1.31205 |
| ZBTB32 | 2.548816 |
| ZBTB37 | 1.74604 |
| ZBTB47 | -1.75691 |
| ZBTB5 | -1.45709 |
| ZBTB7C | -2.12981 |
| ZC3H4 | -1.13891 |
| ZC3HAV1 | 1.70297 |
| ZCCHC9 | -1.19752 |
| ZDHHC11 | -1.52631 |
| ZDHHC13 | -1.7423 |
| ZDHHC17 | -1.0663 |
| ZDHHC19 | 1.628736 |
| ZDHHC4 | -1.84367 |
| ZFHX3 | -1.60974 |
| ZFP2 | -1.15136 |
| ZFP3 | -1.8623 |
| ZFYVE21 | -1.48929 |
| ZFYVE27 | -1.65135 |
| ZHX2 | 2.135752 |
| ZIC5 | 1.015856 |
| ZMIZ1 | -1.03495 |
| ZMYND15 | 1.096864 |
| ZNF137 | -1.13892 |
| ZNF160 | 1.087109 |
| ZNF185 | -2.01847 |
| ZNF192 | 1.249462 |
| ZNF211 | -1.36886 |
| ZNF217 | -1.06299 |
| ZNF25 | -1.15547 |
| ZNF264 | -1.43614 |
| ZNF273 | -1.44195 |
| ZNF277 | -1.04927 |
| ZNF282 | -1.19503 |
| ZNF286C | -1.19107 |
| ZNF3 | 1.128143 |
| ZNF300 | -1.58581 |
| ZNF341 | -1.06195 |
| ZNF347 | -1.59069 |
| ZNF365 | -2.25843 |
| ZNF37A | 1.66848 |
| ZNF395 | -2.2111 |
| ZNF415 | -1.22462 |
| ZNF416 | -1.43023 |
| ZNF423 | -1.19182 |
| ZNF425 | -2.29115 |
| ZNF439 | -1.20437 |
| ZNF443 | -1.2333 |
| ZNF470 | -1.7788 |
| ZNF471 | -1.62876 |
| ZNF57 | -1.40873 |
| ZNF580 | 1.591968 |
| ZNF584 | -1.63487 |
| ZNF626 | -1.15593 |
| ZNF630 | -1.28186 |
| ZNF649 | -1.19392 |
| ZNF655 | 1.13715 |
| ZNF662 | -2.04532 |
| ZNF672 | -1.00164 |
| ZNF683 | 2.021704 |
| ZNF692 | -1.09106 |
| ZNF695 | 1.301364 |
| ZNF74 | 1.017264 |
| ZNF746 | -1.17082 |
| ZNF750 | -1.47801 |
| ZNF770 | -1.23227 |
| ZNF789 | -1.10876 |
| ZNF828 | -1.42556 |
| ZNF84 | -1.06758 |
| ZNF844 | -1.31934 |
| ZNHIT6 | -1.61095 |
| ZSCAN12L1 | 1.551169 |
| ZSCAN16 | -1.5792 |
| ZSCAN2 | 1.224384 |
| ZWINT | 1.213584 |
| ZYX | 1.441062 |
| ZZEF1 | -1.70922 |

| ID | Description | GeneRatio | BgRatio | pvalue | p.adjust | qvalue | geneID | Count |
| --- | --- | --- | --- | --- | --- | --- | --- | --- |
| GO:0003779 | actin binding | 102/2476 | 410/17548 | 3.45E-09 | 3.93E-06 | 3.60E-06 | 7273/29767/1605/6840/4629/8976/6712/25924/4625/9455/54869/10529/5747/10391/2036/2316/84687/4620/9903/64098/23022/83660/199/51474/4608/129446/11034/23242/79778/11151/65108/23499/1773/7140/122622/57617/4646/3059/171024/65979/226/4604/11078/4626/84448/201191/283/8735/83543/23164/9948/6710/4624/3983/6645/23002/7169/9749/4621/79083/8048/4430/10487/4634/4606/2934/7852/7739/79784/4642/130271/55930/29766/10810/85477/55364/5357/10095/4633/4210/339768/11133/2633/84665/1495/27/81624/9026/4137/85358/1729/6525/4645/51088/29114/7168/634/57175/91010/4771/57619/55742 | 102 |
| GO:0051015 | actin filament binding | 54/2476 | 174/17548 | 7.37E-09 | 4.19E-06 | 3.84E-06 | 7273/6840/4629/8976/4625/10529/10391/2316/84687/4620/83660/199/51474/4608/11034/79778/11151/23499/122622/4646/4604/11078/4626/84448/201191/8735/83543/9948/6710/4624/3983/7169/4621/4430/4606/2934/7739/79784/4642/55930/85477/5357/10095/339768/11133/84665/1495/27/9026/4645/29114/7168/57175/57619 | 54 |
| GO:0050839 | cell adhesion molecule binding | 113/2476 | 489/17548 | 4.17E-08 | 1.58E-05 | 1.45E-05 | 3675/1832/9270/2060/3673/6712/54869/5093/3326/53616/28969/7112/9052/2036/2316/10666/6772/6643/8615/928/9748/3898/1903/51474/5586/6164/56924/101/58513/5062/25975/6128/23499/102/90102/143098/6423/6402/56829/51429/55898/4646/57142/9230/1363/57410/3728/7060/3084/226/25978/23650/26136/9793/8357/3683/3913/63923/54874/8482/1499/1982/6868/1004/1012/7412/55357/6159/128866/11082/23348/10606/23164/3337/2335/1013/8842/4856/3694/10631/27020/1525/4430/9588/8826/57228/5214/5493/3939/29766/55324/8839/85377/22905/54505/10562/5861/1366/11329/301/1495/10458/81624/7458/10288/83706/5052/57175/288/64855/302/57126/55742 | 113 |
| GO:0045296 | cadherin binding | 78/2476 | 324/17548 | 9.66E-07 | 0.000275 | 0.000252 | 2060/6712/54869/5093/3326/28969/7112/9052/2036/2316/6772/6643/8615/9748/3898/51474/5586/6164/56924/58513/5062/6128/23499/90102/143098/56829/51429/55898/4646/57142/9230/57410/3728/226/25978/23650/26136/9793/8357/54874/1499/1982/1004/1012/55357/6159/128866/23348/10606/23164/3337/1013/8842/4430/9588/8826/5214/5493/3939/29766/55324/85377/22905/54505/10562/5861/11329/301/1495/10458/81624/7458/5052/57175/288/64855/302/55742 | 78 |
| GO:0008307 | structural constituent of muscle | 18/2476 | 46/17548 | 2.49E-05 | 0.00566 | 0.005187 | 7273/1605/4629/10529/4608/8470/4604/4626/10398/7169/8048/4634/4606/8557/4632/4633/6525/7168 | 18 |
| GO:0030547 | receptor inhibitor activity | 12/2476 | 25/17548 | 5.25E-05 | 0.009494 | 0.008702 | 116372/4061/58530/27121/22943/6352/3557/27122/131/10068/7837/1854 | 12 |
| GO:0019955 | cytokine binding | 30/2476 | 103/17548 | 5.85E-05 | 0.009494 | 0.008702 | 10630/64388/3559/7132/1235/8829/8692/1236/7048/10987/8322/972/8718/3557/7049/8828/3561/2833/7852/8557/3604/3594/659/7293/26585/2633/10068/7837/1441/4681 | 30 |
| GO:0000146 | microfilament motor activity | 11/2476 | 22/17548 | 6.76E-05 | 0.009601 | 0.0088 | 4625/4620/4646/4626/8735/4624/4621/4430/79784/4642/4645 | 11 |
| GO:0030674 | protein binding, bridging | 42/2476 | 170/17548 | 0.000158 | 0.01999 | 0.018322 | 1856/1832/10451/91947/84174/9425/27040/1173/6464/8470/4014/57617/10634/1301/55824/2886/57515/940/8631/55294/5295/8027/10044/10152/287/9402/10346/7159/56961/79083/1310/8557/26747/8935/126669/301/10458/9026/4137/51127/6503/288 | 42 |

| ID | Description | GeneRatio | BgRatio | pvalue | p.adjust | qvalue | geneID | Count |
| --- | --- | --- | --- | --- | --- | --- | --- | --- |
| hsa04659 | Th17 cell differentiation | 33/1111 | 107/7073 | 5.86E-05 | 0.013556 | 0.012457 | 861/3326/919/1147/3118/3091/3559/6777/4775/3122/6772/27040/3108/3932/5594/917/4794/7048/5588/6097/5603/6258/53342/3113/3561/50615/3117/3594/5530/3458/6095/915/5595 | 33 |
| hsa04660 | T cell receptor signaling pathway | 31/1111 | 101/7073 | 0.000108 | 0.013556 | 0.012457 | 5788/10451/3937/1437/29851/919/1147/4775/27040/5604/3932/5594/56924/5062/917/4794/84433/3845/5588/1326/5603/940/5295/1493/9402/2534/5894/5530/3458/915/5595 | 31 |
| hsa04260 | Cardiac muscle contraction | 24/1111 | 78/7073 | 0.000602 | 0.040834 | 0.037525 | 483/481/1346/27089/4625/7384/7386/9377/786/476/782/59285/4624/7169/1350/4634/1351/1537/4633/125965/1349/7168/10479/486 | 24 |
| hsa04658 | Th1 and Th2 cell differentiation | 27/1111 | 92/7073 | 0.000648 | 0.040834 | 0.037525 | 919/1147/3118/3559/6777/4775/3122/6772/27040/3108/3932/5594/917/4794/4094/5588/5603/864/3113/3561/3117/3594/5530/3458/915/5595/4853 | 27 |

| Degree | name |
| --- | --- |
| 2313 | TMBIM6 |
| 2305 | RPS29 |
| 2305 | TRAM1 |
| 2300 | H2AFY |
| 2295 | SLC25A3 |
| 2287 | ATP6V1D |
| 2283 | HNRPM |
| 2279 | UBA52 |
| 2274 | AIMP2 |
| 2272 | CSNK2B |
| 2268 | MAT2A |
| 2267 | JTB |
| 2259 | ARF1 |
| 2252 | ZNF787 |
| 2250 | FBXO18 |
| 2250 | LOC388524 |
| 2250 | WRNIP1 |
| 2249 | SFRS4 |
| 2247 | RPLP1 |
| 2246 | ATPBD3 |
| 2242 | ASB3 |
| 2237 | PACSIN2 |
| 2231 | C1orf123 |
| 2230 | PSMC2 |
| 2228 | CCNI |
| 2228 | S100A10 |
| 2228 | MBNL1 |
| 2226 | CAP1 |
| 2225 | TMEM66 |
| 2225 | PKM2 |
| 2223 | ATP6V1B2 |
| 2221 | ATP5A1 |
| 2220 | DERA |
| 2220 | LYL1 |
| 2219 | ATP6V1E1 |
| 2216 | MAP3K4 |
| 2216 | ACTB |
| 2216 | AIDA |
| 2213 | GSTK1 |
| 2213 | CSDA |
| 2213 | TIAF1 |
| 2213 | BRD7 |
| 2208 | THOC2 |
| 2206 | PNPLA6 |
| 2206 | ZBTB48 |
| 2206 | RBM41 |
| 2205 | CAPNS1 |
| 2202 | DDX24 |
| 2201 | TIMM23 |
| 2200 | RAB33B |
| 2199 | RNU1A3 |
| 2195 | SLC25A5 |
| 2194 | AP1M1 |
| 2193 | SGK |
| 2192 | DIABLO |
| 2191 | UBE2D3 |
| 2189 | LRCH4 |
| 2188 | DDOST |
| 2187 | SLC35A2 |
| 2187 | SNORD3A |
| 2186 | RNY1 |
| 2184 | RNF7 |
| 2184 | CBY1 |
| 2182 | SNW1 |
| 2180 | CTNNA1 |
| 2179 | KHSRP |
| 2176 | UBE2G1 |
| 2175 | CHES1 |
| 2174 | BTBD2 |
| 2172 | ANXA2 |
| 2169 | ZNF195 |
| 2169 | RBM5 |
| 2168 | CFLAR |
| 2168 | LCAT |
| 2165 | SPATA2L |
| 2163 | PRPF3 |
| 2162 | RNF220 |
| 2158 | CALM2 |
| 2155 | GABBR1 |
| 2154 | SMARCC1 |
| 2151 | MFSD1 |
| 2151 | CBFB |
| 2150 | SNX3 |
| 2150 | TPT1 |
| 2149 | COMMD3 |
| 2149 | FASTK |
| 2148 | NONO |
| 2147 | INTS10 |
| 2147 | UQCRFS1 |
| 2147 | CS |
| 2147 | SELT |
| 2146 | TIGA1 |
| 2145 | ASB1 |
| 2144 | PHIP |
| 2142 | LEPROTL1 |
| 2142 | NDUFB2 |
| 2142 | SNX14 |
| 2140 | MATR3 |
| 2140 | MANBAL |
| 2139 | PRDX6 |
| 2139 | ZNF638 |
| 2138 | SEC24C |
| 2138 | COPA |
| 2137 | SLC25A6 |
| 2136 | ZNF557 |
| 2135 | PSMA5 |
| 2133 | RXRA |
| 2133 | MRPL18 |
| 2132 | CLDND1 |
| 2132 | NOP10 |
| 2132 | SEPN1 |
| 2131 | USP48 |
| 2130 | GRAMD1A |
| 2129 | ZYG11B |
| 2129 | H2AFZ |
| 2129 | C16orf72 |
| 2127 | PLS3 |
| 2126 | UBA1 |
| 2125 | RAB8A |
| 2123 | HK1 |
| 2122 | HNRNPUL2 |
| 2120 | HNRNPH2 |
| 2119 | ARHGAP1 |
| 2119 | IMP3 |
| 2119 | CCDC6 |
| 2118 | ZBED5 |
| 2117 | GCDH |
| 2116 | CRKL |
| 2116 | PFDN5 |
| 2113 | C9orf82 |
| 2111 | FBXO38 |
| 2106 | TNPO2 |
| 2106 | PI4K2A |
| 2103 | NOL7 |
| 2101 | ATG3 |
| 2100 | CTDSP2 |
| 2100 | PUM1 |
| 2096 | C8orf76 |
| 2095 | LOC728937 |
| 2093 | MIR128-1 |
| 2092 | IQCC |
| 2092 | PRDX1 |
| 2088 | CLSTN1 |
| 2087 | GTF3C2 |
| 2086 | MRPS15 |
| 2086 | ZNF767 |
| 2086 | HNRNPH1 |
| 2085 | NDUFA9 |
| 2084 | CSE1L |
| 2083 | EIF2B4 |

| Degree | name |
| --- | --- |
| 1191 | PHF20L1 |
| 1189 | TSSC4 |
| 1184 | SKIL |
| 1173 | RN7SK |
| 1172 | NUP62 |
| 1166 | PDF |
| 1165 | CDK10 |
| 1162 | RHOA |
| 1161 | RPL21 |
| 1160 | SNORA55 |
| 1160 | MRLC2 |
| 1157 | C11orf10 |
| 1154 | SNORD67 |
| 1149 | DAGLB |
| 1147 | UBE2MP1 |
| 1146 | PPP1R11 |
| 1146 | MYO18A |
| 1145 | NIPBL |
| 1145 | FOXJ3 |
| 1144 | NOC2L |
| 1141 | C19orf43 |
| 1134 | VRK2 |
| 1134 | SCARNA8 |
| 1131 | NDRG3 |
| 1127 | GIYD2 |
| 1127 | ABTB1 |
| 1126 | SLC35C2 |
| 1120 | FIZ1 |
| 1118 | PTMA |
| 1114 | SKP1A |
| 1109 | ZNF653 |
| 1108 | NELF |
| 1108 | MYL6 |
| 1107 | RPS9 |
| 1105 | LCOR |
| 1105 | SNORA54 |
| 1103 | TCOF1 |
| 1102 | ZC3HAV1 |
| 1101 | C9orf37 |
| 1100 | SNORA10 |
| 1099 | DENR |
| 1098 | C19orf2 |
| 1098 | LOC100190939 |
| 1098 | CCT2 |
| 1096 | UBE2M |
| 1093 | SNORA68 |
| 1092 | GNB2 |
| 1090 | UBE2D2 |
| 1088 | ZNF207 |
| 1087 | YIF1B |
| 1085 | SNORD33 |
| 1085 | SNORA81 |
| 1083 | FAM158A |
| 1083 | ZNF777 |
| 1079 | CALML4 |
| 1076 | TNRC6A |
| 1076 | VDR |
| 1075 | MBTD1 |
| 1074 | RAB4B |
| 1073 | PEAR1 |
| 1072 | KLHL8 |
| 1069 | RBM3 |
| 1066 | GTF2A1 |
| 1066 | LOC389286 |
| 1063 | QSER1 |
| 1062 | BOLA2 |
| 1062 | SNORA57 |
| 1059 | LIME1 |
| 1057 | ATF4 |
| 1054 | FIP1L1 |
| 1052 | QRICH1 |
| 1052 | NONO |
| 1051 | PPP1R9B |
| 1051 | ARHGAP17 |
| 1050 | GTF2IRD2B |
| 1045 | DSEL |
| 1045 | SERF2 |
| 1044 | CDC2L2 |
| 1043 | KIAA0090 |
| 1041 | POLR2F |
| 1041 | RNU1G2 |
| 1041 | GABPB2 |
| 1040 | FKTN |
| 1039 | SLC30A7 |
| 1038 | FAHD2A |
| 1038 | SNORD97 |
| 1034 | RPL7L1 |
| 1030 | NDUFAB1 |
| 1029 | DMAP1 |
| 1029 | ANKS6 |
| 1028 | B4GALT4 |
| 1027 | GTF2E2 |
| 1024 | SNORA34 |
| 1022 | SNORA71C |
| 1021 | MOBKL2A |
| 1021 | IGF2R |
| 1020 | UBC |
| 1019 | UBB |
| 1016 | SNORA75 |
| 1015 | ITGA3 |
| 1015 | TMEM137 |
| 1015 | TRPM2 |
| 1014 | HMGN3 |
| 1013 | SCYL1 |
| 1013 | RAB3GAP1 |
| 1013 | C19orf6 |
| 1013 | SNORD12 |
| 1012 | MC1R |
| 1012 | C6orf48 |
| 1011 | FAM103A1 |
| 1011 | RNU4-1 |
| 1009 | CCDC48 |
| 1009 | SFRS14 |
| 1008 | MRPS10 |
| 1006 | BOP1 |
| 1002 | ATP6V1G1 |
| 1001 | CDAN1 |
| 1000 | FBXO21 |
| 1000 | SNORA20 |
| 998 | CNGB1 |
| 998 | FXR1 |
| 997 | THRAP3 |
| 996 | C15orf5 |
| 996 | CDC42EP5 |
| 996 | OCIAD1 |
| 992 | PPM1B |
| 990 | CDC42SE1 |
| 989 | RDBP |
| 989 | SRF |
| 983 | SELS |
| 982 | ATXN1 |
| 982 | ARHGEF3 |
| 978 | MCM8 |
| 977 | ZC3H3 |
| 974 | FLJ45256 |
| 973 | TUBG1 |
| 973 | MAX |
| 972 | GLOD4 |
| 969 | PNPT1 |
| 967 | CXorf26 |
| 967 | ZNF786 |
| 966 | FAM177A1 |
| 966 | LOC255167 |
| 963 | DGUOK |
| 963 | PHF14 |
| 962 | RALA |
| 961 | ATP6V1F |
| 960 | YWHAQ |
| 956 | SMAD5 |
| 954 | RPL17 |
