# Supplementary material for: Using weighted gene co-expression network analysis to identify key modules and hub genes in tongue squamous cell carcinoma
Source: Medicine (Baltimore). 2019 Sep 13;98(37):e17100. doi: 10.1097/MD.0000000000017100 (PMC6750333; doi:10.1097/MD.0000000000017100)
Supplement: Supplemental Digital Content [file medi-98-e17100-s002.pdf]

NAME	GSM84187	GSM84187	GSM84187	GSM84187	GSM84187	GSM84188	GSM84188	GSM84188	GSM84188
EEF1A1	1391.669	1155.381	1408.695	2126.308	1831.348	1292.224	1332.205	961.2896	1620.286
SLC35E2	68.4285	56.26199	50.70037	81.32558	65.21756	119.5087	92.24084	51.45577	81.39545
RPS28	21095.36	21422.27	18539.87	22068.08	13795.91	17107.54	16378.77	18284.49	22717.39
IPO13	1890.843	1354.356	2874.423	1303.252	3486.569	2619.448	3008.948	5487.047	582.9825
AFAP1	169.3682	95.64756	70.01212	66.9274	52.05152	63.73071	48.32104	145.119	155.0495
GGTLC1	201.9118	168.2476	85.51146	176.9482	74.15343	102.4188	77.38201	95.59639	468.4562
CDT1	5809.804	6777.513	7185.374	6775.806	5932.15	6956.874	5162.282	5632.974	6775.625
TRPV1	34.67157	41.10127	36.19757	38.98831	34.11134	44.4158	35.94931	35.87373	67.56935
LPP	7586.194	8497.279	9052.246	7094.934	12631.49	10143.26	10471.79	10078.21	4802.82
CCNE2	41.97186	34.76241	35.02856	50.93429	36.64701	36.17841	35.83428	31.22423	47.4252
HNRNPAB	1469.847	1829.145	3226.174	2292.505	3378.065	1705.242	2294.415	4170.074	2138.383
LOH12CR1	406.5801	249.7707	126.2971	257.9253	167.5829	181.794	155.901	156.1335	1296.249
SNIP1	38.88057	87.40228	263.9269	69.06408	228.9241	186.5978	214.3971	140.281	114.1257
COL17A1	5488.527	5348.545	9567.525	10650.28	10889.68	12271.65	14692.99	11451.25	2918.764
BCL6B	1996.095	1794.673	1170.517	1603.516	713.1108	901.3072	1132.906	1549.498	1760.64
GRHL1	1557.702	2857.269	1769.997	2538.111	1249.878	1168.221	903.1818	1795.498	55.4306
LOC64461:	36.96149	53.2849	37.24313	249.1295	41.84553	41.51675	56.88275	78.13477	160.8572
ATP13A4	2765.017	509.1944	839.8187	423.0829	1247.109	85.31924	342.3592	1038.424	2766.928
DIAPH2	62.85507	38.02676	56.71022	66.97981	41.38102	44.24879	39.1312	59.72538	65.86638
HMG20B	652.8976	559.1644	251.6252	829.6812	188.3845	385.4793	300.0659	284.5978	1022.828
CD69	743.9554	149.5004	340.8112	75.56936	97.98493	69.09402	108.1673	103.8681	42.61979
KCNG4	42.4479	52.03394	44.77551	51.55121	33.3414	37.80707	37.16734	42.49581	74.13135
MLYCD	1243.003	574.317	228.2583	429.6318	318.1467	530.5049	376.164	279.8732	111.1481
UHRF1BP1	57.82926	75.11554	281.0074	504.9869	122.413	295.9514	86.51105	282.9258	1234.487
ITGA11	875.3518	251.1662	104.9067	156.9062	124.0158	178.0699	103.1232	133.724	706.4405
TICAM2	5847.806	4623.023	3035.867	3603.407	1716.997	4478.072	2576.12	2582.68	4542.128
SERTAD2	1155.807	493.2894	2061.639	1463.384	2741.281	2440.244	1952.531	1671.1	38.77382
SPP1	47.59868	430.2732	218.4724	2807.785	38.58988	562.3168	164.3533	1258.531	75.94032
HBE1	40.6466	45.64254	40.25058	56.63408	50.75883	41.66369	46.80666	37.25775	49.99813
PCBD2	38.7897	40.98315	34.3494	50.37146	508.0545	34.45336	92.56651	33.90906	46.63264
ZBED2	78.05679	65.50915	53.77147	61.25508	41.85393	35.13903	511.9477	36.08022	37.1391
USP21	695.7266	392.1887	135.7457	701.6528	32.53551	217.0998	98.40372	118.7197	44.95681
RHD	46.56308	43.70165	42.03643	47.08219	37.00846	34.64446	36.3331	38.40265	40.3051
AUP1	3185.055	5237.479	5662.397	4336.906	6355.735	4088.702	5393.047	7976.895	5625.279
MFN2	77.31775	79.59517	78.35464	59.59718	216.9118	89.22883	54.00796	64.86144	130.3294
BRD9	18201.2	18755.68	14870.12	17435.49	14889.06	15803.35	16537.94	18215.95	19312.67
DERL3	169.7975	640.8165	357.2742	125.5937	290.7841	282.7196	381.4006	171.3801	252.8985
SYTL2	44.70466	154.392	324.1883	59.55611	276.1444	672.1378	325.1709	438.6144	60.16632
SELM	3944.339	4409.008	3660.22	4709.187	4139.311	3310.551	5301.589	4788.987	2690.512
CTRB2	33.45826	49.04913	33.77181	43.11838	37.13251	46.33274	42.45139	43.78149	39.68839
DCK	65.0332	88.89278	211.0984	107.5319	51.38291	249.842	105.2062	53.60875	172.0043
FOXI1	186.0346	149.9556	73.23193	109.4805	67.19545	114.1079	64.78077	68.57027	179.9962
ZFPM1	93.0562	574.9169	55.77687	51.21912	160.5391	65.55975	184.8842	44.25108	79.3194
UTY	289.6308	203.2789	45.21782	176.9864	41.28842	60.33041	56.22039	89.55778	322.2616
GPR32	59.33947	56.25061	46.34536	49.09584	37.10387	61.50859	41.50498	34.24703	102.3164
KDM2B	92.61031	48.03781	37.70068	58.74979	66.01371	87.11189	59.71702	49.75496	150.5527

CYP26A1	47.94318	59.9198	42.16517	35.69175	40.95688	41.3113	48.71686	41.69621	99.91053
WNT2B	110.4466	114.4003	68.45566	100.0863	67.23918	81.14931	46.0113	94.29903	160.5754
HIST1H2BD	3593.241	7694.048	5449.07	9062.183	3586.89	8076.613	7717.187	8672.3	8070.066
GADD45G	42.21547	146.6328	1243.179	1230.099	499.1094	2431.22	1586.725	2522.752	885.808
ERGIC1	732.6891	1172.211	1149.411	578.4893	535.1775	316.2738	602.6246	1076.036	951.2734
ABCD4	51.40507	50.39623	32.34096	41.15732	40.71888	55.68263	44.15601	37.418	40.66714
FAM193A	1159.861	818.9365	690.6721	948.5804	318.1788	807.7377	381.076	672.6827	1468.622
NCOA6IP	200.1324	151.6146	67.69608	149.3894	120.1886	88.13338	127.0067	77.15234	232.2658
SKIV2L2	87.73213	57.86174	141.3056	61.7998	362.5318	46.36068	183.5952	137.7185	262.6048
GEFT	69.26269	43.4853	235.1319	56.51594	49.05731	65.65419	171.838	301.6391	71.1352
NUMBL	205.0359	159.9213	91.78123	113.4758	99.67046	152.8688	95.38705	128.5312	405.8226
HDAC8	544.7796	744.2976	512.4565	972.5908	451.5029	622.9138	527.1129	310.0785	877.0252
SEMA3E	37.77584	42.48372	37.52037	34.29905	40.77478	38.1195	34.9788	37.25288	35.96736
RGS5	3995.917	3785.583	1713.362	2369.396	5463.882	2493.494	2947.443	3537.557	5834.577
KDELC2	91.79261	75.83938	173.8916	853.6878	733.9053	354.3675	430.2375	313.4928	37.67577
WHSC1	267.7083	130.4315	95.06451	129.7029	90.04247	165.8273	65.38132	105.5186	183.369
MUC13	91.47886	115.2834	57.6124	72.62074	62.35198	94.82235	62.50992	68.11913	80.72861
AQP3	377.1809	247.7214	133.6854	253.4291	146.6817	222.6332	135.2579	84.29006	464.9678
FBXO44	46.3709	60.76202	103.6337	48.74567	43.70436	34.95281	80.67023	49.53064	1872.464
ENTPD2	62.56097	104.3374	60.62518	57.92758	66.08238	69.34254	52.8879	75.25008	126.8557
MARK4	181.0881	209.9447	102.7468	118.0434	68.31576	94.22449	84.07744	87.07331	548.0761
SPRED3	60.55186	76.03976	52.78454	50.31042	58.15833	124.0253	66.23799	55.06161	89.24765
CTNNB1	65.27998	40.21722	48.57371	37.44699	46.6927	55.80885	37.31387	37.8816	33.51468
C6orf201	69.95853	74.1246	61.29899	63.54787	42.68349	106.3062	55.59165	75.09235	184.1278
EXOC6	501.617	1493.61	550.5574	249.6313	381.5129	188.2082	544.5587	621.7432	2408.711
GPC2	400.2884	292.9285	515.6074	417.2107	484.2689	800.0848	303.6602	488.4931	506.67
ASB11	120.5771	279.4663	77.49791	256.996	50.99174	177.3782	90.85793	122.7908	125.8908
RTN3	34.76223	40.36631	38.40668	47.97668	46.15218	42.76717	159.8949	65.49718	55.74463
PSG7	36.96162	53.78468	45.004	31.63328	37.59552	300.1292	332.2044	542.2524	34.9038
ADAM33	73.77969	77.78142	51.32885	68.50499	51.85928	56.43349	50.93767	110.4692	106.1393
WDFY3	48.13548	64.40424	52.73214	76.13995	47.54679	54.80147	47.53292	48.78497	76.3003
KIAA0586	376.1023	535.0964	541.0439	750.9814	365.7341	134.3444	532.6904	387.5864	386.1884
C8ORFK32	58.30744	66.8617	65.4314	85.08849	51.77439	52.30309	53.28235	53.80561	48.63196
STK10	239.4515	936.5477	529.9722	156.1707	184.0402	583.1102	665.8188	768.7386	167.8504
GSG1	173.0526	118.6555	70.28342	112.9438	59.13671	104.0419	67.08311	68.37755	259.9615
EPS8	738.1082	595.5391	51.16618	39.72403	298.3656	44.57534	349.7297	58.61417	45.19117
CAT	618.2823	970.9861	422.6132	1417.455	480.6764	882.1055	466.9473	185.6778	61.10342
RESP18	36.81564	36.6424	39.61389	33.7949	45.56754	39.15348	40.83365	44.36755	34.76291
IQCD	33.01945	376.6996	211.7247	628.3847	46.14657	217.5579	284.7067	37.00128	1053.428
RBM11	273.5378	175.429	103.9464	194.4669	90.48342	147.1028	91.41936	127.6257	306.1558
RNF2	181.9776	266.9232	166.9924	332.716	154.97	113.1911	161.5381	248.0522	338.6164
ADAMTS20	34.32187	42.48428	45.55079	32.17357	43.06471	33.47033	47.88646	37.63826	42.49133
MBTPS1	4588.953	4806.19	2043.156	4243.973	2024.368	2609.958	1701.995	3400.603	6858.202
TGOLN2	1553.395	718.083	981.0789	1542.381	914.5585	1088.296	928.2648	554.0424	1177.646
ARMCX3	130.31	128.0623	90.68453	185.2693	66.86897	110.513	79.37764	80.4326	232.5292
TMEM25	85.30873	84.46807	55.83196	54.95939	135.0558	68.05757	56.09534	154.6161	89.50138
CXorf21	252.3562	87.26234	76.34973	174.881	70.7605	84.78626	64.1795	84.59753	169.3055

TFDP3	160.5867	122.1037	56.18215	44.00644	51.03617	75.53737	37.36106	59.68256	57.53521
MKL1	80.89391	35.49531	33.6997	41.63493	31.175	35.29417	31.64788	41.97265	2873.125
ZFYVE16	1115.584	1029.004	835.7615	1024.113	557.8495	1219.675	795.1751	1066.87	39.49097
RRAGB	40.15685	476.2665	177.366	43.36889	155.756	47.29211	89.50165	149.9216	34.44904
FHOD1	323.089	627.0243	170.0078	403.3682	409.6805	346.0364	896.4639	843.6276	1121.547
MSH5	305.9018	211.2912	101.5139	199.8219	212.7867	138.6533	81.93726	119.3727	465.2366
MAP3K11	66.61873	54.29837	1670.705	40.6106	2837.263	717.4075	2289.021	2952.475	34.92483
RORC	84.85594	45.55247	57.54838	51.89295	74.83092	39.99588	48.07033	58.40759	99.82255
SLC38A2	18312.37	18162.47	18335.1	19239.08	19267.45	18763.14	19439.48	17668.33	19123.83
GSTM4	87.2415	60.92654	296.4398	61.5035	516.7002	1150.184	374.5857	318.707	76.36279
GRIK1	78.82796	50.05814	42.97165	60.36075	45.97739	154.5682	60.25328	160.1378	35.86209
GALNT11	50.82627	53.60879	206.321	55.60531	493.4561	73.75478	394.2314	861.7418	76.90607
BASP1	2091.903	3049.001	5682.659	4757.98	4017.145	5984.461	7810.088	5174.772	1298.811
CCT3	1398.884	2039.614	1997.208	2289.424	2325.9	1951.199	2094.279	2537.212	1822.832
EHD1	8918.313	11754.42	15460.82	12471.04	15972.33	11560.15	15905.19	16822.2	9506.211
RND1	35.63308	38.62511	39.84464	35.21944	130.8327	37.47026	83.82164	43.13483	42.9071
ITSN1	42.8284	73.24229	35.1809	36.40665	39.39613	274.6972	38.96449	38.03769	45.60114
RPS16	24628.77	23774.46	25855.48	25921.68	23712.81	22944.27	26373.29	24765.31	25908.78
UBIAD1	5118.95	4593.018	3620.047	4238.741	2516.785	4162.877	3464.023	4382.702	4929.625
EVC	89.30806	66.98767	184.0493	67.26701	107.2104	735.0371	185.0327	855.8247	133.074
CWF19L1	11296.34	10795	11032.29	10668.86	10560.73	12421.22	9857.606	11617.03	8651.119
KCNA4	42.54099	34.22969	32.33514	43.85036	49.64337	41.34645	34.72724	68.94283	68.00779
RASL11A	36.49157	51.25779	44.9078	57.06903	88.90877	1140.965	40.93882	75.5767	2667.802
ZNF187	68.88732	123.7324	77.27296	55.36718	48.50969	93.81762	47.99081	53.72837	64.6183
SETD8	2482.632	52.34118	1473.458	89.43824	1470.663	1388.962	892.7498	54.61971	58.07455
OR6C76	43.56441	32.12851	36.25624	36.22912	36.8973	38.20078	33.86758	29.73001	81.8597
ANK3	233.4506	142.7091	77.51474	191.6127	71.61179	96.83345	78.01049	68.1543	329.3977
SCYL3	258.9247	620.269	82.95073	138.1191	165.1741	71.74147	327.8031	530.4498	241.1086
TPST1	57.30051	50.46353	42.14448	58.42352	114.0987	56.73324	45.07387	37.11066	49.73359
MGP	3195.252	514.9057	507.2287	220.1067	772.9957	794.282	677.9694	1240.287	203.1631
ABCC5	1066.094	1844.837	2627.67	916.2529	4209.134	4148.459	3076.591	3442.157	869.905
FGFR3	377.3303	139.0678	143.6048	439.4072	130.5864	155.4227	79.38419	143.8387	883.8633
TP53I3	347.565	206.617	111.2328	248.5895	91.19572	161.2848	98.93645	129.8313	461.3221
C6orf129	201.6924	92.57276	187.8362	76.06075	204.3401	80.50669	184.2009	330.729	77.93726
SMC1A	101.2025	2311.965	1644.267	2133.643	1173.656	798.0876	654.2544	1443.035	2260.471
C15orf23	77.79058	104.9665	338.2072	113.8476	131.9804	56.40324	289.5419	140.4193	94.11946
PPEF1	42.22825	58.35363	42.80579	46.541	37.79518	767.1191	1185.72	1129.65	40.64535
DHX57	46.66356	41.48164	116.9276	402.8747	54.78136	43.46004	47.83215	142.2517	73.05233
AGFG2	69.81276	36.89683	50.51969	118.4522	51.0371	34.09553	96.15795	56.05131	272.5911
CSNK1G3	49.17356	591.6439	210.4134	40.74395	270.1013	726.2876	46.15117	37.81488	85.86977
UPP2	57.28703	45.99512	46.658	42.98711	36.40433	34.92185	37.70683	40.54353	69.49343
TTN	806.2099	1968.188	915.7056	44.06654	204.4901	858.4578	1006.232	1930.253	1220.712
KCNMB1	1402.917	76.7086	1093.727	740.0563	590.3688	987.2213	977.7847	831.3162	63.7957
MGC42105	105.2359	47.92746	192.0783	34.9094	451.6115	40.5018	95.88786	41.73771	31.93282
ELF4	45.79105	423.132	1883.749	758.8532	1468.806	1642.89	2173.008	1319.417	1077.877
MPHOSPH:	5084.402	3174.729	5982.942	3240.504	5669.222	4507.849	5826.064	6343.581	3630.45
PAPOLB	46.63482	49.18176	41.87965	43.81965	49.24277	60.76328	48.67647	50.28223	226.2431

KRAS	111.2512	73.70477	64.21771	89.21854	48.80143	57.09495	50.25767	59.18692	76.29041
LMBRD1	568.8683	242.2008	171.4408	288.3571	148.0097	197.7725	197.165	207.4264	876.742
PPYR1	41.77361	52.14781	37.36832	49.45564	34.21651	37.3539	52.979	44.06449	50.64169
MRPL43	773.302	570.2339	210.1693	375.7941	157.8092	329.2207	164.5158	134.3446	1333.937
LRBA	537.2358	274.8293	245.6499	347.8435	218.7728	207.9063	187.4007	175.9901	630.0852
PNKD	195.8368	1184.583	96.59372	170.2245	76.97907	88.53513	152.0409	80.67838	278.4311
DVL2	774.3112	458.2166	880.3347	470.0237	1247.881	275.1497	881.8055	632.5721	370.3247
GUCY1B2	55.52274	35.31356	563.8679	55.47612	292.3891	1181.408	37.49907	42.57478	49.16253
LOC649159	244.6863	173.6245	73.51829	192.2439	76.70596	90.7003	59.44148	104.328	306.3201
CDKN2C	36.82851	35.55888	37.09663	35.44872	32.97691	31.67489	39.26617	36.02435	61.09829
FLJ41733	82.1424	74.84415	72.19737	119.1307	68.7965	58.89619	61.03854	44.70876	164.8473
KIF2B	38.06004	38.3903	38.47943	42.18534	43.16008	61.57331	43.56372	39.10495	74.33461
IL4R	2165.615	255.9203	3037.69	990.4619	3030.687	2013.685	2653.498	3459.081	2255.288
KIAA1632	476.1566	42.31137	54.43798	46.26346	74.23183	176.4483	42.02985	93.22977	68.80294
CCM2	41.32041	36.16809	130.1927	40.82561	31.23041	261.8098	78.95702	183.0867	42.34097
COX4I1	25071.28	27706.86	26403.53	26263.47	26557.57	29060.94	26555	27265.4	25707.27
C20orf112	334.1858	95.51255	74.31648	100.7126	62.16933	108.1503	77.84678	67.40567	130.3552
WDR91	60.95612	54.93015	52.81509	44.51917	179.7139	347.1667	211.6992	57.61731	46.76133
CBR3	1424.077	364.6203	365.3084	53.42573	469.2273	607.5738	233.4118	356.8494	55.07452
FLJ32784	165.7198	65.02047	85.0181	89.36903	72.75472	94.98475	64.345	92.3625	189.4462
POPDC3	172.4539	792.8895	340.8218	1496.219	213.44	1471.328	1071.13	1487.232	90.69339
PPP2R4	48.59121	51.32589	44.85989	52.60893	94.60011	41.83841	127.9184	122.792	55.81448
SECTM1	1134.559	534.4109	382.2914	245.0029	86.82376	358.2484	125.1585	233.5087	577.1782
FBXO32	1710.835	829.5718	1831.865	2740.57	655.3058	2332.795	2380.281	2641.22	3912.151
NOG	87.76048	68.41074	55.81925	74.16432	46.03059	47.91486	63.16807	48.66268	1218.365
ZNF507	131.1139	52.90733	129.2465	75.00764	260.8914	227.1894	138.1106	110.911	279.5025
MEGF10	289.5727	145.4066	122.5216	159.9496	88.78317	138.041	67.437	96.84545	273.5212
TTC8	309.8712	198.5812	155.9665	274.1361	104.7549	226.7516	158.2984	104.8785	266.0473
RNF215	132.0224	73.48975	55.60001	59.57355	50.99717	61.82281	46.15179	54.24664	64.26102
SPAG1	59.5141	62.98119	47.10616	56.18486	51.58947	47.42053	38.36293	42.53789	67.58813
FN3KRP	2173.213	2368.007	2904.244	2552.866	2241.944	2574.225	2518.695	2424.93	743.1091
C4orf50	225.0222	147.1216	85.7905	180.5596	66.33998	91.9961	67.55035	118.4957	353.871
PDHX	147.1371	432.6367	781.9024	489.1255	844.0873	958.4235	704.4405	766.3514	51.23667
NLRP7	67.872	49.57689	49.28477	43.38103	47.87288	428.6563	744.3824	197.8636	34.79655
ERCC1	99.54482	44.52183	39.53545	39.69624	35.43117	45.71323	271.4053	52.21424	51.22667
KIAA1324L	1288.281	452.2592	344.4273	1969.138	278.4122	284.9298	184.4895	367.2464	1911.49
IFNA17	35.82592	33.585	39.10937	34.21225	32.02776	36.00043	34.7702	34.90288	46.50243
IPO7	1040.715	864.6324	249.4798	1346.525	139.6219	40.25297	134.8417	147.1419	65.64188
SUCLG2	513.3139	428.5264	883.3802	878.9856	810.5316	985.2619	1019.602	493.9374	654.2942
SIGLEC5	152.8229	55.945	64.50858	107.6598	97.67786	78.08953	58.06574	73.13155	249.6249
DCHS2	64.02828	42.6468	45.40232	50.43122	51.23337	39.89196	33.58171	35.27432	37.12929
RAB2A	181.8431	407.7755	749.4789	612.1389	670.8546	1424.886	468.1176	1160.513	328.1049
TRPT1	183.8496	71.79374	37.12602	61.25471	38.02681	59.76958	42.13991	40.59146	375.6618
ZMYND8	1558.802	64.01693	77.62984	555.1765	47.14912	110.6504	275.3735	128.7184	2148.914
SPATA7	64.04285	649.8403	1549.755	334.0967	751.4638	1331.343	1757.258	1260.081	74.89286
PHKB	47.26554	47.45563	319.9648	447.007	275.2779	278.8642	134.8114	302.8132	47.78998
UCN2	43.30958	44.01252	596.8686	1226.182	1061.848	1527.145	1991.726	1953.653	35.15107

CA1	47.75146	54.0748	48.91228	54.95761	35.88788	45.366	43.18816	39.47465	34.04251
MTHFD2L	3074.335	2978.085	1896.488	2985.76	1631.685	3146.364	1144.573	2066.855	2277.722
KRTAP3-1	166.5968	130.6164	74.55519	165.486	67.76144	94.77602	64.22292	75.77214	61.47525
RAC1	247.6708	88.36768	83.80019	171.5933	71.834	105.1448	59.30928	65.54983	181.9511
TMEM98	44.56462	59.08245	169.742	43.56248	41.33007	33.94856	39.02312	41.63525	61.81297
PARD3B	104.16	87.11971	47.05361	76.56137	58.36904	62.36019	45.1704	69.09967	85.10588
TUBA3E	39.92632	35.73669	36.66187	44.67875	35.68756	36.44744	32.90403	33.25984	52.69212
SLC26A4	37.14667	410.4254	159.9431	38.17606	225.8796	34.80724	38.05791	49.77448	46.30422
BRAF	32.33101	33.13006	301.5343	35.2239	168.451	41.8372	125.288	172.6889	964.4151
THAP7	91.86748	51.91204	45.49674	57.09374	37.18438	36.64148	44.46208	35.01542	63.52873
RIOK1	93.09222	125.0445	90.15186	90.17754	69.93288	70.88551	68.53537	78.98441	42.09621
MANSC1	52.97935	61.32544	43.95734	42.27274	63.18812	49.92021	40.18437	48.26755	33.10692
FAM139A	39.11843	57.26361	60.30614	42.64155	36.96726	52.00503	39.15668	108.0047	77.81505
APEX2	1514.536	2470.343	4570.103	1171.11	4207.377	3410.353	4190.309	4163.227	1652.097
C2CD2	6070.713	6244.006	4163.367	5139.284	3738.538	5829.64	3809.33	6786.613	6249.619
MTMR9	665.9917	307.8312	379.1182	343.3391	486.7682	400.3195	578.3463	247.3435	832.4296
FAM125B	45.79438	940.2157	976.1227	43.82188	1026.318	1328.996	1719.643	515.6664	39.17737
RELL2	139.7147	94.97285	65.53123	92.25194	68.61114	94.64348	93.63607	70.95795	70.55406
DTNA	136.0095	69.01925	199.804	62.98875	44.02444	52.60535	228.3146	53.88789	157.345
TSHB	53.05359	38.15465	35.50185	46.32389	43.00397	36.91777	41.81285	41.9625	39.98924
POLD1	46.49702	43.64408	42.2088	55.7987	39.12055	38.73237	33.94597	35.1958	42.58187
ACTR5	134.5423	112.8709	1061.657	83.20361	1020.536	1101.166	937.0134	858.803	951.0214
C11orf35	195.1449	43.31207	56.22058	743.9115	40.48116	76.28239	38.33524	34.39227	87.84026
RGNEF	37.4269	34.94164	36.32567	40.33744	47.77633	39.44967	33.76849	49.48675	38.23253
GLIPR2	17939.81	16726.65	20129.31	15154.99	14939.42	18201.48	21582.72	16790.58	12194.94
INPP4A	106.9545	121.0798	80.39565	146.9034	87.60497	96.40508	116.7924	91.58243	260.5119
SH3KBP1	75.24101	1565.698	728.4559	862.5475	428.9514	458.0506	422.5566	546.5226	236.8207
GRB10	108.5882	134.9925	74.00719	89.11579	60.87116	102.2619	58.94596	68.0976	227.0233
FAM89A	148.5853	96.79585	75.86086	126.3797	214.4684	80.67901	130.2865	341.8399	210.8794
SHH	41.44437	39.89366	39.05877	36.30756	44.41983	44.17554	35.18397	31.68663	45.65479
FAM131B	166.3399	116.6922	73.80795	118.0269	65.00801	106.331	48.85559	67.33576	269.9318
C10orf111	40.92029	43.47819	43.73786	48.6845	42.67442	37.03826	41.66786	38.69939	77.49767
THEX1	617.4836	420.6099	582.0962	475.8315	415.3744	579.0602	313.9615	425.9825	822.3891
TMCO5A	176.6739	126.3417	69.91215	96.41775	82.81271	122.9988	75.33436	80.64995	233.8965
C14orf2	100.0774	1260.081	1273.2	1505.371	1639.231	1591.665	2016.073	1564.501	1238.887
GLT8D1	143.694	95.06258	69.19147	389.8162	65.46371	94.46864	55.60961	68.92354	97.61022
ANK2	2797.436	3036.969	777.1501	158.4072	820.507	678.1692	248.3984	1148.961	6260.566
HTR7	149.7323	666.8816	68.75948	634.1169	57.18137	77.15587	59.8552	38.03848	80.0226
RFXAP	55.69317	66.30105	113.0586	245.2062	112.047	152.9883	122.6912	182.471	278.1292
RPS6KA3	1644.664	1417.069	793.0453	1224.491	594.1095	560.2159	467.6157	573.6852	1221.016
ERF	62.09475	1159.146	425.1026	1243.016	195.2477	895.4237	58.32096	274.4874	70.36589
PAAF1	41.8941	33.90031	36.68084	46.82509	462.816	40.42479	337.6916	53.55187	38.44855
ZNF428	14252.63	12456	11129.28	13214.51	9178.56	11068.87	8539.897	10952.52	14243.58
HIC2	235.9412	1480.701	1022.722	183.1665	2315.552	1228.626	1611.761	1292.292	2205.815
CDC42EP2	1154.893	1447.558	604.757	577.0699	474.575	614.3333	529.2053	1233.015	1415.374
TMOD2	48.94192	35.30494	55.23005	34.0044	189.2666	262.6685	115.7907	36.51183	1430.855
PIK3AP1	363.713	195.4093	104.2118	211.4801	86.39493	139.0882	91.39313	91.01497	507.5035

CLK1	3904.844	3585.114	2266.179	2894.932	1548.96	1967.944	1186.572	1542.097	5381.512
FAM174B	7707.807	8019.578	6876.148	4006.322	6353.443	6143.933	5963.355	6476.168	4402.958
ATP5J	806.5561	357.5423	180.6761	239.4771	158.6685	366.767	105.3547	221.4446	600.3185
HOXA3	78.2955	52.00252	45.94883	57.08534	60.24917	48.06433	44.54744	57.69203	45.72923
OPA3	60.92464	44.5143	39.37577	42.54292	175.3602	35.09346	181.9755	39.77039	38.37783
IL10RA	497.1386	887.43	3088.528	316.8835	974.6537	305.6093	893.9407	46.59066	513.1945
LRRC17	1208.658	517.5289	687.4703	841.7827	210.6373	1462.042	1205.75	1342.937	110.9619
GAS2	117.492	63.67263	65.32129	109.172	70.26668	78.50495	62.99098	62.08936	192.5004
SLC15A1	649.3052	323.3015	118.6152	142.9612	569.4848	416.1448	198.9478	358.4252	238.91
BAIAP2	424.1318	215.8996	110.0958	226.837	126.3211	172.6395	82.80101	113.3891	556.5068
LMX1B	73.20944	83.42714	55.46682	75.0924	57.17847	55.62257	178.6964	74.90279	136.0822
UBOX5	484.8092	281.4019	150.2062	309.6906	221.4837	271.4482	174.2681	260.9533	676.4707
GBGT1	3811.504	2265.709	3847.022	1300.582	2802.066	2357.946	3167.701	2776.506	1701.904
CDC5L	1334.323	548.8965	616.9277	1504.738	437.7647	915.1448	520.5941	600.7867	1399.554
EZH2	61.23743	378.5886	486.8779	40.063	109.4556	422.6811	339.8696	143.8336	1161.693
RHOF	53.32417	66.36044	50.50532	61.21829	77.88014	52.12378	206.0511	51.24574	1249.902
ORAI2	39.33458	58.31177	36.04338	48.75665	49.62576	50.40021	38.21305	48.12666	65.17449
POGZ	116.0883	318.8542	298.0352	340.3232	379.5084	303.9653	202.0951	168.6869	40.59997
PRRG3	200.7554	98.76605	68.68114	70.76254	125.6118	61.20811	58.15962	62.11873	250.1579
MGC16703	602.0466	298.9137	54.7236	51.54694	55.59761	59.39813	67.79598	48.35216	63.4664
RASAL2	689.1537	45.45552	230.1549	568.4257	126.0957	103.3865	73.89278	171.3351	62.00553
ABCD2	82.27314	84.83062	57.31562	48.72988	52.2219	50.91082	59.12712	42.18883	63.97131
DLK1	61.43927	66.5837	59.64623	60.97641	51.06823	79.79826	51.75047	50.15246	130.8133
NUP160	41.3071	42.72223	466.8954	59.87793	143.8583	39.09562	331.7126	136.2132	55.77953
PRDM2	40.84801	33.49834	30.58521	37.72633	34.93774	36.18901	85.09749	37.31015	39.84779
HIST1H2BL	35.07756	33.06897	53.43969	54.37524	36.1988	41.99154	102.9037	35.19696	45.63083
CABLES1	877.0979	149.0475	559.1797	123.5976	709.0192	105.67	413.6795	105.3542	220.1967
CSN1S1	78.81232	50.54319	51.21401	70.84974	60.86081	46.09533	40.70575	39.29837	74.66621
PLAC8	35.05836	61.76944	43.51016	39.20013	41.21875	58.5213	40.87267	43.16441	55.26451
COL4A1	4491.851	4866.229	6847.085	9620.222	7589.528	9243.652	12116.97	6294.036	3567.702
PCDHGA12	57.04665	43.26687	33.83148	35.12265	39.84991	33.43958	33.65741	55.66845	72.83715
HSD3B7	85.27613	50.83336	95.82941	105.7221	231.2415	56.34197	56.21354	73.06622	74.75511
SOX3	66.02817	56.64534	63.61383	57.05257	46.0951	32.91851	47.39287	47.24493	148.2053
OR10Q1	41.46331	53.89266	33.23019	41.36142	38.10471	42.42145	40.73705	36.81661	40.73854
CCDC64	169.1747	181.9441	174.7929	100.4705	247.737	133.6921	104.1353	114.4642	291.2866
C20orf152	39.19305	34.86733	36.11654	33.68404	35.83698	107.2057	32.76506	36.87968	33.58555
ECH1	1170.484	1540.448	796.8428	1200.566	1417.073	319.0893	1185.186	888.0704	58.16087
ST6GAL1	47.51638	41.26529	50.29806	46.49109	112.9551	67.91167	37.75686	41.02569	32.39233
PER1	417.7431	257.4592	105.66	231.1995	62.62832	60.4828	63.30447	74.56767	577.3769
CSTF2	7153.199	5128.863	7422.485	7719.534	8133.825	7462.859	9111.338	7066.358	5156.853
SH3D19	34.52971	285.7931	156.0764	336.0511	138.907	37.50551	255.0068	323.9138	37.4815
TMEM188	70.36702	85.87503	129.2688	107.1891	179.2025	173.3631	183.6624	98.36952	133.4467
ECD	58.79898	74.79931	32.65035	63.47563	56.67498	53.20198	46.7588	220.6788	67.00747
MSH4	41.95519	82.35105	48.19688	58.58068	47.85946	79.37444	41.18252	45.2015	72.60064
DPRX	43.2803	54.98389	155.9194	53.31788	119.9834	46.20383	286.0314	153.8631	68.01913
SNCG	1681.165	962.4492	116.0073	837.35	308.9435	492.0901	327.2589	590.8114	2451.433
ZSCAN2	45.30891	44.3447	43.69608	47.83459	391.1684	46.85853	161.3224	60.27647	98.95921

AAMP	168.3518	190.7884	132.4478	136.538	225.897	149.5741	202.4434	245.9632	773.4693
CLEC10A	67.69693	83.40874	578.337	368.1694	465.4735	1029.098	349.9685	376.788	131.449
GCNT2	78.22048	107.8807	58.40229	94.79475	53.60965	74.29445	60.9556	49.49926	1106.436
SLC22A17	204.5227	187.6655	1242.981	176.5714	3796.96	1701.339	1872.229	3097.019	338.398
EFEMP2	5615.325	3716.568	3920.628	5341.486	4585.291	5437.175	4793.85	2192.849	1623.253
ZWINT	44.21252	42.4598	245.6569	51.20372	155.0382	38.45499	86.29204	218.965	35.79741
ATP1B4	44.84758	40.00184	116.1317	68.00787	47.27601	247.7186	103.8809	129.0147	46.63374
PITPNM1	75.16179	791.546	1120.85	125.4391	914.4637	767.3192	501.976	800.9183	74.45417
C3orf22	177.9286	146.4858	92.2296	196.1287	77.94873	82.78422	79.76011	94.90399	179.65
HIST1H1B	512.5731	47.20993	108.0407	67.80917	100.6272	58.4235	55.67225	39.18998	39.8788
GPRIN1	42.68002	36.01204	269.9451	35.30888	113.0287	47.2608	78.16559	152.178	1031.969
DNAJB14	822.9254	1877.074	2328.797	2558.731	1636.487	2436.21	1416.793	1257.954	1568.984
GPR26	113.1828	50.90268	62.30615	63.3989	57.47456	84.5519	54.23124	61.51388	168.6557
MUC20	41.9174	48.33806	922.3433	40.0378	246.3254	342.3966	582.9111	475.5565	661.5215
LCE2A	648.3329	3669.585	165.2154	1094.158	9611.645	1421.872	857.6896	1669.403	943.0405
APP	1533.312	600.7604	563.7723	1666.528	407.4403	591.9049	669.2616	476.9953	1199.464
PFKFB4	3477.802	3838.381	5347.625	9088.812	6084.321	4598.208	9842.06	2962.558	2062.185
ZNF580	332.5849	105.4053	85.32578	129.9105	68.68364	107.1006	57.86675	74.86592	126.1872
MC1R	6473.578	5419.135	3351.886	4307.025	3516.545	4794.092	3094.671	2960.457	5816.308
IL22RA2	35.72902	42.83073	39.15705	44.86676	41.16552	62.6732	44.99912	41.6095	58.98756
HAUS6	46.5222	46.88692	57.11313	251.5672	56.56435	98.29709	52.42564	85.6598	41.34893
USP32	98.93438	64.94624	162.7645	56.4357	49.02234	278.216	83.24326	128.806	1261.159
RECQL5	560.0682	36.26216	112.5499	38.74378	90.67903	38.6025	62.87847	42.05368	31.69776
DUS1L	45.39095	33.98735	139.4808	35.1077	42.32153	40.46574	192.1219	135.9692	35.46137
FUT3	1409.781	652.8549	219.6187	521.9875	893.2836	457.7639	1055.005	247.4042	158.2011
TAF12	79.11684	39.24533	33.50204	38.79255	35.34546	45.74057	117.3445	262.3644	47.71485
ATP8B3	45.34263	44.44103	57.93896	676.6266	363.2238	47.56013	207.9239	34.6447	47.85761
FAM171B	59.74369	529.5089	56.79847	83.14246	58.62669	61.77465	50.97466	62.23742	169.5142
KIF3A	32.44398	104.2362	133.2061	287.4245	112.9787	103.9633	84.53046	94.00462	35.69332
SULT6B1	217.7355	124.0497	79.09311	112.183	72.33546	85.09916	65.12259	83.36437	176.983
NKIRAS2	271.2541	261.4494	522.8652	137.2447	696.1556	450.7487	760.1846	130.3211	99.99867
RAXL1	2386.576	2196.958	858.7099	1252.472	277.9134	526.9717	350.1719	534.1891	2098.903
SPINK9	38.14429	31.99377	36.14543	79.45814	41.49282	39.80251	33.07688	37.33916	36.83858
SLC35A3	63.1357	669.7677	779.2581	608.3134	643.807	998.7243	1211.299	343.7955	880.8885
HNRPDL	2539.587	1818.266	1509.931	1861.119	1615.04	2213.799	1348.461	1219.229	42.40873
DMD	49.83631	56.80723	40.78725	42.49132	149.07	51.29987	191.8392	262.6678	35.85693
PHF14	236.6291	96.09091	114.7809	38.11946	93.4153	108.9539	92.61031	105.9043	33.79342
CDK2	46.78213	37.13948	38.25209	63.05233	45.50735	36.75398	47.57079	33.60263	132.9916
PSORS1C2	127.1565	156.9053	84.12495	128.3655	3597.351	78.40639	148.4122	2059.967	221.6169
MID1	88.61388	97.96956	82.47799	91.69099	85.90149	115.757	57.75517	72.95671	141.1678
IL11RA	2505.609	111.5175	58.38311	111.1626	176.1779	753.976	230.2926	55.96312	160.8028
MGC48628	91.1745	115.8088	72.71718	122.9663	68.1162	84.28628	53.32835	65.1946	63.29075
SULT1C3	55.72131	41.13115	38.83057	75.24545	44.64605	43.40521	41.91927	48.33053	51.2847
HES4	13571.61	13439.89	12302.5	14350.57	8251.438	12046.74	13026.21	6794.764	14737.27
KENAE	272.504	106.8916	77.56101	157.2213	98.71777	125.4989	80.17168	122.5945	654.0482
S100A1	914.5327	1139.643	438.6256	606.9885	150.6611	654.4118	900.9541	1578.104	255.2733
GLUL	41.49615	38.87599	187.4414	60.25739	227.8392	36.81639	244.0457	45.01485	34.25985

IGSF6	368.7973	225.0992	150.143	244.4655	97.40411	238.9834	152.9976	222.8031	489.5317
SEMA3B	38.71584	1904.187	1000.265	3234.7	3304.823	711.0426	4287.086	1580.194	2189.943
EDG1	785.9118	437.4219	136.4758	236.8933	185.2478	164.836	221.4651	411.6001	1631.132
SIKE	73.18922	50.58023	83.52392	56.00274	449.7539	47.51699	308.5827	360.9411	63.0639
TEX10	163.1085	111.7582	62.16956	59.91839	52.72144	84.48167	63.11006	69.91576	46.00356
C14orf80	4774.315	6180.565	6289.11	6043.89	6207.124	5221.53	5327.088	4716.529	5650.529
XYLT1	64.24076	60.60663	583.1121	83.82721	778.6272	66.65221	270.9856	51.95037	130.493
TRIM40	101.1899	53.91556	52.5845	45.68594	50.52601	55.8629	36.70321	41.26143	69.66349
ATP5D	87.77453	2599.615	1437.916	2166.008	1701.696	599.0827	1747.468	1894.648	3132.101
KCTD9	3484.49	4783.177	4183.974	5076.259	4634.355	4282.206	3988.552	1988.735	4317.155
PIGO	192.0571	704.7443	223.5953	151.4857	83.72054	690.9722	469.8529	288.0708	388.0764
ZZZ3	205.8047	70.86025	374.623	73.88923	67.60365	89.6729	102.7581	68.84228	76.15427
UBL7	2170.86	53.03726	729.983	1103.325	458.9979	531.7298	185.4052	1029.145	1347.248
GPR63	118.4592	75.11227	57.83267	137.821	40.29343	54.57635	45.59861	55.85519	75.70584
PTPRC	655.3864	332.8857	761.2283	586.2287	159.3805	290.3582	335.1824	219.9431	47.99684
KLHL7	251.4354	216.8835	77.28245	202.0977	59.79396	117.1087	66.52773	88.66672	355.3355
CUL3	33.45309	32.55086	195.3252	35.05092	175.269	172.9465	42.07825	34.78781	50.36956
GALNT9	296.0939	156.1672	90.13019	184.8489	153.0227	268.61	86.05587	103.1576	468.1622
IL9	85.88596	55.40176	45.58408	71.52891	40.06319	49.75045	52.13464	68.91132	77.24008
CORO1B	34.36352	2202.713	3380.744	1113.069	2996.68	3152.866	4082.749	3352.285	47.70971
TIMM9	175.3279	173.0263	375.6837	536.538	1014.211	165.7948	661.9189	286.7136	62.81498
FZD2	34.46158	30.97257	34.04567	39.8323	257.4778	38.77651	218.9602	482.0886	47.00709
UAP1L1	257.1327	184.8313	229.1242	250.8145	75.63414	390.6837	126.1317	121.3806	403.3938
ZFAND2B	2695.547	908.2697	890.2565	713.4539	1296.323	1024.11	1119.774	1168.927	2203.644
ITGBL1	363.89	241.5854	117.8374	154.3491	146.8688	223.974	102.3568	145.3285	533.6282
ERBB4	94.56912	39.12569	73.42251	73.03588	75.80802	81.03479	59.53569	66.40856	148.6009
OXCT2	8778.715	5719.133	2503.52	9568.867	4361.863	3063.754	6211.667	2335.863	4799.411
CST11	91.01619	40.58814	54.34232	124.1021	58.1459	64.50251	51.17669	59.67357	176.9407
SOX10	1257.173	1941.687	179.2463	412.0313	576.9111	502.5996	299.2025	237.5972	1034.166
MYCN	90.91259	74.58234	59.74055	49.00067	56.02103	51.39839	58.70167	37.15297	75.13626
NRSN1	63.95404	43.26661	42.88899	50.11473	41.22329	51.84756	45.39657	60.2551	62.96768
CCL24	55.53142	54.38145	47.02737	72.47646	57.59522	122.1222	49.26416	46.6987	46.96639
WDR63	69.40249	56.62244	60.4797	64.98193	62.04615	65.21777	61.50302	68.54865	99.84902
MLC1	63.27252	63.37939	72.88086	73.07526	52.67254	106.9305	55.31211	62.74482	274.8272
RNF208	55.44703	44.02044	45.6899	36.66832	39.34258	49.66992	44.10453	40.17495	46.67598
PDPK1	75.67987	951.4973	459.6199	1127.438	523.7104	551.3946	311.8679	31.84584	2825.548
C6orf62	213.6447	1113.196	1081.165	867.0428	755.6624	1129.268	582.7626	858.3029	783.3732
HBBP1	139.9539	67.87801	57.18679	90.54041	58.30211	56.89616	50.38509	68.35239	226.5195
ADRA1A	38.06678	54.44218	54.16796	41.64055	40.07795	51.09994	45.94609	39.17306	52.05215
LAMC2	1992.252	1340.905	374.5274	5588.875	122.1384	1275.098	903.4847	557.6051	2132.371
FGF13	60.10722	82.7588	42.50264	43.29186	44.26928	37.96065	44.34307	61.62227	80.76682
CHFR	2073.838	1051.097	906.941	744.537	1413.428	1266.306	1132.231	788.8702	404.7245
OGFOD1	39.88522	41.15066	153.9496	47.94686	43.18555	52.25172	33.48564	53.60514	74.40265
C11orf41	6242.283	3074.125	1616.177	8666.789	1264.102	4494.235	4013.617	2157.182	3449.725
STS-1	4327.447	1347.234	6336.002	3511.395	5878.402	3905.977	4452.351	6394.483	2472.311
SCMH1	12460.94	11964.85	11679.92	12675.03	11573.54	14216.12	12989.45	10920.75	12478.42
ZNF483	68.57472	103.2805	138.4463	112.2778	234.9775	73.47894	53.29544	61.49205	90.27297

NAMPT	3114.604	1231.601	1750.276	2861.381	934.2792	982.1942	820.2571	693.3184	2905.605
CACNB2	62.44147	70.10029	80.76622	50.57119	132.6798	55.74075	73.98918	46.25616	49.6107
ATRIP	4600.444	1337.283	3207.646	3234.85	3409.615	2960.659	3406.959	4231.101	2435.247
SNORD83A	582.763	38.41074	124.9296	65.67928	96.11635	31.87475	67.71058	36.99874	34.79599
USP2	249.1717	459.7508	94.55968	231.8703	354.649	247.0312	268.9435	179.9319	329.4263
ITGB4	281.6013	145.8361	75.86759	199.6502	94.51541	131.3532	71.92027	179.9572	299.4534
METTL8	55.66803	65.84709	59.06496	42.84327	37.18592	50.39156	36.86599	113.3863	56.55304
SMC6	73.17185	50.41913	141.3956	40.4012	198.355	404.0824	219.2768	373.5667	35.82309
AGPAT3	138.2521	107.606	370.6102	169.3733	57.99361	95.82113	184.3662	68.33096	100.1968
C17orf28	540.1938	947.9257	235.9085	288.8215	448.7573	477.5699	208.6861	286.0173	1199.174
MRLC2	7538.898	6904.451	13497.04	10231.01	17718.56	15764.8	16113.01	14566.85	4857.982
TPTE2	422.469	329.1872	118.2262	307.4835	44.487	57.965	64.07949	119.0748	609.012
ZER1	435.3098	238.9783	203.7534	232.2197	457.6191	379.6694	783.1877	1101.313	527.416
MKNK2	51.78537	63.77481	53.38892	1195.023	113.3253	56.64668	104.9947	48.9467	115.8044
ATOH8	41.12987	53.93022	37.79189	36.97709	44.86844	42.68295	111.8386	66.85447	121.6216
NUDT1	607.3297	258.4127	286.142	296.2576	276.6703	271.1975	207.2258	200.5605	814.6947
CX3CL1	67.2	1615.365	940.0722	571.244	1147.881	1174.229	2183.681	2116.138	92.84186
ITGA3	9394.215	7977.796	3747.836	12096.82	756.9243	4315.131	4274.542	3230.63	10868.2
LRIG2	167.9901	135.9952	596.8526	817.1626	322.0288	476.1309	500.1443	482.9489	170.6855
EGFLAM	8552.773	7523.071	8094.682	7433.24	7267.058	5965.791	7706.9	7975.997	9876.172
PARD6A	507.04	647.711	590.9487	306.5722	604.8859	441.7405	535.6847	480.6916	1067.021
BCL2L1	277.6499	142.177	56.35983	79.94102	263.8489	229.659	263.6522	438.1625	192.6642
ZDHHC20	82.43543	655.2288	1279.895	81.14819	607.2006	1432.552	394.6688	655.7314	109.6095
COX6C	839.9173	354.6841	585.0399	815.9597	1142.358	954.1824	1163.26	1623.88	559.2058
BTBD11	89.69209	107.2008	122.7488	79.91155	64.94218	147.4527	53.38912	76.27722	98.52863
SLC45A2	167.4977	169.2449	58.18408	161.7933	67.88094	81.47993	59.57012	70.18423	275.651
GCC2	46.17957	38.0586	81.14143	205.736	82.95011	64.16177	45.18996	72.95988	505.6458
DNAJC5B	35.83596	32.35464	33.42321	40.32168	34.85456	37.20552	37.94519	36.07073	44.38402
CD1C	2262.8	421.1036	1610.578	85.82236	1235.588	3620.504	841.4656	1941.856	1007.574
DOK7	292.8366	216.9888	76.93114	214.992	70.09372	119.4067	70.26779	74.70007	416.6526
MPP2	58.31124	56.06005	38.09418	625.0935	352.1324	646.6608	156.4657	306.076	113.5917
PCDHGA3	40.6942	53.47144	40.92024	33.05054	44.96318	57.51172	35.87361	50.65823	53.84089
SIRT6	42.52473	42.76485	221.7008	36.4389	607.0065	450.4684	257.7227	39.5472	59.90589
ZMAT3	97.90676	1076.079	304.4194	76.70291	251.8931	92.82976	170.4126	323.7657	596.2414
HMGB2	888.1055	813.3336	788.164	1156.154	380.211	1029.94	557.1898	1105.586	1658.066
ADCY9	43.00133	33.53341	50.0827	49.59625	34.33954	33.9737	36.07036	43.42472	42.36001
ELK1	40.92339	57.0398	1114.35	58.68146	1152.283	488.6401	1347.089	810.0327	79.11622
PTPN23	151.6665	60.10714	61.51828	79.16763	95.77854	62.85653	113.0676	50.73786	204.1382
HAPLN3	30.05428	1227.129	572.3868	38.56707	296.0478	36.4376	448.6458	46.97771	49.53265
ATP1B3	1582.742	1910.615	484.1696	1710.946	320.7172	1233.789	460.0806	660.7596	268.7005
UBE3A	122.8714	92.55119	60.15646	85.97599	65.02808	86.56398	50.19343	70.64429	159.9315
HEYL	4061.217	3586.277	2484.02	3812.505	3184.191	4415.075	5288.072	2883.615	4512.708
HOXB4	128.9354	140.0636	77.24977	147.9298	178.2142	121.8944	98.82586	171.9085	159.993
ZNF24	81.78535	674.9167	61.76129	499.9785	156.236	65.91632	115.2461	160.8918	255.5163
LOC440742	71.76746	104.4409	57.033	52.6874	60.99116	63.66592	51.26293	73.53155	139.411
TESK2	102.5748	60.82462	233.5273	75.10922	154.5762	94.22733	93.8265	69.34355	65.59438
ASB13	31.46706	36.98354	482.3851	33.96979	855.4185	61.00629	199.9605	140.4209	44.58929

C14orf182	61.15933	57.56287	49.65419	36.50562	45.5695	44.27507	36.73597	38.91872	67.54539
KHNYN	34.73918	46.15518	45.53289	63.33032	461.7115	60.89108	933.2321	540.3644	53.7122
RGL1	5370.13	5029.606	6878.75	2300.08	4854.154	6012.43	6021.234	6976.855	3257.07
TRAF7	42.27442	45.7589	50.12092	56.21981	34.99226	56.94382	35.1408	37.50235	54.77516
CCL18	109.3666	339.4963	84.52686	119.8011	107.4428	55.93959	81.81849	71.23543	46.53742
ACSL3	147.4337	89.77171	289.4036	108.9089	155.8714	145.484	96.50524	68.87322	69.56197
PTF1A	33.91541	34.69472	37.11749	37.31557	34.07132	32.85202	35.00323	38.41163	93.6976
MPHOSPH!	1756.801	145.9082	94.1279	128.0333	290.4083	114.4368	196.2705	235.6487	224.9865
ACTN2	625.2175	35.69857	564.2352	437.4989	269.4974	244.9162	835.0665	655.5026	43.96371
OAT	292.2026	623.48	243.1561	205.0038	175.8272	566.7717	169.0487	92.1002	347.2012
TXN2	198.5452	296.018	299.4391	281.733	229.5479	278.4676	237.876	391.5225	195.7235
FLJ37587	503.2392	301.8241	176.2155	285.0333	435.7745	528.3798	192.4315	116.9107	42.63894
SPANXN1	49.95537	58.73878	40.50642	55.12545	34.2985	36.66162	39.9874	39.636	48.79619
PLAC4	217.5603	66.91118	78.409	125.8573	94.12507	118.2256	68.52964	138.2552	315.9932
ACRV1	92.44085	143.2582	67.26587	176.8967	163.5099	70.1815	96.26688	538.3139	147.0759
ZP1	108.5891	35.43573	45.82365	39.19223	31.55371	50.92872	33.27493	35.97279	80.2765
GML	69.20888	72.76829	137.803	53.46526	55.17793	41.12507	42.44794	38.27808	183.5939
UTX	52.16952	33.65271	1074.309	42.09502	727.4646	1188.95	559.1685	37.8618	41.76825
ATAD1	73.19622	692.4537	124.2145	67.59988	279.5793	257.9088	361.9242	192.9147	76.11079
CLEC7A	3903.821	3590.492	2368.694	3003.872	1062.099	2448.433	1848.165	1275.097	3312.689
TMEM120/	268.9435	127.4322	1236.284	1752.484	2341.116	345.84	1710.041	1618.875	197.1013
NPSR1	1050.786	542.6394	176.3014	359.9065	146.3926	96.35538	98.00226	50.44951	175.0539
SEMA6D	413.5982	219.2132	112.0169	226.6939	90.29308	178.3697	84.73882	160.9611	532.0717
ATP6V1G2	187.0625	152.1639	74.4352	156.7848	61.10446	92.88602	58.87434	86.17281	381.3354
C5orf21	1345.316	569.4688	1252.12	665.9786	2303.831	1292.824	1412.035	1306.061	50.35476
MED6	16064.56	18290.32	16974.64	15809.63	18300.53	19811.02	17241.96	17860.46	15425.64
CPSF1	16111.7	15847.72	15844.28	17618.47	12309.74	15221.51	15303.19	14398.34	16278.73
MRPS31	606.9427	533.3958	1429.384	736.783	1286.722	1253.084	1637.379	1568.164	46.73468
EFNB1	2907.478	1347.582	2638.423	3823.082	6439.471	2764.114	7832.784	5953.018	53.2298
HSPA1L	37.79037	539.0628	230.9983	773.4477	147.7438	36.68836	892.8833	356.8517	46.16049
TEKT1	229.6901	148.3999	116.3951	196.4413	203.7606	163.0962	97.14234	92.50957	147.9225
FCHO1	131.1383	104.8234	68.12507	110.4643	68.01003	89.06842	221.0772	70.35641	151.9191
CHD1	1525.797	1542.711	1859.521	1737.537	1289.204	2366.978	1199.782	1464.006	808.7014
RASA3	332.4958	197.4212	330.2213	457.9862	169.098	168.5621	211.5998	112.2374	450.3267
SLC34A2	67.88972	58.10424	73.06424	116.1856	42.65947	58.10831	51.67005	65.36568	65.77798
IQCA1	33.13165	44.07484	43.44871	72.17561	43.73199	34.53724	167.6393	47.04121	36.53122
ZDHHC22	322.4714	184.8049	96.3642	169.4355	99.02016	149.8874	66.35283	114.1369	205.1584
TIGA1	166.0546	109.7206	1454.454	59.2664	2350.585	1433.564	2284.156	1617.679	85.27935
ZNF589	33.77512	1035.741	3423.068	1657.509	2005.583	612.5092	2267.546	2370.153	34.91299
THRA	281.9116	214.5663	99.17713	204.653	98.30999	145.6999	77.03523	74.01257	392.376
TMEM175	7382.596	5895.794	3367.026	2826.001	2971.925	5163.229	3306.715	2218.05	6641.141
FAM193B	1466.705	2403.208	1986.048	1599.31	2481.253	1558.943	2300.726	2980.662	2240.211
RGS7BP	53.50003	42.39756	46.49046	34.7919	43.84201	71.97137	44.18478	58.66138	54.68931
HERC6	65.4226	128.4	1001.043	148.5249	170.2626	1020.054	500.5415	755.6015	1788.042
KLC1	182.6216	714.4662	989.3626	446.4692	1752.289	1725.449	951.8011	995.5168	67.10175
SLMO1	34.12035	32.79528	36.78954	42.88123	78.35891	40.93113	56.8377	103.3731	41.45917
RAD9B	54.4403	56.51525	120.6132	72.81715	43.05762	53.6398	45.55682	115.8036	40.25994

MCTP1	128.9203	52.36623	171.4374	73.08015	247.6561	52.35614	302.088	238.157	93.31246
ZNF197	378.457	205.0288	261.8828	238.6051	246.1651	165.7574	208.5255	141.8425	487.5366
CECR5	4970.028	7364.725	8209.884	7735.468	9899.872	8693.989	9210.763	4699.487	2122.672
FKBP3	96.56243	264.6652	157.4173	205.6546	125.0629	186.7515	82.12263	147.784	63.89087
RALGPS2	80.8929	35.46354	198.7898	44.88811	38.11914	223.2951	38.06027	40.77899	90.22478
C15orf48	50.25046	46.39957	35.00065	35.539	35.3354	35.88395	46.88455	43.10878	41.68649
ZNF280B	163.7537	108.2712	52.16095	99.68876	58.80017	110.8047	109.043	68.87958	81.87812
GPR157	45.87825	459.7867	45.34809	47.2343	34.58194	36.25667	41.8515	52.62405	99.99029
MPV17L	785.3778	280.5591	146.1655	422.8353	126.2221	242.8323	187.3668	36.38196	198.5898
TRIM32	47.9884	52.9516	39.40057	46.52388	38.94413	48.55519	39.89061	124.9507	46.18425
GJD4	104.6337	66.66286	54.96947	77.80077	49.09708	46.98663	52.93711	51.08883	229.2006
CASK	164.6978	2083.42	911.9171	1894.724	1216.369	716.1156	949.7833	1285.171	63.45213
LSP1	3383.933	3060.235	2227.378	1670.469	1640.151	1268.68	1640.235	1206.197	3499.818
TRIM6	62.90853	32.61942	43.55751	38.37397	35.01953	269.1537	30.56254	94.56805	35.08699
C8orf15	88.7133	343.4355	133.1432	107.2265	127.3145	146.0301	83.3151	81.26408	733.2632
UNC93B1	65.0155	35.36411	36.7894	41.06895	43.31213	65.25001	37.28698	59.62773	61.74901
MGC70863	1412.955	177.2529	108.1899	186.8098	102.5421	129.3412	207.2995	100.6719	346.1762
FAM134A	473.9897	325.8188	152.4308	282.1183	186.3251	203.3405	178.1983	278.6785	598.845
MURC	38.68585	415.4316	149.289	438.6925	178.0775	278.5463	198.3909	55.05713	39.31554
DCX	104.6117	85.57815	79.0459	176.7909	53.92016	61.87972	55.2589	59.83632	85.92664
ZNF818	34.61995	55.60714	34.71022	41.2194	41.03373	39.30011	35.79898	53.25931	51.87953
ACO2	660.1994	482.8028	160.4466	477.3454	93.91604	216.9644	191.5548	210.3311	90.96205
C1orf58	46.88081	660.4997	810.7405	51.245	814.0669	871.5496	434.9226	1004.037	148.4763
RANBP2	56.40678	42.99884	51.76126	66.4645	88.87831	38.29511	87.33958	47.21469	102.01
CLC	323.5032	134.298	94.96836	156.5762	110.4469	112.2739	81.31891	111.195	291.7456
LENG8	4325.357	2669.288	805.5718	602.5191	894.0569	1514.437	802.5562	1098.724	2569.389
SCAMP2	1192.708	57.49836	52.27388	461.5497	43.23511	56.00679	136.2721	70.24652	51.40327
CT47A6	312.0556	199.9632	93.98804	227.0135	83.23737	143.4679	87.42946	135.3527	343.6362
KIF7	62.66733	1808.702	1081.032	3679.385	1422.216	1616.88	2616.022	1003.328	50.85714
HNRPH3	119.5156	802.0076	685.4567	117.1836	980.7919	332.3893	657.5953	812.8441	166.806
TMED2	48.13406	58.27528	583.766	1016.021	388.8978	255.6	413.4735	416.2409	98.50865
NFIC	40.94858	38.20252	54.55937	67.66381	43.21936	51.43782	33.66703	55.84412	39.62595
DNMT3A	58.46788	247.538	54.40076	75.59906	38.81339	40.22606	86.13685	157.6521	718.7239
ZSCAN18	540.7507	338.3259	176.2259	345.7655	215.4307	244.6525	207.435	278.9	793.9825
SCARA3	4573.314	3759.346	2664.747	2479.504	3805.376	2534.538	1028.093	631.1106	3548.419
EPHA6	101.8693	127.4769	60.64105	129.0892	68.55159	82.57861	59.37613	70.51029	63.13887
OTOA	51.11897	44.32686	33.63678	56.39324	44.79029	41.63084	35.96335	37.53569	66.43433
HCG2P7	265.5585	253.4873	89.31305	152.9615	78.05426	157.8775	71.55676	100.7442	328.7561
P2RX3	135.3963	70.70622	67.3252	100.6842	69.67844	96.61741	70.16704	68.51347	103.1232
SMEK1	36.77978	56.83155	139.4059	61.49636	50.06461	197.6716	54.58232	70.85778	41.50215
SLC27A4	438.6028	230.745	106.7055	238.8504	88.86263	162.6176	78.16177	132.7154	574.1642
NUTF2	212.8672	405.9454	93.51483	440.6401	67.46204	90.08427	70.33171	159.7279	181.6655
TRNT1	847.2568	992.4688	392.0417	404.0298	347.1994	593.9556	306.5161	439.444	322.8532
TOM1L2	17269.93	15640.37	10900.33	16507.61	9904.87	11866.47	10641.61	14925.19	15980.03
PRDM1	37.68797	231.0668	221.7312	275.4502	277.2676	389.0399	239.3593	182.1464	46.93927
ABR	139.8959	1467.917	682.6895	1735.613	191.195	461.4221	137.5252	990.27	141.0964
WDR19	397.3232	591.7058	3110.944	507.9072	4838.277	2808.472	4652.926	1813.364	411.6946

ZCCHC11	47.82528	684.6571	357.467	36.97905	140.6457	331.3029	228.8617	514.1844	1784.649
PTBP1	22824.22	23711.36	25614.2	24809.95	23619.96	25134.9	23578.92	25532.88	25583.53
MORN1	43.00806	41.43091	42.0435	35.97485	47.40398	36.41302	39.06976	33.56472	74.00584
STK24	4080.498	4301.577	8289.7	5772.303	9720.387	7645.11	8003.264	8206.828	1980.363
RNF138	385.692	272.6308	233.8883	274.7132	137.7724	220.7656	111.8694	97.2972	498.7902
ZNF502	1099.639	51.34453	398.1591	1171.433	183.2297	333.2221	368.9541	399.8614	82.43211
PCDHGC5	54.75904	50.16447	47.24718	37.06972	38.77029	35.27576	32.723	37.59638	44.10744
PIK4CA	900.9355	997.5194	1629.92	1017.518	1751.576	2323.237	2428.172	1403.771	884.2478
CASZ1	12119.49	12831.16	9823.962	11805.08	11943.4	11156.06	10097.53	7485.54	11749.41
PHF17	435.6039	224.7911	157.146	251.3484	97.35044	389.1725	110.7156	85.05373	453.7217
NFAT5	65.82378	65.49171	46.15641	50.38518	39.1174	68.06637	43.73698	51.39679	99.39875
C19orf25	198.3387	165.3853	1379.292	117.9059	755.0541	961.9868	1373.381	1216.791	286.5209
SLC7A11	43.02747	35.26636	53.84379	416.3944	148.3801	43.82104	211.5523	96.51965	70.20063
PURG	249.0751	144.913	79.96399	68.22047	68.87531	94.97458	65.17108	61.8321	87.65575
MDM1	1131.454	1210.293	100.7818	676.3431	106.3097	177.2146	276.6502	260.0531	640.536
ERP27	230.6322	904.9634	2938.903	850.5035	1183.945	1903.436	1540.488	1281.708	42.1845
C2orf66	164.6881	68.2792	62.76017	116.5759	53.62586	99.60345	62.18671	339.9679	208.8952
SLC23A2	163.908	83.49732	43.19532	74.76292	51.34618	52.77744	40.35962	49.21669	54.99019
UTRN	433.7061	2235.741	1164.509	278.3962	1067.988	182.9113	557.0902	636.4931	614.8311
FAM136A	3057.58	3067.768	4964.441	2441.018	7549.977	5074.173	6676.941	4992.212	1872.178
HPS5	146.8405	2091.49	3487.042	59.82814	2398.751	3408.024	2497.455	1744.204	1310.803
PSMC6	115.3458	466.7366	293.7422	51.95844	576.1796	349.0699	536.1934	437.1224	100.2984
RIOK2	94.2341	365.5066	136.1909	61.26952	97.5108	222.2586	127.6629	182.3553	37.71572
SPAG11B	51.57479	43.53041	32.9435	41.94062	47.69884	52.58854	38.56181	44.48582	63.32596
RNF181	537.2267	853.6468	4233.603	2113.268	4635.89	2914.996	4786.161	4762.247	1095.812
PLAC8L1	50.73357	81.16396	55.10248	54.02541	58.75775	51.03296	96.35515	45.73743	74.0575
SCGB1A1	186.327	85.02071	67.9127	112.2889	66.26141	4008.022	270.2668	435.423	135.2109
GPR1	115.2569	101.4285	101.2337	202.724	100.2575	148.3978	111.7015	125.6902	597.0342
SELV	43.33703	85.30743	50.16137	69.2508	41.72785	61.92802	50.91338	55.61544	66.52588
MRPS22	1356.148	1661.832	1668.604	1604.125	1542.133	1519.627	1684.569	1944.886	729.8383
DCLRE1B	112.5073	32.41221	321.3968	47.35927	241.3599	43.10826	43.82228	334.9037	45.00371
PRDM16	35.89493	40.60084	37.22792	35.79126	35.65577	47.54353	43.41261	37.18696	57.66917
SCARF2	50.25939	38.83018	134.6754	418.2792	305.9183	44.46342	246.5163	113.3215	113.285
TNFSF14	96.84604	51.54704	99.33687	81.59881	52.40419	76.53806	58.29892	97.30066	357.2763
CAPNS1	6933.9	5255.358	10091.46	5982.977	14350.87	5238.979	11918.29	12214.96	6450.868
RPL17	7755.58	5183.005	2749.723	5640.062	1835.619	3720.756	2320.874	2078.079	7724.142
KLK3	250.7851	136.5663	113.0232	141.6891	90.84935	121.9697	72.84232	82.87218	194.6423
TNFAIP1	1505.247	1515.27	1207.116	250.5032	1797.079	1643.926	1017.404	884.6158	559.0077
BCKDHB	192.3514	168.7609	77.4213	113.4058	66.06128	118.4796	59.08899	88.78113	272.4158
XKR6	36.90542	35.1438	38.97083	35.10784	106.0014	36.32812	74.66982	97.4986	34.04467
OR5R1	59.83221	75.91301	43.70385	68.8687	45.36339	61.65407	48.63013	44.4557	63.16847
DSG3	1426.204	793.9899	848.6797	1630.128	1987.021	396.4811	1053.261	913.2581	36.90325
TSPAN3	1846.463	1062.507	619.9893	107.9129	748.8444	1326.437	583.9282	598.9177	46.44099
RNASE9	41.56995	38.6355	34.17279	49.16977	39.59208	33.08534	37.51014	39.12351	36.9598
TRIM27	55.58305	98.07166	48.61301	82.01878	43.63229	64.1117	53.93333	60.13423	56.48695
C1orf124	185.1157	112.3251	78.62582	359.3395	237.936	246.03	222.5011	74.24928	974.0236
EIF4B	2720.028	2606.489	1655.461	2611.468	1199.215	1243.658	1194.062	577.8132	1083.447

IFT81	111.3254	59.25559	42.49403	185.3208	61.67311	74.22375	42.76599	47.60448	96.04063
ASAH1	242.0472	94.00262	79.81969	96.53716	66.45916	60.54221	67.23161	204.8963	152.1058
DMRTA1	34.95909	43.18961	44.73688	39.15219	101.8611	33.75663	35.10009	34.73368	54.34969
FLCN	1251.086	1240.553	110.5105	246.1046	226.7084	194.0374	236.7664	264.74	423.6606
ASB6	35.82766	38.32301	31.62486	38.48239	33.49828	49.23699	163.0065	36.11528	48.30563
ING1	148.6528	100.2209	74.29998	122.9	72.48953	62.36497	73.95349	102.6618	193.5018
ZMYND12	54.81614	44.51231	59.51836	56.89561	159.3205	57.07763	137.3472	56.1857	58.72598
SIGLEC10	73.93675	66.26456	1096.876	33.70096	272.5356	35.40111	507.0179	467.896	46.01208
INTS6	542.5624	801.2691	773.0547	310.0128	599.1793	725.2578	391.374	536.1921	1201.29
ARPC3	2321.296	1900.359	888.3015	2473.076	1040.766	1508.23	921.9316	1585.858	3341.159
KIAA0427	94.4828	73.32012	143.4262	109.3054	107.1628	235.1	88.90709	119.6417	141.8253
UGT3A2	47.80635	70.47758	36.90654	1437.146	164.8942	37.99304	180.1085	32.78782	38.36057
C6orf59	39.07472	874.3125	43.22886	36.68917	36.05386	80.1187	213.3294	45.57583	49.40825
TIAM1	65.17616	1379.371	2998.74	112.2921	3234.825	2054.951	1333.981	1660.237	201.4226
SERPINE2	24229.29	17176.61	16800.39	28556.74	23912.06	29044.41	27706.86	22059.97	16414.79
KLHL18	156.9848	92.33984	113.8348	67.24906	113.1232	41.79672	71.58927	80.15929	627.4019
RET	55.67585	56.36964	150.342	45.3018	127.3316	66.2719	301.84	170.3306	101.4996
TSHZ2	1102	46.45827	1266.219	60.05219	2126.426	699.257	1644.796	1498.143	1847.265
ARRDC2	2222.329	642.0706	1452.247	104.5727	953.7435	82.06505	1587.023	916.9686	124.2821
DSP	1623.418	2516.742	2056.282	3982.139	3912.822	2363.557	2595.71	1475.357	102.8651
PRKRIR	170.6591	110.4392	437.1778	140.9945	1384.434	83.64166	1376.151	935.2479	149.7479
GPATCH1	94.3567	286.7967	112.3505	43.66733	154.1307	148.8791	182.9414	131.1845	38.19648
METTL3	175.1594	92.71764	563.4005	161.1053	258.3546	104.8882	209.2465	225.1089	102.2874
UPK1A	23084.17	1926.648	4180.321	14316.58	6641.185	5526.832	5992.268	4508.467	9802.412
FANCI	815.4862	823.1591	689.1605	1238.752	808.7482	542.1945	789.6089	613.2552	945.6831
ZNF804A	84.79801	97.14416	46.24076	109.8788	69.46693	69.38303	45.54204	57.88055	72.9781
AK2	1122.314	576.6567	453.6329	1046.003	127.2011	393.4756	598.6778	1084.781	40.38149
MPDU1	113.4541	87.56087	75.71833	55.61442	357.9998	46.96827	173.2803	518.9094	76.70783
SC65	390.6583	244.4161	125.4562	260.3933	264.8983	169.9574	862.4019	327.6409	551.5026
DGKD	1335.457	47.7083	33.50512	34.72837	33.29512	331.2851	90.76122	32.0204	325.9871
SARS2	6761.926	7608.919	11574.59	5311.502	10501.64	6797.37	9943.693	13430.44	9379.044
CDR1	63.30635	61.27712	56.94025	40.00805	44.0764	46.83062	52.08197	59.84053	111.3371
ABHD5	52.36539	37.86214	425.8306	42.14262	1218.308	401.8254	871.8232	373.3652	41.49112
PRLHR	58.14275	67.94168	61.95189	55.66577	42.19539	43.65392	44.86464	48.23658	46.67764
OR2M7	52.32206	113.2465	49.47093	40.29116	45.63602	54.80598	45.22543	41.4527	69.20928
CNNM4	104.8976	90.17753	76.66835	64.68433	146.2526	67.46068	101.7101	73.81645	146.9298
BCL2L15	47.70718	58.39522	47.5879	43.35653	38.49502	49.07472	46.53387	39.57443	55.29545
RFC2	65.49627	121.1061	123.1507	55.27094	108.1041	109.9361	83.61057	115.669	42.22881
RPF1	47.13743	125.3181	237.2867	249.7778	273.736	219.2357	305.1288	238.959	40.2942
SNAI2	340.8856	177.2468	1613.698	1456.509	1349.923	2100.969	1496.058	1179.94	228.7723
ZNF323	5338.909	5543.818	4794.942	3180.413	4479.935	6850.07	8049.976	8498.281	4326.176
MRPS21	4034.003	1415.741	1954.769	2950.346	1344.681	1061.591	1527.287	1382.527	37.38881
ANKRD34B	35.12387	34.13562	43.16051	37.22889	31.93737	35.81524	34.1939	36.64906	33.66473
METTL10	148.0652	246.3136	124.8939	172.5775	104.4595	176.1565	137.2444	100.8193	320.7137
ZNF780A	54.66228	54.5346	48.4073	470.4004	64.18035	43.74168	126.5334	124.7292	238.3559
43527	994.0253	371.8577	51.67216	57.44342	114.6443	76.28282	187.1076	44.67278	58.76345
SF3A1	170.0788	70.17577	74.60869	123.5321	58.63881	73.33114	66.6077	56.61446	125.9905

CAPG	626.878	340.8476	120.9322	539.0944	203.7238	83.78934	69.92441	72.87818	912.2038
COPS2	42.48993	373.6591	355.4709	668.7793	229.2305	309.8626	140.248	215.3378	56.12613
TAF1A	67.42847	34.3423	139.1343	38.60999	104.7066	255.9587	150.3524	40.49992	136.4519
GSC	77.04868	60.09713	54.84237	58.71208	55.6614	69.36288	54.85124	68.92042	143.0092
HRASLS	46.31748	37.12175	385.4236	41.70388	481.0844	65.80414	169.1088	199.0841	50.19484
TMEM159	164.2329	63.08729	233.2021	61.51764	794.3958	73.44775	561.4114	307.7969	371.4646
CHST11	171.9265	120.075	61.51923	147.7178	63.40539	87.24551	52.87168	61.49168	48.9586
ATP2A2	119.6662	1219.402	1152.943	2041.203	940.8607	592.2787	1075.197	1783.242	1366.724
MMGT1	51.83235	53.65984	37.79326	37.65872	32.99597	58.12981	38.74416	48.78095	53.61314
C9orf93	75.0966	44.74556	55.62107	67.42422	46.60854	40.98414	39.81474	47.54171	73.5132
MYL1	84.21942	51.78633	59.32832	57.15837	36.14407	43.9535	52.43955	77.95845	40.79161
ROR1	100.4155	51.97903	134.156	66.97134	61.2972	63.18197	47.52023	67.3091	63.21285
FBXW7	2631.769	2151.215	843.4711	1222.236	554.8565	1017.924	434.303	677.7935	2556.493
NUCB2	1965.899	1363.364	2873.126	1099.071	4246.487	1711.081	1648.724	1835.199	727.9172
MMP11	760.5447	61.18348	1129.91	4456.405	441.6375	2398.119	1060.928	1025.992	2926.896
GTF2E1	57.22609	48.88727	304.2238	601.733	42.90777	44.47329	252.843	49.62074	138.9585
ELL2	38.63507	724.8741	577.3001	1580.604	502.4428	289.7812	355.6706	273.3395	42.02889
RPE	163.5678	86.69598	69.73032	142.5444	43.41565	45.67437	40.84172	45.51514	92.1039
ADCY7	292.0007	233.1191	79.13512	280.2654	95.39076	172.1999	111.3705	75.93431	506.3914
ZHX1	57.13587	78.72635	83.92747	52.16953	88.49677	201.9001	118.5409	56.20369	60.36877
LRRIQ1	46.97754	40.24765	42.50496	35.05895	38.30944	39.17177	121.442	54.9081	67.74576
C7orf54	7738.179	9513.822	9285.821	4143.535	10646.26	9457.718	9484.739	8691.478	6220.185
PDE6B	53.94498	50.46578	201.3537	50.4007	40.95092	43.29982	36.96611	47.85941	82.5059
CDC14A	260.9257	1780.237	745.0204	837.105	47.88762	428.3458	417.4318	221.0487	118.662
GALNT10	117.1398	71.07642	55.01729	65.89765	62.36128	96.16272	45.32857	56.18181	60.03917
STAB1	44.96885	39.04201	437.4946	34.25565	1077.865	52.08264	701.7059	1362.838	50.42344
CDK5RAP2	8203.338	7752.776	7442.794	4803.37	5631.394	9896.859	8312.688	4236.071	6791.366
RGS1	50.70421	331.143	350.844	68.41626	310.4454	299.5843	324.1068	189.5494	51.18925
DHX9	52.23075	250.2084	143.2979	132.5097	132.9811	484.8997	214.6793	158.1088	89.39101
NRF1	65.6624	56.50761	201.5302	68.08242	241.441	38.26051	193.7485	290.6526	362.7063
NTN3	341.9037	273.2959	119.333	201.0965	135.7325	166.2735	109.3882	183.0612	309.0138
KIAA0319L	153.4415	59.54397	525.8276	1213.734	242.6777	71.27103	240.1903	424.868	171.4997
BRUNOL6	1803.298	70.3956	639.3384	893.6517	844.9025	556.5562	797.8922	1103.397	257.0508
KRTAP19-4	43.09417	63.1139	54.2084	60.26678	45.77517	44.92449	44.96042	72.10802	82.41837
PLAU	395.1772	216.599	1336.377	771.9583	1060.34	1791.608	3606.994	3005.866	485.6003
TNPO2	9622.351	8050.688	5829.688	8299.528	5215	8228.724	5367.668	6455.496	9637.253
C17orf55	632.0341	377.074	425.7305	408.2087	226.7753	311.5449	281.8304	383.087	894.1324
KCNQ1	39.21991	41.56326	32.94586	37.34471	33.9786	39.6579	29.94045	32.19399	53.03811
C1orf110	10048.79	6468.389	230.9072	2293.207	12200.55	7427.488	8695.749	12264.82	19445.59
SLCO1A2	44.04663	42.69198	43.86913	59.50986	37.68826	44.46281	43.54417	47.92781	50.61182
MYLIP	37.06922	45.40788	343.6382	34.40018	909.6998	40.8468	456.4203	54.24541	1388.39
ZBTB7A	460.7374	212.1344	105.339	250.3449	182.736	174.5838	200.9827	106.0366	970.4596
SLC39A10	62.3353	40.07038	33.17182	46.80363	37.07599	50.60775	34.03286	35.31278	54.38146
PNLDC1	50.56643	52.05501	42.85527	41.61979	107.6761	33.74349	44.76689	121.9742	44.48286
CNOT7	1555.907	688.4617	1448.484	1470.381	2052.951	823.1363	1084.926	340.0843	2214.123
STAU1	55.36185	592.0455	670.653	76.02239	681.2524	41.60397	470.8296	431.8606	55.64261
MAPKAPK2	1867.97	32.70434	277.6399	36.47816	33.84557	33.45305	33.50653	33.16593	40.95118

GOT1	11648.75	10986.18	10464.26	9323.832	9091.213	9369.563	7205.763	12137.25	12386.7
ATP10D	44.71503	53.40508	171.0763	302.6902	332.4247	710.9863	152.9402	270.1542	60.89207
USP9X	48.72166	54.14886	169.6579	50.87877	839.2093	976.4787	719.643	490.2607	65.01257
ADAMTS19	37.91671	33.31796	39.82444	38.2144	31.91649	42.05444	39.66476	39.44008	52.56646
DEF8	185.8646	118.5474	2089.57	566.5386	2164.389	874.2365	2040.241	2032.558	269.7008
SLC41A1	425.244	62.16365	143.4002	250.9461	114.7436	86.62173	186.876	154.7959	48.75665
MGC19604	48.87325	39.01877	46.74862	38.47012	34.71812	41.02378	39.62088	49.01824	35.5918
ZNF704	169.286	119.4544	128.4057	105.4261	58.38966	271.3926	154.2085	62.91713	104.5277
MGC33407	49.31825	50.67399	39.08393	53.09852	33.00789	45.50687	47.20887	49.30596	41.12601
TSPAN10	3252.284	4470.284	2476.675	3826.652	1500.132	4089.02	3526.423	1877.41	3083.699
E2F6	153.9332	577.4703	986.9154	415.1162	1459.726	1635.492	1239.361	1627.352	101.5607
KATNAL2	49.56327	40.48603	116.721	32.09042	63.97943	219.3221	78.68443	78.92197	160.4295
OR2T27	62.12339	59.44491	40.71192	69.92615	54.82846	54.57196	42.59466	41.23322	34.05705
DUPD1	133.1634	71.99427	64.10733	62.64599	46.01965	59.86247	38.03853	42.02644	43.83986
ASAM	458.4581	246.4013	68.06482	39.24995	46.53702	104.2789	57.40624	61.47485	62.4054
PRPF38A	54.57464	3021.894	3466.565	2278.904	3999.86	2431.259	3405.362	2967.959	107.2433
METTL7A	3361.942	4397.227	3616.296	2429.99	4689.168	5665.463	4941.514	2799.973	2988.855
SPOCK2	2764.178	4464.569	6099.256	911.7224	3471.176	6088.625	3502.277	2959.714	3627.566
TSKS	36.66409	33.57513	56.38409	647.4904	38.71449	41.35115	88.66338	274.7221	37.85306
GOSR2	975.004	432.1629	333.1425	146.8267	303.8601	214.4035	242.8829	454.9626	49.12421
C21orf57	348.6339	217.8052	111.6075	197.6412	103.6121	158.8961	199.5069	117.6807	487.2305
GFRA2	3211.452	2217.179	1303.589	991.7727	410.3578	1461.579	869.1568	917.1598	2637.311
SNORD28	484.541	274.0672	120.1357	316.3596	99.57705	47.42235	89.32427	118.431	40.88298
INDO	445.3351	307.8563	349.7531	39.14192	117.2058	199.3093	216.7943	129.9174	40.60242
ZMYM3	90.84381	117.0531	69.37914	142.9617	65.64201	71.67923	62.92236	69.47069	185.6274
LTBP1	37.74428	36.37566	192.8832	33.52452	247.2894	40.96614	58.12138	35.96791	33.60227
GLT1D1	195.0503	104.454	108.6512	113.2759	70.29583	123.5601	79.20097	95.32866	233.5659
CCKAR	119.5712	527.8279	59.38161	68.98595	274.7932	647.843	93.94656	176.7628	88.45228
RIT1	37.69897	39.24865	206.6305	864.3538	781.5014	49.33221	483.342	39.29219	37.00797
PTPRS	472.8107	218.4935	75.69036	197.4356	59.51603	160.9556	66.42291	68.36884	655.6168
FBXO48	54.18136	44.79623	40.98637	32.04728	40.87585	103.1385	64.07962	33.33852	45.34821
LOC201175	3881.995	6474.389	3951.784	3374.856	4874.738	4744.07	4023.566	5211.264	6763.502
ASB12	171.6971	92.63621	349.7021	88.48997	70.44501	73.80637	404.5617	174.0762	105.3768
ALDH4A1	15431.39	13783.57	12285.56	13264.23	11274.52	14170.93	12329.1	11142.63	13136.33
C8orf4	3062.506	4595.501	4784.565	3451.057	3528.364	2027.49	2130.106	2600.231	7909.124
TRPA1	39.47961	62.35744	39.20936	81.45901	51.77199	51.5215	46.56347	70.10946	60.75525
PES1	1099.014	1167.775	657.7424	1077.33	488.9178	687.7582	507.1355	781.5013	1304.18
BNC2	593.3936	291.4572	196.0611	267.9637	126.8554	183.4722	149.0613	53.26987	126.7376
NMT2	112.343	379.8248	247.2776	620.685	122.3209	272.1412	167.7476	220.194	73.07122
SOHLH1	58.55666	59.70242	33.64945	38.63511	37.22371	55.24333	36.01136	30.90051	34.70395
SEC24D	72.47342	257.9334	144.783	93.50272	122.1815	114.9917	148.7643	102.7449	73.13377
MYOT	96.43726	109.0542	106.5086	236.7924	127.7503	180.8717	221.9386	234.5586	50.96616
TCEAL8	729.7835	736.614	724.3615	624.9196	647.5718	816.6209	792.455	562.0931	806.0811
STON1-GTF	59.48511	47.90693	47.93653	58.1184	55.20286	54.48264	42.65868	39.24023	114.0874
TRPC6	34.39775	273.6219	150.64	43.06641	217.0998	241.9241	142.4137	310.7263	63.25919
ZMPSTE24	3751.669	3196.556	2770.791	3318.869	2408.981	2651.227	2389.378	2602.099	4089.756
RAB11FIP1	109.3242	79.18301	59.36765	120.791	59.67108	93.94972	49.45892	63.03693	71.97549

MGC3020	1923.715	62.92904	806.559	35.26908	349.9401	55.4713	836.1597	990.6511	56.10293
GPR87	1664.757	2189.75	3004.154	3174.239	3742.214	5343.368	3177.652	5005.502	898.5895
IP6K1	282.8632	159.882	216.3901	567.53	90.1797	145.3013	79.1528	103.4401	268.5526
RBMS1	66.33477	49.40921	130.8455	578.7618	147.9076	209.0245	38.732	196.6328	37.89991
C16orf59	34.87384	39.40268	1539.673	36.59921	2277.937	1034.837	2290.261	1509.197	42.27468
C11orf73	127.3376	81.04549	63.92424	98.86619	92.26138	58.45493	107.9394	92.91367	222.9821
ARSA	43.38554	46.07852	67.7999	37.01417	227.5457	52.83812	45.8069	49.77541	67.17985
OSBPL2	1495.254	1483.265	1753.606	1020.968	2478.33	2509.707	2231.715	1882.982	1007.495
DNAJC10	325.9574	161.9041	151.4083	149.0087	146.784	84.59879	85.10206	69.00624	217.6181
FGA	328.3471	215.7299	97.84999	226.9698	102.9046	149.4268	85.54166	126.8389	451.2291
EPC2	109.077	70.68781	61.73756	146.6492	71.47466	70.77335	54.25788	54.2789	79.45812
DUSP5	3235.679	2132.371	3689.691	2104.263	3807.667	2785.233	2641.681	2990.257	397.3896
ZNF304	522.3375	295.7712	151.1264	311.558	243.5282	280.8647	243.7399	232.608	791.2513
CGI-96	316.3035	180.821	97.84254	157.4198	161.8154	139.634	173.9924	111.8094	289.5523
SNRPN	41.83471	31.91061	320.3945	40.26328	193.0599	259.9143	56.20197	298.8937	35.07873
RUVBL1	193.6378	289.4454	210.7791	627.6032	248.8514	399.109	283.6936	416.8948	224.5278
ARHGEF11	43.35685	54.70109	31.46135	51.5241	42.68806	36.25167	35.25262	144.7872	55.40369
ARMC8	123.1064	78.85992	52.57447	156.673	48.66599	50.50595	48.78601	60.7084	62.15111
PARM1	50.28884	89.99967	47.74373	43.94107	132.3362	40.12436	70.0477	40.94479	52.13977
PAX8	37.76334	40.292	36.57644	52.9067	37.19093	33.52668	35.06517	54.27759	40.91147
PCGF6	133.3852	245.9469	139.4443	214.3537	171.4464	216.7834	167.6696	200.0671	104.0327
ATCAY	64.3646	102.1169	74.67279	111.3118	59.47247	105.2835	55.42519	55.46629	146.8106
KCNH7	42.22462	52.38145	34.84986	36.07937	34.65893	40.25766	37.27103	35.0063	37.52738
AK5	42.22867	36.62463	55.54917	56.74149	45.0941	61.15769	45.64443	58.14075	35.16854
STAC3	231.8178	87.47158	99.77966	149.7705	74.4554	124.0599	63.28241	112.8115	370.2823
LOC387856	34.02731	35.99944	47.0675	76.48343	43.83246	48.81794	43.17897	44.68491	54.99368
CHMP2A	106.7695	51.6895	51.15119	58.24138	52.38624	60.92425	46.24601	54.60966	75.13807
RPS24	4258.892	4345.608	5114.989	5491.007	4061.685	4501.146	4504.917	5837.816	4608.093
WDR4	5744.191	6648.09	9042.129	7213.077	8796.956	8328.29	10025.52	9560.898	3150.053
BHMT	54.43191	43.60848	40.96117	43.5594	41.48068	33.55128	110.4294	353.2115	32.726
COX19	1548.991	1022.008	359.6679	753.1699	165.2344	474.9334	227.6426	389.1142	1592.647
HLA-G	334.012	200.3446	103.0995	582.4949	150.8438	118.9019	130.7025	91.46423	175.3593
ZYG11B	8885.779	7241.927	5797.947	6783.696	3913.217	6114.382	4312.385	5083.177	7969.13
AZIN1	43.43796	340.5555	409.0313	448.5433	1159.774	409.7657	1269.72	466.7433	42.4994
CCR3	40.14269	37.05615	34.13837	31.97267	39.24735	33.21476	32.27927	59.23637	39.2131
FBXO4	38.91782	42.82728	36.93777	38.28097	147.2435	41.07748	181.1444	49.83886	67.88972
LCE2D	749.9271	2534.753	86.58389	424.6042	4251.554	553.627	801.2446	2042.239	344.2715
OR14A16	46.1256	57.0627	52.17791	41.08572	39.88769	69.43815	46.62783	41.91687	102.9421
MIA2	45.68068	68.88596	48.20845	51.98042	39.68421	39.24054	47.19078	41.69299	188.5757
TGIF1	55.20199	42.58032	46.7231	340.6334	47.91807	57.25174	57.63958	44.70757	36.14622
FLYWCH1	1641.891	781.009	1263.786	1627.269	1075.522	1546.072	1807.688	1401.911	2198.348
TIMM17A	271.8554	142.8529	134.3431	191.3813	96.06774	117.8083	89.84606	106.3049	436.1741
LOXL2	37.81477	72.74787	36.40384	44.775	39.57679	34.80658	36.18155	41.16382	33.59362
RPL27	11818.44	10067.27	7958.713	12162.47	10931.45	6107.248	10709.1	8205.249	12551.92
ITGB1BP3	50.25636	66.85286	47.01046	808.4055	45.81031	40.42278	37.17353	631.1866	84.95673
GPR141	46.44923	43.62239	185.7763	35.81709	38.29581	367.1007	57.87501	209.1375	41.41837
DNM2	220.8672	141.7614	198.1333	133.7883	62.72411	102.7945	315.168	65.34209	267.0023

SH3RF1	55.38902	52.4035	285.0672	148.4969	219.0786	65.03947	239.8929	200.3709	61.81887
RBP1	6781.596	4548.058	843.2552	9436.054	2519.003	6556.571	3582.548	5357.051	8738.622
VPS13D	48.43252	58.23254	59.93319	60.58628	47.15778	32.72872	42.10827	45.35892	79.78755
GBA2	148.4173	80.55338	61.90886	61.10424	54.72319	82.19003	61.01374	57.0971	286.6045
SLC26A8	64.72102	54.55022	44.38821	60.82115	37.24485	39.97977	42.12849	67.94567	56.9375
LIN37	79.38534	53.52952	627.2464	72.40568	826.8134	645.928	992.2342	853.0468	56.74265
HECTD3	13943.37	12571.46	15168.5	13175.82	13710.94	17527.22	14951.67	16695.87	13631.19
SLC1A4	72.42713	94.73004	700.3074	573.0513	1980.547	1488.225	1595.579	992.2753	47.15456
MDH1	176.0969	117.7146	951.1135	233.8859	971.1127	836.3256	1202.685	866.3491	69.00335
PSD3	1129.339	370.8895	135.6871	168.0645	189.5603	248.096	136.3412	74.5983	149.8516
CRIP1	2806.01	2707.718	4702.871	4072.404	7235.9	4107.833	4833.35	5539.284	3624.521
SEMA5A	115.2261	36.62912	56.77521	73.5048	46.46203	35.7299	46.66689	39.99349	39.76126
REPS2	49.47374	48.34234	1834.471	610.0501	709.1469	1173.807	1652.027	1319.317	34.33029
LOC731486	8136.421	7436.801	9603.094	10019.75	9343.342	7551.849	9845.497	13152.69	11237.65
ABLIM3	312.97	288.5504	335.8132	439.3763	408.8956	290.4346	450.3028	386.9976	498.6904
PRICKLE3	94.74155	330.6777	203.1718	84.59403	99.89078	262.1648	181.0345	126.7144	40.34158
APCDD1	83.28566	1984.603	1114.931	57.77047	504.8504	889.1434	597.194	241.8505	183.731
PCDHGC3	90.1071	562.0144	191.8092	77.17915	264.8227	50.12393	100.5658	255.8386	85.88241
HIBCH	35.56215	351.3326	1672.424	62.54519	1181.617	702.4352	1061.834	1198.097	35.04228
ABO	2781.581	516.8455	1846.568	879.6451	1833.687	2579.264	530.4657	539.4067	2579.324
NLRP10	275.1046	430.3883	149.9777	737.3794	130.3153	223.7147	89.25731	260.4647	753.3191
OR51B5	137.3931	103.8876	50.65225	110.3061	55.1658	72.43485	48.33919	60.24924	137.6242
NFASC	224.1245	145.3223	194.1064	166.1831	128.9238	130.8599	74.44384	125.201	496.5399
FILIP1	442.6505	745.8639	688.6805	253.4495	490.1287	876.652	427.6853	696.2061	1344.847
STARD6	73.79927	70.69868	48.2069	64.1229	39.98824	58.73819	45.92258	41.54845	58.77654
ARSK	400.3323	239.0112	120.4968	272.6501	94.70828	169.1479	150.2437	136.5451	512.9035
PPIL6	52.3349	51.80664	70.38577	43.9684	52.89389	48.13221	124.6316	321.4253	51.71493
C6orf70	112.6602	43.26915	59.71627	42.05516	53.81612	40.25724	190.148	201.9267	42.33751
SOCS6	497.4258	265.5536	237.1155	318.4407	174.7621	239.8416	176.2576	197.8101	636.0867
ZNF533	57.71968	91.08997	34.15495	120.0292	44.34537	41.73143	55.48699	35.41552	40.48587
STARD3	417.1703	178.2921	167.4595	207.4779	142.4559	178.0084	133.4638	203.9067	325.1837
PTPRU	68.51804	164.4185	102.3532	119.4604	70.3351	64.92979	74.54357	94.17054	148.8563
ADAT1	92.91045	537.0409	237.0549	621.2177	227.8806	193.6671	262.5652	176.116	49.13135
FMR1NB	38.39925	45.85017	44.41838	57.27724	40.70979	42.3458	38.32838	41.33247	38.68639
C1orf69	42.73372	69.17193	37.4628	55.37012	176.1985	60.52673	57.24856	243.4433	55.65559
MEOX1	162.2653	604.2783	295.6414	73.38496	734.483	481.8401	1230.845	710.7078	58.72514
C19orf23	551.4509	1093.844	1361.268	1772.093	1399.351	1465.933	1267.704	1511.658	180.061
C8orf46	35.01491	47.19967	39.10629	73.63578	49.70892	48.8158	47.03871	36.0862	40.55508
ACTR3	3860.279	4454.156	5862.332	4440.604	6948.036	6090.822	5185.044	5268.751	3165.232
RNF168	2102.82	2458.615	1232.4	1205.76	313.8368	943.4636	836.4323	1717.862	419.3618
KRTAP10-5	71.9005	58.73782	54.69363	45.36979	60.30376	65.94171	56.91066	85.19799	335.7792
TSNAXIP1	144.2687	60.07659	54.07246	43.49202	51.81733	50.33162	73.93719	52.88424	72.81739
SAE1	13253.47	12332.61	19029.93	17511.12	22408.6	18052.75	25205.35	20223.07	9602.432
CCL20	1130.375	128.1304	630.2021	184.9094	412.3263	327.5173	733.6508	668.925	173.4527
NUBP2	45.95064	48.12987	39.31431	35.33049	213.1275	38.39271	35.5945	35.46447	56.4357
EREG	483.2115	236.6085	83.72299	301.4473	170.2921	104.4371	96.85505	150.5073	720.1183
SGCD	49.23314	39.15778	39.59126	791.7012	47.83693	298.7188	45.14441	241.5573	51.69965

ALKBH5	6433.928	8512.665	12670.61	9862.486	16639.54	12746.12	17383.32	17082.41	5560.851
GUSBL2	200.9642	212.2845	98.18545	223.7118	74.12381	145.251	370.9205	101.6398	202.6866
CPA6	134.2354	41.05631	61.50852	44.9571	39.59233	290.8927	87.73171	127.6132	107.1724
SULT1A1	84.87827	56.75168	44.96376	53.0906	53.87252	39.85651	35.84229	37.94773	37.94434
CBWD2	38.54902	48.80762	32.2397	36.73889	42.76986	36.54602	31.36831	40.52153	36.53309
IGSF1	79.05808	61.28096	42.162	41.43427	43.78508	42.00548	55.65239	49.53469	59.12938
POLR2J	59.6627	50.94151	197.8541	39.41947	60.20689	48.83542	99.41524	367.8304	54.06402
CCDC85B	148.487	675.9653	126.4701	876.2554	98.45203	287.0106	129.8159	241.6535	2000.763
ZNF607	113.7703	72.64963	66.08037	113.7277	57.42246	69.3161	60.33124	59.97747	236.8143
PLAG1	137.6649	68.99771	53.04575	426.1086	48.22731	50.69214	180.7407	47.55653	1137.071
PODXL2	70.33529	73.18749	59.08169	68.99265	299.1899	411.0533	306.6823	69.02025	58.38986
CD200R1L	46.07075	40.84455	50.26696	48.65886	51.74129	64.30309	43.34588	43.78187	51.52761
CBX7	260.9975	281.6696	113.2865	149.6409	339.7906	125.5937	77.39291	87.45735	339.8037
LEPREL1	5445.922	4659.137	3895.452	9006.304	2931.994	6959.087	3904.728	5617.954	5354.863
RASSF6	885.0673	48.05133	38.20684	41.49504	140.0878	47.25111	170.2366	39.6369	44.58572
HMGCR	1677.144	1433.292	1479.863	2120.101	1921.664	1723.805	1037.675	1076.116	1004.968
LOC284757	628.7983	32.95548	102.6624	68.18096	61.15709	222.5245	84.8593	56.03174	710.1944
RFX6	82.69124	38.98421	38.8132	37.59763	53.41145	53.96835	41.39525	56.1628	58.96057
SPPL3	134.5553	109.7673	70.79635	64.01523	143.8592	77.68737	53.40521	77.85703	132.1965
NUP50	38.91247	41.39073	40.06429	1559.903	174.6273	43.24721	441.2565	746.336	48.83774
SPG21	38.53001	50.49678	55.73014	51.45933	47.26137	35.79108	37.06161	42.88192	132.2073
SMAGP	61.81073	42.55266	38.45957	71.77153	50.68259	36.46634	39.28765	44.77394	71.7106
MT1M	60.5034	50.54477	45.75403	425.7941	68.60544	41.67436	151.6583	38.36544	37.10735
FGFR1OP2	770.7724	547.2115	492.9631	745.6848	459.2651	623.1699	483.9936	660.8775	948.6317
KCNC2	36.37629	37.91534	38.26412	37.078	35.61332	44.22866	41.75848	34.1973	46.70071
ADARB1	57.64414	36.60313	33.43495	36.68455	221.0208	33.42855	128.452	36.92624	49.18489
C1orf57	55.03276	283.9096	1447.459	228.5878	1438.7	1146.582	2302.028	1436.675	2021.125
SRPK2	238.8246	631.1782	272.9471	598.2629	110.6369	496.0965	136.5353	155.5627	128.1894
CEACAM4	316.7001	169.86	84.8046	168.1649	77.22267	109.3576	86.13156	75.21106	336.674
YTHDF3	362.7487	305.623	152.2121	219.9323	140.6579	259.5063	124.5978	173.558	432.6979
GALT	865.9407	1064.606	1334.077	818.0188	841.4464	1484.442	1006.265	968.5839	540.2979
MAGEL2	404.3637	217.0149	115.0359	255.2844	129.095	173.319	106.3735	161.7511	607.0899
SEC23B	49.05031	881.4637	1420.194	53.38262	3192.209	372.8658	1781.789	1421.671	47.18412
OR2AG2	74.34821	721.3474	75.06649	60.10329	47.9767	70.26839	48.34441	62.77335	53.75498
MLEC	257.1472	972.7487	1188.973	1518.073	1257.008	1299.069	1198.458	1493.549	2135.114
KIAA0319	50.19617	392.2403	209.4743	41.22353	50.49651	273.611	102.0636	99.82034	35.11397
HR	63.47787	65.11551	57.20719	55.08928	845.075	63.21168	48.25473	67.89979	96.68866
TSEN54	64.0303	60.53907	794.6414	35.66357	1435.565	379.4056	828.6284	946.2786	37.59723
RPS6KA5	70.19802	69.35982	55.78889	70.5461	292.1618	61.83921	217.6044	185.5541	50.85116
CLEC12B	46.43073	77.71818	61.39579	69.85531	53.0113	42.151	44.96384	47.15429	74.98647
CCDC34	3434.649	2578.591	4322.988	4349.101	3630.155	3002.523	2376.707	4053.289	3424.033
MVD	40.51254	40.90263	509.8181	190.4942	668.5419	699.7292	373.3932	790.3766	368.2702
TSPYL2	36.35184	71.0789	38.33067	46.84927	41.986	33.19418	38.37905	53.0573	36.07686
ASNA1	387.8376	173.063	111.8943	164.0795	114.8721	159.9248	103.2055	149.2325	448.3475
SCN3A	64.22599	49.45083	43.15612	40.16448	84.54824	46.51279	74.80467	45.65171	49.33828
NCAPH	85.46661	89.24765	63.60963	84.78841	63.38089	77.76122	66.03303	63.41102	103.3888
ZFHX4	32.66175	41.14327	35.0689	37.08202	103.4632	33.54119	78.43593	115.3945	35.39494

NRG1	79.51493	55.59771	86.36592	190.8379	61.01725	75.36553	53.19397	65.9783	54.98808
WTAP	224.733	59.02852	37.34996	50.89253	65.0102	85.6194	51.42513	95.30902	97.0678
NSFL1C	1286.994	2292.333	907.186	1812.533	828.0552	390.764	833.2929	1422.131	3113.965
CBFA2T3	3393.758	3771.142	2833.625	1585.297	999.8654	2070.849	1365.253	1375.096	3740.227
STAP2	79.55856	84.18028	185.6765	99.98684	538.2792	77.57685	373.7294	564.8272	1715.255
ZMYM6	102.7951	69.61404	305.0841	448.4868	388.0363	613.4382	595.7014	563.7133	161.9631
FANCD2	1096.151	3214.137	2257.323	3873.542	1033.834	2857.87	1531.487	1417.65	3532.991
EDEM3	339.9755	137.1399	102.9168	118.1607	56.53221	110.2434	59.01734	38.5084	53.58466
KCNIP2	34.73687	43.88054	35.64677	32.97779	35.57003	56.11454	45.98579	37.36725	34.22286
EXOD1	2126.504	1621.906	1241.375	1195.312	930.6979	2109.908	1059.076	1590.326	967.8423
ZNF644	1359.118	1531.547	428.2659	46.26704	163.395	629.0394	97.92666	37.70718	1638.26
VAV3	410.8542	114.2044	278.2186	165.3169	239.942	227.3264	266.4215	151.6177	613.5687
CCDC69	452.479	317.47	308.2111	327.0963	309.3869	279.609	269.375	590.2135	663.8948
GFM1	4847.749	6868.73	6345.631	4949.333	6814.031	7666.547	6887.163	8289.873	3602.619
C1orf198	210.2238	195.3757	386.7375	408.0089	576.3432	533.3077	314.7313	671.2187	143.7835
SAR1A	534.241	238.972	896.3854	571.3769	1297.54	1055.784	1321.474	934.9471	74.7207
TMEM137	20688.13	19525.97	15744.74	17545.28	11572.89	14077.81	10973.5	13700.88	20165.01
MGLL	160.0162	103.1667	98.63976	499.4947	634.0674	120.8826	350.9035	93.5435	269.9525
TRIM34	113.8366	38.46409	37.87638	36.17298	38.21664	33.50171	37.40766	46.3817	32.88332
ZNF254	47.97258	54.88927	41.01427	46.33818	55.06163	44.02106	52.04228	33.45668	50.97894
C15orf17	8575.583	8060.696	7703.127	7244.711	7131.379	7871.773	6726.145	7869.089	9760.17
BPHL	3820.198	2763.538	2099.618	4423.003	1314.639	2967.898	1762.288	1769.729	4099.763
CYP27C1	1944.383	1567.995	475.4902	2452.151	1222.148	1644.79	1240.639	1341.055	1560.97
CNNM1	66.46651	51.23848	77.45724	101.0986	487.3829	93.36562	361.2596	77.10517	133.6604
RPAP2	337.1129	127.7681	479.0313	198.3401	543.4297	498.7348	344.3193	404.1829	369.0341
SYT17	40.35452	46.22032	45.07382	41.547	461.3341	466.8689	103.2349	182.2831	64.56327
TRIM29	128.8996	488.7944	574.8572	415.3091	457.3517	485.5366	229.6039	333.2195	916.0065
EIF5	52.13589	56.46912	35.54476	54.49635	42.87659	36.10624	38.81753	55.56417	57.90086
CRTC2	660.6563	113.3057	121.5387	90.31048	73.18833	94.7622	74.5022	63.77789	223.927
SFRS11	426.1127	257.0353	160.1275	271.7385	193.1109	264.3238	257.4134	149.5776	461.9785
STMN1	56.7291	105.7961	94.54949	89.30118	346.2894	101.7292	143.3474	76.66345	198.8136
FIBP	15664.21	14752.4	11019.71	20701.73	8746.505	12318.43	15932.78	8467.492	17422.59
CFH	4699.014	5801.649	2463.333	2927.339	3327.038	3688.4	2272.694	3727.152	3879.805
PPM1M	29524.45	28831.23	26978.88	25178.05	23069.88	23643.86	23587.45	21050.72	27423.24
IQCB1	44.35253	54.74792	107.2755	72.63656	85.73554	132.4858	54.07155	74.22348	42.26546
PMEPA1	81.65643	76.0395	48.93915	68.40224	58.42738	80.01257	57.11237	77.4464	168.7522
PLEKHG2	120.1753	66.05994	47.24387	47.00254	38.21174	39.35216	237.6538	57.81382	35.08127
ZC3H8	1560.125	767.2477	785.1417	947.6876	551.9551	866.7566	513.0974	658.2883	1203.523
JMJD5	68.30262	70.48064	41.47091	42.92284	46.75011	48.43992	48.82352	110.1954	55.4437
SUNC1	80.00804	34.13809	181.9949	93.35926	199.9741	46.34515	280.037	177.0837	58.2555
MITF	50.95513	32.24549	61.55114	36.93145	33.00224	35.64471	37.44793	32.53484	32.34247
ACTL6B	54.58577	99.49642	62.59172	91.27597	62.66005	87.33929	50.17051	52.58344	71.62637
TMEM14C	46.33024	83.07552	144.7871	78.14703	130.2202	63.76869	146.073	134.8089	87.64207
C1orf151	42.09008	55.23376	46.11892	32.11193	43.64456	35.15897	36.13112	36.24394	141.872
AHCY	40.69669	603.3954	560.3638	557.5041	377.4805	872.5587	483.8591	545.1758	45.74417
TMUB2	144.862	151.8534	77.9522	138.5458	242.5517	93.52263	193.5308	210.4192	105.8103
SIRT4	54.78302	65.58648	42.51104	46.74884	57.39468	47.77216	55.21501	218.5193	201.9748

ABL2	57.19894	398.6038	156.0186	421.5374	41.89751	262.3361	166.3559	71.30347	48.32405
RSAD2	86.01826	2698.129	741.1428	532.2708	58.16039	698.1392	1514.971	528.3642	40.55906
XPO4	44.56485	38.86074	241.528	53.5929	474.798	256.8533	508.2188	342.2493	44.62994
NME2	73.2943	78.9212	38.61415	40.39542	49.93099	42.07285	34.53192	36.29063	42.05798
SALL1	37.29851	30.28938	31.79564	41.2711	36.87385	36.73667	80.88313	33.42771	34.14264
TNKS	81.7793	37.30402	599.9545	872.1401	148.1401	48.94912	236.7735	357.1727	47.78823
TLR8	154.3871	157.7882	101.1178	126.8498	71.91019	93.26984	86.92322	92.47351	115.0794
TXNRD2	6551.118	7875.711	5462.645	7668.694	4731.326	5789.149	6728.383	7185.987	7386.588
MTP18	45.04264	57.51809	41.67322	42.34513	36.37268	197.7108	36.35487	38.76122	36.02793
C1orf84	2239.277	43.22342	33.4501	1415.092	160.6982	60.04436	96.2822	32.33816	47.46324
RHEB	343.7423	202.1797	148.6449	237.5383	179.0698	186.8282	165.8306	133.6775	298.0443
FMNL3	2008.664	1929.973	2477.371	1044.738	1983.026	2969.841	1764.774	2253.344	36.56809
LOC72894(	91.03255	47.99889	40.97689	41.79993	49.10378	41.2962	49.02385	62.47483	84.20084
HHIPL1	65.29717	117.6842	89.80176	91.84428	70.13626	99.33378	62.07056	51.4272	334.5294
MSRB2	14406.7	13676.21	11401.37	11058.02	8276.692	16156.24	12425.86	14031.66	13192.47
TERF2IP	330.3851	176.6291	704.1411	496.126	1554.714	626.32	994.5159	672.9333	504.3635
ADNP	266.9254	40.74746	118.8895	39.02797	126.1631	60.47873	137.9383	39.82605	51.57491
CENPI	74.93149	53.70314	181.7906	53.69961	45.92514	48.49743	137.1875	37.78045	47.29309
MBNL2	564.2059	452.8524	1074.252	670.5137	1937.844	1325.169	1067.615	799.341	451.1107
ZNF831	1689.529	39.53938	43.10375	44.25056	272.911	39.2443	389.8619	738.6016	31.28182
SPN	76.93882	81.8854	70.83537	91.14426	50.56643	67.70508	50.89912	56.52813	149.6787
RAD54L	119.6145	346.972	735.4804	985.8644	737.9205	640.4785	929.2626	996.9597	229.719
CRABP1	45.61908	874.7795	757.5542	84.86872	602.5062	46.02747	192.563	41.42652	166.3461
ATP1B1	1729.51	717.1862	1764.709	1169.191	6076.337	4026.536	3876.427	2354.819	3956.589
ZCCHC13	36.11774	52.99406	35.24021	50.38826	46.01773	56.36271	38.4951	48.03848	62.01956
ZNF573	674.3377	478.4492	742.2001	333.7008	418.8637	745.1394	633.0364	334.333	802.473
FRMPD2	34.12775	34.13311	37.27487	71.94092	30.17672	33.7647	36.47328	36.39879	43.77934
ABT1	55.81858	171.8427	139.8258	207.9942	98.14561	152.3176	117.985	155.4862	117.2039
ZNF365	63.52755	41.78535	161.2395	56.49757	119.4607	38.76155	154.637	34.31425	34.94933
KBTBD6	475.4547	319.6707	118.5321	304.0454	110.264	168.9952	106.5954	140.932	706.6577
C18orf19	65.48537	1015.074	181.3495	3000.099	138.4489	284.2292	581.136	1010.949	65.20953
CFP	63.66023	50.34911	401.4729	57.79963	126.6942	52.90004	230.3718	186.8811	158.8341
DAZ2	43.75333	50.06175	49.37436	66.95515	54.1642	55.76975	49.14279	49.10625	79.57372
RFC3	84.71642	33.94168	175.2033	362.8177	34.23011	355.9544	208.762	174.6778	50.57687
TRIP11	340.3786	346.5847	699.6898	384.7996	1019.619	689.355	783.064	421.0574	417.4113
GRLF1	30.82508	88.4445	186.0099	32.94167	48.63795	34.94872	33.47803	31.43866	55.28394
MAP3K10	659.1808	438.9465	228.708	362.4193	156.6491	277.669	249.7952	300.8093	852.5893
ZNF501	75.72523	118.7997	48.77014	64.6479	54.8207	80.24706	51.12227	56.65403	123.6295
FAM156A	59.24425	411.0042	462.9917	463.7766	764.7897	54.32961	341.1983	303.0495	154.2773
NR4A3	65.1394	686.1069	50.96805	49.07552	53.02884	46.37373	99.64516	49.45183	47.89953
ZNF418	3747.498	3614.985	1889.262	2415.563	1411.5	1407.135	1025.97	1340.577	3969.552
GGA1	41.44117	39.14581	257.7147	54.93714	46.79623	552.6849	102.1887	55.66647	32.99291
ABCG1	33.3665	34.4247	50.28857	40.16721	39.24725	37.72992	40.17722	35.50024	34.79672
MEX3C	61.28616	48.98418	77.77142	61.21569	108.0082	82.2112	63.78165	69.94498	733.7759
KIAA1026	86.25096	633.2152	198.495	62.66371	44.35464	65.39281	56.14907	54.77304	202.3265
C1orf102	48.33045	38.70629	54.82316	33.64464	168.7681	48.5587	37.49947	41.27765	42.86381
MSLNL	361.9924	218.4593	119.6682	246.2012	106.8702	173.8569	92.57512	127.0832	495.8503

C7orf60	32.46648	35.89715	41.92014	34.86139	42.79968	38.11627	32.43382	46.10439	37.09869
DRD2	89.60268	45.62856	58.59048	50.92394	43.65869	36.11102	49.47931	35.01095	31.88183
POLH	473.8895	324.0273	446.768	375.1494	668.4308	589.4974	290.0045	588.7504	684.731
NACC1	249.8575	149.9424	84.63205	179.7456	125.6291	105.2923	102.5401	106.0891	208.1973
FBXL10	290.9815	253.2124	127.0821	170.4154	108.7545	132.5178	94.69859	77.70898	308.0887
KHDRBS2	82.5127	64.79427	53.24554	75.34702	58.20285	68.34785	45.13437	60.71697	44.14449
OAS1	136.2213	1670.845	4173.983	1247.917	1311.433	3774.964	5898.357	3805.081	207.0856
ABCB5	54.33392	40.8244	40.96454	66.50352	41.01765	68.6289	36.42184	34.29588	35.20549
DRG1	74.14082	54.64858	466.1789	60.55455	563.7442	48.30146	278.5872	764.23	162.7915
MAPKBP1	37.31416	54.79774	48.18983	39.59894	34.87446	36.93261	88.85342	190.929	37.93964
LSS	36.58658	824.1921	51.12365	76.6274	41.3227	49.76513	36.83739	233.6275	57.01888
L3MBTL	30.16253	39.39724	160.6388	54.37032	37.84683	34.5131	38.54718	46.41557	150.5483
WDR54	5620.795	6214.653	7413.592	9230.081	6558.891	8706.205	11042.86	10829.75	6178.014
C16orf68	99.17162	491.616	386.9032	39.0578	441.1065	292.8493	313.5153	410.6061	40.62527
FAM83E	39.27167	50.11657	35.40007	36.91595	31.02982	45.66228	42.51018	40.4707	36.63062
PCYT1A	284.469	202.6049	105.985	182.0647	77.01225	137.1899	81.5308	95.49444	312.5284
BAZ1A	61.3355	65.046	51.70193	47.64441	38.81272	33.03158	176.9023	52.501	43.93037
ECM1	2052.601	405.1209	316.8814	1526.039	329.9272	506.085	197.3789	63.4999	1116.374
AKIRIN1	528.9502	2209.109	2758.731	3234.658	1506.823	1573.882	2055.987	2462.524	239.6273
FIS1	42.62878	40.30103	249.2712	47.8091	329.7651	42.2219	213.7149	361.1128	40.74594
PAMR1	5227.11	4425.316	5113.497	3427.95	11533.33	5858.404	7326.583	5301.589	4328.661
TACR3	409.2948	158.9951	126.3319	212.0318	164.0989	213.8392	119.4431	191.8616	367.1012
PAN2	45.92556	437.7303	1669.661	883.8251	2419.374	2023.865	2961.57	1184.503	37.74221
CCDC114	122.4623	63.42889	46.58773	65.27932	50.57937	49.28579	49.51273	39.80405	93.97398
CRTAC1	224.1919	332.9528	125.318	98.13383	217.6582	47.7797	145.1328	65.11638	223.8915
DAOA	90.97206	45.42582	41.04365	58.92455	44.26712	42.40865	46.23332	54.69424	51.6832
SPEF2	74.39872	88.95511	105.9212	50.47241	46.62022	57.48045	49.91575	41.03652	59.77273
KLRG1	2797.498	3421.644	1095.301	821.9088	461.6818	951.5802	714.17	1230.485	64.78802
SLC43A3	108.4048	46.99722	51.54148	49.78297	222.7209	74.45247	438.7809	429.4909	95.75886
CHD4	4742.476	4814.658	4547.511	4540.886	3202.833	3449.554	3856.768	5013.762	2074.859
HYDIN	45.26577	74.91042	63.65507	46.55499	47.06877	36.82447	53.84889	61.19661	49.60483
MRPS18C	212.7756	351.6471	165.7533	280.5256	267.7034	95.97446	324.3855	468.8853	247.0713
DSCAM	352.3097	202.0567	100.9244	218.6452	95.38171	163.8828	86.81255	85.72823	516.2315
DAG1	40.58514	53.19063	476.6287	70.47188	1093.743	53.51614	585.4255	332.267	97.37293
WNT10A	107.6543	1009.007	701.3658	134.1086	733.8692	1619.998	699.5894	1239.039	1899.557
SFXN4	2307.549	1831.265	878.8181	1260.807	662.5181	1437.255	655.8967	1210.884	2252.492
FAM53A	2706.865	2290.025	941.6477	1624.482	789.5787	2039.579	948.5413	1244.919	1894.904
GTF3A	4952.238	5936.639	10211.01	6665.074	9829.668	8365.889	9876.012	10847.06	3904.462
TP53AP1	36.52413	50.53511	46.48756	34.77355	42.78168	40.45762	42.63298	38.1118	68.25463
APH1A	100.1654	1195.773	1084.248	179.2998	58.31046	634.5222	242.6016	61.21002	77.69534
IL1A	4338.115	1635.959	2403.262	5790.185	1309.761	2328.403	2364.531	1195.19	3286.277
MRPL54	283.1888	168.1623	173.0454	112.2605	78.3959	128.8931	269.4912	423.4725	383.6803
C13orf15	2752.355	1844.468	985.2333	779.3475	2050.541	2134.271	1866.809	2535.563	150.3847
SLC44A3	74.77966	75.64355	50.50979	81.6767	238.6697	48.04751	87.50253	46.10516	147.4882
SYT13	58.56826	51.12718	46.35114	50.61424	51.13808	297.7216	104.8523	55.89143	50.69301
SCAND2	63.20126	55.2643	120.098	71.19331	113.2302	171.653	63.0798	51.23702	52.91399
CHKA	106.5586	1611.341	2326.711	581.5579	2321.506	1880.723	2607.066	1932.46	58.56851

IQSEC2	333.0763	176.7534	230.2169	190.6651	70.4735	955.4636	282.5499	96.13257	268.7778
SLC13A3	119.2984	115.4634	70.14505	82.08855	63.92157	87.28297	59.01748	49.45655	176.2891
RYK	253.0207	527.9142	509.5295	763.6641	270.6324	271.4145	319.9929	276.2276	228.0314
GALNT5	52.34244	41.62203	49.46347	60.34337	51.48262	39.75348	44.9952	53.49881	45.33919
PTPRN	47.89961	40.11005	42.09649	44.82911	42.26389	54.08072	59.03777	128.1124	35.65503
CDK5RAP3	55.77676	1016.599	683.8256	107.2268	391.7826	547.7532	212.9983	853.3828	212.3214
LOC73001‹	47.03976	41.21808	36.70629	691.3609	52.43605	34.17514	38.31707	35.17139	35.28912
WWC1	45.34283	37.42126	42.88582	65.28699	175.0765	38.50016	46.66254	44.64445	81.54324
MAGEC3	44.20289	42.93487	54.44425	41.31199	37.12997	41.83554	46.193	38.19263	60.82203
UBXN6	40.6695	520.5817	263.8977	38.55582	264.0045	186.0542	434.1552	439.4453	987.076
RREB1	3681.064	4433.987	3168.034	3672.911	1694.19	2653.975	1606.233	1564.646	4496.645
CLGN	34.23744	37.10475	59.25122	35.23122	31.51143	34.8702	35.32184	37.0096	46.69523
MRE11A	156.1526	76.28136	68.76881	117.3588	95.86602	106.6892	68.66364	87.35049	152.8767
SLC46A3	1030.319	61.15291	46.03679	63.7731	148.3019	89.88435	174.9442	38.22749	53.6197
C3orf57	52.18544	51.21896	45.90306	55.03631	123.5935	48.9789	50.03955	38.78445	39.94535
ACTC1	738.9576	484.7146	382.5266	128.4671	430.8968	758.3155	1318.613	1641.343	1096.067
CDH23	66.08277	88.23403	151.3306	62.00883	129.1826	233.7359	69.41497	341.0454	109.9311
DTX3	49.52887	47.33374	41.3334	56.88902	133.1385	336.6819	292.2845	46.60506	38.48573
SAAL1	113.035	738.2892	1421.649	130.6199	1489.573	1403.479	1015.342	1741.395	107.798
YJEFN3	247.4251	63.05805	72.10826	102.2147	68.53696	107.8677	70.90195	54.37255	71.88826
ATP9B	2015.476	4103.706	3304.082	2351.047	3618.593	3690.003	3855.937	2889.109	4657.003
ELFN2	69.93867	54.77728	54.10243	89.21981	47.86811	57.12739	49.5692	42.73579	97.54313
NUF2	438.8715	230.6753	523.1114	1244.861	145.5994	819.0698	406.4299	118.9336	559.1343
HIST1H2BJ	3201.359	2278.884	1494.795	3826.688	1212.008	1683.156	1548.887	1150.997	1924.891
ST6GALNA(	511.9387	379.5668	591.4177	523.0499	560.7466	749.3923	644.5175	903.1772	163.5676
LAMB1	2545.03	1390.486	918.3976	3988.152	522.9255	1368.156	929.8558	613.0037	1254.28
FAM19A4	194.4525	114.7929	82.14011	227.9317	56.38945	127.7015	57.26062	59.80796	134.2562
TNFSF11	44.22145	34.34953	33.86586	36.29165	36.1232	45.27028	36.7221	35.72161	53.62498
NRXN3	275.4502	160.527	172.6511	204.6723	96.76077	148.9807	88.69827	117.41	461.1104
DEPDC5	260.719	112.0459	180.3926	251.4155	153.6157	181.5781	160.2026	151.9869	568.1096
ZBTB25	166.4975	248.0265	159.1137	253.1958	173.3985	206.8687	158.1519	252.089	384.4665
CCNDBP1	869.3386	34.46697	341.7581	32.76312	192.4691	33.63956	44.98419	40.19498	47.66159
PTPLAD1	432.6078	763.4952	284.1823	522.0341	264.3481	343.9892	139.5191	340.4488	83.90103
NPB	102.3446	38.36766	47.81075	54.27337	41.1697	40.99054	41.64618	51.26744	42.40802
PEX19	453.2994	244.5102	265.2187	282.2148	751.1283	554.3712	454.2989	545.6536	587.561
PIGF	92.9136	140.2956	61.86893	381.7314	116.2423	234.7579	105.5444	150.9204	892.5706
MEGF9	41.84108	61.29498	886.503	62.27236	359.7504	929.6333	381.3123	399.3924	39.38448
BRPF3	492.3878	577.6398	751.1989	294.5051	882.5472	1165.997	884.9623	727.1588	638.2977
KRTCAP2	15015.51	16986.6	15587.79	15947.07	14408.97	16308.8	15655.55	17581.15	17680.71
BRE	41.83794	59.92	52.15454	38.04986	44.219	37.60628	42.9317	50.99336	48.75694
SLC5A10	339.9809	215.5835	97.3754	225.9709	98.18483	133.3982	86.7002	132.8324	306.0068
HMOX2	58.55923	236.0888	131.1779	69.81915	166.8618	72.90694	136.6767	156.3371	45.28694
SEZ6	52.09159	42.5625	46.53153	49.921	35.24228	44.543	43.58454	43.53428	34.87538
CAMK2D	90.51953	44.96729	64.97534	43.91644	48.81291	39.4408	50.38792	77.50506	96.02311
SAMD1	794.2971	60.87401	64.33328	65.51882	62.76418	65.88728	95.99659	54.35617	42.92672
C10orf26	53.4996	47.05655	57.55627	70.69981	57.93996	47.95298	94.16705	64.35513	148.0713
ZC3H18	59.99109	33.95678	34.51092	41.72255	33.96357	40.73512	45.55119	37.03656	42.18455

CAV2	51.8306	264.2115	152.727	295.2213	124.7689	151.187	175.5944	105.9026	734.8226
ARHGAP25	71.14662	37.75073	885.3559	547.0907	38.20463	312.8618	180.5361	498.6127	68.34445
SP140L	67.49697	1109.06	495.5303	66.1664	47.81616	552.0334	84.80178	72.46564	72.5954
ARAF	209.0833	316.7036	372.2774	504.3635	129.8247	233.2902	201.8101	284.088	405.7975
ATP11B	1592.17	1808.224	500.1831	822.5284	503.8057	1218.606	562.9021	653.6887	4422.381
DAGLB	4340.302	3114.93	2482.709	3662.166	1477.207	2324.936	2026.56	2321.597	4699.514
OSGEPL1	428.3033	296.8223	119.0933	256.5371	99.41556	204.7222	101.5141	141.0571	608.6005
SLC1A1	33.01883	52.76759	112.2185	43.03538	207.6246	46.02105	85.74771	85.74022	41.5982
JAG2	54.55482	72.56781	49.91435	473.4344	46.74567	63.6573	97.18181	52.21238	60.29965
NOTCH3	17644.55	14501.09	11082.93	14063.27	13966.66	13504.19	12258.79	13679.41	17773.06
GNB2	4127.109	2960.129	1086.793	3808.04	1058.534	1447.846	725.828	1117.385	5756.805
DDX25	273.2744	192.0666	102.7861	160.8756	85.28245	119.4154	84.53871	109.5873	412.681
EIF4E	63.07679	59.23224	54.90466	74.62301	55.96291	44.95772	42.95924	41.90857	61.36423
LCP2	1601.62	1249.607	902.1107	832.2372	153.0226	738.9517	402.6547	251.1344	1246.085
GABBR1	143.1497	69.01691	60.83834	93.93046	57.02562	101.4791	61.99586	60.64609	85.60564
CREB5	103.2071	127.391	65.29427	121.6258	110.2069	86.9847	54.79208	68.81092	201.3342
MEX3B	45.62543	151.2413	104.1071	125.0691	161.2935	167.2929	305.1737	151.8585	95.01379
DPM1	719.8411	163.9594	391.415	864.68	315.4995	343.6297	221.7449	228.7046	397.5748
ACOT9	59.34198	37.04311	163.5901	880.8987	362.9417	417.8999	546.8681	438.4258	48.20461
XPNPEP3	107.9359	81.36	47.23294	88.82786	42.83967	66.32856	42.90305	48.6546	114.7284
NANOG	51.22559	70.88056	41.10414	86.42557	45.38546	40.96915	38.534	66.6187	54.23406
C11orf74	51.85709	370.626	155.927	47.51744	198.4976	655.8044	183.3965	151.5518	63.39787
TMCC2	43.15472	35.61102	152.4958	62.1739	41.73579	48.13527	42.0345	38.43196	48.01732
POLR2K	1568.263	1748.385	697.2556	1830.854	283.4552	557.2784	408.407	629.5116	1535.675
SLC2A1	7446.289	9019.857	7370.873	11550.12	10445.22	9145.32	8542.954	10152.16	7073.687
FAM116B	13396.06	16647.95	7937.087	13622.66	10195.18	11485.52	8580.325	9129.373	15269.77
PPP1R10	61.54171	35.28912	268.0396	51.09691	833.4652	47.95591	502.4803	746.6256	36.28682
OR51G1	46.69439	53.8966	36.98091	38.3825	39.96078	42.22109	42.70045	33.03497	57.95532
SPP2	43.75224	74.52519	53.59446	41.41411	35.65018	60.62157	41.44606	61.76755	50.40891
HLA-DOA	145.9321	115	1139.556	141.8603	809.9021	247.1638	619.6301	854.9916	134.0468
CD40LG	35.16083	69.72172	136.3026	65.16593	239.7059	355.7658	118.3334	50.21048	134.6618
C5orf25	35.9765	300.849	35.78131	42.21693	87.90083	45.70457	55.51705	52.24249	57.84225
ZCRB1	438.9795	394.5211	209.6756	280.8203	212.9787	236.4373	253.3575	149.1915	618.6376
WFDC12	49.07092	64.92316	66.08859	59.98828	130.9671	151.7833	72.483	191.1202	61.84451
NEFL	2214.155	1160.191	325.8534	1500.047	956.7374	891.9365	1388.726	1467.13	3597.361
ROCK2	64.83733	40.86503	112.4697	294.097	35.48985	37.5749	38.41399	93.57748	57.77174
PSMA8	37.51582	52.17671	46.78947	44.09407	37.02559	44.61893	40.30877	63.68539	36.22032
PHLDA3	53.26888	55.87207	222.3189	38.03619	61.5699	361.27	137.0662	119.1319	120.9528
KLF10	1852.064	2941.832	947.4108	3943.41	1300.737	1658.392	1751.136	2065.791	2797.597
CACNA1F	46.71626	40.25961	35.66861	50.83561	39.02357	51.01169	45.01638	43.47242	32.29431
MDK	263.6803	63.41864	41.36749	59.53844	38.33472	41.56224	43.31522	49.05396	52.72522
ARL8A	324.3136	177.1576	85.8047	200.9276	82.813	147.6346	79.94607	105.867	294.1807
ANKRD16	104.3207	1313.68	1590.174	65.77805	1355.683	1957.17	1674.15	1745.437	82.83933
SAMD3	43.30968	47.11663	49.92035	40.13636	40.94843	41.46783	40.93982	54.1813	56.55658
SLC6A15	67.30823	86.98629	40.95524	46.73827	38.08859	50.70274	41.29694	39.56828	50.29085
C9orf89	42.58854	827.9918	36.27944	97.78224	40.54698	47.27406	46.6888	36.28554	77.91862
GRIN1	35.22515	813.6456	505.2026	59.6141	46.2571	41.54957	39.0736	39.6772	48.58992

B3GALT1	63.04482	62.73108	68.28899	91.64672	54.05973	48.96088	128.9408	162.4074	55.42725
RARA	17130.1	19137.93	15954.85	18035.86	15409.16	16742.89	17644.93	15841.49	19317.27
CTXN3	40.21481	40.42369	39.86407	37.04605	36.37549	47.87735	53.75339	41.33794	114.6022
CYP4B1	54.47183	86.25027	50.98447	61.62414	152.3593	55.04853	47.33664	43.68561	128.8666
CD79A	118.6892	61.20928	39.86909	63.4173	46.22853	47.38373	46.16913	42.8922	42.64019
KRTHA5	39.80019	42.03233	47.74697	41.78862	33.48612	36.99474	34.48825	30.97717	60.70999
MTMR14	5757.201	5322.911	5877.439	4119.518	4054.677	5402.548	3691.584	5189.896	5001.88
RP2	39.78098	39.44271	151.9991	226.5082	154.4874	37.01068	104.0303	282.9546	36.79709
TNFRSF8	255.7363	108.207	150.6611	100.7371	62.16835	121.1642	68.19603	130.044	60.4541
PRPF6	1089.347	806.3743	751.6779	1578.884	444.9946	846.6025	502.5121	264.8516	224.0555
OTP	454.7358	268.5837	138.3921	301.7263	119.909	197.1131	168.9222	184.6184	557.9584
PSMG1	929.6872	437.845	37.98768	44.40968	156.2112	256.2422	247.4067	130.0448	44.63105
PLEKHB2	57.41771	44.77142	41.01962	49.61931	37.1728	34.14662	37.77593	36.21756	68.06853
FZD6	35.32147	358.7811	246.3864	358.0949	186.3372	291.7823	396.1861	426.2521	62.11121
SVIL	271.8951	638.669	233.4024	1126.212	69.36129	368.018	235.1099	985.8513	1743.408
PMP22	132.2331	80.2578	48.73198	88.31422	59.18513	78.53564	53.53809	51.58951	101.3493
ELP4	58.53613	74.72689	43.633	34.35868	406.7678	390.0078	335.6429	46.17643	37.12162
PPARA	33.87447	45.00438	38.44963	39.57079	42.06001	50.33981	41.03079	44.80184	75.75293
CASP6	41.69088	40.66945	65.71893	52.32411	44.0703	60.13803	49.87884	108.1749	109.3755
KCNK13	79.83234	97.01627	62.05939	78.68427	79.00361	88.47269	59.62588	50.56474	86.0864
GH2	41.97483	56.81113	43.89505	44.68206	35.35888	33.81895	40.37784	39.88542	74.11448
SCUBE3	173.632	108.302	61.30975	102.2692	235.9549	54.27184	63.67538	49.23621	111.4178
RFC5	969.1576	148.2684	1119.478	1442.89	1028.169	1011.603	1005.556	1557.641	231.9527
ADRA2A	1226.171	192.6722	209.2531	226.7822	399.8047	89.3892	73.11312	115.1444	224.1835
TFAP2D	43.23468	44.13111	39.98321	59.57444	42.24896	38.61189	34.82198	48.38124	74.32916
POLR2C	101.3052	81.48888	65.98112	89.03584	168.8721	59.47463	53.15624	49.46734	970.6592
MAP2K1IP:	4865.072	4074.476	2557.564	1818.318	2789.802	2830.559	1812.903	2418.843	4509.278
TXNDC17	1897.342	1335.383	3055.162	1309.266	2948.468	3797.159	3248.108	3502.079	2113.568
KIF1B	360.485	78.95787	686.7686	668.63	232.5643	372.0799	290.444	754.3918	1538.642
AAA1	289.7795	155.3821	112.0736	229.1854	74.403	102.7882	89.54998	110.5256	222.8754
DUSP23	10819.52	11561.38	16171.49	12610.35	16077.74	11971.95	14726.84	18204.74	12826.22
APAF1	3272.019	3290.776	2074.685	3244.479	1079.411	1833.062	1418.061	1151.532	4703.55
FAM71F1	167.2292	318.2643	465.5407	287.4697	102.3047	90.43283	195.6049	70.1716	133.7845
PBRM1	925.4293	52.54523	583.4047	658.8677	917.3734	57.34178	813.2817	300.995	68.96449
KRTAP4-5	38.4147	38.11773	43.32725	70.28432	39.83709	58.15528	39.79984	43.33709	50.9807
HBS1L	153.6001	97.46252	74.63112	200.2594	78.78053	105.0863	57.36869	78.6215	118.6722
WDR89	127.0147	58.50586	65.20222	60.82745	53.62342	61.61446	51.11914	57.93366	148.1489
EPS8L1	52.91665	130.8067	45.83549	49.29429	40.2291	57.89614	46.9617	54.60968	63.66585
USP39	40.73558	45.60586	1258.278	35.83055	2242.456	421.7267	1201.769	997.4803	45.76113
MED8	91.16819	69.91924	189.6443	38.40528	43.6504	53.22203	127.3929	281.1773	52.79474
CRELD1	384.0565	189.465	120.0417	193.2742	153.837	201.7914	141.1749	207.8062	524.1172
STX3	137.4727	140.7452	251.5885	117.0639	762.9497	833.9374	860.6835	230.4401	220.8529
SEC61A1	15812.57	17794.87	13615.69	17098.78	17614.19	18634.87	16563.55	11578.59	17294.52
MFAP2	33.13234	34.67428	39.78773	35.90435	50.97601	67.68331	45.62706	44.53634	41.60128
AR	56.31137	52.3712	60.52161	86.13831	42.88439	43.93105	41.60035	47.45344	64.15127
FPGS	34.86611	51.55982	34.42007	46.4053	44.75887	33.08364	43.65621	35.22723	34.77532
ADC	1179.354	472.9804	923.0479	60.23701	169.2575	767.051	537.8499	310.1661	90.03738

CCL26	286.5307	117.8064	95.46779	144.9636	194.0676	325.457	75.03117	90.36348	325.3695
TJP3	177.6048	86.5506	77.39833	147.5316	82.84486	102.0854	131.8452	340.3962	173.9989
MYCBP	84.39626	1087.499	242.7904	862.2534	538.9507	42.47164	158.4425	821.3976	74.20046
PRG3	33.47469	37.57479	50.82564	35.3283	37.63807	37.41024	43.57592	47.28054	34.24794
SGCG	76.80585	116.1372	218.262	54.23701	820.099	80.42965	1217.362	775.1836	119.2616
MAGEH1	471.4995	305.4487	415.7256	329.0612	246.9776	292.9872	212.0982	232.9995	545.3114
GABRA2	113.6502	82.20243	70.57645	142.0747	45.6336	67.45014	50.6415	41.84447	81.94112
GALK2	69.64341	1653.599	311.887	127.1954	490.3952	84.05564	440.8522	51.14451	77.2078
LGALS3BP	550.2715	305.8343	127.1463	282.6751	95.33096	168.9309	82.17007	63.84384	44.51849
USP6NL	747.8593	52.06846	568.6643	423.6126	444.6402	252.5621	136.1751	453.74	1056.994
WWP2	1346.856	1442.012	1582.162	3059.829	2661.584	3798.78	3608.568	1580.391	3492.36
EXOSC5	52.70533	51.22211	62.87509	48.34745	311.285	422.0463	337.0789	57.5797	61.82801
CDAN1	948.879	1822.133	3056.524	3004.346	2067.039	2377.051	2002.542	3264.447	2115.328
PRMT1	117.7093	105.6682	75.37408	724.1332	60.88054	82.91476	49.99805	427.4468	191.205
LAMP2	41.30962	179.1752	43.20762	59.67756	85.23239	128.5565	70.48454	50.06345	38.24971
BTF3	4709.644	7439.947	5230.922	9081.003	5014.828	2899.566	3621.637	3599.278	2136.186
BAG3	4805.313	895.1986	5166.235	4476.576	9147.798	3455.142	8137.335	6050.103	2650.926
MRAP	117.3544	99.61924	69.26543	112.2739	58.7403	58.17598	61.17219	55.91102	46.46687
KCNJ15	73.97858	37.98549	44.2254	46.38392	48.76762	76.22605	45.94141	45.86438	46.4333
CNGA2	48.22623	37.06694	50.72781	65.92379	54.49969	114.6422	40.8867	36.54411	56.4777
PCDHAC2	213.3978	127.7173	89.50924	135.8352	78.49512	84.84007	63.54176	89.25574	421.0838
RUFY1	67.94198	41.47045	72.31595	52.94243	117.9406	70.93161	94.17051	44.50855	56.01609
CSMD3	36.28924	47.91383	60.39799	72.90784	39.50384	35.92235	36.26792	42.8023	40.13948
STK19	47.7693	57.79863	48.74862	69.98986	285.2041	1175.277	841.4611	1204.777	43.80615
CCDC76	85.05781	666.8549	589.1238	475.9357	778.4919	401.4526	517.6277	174.2196	117.3822
HOXD9	46.52502	347.194	57.38997	43.92046	36.46044	37.49192	41.01106	84.54987	39.7256
BCL11A	3606.725	1883.746	1378.037	973.5123	518.9471	480.4029	743.4783	1283.39	4532.925
ATP6V1C1	33.7326	468.7788	647.0615	37.10696	1045.827	680.3024	1054.571	1368.617	1403.688
OLA1	57.92476	45.90086	51.98577	56.29105	49.75991	37.92544	107.8043	48.71119	69.98737
PCDH1	59.24077	57.08684	57.99163	128.6745	59.90363	52.99016	59.70112	62.46809	149.1024
RAET1G	51.01417	293.538	1940.126	40.41803	2162.623	2051.618	2131.064	1942.147	34.03685
DNAJC3	3014.027	1126.911	928.4765	2812.669	1644.694	1034.714	698.5153	1702.291	4314.419
KIAA0355	75.91189	653.162	228.345	54.78677	1787.115	55.63503	698.8215	534.1961	44.82685
LOC38983[illegible]	119.9669	155.0825	70.94006	87.36292	78.32257	86.54449	58.58312	62.19274	189.0619
PWWP2B	76.27615	57.44514	58.46811	81.33658	63.48363	90.85124	56.84214	39.61483	134.7652
FAM167B	386.0318	71.2467	97.93909	73.08266	106.3871	122.0826	126.0812	128.5921	186.7053
SNAP29	33.1236	32.83709	605.5684	40.57416	632.8288	464.7685	523.8784	302.0967	46.75831
SFRS15	2113.197	1196.664	721.5852	706.5088	302.553	558.8193	272.6487	644.0026	86.01898
ACSS3	331.5066	162.0599	105.114	185.9951	69.28394	129.7274	65.34201	61.16177	360.0068
PPP1R14B	59.29872	449.7195	1749.921	1372.912	1504.008	792.9082	2701.741	2094.705	1006.333
43800	58.7115	67.66171	60.614	53.16629	56.57868	65.44248	48.33171	52.13393	126.7474
MSN	73.08784	617.6531	1648.328	1158.718	2921.96	1179.045	3100.417	2841.035	120.1703
FKBP10	46.59739	352.2856	41.97839	44.79299	47.71632	63.93176	130.5603	52.57101	54.94932
ISG20	11873.96	19364.9	15934.54	12071.49	8598.467	20221.81	16217.51	14418.21	11955.16
C9orf98	235.8591	143.22	59.5838	105.4473	80.9992	70.98308	133.5966	92.47366	216.072
GNAQ	38.36101	62.41544	127.1937	290.1592	78.03776	215.0968	63.96243	88.94261	762.6223
ST7L	3764.68	5024.514	3186.33	3601.995	2024.99	5421.222	3891.288	3747.162	180.101

TCF1	226.6598	99.40424	77.74496	86.5133	74.11517	126.9943	68.5998	101.6773	172.8035
PPP1R15A	10464.47	9304.771	8486.694	9918.661	9499.692	9152.03	9185.572	3665.407	10910.63
ZBTB24	169.0312	232.4785	705.3496	369.9572	619.7067	503.468	594.2368	541.8497	248.3485
BACH2	83.11852	90.01737	57.80902	92.95824	53.14037	62.31739	46.65701	272.2355	97.32854
APC2	53.30671	49.57354	50.20839	58.52401	51.50173	45.72923	51.47506	45.69037	103.5481
MTMR2	126.2988	1249.138	1708.361	667.2612	1964.943	2053.985	2383.546	2260.105	1963.954
Septin 3	98.14926	484.797	276.9217	63.71373	134.1429	62.64008	77.04635	65.30758	66.10542
IL4I1	78.24659	51.42909	37.51886	59.49331	39.27078	57.30978	83.29957	121.2982	65.74626
C1orf216	153.3468	534.5154	598.2014	153.8725	513.9938	329.879	635.5056	559.0523	215.306
CLP1	422.547	229.5322	190.6743	254.8753	168.0027	191.2811	205.0462	152.881	590.6064
GPR152	163.9142	104.4885	75.67725	95.70358	70.55358	102.1367	66.79465	85.37805	299.3539
MEP1A	55.2943	47.57683	53.97889	51.68639	53.81797	57.25646	43.41866	34.24774	150.19
RSPH9	74.08549	43.5883	46.05176	63.48358	141.8663	42.15697	44.09653	72.37895	57.73643
C7orf68	460.6242	271.4178	248.7451	412.9755	208.0695	269.7033	152.4888	194.1676	558.2825
SLC34A1	169.5456	137.5695	78.4847	152.7873	79.03677	86.90761	69.02604	85.19259	291.6685
FLJ32658	93.60307	67.59239	48.25433	52.02197	43.60286	37.34152	41.82262	49.92548	62.1748
M6PRBP1	738.8247	438.6354	2773.238	659.1441	2733.119	1094.726	2161.165	1922.442	106.5478
P2RY6	272.1187	74.49448	571.3405	80.69475	123.1597	438.4052	730.3463	150.2464	174.8562
UBXN11	423.6667	234.2674	128.0194	273.621	153.9007	200.4447	113.8849	226.5758	533.7501
POLE4	295.8739	859.0346	2362.187	904.2186	2034.918	1375.845	2747.547	3106.306	381.1371
RNF44	36.86001	435.177	410.8004	358.1114	907.8981	938.1265	748.4528	485.579	39.75035
MYH11	5139.327	12899.13	4840.107	2119.057	15364.73	6916.305	7767.448	9635.235	3391.233
HTR2C	38.49196	61.41119	41.83015	48.14167	34.36117	66.44122	34.52689	63.48826	61.76262
UCHL3	44.91751	802.7775	763.2823	1455.147	1548.259	1438.36	837.204	1167.936	88.42072
CDH7	49.21684	39.20499	43.27158	88.21371	49.67864	41.8971	40.47356	44.04761	75.84343
MMRN1	2290.46	2990.92	1951.103	119.5137	3921.424	3714.261	3055.922	2589.644	2778.955
SFMBT2	4760.343	4845.662	2196.292	2810.161	900.0891	1694.277	1465.588	1508.362	4612.222
ARR3	56.6992	62.17092	55.48435	38.57198	39.13054	40.22976	45.71331	34.33313	78.20059
TCF20	446.7517	235.1293	470.226	925.8303	748.4708	565.2886	426.8282	514.7127	585.0976
ZNF582	90.09869	70.8901	104.1363	113.8176	56.00872	50.98983	59.58345	109.762	67.05681
MATR3	94.07324	101.2688	52.09003	106.508	35.70495	64.58552	49.31255	64.7487	67.91604
SYF2	3871.454	3920.987	3281.667	3643.445	3014.865	3700.257	3166.456	2872.571	4383.986
ZNF529	1987.074	2633.834	2825.692	2724.542	2406.064	3158.042	2897.268	1611.161	1804.903
IFNA5	38.45705	75.0434	44.21216	101.6033	35.77123	41.05944	38.22523	49.5787	64.55316
LOC643923	56.79456	33.97546	44.0404	51.61509	43.10198	53.02268	42.88895	41.16269	55.49408
ACAA2	462.0555	473.4466	684.5691	35.43156	1371.291	48.9163	799.9099	536.3947	150.7237
HCRTR1	57.30088	86.09048	53.37308	48.08733	56.95123	66.56034	44.53697	42.2165	152.4797
MTBP	36.69139	47.23248	103.6723	48.73724	55.06903	37.53318	290.842	131.2052	50.45757
CREBL2	501.0917	301.2628	218.2642	343.0928	172.8999	248.2941	182.8155	217.0683	628.1439
PEX5	412.1064	222.7678	236.7957	289.3765	300.0493	376.5681	386.2413	193.6671	564.5897
TDRD6	138.2795	102.9425	94.56918	251.643	94.22417	89.57675	67.97746	97.81964	233.4615
C2orf43	1033.913	820.9226	209.4674	122.8278	82.87151	309.4552	211.5632	279.9256	125.3932
POPDC2	112.1916	510.5793	148.9166	78.03935	139.5633	299.9362	52.45591	277.7412	165.5899
H2BFWT	35.65124	82.48025	50.26473	56.43641	42.59227	43.58524	34.0629	53.44308	56.13858
PDE12	42.56609	247.7526	240.3521	38.98427	398.3595	191.0344	319.843	139.4866	63.83908
ITGA8	3114.931	3617.259	2125.489	1062.776	1985.384	1584.717	1221.037	1792.322	2983.188
LEFTY1	119.7836	53.56353	46.6652	47.76419	44.08265	48.09161	55.83891	64.19376	51.34015

CDH24	380.1544	183.1062	98.96957	206.011	102.0193	151.7992	79.50054	110.0409	408.2509
PPT2	65.71672	189.7065	72.09143	70.60775	55.85479	65.64848	62.5573	42.14936	132.1304
LRPAP1	279.2808	113.2837	76.50199	128.3085	122.0443	95.64507	107.4224	143.6925	247.0984
NGRN	2993.396	4052.447	2518.212	3341.76	1168.164	2044.357	1172.891	1956.173	4014.041
PPP4R4	46.13604	40.96927	44.43505	43.68139	35.26807	445.7181	108.1597	50.39891	61.79569
NPY2R	64.09749	37.18877	37.68593	65.91172	50.41403	53.60457	43.86459	46.54379	94.1488
PSCD4	2879.94	8394.007	17667.9	3691.473	11556.95	6625.898	14605.72	14209.82	36.49696
TRRAP	131.64	138.1537	1270.182	235.1875	2498.88	667.157	1864.368	1318.969	130.1704
GRIA2	50.22452	53.54772	35.48542	46.27927	41.62376	33.42464	39.92721	35.23297	52.08556
XAGE2	60.88801	67.42638	32.36356	66.34957	34.70159	58.01075	37.44867	36.94973	40.34781
C22orf40	892.9733	51.00606	168.7699	43.30256	47.61101	275.675	136.7879	115.8878	89.47447
BRUNOL4	61.09324	40.38449	33.77719	56.68082	36.82296	37.62039	39.60629	47.73895	58.46427
GRINL1A	35.44587	39.22213	34.84973	55.93959	37.60778	35.43919	47.26894	41.39029	51.07172
OMP	33.83433	38.45916	36.85532	39.91027	32.99458	35.26948	34.27325	40.08798	36.69006
SGK2	236.44	60.6656	74.14422	71.14357	68.02114	79.44357	56.66475	74.88194	97.75066
CPSF4	22613.64	22614.73	24232.7	25474.22	21630.46	22847.43	19244.16	25497.43	20448.87
ARID1B	673.7322	67.37408	119.6398	66.14884	46.49744	272.1202	64.15127	85.35071	107.3549
HSPA1B	78.15678	890.8872	1618.806	925.4483	2969.604	1550.915	2554.455	2198.802	263.6844
FAM108B1	53.72593	45.63863	236.2711	41.03424	44.12172	51.04344	119.0044	114.2306	39.35196
BTNL3	42.46608	38.90555	32.51259	36.68349	33.02702	34.69363	32.84651	40.35357	48.06239
MCOLN2	134.356	62.94355	331.84	63.91656	252.8664	298.4314	481.2895	218.8117	164.4688
RPS27	1003.67	1167.98	1779.704	1280.985	1870.023	1099.617	2478.544	1625.455	38.33068
LY6H	941.668	613.1489	485.0469	529.5964	117.6479	915.1391	841.135	1007.82	66.16621
LOC34006(	73.1835	60.12643	63.16035	65.29117	55.46086	96.33435	55.92245	57.51521	75.75956
C3orf70	36.4382	108.842	57.5602	130.5547	64.66595	65.84167	62.65881	50.47581	1537.575
RPN1	321.1321	2769.97	2735.412	2320.942	3937.034	2530.871	2813.373	3869.787	1461.801
USP50	34.95426	44.37575	36.06585	52.67114	37.7458	39.26643	39.14399	38.08768	37.7284
PCNXL2	37.81898	336.769	340.0441	781.178	94.38359	242.4131	229.9794	177.1061	63.29234
ARRDC4	53.07829	460.9831	252.0024	663.314	47.05403	712.7337	345.345	363.217	76.09725
ERC1	297.2819	184.9777	90.07113	179.7355	77.43362	149.6646	78.9516	102.8546	251.1247
GPR177	73.72914	45.75481	185.3502	60.43188	276.4097	201.5067	201.2956	164.7459	120.7379
CRAMP1L	92.20216	57.4286	45.74289	51.87623	50.57376	54.72115	53.76881	58.14631	54.37579
BMP5	125.3934	139.2205	91.58891	181.316	69.91858	135.3912	61.62197	93.43574	120.2737
ATP5G2	4033.563	1990.223	3361.562	3623.852	1511.921	2412.86	1955.392	3473.252	213.1045
CRLF3	992.8145	541.0287	1459.247	1604.688	211.9842	916.0529	779.3806	1075.717	274.5209
LIG3	551.0784	1209.466	1033.502	1694.273	759.7958	1088.255	1088.965	1091.721	52.63437
C15orf40	304.7195	237.8507	136.5117	260.5006	115.4713	190.2269	107.8635	146.4297	588.2191
C1orf43	138.0378	253.2485	227.4221	281.0076	107.7867	261.3554	187.888	204.0266	117.0349
GABRB3	57.54707	36.02535	36.09621	49.38864	209.9451	37.15471	39.01178	50.64735	59.69303
PATZ1	39.19395	36.75363	38.1676	36.48305	35.81177	34.4285	35.85727	34.16598	34.5608
LIMK2	2791.637	1287.133	1859.804	2193.495	2399.09	3142.557	2331.104	1267.224	1114.73
CAMK2N2	37.28687	42.51067	36.96433	34.59254	37.67056	47.80532	44.48402	184.4449	37.39253
LACTB2	606.9094	949.842	494.1017	546.486	466.8648	1081.485	690.7859	819.0501	651.2328
WWOX	459.5164	151.2193	103.1206	295.8078	133.3788	77.97575	163.0283	123.4012	703.1288
TSHR	32.83297	34.61536	36.67376	32.73606	41.00766	41.54451	35.21442	46.86631	73.48065
CDCA2	78.30961	1309.882	70.98555	800.6738	368.3662	462.9138	295.9279	76.23515	69.19352
TCP1	104.1411	86.30814	429.6897	106.8868	105.3018	325.959	264.8082	231.8394	233.944

DYRK1A	57.80983	751.4114	598.256	594.0868	319.3652	302.398	324.8208	190.1298	77.20582
LOC642181	88.31122	260.4146	58.62209	74.11311	76.20881	104.1914	65.04443	71.16937	242.9421
RAB31	3274.699	1661.635	7356.427	1789.087	9215.931	5463.433	10914.02	5984.046	115.039
SYN1	62.81442	51.28832	35.58222	61.38823	40.37551	33.32607	30.69885	33.35686	48.06543
GTPBP8	771.7725	596.0259	386.884	647.4297	270.4458	664.1087	357.5545	532.6254	809.016
GABBR2	68.04102	44.34373	39.93139	48.46106	39.10708	41.36692	37.33401	37.32035	53.57978
STX1B	50.21141	64.53258	32.03599	50.24868	39.06601	46.83485	42.74054	52.13225	72.24562
RDH13	42.98037	40.23928	2713.038	1478.329	3438.616	1022.018	3124.501	1641.048	2549.145
ENPP5	1899.839	3269.559	1432.785	42.4966	1506.061	2239.504	520.774	1533.393	1360.381
ATP6V1C2	57.17594	689.8407	33.97178	35.92809	1023.859	37.99437	156.1154	34.86388	35.44593
SLC7A3	121.8386	40.2518	54.88472	55.78835	67.59653	61.36915	58.20177	53.6018	108.4621
PPP2R2B	2023.793	99.51443	2931.508	4690.752	1858.505	2523.516	3164.473	3246.553	70.22736
ATP2B3	151.7014	90.62343	88.68275	132.7513	82.57178	97.40911	65.23595	57.71585	145.584
ASPSCR1	25616.11	25854.38	23181.99	24911.42	19455.51	24459.2	21247.43	25330.14	27564.08
LOC650152	1992.334	2171.238	2317.769	2725.795	1766.86	2356.989	1763.837	3235.745	1672.709
HIPK1	32.93708	39.35427	279.4846	532.4313	41.02279	44.9535	95.40641	36.18217	36.72844
SUCLA2	47.92506	548.4078	629.2741	750.5586	72.09344	40.35886	282.4808	175.0055	2135.764
PAQR4	8524.185	7522.006	7711.681	9781.189	8082.354	9522.54	7885.064	6119.518	9498.492
CSRP2	2165.031	2969.041	1997.192	3104.558	1709.011	1807.666	2332.367	2495.445	2743.287
WFDC1	35.27842	57.11974	35.62781	49.71111	40.16922	39.2316	43.51962	161.8333	62.74252
TTC17	66.42146	48.87577	81.86882	70.09754	130.1171	68.90116	134.3398	113.0408	85.09428
C14orf11	85.84021	131.5903	116.1462	179.5494	113.0909	193.8047	61.87605	183.7691	323.0988
CAB39L	40.46678	58.04539	37.11991	36.15635	40.11838	36.57401	32.91796	36.85615	78.57677
P2RX2	138.6242	125.2381	74.95038	112.4033	67.25536	72.09785	59.20231	70.02815	344.3909
DDB2	138.8726	1096.127	581.6406	646.8166	378.2536	63.97944	545.4285	331.6139	1723.931
CLCN3	3733.78	2661.979	2829.146	1553.203	4308.076	2460.06	2077.632	2300.299	2630.869
PPPDE1	2062.073	1138.868	782.0968	1211.326	408.365	55.28616	351.754	659.3787	73.86929
INTS4	97.49883	65.57137	252.1245	86.93246	51.94432	393.1985	542.563	445.3434	82.20657
ZC3H6	173.4086	76.45712	92.69603	101.4477	108.9789	89.55856	139.0482	100.5083	175.339
AMPD3	80.3327	65.19648	54.11771	99.72412	51.11777	54.05624	46.65547	42.25357	43.97939
ALG11	141.5526	129.9558	140.4828	127.3502	172.1846	73.66032	84.46355	104.0105	70.45739
RIN1	490.8946	1023.023	2257.182	1396.555	1003.007	820.5615	1618.431	1927.283	622.8328
RHBDL1	33.90156	46.43346	49.42658	457.7797	321.8658	363.1953	246.8814	298.2632	47.1678
ZBTB8OS	782.1144	51.01896	141.9404	387.3067	119.8534	54.93438	93.73971	250.4518	37.8404
NEK6	5930.143	3181.393	5823.136	7391.389	2072.292	5190.824	7190.766	7322.716	2146.506
RNH1	47.95416	57.33696	958.5292	413.0541	2449.716	538.0687	1228.065	903.6179	66.60202
CHRM4	44.23072	49.24171	46.62708	41.11728	33.09477	36.39897	36.599	42.84861	33.30989
ELK4	670.1839	522.4497	232.4796	357.4137	178.2926	217.5486	250.1281	178.0692	857.6896
ACADL	40.14736	42.52179	35.40668	477.6828	43.56286	38.43431	34.72848	36.69416	50.72037
TRIM15	36.57191	35.92892	35.10218	35.44095	32.47683	36.03152	36.43729	33.03197	33.11575
PTPRE	154.4879	119.8527	87.9837	142.7615	69.19984	219.9492	59.55079	81.15463	139.8462
LOC284296	271.6958	116.5003	77.35201	186.6966	78.38869	125.2828	62.43397	90.14453	282.551
MGC5139	311.6676	153.4524	80.43461	209.2275	90.13683	141.278	76.68216	109.8607	362.5955
TOE1	176.1131	99.62103	67.77425	96.7553	62.71711	52.12016	62.90635	89.65745	198.4755
CCIN	39.62867	68.70074	46.6457	55.04529	43.7322	57.72019	51.76805	64.47746	58.13361
PRR22	47.21168	810.1584	50.18614	35.0996	34.4329	40.08586	258.601	34.38513	41.09842
ADAMTS12	51.39775	63.26344	38.11569	56.22667	55.62693	52.13062	48.58453	50.55781	60.9561

GTDC1	46.13383	38.51039	51.54193	42.61959	40.67255	31.69956	38.75241	41.80346	38.636
GAD1	728.3054	1027.716	555.0368	2193.221	447.9961	1691.479	1927.703	543.1385	1156.258
OR11G2	112.9136	96.63383	58.31092	166.481	76.33056	109.1047	67.47852	69.33314	298.2282
PER3	178.7208	76.68283	74.07938	161.036	60.72155	75.6641	60.11338	69.9818	75.15861
AMH	37.25711	56.49374	44.30427	41.95787	49.10199	40.73388	100.7368	157.6921	49.54043
RPS6KC1	11388.57	13197.64	13373.56	12381.81	9917.952	9745.934	9448.872	12295.28	13005.07
RFWD2	1037.827	3187.986	3778.487	2818.58	4449.814	3948.016	3739.753	4739.214	1364.882
TREH	117.9591	153.2526	63.2121	53.43093	65.08365	99.91025	53.2127	47.89246	237.1711
FLJ25328	40.74257	34.59879	34.3991	33.90703	52.93982	48.16249	37.04931	55.15306	40.33526
ZMAT1	84.19365	105.7701	72.41068	99.14754	275.1791	86.61813	175.4261	72.78719	92.99266
FAM26A	47.24938	41.79838	40.5564	38.45906	112.7543	36.32615	175.4485	41.95657	50.77085
MRPL30	151.6643	128.1838	615.7431	562.7267	258.9579	372.4618	235.0046	439.48	157.59
WFDC10B	259.2989	95.63431	75.89137	459.0648	110.4538	232.1292	93.01865	125.5529	248.4376
NFATC2	800.3942	63.77374	653.3369	190.9783	264.6656	229.7596	173.6191	143.256	51.51225
ATF3	63.03383	50.16418	48.92253	40.23338	35.92374	34.51476	39.76294	48.86666	289.9444
PPP2R2C	40.43985	272.428	44.43817	485.6156	117.5463	54.55349	138.2542	93.8938	799.5858
SH3RF2	4904.698	5518.041	3165.574	4108.219	2573.16	3301.067	2704.043	4693.75	4515.242
GON4L	33.89518	1924.042	2139.829	1519.988	1975.634	2156.569	2142.758	2310.052	1276.974
TMF1	48.61131	499.8933	499.9241	61.12586	633.0021	51.10912	291.8965	306.2923	93.82457
USP31	421.724	349.8411	103.9349	450.605	85.6067	219.203	76.85267	94.71046	848.7252
SIAH1	39.82752	48.8408	45.09517	48.88073	51.21137	46.42537	41.26315	47.41972	73.57307
NDUFB8	21492.18	20447.86	24072.06	21136.44	25628.26	26234.89	24935.72	26387.52	17949.15
TRIP4	74.73186	735.2157	527.1808	702.5412	70.67892	824.1482	233.6305	322.159	42.30886
NR4A1	1778.419	152.0206	265.0583	146.4463	632.9296	96.13387	139.8474	81.74466	279.9827
ZBTB41	103.574	77.50249	56.54239	63.52695	68.60688	67.5451	58.53604	56.53847	114.1972
CLDN14	949.4986	1042.882	576.1038	441.3199	443.9357	886.9173	372.9805	470.9736	877.3373
CSF2RA	56.63269	48.43449	103.8842	34.47752	79.696	203.6612	88.42809	85.559	57.42718
CLCF1	57.9523	67.59802	55.46598	57.21897	386.6869	72.6127	553.8459	56.39219	131.961
SPAM1	58.28193	65.77171	51.57722	98.24429	49.58581	45.31959	47.22522	46.68009	67.46654
SHMT2	7420.206	8481.013	7109.201	10728.93	8415.439	6997.61	6584.881	6906.48	8350.959
HLA-DQB1	96.89895	123.3581	130.5537	73.21143	57.87778	57.84568	50.66943	119.8255	106.9814
ZNF224	126.3773	529.1895	156.4152	69.63506	167.6739	189.0216	244.9124	348.9509	64.68913
TLE6	49.77355	352.7618	129.2879	77.50595	139.0093	213.1189	70.48238	148.9032	620.106
JRK	135.2694	93.84816	55.08797	123.014	65.1512	60.99339	153.1345	255.155	142.418
XKR4	39.18188	46.77179	48.27866	43.89198	35.76035	37.09697	44.68931	46.6727	37.74154
KLHDC7B	53.11203	55.74485	43.47592	44.67062	36.33795	47.67575	42.33799	40.17863	38.29213
PKP4	39.46046	494.451	349.5882	492.3512	196.5983	283.6339	36.95341	338.1775	39.89965
ZNF334	47.27683	45.97535	51.71552	57.54956	45.58736	49.34223	43.29403	42.15244	100.8664
SPTBN1	6334.952	3899.655	3109.654	5859.812	3841.814	4221.727	3508.159	2720.198	6063.753
SRM	694.5609	1677.767	2937.911	2916.048	2984.188	3059.493	3514.303	2840.198	138.12
Septin 6	54.25412	42.44943	43.43667	34.72855	50.36466	34.7607	57.3592	33.71883	42.63942
C17orf81	43.24528	38.10479	36.48884	34.07542	45.94964	37.8313	40.75887	36.04845	57.06503
CHRND	128.4598	479.0197	54.85213	54.96109	55.40796	77.37663	48.07324	37.84505	38.49945
CXorf65	59.50839	55.10406	51.05113	38.66829	50.95112	72.37329	41.82093	37.09036	110.848
CDC14B	69.71248	37.93373	43.5883	43.27053	49.60374	38.4458	33.17539	38.29836	39.40858
JUB	48.90359	786.7641	273.0479	250.6631	189.931	51.42664	40.33996	34.11285	2354.887
GRIK2	140.7013	115.8426	59.80278	96.53996	57.07693	136.1565	53.94506	47.78257	169.0397

LSM12	35.35945	39.41999	33.98095	53.01615	32.1317	32.9254	40.69088	44.90232	39.67011
C3orf63	137.7118	45.64012	38.26763	67.79006	57.37714	82.43274	44.74136	116.7628	116.2862
MAGED2	138.187	226.8997	94.29648	131.7716	113.7166	173.4607	89.23866	109.9377	251.1423
THAP6	40.36166	437.2822	107.3107	258.4417	46.05212	60.12806	72.38798	43.95066	45.17728
RFNG	89.84525	45.267	44.37547	91.7592	65.61259	48.14963	130.5466	50.49961	95.32255
ATP11C	43.44676	559.883	590.4082	72.85926	378.329	49.48589	429.1555	459.8683	57.95889
NUP43	5918.444	5631.106	5324.629	5276.686	4302.873	5222.367	5140.838	4973.035	5868.609
ACSM1	41.117	46.48058	115.854	35.39839	46.27158	44.64133	32.68747	42.43474	41.19134
FLOT1	2116.579	1170.539	2834.955	496.4633	3776.118	1836.023	3121.238	2713.994	168.5816
GRAMD2	46.02126	52.43187	173.5918	76.92611	46.43663	51.20237	90.18386	34.12481	35.93426
DGKA	245.4474	197.9066	106.5835	247.1907	72.50558	127.0527	183.6935	69.75334	453.9756
ARF1	454.3746	313.5012	174.0057	299.1777	143.5728	210.9647	157.2371	140.4609	637.2484
TAC4	47.4535	72.94927	43.21011	51.17542	41.93309	63.25432	37.68206	53.68504	71.64082
ZBTB45	656.7954	314.632	271.4998	417.9164	182.8864	320.6098	207.6974	288.4616	664.8262
RBM34	552.3765	39.73271	129.1151	309.0058	153.5619	210.8218	198.7824	230.1309	35.37135
LOC72990!	166.9988	54.0725	69.39156	197.614	63.04608	116.1036	66.18366	90.93792	585.3915
PFDN6	627.5558	631.4351	406.2214	602.3316	464.7907	427.9342	442.4005	594.9483	853.6382
SH3GL2	42.56417	49.62535	642.2653	61.18872	160.4377	419.6056	135.1817	47.3235	170.5272
ARRDC1	34.66397	33.70609	40.59528	35.83401	118.7757	40.50013	99.18938	42.93048	32.76641
B4GALT4	3723.529	2747.842	1031.816	3980.557	1290.113	1948.159	1309.527	1826.357	4349.806
LOC38982	154.2012	106.6099	65.08872	63.473	40.88608	61.69304	53.27724	63.0639	128.8254
POLR2J3	50.89922	55.72773	35.3583	47.34241	53.00513	38.91842	37.70272	41.523	73.44535
KIAA0408	110.0974	207.8957	82.15459	108.0262	74.33461	115.2499	68.7485	106.3027	355.4018
TTTY18	72.98818	49.67495	48.08337	62.33711	44.76582	81.69402	52.05313	64.32294	51.0434
LEPROT	3908.741	3782.81	3309.94	4614.907	3276.339	4821.612	6945.156	4922.044	4184.037
DIAPH1	67.83285	1502.875	1447.273	4036.447	1449.933	1018.848	1212.094	2647.424	3435.1
FSTL4	86.68434	44.94957	45.76391	50.27443	39.70295	49.07621	53.42331	58.31495	73.48358
APOBEC3D	52.995	103.0966	71.17069	58.77909	66.75652	95.46108	52.42888	90.01825	426.6088
MAFA	35.74409	38.76068	41.22577	61.11275	54.06908	589.3043	167.9207	330.1576	44.22407
CD151	1702.036	175.784	498.7186	2202.717	108.3395	176.5467	230.1188	401.1616	478.0302
FAM18B	1548.938	1292.534	308.2747	789.7974	455.7786	433.9143	299.4323	240.5972	1219.114
C2orf42	34.99036	42.80635	37.9773	54.32968	41.83867	56.04337	42.23266	39.25209	41.65799
C1orf53	53.81292	59.13139	230.227	63.80656	225.2448	40.50339	558.0507	182.4862	144.4554
FLVCR1	735.4535	640.098	500.8398	970.9644	338.2679	448.2208	325.5994	544.9468	953.5006
DDIT4	2061.69	675.927	1827.912	3254.815	741.2305	1023.418	1568.585	738.2302	1635.043
FSHR	35.73213	39.78381	35.3017	33.04606	38.00105	34.85534	42.679	47.18018	47.90652
C20orf85	92.39522	132.8696	63.85213	130.2818	78.87316	101.8217	65.91802	77.67902	141.165
TBC1D7	1266.926	594.0492	998.8887	36.91039	940.7525	71.09219	788.627	597.7505	99.21771
ARSD	91.25835	58.28231	50.09721	63.62671	46.69417	62.73061	73.00768	47.89618	96.52863
LZIC	1919.63	2082.858	1616.006	56.84171	1766.285	503.5851	1502.094	1053.317	41.69406
LILRA3	146.2937	85.98157	1147.173	1311.027	857.8773	1021.634	1228.542	1013.797	54.3164
ZMYM2	566.2924	311.8393	744.3768	619.9968	715.8267	810.9335	674.3716	557.6004	614.8577
TRPM8	82.89997	50.90675	142.8268	246.9604	98.96914	345.827	251.0491	425.8638	56.63836
BANK1	107.0207	60.79672	55.39281	120.8381	68.0121	73.39764	58.38349	74.16277	147.3697
SMEK2	233.3031	186.5168	109.2971	140.9959	95.38847	183.7226	96.52994	76.37082	38.90823
DET1	81.12984	46.50118	140.7342	944.8663	48.66057	53.95483	122.8232	47.95726	70.32265
VCL	83.90191	44.55315	46.28112	81.54185	53.22414	47.30717	67.96465	44.57848	48.92051

IL21R	291.9448	2393.657	120.4702	200.0577	129.2973	151.604	148.7102	324.2904	2474.033
IRAK3	117.2485	2237.177	2320.965	974.1066	1454.78	2078.719	1331.166	1751.896	1508.796
G6PC2	47.97313	33.97609	42.00821	38.37987	32.37167	55.88275	33.005	41.07764	31.90177
LGALS7	18909.47	17800.99	9708.977	19250.99	16770.14	18627.98	15609.75	8391.838	17097.54
TFDP1	4755.138	4052.945	4210.965	4015.019	5019.56	4898.052	3681.421	5884.014	3088.009
FOLR1	47.49277	42.15142	33.48914	38.8543	203.1316	42.86783	1779.466	37.57954	46.07763
C17orf103	36.90023	45.35238	36.40401	32.67807	30.44038	55.70415	67.14977	42.53374	37.93564
VIL1	68.88614	64.57948	59.97665	53.60875	49.62757	66.94353	50.69544	51.71153	50.1043
FAM129B	19897.5	16112.7	22827.17	18641.64	24702.79	25179.63	22962.91	14749.16	16867.67
JMJD2C	102.8292	929.0679	66.93208	48.74303	209.701	48.35535	96.17589	40.60997	50.05928
CENPJ	42.53773	479.6867	304.3434	1230.675	419.1841	345.7238	394.0051	177.9828	35.10226
NR3C2	104.418	239.2011	64.70143	88.90614	74.66138	81.24854	54.64087	60.93895	273.4049
MTL5	56.82414	41.56581	41.07934	47.34501	151.4117	38.21687	88.70883	35.1342	55.84874
WNT5B	42.41951	408.7798	274.8346	1080.862	38.69735	285.8864	113.2616	42.02094	84.16033
HIGD1A	51.97223	674.5716	891.8605	714.0234	1440.781	929.843	1052.537	519.9597	79.32614
RAPGEF4	219.583	275.9119	122.2707	177.4476	156.1457	139.034	101.8936	107.3035	283.9186
CTF1	410.4647	251.9367	391.7168	252.3462	1222.008	1118.636	908.9045	507.3658	574.7385
PREX2	67.88358	593.1576	35.28643	49.65656	44.84163	45.75359	142.4054	50.33038	58.08254
PRRG4	36.92231	513.5178	544.5159	33.27745	348.6949	218.9564	201.8005	256.6027	60.58492
TNFRSF6B	61.99369	49.80786	45.35839	68.63568	49.91136	40.07728	38.36945	41.04365	60.42926
ANKRD12	422.538	111.1534	73.33779	89.48576	149.2189	80.4044	267.0946	134.9402	48.10665
OR52A5	90.20429	49.83504	65.61995	63.97832	62.1093	61.26586	59.24441	54.79573	61.81878
CNTN1	44.91206	36.17438	62.76223	52.56133	129.4956	59.94041	38.6901	63.73212	57.32052
CSF2	77.51011	52.2517	259.1963	1494.626	80.74277	827.5399	907.7486	189.5915	48.63526
APEX1	937.631	506.4898	1474.209	2401.156	1491.619	893.5291	1489.197	864.9671	3053.612
MEF2A	38.16708	48.98269	557.2094	39.08121	199.9401	333.23	219.1664	347.3421	53.35059
PI15	696.7665	141.0407	272.2166	1661.515	203.9873	453.1433	403.5186	158.19	235.8489
CYLC2	114.3627	85.90002	63.32391	64.82658	54.06836	59.51017	55.16868	56.01997	133.2442
SGSM1	37.23734	55.37263	35.32148	34.90379	45.92482	36.01023	36.00012	38.48148	47.86683
CAMTA1	130.9363	96.46047	186.3867	52.00151	332.1329	57.58896	166.6198	43.46241	264.0712
C14orf156	46.48859	41.4322	346.6412	655.8055	1241.808	300.636	992.082	747.0464	81.23097
UGCGL1	31.99903	35.4883	37.18036	35.34763	35.92419	39.34766	42.33448	42.31002	41.83101
ESRRG	59.35423	180.881	42.84731	67.36931	52.69635	153.1809	96.00922	94.46906	39.12688
METTL6	52.31416	499.9977	186.6931	509.6266	120.7135	528.834	274.8313	162.7345	37.67826
DLK2	148.5299	83.49886	67.77711	77.21218	66.32991	69.64145	53.90852	52.94519	156.9843
SCRN2	127.6548	117.5047	80.66528	125.4502	84.08527	109.8032	73.70407	124.8417	388.7003
ZNF326	279.9751	627.73	109.7861	646.4275	263.9753	128.908	236.2079	244.0806	252.7864
KLF7	286.7803	165.0148	94.3856	279.3814	102.0278	150.6242	85.00453	120.6808	483.0813
BTK	275.4766	458.3699	789.1619	220.6068	572.5366	520.7932	594.5648	408.3321	247.3541
LARGE	14439.05	14200.43	12287.1	12216.33	9648.699	12695.15	10117.25	14562.86	13095.21
AGPAT5	45.36343	44.58316	35.84857	43.70155	42.29697	46.51909	34.39025	34.23505	37.65356
WDR92	4655.774	5218.935	3730.635	4447.55	2929.912	4596.5	4364.148	2846.685	4712.438
HTR3A	84.97706	57.56979	44.85179	61.83667	209.8292	47.54099	46.08908	53.08845	48.47289
GUCA1A	204.7525	123.3952	94.17428	153.7461	82.72586	129.7985	74.8451	39.70821	342.9028
FLJ43860	216.4023	207.9002	87.40524	214.1031	68.16682	146.023	76.13327	114.0234	467.4979
TCP11	37.44499	33.42224	39.14935	36.95418	152.4834	36.75647	183.578	127.8751	76.61855
LOC38772(	68.44191	43.79336	48.22276	71.55965	42.62555	40.43648	51.14076	48.12296	37.16785

MATN4	33.80436	38.03673	37.1769	35.85544	36.76716	34.72125	39.0423	35.67863	47.81837
ISY1	690.5803	47.99859	118.4042	245.7205	167.7234	445.9253	162.7926	308.3109	165.4258
RCN2	71.0368	51.44124	152.7788	89.19964	250.5658	72.97221	384.8817	285.614	80.20934
SGPP2	37.26855	36.12143	46.69164	32.4103	42.59406	50.70648	35.4204	38.27932	35.42083
CPT1B	65.5066	146.0186	85.28959	93.13663	124.6427	141.5831	90.46631	104.9781	75.80058
MANEAL	736.6187	1225.675	931.7745	55.66543	1705.741	1750.981	1075.517	430.4101	50.36226
SLC22A12	38.17524	46.00239	43.45831	46.06348	34.83039	51.3231	40.31322	38.08619	48.85172
PRKCZ	47.81825	92.65212	44.8943	48.81947	56.48901	54.26168	53.25823	60.35423	51.20623
TBC1D13	3407.789	3352.479	6510.254	4084.427	5175.079	5164.77	7069.975	7191.103	1663.82
PTK7	34.51855	35.81704	501.6047	2210.312	711.6683	560.4766	642.8413	845.837	1870.744
SP3	70.3961	359.25	42.43214	107.4175	43.56217	60.71312	70.47394	51.38319	105.5706
ORMDL3	1968.016	35.78248	241.3315	1526.398	658.4891	41.69063	647.2529	987.1877	37.20391
C5orf34	80.89064	118.0691	76.99886	172.2437	67.45065	569.5898	578.7973	55.71427	356.1332
WNT11	73.63599	44.71265	47.12379	35.05534	126.2084	177.416	319.8076	191.3654	66.3529
ZNF248	1027.616	1442.622	1119.8	855.2458	1086.163	1282.402	801.7441	1280.578	972.6571
RANGAP1	8324.237	8883.614	9196.869	9726.494	9114.103	8270.128	9560.998	10263.63	8565.805
PAH	160.4574	73.16496	61.31252	78.31996	43.61575	51.89542	68.46293	44.02782	67.28232
DCTN2	7849.465	8477.112	11510.49	9535.178	12701.8	11028.05	12277.95	13210.32	7846.601
PARP6	43.98168	34.52666	37.32017	35.20221	49.36882	60.27428	40.85277	49.94355	47.49314
MDP1	211.5582	146.3285	79.43484	162.8216	86.85697	130.6745	70.47281	99.42562	361.7473
GDF1	381.0508	58.39084	122.0836	201.3498	95.83009	144.4035	71.86097	65.50834	447.455
IL28A	51.65835	50.33192	55.18613	66.00389	45.40581	37.14421	48.63543	61.27412	60.74669
RABL3	459.2125	606.5924	151.4467	336.8468	138.6476	374.038	167.6216	190.3157	630.6793
VPS33A	193.9464	129.9378	81.46499	147.196	79.27753	123.1874	65.0753	100.7225	232.889
CCDC59	36.44706	37.91289	1076.05	37.78343	424.1832	342.3967	758.2381	952.3029	33.36053
ANXA7	52.23895	42.73656	44.61565	100.8436	65.80832	57.75289	44.86379	50.30269	50.52536
CNNM3	2778.515	3106.353	2560.079	2774.11	2137.819	2468.381	2458.687	2533.231	2920.647
PDSS2	37.14259	32.78802	559.447	461.6492	449.4063	186.4612	611.077	555.8625	59.77677
DNAJA3	2350.365	1794.132	2813.513	2555.99	4323.336	3658.825	4141.009	3847.799	80.21098
ZNF358	7155.166	5895.204	3621.666	7023.622	2605.119	3764.774	3501.703	5272.705	7216.309
MX1	33240.11	35563.28	38205.19	35431.99	32326.04	37293.57	34494.49	37234.33	35793.66
LGR6	66.05865	57.92125	59.96474	58.88524	48.02431	37.28519	47.16985	35.14328	48.3672
AARS	24257.87	21641.75	20382.26	22573.28	25008.16	24215.16	22453.95	17352.65	25328.14
GFRA4	97.15856	82.09841	70.40759	53.86539	67.78976	81.86032	44.02869	53.6361	180.4338
ZRANB2	5144.687	6582.142	5109.733	6711.438	5101.68	5510.525	5195.772	4399.222	3259.465
ASPHD1	46.41421	41.68585	153.3685	51.75792	34.29631	39.77335	35.6105	37.98318	33.05323
LRPPRC	274.9116	1138.17	577.7625	118.1473	1190.757	384.773	1227.11	1144.228	287.772
COX7A1	1805.949	1846.784	873.0674	48.21484	1302.896	508.6249	1479.471	1651.568	707.8091
AP2S1	49.99159	375.7007	139.3597	80.08801	99.708	210.297	220.5033	58.0602	90.20537
SOD1	3595.565	3394.713	2750.665	3443.926	2992.986	2741.48	3351.623	3749.922	3959.634
FCER2	116.6792	85.81898	217.5385	57.69067	47.87194	61.99543	120.7979	53.66844	73.64182
C10orf35	1924.703	37.42531	669.3807	52.32658	375.6789	1775.162	1752.871	1875.423	59.15587
HTN1	35.70495	35.52604	33.05504	44.01186	39.76808	34.94207	34.99997	34.87893	41.39354
SMA5	140.9944	385.9106	198.7157	57.02217	142.3073	231.9099	106.8737	141.7611	63.76318
CLDN18	135.817	117.8097	70.18522	92.77429	104.4921	102.2606	74.12621	107.1365	170.6237
MED23	42.81566	528.7902	56.74546	55.16585	39.30472	47.93428	102.8459	39.91846	59.92155
GDNF	208.2898	90.36642	71.934	103.124	77.80876	97.35995	81.69672	62.18481	108.9499

LOC392395	74.67534	46.50649	45.47918	49.6813	40.3109	33.84418	36.90928	35.53125	43.38153
AKR1C1	901.3238	245.1947	138.9816	222.3548	102.4013	182.1037	111.5966	125.9575	685.2883
C3	71.77658	78.57328	149.1031	75.46297	174.9193	278.1626	94.7636	167.4195	694.4308
CXCR1	122.4363	85.04095	89.0237	88.65186	125.0933	133.6238	75.23662	90.36748	77.38895
C1GALT1	999.2018	719.6872	1056.076	924.7903	487.6425	799.0588	856.9467	717.0601	733.7458
PNPLA7	9017.073	8319.138	7192.11	3251.824	5487.901	6944.54	4197.389	7580.529	8473.821
PPP2R3C	459.0377	109.7711	381.1991	93.38543	235.1676	247.4838	74.41978	270.2386	88.5614
SQSTM1	3632.467	5400.621	8243.882	4898.022	8750.652	7737.187	10656.36	9917.429	759.0215
TFPI	61.09892	851.1029	448.6156	48.47469	415.2119	34.52185	327.9755	386.3303	54.1905
PUS1	10355.2	11506.77	16312.29	11984.69	16429.67	13871.56	15997.11	12773.08	6693.312
APC	175.7892	44.14229	221.6541	287.1925	374.536	248.1236	226.9629	308.8517	157.5373
ADAM2	258.577	161.2001	100.9239	95.84006	106.6875	132.8823	67.68745	95.80964	392.3637
AMBRA1	74.2528	53.85231	85.64109	73.47825	113.7367	136.6427	128.8436	89.12217	48.88954
USPL1	1053.479	2668.34	755.0721	902.4315	521.1354	445.0557	413.0429	495.484	48.59048
PKN1	54.5486	61.57639	44.24393	48.73492	45.42358	54.76688	46.38249	45.93251	66.53743
SEC24B	96.84868	45.26735	132.8326	49.64498	38.88247	37.82379	142.1559	39.4192	38.41887
GPBP1L1	2863.371	2869.444	2091.762	2268.274	1985.303	3067.975	1875.586	1407.509	1635.403
COL4A3	57.05282	39.20538	130.8232	38.4251	119.5489	46.72665	49.17718	51.96835	43.10357
OTOP3	152.9188	97.86901	64.12844	90.70236	63.23925	65.09283	57.78936	53.26391	119.3574
OR5K2	173.1855	98.83577	76.55397	98.47189	70.43155	105.7658	44.44418	64.86575	148.5812
ACSM3	43.56836	37.04132	37.86445	57.01192	42.09822	52.90298	39.57236	44.23029	52.74004
EDG4	1586.793	2539.541	2917.438	2232.47	2633.997	2125.029	3399.796	3705.93	630.3544
EVI2A	37.87541	39.07791	34.74401	55.06916	45.2524	53.58274	37.19148	172.4576	36.48978
NHEDC2	107.8245	120.3749	71.68205	78.15402	343.0112	58.80228	69.75214	64.0537	111.5826
ZNF208	35.88018	38.75348	33.94015	44.12687	34.12913	35.51286	33.33987	45.58003	36.11787
C10orf68	38.71235	59.91691	166.8688	58.73361	34.72747	62.18947	37.06774	170.0694	66.47953
CA2	53.97196	1418.52	32.9422	803.5067	299.0452	1036.666	956.1411	974.9289	31.11132
GPSM3	259.5126	433.751	75.46986	124.6158	66.67713	102.699	116.8631	125.0354	337.6888
MADCAM1	37.71516	52.66659	33.71755	45.19509	41.55119	42.44628	33.37925	43.558	36.99798
WFIKKN2	35.40535	33.15797	32.9748	40.28607	40.5405	49.2177	41.15029	36.6617	60.13363
PLEKHA1	55.96029	94.13171	186.5007	327.3703	412.7131	366.9292	292.5862	247.2294	576.9111
CD53	222.4389	147.0679	716.023	103.56	261.5453	310.1559	513.1501	311.3501	693.9243
GPR160	61.84215	520.6849	607.6475	479.4173	222.6939	639.7073	249.1084	361.1543	743.1628
TXNDC15	3347.558	3223.214	1403.251	3996.484	1512.487	3010.432	1258.708	1477.397	4241.064
IQCK	101.8127	70.71557	58.2615	59.11097	42.74845	86.73162	45.92566	65.41829	43.20335
CPPED1	291.5518	162.0504	158.8176	423.3863	87.69167	239.0647	115.4886	138.1534	333.1216
FIS	92.30898	50.5864	51.16009	115.5894	303.0063	56.81334	409.6484	676.4486	132.0229
TSLP	88.67017	80.57269	49.00617	49.5737	53.2008	44.92387	61.30507	71.25749	209.9788
KLHL34	33.51944	36.9747	35.74913	49.87391	43.22088	38.60451	53.24529	248.3521	43.95508
BRWD2	140.4387	378.5854	771.0325	1319.002	85.80018	75.22326	483.0762	330.8259	110.6721
ERCC4	76.55388	56.83551	34.31619	57.02046	47.58889	40.96807	40.29355	226.978	35.72125
ANKRD13D	89.13026	139.9479	79.1227	136.5815	229.2253	65.83441	70.42377	69.4316	209.2748
SFXN3	2529.098	1940.34	302.6097	2570.896	306.6074	629.4745	452.8182	366.6286	2764.262
CTAGE5	75.66016	44.29097	47.68463	102.0343	61.00232	61.37808	49.98171	77.33915	73.31762
LMO1	43.1644	55.07474	309.2488	35.38301	35.05051	614.6631	122.3345	486.438	39.96978
LCP1	2985.559	1765.85	6741.231	813.0942	3266.283	4692.401	5191.7	5881.985	1320.823
UBE2I	182.7701	94.48815	69.5563	124.8442	81.90032	85.43587	57.00015	108.3977	396.8085

C1QTNF7	150.8328	61.95997	43.24207	64.0656	157.2008	218.6793	49.78071	59.18637	109.2842
PON1	150.7666	77.2121	59.93912	155.187	61.02333	61.86852	58.57298	173.6977	48.68142
FAM132A	64.04458	56.32878	51.01446	44.62619	34.2062	55.37465	41.87074	42.97938	47.63683
ZNF687	86.38583	88.66231	57.93552	50.83888	170.8699	84.63453	66.73048	70.61488	169.7374
CCT7	47.86427	1436.34	1936.98	2741.068	765.5527	893.3223	769.9844	1169.757	74.68386
GRHL3	49.49439	982.0635	1317.749	51.73387	1079.013	850.5659	467.6444	1123.826	1632.39
COLEC10	30.47235	60.0535	35.32217	35.41071	41.11216	37.56332	55.42119	112.9577	32.43932
PSD2	171.5857	173.1862	81.09155	179.0222	85.05715	96.18289	71.17359	109.0232	307.741
PRMT3	37.80453	43.80763	188.8575	239.3707	320.6194	59.00018	252.3816	140.6264	202.0715
CD82	318.1291	162.3676	101.2512	229.3451	95.73968	137.021	81.51496	91.51659	440.6919
STOML2	7278.827	8920.289	13478	11678.92	13045.53	14453.01	14064.41	15177.45	6235.822
HIF3A	55.94571	44.31708	59.38266	71.29951	84.00587	55.63863	118.5105	360.4794	136.5963
SNTN	33.42334	48.55272	47.93814	42.58516	40.29869	36.16912	35.9353	59.60307	45.45021
FNDC4	848.8682	76.9901	245.9009	69.43804	126.6754	74.46766	121.2936	140.7324	183.7125
TMEM129	238.3039	93.478	56.90237	147.353	57.72886	56.88021	56.71092	53.40763	156.4147
SREBF1	256.5633	486.8183	2719.891	605.129	2735.351	1571.385	1831.071	2320.286	303.5196
LRRC4C	66.49651	43.11321	51.10276	58.38963	48.30957	55.82533	42.42367	45.99257	45.29644
SDC4	34.23602	33.41406	34.15333	48.60777	37.80952	380.6678	113.3355	34.47062	2085.665
FLJ41821	136.366	104.6745	69.86863	61.21271	61.81845	58.29992	53.82117	72.33832	278.4468
CCDC67	57.81241	75.01575	54.10302	63.82697	49.17075	39.90377	47.7303	57.07793	158.2155
MGC33556	37.23518	37.01133	207.6627	41.94286	51.63534	248.9791	63.4124	58.04116	40.26651
LFNG	5505.791	4948.768	11306.21	10817.77	10270.38	11177	14917.02	13707.78	70.26313
LOC440925	66.57687	74.06944	63.4211	58.09354	45.74041	118.3865	260.0983	65.95351	51.03108
SON	46.05937	41.41938	43.25735	41.609	95.28458	44.65103	68.28027	115.9305	68.07489
CITED2	1041.552	34.69032	149.7226	1214.947	738.7357	946.1985	583.4365	108.9084	37.72166
UBE2B	50.4828	263.6927	363.6051	433.5157	287.4225	394.0466	219.3036	52.77565	43.48555
SPTB	175.3817	83.29944	55.53168	65.36104	67.25069	58.39057	84.66944	56.06445	256.9443
TTLL12	98.14715	390.0015	1596.156	430.043	2851.051	709.5542	1991.2	1731.071	53.35637
RASGEF1C	204.0594	106.164	68.94997	165.0468	58.74503	88.94828	60.00388	60.98995	90.43983
DSC2	4744.357	3831.2	5453.441	4402.753	7415.931	4414.856	3874.034	6045.488	4155.157
LYST	57.51278	67.66425	53.38334	110.4285	47.95592	54.46965	40.8172	45.02841	63.56617
ADCK2	2305.209	1361.151	3229.996	338.9887	5715.699	668.113	2694.358	3280.881	2196.431
CLEC12A	941.5557	570.5615	152.6267	269.5236	460.7733	230.18	268.3542	361.869	48.54099
NOMO3	32.1839	596.1887	35.98434	908.4766	61.81938	222.3004	58.5922	233.8554	760.4023
ZNF238	39.87794	52.57766	36.79171	34.94651	39.23831	37.29219	37.73026	39.20624	193.83
DDX31	36.97341	45.46291	43.93631	48.89054	38.50991	46.66195	35.89735	38.12987	45.44033
ZNF174	348.6303	219.0504	125.1255	231.5179	103.0876	159.8654	82.01592	126.991	400.9909
ZNF337	37.56798	1483.229	2663.857	34.02827	2883.971	2423.656	2893.19	2674.799	36.13542
MATN3	162.3458	124.9232	68.75809	401.3308	67.24217	188.7039	80.5771	64.80703	199.9148
LOC442582	356.4898	677.6401	258.8218	750.6221	192.7083	261.8258	216.6384	271.5869	765.9978
PROCA1	259.1067	383.308	154.1211	114.4827	118.7781	216.189	96.75527	413.4406	260.2179
PLEKHA8	149.7847	90.6919	56.23438	102.8606	75.25359	106.9809	55.73734	81.63078	292.0649
MCM7	250.7505	1463.438	4790.763	4219.56	6086.285	3635.532	5363.876	5457.726	247.7383
ITIH5	43.28529	574.1867	430.3562	36.13789	569.0234	997.0376	151.7176	278.8672	1063.431
MRPL22	59.54402	67.73242	136.9912	369.5737	127.5385	213.1925	107.9377	113.4324	108.682
KATNAL1	473.0845	315.5298	97.80172	149.4423	108.8805	95.188	78.3402	128.6679	235.4148
EPHA10	381.6634	202.8848	77.7637	260.9401	124.513	143.7697	92.62206	125.7396	392.7076

ZNF702P	34.46002	64.17532	44.84274	47.08161	35.60805	37.24916	36.63128	45.0153	49.89939
SOX6	814.9791	402.1093	442.7373	47.85912	170.1789	247.4762	112.5831	108.0624	71.53178
OTOF	2828.648	1075.558	3268.769	2381.786	347.9679	2970.26	1958.792	4697.085	3758.49
SLC2A10	604.6729	292.1454	234.0907	67.3451	167.8479	193.5293	133.1944	238.8154	810.904
RPH3A	307.0633	159.6901	84.7344	165.873	89.88885	151.0336	78.60243	91.55401	346.996
CCDC62	41.16275	43.94497	37.53027	67.06948	46.80365	37.73137	109.007	246.2631	40.74798
FBXL15	47.23972	50.65405	611.4517	43.16195	637.3294	47.40828	874.9887	1339.456	61.13276
TRAF3IP2	42.12418	47.26231	33.76324	39.44252	48.198	43.57877	33.82303	61.83821	32.95042
GEMIN5	267.5362	198.2461	221.1936	248.0403	74.76655	80.15131	148.5545	65.40922	477.2038
CDC20	7593.027	11945.26	11620.03	16223.35	11089.34	11312.95	14808.72	13785.69	10810.5
CAMK2B	67.99712	54.22608	67.3462	91.89405	59.97145	69.65527	64.00266	357.3279	145.7887
TIMP4	185.6876	187.8186	94.1491	158.1622	87.99194	116.819	155.3056	80.23354	296.1652
LARP6	120.8024	378.4194	420.4601	773.9898	773.015	1167.067	1775.554	522.0355	221.6146
SURF1	2202.513	69.01009	513.0134	76.95296	1250.826	907.7708	1415.942	1379.89	1643.661
C22orf33	303.2494	196.8701	103.8143	194.611	99.65783	140.6685	88.86272	113.5878	460.5388
RGL4	420.1332	261.7039	146.6478	291.2167	195.2782	385.2641	209.0816	412.0528	487.6548
MED12L	45.63235	35.80826	38.1797	39.78936	34.34992	43.52132	40.11301	39.46537	40.04416
NECAB2	85.46003	713.5614	84.45089	167.4559	166.9764	61.07595	128.5861	222.5597	150.0554
LIN7B	68.77209	88.83942	41.83739	614.733	273.7856	318.5249	144.9202	533.3069	3793.73
SORBS1	51.35268	52.33686	35.56452	1021.294	218.9726	315.1197	287.5431	523.7095	49.60905
HNRNPA1	39.10018	39.75434	51.28053	49.91623	34.8941	417.3475	78.31141	32.71481	55.64379
HIST1H2AI	37.95261	62.37361	394.0706	419.4427	278.5432	619.0121	277.3092	351.1389	1365.836
PKP1	85.38104	91.13188	480.5023	63.23594	820.2603	101.8847	2696.885	771.7117	189.0026
C15orf63	118.5699	69.47414	80.75851	54.86174	141.8336	150.5461	59.38393	68.17832	166.2663
TLK2	494.3124	127.3126	114.336	121.2126	111.8204	167.8346	97.73778	127.3149	161.7605
UBR2	2312.038	1625.133	1487.663	2389.065	1352.753	1087.368	1021.15	1216.59	1256.733
ZNF541	36.70248	49.65002	75.32924	52.34668	34.08409	153.5905	66.12896	63.33041	262.5987
MXI1	133.8666	50.89645	68.20599	76.14828	67.60696	78.08237	54.78902	67.35557	68.63554
COX5B	3961.179	2947.155	1793.335	3616.943	1161.294	2452.249	1299.263	1624.063	3932.333
SLC24A3	150.6049	348.134	686.7531	421.0285	740.7041	787.4696	997.59	749.1481	101.4835
RIC8B	1476.292	687.2888	2347.764	615.1904	1644.294	2305.235	2078.5	2143.225	103.2321
CLYBL	8114.684	4183.395	6192.124	2808.246	3653.081	4856.277	3982.764	7834.745	5925.932
B4GALT7	396.4176	1243.023	1300.863	618.2189	1382.421	961.5496	1394.95	2528.437	511.8773
TCF2	44.38881	41.3419	39.49716	59.67622	36.82098	49.36642	33.28615	42.40368	82.88135
FTCD	1559.836	885.3552	290.7417	1136.082	297.6789	688.6521	157.8464	167.6496	2458.722
S100PBP	219.7761	299.8877	96.68344	268.9435	110.0316	159.9391	96.43981	112.9324	192.3346
DPP6	174.8424	168.9734	79.90649	107.6056	68.39369	132.9374	65.66118	84.13445	125.4442
BBS5	138.988	168.6884	50.94582	174.0204	52.37722	60.52171	54.86589	67.28487	100.3005
RNF123	2966.968	3733.866	3792.756	2581.035	3085.371	3425.578	3678.793	2981.762	700.675
DNAJC7	404.4627	257.4678	176.6087	309.9096	117.3755	183.5229	107.2678	164.9007	530.7007
STAT3	2341.674	1201.179	1437.804	2029.816	935.2139	885.0808	711.2682	487.3849	55.35175
ELMO1	242.2586	71.93061	447.6507	86.91338	349.6212	301.8816	268.9384	144.558	158.8221
MAOA	6993.128	8353.023	10114.62	14962.86	11212.25	14858.36	12023.92	11916.72	7123.816
DMXL1	39.24166	67.35624	47.3468	36.50978	55.15532	85.863	49.75221	42.47133	193.0344
MTUS1	1506.645	1019.316	60.07457	68.41251	313.1274	426.247	280.8122	275.1112	65.84813
MRPS10	2007.538	2157.953	3817.069	1605.854	4429.25	3936.125	4971.779	4904.622	50.98131
C9orf171	35.79512	85.49086	67.47	91.71556	63.99387	36.48618	55.28241	61.85846	119.1057

FLAD1	5515.579	6405.334	5037.583	3924.034	4301.69	4088.771	5815.223	6114.384	3949.134
SNORD54	34.33709	45.09547	76.45119	38.51798	33.56209	33.29665	55.80606	43.80906	52.07733
HEPH	42.74366	56.94178	120.7238	36.71148	31.99041	34.02006	51.89283	31.57947	36.18488
SLCO3A1	379.7077	152.074	77.99037	125.6613	76.71662	78.21558	66.04697	66.07934	355.1042
PCDH21	202.2021	475.1149	382.3546	487.804	548.0295	608.6988	411.312	287.3644	419.0951
LHFPL3	40.12023	34.99915	37.01195	37.29684	37.76214	40.57307	42.26832	36.36641	37.79234
DNAJB5	53.07667	48.09399	35.55317	62.41681	47.79584	37.94386	42.75476	46.90349	84.20793
SLC13A4	1075.18	628.2414	184.1861	43.52954	407.7932	304.9348	228.9001	486.9514	1545.381
ZNF442	164.79	78.50259	100.11	330.0151	110.2428	286.7527	129.9962	94.35336	83.8349
SIX2	131.7134	96.15934	68.71826	75.85082	108.9978	43.12722	76.83633	38.71264	164.6281
WISP3	217.8853	116.5733	74.2839	97.12193	81.24815	94.51414	65.4562	76.70559	176.3404
EPB41	547.3426	303.5725	287.2665	377.8027	333.5099	287.4548	252.6176	276.1716	821.0016
MIST	96.68919	94.33874	47.51581	79.4373	49.4662	551.528	150.8834	166.6174	73.14653
UNQ1940	87.41729	97.31857	48.6697	67.89974	45.1053	62.93628	41.46956	50.49328	42.40715
CCDC148	51.4642	38.07605	38.71785	37.73662	44.99015	32.45794	39.32724	33.07867	39.65258
SMYD5	53.95612	47.79277	182.3792	51.97749	37.45038	310.1889	168.4825	264.7245	48.45991
ZNF286A	42.04608	44.21377	36.02327	36.82602	52.89902	60.45597	60.4143	39.91216	127.6993
TGFBI	7897.544	6060.813	6753.811	13131.48	7341.964	7982.199	14131.37	10046.92	7292.755
MMP13	2441.449	2788.613	81.31007	1271.75	41.85883	1406.893	3704.975	545.2089	808.1075
C21orf128	387.1015	241.621	118.7346	259.7361	109.8599	180.4673	98.52309	133.5817	539.556
ALS2CR16	36.42382	323.4104	144.5148	70.15767	329.4728	584.8273	246.0217	147.8137	865.4429
ARHGAP9	1259.396	1592.635	2438.026	719.1696	1200.425	1856.985	1522.285	2282.802	1277.759
TFF2	34.13106	35.41254	46.34705	40.15425	36.5299	37.13709	43.53382	54.49692	57.41347
TH1L	35.14507	1066.127	690.3186	949.5574	1556.146	691.3016	1097.081	1137.93	852.364
LOC441233	47.73177	60.32431	37.23225	49.64129	42.8721	57.29542	35.04252	37.79536	37.73087
CNNM2	65.10797	717.975	317.7819	933.3157	234.8429	672.9902	341.3498	212.1344	56.28371
PDLIM4	33.46592	44.04798	38.32502	35.77151	438.0822	36.82404	34.38301	40.95518	46.9774
MAGI3	40.7405	53.86399	161.6938	41.80132	52.65594	66.51004	73.40072	39.13562	33.3859
WNT8A	303.9264	141.0389	74.43142	72.72448	58.65878	94.26405	69.8048	76.2532	250.2665
ARSF	43.45572	52.92623	153.6357	45.46395	35.44349	46.66404	42.54755	43.13087	51.70552
AGL	44.50638	381.8631	124.0783	351.6942	53.0754	232.7953	89.13098	89.40052	50.32303
RBM46	62.08549	44.19316	49.13838	50.58816	43.30711	81.59329	44.0119	58.11627	47.23182
ELF1	4153.83	4158.837	4522.963	4779.157	2171.163	3525.086	2020.668	1661.845	3457.369
CANT1	124.2961	673.9584	942.8413	107.5905	708.7875	680.4272	989.6108	1443.335	101.3651
STOX1	49.86	57.39265	50.72854	45.28681	43.84177	49.74471	37.84475	42.86498	57.5121
ARHGEF18	6070.686	6452.117	10189.21	8380.651	11670.84	9735.213	12389.88	10230.83	6376.996
APOB	154.5986	55.90614	52.83025	99.57103	36.23233	41.83695	37.08879	52.36295	65.57808
PXMP4	110.3445	61.58495	144.715	69.8004	63.34649	183.6454	91.03516	67.8446	183.7683
CENPB	7028.742	7911.211	7176.794	8713.796	6391.592	7436.346	7918.564	7159.549	8283.655
RAB1B	6402.601	4306.658	1534.536	5109.598	1039.441	2061.259	1307.727	62.30164	4964.715
CACNA1E	42.81516	36.49391	338.6675	54.86646	42.82224	42.7108	231.9874	49.66194	60.72963
SEC22C	38.8251	39.72053	32.72186	75.59795	31.52426	36.34595	56.41435	31.47037	54.64072
FAM129C	227.108	159.3371	74.37965	172.0663	60.86753	83.73424	60.77626	85.51525	165.0951
TNNT2	13028.42	11341.31	8617.561	13185.48	3905.439	10666.65	7316.937	7481.366	14020.57
LOC286076	1631.364	849.5362	1258.1	1750.961	611.6219	2454.964	449.3291	1031.256	1385.19
P2RY13	3278.555	3403.669	4555.372	77.43398	2820.308	4263.938	2836.274	3144.365	3293.593
FASTK	143.0544	87.35934	657.909	56.96115	1204.568	346.4928	773.5568	1537.351	47.00768

GLIS3	49.00468	51.47769	33.29443	42.88152	35.8171	35.44126	42.78714	38.67533	48.6074
RFFL	70.98026	81.16692	244.5876	44.77779	57.42084	57.80725	131.9097	53.94894	57.27096
PTPN6	183.143	126.1512	64.97222	135.2566	54.87027	92.81738	68.66001	74.35216	194.4391
SLC30A5	86.52254	57.65806	810.4297	134.5082	614.561	491.0397	338.095	284.58	74.98618
IZUMO1	62.25437	84.8896	50.81979	98.75263	107.8029	77.28297	52.79444	66.95653	118.6108
RPF2	51.11171	521.8258	491.8203	67.68438	657.4211	263.6148	672.5087	355.9815	1117.163
SLC25A11	3570.955	4670.084	3533.828	3305.598	3363.842	3334.227	3252.073	4631.42	3018.792
CUGBP2	43.97972	37.70455	35.33414	31.84614	33.55854	46.8038	36.10363	40.65459	38.18835
FBLN5	1196.508	234.4769	204.489	537.5627	218.3782	165.8643	193.7541	134.7047	484.8103
ATXN7L2	972.7545	478.4339	861.7956	892.48	1147.223	807.14	818.6914	886.7242	130.695
MFN1	855.3093	1006.814	1782.879	1144.487	1668.803	1455.656	1472.472	1767.327	255.0594
NKIRAS1	65.50113	526.749	537.3414	319.1328	504.7799	467.6558	651.2993	462.7038	76.08791
TIMM23	553.2522	263.364	396.8617	900.2796	256.0578	175.7934	334.6907	439.845	1013.017
BCL2L14	166.25	121.497	75.44283	129.5353	58.18588	86.29882	55.37615	64.57224	183.0852
ADAM28	34.03461	32.44992	39.22168	35.7111	45.79948	39.43799	31.82157	36.11941	41.21923
C20orf12	279.6175	184.521	294.9793	1001.187	257.9169	128.2892	165.0622	112.1705	311.3397
USE1	74.04325	67.07291	547.4153	84.97444	44.81071	51.18941	283.1492	242.0907	128.9397
MGC40069	1138.712	115.0901	305.7286	157.1824	250.1699	216.1189	150.8268	355.0427	237.6536
EPHA1	1622.55	1440.804	3388.585	1766.961	5951.175	2272.386	2471.085	4296.258	305.709
WBSCR28	121.1378	113.3489	64.79139	69.44983	45.4208	64.65291	63.74763	56.87258	48.60681
TBX22	258.1562	150.0953	79.12344	149.7359	69.23453	118.0009	70.57475	92.88099	410.3312
C9orf75	232.8399	522.5941	844.319	406.5741	1032.381	372.6715	772.2106	1035.145	287.8201
LOC44057(	63.34821	348.496	56.77205	76.67846	57.36582	56.85083	52.38181	314.8518	132.12
ZNF415	207.268	132.8606	79.33028	76.37762	72.56264	249.5353	395.8664	341.0365	155.7714
LEPRE1	298.72	1850.576	1276.512	189.1732	1535.837	1394.821	2532.765	2431.589	474.7318
HTATIP2	567.2507	381.7494	282.9054	403.8683	252.4143	591.1859	253.1518	234.2363	618.2729
CCR9	375.333	216.0822	120.712	248.8231	97.40123	171.9132	95.23608	149.4222	543.1886
SLC14A2	39.09872	50.12655	133.0708	57.43836	44.72712	55.17056	55.1614	39.05564	99.87396
MBIP	52.62766	175.39	171.3714	645.1336	128.5563	190.7969	61.4888	160.0563	45.87045
CEACAM1	51.66539	65.78799	781.586	93.5828	352.7343	922.0729	405.1236	45.11967	2504.564
PNPLA4	34.38018	55.03674	52.63175	38.17579	51.60003	93.61128	58.63473	36.07151	40.34745
GBA3	37.06631	44.45898	38.56423	33.8552	157.4268	67.09817	94.70951	32.89694	33.89803
SGIP1	1692.387	791.0526	561.9219	64.88297	646.6543	506.6001	751.275	429.5441	36.78808
FLJ40288	55.60551	113.3288	50.15408	71.41459	69.0229	78.23995	57.05014	55.34945	99.0641
COQ6	68.51154	76.08277	56.86079	55.25853	42.93487	67.30946	118.2443	50.31702	87.95776
DHTKD1	236.6796	136.8991	75.45806	192.1389	83.20972	124.0338	74.58028	106.6887	327.0399
C3orf47	52.26267	38.12694	34.37699	724.7693	32.99833	34.56709	39.2789	38.74714	67.82227
MEIS2	670.788	619.8733	414.207	85.67255	544.45	423.7327	311.8098	292.9828	754.3888
ZBED5	477.187	181.7184	117.4796	237.2562	113.8145	142.4694	91.87859	110.9334	487.1183
TCF3	111.3702	75.27821	139.2335	57.39607	41.67828	367.7515	153.4643	103.9481	54.02998
TAF1	176.5955	91.66177	56.24426	70.21516	54.80379	79.17528	59.0634	66.22565	75.63825
TP53BP1	59.89091	63.3687	45.01547	44.84893	43.72138	39.35994	46.84598	43.38779	37.22673
PEX12	50.89853	47.25172	61.71729	254.1292	44.12429	44.59543	52.45465	38.23471	43.30831
ALG5	1760.22	2238.293	1547.264	2444.897	1925.676	2385.044	2204.521	1477.694	1128.304
ONECUT2	34.12682	35.78103	41.93871	45.54797	36.10397	32.95658	49.70449	39.62546	31.48697
PTGDS	112.1081	61.02169	475.2799	59.44563	293.0893	556.2577	444.1624	294.235	43.54749
KLHL9	107.3559	145.2645	78.13384	315.7287	204.178	97.51447	107.779	74.5784	94.95638

PLEKHG7	509.4734	74.66522	107.1096	54.8009	80.87574	68.78545	70.60136	54.71513	141.3292
CRMP1	126.5395	52.23491	42.83165	91.06676	43.50692	52.29525	49.59085	74.46843	41.03464
NDC80	246.4489	127.6815	148.3602	413.9116	114.849	120.264	96.50911	207.8505	239.5795
CENPF	2457.629	529.755	1270.362	2900.905	1163.534	2183.441	1666.549	1297.511	119.3319
USP11	839.6636	301.9126	732.609	561.9088	1058.271	1304.648	1323.966	573.4272	57.26648
IFIT3	49.36622	1637.376	101.4877	505.0757	114.9462	604.5744	286.9753	195.998	42.21545
FLJ33360	44.9203	35.32819	36.35671	34.26781	56.15572	39.18132	40.99306	167.6837	59.20974
PROZ	55.86631	35.88526	35.96264	59.01688	40.69003	42.14161	44.10412	42.547	40.16967
DLD	117.8414	49.04455	131.2724	292.1608	93.45143	183.3026	101.574	151.842	67.90221
MICALL2	102.9873	97.40534	62.21852	68.97042	70.00467	76.47541	57.67297	50.11011	161.8502
C1orf95	1634.204	196.115	187.7185	166.3657	69.82895	104.2859	63.06166	234.3698	408.7303
NRIP2	499.7984	166.555	93.49552	209.5702	70.35308	133.0495	79.61127	84.4748	170.202
INPP5A	450.9036	257.3038	183.6343	278.4044	238.7422	212.0486	183.0565	315.6281	513.8785
FAU	3218.636	3994.352	4336.393	4314.388	5153.716	3323.568	5688.251	4416.949	1696.537
CHEK1	578.6562	85.00663	133.2708	200.524	158.1071	117.5602	117.3293	69.82737	226.5095
MED4	2030.011	1921.809	843.0961	743.5696	1850.348	1432.029	879.8825	1287.019	1629.88
ATG16L2	387.6459	264.641	315.0649	208.382	552.4807	230.8084	456.0952	190.7328	493.6072
NSUN6	2041.278	1342.473	1021.807	1445.373	592.4153	805.4536	567.9592	688.5917	2034.767
CADM2	114.3881	43.92406	51.61327	56.01609	106.3761	44.75999	56.40734	170.4909	92.50869
DNA2	569.1553	308.7193	209.0816	397.4523	239.0278	267.4108	204.3937	202.8908	528.5963
DAPP1	1296.565	460.4886	745.5785	488.0765	623.3675	1060.835	517.1838	279.8507	614.4586
UNC119	1938.752	1732.207	1354.153	2099.648	1128.86	1470.311	1231.016	888.5977	1494.846
FLJ14816	33.85156	39.37128	52.12772	52.9538	52.57037	49.14608	38.79512	34.78273	81.8392
LOC65360[illegible]	42.67125	37.63079	34.32541	32.15496	39.88382	35.59178	85.03319	42.31104	46.09021
CYP51A1	635.6423	497.3453	864.8143	1508.462	1796.83	1171.723	797.4373	764.2134	750.5779
C11orf54	236.7749	113.0365	82.38098	193.1906	90.86809	133.9376	64.17101	72.86791	143.1216
C10orf72	102.3852	85.05896	56.98698	69.02428	42.95887	85.85052	58.41531	55.2394	161.5865
SLC25A25	116.4893	55.32621	40.16445	57.42706	72.86259	43.48587	44.60852	57.45244	39.74694
TMBIM4	40.9864	45.13208	825.6829	47.95036	968.6847	685.1105	723.3325	737.0955	71.22203
KPNA4	34.92807	391.8371	835.3483	478.1568	1139.434	1064.414	1269.396	2014.968	37.01567
TMEM138	2940.063	1983.529	2799.587	2431.545	2010.346	3115.347	3754.235	3896.231	1047.651
SH2D4B	54.20354	45.91473	37.31744	33.51295	33.99639	34.88103	35.41332	32.66315	44.69554
OR5I1	45.40941	46.8263	40.0412	36.25339	34.09947	37.22939	36.99736	34.02431	79.80455
ATP2B4	65.51066	66.51846	59.26952	446.0305	416.4995	57.6287	120.5012	378.7993	65.26944
C11orf49	2214.094	37.88688	40.62436	38.77404	474.3236	648.2525	420.3763	372.2793	45.15598
EFR3A	5776.053	8443.279	12639.34	7666.806	10615.39	11502.76	13745.74	11880.85	30.79852
FBXO18	35.0111	35.37448	176.8632	34.46036	32.59078	45.71122	105.8971	243.1181	49.35122
GRHPR	1353.478	387.9037	1782.028	1619.457	1768.31	1960.868	1896.417	1857.977	2275.46
WSB1	698.5402	945.5052	863.1135	920.7939	840.1472	1154.629	919.7421	587.9644	738.7667
TAOK3	256.3734	132.335	90.21687	178.8838	106.622	140.9737	70.42515	107.7738	356.1028
FBXO33	64.58054	50.68601	477.1547	43.94882	1015.976	40.24239	746.2582	674.8594	49.19492
APOBEC3H	63.52936	71.28607	529.5176	46.43063	219.0088	167.2763	323.6457	556.4061	63.56031
MRPL50	38.24623	60.17303	376.6081	43.77364	713.5217	1224.32	821.961	636.3324	40.85872
PPP1R14C	34.96604	2116.835	865.9819	1997.548	1385.899	918.8593	1842.41	1402.781	64.73478
ST8SIA1	61.62453	65.52469	286.4727	62.57934	126.1764	41.84755	69.27544	46.20487	53.27801
ID1	51.06525	1070.81	482.1852	53.63901	1597.489	309.8731	579.7354	653.5128	42.80296
CTRL	39.55465	1432.514	737.3176	35.78386	1121.763	770.1048	813.1385	1319.17	1166.407

CACNA1G	47.28022	55.25846	46.40993	84.58943	48.8414	45.6674	49.28117	46.52801	60.04412
NLRP2	203.3986	99.41064	211.1404	157.4391	58.93482	86.18281	116.0789	895.3397	143.3796
RPSA	971.6017	926.5251	862.7098	1047.306	572.3751	759.8206	555.9055	332.1712	883.1734
CT45-2	40.36573	46.48967	51.96854	34.4599	37.46816	35.00739	35.98981	36.02645	39.85622
KCNK9	302.6203	208.3568	111.8088	233.2077	120.89	180.957	89.60482	136.821	516.4299
C19orf12	11632.19	12529.78	15052.91	11343.36	12661.13	11958.96	13528.53	14895.67	10562.74
PPP6C	9609.8	10252.07	12554.42	9830.42	12753.16	12381.67	12974.7	12773.86	9289.594
FLNB	123.8196	50.502	794.0655	2609.179	1771.181	535.8958	1739.083	1002.708	58.92324
ZNF599	76.16277	34.19937	45.03188	55.76407	47.6591	44.01426	46.71024	39.89381	60.4897
GTF2E2	57.10515	3782.046	9838.773	3772.606	13829.42	7883.806	11439.26	7170.915	5556.169
ZDHHC16	34.21529	41.42249	36.60998	33.4244	41.05941	31.96999	35.56053	41.0612	37.1172
SSU72	4710.024	4980.201	8495.301	6858.189	10977.77	5803.406	8860.068	6416.673	1517.009
KIAA0284	459.6448	527.1399	854.1066	49.37593	1917.477	1576.049	1002.302	1039.849	40.41093
TJP2	796.7687	955.5038	639.2341	1449.424	971.8591	354.9113	811.8894	1341.656	1180.384
MINPP1	259.5952	86.97325	67.28397	154.7245	73.52161	178.1502	72.82336	103.0163	157.0937
CASC3	1879.668	907.8012	273.044	53.4764	198.6348	281.2811	231.125	299.389	39.28765
LHX3	91.3255	38.78726	55.39037	48.09114	53.76439	67.66388	47.38639	37.15801	36.87785
NPR3	120.428	107.2149	64.94841	88.05429	153.8403	82.8832	63.40008	229.8235	152.1237
KRT14	29093.71	28895.71	32388.43	30519.37	33124.92	30539.99	31887.12	32337.13	30578.44
RBMS3	103.7639	133.5198	46.99268	89.77745	57.97475	421.9738	101.6363	227.4983	87.20242
SPG11	46.75269	1012.715	1097.583	39.11361	785.0587	1184.047	1068.333	741.9004	43.07751
TCHP	78.60185	1465.99	271.0485	96.67702	228.4076	483.9643	238.8932	385.3099	134.7227
SERINC3	561.6521	484.3828	483.2117	40.74533	286.9774	437.288	376.92	598.6193	39.20406
C4orf14	1893.849	1890.298	1291.558	1169.647	453.6759	149.5459	739.7347	261.8192	1702.067
SPTLC1	33.64371	45.86725	71.57107	147.2181	73.42948	96.97449	67.79671	55.21326	41.47046
NELF	3667.015	3118.684	1394.061	3490.093	1222.603	2241.4	1353.503	1135.885	4331.274
PTGS1	257.8315	188.0372	96.9573	931.0398	457.4041	204.1422	132.9057	76.45531	466.4449
ITGB1BP1	43.55124	31.914	186.0993	37.3882	747.8036	45.66377	218.4602	431.0689	35.31789
C6orf89	32.79465	37.89259	34.43486	38.15149	34.89131	32.73664	136.0593	37.97352	36.21785
NMNAT3	42.21196	42.28829	36.01889	42.76963	33.39341	35.60617	36.01179	38.66122	37.93756
CD36	41.68517	40.62317	40.47516	38.20815	40.80957	47.74634	43.42436	34.65772	40.64357
RBBP6	288.7163	241.2389	108.3887	155.6214	142.3389	165.8007	110.8707	88.07085	142.3232
HERC2P4	34.11715	51.62639	31.28021	34.94105	30.09383	33.92834	40.31649	37.58889	32.98203
FCRL2	35.07327	58.12919	37.88324	71.34023	51.06189	63.16462	55.52274	46.45343	36.95833
CTR9	88.43089	55.09626	55.97382	65.06554	47.93442	186.423	70.62327	104.6745	66.68361
RORA	1206.279	545.8857	314.5984	439.9279	197.6507	286.4192	152.846	159.1223	1298.821
OPN5	156.9104	106.4577	67.22104	84.84852	55.48665	74.71297	50.26011	68.33536	62.87484
NUDT6	66.6507	95.96091	84.05424	68.13225	51.43612	80.2669	112.9348	66.44381	135.118
SUMO4	60.19252	373.8316	136.1773	284.3046	43.94192	132.5531	153.6861	131.11	87.42076
ZNF260	2196.766	883.3636	2753.403	47.38815	4277.308	3906.491	4606.136	2230.728	547.2704
C10orf85	200.2181	121.4396	179.0618	138.9081	79.92207	261.4568	62.27051	247.7971	363.3412
LOC441426	38.14202	283.878	247.9541	51.38543	310.7668	150.1983	161.2488	176.1839	731.8348
EDC4	36.63291	843.8101	1510.795	852.3104	1974.38	2112.43	2098.875	3087.252	55.49273
C3orf34	35.12465	43.66743	38.37749	41.05097	40.37053	54.20819	94.49037	124.6887	63.81955
LTBP4	1801.766	4883.573	4650.712	3968.89	9167.015	4548.971	5990.233	5215.162	4061.321
CRTAP	1144.273	887.5021	544.402	1062.103	632.2953	281.2216	684.9294	299.6204	1712.448
FKBP14	5393.042	3592.434	3298.319	4881.531	2249.635	3010.663	4191.773	2482.937	4793.584

UMODL1	75.41534	126.5569	65.67464	72.59692	54.22723	60.61126	59.88982	61.76782	160.9121
RIMS2	312.0125	186.7226	159.0679	148.3881	127.1537	250.6805	267.6911	147.96	120.9013
FLJ25996	137.4885	77.99384	587.3121	138.3438	419.69	290.7552	268.0998	61.75974	71.1991
SPCS1	9259.676	11770.86	9042.731	9773.647	7493.993	6257.812	7732.389	6248.851	9025.085
C1orf25	76.69653	46.75544	339.5966	40.88346	629.4745	1407.111	743.0978	411.8324	33.35316
WDR38	71.94969	50.09199	55.86284	65.07106	60.31401	67.25677	48.15913	67.80739	363.0272
FOXN3	61.43632	48.53876	77.72188	54.6171	37.13824	38.61279	76.3871	65.94556	70.0143
STH	101.7952	85.8516	63.9971	63.37774	59.85791	66.75829	57.37708	76.95324	162.3182
NRTN	47.69016	39.27301	41.26799	36.46537	245.4877	37.37301	37.12777	50.24766	49.86074
AMT	2935.844	3187.625	4134.523	3178.953	4843.026	3352.043	4372.887	4356.837	4881.527
DIABLO	92.10936	131.5271	193.3962	139.6303	184.6055	78.97575	60.29052	70.99805	356.2567
EPS15	63.10368	544.2084	815.5831	433.583	786.0634	794.3619	652.8457	411.9191	52.31603
FXYD3	38.79111	38.55391	235.5845	1105.698	213.7024	37.11663	39.65745	277.7985	41.92502
COL9A1	172.8764	103.1167	49.08841	151.3616	64.18629	82.24239	49.47995	72.11767	330.2954
SH3BP5L	74.4554	60.04839	36.35489	46.96293	233.2035	61.21053	205.6202	250.5196	58.23634
ZNF445	31.40636	36.89457	39.01952	37.74466	32.63235	39.64649	32.8588	37.73026	31.84961
ZFP91	7999.23	7619.241	9525.155	8843.037	9257.588	9658.244	9902.926	8837.375	8088.336
RPRM	1171.282	86.53876	681.7117	106.3735	735.4781	1505.586	554.4425	1567.428	1702.738
GSDMD	15240.77	18581.73	16645.52	15692.99	10267.83	16954.08	17220.65	14025.92	16592.13
GPER	74.98467	121.3819	53.13904	135.6271	63.25389	67.23985	55.55651	83.84428	173.5603
CYP2E1	896.7825	451.8322	1249.961	36.63974	838.2419	1363.976	1480.203	176.3321	1315.772
NOL6	86.96818	65.34459	68.44955	54.99447	51.78095	67.27657	125.4345	50.06234	171.0659
ROBO4	76.33688	133.1914	49.36496	74.60255	58.98686	50.50447	130.0101	64.559	70.55719
DCUN1D3	748.4456	1415.249	2682.149	1636.418	4806.569	2673.697	2907.119	3324.589	2318.491
CLEC4C	70.81719	40.24539	39.08817	37.31748	34.31573	34.89241	35.69794	41.94605	65.97162
PDHA2	90.85725	101.0429	64.76343	60.32661	52.49804	64.34464	53.6014	53.35103	69.95938
C2orf54	48.61371	51.18427	39.82248	46.65496	207.4898	66.44724	50.34526	80.59848	66.0539
KIAA0020	377.2637	211.4958	1041.311	220.7723	528.5245	391.8158	587.7499	522.0173	391.0996
SORCS1	267.2399	176.6932	88.47652	189.0922	230.6741	139.6302	73.47322	97.60099	376.3782
C10orf12	67.86058	89.96955	813.3254	84.99697	424.228	83.91602	446.8879	393.1897	58.81101
ERRFI1	118.014	72.21183	513.8923	74.87733	803.7001	57.23172	731.5342	646.6234	109.3186
MGC4677	362.7851	239.9873	163.374	421.2577	76.25272	165.9886	160.1507	130.7609	480.8829
STAMBP	35.29052	38.08451	51.16772	51.23663	42.02002	42.74948	48.52208	67.62606	85.20041
TCEA2	3579.139	4742.436	3218.091	4050.735	3270.306	5391.935	3452.179	2541.01	3594.245
SKP2	2143.482	3184.631	5713.677	4747.331	5201.939	5722.065	8707.027	5940.383	1438.65
CADPS	168.7149	72.00099	55.36292	108.9078	61.54423	71.40339	57.2094	63.10638	95.40001
BRF2	143.0196	89.70605	627.7845	158.4289	587.2545	105.1442	337.4632	101.9588	256.4435
USP15	676.4053	466.1312	432.5641	324.1802	387.3346	575.8512	410.7868	404.2033	288.5625
PLIN	46.20809	40.06507	35.94827	41.29745	57.78971	42.28097	35.70955	34.31487	43.39822
WDR5B	44.58332	51.6007	184.7177	320.7915	426.6428	41.57114	394.5385	35.04131	47.43958
POU5F1	136.2657	355.0345	69.97112	135.9886	65.52025	88.27766	80.47191	160.9463	370.3656
CD180	1400.11	122.6771	370.7315	59.15907	149.0084	61.34742	57.72277	81.01855	103.3409
CTDSPL2	120.1452	52.00134	53.43797	106.8907	92.32882	67.92834	65.32359	107.3066	284.7459
LCA5L	879.8091	74.28574	312.0838	61.24316	145.4286	298.6806	83.84689	144.8954	34.85764
C11orf17	223.3795	168.3962	133.6247	226.7688	131.4339	173.3176	104.515	132.3551	252.0016
EPO	39.28331	39.11662	33.21041	48.7182	34.84658	47.0882	72.98881	251.067	38.38247
PEX10	48.25968	64.04765	37.14712	59.8656	35.73174	37.98579	38.27521	46.12816	61.19786

IL15RA	39.64497	79.10966	36.21444	60.33232	45.51279	48.47755	36.84247	63.65257	49.10052
FAM38B	276.499	177.0515	142.821	177.6851	97.70793	257.3791	94.98705	158.3727	473.6766
ACTN3	91.65665	73.34782	54.66892	84.03684	172.6837	79.31051	168.3574	163.6861	169.3082
EFNA4	212.1417	120.4338	69.29466	97.32099	67.29503	436.9257	124.8284	58.46655	300.0466
C4orf22	49.2286	50.02235	51.40655	46.62477	48.00972	35.13316	44.67384	44.60118	45.78249
EIF2S3	1061.117	657.6289	522.2443	601.7384	675.5307	732.6976	641.5429	489.1872	796.5543
ABCB10	718.2004	330.3365	772.3915	1332.592	743.6527	781.5638	499.238	523.5761	59.13733
UFD1L	175.3777	365.4512	424.547	320.2868	479.1596	454.6898	591.084	566.5674	869.4069
FLI1	167.8033	468.7229	667.2576	515.1206	456.0945	528.731	530.3864	507.5659	650.5659
LOC14995(	59.04925	33.2231	41.51396	32.55893	40.81274	35.79978	38.15838	38.43902	53.51589
DCC	44.11775	72.49017	48.46646	69.29594	55.78266	64.53981	45.26483	52.7244	79.90761
BCL11B	73.83021	659.7215	176.1255	42.55114	251.8497	348.5513	260.7367	67.24443	83.60725
MOSC2	170.4665	380.5111	156.1784	64.55775	197.9301	169.5554	242.1974	99.90624	60.19801
ITGA2	2461.013	1860.078	885.4639	3733.666	874.9373	1632.727	684.2122	886.1072	1382.355
CSE1L	1640.256	1495.105	2632.885	3657.424	3040.706	853.8406	3011.627	2928.869	2424.479
OR13C2	94.57993	58.47403	59.86052	52.57596	65.08015	75.66335	43.67046	58.64805	129.2782
C6orf218	80.96468	95.72473	58.09359	92.23238	57.72073	57.25072	58.90964	52.31603	149.4967
CLPTM1	259.4658	1273.101	582.4403	1114.715	2219.821	108.5829	1542.754	2254.013	304.9695
SLC25A24	226.7318	105.839	163.1982	628.9958	74.50638	126.3424	60.99484	77.79099	319.3358
CFHR4	38.44807	39.11066	41.82905	35.58828	31.51259	36.88445	33.25681	43.44442	34.87985
CA10	45.89363	50.21546	45.02974	49.39395	52.99086	57.65889	40.95192	54.04529	53.61742
RAB27A	1445.81	413.8173	320.4646	47.14767	107.8939	227.2528	150.8172	147.3869	36.15665
UBE2V1	112.6558	129.9993	74.91404	127.725	80.71837	97.51604	61.18562	61.77294	75.76256
IGFBP4	9749.637	11307.51	13847.39	8739.367	20804.49	11556.21	19409.9	18860.9	8403.585
ANGEL1	301.9932	187.4994	377.3811	107.9559	448.8682	91.50441	409.9107	450.4127	484.0401
DST	1059.653	421.1569	380.031	419.8724	207.5621	207.8709	298.2433	154.7899	38.55579
RNF149	5562.294	4983.117	3344.451	5260.661	2830.506	4226.814	3592.098	2879.961	5796.619
REP15	47.51241	34.55052	170.5452	39.34623	224.6669	235.8213	218.5691	140.2797	48.47856
WDR61	36.18466	39.16467	61.40439	35.04579	146.5465	641.8134	106.0286	103.3432	36.51542
FOLR4	112.1006	52.18595	44.71295	67.64565	45.52662	40.2928	45.90343	40.32979	36.22458
KIF25	52.19221	48.08081	42.80541	42.75938	36.32941	51.34753	44.85606	57.00883	68.93121
LASP1	8408.71	7015.404	8185.147	9399.807	7712.854	7237.953	7825.817	8999.865	6357.477
HYAL2	458.3572	128.2279	33.35767	212.2028	60.46843	157.7779	46.64564	83.03947	589.8599
THEM5	160.8878	88.99737	94.34822	123.0243	68.72176	123.8036	81.27324	109.0794	235.5892
MAP4K1	892.7474	2417.647	3573.565	446.4864	1867.85	2409.948	1733.199	2114.334	1136.014
ACBD3	904.2597	600.532	1758.316	1113.461	1835.031	1466.771	1709.163	2053.141	757.2717
GAB2	130.4758	48.09796	128.8191	109.893	135.1129	46.28591	143.5785	146.9341	101.7631
AKTIP	36.4954	296.2354	1020.893	572.9206	1630.428	779.8358	847.2644	1053.665	57.22299
ERCC8	36.69977	313.1811	113.812	63.12114	196.5398	45.85653	131.2089	148.9513	52.2571
SILV	98.39925	105.6112	1647.016	79.3602	184.0857	83.52561	876.0743	499.216	102.5106
BCL7A	235.6177	142.7209	69.429	111.9288	63.28863	105.3499	70.90004	80.34969	218.7954
HS3ST4	256.6329	130.1945	71.36327	158.1944	76.17286	92.22826	65.86534	64.46031	249.1172
WASL	1350.836	61.49065	262.8307	68.95338	1283.908	804.7356	398.8136	822.4422	112.0499
TRAPPC6B	66.87236	53.13912	92.63388	233.2651	101.7034	145.225	121.7765	118.9063	64.09161
TNFRSF10D	55.75219	46.47274	46.3972	325.2103	56.24922	47.49123	75.36578	52.3532	35.83725
NUP214	203.1414	91.18419	359.965	49.0656	48.54944	61.72159	47.93491	45.04988	63.58094
TMUB1	126.6361	3322.991	4381.125	1309.186	5167.868	3394.028	4394.267	5918.445	42.38692

REEP2	570.9634	378.006	125.3672	74.18294	148.7045	238.7266	133.0998	307.5677	58.52348
JMJD6	71.61883	46.87471	52.1365	56.45068	53.76939	61.03703	47.91024	45.77756	124.2832
CD8B	33.97357	41.3132	32.42443	39.51907	44.24292	32.71795	32.62329	36.94148	57.23903
ARHGEF4	42.14727	32.79465	936.4587	36.54401	1602.891	629.533	994.4266	607.6888	42.34842
HEATR3	52.75212	49.06416	39.46446	45.33638	40.38546	34.96482	37.20984	45.77503	135.6234
OPALIN	45.15261	38.53798	39.32541	67.36342	41.43715	67.71347	33.5371	49.72112	37.24839
FMR1	60.75294	38.22309	38.05334	37.42597	35.14505	49.06851	36.82696	44.67901	35.92459
MB	43.68002	2503.925	409.5943	42.88663	2045.147	1792.386	3652.053	3948.036	46.6456
YTHDC1	42.95515	308.3967	294.7789	32.69092	187.33	244.3914	191.6432	164.3634	48.96359
FKBP9	40.53265	162.3295	93.25393	250.1092	69.89735	129.4159	74.42568	54.3326	91.10226
OR8A1	104.8209	114.092	63.49783	63.68409	51.84356	58.32983	45.15886	48.46056	31.12901
MORF4L2	889.0344	745.2043	742.2108	562.4337	884.1537	910.8166	767.6136	633.4681	1304.725
NBR2	48.58191	51.46257	44.77639	68.3884	42.07043	39.17547	47.18434	40.54847	50.05741
CECR1	126.7243	101.3088	61.82122	106.548	64.33675	88.30339	45.24491	58.41503	135.4443
IL22RA1	253.2068	89.8605	74.03348	154.7156	86.51118	131.8901	75.04666	90.28334	360.2746
TP53I13	2779.66	3154.206	2157.365	3073.121	1499.224	1866.371	2066.848	1996.801	2784.954
HIST2H3C	34.13963	35.63091	33.04304	753.2918	37.77272	37.39614	36.15854	35.18001	34.81114
KLK13	4330.135	1310.029	6833.929	387.3251	7040.069	3503.441	2616.517	3034.502	4040.356
DCTN5	3336.241	2858.222	1210.303	2056.942	567.1091	1198.928	1001.78	323.7111	5069.859
VDR	90.79265	59.45682	233.7325	57.05583	53.50861	68.1192	53.91608	249.3345	61.13476
GSDMB	4005.211	10491.51	11045.96	4400.786	9972.953	9085.202	10120.3	12009.54	5165.929
C14orf106	8711.483	7863.714	6327.974	9717.255	4428.876	6942.146	4904.8	4293.708	7833.851
ANKRD19	69.60738	198.09	36.07254	50.15648	37.51731	36.38006	57.58901	60.96119	44.14486
PHACTR3	36.33043	41.45598	34.9422	42.20005	165.2705	457.93	190.6685	37.3546	110.5503
IL17RC	137.4455	80.35365	65.63748	73.91779	61.7693	118.0419	47.63616	50.16065	53.44516
HMGA2	160.4205	99.11388	162.7608	359.7818	107.4613	89.92355	107.3537	55.75486	211.5631
AMFR	49.09935	61.47539	44.77658	40.06815	210.2977	166.1186	320.1195	132.6073	69.98486
CTSZ	1390.765	36.4227	2694.493	1328.546	3530.395	2504.757	3871.55	2528.16	61.08009
COX11	67.98245	41.42685	128.0732	36.03353	119.8912	40.23756	37.01007	94.43822	35.17947
CDKN3	521.6315	334.0729	281.9719	506.1136	186.1247	215.6937	267.2094	160.9068	44.14466
SRRD	112.5334	518.1364	1355.269	209.7137	1411.194	560.9713	1898.157	1764.123	123.2821
ALG10B	41.71889	38.67746	108.8333	46.13017	55.96272	134.4264	56.70471	53.08046	46.81585
ZPBP2	66.90336	46.21537	66.35316	69.98399	36.51278	56.11145	52.35598	36.42186	42.4336
FOXN1	212.512	158.2424	87.27587	152.5542	74.39462	95.45403	86.49728	230.3551	248.1799
COQ10A	610.6152	275.6325	263.6443	283.6911	99.7863	239.0773	208.9924	198.5215	839.4128
ATP5H	5974.284	7570.033	5837.31	7499.095	5725.073	5367.242	5903.827	6064.062	5923.726
TRIM56	824.5385	1272.304	665.5405	1457.456	332.2282	689.0104	597.2796	515.9986	1229.105
MAGEC1	64.08897	44.17908	298.6493	48.16677	49.03461	35.03231	39.43342	38.45784	61.63994
STARD13	170.7793	73.53383	48.69417	58.0416	136.5251	58.15464	86.72124	45.20441	82.45771
CALM3	5602.402	7099.918	8118.493	7805.064	6391.806	7410.549	8429.063	9326.009	4786.95
WNT4	2091.037	2797.151	686.9225	2705.763	898.2001	3520.633	1278.352	1114.127	2364.776
RING1	16702.05	16980.87	22458.64	16514.96	25286.7	23558.99	25867.47	25585.88	16544.89
PSMB6	17758.18	21703.5	20232.61	20679.45	22706.27	19496.68	20923.09	22824.63	17733
CAMK2A	43.79282	55.22619	39.91273	54.97572	47.23011	492.1783	129.6709	262.4829	60.40805
FAM134C	366.0051	156.1573	113.5518	202.9416	106.1595	148.564	102.8605	115.8435	479.0392
STK3	7754.588	7822.321	7753.792	11954.79	8663.491	9443.226	10324.27	9053.95	6918.165
ROBO1	36.76118	39.19841	35.2239	34.12392	32.47745	45.81159	36.6785	37.4066	47.90969

UQCRQ	1353.08	964.6954	814.9111	42.52593	718.1028	491.186	1132.711	855.6147	50.2686
SP2	169.4093	90.353	118.0803	281.8756	132.6947	154.3553	151.7221	139.4971	153.0744
CCR10	84.72975	94.97066	57.02718	86.90917	197.3088	414.2787	91.16719	588.185	74.61034
CRK	333.1205	656.6123	251.4986	688.6604	231.8486	169.1791	368.6503	283.3396	521.4634
SOBP	1544.845	379.715	506.1962	635.1727	813.5574	1326.608	799.654	877.1492	917.6177
DENND2A	5629.42	5035.718	2668.845	6525.969	3193.213	2963.663	4144.278	3325.203	7146.722
SKIV2L	2820.547	7984.249	7765.772	5438.297	8782.73	7774.543	8042.978	8190.781	5779.535
COL25A1	80.61732	63.7136	59.23466	80.72296	61.511	76.58978	46.62116	36.08564	244.7432
PITX3	109.6631	75.10651	72.81727	82.18563	61.65657	79.83003	49.02902	65.34439	168.65
VSIG2	518.8945	1321.431	1019.19	673.6763	1350.612	709.0797	557.9462	1322.349	6794.294
GCNT1	2556.046	1714.955	2259.06	3316.314	370.2142	2375.069	2419.277	1109.382	1623.141
DUSP14	146.0399	182.2138	110.7771	110.4556	194.2334	155.0356	129.8919	130.7583	318.1888
ZNF75A	506.2982	348.0622	198.5724	339.4466	154.3254	294.3035	214.1331	183.6272	572.3735
SLC25A19	1352.753	2629.389	3742.161	1688.036	3994.6	3129.136	3341.137	4223.563	506.9679
TRERF1	174.0401	141.8032	193.0263	156.9029	131.612	81.34769	120.2038	55.60146	139.3062
IFT88	45.74651	40.88144	35.03473	40.7546	39.22623	44.22853	50.59153	32.38927	42.20476
IRF8	338.1171	163.8034	423.5685	497.0573	154.5112	273.2532	239.9195	187.1586	367.0338
HSPB9	102.6991	60.90211	50.6594	81.25864	55.72674	52.98425	92.9401	59.36386	91.14965
PI4KB	7187.468	9984.64	9825.265	8228.68	10865.94	10420.64	10591.36	10407.26	5973.402
SNORD30	3250.533	4129.681	3217.594	4155.203	1044.017	2415.847	1458.264	2237.485	4717.696
NARG2	48.95995	751.78	294.4701	592.5239	260.1469	39.8997	253.5953	284.5394	35.76813
PREPL	1372.249	777.8591	1013.435	746.8815	2723.132	1657.056	1995.34	1296.323	1458.639
RABL2A	279.3237	142.6676	92.67548	172.2205	79.33598	145.389	83.6815	105.0522	303.5442
KIF17	61.31745	59.17025	51.71691	73.98595	49.09271	36.57737	40.68742	58.2995	2454.071
VPS52	2070.052	2509.707	1887.823	2014.87	2329.811	2148.017	2146.543	1545.068	4056.69
FAF1	78.66082	693.7359	1012.913	369.5538	220.3059	411.7429	282.1108	556.0452	1073.349
RPS14	3966.054	3190.399	5491.101	2655.519	6366.893	1951.093	4739.363	6263.662	520.5647
BTBD3	83.60219	87.35135	75.12217	79.97402	87.3193	88.95518	89.03101	81.46655	119.6656
KCNK2	57.72321	172.9804	47.42599	44.53703	40.33978	138.2845	33.92316	33.3318	633.054
COL23A1	293.9163	111.2846	77.48244	113.7522	85.86079	137.6348	68.37962	96.57407	273.829
RBX1	278.4816	160.0872	1667.679	1363.704	2103.947	1072.587	1489.529	1936.701	159.7316
ACRC	2150.394	2850.37	2697.582	1552.907	3288.046	2814.788	2568.342	2875.078	2128.695
LYPLA1	734.0386	479.2502	229.8271	1082.599	261.6329	104.1355	135.3404	158.7444	198.7827
WHSC1L1	624.7551	366.8905	816.8658	432.8599	426.4258	174.9692	376.3325	408.0937	799.5323
ZNF586	4175.565	3146.02	3347.98	1949.741	2721.059	3422.785	3394	3665.174	6898.729
C3orf32	60.08339	68.93398	46.079	76.4587	53.92725	73.70171	131.1389	300.9771	63.93322
IL8	460.5092	281.2257	125.4078	305.2809	120.7374	301.2593	126.5277	166.6979	635.1613
RBM15	17449.24	17139.71	18545.1	16922.3	16779.43	19229.73	17101.66	17325.26	17897.31
C9orf72	46.19182	36.21649	38.50246	43.65058	35.76632	45.29179	63.05189	33.15351	42.54291
PPP2R5E	1388.005	1282.582	2817.552	963.668	2624.396	1812.904	3030.72	2395.352	763.6135
C21orf90	76.42934	69.00359	63.94987	81.4221	65.50928	99.43394	50.7275	54.88412	179.5504
CACNA1C	73.59213	94.78002	207.3725	669.0481	164.9872	86.48402	109.8778	198.2934	88.97307
KCNQ2	34.11798	43.19564	151.1914	46.47755	111.1168	247.4455	107.2065	36.53468	42.30761
SHOX2	598.5245	362.6865	138.6246	391.8745	212.2373	200.3333	242.9777	275.8461	771.3865
SYNE1	2195.628	2495.648	801.3842	1202.406	384.5913	891.0322	654.6102	600.4187	1545.999
PHLDB1	4532.303	3672.22	3550.359	4672.089	3590.172	3784.115	4240.283	4220.366	3860.255
KRTAP23-1	47.17736	40.00499	54.46003	35.65332	51.86021	56.7293	48.36042	39.09809	90.56399

C1orf229	1042.105	50.849	166.7022	585.0802	457.0198	521.1267	343.0149	364.5969	40.13198
C4orf41	49.25655	59.62349	235.6508	48.04042	50.08115	74.1533	51.627	73.69019	73.06979
GARNL4	66.76691	39.59702	277.1512	1202.296	55.66486	39.87764	156.9515	46.90403	116.7863
KRT17	38542.16	39264.54	33541.39	41217.13	40237.69	33727.32	38226.37	37688.13	41897.78
TRIM62	120.4311	454.626	104.0767	351.1622	177.8568	276.8173	133.8434	200.5403	1057.376
DACH1	41.84559	38.95411	51.34157	52.18111	46.18864	43.9735	38.86588	48.61963	36.23241
ATRX	52.34738	88.73634	57.25965	51.31401	49.56356	52.22622	70.26714	51.02195	1020.805
KRT28	35.74001	39.60917	35.93198	31.92187	38.90095	35.90519	42.10959	34.65842	41.89537
TRIML2	39.77943	49.3837	659.0718	547.4802	48.64	44.57263	354.8195	54.9546	45.50769
CSPG4	5446.462	5376.632	2430.779	7007.344	2182.914	4934.229	2638.164	3328.998	5428.955
GPR114	2999.694	1733.455	7168.087	995.6926	2562.18	2485.51	3873.431	3485.319	2168.684
SLC17A6	70.07545	41.50787	42.69608	60.42054	53.50707	53.35268	56.69436	38.2086	51.24743
GAB1	35.62784	333.9484	287.8929	35.72117	114.1789	165.1806	70.2951	89.72414	927.0343
PINK1	310.3785	291.8097	234.7053	566.5248	484.9247	267.0317	430.4239	620.6954	687.1749
FCGR2A	387.088	1043.13	1291.048	2503.494	419.2037	804.9567	802.5247	676.927	1764.059
ASH2L	119.1747	162.5313	752.7312	613.3226	692.3071	592.475	525.5251	297.558	81.96431
NOP2	218.523	78.13749	127.2076	341.709	249.9726	101.2592	155.4473	121.4643	164.8923
INS	148.4737	124.3857	66.02717	1347.878	61.14175	87.14755	203.5918	113.2577	169.0833
BRP44L	879.3007	900.9355	683.1136	1018.489	458.5346	331.0969	331.3979	814.3528	1376.199
TEC	446.6982	231.5444	131.819	226.1267	125.8991	182.2646	105.7075	133.4514	611.3669
SLC5A2	38.29081	38.6902	40.68827	33.02964	39.84435	36.96345	39.30117	46.87207	49.33494
PLD3	12599.04	10555.08	8044.41	9842.269	3614.824	9036.902	5547.088	4939.707	10555.44
ACE2	53.29506	94.1951	244.1598	60.33384	360.6997	89.95831	547.5826	419.934	99.04987
PASK	213.9399	102.9028	92.22864	162.464	125.42	317.4734	62.0788	67.92471	179.9932
HSBP1	136.5186	2178.666	5748.698	2770.088	10567.23	3739.995	7316.451	8076.878	153.2672
PDPR	185.9003	67.14481	204.5612	95.38113	267.3261	128.191	279.8536	269.3406	102.1049
MFSD4	43.0309	57.85417	37.60991	37.08363	34.39365	43.99576	42.20176	34.54724	59.66751
EIF4A3	2760.576	4804.634	7418.857	4960.678	9178.188	6806.804	8993.974	5550.171	159.821
TRPM7	838.9528	457.8234	249.8394	689.1608	157.7097	344.79	232.7238	182.6106	919.8983
PRPS1	77.69072	65.38682	46.20456	69.17907	45.91729	97.13015	97.49841	41.45068	65.43809
CLU	36.95269	32.9671	47.96115	37.55636	39.71283	34.92247	41.26291	31.76103	35.05884
HSF1	37.21839	39.83777	35.18383	56.389	35.3332	34.27553	34.41275	41.90203	36.8785
ZC3HAV1	2473.812	2526.207	5326.06	541.972	5843.505	6034.511	5544.991	4144.642	1745.462
SPTBN2	308.0943	151.1745	149.791	133.1526	548.7947	164.6162	344.9393	282.9751	434.8426
CCND2	79.08114	154.1075	75.72334	82.07095	57.71433	90.72557	209.8894	65.25866	965.087
AP3M1	442.6743	237.8685	178.0907	256.5159	159.2351	169.5609	128.5509	138.1636	613.1959
PRM3	67.49389	136.5621	61.3498	90.9909	62.04122	95.77156	57.84827	49.78966	241.5605
FBXO7	355.155	164.1907	136.5819	304.901	162.5942	184.8851	96.25623	139.221	515.2515
LOC38852(	45.61581	119.4134	57.95465	62.88652	55.99541	55.49711	51.28206	37.8671	51.65907
TMEM61	145.9893	55.64795	164.1578	45.8365	128.9912	131.8367	93.38324	36.69919	36.98963
FBXO9	65.28957	46.49066	49.13069	51.73538	35.04272	50.51035	43.98977	46.17212	35.26028
RPS6KL1	308.6269	132.9948	365.0717	173.7206	200.6363	115.305	353.8083	80.25339	369.1348
NANP	270.3583	330.3966	141.939	201.0503	111.513	155.2958	123.79	108.527	353.3375
S100A13	77.93613	108.1031	59.64523	115.2113	188.2186	67.59863	57.28292	69.69603	54.1186
NKX3-1	60.47475	51.37874	44.52357	57.11039	50.39164	56.47345	79.80867	44.21758	90.28074
AIFM1	766.6382	228.8341	194.5792	262.6588	107.7757	158.3012	113.7212	164.6947	493.1825
SQRDL	88.26423	58.15792	58.45657	83.48032	58.38836	82.70473	47.664	55.82406	88.1397

WDR51B	133.7936	61.42654	75.86211	82.60074	61.55322	92.30624	66.1288	73.33202	61.56489
DFFA	2791.396	2121.823	1961.627	2460.022	1372.599	1935.119	1875.097	1635.868	2068.745
MTX1	499.907	559.8669	961.2235	755.9376	1263.385	739.8718	1251.623	1246.285	64.58241
SLAMF8	2539.662	2532.75	4230.92	3047.09	1718.83	3621.128	2755.336	3845.205	201.5303
PDE1C	47.93124	39.7788	37.10924	52.48587	49.82194	38.52774	42.86583	38.12586	135.2928
C21orf59	156.6518	126.8144	65.38247	157.4925	55.34937	85.38967	49.57853	70.91888	66.98754
KIR2DL3	41.25406	2776.419	732.1326	62.38769	45.60986	1942.473	36.23788	776.2144	49.28842
INPP1	1242.336	3051.628	5797.105	1740.615	6290.16	4994.458	4681.876	3169.648	3061.22
SDHB	4382.653	2928.902	4010.797	3255.2	4351.372	2172.884	4255.386	3075.031	2673.493
MAPK3	13721.46	10936.11	6729.87	11511.43	8218.171	8052.344	6580.17	2519.733	14161.89
PJA1	60.85992	39.28231	61.31631	78.51884	112.4323	115.2864	71.69242	74.33494	712.2733
PCBP4	69.73795	85.94054	56.66531	63.40152	48.12399	45.75891	52.81086	51.21749	132.065
SNORD73A	374.476	269.2076	163.7928	311.7404	85.54195	135.2364	85.71984	102.1527	163.5702
VASN	4163.841	2856.088	1959.757	1926.452	2201.321	1512.561	1562.188	1731.036	4888.62
LOC20122$	47.03283	233.2377	163.4063	41.56273	310.879	331.5267	275.6632	244.9758	226.2967
RANBP10	37.5559	30.70138	40.47816	36.64021	89.80116	35.6698	34.81424	53.67045	53.32002
LPPR2	208.3294	131.8704	312.461	147.221	560.8231	116.8074	327.0656	442.3513	2422.725
ZNF577	193.9397	74.99508	54.27995	115.0563	58.30423	74.32133	52.84882	51.73631	90.17642
LOC11338(	185.4208	106.4965	514.0486	566.5067	226.1962	105.6267	189.7912	295.4863	304.9245
CCDC128	3002.904	3465.043	3408.707	4264.191	2858.064	4242.286	3329.349	2644.676	2723.398
COPZ2	97.96205	759.5301	237.7326	45.99267	412.4973	569.381	455.137	842.6123	69.10517
GABRB2	53.56929	54.14293	60.82602	53.82454	50.11066	49.74105	44.74208	56.05718	43.36733
SLA	89.37858	51.38013	63.30067	36.42541	50.66335	59.6843	56.08416	44.76146	40.51428
C14orf149	55.61004	57.39506	45.38401	52.81816	44.22791	44.66718	81.57384	78.78928	44.39949
CAMKV	4636.408	1431.826	1557.718	961.9616	1012.42	548.8181	900.0289	1290.474	276.5238
OR13C8	34.55239	45.26246	33.74812	33.7714	47.12539	35.01461	35.16512	48.60481	61.04792
C9orf100	42.68063	48.88021	40.81649	70.68127	36.78284	40.12095	51.46629	68.82476	36.31232
RAB23	43.22918	40.68471	119.3943	40.41869	39.81187	34.2525	176.6948	43.52203	101.4772
SLC25A20	48.72734	35.33062	44.43706	46.56658	45.23816	52.70354	69.74117	59.65585	46.19001
DEGS1	47.96469	1475.266	333.5013	550.3782	614.9629	1241.072	443.8198	694.9318	50.67273
HYAL3	57.02298	111.03	219.6609	95.60534	778.924	115.9789	811.6027	281.8771	70.10751
LOC40211(	139.7161	84.84386	74.26017	159.8555	76.38577	129.9041	66.11304	73.9073	110.8863
GCET2	49.07533	47.17336	196.4279	71.17171	67.91815	60.89991	62.41988	79.51133	80.99466
LOC44120ξ	133.9999	44.17978	152.3008	71.57999	90.43349	199.8043	134.1072	210.1424	751.7673
HINFP	106.6545	768.4606	1449.735	1102.094	1165.147	1194.458	1267.504	1669.603	61.24543
SULF2	1838.913	2863.371	5217.545	5027.444	4585.641	5045.119	7925.72	6923.488	187.3757
LTBR	170.8513	528.9249	880.873	536.7641	1093.622	275.0887	1065.324	942.724	59.16721
C12orf65	605.3873	728.331	1478.434	1183.008	1065.985	1202.051	1154.447	1477.623	709.8022
SPINK5L2	45.76484	54.00484	41.1905	40.77736	41.19009	43.01741	35.69874	85.10216	83.28566
IFRD1	36.93755	233.5527	155.1468	41.36889	669.9594	227.3284	401.1428	184.1052	53.78864
ALDH3A2	817.9187	1261.073	207.3315	60.29158	449.7521	57.54775	414.6516	580.5867	97.19104
IL31RA	106.6112	89.87333	78.76741	242.489	76.72059	85.43073	85.69792	78.73734	256.7756
LCMT2	88.36602	49.13255	499.2531	44.45118	324.9811	458.6366	460.3194	539.1289	37.7702
DUSP26	1653.902	1760.861	752.2386	163.1674	940.8339	1930.331	3343.709	6139.496	801.0148
PARVB	105.5679	158.1559	71.50603	61.02975	284.7477	1066.24	424.843	69.13052	246.9114
SNORD35A	1397.448	1001.12	149.8256	459.2178	275.5475	41.5713	266.5801	330.1873	1841.555
EGLN3	3142.156	1921.084	1711.226	9435.146	1498.037	2593.46	1627.304	1223.751	1427.818

ACACA	48.00878	608.0164	408.7816	752.0261	407.1272	444.9582	112.2728	57.1349	43.90253
RAD51C	40.10163	37.58392	39.81558	46.77168	38.07356	41.62898	38.63591	43.48175	61.39921
TPP2	137.9738	72.97919	83.61894	106.9332	82.94433	124.3967	60.9043	75.38108	96.36247
C16orf46	78.96861	69.70664	48.80081	71.55345	51.4017	52.68124	40.69548	54.75405	57.54859
PTGIS	2361.943	1021.638	55.18885	50.39648	163.6774	56.51242	203.5065	477.3694	1757.69
SPCS3	701.6361	33.5806	121.1285	400.5264	103.9709	166.8486	102.6133	96.11118	509.9316
HRASLS3	56.4836	512.9889	803.388	36.50023	1018.418	673.0563	1126.178	1127.771	49.70639
TMEM101	460.6171	255.0822	397.9616	299.5487	672.028	275.7664	642.3652	821.1	188.9678
TCTE1	43.21569	35.49744	37.21766	47.77179	46.22108	40.60214	39.79339	46.49591	74.65988
DNAHL1	32.50232	36.39934	44.22712	34.01513	33.71589	35.07005	43.88083	33.26541	33.29702
ANKRD33	84.8236	49.87661	52.45987	47.8622	48.35753	40.66177	41.02129	55.5518	55.05643
SLC4A5	36.11663	241.3772	44.59487	57.00974	34.34587	36.71782	34.98338	39.46254	45.65053
PPP1R1C	35.11711	35.54867	31.87782	36.73821	36.13365	81.11079	41.71452	34.85459	43.42256
MC3R	42.60769	52.11679	40.4216	59.07979	51.69487	55.75835	41.25519	48.58152	63.8531
C21orf100	37.49057	39.17825	39.71133	35.2149	39.48752	45.27116	39.06086	40.3149	50.86317
PPFIA4	2382.226	4037.392	2664.206	8521.858	2889.452	4958.632	4263.443	3264.729	4941.71
OR56A1	42.76438	46.40364	49.32779	54.15351	39.84951	47.66577	44.1965	49.83922	58.46372
MLKL	10855.2	12767.98	12179.16	11083.05	9962.978	12007.3	9685.331	10951.72	10818.71
HOXB6	205.148	170.751	86.00421	219.0695	82.04885	124.8544	64.88654	101.6496	381.1557
UBR7	36.42273	44.61573	168.1918	95.30019	663.8959	357.8099	536.5389	217.303	60.15147
CMTM3	127.426	120.4164	59.9823	124.1296	59.57021	66.76706	60.63562	54.42893	225.2585
XAGE5	35.09365	32.29272	42.64141	60.26945	33.35909	32.03518	32.59288	35.41007	48.94159
C12orf52	1071.621	2442.062	2834.064	2234.803	1188.583	1580.792	2135.642	2436.006	1203.359
THOC5	14603.44	16493.16	19134.56	17359.47	17717.85	17566.46	20498.07	21041.21	16572.52
TNS3	47.26415	2599.535	1482.623	45.31232	1398.174	2524.076	2944.426	1031.692	49.61436
NR2E3	44.68346	41.11246	35.21397	58.67692	35.24685	47.8527	44.76658	32.79465	57.72987
TRIM67	396.7966	196.8969	117.0321	223.1482	124.7125	155.7664	111.9436	150.9522	455.2461
BRDT	48.71473	42.97142	42.35835	34.70171	40.63958	41.78974	39.06138	40.53333	33.29845
NPY1R	58.60614	68.36168	35.94437	67.47063	100.3615	52.22134	40.82518	43.04123	108.6378
SPATA18	92.03574	55.82484	490.6086	45.45588	241.312	72.79947	362.3663	47.49903	68.80987
C1orf24	46.46091	86.42803	116.1624	72.28728	34.41489	59.18756	47.80094	54.40747	103.1327
TAF1B	34.45099	48.26835	37.66808	81.30461	47.14542	51.15862	216.0924	37.5984	55.64094
CACNA1S	298.3488	536.482	72.89616	156.8184	64.86817	137.0853	123.9822	76.92148	328.6997
AMD1	98.95076	55.13423	164.5544	84.26134	175.3051	479.0435	161.8711	56.96098	1316.078
KBTBD4	42.2491	42.63532	36.05231	43.0397	36.16228	63.1379	37.28486	70.74344	46.79576
LOC64386(	68.10905	34.88862	42.26167	35.56203	36.97217	59.12921	41.99442	59.0773	45.7816
SLC25A13	3753.861	3553.159	4150.133	5588.438	4389.765	3671.143	3040.131	3845.951	3286.531
EPHA5	43.2409	72.88419	43.62627	60.57251	50.9787	37.30235	42.17864	45.92817	58.82498
CRH	63.31919	48.52506	47.00594	34.21501	42.84454	40.14688	40.61512	38.92548	57.10307
GYPC	63.27896	64.85421	1360.849	1621.478	568.1671	54.13157	675.7101	553.2909	3963.691
FOXA2	55.43652	49.69091	114.3193	559.75	38.5592	357.467	170.1152	283.9105	34.96688
SAA4	78.07636	41.68287	40.93984	47.77562	48.96962	52.14114	230.6065	41.67976	38.50989
FCN1	6100.323	2802.943	2908.765	2701.06	1087.684	2957.058	2532.212	1980.85	6778.067
RBED1	159.1298	62.45806	537.5715	73.57433	463.5191	76.96284	423.5379	672.6981	47.17143
PCTK3	51.0151	33.68332	42.99966	39.18495	118.1451	38.02339	34.60193	38.42899	38.79386
PGAM2	34.33671	39.49602	49.2257	52.29994	33.85418	33.67287	45.20663	67.49327	39.13784
STK25	767.6121	2797.305	3105.956	2406.483	3122.751	3468.544	3166.552	2153.169	1647.748

ESAM	1204.799	2940.85	2955.669	1793.075	4602.379	2870.512	4168.796	4400.413	2385.919
CXorf61	119.7907	59.82134	49.21433	43.46199	52.91885	58.37457	40.82154	48.34533	79.76597
A3GALT2	41.91154	39.22079	33.1659	34.44364	33.18135	45.07476	41.98394	45.47064	33.80387
MYRIP	30.94448	37.28146	33.84421	33.38745	125.6427	34.56215	104.2515	53.10398	56.96337
GSTM1	41.78057	44.16679	40.67179	51.28866	37.05529	38.49051	63.00922	43.82606	95.40231
PCDH9	57.88736	103.8942	70.91634	88.67813	60.90653	62.51722	56.09286	63.6064	70.5152
DGAT2L3	59.73916	41.45325	55.30251	33.78177	32.63963	46.61613	40.49331	49.12922	53.03279
HNRNPF	233.8856	124.9972	703.9918	100.1838	774.8506	852.2317	837.4567	964.2025	163.4057
ZNF282	49.19864	40.80668	668.3634	56.87163	615.7377	47.28844	530.4277	309.1061	91.95103
LMTK3	4072.551	4423.149	2014.269	5838.595	1335.164	3439.819	2305.358	1602.578	6528.243
KCNB2	101.5666	97.50945	56.08156	84.75777	55.9063	54.5448	54.02076	46.8502	177.2883
PHEX	54.31343	77.20555	57.87574	61.54813	49.26823	76.66445	48.77449	57.28838	226.0069
TMEM52	54.56236	53.74929	38.60322	47.59261	41.41125	44.96815	31.36709	62.45415	61.0054
LTC4S	89.06029	48.34859	39.166	53.77003	32.72876	34.15731	36.6246	36.01611	35.07198
LMF1	57.28604	69.9556	50.50824	46.94313	237.9324	595.0562	340.8714	53.23165	53.41337
ZDHHC18	52.07605	56.15576	185.0538	279.4791	88.92266	57.26692	167.2393	246.7168	103.4029
BLK	41.96789	49.53996	249.5156	41.14376	37.89639	39.871	75.10784	150.37	52.69489
APBA1	56.6282	59.18821	51.30116	49.42685	40.96046	40.85915	39.25169	36.73659	63.00934
ZNF780B	284.0906	270.3724	164.4619	55.21584	86.5462	57.73105	163.8603	140.5797	122.8052
SLC2A9	67.96471	49.54004	349.473	1183.404	41.57742	54.31385	248.0563	42.41774	70.07004
SYNGR1	46.44397	56.19268	38.18529	71.27347	58.17107	47.54802	42.69245	49.2994	67.18075
TMEM148	115.337	66.24426	92.40964	52.39717	54.86345	58.24122	55.90803	54.18578	41.76276
OAF	122.8691	90.09672	1414.627	58.09025	2690.751	1292.39	3608.099	2911.92	160.6969
TAF6	140.1719	68.29712	57.06324	85.35792	68.91849	53.93214	35.1527	74.86095	143.5174
CDC37	35337.93	34749.89	37847.54	35033.52	33711.43	32669.15	35243.12	37971.51	37434.5
ITGB5	3055.482	4822.76	5680.496	5722.727	11752.11	8415.993	8835.246	9366.818	1836.12
SFT2D2	2920.621	2081.365	670.1521	1328.655	264.2174	263.4504	232.8698	271.8643	1689.415
LCE1E	161.1992	81.5272	67.59563	162.3945	59.46444	92.96024	50.47755	54.61121	103.388
FHL2	120.259	404.5342	519.7017	66.98879	762.5762	421.3555	811.4759	340.379	148.2297
WASPIP	49.03365	49.84021	147.054	58.76065	165.6638	41.25053	179.7218	220.1394	102.7969
SPESP1	49.48325	44.68387	142.4556	65.1151	58.83163	71.87661	79.39312	52.37782	46.13587
C21orf42	397.2378	257.995	323.4269	236.8969	99.03388	177.6171	94.98035	125.6462	590.4775
ABCA9	695.3062	302.3596	145.4305	131.1765	59.98605	79.27047	144.2058	67.65834	818.9283
TRIM36	405.5502	214.4098	97.75229	215.9337	121.226	163.7298	90.1563	127.4544	499.8637
PRTN3	200.1024	114.1105	100.9852	182.8371	82.36776	118.9685	76.26913	105.1128	314.8559
KIAA1609	90.35398	75.41212	41.80127	58.5488	41.31465	48.43314	52.18129	48.58867	65.77045
KIAA0922	92.7225	568.7682	688.4541	595.4192	371.7892	562.1414	408.2509	832.2205	36.44276
LRRC47	38.77809	40.49163	47.70099	38.36955	73.15384	45.84613	61.37987	36.07127	57.47797
CWF19L2	54.30605	459.7162	385.738	84.26176	55.31447	309.2199	144.6487	55.11065	120.2669
DDAH1	40.52417	42.43041	34.83258	52.70877	100.6702	213.9586	64.7894	134.159	54.4922
GLYAT	55.91558	51.2855	53.7301	70.40001	37.89821	35.8448	46.22141	55.63404	51.86112
PIP5K1C	1936.907	43.82003	4244.166	2101.248	4099.745	2994.247	4082.625	4666.807	34.33761
LIM2	32.55587	35.09196	45.43745	36.27093	40.1785	34.9412	32.77414	45.25666	36.11193
NR3C1	168.1063	92.82877	195.6234	157.8916	63.43049	118.5362	66.87108	183.4095	1877.015
UROC1	270.8343	175.5713	75.27588	111.9954	80.41515	91.69011	55.98791	93.07707	133.3247
JOSD1	2778.693	1699.442	3005.101	3297.557	3387.222	4664.404	4179.467	2624.949	49.43209
ZNHIT6	54.91302	585.4419	1877.585	1486.181	2105.877	873.0284	1535.291	1002.493	62.10246

STK31	51.92926	37.36463	54.93438	63.82707	56.39027	66.07149	48.00374	68.56059	40.76909
RGS10	45.27439	36.26352	38.87671	42.80243	42.22337	35.52948	180.8668	53.90891	43.39632
RMI1	272.3833	47.44864	40.74746	46.79546	247.4483	57.06782	96.51176	49.50919	32.68814
ANUBL1	658.0254	398.0476	285.7445	58.33437	243.2032	299.6711	288.0518	213.9379	1253.11
CRBN	204.5962	417.671	398.9057	218.242	376.1296	149.302	482.5528	473.1465	197.4755
KCND1	243.6185	184.3094	57.47482	307.7976	112.5396	212.1344	84.15252	63.81558	104.9176
STON1	39.97377	35.75393	34.41532	35.7097	324.7024	34.57449	36.44301	31.67445	40.32412
HPX	108.823	89.21764	62.30206	76.11736	61.44829	73.15539	61.62074	66.78472	325.9933
NAAA	48.1706	70.1061	229.7666	86.74426	57.90971	66.80075	80.77436	52.1541	107.4208
TTC13	64.5378	64.13345	46.22513	59.63891	47.82415	91.15304	41.36926	54.71358	82.61187
RBPMS	35.9216	38.785	44.1609	48.71803	52.13243	57.89162	46.71439	118.6596	48.44995
KIAA1467	104.8787	49.47469	55.99031	60.45078	49.85847	52.97032	45.81987	68.12705	235.2522
TBC1D10B	67.88992	61.77425	44.00314	65.82791	390.6833	400.5951	45.44493	206.1002	90.05987
MYCBPAP	46.13042	65.61459	58.83369	93.06305	58.2749	99.66161	56.73063	57.2479	83.84806
FLJ38973	669.1428	298.449	289.2174	37.15214	479.1496	51.61127	513.3757	426.4553	59.1256
SNORD42A	197.8304	92.61935	38.81631	56.50919	41.50352	38.08428	30.95896	46.8722	53.78853
MLL	55.84345	124.384	137.8829	489.7717	299.5624	73.55313	106.7752	221.7934	1311.77
SIK2	83.96799	95.4575	306.4578	60.92753	218.2034	48.37447	48.06744	341.9914	46.39482
CCND3	5960.875	4283.906	5740.415	4711.892	3164.679	6739.739	4512.491	3237.073	4137.127
NFYB	55.47287	146.8373	64.38738	74.71843	55.43264	47.16674	51.10126	75.98591	126.6638
ARSB	33.08652	37.78159	78.68427	44.42188	39.70233	41.67026	47.99774	35.90204	35.2415
MED15	67.94	368.5556	524.3342	59.28562	566.793	78.04018	670.562	687.935	52.96238
FLJ20850	139.2694	91.84896	141.3522	104.1586	63.38757	105.497	103.8648	68.19572	66.45304
HAS3	57.75644	54.36303	262.0179	784.9525	120.0457	501.6142	47.81905	58.39289	43.26295
ROPN1	41.40999	60.22965	46.38862	44.22295	54.61817	65.5962	34.29133	50.06244	73.35746
PRKAA2	40.55563	55.53291	42.14434	38.04494	40.83737	42.76487	75.07036	34.99601	76.82984
GPR17	47.96371	43.97279	39.00365	54.56046	38.74474	40.2003	34.53467	38.16724	99.56551
TTC30B	124.6155	229.1557	46.14191	221.2286	65.99641	118.0122	73.51072	46.41494	80.53017
CENPM	58.62511	44.00438	45.52927	54.24255	49.87202	43.91185	43.97188	47.95727	97.37673
BATF	1154.063	460.0521	1092.526	291.3511	200.7687	803.148	416.0414	702.5098	794.5325
RSF1	340.1644	428.5472	240.2009	544.8596	208.9292	316.0052	324.8422	264.9984	705.1394
ACOX1	64.61129	48.54237	46.04071	81.6216	46.50943	43.88407	47.14548	143.7526	239.3941
PCSK9	82.66994	134.5767	90.98075	89.89996	113.1547	113.6702	67.17239	87.14158	63.26062
AADACL4	43.2909	42.37897	61.16616	111.3873	66.59711	71.63614	61.22526	78.97692	66.02955
LYPD1	542.494	324.5869	264.4052	358.6926	121.1359	331.9581	307.1576	220.3907	349.6313
SLC2A14	37.88041	50.33029	49.29041	50.25709	42.68002	45.00434	40.8299	51.52593	187.7811
HECTD2	59.02455	66.51545	84.46243	78.41132	227.807	79.00259	177.2639	277.804	70.40253
AKR1C4	92.64806	97.21107	48.80468	60.83797	44.25373	65.27282	54.13981	47.27461	53.91531
OCLM	249.188	73.13223	77.73759	120.3889	106.6385	69.9319	65.34486	72.30127	135.0751
ADRBK2	1506.138	702.0381	958.687	855.919	400.7074	1667.526	1292.551	1134.624	42.07049
MAP2K3	54.43629	61.18233	36.24382	36.33652	46.82803	39.35231	36.27642	53.4222	47.96173
CIDEB	102.3612	685.0667	2757.988	2199.126	4075.836	3487.028	2415.135	2246.278	68.45683
C14orf48	98.89601	98.92728	45.47746	145.5989	58.77454	118.4578	48.69803	66.01041	79.99925
BEGAIN	81.51955	54.80075	39.48136	66.80273	45.39691	50.55291	149.231	45.20641	45.46178
USH1C	35.21502	38.18919	37.70042	48.80423	34.70176	60.48363	38.43633	31.93845	33.54756
MID1IP1	314.2462	122.3332	447.7675	136.9865	236.0373	322.3547	199.3215	201.0711	266.7408
MIXL1	45.90224	52.55444	47.62816	57.74173	46.40239	47.3828	37.35221	37.69374	38.0244

ACVR2B	38.28465	37.02317	35.13	37.68585	39.59526	35.50469	37.83179	96.63055	679.8668
SPNS3	108.0064	178.1444	259.9873	141.4487	58.66822	64.59367	52.75326	57.53215	93.85021
PRELID2	38.34187	509.0394	206.8043	632.5214	33.87075	238.8703	43.70679	44.18209	43.93103
C1QBP	379.6883	237.3146	123.9766	244.0204	129.2095	171.5723	120.8116	157.5613	322.6597
XPNPEP1	6497.087	9371.426	12187.16	8562.099	10003.42	11355.85	12338.29	11574.45	8317.925
FHL5	1243.128	554.1632	36.12886	46.78645	35.38851	68.06317	207.5519	379.6076	1643.28
NCOA1	2821.908	2363.199	2475.423	1698.91	2296.367	3072.63	1605.281	1187.233	2045.526
FOXQ1	2005.558	1380.408	2601.951	1156.223	5552.835	3774.263	3181.396	2439.367	1634.178
LRRC8E	224.0047	161.5366	259.8218	190.69	1743.795	564.0621	641.7649	1314.297	407.6942
ZNF823	195.9753	118.1556	72.22036	130.8225	179.934	192.442	129.8949	112.5537	155.1228
RUNX2	38.27973	51.49026	43.52422	761.0199	197.5361	69.98449	249.222	302.2811	55.59618
MIPEP	56.23101	37.27446	56.63846	44.23836	236.9061	54.52312	65.58176	51.93186	35.16699
ZNF672	92.69191	93.98392	216.2306	85.84053	320.3127	278.7382	427.1708	513.175	127.9755
ATF5	961.876	2262.919	1248.003	972.3036	876.2554	1184.17	1155.288	1759.622	1373.111
WNK4	51.61835	34.6973	513.3221	576.0988	665.5223	1222.916	159.9935	32.52934	63.34013
FUNDC2	116.8386	124.2668	75.95451	125.5008	67.22829	117.4503	60.54597	82.32505	143.6377
OR52N4	165.5626	55.78123	49.4543	82.38121	49.1797	51.27033	53.36053	68.35205	173.3742
NARG1	37.64071	54.59778	352.5452	548.0698	379.4504	411.5388	291.6465	197.1465	40.17458
PTPRR	314.4584	183.3271	101.8849	218.5821	109.2338	163.2356	79.80701	126.2294	337.189
IL4	47.39392	54.05524	34.94913	39.51995	42.83681	54.78041	36.026	53.6566	34.01957
DHRS12	70.59719	103.9637	64.15636	62.5728	33.60707	45.3456	78.55267	64.0134	75.7532
RASSF5	69.63506	672.7689	699.6732	39.74619	331.3452	429.7964	276.097	137.169	39.72504
ABCF2	53.4747	53.36907	65.81283	64.77959	363.2094	62.36124	224.4117	361.8406	70.89347
CNOT1	13157.65	13208.84	14928.18	15028.61	14112.18	15804.14	13472.42	13876.42	13744.17
BZRPL1	69.89464	63.5809	64.66309	55.6753	44.59702	55.34968	51.34222	70.85309	54.60317
SNORD16	47.32988	53.26595	499.2347	35.53784	41.88194	50.10299	86.25317	98.99847	1374.611
CUL2	48.44427	83.89462	585.2849	87.48647	296.519	69.53511	644.6823	626.0761	43.61732
ANKRD49	39.44915	40.2584	158.6356	38.42862	193.8404	35.82673	137.6518	36.68132	67.29085
MEX3D	5982.923	3795.05	3131.336	10790.49	2256.048	2590.105	3052.872	3667.316	8658.151
GNAL	53.43426	65.64507	59.81148	56.24549	37.71042	52.80083	98.81623	54.16147	74.67593
PPT1	1051.836	1202.406	4737.165	1770.947	3270.299	3499.191	5296.879	4656.03	1203.057
ZNF16	363.1063	202.7736	204.5045	200.2182	222.11	130.1961	148.4684	103.6415	473.2464
CLN3	50.94629	706.7589	122.3945	73.9727	228.5527	63.72871	220.371	223.3167	46.12754
YWHAZ	142.2238	493.0012	374.4106	581.2167	150.3687	596.4808	321.6098	174.1468	127.7034
C14orf165	72.01837	65.10972	49.26185	42.75796	65.9142	59.83406	43.907	45.23706	58.09332
UNQ9438	50.5153	61.69368	36.24325	45.22898	40.13667	38.6167	33.52837	41.49331	55.89117
KRT2	46.29452	35.80537	46.77463	39.04937	43.10564	47.73385	35.19933	42.69191	56.07217
YAF2	94.01572	324.9012	337.2904	337.3019	560.8984	428.0139	473.1436	792.497	50.72868
BRPF1	139.3574	111.3333	1032.767	97.92905	421.3253	369.2997	522.4149	326.1327	305.6833
RHOT2	104.5002	1634.138	3000.477	3812.077	3963.354	2068.984	3632.781	2419.685	65.12392
GPR77	82.85828	41.70735	105.6768	52.91297	43.97066	59.36404	47.17143	54.83821	68.11745
MATK	42.4755	37.6728	37.73186	34.19673	50.05008	38.13068	70.39265	44.99878	75.79517
BACE2	2048.957	1890.786	2986.424	3855.428	2285.573	3310.784	3189.457	2033.943	1000.23
RUNX1	521.5062	2310.719	1696.15	2418.08	747.4985	5234.727	1430.589	962.6021	4260.638
CYP4X1	207.1998	156.0435	84.27197	85.26375	69.42989	116.6229	64.92172	129.3545	287.1745
LY6G6C	1711.397	2217.008	2209.893	379.9326	4310.296	1643.195	954.496	1757.471	341.3588
IGFBP6	225.5764	138.8032	51.22925	102.843	1028.215	497.0674	857.0594	1107.554	391.5226

TGM4	251.6872	39.11999	40.47745	38.37687	37.04197	41.69561	40.80077	41.40044	47.42834
RFX4	82.51998	89.74061	64.24919	94.30532	70.62662	49.92117	49.70725	80.06154	86.48272
DRAM1	44.60015	77.10474	38.1378	83.94256	39.7666	51.06163	46.33525	38.17692	54.24248
AP4B1	41.21939	39.44358	2478.02	48.78838	1997.371	1604.867	2156.612	2360.125	59.52861
LONRF3	751.0967	36.95739	37.4572	59.4403	76.10489	51.62661	67.12162	124.1925	36.13789
CPLX2	79.36191	99.93065	75.81641	153.3197	60.05154	93.85015	51.95163	63.11023	159.3534
PPP1R13L	4479.257	3350.903	2954.63	6536.39	3099.381	3200.272	3133.712	2496.471	6686.306
EI24	91.44811	286.8292	173.8377	360.4509	225.1589	222.4444	256.2253	269.9001	73.88593
KISS1	119.6173	90.29634	48.08119	89.3378	57.37402	56.63652	71.57992	67.2085	64.99298
VSIG4	454.322	218.741	97.61093	138.0684	101.2483	108.1292	70.56696	103.3844	270.7023
CHGA	146.3882	121.4517	74.12714	97.58112	75.06288	63.34313	53.13776	66.81052	60.00229
LOC646531	11585.89	10751.37	7840.604	13596.31	6160.403	6975.075	7531.821	8205.514	13066.86
NEUROD1	100.6532	60.57786	68.06457	78.62407	47.22091	53.77167	61.16984	36.79971	85.5684
KIAA0913	217.0998	572.8165	1773.286	128.125	1580.026	1024.504	1484.13	1592.498	1260.081
KCNJ14	135.177	104.3181	66.90821	179.6013	79.18966	89.80446	63.14353	78.77713	201.617
PYHIN1	87.06019	78.46358	50.87876	42.96695	70.27741	178.3657	65.69064	111.5684	34.76405
NME3	56.63447	2353.868	2556.438	1021.422	3369.671	1528.164	2679.358	2518.578	204.2874
TXNDC2	41.82347	42.33606	36.03339	36.62323	41.70908	32.57753	33.87252	48.16877	38.18333
EFCAB6	80.07531	758.9474	46.88616	882.1841	197.0568	44.70097	109.677	54.83109	81.21199
KDM4D	40.80819	41.84916	33.43412	54.01593	35.83257	35.15054	39.38701	30.02165	36.26633
PCDHA10	43.03318	40.30602	36.35359	34.64369	32.53174	43.22815	37.62072	37.75678	35.94211
MEST	47.64751	59.70449	34.62015	46.5331	48.30788	47.14004	38.4278	33.40317	40.0412
WDR6	1174.7	1131.92	2761.591	1135.079	2852.419	1884.794	2992.237	2765.783	1580.469
ADAM3A	55.84364	31.64173	61.98359	54.98731	39.07833	44.99627	33.32188	50.62817	71.89648
OSBPL10	1135.244	34.92072	503.9982	35.69764	1039.684	274.7549	801.6229	347.2413	37.23646
E2F3	2862.199	2615.247	1990.626	3066.424	1067.885	2475.796	1619.047	1202.57	2068.2
ZNF229	97.95986	70.35719	53.93753	62.37758	64.21989	48.43054	52.30187	64.00347	111.0732
TIAF1	178.6136	135.082	71.28393	107.5823	74.66189	130.06	143.3176	65.4145	276.5806
PAF1	9832.202	10245.74	14884.47	10965.43	15486.88	11467.68	14742.69	14822.4	6134.244
ZNF193	99.9696	72.08563	48.4757	73.20788	270.5903	68.87478	218.7666	59.43425	311.8018
FOS	36.67062	891.4316	261.3073	36.80854	1719.564	49.69647	486.2083	457.6837	36.54123
FABP9	63.66932	54.26938	47.05809	43.82003	41.73222	54.66258	40.7099	35.78717	55.89182
MAD2L2	77.75439	55.67613	46.50138	109.0174	489.1231	447.7369	301.5599	182.7005	63.27346
UBE2E3	250.7648	1108.399	490.5592	220.5456	709.4752	303.6377	715.4392	1342.506	1992.588
FAM48A	63.48725	53.66797	598.4968	1394.181	330.5172	1050.31	272.7993	530.5781	43.27179
AIM1L	49.29593	43.69255	39.39764	65.45592	173.302	45.10208	38.51595	39.51089	31.51831
RNF126	60.72025	107.2258	395.8389	1755.221	452.0733	67.32555	59.99452	231.2617	2624.15
ILF3	58.06845	875.0971	486.761	44.25523	357.5046	54.08494	375.933	234.4955	48.64692
43526	159.6704	87.27666	51.88328	74.67601	113.35	67.56915	81.06539	36.38659	107.6456
SUV420H1	60.23577	1432.245	567.0109	573.3762	754.3888	572.2231	720.7578	498.0246	119.8218
DENND4C	10240.87	10469.12	9693.909	8179.694	8327.97	8434.494	7242.672	8732.426	11446.97
PHKG2	107.5067	42.47685	281.476	60.68193	577.0463	77.90507	486.3129	632.4863	55.50801
CCDC13	35.05318	36.55179	46.85619	38.43526	42.46095	53.35807	35.28605	43.38327	42.35994
LINS1	53.88409	58.14979	47.82132	40.75201	40.80854	69.00335	48.46245	51.08942	61.13872
GPR142	105.8527	109.609	57.06477	64.21063	59.91391	73.99471	55.67876	44.78561	217.9763
GLRB	49.6138	54.56463	32.64658	46.48299	38.87275	35.78212	45.16508	39.25347	55.78129
ACP1	52.13533	35.90904	155.1696	37.24887	34.54142	194.6942	67.68085	118.2297	45.94335

NUP85	35.13815	1064.214	136.0017	43.53653	1369.582	39.55868	745.4669	1156.89	46.46226
PKD1	75.23265	51.26017	38.7664	52.02995	351.2283	35.64989	147.1072	59.05322	128.2378
PDLIM2	42.45927	34.53436	42.19135	41.26139	40.47393	35.02012	36.57211	40.08863	52.69745
KCMF1	300.9807	192.2016	581.8953	226.4371	149.1474	351.5408	114.9789	105.6535	374.6546
CNPY3	92.70482	46.60564	177.1934	102.5058	41.66019	48.94825	158.2484	177.0775	70.56264
IKZF3	2135.177	2783.463	1386.378	1392.908	405.6127	1142.461	635.579	610.6784	2759.688
ZNF792	2748.592	2301.318	2168.383	871.4273	1676.179	2387.723	2329.194	1866.962	2048.057
KPNA5	49.57579	65.93956	46.62828	37.71983	67.97314	50.82508	71.02697	49.49754	42.05837
TNK2	8593.599	10755.62	11680.83	10122.23	12272.69	12549.54	11439.61	12372.09	7578.83
TMEM108	39.9207	40.11541	36.01214	40.55234	35.28476	33.37397	35.78087	31.99866	69.51951
MYH7	7548.443	14546.05	11672.77	10228.23	17496.42	15874.06	27857.21	30965.6	15087.01
GTF2IRD1	98.69514	92.95515	62.46243	114.3775	54.63762	80.96619	59.45337	52.22062	178.0569
PSENEN	472.3274	330.4116	657.2891	314.94	560.7292	440.6527	704.031	493.5005	953.2234
LRRC61	282.6622	183.5177	86.0508	158.6026	66.46571	82.36173	138.721	73.22209	317.5285
WAC	66.48527	43.36333	111.6169	106.8819	95.58878	65.30427	50.36334	59.88817	737.6037
ERLIN2	118.113	88.85623	72.84624	65.31121	74.70199	71.34589	84.00803	88.04301	78.31049
RAD54B	38.37581	37.95872	32.97618	38.48726	38.25508	54.47848	34.3714	37.14922	40.25515
UNC13B	190.8425	72.69487	609.9232	112.1522	918.1971	76.70335	419.5296	342.5103	76.49089
PDIA3	352.0552	195.0588	77.98199	209.3278	105.5432	215.3432	93.66957	107.4082	68.35003
LRP1	156.2638	1064.416	221.3362	572.2469	112.8051	94.96143	299.2921	443.9745	2760.958
ANGPTL1	46.61629	35.68804	36.94196	57.95925	34.36777	38.06574	75.39787	36.77268	46.76117
PRLR	456.1552	187.9249	197.3505	240.0814	68.07537	210.3738	217.9122	194.8016	239.6479
NUDT14	1548.261	1356.426	4831.333	1478.038	3984.751	4274.75	5803.775	6365.835	1918.246
CYP8B1	47.75567	627.8568	59.62117	46.38707	55.73735	85.61882	70.11996	60.15732	75.52748
C1orf87	43.3849	41.30744	50.97246	60.01903	39.6214	35.2044	35.64975	35.45748	39.70549
EYA2	36.81569	70.40568	39.70847	62.29099	39.94932	56.54968	43.70793	33.32847	60.42476
CACNG7	37.78528	35.87523	37.96962	49.14547	43.75346	60.02859	37.13063	45.37088	61.93885
ESD	231.5982	81.7225	68.25859	146.3822	64.55431	119.1863	93.263	129.5498	165.0417
C6orf192	5119.725	4799.092	7288.839	4365.122	7229.22	7430.865	6575.278	6349.156	3344.448
TCF12	55.54918	1094.428	409.0093	571.6805	824.3398	636.5392	719.9836	349.3374	47.74509
SH2B2	43.59717	51.19832	37.89849	52.60868	38.32985	31.48608	42.0841	37.18663	38.46678
CHAF1A	5508.292	6103.489	5821.517	8821.157	5914.727	5579.089	6052.736	7654.511	9315.045
SLC25A31	115.2423	96.94852	61.83941	86.13184	65.29286	60.67218	53.50983	45.6624	367.783
CUX2	31.98328	34.42616	434.9167	35.22297	32.60226	36.29483	360.005	198.1302	66.50664
GUSB	100.7668	72.98926	1541.064	56.47147	2120.116	1604.756	2208.977	1689.547	38.52837
TSPAN13	39.64766	51.6909	50.2445	63.45932	50.51442	44.33365	136.1314	46.33379	35.7063
CSTA	21512.28	21666.01	20741.6	16611.45	25979.89	19279.77	16529.79	19317.12	20505.44
EGFL7	1162.322	66.4474	73.01649	69.60484	41.45341	75.43478	51.8957	58.54126	81.02477
DCP2	62.35579	66.69129	766.5948	57.0703	1552.969	1559.915	1398.501	1224.513	103.4604
TEX14	76.17934	70.09441	56.22694	68.42658	47.10428	46.90272	52.29335	70.45918	54.27673
ICOS	312.2797	1575.172	3117.69	88.13551	367.5562	2239.913	1463.452	1772.552	1692.907
ARHGEF16	7172.104	5621.749	4426.429	7863.257	4451.695	5073.135	2943.63	4678.343	7815.906
LGALS8	127.4259	63.26358	42.14758	62.81902	54.80944	69.2236	56.36276	48.49822	232.1859
TM9SF3	1254.034	1143.792	695.0596	1409.903	599.5391	1219.052	772.6628	1030.426	1353.108
TMEM38B	1133.269	1337.539	1101.359	625.8071	631.2432	1844.673	1668.405	1408.333	446.5233
NDUFS7	11982.77	14508.91	12259.13	12028.25	8613.649	11848.9	10087.84	11604.24	14202.77
DKFZp686C	370.4944	143.0746	106.4132	229.9477	75.14117	422.8565	74.10636	93.74503	357.8854

CCDC85A	2238.169	1615.925	827.7189	44.68009	1957.278	1107.312	767.9892	990.7124	1380.076
RGS22	43.81389	51.77372	47.35971	48.8483	31.56113	37.96993	34.70161	51.84131	36.56028
OR7E24	34.91063	40.40183	36.52635	36.52148	39.15505	38.13833	37.84016	35.25983	37.01672
DCAF6	11759.49	12338.34	10732.79	10663.58	9069.446	10479.11	11303.04	16040.96	14867.7
PEG10	91.10193	44.4249	48.92121	50.58414	51.7513	62.12763	142.4158	64.17605	82.43875
LPIN2	68.50455	81.19647	285.9745	924.7055	196.4694	88.28744	127.5217	63.00579	107.0091
HIST1H4I	58.19394	39.40565	38.01846	35.11279	41.37793	37.69236	38.98225	41.0749	34.39904
POLR2L	33.63756	45.96828	326.9266	41.57528	361.0025	51.8925	986.7372	619.6154	59.09949
EXOC3L2	72.1231	351.0671	78.50001	134.4955	55.16188	58.34717	68.49674	76.38194	112.1223
C1orf210	1765.28	64.00998	236.1547	51.84022	62.30475	53.41211	62.56297	51.11816	42.7248
FAM155B	107.7021	89.95298	68.86572	91.76643	1192.117	68.83618	212.8677	348.5052	128.2291
OMA1	42.31652	43.49476	341.9464	60.76268	686.2689	391.7451	320.281	768.7874	36.61609
FBXO43	267.5377	148.6711	95.36694	145.6551	103.2433	155.3675	72.42296	122.3874	528.2599
BPIL3	39.53022	60.8666	61.58192	44.52206	40.3358	48.94247	52.49561	54.25795	84.26578
HIST2H2BF	216.1894	125.3195	77.71044	104.0775	70.93027	301.0602	79.7739	83.11594	168.6779
NRBP1	1008.521	464.8906	318.8512	57.94404	304.415	49.49767	575.3346	375.6724	56.60329
NOSTRIN	2748.509	2309.436	1478.989	1561.771	1901.912	1567.078	1239.936	619.2429	218.7343
CLEC2D	62.79172	36.71501	103.3458	81.55356	38.58298	42.59303	38.60868	44.48554	79.19127
CDKN2B	62.40504	483.0864	347.0304	488.655	182.4928	53.2506	181.2236	362.4998	125.6079
ABAT	61.72932	59.61977	68.71847	86.47314	63.63086	47.06878	50.48389	61.33571	113.9802
ZNF624	305.0125	110.7041	105.8837	166.6367	84.46661	139.5309	70.88401	75.18458	281.1259
ARID3A	1904.065	1899.328	1142.866	950.2889	529.5997	1393.348	853.7435	744.019	59.1938
FADS1	38.93454	439.994	81.0243	39.81131	329.0185	40.81404	185.2542	223.0401	45.03553
TEX28	83.59937	67.78609	68.09513	70.71686	47.8689	51.93978	51.87448	53.01564	94.36768
C14orf70	43.7328	60.30268	34.79445	44.70407	45.91749	44.76336	50.48521	46.27298	100.096
OR10H2	74.08367	67.23697	53.05291	79.96902	57.81354	88.55251	48.60261	62.29596	179.7388
DFNA5	3304.141	1556.699	2616.287	6108.41	2013.665	2989.178	5263.854	1757.22	910.0955
PHC2	33.43685	35.31367	40.20861	47.84887	40.73049	54.10196	44.77569	42.28725	69.77792
OR4S1	339.0737	214.1575	116.4567	242.4373	108.2062	157.4844	102.8203	133.6318	391.5406
FAM83A	34.41352	455.9592	172.7347	506.5555	132.9444	38.11214	785.0953	783.006	1338.381
WDR33	656.0854	921.9011	3759.611	2277.485	4885.93	2981.016	3109.548	2152.297	901.9897
SSH3	37.56231	38.85797	40.99128	728.1686	325.6111	75.8969	60.0172	39.46857	57.90649
ACY3	50.81855	51.79241	48.25141	48.07659	40.85781	105.3297	47.84977	44.11427	35.87618
FLJ46836	122.0812	145.7401	76.65788	185.5697	94.49236	76.38966	78.05143	126.4265	160.9527
MCF2L2	287.7565	125.1915	69.90276	117.8333	96.26975	116.7094	70.28683	120.8697	284.4003
EXOSC6	11987.46	13546.35	11104.53	13606.13	9346.265	12708.72	11482.46	8118.045	13121.81
F8	44.58976	54.6868	155.0905	55.23674	62.61584	245.7108	54.49095	51.0029	87.2492
CDC45L	55.93718	800.5684	1731.636	1683.085	2635.844	1444.609	2821.796	2175.703	945.8057
KLF3	314.4463	275.9833	104.0244	180.1522	119.6953	153.5909	78.55655	95.26162	427.3585
PDK1	420.5355	429.9707	416.6278	408.4894	481.1716	585.9311	375.5036	212.5139	389.2163
C1orf71	61.92027	244.8663	142.5388	58.66901	323.6233	203.0223	140.866	182.7832	439.0944
LRP10	776.4547	447.829	388.8727	415.3365	616.8537	277.9564	517.9115	391.9311	496.7692
POU3F3	46.02652	52.27615	34.0757	41.56077	37.14188	34.80402	39.44222	43.96236	64.04099
AIRE	2430.556	1007.832	889.8521	40.96712	182.884	930.3394	263.4863	157.2467	39.96972
KIN	109.3292	1137.973	357.9874	109.8583	289.9274	1147.241	361.8496	354.3927	1071.444
HK3	41.17673	31.46025	47.96116	36.46514	56.3244	37.146	47.21243	34.55506	40.37093
SERPING1	953.8572	133.1684	690.6974	78.05468	871.2924	301.3483	665.2699	482.8256	160.7821

SCGB3A2	127.1859	186.0048	94.28928	122.7415	97.17616	161.4034	78.76797	66.36063	163.5796
GPM6B	156.0211	83.17035	48.82665	66.31736	52.96255	89.11951	54.06924	59.62313	180.9317
FCHO2	624.1206	392.8358	1454.475	327.7911	1962.629	846.5767	1206.192	669.812	36.69627
SLC47A1	4363.284	9452.408	4017.731	1602.767	6137.083	6335.037	3459.074	6816.333	5240.061
MAGI1	70.63293	68.49081	60.82357	84.58204	71.27869	103.3078	54.05317	89.71814	116.2881
RAD51AP1	232.4122	212.254	132.7262	186.7814	132.4399	250.5384	134.624	142.4727	193.3627
PLCXD2	97.92921	77.07187	62.76554	73.82065	102.5162	62.57233	55.65881	48.56897	61.84863
ZNF20	6962.656	4841.574	4816.408	6170.325	4907.195	5681.09	4818.37	4824.232	4993.444
ANTXR1	23164.14	21850.95	22280.39	20938.77	23295.97	25993.74	22172.43	18105.24	20737.6
PCDH7	42.4937	74.37555	136.3508	88.44831	108.7025	71.98961	219.434	42.18722	67.26296
TUBAL3	75.09779	57.55139	271.9126	43.06243	540.3499	518.6533	117.4739	50.10528	45.54332
BCR	45.87329	36.95159	44.49434	50.07529	41.27433	35.31348	40.2457	39.41901	123.4507
METAP2	12045.52	11280.57	10824.27	11998.11	10719.25	12176.57	9964.539	11356.91	11959.05
FYTTD1	1230.844	1649.912	1785.233	1745.55	2108.565	2742.42	1921.104	2364.507	1222.59
TEPP	116.6304	77.22927	54.06575	57.15623	175.0152	243.2089	55.53615	49.40855	54.13726
RBM42	7899.653	5954.101	7827.47	6841.908	8271.761	4518.477	7337.511	9497.666	8388.733
HUS1	338.1033	68.41968	86.79699	199.1006	77.98644	104.6745	105.4764	75.61623	288.2384
FAF2	5757.766	7613.851	8196.797	7897.318	9606.803	9727.107	8486.407	7692.103	3332.601
PDPN	16374.8	12482.62	15265.78	19371.52	10065.07	19363.05	18865.98	16696.51	12954.44
AMMECR1	889.7163	90.49715	220.2288	61.3909	124.5397	178.1674	156.3624	151.6051	58.9278
SAPS2	122.8109	49.13532	39.25914	43.99865	45.45991	53.3111	39.4552	37.61562	36.66504
NDRG2	742.1944	1279.787	400.7677	422.5687	432.0274	522.7482	351.3541	826.9036	1451.199
LOC92017	7104.192	3771.01	4573.04	4658.969	3386.333	2846.235	3583.158	2891.731	7353.425
SMAD1	67.60286	120.9136	586.2404	76.66984	62.84689	627.2767	42.86966	493.0018	61.4203
GRM1	34.70016	35.72356	83.64924	35.92596	59.54329	39.66265	38.66071	37.13355	37.87692
WDR78	77.33612	75.44167	54.82381	61.82554	48.21926	78.02588	65.22827	76.19887	89.25809
HSPA13	42.34286	50.70352	229.805	50.16618	83.81245	272.4486	85.99841	59.58421	42.12347
IDO2	114.2553	115.4792	567.0462	178.2829	61.55062	59.35438	322.3644	90.46832	193.9492
IRF5	47.14303	63.34346	38.14522	48.19472	45.34813	56.65205	88.28123	38.39584	82.67375
LBXCOR1	111.6822	46.38829	46.37017	116.5448	56.33301	63.03526	49.77181	68.28062	133.0539
GABRG1	73.12868	64.79836	49.30237	46.19311	46.09842	48.71321	37.85011	67.06889	42.5606
ATOX1	6797.583	10316.69	15256.31	11255.91	13324.76	13213	15476.6	17131.59	7344.557
MFSD6L	348.6149	152.7047	278.901	164.7642	104.9839	459.5041	74.92696	105.7283	532.3523
CT45A5	32.06646	36.37034	37.87628	37.41244	36.298	38.56886	32.68025	47.87179	34.94973
PITPNC1	52.78505	572.7747	344.2976	32.94932	297.3934	654.8296	262.0694	387.0636	36.22523
ZNF664	82.43634	298.1874	130.355	306.6061	82.57977	126.0814	104.7303	129.0721	68.05158
CYP2C9	96.52148	41.28461	49.30264	59.90572	146.1141	185.086	46.4162	81.14684	52.87838
CCL15	56.48777	43.15601	35.32989	49.29728	37.94936	38.25821	44.10853	35.97537	52.62598
PPP1R15B	609.8726	387.3424	237.069	318.8549	140.6858	203.9128	96.71082	132.8912	667.9054
PIB5PA	132.6035	139.3522	53.82842	128.1027	63.12182	78.54722	54.59126	64.55208	348.8298
SERPINA12	38.67205	35.20945	38.23906	52.10545	40.53054	52.99848	47.63339	34.05429	51.36154
TMEM37	56.14288	75.94955	46.41148	875.4108	51.39771	61.22789	127.204	226.5294	42.59671
TBRG1	37.81853	50.37904	40.39414	64.569	106.7392	44.63801	33.02057	38.12094	57.38498
PHOX2A	36.98621	37.32246	44.72948	55.25071	40.48055	50.48844	36.17987	38.49466	39.97728
LPAR4	1970.626	660.9211	833.5461	403.8288	553.405	604.5658	535.5854	285.4343	1267.898
LIPE	68.81813	68.34182	59.8266	62.84743	52.52672	63.57928	48.10955	46.59631	99.28071
STON2	39.88828	41.84883	207.2323	660.525	34.63221	364.5109	434.6961	421.6996	332.7349

C17orf46	303.681	183.0712	100.6158	211.7516	90.76043	156.4633	78.33648	118.4059	483.661
F10	1973.766	1664.471	344.9861	54.27819	513.1968	58.75932	149.0378	46.89995	2988.223
C18orf51	34.54154	46.12037	64.46909	49.87227	58.26218	115.1481	51.16781	82.70287	204.3632
TPD52L1	76.80156	59.41323	53.55483	90.0182	47.79447	110.6913	61.34921	57.98351	80.24155
MSL3	96.75845	60.61509	354.7328	60.37738	179.1483	586.2702	487.3076	439.8226	65.33372
SYT16	333.0861	88.49123	69.8915	160.6186	99.51601	129.8285	67.92931	73.36302	329.6229
BOLL	127.803	100.8229	55.23696	90.42422	63.50111	71.03664	47.39077	47.93342	115.2403
GALNT7	50.84492	51.30681	114.8252	38.93082	91.29824	171.3461	65.92064	58.24044	46.50975
KIAA0907	84.24609	64.13354	130.6416	61.04219	811.3711	481.96	609.4344	242.1891	60.26572
USP25	40.86576	56.06536	52.9977	227.2723	67.45724	62.37271	51.6142	50.23064	41.75122
LPO	44.87567	45.94022	49.07278	37.93376	39.14015	50.92253	34.45402	42.35153	46.28716
CENPQ	34.77847	33.35368	103.4668	357.9488	229.8816	33.06728	151.8735	113.3645	41.80832
DPEP1	197.9379	127.7027	71.24318	100.5221	59.91739	90.30056	61.81318	62.84285	278.8202
POU3F1	48.25287	44.04344	34.96349	47.1683	38.70356	45.26197	47.49228	34.93528	55.47672
EXOSC10	8620.129	10634.97	13335.62	11694.21	14021.19	11648.24	16239.7	10979.78	9659.659
MTR	239.0193	889.8044	1599.154	1470.928	1564.734	1953.409	1889.874	1210.345	710.7183
FAM84B	382.5201	1070.929	1865.063	3239.088	3388.466	2818.584	3372.973	2729.367	256.0784
NRM	328.2195	149.3963	1534.995	914.1119	1897.945	979.1735	2507.149	2431.425	184.4967
MZF1	656.1604	271.91	205.0629	305.1362	116.2738	170.7168	116.0203	49.72463	696.734
GRPEL1	384.0412	363.002	655.0854	621.2606	595.7049	288.6899	587.9393	500.8853	577.6742
CYorf15A	36.30012	782.2781	49.83757	572.8362	39.87554	37.78056	459.896	581.8071	56.10445
ASB17	327.1616	118.5581	69.11957	184.9439	62.33905	94.22977	65.46801	72.29698	292.5028
LOC44107(	136.3302	102.9631	77.69923	65.47678	83.18031	108.0627	83.08613	77.95936	160.269
KCNG1	61.34911	55.25156	189.5009	1124.119	56.1666	45.34826	47.80861	36.92564	53.91305
CPNE1	32.24698	1227.313	544.8265	46.20707	861.0033	2060.473	1065.634	319.6129	36.86584
OPN3	56.61516	57.06443	40.96538	42.16339	37.12374	46.22018	138.5223	42.26988	42.62322
ALCAM	3360.487	1668.891	1844.787	5238.856	1955.155	526.0807	2797.941	944.5679	568.3816
COLQ	63.18993	58.98049	42.49546	63.52518	46.61735	36.88319	36.43019	37.2527	57.09616
PPM1D	551.7153	194.9711	236.1387	42.1088	261.2975	327.1626	282.3362	163.6114	34.83595
CHST6	68.62478	46.33656	278.8459	61.57242	119.0436	50.6377	172.6366	51.09269	59.92189
ZNF207	17892.04	21115	19396.74	19383.79	19363.98	22922.21	20409.11	18185.52	20253.08
FBN2	2610.837	1920.676	1000.846	3700.507	1921.222	2288.356	3169.468	1267.522	1733.966
COX10	1250.926	965.0792	1335.683	1337.742	1627.271	1810.656	1683.796	1922.978	990.0627
NAT2	52.42683	40.73397	71.39448	50.94619	39.24054	57.63489	52.49411	59.02417	57.02077
ACVR2A	46.71638	127.9659	38.25513	48.34501	36.94384	75.61664	36.78201	39.59621	49.26805
INSR	363.5399	227.3586	104.4044	215.2115	119.2073	176.3974	88.08418	113.3928	426.5271
FBXL18	116.2863	125.4596	238.7671	82.37319	54.41432	106.1939	122.4232	60.632	103.1698
FAM166A	94.12666	56.57737	76.64821	66.534	59.3629	49.46769	49.71205	52.04198	54.27724
CYB5D1	128.856	119.8489	55.76822	138.5506	333.7885	824.9955	268.899	363.115	331.6073
CHST15	90.13276	54.74029	553.9313	75.62547	1248.627	1815.53	1357.208	376.27	94.23096
PDS5A	843.2866	1114.595	1009.336	344.33	305.5738	376.1046	587.2511	871.4338	3346.594
TSR2	6058.472	4915.586	8950.646	5061.275	9341.714	7587.499	8872.247	8351.689	45.5948
KCNA1	46.67339	47.37516	37.70033	40.34468	47.75836	35.09401	46.73894	36.84568	46.80511
CEACAM5	1685.502	342.3577	586.5544	42.36418	400.1572	588.4645	69.90025	319.2726	1136.371
PPP3CA	603.5405	308.6095	256.6512	339.7501	209.4922	188.7025	144.3505	193.9021	311.6899
C8orf47	252.7063	38.64288	50.61897	61.05486	58.12885	84.12325	63.27277	71.98263	177.1789
POLG2	65.50159	46.56174	148.3265	49.63816	294.9292	41.97153	140.4528	145.0163	101.8031

IRF2BP2	100.2836	3022.302	669.5684	2827.977	506.7688	795.0128	667.265	1106.052	3910.689
ZNF267	117.1262	153.247	60.43061	98.30917	63.64307	65.37495	55.6544	56.38075	97.97354
THRAP3	4089.463	2476.296	1631.308	3318.558	1091.313	1912.599	1467.104	606.7493	4316.634
GALNT3	1920.481	66.25105	1703.164	1914.688	1090.719	1230.995	901.1813	533.3958	107.1935
HSPB2	2006.169	1568.263	384.9819	882.3308	327.2882	161.5657	1628.737	1506.671	377.9136
CD276	52.047	89.50279	69.32068	2603.311	55.95395	504.9243	278.5998	69.57772	248.4681
GSG1L	45.89941	40.90882	34.80562	40.60664	34.26955	42.17332	40.21479	35.73932	61.43629
CDX2	180.9071	295.7437	93.61926	102.4144	89.08959	104.4461	74.42072	63.81142	212.3373
MED13	53.54822	346.9322	438.9643	392.5396	352.2431	323.8989	312.1714	544.1888	881.2405
BAI3	40.99046	43.30253	48.73767	55.07189	143.1764	40.95377	39.9674	46.69571	39.62403
C6orf222	263.982	127.9661	82.78868	96.66294	75.84518	105.2928	51.65408	70.61459	61.80784
SOX17	47.57013	829.5715	35.15151	39.92498	303.9148	48.94427	128.2121	53.41362	34.79712
ZNF592	248.1654	126.2291	77.2283	173.9714	107.1644	69.23557	74.089	66.97023	199.3424
GJA4	53.61621	397.6183	234.1377	655.6094	410.3192	257.9417	553.3303	500.4312	100.2194
TCBA1	41.99364	34.93417	52.18327	37.64369	38.92346	77.31697	39.60677	54.87772	41.40171
PIKFYVE	81.30552	1640.744	702.8638	64.64268	155.9601	406.4779	377.0047	163.3473	43.33469
C3orf21	2620.7	2523.044	2514.689	3312.2	2700.712	3873.791	3269.849	2349.719	1794.659
MFAP3L	46.22577	35.43715	38.68589	42.09314	58.01247	93.83828	57.23462	53.99524	35.84729
GPR68	56.09238	40.50365	749.7393	59.05412	1233.122	54.73981	1290.987	1182.448	48.70396
MEG3	40.34608	55.30382	40.85346	42.453	47.70275	75.78874	65.24883	67.50174	73.09152
RARB	36.32884	40.86442	38.21199	44.92981	40.57016	65.37068	32.83894	34.20082	43.57893
TMPRSS13	119.1766	107.6889	100.0467	129.1107	60.71555	102.4067	64.71497	73.6133	117.9259
MRPL13	2698.234	3391.069	4974.582	4235.935	4063.822	5305.362	6015.011	6181.288	1602.172
UQCRC1	85.69193	164.1964	1048.817	128.6125	3356.666	567.0139	2621.167	3256.767	145.6005
IARS2	1486.158	569.4632	840.3398	2424.028	1896.747	415.6692	1500.032	1294.148	41.14062
FBXL19	139.5889	106.8901	90.81802	164.9845	82.4099	60.63427	65.14436	81.40762	94.42422
GAPVD1	2915.615	2326.626	4794.747	2382.27	5081.622	5639.795	5010.167	4244.467	1652.209
GPR61	209.8304	172.5625	93.85135	214.5011	78.19762	142.0984	71.48214	105.8395	432.9395
GNGT2	271.6563	116.5308	285.2934	166.3643	161.192	121.4576	67.87473	71.0557	397.1852
CLIC4	903.3313	849.4557	687.4318	1432.664	462.9061	649.7231	643.6489	580.3098	815.0449
DKC1	4319.12	4327.901	5231.821	5905.25	5392.057	6032.921	5275.805	6339.037	3532.331
CACNA1H	97.24758	488.9194	476.3757	403.2429	494.2347	398.8616	797.4579	833.4409	168.6908
NF2	33.50325	2736.451	512.92	61.67426	472.5275	35.29778	535.8826	843.0159	34.97438
EMP2	183.1265	40.15815	85.50226	89.48097	91.08595	65.96246	54.5041	78.76471	78.2495
PKD1L1	130.9556	86.30509	76.45993	51.29322	57.45087	58.62471	53.08321	60.42081	149.8721
LOC731823	60.9283	34.50541	32.30917	33.57845	32.357	41.29745	90.641	32.55611	35.85291
RNASEL	4971.779	3628.359	3260.251	2853.249	2658.053	3950.198	2511.566	4077.909	1970.978
ODF2	358.199	218.7174	248.4488	193.8947	322.2491	121.6431	245.6868	607.0273	459.1523
EIF3I	239.3674	185.4885	396.1394	1071.155	400.5303	112.4828	652.8197	537.2432	293.4166
CCDC3	158.2595	110.0009	80.72326	180.1858	280.2635	117.7454	507.1989	176.3273	114.3584
UXT	97.6487	452.6906	554.6888	62.41544	878.1481	336.9692	735.1075	892.3491	69.77437
EIF2C1	174.914	177.2948	410.2294	127.7108	637.174	158.1966	517.236	575.0405	376.4621
SLC2A5	1376.765	232.5584	183.1134	232.326	67.0209	430.4207	286.3772	684.8235	430.7177
MAP3K7IP3	525.3268	701.6668	216.1148	666.637	165.7686	332.8483	191.1112	184.2214	901.8222
IL12A	37.84483	260.6161	70.12061	240.8688	35.20853	201.9113	49.57391	53.09303	54.81479
IMAA	1126.834	103.8679	69.55893	158.9293	100.478	70.15149	146.1897	50.43604	288.8822
UBE2J2	33.21172	61.0906	33.8512	50.75626	35.98916	59.2226	37.15226	45.40034	35.631

PRODH	65.93645	48.89168	821.7497	68.85378	1097.56	434.7736	953.6602	1477.623	40.47604
C19orf53	22447.57	20935.06	20564.26	21514.27	19861.67	23831.49	22004.55	25036.27	21671.59
C3orf23	38.778	43.93548	42.04435	41.79678	160.0434	34.95303	91.92177	142.1566	143.466
KCNF1	954.2617	650.1851	406.9619	485.9714	220.5254	549.6088	959.2073	1027.416	1067.748
FBXL4	35.61232	41.76219	52.40541	74.57487	47.42389	31.99305	41.58593	39.26677	37.58133
NPAS3	76.16872	58.87949	60.59051	81.40519	53.81845	40.11889	58.11164	51.78205	63.19885
ARPP-21	1950.845	3224.411	596.5687	1588.934	315.0334	1165.377	1348.113	2665.794	3141.442
PCDHGA11	137.5763	34.61931	59.48832	56.16634	57.34221	38.49365	52.92503	41.29745	39.32736
FBXW4	80.15274	737.9867	1894.939	69.5454	2978.438	1465.002	3274.132	2861.467	78.4716
MAP3K15	120.4943	68.43359	51.97207	97.16553	227.1788	103.5066	58.86216	82.9397	224.2633
MRPL44	1278.387	858.0603	1011.547	92.96535	1267.537	1334.385	1164.982	1035.013	178.7095
EHD3	171.6586	84.01236	172.5886	60.6909	52.81868	39.47648	59.57306	62.19965	201.5526
POTEA	51.26567	32.22439	37.36763	40.62749	39.28765	35.54935	34.65984	36.50381	40.33464
CKB	68.06599	58.66628	368.8654	273.3113	1547.451	306.4956	1185.944	355.3664	36.08846
GALM	99.06386	544.9992	231.6714	34.0801	122.906	49.8266	85.30602	140.9793	37.83449
HOMER2	118.5154	114.6055	451.5393	785.6625	310.2333	65.89324	490.1631	268.3247	65.5192
PC	37.94421	40.75324	1322.645	1218.281	2467.035	1417.079	1650.64	3436.16	46.3907
USP5	58.01278	1004.188	1669.952	997.659	2670.09	1845.197	2361.25	3158.343	125.9563
ZCCHC5	88.47369	66.48228	49.71741	111.0773	53.47279	52.78893	41.49178	67.03062	106.8822
CCL3	204.5385	163.9883	85.90761	162.6834	64.90504	66.99591	342.6528	108.4871	1880.72
CIR1	2064.611	2053.932	1513.883	1648.392	591.2465	1634.401	1161.035	1493.97	1515.79
LPIN1	57.02268	40.03027	782.3961	878.7545	1813.859	31.68157	670.2519	1189.503	53.05442
PHLDA2	1739.799	1736.121	889.7579	1197.966	871.3517	1230.86	413.1706	907.2233	2377.366
RNASET2	6379.787	5332.731	7394.45	5073.475	5507.692	8418.74	5177.879	4525.197	6042.602
ECHDC2	17179.3	15954.33	21348.12	11856.23	22779.23	22127.85	20849.16	16734.61	16633.17
MKRN1	4740.446	5615.595	7022.005	5127.479	7377.324	7242.408	7444.106	8148.523	4004.706
MED30	44.51528	33.20351	42.75498	35.30024	34.44693	32.92896	36.69912	37.1978	54.22065
HMX2	116.4612	138.056	72.86584	117.9423	67.23796	78.6832	60.4882	69.3153	133.5406
PCMT1	20402.6	22410.64	21866.18	22740.8	20740.76	20875.72	21542.56	22932.37	21470.76
DLGAP3	44.41628	54.04988	33.9977	45.87679	39.1715	44.0044	40.71255	43.11594	38.16491
EDA	192.3968	117.8161	52.55066	60.01058	62.08682	62.75481	59.85272	45.4126	298.1034
EPB49	1470.709	397.4471	901.8897	336.5581	1196.832	511.9533	684.3624	357.152	95.18537
C18orf10	2155.697	109.267	318.6222	655.6779	174.2508	603.6214	77.15543	367.1298	54.21579
ACTA2	14973.02	14253.88	13120.93	14575.85	23432.79	17395.39	17853.28	16580.68	10728.46
MADD	86.12873	338.0751	42.24274	43.60602	93.53706	50.79335	72.26941	54.8708	68.11201
REPIN1	363.7289	230.5405	114.1187	216.5333	114.9987	172.3	153.9433	158.9316	436.4166
CARD14	321.3789	149.5596	76.48075	116.3085	76.64297	142.5303	64.75481	121.7656	387.4376
AVPI1	16045.61	20558.65	20126.29	22358.78	15889.95	16064.75	18168.58	15712.5	14235.01
UPF3A	873.6438	35.1288	376.638	299.1975	366.169	455.1766	252.7635	269.8048	807.8901
TRIM35	35.39818	36.3947	39.5331	46.40646	41.38844	45.05946	39.23258	40.19722	34.92639
F12	1593.2	2372.952	4731.344	1755.967	5972.784	2596.291	3540.194	8042.855	1352.753
PCK2	44.45895	43.97813	474.966	907.0358	851.2899	423.0886	689.7199	745.6622	63.97141
DUSP22	62.65804	400.3733	271.2206	77.37454	1004.328	285.2336	698.7872	595.7955	51.08976
ANKRD21	36.52852	37.52934	34.16636	35.71682	33.51932	38.54163	41.78653	33.92762	59.74948
UTS2	217.4876	115.4067	94.99012	97.57868	74.8813	106.1817	64.45657	70.25659	69.06976
TAF1C	13446.34	13560.42	14055.55	13381.9	14611.29	13541.78	13774.04	12871.02	14253.13
PSRC1	3125.596	1596.531	4006.923	4971.779	3525.846	3567.722	5311.485	4017.65	117.1886

EYA4	95.33414	64.60554	60.30628	68.52142	131.8678	57.67828	77.31474	62.82647	80.37253
ITGB1BP2	132.992	204.4587	84.42074	189.3029	77.17749	92.05098	80.35398	115.9818	491.9651
MLF2	48.62134	2156.578	1258.375	1090.216	2044.755	64.61113	2214.844	1864.298	2814.087
PID1	44.53279	44.57626	120.0736	52.09845	60.76615	118.9042	66.15845	90.65862	113.27
ZNF613	6695.059	4848.821	5323.767	5514.857	4767.899	5083.057	5922.614	5640.671	6241.492
LMX1A	38.56343	47.85633	38.88448	37.3139	234.469	39.13866	34.26106	96.30736	44.66529
THUMPD3	66.10218	106.2391	69.14426	93.80489	116.557	253.5398	171.4269	45.36209	93.5961
C10orf78	76.69854	47.9126	357.9776	40.46069	157.1783	42.61406	283.2354	223.7063	55.19053
MND1	51.54355	379.9817	213.0803	406.1704	127.5094	325.8268	94.94869	425.28	47.14239
MTA1	363.9335	171.9102	104.4581	184.4524	151.9997	103.7413	244.5896	105.0702	484.4477
TRMT2A	363.6071	207.9902	124.6948	186.5981	182.9079	246.761	144.8778	190.8273	530.4424
PROS1	2915.306	4021.407	4608.846	3289.598	9550.071	7649.971	6325.771	4748.153	5102.237
GPR52	49.84824	39.98794	129.06	46.17971	52.42408	43.54378	70.12544	49.74646	60.5976
SAMM50	35.81163	976.7836	4422.285	584.2421	5655.32	2010.837	6444.347	4861.353	39.5811
DSEL	40.39921	33.02258	43.58689	37.34682	34.49211	36.47585	34.68827	34.66184	35.0885
FLJ44076	66.689	49.95406	65.37759	82.71279	79.60702	119.4471	73.63228	65.76749	57.75928
LOC64246(	50.38882	39.83074	38.88073	37.88432	40.46928	34.53312	34.22701	38.32903	42.81992
TOB1	62.19022	2512.638	3273.691	1556.492	4039.991	2055.687	2485.307	2443.053	36.3696
EPHA4	115.2611	80.19675	58.23667	394.3796	63.35686	356.7732	148.1111	68.90437	45.57288
DOLPP1	45.72923	313.6885	254.8246	50.34272	434.0968	417.2206	320.3153	575.0056	48.77739
NR2F6	112.7465	68.93967	586.4443	77.01118	553.6122	258.03	556.4984	573.1584	68.11181
NEURL	89.23772	84.97695	74.81588	78.87177	49.76156	64.53065	55.64768	53.78195	62.62346
HOXD4	325.7452	142.2475	95.46435	156.8262	251.1622	141.5418	82.88322	90.54975	506.4294
DLX1	156.6595	113.4927	74.30202	136.0737	839.1662	227.1031	528.8436	196.0908	481.2631
CD86	32.85448	35.18848	36.37346	39.9409	34.85658	43.80964	37.27934	35.20335	52.07938
PTPRB	156.8593	57.63784	263.9022	53.69079	312.0387	56.86093	289.3662	63.89868	97.62684
TNFRSF10(	372.6402	186.83	88.69691	218.8608	86.03162	128.7386	86.31942	99.98687	404.9873
KRT73	6047.223	466.9677	1377.895	2375.21	1542.646	1439.595	837.5094	2011.753	2273.313
P4HTM	2362.538	1736.649	315.8805	751.7454	276.3867	568.3926	339.5025	234.1062	2592.198
C4orf18	39.81843	38.86855	36.11016	48.08356	43.82928	46.6898	51.20026	35.65347	35.68386
ATF4	27724.84	30204.34	26701.88	29112.42	26099.15	26722.53	24971.36	25575.82	29214.78
ZNF615	124.3222	85.40675	71.68029	682.8229	516.1326	387.4376	680.5289	201.8451	166.7878
AKR1B10	567.9927	64.83272	299.9687	70.00834	761.5295	48.08209	214.7082	216.8099	35.00122
CHCHD1	49.30756	48.16989	152.3961	80.87539	562.2252	319.6844	262.7642	190.2191	47.77431
ECE1	3698.287	1986.815	468.3294	2721.552	870.6678	1310.589	762.7369	490.1144	3435.534
SMPD2	157.2958	136.8403	78.17184	128.5787	72.96215	116.9574	58.28635	94.19595	138.3436
RNF8	315.901	159.9338	94.23613	184.4232	87.02995	141.3797	79.77393	86.99626	442.0116
ATP5F1	59.98355	76.13777	120.4749	61.42838	126.2969	158.0188	126.2043	39.80557	75.71875
FGF12	52.20792	65.21429	44.54557	72.85072	54.89667	49.4531	238.1595	753.6105	96.20197
LRIT3	92.96725	76.16556	967.8893	99.57565	657.5773	965.087	375.2916	62.63322	54.116
FAM55B	53.60118	34.25684	43.22422	72.11835	39.4002	40.75282	36.22583	47.98777	36.12106
CXCL16	47.58908	37.63534	49.04283	37.38749	33.46999	38.41352	35.15006	64.68862	68.99156
MYNN	5708.834	5622.904	3406.06	3908.023	3194.604	4408.521	2870.591	3469.766	5518.568
ZC3H12A	40.22061	2245.289	462.0732	605.8463	4442.389	1603.933	1987.896	1510.694	1712.772
STK32C	115.2343	82.82914	130.9037	128.995	74.34819	150.2823	62.92477	63.83238	83.47243
BMX	149.7408	92.17441	52.51585	119.0665	77.19599	81.70628	53.90053	38.48657	85.02495
PCDHA2	32.47816	31.54697	37.48805	42.57189	31.99499	42.48079	36.41901	49.71014	40.83043

MAP3K7IP2	827.8332	830.189	430.8963	922.477	324.3343	310.4838	275.641	430.986	1023.617
C3orf60	52.3333	36.80268	43.21362	47.61583	56.61679	51.79556	44.25565	57.38129	142.6643
JAM2	126.6679	104.4433	371.6696	83.89281	944.1909	108.5374	348.6281	652.2177	340.8862
PIGY	682.5789	40.3409	141.3091	396.3862	105.8676	68.86845	235.4034	227.3018	77.78854
ZP3	62.0662	94.75057	57.71973	139.6969	60.38722	59.1243	60.66924	74.32014	65.56186
SLC38A3	47.65951	46.02004	37.96755	55.676	91.97215	52.53939	44.28313	50.58925	93.59454
CRYZ	53.35546	219.5703	142.5275	300.3687	287.3371	333.3377	225.6162	119.2058	39.51264
ZAK	43.60824	884.6684	542.9225	1056.563	681.2798	533.6104	850.059	905.6033	37.70269
CUL7	59.17593	114.4568	82.4617	49.75264	140.9195	196.5186	128.4031	109.6098	59.65731
ADAMTS8	162.81	66.64732	64.71246	100.1769	92.30305	76.82088	48.27718	98.16823	109.8018
TMED7	511.25	370.3476	218.8215	385.6805	232.1637	399.6415	188.6643	247.7041	74.7873
FBXO3	223.2876	108.1871	45.30257	118.7671	73.07673	81.03975	65.69366	61.47552	238.1919
PTPRCAP	6753.63	7018.292	9560.135	2875.411	3085.099	7400.391	3782.046	7939.293	5342.402
SMARCA2	39.16226	198.5007	115.9184	42.96491	49.75619	38.57987	61.51856	74.14868	112.4664
QDPR	235.574	153.7666	160.4074	117.1283	140.9962	82.72215	176.584	171.9804	193.7683
RPL11	9736.045	8796.457	9454.584	10456.27	8288.407	7599.748	7917.137	11164.27	9257.367
SPPL2B	1415.547	46.82227	684.0325	44.16792	902.8047	427.2737	705.1772	415.8769	43.88363
PRAMEF8	119.5744	85.46935	71.61292	127.568	85.20665	137.825	62.43859	65.94102	245.159
TRIM55	47.69128	37.87229	41.16084	38.64589	35.16044	31.42441	32.2275	35.22465	54.03796
TCF7L2	49.20448	54.14495	299.332	73.24341	397.3828	591.7622	346.6402	325.5071	976.989
DNAJA1	2298.182	1910.256	3036.816	1831.645	3043.753	3985.078	3275.584	2091.178	1702.863
DPYSL2	10279.83	7800.789	7104.839	8489.863	8953.348	7445.846	7126.648	8308.701	7937.863
PDXK	12038.42	14550.42	17949.15	17373.04	20923.47	16357.41	20387.7	15050.69	14221.38
LOC389834	863.5156	281.1825	138.3394	46.25458	85.44204	239.7626	72.96896	107.1866	605.4377
FBLN1	13777.47	14529.74	10723.05	10209.29	12241.95	11361.43	8978.952	11713.4	14053.18
NBR1	37.1605	34.44447	38.08991	67.24017	197.8438	48.03305	255.6954	80.55982	59.42115
GIPC2	55.88335	44.81926	39.07825	62.12548	150.162	47.95078	98.65501	38.74596	78.25082
MYO9B	1314.123	694.7773	2551.082	1539.984	2469.455	758.4806	2023.118	2774.599	1979.099
GPR110	303.4137	31.00674	78.3213	102.9398	53.03439	88.63728	43.32274	61.44836	58.52551
FMO6P	368.3955	219.8447	118.4979	251.6786	130.1383	157.4768	112.911	106.5926	507.2707
RG9MTD1	36.0895	266.0399	159.2762	291.3901	207.7599	209.3576	134.1028	198.6848	36.70628
TFIP11	1204.61	483.6985	868.1724	826.9761	516.6257	809.5889	605.0985	743.3447	1946
SEMA4B	767.1957	57.73179	750.1465	411.8227	958.2008	1113.491	1589.755	752.5383	75.35522
BCAR1	36.56445	268.1863	39.55162	43.7479	40.64414	46.83866	65.34603	43.87929	39.36794
C7orf41	10176.67	8310.671	4874.42	8155.706	6038.835	5579.336	7681.802	6445.273	9519.746
PPAP2B	116.7524	80.8512	54.5783	51.70774	69.36686	67.37083	53.46629	51.02276	149.8395
CDH11	56.03527	53.30747	476.3257	1399.875	1980.195	2115.33	1210.692	682.7593	38.66128
PKM2	8890.09	6835.908	4538.275	9290.52	2062.322	4301.63	3411.216	3791.531	7079.866
OR6B2	39.62333	40.6338	46.28812	41.01839	35.58377	47.37578	37.88346	31.98101	42.54251
MBP	36.18311	43.66967	3236.362	37.58334	4353.172	2187.763	5094.136	2948.886	56.80685
SP110	3283.249	5190.209	6170.563	5746.646	2095.346	5326.238	7535.623	5892.95	3476.741
SLC20A1	65.15732	59.91291	525.5455	633.0065	549.5947	62.48912	1053.259	422.672	60.04429
LOC645447	58.78309	48.25739	38.5779	50.39224	44.33671	45.26806	35.48188	37.38457	57.19276
IGSF11	160.8294	82.20501	67.13975	70.30785	58.85817	58.21329	45.96428	54.00871	68.57282
C10orf4	128.7932	41.76672	44.10392	70.16615	48.38319	54.89133	49.49006	65.84978	78.99745
C10orf57	722.809	300.665	312.7712	485.5954	311.6466	316.1133	322.7305	185.6301	870.4789
PRSS12	362.4277	105.7743	68.04925	128.3049	72.53922	68.18102	57.66302	56.45242	46.52639

KCTD5	190.9382	460.8221	860.1938	665.3467	1233.912	1120.664	1372.63	789.8621	132.4414
CCDC109A	48.19036	58.32286	119.0876	297.7144	298.6812	336.1563	408.4495	320.4855	35.25633
COL10A1	38.59031	232.7327	39.80071	35.7711	35.61856	37.00324	50.62342	37.66372	76.18316
CT45A4	53.70806	98.75938	50.99421	51.96835	61.30291	50.21725	60.17603	46.11155	88.46835
CA5B	586.4209	256.4128	242.807	236.6796	300.5209	238.7732	200.387	129.9061	45.77878
C1orf159	6476.821	4436.727	2928.964	4881.772	1779.007	4966.525	2641.89	4293.231	5071.447
SSH2	68.83142	44.87975	51.658	117.7848	183.861	744.5412	279.503	64.63168	203.6401
FBXO8	128.1312	93.03529	74.13528	104.3578	71.72851	89.93994	76.68619	105.0078	86.82812
BRUNOL5	52.71346	39.81363	40.87629	48.3321	43.57159	43.09752	46.04287	45.09787	56.1335
FGL1	74.11973	60.19851	63.38189	88.84541	57.16208	56.10005	55.35125	65.95772	54.64963
MFI2	33.33281	48.27678	35.2659	456.7388	34.80454	39.55004	33.34588	40.56638	48.8813
MPST	502.9663	282.2	174.9026	342.1858	171.2428	228.1873	152.2631	254.4518	687.3977
MTFMT	32.75684	392.0132	366.7567	380.684	177.2328	321.5282	296.7749	251.5562	59.80908
POMC	51.79607	55.32092	46.19622	69.30358	93.9399	35.92892	35.80727	135.5983	107.035
EIF4G1	38.46471	45.61943	49.38239	36.10415	52.7551	43.10456	78.12159	124.4132	40.48362
TWIST1	180.1274	43.29559	230.0614	76.88779	296.3601	417.8591	102.7128	220.0604	46.2396
SLC26A11	38.01477	63.93692	37.43301	37.42578	54.1828	51.91046	42.9499	159.9711	78.4544
ZNF562	63.30644	63.19766	254.1572	271.6939	165.4027	201.0378	182.0614	168.5079	45.29007
RPL32P3	41.06429	37.99767	48.90106	35.79638	41.38041	50.14487	35.02754	55.03815	126.3323
WBP2NL	49.52967	44.08555	38.37573	54.06488	67.36297	37.01729	54.94575	51.39385	78.52344
RCP9	964.8894	469.107	162.6913	507.2932	69.73302	106.1719	235.6197	490.8946	47.08072
EP400	260.7153	222.6919	582.0229	202.1904	688.5156	118.3399	747.9613	281.8882	352.0826
RBM15B	38.08763	32.88793	35.91647	41.76844	174.2397	548.1092	343.2319	37.2681	34.96675
CHCHD3	914.4601	632.4848	178.6533	367.8609	160.991	174.1812	125.887	256.5932	1133.043
CMKLR1	72.00793	1490.403	375.8321	58.45389	161.0486	701.2908	103.8008	245.0778	60.8028
DPP9	1920.444	1522.974	868.9384	656.3051	1556.096	74.40138	1197.837	1027.83	57.9414
WDR62	47.48786	45.50387	35.97063	34.38814	35.09753	43.50164	177.772	41.62785	63.54472
AP2A1	39.9624	58.07314	66.98684	62.6908	49.44867	40.44115	43.22728	41.27198	57.67924
TSEN34	1023.731	813.0614	545.8721	759.5698	724.3895	691.1332	747.3871	756.5819	1292.124
ZNF32	105.2196	69.45983	58.58528	76.16036	71.86486	44.65411	57.81363	63.70444	80.50101
F2RL1	36.42868	398.7631	37.41302	32.74288	112.1764	33.80668	78.73264	82.5071	61.98699
AFF1	1809.174	1400.863	792.3518	1171.21	464.8803	747.7661	372.1829	453.6273	1899.75
PCDH12	204.0154	114.6151	250.5086	115.581	232.0574	372.8862	369.3196	74.74518	2470.124
ZBTB33	1636.597	2305.945	4280.391	2222.651	4748.312	5196.044	3425.753	3943.524	1221.626
NRXN1	54.78009	63.106	54.67961	47.08843	126.3024	39.61037	33.80154	129.2641	31.74456
IFT52	41.82195	63.51988	41.82818	49.29913	41.86616	58.48991	52.64877	46.62652	40.42629
THSD3	68.09563	65.67728	66.19084	81.54682	52.97267	49.49328	56.92725	52.20742	85.48168
SNORD100	1507.08	2621.493	689.7167	1382.869	386.1537	902.4609	374.935	672.6929	1884.244
TPD52L3	89.91748	77.75068	82.43076	90.77444	70.37592	122.6511	67.66553	54.26916	98.97203
S100A7A	5829.076	11823.13	7831.285	9173.728	12988.25	11352.5	10524.71	11147.96	13031.37
KLF11	106.3456	90.449	52.07167	115.5501	168.972	45.36612	296.084	336.9986	59.67682
PCBP1	64.67868	331.429	324.6406	64.33761	986.8856	293.7653	788.7567	337.5081	89.84479
ZBTB10	575.5616	395.4939	72.13454	146.9282	164.6752	357.6601	66.09519	68.10641	1080.123
LASS1	141.0969	94.31408	68.88371	149.1443	62.68046	103.9646	74.38799	103.663	268.5991
PKDREJ	161.4347	80.03791	66.07793	121.4451	69.50753	88.30296	55.13529	93.37574	85.23202
CLEC9A	112.3386	67.62537	76.84582	69.41492	65.40513	91.52565	48.38783	56.07219	104.1376
HMGA1	51.80008	55.50139	52.91367	73.90499	45.24075	49.8249	35.7822	50.68449	261.9618

AIMP2	70.55364	381.4204	4968.54	1759.394	7440.457	3510.444	8388.539	6799.328	110.5962
EPS8L3	134.8449	67.70774	66.8753	85.80787	56.68835	234.6969	55.63247	98.01794	64.54974
TRAPPC2	40.21867	38.6521	158.5507	48.9379	418.5668	488.8463	160.0618	151.6093	38.56185
ZNF80	210.5364	109.7165	72.99764	120.4218	74.14391	95.39247	53.43827	56.40758	288.0562
RHOC	6348.633	5040.74	4647.656	6838.017	3190.031	3751.63	4194.32	6016.705	7588.915
C18orf34	64.66551	120.8138	40.80836	39.71833	44.49792	195.6507	94.98664	152.024	43.97536
ADRBK1	38.64367	38.40303	40.69038	32.57191	38.93	32.41027	45.17619	38.17643	40.74813
IFITM2	5131.805	6411.673	10987.48	5159.384	11029.91	9116.083	11441.75	9548.741	8029.028
SMR3B	42.94424	35.83352	49.00147	37.15688	41.19823	47.72793	38.82149	35.30631	45.18372
FAM83C	937.3494	1333.926	776.3832	1367.137	1138.627	430.0283	613.5253	779.2279	2367.659
SLA2	109.5033	58.34809	149.1635	81.21462	223.8291	522.981	667.8489	68.34977	65.17957
ARHGAP6	38.69703	39.14346	49.83406	50.01133	41.17303	54.02086	51.3787	50.43197	67.68615
BDH2	41.7819	41.81749	62.06245	79.66084	39.77503	182.9642	69.34814	36.3399	61.17044
CD97	114.5304	443.5697	506.7586	510.277	1007.561	251.9707	1238.744	1161.944	39.18148
FBXL5	40.42955	60.09279	723.6584	41.96288	271.6287	287.8938	514.8959	165.1342	44.10361
TBCD	77.79142	61.90677	69.14106	69.50713	38.38195	55.02148	151.4974	302.2632	66.74856
GNG12	5205.396	3938.52	2967.173	7305.393	1460.527	2571.347	1550.997	2826.595	7052.082
GEMIN7	41.83053	47.13255	50.77029	54.1091	54.01486	40.92634	152.4541	41.39631	38.20766
DDN	153.03	393.485	435.0369	153.0673	238.7733	812.7825	2405.552	2435.261	137.5578
MECR	424.2008	236.4548	111.5892	271.159	108.7128	503.5917	98.11057	385.7213	542.2512
OR1A1	75.13998	42.29631	38.05546	59.78634	46.76807	41.59156	37.9049	37.67431	44.67314
C5orf5	581.7342	474.3178	2758.068	580.5734	2140.063	2333.7	2088.225	1735.717	75.66608
LRAT	98.2975	47.90947	56.24593	68.8885	59.55474	395.7928	165.6909	78.68517	60.2573
CACNB4	152.2617	94.35883	80.74297	142.266	73.36404	96.758	67.50948	73.05196	233.3092
RPL28	165.2271	94.53954	189.758	138.5255	487.7928	76.29372	318.6755	186.8286	185.7931
BRWD1	196.7368	199.3458	280.1436	834.4135	572.1161	498.1561	651.6454	459.5723	403.1023
KISS1R	42.1053	143.8293	45.67157	68.35146	56.96898	45.65643	36.28793	33.32875	40.46696
MOCOS	107.4207	67.07205	56.96806	78.78362	362.0692	81.81753	482.5716	236.2126	78.08639
AGXT2L2	230.935	173.1044	158.1839	222.8047	148.0401	195.339	109.3802	140.5262	737.765
LOC34015(	37.17711	232.9846	240.4707	65.86826	423.4019	54.04332	270.1826	247.3785	67.4275
WFDC3	42.29892	57.38395	45.49113	47.76157	36.00418	35.39833	39.71765	41.61905	41.51584
AKAP14	80.42778	1393.265	38.57887	47.26518	127.9455	48.81734	113.7745	349.6018	46.63897
PGM2	581.371	74.00541	302.3104	49.82097	411.4808	237.9354	348.1388	121.605	33.78953
C1orf83	255.4296	165.683	182.3334	501.9279	79.44941	113.8351	80.0508	103.8111	260.4034
HSPC159	2262.802	3903.773	2749.287	1912.747	5791.228	2799.13	1977.088	3974.397	3639.706
PTH2	75.42512	114.9876	104.9394	109.4658	53.19757	123.2023	57.79036	63.1327	313.2823
ADAMTS1	215.6246	462.4789	685.3612	185.7155	237.5972	272.947	184.3384	223.5762	1992.669
SLC6A6	47.14115	62.5305	1741.864	764.9629	1297.691	1311.169	1646.892	1359.146	1904.68
BHLHA15	152.0124	1670.871	61.39441	130.4859	244.5294	97.4452	54.79162	76.92163	264.7499
CYP3A43	71.61335	85.45365	55.80344	70.63947	195.6215	73.27404	50.91336	51.99361	130.0929
YIPF3	7390.951	5946.25	5481.376	7420.704	6165.859	8910.786	7485.922	7866.904	5865.201
PRSSL1	39.51431	69.30811	58.80506	64.85365	50.03479	41.97084	51.69201	56.62008	217.2742
ISLR	58.69395	46.38856	46.95755	57.88128	122.887	34.89485	38.34544	39.76738	46.88291
SLC10A4	34.36679	35.99627	35.37095	34.7726	56.94918	38.55917	39.30053	53.27352	53.16646
ABI3BP	1612.571	979.729	1565.527	508.7715	1256.026	822.7814	865.5042	776.4547	984.9828
PAG1	2231.259	46.16535	54.53926	37.24177	47.47908	49.50244	33.5657	40.90742	61.0004
HYOU1	50.74477	814.4244	455.8671	35.88321	190.0223	49.37087	265.0731	63.58828	88.16993

C5orf4	56.15964	59.07784	46.56283	84.65608	61.24478	55.15399	51.60451	60.19528	46.4947
PBK	908.358	897.6177	1289.68	669.7916	940.6588	1302.296	834.3807	1098.108	894.4033
SNX5	193.7336	146.4252	82.9269	131.1535	71.49849	86.14436	74.59583	77.3147	53.07612
SLC46A1	374.1164	176.3699	101.0194	123.2592	85.4117	115.7701	76.98103	70.16592	311.207
GATAD2A	8243.562	8816.106	10290.82	11072.73	13159.43	10260.74	12556.38	10381.7	5193.739
INA	54.97878	45.46563	41.02177	48.41029	152.4794	45.75229	45.06815	40.12309	48.36562
HDAC9	41.76282	52.57518	36.02289	40.07835	39.8692	34.40513	47.41626	51.20486	40.08875
HSP90AB1	52.46733	524.0129	42.4536	1221.632	774.8755	920.2275	756.197	931.4338	59.4344
GPR149	148.3789	176.7789	90.63974	155.203	301.0705	99.61877	62.3238	101.6573	223.6246
KIF6	170.8495	142.6054	86.46214	61.97766	72.64572	93.63152	59.62099	95.28452	204.6165
EXO1	94.41913	264.3446	150.4364	960.5688	226.217	280.3297	197.3547	289.6844	48.74667
RHOBTB1	207.4802	124.8176	61.44724	139.6303	73.84775	117.1148	62.69942	91.79138	274.1252
C22orf26	273.309	290.5735	149.743	159.9867	71.10523	77.25912	122.6886	133.6663	311.0215
ZNF81	45.0466	54.38385	248.8386	34.31141	40.38935	47.31924	181.2616	186.6698	33.66487
CASP8	849.7747	767.4698	451.7307	780.7095	285.9971	238.9232	445.5775	575.5241	49.62757
GALNTL4	93.3655	48.40222	44.51808	70.38872	352.4385	49.36606	144.9068	43.60796	42.16383
CDV3	3875.342	3446.374	2655.01	3287.841	2398.72	1717.557	1807.784	2772.97	3803.424
HSD17B4	1500.507	1573.591	1877.185	465.6216	1814.382	1197.753	1162.026	1713.87	2077.174
PPOX	53.12975	2435.941	4317.521	1351.603	6074.271	1780.559	4107.572	3640.695	154.3752
ZNF426	649.1733	39.63784	34.09806	476.847	124.1112	198.2455	60.96739	84.16455	48.42719
GIP	67.24073	68.09485	52.0909	57.88409	55.84504	48.22084	46.68245	57.34077	117.2322
ARHGAP11	35.79068	43.68296	32.33587	40.52325	34.0438	48.67057	35.54416	34.42411	58.65981
HLTF	772.4552	229.2897	129.7151	257.6408	109.2744	187.8678	161.9154	189.6931	453.5602
CST2	48.53327	68.99527	40.8864	42.47892	47.78889	53.86157	102.9688	64.8374	159.0466
C14orf173	49.7542	47.0814	80.02499	34.12694	39.2893	179.6052	34.38451	83.6478	44.91505
LIMD2	937.3156	601.0887	719.097	88.78923	62.23766	268.0592	85.90269	175.775	1167.937
EFEMP1	3985.419	61.0593	41.88305	56.84374	258.8107	429.8287	529.4546	43.51796	2264.115
SIM2	68.87877	97.62576	468.4227	385.2317	43.42329	55.45122	440.3181	56.10484	55.09249
GK5	5030.664	4150.006	1942.69	4185.159	2950.676	4248.369	1870.541	3295.636	4762.729
MAP3K13	36.17461	48.74011	174.0808	47.03763	49.34666	241.8398	153.1123	251.5113	96.09682
FAM57A	56.71267	48.07865	34.2679	40.50928	141.9304	39.94764	76.76646	33.46383	1493.456
GTF2I	2195.516	3257.185	1724.367	87.90215	1545.856	924.1738	1467.032	1672.161	4498.484
KHSRP	14876.76	14387.16	12054.05	15036.3	10009.27	12051.34	8781.317	12135.79	15611.03
RBM24	282.7014	201.6855	163.577	187.281	114.4864	142.3729	169.9627	213.4006	991.4465
MANBAL	146.6582	62.17847	568.515	114.2067	874.1212	120.9864	767.9292	644.2839	254.6846
STBD1	403.0114	246.0101	170.9599	263.3455	229.67	199.295	143.3895	251.0805	500.8915
HRH1	354.5998	162.9499	94.38538	207.5305	101.0985	131.189	87.07516	120.8385	364.4511
MAT2B	201.5569	132.3882	69.00819	219.3367	57.87391	93.69978	118.5492	57.11009	168.8072
NUP205	1796.877	796.0597	2334.744	1165.038	2745.705	2122.123	2464.199	1012.235	1049.56
POLR3F	1058.931	1785.049	1396.134	1385.299	1474.991	1792.177	1526.399	2063.267	1465.658
LOC374491	34.26291	35.66833	37.55516	46.75675	42.7234	41.91772	43.24298	43.98635	41.36667
C11orf40	56.15505	54.72821	46.45019	50.3455	38.52785	37.75457	38.27185	36.6831	59.22246
DEFB134	46.72098	32.53042	43.34669	40.63414	33.84791	42.85167	33.74323	44.39209	53.98709
ATIC	2604.606	414.2327	3466.69	1591.519	4973.472	2678.245	5810.396	7341.344	812.4404
SSTR1	401.2038	231.6436	111.9974	260.9001	78.85215	130.1137	89.31473	120.1879	534.5747
TKTL1	846.973	594.2913	326.9925	1158.555	239.3587	450.1443	471.1018	507.4698	1108.769
C11orf53	190.8927	117.1296	67.85372	107.5959	70.54548	128.3499	63.85754	77.89864	292.5714

IKZF5	189.5654	439.4704	163.8508	470.7425	117.2713	250.4441	49.33151	89.42652	87.01367
PKIA	69.29618	40.61869	43.10997	43.59348	44.63918	37.32626	50.45956	36.42508	40.9176
ABCD3	69.3327	382.7102	66.36898	154.2415	72.32854	101.3984	93.83802	47.69573	106.7477
H2AFY	34.57777	1074.293	39.97412	1029.28	231.9102	615.7427	233.3066	346.6279	33.2936
BIRC7	51.51341	80.2925	71.13732	114.3988	64.25683	86.69316	451.4487	73.41261	80.69
CTSD	533.0934	794.561	439.6818	1094.516	496.3326	871.3822	352.1017	954.4697	794.9492
TEAD1	53.88301	46.82442	238.3044	892.3473	88.64234	232.3596	172.1015	123.3225	47.74647
COL8A2	349.3277	197.7918	98.51269	363.7207	125.6467	233.7439	132.4812	93.04932	489.5858
CCHCR1	244.228	177.3455	159.6537	150.9428	178.4591	147.703	97.71571	240.4139	162.0661
OAS2	22705.25	27617.83	27104.44	27629.06	15117.38	29458.33	29783.12	29352.15	21393.72
ZNF225	797.3422	474.101	502.1087	521.4151	128.8835	195.6401	267.3275	423.4418	137.4227
RBAK	61.00188	99.8683	39.76926	66.73206	295.5032	48.17398	145.679	56.80862	67.93193
SORBS2	145.4269	94.18145	45.94036	82.5001	46.09789	70.53346	47.63787	44.91878	244.2657
S100A7L2	44.79972	46.15377	46.9557	36.39371	33.54604	38.55809	40.95647	34.54087	34.21529
FLJ37543	213.3079	60.21141	55.91506	81.28529	62.86734	101.8563	56.1844	48.56491	48.57012
CWC22	734.8038	549.3992	187.5074	372.7716	203.3426	292.8999	319.1184	230.6186	1171.429
RALGPS1	153.8718	278.8814	2228.556	137.2644	3887.461	3733.473	2967.517	2080.359	84.28996
GCOM1	62.95279	400.7035	299.4287	486.1949	421.362	258.3898	210.0235	166.7577	457.7887
NFKBIB	43.5883	65.87659	155.9429	53.95816	98.2434	57.51683	44.65381	75.95057	49.21764
BIN1	2502.15	408.9584	972.6714	235.6847	385.6014	358.5943	1536.817	2926.516	577.9975
DDX42	39.91367	41.32262	46.19387	39.81837	48.32618	40.22416	43.72965	35.03357	46.71324
IL10	75.68683	36.50432	33.34673	38.09019	41.17972	175.0398	39.47506	49.03136	53.38587
GPR125	69.39778	69.48108	39.76151	86.11267	66.34346	54.01576	48.31699	61.88562	167.3862
EMX1	78.68264	135.5849	40.3188	72.37521	48.12115	49.40357	53.20338	50.49141	82.71107
ATP11A	60.14645	94.7525	46.14158	54.88426	50.16639	45.45091	114.389	59.3153	209.0909
CHAF1B	51.00745	444.5989	3186.265	35.62961	1778.178	3299.907	2479.053	2017.061	41.11908
HSPB1	10248.78	12715.43	19817.51	12458.4	25451.24	18090.41	22903.61	26025.12	8570.457
TFRC	99.08291	46.47516	570.7785	39.48978	232.3128	39.5845	570.4091	620.9175	1445.521
NCKAP1L	7749.524	6771.01	7146.044	4037.456	3459.707	5430.844	4568.258	6260.022	5911.217
AVIL	61.19855	93.65582	76.35583	142.8232	59.88366	61.33914	64.28729	75.12493	97.95647
PPARD	335.6561	228.1035	112.9832	244.3106	128.5879	156.9152	101.641	119.5997	542.6342
LOC401233	149.5037	94.75623	376.0371	133.4236	191.3972	670.8454	261.3646	575.7328	189.2829
HCFC2	472.5929	236.0738	150.1968	253.258	147.6368	115.7338	192.7397	112.142	37.29066
PPAT	94.9486	975.2456	59.73437	50.78403	626.6738	62.01867	526.7977	57.79431	174.3772
FKBP2	214.7704	634.4964	163.6394	494.0858	353.1568	309.2349	389.0662	385.6649	368.8889
NXF3	1913.407	100.4983	737.1441	111.0014	530.8382	439.7244	388.3208	648.1954	535.2579
PARD6G	44.56976	40.20201	209.2973	717.9353	521.4078	549.326	308.5079	338.6723	45.68484
HRASLS5	36.28161	48.51716	36.80081	42.1944	48.518	46.64306	31.78955	52.00988	40.35907
MIOX	55.07953	70.5348	47.78655	36.00806	42.47356	38.45924	41.91873	58.35063	116.6874
LHX4	2945.973	169.1727	833.8407	612.1264	6048.958	334.5719	881.1517	1003.743	6219.06
SPRR2F	598.7847	1324.69	508.299	57.74881	18057.3	64.85859	5938.801	9519.746	55.17778
C10orf27	264.269	943.303	80.07217	152.8567	432.4123	86.93855	69.15154	56.64515	411.8701
ANGPTL4	332.2118	169.1072	94.38539	226.5071	87.66716	151.3896	96.60844	104.5484	380.5091
TRPC1	103.2759	532.4299	48.53417	55.18769	498.8031	1298.099	420.4665	49.31218	36.51903
YWHAQ	5697.982	6429.986	8568.184	7171.489	11918.2	10786.46	10172.98	11868.29	4982.592
PITX1	28228.42	24187.9	27706.86	22560.11	28400.18	26008.2	20419.44	22891.99	29661.26
PRKAR1B	52.04035	35.91265	314.8391	516.9634	137.2532	35.37405	65.4936	501.571	40.56219

C20orf3	199.3672	175.8397	120.5273	95.57426	101.4193	166.7427	95.01293	85.72357	214.9304
ZNF143	53.5071	447.2419	323.466	36.83075	563.028	37.06216	247.5914	125.9435	1202.403
TMEM71	39.85315	86.17426	195.4876	64.69646	512.4694	764.7229	304.3719	192.4598	42.82195
CEPT1	261.2392	158.0605	92.49592	183.8519	82.97594	116.8573	72.75009	108.4511	284.7355
CKAP2	164.2161	102.127	154.159	62.84309	63.39651	61.82833	53.69926	115.9587	238.743
TM9SF4	866.6525	590.5632	376.663	553.1109	542.5554	494.8453	569.3699	452.0949	1103.683
SH2D3C	192.8385	452.2976	65.39987	78.88216	60.00729	58.71103	44.26887	63.03044	62.99393
ATP2A1	46.02318	1034.178	38.04181	39.75336	79.12511	38.6208	222.4911	863.6727	51.94961
KCNN1	666.6398	103.4721	134.1458	176.1783	93.14575	163.3759	86.23084	87.58879	433.1332
C7orf46	97.13984	50.003	47.89593	94.36768	99.11959	55.37744	42.30195	103.1916	43.52173
ZP2	54.50363	35.40857	39.54131	33.90121	42.93358	47.21879	39.57082	44.71397	58.59402
TRPV6	156.9188	89.97851	776.8375	89.01073	582.1166	104.6836	72.73418	108.5885	163.2785
KIRREL3	54.49225	56.11449	53.55048	47.23442	51.67802	50.26186	57.19146	55.62895	56.17615
SMAD5	85.46597	768.7224	1610.177	884.3564	2983.198	2132.995	1910.899	1025.317	51.31588
VNN1	151.8123	77.76846	71.35661	314.8749	61.6983	187.2225	61.31356	63.94994	180.0249
HAO1	145.922	149.0954	73.38546	112.8041	63.38867	85.19731	60.18468	73.16265	410.4781
SIGLEC11	77.74036	68.98129	57.17803	69.46675	60.93781	63.75436	57.21511	79.67846	78.77296
FAM169B	53.60366	62.08352	47.29994	39.91302	38.52871	33.74934	37.76009	40.0009	75.34884
CLTB	34131.83	33229.14	33336.2	33817.67	33942.57	33690.41	30825.13	35364.18	36076.01
TEX13B	140.2928	182.1396	95.31851	68.34819	91.91715	154.9986	86.07414	79.72731	345.9131
SGCE	88.93806	66.25694	57.92395	84.24314	181.335	38.58287	184.4364	60.55697	144.1841
C9orf3	119.893	1970.026	47.38346	98.90597	624.6953	75.54279	1144.014	710.4546	429.979
UBE2H	46.78702	42.3573	175.8402	38.31377	219.0125	46.67185	151.0993	191.2796	44.49667
CXCR6	37.72516	682.4378	464.3441	57.2577	309.8856	36.41233	200.2292	53.99478	39.92642
TSGA10	41.18396	40.64885	44.75253	39.60003	44.5433	42.79988	34.90659	46.60685	72.05473
IRF7	36.16153	42.83949	38.38607	41.51554	47.40517	48.42771	45.70503	39.29765	51.7071
C9orf95	2451.037	2006.981	3339.767	756.8641	1543.385	3709.356	3233.08	1629.417	1019.134
COX7B2	37.83933	37.70996	37.27006	36.78187	41.92019	35.23151	40.91392	45.70981	73.69317
OR56B4	126.7426	95.66312	62.92965	101.8641	56.44117	82.13763	51.09352	55.91516	310.1288
CIRBP	524.5802	1786.505	1270.253	1867.8	2190.537	642.3074	2446.936	1712.899	2856.106
C15orf42	57.09634	58.77656	48.77263	65.36475	337.8917	65.71945	260.4329	383.6961	51.86586
CPNE2	42.79768	53.56236	41.42115	51.75558	45.98519	48.37824	56.07055	56.62638	74.70196
OLAH	106.8222	111.1733	52.80119	134.6044	58.94328	80.57836	50.95464	66.16878	79.42178
AARS2	5828.064	6133.135	6378.877	5989.621	4541.088	5816.767	4731.344	5315.303	6119.342
UBE1DC1	678.3624	714.9444	556.7761	324.1371	882.4027	1166.793	616.3765	453.9706	55.63212
MTHFD2	77.37211	3463.408	5730.398	5666.492	6098.179	5387.91	6345.561	7215.309	1435.249
NID1	1170.025	971.9007	174.7506	75.31231	395.8272	729.0576	755.173	413.8373	1652.051
FBXW12	65.80429	37.8101	43.66317	54.06228	48.54537	43.54605	47.81395	47.45348	67.59608
LPCAT4	166.4718	136.1756	61.34345	90.6434	56.97601	118.1048	56.60131	53.58115	313.8563
PCDHGA4	39.74347	45.43771	36.49351	31.92117	40.31824	34.52402	41.43922	32.30019	39.33991
SF3B1	2308.766	508.9301	2461.354	1880.612	1623.15	932.3783	1361.387	2590.225	35.38063
COL2A1	111.9075	47.00504	44.85442	567.6572	50.43375	69.47455	128.0145	142.1091	231.9093
OASL	150.0524	364.7505	75.9509	117.7607	123.2545	119.449	249.7771	323.2855	229.9983
C1orf115	59.67925	50.49913	209.4931	53.90631	355.425	500.1574	359.4316	332.0782	32.3776
PPIE	1157.931	1156.316	183.827	451.6525	129.0843	307.3381	225.2374	113.2375	1317.949
FAM124A	120.6008	75.43416	61.40973	112.79	257.2668	71.57051	138.4253	225.8492	81.66058
ZAP70	55.2685	31.58172	61.6949	44.42	51.5406	67.88578	60.62158	76.92749	49.58447

TRAM1L1	58.96081	80.1274	42.08122	76.47766	35.68854	37.12425	43.85094	42.03872	46.84761
TADA3	480.9214	330.4359	667.3589	742.4989	664.2322	1345.164	852.7019	928.2255	646.2968
MFSD10	22934.85	26962.28	28292.36	28975.07	24102.2	29227.01	28560.87	29517.62	23951.3
HOXB5	1144.228	957.2427	106.8465	2384.906	1209.818	923.7501	644.5759	1015.128	1231.506
PRR17	136.0786	498.7783	502.0599	454.9641	314.6118	1164.893	275.3609	531.2151	1809.647
C19orf35	58.58073	53.74104	46.29654	41.04478	38.57071	42.11718	39.0486	43.78883	36.43667
FOXF2	489.1468	169.3687	108.8026	210.4094	80.3772	136.8075	75.81828	122.6544	284.2632
ANO6	484.6758	320.0773	204.0069	385.1134	132.052	240.5654	152.188	220.9769	817.6243
GDA	35.09507	35.86948	63.4604	39.9247	46.87782	39.45834	43.28641	34.56837	46.54593
SCP2	73.84641	71.74938	107.3669	54.7265	35.32793	54.92809	57.27945	37.28633	53.04498
LPAR1	59.47329	40.66779	33.45803	307.1734	63.57473	39.58197	90.10293	106.3468	46.75097
TMEM51	49.64555	1968.647	4463.814	1060.685	4231.317	3013.714	4625.782	5485.74	4227.562
OR14C36	44.68758	70.85579	55.66101	53.68641	46.02917	68.84884	50.51584	56.24996	50.66159
ZSCAN5A	39.77937	1911.543	2252.63	2855.88	2659.902	2411.38	2599.182	1667.074	3287.949
NMUR2	75.55009	31.30547	35.46014	53.5243	34.96017	42.36765	35.75637	50.69259	52.83338
C1orf165	69.12016	34.93381	182.5853	63.78211	32.55651	59.65871	43.91083	42.74608	103.0581
PRMT2	34.45617	611.8406	755.2485	42.27416	639.8577	35.09671	259.5552	460.7624	29.0524
IGF1R	61.42176	57.8769	147.3218	42.45418	45.19272	50.40422	255.808	183.7914	164.1598
SYNM	62.24032	64.63979	56.22598	57.13876	50.09009	65.18261	59.55574	55.04897	70.02382
C1orf166	1248.204	383.8402	1182.002	620.8315	1068.525	1003.223	1371.244	1094.099	238.6062
MYL9	2725.895	3322.664	2519.909	1521.312	5045.303	1869.816	3385.966	4415.134	1951.546
UBA6	835.0054	1239.984	2069.603	1161.216	1535.396	2918.995	1852.351	2381.625	752.451
VLDLR	47.22427	67.51087	142.779	42.60593	46.25943	47.49417	34.34386	35.22078	49.35276
C17orf66	49.7239	45.4224	35.85137	43.99008	38.4808	60.33932	46.26646	75.33488	97.03701
EDNRB	57.74192	59.92902	75.78664	115.2542	51.55482	63.0436	48.70467	76.5535	456.6827
YIPF2	1842.358	1590.64	868.2114	1542.148	612.5991	813.2178	580.8532	1247.905	2059.254
HSD17B11	63.89411	52.75101	53.19174	50.39432	87.771	43.05062	49.03136	61.8624	50.45966
DDX17	1447.655	1398.448	1019.418	1356.955	1251.232	949.7603	900.6247	625.3447	2208.134
PIH1D1	139.2939	678.3093	212.1001	705.5165	340.4458	76.24116	455.5103	261.0139	111.9326
HKR1	822.2183	393.777	352.7971	480.8994	253.0936	418.6742	310.2047	350.4099	789.9611
CDC42	37.484	38.47321	36.84089	34.86354	40.09661	41.87817	35.62934	48.15772	45.98442
RGS9BP	45.77788	53.33681	447.8923	60.9056	211.4421	60.25924	342.9266	709.4045	54.27628
ME2	49.78337	42.34946	1180.706	771.4482	788.0396	1176.082	1104.151	744.8024	1727.12
RETN	43.93954	36.02711	35.54832	50.24165	33.81396	33.25042	39.86634	35.99124	54.54774
GAPT	71.91178	58.58073	354.5794	102.6302	169.9747	57.80923	150.6526	414.6815	72.7953
IFNA21	176.9503	101.8987	82.29036	185.5266	77.49039	128.262	68.51672	91.76145	337.3935
ADAM22	654.213	399.5622	185.8646	373.6756	106.7403	335.6814	249.8165	245.3625	705.9587
WDHD1	319.682	125.0556	124.6481	738.0778	61.00152	208.5093	139.5698	76.52197	333.8534
DGKZ	55.75884	33.70987	126.7218	37.75977	139.5309	42.67443	173.3163	114.9278	1026.485
PLXND1	645.4268	345.1624	221.5135	343.773	172.7492	260.4082	183.6851	198.3028	795.2792
NDUFA7	954.6688	46.16827	273.9252	539.8911	414.9109	850.508	462.6201	679.4202	43.92897
LOC441268	4055.682	5672.401	3120.365	852.9377	2592.818	1387.379	1051.483	1206.519	2507.239
NAGPA	50.583	910.3948	2565.049	454.304	2223.688	1098.277	2000.031	1557.748	251.6453
LRP4	1765.231	42.35383	40.31625	1574.182	842.6915	35.66217	1369.12	697.6381	40.13461
PPIL2	51.49448	59.94041	36.79645	63.90901	38.06317	47.53363	92.77716	44.57365	35.03552
FLJ45422	1533.314	1186.843	376.6743	84.50881	507.4829	1432.698	195.3005	499.4552	81.45237
PSMC3IP	40.86407	42.00724	46.46718	45.00118	36.81589	42.40207	50.66284	61.24682	36.38285

ENPEP	1996.582	3188.988	1523.951	2742.731	1360.844	2536.314	2036.747	1940.646	3409.871
PEG3	43.43563	48.09184	612.7736	49.79024	45.84063	35.00021	49.38607	40.60286	98.72464
FLJ44955	92.68015	80.35806	95.68214	133.4105	92.90234	116.4684	68.58875	93.92139	319.8789
NPPC	582.1827	33.87587	42.68002	43.47593	42.39875	57.81036	35.39878	44.2769	75.71111
MYADM	43.88964	50.87777	44.57	52.20865	49.6776	36.82845	53.67483	61.32467	135.9998
NKAP	359.7513	200.3216	107.3066	230.6286	96.78363	155.3828	77.54556	119.4745	532.5862
SOX14	47.17016	36.06793	51.83562	45.38356	34.169	48.21458	41.69241	36.32677	57.71527
LARP4	324.1271	227.9804	209.3551	194.8711	230.5329	245.0951	200.2048	246.4418	632.7139
AURKC	35.12432	39.76617	35.0893	44.06434	31.61887	41.50942	38.1699	32.7999	35.22268
LIMS1	222.6116	194.0053	92.99528	463.9525	128.6861	199.1641	108.7528	65.79525	1236.251
SLC24A4	75.93195	76.76483	53.5324	77.33995	55.27469	52.86964	134.3203	38.91978	51.20473
ALDH1B1	852.6545	145.3258	298.5813	125.1119	64.3449	63.99869	182.4454	85.66254	214.8852
PPAPDC1B	47.21231	50.88604	48.26767	62.18858	43.65739	37.40177	126.6402	76.17954	200.3298
ENPP7	140.7467	84.14445	63.00037	139.4485	58.51511	100.5962	63.93162	101.4293	198.8827
ANO7	41.15841	55.88069	37.26993	49.70669	46.89206	39.60965	37.6575	38.13905	83.66383
OR4D1	135.9877	110.1289	45.11404	80.63908	60.24173	35.7792	48.65494	60.05864	82.45209
SMURF2	172.2736	176.3544	84.83717	180.3563	93.19673	104.3953	73.32575	81.62607	137.7537
FLJ34503	57.28791	49.52921	38.95435	61.16757	37.22185	66.97937	42.90034	54.1705	56.5658
KIAA2018	30.93642	268.997	90.27187	36.00117	72.57446	47.29911	65.62055	40.06017	61.20527
ZFP36L1	4078.253	4531.204	7960.084	6056.025	7439.116	7378.3	7096.541	3833.296	1300.934
HHIP	123.9752	54.10645	51.47662	96.27227	39.8354	86.2408	64.17199	51.6302	128.9261
CDH9	37.2485	60.78767	57.43322	64.31847	36.23423	51.22914	41.4761	42.43714	200.1166
LSM6	64.84371	76.70718	557.3366	38.06526	675.2296	1059.161	761.6791	714.5787	52.56441
ZNF646	40.24584	48.4286	293.4733	65.24105	40.63082	74.41352	50.72957	56.28282	65.60569
ANKMY1	4691.544	7008.191	4212.764	2977.23	1259.669	6232.716	4103.319	3665.217	6824.974
SENP5	40.38278	85.35208	267.7178	89.29097	297.0931	65.78049	211.5666	150.228	58.69642
PPAP2C	466.0196	244.548	135.1906	230.8618	128.2969	197.4709	142.5534	225.4134	682.0663
PRM1	68.21631	61.00841	61.5067	57.36427	50.59501	43.88476	39.28769	56.37878	75.59597
KRTAP27-1	182.7893	86.11235	46.84248	78.15433	74.15007	104.4202	46.33377	74.44137	301.075
TRMT61A	296.416	152.847	222.2373	126.3035	281.8734	88.27808	298.7693	75.57945	356.6201
SPG7	400.1446	2139.368	607.7271	1815.517	871.4031	925.3774	98.81164	250.2023	524.4724
HTRA4	236.8143	147.5744	81.26694	152.8522	77.95828	121.2921	73.84656	98.16715	178.3829
SLC22A1	221.852	56.13531	67.35851	78.7236	56.86977	59.36843	53.67086	57.33403	79.53661
BLCAP	164.1091	756.7859	1501.215	707.8268	3942.086	1741.911	3398.98	4019.485	85.67716
C12orf54	886.5215	551.0086	2320.929	67.0964	882.0709	498.369	2161.455	1775.1	1847.189
NT5M	45.93657	38.4903	32.2687	36.14151	52.40733	51.11957	35.6016	46.07274	50.01986
FN1	203.3167	103.634	54.82074	567.8125	54.01919	113.8142	92.42517	42.08951	63.29168
PPFIBP2	7368.458	7150	9079.765	4855.749	11299.11	8735.713	7331.158	10471.32	7483.785
IBTK	3288.893	4678.097	5979.742	5038.344	6622.223	6471.139	5758.756	6298.783	3513.438
ICOSLG	102.6975	1642.495	3187.085	1001.766	3279.875	3222.378	2429.151	2210.018	329.925
UBE4B	119.8173	275.6685	352.0555	90.82649	503.5346	306.4769	556.4536	477.3967	162.3281
TMPRSS3	52.06819	53.5865	37.99339	65.75207	38.35262	49.46289	35.09696	76.2086	39.6302
APOBEC3C	89.7553	471.4008	179.4217	93.0022	147.8324	350.7449	320.9012	51.40309	110.7811
PPP2CB	48.88749	75.36441	262.8065	70.99796	395.3873	53.13521	213.7708	339.4987	41.16936
PDS5B	58.30371	37.49187	157.6263	35.18989	163.8391	269.2264	229.5464	123.2888	128.3032
FAM114A2	494.7345	39.13359	220.4165	333.3605	52.08472	191.6673	127.2832	34.26685	37.41129
APOA4	61.93617	42.10154	43.45973	39.44422	51.83228	52.93388	40.83834	35.6739	61.637

ARFGAP1	54.5683	57.95333	2001.21	56.4758	1950.104	480.7216	1007.23	1475.45	226.6024
UBE2R2	318.689	194.3537	119.1205	202.482	138.2658	185.6412	102.4588	145.5442	526.6552
PCDHA1	33.34111	49.7975	55.67403	45.9305	36.93196	35.82289	43.37278	49.24705	55.4784
SEC14L3	175.7626	129.8968	78.39146	126.3367	65.45306	89.93454	69.63506	82.23845	257.1023
ZNF175	6355.933	4877.763	3545.131	4140.52	1695.544	4042.447	2960.192	2888.559	6064.728
DDR2	60.93086	82.62265	57.18322	61.50198	53.30156	37.9566	46.72894	78.44452	37.75539
EPDR1	928.2882	796.7339	311.2923	635.3651	155.6846	431.8975	243.2769	396.157	34.8299
TCHHL1	44.48936	34.3363	40.34851	35.01776	34.44989	36.66928	64.18883	35.89095	42.65952
FAM101A	151.0629	104.2566	57.26471	107.1298	78.11865	94.81657	70.43686	86.04626	75.57755
PCDHA4	329.5983	74.75687	59.71106	78.54436	69.8379	63.3615	49.64905	64.013	42.46878
WDR1	2244.781	1380.501	481.4254	2353.706	697.9046	917.8524	778.2926	424.7596	1027.02
MYL12A	15527.09	15758.13	18247.63	18626.85	15538.02	17435.03	17744.83	15846.15	13951.64
COL4A6	166.9049	78.68721	51.29563	89.47391	44.22738	40.52608	281.1386	45.68247	102.0865
ARRB2	440.2366	254.6303	183.1679	268.4055	149.6095	194.5712	167.2393	197.389	594.4957
SH3BP5	50.43916	51.715	40.37845	344.5464	50.8714	42.32255	103.4305	53.24819	77.95143
NEBL	55.25008	49.68278	78.67425	563.5072	42.76117	31.88827	84.94405	39.23491	59.05815
HIGD1B	47.78086	56.15468	51.22622	59.80985	49.07501	38.90446	48.75453	68.57539	70.09814
ANKRD9	46.10701	61.34996	52.02048	49.68128	46.35744	45.86789	40.96079	52.56032	49.70412
IFNGR1	48.75681	40.58748	1125.133	49.36194	2047.141	1921.829	2584.095	2151.935	982.2598
DUSP15	55.5395	37.49992	35.12196	36.78202	48.94891	32.72235	43.22418	39.22634	54.43933
MT3	50.43621	52.6465	37.64401	39.32383	213.3573	287.617	71.78885	129.1701	48.03996
GGA3	1885.146	1020.782	254.4605	822.8521	221.4084	79.00716	251.9655	400.7699	896.428
ARHGEF7	51.75626	99.91258	191.5665	891.6666	151.7837	342.8226	176.6564	191.8109	1553.09
QRICH1	4428.818	4484.689	5455.105	5801.866	4951.767	5303.87	6261.344	4149.356	7296.969
PNOC	43.59307	45.91728	113.2387	43.96192	42.81842	59.98604	127.5128	39.30708	37.45272
KPNA1	2205.666	1508.603	1289.697	1624.639	1088.264	1947.78	876.0968	1987.162	1565.798
SP1	60.91133	364.3574	1247.107	1140.378	1485.1	2119.276	1596.409	1337.298	205.3644
C1orf152	37.84788	48.28113	488.549	52.72312	200.3976	548.1968	41.85113	44.65602	36.30546
MRPS26	3491.019	3111.18	4534.248	3543.589	3379.167	3437.092	4069.363	3126.291	2289.393
MAGOHB	788.6382	765.9304	275.2657	1016.955	163.7428	336.1658	184.7525	202.7006	1079.32
CLK2P	262.8451	160.761	54.96218	741.767	162.6598	87.07294	164.0029	78.59611	300.3605
DIP2C	57.05086	45.51859	125.148	332.4778	158.9576	207.7445	259.3592	125.8773	34.69791
ASB4	67.06016	42.29507	54.26381	49.45086	104.538	42.50425	43.75177	52.94927	67.43474
SEMA3G	70.3662	447.8706	166.135	55.60788	115.1351	44.03015	119.091	262.7063	967.2989
KRT82	48.52062	39.87753	37.77116	50.58172	37.68429	56.77761	43.83709	48.58911	92.18858
MSRB3	71.46878	65.05568	172.4033	51.3003	278.3217	46.20297	47.86301	141.7684	69.15443
SPON2	35.92984	79.21048	261.9402	58.60427	709.5718	49.78233	750.497	683.2471	36.79369
NHS	59.06938	36.78259	59.67947	46.37714	34.9167	51.06944	88.07698	242.4207	48.77157
MST4	8069.074	7360.025	6218.596	8304.471	5485.679	5881.168	4901.132	4153.548	7120.988
APOL5	81.68064	53.86456	53.40679	75.41828	48.75985	59.31088	47.20547	90.47874	123.0578
DARS2	5941.604	6247.446	6862.093	9509.633	5963.277	8568.305	8375.917	4774.234	6815.691
SLC13A5	2426.803	2470.406	923.5393	3025.626	1790.481	4057.77	3654.682	8487.935	2593.989
LRP11	157.3506	266.576	129.9808	404.6191	214.7836	58.05122	212.1802	89.32615	770.1768
SRPX2	2512.288	1221.55	3680.7	1985.259	1655.274	2703.796	2316.043	2089.573	1319.469
DLG2	49.80404	46.17259	757.9666	63.12796	378.4422	51.4697	131.2717	214.7607	175.1963
C16orf89	392.8308	207.5179	87.84264	149.5192	96.25365	149.7842	79.73042	101.9817	458.4083
ZNF460	55.79358	42.3545	278.428	460.1447	188.3952	102.0033	375.2667	358.1017	84.28537

BCOR	1699.304	585.7535	1308.578	858.9297	1421.345	1285.606	940.7627	431.5638	38.89702
JPH2	316.181	155.9225	145.0948	158.3941	94.5631	129.4127	164.7273	199.7227	327.3045
SEMA4G	51.32409	53.83313	53.94115	57.18829	44.82425	63.24064	38.74512	41.27151	90.23522
MTMR3	77.7719	36.75408	47.10617	89.54037	37.45914	520.2488	37.96383	203.2651	46.90286
TMEM211	31.68343	35.42548	34.77011	34.37833	33.05182	40.87053	35.12539	36.93941	34.27505
LEPR	182.5632	87.88387	65.40093	100.7561	272.1135	96.71312	49.01425	253.0989	126.4982
ABCC2	70.36879	66.64494	65.32384	61.26829	56.86367	47.74212	52.94413	53.51455	101.7463
NSUN3	183.7301	101.3463	78.05338	123.9832	65.32608	96.05929	69.05054	93.50547	202.3667
RIMBP2	50.20574	59.67777	48.36844	66.24889	50.42838	43.95925	59.67121	50.44377	166.8816
ACBD4	136.1912	56.04111	60.52874	91.67091	66.51505	190.3666	58.70252	201.6194	160.8441
NCAM1	281.3755	283.0764	74.6434	140.2767	90.36642	127.1272	74.41505	116.04	466.0706
BVES	37.61999	46.70271	65.90424	46.8994	50.28428	76.10722	65.06326	57.82827	36.51808
PPAP2A	266.8566	100.829	90.39095	111.1245	93.89486	206.7544	80.62632	107.1013	246.4757
FAM113A	62.8014	998.5809	2744.509	1680.33	2671.988	1929.402	3074.414	2610.163	51.71626
PTK2	2052.336	2112.785	986.8201	4612.785	818.0927	2010.66	1201.421	998.8828	1869.829
FIGN	72.31646	55.7364	49.28908	92.86871	140.5028	137.9419	51.90385	54.74416	183.9378
COX18	57.93145	62.52744	47.47078	73.55003	49.47874	45.56723	92.84936	40.87089	123.491
BAZ2A	561.8513	262.3023	74.80917	284.6592	106.0469	121.3162	84.10022	89.78812	102.3906
C1orf172	49.37146	46.4276	47.89944	48.46932	90.19838	51.19102	43.16401	42.36082	61.1534
AADACL1	48.82603	308.217	54.71183	73.28237	110.8896	240.899	158.6572	141.0903	52.75633
CXorf36	2889.627	596.9888	330.3539	53.46027	236.5094	42.94781	44.60918	232.3546	33.86088
HTR4	155.9194	112.635	89.62094	146.555	72.64747	89.55053	62.29674	60.73071	193.8476
RALB	60.38742	781.9272	1143.583	3059.863	1747.931	1325.125	1052.51	1033.398	43.41311
ARHGAP22	995.4246	1138.803	903.9474	2415.835	713.629	1404.671	1591.09	726.2758	177.8631
SIGLEC12	1117.295	42.279	417.6666	49.34144	41.36339	501.2863	83.4227	40.58807	50.77318
CD209	42.08569	35.66766	1259.743	39.8367	924.1104	1669.967	1534.278	897.1841	50.37688
ENPP6	55.29042	38.1842	36.95288	52.67432	37.22328	34.8214	37.62965	46.66266	58.9429
PCSK6	33.73898	30.40451	32.54649	43.0649	211.9842	35.02983	133.5659	256.8977	38.08768
PAK2	1120.273	90.57299	324.4662	882.8796	577.876	327.5766	509.1436	392.877	1564.477
ATP5G1	11992.38	11852.27	14275.87	10723.41	15228.5	15711.07	13499.82	15164.98	10767.88
CTU2	146.6243	96.68111	55.58429	103.5949	70.91656	77.69875	90.56549	67.91001	218.3884
TAGAP	303.1787	196.7515	221.1253	200.1104	164.3157	160.7051	123.8527	109.4282	335.2124
VSNL1	10312.74	10702.28	9478.293	11093.65	10069.47	10507.19	9216.083	9602.298	11570.62
CCNC	46.49679	1284.392	2205.311	750.6267	3261.167	1698.794	2693.132	2801.33	49.97205
METTL1	60.06474	46.55955	43.12889	47.88531	51.60591	56.70153	41.84278	45.99724	67.51009
GCK	54.17595	57.47362	36.82595	40.1334	45.54037	49.4752	39.93379	60.72554	35.05652
GPR173	61.22323	61.20909	56.28963	52.82019	168.2078	58.65955	50.94898	58.8578	74.55132
WDFY1	10824.8	12306.6	13799.44	12273.82	10638.53	13516.45	13416.73	12046.24	6839.403
SLIT2	214.1248	156.9532	488.4205	188.2941	894.5832	283.5755	671.2506	982.9933	249.5923
ADAMTS14	36.19081	41.73665	42.80652	49.87307	47.29633	432.5755	80.81544	50.44413	37.58916
MRPL11	70.05904	1166.101	355.5055	1565.527	37.77021	285.092	172.4613	202.9988	2685.849
ZNF785	43.43737	50.97019	40.45364	49.98883	51.42314	42.89175	53.78189	157.2897	49.11749
TCF7	152.0101	155.1223	368.6284	176.2406	62.65901	117.0782	207.2146	97.38171	212.509
MIP	117.5541	56.18382	54.04927	85.0639	54.46056	45.12082	50.60047	34.9372	70.49215
RPRML	716.758	538.1048	127.3381	437.5071	43.23507	135.8104	102.7355	159.6766	749.687
IMPDH1	2911.04	2604.066	2132.371	3631.172	4264.741	4167.053	4167.478	4124.177	3635.233
CCDC91	546.2751	453.7076	245.8795	615.9342	202.8121	384.5556	354.0326	114.0085	84.56377

BTN3A2	37.14153	1327.979	297.9441	468.6945	345.838	509.0407	381.1391	416.2319	1245.788
BZW2	57.32079	1601.786	2880.265	41.8957	4273.077	1610.059	4212.402	3854.932	50.94033
TTC26	70.62874	72.15295	937.4893	76.23455	542.764	205.7605	537.418	669.1585	105.2576
HTRA1	92.82198	2135.807	4098.738	6279.646	7283.271	6289.792	10715.28	5471.787	108.9681
IKZF1	1357.539	1150.978	2682.946	1587.184	764.7142	2853.248	1562.788	1314.829	2222.01
HIST1H4D	44.70971	36.53658	58.69999	42.85418	33.88614	36.33516	41.70604	87.44699	36.63272
SEC24C	121.0679	2750.79	4944.142	1642.762	8183.061	2402.337	6018.821	5844.696	2625.086
SLC7A14	57.73865	40.17999	38.53728	37.07338	44.24974	54.82872	52.91275	49.34929	46.26472
ANKRD42	1104.605	803.8268	226.0005	62.25425	279.0078	434.5696	468.6226	247.3127	66.61515
PHPT1	191.1728	744.8447	1038.755	143.8685	734.3991	100.2318	756.6831	1645.587	2185.261
PTPRZ1	98.62115	101.9426	205.5303	99.60072	56.94355	240.6374	55.68859	76.66698	68.54909
CHRNB2	66.49203	41.37522	49.64813	65.36248	57.99135	44.7488	39.82423	53.13999	48.74703
SS18L1	430.2072	245.1745	162.5041	215.8103	158.438	218.2732	127.2382	134.3175	186.3648
ARID4A	51.8972	232.6508	52.75326	61.63931	84.97431	75.38757	74.37173	48.86103	47.97534
INSIG2	169.4153	57.00937	92.54105	44.69274	88.242	50.49566	68.12277	68.54312	45.88382
CCNO	120.9577	83.80875	1526.963	93.20621	1983.135	2299.572	717.4433	1209.431	266.0376
DISC1	1265.211	1606.167	2793.818	1173.478	1579.705	1229.611	1796.466	1035.871	1570.538
AQP4	134.3197	84.86748	56.00719	91.76737	62.45274	66.75072	51.2085	57.75084	146.9019
DNHL1	53.89265	46.86374	42.25189	59.61892	51.91553	55.32084	59.9911	51.43854	102.5692
TNFRSF11E	243.5076	179.0143	133.3152	141.5053	37.06959	149.3296	68.34692	61.94201	126.9631
CUEDC1	906.1104	525.3848	412.5074	522.6284	346.0142	568.0756	476.8181	502.2523	712.1979
TMED8	74.42677	60.32999	54.08644	473.5356	69.48624	68.11889	73.08223	182.5263	168.0228
IFNK	140.0154	52.29502	59.99358	452.1572	64.33098	63.30545	85.72163	62.07393	84.39228
SDK2	9629.042	10529.75	8338.472	12345.65	6069.911	11951.82	8321.899	11974.61	9165.571
C1orf116	83.17913	204.9807	166.6381	547.4534	87.06403	37.15701	85.40308	179.1864	54.23742
C17orf65	2799.679	798.1902	162.2722	1662.201	38.4259	893.8635	206.0482	186.1182	2368.075
Septin 2	990.7316	1264.859	735.0371	999.7407	424.8093	632.454	285.459	501.613	516.162
ZNF142	7620.891	7226.714	6819.49	7384.166	4866.685	6371.905	6205.844	7113.093	6940.142
C3orf15	48.43692	59.57251	38.08628	63.42144	263.2352	57.13725	148.0293	43.96986	71.20734
DDX27	1832.646	1194.689	662.1194	1187.354	558.2563	623.965	701.415	1011.701	1119.466
SIRPG	52.57041	68.33617	53.63424	57.07993	53.34313	44.88331	47.98024	38.96152	64.78915
REG3G	112.1352	137.1396	93.39098	167.7095	58.51359	68.95023	50.65419	56.78452	68.06148
C8orf37	95.48162	37.77053	199.7131	45.52705	51.43556	50.42493	122.1235	202.6464	69.4459
MFAP1	2470.811	3104.859	1278.441	3270.374	1559.655	1310.252	1770.789	1281.383	41.15173
C2orf40	41.55532	40.13697	32.46736	44.81456	330.8504	52.14751	156.4218	247.4385	47.92836
CYB5RL	38.03596	69.66354	47.69578	37.87392	47.57452	49.43023	42.75123	173.8145	39.45937
BTAF1	8565.063	9092.584	10237.28	7765.46	11501.61	9366.94	9197.014	8955.114	9111.321
ABCE1	4831.674	4907.061	3703.419	6033.184	2389.646	2784.002	2084.331	2820.108	4688.989
RAB37	123.5942	60.88678	130.5202	83.68021	72.66634	87.87331	62.64019	79.97915	210.2021
GABRR2	57.72346	53.27399	221.0853	51.13206	40.64272	45.08295	34.28821	48.56068	105.8902
CORO2B	35.93282	40.22593	47.23471	42.10237	42.62516	35.17899	39.47798	44.55733	43.96566
CDC2L6	6975.188	7056.567	8545.575	9620.715	11141.08	9208.2	8736.678	9242.677	7928.59
ADCY3	7794.533	9978.57	6143.493	8483.848	6050.292	10716.98	7633.832	6758.959	7631.54
HSPA12B	659.0105	817.4555	717.2821	36.86314	487.6709	506.2187	708.4606	517.8622	34.8986
YEATS2	86.26303	82.73079	280.8841	55.50179	53.80293	60.38101	201.213	452.7568	66.46064
TIGD7	393.0609	338.1777	1058.139	334.6765	651.9565	1136.25	553.6773	623.6801	50.83521
CD247	1461.415	651.7296	3374.175	835.6357	1261.943	2744.312	1332.902	2325.615	399.314

AP3M2	8842.83	3849.213	7349.15	9128.561	8008.211	7969.225	8194.469	4920.283	3499.377
LPAL2	39.36693	34.20924	37.14931	33.5834	42.54966	41.54413	35.19021	49.25283	71.86593
TYK2	16349.16	16012.83	14970.84	14390.2	13205.48	13978.25	12723.16	13629.15	17355.16
CABP5	40.19537	38.42387	33.80228	34.72813	39.9269	34.61687	34.32483	35.27319	46.52618
SNX15	2049.788	69.63806	63.76798	84.08247	77.15115	49.73006	58.02766	74.31815	162.1647
MTSS1	129.704	167.3477	79.68059	151.9252	112.7518	111.5147	88.95612	140.6832	173.6034
ZNF300	34.13725	36.61598	233.9445	38.37004	654.8007	44.87936	148.6614	252.6681	38.6977
DDIT3	463.1584	621.5112	336.2073	1071.44	70.16149	414.35	407.989	394.6844	839.5175
TREX1	200.9602	661.5458	67.25536	158.9865	73.14754	123.491	123.3324	83.74536	128.1986
SCN2B	60.78182	44.1259	133.178	37.76469	42.21124	32.51305	78.59904	35.3002	50.24922
SYNE2	39.79883	49.79495	39.63802	42.72818	42.45235	34.99705	35.57798	41.19689	38.56185
HOXA4	42.47085	37.59221	40.91612	37.62425	47.20179	38.46688	95.86123	181.1401	40.43938
CACNA1I	43.20607	41.9527	36.34361	48.96745	35.02632	40.6039	36.03568	47.44843	51.31317
FLJ21986	11857.34	11336.34	9300.386	8914.86	12243.03	10640.57	8615.787	10177.54	12039.55
CHUK	748.3034	60.44954	406.1828	126.9369	737.6876	466.123	566.0347	695.5117	63.16913
AKR7A2	3645.208	3304.562	3292.149	3139.648	2310.328	3047.868	2321.034	3262.82	3242.696
C11orf60	76.40273	89.6068	176.342	1633.373	953.0363	38.07224	644.6509	804.3441	95.71014
RGS19	608.8676	506.3717	727.0606	282.0605	493.9023	675.1072	671.0974	414.4813	287.4752
YPEL2	46.39221	51.15935	250.1616	54.33297	54.4217	40.34008	163.5449	48.41619	75.60825
TRAF5	35.93982	50.40972	42.31252	44.73952	32.54224	47.15322	35.0794	48.83148	59.08027
CAPN13	2058.856	42.79401	2874.407	47.2832	38.11814	1274.182	666.4316	307.7631	57.29344
FAM168B	76.67793	82.31316	50.4255	438.1365	414.1561	34.5547	41.60841	55.41479	39.3826
MITD1	153.9965	763.9929	1105.319	1089.577	648.0216	466.7881	590.585	885.6364	112.2346
OPN1LW	36.58229	37.24999	44.55549	34.8517	45.5662	38.03663	35.9127	39.43906	89.96951
IGFBPL1	432.745	230.8886	114.242	267.2946	112.3556	170.3257	108.6386	112.6081	605.191
DEFB121	40.71552	44.20832	36.35371	37.15208	35.54281	49.82128	40.04869	52.79965	64.78445
C1R	1543.824	1769.574	659.2812	2053.374	1637.162	1820.543	932.2445	1033.241	129.1521
CYFIP2	5106.395	5789.646	7164.589	3657.974	2611.174	5239.867	4083.886	4833.504	4883.335
ARVCF	252.9913	152.2635	93.79324	188.9838	73.39077	109.3442	106.9147	86.02411	179.8431
CCDC14	7250.075	6936.567	9925.102	8203.4	10868.87	12539.24	10866.06	9436.995	4833.232
SLC10A7	54.17329	45.65934	40.83213	366.5469	190.3206	44.26061	35.52085	35.28674	37.07123
LZTS1	66.1039	84.42544	33.50553	53.18003	48.30276	54.34692	114.7428	289.1082	43.0036
MAGEA2	44.86291	58.10088	170.7519	42.46988	38.58277	57.45506	35.40044	35.99591	32.45274
OR4D5	228.8661	168.0738	96.62029	211.2852	70.16747	92.60822	80.13232	79.34596	305.4102
TH	148.0938	86.94946	60.81967	1231.699	57.76421	73.92856	1618.501	618.8894	147.2514
C5orf30	5209.248	5064.641	4322.696	6141.666	3323.397	4192.597	1890.524	2520.23	6485.678
C5orf42	69.90236	69.42381	114.3729	87.95531	51.26271	54.28783	76.15533	122.322	39.14231
PVR	811.9764	1834.727	1013.025	2086.285	1180.486	929.0969	1295.41	1406.081	2671.701
HMCN1	651.7567	506.9869	349.5955	263.5349	1028.281	702.7036	418.4087	329.1401	658.869
MUC1	145.1157	270.7681	138.4181	280.0229	64.78286	226.9588	85.68693	95.51588	74.5285
MTUS2	125.6603	112.8665	51.67601	102.75	47.50287	85.802	52.3749	42.2366	192.6125
CAPN10	40.6926	36.59919	35.9316	64.94338	40.26159	90.51515	31.89907	34.22638	41.55296
CHD7	3678.658	3063.056	1787.473	3906.486	1425.483	1581.801	1138.782	1966.741	1424.125
PTGER3	52.99572	101.5821	45.29179	88.76403	93.85601	58.03556	51.6748	57.22527	49.88978
C8orf40	49.49629	77.02328	798.6635	49.6452	591.9194	712.2778	1778.751	246.7817	1430.088
KRTAP10-7	46.17806	50.40571	42.98507	42.42994	37.20753	37.73793	47.0708	39.01564	38.89098
NDFIP2	6163.209	5060.782	3989.523	7271.756	5777.206	4783.146	4786.858	5027.966	5417.737

RAP2A	37.55378	35.49421	43.45026	398.3568	340.2256	37.88484	268.635	80.99181	35.24438
SRGAP1	3508.083	1556.153	882.4698	2773.275	655.6758	368.1081	996.3065	834.4822	4996.426
GLS2	353.9767	193.94	66.11533	174.4003	69.2571	93.48886	64.89089	94.71692	577.8599
ATP6AP2	3122.447	2858.227	2098.079	3730.782	4081.859	2237.802	3544.542	2863.715	1397.183
REXO4	5197.164	3803.849	2678.494	4717.551	2502.845	4256.739	2911.506	5237.704	6702.427
FGF4	108.4685	109.2432	71.43267	89.28919	55.1707	108.44	49.34013	58.92694	134.1031
DUSP6	1916.093	45.54746	281.3715	33.94305	205.284	35.11975	301.8241	33.1689	42.74882
SNAPC4	15582.09	18490.75	18161.65	12538.72	14438.73	17570.79	16422.71	18274.4	14029.29
MACROD2	57.18818	49.21885	45.81905	45.4208	59.77413	35.85198	58.26803	69.64154	191.6117
ANKZF1	363.3696	190.5186	118.3689	208.6205	416.9164	181.4942	266.4893	184.0211	386.0896
STAB2	46.67915	43.5037	256.3936	42.02763	39.08686	38.301	123.252	356.4618	41.86337
CCL21	6099.644	4575.749	4174.338	5919.949	2661.809	2382.653	4572.661	3100.55	5809.892
PRR7	66.64387	61.15932	38.08634	37.20745	78.61354	32.56692	58.40916	49.09954	41.61177
PTGS2	43.21856	40.438	98.09462	38.64259	134.1521	33.90522	205.4427	150.0776	38.78125
TARDBP	442.3279	214.4472	165.1208	250.7313	142.2002	204.7798	142.2731	144.9639	388.1182
SCAP	6963.254	6382.594	5421.947	6184.94	4556.822	5302.82	3789.971	4222.064	7190.211
TRIM14	57.59565	804.4969	48.70653	65.29996	43.8628	70.45345	42.9269	85.41199	108.1505
TLX1	166.5861	136.6402	198.5462	63.11372	64.8886	452.4416	61.84849	67.97493	357.6609
EPYC	37.48632	39.91671	44.04553	42.83331	49.9467	78.7039	45.73703	44.44001	45.72972
FSD1CL	56.26139	77.83771	64.00553	123.8988	44.72878	82.24529	78.26728	40.42148	61.0064
GALNT6	90.34253	38.52724	52.56517	57.10898	54.45584	69.18842	50.35792	78.26054	240.3821
RPGRIP1L	50.08965	97.06699	43.69423	118.6188	61.95021	53.37152	58.50085	44.96755	317.637
KRT83	177.8878	102.2508	53.69235	137.602	65.72317	89.50955	59.16811	61.13613	188.8225
RAP2B	175.8473	143.4103	99.10151	167.048	166.5896	219.7277	98.65334	103.5772	238.1154
C1S	982.0842	472.3704	37.17115	322.6016	196.7451	37.05556	156.9306	64.21928	43.86275
SC5DL	786.9097	626.674	1184.221	809.3366	1742.446	2345.731	878.5304	1306.777	728.6642
NEUROD4	53.70422	70.17959	42.38888	49.19732	44.80739	48.83786	47.69502	74.86895	92.39874
OVCA2	146.1385	718.0851	149.8669	225.0069	105.0239	107.2611	54.75049	70.3904	81.15238
UCP1	41.72829	36.47302	58.8904	47.92168	51.25443	53.91498	40.80752	86.42722	40.94376
NXPH2	185.8255	138.9692	71.99668	102.5914	58.74177	75.56315	248.3518	183.0497	132.7809
ZNF268	39.13211	35.24839	35.68998	37.80333	31.91953	36.37012	35.02089	51.73192	48.88657
NFATC3	53.25168	41.55766	38.5237	49.68476	40.61133	39.83924	47.47268	33.01233	42.8141
TUBA4	33.57982	43.45734	37.25431	41.31302	32.91836	33.92316	92.72582	39.41746	42.73215
COMP	4671.262	2472.413	1641.811	34.35694	6820.788	1307.165	1642.978	4196.009	2575.736
BDH1	60.87764	60.88905	225.1195	68.82899	50.85804	50.95373	43.41692	56.12122	44.7788
BTBD16	33.30428	36.19475	222.2402	33.64677	37.65508	35.40285	68.25678	33.28921	80.85316
GPR109B	136.5144	217.0998	92.93162	227.9626	50.41787	165.6277	51.90953	34.37751	117.9784
CHST1	108.0297	90.2445	59.68024	69.92519	65.75499	87.73496	46.81383	44.49342	80.99863
ANGPT1	91.90807	71.97339	42.11472	111.1393	82.73465	65.05731	61.98957	70.16888	35.37694
WFDC9	36.54004	41.23361	41.79401	35.45681	46.51007	36.8517	37.99041	48.76098	32.6194
TMEM104	34.54958	53.71365	34.95414	40.31927	37.45753	33.12062	147.6403	37.89954	33.54092
CABP1	56.66503	40.41662	37.89602	41.46477	37.2868	35.73973	48.16135	78.69357	38.99116
TMPO	882.4879	692.0056	637.2441	1171.712	326.4099	330.3583	343.6534	386.6179	723.3197
UBE2D2	8353.175	8389.343	6719.533	8026.815	4069.225	5569.875	3844.183	3995.075	8932.713
LRP8	1848.593	828.741	960.983	982.4597	1102.526	34.36283	694.6746	612.0726	60.79168
POR	198.6964	166.3883	85.9298	96.33363	74.21729	90.19307	243.8564	463.4107	324.2904
ZNF559	597.2595	819.2692	1488.223	483.0327	1234.447	1464.885	2086.47	749.008	176.1707

P2RX5	70.48419	637.1319	65.8906	48.4492	31.50415	40.67502	38.22497	48.34616	38.81971
BRCA2	863.621	440.9506	516.6369	400.5209	109.5014	240.1413	160.1723	214.3383	815.6874
SGTA	49.92911	1407.785	51.31501	817.3317	54.86476	53.38589	302.5363	56.16829	2154.063
ABCC3	1348.547	786.801	459.882	418.2284	360.2207	931.7733	251.6365	242.2395	70.9344
KCNH6	61.46824	50.86775	48.0565	98.70573	49.7916	50.00519	39.91061	53.06149	76.3102
RAB4A	69.18259	52.88317	178.8797	440.7526	40.59949	45.26789	88.20794	35.69073	129.8735
TLR7	39.80628	286.4902	365.2141	56.03308	262.4499	98.84853	354.6291	189.856	68.51509
SLC9A6	65.87326	52.30516	190.5397	69.46172	132.3372	60.65543	75.04154	106.3349	999.7692
RAB24	3202.068	3809.214	4182.111	2937.049	3883.597	3246.174	3586.315	4778.678	3025.322
RARRES1	565.3994	548.582	452.0677	444.7047	322.2607	1112.41	337.9327	196.7925	946.0991
CCDC105	55.92835	68.6674	65.60017	42.95686	70.1432	57.9101	69.78111	53.88346	59.50987
FOLH1B	79.57904	62.4353	39.95715	41.26761	38.979	52.63876	40.87979	49.11457	81.22659
ADRA2B	336.7272	233.002	129.7903	225.6498	240.7815	209.7184	116.8277	142.5571	501.6142
UBE2L3	2785.691	4659.108	7969.438	5154.299	9817.484	6505.724	6530.629	8581.484	35.74261
FMO4	104.6566	73.37152	428.0529	76.60464	333.9802	961.2399	461.0562	137.5453	765.3105
PMS2	57.29696	431.1298	41.7072	52.34458	61.70388	53.89065	47.52765	56.26205	88.2734
GMPR2	347.1274	403.3647	208.6329	303.8225	224.0028	201.674	235.6202	212.7243	526.4283
LTF	152.81	75.71495	223.3026	118.8793	68.41605	83.87568	107.9555	67.10064	187.8824
SHISA4	216.2208	150.7183	85.29717	208.3932	92.84421	116.6619	75.69985	140.4369	446.8545
AFF3	53.09639	338.8003	570.1499	47.22578	36.73168	271.4989	104.6507	56.02267	44.34495
OR4K17	42.11725	49.46984	40.6436	57.50861	50.34013	45.48146	40.54757	51.91741	37.95415
ENTPD6	57.13314	849.1478	33.35732	931.3608	198.2555	44.62636	117.6574	37.27911	44.14341
GPHN	52.39551	65.10805	435.3297	454.3079	176.9594	36.49816	278.0592	268.5679	1421.406
TMCC1	57.73016	37.34599	43.75013	50.01199	33.12646	38.05379	37.92213	40.25887	49.60516
KIAA0649	37.53169	49.12793	904.8959	41.8854	954.5421	445.9305	725.0583	772.5046	42.0919
C1orf129	142.6942	119.1459	67.28867	139.3545	69.30704	63.02407	62.3331	78.12624	155.9763
MAZ	1104.106	973.3823	745.3987	880.3271	570.7342	784.0204	504.6028	887.3688	1325.552
TMEM41B	871.226	2385.854	4945.191	4010.547	7048.336	4172.459	5797.586	5208.308	2215.819
MGC88374	55.33212	55.88446	71.4807	43.9523	38.12961	45.43385	41.67761	69.3196	68.5453
NR1H2	37.83287	1218.755	46.05797	49.41499	263.2536	71.16012	167.3796	681.1866	3411.427
HIRIP3	7739.222	10882.86	11224.99	9554.375	12283.77	10277	11510.96	13075.53	10961.89
FGB	372.5206	176.8192	101.8513	200.1537	104.6403	171.4107	79.46926	108.1827	460.7505
C19orf24	337.5469	1151.399	353.8074	147.7202	604.0284	149.3797	184.401	1138.347	3245.159
TRIM21	1062.925	576.1169	2366.383	584.7035	1028.204	1660.445	2123.279	1692.967	54.4775
CASS4	38.32667	42.21643	47.52411	49.24492	183.3845	40.27877	33.89905	49.96957	86.0062
HCRTR2	387.4376	202.8775	108.4877	233.2916	118.0253	173.0325	77.08627	104.1399	477.6157
CDYL	424.5814	238.9618	1704.168	1152.014	3057.587	2193.074	3455.487	1470.23	434.3131
CCDC74A	89.83897	56.14114	300.2985	59.45757	53.84987	44.26993	48.06275	36.62543	60.56726
MAP3K4	67.54414	50.66055	2809.307	48.51369	6220.111	2695.043	5879.313	4102.904	42.28435
SMPDL3B	101.5738	57.47433	37.86766	57.23367	56.04763	53.55403	45.42557	60.42358	43.79685
TTC4	9439.906	11150.03	13132.24	10262.67	12846.17	12478.83	13167.94	15225.49	9011.437
ARHGDIB	22044.19	23995.72	28178.96	20576.78	24758.67	22875.61	25156.18	25639.25	20102.03
OR6S1	68.00652	53.9101	49.67105	63.40465	48.32907	51.33632	50.48808	46.04269	60.1869
LSM7	3867.691	1624.996	517.4501	822.105	651.2498	144.5835	352.0238	854.4095	451.0005
MME	42.66671	39.26358	291.5088	34.94977	39.49927	38.77282	77.70231	190.1057	33.27943
KHK	72.46319	65.59983	65.08885	46.80506	233.4265	35.51314	37.5806	38.33931	68.99632
LYPD5	47.00488	40.38884	34.28421	44.07705	35.51102	53.35216	39.53836	52.17141	70.92339

ATP6V0A4	33.6483	60.29173	253.5851	49.49253	114.5376	642.0562	158.2049	191.9906	1336.894
C9orf153	56.81044	40.18339	39.02777	40.41036	42.65682	50.49847	40.34866	36.61728	47.08831
GDF10	41.14894	87.14555	57.82015	59.91003	76.10963	180.4271	74.57038	86.98379	44.90812
RHOJ	3971.526	1945.348	1860.511	1915.167	1562.752	2361.223	1874.39	1810.583	2284.3
CHRDL1	34.39606	39.73587	45.29542	48.68331	48.74511	49.57709	72.17932	136.5039	48.03562
MGC44328	50.2172	65.3757	73.86846	53.46466	59.10444	73.20887	58.11378	56.81105	222.0684
KIAA1267	50.12371	908.689	293.3097	689.7703	443.0126	617.3617	229.9552	368.3578	60.45495
ZNF683	2118.126	1909.714	2207.334	143.5529	242.1808	2876.489	806.7402	1628.053	950.8158
HDLBP	35.83059	37.49089	33.44181	40.7654	33.46079	39.19878	87.23371	230.5177	40.0117
ALOXE3	248.5155	919.1473	207.8322	504.8981	1229.406	99.83014	460.4059	685.9011	184.9682
BCORL2	45.31162	33.09466	40.14486	36.24697	35.97652	63.24782	36.83248	40.4248	41.41643
VPS8	44.47937	47.28576	306.9203	84.28184	616.8263	595.4834	588.2529	45.55009	34.83696
HMG20A	335.8726	160.2777	181.9021	186.2497	218.7551	97.29585	335.9752	154.3817	416.0383
PEX16	45.46989	43.32533	43.00705	42.50433	40.52429	56.61908	39.12117	45.0743	47.60757
AASS	76.45032	76.20608	209.3578	103.5147	335.0759	635.3789	342.4697	145.7738	89.10091
MAP1B	59.87761	61.38163	39.32208	83.26337	38.53596	45.52743	44.01265	48.81332	40.15714
FARP1	53.82946	570.532	1377.078	1084.269	1835.649	1531.568	1852.542	1222.008	91.59475
INO80	779.9901	602.5075	734.1505	2221.384	601.611	995.9659	670.3928	459.4001	1038.379
TMEM178	44.4407	52.81918	35.17811	513.261	125.4848	45.26883	39.82933	31.41745	65.75465
FBXO11	857.3219	1342.175	1003.949	1018.279	958.8654	1427.782	832.3336	574.6472	926.9343
DHX58	62.8554	828.5229	3325.206	778.4573	1362.992	2386.817	3850.667	1325.483	37.32251
SPA17	115.9863	127.6874	94.76105	127.4729	125.4186	162.2381	87.52907	94.70756	107.1295
WFDC8	40.2084	49.24131	41.23591	38.65018	40.06058	44.5801	36.33794	40.84981	39.71715
SRD5A3	64.73629	38.56169	58.9215	60.60396	51.51723	88.81378	44.81834	58.70542	55.21739
FRY	397.9785	109.0808	143.3465	128.3818	164.2827	171.715	126.3123	173.1953	128.0677
CASP4	900.1531	1209.447	2359.146	916.0922	1917.099	2049.788	1801.506	2354.684	805.0617
ZNF600	795.1073	1161.7	732.5996	537.3022	466.1965	411.0888	351.2041	708.4769	983.5774
VAPB	38.26598	46.71301	150.4554	65.70079	117.8795	183.1266	84.5808	96.22845	36.3211
DCLRE1C	46.3732	48.71926	34.72662	50.98669	41.68054	38.71986	37.6064	35.72569	50.24066
C9orf85	560.208	38.99829	143.6881	321.0061	277.0461	295.494	256.0259	207.0558	39.08408
CHST5	118.7455	87.86875	58.48993	125.0975	62.34903	79.04473	54.60245	49.34414	104.3979
CBY1	37.16362	52.99852	429.3907	58.79784	37.88216	42.36224	35.36324	36.24319	33.73395
RILPL2	37.11913	47.74722	313.259	35.58323	221.9943	43.95979	140.1233	48.68967	44.83463
CHN1	54.95724	81.96185	54.07965	61.84354	877.7239	854.6764	495.2203	461.9854	77.66319
ZNF438	61.57817	48.11686	50.3965	59.20258	151.2153	42.23026	141.8367	107.3767	39.33376
ADD3	500.9053	467.5942	305.5214	149.887	124.3298	256.1366	113.7932	76.55051	259.8956
RHOT1	1001.609	2615.431	3681.499	658.2234	3166.702	4469.42	3808.862	1512.04	2125.507
ACSL5	70.22252	735.3687	46.6357	528.4456	39.14713	50.07317	89.82944	50.01316	46.19363
N4BP3	84.70611	59.5246	417.6085	86.86766	44.11001	47.22456	128.908	146.8849	48.95964
ESR1	40.59504	50.4465	44.50204	42.82647	78.60917	51.93037	41.81812	76.28075	181.7984
ATRN	53.87018	295.1409	33.76135	40.30134	119.8599	191.5405	85.62831	107.6561	46.73555
PEX11B	541.1811	454.6543	915.4744	440.4529	1329.182	981.2499	1259.871	834.7795	1096.844
CPNE9	54.22974	61.44338	52.73176	60.7392	61.59721	53.01421	47.0391	58.20559	41.85754
CPT2	76.05271	272.6108	454.8329	71.32225	456.0304	364.8075	581.9315	424.9439	97.29527
CDC123	1584.234	772.7997	733.427	1515.251	194.6921	1611.085	466.4469	1181.148	101.9588
ANXA6	115.9376	535.4361	865.8467	92.84443	998.54	632.1013	765.009	503.113	1798.963
ELAVL3	94.37708	68.27654	40.96954	41.32082	36.65914	49.97794	52.87639	45.38267	110.0783

DAK	46.18315	315.1647	36.94368	39.91086	39.79051	42.56249	33.4132	33.88786	39.54777
RUFY4	63.97332	49.41173	2317.338	57.35205	58.31363	1686.938	1634.125	2343.763	111.7521
DOCK7	326.0766	425.6129	84.98555	121.8077	115.6047	118.0388	155.6792	79.50602	351.5511
SLC5A4	40.75939	38.62854	34.63537	37.07065	46.25346	239.4878	37.44087	39.36212	45.0587
C10orf61	66.02912	40.74316	35.95744	39.93805	59.34238	41.30793	40.80277	48.06809	49.02253
PNLIPRP3	1571.226	562.462	2022.116	1748.348	3529.036	5114.104	4835.535	2050.089	1612.553
RRM2	46.60883	741.4013	248.0054	1575.028	202.5559	43.43736	387.0789	481.032	39.95728
KLHL24	1256.581	1180.845	1556.878	926.0178	2292.464	3731.611	2372.837	1766.276	1252.847
SLC37A4	4270.566	4151.292	6064.617	7136.511	5934.168	6042.5	5297.138	5561.099	4150.584
DBR1	238.2197	1020.643	617.9304	460.5427	232.4296	600.7142	408.245	621.9484	1280.451
USP1	44.93988	42.67768	35.3129	37.23516	37.81609	42.00788	35.93331	42.97426	80.61716
UPB1	89.04641	2191.764	1034.311	53.0606	726.9744	1473.676	735.8419	578.9651	69.5829
MPRIP	296.3515	125.0788	69.85004	91.84142	77.35959	65.23315	80.75878	186.0341	273.5676
RBBP8	70.62708	36.03414	89.90833	52.5741	45.36645	41.01204	89.03432	72.98773	38.18954
FLJ35220	2176.736	3304.224	601.3708	1209.995	1050.037	2151.635	809.6766	1408.845	56.89157
TAF15	5536.935	3315.708	5895.175	4861.748	8838.835	6800.017	8139.392	4048.054	4427.545
PHYHIPL	51.52658	52.11658	37.98832	78.38766	39.23249	55.02819	44.59123	56.01804	58.63328
LOC38911ᶘ	224.387	162.5077	94.24639	149.8044	91.26369	121.9002	68.719	97.73294	200.2758
SIL1	19211.73	24886.93	19597.53	20972.68	16925.1	21535.2	18737.33	25349.14	21432.87
NUDCD1	45.058	46.54611	147.9076	55.70792	581.712	37.03888	577.559	259.494	47.18306
PFDN2	429.4707	220.8869	152.7618	272.2017	191.7511	211.5454	158.5057	229.0052	452.0083
DYNLT1	162.7643	68.54939	405.9709	1775.427	128.6602	293.9004	320.8054	252.5485	52.27299
SNX26	7785.444	9869.962	5014.179	5840.448	2734.993	6115.264	2744.188	3685.026	7563.859
HAPLN2	241.8333	116.9782	56.90019	113.5634	67.42292	126.1728	67.69935	74.30504	228.4905
ADD2	39.4783	33.4055	42.96418	45.88993	31.08989	44.5387	33.18698	33.30925	44.63194
RAPGEF1	47.72927	34.79266	44.21041	35.79162	33.81652	41.88373	316.0631	46.26633	59.07435
POMT2	157.8541	787.7525	1460.674	1517.453	3451.245	809.9491	2285.602	2397.218	170.3384
KIAA0831	89.53633	39.09503	201.447	43.29491	121.7955	221.3622	225.9197	255.2764	52.16188
HAPLN1	53.06138	50.11169	46.00438	48.90384	38.59633	80.28707	46.71558	43.82753	57.26469
UBL5	327.3212	198.6179	99.87351	205.1241	101.9184	139.9237	84.3877	118.5802	448.4915
GREM2	41.69302	51.06562	44.35576	40.99547	34.66394	43.61684	33.51352	36.86684	70.75383
KRTAP22-1	57.4845	38.13967	50.34336	87.47308	56.12659	39.04419	42.86081	44.79465	106.6392
CCR1	59.94868	198.4836	97.97909	271.4566	57.59007	68.10329	97.93431	50.01234	131.8342
UBD	883.8757	1054.029	2091.433	55.1352	268.9651	888.1624	773.9254	1394.561	1522.649
THBS2	7019.363	4710.758	2629.664	4737.204	5470.333	5136.84	5619.624	7290.331	5524.264
FUT11	66.46149	217.7216	148.855	285.9727	109.2935	183.3806	90.79979	90.11785	663.5526
C7orf29	145.446	87.41315	70.15861	123.7949	66.89253	103.7928	61.43322	90.90229	402.1849
ERN2	211.614	166.6258	89.75124	191.6428	78.64031	949.3121	614.507	105.5406	351.393
ENO3	2102.813	5652.92	2358.879	2019.716	3237.025	3800.512	4572.695	6595.401	3248.018
YOD1	42.76062	733.1855	310.6145	64.59818	863.1574	700.8806	347.2762	459.4863	1267.052
HERC4	51.50658	57.26868	292.9973	268.4803	620.6106	376.4718	639.0462	362.4609	64.33586
POLR1E	36.86801	42.28462	258.232	37.85554	44.21946	45.24995	48.92783	68.73617	105.4127
VNN2	898.704	665.2646	799.6191	544.4553	381.1562	484.0344	442.672	933.8304	996.3571
HSPBP1	128.5745	90.99313	67.24725	71.32993	104.9557	118.7766	49.0621	108.3278	92.84876
WDR55	36.72532	31.75294	1309.416	850.2723	1182.435	839.6155	940.1037	709.3166	36.41314
FRMD4A	682.9831	439.468	512.2648	349.2022	446.5823	343.6253	345.6774	415.493	56.86994
DFFB	407.8694	200.4725	185.1085	175.134	79.78349	89.97383	197.1999	97.58251	357.2002

FABP1	125.7185	36.44831	34.28308	34.21529	44.99894	36.27061	34.67205	37.50774	39.6703
SNRPF	508.6158	42.76498	274.4421	303.4658	197.3716	108.4982	137.8958	185.7805	41.5857
GEM	66.26281	1338.387	526.2479	65.98041	559.1141	661.6462	369.0058	88.79507	247.161
LRRC14	4000.689	7898.166	6027.309	7479.657	8052.673	9835.268	7484.25	5352.678	7131.575
FLJ11783	217.1283	146.141	119.4683	224.7754	132.4872	74.75344	69.88192	104.9972	292.0557
SLC12A3	51.46972	47.09958	390.6099	51.83096	151.3345	338.5117	111.7342	55.12928	62.54984
OR51A7	59.41984	61.29899	61.63773	102.7128	63.48328	66.74674	53.5717	46.44621	90.15372
CASKIN1	222.0995	104.5822	69.92632	106.5493	70.7206	82.0274	60.33696	56.13495	104.1877
SLC3A2	78.41137	885.554	563.8745	1252.991	585.2989	628.8491	1133.388	559.6237	138.5135
SBDS	3219.486	3436.973	3188.236	3984.891	2775.348	2727.728	2672.748	2236.353	2808.732
COLEC12	86.17453	50.46233	33.95583	48.02948	34.38083	36.40746	38.23514	50.80017	127.7329
MTX3	48.26183	57.55336	723.6292	837.2249	592.8763	929.1982	210.7779	290.644	68.72217
RABEP2	40.32547	49.15583	37.78899	41.27476	35.72071	66.40784	38.71761	44.6419	66.88137
RAB9B	1459.247	49.40527	250.755	38.09642	318.7108	52.78195	187.9144	43.72192	72.38691
ZNF581	204.7761	112.008	64.35149	146.0496	466.1536	120.9101	389.2281	504.2961	97.81812
TPH1	135.5744	282.5153	55.55741	162.3448	63.79224	82.68152	73.68261	65.91161	287.4373
CCAR1	818.2629	587.725	516.7998	617.727	337.713	516.8982	534.653	358.0968	757.6125
FAM26C	33.64541	35.04733	35.06432	59.69069	39.81368	35.79513	31.71463	31.58841	35.30275
SERPINB1	2658.317	3525.786	7017.841	3317.309	3920.496	2974.418	4118.078	3664.771	3206.865
NSMCE4A	2104.05	3869.545	5317.118	1323.084	6214.549	4739.161	6009.176	4385.208	59.44349
HORMAD2	89.14069	37.92992	39.52773	45.12098	34.77103	42.11684	35.80232	56.87783	37.49214
LGR8	44.06772	41.57739	43.5535	47.84722	40.36702	37.40088	33.75043	35.47796	44.29804
FAAH2	185.2049	127.0098	84.37458	129.5892	87.50285	163.3389	63.75377	67.57903	61.81199
CLCA4	4349.564	1064.641	851.6928	1705.495	1008.648	888.0247	356.9627	999.8918	3037.856
MARS2	117.1385	289.748	56.36399	75.27135	59.1438	73.327	37.46859	46.54977	72.47997
LEF1	69.2876	93.17787	217.0038	126.5915	177.6077	342.1554	145.9252	159.1001	176.9066
ATP5S	131.4639	74.27688	99.18748	121.9804	84.46355	165.3294	58.03718	91.12578	96.50814
UGT2B7	60.48028	35.79974	44.17247	35.05256	31.31943	53.84042	130.5727	46.47029	35.87271
C20orf24	1803.371	1928.416	3720.766	986.1549	4925.456	1971.58	2650.162	3232.837	2352.619
HSPA9	4583.942	4413.987	4240.913	3776.229	4275.371	3598.85	3862.737	4968.83	5479.605
WNT16	96.77235	66.23766	53.89552	56.79014	63.49889	99.94975	57.18006	68.37885	91.36553
FAM110B	106.2251	2078.562	2073.933	916.0112	2606.198	2316.539	2326.448	2066.427	107.3271
KIDINS220	51.88882	661.2896	455.352	40.12394	269.9619	312.4424	227.7416	98.21482	31.30265
LENG1	263.7427	238.1434	103.3462	204.0641	97.28282	191.2235	86.12032	138.3339	457.8046
C8orf42	76.30235	58.98922	45.88851	395.8095	141.1857	45.14302	76.08472	77.56206	65.03735
ZNF546	2914.309	46.55692	269.7997	34.32945	471.0822	39.20745	125.674	34.76891	33.2705
AP3B2	42.30077	46.3858	41.22683	58.88186	144.9245	40.14545	103.8499	184.6631	36.01415
DPYSL3	9414.956	8848.525	9619.75	7006.515	25667.36	12280.55	16998.32	9361.775	7255.761
TGM2	3986.944	935.7005	2793.267	8733.292	595.1062	2358.757	1611.592	2416.921	4136.796
PELI1	94.53889	78.93323	611.0238	66.09435	391.8572	474.6493	311.3049	566.9146	301.3002
SYPL2	61.18745	52.0122	52.12087	103.7469	59.38787	50.3321	72.19905	176.4265	62.21404
LTK	42.7751	39.12301	39.84013	49.78936	129.8953	37.78582	46.19063	49.21714	46.51582
ZFAT	60.22487	66.65404	35.60519	81.58165	55.91185	77.91726	51.87964	44.14998	87.42287
ARSJ	73.83545	74.77353	44.01427	92.73024	129.2744	53.60013	45.3275	44.66777	49.83397
PSIP1	1415.323	2399.722	1632.157	1669.551	671.0872	1235.653	795.0362	1324.444	2497.901
SH2D4A	2691.121	2050.609	2377.797	1408.569	4850.498	2445.091	2238.271	1920.087	4275.142
EIF1B	52.47035	55.88672	115.2225	280.6424	103.0024	94.65908	104.4554	78.95769	53.00572

C3orf62	60.05227	70.7511	53.08057	91.01933	56.36807	110.8512	58.36786	67.87064	292.9118
SPANXN5	61.14966	44.03514	46.64656	40.75903	38.8565	39.99279	34.14852	54.71637	44.0332
ZDHHC5	1531.424	2674.737	3609.67	2774.02	4059.717	3461.146	3534.302	3664.302	1482.567
CCT2	8401.404	7346.844	4846.615	8803.604	3594.334	3666.474	3900.991	4443.288	8659.875
MAMDC2	42.91018	238.5022	133.1694	50.57819	981.1866	205.944	242.8931	81.29208	64.84174
TRPM4	16832.5	20260.9	16098.56	20784.23	17061.56	19012.73	14294.8	18775.48	20055.27
DDX56	42.85077	4509.268	4295.399	2450.656	6275.478	1594.34	5666.057	4187.582	34.72356
DDX11	80.65937	67.56242	60.53626	67.57137	53.45955	447.0543	50.70591	47.54129	52.87025
UPF2	36.67777	37.13987	42.45308	31.8307	44.43385	31.92476	33.8284	36.53841	35.18923
CHIC2	1669.393	1441.404	558.7264	885.4258	956.8707	38.0841	849.2113	848.1844	91.13485
MLF1IP	41.9212	63.6177	1315.713	940.131	1854.799	1506.552	852.2097	1682.163	41.01516
PPFIBP1	1301.691	497.2111	1121.006	1975.745	855.5682	1157.751	1535.144	1055.167	838.3999
BNC1	48.54908	36.96165	49.97258	57.75596	46.03252	50.6384	80.96408	50.61639	33.43578
GART	95.89292	1606.79	834.6773	295.6072	1544.715	1041.736	1963.18	1582.185	89.00745
TUBA3C	52.2636	39.73873	44.689	78.774	45.61996	51.47961	36.36994	51.4446	273.4459
INTS10	38.08714	49.84396	3527.413	53.95954	8015.85	2451.81	3911.548	3221.788	116.4621
ULK3	77.24608	317.0042	68.71832	353.9484	52.61428	86.38036	52.31008	69.00031	60.78121
CSNK2A1	43.42	55.44255	304.8343	604.3771	134.6851	43.38251	93.60201	35.81094	61.21857
AGPAT1	166.7677	59.3332	69.98739	57.10125	535.3473	63.30538	276.5368	329.2338	280.7842
IAPP	69.84033	33.47052	50.62834	67.32217	55.8815	58.39813	46.86161	50.29221	70.7705
GRM3	39.23925	48.56977	34.24844	35.34425	39.54508	40.27168	35.83408	36.92492	125.5677
KPTN	283.893	91.15829	86.0565	193.2208	76.57723	110.5719	59.79703	93.50526	152.6381
LOC28387	4491.176	5799.021	2722.839	3053.908	2284.671	3100.846	1684.542	1736.478	6257.622
SETD3	65.52146	40.49698	47.02493	59.46116	38.57047	47.93832	54.82562	40.43085	53.42627
KCNIP4	53.22937	36.0423	37.25882	33.3135	35.03796	31.74815	33.07124	50.4039	41.92176
GRIK5	328.4225	165.4484	89.22413	148.0423	85.08296	154.829	58.02869	87.65813	496.2451
ACOT8	31.09871	297.1529	107.4171	309.8904	104.2344	56.41802	36.27895	34.94909	56.02624
SGSM3	100.8642	111.2998	220.3446	722.6878	380.0954	448.8445	477.8952	565.611	85.5649
FXR1	2909.47	3595.358	2739.795	1321.597	2421.246	3680.726	3092.381	3354.149	3887.197
FAM120B	7365.823	10391.48	11496.37	11170.82	12224.01	11942.36	12775.8	10738.27	7124.579
CHIA	37.66376	38.95302	44.07791	48.30701	39.86992	48.44028	38.01117	35.99372	35.35226
WDR82	5609.227	1263.33	1456.68	3386.381	413.1472	387.2537	731.3812	578.6459	3172.283
SCGB3A1	67.50983	51.33866	47.13452	56.36926	45.86405	45.67433	42.00129	55.11184	76.38635
FSD1L	111.6731	116.0544	50.45271	901.0383	50.18627	66.77517	51.58249	68.5723	269.2953
IMPG2	182.1029	202.9529	90.60513	192.1674	77.26156	94.77985	78.68427	104.5832	248.9922
TMEM87B	128.0061	97.08366	59.57294	67.34507	278.1855	79.83972	263.4453	84.83895	195.7075
MMP27	43.76161	64.05949	54.93789	57.75546	54.37524	41.7025	48.55582	50.92742	47.98399
EXT2	34.06178	36.02316	33.68438	33.31971	37.60419	37.29261	45.37106	45.37733	34.93012
LOC72939	40.38423	52.2465	39.05551	43.80432	39.7776	35.12344	43.78799	50.16611	53.43713
CYB561	167.9735	134.9983	61.79889	106.705	49.91261	49.92968	47.08168	104.4628	222.3529
SLC15A4	123.0066	80.72806	59.57379	52.46722	54.05244	76.89384	88.28764	39.93583	53.76189
ADRA1B	157.8012	495.7474	342.4006	86.02003	201.8657	339.8517	327.1325	254.9746	776.8478
FLJ45983	188.5063	97.44099	68.32394	54.29633	54.48055	84.38397	64.43233	74.29298	75.21387
ENOX2	35.93394	38.02449	35.5936	44.94045	40.15681	65.50981	40.94845	40.98055	50.41332
BLOC1S2	166.1969	126.4988	67.77151	68.22002	64.6384	572.6777	64.05299	345.8416	250.578
TOMM20	4589.125	711.4267	1594.043	3636.229	890.6183	1777.801	838.4948	784.7988	4025.402
TLR9	57.5878	59.79131	1250.861	38.74822	97.96397	945.2422	129.7984	343.3592	39.95293

BRIX1	47.83817	2407.009	2028.705	1783.092	1862.597	1139.923	1486.02	1400.644	2129.955
GSTP1	7413.781	8532.577	10203.7	11982.14	12847.46	9884.527	12258.69	13379.5	8760.563
MBD3L1	56.77008	44.36345	49.88	39.90121	47.49044	43.18221	33.03239	47.74147	147.7016
CST7	294.8284	683.0485	756.9506	207.435	271.5009	457.0044	762.9949	1031.526	463.4702
SYT8	90.04575	57.83402	57.65088	66.25697	52.47188	70.17328	39.80487	50.23417	71.95417
SGPP1	34.47882	43.26165	37.6518	33.28349	35.16968	78.47345	37.11066	53.11948	42.47224
WBP5	6159.339	6670.864	4983.79	8007.286	7022.952	5997.712	8123.824	5773.859	5017.226
OR11H12	63.37654	96.54247	44.09596	107.5123	49.59175	47.46863	102.4425	49.30418	118.3663
THOC6	46.45847	37.29221	308.3551	48.74323	390.7769	34.27435	234.8691	35.21943	36.05698
WRN	228.7501	455.3795	1217.411	478.7095	1133.021	704.092	1024.861	815.6566	1900.951
NAF1	84.86665	100.5228	44.9078	78.37505	69.44835	40.01102	63.85242	64.60923	129.8381
IGFL3	3303.538	4178.921	1046.003	7585.825	2412.912	9833.653	5135.697	2202.394	2114.068
ALAD	32.17851	48.44384	34.95569	35.43505	36.17874	154.6392	35.4197	34.02156	37.43982
PPARG	1616.828	1275.461	376.9424	52.38094	246.3365	46.08014	55.14728	53.81699	63.05232
DPH3	64.27851	55.47346	65.43903	54.67593	311.0571	448.4007	192.2981	61.65344	111.8631
ASCC2	16854.3	16968.98	18350.26	16918.52	18530.84	16883.98	16840.37	18286.07	16995.63
KLF13	184.0273	128.8177	85.31216	131.7178	339.5412	429.253	435.6889	63.82982	120.3506
DBF4B	249.0336	57.58266	57.89102	52.08917	48.20915	59.8983	45.33966	53.47831	34.62011
SLC25A23	13544.93	12914.97	11795.95	10369.32	10767.83	13787.14	10017.55	14138.28	15908.52
TBX6	41.20432	60.65359	54.83511	53.62921	50.81546	60.07818	45.01973	44.19632	37.15795
LCE1A	37.83203	33.7877	37.24917	36.44503	35.68764	43.82891	36.49424	43.16549	90.64428
GPR31	62.48839	413.9038	60.64396	65.69872	57.09616	69.79811	58.06291	62.79189	124.3371
NMS	51.8639	47.20837	42.48123	51.52858	33.73181	40.37344	39.80002	65.15848	37.96141
CCDC123	199.5081	115.1351	286.7689	145.3956	72.88855	95.246	71.24286	87.07542	115.1551
OR1J2	160.9662	94.19702	58.28183	94.35903	77.06246	103.3165	60.86055	56.98092	205.743
WBSCR16	85.56326	1452.316	831.0219	672.0419	342.5731	446.8022	205.9581	340.9244	1059.621
PLK3	134.8413	110.222	107.9644	103.0324	122.4757	171.5574	103.642	123.8609	187.7469
ZCCHC12	43.86016	34.36249	36.79904	60.21714	35.44284	39.56911	33.58069	35.93808	59.68953
MPP6	59.65167	50.94297	364.6977	51.05916	167.6702	394.9431	149.3861	58.16738	36.56853
KIAA0753	73.54483	57.51233	119.9252	48.75082	184.5183	53.9175	175.8671	45.14186	51.49383
VSX1	44.67861	58.88582	46.96293	51.53896	41.6923	35.13751	44.54328	55.21861	74.81155
RIPK4	520.1843	239.8652	137.1605	277.6546	192.2898	189.1412	110.4494	161.2952	586.318
SLC7A13	45.37548	32.54388	40.7076	48.13536	51.15947	37.11956	38.54864	37.57347	61.85725
FAM65A	6750.867	6060.079	4245.706	7835.653	4012.499	5948.181	6043.868	4380.421	8005.583
LAMB3	4443.91	1800.481	1371.599	6121.37	893.4153	2079.932	1357.544	974.1644	3491.185
B3Gn-T6	298.4898	200.0096	100.535	211.1098	111.7293	150.9214	80.7564	117.0939	444.4176
POP7	2069.692	42.42202	1189.224	1845.454	1678.797	62.6945	828.5432	837.0494	39.47455
HEMGN	38.1142	38.96613	40.5694	42.34709	32.54532	44.10566	47.0304	36.7754	43.74338
C11orf45	66.56317	40.3262	46.46759	85.41291	231.4394	43.39938	138.0603	51.73499	74.16711
SLC35C1	47.14981	346.7496	653.0514	119.2928	738.9658	390.8375	874.3103	371.3956	89.27203
COL4A3BP	2392.739	2386.312	3928.581	2810.279	3755.047	3290.019	3335.275	4175.447	1933.363
C10orf116	38254.48	37631.04	34651.82	36599.09	32388.6	30288.61	33494.57	31729.57	41136.41
AHDC1	1881.367	108.1612	1355.652	101.2847	1253.767	499.401	1060.314	1020.36	96.91686
NSUN2	3220.652	4861.496	3602.359	4143.819	3205.758	3609.925	4759.079	1959.782	3128.848
DYM	94.40505	61.17894	197.7561	34.96228	558.1208	43.21019	295.1671	409.5892	89.63724
CADM1	129.7029	3060.058	2683.545	212.6779	1345.903	709.7816	1681.028	196.0164	63.60361
CARM1	11887.35	12060.87	15936.77	13810.51	15101.65	12073.69	15164.8	15728.83	12480.77

MAFF	33.61356	39.79506	108.2178	308.9576	144.4582	157.6868	115.9624	42.18545	39.00122
HLA-DQA2	105.5203	344.9774	121.0376	361.3114	86.54159	275.8987	52.49551	61.6917	879.3739
SART1	35.87196	48.4603	35.20779	69.60024	44.6116	48.39581	36.37128	44.44291	46.52206
MTERFD3	105.7227	67.82529	39.84316	71.2615	101.2601	50.01611	68.32916	141.652	93.67119
MFF	1031.53	35.94007	722.9336	36.26928	870.7026	802.6732	655.5748	455.89	1651.552
C13orf28	56.23476	74.15809	62.89113	49.41186	38.38522	43.21577	44.06213	52.95101	68.25384
APOBEC3A	17873.87	30222.16	20005.31	20375.29	4642.213	26454.82	22495.19	18476.57	18170.05
PAXIP1	317.6966	137.577	90.49379	158.6161	272.2256	231.9223	239.6078	174.3651	156.8017
LAPTM4B	906.2023	672.5038	493.0698	1095.354	251.3635	320.8111	332.5726	567.2468	2574.838
GEN1	205.878	142.0436	113.7278	139.248	55.86305	100.4278	112.8862	146.8979	235.3159
PNPLA8	173.2223	318.7111	314.3471	201.5888	347.172	288.9037	217.3035	209.3272	314.9357
OR2L2	63.3254	97.5137	40.92512	57.25501	41.34241	40.54704	35.24636	39.00774	39.5767
FAM119B	31.76667	31.06844	33.68616	34.38714	37.21982	38.73926	77.23412	42.76339	36.65812
NUDT9	39.72934	1314.685	462.7689	806.0596	435.4074	658.088	408.1507	338.4149	916.8779
MYOCD	48.41265	50.40403	40.98919	48.91052	42.4489	64.19231	36.38544	49.71752	38.10575
USP49	752.2941	1493.601	926.1172	617.0787	370.8838	343.9003	525.0049	798.3984	957.7469
BCAN	346.4149	166.2295	79.09929	172.8043	94.16709	112.6465	68.18932	76.24747	415.5362
ABCG8	122.7517	69.62323	51.32943	73.66663	49.13528	58.64672	50.28075	50.23292	118.1304
STX4	862.9485	1541.667	1445.961	1077.533	1126.884	1244.468	1285.388	1760.554	1029.592
TXN	2855.964	1437.34	1131.884	1166.539	2545.465	898.8835	1587.735	1315.119	4142.125
CDKL5	35.69136	35.89798	38.51116	41.09274	46.24639	38.32726	34.08185	38.51123	41.89864
GBX2	176.3928	143.2391	85.63271	145.9763	81.34208	85.94695	165.7412	45.27202	351.6863
TACR1	102.2899	49.1671	35.78568	40.63168	32.58358	44.48824	36.13248	34.79302	33.62521
PDGFRL	2169.129	968.4909	1842.793	94.67617	2867.529	2777.831	2491.997	2550.801	444.5023
MYOM1	3545.359	12416.59	6810.042	6463.388	5795.06	9020.488	12388	15626.8	8821.157
ZNF317	130.9268	2333.136	3797.067	3296.056	3965.531	3144.013	3726.561	4082.439	2118.154
RIBC2	41.46521	40.42182	42.79934	35.59954	35.45418	40.68129	39.98693	36.05824	38.27433
NSF	833.6893	716.4268	2843.373	626.7923	2907.031	2605.135	2781.497	3130.85	835.6249
CD109	55.12461	61.38405	60.63258	95.69195	83.53592	73.51412	44.88962	49.138	68.53632
C10orf90	50.68488	37.19836	54.25807	76.74237	63.77311	63.16839	51.67368	39.25418	43.78809
WDR17	123.0852	121.5241	72.90373	74.67914	53.98877	61.1865	41.48551	55.23238	61.2421
RBM45	953.3318	1224.01	2719.939	1853.494	2352.38	3272.525	2582.201	3254.211	747.9794
RPP38	68.0097	73.54302	216.0574	138.086	69.6344	59.90807	65.22772	188.5618	71.41111
FLJ45337	172.7937	86.51302	68.55962	167.869	53.32523	215.6961	99.27599	44.9027	102.5149
SNORD55	9305.679	6826.754	4341.93	8100.827	3413.542	3987.517	3731.923	3501.26	8832.021
IL8RB	1157.175	109.8619	263.498	167.1161	85.43378	102.4055	64.59277	72.09789	277.5832
SSR4	476.8122	768.3583	651.8577	521.5244	1149.333	533.9135	912.9817	1034.161	948.5974
ASB7	976.4791	997.6888	322.3347	746.4916	202.5456	651.9845	314.7112	329.5794	832.74
NUDT13	45.84024	36.12918	40.79133	35.2046	44.54421	44.96423	77.31551	39.90612	37.08826
CTSG	23052.12	22903.43	14133.02	13924.89	9870.82	16437.87	15529.4	12147.1	25530.53
MAPK8IP1	64.34868	52.41314	41.52854	232.4059	36.41981	36.07471	109.4604	48.87714	68.32372
ITM2C	180.3068	1929.713	1077.705	162.9205	2063.305	502.6817	1924.979	1262.198	288.2259
XAGE3	83.59081	82.34503	60.13591	65.82645	61.1575	54.69063	50.55682	56.78986	60.73135
ARL5B	66.15072	49.30418	52.704	50.18626	115.0178	59.39506	60.77234	53.30712	156.1163
FLJ12529	66.35849	52.45812	56.66427	102.7511	49.02912	50.3299	49.08656	63.93024	64.12871
AVPR1B	137.6572	57.49438	56.46934	73.45063	57.90791	57.61927	48.45846	58.43215	100.0468
SSX5	222.1615	66.42388	61.58184	182.8394	66.81128	64.26024	55.82249	58.31783	65.08045

RTKN	3610.488	3355.773	2755.305	5154.666	2284.963	4416.36	4586.908	2818.567	2295.133
LOC440248	76.50928	57.50395	40.35913	73.15028	109.6238	43.94524	149.3196	50.45475	58.47421
PLXNA2	362.2993	168.7491	93.43713	228.1922	87.06704	120.3358	83.22851	120.3339	445.9111
MYC	1436.946	843.3384	463.5734	1783.814	291.0194	567.6784	475.6065	504.2638	1434.599
CREG1	2417.281	1654.836	1315.792	3389.755	2001.16	1274.457	950.0295	758.0032	718.303
PDIA6	1574.78	1016.962	295.1057	489.3154	176.6356	790.2324	294.6137	231.5662	383.0605
ANKRD20B	49.48485	46.96613	51.63121	38.12932	41.74174	72.92384	35.01639	50.7072	45.07256
KIAA1333	634.4117	188.0057	268.5781	391.3408	253.7181	399.8837	339.7017	164.984	759.7939
MIOS	313.6621	185.4685	1163.33	466.0016	1870.227	1004.386	1504.476	1381.688	423.0709
SELENBP1	295.1579	180.0341	166.748	176.8693	262.3079	192.8592	222.2635	113.3181	160.3596
C11orf63	188.597	162.4023	91.06953	57.54645	133.9098	109.1544	283.6024	88.6436	217.5354
NT5DC1	751.3096	776.299	872.2223	523.6847	952.4916	503.7739	624.7357	595.2849	1009.969
MRRF	1370.87	2972.433	6401.163	5060.569	8081.35	7181.78	8821.157	6619.152	49.72906
TADA2A	263.0285	120.1361	138.0687	468.3946	148.4782	186.4855	124.9804	144.2575	307.2919
NUCKS1	3827.17	3039.176	2042.405	2677.932	1580.579	1904.092	1820.896	1934.91	4970.939
ZNF419	4118.796	4346.653	4404.838	3683.863	3969.766	4490.854	4700.081	4155.311	1188.146
MRPS15	1231.111	1484.701	4974.664	1931.919	6233.64	2866.593	6097.774	7233.53	4156.767
LIG4	770.0955	359.1413	157.2385	559.3943	301.4948	263.1298	153.785	188.9635	1051.236
CAPZA1	56.60922	50.91024	44.04088	48.03325	47.31219	40.06916	50.87749	51.29014	53.61064
SYTL5	254.7046	155.6761	66.68655	139.3125	88.96518	99.97629	58.64317	75.51447	259.0358
GLRX2	594.4409	398.5425	416.5028	526.3413	404.8939	282.8816	468.2283	408.377	664.0768
B4GALT2	66.57872	65.86068	43.40673	59.65563	34.97245	58.1198	51.73619	54.71707	83.23434
C5orf13	2568.938	1065.489	1065.612	887.7963	1374.669	1647.724	1177.379	611.7026	31.08514
HOXA1	51.23461	51.82814	36.09771	40.00144	44.52107	234.8269	89.93671	35.53126	44.72912
A4GNT	179.3378	127.819	93.97446	225.7286	78.13957	126.5129	68.08276	94.04104	164.9456
LRRC26	2205.784	3197.356	7833.203	1584.642	572.2378	2157.279	2099.511	3114.342	7956.491
UBQLN2	862.521	753.5506	1836.734	454.4698	2722.578	1738.661	2751.095	2205.664	55.06714
C14orf135	760.0211	185.0329	244.9737	442.9254	93.8477	287.0278	210.6499	271.6684	1040.145
PATL1	158.2146	473.1083	177.7653	573.3528	345.7548	548.7262	573.5588	406.4197	315.1823
IL28RA	865.7643	1937.92	751.7068	773.418	925.7005	1436.282	873.6152	973.7305	1426.026
EDN2	53.12027	66.36137	121.7453	57.63742	60.54752	59.14956	50.74291	40.4968	76.50059
KRT27	62.69907	57.45315	37.22609	62.69321	376.9149	41.61639	33.41396	37.08879	36.37019
BAZ1B	50.4791	94.24007	46.7776	87.55868	164.5865	42.3759	380.1571	226.0448	34.39704
RECQL	217.4924	80.98953	75.7886	175.2896	60.54118	119.8685	72.49479	86.0895	45.92561
MAMLD1	991.6135	119.8632	545.3044	884.5946	572.7058	529.9431	362.1331	256.1821	237.1466
LEMD2	5686.793	6493.765	6905.359	6303.817	7439.411	7630.545	8143.109	5513.412	6837.446
TUBB2B	3389.332	1453.889	2529.355	1134.52	8682.532	1491.833	2132.226	3265.551	2894.705
OR5H15	47.38467	41.82418	47.13018	78.75126	40.56108	39.70685	43.73809	37.67214	84.86273
TREX2	41.5495	40.61446	57.10187	76.51181	370.2435	54.27485	141.6656	78.53587	83.97121
SLC9A3R1	662.4136	509.2395	1150.779	321.4658	927.4903	728.3431	682.4653	1637.8	708.4945
CASC2	54.24612	56.95393	52.90213	58.84941	80.94212	59.66843	42.46995	44.79313	71.46763
DNM3	55.55346	272.993	48.12231	49.97945	68.04645	150.6806	68.82013	41.4346	49.68204
OSBPL9	47.3704	47.99264	37.41329	53.87486	98.09529	42.31477	42.39364	126.7329	46.10036
HIST1H2BC	3997.534	2439.82	2724.601	3091.511	1588.889	2277.595	1212.206	1541.388	3436.282
LMOD1	85.17783	800.5394	49.39443	69.63397	161.3112	57.43225	222.3484	506.6953	118.7541
AURKA	89.95584	97.74427	1003.323	486.6722	1176.307	896.1363	1193.082	941.491	86.39021
LOC728454	3411.39	3495.465	8494.947	552.7666	26066.15	1680.792	8636.69	16924.39	5005.626

CIP29	833.8227	413.5454	1047.674	39.34896	1198.56	1845.026	1122.521	1661.357	38.29319
FOXF1	117.4873	71.73312	73.7674	93.94693	78.65289	172.6873	73.5397	55.0808	124.8397
HAND1	107.2022	44.66563	47.97922	97.37027	44.57987	40.33735	104.0615	47.90495	76.97186
TRIM10	53.27616	80.99202	42.30085	48.53371	38.77721	34.42872	49.40604	37.04471	74.00945
ALOX5	5425.109	1087.039	4769.496	645.202	5776.323	3537.996	6082.812	2542.07	1188.291
CGRRF1	179.1108	129.8144	73.32146	129.871	184.8985	98.60863	92.60651	126.5352	280.699
SPNS1	1731.028	1138.513	487.193	1134.876	281.3271	923.5125	479.8114	430.7842	1593.936
C16orf54	316.6797	151.1127	88.94874	164.0339	88.84545	123.9499	80.16372	98.3909	320.4858
ALLC	331.8502	149.8645	92.72239	159.5866	92.62392	141.4242	76.86719	109.083	165.8648
PIK3R5	277.1427	89.1695	79.19735	135.078	90.42353	114.2545	61.79816	75.66723	51.10635
AGPAT2	804.1022	146.0321	79.88422	632.2086	144.8776	272.0182	168.2124	547.6329	1247.907
SLC7A10	124.9476	86.4484	143.538	104.7371	248.1059	60.03225	99.61283	216.4063	83.8376
43525	105.9761	84.41988	147.4722	149.072	70.84248	64.78002	118.1323	64.14938	47.688
AQP1	186.2056	835.1185	496.5884	120.636	455.5635	303.1095	349.9493	595.3824	1150.827
NPHS1	64.6965	76.1367	56.46883	63.08435	36.3749	69.12646	55.41473	43.64246	168.6028
CAPRIN2	971.2446	802.6512	789.0914	2338.396	868.4148	62.682	687.8237	583.8445	147.7622
C19orf48	55.1754	66.4587	57.98196	36.36535	50.51767	39.89243	48.53304	44.96114	62.04768
IL12B	44.81932	51.61293	55.40113	74.23319	57.48455	48.14167	98.80034	52.41129	45.19857
SPATA2	42.31452	60.00691	51.20967	77.76342	43.96944	55.77671	134.8588	59.7645	85.82964
CCDC33	50.90413	135.4117	77.60078	74.85141	113.2469	106.0597	65.23915	117.769	189.9523
ZBTB39	116.291	144.3924	89.79985	447.9095	449.2998	546.1522	366.1315	268.966	209.434
CCT6P1	40.30725	36.00071	35.56911	81.98341	39.24404	35.07485	57.81311	44.07222	40.31423
KLF14	46.70693	51.71084	34.51257	85.08469	48.63792	40.26484	45.03096	54.13018	38.05803
OVCH1	74.09019	86.31612	59.57562	61.41098	49.98479	51.33005	47.94774	51.86579	180.4848
TWF1	559.4581	575.5327	797.6075	687.0346	607.7838	336.7134	595.0149	908.8129	225.0511
CACNA1D	229.349	75.23422	76.17902	108.9679	64.71307	94.34911	62.40847	91.76346	179.5804
MRPL4	35.84147	50.4884	510.7086	51.66711	33.3045	36.39123	38.50655	509.067	38.89698
TNRC4	65.9878	50.52682	53.52712	33.9862	47.76482	56.49911	42.91335	54.09589	47.99793
CLPB	814.3995	57.13076	57.67255	36.3834	114.0908	46.92308	90.05334	59.15253	73.54488
MYO1F	40.52374	34.40746	37.41988	49.2737	37.46534	42.65623	122.8749	35.02216	37.4185
BLOC1S3	46.63377	70.72684	36.34443	36.96182	38.95918	39.65588	35.41006	33.66111	43.99529
KIF21A	797.0728	319.3158	481.9189	1218.159	1920.538	568.3265	564.8154	749.6508	933.7986
SPINK4	68.04051	50.4977	48.80326	49.98788	37.44217	46.45735	69.16925	49.28915	60.15448
DYRK4	65.28499	49.41343	615.3782	63.87498	562.0741	747.024	766.8787	340.3828	57.04658
HIF1A	2913.942	2892.724	1645.44	3144.866	1050.081	1548.093	1390.792	1254.992	2607.534
ICAM4	38.71159	49.66425	48.73473	53.5452	36.53385	48.6073	41.12206	39.8359	42.74272
AIM2	2668.098	4093.05	3214.555	2847.774	903.3447	4104.156	2918.914	4415.758	2199.761
PAN3	521.5258	321.1418	189.5928	331.9455	201.7052	249.8206	193.6286	261.1695	742.6793
MFSD7	831.1774	453.5039	171.192	85.60288	442.7711	287.5808	203.8278	271.6147	802.8452
MRPL49	40.02308	44.38749	46.20189	46.16074	713.8359	43.95573	423.4013	259.57	33.36575
C1orf41	46.75838	33.38704	76.72914	52.57743	36.66162	34.48003	54.62762	39.64214	117.3108
PDE2A	247.9158	144.8063	124.0154	128.9881	194.284	101.7649	98.86013	68.22118	142.8737
ZNF642	128.62	109.0173	97.67671	43.34758	85.83125	50.55542	63.52373	55.21082	67.47878
ALDOA	242.3672	129.2094	75.72141	137.7584	79.15398	119.3395	70.30602	105.6904	183.7759
SIGLEC7	198.7407	117.0511	85.06793	147.6907	84.2403	125.4546	75.34718	70.74032	231.2194
DCXR	19932.04	16509.08	13515.23	15756.62	14919.17	19519.91	13074.45	13817.24	21590.75
RP1	31.92743	38.92665	33.28673	34.41033	41.5831	37.91116	37.38446	32.84112	35.74393

FOXL2	33.77775	38.54982	34.20651	32.60546	36.40371	41.35277	37.79922	39.11588	37.28357
OR8H1	46.0237	36.46284	35.0167	40.95513	45.6451	46.16003	42.8732	52.50374	38.84533
REG1B	59.8947	45.23667	195.4902	43.3785	48.69191	36.49495	574.014	1853.52	2264.351
RAB11FIP4	49.02362	41.9696	46.76085	36.88675	57.37782	59.29235	54.28656	52.83834	60.4113
UACA	972.7964	623.8328	1964.972	797.9457	3512.228	2786.988	1263.085	2836.382	786.6844
MCM2	57.55745	48.52994	255.2408	43.056	502.9438	34.89906	554.8491	371.6342	42.53507
CRLF1	177.3067	158.8113	88.46134	130.4448	82.11348	151.477	82.1686	87.92048	351.5081
DGAT2	275.2184	193.4924	88.34541	75.18808	103.4645	143.1605	72.48747	121.4845	137.8208
SLC5A1	2343.478	4423.081	2645.985	2388.437	4800.775	3063.233	1389.83	3661.828	67.53159
HIST1H4E	61.21798	44.32712	211.3835	925.0684	183.7326	295.2802	261.8716	60.30926	68.032
RHBDD1	218.978	864.8735	877.7987	187.1505	1184.579	505.6874	827.4671	538.1266	301.5684
IQCF2	83.15357	44.213	41.59983	75.12968	54.8338	37.02674	50.43501	43.03241	66.08253
BACH1	138.9702	502.5247	69.89529	166.7355	85.76347	316.2881	69.81142	312.7649	397.2642
LARP1	1639.686	1554.085	651.5164	465.7138	476.3607	593.0356	428.9239	366.2751	40.52311
PTPN1	41.30408	1012.114	746.522	1221.215	1257.683	632.7652	1629.493	933.6326	122.0577
PCDHGB7	52.8254	32.68847	47.25534	39.21919	33.46568	44.15928	38.36892	40.58654	71.95845
SUCNR1	67.3321	50.80795	36.9803	48.15665	64.01533	40.98159	39.57593	49.64179	55.91788
UBE2A	35.63893	34.19829	36.35577	43.08976	36.59692	38.49032	38.61253	41.03302	82.49419
DMBT1	49.07153	61.06373	45.75139	48.66403	49.44379	54.1422	41.59135	56.02224	62.03296
ZNF277	69.41838	42.09236	198.1647	42.70208	198.2174	232.8094	182.2422	120.3994	52.67324
PXMP2	66.78101	74.51332	620.1436	843.739	420.1223	1124.201	884.4188	681.714	2111.313
DLEU1	44.24052	77.57822	162.085	42.47528	331.3624	45.64428	151.388	289.8016	77.34172
BIRC3	83.78333	61.02942	533.9584	639.1169	170.4355	311.2134	495.9951	426.5352	65.23126
TPD52	41.20385	36.43708	30.62049	29.41955	38.07153	33.62252	33.98203	31.09129	39.15839
FOLH1	35.18023	47.44621	40.84292	45.47244	68.14394	42.01401	43.22701	40.07431	37.71497
MAP4K4	91.13329	67.75828	51.2805	90.19251	46.03044	51.7687	49.75349	53.24585	116.4495
SLC25A4	60.18899	1550.664	154.7068	65.70763	266.0012	306.5506	556.6057	465.5093	41.21378
RBM16	778.6992	220.4218	603.9686	607.8311	279.9789	307.9033	285.0141	495.2515	107.7278
CTDSP1	2276.996	2644.227	1265.694	969.3271	454.3875	42.26113	1079.37	743.9449	3268.314
TSPO	17900.54	17453.54	20238.99	18974.39	20675.67	21568.33	16514.87	19328.66	19850.74
DNASE1L1	426.0964	331.5652	229.0175	361.7149	410.9726	668.1885	299.5063	464.3047	651.0177
FOXO3	8752.332	12532.04	14465.5	12075.68	16324.16	12113.53	13451.61	15887.1	9778.084
LRGUK	140.6911	94.72103	49.55381	106.5499	57.61544	77.72077	50.45037	54.40219	73.05696
TMSB15A	63.54967	42.12838	38.26991	41.6525	89.12408	43.27017	40.2924	50.11771	57.43284
C1orf31	150.0711	95.73945	68.8495	99.84487	64.78819	80.9546	63.30505	64.37946	142.2122
LOC34884(	189.1382	156.4852	97.74204	77.81345	106.0807	92.07284	87.22448	119.2992	87.26284
GGH	1103.342	894.7101	701.4651	951.5892	537.0571	659.4427	544.835	904.7991	1639.691
FAM80B	45.91804	33.2198	44.03439	35.68082	218.9211	47.89555	37.87376	35.09524	56.90104
ZNF320	143.6834	150.9934	102.5602	66.58459	76.218	84.92181	66.46497	59.28659	66.51629
PRM2	37.21789	43.70424	35.60423	45.43275	42.31017	34.549	46.69546	48.95043	59.83275
SHROOM2	199.0069	142.1	70.21394	198.3847	498.9879	152.7205	261.7221	111.5744	2964.492
MKX	38.33524	38.99117	104.6924	39.44098	113.7904	160.6309	51.27747	34.06286	42.19999
GRK6	39.35699	52.89413	36.02774	38.02817	39.20374	36.88847	39.98889	334.7458	49.44756
ZIC3	81.29803	74.26778	44.89339	75.36956	76.49754	85.94803	53.54252	52.49623	42.06107
HIF1AN	62.81921	61.55862	441.3721	57.47456	193.1048	437.4911	356.2143	450.4162	55.94989
CRB2	211.3177	91.22383	67.03626	70.16517	69.29851	56.37894	56.95541	43.51902	124.473
ZNF606	86.37153	356.4829	670.5106	49.5626	696.0021	620.8229	920.3929	745.6407	79.32124

POLR3A	49.55236	922.848	708.2159	1889.262	668.3901	527.0859	787.1565	1023.163	66.18042
PAPD4	81.84709	75.90323	108.4592	137.9289	84.13052	120.2722	103.9033	127.3212	49.54814
ZNF540	1822.382	2255.83	2371.669	1208.239	2658.172	1910.793	1604.118	2470.482	39.61435
TBC1D25	327.0954	435.5598	704.4797	205.9414	748.2328	364.8585	562.0546	240.8428	328.277
MYL7	377.2138	183.3613	99.69749	243.8098	110.0607	159.9544	87.8998	131.7434	527.2925
CETP	584.3204	321.4025	188.3751	329.4254	84.46313	229.6647	127.4856	177.011	642.583
ADAMTS5	45.89743	52.83105	63.30493	56.09733	49.89891	61.63534	50.7288	60.24187	130.1446
PRKAR2A	244.0542	124.4908	67.64506	432.1231	213.9572	100.8352	202.4845	185.3056	53.67992
DYNLT3	2573.446	2612.217	1687.855	1623.349	2147.798	2107.518	1331.276	1351.415	1944.193
OAZ3	184.5275	132.5265	70.34756	111.5033	55.31098	102.0363	74.23952	62.66755	150.0912
KCNA6	180.4515	98.05649	68.66882	172.0128	74.43636	98.49532	74.00268	84.76337	269.3192
C1orf92	52.06742	69.30034	123.5483	65.8157	46.66233	67.30489	129.9728	50.2126	64.62992
HDGF2	170.8674	805.5781	336.713	580.1346	175.9022	280.6006	183.9791	289.3989	1036.68
ALDH8A1	35.94992	39.84787	35.57961	45.63655	43.64228	52.02591	76.27226	39.28242	47.67848
ITGAD	35.30211	186.9445	75.73872	49.24822	45.61423	60.22454	42.1678	76.24187	79.33929
PDGFRA	46.50924	515.6124	139.2383	41.87031	153.4015	40.79114	94.32886	186.0048	99.86525
TMEM69	39.67029	37.17695	192.7776	638.3578	48.54269	37.31596	55.37893	35.72179	43.69059
OR52N2	367.6547	192.7542	102.4203	228.1428	96.95792	166.2639	92.52359	127.2475	322.6968
JMJD2B	67.6519	41.16793	34.85681	39.83385	33.20003	35.36801	35.23415	39.3024	37.58711
RSPO3	106.4277	37.67213	43.3606	57.06594	324.4088	539.6281	289.4349	44.01298	80.33948
AP2B1	46.14682	38.9713	57.6128	43.0046	34.0615	42.84409	42.66917	63.83233	99.99902
GAL	35.93892	42.77345	34.80788	33.97166	36.74879	36.24205	34.07758	35.95686	40.64668
HEY2	14052.41	10295.49	7792.601	7955.641	8203.374	9601.686	10713.15	9530.36	12563.59
FBXL8	39.85317	47.50916	37.73122	40.448	42.85561	68.02803	33.29301	114.0843	193.778
SNORA25	583.6196	390.6943	230.8294	870.4677	133.2422	194.8399	203.835	168.7484	782.8187
SRI	135.9792	123.073	107.9866	213.3756	129.8654	156.2597	96.49579	113.2501	220.5374
RYR1	67.88225	805.9028	274.6071	47.82015	56.44652	472.5048	122.5778	361.4737	123.2182
RNF19B	947.8057	51.12437	271.8702	357.8047	161.3116	186.3854	159.0009	275.9871	59.02579
RCOR3	93.32583	502.3745	1671.152	469.9971	2419.344	888.1359	2071.773	1447.794	125.4258
PAX1	195.7988	79.80655	57.25063	94.66758	53.65934	72.36529	48.86099	61.86926	96.18576
PSMA4	672.0026	633.08	449.4491	612.8632	278.3078	511.0227	360.186	454.6168	705.2269
TNFAIP8L3	1920.136	2563.472	2553.092	1208.396	2490.049	2179.185	1962.979	2308.072	1490.212
IGSF10	71.34	55.60551	69.206	75.79977	125.5184	56.29063	43.6697	52.93172	58.47252
C14orf119	367.7873	213.2572	114.8048	228.9638	103.2785	160.0469	89.04417	133.0358	484.6421
HOXA10	48.46125	36.47706	34.66797	34.98497	44.4561	35.14543	37.83871	40.14812	41.64145
THEM4	381.5372	75.58904	51.46274	73.47379	1321.065	71.32987	885.6827	383.4148	48.23617
TTC9B	57.3607	42.56771	40.46628	63.08473	42.41961	43.01775	51.29894	36.30099	64.07779
RAI14	48.31149	40.70242	664.3843	773.5666	761.8651	938.3867	1722.067	1095.237	984.1718
HOOK2	1461	2719.045	1566.049	465.3976	2270.56	578.4976	1415.534	1741.48	2882.963
NT5C2	10916.14	9976.657	7712.264	9136.16	5301.589	7946.291	5329.389	7460.729	9955.781
CLEC3B	4859.799	2025.46	435.765	2428.464	398.0965	55.97828	256.7954	676.1276	4766.773
VCPIP1	321.6221	234.5747	113.6744	250.3203	108.3098	134.3858	122.7326	133.2312	550.6542
NFXL1	814.075	733.6179	1686.71	1134.117	1673.25	2167.208	1376.25	879.9499	47.28946
CSTF1	67.64206	78.29539	65.25895	80.27036	47.64119	65.62695	51.96909	41.53519	247.0135
GCC1	1609.433	1660.183	1293.295	1456.471	890.5862	1462.247	965.7951	837.9003	1311.279
FNDC8	101.4722	50.86234	59.12338	69.13737	56.25285	66.44468	40.48862	39.60211	68.91029
ADAM19	247.8053	87.16669	41.33844	58.14878	45.91359	65.47137	39.66237	70.07197	76.19209

CLDN15	2936.944	4086.427	5062.61	2873.903	4565.604	4314.456	3639.2	3243.662	4265.331
ZNF404	42.86194	39.22961	44.33526	40.4712	40.13757	62.1688	49.06619	38.0167	59.04117
TMEM200I	79.83595	326.343	107.2654	76.67214	33.16136	76.3543	44.22496	58.85435	77.43405
MIER1	154.9149	201.8989	943.9134	241.4036	1149.904	1200.827	1103.645	1226.815	211.3304
ESCO1	397.4206	38.64264	44.18468	37.382	59.66203	78.39626	54.23747	32.39331	38.16018
IFNB1	56.86522	45.89179	35.85234	42.42904	34.21146	43.8408	36.49799	36.51759	36.39386
C14orf183	70.92898	59.76645	40.50017	49.90422	35.09918	38.70974	38.00061	271.3434	60.61527
DCAF7	124.9608	1342.798	3517.104	1738.605	3323.448	2974.854	3155.936	4418.157	116.3458
FGFR2	812.2169	50.1824	35.83481	41.20487	35.30719	46.04968	46.85608	50.62856	36.40885
ERMN	103.9462	511.2294	797.3354	132.1746	251.5119	349.938	268.8198	198.5344	131.4445
OPCML	39.63386	33.62291	43.7893	49.32152	37.18626	34.11545	36.10033	37.74957	38.05921
CYBB	101.3267	48.70896	51.10398	45.67673	40.333	69.89635	53.68269	43.5883	256.8426
TRIM33	1586.111	634.318	623.7974	1106.547	780.7734	53.74942	553.8032	239.3098	98.59836
STATH	41.36691	33.10175	40.16438	32.5089	48.53279	43.63633	34.42559	39.59998	198.2177
DDX51	113.2601	67.40915	70.3998	389.3572	85.83121	262.7755	50.29641	116.3306	116.7271
PCP4	33.9257	41.94254	44.99508	34.54896	40.0516	50.3362	39.12112	38.14409	40.03614
OR13F1	37.3211	41.11621	34.30091	39.19357	45.32721	42.13843	32.40136	41.98569	49.14986
MASTL	38.28136	46.96276	41.01429	40.50303	50.08995	46.44702	40.33091	39.58685	37.97798
C19orf57	271.5212	181.3093	86.52041	167.2643	65.48029	95.93761	67.34406	72.66319	292.2573
SNORD33	10689.63	12262.3	6269.489	9067.588	3969.752	5646.877	3801.718	4700.343	11781.7
LRWD1	148.7082	507.7733	913.0039	1375.099	775.324	531.4856	801.2395	596.8807	1197.6
ATPBD3	2424.013	2134.112	1259.524	1990.466	816.6877	1478.238	932.821	1225.109	2448.458
CLOCK	103.1232	151.7645	66.24851	74.06438	108.196	153.3899	67.6934	98.11558	402.1593
SNORD46	5294.358	3223.344	1800.57	3034.63	1583.295	1671.747	1335.511	2466.451	4173.505
GABPAP	102.8517	55.94375	74.83253	60.30384	147.8335	55.23967	105.6102	258.3144	65.65549
SETMAR	74.38734	108.9605	261.1382	58.00439	51.39932	41.09834	339.3061	524.0932	145.1032
C1orf59	131.4444	143.104	298.3557	108.3173	337.69	101.5943	963.5363	69.53346	325.8533
C6orf35	578.9367	67.66901	47.28915	308.2217	57.20382	46.03254	68.6242	94.8805	120.3188
ZNF518B	159.5611	115.3376	82.85839	136.4398	68.84447	133.9908	49.79672	81.2318	244.5608
TUBB4	93.88991	53.68662	235.6406	635.2715	271.371	442.134	285.7825	395.3622	139.8547
LOC28317	128.2903	157.9181	59.99537	103.5306	181.2621	77.10408	62.13683	247.8779	144.8164
GPR133	72.61452	57.32529	50.08198	417.7133	55.99453	56.03024	50.1497	62.48159	70.94155
PRDM15	74.15821	1552.223	164.4067	595.129	298.807	364.6097	183.9336	582.0334	100.8165
RSRC1	47.71917	362.6337	111.157	287.6862	128.3029	177.4683	109.2184	53.76626	47.17841
NPFF	123.1889	123.0087	150.4061	107.8736	162.5173	71.58027	124.9851	142.8867	172.9429
CLUL1	402.8468	213.4846	108.0879	232.59	111.1274	164.7642	102.5229	139.6281	586.4514
ANKRD2	46.85232	40.73953	129.0806	38.12923	35.22442	44.09525	533.1191	94.35405	46.97946
CCDC106	9076.019	7925.818	4482.901	5791.799	3895.372	6958.025	6176.469	3239.789	8852.368
KIAA0528	3876.161	3667.323	2665.693	2745.624	2096.463	3884.286	2489.033	1858.234	3270.561
FAM69A	241.8967	170.9737	88.20367	106.8015	139.5992	139.9468	78.41185	150.1043	481.2825
IL19	149.4861	126.7823	82.00121	139.0702	68.1655	87.39874	62.04574	444.9024	162.7014
GPRC5A	113.8507	882.3776	835.3218	2119.212	885.2845	306.9152	1281.768	393.5142	71.55934
IQUB	69.71839	64.88626	56.85406	73.92428	43.26902	71.69746	45.4068	51.53416	56.4592
RNF146	73.4307	99.17695	57.37944	66.85245	65.94884	57.04224	53.05963	69.57727	71.21178
CXCL2	921.241	84.4178	546.8383	361.7289	307.092	158.6396	252.5343	176.3159	1124.244
EPM2AIP1	606.465	53.83421	460.2518	457.1335	493.5032	701.7888	432.3625	421.0093	45.49216
BMP3	307.0754	197.4368	78.23667	212.6466	109.9669	155.9964	92.39525	101.1409	435.1368

LOC203547	916.6488	790.3918	824.5385	1531.172	978.9603	1150.632	854.6674	818.0789	1025.876
PBX2	4472.303	4467.542	3166.776	3836.813	2510.638	3500.611	2426.802	3185.55	3703.708
IER3	4655.367	2618.322	2095.769	5518.106	2432.966	1462.864	3610.486	978.8923	7334.655
FANCF	265.986	142.3691	379.3576	544.353	153.386	104.5181	138.0133	151.0504	322.4535
JAZF1	78.66801	697.6072	228.1419	62.10581	565.3367	63.19524	346.3782	370.3544	100.3626
CHRDL2	70.75803	60.57615	1355.772	45.32065	1562.535	55.28466	2010.54	1738.163	71.0698
SMAD3	756.0232	297.2047	739.9745	831.7496	1320.58	1358.477	1144.378	1645.065	2044.503
FANCA	193.1122	115.5214	53.98832	117.2101	51.90183	63.90784	54.84309	80.69486	250.1155
C17orf87	137.8759	74.48814	115.7715	64.44958	94.09816	189.037	119.5758	135.9565	50.26538
IDH3G	59.33643	31.85581	43.49417	44.06135	112.6658	55.01462	69.38935	36.59874	49.53079
ALB	54.13791	42.88211	41.22898	41.91405	35.62424	65.54967	43.94341	41.77507	51.54357
C13orf27	127.5937	74.4554	134.0835	37.35616	101.1379	224.5766	69.96549	62.8621	38.02745
EDN1	897.9163	2783.575	3253.882	3227.671	4587.4	3066.458	4616.916	1946.344	1241.017
TEAD2	1630.583	1945.638	3062.221	4358.765	3234.922	3683.759	4084.801	4174.869	78.8021
TUG1	2007.834	2065.706	1352.071	1579.09	1093.102	1312.085	1137.83	2069.459	2327.05
C3orf27	59.97693	59.2179	48.06696	69.88264	37.97192	55.93206	42.96802	52.08278	46.1527
BYSL	37.29075	865.3974	571.5057	43.76336	1390.937	492.9043	1329.956	474.4658	37.8658
USP29	38.00256	42.06567	61.69533	50.45006	53.80857	44.36026	52.67352	49.77678	54.58216
STAMBPL1	38.64495	865.8762	1642.689	48.65723	1635.03	943.17	1589.741	1227.719	48.29006
C21orf33	39.21251	871.2479	617.0282	899.2354	381.6875	1369.946	551.8277	1192.635	3558.698
PRKCD	949.7925	529.7847	48.21026	74.67337	65.19852	57.26361	46.72555	275.8065	1525.63
TTLL3	48.88786	60.93781	86.22334	76.22281	109.8514	55.33839	63.93382	99.71449	148.2127
NEB	546.5812	681.9919	159.6749	70.03807	153.5349	217.8671	209.4881	340.8921	1050.334
NDUFAF2	1009.096	664.4922	1352.321	1222.947	1512.061	969.1856	1066.206	1464.349	36.5021
C20orf151	584.7646	246.7086	219.6878	78.50478	268.8365	186.7122	106.0168	157.6245	836.1902
SPSB3	8800.312	9059.01	9606.361	8472.344	10085.57	9657.572	8667.063	9426.806	7912.595
C21orf66	1447.561	84.2513	352.6021	86.27419	398.0521	196.1208	506.1186	216.6304	220.64
SLC35B3	633.9683	409.5648	348.5758	375.7732	307.2357	429.2441	435.1061	262.8313	34.02751
PAFAH2	555.259	331.1571	521.0811	1955.753	195.9249	136.4601	267.9322	400.0987	659.4239
CPVL	2032.54	1853.023	1040.718	770.1778	316.1708	897.0634	454.2947	380.7047	1342.617
SIPA1	18769.04	19194.97	16349.62	16000.27	10627.09	17561.42	17542.36	12315.65	17860.05
LYPLAL1	116.0082	199.185	201.0569	74.7981	66.8989	422.0511	133.6857	159.9577	77.61938
RSPO1	1145.4	1312.305	298.7717	563.9014	514.4313	49.82917	184.556	131.528	51.61516
ARF3	552.9509	271.0021	124.4434	284.1455	164.2119	72.84922	98.40598	182.3168	143.8938
C18orf25	38.98322	612.2419	58.33168	56.43465	55.35001	59.85628	47.76143	39.15767	37.39053
GTF2A1L	52.11597	34.39222	40.54349	49.35216	33.73405	38.12986	41.05381	36.63928	38.63491
PACSIN3	1407.678	1682.028	1623.387	3269.659	3703.685	1496.468	3226.162	3425.086	2393.201
DTNBP1	65.12357	39.84426	56.12421	69.18327	52.7474	405.3165	138.5095	266.687	113.0289
PDZRN4	63.38772	62.86892	49.45951	43.29199	33.13063	39.85102	36.94429	43.55608	35.8597
RNF34	176.4019	109.7889	86.07042	134.184	96.1021	105.178	74.26929	61.63326	245.8174
ADAMTS17	55.95334	67.73839	46.24691	57.85968	64.79204	49.35636	44.47956	94.68108	82.19436
NKG7	1400.069	1171.121	2841.534	60.86856	796.5505	217.7271	1810.473	2052.129	67.98405
VWA5A	783.4365	59.56904	473.6673	43.89721	202.5293	47.03967	157.6235	49.57588	43.61996
PLA2R1	52.98486	36.96287	35.77499	47.54499	161.6873	43.81264	96.85805	46.55962	43.51494
PDGFC	143.7655	98.38702	122.7423	120.6589	65.32288	151.881	77.40884	65.83119	97.15057
PSMB10	14793.61	22036.17	20624.09	15685.68	17338.72	19841.1	17577.97	21785.94	17491.64
C21orf87	143.5933	130.4036	84.95872	133.7339	74.54089	76.77137	55.13704	56.57811	113.2645

PPP1R2	286.2928	1633.328	474.735	640.631	518.3611	411.5226	458.0133	475.8766	177.6309
OR6Y1	101.2296	64.14675	56.87255	67.16611	53.40824	49.86041	60.31841	62.71504	115.2692
SNRP70	35.3905	753.8069	321.1255	487.2857	316.8864	459.526	233.6953	142.4792	40.25714
PTRH2	157.9459	111.6176	64.08705	131.478	59.95503	108.7819	57.04547	56.71404	229.1328
SIRT5	96.16359	88.39191	1457.639	137.6432	1744.392	1437.607	2555.204	990.1641	2533.345
PDE9A	324.9113	182.2176	130.064	210.4264	95.70844	177.8093	94.54182	145.6505	398.361
C2orf64	195.6047	827.2634	2132.147	807.1884	3852.444	2676.198	3118.282	2845.549	57.43084
ELSPBP1	46.56396	88.16493	36.09624	38.50541	36.37442	40.22037	44.96826	36.06236	36.89988
RPUSD1	44.9026	56.67077	2642.139	1101.882	2907.698	2397.544	2283.189	4186.165	121.7706
GYPB	52.52095	53.55077	53.75003	39.70662	48.30538	60.50295	39.0868	35.6791	67.0208
ASB1	6209.887	4925.649	3653.577	5322.924	2718.302	3983.636	2736.602	4428.564	5661.647
SLC26A9	1034.55	937.5367	290.4532	111.7496	795.9624	68.18515	247.7249	189.4367	150.2161
FANCC	1260.917	740.7441	583.158	398.8477	598.4475	1073.451	883.2048	748.1995	104.7742
UNG	109.9352	109.9535	308.0691	398.0208	947.6619	1023.937	644.975	558.8474	173.0373
CYP2C18	73.75655	37.58147	252.9429	46.8179	975.5421	38.13099	108.1202	37.60744	34.9767
ZNF281	649.1747	934.0021	1698.021	985.6902	1430.51	1039.798	1874.13	1114.511	113.0101
CCNL1	2684.757	4931.97	3689.158	3254.911	1298.938	1630.79	1178.213	2276.289	4765.4
KLF15	213.0925	112.3544	80.97132	133.4035	89.02583	161.9171	107.0017	100.5723	405.9405
FTH1	917.9197	929.3883	1302.929	1298.503	588.5599	1196.321	1427.6	708.1352	864.5887
PRICKLE2	838.6906	197.4054	815.095	1140.652	762.1108	127.1944	847.9628	412.8815	301.3057
LOC388882	210.373	115.2751	71.78253	163.4638	67.68448	87.29641	58.22091	45.4693	269.803
FAM20C	877.0886	65.51557	37.5722	40.20626	48.85094	270.9431	216.5333	137.946	89.14494
C9orf23	369.9746	1042.873	886.3094	229.6016	372.423	153.3655	753.319	159.1856	600.0526
JAK2	110.2407	487.3084	683.5209	546.8	57.35519	783.4964	113.1864	236.8143	232.1643
RRAD	568.3873	108.1318	52.89403	339.9039	109.1071	56.82122	68.82139	88.70815	38.66748
DCN	5751.302	3215.456	1301.271	851.3337	1563.403	1781.97	858.7085	908.1934	5383
GMEB2	4672.023	5998.533	7247.091	4821.962	10298.03	8245.733	7412.108	9441.38	4301.29
NCAN	404.7119	192.327	126.3732	262.4957	96.97717	169.6343	106.6912	145.423	531.2995
SLC25A32	2372.822	3359.987	2411.724	4930.276	2452.35	3182.994	2863.414	2028.115	1644.846
HSFY1	42.03183	59.47801	58.72673	51.68451	53.78095	55.0631	47.17915	45.92628	205.3324
ZBED3	276.0488	110.5286	75.62614	132.4015	79.45613	113.3435	80.57115	102.4409	280.0171
FAM83F	2352.341	2461.467	513.8821	2391.569	545.0204	1177.595	741.3223	458.1106	2790.84
C9orf128	75.20313	104.5779	251.9334	58.38966	35.58236	57.49136	50.952	73.33711	66.84884
DNAJC1	720.5517	305.2675	345.1867	495.5141	155.806	354.788	197.0875	282.0924	1838.02
RASSF1	36.4662	593.2162	289.3744	457.4811	144.8402	620.6859	33.42959	31.7183	35.65857
VPS45	138.907	90.54249	780.937	1221.991	663.7938	227.2395	444.211	405.447	159.6384
TSPAN8	55.1521	48.90018	46.47038	56.23506	85.86882	55.80968	49.56079	48.68604	202.7614
SNAPC5	3813.585	3229.236	3783.558	2413.364	1228.963	1526.123	1266.407	2834.158	3237.526
PEX6	192.3918	556.0219	1534.705	271.4608	1737.474	625.4351	263.1809	1706.286	454.2014
MEN1	128.6324	236.7336	112.0943	281.7211	96.55647	128.0104	88.93455	95.02702	93.88799
BAX	93.95538	143.9516	146.4255	455.152	131.3024	97.74271	107.3603	87.26125	1101.21
SMC4	298.0465	138.1658	68.77679	74.80433	61.61462	78.04038	62.35403	75.55517	530.2177
COMMD2	54.77516	334.49	109.8889	553.5989	281.5522	223.062	238.3859	156.2069	37.07587
ST3GAL1	472.003	299.5695	165.0413	339.8205	293.8305	311.0124	251.0132	256.3677	2429.82
PRR18	207.0585	88.59326	72.72425	119.9667	73.3416	107.3001	56.71807	80.20451	90.71355
IP6K2	85.60288	466.4262	337.0009	705.1351	258.2107	638.3289	418.8012	148.6742	1172.768
HFE	46.99219	49.43511	40.57669	36.34924	43.40709	36.65715	42.02414	39.14515	114.8583

OR5V1	196.5795	103.6679	68.00547	127.5501	74.23318	79.74168	58.26042	72.71236	334.3733
LRSAM1	81.00037	583.5701	343.5274	36.09968	187.4073	403.0859	97.45499	192.7687	131.6949
C16orf71	59.84681	80.79557	74.8803	90.26443	62.55722	97.80927	56.8699	55.45501	89.34878
MDH1B	231.8156	116.4521	72.88472	144.6878	166.1622	75.02969	272.4031	88.57153	234.3067
LHX1	217.8817	72.88825	140.2313	1705.92	155.0095	97.63287	870.5875	222.5861	314.0536
NCAPD3	1548.596	667.6705	1173.207	2055.168	950.0569	702.8105	571.6586	855.8592	173.6987
CDCA5	51.1695	50.30659	2224.287	1138.738	2816.644	1037.526	4773.933	4813.403	1931.911
CCL7	86.52724	31.80973	39.98143	39.68959	43.67783	32.78707	35.21021	33.36256	50.58771
GSS	53.60875	401.7359	146.6904	459.8294	535.442	296.6238	221.8795	439.1402	86.04927
TMEM139	39.83114	41.15974	34.25702	44.39471	43.32928	335.0115	85.20949	38.95654	52.44381
TOMM40	2059.943	1840.52	1457.295	2743.586	1125.835	2287.134	1559.003	2069.307	2080.26
CD34	102.142	1475.884	331.4439	1182.105	530.8926	92.88911	393.9572	436.0836	2494.862
ZNF154	77.84813	40.49614	210.6113	441.2755	116.1928	59.62859	86.65304	119.0436	58.13391
GPR172B	46.37355	55.05147	752.113	43.572	675.8441	549.0756	779.6743	1210.048	44.11464
TMEM154	697.2966	479.3416	500.2081	581.3341	506.9944	503.5493	255.1758	508.605	858.8691
CCDC66	36.05543	45.6934	35.97089	37.65902	91.05028	34.49654	61.85989	35.73923	40.56881
CSDE1	1415.762	622.58	555.162	846.4701	824.3528	690.27	943.6177	988.2235	56.98356
ZDHHC4	711.7462	100.7441	146.2124	116.0688	63.07383	231.1837	85.02707	67.05409	112.0227
SNRPG	13162.02	13667.49	16486.6	16513.47	16394.16	17055.48	14821.33	15678.81	11549.65
TMLHE	112.4222	74.76162	57.23265	70.64669	63.57537	71.45355	100.7047	69.45395	1095.637
PPP2R2D	188.8018	66.56326	52.31617	95.93448	104.6957	94.78642	55.06725	53.14194	142.5426
MBD1	36.85521	38.05729	39.4118	57.30656	39.69606	46.47302	172.7389	55.49213	35.54014
ACSL4	36.66154	51.6019	127.5845	275.1484	158.0266	174.5636	120.2197	130.2404	67.00753
CYP1A2	58.74756	82.33809	190.5821	60.29227	56.53609	1321.479	368.9255	395.1866	5162.695
UBE1	1043.815	509.9108	570.7698	445.5523	1344.258	598.2063	592.5239	798.9149	79.58939
ZPBP	110.8645	49.11573	45.04043	43.52732	38.6815	35.45031	45.35995	56.68457	43.19399
C11orf70	75.49966	74.46159	62.2589	707.9299	98.88052	91.22965	301.6583	68.43745	36.33202
FKBP1A	1585.46	297.222	1536.351	820.8348	1519.35	1612.013	1712.704	866.5001	365.3471
EIF3H	1899.534	1733.309	2508.231	1644.053	3294.541	1570.832	3240.238	2564.679	1455.76
ING3	885.1978	518.0757	315.6469	300.5369	129.5299	48.804	40.77833	187.7931	803.1437
SLC10A2	41.41586	45.71254	75.91503	39.03746	69.72746	60.68892	69.68	78.86494	46.58959
SPATS2L	1026.547	1699.686	1268.158	2293.676	802.507	1669.424	1783.081	805.8691	1234.349
DAGLA	54.01677	47.49853	267.2114	1111.503	443.5204	593.0908	573.3601	925.7883	53.44165
LRCH1	40.1871	309.933	34.28501	29.07896	33.23532	34.03372	35.69107	40.11319	35.34075
SLC6A4	39.98904	32.78279	33.95427	45.04172	114.968	47.18483	50.42498	47.10965	36.15114
TNFSF12	70.26369	58.58493	38.85612	58.34989	279.4208	218.4931	48.25293	180.1591	39.17183
THSD1P	52.67714	35.6035	247.7917	512.6339	396.0247	177.0029	385.8109	407.3471	479.769
SNX32	258.3157	210.3595	98.5659	214.395	80.11076	120.5062	132.2387	109.8291	357.0941
TOP1MT	48.36986	45.12725	34.89661	66.00122	283.0116	49.3371	40.37463	195.7767	1175.029
LHX6	138.2053	115.4047	81.05254	87.12451	135.2071	71.81334	53.35065	77.12453	77.56732
SOX13	51.52925	664.3473	828.7649	691.8275	1560.251	1135.39	860.3862	923.705	67.10035
CLDN9	123.491	105.7181	58.80636	77.32572	65.68578	82.95702	60.32047	56.5282	103.666
CXorf48	38.61417	50.71001	43.4598	39.58369	40.25907	35.13315	36.17365	37.23774	42.7794
INVS	45.24295	57.9871	793.3196	1636.529	198.9988	67.2886	543.1342	257.7455	320.0354
IL2RA	10996.45	7495.341	7844.525	5886.894	1567.784	6673.326	5134.383	8063.933	11055.11
ADAMTS15	113.201	88.35892	70.6685	90.30522	62.75502	92.79019	50.27507	58.16594	212.8663
LAP3	1473.721	1217.571	797.6461	1056.403	280.5358	718.8221	763.3618	971.3701	896.8564

MRC1L1	52.01427	36.6132	128.4997	68.26703	53.29218	43.24692	52.46895	73.57881	49.13699
MGC40168	88.47588	51.31007	42.09026	55.94439	51.26354	54.61029	46.03286	57.02658	40.94131
TNPO3	249.1842	138.4519	846.6905	119.0436	1240.559	786.9156	885.7253	643.259	1678.765
UBE2Q2	2375.339	1738.136	3117.454	3805.74	2620.389	4731.344	4469.356	2698.905	1435.28
HSD17B13	40.38896	36.23919	35.27209	47.89259	33.63194	34.53864	37.9618	39.6848	32.57601
ZNF484	698.5813	279.8405	384.5815	307.3377	188.6804	370.8162	204.8647	260.9131	357.1676
SLC7A1	7088.729	9364.881	8241.512	11124.03	10459.91	11319.14	8871.075	9739.809	6455.733
CENTD1	33.08085	32.42142	32.04752	34.34136	34.95159	35.47404	34.8793	34.68609	33.22799
ACY1	44.96161	43.04128	40.65593	928.6265	35.83274	35.10954	35.94214	37.95993	112.056
DEFB32	49.05363	64.05298	50.54425	60.40704	55.49296	54.92269	52.20454	56.11376	244.7281
SRP72	7245.721	6129.846	8131.81	7398.519	10480.8	8037.641	10790.81	7861.412	2567.329
TFAP2C	1284.76	621.7241	1606.794	56.70002	1841.186	1564.893	1275.368	1092.243	60.81544
AMN	95.73767	50.57798	40.08377	48.56228	46.5099	64.08061	48.48098	40.68204	219.9433
C19orf21	972.2169	746.6642	601.9544	3055.148	425.8097	936.2582	1521.102	1161.761	1448.762
TRPC7	176.848	142.917	58.53882	98.22613	66.83157	78.62212	56.88439	60.14681	55.14503
PEX13	59.53569	65.89485	108.3484	48.69477	149.8298	181.2375	184.176	108.7228	35.33438
MT1H	112.5672	397.0696	212.8987	413.6045	263.528	339.8478	277.5999	204.7803	54.53261
ITGAE	265.9074	325.4108	1119.244	488.5447	898.0031	426.9254	1187.363	1081.868	53.33239
ZNF425	38.49521	41.22503	158.0253	41.20961	104.0169	364.8194	136.9261	233.8506	37.66231
SERPINA9	239.9522	144.9095	108.2997	174.1826	151.3527	166.1831	83.49939	116.8767	285.8924
VIPR2	208.1634	65.077	53.40041	120.0479	176.7665	70.00572	50.35079	213.6215	87.06258
PCDHB2	56.1028	43.47284	51.78436	64.6926	55.98666	73.99524	47.61236	37.60436	58.10588
SNX11	3282.479	3668.407	5208.235	3512.019	4356.104	4653.238	4022.852	4892.349	3915.869
MED13L	452.5861	180.7538	106.9336	240.0151	94.5757	150.8002	80.02807	83.82711	322.1549
C1orf89	52.31787	638.8927	40.77912	49.38744	383.3004	41.11127	505.2261	1448.773	101.4255
OXSM	232.5892	133.4635	235.1382	113.4014	171.3095	134.6521	164.1092	101.5489	119.2021
CREG2	145.7609	90.81115	67.33571	133.9949	68.89896	55.13981	112.8093	52.24572	221.2185
CXorf1	77.95222	49.89071	58.46675	107.0379	182.9083	66.95773	52.4565	47.42597	46.89969
GYG2	124.7261	139.2339	69.00256	80.41558	61.50165	55.11367	68.72202	81.60336	113.2233
STAT5B	36.25236	959.257	355.9809	1207.143	575.0344	59.21115	568.244	465.097	45.52541
CLIP1	329.9448	164.267	102.6354	217.1312	99.1609	149.6701	82.84564	62.41544	490.5166
C6orf190	51.38512	70.56282	151.816	42.26753	49.96708	258.2712	119.0125	43.47216	41.17698
BET1	59.32135	64.02399	276.7994	102.4405	107.9409	53.02733	222.366	55.88318	80.60541
CDCA4	2472.844	1133.3	2646.996	4151.498	2797.14	5638.775	5099.885	3514.151	1567.12
WASF2	3538.79	557.6101	1004.242	3171.687	385.542	581.8942	395.5028	642.4121	4765.969
NUP98	148.4737	346.6371	135.9177	163.0152	135.725	193.5562	85.16103	64.8277	86.74035
NETO1	107.4514	56.3587	49.60369	75.09187	53.20438	41.04657	46.44781	51.03544	50.61339
RNASE10	287.3414	144.6782	67.77925	146.9244	83.94398	109.064	69.99498	75.82541	251.3547
BDKRB2	106.5975	268.6169	295.4573	101.0696	525.5867	266.3741	302.8402	540.5324	118.5795
NR1I3	128.0955	43.55513	54.9721	39.31244	40.05976	53.83978	39.80495	47.87194	39.40663
IRX4	69.53999	64.51607	129.0882	455.429	102.8814	234.6644	208.5739	145.1451	88.51835
NPTXR	98.17527	63.97211	39.28018	44.65364	52.96237	52.91824	36.7989	53.13263	86.52167
FLJ23584	429.8023	214.8767	111.9441	232.6675	333.8511	166.3399	487.3484	488.7475	599.6885
GPT2	15158.24	15106.02	16332.97	11261.92	20157.94	16136.79	14122.04	16574.01	14979.46
GSTZ1	459.9221	428.6754	329.9712	335.3644	354.0027	314.6313	283.3052	538.9026	692.596
RAD9A	58.6161	76.06155	205.7691	45.95411	161.9699	46.80692	211.6857	155.2185	1722.779
DYRK2	279.7623	52.6669	73.39755	42.26931	86.06922	105.249	52.97024	74.01081	96.47884

C1orf141	56.76986	68.63663	42.18702	57.16832	45.96498	43.07261	41.6768	41.57186	156.933
C3orf20	36.96587	40.16665	41.98385	38.0921	43.31396	46.93232	46.97038	40.93581	40.94751
WIPI2	62.74758	57.03412	56.60365	48.53748	48.78001	60.45275	41.47209	51.30877	71.76026
GPKOW	1025.984	1960.234	1143.315	1166.542	1087.449	1291.919	964.3765	1956.331	1229.597
SSTR4	36.05425	59.10954	54.01827	49.43042	42.17088	37.61487	44.07645	45.84587	41.44926
CIB1	33645.48	32307.49	35516.06	33194.8	33207.48	33076.25	33391.74	31955.29	32427.51
NPAL3	152.8167	91.59961	177.7743	168.8737	259.0235	190.1979	416.048	192.8521	112.2309
SEC14L2	68.47114	37.45109	36.88095	45.72398	50.80939	67.10609	143.3808	45.07656	118.7463
AURKB	34.80289	40.33571	201.8925	45.12486	63.53519	32.58874	365.0127	37.68503	34.62411
GPR146	456.2005	324.4784	175.073	330.3661	171.9519	296.8047	259.4698	196.3013	721.9061
C1orf125	40.71453	65.53252	231.7847	47.4866	64.93021	40.70908	73.71902	209.2148	94.29264
MYL4	627.3782	591.3019	422.9821	79.44257	183.3017	217.9084	308.0116	397.6036	674.4449
EFNA3	156.6096	74.32178	54.46365	136.7454	220.1852	69.98129	54.03847	59.77958	162.0623
ACBD6	103.9244	45.84905	60.63258	63.74992	1165.629	821.8073	1140.871	758.2388	94.33289
SNORD38A	1003.88	50.56128	250.9193	66.7997	271.2793	77.36135	131.4461	368.2506	47.90712
PNPO	2918.916	804.1846	2617.472	1948.529	2068.836	2286.078	2398.604	3220.659	1799.603
ANP32B	11367.77	13159.44	17026.52	14690.15	18697.34	17118.37	17088.52	18407.1	12895.92
AK7	287.6921	105.6328	74.13409	146.34	79.97197	134.2894	69.35233	70.70441	271.9712
S100A4	1082.777	958.743	266.0741	705.6711	733.1486	53.35957	434.0007	311.5652	32.89919
NHLRC4	54.77452	37.73914	35.96745	51.90047	50.35563	39.43172	32.27556	53.94405	55.76947
SEZ6L	39.87981	38.945	40.31457	38.29006	35.99167	37.36023	32.91246	50.03826	41.64917
CYB5B	785.9498	668.1374	1059.801	1518.517	806.8021	1092.066	1098.496	1105.323	1039.093
VEPH1	70.67678	41.64792	43.536	52.1715	43.59136	44.30363	49.27356	40.33152	55.91475
LRIT1	159.7061	68.68613	46.04984	79.06968	51.14435	54.40847	43.20157	58.19977	47.67721
TNFAIP8L1	894.7155	473.8862	1466.156	1287.401	1460.589	454.5028	1343.151	2516.113	172.1794
IL2RB	692.686	320.0028	127.9928	186.656	151.0269	130.449	270.1111	242.3961	439.881
TRIM45	127.093	51.38607	41.46358	43.48234	145.5391	61.94765	151.0492	501.7286	133.5838
C14orf139	98.06306	440.8386	383.8703	677.8884	1291.573	554.3602	1194.317	787.5728	218.801
C5orf62	251.2327	555.7875	185.9962	548.4575	429.5134	355.3178	605.431	329.3391	185.3682
NAIP	222.6341	74.62576	77.7658	167.5931	80.79918	104.6556	63.18193	66.50633	262.4717
COPB2	7251.028	1103.789	1292.955	3213.834	2488.976	1568.011	2233.386	2199.083	721.1625
PLOD1	11372.22	11613.91	8677.957	13794.74	7458.576	10622.16	9919.988	6982.591	12184.09
FMO1	48.1925	57.2807	329.2975	60.10201	234.853	262.781	158.3153	405.3336	70.38689
STXBP5	165.6695	97.91088	496.343	557.675	1464.485	769.751	785.024	961.9744	153.8652
SLC17A5	82.21745	93.74984	50.4145	117.481	54.32336	85.73242	60.13031	74.21656	418.3238
MLF1	551.2859	318.3564	58.09413	317.423	370.8857	187.0265	213.182	240.9301	117.7796
PXN	83.26613	100.3373	70.36396	122.7261	76.03846	76.15577	53.05989	48.2807	82.35894
FCRL5	175.8327	93.49055	42.90598	104.6561	58.14841	70.16828	59.50748	63.32717	119.5341
SPAG7	150.4916	2356.732	2247.634	1129.153	5980.473	3946.452	5363.5	4664.32	3097.465
MDGA2	42.66071	64.26291	40.87381	42.53759	54.13059	36.42437	37.73342	57.53362	38.7776
UBASH3A	93.76598	42.58626	53.06769	60.95804	40.12214	49.70932	37.57024	50.16756	56.14746
CADPS2	3390.78	3650.83	2068.938	1531.17	1910.473	2924.008	1795.299	1659.975	3534.072
CILP2	61.72811	48.82864	38.06458	51.08857	45.02309	55.19994	34.89361	35.09814	53.40643
LEKR1	342.253	193.8746	110.3811	221.2713	102.419	146.1538	71.44501	105.9768	483.639
C10orf33	52.60813	54.04858	221.7219	53.2066	384.9824	211.8893	614.6883	168.1423	42.7126
TSTD2	145.4941	67.75574	101.7034	87.63104	61.53051	76.35192	271.409	295.2209	106.9118
KRTAP15-1	49.36064	34.47935	54.50625	58.49515	39.5205	46.64839	40.7899	34.79771	45.70274

SNAG1	60.36796	67.12978	56.34074	42.481	172.5222	60.55392	151.3817	249.218	42.59541
EPB41L1	1629.782	409.0097	930.0718	1894.619	545.5115	884.6865	478.2718	443.0826	4006.662
IFNA6	57.86809	54.70283	46.05114	47.528	35.01674	47.84681	40.72066	43.69293	59.69961
RHOH	149.5954	199.867	108.4467	69.46448	72.38397	140.3486	60.33343	59.95226	82.72656
COL16A1	3042.566	2817.82	2425.959	4218.735	1824.016	4021.5	4585.657	3832.292	2197.248
C1QTNF9	48.00644	37.31993	33.48292	34.60927	56.9599	39.03478	42.01821	86.78187	43.46446
SPIN4	5840.237	6402.584	6587.29	9614.243	6664.48	8321.288	7695.823	6794.125	4975.718
IL29	66.31169	82.97591	54.8682	46.1722	38.9906	53.14081	40.26287	36.09755	63.7985
RSPH1	88.80946	506.7882	50.17429	80.07589	44.92307	307.5155	49.0766	64.99155	65.69768
CLN8	38.58207	35.66	31.84916	38.84179	156.6765	35.41053	90.75894	31.79107	37.90419
ACSL1	80.25162	64.45144	119.0614	259.8509	162.0183	178.745	100.0845	123.6818	85.91571
ZNF434	1555.317	2501.206	1389.732	794.1697	607.4965	334.3274	627.358	376.1291	3104.568
TNXB	51.24188	57.44247	36.88164	75.9893	37.396	48.68303	35.7759	47.9489	48.90887
SPAG4	61.38853	38.02476	38.1964	63.41115	50.06643	51.21257	46.06804	39.95997	68.24191
ZFP90	2398.693	2981.306	2470.768	3554.002	2183.329	3271.26	2650.549	1521.363	2339.045
C20orf26	45.4721	69.66801	41.24127	36.64412	35.57932	52.36993	37.66214	48.41034	50.69838
ILKAP	743.6042	586.5839	558.9642	559.4903	405.8476	941.8324	520.4586	253.1041	723.3965
GPR115	31.80312	34.58298	39.79216	44.2006	38.3928	45.11207	32.12373	41.2306	47.07712
ZDHHC13	130.1292	89.62885	245.5529	161.4751	636.1137	68.97829	399.0957	78.00973	138.0086
HDAC11	1636.65	1117.409	554.2439	1978.901	483.803	1348.92	710.918	498.5958	1705.224
ANK1	92.56083	86.83208	81.53561	66.4723	101.8988	63.60633	118.2639	81.56962	100.6912
ELF5	39.47665	41.03035	34.92795	40.37122	38.43155	35.68853	39.27228	37.57282	36.4829
LOC400891	100.1901	55.26515	387.4376	50.23891	35.3513	35.82552	165.6053	211.3539	87.69377
CR2	49.11631	73.71706	39.70578	40.31992	33.74583	33.78474	42.68117	42.42167	42.76061
C2orf27A	61.05009	37.90958	40.76547	38.54242	39.22018	38.61092	68.25277	159.8015	58.24479
IPO11	7875.928	9357.623	5705.756	8940.039	9326.234	9548.632	9284.697	9774.022	7677.922
CNTLN	311.7322	206.2108	292.2098	206.9751	98.11942	134.9098	87.71674	102.0712	431.7932
KAZALD1	63.60358	62.13285	49.62663	48.26403	252.4994	54.22344	276.2234	239.0742	130.5197
ETFB	178.4578	150.3076	74.66857	88.55071	80.59959	120.4387	48.17976	88.96853	362.6336
USF2	57.5107	39.08794	184.8913	49.86286	98.34071	50.48217	53.03939	113.1732	136.2496
PGRMC1	39.12784	1055.019	642.1919	2026.262	1151.852	981.0869	760.6852	767.072	1301.899
CCDC101	3450.662	4296.584	3610.745	4096.543	3591.611	3362.361	2851.267	3799.643	5684.807
PRND	892.9645	1865.966	1561.517	2049.788	438.6749	1853.794	2078.092	2567.256	8548.476
TAF5	35.32504	30.84685	147.3917	76.02438	175.1779	132.3501	241.2415	259.8128	139.8736
AKAP12	150.9304	203.0888	95.56693	118.1368	100.6332	85.82983	69.56784	75.36871	295.0047
PRKAR2B	753.5817	554.1585	279.4746	284.4092	218.7087	379.439	183.253	235.775	819.9891
C19orf41	31.01542	43.88923	40.00968	45.34836	49.97608	41.24974	41.09745	40.31145	41.10646
GPR22	89.61576	73.27477	40.67713	104.1471	44.83837	69.60658	56.39916	55.90465	108.3082
C21orf49	42.94653	42.35048	221.1396	36.96276	39.50714	51.18209	40.56278	243.6721	2101.42
VCX	47.4953	41.25238	32.78115	60.45827	45.46778	40.79293	33.04923	44.88006	38.98368
SAMSN1	440.7299	212.2689	247.4703	232.957	133.3113	284.7797	77.62582	203.4377	550.9105
OR6K2	47.89329	38.50307	36.72319	34.53819	49.29436	43.78498	40.05489	33.79398	189.6057
ADAMTSL1	40.87748	33.69024	41.03777	39.18582	40.20092	36.37672	37.7125	41.50835	40.60709
ANP32E	324.2249	184.145	88.35623	179.5285	85.8651	138.6761	85.06409	88.34236	303.3593
TOPBP1	1192.188	1833.985	2616.196	2150.457	2199.078	3151.027	2341.861	2526.069	1209.966
GPR119	108.9729	51.89427	49.92995	114.0615	42.07954	44.59618	48.57161	53.05112	105.2217
TRAT1	136.835	108.027	69.5315	145.6212	59.00126	96.19727	56.03897	188.4056	325.5985

KCNJ3	64.11458	31.11855	43.9575	51.65437	47.46206	42.60112	120.1793	43.37412	141.5578
ASTN1	45.94694	50.32888	43.85492	44.57911	41.96759	37.1057	45.44519	69.80375	59.06496
ZSWIM3	223.5406	64.73621	47.64215	72.74122	56.69611	57.16468	54.3672	531.2513	77.7709
ZNF212	77.35038	3320.177	4010.85	3706.93	4168.803	2754.672	2765.498	4516.676	118.0222
PDK4	41.96375	35.75351	46.66076	40.47528	54.1959	51.91098	47.30383	72.33035	768.0073
OTUB2	147.8526	206.4243	59.81572	98.09985	78.09698	65.12048	54.59711	62.28151	181.0815
RCE1	124.3419	3690.18	5608.45	1701.399	4412.275	4354.43	6682.391	4899.75	179.972
TNFRSF1A	48.00517	40.5752	43.5371	37.83759	250.044	41.49643	310.6852	197.5202	59.20993
ITGAM	177.2956	464.8692	124.1356	244.1672	267.9825	180.6467	110.6744	229.2629	445.6393
EAF1	37.5605	43.63431	246.6063	35.08707	571.4925	337.0444	310.3923	258.9234	32.77277
CAPN7	589.0575	306.1986	136.4768	157.2194	66.5906	218.551	89.98257	102.2565	355.1125
ASL	40.71912	40.23963	49.6723	646.1354	133.7099	46.59673	87.35847	439.5724	47.43487
TENC1	143.7544	44.90256	56.68414	71.50997	93.11574	57.31761	62.54684	113.5584	109.0864
CYP3A7	41.98089	53.23777	38.48489	37.4431	47.76315	57.16043	46.1502	39.15969	56.76205
CHI3L2	37.17102	57.67046	42.43047	37.96686	36.15754	41.0051	44.49343	38.42352	82.01943
DNAH1	10002.38	12966.39	9822.334	5292.194	6737.396	7335.14	7881.314	6792.35	11374.83
FLRT1	62.54993	34.70333	43.20137	46.42579	40.88573	46.43832	35.31435	53.31355	53.10679
SLC22A4	44.42236	48.83637	35.04033	81.26782	52.9636	45.74788	94.56698	39.06756	65.48273
SNX7	154.6005	73.18281	77.52793	97.2239	58.39222	61.93535	94.44643	70.18782	389.0087
TELO2	504.683	255.8019	148.2166	279.2762	132.1364	162.607	103.5669	115.9586	498.2177
RPL36	40.83316	712.8275	1763.91	2887.615	1731.914	1182.262	1468.273	1908.354	1019.051
CNO	411.887	373.2241	1261.319	337.3482	1389.428	1790.65	1601.917	1516.905	994.884
MCHR1	56.20081	87.36145	73.40183	102.3803	64.81783	118.3076	69.30442	62.36179	209.5826
ASCC1	1221.256	1427.124	2933.612	1972.655	3436.552	3243.763	4095.898	3770.453	1903.248
C9orf6	116.7721	75.76841	66.10507	74.95193	44.07104	49.29207	108.1266	46.51423	77.65916
POLR3D	86.86275	92.09582	125.4496	107.2628	149.8358	90.98758	63.58659	62.15401	220.468
TACO1	4514.046	5811.74	5267.033	5050.565	4030.886	5101.556	4621.661	7949.952	3578.954
HEXIM1	106.421	76.78975	68.58677	106.2916	64.47021	66.34704	68.70794	38.8713	67.79275
COL9A2	35.05544	44.12642	976.2041	49.58054	169.1888	737.089	1459.091	31.77738	32.55832
TCTN1	171.6902	140.6256	94.62646	187.0631	218.4521	97.92965	238.1685	150.1925	231.7609
PRRC1	205.7931	100.1002	334.8846	102.8287	532.2444	62.61997	271.5386	68.78757	321.1341
ADCY6	1720.747	393.1802	459.1848	48.01288	1384.654	1273.786	655.0455	771.1821	2291.012
ANXA11	8195.373	9822.367	8251.302	6445.924	6325.813	8004.375	6847.467	4887.898	7688.153
CPB1	303.416	162.2979	69.87526	152.3759	89.7398	115.5306	74.68039	96.81076	286.2958
SGMS2	61.35887	418.8344	748.6088	649.1747	829.4642	349.6943	555.9935	542.3902	632.9131
CLDN10	80.2347	59.04794	64.19031	60.38339	58.57002	50.75635	35.33922	54.032	42.7856
CACHD1	67.09375	88.20763	116.6442	96.71127	49.92974	57.68626	57.64134	65.29447	49.0551
KRBA2	69.78831	90.78349	53.91053	1245.085	247.0061	67.21968	272.1984	64.06073	57.83475
TMEM14B	792.438	875.181	1618.917	1438.366	1980.779	1136.851	2705.94	1614.272	61.99876
DNM1L	46.06715	91.07196	364.4878	1313.829	306.931	731.1948	422.5433	226.1694	126.2268
MCAM	1149.07	746.3853	768.1107	497.4833	2100.95	494.294	1515.522	737.6354	668.1821
LOC37574{	60.2551	57.32925	43.50672	59.6097	42.74549	47.40409	41.11596	37.63684	46.39046
HTR1F	45.86547	42.60031	33.0796	46.94558	38.25985	36.70728	156.6518	43.20602	38.58038
PPP1R12C	642.4127	1319.114	328.9997	460.4846	447.1697	214.5239	295.6872	370.6271	1719.772
C16orf58	24147.25	25363.77	24030.94	24881.23	23814.23	23540.85	22766.52	20905.21	27680.91
PLEC1	47.41406	60.87117	67.84113	58.41775	44.2624	512.5634	53.6615	57.21661	114.703
EN2	449.3167	225.1904	128.3473	266.5951	100.4241	195.3325	103.1152	137.9336	567.7333

TPM1	630.0294	462.3102	315.265	1116.984	251.4501	377.1213	546.5024	239.8324	425.395
NUPL1	2791.715	2366.881	754.3888	2339.64	560.8396	1079.931	520.8734	515.3179	1857.735
RTN2	44.92276	40.94871	33.00497	33.29834	40.90043	35.95752	33.12434	38.00482	34.26571
ZNF773	429.5908	564.8289	155.8281	213.3416	157.1283	90.79517	190.3979	101.3055	435.3647
SLU7	1434.288	887.5233	794.3249	433.929	547.4807	372.1641	552.4559	399.1415	46.80993
SUMF2	42.76344	155.5446	119.7384	321.7795	74.37749	166.0805	94.17835	68.5593	49.16425
PIGR	46.60334	36.28187	46.77791	42.86512	35.08665	354.0902	91.02616	75.22017	42.60808
BAD	39.81948	41.20639	51.0429	62.23283	51.52209	45.44205	51.80909	40.92259	64.25056
MMP7	83.75356	58.60525	57.23049	497.3354	41.98137	341.4523	751.2087	425.6289	63.19137
ALG8	167.7968	286.098	559.0747	165.3001	464.2235	371.0704	600.9863	338.8976	169.3456
HBP1	143.5996	88.66409	202.34	362.2464	161.3378	79.76314	68.122	116.5748	83.07252
TRIM3	73.4661	47.80211	42.5917	46.35046	42.37517	37.62336	40.69766	44.25668	34.65189
DZIP1	34.50172	52.83309	243.9901	221.3232	196.333	553.715	536.9286	271.4706	37.20423
COL8A1	7821.409	4342.064	4922.361	4412.628	9712.947	11130.74	9771.503	4591.498	4864.416
ASAP3	9077.191	8138.688	8524.519	5747.771	11130.52	10881.85	9266.85	8240.832	7922.096
NOTCH2NL	1308.293	1334.998	1068.24	868.8219	635.2441	881.5071	586.5642	541.0325	1425.877
NARG1L	33.17559	43.80572	65.6688	113.3187	71.1866	153.8002	62.44644	49.72748	40.75138
ZNF397	86.66732	74.29849	86.41348	105.2858	185.4312	59.33796	123.6095	57.46179	72.82831
LRRTM4	78.67873	51.96835	41.34085	116.0183	168.8615	64.80249	50.37227	68.74588	91.00507
TARS	69.97195	280.1737	242.8677	277.4781	662.3798	177.2003	459.0804	281.6337	162.4285
FETUB	190.8149	556.391	230.1076	531.1867	1337.794	462.433	126.9579	823.5818	355.2566
TMCC3	137.1571	47.24617	101.9529	69.16013	113.4644	134.747	135.3555	112.3625	64.07484
RGS7	90.71611	74.09682	64.61433	67.85172	50.83444	61.37183	50.54628	40.37671	50.38652
KLRF1	192.3306	93.4386	141.2649	128.537	91.95778	73.47149	75.65544	80.11684	141.3293
PILRB	46.16631	41.48903	985.609	49.59371	39.44428	1400.679	419.9301	730.8765	3297.839
LARP1B	128.6733	267.6443	569.2376	372.4012	251.1526	313.6704	121.9732	417.6608	212.6169
SHRM	672.1023	657.2584	696.7285	1870.215	1022.03	1092.975	340.0607	770.5562	71.23311
ZXDB	410.4265	204.1642	91.1865	280.9711	112.1419	73.86839	153.5068	90.94775	237.6104
TGDS	271.9409	248.8914	120.0806	181.0831	155.5314	207.7856	120.6808	158.5856	330.688
TSC22D2	256.7308	567.9557	1045.834	2425.552	777.8583	371.2175	533.7734	317.2476	151.2769
CBLB	1017.974	530.6082	1322.894	509.6639	1505.155	944.2933	1251.713	1311.434	226.0958
PNLIP	61.78617	69.78145	44.73389	54.87967	51.0294	62.00403	45.88916	47.31728	82.23108
TMEM41A	1810.656	699.0141	453.9405	87.24789	665.2326	712.0934	269.6408	839.9733	2289.795
NPTX2	54.5965	35.49345	52.49355	43.09086	434.186	46.85361	53.17063	58.60595	165.4385
EFNA5	2115.426	2204.255	1009.974	60.42178	416.577	509.0644	47.3591	57.10656	269.8207
UCP2	39.0341	37.37121	46.26027	46.29142	393.1335	42.2146	195.2265	562.0144	57.10477
KIR2DL5B	38.03842	50.54689	44.08907	51.19126	35.80206	56.03012	40.61793	42.74771	45.65163
KIAA0892	2470.478	1074.955	1124.869	3181.589	1376.007	1203.117	683.9135	761.96	2014.214
KCNN2	39.78698	44.94925	46.72414	38.82401	38.71798	36.54007	35.66884	41.91119	44.80207
FLJ35776	64.67294	40.43736	39.83293	37.41677	33.28045	37.7609	60.20339	71.89671	36.27253
BCHE	121.2431	107.5869	133.5508	343.9934	69.40118	78.59323	49.57647	44.06654	114.3478
MYOHD1	191.5098	63.24022	61.39465	91.2041	69.73694	74.17857	54.95497	77.39665	112.9865
RRP15	93.12785	3453.07	5699.672	5329.391	6486.996	6450.574	5919.603	5550.403	4567.051
CYP24A1	48.64627	41.81182	36.80035	54.64338	48.4634	70.08549	193.9299	169.1117	41.10438
TTC9C	53.67392	38.41323	238.2876	614.965	166.8136	486.2633	112.8234	119.9125	81.21533
EEF1B2	51.0623	57.21069	56.54829	50.50429	45.67411	63.59464	55.54665	46.85727	50.24586
SPATA1	53.75406	68.5638	38.39388	60.04269	49.18302	59.56771	44.04787	40.48059	47.04256

PCNA	34.28278	47.37294	41.70118	41.24549	34.25858	36.58864	38.34739	60.43188	164.4104
JARID1D	680.26	1120.743	45.58334	1057.296	30.76155	41.7374	435.9518	326.7753	1065.6
PRSS3	59.49665	91.44642	254.7267	98.02628	471.4816	86.9053	357.1933	62.34183	126.5044
ACOX2	1066.573	666.5488	547.0501	36.8452	1395.963	1544.103	928.6812	671.7675	47.04418
TMEM125	82.65412	89.28329	871.7418	101.2387	1483.989	72.79179	364.6814	1206.065	172.9242
INHBB	349.8264	192.5242	104.9601	229.8977	419.9348	116.9097	284.7557	263.6907	410.9015
NTSR2	75.95454	57.76807	48.16838	57.92785	51.96104	47.86592	47.90633	54.02206	435.6367
EIF4A2	11453.69	12331.79	12336.63	13317.85	12193.36	12735.33	12048.02	11557.71	12298.96
NIPA2	342.7244	454.3939	279.1625	197.0333	222.0953	144.3645	246.267	430.6576	312.1113
RAB3C	42.49955	34.28736	37.27525	40.61345	37.69752	47.36293	40.3918	39.58656	59.26803
WFDC6	86.00702	45.56952	44.3044	33.48637	38.1241	38.37109	38.70912	33.14259	52.54549
WDR45L	54.07905	60.41402	298.7057	84.86148	41.34408	533.3958	212.423	514.37	46.46796
FAM3B	50.31271	59.7858	38.59434	49.09273	41.24891	33.60211	39.44625	54.17887	49.57314
LOC64734(	954.515	1113.827	881.0094	1347.048	1627.914	603.3921	1243.594	919.4082	710.7988
C14orf142	30.65015	44.28548	173.5521	342.8429	288.1774	249.7869	385.2681	218.5106	35.67276
AKR1CL1	109.197	67.63981	58.43576	66.44155	61.93241	64.94394	55.59617	68.90122	60.36407
B4GALT5	672.991	628.5306	803.7943	447.5932	881.3796	641.1488	1089.82	470.8532	770.8284
SLC7A2	35.33277	42.14133	48.48251	33.17123	41.62345	48.28919	75.52188	35.09509	34.55134
AMPH	117.3289	75.70921	348.0365	113.903	178.0702	98.06177	331.8792	423.2716	55.58031
KIR2DL5A	71.81188	67.74739	49.90472	66.10271	51.64862	51.38819	50.63981	42.77281	60.41045
POLDIP3	1521.931	1767.695	1740.676	3971.51	1011.797	400.9396	908.2463	1649.27	36.07221
C5orf53	95.38299	47.19053	455.8824	241.1293	530.8405	628.7729	416.0985	668.8512	37.44105
MLN	224.811	144.4874	89.36285	115.5181	75.31936	74.46642	81.63592	72.46982	79.79816
ZC3H7B	147.1011	107.1261	75.51894	86.32446	85.82009	125.8201	62.08735	76.55505	46.53178
MAPK4	465.922	250.4944	117.874	194.3222	137.5276	91.03174	90.7702	83.73182	113.5603
ETV1	67.51031	44.12727	53.60331	46.27351	45.53757	76.70548	42.94088	58.36784	46.42031
SRC	57.77385	46.30797	50.03536	60.05169	94.1684	38.52461	38.46584	41.83627	41.81657
TEX15	104.212	29.95001	38.80581	62.62847	44.73612	32.7866	52.00582	53.03462	35.21658
KIF2C	128.2116	307.3167	290.313	350.9123	373.4694	670.634	489.2156	515.5014	87.19545
EMCN	155.7617	59.90346	55.03519	85.42854	103.2546	57.90752	61.79728	115.5146	69.08289
WDR34	212.365	154.135	495.6673	88.71744	537.5381	74.82264	333.6449	393.1465	1240.459
VANGL1	66.18588	43.98279	54.3986	403.9398	60.15108	50.80211	58.86813	68.92948	98.21949
DNAJC24	57.7671	48.09081	42.27946	44.71299	38.60892	52.72665	44.48323	47.57845	56.72745
UPK2	36.49913	55.22268	242.7993	61.41446	40.97055	65.50745	109.1803	34.9699	51.64644
HMBS	112.7097	80.97958	56.96909	425.7369	53.25473	71.35635	67.25211	70.06746	341.6953
CDH26	58.15921	70.81064	59.26044	104.7901	46.10759	57.17998	44.58433	54.02102	72.36555
SLC25A33	269.1591	68.75782	140.7258	169.6351	123.86	174.6026	110.5435	102.7453	34.28378
ATPIF1	63.57157	235.386	108.452	265.2735	90.35215	71.83862	83.36911	99.32166	644.1292
PDGFA	1055.699	51.84446	70.10506	46.40786	58.19575	49.43215	55.60551	68.39946	47.2894
FAM164C	56.49471	45.48483	144.6406	354.6617	329.197	296.5978	270.8067	220.5342	42.57652
C14orf79	831.1597	586.7898	815.8113	655.7845	775.4499	1397.25	902.113	700.5519	750.2296
MAGEA11	49.28786	41.6091	39.36129	59.85908	50.6992	101.0932	49.20586	39.00926	39.94981
GPR162	38.11847	42.57152	39.45791	65.81794	41.28449	38.51839	39.944	36.19704	49.14589
SPATA17	57.16889	65.22798	58.26673	58.92212	52.53659	62.51538	47.76545	35.54504	119.3446
TOP2A	38.60122	1777.627	1385.034	1006.086	1669.751	2188.004	3349.942	2494.682	4532.816
CCL23	4355.859	2092.42	1767.018	91.56246	2858.708	2460.028	2543.855	1885.112	2418.508
THBS1	589.3798	281.5817	163.4385	311.1781	75.54858	47.21853	275.2542	119.3559	41.19852

CCDC45	673.3505	3443.976	4919.859	5352.667	5876.889	5283.546	5547.055	3987.84	1438.418
NSD1	530.5759	731.1326	422.6291	428.3744	259.7854	462.7128	212.3436	348.4849	1284.111
CRSP2	168.4828	83.28313	76.92116	130.7837	72.29881	77.16093	73.84412	87.58222	173.2507
GGA2	13478.5	13114.26	13217.04	12858.33	11884	12024.25	11371.58	13614.96	15397.51
CUL4A	395.935	115.1216	85.83749	62.22159	88.59591	112.9844	68.37553	93.00154	295.3055
SDCCAG10	97.00912	266.3397	136.6663	343.2375	171.3878	195.924	269.1242	228.9084	176.8515
GNA14	90.05033	50.47511	56.91033	48.68451	42.58727	49.21904	67.55372	60.14188	62.5029
GNPNAT1	844.1873	1073.719	998.3713	1520.615	1098.201	985.5023	835.8515	1139.078	2421.456
CBX2	177.6358	90.64954	50.91225	83.95933	49.58607	67.1865	49.7333	1036.191	59.55118
OR6A2	108.0583	71.47221	42.61549	51.16612	56.6375	50.43364	37.42029	43.73379	72.80556
OR6Q1	53.66116	46.28387	33.11616	42.16914	32.87649	47.70565	35.20178	38.15321	40.11345
OR1J4	97.71851	73.50317	52.21765	110.1961	67.97319	67.86702	57.76846	58.79712	196.3306
FAM127B	37.53409	681.7463	310.6657	71.17904	1226.035	532.4174	1078.28	1145.736	48.66215
PRODH2	343.2376	59.63849	66.14161	52.40949	50.33299	100.3773	39.11824	54.51072	546.24
DIO1	99.23395	44.5987	46.28072	36.07171	38.40197	37.66826	39.68748	49.15498	44.14846
PLXNB3	36.86797	42.84547	51.28015	70.77486	126.0156	65.99371	112.9405	34.31978	61.16531
USP37	91.13853	68.49479	238.7916	72.2284	451.6848	294.5565	401.9542	129.0515	66.94646
PGBD2	110.7231	82.27	51.80519	42.77476	65.5353	76.1153	55.3899	39.00056	69.99194
CRYBA4	75.47847	61.33548	47.82515	63.37368	1273.89	306.3155	264.0775	303.452	52.05549
HSPA8	8912.956	8288.791	6564.515	7940.387	5647.712	4456.242	4403.608	6417.95	7985.074
C15orf26	59.9856	44.5242	53.46343	45.45921	170.8398	66.81175	69.51697	42.54262	44.6917
SYPL1	39.32304	42.68002	43.64019	33.46976	34.61473	35.77464	33.80752	47.54438	32.69612
PYCRL	47.82229	45.06437	39.84095	60.54833	49.73616	33.67379	92.15373	54.43491	34.17942
NBAS	93.35961	79.2095	167.0088	107.033	331.4941	178.5642	150.8698	307.3736	707.4895
KDR	96.45026	82.51995	108.4608	141.6175	59.50568	150.1712	86.08912	68.1329	107.2171
ZC3H14	46.25973	116.1375	717.1469	1814.279	396.3797	1017.072	285.1359	938.7205	69.37299
ACYP1	167.5373	58.72998	457.3398	47.20185	342.0242	589.2529	191.4334	387.9944	38.02068
TXNDC6	319.2948	159.6057	88.95619	140.4729	68.04176	120.2247	59.64145	67.64884	334.1458
HILS1	66.97579	47.97557	51.62109	56.87457	56.52048	73.23153	49.90057	51.65988	86.35255
TACC2	64.52022	42.56253	63.59407	57.5026	42.49311	53.04469	40.58298	55.8658	242.693
TIFA	635.9058	55.16956	209.8861	49.09774	176.1118	60.11967	145.381	65.23426	97.47654
CEP152	470.8	271.695	411.0486	49.03261	166.7504	263.4643	126.1687	131.1286	41.89669
THAP2	109.6421	116.0747	53.80673	83.00774	43.30745	50.27685	47.85032	62.59624	170.8488
SLCO4C1	681.6886	346.5925	178.7392	83.52326	60.11563	542.3292	124.6115	55.54686	109.104
FBXO24	54.6265	55.25693	51.99525	53.7595	48.94875	83.97522	41.4151	42.70272	78.41125
HIST1H2AG	89.174	100.2291	65.93802	64.48249	132.1697	61.27635	58.00311	467.8029	172.6756
DDX59	126.4615	124.3012	70.87663	48.77591	61.1883	103.0671	66.60213	56.85319	107.5378
CUGBP1	87.36127	65.30473	281.4931	592.87	208.6187	94.96579	384.1205	407.494	65.65327
AK3L1	37.82105	35.28558	36.3176	33.60033	195.0259	50.73986	34.00134	40.06689	43.86207
OR2V2	65.59457	61.68274	41.88008	89.59472	50.07133	65.38002	45.06317	43.75223	126.4346
SLC37A3	55.7247	71.08743	68.10468	70.17867	49.62605	65.85435	42.7188	48.87197	41.24667
INTS2	41.33746	34.78661	38.22709	37.19829	410.4027	332.1446	431.9157	433.6277	35.96673
FYN	1926.013	1360.446	2337.682	1933.141	1692.435	1766.363	1644.192	793.6549	218.7381
TIMM13	112.7638	128.0465	63.71237	75.47546	82.9364	115.7633	58.01181	106.5284	97.44579
KIF13B	37.84909	387.4874	149.4584	49.16223	354.5742	248.7507	213.4956	48.17527	34.97604
DEFB1	105.3163	146.1724	434.9317	103.0147	2665.891	668.4274	1344.718	194.0463	225.2121
GTPBP3	240.2542	71.29346	56.68186	78.03365	56.69403	61.26513	44.86357	59.69527	56.82548

APBA3	152.0891	1140.216	2481.018	75.29974	1944.893	1408.315	2264.3	2470.333	164.5852
CSF1R	16479.61	15443.35	16865.22	11862.91	12310.63	14252.77	13706	14308.98	14355.97
BAT1	6806.469	7954.784	10224.13	7434.348	10767.82	7549.378	10822.46	10162.24	3373.874
CXorf55	51.83925	49.9317	59.00137	70.17139	55.47564	66.74026	50.22866	45.73786	51.56679
UTP14C	1015.642	2889.292	2472.465	2225.069	2947.301	3009.247	2220.544	2211.831	2538.018
OR1I1	70.77203	73.86848	42.35831	45.22456	56.08699	88.6156	49.30429	67.72499	84.59152
MT2A	3356.467	3238.835	1952.309	4698.876	524.1464	3171.704	3257.111	1573.974	6098.812
GFM2	90.055	61.511	51.1371	65.564	47.88403	50.51245	45.52621	59.83577	95.4668
ZNF462	53.81272	57.95657	1826.152	73.78103	1549.392	1239.05	1402.088	1071.962	129.6332
FBXO38	86.04704	40.99312	296.6015	59.23017	336.5613	298.9952	330.7169	238.4326	68.62758
DICER1	311.5909	141.197	85.52486	190.3638	98.79398	144.9825	77.24542	120.4711	197.55
TMEM9	49.98717	39.15138	40.52491	36.1042	360.3297	574.6166	125.3031	400.8563	75.75662
MGEA5	47.29763	272.6547	344.2501	40.80951	268.0126	181.406	196.2394	246.5419	45.41097
RPE65	34.80039	39.55632	36.9725	39.67107	34.30893	41.25735	35.88157	40.70935	42.74044
FLJ44653	91.30463	61.15196	42.10645	65.4687	49.52298	173.0108	52.34031	53.64553	64.29897
KCNK4	55.99427	58.7332	65.15895	66.83558	49.90542	61.49154	41.02964	57.65609	47.17815
CCRK	144.9734	137.257	244.1141	62.10709	325.0182	55.97062	341.948	423.3049	165.2178
NR6A1	69.44454	60.76719	44.87017	264.0055	60.4206	56.8679	229.1176	380.547	136.7697
HLX	503.4361	246.2062	71.92109	101.3219	65.00282	68.85335	59.38724	68.16428	137.7764
CLCN1	46.3802	57.01891	42.50391	51.15385	52.71682	43.81194	54.99004	64.48354	93.16123
GJA8	117.3637	147.6864	70.42061	97.5533	52.29389	63.18509	64.11492	86.01051	128.3781
PARP1	1370.764	840.3539	462.7852	750.0383	428.7583	1113.665	931.2953	751.1859	1153.232
IL1RAP	101.0542	238.4039	393.443	439.9022	470.8463	452.8727	357.467	312.1493	53.81849
C6orf225	50.57786	235.5503	84.42519	41.77486	108.1293	48.3676	92.1258	86.86302	49.34604
TP53INP2	94.22366	113.9549	884.441	124.2353	1646.502	666.882	841.2381	1009.846	2805.026
CCDC58	562.0761	1219.633	1079.534	959.4258	1714.438	1707.61	1216.214	1294.839	56.65877
GPATCH3	49.83496	56.36414	38.19362	46.75492	88.92019	50.38795	88.33875	141.8216	39.94033
CASC4	6546.475	4930.604	2947.197	5809.599	1687.248	1679.306	1445.626	2698.238	6066.19
TM6SF2	49.57513	59.25214	124.4206	49.9695	98.07342	281.1335	250.1975	133.658	48.11397
RPS10	34126.27	34475.65	36976.13	36060.07	34881.59	38272.89	33784.71	36774.95	36817.89
SS18	33.7906	434.0381	32.36516	335.3804	71.32935	139.8716	37.30724	36.53852	39.88504
FGFBP3	50.11927	67.79436	37.30602	56.74991	226.4784	200.3466	233.2223	116.8887	948.2083
ZNF362	78.72647	53.78083	1467.803	1107.481	1262.227	2115.094	1463.376	872.3822	52.82502
MTM1	1149.359	34.91362	32.77771	34.27843	29.86913	32.01777	90.41759	33.78264	37.06456
ORC5L	49.83073	36.16178	48.94135	41.11844	35.08949	43.00538	37.26706	34.34554	36.53936
ST5	354.8126	899.0923	622.165	2416.077	248.0102	120.6921	447.3867	576.3296	486.7295
INSIG1	33.63258	36.4803	38.5873	38.17064	188.9699	45.06878	32.75726	52.10739	43.77126
TEDDM1	142.7204	190.0572	77.51595	169.4767	77.65966	101.548	71.4459	76.21049	392.3334
OSR2	212.4295	116.1472	73.39436	151.449	69.39429	127.2892	152.901	85.20083	95.64304
GAS7	201.8123	147.2174	73.82899	144.0741	80.437	89.69727	72.81807	77.7913	463.127
GJC1	315.2907	293.5563	149.4321	453.0174	124.0094	102.7757	129.6011	106.2867	769.6096
ADAMTSL4	89.95915	961.3895	717.945	781.7448	678.3995	97.03403	985.6155	1063.553	111.4731
MRPL47	145.0666	34.72498	48.10299	133.1177	46.08713	34.2456	49.071	51.56723	35.48252
IFT140	128.7623	57.51442	48.72636	44.9098	117.9191	62.2059	36.14796	48.01585	55.47647
OR10A2	53.82809	85.60288	42.49695	42.24182	54.19753	40.04841	36.31847	50.53815	69.54897
PAX5	106.4599	51.08695	65.58984	119.1894	48.20138	63.08994	60.45929	295.6877	56.1417
C20orf117	1135.255	536.1257	356.1671	1331.379	468.6929	67.52422	109.7691	47.8902	1687.412

C3orf64	715.6104	465.9165	429.4895	1162.014	468.8533	595.2683	542.9302	421.2301	748.0457
RFWD3	1019.88	2018.083	2378.55	1626.318	5100.196	2576.454	4177.528	2660.306	122.4392
STARD7	61.76844	42.28067	31.39758	36.02079	37.0307	35.05029	36.6306	40.48448	68.22955
BDP1	1645.332	1370.663	79.01872	177.7576	269.4455	88.9633	128.3466	271.1494	221.0584
CLTA	71.35759	41.76009	30.62563	42.82343	33.37277	35.26434	36.66302	34.67795	40.51533
APOL6	85.93771	79.55199	99.47878	70.26321	51.05431	53.80555	51.30434	54.13384	132.103
C8orf13	1739.229	300.312	1366.263	61.85649	4904.515	1700.423	1641.579	1065.833	51.74609
PCBP3	41.38438	64.03601	48.14167	50.32372	50.18536	47.46807	42.74097	48.68525	34.98669
DMRT3	65.23841	284.4416	55.9032	58.05151	371.6325	51.79276	195.4153	53.77154	53.7927
ZNF514	4055.144	3092.971	2392.912	2707.783	696.7482	3176.504	1749.443	1528.614	4633.906
HPN	115.1471	71.73869	41.55291	93.1665	49.93765	47.42502	42.9393	53.50843	82.95561
SPATA20	11856.38	12161.29	8115.473	13009.5	4904.596	10980.24	8283.184	8835.319	11398.81
NXF5	69.29934	63.56879	74.7618	58.05286	55.40195	56.33149	53.68088	82.6071	112.9383
DHPS	197.4656	97.68446	74.37846	147.1563	285.6913	86.30911	117.7401	188.5316	325.9717
USP12	62.97768	47.57047	55.29758	62.85968	42.84991	69.46509	47.85307	36.03883	42.25751
CDNF	49.35181	335.0648	168.5856	64.07692	81.64726	343.6792	103.3997	228.6523	68.7588
VCAN	1523.984	38.69444	868.3558	53.53303	1531.406	548.1443	1180.232	388.6044	38.41394
ACAD10	1361.448	1597.208	1466.474	1299.18	988.1266	2016.971	1537.113	1007.078	1252.063
LGALS2	287.4373	120.4143	145.5681	141.8442	151.6653	130.8687	82.17452	71.59235	823.8974
RXRA	34.86972	6169.369	8987.839	3125.59	12081.74	7348.223	12834.35	14951.14	4356.068
NCDN	104.7287	115.2308	1167.747	1059.168	1403.941	1447.114	1408.141	2500.812	100.3092
SLCO1B3	1067.101	39.65536	39.62564	328.5343	50.95605	64.57603	167.6777	166.54	39.39956
KCNMB2	270.0255	156.3898	86.72009	153.9866	76.97865	96.57573	65.44093	106.5963	372.2765
SCFD1	52.66256	220.5455	59.13353	33.52938	44.04049	84.59232	52.23266	32.23376	497.5385
FLNA	939.9758	708.4292	360.855	801.5483	300.5957	448.3338	289.827	578.3636	1158.823
ANKRA2	37.16385	1290.372	2721.866	1600.709	2249.974	3517.681	1443.272	1913.68	33.98991
TMPRSS4	43.30185	32.70028	56.27192	33.78605	38.41007	63.60725	38.91576	48.92226	46.111
MRPS23	157.0784	117.6936	573.2263	115.1351	771.7169	512.5436	754.5032	1194.852	172.0809
IFI6	20112.75	26543.62	25446.5	27315.44	13920.7	26265.01	24431.31	24897.19	20813.86
MGST1	32.13966	32.35239	32.71067	41.03227	42.82643	44.10649	38.47817	30.97586	49.06272
C3orf35	1887.094	929.6494	471.7457	167.3399	595.9647	102.785	174.8752	101.3521	280.2982
NRK	433.1914	258.3203	114.7816	209.4802	101.3502	147.637	78.6958	118.8151	676.0062
MRPL40	1185.243	887.3237	939.4196	165.5274	1043.682	987.924	996.002	1145.088	269.438
HCN1	44.63416	66.17191	55.39443	94.74976	57.64014	64.76452	59.37943	38.77521	70.61806
CRYGN	128.4965	84.12456	68.20406	68.99827	81.15091	100.9946	57.22506	66.08845	123.3747
OSBPL11	41.19059	33.13494	39.86652	34.36708	164.4794	37.42059	155.0107	110.5238	51.18753
DHX8	5846.292	5410.381	3169.394	6048.763	3081.359	6090.004	3464.876	5121.26	6115.115
EIF2B4	44.00333	2406.06	3986.449	45.56955	6286.718	3086.179	4601.367	4239.303	38.42039
RNF6	542.0974	239.5581	114.0561	246.4956	68.68063	180.0096	62.59461	126.9164	143.494
HIPK2	6458.431	5770.272	4284.955	3985.36	3745.556	4166.579	3136.697	4160.486	7175.269
TNIK	47.15002	57.30128	46.90876	45.41201	38.70588	57.41074	41.88109	37.91362	60.43188
C2orf39	36.38381	43.7818	35.62512	66.09601	38.74911	34.87546	43.21293	57.87619	53.18511
ZFX	54.47728	32.71688	123.632	348.5107	135.9178	311.3413	296.5026	41.99786	915.2385
SLC37A1	279.3065	478.8641	326.4142	168.8861	169.212	116.9756	126.3302	249.4416	1627.406
MOXD1	2571.885	1743.09	2946.023	715.1429	5643.463	3732.006	4974.947	4058.849	3362.848
ALDH7A1	13800.71	11823.67	14819.42	14537.59	10407.03	13751.54	12253.71	9493.434	11820.5
HRNR	67.50639	83.22198	41.54215	56.73489	43.32038	49.37051	51.74831	38.27771	113.3348

SNAP23	4108.626	3467.49	909.0989	2771.233	635.4315	1277.356	876.283	961.6841	1526.997
SEMA4D	665.3553	628.4406	865.3861	307.7534	314.1329	772.4523	344.1888	386.4927	554.0774
ETS1	1303.676	1001.175	731.1691	619.9265	562.2606	584.0112	682.7067	914.0369	1206.071
ZNF567	49.93739	457.6357	44.72446	825.5648	115.2647	268.5997	232.299	176.4559	53.5209
HSP90AA1	305.0414	215.6138	97.51913	205.7405	70.96813	137.0911	164.7851	96.23663	279.9843
DAPL1	4822.509	4231.599	5896.846	2939.609	10537.13	9674.378	8564.621	3872.32	3178.5
CUX1	1190.415	509.4591	75.53664	91.09292	240.1156	79.00262	60.41078	73.91883	112.6087
CAV1	76.9781	52.98078	200.115	1125.062	203.5847	400.6073	355.3029	149.4886	97.02271
CPT1A	416.4704	235.7494	118.4771	246.2764	114.9648	186.9645	116.1758	124.1346	567.3532
WWC3	35.36274	846.647	673.08	41.70993	582.3447	555.4105	486.1061	418.073	46.27617
AATK	273.5269	106.6858	66.6603	116.5118	78.83941	117.1179	60.03427	103.1549	227.3285
DPCR1	50.61687	52.29871	46.22633	60.07435	151.966	216.4438	50.2103	37.50368	57.41211
ZDHHC24	68.84453	130.4345	72.58526	172.9325	71.32894	137.2985	149.2807	232.12	61.93406
ABCA2	103.5523	81.66417	59.37896	76.56571	68.56945	63.63225	61.31337	60.12633	114.3433
TGFB3	1009.325	1784.165	4187.683	678.7573	2847.795	4070.132	4918.572	4973.512	112.0366
DNAJC4	93.89784	135.9787	66.34187	101.161	54.97043	78.15045	58.53842	85.57409	147.6361
ZNF576	329.018	1004.961	463.4357	606.3013	1058.896	652.4549	791.6547	749.1221	433.3882
C14orf178	73.21658	48.52132	54.33784	78.34939	59.06496	38.11032	54.10898	45.74316	59.51931
PROC	32.41316	34.55416	1462.556	1342.167	1035.697	1363.679	1264.118	794.0884	43.80105
RAP1GDS1	488.8722	69.1235	89.16446	52.21677	76.41576	43.68889	54.71945	42.07277	65.17667
BTBD7	160.1322	101.3052	195.7017	348.4849	198.6036	203.3436	125.7941	155.8952	822.91
TFEC	70.49349	87.68183	53.471	62.00613	51.15902	59.68049	43.62778	77.63821	45.21163
BAALC	365.6201	249.9291	93.82357	267.2744	146.3557	177.5317	93.0683	112.4605	425.2922
ATP12A	62.96347	85.10714	56.61667	77.69167	266.4351	82.20171	51.64074	41.92432	156.5993
NCOA7	131.9579	118.5815	963.4426	486.0694	853.1033	688.7769	761.5458	243.6821	1442.016
RAD17	670.5568	514.6797	316.7169	756.4734	64.05937	128.0963	108.0275	169.7787	1186.124
PPA2	712.0716	284.6215	728.5826	591.5056	1153.517	767.3188	953.9727	845.1541	718.2928
CCDC38	44.70875	46.02771	39.09229	358.2484	111.6618	41.36784	70.60883	64.83942	33.99729
C16orf45	156.9947	398.5573	141.4819	122.5578	174.1772	111.1203	84.45897	82.68198	411.6903
SEPHS2	2396.118	2077.582	3872.822	1496.703	3323.481	3052.534	3417.083	5029.033	1632.225
CD226	1827.324	1378.535	1859.324	485.7444	1156.92	1876.117	851.0708	772.8104	3524.927
ZNF311	41.03606	43.74199	273.532	32.91164	184.1974	35.01759	291.8386	126.2615	42.10596
HM13	159.8864	84.0821	58.77398	112.7503	60.61868	92.23912	119.797	64.63342	136.485
C6orf224	37.96077	57.76607	117.6697	37.02478	36.99001	39.7452	33.9772	46.99751	877.6887
ABCB7	961.1027	1848.236	1390.448	1802.989	1089.211	1138.576	1274.439	1384.011	2312.76
C7	77.82108	423.4969	69.63843	57.56463	675.3193	253.7779	145.8048	282.9386	52.66652
ST6GALNA(	881.9421	75.09396	366.4207	98.38702	249.7374	374.6991	199.773	224.7541	169.783
CALM2	138.8712	945.1246	3028.447	1011.788	6065.393	3214.592	5193.612	2321.047	1414.501
POP5	54.1623	614.578	1774.395	51.91073	2077.291	1603.567	2659.526	3043.453	34.33631
TMOD3	244.699	49.39501	48.74446	31.80512	94.33304	38.84191	32.51269	45.85727	58.5465
KLC4	57.58975	62.4241	65.59855	41.53483	277.9967	63.19247	57.26696	82.6357	33.54425
UNC5C	347.0077	206.0958	98.8172	212.0202	106.8081	157.7104	71.19156	124.9063	353.0831
ZNF2	244.9008	685.7172	853.0118	184.1286	1285.323	1424.281	1058.076	1328.74	210.1143
PSMC4	57.34214	480.5859	136.1709	106.4744	89.82686	59.01584	110.8652	239.3994	722.065
BTN3A1	498.9199	288.3788	415.2742	251.0824	104.172	237.3218	158.7909	319.1057	678.9982
PIK3C3	87.25084	283.3993	275.3555	135.1199	434.407	290.9196	346.4939	250.4547	194.2126
JMJD8	28068.99	25160.42	20700.9	24959.92	24885.88	24708.17	23398.91	24338.41	29465.39

RP9	9658.791	9144.808	8735.377	11932.91	8966.625	9876.101	8956.933	7954.701	8813.173
HIST1H2BE	339.1318	112.6041	78.50652	409.1781	48.39268	144.2901	79.51162	95.46608	251.2798
MMP21	240.2539	54.37471	104.426	58.21852	68.57085	65.1835	69.45837	61.74668	181.3081
SLC25A22	2820.998	4970.307	9187.259	5321.313	5165.981	8783.798	8089.7	8158.858	4255.969
NTRK3	50.61737	83.76318	50.52633	44.70384	51.57045	54.98569	50.52813	47.47963	62.90746
SPO11	91.03172	65.34418	62.5056	65.88786	48.85667	50.30459	49.41585	62.52605	52.85399
CAPN3	1756.655	91.5658	559.5621	63.95295	202.8835	211.1785	957.7012	930.9207	183.4852
PHLDA1	47.91655	470.2137	265.4741	499.2833	654.3018	49.34615	534.7542	184.9909	140.6971
SLC6A20	305.0916	102.6335	87.15215	207.9283	84.64896	108.1625	64.45373	78.26762	283.3182
LPGAT1	95.38908	266.4218	94.76211	296.3896	111.1421	164.2524	146.8093	103.032	614.0302
UPF1	64.17348	103.6476	68.17451	119.8235	157.5728	219.332	220.0952	205.2129	218.9594
CST5	171.3727	71.05062	75.72341	96.84823	72.12611	63.46083	64.60924	57.34231	107.3453
FAM161B	35.63872	32.48885	107.3687	41.6	34.17858	41.8574	195.9379	205.0399	62.15191
COIL	3085.978	2087.477	4199.245	2620.9	4612.001	4791.178	4756.975	3929.211	151.598
TMEM53	795.0245	459.5126	466.8878	649.6644	319.7763	471.8665	363.9022	496.8019	833.1655
Septin 5	2217.243	68.38032	533.3049	1749.409	233.5343	564.8438	39.20614	35.8498	54.35153
NAT1	51.33937	39.87032	71.73312	78.79099	48.61152	52.08432	41.23463	156.4946	43.49802
OTX2	101.3435	97.30926	64.10499	151.3581	70.17928	49.7656	90.43536	54.79882	228.7785
PEBP1	1411.124	1781.023	1531.442	1182.697	2092.301	1184.931	1576.054	1881.777	1849.908
PTER	60.59079	66.03057	36.77437	1030.862	49.9695	39.24665	38.39054	577.2091	36.91576
TBC1D4	1466.826	2077.36	2271.82	1391.536	1573.048	2835.314	1333.235	827.1247	1244.231
BTNL2	53.85611	49.1184	49.4347	38.73355	46.56826	36.30935	35.1459	44.92103	44.85464
CTNNBIP1	47.83623	1430.101	412.6177	60.98922	2421.828	702.3699	856.3757	1192.47	2287.103
TRDMT1	67.73802	88.8017	57.22746	65.54291	76.51486	85.37177	53.00114	82.87247	122.6344
LOC645974	315.8184	124.0295	75.04115	116.5347	78.91995	114.6135	86.01131	83.60793	223.1782
PCDP1	217.9279	130.1643	67.19138	104.1078	67.3589	61.31963	64.91579	73.43577	355.8797
ZNF713	241.6301	150.5163	79.89085	176.4393	74.82967	111.8475	70.94787	74.18504	176.2525
IL27RA	107.4913	157.7825	715.1491	183.5849	640.1536	397.3672	429.3242	641.0586	224.1104
MST1R	34.14184	38.28946	556.7152	1628.396	334.267	492.6594	1364.512	275.8146	33.13781
CYB5R4	53.60954	354.9435	135.4641	363.5151	102.9825	45.35831	91.67195	74.28703	40.52361
WDR27	59.60865	54.46363	505.3107	324.8729	347.1154	634.5267	394.5851	138.773	67.89571
SLC12A1	65.32339	39.27309	47.23589	53.98891	34.10079	46.90345	34.56083	45.10924	37.98833
RRP7A	15659.52	16568.61	22897.38	20798.02	22856.66	23840.45	25217.51	25560.4	15169.14
CSPG5	68.92136	70.31078	64.18782	101.4204	165.3887	46.56757	54.48705	64.15127	707.1357
KLK10	2367.128	893.1376	985.278	929.8203	3176.409	826.3202	616.1547	1267.974	1632.875
SYNPO2	229.4232	146.1621	167.2211	120.0714	134.358	82.45672	90.2508	78.98568	149.9281
KIAA1409	33.27417	35.96118	33.0878	34.74939	33.17158	48.75059	40.63435	123.0294	41.28161
HNRNPA1L	34.93355	257.2073	125.2473	41.7203	80.25563	121.7566	71.4296	40.91526	60.99464
TREM1	479.3959	259.3421	141.2135	357.6142	89.29105	161.352	120.1531	112.4938	548.4512
ASS1	44.93336	58.75838	66.88156	103.9606	53.12685	57.66523	41.5722	54.01484	84.80965
PHOSPHO1	72.17702	102.9365	56.6987	67.10456	57.53738	63.16741	59.90947	132.487	59.54191
C6	92.11596	677.251	54.72397	678.9028	272.3508	62.05061	54.68177	248.6879	151.4328
C20orf144	32.20134	282.4584	37.5221	35.32349	38.80732	178.0947	53.57962	38.00697	35.6327
PVRIG	600.612	75.84315	137.4993	37.8909	54.92817	352.7085	139.0921	146.1487	50.33694
ZNF219	1158.082	402.6638	645.2871	420.525	557.778	342.1761	414.645	465.5976	1701.919
RCAN3	1710.869	1887.487	1724.86	2469.085	2037.913	2374.3	1948.18	1670.569	1492.053
ZNF160	2761.53	1598.331	1665.003	1868.672	809.9311	621.1187	1457.768	409.4111	770.3315

ADIPOR1	127.3108	1037.54	985.8328	116.5445	930.5054	641.8246	655.7187	573.0255	2626.988
MAGEB2	127.6793	36.19305	271.8552	62.19047	53.49215	44.34604	37.33194	38.38993	64.04625
ZNF800	54.1373	119.1138	79.16831	47.71605	59.04235	88.57597	63.33842	54.93229	89.17143
C3orf17	529.3255	298.6721	208.8336	311.8979	158.2765	241.0195	142.5121	310.1511	743.0867
LST1	6377.214	5319.214	6732.75	3624.51	2169.158	4809.749	6215.063	7567.975	6574.037
BPTF	266.6202	1615.177	604.0319	175.3602	238.0591	102.8262	240.4078	270.5482	235.6192
ZMYM4	2864.02	1132.84	1253.938	2374.849	844.9397	1857.008	1106.644	839.1629	1483.44
SNW1	2419.293	2174.211	1254.87	2427.955	703.139	1107.597	892.2932	1344.76	2697.112
MYL2	309.8091	162.7403	82.77225	304.5866	78.66854	115.0184	163.0103	275.3428	360.7188
C17orf57	153.5388	149.2005	67.58073	152.6918	53.05316	60.48773	86.25652	66.78862	210.2527
FCER1A	705.8695	67.41981	86.82472	229.0784	154.1292	191.5902	145.1553	156.948	189.1429
LOC15506(	879.3262	42.15726	42.62235	33.98819	201.3495	60.58019	41.3069	39.57627	42.87848
LCMT1	136.3502	1537.873	259.3047	1074.876	119.5949	61.23774	260.8305	149.5362	1536.773
KIAA0564	145.8905	806.5024	752.4236	62.10857	1452.716	1344.775	1072.202	1285.894	1045.418
CELA2B	482.2068	242.1471	177.6581	264.3752	264.9696	275.5993	167.6759	295.5736	619.5161
MAP6D1	431.2135	3362.76	4131.442	1553.418	3747.742	2968.619	2818.934	4947.103	714.9989
DGKB	129.2083	56.70041	42.88367	150.7854	51.29456	55.92896	54.12776	40.58186	96.13075
LRRC42	3967.706	5286.201	8302.516	8728.705	9224.433	8145.878	10119.15	9583.534	4910.58
CCND1	13410.97	14271.46	16410.41	18763.91	15294.58	16956.45	17379.5	19723.17	14174.57
PCDH10	58.67093	40.0504	46.81699	65.76543	48.26917	67.71719	45.4244	165.2381	34.38563
OR1L8	286.965	231.6269	118.7952	270.6755	106.2377	167.4071	99.57502	131.1745	491.5007
ZNF195	3663.769	2775.314	1766.127	2755.672	1186.331	2289.146	1066.127	1597.452	3003.904
ARL5A	19562.54	19848.94	17754.88	17186.84	15709.15	17027.17	14055.92	17363.96	20196.83
EIF2B5	3032.884	3604.666	3795.963	4806.535	3866.351	3980.36	3362.956	3614.663	142.4645
OR10J3	32.49074	47.55812	39.84636	35.62529	30.81844	48.98861	41.80694	40.26455	37.07675
APOA2	50.24394	38.90108	33.11481	36.54047	51.90728	37.38298	43.52062	42.48301	30.48726
CAMP	1752.702	1951.673	619.8588	2892.87	344.765	1914.519	911.9619	931.2786	1775.069
ATXN7	148.3873	84.7995	54.67268	89.24468	55.57298	64.18823	57.83503	85.75041	81.12434
RNFT1	128.6281	100.3989	54.36403	73.50127	198.5394	84.19001	56.05891	57.40565	58.30382
GRM5	278.3997	174.7033	87.46588	152.8812	78.93742	113.7028	74.07669	73.99522	274.0284
C9orf80	688.4098	301.4566	499.2398	656.9858	375.9084	586.7457	553.2845	477.9231	58.91693
UBQLN1	1066.635	1127.817	1472.368	1184.99	1653.494	1512.077	1575.895	2299.64	608.9307
AIM1	840.5844	541.5444	381.9706	464.391	190.261	233.6412	229.9507	197.0665	991.2891
AGBL2	94.6882	45.67466	55.48903	599.5236	140.1835	51.21821	48.22796	157.098	120.1909
ZNF274	61.39164	1779.072	759.0497	666.6104	685.2147	592.8141	302.3196	892.5248	1667.739
RECK	1891.909	817.4329	634.8062	1745.198	706.8005	537.8962	397.3194	276.0717	1925.089
APOL1	36.94544	1307.883	33.58944	49.92389	44.06146	52.15605	174.2649	146.9678	78.96887
CLCN2	85.87896	166.9359	59.01896	137.1246	82.81217	91.89655	56.25291	85.89016	275.0805
TMEM198	203.4256	127.7657	70.47378	70.33755	67.62308	113.1928	99.35774	123.8698	272.5801
FBXL2	148.182	436.1622	267.3273	181.0089	334.9992	144.1363	288.1429	106.3072	332.0968
LAMC3	11074.32	12184.44	8592.66	7973.661	6960.451	7682.041	8881.721	8375.603	12865.22
LCN10	118.1732	120.3481	64.04591	113.2352	66.95618	59.79176	61.54632	63.93327	413.8226
IFNA1	35.71192	36.45685	36.88005	41.20367	38.5406	35.96743	34.8597	47.91115	105.4322
HIST1H2BH	1007.466	901.1356	590.7979	1239.905	236.5011	1097.347	566.051	475.7652	727.7042
CDCP1	79.72829	558.3092	582.6958	83.98598	442.0232	658.3406	414.8868	607.6505	137.5628
SIGLECP3	106.7685	60.00737	64.48702	56.86281	52.85856	66.85036	57.91159	63.54498	161.3245
ZEB2	483.9613	265.6298	168.5889	289.421	159.8791	199.9967	128.4117	146.0719	594.3002

PJA2	8146.784	8829.229	7357.771	9349.774	5465.487	7673.17	6940.875	5926.667	6420.483
C17orf47	387.021	204.0906	102.5655	172.295	113.9697	139.2948	81.06635	96.37406	409.7992
FLJ44048	1151.545	1086.667	1127.545	42.70747	329.0492	1276.543	338.5691	1237.719	82.92448
IL13RA2	34.56895	36.44538	48.75665	125.7396	76.88963	68.02729	70.07494	45.57306	33.67565
C17orf95	47.73982	38.42313	47.58614	37.88086	48.44377	60.93781	39.33343	46.14694	46.17234
DNAJC18	2438.331	1198.63	1237.646	1540.961	1794.744	2686.072	1485.779	1720.656	1431.97
PTDSS1	38.40149	590.1797	513.9801	436.7435	1735.714	650.798	1598.76	1118.03	49.67122
AAK1	118.6339	49.4541	142.3562	36.59204	123.7797	260.2198	95.38817	134.44	43.48096
ZNF221	465.0228	48.76619	68.03558	36.28817	94.5982	45.2647	60.46043	77.024	586.3189
GPR39	118.0285	77.48029	38.38921	86.63032	47.89392	51.29498	58.21659	82.94671	81.37547
JPH3	57.63629	61.61742	52.66583	61.57682	50.36585	73.03801	45.91224	53.66488	54.60011
SPRY4	897.3376	78.15901	166.595	90.36642	77.00052	124.0424	65.98615	235.1015	101.7975
SERPINB6	44.18167	33.22456	37.07151	37.91659	41.5367	38.09788	34.35784	52.17316	44.83307
OR4X2	92.49468	44.05877	41.22036	45.24137	46.94426	37.68736	38.66053	35.47517	44.60719
SYT7	70.81064	58.65561	52.92995	79.09839	55.67466	418.6132	51.53144	76.52113	53.20225
OGN	58.38825	51.91896	34.99998	32.23446	32.68068	45.76004	35.95605	38.00492	62.03778
GRK4	89.05061	60.6687	40.58466	45.23685	58.20792	56.61914	39.93025	35.13009	53.13786
TMEM44	3265.897	2820.779	1385.996	3654.993	1261.92	2640.04	1000.036	1600.541	2725.158
ZBTB9	53.18047	736.0482	1125.514	52.55775	847.3781	971.0647	1613.496	1541.419	78.47785
PCDH11Y	33.1701	34.11317	31.61663	46.56864	38.70446	42.82034	37.75004	35.49356	32.4008
CABP4	59.30445	40.08258	35.29532	65.41508	103.8237	43.5041	109.3315	121.4584	49.72713
PPP1R9B	6931.909	6256.637	3995.917	5550.451	2523.796	3298.612	2203.692	4341.051	7768.531
DIRAS3	241.2838	149.0331	85.49579	163.084	78.53488	94.43647	72.74728	94.74603	301.0461
GPC5	721.5078	61.11957	58.69072	96.56599	110.4795	58.91408	81.7224	227.2109	64.05073
LAMA3	90.33832	1665.912	469.1164	5651.144	634.9509	898.0954	1015.301	542.6896	2721.551
RASL10A	65.76095	39.7934	35.97431	40.72034	37.72277	38.4447	37.00875	54.94453	67.42406
NR5A2	58.11104	55.78467	48.43109	53.23224	36.19746	61.22097	43.37125	45.70857	67.58695
KCNRG	44.87652	34.30109	45.18883	42.83522	260.3698	34.47272	111.7611	39.26888	34.5315
ARHGAP19	52.18369	45.08976	42.16707	37.28498	113.1726	50.63303	103.9321	232.5356	39.97279
CD27	565.304	1288.508	1872.371	438.2582	2203.082	858.1188	1222.283	1654.656	1035.277
NOL11	59.97379	559.1581	2633.256	54.27286	2278.449	2439.391	2848.603	3403.762	82.11937
C17orf58	69.13901	130.7242	67.00665	58.30756	68.84752	42.81617	50.11937	61.58005	650.8909
CDK4	70.79144	1597.306	2717.167	1568.077	3478.49	2675.608	3281.714	2720.792	1931.686
DTX1	121.0214	133.2141	78.37385	78.37552	147.1994	221.0303	109.449	64.21404	158.9885
TNFRSF12A	923.1073	1440.383	696.2836	2048.671	382.5753	578.9415	799.5858	722.9008	1867.468
SFRS14	52.14754	38.96037	205.526	49.59415	552.0189	37.30158	70.8961	50.84033	39.83335
SEMA6B	218.9644	66.70966	51.91513	105.8975	66.6871	141.9414	55.00551	93.87621	336.3839
WDR7	44.40441	34.54908	54.07872	40.36155	39.01811	58.97628	40.25973	39.26456	45.39286
LIPG	50.25087	201.1723	204.6616	343.6852	170.872	237.2672	100.8041	368.895	65.02898
DRD5	44.68487	36.67088	41.06004	40.94063	40.22643	36.64379	44.55944	75.73201	35.16306
FLJ20273	698.6294	462.6264	2615.915	795.5425	2637.755	1512.136	1714.771	1436.236	47.51994
PIK3R1	52.80001	40.85286	401.6616	61.26194	265.9988	263.3641	275.5414	117.7198	49.44219
ZNF329	46.06862	53.50665	407.0251	48.86407	616.9948	264.6741	669.7016	545.6489	40.83718
COQ7	45.09735	50.56046	43.81638	66.5051	170.6058	50.056	199.4076	228.8011	40.22995
CTSC	68.33304	311.5642	59.98585	273.8153	45.20401	53.38356	68.95114	103.3324	257.7983
NAT5	14462.34	15485.65	24439.23	20252.12	29075.08	24077.64	24946.79	21460.32	14298.11
ATP1A3	159.9579	90.95146	77.7552	152.337	61.82887	96.1562	66.12555	61.21852	136.8281

C1QTNF6	183.8657	104.885	67.25507	106.5491	59.86955	80.74164	68.5071	73.25437	99.23502
NOB1	113.3709	85.96249	99.88002	68.66632	486.1419	107.9234	182.6772	99.57202	36.29076
CXCL12	3634.599	1796.753	815.744	57.12076	169.6278	49.53768	736.1143	1045.079	119.828
PEMT	74.89341	85.69425	46.07315	102.0774	48.34377	55.56591	51.09977	61.9601	38.57062
FRG2B	40.40439	35.14168	39.92026	37.2113	36.37587	45.91388	33.19373	41.58734	37.20389
CCNK	4227.349	3140.347	2669.97	3715.983	1669.589	3692.059	2491.653	1641.92	3751.108
OPRM1	90.29465	127.0613	83.03368	177.5446	65.16537	68.94096	63.80785	77.19698	326.8723
UBE1C	134.8025	156.4845	170.3944	282.7744	136.8018	144.3915	121.6957	152.1037	440.8783
GABRP	48.49842	55.38371	43.32961	79.56059	49.83272	49.43673	93.67085	49.21168	107.1426
BTG4	60.37239	42.18153	51.99802	54.57033	60.53415	65.5503	51.47038	74.97501	31.42902
MMAB	162.0522	120.5052	79.54388	121.0555	53.29523	60.52347	52.39811	39.34364	166.4451
DPEP2	3393.531	3873.061	4542.278	2242.482	3333.435	3959.14	3076.464	3375.407	4717.988
ACTR8	137.0031	232.0941	137.4528	63.20933	70.65596	165.806	89.68334	73.34004	49.58958
C4orf31	108.5055	309.1993	55.38494	92.44325	244.0741	254.9002	128.5154	126.466	380.7376
PPAPDC1A	77.87583	55.61464	80.11095	60.4109	63.96324	35.34073	50.58606	49.95565	48.18475
ZBTB3	2945.072	2574.194	3005.063	2444.49	2617.596	2968.636	2987.776	3197.26	1434.615
DHDH	3771.911	3491.121	1519.35	7263.341	1030.696	1832.382	1699.468	1368.432	1266.463
RSHL3	1396.05	3380.347	824.3586	1416.385	544.9065	1448.758	531.1019	702.3937	1446.774
ACVR1C	61.09783	112.9744	62.31313	83.81161	41.87141	64.63903	73.24435	46.62011	119.0387
IQCE	2496.692	72.38511	434.069	906.0511	504.7229	795.1578	296.1871	400.4398	144.1418
SPG3A	331.9155	203.7005	714.7327	230.0701	1169.823	402.183	371.0667	1021.493	429.739
MAEA	1979.24	758.0655	284.1749	787.8199	234.8088	379.2841	100.6889	498.3235	2283.338
GUCA1B	42.95493	37.85222	34.2654	398.5035	37.23837	50.35022	63.46826	121.0376	44.53542
NIPA1	776.3828	396.9838	764.8967	308.4014	781.4427	1180.554	805.852	736.1146	199.7635
LRRN2	346.6695	160.3084	77.05859	170.2982	80.17905	144.5658	72.46199	260.6635	288.6526
LOC85390	208.521	139.6579	506.1397	880.7646	50.79678	489.2002	216.7249	307.2529	89.71928
ARMC10	331.8792	202.7646	127.5393	178.8048	85.91818	156.1111	149.3708	98.0589	310.9549
TMC7	239.0435	171.522	477.1968	186.6341	104.6101	510.3964	77.01304	413.9315	464.2848
NUAK1	480.2386	733.3225	456.5952	656.2139	999.3846	910.3831	1663.097	1831.717	67.79623
SCD	41.24037	54.41983	1979.465	2746.649	5380.518	1838.161	1826.533	1776.742	40.93375
NUBP1	1727.402	1317.46	306.3672	919.1555	828.506	1545.09	962.8102	1196.069	2137.202
APLN	97.64602	110.3713	57.05085	89.34526	57.56352	82.30536	52.53978	64.86611	113.6677
GIMAP2	58.3749	82.33646	133.2116	47.80267	80.88303	169.8883	65.60132	37.55727	49.23993
CCRN4L	3827.755	4232.071	3184.606	3884.583	2184.407	4168.104	3267.345	2846.047	2805.029
SF3B5	51.44061	99.61544	1073.333	103.1438	2430.278	483.0881	1376.314	2270.501	194.0155
DNAJB13	79.30124	49.75265	42.95887	65.02426	82.7032	86.47447	60.97073	51.04502	101.1889
APCDD1L	521.92	4567.824	2548.067	966.0288	1573.688	4718.926	2781.581	4540.017	2324.293
BTRC	37.6295	48.72157	40.05667	37.50715	33.78815	47.38694	128.6086	266.5659	39.26949
POLR2H	11857.82	12702.9	15465.78	13285.27	20064.99	15915.22	16559.01	17949.15	11392.32
EIF3G	134.5082	544.0065	1202.836	418.0251	1613.232	271.3791	1505.216	1927.143	87.87189
ZBTB20	1327.736	1185.854	669.1481	1265.023	711.1522	1209.934	720.5323	632.3585	1775.225
SP7	71.06646	118.2164	54.07158	38.9832	49.34528	52.05544	43.05527	40.93357	73.9334
ATP6V1H	371.2861	781.1008	138.8221	227.6627	84.22591	180.2177	69.23122	113.3268	663.559
CPLX3	93.20517	89.58678	285.4124	70.05642	57.80473	436.059	145.0026	62.33801	220.4469
PECAM1	894.1628	1417.791	947.2192	836.4832	1710.543	1188.46	1244.799	1016.786	54.15746
ZNF136	79.7273	51.10163	363.9511	36.90542	197.3831	40.997	168.791	35.83987	46.97304
FAM63A	915.4154	576.6407	261.7847	365.0392	136.8111	298.9955	205.9783	246.9767	1236.932

C14orf181	175.4893	187.9108	111.4376	146.0308	107.9476	178.0824	119.8061	99.75118	143.7377
TLR3	70.64279	62.93845	38.94199	70.92937	37.41574	467.7013	172.1494	42.58811	61.34284
OR5J2	49.93132	35.90776	33.17906	44.65778	35.79841	44.31008	32.78642	37.76548	46.60328
SNORA66	6793.827	4908.052	736.7888	5272.37	1458.563	1018.706	939.0498	2101.108	2772.946
RNF17	40.59063	45.91029	42.9861	38.43921	40.53833	38.68041	45.91808	36.94016	76.09351
MINA	673.7109	277.0911	127.0276	315.4662	145.3848	154.8194	100.257	188.9024	731.5124
RAX	37.09786	35.26146	54.91999	38.2459	41.89783	35.7811	37.21304	36.03838	44.15844
FSCN3	69.06964	61.79087	61.67728	46.10736	52.1349	50.62113	46.13594	38.56808	86.11026
KIAA1755	877.4427	553.0982	236.1441	187.5283	143.1096	139.0122	123.7659	168.6383	222.7611
RNMTL1	20293.7	18865.11	18847.54	18529.53	18529.69	18910.18	20246.1	19846.33	19519.95
HLA-DRA	4106.869	6375.8	4066.214	3852.114	1542.933	3832.288	3325.262	2944.085	4831.87
NAE1	941.9581	515.0839	345.8925	617.0814	245.4377	317.6775	290.7221	131.3105	1054.4
ARID5A	127.1249	320.4988	263.3853	1021.138	233.8462	50.98122	420.7378	45.51421	35.58906
STAR	35.2892	38.98197	51.62679	33.98476	35.63989	40.96423	35.84426	38.29359	46.55294
TMEM5	46.69468	567.3712	1270.285	361.3268	920.4111	896.849	1216.431	888.2498	48.2943
CHKB	1005.424	455.41	314.2222	529.2941	197.7706	260.3463	273.4501	210.328	49.07763
IL17RE	304.688	147.0379	102.0012	82.12266	136.6694	176.344	85.90256	112.6049	245.2227
PPP2R3B	1433.828	64.11608	150.3379	163.9318	66.0575	502.6716	49.08098	481.9721	36.99182
IL1RN	54.47214	934.45	255.7955	1024.767	216.6527	525.9023	261.1809	152.9818	62.03795
NOL8	640.0744	1229.729	1185.941	828.9104	1036.599	1579.697	1053.82	1254.197	1168.981
ARHGEF10	571.8464	366.9909	345.964	335.9246	517.6277	540.7493	328.5341	550.3731	715.1173
MRFAP1L1	6506.362	7179.652	9685.79	6872.047	9477.074	9496.416	10903.45	8998.734	6126.137
MRTO4	2092.362	3296.069	2927.046	3757.314	2058.715	2099.126	3746.549	3959.184	3792.447
DUSP13	50.41928	35.44342	35.75515	79.38546	53.4657	39.27308	38.48668	48.84147	50.45775
LCOR	122.3554	493.6849	82.25252	159.3684	71.42832	268.1801	145.3337	201.7489	62.66092
DMPK	3145.297	3454.038	2986.182	2977.631	1918.349	3326.91	3151.557	4139.542	2104.652
DNAH5	82.65598	604.1929	68.27925	114.4001	78.42075	100.9155	158.2317	104.2692	128.2567
C5AR1	94.57274	58.88959	52.2181	44.31287	195.4092	38.14177	188.6829	189.5532	52.00754
SC4MOL	65.93478	77.92168	35.41766	87.04346	83.95498	168.9711	63.29776	55.48279	103.3254
ST8SIA4	148.5699	33.08692	34.40652	39.0999	53.68861	43.98934	44.64138	60.43516	52.08531
PAQR6	211.6907	74.72168	69.00671	132.2572	79.84341	116.6982	55.71278	83.87048	182.3289
TMEM80	1815.133	1227.873	486.6166	805.6448	382.7971	607.4631	320.1842	151.9463	568.5993
HOXB3	34.72713	79.3008	32.38118	490.6581	41.31967	243.135	72.14113	78.35495	44.60779
MTERFD2	83.58129	98.96114	56.13958	59.46041	51.28725	87.13431	50.90718	53.81925	59.76913
ANKRD13A	67.98146	75.51316	2223.606	541.1254	2118.77	1336.955	2253.003	1514.562	117.354
WDR85	39.83463	749.7944	247.8623	61.79003	111.5946	64.33208	202.8703	451.8047	155.3534
SPATC1	60.89536	47.42805	62.08842	52.28198	46.738	49.92516	42.53654	45.93815	202.1212
CD81	14584.06	15308.69	19829.12	17376.27	20774.93	19598.94	19304.51	19910.66	13848.17
KIAA1432	1583.395	1739.939	259.129	1587.216	68.38478	868.1025	358.6961	57.87703	46.73681
PLEKHO2	46.38641	622.1806	890.7892	548.7081	1042.993	49.69402	1174.693	1393.084	45.10727
RRP9	39.79117	35.53249	34.56018	37.42294	37.35349	39.31197	38.7468	41.34597	35.2651
EDIL3	50.0671	42.51328	54.52954	89.05547	42.19423	60.24704	49.2131	43.46687	38.16425
CDC42BPB	49.80816	63.81809	757.3514	83.57639	551.3733	49.56847	493.6069	342.9075	75.33552
OR4K2	88.28327	59.03615	55.39071	135.7172	59.23047	52.056	52.2371	66.94003	50.30505
SPINK5	8072.716	4945.261	5493.478	1605.302	4787.158	3306.529	1409.246	3299.819	9854.938
SOD3	707.2856	1798.857	917.2264	45.44871	941.3533	63.84535	606.9196	1139.079	3453.385
TM7SF2	102.4269	47.58537	178.1673	58.58772	176.6962	55.94802	85.85281	291.4765	191.0431

NGDN	63.16216	40.78421	57.33346	591.8265	44.55657	49.03236	163.704	194.6239	127.9689
TNNI1	150.8268	64.62088	61.49685	306.5972	53.39867	63.89639	216.7648	161.9055	55.87767
TIGD2	158.3969	119.2654	94.2718	158.71	300.8039	148.4586	61.1915	105.1467	118.3016
PPM1A	602.9935	1039.733	392.8509	499.0305	424.3794	592.2283	295.4477	691.044	2432.878
STK11IP	67.80662	56.12321	634.2029	55.48812	638.106	525.8511	427.1731	321.5254	109.2735
PPP1R1B	558.8025	305.7147	182.0246	310.6282	191.1468	244.6759	162.9416	280.5737	703.5618
FAIM	35.09298	259.4824	388.4662	262.3114	200.0906	266.7636	409.9394	452.5664	31.64634
STAT1	38.74945	1607.564	2905.999	1208.165	808.1465	2764.741	4155.32	3690.574	63.98704
PTPLAD2	14072.36	14681.23	8994.244	10839.22	4939.714	6479.559	4988.397	5904.368	14378.01
P4HA3	232.7464	133.7484	68.72999	103.3413	65.03899	92.45876	66.25576	100.3548	200.1476
CRKL	2509.238	1459.247	4817.949	3971.792	6709.748	4259.668	5167.693	4126.922	1798.585
PDLIM7	6055.568	6631.208	2241.657	7202.017	1031.901	3764.523	3396.287	5997.554	3360.91
NIPBL	7094.663	7300.917	4818.299	6153.108	3030.179	4623.161	3821.769	2837.635	8298.529
PHF10	118.5095	67.24516	72.81639	79.36122	76.58083	75.67419	69.52496	145.8459	141.5305
ANO5	38.99403	51.05478	46.6759	43.94366	36.39971	68.50908	39.91211	208.9241	61.31854
SCN5A	80.73676	56.74792	62.02141	66.86553	39.08947	98.02774	49.54493	66.22116	150.8499
CRABP2	3500.971	5882.21	6140.212	4271.382	10294.93	9600.547	9355.77	10126.27	4615.599
DLL4	347.3753	198.4165	123.0046	217.5732	171.0109	200.8224	130.1231	167.7082	818.5507
CRYM	338.7323	199.9043	108.8148	217.7059	106.2238	122.5975	79.39149	245.0244	431.7273
CER1	264.7281	154.0333	96.05179	107.56	65.15513	118.1715	75.43409	90.32268	390.6454
OR4F4	87.43571	97.25459	75.60056	78.29643	59.45465	378.9756	61.07098	76.70387	105.7591
C1orf186	550.805	242.5219	57.72273	32.93414	116.5007	183.9036	58.22841	205.9069	70.5917
BFSP2	312.1466	152.0998	69.85394	128.7701	69.62852	109.7879	99.32319	65.79095	337.3125
CNTNAP2	62.87339	41.76278	252.0895	42.66117	316.034	320.7478	689.6154	306.364	56.47924
BATF2	965.7032	40.01103	49.40681	58.9572	43.27966	315.9175	176.6217	158.3551	79.55585
ALKBH2	171.8631	66.98648	751.62	700.4661	617.2036	381.504	290.591	650.5775	89.45356
SYNPO2L	9661.35	8825.441	8866.271	6575.099	5702.852	7142.823	8880.974	12194.75	6879.894
RAE1	86.0907	1226.75	42.1387	462.339	44.34636	32.24284	45.9985	39.01762	57.84972
SNAI3	35.01641	56.92271	53.85468	66.03413	45.90505	59.78372	128.7844	55.12793	95.44635
HNRPUL1	8223.235	9770.029	11663.87	11575.53	14095.96	13479.67	14448.82	7888.14	7924.215
ZNF354C	42.4024	49.36398	43.88028	32.15355	90.70714	146.3776	56.24325	53.3677	40.82947
DLEC1	238.1697	141.2838	89.72115	219.5242	89.67194	148.5838	78.11669	104.0509	322.0421
CRADD	71.48445	149.97	336.2134	115.3006	293.5182	322.73	63.76051	239.6499	310.5868
NDRG4	38.46156	42.22386	46.00423	68.8225	33.43592	52.51613	34.98823	47.11412	69.66241
DUOX1	8367.313	9551.808	15458.21	8446.252	17749.27	11610.4	11717.2	11844.1	7937.718
CC2D1B	55.33258	39.67588	183.3192	36.15882	48.56567	46.08343	50.56594	47.44547	32.70615
OR8S1	42.22672	46.48404	43.93832	76.01522	44.58799	100.3221	41.14512	48.10821	71.1812
MGC26718	502.8744	41.85097	281.5044	77.39964	57.72309	198.0696	75.64492	43.49518	65.95309
TPRKB	369.3279	230.6521	130.7592	239.4385	119.5603	175.9413	103.6568	135.604	500.7627
PRKACB	67.63326	49.32936	107.9407	169.569	59.72453	99.65831	69.47408	80.55289	550.9548
TTC23	5799.071	4350.057	5358.795	4882.675	4304.132	5893.037	4085.948	6656.877	5317.682
NFYA	39.73895	384.1785	122.6607	33.75256	33.56527	53.80119	70.27016	43.74479	31.8495
CLCN5	246.4361	142.2528	69.09396	163.251	72.79479	101.7345	195.2915	85.86038	337.8887
FAM118B	1184.969	1635.045	921.4456	157.6711	817.9127	517.393	797.8541	908.1055	4530.08
LTA4H	135.1362	497.8678	1720.003	768.326	1710.074	2829.862	2312.741	2528.351	126.9267
ADO	92.55262	121.2817	1305.832	394.1994	1164.49	1617.173	1777.491	2043.836	52.71601
CSRP2BP	46.79387	1871.492	945.6187	1463.49	1197.441	1491.849	1051.048	1250.88	1718.923

OR10AD1	217.9596	110.3699	67.65375	99.95382	61.25385	79.30398	68.52541	84.7097	311.758
COMMD3	440.8931	1138.32	3306.815	113.4074	6923.813	3770.09	5252.517	3010.571	112.396
DYNC1I1	67.7756	46.18985	50.49128	54.9788	213.6811	490.2191	114.8415	66.38083	39.27791
GFI1	350.5255	1229.719	713.7548	238.1256	269.4691	447.4927	325.9368	741.1945	499.1832
C18orf45	3977.169	2071.349	1501.354	4308.828	964.1483	1215.094	247.5465	910.6268	3225.073
C3orf54	644.7681	837.8823	3013.497	2478.293	4817.068	2101.27	3670.271	3163.325	156.9659
TMEM20	36.97944	37.8159	34.89959	40.95263	38.64533	53.69263	33.06893	49.17941	52.44593
ANKRD11	51.29616	82.93458	49.57004	47.5355	179.7443	41.53039	38.06414	156.5035	47.09021
SMG7	243.5048	166.722	83.1198	160.1757	56.01033	109.7328	51.82286	82.30424	345.0697
QKI	67.42361	54.27179	325.2305	631.3071	151.2903	40.66298	256.7133	195.5249	51.53296
KIAA0895	48.28972	513.2856	38.55934	41.31795	39.65669	34.2802	39.91883	43.1834	61.84369
PCM1	56.117	70.28635	547.1292	76.22884	494.9001	369.4485	327.4512	277.7853	53.61514
ZFP41	40.12426	41.81844	37.22528	58.76279	39.98493	55.46934	42.22765	47.24678	51.89715
LRRC20	2002.002	3549.068	4296.565	1926.177	2570.416	2761.728	2215.235	3801.82	1254.407
VAMP7	568.832	45.3763	424.0363	42.12091	482.494	553.0589	462.1822	426.7179	41.66724
GTSF1L	52.82909	34.30238	39.72938	34.72215	34.8765	35.0182	56.43449	37.19433	35.73231
TAF11	145.28	754.2855	648.0491	765.6107	270.0352	711.4872	239.7078	69.57915	225.7155
PPP3CC	787.217	343.9129	170.0268	51.97415	173.4234	174.0073	326.8745	106.0687	48.38008
GZMM	51.01499	61.91014	36.10904	51.03738	48.96053	47.51373	36.22625	51.69709	44.16994
SSTR5	34.97031	35.15258	36.29933	43.98838	51.61525	46.647	50.07441	40.8868	44.22056
RASSF4	8864.574	9211.247	12927.08	5825.392	9429.812	9506.829	11405.52	10897.6	6698.79
ZNF709	63.94276	82.44018	52.77597	53.93746	56.97767	54.89953	44.87734	51.44326	61.3709
ASMT	108.933	88.14536	63.61957	80.20783	44.60541	73.21387	48.85397	60.39854	52.84449
RALY	39.71409	280.7647	3590.245	2254.652	5951.42	3015.263	5082.171	2537.184	1364.593
GPR98	206.7957	116.5227	183.9755	136.8637	324.8288	441.6533	259.3546	374.7667	392.1107
SLC9A8	87.60024	39.46359	223.2074	41.1887	230.8322	56.00187	209.8972	252.2042	86.41081
ALS2CL	52.90089	34.85539	41.46846	36.81238	42.54115	35.79816	35.41481	54.73901	126.4962
SATB1	49.88501	559.7747	171.9777	744.8993	208.284	303.1659	267.0554	382.1337	1285.903
CDK2AP2	7604.24	5580.496	3153.262	5801.731	2413.969	1332.521	2883.645	2224.812	8514.975
P2RY2	32.47499	48.77761	32.73989	38.7117	37.56493	32.32476	40.6854	35.24963	51.66273
B3GALT4	67.0405	59.95082	42.68585	47.07378	859.8583	41.3774	151.2592	33.82615	35.09739
SEC23IP	232.1729	132.004	80.40003	631.0608	382.2757	89.94287	63.3227	66.43313	196.2411
CTPS2	34.49958	36.37685	34.3973	55.33887	39.26771	44.62062	32.98528	33.6733	56.58956
PEX11A	2670.729	1196.84	517.2817	787.9137	531.7231	810.4196	834.3954	1171.193	2488.643
OR5A2	49.88011	42.94585	43.91282	44.66361	37.41088	45.8618	33.56257	36.22641	36.38958
C21orf34	6274.52	7059.126	5417.718	4489.395	5603.535	4180.293	4986.074	4724.077	8268.899
SNRPB2	42.06908	852.2617	2166.216	1476.37	1936.748	1645.576	1827.823	2068.521	45.90054
EGF	236.0535	163.9559	89.69174	136.9673	93.73529	136.6267	92.27633	103.9847	485.5558
RBMY1A1	44.46032	63.19434	40.30444	58.9786	41.12202	63.29599	47.35543	63.50327	43.77267
SURF4	136.2461	106.3653	73.49887	72.24722	220.9997	61.24747	212.1556	586.4534	43.81226
RFK	462.7681	197.4099	118.0613	267.677	115.8565	149.2823	113.5586	113.4437	568.2895
TREML1	57.04625	63.51152	63.2538	54.98575	48.02214	46.37793	43.17455	46.14734	89.30976
TRMT112	1169.518	767.9828	495.8609	1153.618	485.9537	496.3279	577.5529	417.0655	1307.944
MKL2	430.8069	164.7898	86.5983	187.8103	138.6954	135.1126	85.20424	88.07629	537.6482
AKAP5	90.32755	60.67539	74.38367	96.10525	53.42656	61.14201	54.14578	88.17213	110.228
VAPA	195.6324	133.0646	407.7536	475.8202	173.899	467.8153	263.1492	294.5774	394.8844
TNKS1BP1	75.43137	914.972	706.5135	2042.636	608.99	65.7251	397.4928	426.6613	1624.448

AGER	46.8499	46.21183	31.11301	48.58433	48.96637	33.00037	37.52647	32.36768	35.6375
KCNQ5	31.15321	53.31201	37.87572	37.36094	36.00741	40.32157	43.14464	52.74937	43.87988
PPAPDC3	42.39869	35.83752	169.7908	535.0281	47.05991	47.5405	75.88538	248.0636	33.78403
ZNF470	46.10368	732.6841	48.26038	50.30238	166.5396	416.7556	349.4243	254.615	53.69407
KBTBD10	69.32104	405.3332	154.9207	315.431	198.3263	168.7782	490.8397	973.8667	808.1479
APOA1	34.06403	36.84251	470.313	33.83421	161.826	34.26859	243.1691	278.8341	37.45118
CMIP	45.03876	95.72134	42.34611	309.3981	55.40755	94.18882	47.04401	49.53671	95.66883
TRA1P2	147.6019	57.5362	50.31839	50.75003	53.14769	53.76782	45.21045	39.71103	47.30987
CCR6	252.343	1087.559	1865.43	605.05	526.8454	579.455	455.3459	446.4195	4457.313
C16orf87	427.4688	244.0616	134.8986	1142.792	258.5337	252.1546	244.0422	272.2001	433.4091
SP100	67.78613	45.68562	39.04298	45.97498	48.93393	40.40677	323.3854	161.2965	38.1245
STAT2	17105	18100.41	21099.54	19065.15	16187.16	20775.5	20231.38	19286.14	15561.06
MAST2	434.2733	280.5831	174.9442	262.5673	228.1531	253.6061	244.0203	229.8271	552.4452
CAPN9	36.14002	41.09444	186.7333	43.57657	48.28804	55.21897	42.83981	50.61712	54.17054
ECGF1	87.79755	1220.467	654.5972	717.0041	34.08458	94.82915	223.5809	367.6493	34.78234
CPZ	48.91544	50.67968	47.09907	43.93953	38.9967	37.01461	38.00726	41.98324	40.31933
ASAP1	59.23596	567.9768	2289.595	1987.64	4052.105	2813.179	3380.409	3317.988	58.25967
SSX3	327.6132	147.9941	69.14918	142.2081	64.41434	112.5233	71.0733	74.55671	167.3006
SLC17A3	187.6219	131.5401	79.06242	117.6254	83.01567	119.2704	62.87938	71.01728	300.9269
DDT	7651.671	5953.666	7809.766	6939.334	11120.56	6561.43	10031.13	10842.3	6628.784
NEUROG2	293.5818	191.0308	93.82638	205.9175	96.98035	110.215	518.5989	112.1141	410.2062
PNPLA3	98.72598	56.50743	44.10343	50.23256	56.64835	64.71609	45.95217	51.23835	40.07094
SLC22A18A	158.0391	74.34563	182.0969	114.6309	67.8623	119.7923	67.08517	201.1397	282.5984
FCRLA	39.32016	578.5643	1900.148	35.35042	654.5952	1369.212	1040.162	1059.839	30.97907
TEX11	40.51165	42.75836	46.49944	49.36545	46.81101	32.22556	43.21239	51.33495	74.07358
OR6B1	53.99245	37.89384	37.6699	63.02773	44.11576	57.63168	38.85236	36.81345	37.06571
MPV17	1927.312	249.2758	743.6398	324.4991	1089.623	1576.57	794.2413	613.6227	1839.684
LOC729692	330.953	211.6745	120.3367	220.3326	132.0662	172.3342	90.31348	119.5893	387.3626
SPATA2L	10070.28	9860.001	8010.431	9236.852	6490.214	7960.629	6087.24	7598.059	12293.12
PIGN	1076.272	505.0531	1724.054	59.21345	2623.215	1796.821	1194.74	1042.412	149.0717
DNTTIP1	59.90995	95.55878	763.1038	674.2795	982.9023	542.0156	773.3185	460.6894	94.16642
RNF122	482.9829	284.2863	166.3182	275.8554	132.9806	209.5128	170.4062	183.1859	632.0245
VTN	33.66332	40.62915	36.18385	38.12373	34.91558	41.92373	67.77014	33.03416	35.56418
LSMD1	517.2745	606.2018	936.6411	406.3887	634.8903	627.4488	837.1932	593.2537	37.61847
RAB18	839.5117	473.0017	187.7329	331.1229	180.6441	291.3941	166.6884	183.6792	38.94156
MT1A	34.42954	34.33352	40.53513	40.75697	32.82246	36.84586	49.44197	36.06163	120.5546
TGM7	270.5431	161.0755	76.10788	118.26	65.10294	78.83452	62.32194	93.07539	369.8492
CLSPN	542.6433	53.25018	51.49194	293.856	77.5713	161.4302	70.21846	106.9683	64.52056
ATG4B	455.2766	257.2171	92.45243	248.7065	52.42487	121.2387	65.44707	57.93773	481.6313
ADH6	406.8351	225.1098	107.2323	249.1045	122.5989	199.6975	118.779	158.2889	550.2363
OTX1	41.47964	33.92879	99.11379	35.55022	190.0512	32.35201	88.50465	32.18959	37.60628
TMX1	765.2498	947.1649	2184.483	1261.708	2378.284	3724.204	3127.433	2751.895	725.5533
UIMC1	9057.806	8033.794	11418.64	8953.983	12707.39	11970.34	9234.938	9494.272	4435.561
ZNF566	327.2746	128.2951	86.49166	140.2394	64.2431	105.718	59.06486	89.5954	245.1921
C20orf197	46.95204	57.14646	48.37696	48.33237	49.06497	34.56192	133.1532	51.99144	98.96695
CAP2	268.2734	259.7813	605.3412	369.7124	1007.157	1513.595	1502.018	1028.03	60.15427
FBLIM1	294.0366	587.1138	198.2061	113.3279	301.4241	129.2564	74.39742	72.33665	344.0302

NAT8B	1341.989	1936.382	3252.288	2450.943	265.2693	2445.714	1432.802	1835.635	3773.021
CXXC1	17358.59	17415.94	12613.91	16490.16	12310.35	15601.31	11956.64	12862.74	17899.56
KLRC3	57.73745	92.50103	50.6584	60.21761	36.84927	39.81041	43.11314	47.2488	63.37976
CELSR3	8057.46	4291.77	2994.233	8203.959	8883.266	5771.764	10074.19	1924.32	7653.333
PIGS	69.37873	41.55319	87.77861	63.04467	528.764	58.85353	557.1888	70.67971	62.15253
RNF185	55.43285	57.4014	97.27807	60.40015	705.2809	44.36148	753.563	436.8866	977.7281
CTBP2	56.93879	85.11579	43.41453	64.19656	80.15856	42.74177	56.33148	63.24547	69.21494
PVRL1	326.3702	206.1725	99.32968	192.1801	105.143	151.7928	91.5356	118.2507	387.3785
C1orf192	310.7409	50.88815	49.9695	97.18311	104.5065	103.0262	55.11417	44.34846	49.1609
ZNF57	90.64889	428.8698	174.135	68.64883	239.9031	120.7686	247.4324	192.0761	344.7679
TXNDC3	48.7565	67.82135	39.66269	76.6173	46.29731	83.67733	53.29067	54.2756	123.4446
CLEC1A	138.1356	298.0199	63.01644	127.1974	117.7596	80.24056	107.498	86.95827	107.0729
CACYBP	53.18364	176.7	166.544	41.22694	50.13403	38.91853	41.88131	46.87597	152.396
HHATL	441.4046	2819.801	1027.774	2168.805	710.6974	1155.282	2173.54	3996.16	1296.342
JAG1	797.7344	733.5438	1514.756	1879.809	1736.121	774.1409	1908.908	1995.676	109.0464
DNPEP	172.5555	124.4816	147.5082	189.7726	171.0919	105.5882	162.7452	97.21526	267.7852
ZBTB11	419.2634	44.24884	208.2322	198.1593	530.8071	290.9709	406.9583	153.4389	37.83027
SEC31A	980.1391	324.2904	393.1975	935.8234	336.6447	517.8712	390.0044	620.9258	276.6124
BAMBI	11705.91	8619.236	6494.705	5927.333	11046.12	6174.497	5529.263	5876.082	10405.65
NNAT	107.2683	101.2182	1525.422	1014.612	1887.626	871.4418	466.8174	502.7721	235.5876
CDRT4	1529.453	906.8285	3697.459	1225.32	3240.391	2678.117	2737.953	3311.502	40.52932
LDOC1L	835.5552	272.523	162.4434	403.7996	150.374	380.7474	192.9905	124.8595	762.5281
GSTCD	105.1184	96.67731	108.4615	133.5929	86.49359	113.0745	55.44072	115.4739	259.4815
PRDM4	5853.635	6795.72	9241.482	6509.501	8783.47	6964.696	7943.626	7420.023	5536.14
BLVRA	796.1829	531.5792	1782.289	1836.309	1608.555	1475.318	1870.69	2275.37	585.1182
C1orf126	68.43124	43.78719	48.76535	55.85782	48.95012	46.28544	48.3235	64.9493	50.75822
UHRF2	374.0154	198.4805	62.37982	86.62975	54.19695	153.1965	58.06538	51.4409	48.74953
YES1	3196.122	3713.236	4855.722	5578.055	6470.034	5935.398	6106.924	7539.131	1108.032
GSX1	115.181	92.8617	54.30149	46.58592	73.01021	73.74127	71.08595	86.22646	94.78905
MYLK	75.7649	1042.79	497.006	52.35452	1306.244	60.92274	727.9864	293.7403	41.11299
LONP2	90.78475	2067.333	2509.905	1779.443	3280.057	2015.354	2708.183	2425.554	4831.436
GTF2H2	680.8233	107.3693	161.4504	75.94328	50.76193	270.9389	81.69	78.6808	135.9422
TRAF2	43.1361	310.7032	456.0572	39.55602	363.3627	418.791	214.8685	298.9839	3013.444
TJP1	251.2347	379.8569	517.2872	375.0839	560.6543	430.1327	607.726	270.4079	410.709
C15orf5	6707.386	5594.764	3000.971	4022.766	2535.547	3643.363	2518.068	1188.865	5569.72
PLAUR	58.60037	1457.036	2197.408	3133.074	1592.524	2349.606	4374.284	2864.038	51.18179
MAPKAP1	177.0489	165.7952	102.0968	160.8311	100.1313	126.6217	75.67202	97.42316	123.9407
CUL4B	39.97135	420.9245	33.66624	39.36449	159.8288	35.02291	101.1125	89.65735	35.12419
LAT	12188.69	10964.92	10935.75	6555.155	6979.287	9268.057	6839.403	8825.459	12037.95
SNORD26	345.0075	210.6581	145.1496	265.9322	41.15236	132.2058	65.28266	88.40464	564.665
ELF2	1005.058	43.87542	753.4772	44.9052	1451.041	592.5239	648.3722	434.718	48.99755
CCDC140	51.34584	58.82782	70.25358	47.26682	60.87918	49.52898	46.3746	60.33178	77.18832
GNPDA2	66.87547	58.28788	151.6633	42.40313	41.13184	36.41247	79.5305	113.8699	41.11263
METTL5	2335.643	3232.397	2152.351	4596.871	1343.964	1605.329	1443.514	1086.06	1103.625
TST	3535.074	2453.927	4977.866	2602.996	9347.028	6741.035	4184.379	4184.633	867.7248
SNX2	69.54852	367.9343	544.1112	86.12196	643.3169	710.5643	580.2256	338.1557	307.6427
LYG2	72.73883	510.5201	59.98298	638.8729	709.4565	1033.595	53.18784	837.5396	85.0356

DAZL	64.97405	76.87143	59.91499	54.32244	42.70782	45.99688	36.97086	55.42244	41.38696
CITED1	98.9959	41.2392	40.35519	55.51244	48.30217	57.27969	48.31195	44.34772	47.26436
TRO	45.53683	30.74731	38.37052	42.90192	36.52779	33.38937	44.90866	32.64665	47.71449
LOC100101	40.54353	65.79987	56.61442	38.4336	44.54462	74.42694	37.91489	47.2976	48.89509
C6orf154	117.6578	83.89143	64.89556	145.5673	60.71753	82.7603	50.00817	53.71623	76.59276
ZNF775	66.10392	94.6012	59.43493	85.74368	729.2911	85.82325	440.0462	868.9971	213.1092
RHBDF2	8610.704	9191.278	9198.442	11243.36	10441.61	11772.8	9563.051	7482.822	8007.244
GGN	37.32155	43.47904	37.37915	49.62544	37.97399	51.5322	46.54778	55.82376	40.93668
PARP14	415.5021	514.2176	1317.211	597.7567	510.7228	1505.551	1210.706	988.4047	84.98412
ST6GALNA(	349.3726	554.9738	480.4602	233.6347	578.9202	395.394	707.7914	107.9767	412.4926
GFRAL	48.46296	49.35715	35.17179	40.84671	40.71212	33.27822	50.03321	45.76794	34.49294
GJD3	57.85229	45.00774	762.6152	84.48836	751.7804	50.97476	157.8608	280.2684	51.62244
KHDRBS3	1022.576	478.8797	1515.349	239.2994	2654.841	1572.069	1841.513	1439.636	443.4396
C2CD3	155.5317	751.5884	325.0391	1886.818	271.0075	194.9937	152.5411	490.8199	175.1296
FAM45A	1453.768	1420.969	1306.048	1727.062	1033.735	1638.576	1179.778	1562.601	1358.675
ZSCAN29	1939.32	1521.353	1089.176	416.665	1145.806	1332.728	843.6847	1920.882	212.3913
RAPH1	2038.289	410.8958	1132.794	2578.404	895.2502	40.39216	576.7246	612.5096	158.7901
SMNDC1	960.8106	1623.943	3217.064	2863.371	2821.57	3931.931	2839.563	2137.922	900.3033
DACT2	76.00983	48.10034	49.41505	60.27347	407.1023	304.0323	223.0054	39.09058	147.9427
ZNF26	303.6476	210.6834	1016.339	217.0592	735.9137	862.1218	856.2107	828.713	433.0318
KIR2DS5	111.7231	68.86293	164.0453	71.21965	46.72483	86.3018	55.98816	64.49442	65.97581
PSMA1	42.24428	558.2089	52.44596	626.4415	53.53925	45.26755	125.3604	180.3761	85.56873
OR4C16	350.2753	168.7329	107.2801	215.7916	82.67911	144.5372	79.38386	99.535	426.225
RNPS1	826.592	427.8073	1381.527	142.8529	2628.679	1023.776	2862.213	2149.854	987.4591
G0S2	3864.608	2273.404	654.755	2916.777	226.7483	158.7282	555.3785	589.7241	4356.553
LIX1	252.3072	189.4986	105.3285	201.7401	83.53632	146.8566	86.12571	129.743	530.0008
LIN7C	777.4055	610.1503	335.3094	523.6845	365.8439	327.5993	217.3825	332.7849	845.2136
CACNA1B	152.434	89.37016	66.01282	112.9617	73.40463	97.06315	65.01961	58.79362	206.0325
SPRY1	140.9148	239.1833	62.06144	94.95714	146.2007	217.4174	147.6854	281.1558	75.73763
RABIF	172.0284	136.7175	142.9003	128.8948	55.64124	82.32303	149.6304	121.2998	325.3708
STC2	93.37529	68.28221	1252.39	3257.356	1479.872	2542.119	2081.437	948.697	53.43657
TAGLN2	1885.392	1242.759	1505.823	3243.469	1475.497	1631.202	1045.608	1170.895	1352.197
C6orf1	163.1447	119.8792	61.02585	80.55333	133.7619	49.58191	65.55215	140.2776	297.4489
KIAA1919	611.7408	44.98907	54.88331	268.9811	248.5324	45.72969	106.2412	150.1865	37.13966
HADH2	60.46603	59.97704	366.5659	52.02954	891.9624	47.60152	588.0663	690.27	79.55245
BTBD1	518.1384	398.2182	216.666	45.39437	150.3425	207.3669	124.4686	193.9556	648.6978
CBX6	11343.71	12633.42	10234.05	8878.578	9572.45	11168.74	11626.5	9839.917	10733.09
CSH1	32.7282	44.68687	57.80221	38.14315	42.05688	30.12034	35.48363	37.05442	33.26658
CCNI	52.05017	2485.293	10167.07	3769.679	14872.49	5340.886	13374.96	11563.99	1342.906
FAM173B	49.12262	1191.122	173.9798	704.6553	662.1131	69.67105	334.3279	64.09568	51.95262
SPANXN3	55.27668	62.64625	33.02821	34.86658	39.88349	38.66165	60.14248	36.20238	34.96199
OR2H1	39.69197	43.99211	35.02359	50.46193	39.46307	52.61404	42.50305	46.75277	247.2191
FAM126A	2224.72	625.2646	745.8644	1147.48	467.043	699.9407	536.7606	449.0589	1017.728
PRSS16	50.4697	53.55063	49.49489	40.31379	55.94075	38.60822	44.24834	47.77279	47.91865
POT1	758.4572	405.457	520.9588	499.5621	770.2468	1091.234	815.9144	654.7962	55.53885
SLC7A6OS	1248.203	2345.242	1283.841	2078.951	372.503	734.18	964.5112	809.7599	3161.456
HS3ST3A1	830.0894	676.0659	444.7988	401.6813	920.525	517.2494	879.4223	499.7686	961.5555

NDN	2340.923	2743.074	4018.11	1754.002	3660.252	4026.224	3680.466	3586.89	1260.837
KIF21B	37.02725	38.22271	34.53823	42.31401	108.3362	35.29585	34.33121	33.5129	44.958
LOC728006	3964.06	3346.216	3163.454	3468.303	1972.511	4204.806	2767.444	2689.665	3101.211
MXRA7	83.85076	112.5435	48.04824	111.3291	61.42004	53.46922	275.1909	51.4878	105.433
ANKH	59.76733	97.35394	61.94136	61.50594	48.09687	69.23228	48.78833	51.37544	44.60215
GRIA4	40.68285	50.68034	49.1102	48.53343	36.07058	43.42721	39.94902	40.36531	32.03378
ZNF35	40.7897	47.87711	1522.304	1058.251	1721.54	1414.582	1610.055	1686.204	39.08347
USO1	888.8271	687.6736	427.8021	44.08559	1639.033	838.8806	1084.973	539.3409	97.80386
ELOVL3	286.263	101.4334	74.17353	75.55473	59.67709	70.50146	48.08469	57.81094	316.3323
ZNF226	130.1326	1896.183	632.2133	576.0424	877.6703	526.254	583.9089	532.77	1466.413
C6orf114	522.1895	768.196	1002.636	1472.878	1438.676	1295.009	1834.209	1264.977	301.5544
NSDHL	16136.76	15067.85	13500.51	14524.33	16231.84	13313.8	13081.05	18381.64	16610.55
UBE2Z	113.7287	95.46797	94.88033	60.68911	72.1392	90.21674	99.51011	60.69553	129.4862
TSC22D1	64.58188	70.02619	199.8764	37.21812	174.2954	51.05396	242.4795	120.4947	51.75548
GNA12	186.4988	122.5646	280.4528	966.4143	238.4302	113.3296	197.1922	72.82494	435.2395
RNF103	151.0391	131.3106	167.063	305.9648	159.7425	231.4886	147.8438	166.4397	182.3991
LCN2	2933.218	2851.19	8442.352	2629.774	17439.11	9868.097	9114.903	13664.96	159.1962
DOK3	36.09642	3024.654	1050.728	2187.806	597.369	1611.176	626.9442	1409.664	5530.892
TRIM7	162.0104	60.69511	67.00419	119.215	69.88222	97.24494	57.09723	64.58602	298.1938
WDR45	61.18568	59.51007	52.07584	70.08949	60.09772	62.46029	48.20898	56.60378	94.57869
EPHB1	307.2631	203.5115	90.42209	224.8376	213.0383	114.1931	197.8745	94.01982	497.4659
B4GALT3	1684.661	2075.434	1795.294	1765.432	1761.691	2096.534	1543.302	1655.046	2009.507
TEX261	773.1456	50.4758	1085.598	421.9262	1515.729	1112.802	1345.085	1579.465	50.51568
GGPS1	1237.775	1857.213	1240.957	1443.701	769.8421	1530.562	1368.727	1190.918	2673.023
NKX2-2	48.57608	36.98341	43.11708	65.22005	51.96835	51.24859	40.11513	65.80588	48.05309
DNAH10	32.30548	35.69725	37.57065	33.06481	37.30278	34.9705	37.28165	44.48323	36.20922
MYO1G	726.1176	224.1136	866.8033	374.0361	756.5726	465.8098	994.2455	316.7826	873.1946
ALOX12B	4679.233	8416.711	7136.935	2773.085	18135.16	7042.94	7995.25	13628.15	3691.764
ELK3	62.22401	380.6022	277.9995	69.72626	36.44466	66.40969	113.7535	46.76933	76.6819
ZNF662	56.01609	38.85818	83.48672	44.26372	44.91378	35.98488	138.7532	221.5036	32.56392
CDC42BPG	1231.733	56.68992	178.3083	213.4276	39.50448	46.08023	211.0625	153.667	37.1196
CARD18	64.82926	74.13938	696.0876	52.03034	1091.34	1352.753	91.23227	568.4041	32.78995
IRAK4	39.51028	45.21869	309.238	38.56861	144.2409	44.4493	113.1506	34.3028	34.05321
DLX2	94.45667	103.3452	72.62124	103.7177	64.98032	78.23077	58.9382	98.53983	278.8776
CCNT1	115.1018	144.1901	260.1889	106.4931	428.9184	153.9055	159.1283	125.2498	143.5858
KIAA1024	66.09932	49.38815	720.7244	49.91476	193.4834	508.6817	560.3456	513.0616	48.13225
CXorf58	50.02786	38.94569	42.04694	45.59165	46.56485	48.16883	37.5708	107.0833	44.16333
CNTNAP1	1479.171	3058.52	2693.955	1606.252	2996.644	2793.661	2237.625	1232.684	2006.337
NMNAT1	176.1853	93.18585	76.05898	163.2904	98.08371	135.5108	72.29058	156.5963	287.82
PQBP1	57.69452	57.51134	68.278	67.18253	50.39119	61.50073	40.11922	60.92759	149.3873
FLJ20699	141.2164	85.00772	446.5948	94.92814	573.1458	55.51961	747.5573	895.2673	154.541
MGAT5B	372.9407	218.3019	111.2906	251.2633	106.9905	160.1917	103.1425	132.5388	516.2065
ZNRD1	247.853	129.6456	96.34971	572.1419	441.3208	131.1007	434.0901	350.976	228.3552
TMEM106C	2346.706	1899.833	1688.842	2240.063	1161.197	1299.275	1410.286	2049.788	2794.605
DPP4	222.675	378.1182	317.0083	210.9687	731.0277	943.1878	514.3619	156.8887	148.7676
SH3BP1	139.9622	68.51875	290.826	94.11881	532.2429	75.02977	341.839	453.164	126.7447
PGK2	58.04877	68.61012	44.70388	58.53818	66.15057	47.8597	49.75148	69.68978	146.1977

C10orf92	98.36018	1475.421	195.1121	55.6288	52.47717	518.8517	298.5114	367.161	68.06091
SFRS8	316.4505	149.789	167.2222	152.0742	485.5965	201.7757	200.8512	301.9575	1171.391
RNF183	196.6982	170.5688	95.84969	185.1728	87.50647	110.029	68.39507	93.85973	293.9724
ZAN	343.8679	210.7616	97.80154	189.5538	95.38296	137.5764	75.46612	128.3038	428.1963
C4orf35	53.98596	53.19932	60.73299	76.56399	51.64064	53.62193	45.65461	57.25823	106.9819
ZNF263	350.3388	215.3374	1242.552	235.3363	2202.638	905.2302	1844.919	2245.688	505.8884
LPHN3	45.76823	64.78123	89.67801	54.05422	81.33559	53.11177	55.63534	48.91436	147.976
MYH2	7354.803	10556.68	5062.536	4527.3	1598.484	4400.063	4096.987	8879.167	8561.994
HSPC072	46.35535	48.64271	48.98855	51.39944	53.1587	34.08616	37.01389	46.08807	243.4107
PHB	8387.391	8850.256	9778.295	9807.932	8298.058	9348.428	8616.506	7852.975	7631.443
AKR1D1	65.25469	44.75188	49.56991	43.12781	52.93592	43.94947	41.15535	43.35477	100.2066
PRR5	301.252	770.064	557.2881	1384.768	815.6493	444.5091	447.9524	240.563	384.9261
UNC5B	43.76901	76.94021	40.30134	809.0248	56.49666	36.39755	102.5342	71.19011	89.02758
NKD1	45.40561	40.14298	43.87109	39.40928	43.52684	47.11356	45.4208	44.05912	56.08848
SMARCD2	811.6983	36.73663	133.2731	649.024	44.43521	243.0683	64.90779	35.60755	846.7982
VASH2	37.32454	219.0385	55.49031	35.35249	48.56395	46.14859	54.46296	33.92797	57.06143
DCUN1D2	2254.288	1065.171	1257.792	1303.733	1473.499	1912.975	1230.799	927.1761	1631.749
C2orf79	790.77	71.48381	2406.836	1641.165	3150.163	2253.098	2912.652	2591.831	1643.794
TBX21	147.171	63.10187	85.75883	137.8969	70.56666	109.4073	64.18054	106.5883	134.6496
H2AFB3	94.70451	71.10971	45.27319	94.444	35.68613	53.86638	36.35641	38.0192	57.40894
TTYH3	1574.723	617.2739	2477.28	1366.265	1280.333	2910.264	2959.332	1574.618	55.13924
ISLR2	77.13558	44.29158	39.19772	43.53013	128.9298	45.56493	37.79937	54.9369	44.99915
DENND3	47.74138	48.24858	405.3754	35.00625	844.9731	49.71038	829.1345	213.2781	110.5616
TMEM49	3574.825	2456.513	5423.087	3976.892	4323.271	6839.403	7821.409	5489.339	3334.815
PRDM12	57.43112	94.49834	43.61083	36.90639	37.41146	34.0688	31.24979	45.13948	49.31216
DPAGT1	7218.166	8496.46	11255.07	8948.06	10429.7	9161.592	9612.264	11649.32	8663.864
PRPF39	428.8171	117.6864	128.6505	293.3037	120.9456	154.4638	107.8014	105.1383	639.7877
KLHL21	160.6459	121.9141	65.59282	2258.913	59.57948	46.76719	150.994	46.47508	200.4758
ITGB3BP	54.08246	49.66991	41.88596	119.3529	130.7313	47.88667	97.41096	34.34637	46.71779
ASPA	2277.012	368.2334	178.1104	583.7046	51.22379	64.49657	133.9048	137.4885	70.68102
UNKL	62.2609	504.6656	199.3243	62.95849	63.52912	79.0335	56.66338	374.3487	104.9286
HIST1H1A	82.05109	68.23101	164.1219	494.1973	120.5229	51.31499	123.2426	202.4347	98.68605
C1orf26	460.8531	294.9148	183.8084	313.8099	151.3729	264.1507	167.9696	172.8867	499.9436
TBC1D19	391.4677	175.3015	270.0164	203.4656	141.6944	170.8751	341.7831	107.4416	338.1138
UGT2A3	39.22363	34.95958	37.16575	41.73219	31.36614	34.31728	38.11123	46.44764	45.85304
PDLIM5	287.0345	187.1522	430.8949	109.0697	329.7185	107.3618	193.8985	66.99873	451.0342
VPS37D	859.3769	1426.874	518.3893	665.671	454.7567	926.1708	594.6661	941.591	1648.371
PIK3R3	182.4376	126.8046	81.6498	128.4852	100.1356	154.0831	74.11487	117.9717	76.09972
JMJD4	60.00832	48.97555	49.16243	45.85392	278.6439	43.65636	503.747	355.7666	88.53675
NTN1	183.9415	88.80044	66.19821	167.8494	65.73788	71.4159	151.5649	94.28653	286.6698
OVOL1	59.82285	50.15376	50.94619	66.07369	136.5362	43.69986	58.87354	69.67919	55.45756
PSAT1	57.4187	1235.878	1055.202	1552.575	3468.943	1491.759	2076.026	2583.592	1923.817
CTDSP2	34.5839	39.67391	1751.561	495.7056	3251.126	1614.323	2761.413	1638.517	73.53167
AXIN1	34.11123	39.67644	238.5504	44.09421	323.4525	504.7988	34.92315	34.14754	39.88684
BICD1	56.82973	87.66454	40.99712	45.91101	138.2789	65.81474	57.55456	52.23664	95.18995
SULT2A1	87.55464	43.36012	47.90466	37.85659	42.98245	44.48526	42.23554	65.36475	45.36603
FGL2	1282.539	534.7028	712.8255	206.4602	294.2296	766.566	390.2577	91.3818	455.8322

CEBPB	15444.84	16440.2	21258.97	19164.5	23950.09	20910.25	22808.35	22500.35	15003.17
ZFPL1	1252.218	657.8522	1345.341	159.0419	1491.895	1434.958	2196.089	1373.917	83.05139
WDR90	788.4059	359.567	37.07862	46.6519	46.995	46.24844	36.90559	47.82763	48.02052
TMED1	13843.79	15331.36	12661.75	13403.41	11313.79	12871.38	12139.28	13151.14	14569.71
VEGFA	96.10026	41.87969	41.51412	1958.635	35.36032	36.77296	46.37334	57.15514	121.0243
FAM92B	182.5861	155.4029	69.32958	103.2677	73.96315	74.2298	74.73536	57.69427	231.4696
ELAC1	1214.222	68.67658	360.5464	167.5699	59.91375	894.1827	315.0295	249.7003	43.18853
SDC4P	77.97496	52.70653	59.28534	62.82198	53.31427	68.58123	42.91945	45.66692	109.6113
CROT	6597.887	6428.283	4689.549	3821.549	2358.102	4324.619	1638.34	3192.583	7285.81
MLH3	59.72904	54.88422	43.62505	49.49305	40.99493	37.34586	193.6725	54.57947	116.4958
SPAG17	49.49889	51.14695	46.68512	59.06781	93.67341	48.72813	34.43223	50.02408	45.71025
LYPD6B	83.92096	84.19793	60.17966	104.2105	57.24157	47.73452	559.4257	292.5081	76.22113
SDHAF1	73.18507	55.49374	53.43351	61.84296	52.98374	45.19479	44.21253	320.6776	118.5417
VTI1B	82.06551	102.2755	77.52361	95.28416	108.1481	163.7093	90.42963	97.33576	168.067
BUB3	12366.87	13307.52	14171.03	16446.59	14564.14	13522.37	14245.29	15459.21	11728.34
ACAN	36.31039	36.14778	34.78073	38.58875	35.05553	49.46608	37.58875	37.5258	32.28293
FUT8	41.08348	48.54364	37.44112	44.70355	36.18106	44.41975	47.47285	50.23703	131.2571
PI3	2485.347	3283.969	4428.459	4900.99	10283.35	5179.865	5999.737	11274.7	4859.017
KIR2DL4	42.41273	64.57555	36.75594	38.41448	47.74732	42.83536	48.06406	42.30384	59.69133
NSMCE2	1630.553	38.042	1600.731	987.642	1826.857	1922.019	3336.939	2861.765	33.26806
ART3	140.3919	68.47359	66.50595	83.77535	57.6325	82.26287	103.41	123.0729	100.4197
AKAP11	65.55576	321.6511	61.23184	83.28417	46.37307	208.7476	113.9958	103.9983	843.4186
CENPH	299.3643	188.8566	85.48113	230.4638	90.64606	122.112	80.58744	109.4392	343.3238
C1orf212	7161.926	6511.562	6170.327	7288.327	5313.612	6773.528	5727.577	5826.239	2931.984
ZC3H7A	4314.683	4918.487	4490.097	4775.339	3483.081	3740.696	3781.43	4956.058	5945.082
HYAL1	58.0632	76.54994	71.38109	49.2692	47.6857	88.75101	132.4595	263.7716	46.59202
KIAA0513	40.31405	40.19196	40.6722	37.99973	379.3606	66.95711	318.7787	33.68448	48.22061
ZNF296	11891.7	11129.99	9925.869	12398.22	9196.296	10573.79	8832.139	8239.444	15758.92
SMARCD3	59.25895	52.02908	54.38987	50.12066	50.8567	174.8352	54.37524	32.44043	196.7822
ACBD5	717.3408	95.11866	327.8836	694.3768	38.41427	265.7608	45.854	63.23393	48.33219
PDCD6IP	65.38519	91.5843	198.123	66.36617	162.5137	67.08747	90.57102	62.89814	51.06949
GNG8	60.96389	110.8262	68.90533	149.9613	54.68234	76.20371	141.9023	232.3126	249.3964
SUSD2	13598.82	11244.55	7890.56	7923.822	7186.202	10166.26	11326.92	9610.617	14048.2
USHBP1	64.84483	66.46485	60.45449	58.00624	59.45901	47.87881	54.6907	46.88288	119.7346
POMP	874.951	1078.892	1978.989	1314.093	1516.736	1986.272	1751.557	2472.505	1297.731
METT10D	164.3147	174.4968	99.37981	193.9684	156.3192	145.8459	74.31567	112.3177	486.8053
TAS2R41	221.1814	98.76089	78.58507	103.9553	58.64155	92.41141	57.01132	78.61322	116.483
ITFG1	46.21249	36.34699	204.7108	35.52437	264.0108	59.18598	283.5116	50.76243	39.25307
TMBIM6	2796.488	3585.683	9467.702	1602.701	12574.95	4781.045	12592.93	10992.2	49.74493
TTC15	49.41783	60.40763	284.4518	67.53005	48.53165	34.99134	42.08606	56.10493	34.73527
C6orf162	35.73926	36.92058	33.96302	46.62435	49.36435	37.3993	43.7659	32.17118	31.39641
TMEM19	475.5513	219.1597	36.0941	258.5528	54.40957	84.52903	67.87125	42.44126	78.53355
P4HA1	66.2261	292.8901	123.6413	74.10358	107.8056	164.0042	99.01154	165.7506	735.3306
CYP1B1	72.83628	316.0991	81.29514	79.24395	163.5654	171.9784	76.56538	303.1015	52.50128
RAC3	370.4041	194.0604	102.9684	193.4421	106.7789	150.7202	85.55456	105.0427	440.442
C15orf21	548.4707	83.98403	163.8627	463.544	94.11419	127.7085	61.81807	178.3083	293.7292
CPEB1	35.16008	42.49758	36.80801	37.84142	48.07454	39.4031	41.43165	44.63837	52.85904

NOXO1	185.2409	116.8801	65.76617	66.94269	70.6791	90.80362	56.26343	63.78898	233.0948
RGAG4	206.3413	65.27726	153.0842	753.1444	64.76532	91.83742	50.11557	95.43778	2188.17
BCKDK	27147.11	26137.37	23649.7	26632.96	23606.67	25150.59	23529.74	24489.3	27157.5
PSG4	80.38634	78.22398	69.43533	40.64214	44.27944	78.89577	101.4532	164.4487	53.5486
KLHL28	140.7759	98.33278	185.7793	133.8195	333.0857	74.76853	252.6022	232.1309	145.5231
RPN2	8742.283	9754.941	9969.804	8967.953	9378.256	8499.383	7914.982	10506.95	10178.87
TNNC2	53.90055	2113.198	584.2174	925.8953	171.3688	541.0784	281.1924	1922.262	1218.533
NME1-NMI	288.2728	130.1223	68.5422	174.3351	73.63826	111.9387	70.10044	94.6153	244.8486
C13orf31	1093.371	321.5333	293.4671	303.3782	210.5208	348.9509	239.6862	407.6011	55.78381
FBXL12	57.31281	785.533	725.3674	584.5475	907.7547	1017.879	826.2065	417.8665	58.30956
GAL3ST4	638.2472	291.7136	353.2797	288.8795	374.9053	585.2321	321.7919	273.023	338.2136
KLHL35	4824.833	1271.1	4192.491	1553.408	1545.198	3652.127	3613.698	812.2233	1768.328
FGF18	557.8853	251.9432	156.9491	51.86319	129.7577	184.4607	66.13894	79.9571	699.5208
OR2W1	52.07509	51.9984	48.07053	48.30375	46.08599	38.11598	47.9507	40.86295	43.30325
SEC11A	68.50157	440.6742	413.6016	480.9307	219.4339	264.2425	406.251	464.0154	57.05001
CMTM1	4067.572	3361.496	1844.027	2515.3	650.5743	1836.884	1508.217	1558.307	5666.567
EGFL8	61.1771	34.96453	46.45472	46.02813	40.58741	58.15042	42.78637	47.5437	41.08054
PRKG1	233.0347	66.13788	65.08591	129.1801	78.0238	114.4538	63.31564	93.62755	402.21
LOC441046	230.1242	109.1579	440.253	165.5877	537.0522	1292.876	290.2091	289.2088	308.9382
PSG3	40.63913	34.08939	51.95662	258.5132	34.57037	41.99374	55.43643	37.28262	37.96416
STK36	18518.92	15360.32	13760.22	15751.07	11506.47	15762.21	12954.03	11767	15493.5
CD300A	65.03621	49.27622	36.83769	37.20718	38.64787	48.6649	57.23686	49.43156	56.05632
DOT1L	578.3766	232.483	142.7935	250.9753	191.9503	223.2824	134.2599	164.8989	490.3866
CYP4Z1	52.55244	59.20122	50.41573	62.78079	44.92245	481.3301	46.87226	250.5531	125.8693
ZNF287	39.6394	422.3028	458.2271	810.8676	282.9158	44.05064	300.742	545.1478	33.8393
RTN4	2096.919	1250.847	439.3196	477.7156	177.277	428.5064	301.5549	290.3238	2857.338
GRM2	1781.629	1044.656	612.8852	647.869	367.2503	555.1	452.8863	304.7345	1125.95
PGM3	102.0805	417.4763	151.1599	60.73877	258.4106	269.6964	221.4189	168.7293	152.7545
C16orf7	3657.33	5821.598	6736.958	4214.939	5508.218	5333.117	4342.969	5873.206	4298.069
FES	1568.189	1746.222	4797.659	1497.691	3067.522	3112.758	3526.534	3035.292	669.5424
POMGNT1	7053.011	8373.277	9478.263	9059.805	7838.695	9249.697	9418.831	10319.08	7276.553
KIAA0261	83.23766	59.26572	142.2422	80.80961	47.04176	58.34971	137.4005	44.66056	236.4556
WDR79	1616.893	1072.245	597.4062	1288.257	476.6809	816.7008	578.2352	533.829	1215.372
TRDN	202.1019	153.1244	89.36971	161.5656	71.44769	112.7275	76.17053	78.46503	321.7658
STK16	187.4944	66.85839	50.26325	113.6773	67.91573	103.024	48.69685	62.47281	78.06718
PDAP1	247.4325	185.7053	89.75134	271.9659	96.43042	156.8792	91.14085	154.5308	661.5177
LOC205251	1505.67	143.8079	153.7509	534.782	159.4653	308.2208	263.5223	559.5443	2247.352
OR5B3	44.05396	42.29115	49.37714	43.25523	34.38966	54.50302	40.9456	41.08837	40.86212
CCDC141	40.39694	56.21666	40.7023	138.1998	47.45228	44.46618	43.94519	45.12084	40.05387
RPH3AL	432.7855	222.7558	106.1301	280.3086	69.96347	177.0377	82.82615	89.83601	559.8408
TBC1D10A	147.6565	323.627	125.2385	70.94754	301.8241	218.8922	159.024	228.232	145.4695
CTSE	80.96684	66.13961	76.59479	67.82005	61.74366	74.53381	50.67059	50.5612	175.5586
BMP4	53.75751	37.05703	51.96835	80.06688	56.32867	47.10519	47.16338	37.23386	54.96062
LRRC48	62.48932	45.3562	33.58513	50.3148	158.7046	61.58957	38.32513	57.35415	51.5769
CEP135	3385.178	2455.6	3446.58	1980.586	2332.393	4999.306	3625.719	2094.717	1858.795
ASPH	332.4915	388.4759	138.8139	290.9965	113.9307	223.5935	172.1636	278.961	84.74923
ZNF432	603.0822	231.8689	281.8843	413.2312	244.6485	419.971	247.41	210.7213	678.7877

ALPP	486.8798	673.2191	1036.988	750.897	432.7695	276.9121	524.0355	771.8586	1152.133
HAVCR2	6907.858	3542.773	6495.041	3893.433	2417.621	4337.83	4002.92	4130.325	3462.53
LACTB	1673.138	576.139	462.8954	452.3612	201.7001	607.1799	283.615	855.6455	182.4039
HELT	46.66132	48.27105	51.69244	86.00713	43.70547	50.35923	55.23928	62.94843	115.0749
HHAT	58.32729	279.9948	237.6146	268.8463	180.6046	200.9399	122.1575	138.3517	696.5622
MGC70857	210.8482	113.9619	343.354	475.5529	391.0984	54.06771	379.6453	204.1064	1147.732
GMPPA	548.8633	202.12	251.1749	238.2109	181.6662	331.6254	195.3315	105.6657	520.0873
RANBP3L	111.5007	65.24751	50.32251	39.07815	34.76316	38.0999	35.10452	31.65055	42.20617
TAPT1	161.5283	305.4958	592.1915	453.0543	701.5783	663.5306	599.4763	586.9578	122.04
MOGAT2	215.994	196.0218	88.90996	318.3198	83.50142	146.3178	95.11322	110.9484	491.9608
ATP8A2	282.1096	567.5079	203.6063	165.5025	182.1227	159.441	83.01351	117.5814	510.3142
HAT1	291.9435	198.737	103.704	197.7975	112.4142	159.6578	80.29591	99.91947	414.6097
SLC47A2	2473.172	5132.476	3926.925	4141.711	3817.508	5403.577	6642.093	6919.389	95.98987
OIT3	204.815	93.94444	62.26841	142.2639	65.2857	120.3386	51.63249	68.77613	254.5869
IGSF9	1233.083	1144.035	1420.815	3558.581	4822.941	3235.784	2880.717	2450.481	38.52465
C14orf180	201.2046	123.3933	79.82879	94.29854	64.1389	131.1735	138.3378	73.45021	138.2232
CATSPER3	108.7123	1086.686	1192.301	55.73156	363.1838	1098.557	508.6015	835.399	78.38634
KRTAP9-3	38.74416	59.13639	58.43061	39.35524	45.62163	45.34699	45.5719	37.37802	43.89814
SH3TC2	189.6389	203.9318	85.60288	183.6358	91.29625	83.26685	68.88296	110.4557	257.1438
JPH1	2744.737	2301.791	950.5064	2639.983	1289.805	1334.92	981.9333	2010.296	622.8448
MLXIP	44.72909	380.904	328.7733	58.85698	34.49168	363.8435	104.408	353.0399	52.40852
ADRB3	247.9581	55.0431	53.39414	36.79984	69.86177	67.57339	44.277	47.95378	55.03937
WNT2	297.2479	199.5253	112.7116	169.7452	112.0047	153.3395	94.29205	116.058	418.4514
SESN3	1116.863	939.2014	2015.402	1092.411	2112.235	1151.306	1819.63	1885.745	48.51611
LGALS4	64.2041	70.23598	46.40404	35.10742	53.74963	50.95313	56.56925	45.06184	53.9928
PDCL	476.8793	231.113	228.6589	255.9038	248.7224	205.7626	163.2915	162.4814	874.6904
OR7G2	237.2141	124.1143	72.93217	139.4865	64.60709	119.0436	66.83954	77.48584	99.48671
EIF3K	3922.235	4216.061	7100.363	4847.521	9022.153	5653.628	8389.825	10293.77	2991
IFITM4P	41.07188	38.63245	53.31538	42.95887	42.1208	61.29969	35.2346	40.22136	73.06419
EFCAB2	1179.634	1252.12	846.5219	442.5825	747.0773	860.2844	847.5833	832.2743	1127.337
FAM114A1	46.53886	40.49447	48.81269	34.097	170.2693	51.34826	70.87133	41.87223	43.92975
GADD45A	571.4331	272.0362	63.87332	503.5662	102.8214	57.61967	107.1038	191.3389	82.83537
TAAR6	57.25956	41.1003	40.08752	55.21623	53.14729	53.256	37.50101	46.44525	62.42976
ZNF804B	90.09447	84.1379	52.46239	124.1335	52.02535	77.09039	53.471	64.37845	242.0169
PSCD1	636.3912	502.4523	1827.563	1111.093	2731.039	1818.419	2315.852	1629.17	804.9012
PRIM2	406.3878	216.0029	243.3735	235.311	84.61986	78.98056	128.7579	61.06955	526.0624
GPD1L	497.1715	2871.121	4420.478	1213.217	3874.103	4841.41	4139.921	4455.359	1810.456
PNKP	55.62632	885.0336	768.5182	1020.381	642.9073	301.4554	931.2745	1039.037	138.3712
KIF24	42.27523	50.65974	36.92626	64.22967	43.76151	49.23679	129.7821	40.84337	85.98489
C4orf45	32.36756	34.90313	42.36858	39.0312	45.32409	56.2716	35.11842	41.08447	75.77826
AFAP1L1	1494.054	56.91563	201.5414	1266.92	392.9281	423.5468	347.6142	368.4486	107.1607
PUS3	95.23809	47.48673	64.66629	98.46148	66.24479	106.6805	70.86342	103.0478	57.18445
HIST1H2AA	79.75733	82.1223	56.99228	79.34946	56.06276	117.4991	48.10719	45.0779	124.9383
TRIM68	33.42041	39.75166	188.393	44.71628	66.42696	34.96425	93.61336	39.5943	57.58124
C21orf84	162.885	86.0957	67.77676	66.76317	47.9361	76.0882	49.36647	63.15064	154.3957
SCAF1	1524.316	54.87416	46.16579	86.02654	366.7668	412.5796	458.8687	338.7141	126.6015
ZNF141	39.19275	132.2584	100.5458	223.3935	60.35138	82.33636	70.08331	79.68653	58.00216

PLEKHO1	3139.66	3622.589	3176.12	2572.235	2243.693	2858.293	2772.682	3568.662	3009.654
ARIH1	40.27026	388.1002	411.392	451.703	174.3441	417.167	183.847	280.2231	45.58551
DGCR8	2509.707	929.5477	1250.262	787.002	1992.274	267.2427	1158.228	1981.73	674.7074
MAP2K1	399.8946	182.6352	555.8288	187.466	1222.028	1266.088	1242.974	1151.792	454.3927
HES6	6746.328	2026.375	3726.012	7409.132	2393.738	7991.612	4290.242	2932.641	3377.688
FLJ10996	49.55758	48.19513	42.98665	36.90783	588.2632	34.06962	330.5208	413.7313	37.17044
HRH3	45.87439	46.65862	40.50547	79.87833	36.8414	41.93954	42.84558	51.40398	128.9108
NDUFC2	463.6779	518.3638	1448.629	1011.539	873.3707	1461.423	1444.704	914.0129	592.508
GLE1	40.4864	46.89133	445.3202	51.08529	161.5921	54.64638	129.707	113.6487	39.93786
NFIX	4286.2	3669.263	4228.869	1762.475	5443.4	5944.438	3857.219	2488.392	4330.816
TMEM89	49.49099	51.91739	47.28485	62.64434	143.4547	67.68831	169.6723	61.96019	50.96071
NDOR1	955.4908	872.7979	261.9231	62.1204	39.8699	60.18191	322.8851	128.0007	61.65551
CRHR2	40.73793	46.74721	43.87185	37.39587	36.93133	64.3478	33.58909	192.5426	40.80508
SNORD35B	724.9243	124.9298	162.9644	105.0845	105.2198	178.3083	123.4127	137.7854	190.1072
YWHAB	4858.805	5954.492	8671.313	8632.033	10172.9	9806.479	10090.35	8710.473	5649.418
MYLC2PL	68.49717	56.55445	43.52485	45.41278	50.68311	41.97612	37.53883	50.87659	39.45833
EIF3F	1201.386	663.633	465.5237	339.1999	250.8799	270.6585	214.6829	382.874	513.2646
MSR1	340.817	253.2753	124.0331	264.3447	301.809	226.7952	256.0339	265.8114	348.0912
RASA4	810.5334	1017.853	358.4146	39.09676	320.7025	106.691	402.6258	650.0884	2893.18
NANOS2	47.23216	42.15284	52.02005	59.51441	44.0457	58.81124	48.14224	55.87203	52.09624
KRTAP8-1	85.99212	55.47725	56.55646	72.96611	66.83344	51.69304	50.34596	56.91919	65.23676
ROR2	2640.005	1735.562	1537.456	668.8557	1862.932	1160.784	1180.355	2256.12	2926.881
CRIP2	858.5947	1067.979	1010.812	904.4888	4568.924	1716.056	1570.925	2372.052	49.55103
ZBED1	1908.262	241.468	1750.802	1075.899	2730.663	2290.678	3336.63	3096.72	44.0529
GTPBP2	4348.607	2419.686	2417.044	2780.067	3429.064	2119.123	2631.667	1650.938	1502.083
WDR18	20018.04	24690.79	26888.44	25662.46	28014.09	28846.89	32186.19	32838.98	23472.54
CELSR1	116.3924	912.5154	57.41595	79.85037	51.64824	59.80401	46.54708	37.95086	84.76836
GPR50	66.05953	48.9276	40.10643	42.56465	37.2671	35.89592	43.19269	41.27528	112.222
TAS2R19	39.95383	71.68959	49.10023	53.91038	38.12023	241.5751	46.59418	37.72846	31.93359
TCIRG1	80.96468	406.5923	323.4567	682.7206	379.616	424.3004	243.1475	565.0462	930.8237
CCNG1	671.2676	593.6937	483.3359	585.3785	507.466	401.2972	529.0157	748.6647	940.2745
PRIM1	43.37212	56.47337	65.59222	1713.698	44.616	51.46864	111.0653	46.34347	186.4116
RNF220	41.30274	2725.304	4281.945	2974.204	6979.883	3577.843	7921.589	5057.175	81.6693
ZDHHC11	120.3431	1020.056	856.1138	148.3631	7905.053	1429.207	1321.494	1066.115	443.5803
DDA1	8641.881	9290.96	9387.986	10724.32	9532.019	9697.255	8836.257	9262.895	9062.673
RMND1	300.6597	297.6917	185.5181	259.7282	87.7013	292.4448	116.0725	146.2843	328.075
PDZK1	36.72712	42.40163	41.10626	48.10134	34.44339	35.88865	37.40123	40.92271	35.36845
MAP3K6	1237.006	2042.713	3335.274	2670.149	3297.344	1784.191	2929.97	4118.776	4141.459
IFFO1	72.03131	970.442	50.43305	42.78534	48.74439	46.67117	50.34479	39.81469	54.26719
ANXA3	48.90833	57.97871	425.8752	363.4335	409.0354	395.601	364.473	155.0556	34.05172
CLTCL1	38.57519	47.17143	41.7712	39.45817	36.09453	45.56665	76.57638	51.20895	65.67526
C4BPB	80.87226	49.35611	47.4745	97.9012	39.86525	59.34386	59.43011	55.44552	61.98936
PAQR8	2085.647	1913.424	1450.467	854.4384	1428.19	2728.625	1022.068	1206.147	1879.835
FAHD1	46.77679	49.59224	45.95414	39.79925	200.3666	52.61301	41.89788	60.89967	49.84137
FAR2	54.20135	39.11866	39.61264	56.85323	40.66624	48.23579	34.37441	43.03313	39.32944
SMARCC1	38.2088	1036.177	1518.721	50.08778	2485	469.5281	2184.462	1259.014	95.02808
C1QTNF1	40.2395	59.07532	36.4455	58.58897	155.0335	52.73119	92.31991	54.46987	64.09605

MOGAT3	120.4659	54.91815	49.23091	61.92401	40.71636	54.7855	47.22295	47.2756	57.16805
CNDP1	69.67628	73.1561	53.4334	61.65073	45.75657	68.14173	38.62556	48.17841	45.53484
ZFAND2A	13815.27	12149.21	12524.33	12473.23	10075.61	11625.04	9931.192	11587.48	14607.08
HIST1H2AK	1125.082	140.435	82.49937	407.9456	92.72875	132.5289	94.85062	90.04854	223.7477
MAD2L1BP	48.54347	49.24346	34.09959	77.50615	43.52585	40.91285	124.1257	41.8017	63.6425
RNPEPL1	242.0724	158.2148	102.317	142.9033	96.67271	125.7396	74.13972	105.35	228.0107
CLCN7	23827.22	23599.82	23857.21	24760.57	21395.91	23142.29	21325.14	24311.54	26577.69
RUNX1T1	41.70637	53.60875	68.50746	61.42333	59.03442	56.62074	46.38559	53.3563	162.7878
RPS29	3499.144	4836.482	14002.25	6034.806	15882.61	11168.57	18335.11	14355.97	3880.496
FSD1	55.16382	34.17463	44.0364	40.43626	44.85611	45.62882	37.84074	90.12516	601.8399
CTSL1	3945.946	140.7389	605.2469	1101.592	207.7531	90.77785	550.5588	572.3926	2573.392
C19orf42	594.8153	470.5525	147.4046	349.4153	190.7736	138.8583	183.7721	277.983	85.64648
EXD1	72.8156	57.35938	44.44058	76.77785	47.96423	120.5533	54.00912	57.04816	99.76668
OR52J3	49.63756	75.56133	44.86883	53.07714	35.58891	40.93694	42.80386	37.46884	36.31144
GCHFR	285.5683	186.9263	106.7534	186.8801	410.5867	121.3503	113.1647	107.261	262.3193
C5orf28	71.8365	132.3244	73.10938	66.73224	75.652	49.33979	82.50467	44.38648	120.6048
PIAS2	495.048	991.9349	1250.691	286.0508	700.5054	214.794	498.5582	672.2002	696.9676
SLC39A4	43.95351	40.9071	40.7669	39.61952	35.87805	54.65451	32.62865	36.06517	66.59269
PANX2	6257.65	3394.888	1189.579	1523.627	1020.719	4961.466	1248.241	3000.306	4545.614
TRH	59.87913	43.64858	35.82217	37.79182	41.49393	53.30629	39.16938	49.54623	66.16579
PYDC1	111.179	88.22112	50.62638	39.73905	58.51899	46.00229	44.53127	38.0051	42.64165
DSPP	101.4091	58.40292	52.09425	70.78854	54.5132	48.24309	40.7609	37.33413	97.79092
MATN2	1457.93	2416.509	2807.907	60.59269	5137.675	2627.424	4938.845	2641.144	1777.01
TERF1	56.32301	200.1381	65.34865	61.54117	141.1962	49.69803	109.2603	116.1204	47.64029
APPBP2	205.5199	107.4747	76.33517	90.83282	62.5653	109.9412	51.19849	65.20004	163.2659
TAF2	78.92498	60.88078	73.84031	286.3824	68.83281	187.9491	86.1617	71.97638	64.50021
UCHL5IP	50.56975	83.07281	67.22286	61.73165	55.45742	57.45123	52.8279	56.25549	139.4271
LMO3	165.2528	114.7499	60.81614	92.436	78.36555	102.3765	68.68793	56.41718	134.7458
SERPINB11	702.4555	321.7089	57.69723	65.04002	450.0618	262.4012	91.06291	126.6074	576.1731
PTPN9	111.4552	331.4702	39.75375	343.6067	131.1114	31.82024	183.1225	53.35514	128.0987
TBPL2	41.25476	36.33962	41.212	45.87319	46.63447	43.22045	35.28677	44.28368	209.3911
CAST	177.3834	62.678	52.7749	94.14254	60.58542	88.24792	91.73439	62.11647	178.9207
MRPL28	299.7715	182.503	97.1284	181.6849	270.4683	76.51205	176.9233	339.3954	299.3358
ASGR2	844.7697	54.27074	59.59984	44.48323	47.82395	44.87402	127.5725	147.7536	56.08526
DEFB127	33.2437	39.04323	35.12275	41.63338	35.67565	40.01614	38.66076	33.63768	38.00768
TTC29	68.45272	45.46474	35.69188	61.55547	34.20966	48.60341	43.68401	44.78725	37.37046
NAGLU	302.7964	310.3085	2284.755	1825.832	2716.399	586.7042	3377.58	2220.696	2327.807
DDX20	3447.12	4132.553	2322.266	4511.329	1429.982	2857.185	2018.043	820.2356	3796.011
AKR1E2	61.19809	65.80331	133.5273	295.7221	195.1902	40.94443	139.7674	57.05494	811.763
PKIB	76.20431	61.47114	58.82981	92.18534	97.85976	74.51516	40.64171	40.91252	88.8543
PCSK2	66.97883	102.0648	41.78254	36.96743	64.53713	58.03312	50.48521	35.90768	37.15082
QRICH2	61.04165	33.22564	121.4608	84.44411	114.0419	48.88445	101.4277	37.617	53.61282
NEK3	318.9285	228.576	113.6795	184.0314	410.3197	164.2714	303.4523	197.7757	403.7948
CD2	127.7283	120.0216	714.1077	77.88835	696.2744	363.0251	469.3315	975.3654	84.41003
TPPP3	69.59955	36.84733	46.99493	42.20815	45.3618	40.70415	37.3792	38.20877	65.89742
GATA6	115.5622	58.47243	77.49847	86.75642	57.92468	70.5662	54.82611	46.25189	103.1858
SLC38A5	973.5049	366.4869	131.0813	1302.525	237.1568	313.2892	198.5245	209.6445	1192.412

ZNF101	741.4407	418.4432	606.2638	509.5255	305.1405	804.28	345.4677	416.059	91.84047
KIAA1715	230.7589	182.3296	76.40848	459.6961	86.82574	128.9483	76.44154	146.1365	249.2272
WRB	49.35149	41.54562	41.86703	87.77132	47.67674	48.61646	47.28857	50.39143	45.16175
SLC7A8	45.0663	1842.932	47.02188	1098.643	424.7644	1003.792	832.6103	414.7407	37.80794
IL20RA	462.4435	240.7164	172.1682	282.2222	330.4101	224.9438	270.26	327.7266	594.2589
RSPO2	47.59418	46.40532	34.91817	41.2079	41.09098	33.99444	32.03475	36.6417	37.58635
BCAT2	1596.961	594.544	175.9026	471.8545	423.8051	87.72588	375.3799	441.4179	233.7687
ZFYVE9	56.81414	47.17352	46.27408	36.18845	36.71689	49.63783	39.38409	66.33295	56.3267
GCM1	149.6347	144.5679	64.60455	173.2828	70.60383	101.5943	65.58862	69.09039	235.0102
CA4	2295.361	2463.587	569.666	97.60072	78.38631	1171.479	575.4117	3192.156	3301.43
SCRIB	95.32613	52.81771	48.37817	62.86722	46.72238	36.76214	54.25175	43.75809	44.10346
GYPE	105.7145	36.94372	142.9332	37.96516	38.15499	40.13042	36.01691	40.7701	36.3451
ANGPTL3	275.2815	159.8413	79.0085	213.9542	73.9323	101.6532	73.75902	108.0124	274.5888
DIAPH3	209.6462	121.1354	79.73272	95.94273	69.30516	116.1892	98.63693	200.3685	221.1802
KLHDC8B	106.0785	1547.625	2691.546	1434.796	3097.655	1856.387	4314.69	2768.451	155.3126
RPP25	677.9044	507.1316	134.5321	334.604	303.676	453.4341	188.9322	287.5738	948.5283
MAPRE2	336.3869	267.6708	237.7948	318.1733	326.4092	263.7519	229.4887	247.7539	584.3633
SMG6	184.453	267.5951	102.8809	375.1454	98.43085	61.72181	97.05734	113.5009	213.949
FERMT2	4553.204	4777.414	3397.918	5812.779	4092.824	3618.71	4573.092	2952.227	4882.542
PDLIM3	88.43771	2068.533	768.755	113.7202	502.8855	1128.048	746.2307	969.2355	1547.34
C7orf69	57.12235	42.62082	43.75049	45.05695	49.57973	81.82738	35.39083	53.99776	40.50353
HLA-DMA	6232.051	5116.533	2471.954	2940.347	696.2971	2921.081	1659.209	2579.545	5167.661
SLC39A8	468.9745	44.00144	68.28298	65.90293	54.60267	204.0995	60.99376	55.08109	88.3474
RCBTB1	70.13245	83.93841	67.69334	89.37064	87.22556	61.5275	109.7413	218.3517	90.83588
PYGO2	1245.572	488.2601	254.2859	115.6495	154.7543	101.3535	257.6072	227.7121	476.2935
KCNK10	75.25584	65.29069	57.60745	61.18602	40.73828	48.51406	45.16981	35.68376	52.87175
ASCL3	53.45889	35.26709	35.54494	68.83798	49.30091	48.69453	37.73296	42.31502	52.28269
BMF	1135.89	33.38276	32.69844	35.11437	159.2994	34.34765	101.8206	194.2731	30.66075
FOXP2	1552.319	148.9725	183.6763	171.6802	164.3623	98.54322	69.24971	69.58709	383.0878
RNF166	39.38327	43.17212	130.3613	47.54596	43.06956	47.35376	90.19036	57.75046	91.30025
CCDC99	29.80635	38.44164	823.1346	370.8996	104.3733	676.6818	402.0134	221.2308	34.23938
OR8U9	39.84856	53.14635	35.80634	34.48552	43.42809	44.58584	36.92514	49.85067	49.35257
TSC22D3	35.94107	39.39119	139.4425	54.97345	33.99878	32.82373	74.00197	43.27354	33.08565
LY6E	4754.542	3077.673	2574.269	1636.685	317.3738	1853.957	2152.461	3251.958	616.6359
ZNF429	110.3045	73.63298	46.13328	91.22041	57.96508	84.33376	50.69876	64.11249	205.5191
ASF1B	99.50048	259.2697	498.6238	560.2319	653.7437	521.5549	1044.547	765.9718	57.66047
IQGAP3	445.6358	172.5139	147.9498	237.1645	114.2486	185.9813	99.68388	86.28366	562.5049
NCL	548.7659	245.4494	138.8765	485.6798	164.8399	240.7991	137.7428	156.3195	637.3196
CD9	54.08911	48.06433	42.37314	62.87379	543.2047	638.7619	106.1131	48.44357	2007.698
TPST2	38.45771	1589.706	1264.68	1921.621	776.463	1145.777	465.9647	622.3702	854.3309
YSK4	49.5107	31.9075	34.74257	43.00924	34.46296	38.10485	31.15447	36.6514	36.97045
AMBP	161.7439	69.88065	61.12106	125.3128	65.33471	75.19128	65.49333	74.63659	254.5511
PTPRA	253.2825	174.5098	98.87771	124.6218	85.4952	143.7756	74.62079	107.5135	294.2255
MRPL10	858.9163	1338.339	336.6725	50.36736	280.633	951.1029	314.6071	504.7529	56.9367
SEMA3C	42.71952	50.38141	55.47303	45.80854	35.53529	32.04016	157.6465	74.49386	102.5863
PLD4	289.8224	173.0434	120.8277	109.4149	76.14365	95.70363	61.80757	93.96326	460.4762
WDYHV1	118.4714	66.90211	75.66726	343.4485	123.1719	91.96913	187.127	73.28469	108.5974

DKK2	175.7903	97.05299	53.40142	61.37814	52.07116	68.59232	48.35322	62.97594	54.63294
PTPN12	887.6915	637.0967	476.5198	1217.671	305.4686	529.4139	305.4531	417.5176	1046.381
MS4A3	46.29525	57.30584	44.07418	50.08012	45.11781	52.77246	33.63191	38.63598	60.26944
OR10AG1	95.45682	118.3789	102.9579	152.8104	68.85242	87.25955	61.97684	71.05212	103.8083
MRPL24	129.8268	294.5207	360.3133	76.68156	391.9671	314.9134	575.1146	374.2316	851.2192
CIT	2412.08	2493.873	2196.553	3786.75	1186.637	3101.218	1913.406	2368.364	2732.95
HNRPC	125.4377	157.3439	171.7359	114.1886	218.8698	128.5392	302.7507	90.577	86.81544
ADRB2	5481.899	7516.924	8622.202	12292.63	11247.33	9036.323	10874.32	12234.99	7079.598
DCBLD1	1278.359	1467.685	1110.025	2608.413	534.7103	1443.418	1270.402	1780.671	1916.365
EFNB3	35.72956	37.29679	284.9897	35.9205	2477.795	1304.698	846.647	581.7122	40.2757
KRT3	23209.38	3098.339	11479.48	721.5763	13980.27	7340.421	1566.106	2048.28	10177.14
CHP2	321.2403	83.38967	96.66029	104.8636	250.631	94.11233	95.97153	187.2772	252.6733
MAX	74.7171	37.09715	49.02575	37.62176	35.32664	44.25876	33.75086	67.75381	46.27614
PTPN22	594.3992	359.4815	161.9407	39.19188	70.0666	268.8759	135.9905	182.5091	118.5311
CETN2	2296.085	728.7572	1415.596	1547.056	2099.349	1571.692	1881.106	1322.67	1022.148
KIF20A	43.97768	52.31603	493.9228	59.16332	305.839	40.34835	323.08	490.5164	45.44737
SALL4	36.06534	35.93997	135.9748	41.85749	107.3476	209.2158	275.6687	277.6642	44.15393
H3F3B	2099.342	3400.934	3680.371	3202.574	4083.659	3347.844	3483.163	3979.983	391.3741
FAM105A	585.2011	391.5395	357.7095	407.8869	343.8638	569.4188	231.557	553.5783	800.0272
RBM41	215.0783	151.4014	162.5633	153.2407	341.3976	314.5212	173.5269	313.128	362.257
EIF2C2	14439.4	15253.06	14002.73	15037.69	13428.17	12638.01	12871.6	13348.53	14707.73
TUBG1	3590.188	8012.891	10530.22	8396.798	10780.12	11823.17	11461.28	10493.29	4573.201
C1QTNF8	167.9074	102.5384	51.72565	100.2419	57.75467	103.0862	65.24778	54.22636	253.8436
DISP1	143.1366	948.8521	744.4786	419.2559	511.3446	1115.598	686.0964	645.2097	1160.037
AMDHD2	107.3178	113.7265	57.46072	64.26642	58.08961	74.34554	51.3503	240.604	120.0948
PDIA5	132.5143	146.5788	92.18946	119.0993	269.9977	139.461	111.0279	78.2708	85.87108
UBE2MP1	7680.223	5764.253	3654.153	7150.104	2607.249	4138.205	3047.391	4472.721	9344.948
NCOA6	215.4059	90.64283	296.9716	643.3975	1297.62	583.1347	808.0869	695.6516	104.4505
DYRK3	45.68844	147.7215	40.79185	40.80474	36.04545	38.01708	39.27406	35.75594	41.79833
KRT72	87.99315	130.6402	602.803	81.47395	424.2501	549.8682	60.04263	46.23762	319.6027
ACTR1B	3628.778	2844.668	4483.22	2162.633	6098.76	2142.721	3957.402	3265.48	41.77242
PPP1CA	366.1606	219.9401	306.7	618.8689	164.9872	340.9822	79.84745	112.9534	460.3906
TTYH2	40.31292	35.02089	154.0482	38.52743	275.1189	36.43007	230.5616	168.7657	33.6031
ZKSCAN5	36.32946	532.5999	843.2015	778.7956	1013.46	1182.355	1069.278	1234.385	39.36597
PARVG	8834.934	9526.933	14121.6	6899.329	5383.133	11587.26	10190.95	8665.985	8520.758
C5orf38	41.10534	35.28786	37.01491	49.47883	35.7161	33.7613	38.99527	46.42081	45.68409
CLK4	568.7054	216.3481	112.9657	253.523	117.1614	199.3695	138.0761	109.044	745.6407
FAM44A	135.3484	337.2303	363.2746	48.47586	207.2896	224.849	200.1774	138.4393	47.56142
PRICKLE4	315.907	554.0809	353.6221	573.765	632.8138	221.3248	502.0671	653.0001	137.4119
TIGD3	251.3605	111.2812	53.38556	53.54268	68.55806	89.21986	49.09937	62.66236	167.7242
LOX	35.43067	38.2927	34.87504	32.32122	260.0818	340.7108	146.0997	57.01942	92.22686
ZFP1	133.8408	219.6552	57.17868	120.743	60.51614	142.3147	55.51764	45.69499	132.2786
C5orf15	1803.167	4226.222	4878.337	4532.056	4168.435	5360.957	5834.337	4810.676	1489.871
KLK11	15419.94	14030.34	14356.54	12856.06	12962.25	13498.76	9702.643	13585	16003.48
NECAB1	81.71312	55.02524	49.82767	56.85252	41.84352	42.00036	42.0696	48.75665	38.10472
TRNP1	286.8132	105.8223	56.22572	95.48488	46.56667	70.62087	46.60611	68.90839	37.14175
SCN4B	145.8955	105.7396	45.1322	79.47243	129.9985	68.48458	66.03348	77.74144	330.614

C21orf63	312.7937	202.5496	914.5397	175.5963	1164.587	123.0896	300.4639	715.7202	260.9078
CLK3	36.66884	51.9354	52.91116	38.07927	52.99818	41.76643	162.8766	54.47503	47.39223
SLC12A9	7068.212	7507.399	8732.947	8506.737	7813.293	8614.994	8064.346	8690.58	6185.82
PRSS7	63.15458	72.43459	49.32304	52.88295	42.63507	39.51672	39.87654	34.2037	149.8492
CCDC89	67.65005	72.71651	70.43447	66.331	199.2001	42.35028	37.32988	139.8476	90.978
LINGO2	33.55046	58.52863	162.1607	47.30978	46.26348	2145.148	34.97523	285.9333	42.50664
PAGE5	37.08925	33.47305	33.50482	31.89237	39.4819	38.45596	31.7013	31.41463	41.03048
YKT6	304.1436	138.7048	82.19347	124.4797	83.61023	163.0533	73.78136	110.2193	500.6245
GNAI3	678.4726	1034.463	1887.795	2096.296	1608.202	2090.286	2289.446	1457	951.6901
LRRK1	266.6076	89.27707	150.2069	281.1123	189.1709	196.9397	147.5221	154.4631	156.5404
KPNA6	70.12184	56.76405	850.5904	637.4808	1132.894	508.8425	743.5013	454.6182	153.4008
ETNK1	1420.107	3091.286	2377.087	2597.999	2038.593	2133.867	1408.106	2446.052	1283.548
FLJ36031	55.17221	50.60712	59.81464	64.73439	137.6242	302.5998	79.86314	51.33303	89.49084
C12orf8	772.6492	37.99986	40.40972	50.48486	40.8847	42.8036	44.29947	44.67137	566.5297
SIVA	1757.766	1595.163	1496.853	1700.217	1136.743	1321.016	1143.265	1267.754	2073.739
C13orf33	40.42674	170.6491	72.41949	51.59816	54.47801	171.2943	94.83823	41.22155	49.85539
SMYD1	42.77626	2013.189	897.1892	43.62071	35.95333	1249.604	1720.995	3050.82	63.74128
SLC8A3	54.39302	100.3589	50.25007	85.1693	37.16911	45.10273	36.58459	298.5309	165.4075
GRM7	31.46815	38.733	33.06431	32.86107	39.13104	33.04524	33.14271	33.64598	34.99507
SDF4	33.75389	33.01571	128.3941	36.28407	36.21488	36.07408	41.17896	45.45146	52.79093
CARS	73.5163	330.9081	296.9404	365.6955	738.2088	218.9475	489.037	402.0113	171.2677
DNAJC6	83.87852	104.08	63.63807	57.72601	69.4046	61.34615	62.14995	47.07135	126.467
PHB2	2285.169	1110.669	1045.839	559.8287	1060.317	206.2483	498.9097	626.991	84.67897
TUBGCP3	36.82442	254.7905	131.9558	36.01731	108.6676	39.48434	85.35142	90.98637	38.14835
GTPBP10	283.2592	158.9142	89.67548	174.0442	77.05441	115.273	81.73715	95.14088	294.9148
STX18	121.2299	57.99384	60.28324	51.79354	55.96117	74.6237	54.9299	54.22035	73.15931
KIAA0240	59.4973	465.0228	399.4932	435.0458	551.8434	673.888	512.3965	624.7479	46.73051
ANKS1B	106.9077	348.2719	43.49136	51.61392	47.17555	47.95009	46.50591	50.39585	60.42033
ATP1A4	58.76476	50.25586	40.93771	71.11923	39.75389	54.77516	44.0948	39.6225	42.84986
SELI	1141.797	96.28502	71.26181	575.8013	250.5273	327.0083	179.5741	65.61199	218.876
MON1A	55.03407	560.9189	51.56894	131.3696	63.68686	74.92882	120.5446	58.52603	157.2018
SYT6	62.26088	873.5579	47.30721	37.39677	43.95967	50.12218	33.56592	59.98549	3085.825
HBQ1	59.32586	61.18804	37.88486	46.39769	38.98323	61.96988	45.1918	39.68364	52.21112
PYGL	703.9684	567.8953	472.0363	1664.651	513.0995	433.542	796.9772	749.3762	600.9586
KCNAB1	274.2574	184.1575	95.53342	247.8822	108.4298	170.1656	95.17548	122.0861	492.7591
STRN4	86.52379	426.0422	2093.138	1690.46	1812.284	2352.081	2002.089	2074.107	939.9338
NHEJ1	60.81721	63.34539	48.73364	75.47038	165.6727	58.26394	236.1732	438.8681	48.34084
ATP7B	345.7609	190.8672	144.6887	195.6076	230.1203	119.54	158.2303	319.5014	343.678
RAB43	117.0787	1244.321	80.75473	110.0962	192.4798	70.80431	78.92026	101.8909	135.7284
CYB5D2	72.08209	288.0695	429.1261	489.4292	623.1572	725.4854	441.856	532.9861	64.92072
XRCC3	173.0765	466.8842	235.4978	164.2534	437.3936	79.96741	431.2387	598.8328	227.4563
ZNF653	8286.446	7508.719	5902.459	7091.727	4853.138	6854.486	4802.899	7379.223	9950.094
SIM1	51.20329	33.98062	42.29189	53.06137	32.31669	49.1127	39.28735	43.36974	63.30841
SNX6	133.5797	49.4311	42.68552	46.81693	36.4313	38.16793	42.33706	54.77632	57.23109
LY6G6D	49.55139	39.9516	31.88709	32.41692	42.4984	34.03145	139.9632	47.79761	46.16598
IMPAD1	74.97539	36.16595	35.86703	247.6515	94.0268	102.8089	66.04345	91.87838	41.50513
CACNA2D4	44.42337	64.60507	38.22124	63.59558	53.96159	44.43657	437.5902	40.18658	1880.766

GUF1	2365.618	1488.299	2452.636	2633.256	1414.157	1922.336	1713.007	2210.356	1284.543
ZIC2	78.54588	94.14391	50.50755	985.9293	127.4337	374.2367	125.3049	49.39448	60.08675
OR5M3	181.6983	192.5279	86.93846	81.29041	78.80413	136.7095	77.20839	82.45844	397.6531
CTSB	53.21373	40.25705	73.33804	37.79012	47.1242	42.4161	52.77548	71.23536	106.9679
TTC31	34.36845	35.05475	157.3263	43.5791	507.9194	297.8508	448.6907	572.2324	44.25613
GHRL	1216.244	649.1096	1052.804	69.95587	166.6432	1430.996	1354.154	1160.004	55.10575
POP4	32.22817	42.61687	70.6611	44.71096	174.3697	37.04296	166.0785	93.45939	37.77923
SPDYA	396.1061	215.5566	160.6063	219.0164	222.6704	226.5265	118.8514	201.4449	453.2541
IL6R	59.44757	44.69816	312.6374	58.54072	41.56755	41.9428	43.75922	43.76952	1785.623
SFRS2	125.2367	427.411	645.6433	363.3058	1114.87	611.0433	823.092	487.1605	745.1068
STOM	847.1955	330.465	494.9378	671.7031	425.6527	1063.707	389.0291	516.3389	299.4149
IDH1	277.4411	84.39973	73.92912	103.3788	60.1784	160.3875	58.02994	63.64672	177.8743
LAMA1	41.26054	48.18729	44.20491	46.24714	35.97989	43.33374	140.2642	41.13765	101.3005
KLK1	843.4323	315.05	150.9656	311.4886	270.2148	621.679	81.44344	216.0001	857.379
SPI1	3941.007	3238.9	3256.054	3251.035	776.4547	4030.048	1673.596	1940.008	4571.833
ROPN1L	78.86045	57.06075	67.55537	69.5547	64.63353	70.70854	55.63488	45.53847	147.2055
FGF23	212.7098	129.7654	79.7739	146.2238	74.17793	85.80081	63.13245	66.38161	214.7162
CMTM6	302.749	491.5526	177.6533	77.14303	154.121	108.8239	221.5596	184.8956	308.8897
JRKL	117.7507	127.4009	172.8012	100.5235	137.8673	72.0779	286.4711	358.0824	136.9515
AHSP	649.1224	1238.629	958.2199	202.1618	311.4135	1609.604	354.8385	1255.742	482.079
EGFR	49.35343	46.94111	489.4883	1381.042	1342.675	1972.83	1009.21	644.3524	211.301
TAS2R13	30.11759	33.7128	34.97556	230.19	37.56218	37.98569	50.29447	48.601	37.70738
RBBP5	33.90188	33.95561	43.58998	49.99264	43.69215	30.78561	36.40755	34.76543	32.48246
DDIT4L	366.8871	3846.577	3633.235	216.4491	1592.175	4575.143	5564.371	6128.772	263.7082
FZD1	45.24978	66.44299	61.48671	2283.904	513.5366	69.82377	529.0426	558.2086	155.5291
KIAA0133	438.2787	251.2941	176.844	271.807	711.0478	421.6821	437.1715	120.0771	560.2343
LY6G6E	40.55793	46.68716	39.89211	47.91153	34.33855	40.05353	38.97759	39.59675	57.64675
ATP2C1	177.9999	60.17316	415.3135	513.9485	503.92	253.7221	601.0655	931.194	43.38705
ZNF169	35.69935	43.75517	128.5695	43.89993	39.87109	39.93798	113.8307	40.50809	68.14489
ORM1	204.916	49.32785	41.95688	35.54631	51.82584	41.16764	49.58362	46.31674	120.2409
FERMT1	39.53333	38.05955	32.90773	554.2105	320.0194	36.21374	455.4437	324.6303	38.11122
LRP1B	49.52273	41.86494	54.45461	54.30548	45.42747	305.7781	39.85423	42.02802	51.77291
CHST4	107.1358	51.33464	38.47498	44.50568	155.7975	54.6868	46.94955	57.43413	55.9278
RB1	45.11722	47.28385	173.5353	38.63482	194.9546	309.01	271.5016	43.33935	54.32544
C3orf37	606.3761	961.5575	234.2302	897.4978	305.4043	309.4726	196.1389	351.0309	781.3156
LOC44117{	1327.848	67.92619	411.1765	34.13652	117.3591	222.4035	79.03783	59.76594	903.6166
VARS	3185.728	1161.289	4220.739	3235.331	3624.633	3237.855	3877.251	4023.728	1432.275
CA8	45.81031	437.9099	499.4337	42.74628	871.5137	284.8291	178.8993	682.2604	1319.564
SLC38A6	101.4865	313.7903	252.0106	295.8295	113.4813	238.1484	215.359	110.0623	78.43425
TLN1	9436.12	7942.945	8062.272	7863.469	6127.208	10058.98	5815.838	4387.015	7791.518
IFIT5	201.7291	123.9882	108.2826	153.6603	174.3055	165.6408	92.42258	143.094	231.1106
THSD1	88.55998	50.63358	38.95726	58.33014	42.21894	37.87443	111.5886	39.39438	48.50679
CES2	10852.38	8655.799	9377.43	10847.97	12412.8	10558.37	14075.9	7822.244	11681.66
ABLIM1	144.7719	88.20892	66.6517	84.18345	56.77732	99.1758	61.1439	73.35815	207.2294
THRB	114.4748	64.87684	80.74363	76.33317	93.52821	68.34387	61.41153	51.72795	77.67647
FAM90A1	36.99952	38.54273	35.48145	47.96239	190.2758	46.46314	38.19421	34.81432	44.55109
ZNF280C	242.5645	147.1389	119.9979	174.7632	84.10091	83.68342	69.00335	84.35005	322.4478

FAM184A	209.462	581.7395	83.62674	103.3329	449.8746	90.08157	111.579	250.1673	440.3513
NEO1	1609.665	1500.319	1471.619	1681.817	1192.827	2231.622	1206.096	2371.517	1533.096
TLE4	50.25969	46.22416	133.4533	49.29662	54.73275	58.76797	72.66679	61.92718	66.09521
C14orf166	743.9059	33.15217	1015.118	578.2675	1278.335	903.5022	1468.237	709.9158	34.31066
POLA2	8485.778	9927.211	9425.046	9877.387	8184.361	9512.42	12062.33	11751.05	9538.558
LMNA	6804.936	3909.302	2013.646	5865.401	1508.147	3292.648	2325.009	2753.534	5850.688
TTC14	5662.695	6469.842	6185.887	4384.556	5792.658	5836.695	5679.352	6007.276	4949.131
SERP2	54.20598	40.29689	861.8117	36.38425	875.6666	223.4246	1277.329	781.2187	37.68986
CTNND1	62.11362	294.4507	221.8889	312.6814	213.1316	144.6157	248.3757	141.3286	194.4245
GPR182	291.7724	180.1805	98.36825	211.5757	81.29594	145.6629	73.66676	111.6818	449.1619
SEPSECS	44.02363	46.82323	32.50452	36.83909	138.1213	325.2542	35.6378	37.18441	35.88813
C11orf47	1583.642	173.025	95.49594	158.5915	206.628	112.7647	75.14273	96.78292	345.8627
PARS2	238.5705	183.4165	77.50767	122.3435	64.77941	97.59928	69.96642	53.49541	359.0564
RGS14	739.3458	511.4838	137.1478	383.7315	93.40041	38.32974	32.16643	41.24172	997.8112
AMPD2	39.0537	64.40035	34.67244	43.78467	50.0375	40.21395	35.53626	36.39883	33.91212
TGFBRAP1	142.4977	50.84073	293.719	489.2017	261.2772	61.53767	224.3988	227.1156	37.9694
THOC2	780.2687	658.5605	538.4631	578.6456	396.7176	460.9446	424.2082	477.6758	779.9922
LATS1	63.14025	425.1342	996.5525	1283.928	308.834	767.2638	581.591	615.8311	1130.091
OR4K1	60.80451	100.544	49.66256	106.2487	45.85643	60.59911	51.36602	43.9148	194.5759
NLRP3	282.0212	139.4571	95.1423	195.2753	68.64018	94.0815	73.54285	108.2419	389.8332
RBM39	122.6978	312.1102	789.9442	413.9464	727.5383	773.3583	562.5903	462.1805	116.5682
PDZD8	2107.159	2766.947	2429.785	4011.912	2319.238	2234.834	1721.117	2083.025	2780.938
ANG	422.6382	221.6384	132.8913	264.4646	115.4355	165.2368	151.1333	140.288	581.9972
DYRK1B	63.1631	78.29757	49.59121	59.56166	48.87222	47.73302	40.91996	56.74458	50.40162
LRRN4CL	36.90186	868.256	452.4036	716.9446	355.1867	607.3569	670.6348	531.163	3015.765
CSF3	53.11604	70.51303	43.43312	67.07339	51.08825	44.17144	111.2304	50.57534	45.04473
GAL3ST3	49.9446	58.55902	51.01173	63.06984	51.81259	56.82167	55.45285	39.60413	140.2166
ARMC5	1310.997	984.7137	1474.23	598.4548	1360.417	461.889	913.6871	2118.57	1569.309
TBP	40.80626	1619.918	2270.185	1555.464	1669.078	451.6428	2519.561	1102.8	35.67997
ARMC6	509.1293	110.6978	94.90556	263.4812	56.27917	86.58297	89.49014	63.46277	319.4839
TAS1R2	43.32788	42.68734	46.90677	40.93189	39.9057	53.54877	43.15852	54.00598	40.38847
ZDHHC17	62.22341	83.01727	455.0941	63.09591	405.2637	58.84613	302.107	229.6451	63.32883
TTC7A	130.1862	78.13139	56.06331	122.478	49.38759	61.22858	58.07051	58.54729	249.4333
C9orf78	1088.734	1626.73	2157.23	772.2063	2655.267	3286	2833.302	2889.061	1202.032
PRPF4B	39.03316	40.338	42.66735	60.12528	44.8124	69.63506	58.70171	46.89235	100.4116
GFAP	92.2782	36.92138	49.91984	69.90028	48.61033	68.50654	44.27041	43.21587	57.12104
PNCK	1644.263	762.7089	68.78352	5301.589	538.2808	1755.962	203.3196	548.781	476.9667
MON2	717.9899	1456.202	1304.939	527.7302	739.9686	922.4734	841.361	1070.495	873.4047
MED22	3648.254	3387.249	2351.762	3530.463	1946.751	3482.99	2081.649	2234.197	3782.046
NT5E	714.4505	329.9337	368.239	2292.055	126.324	56.16504	309.004	98.45716	915.4714
GPR56	242.6781	180.5529	86.54647	200.9479	80.39853	110.9932	75.19077	107.4701	68.64114
CNBD1	31.5946	37.77061	36.19313	41.89909	35.28623	33.06212	36.04152	32.79755	47.87616
EMILIN2	691.1827	69.44037	330.4057	68.30285	400.1205	761.3568	988.1786	830.665	34.56973
ST8SIA3	272.695	161.6985	81.47317	135.0471	68.86134	122.245	66.16409	94.74812	450.2181
NCF1	44.95111	74.74061	37.67349	58.02404	107.4665	228.8039	37.28714	35.10406	37.66931
GNAT1	295.7001	693.1806	470.8848	1015.469	796.0472	963.0079	196.0475	271.3308	579.7695
DYNLRB2	2794.451	1997.338	1229.67	767.42	1192.936	1096.525	724.9056	1030.762	930.2883

SPATA9	70.34721	36.82637	72.60143	48.00013	53.38149	37.00652	46.20522	50.14585	41.07572
SRPK3	40.34351	839.9847	37.37728	64.07217	36.98199	47.40754	279.457	53.31668	44.67398
HOXC11	98.71692	64.00887	50.67598	113.9857	68.53178	55.07426	56.50159	46.28591	62.76304
WBSCR22	1626.204	44.96541	895.1381	45.96077	2018.213	48.75665	1305.328	2215.029	2189.911
OR4F6	86.84573	57.55025	33.88782	48.8732	46.32274	46.35081	42.08185	46.61694	76.31384
C20orf27	4471.995	4128.696	2979.145	6043.956	2040.152	4729.425	2204.262	4035.324	6354.878
LOC38891(	38.42508	31.62406	37.70553	35.94941	35.15593	48.68092	49.49895	34.10079	44.04668
TNFRSF14	23474.74	26154.71	25164.63	18904.45	19931.64	21714.62	20960.1	22633.4	23648.19
RHBG	75.78349	78.58869	36.66162	44.08332	44.33847	40.72758	75.99738	67.77937	49.65995
RBM9	1584.146	1031.522	530.9571	1768.953	386.2536	231.1436	387.4245	425.9916	1239.966
TINF2	384.1377	38.11268	44.52288	37.46096	32.497	35.31828	52.27629	33.21637	40.46842
CYP46A1	138.2908	811.8008	202.494	65.0837	251.9458	444.8096	212.9522	39.97523	61.71465
PRPF4	40.07301	459.9407	646.459	39.08089	852.6319	285.2185	1160.688	888.3986	1356.813
IL1RL1	43.39509	38.06425	233.4893	59.23297	146.3364	439.5171	262.5673	119.1295	63.98802
TXNIP	4412.884	5915.588	9305.283	4180.377	10490.28	9994.701	12797.14	9954.28	70.04641
REEP6	35.51421	41.61364	197.9112	34.52448	359.7231	34.18555	171.101	38.19796	53.13073
OR5AY1	208.4161	147.7218	64.6162	175.2749	55.59883	73.71393	67.29411	69.25352	218.413
SFRS6	14381.26	13641.44	14152.29	13787.81	13419.46	15488.99	12334.78	12617.06	15197.81
TM2D3	1832.818	1221.694	180.9289	201.4038	161.0661	175.0894	375.0244	885.9436	496.7694
FLJ14213	93.51108	39.09714	1986.861	1574.799	2608.765	2661.381	2105.637	1697.91	44.00001
HLA-DRB5	63.91956	65.95528	4257.595	47.06543	45.77138	35.92017	45.47161	35.90034	112.9653
DHX29	73.45856	108.8048	238.9171	322.9576	139.4437	456.5271	84.78262	50.25661	49.21658
ACSS2	70.07811	72.77303	58.97792	69.84608	54.68849	72.91893	57.88797	53.01256	45.2635
SLC32A1	60.63287	40.90668	37.60628	37.35812	32.88636	49.8148	50.99379	48.61882	67.24468
RNF10	43.68884	315.3514	258.4342	40.69747	127.5793	34.98468	178.6731	110.8733	89.02131
SLC25A29	119.6866	98.48064	188.7206	67.53981	337.4961	94.98141	259.0585	98.2663	115.3962
BRCC3	70.32203	38.43959	41.55739	47.8092	68.34277	49.95092	63.09414	30.70886	41.47863
LPHN2	454.6168	230.5258	161.7755	34.71221	214.9599	218.8307	139.5944	94.49635	578.1077
ACYP2	147.3929	159.7904	84.45598	195.7572	70.25709	113.7701	73.72701	95.02681	305.7447
SASH3	70.19808	64.40856	604.144	70.28693	469.2921	428.0986	810.57	624.0211	36.85226
G6PD	1719.695	828.2404	284.728	44.49319	102.7684	48.51363	389.3545	68.30307	43.54899
FBXL16	60.34606	68.2545	49.19736	55.61121	52.85591	65.85444	48.56503	62.56203	93.1505
TPM3	5994.042	5827.633	3324.574	6509.617	2577.09	4885.215	5117.377	7368.491	5717.542
ACTR6	2115.39	2650.78	6664.196	3984.155	6785.142	6641.038	7521.37	6028.266	57.40553
ANKRD28	46.14355	91.97942	132.7314	69.85608	85.51104	135.1494	93.80408	90.6972	59.03279
KIAA0494	69.45722	198.8032	63.39922	75.87405	89.18463	148.0819	104.8417	84.21601	56.31776
NHP2L1	37.92268	52.84552	36.02195	38.52459	32.97131	33.90919	32.28034	33.46496	48.85254
KCNJ4	221.9565	139.4963	51.49331	65.6724	55.53374	99.98684	68.18758	58.79125	106.9728
PLA2G4B	20557.34	23104.47	19519.12	18829.2	22134.64	20952.59	15995.73	22721.74	23162.96
FLJ35894	40.00735	43.75181	40.15057	49.39774	40.72712	43.19867	47.96648	49.99203	59.56708
OGT	1771.169	1771.249	2525.092	2068.627	2939.521	3416.068	2796.987	1686.147	1316.082
BCAP29	43.11475	40.28058	86.94439	2395.476	351.1015	739.2617	255.5088	79.10647	51.50845
PLA2G6	396.2186	243.2916	126.7414	258.817	134.2772	192.7296	113.306	192.6774	448.5417
NRARP	71.14413	47.68528	228.754	804.4497	192.9023	490.6974	313.2224	842.1651	38.264
SRRM1	2428.487	3417.417	4151.331	4114.184	3682.344	4376.355	4070.186	3400.363	2133.724
FLJ42280	48.57776	39.66592	37.19121	41.79695	158.1121	75.0499	50.94805	40.41562	66.56034
ATP6AP1	7143.811	7477.055	5874.342	6641.174	4087.482	5846.311	4702.808	5728.973	7608.337

PLEKHJ1	226.9247	99.33649	88.85	69.41197	73.45275	78.34715	57.34832	76.57281	151.0088
HPDL	2072.916	211.7792	54.00645	1079.773	738.6829	914.1998	521.9522	303.5656	58.89454
KRTAP12-2	73.647	102.5925	41.5364	59.61829	44.94254	66.48864	56.4653	63.77131	106.358
IL1F7	42.07641	38.2974	32.14127	33.76606	42.1977	41.41176	36.62614	93.44805	52.63077
MAP2K6	32.84039	38.76955	52.89253	38.24054	62.50865	50.37326	49.82733	86.59545	34.18699
CST6	39.41165	39.14816	562.0508	49.12478	907.1541	199.5934	1067.042	1070.75	120.7296
EWSR1	3237.534	5116.055	5596.171	5133.018	6800.626	5166.595	6469.45	6310.522	5797.677
EXOSC2	84.37766	63.48288	1144.923	1335.064	831.9437	1730.007	670.6573	1718.266	45.62035
C3orf38	104.991	76.61124	194.7626	47.52207	139.177	81.81426	104.0056	161.7271	69.49197
GINS1	369.2561	140.5287	84.928	196.9093	72.90229	76.41334	51.42804	78.48521	74.84171
OR51L1	56.43719	39.22029	48.62496	54.03435	46.34204	65.06627	36.47101	50.39523	109.6082
C3orf67	217.6449	148.9471	76.28945	199.0608	78.4728	106.3165	67.2329	67.77183	308.7832
TSTA3	94.19019	105.5409	1077.593	118.502	395.3743	85.47588	444.1686	361.1109	182.9072
SYNJ2BP	4261.36	4280.548	4366.37	5149.763	3575.849	3919.771	4232.347	3267.091	767.1125
C16orf86	73.82019	101.5932	61.71516	75.61165	51.17606	89.62817	55.87837	58.98839	128.6483
NEGR1	33.30555	222.1824	44.93252	60.44827	43.50698	42.83007	56.14867	41.08725	35.57466
TSPAN11	75.11796	76.68004	60.77447	54.22816	65.321	89.59793	52.96674	52.55807	267.288
HLXB9	128.6282	63.8298	60.18112	93.00459	65.20591	97.48633	53.83394	59.85471	123.1124
PANX1	567.5032	345.8868	267.5982	388.1233	178.4748	262.5673	236.5012	330.1302	1586.232
HINT2	1588.247	2406.577	1831.245	1737.429	1778.818	1466.291	2993.411	1589.346	1925.508
ATP2A3	459.6891	320.9058	248.5119	294.4453	242.3479	248.286	199.6337	304.1459	589.1511
MS4A1	141.2927	63.25801	108.6701	42.43096	97.89554	156.1043	76.02329	118.248	70.96627
GNRH2	35.50808	36.92091	37.42683	39.75702	36.65757	41.64741	34.56039	31.25402	39.91665
CXorf38	61.71157	44.32007	236.7365	759.988	904.9273	479.9504	894.2598	492.0717	58.99944
C21orf121	43.06211	45.13501	258.7189	43.87829	33.61883	203.2793	34.95022	234.8885	52.22158
PLD2	2417.868	864.7867	1302.586	527.9467	1654.417	1286.281	1337.828	1616.869	66.63124
WBP4	45.96103	59.26912	113.7988	33.42828	67.21365	91.11943	72.50694	47.47866	37.7243
GYLTL1B	161.8209	105.7567	81.19312	120.2247	64.58231	105.6358	65.36282	75.36477	201.0989
APOB48R	494.0273	41.24316	137.0189	54.04516	64.00655	182.7358	120.941	47.36024	41.97175
XKRY	61.80851	45.72923	33.96601	59.2077	32.92606	72.25069	44.67095	45.10507	68.82917
OSBPL1A	193.1644	114.4424	47.7971	146.801	66.60989	123.0163	69.28241	80.74857	103.0125
C22orf9	411.9348	47.46274	39.37688	40.75346	57.66505	35.67654	42.77704	48.85685	44.83482
SLC35B4	3052.852	2480.818	1832.144	2383.777	1086.014	2661.84	1154.148	2620.055	4253.201
NSMCE1	1452.573	776.9584	982.1116	1432.41	1268.526	857.5486	1198.821	1395.504	332.4901
UGT1A4	148.5131	41.85275	45.23642	43.757	52.92826	40.63196	39.53227	41.11285	43.76881
TRIM38	662.7245	48.83878	1910.72	1924.366	1241.011	1338.28	1755.333	1213.39	1906.167
SNORD101	57.79269	37.40807	129.5961	270.5656	114.9096	84.73159	76.68612	61.26614	564.1584
FAM54A	259.3411	155.9194	147.6164	164.2741	79.68114	111.2503	392.5606	330.3507	264.3704
NCK1	84.73045	229.4038	279.5185	250.0179	574.5145	645.3583	283.029	279.7882	555.6296
TBX1	52.10565	76.24007	71.28912	262.8028	93.18011	159.3761	98.02382	144.9689	39.48774
LGMN	468.4376	3514.153	1865.985	2966.226	1936.14	917.0309	1699.248	2137.159	145.8183
DLC1	8004.819	9819.226	4766.944	5370.924	5030.68	2878.883	4523.514	6421.913	9865.712
FAM188B	48.63275	2269.045	1160.962	676.8473	741.4839	51.65847	1343.607	828.0827	49.27586
ZNF619	155.2757	48.63781	33.8082	75.27592	37.6597	52.50012	34.26384	35.20853	42.73445
PITRM1	682.1716	1153.608	528.5898	1084.082	541.8879	640.763	631.9371	716.9302	896.5463
C14orf21	259.9353	151.7856	85.63984	150.8527	193.3226	137.4069	228.817	204.4158	258.8942
DSCR8	159.4478	155.9517	61.53854	97.33938	56.0294	86.20118	55.67388	68.6071	174.2767

TCEA1	50.4018	1045.019	425.7997	1691.414	413.974	915.5725	324.2149	410.1525	1594.977
LRRC29	47.5059	46.19064	48.32676	60.38731	45.90849	63.41005	40.24915	38.88942	46.70442
CHD5	1062.227	574.4168	189.7977	51.32565	218.5569	325.762	266.0938	184.1746	56.54039
ANKRD6	3493.438	60.40293	125.4235	38.87926	408.0378	38.07914	36.6263	56.68328	37.88748
C21orf91	256.4621	173.0975	261.8083	175.6768	67.7965	144.809	204.4388	134.96	440.0371
INCENP	82.73387	60.76896	45.10551	49.66506	53.51332	88.19842	104.7208	42.0117	38.31409
TAGLN3	48.19734	493.3878	177.1578	759.3771	235.9538	1441.986	422.665	258.7291	57.02972
IFNA2	55.67236	45.99452	35.86418	64.78577	35.6902	46.67568	39.10527	51.09512	113.3048
SCML2	228.8752	169.6157	90.56725	186.7576	100.8857	240.7039	73.02631	100.9585	232.3462
AGPS	481.3468	940.3041	805.5592	998.9619	766.6932	702.2222	818.0717	628.8717	64.50059
RBM3	1592.152	1186.291	786.5357	1632.295	509.161	796.7887	664.1136	559.2958	1878.547
TRAF1	595.6422	465.3372	569.3401	368.7211	239.9602	554.2388	309.8865	245.6091	709.2811
MYO5A	1455.983	1459.993	1446.703	2240.763	1562.004	1607.805	1869.724	1576.91	693.3766
RRM2B	60.23227	96.84921	154.9037	530.4935	143.2066	51.67998	111.8735	137.9925	92.73959
RWDD2B	501.8842	289.8788	145.0668	301.9828	102.0588	293.1453	129.9929	149.0363	567.7106
TRIM17	250.8926	153.7673	91.56246	147.1487	73.41917	122.8357	57.07927	110.7649	317.4077
C1orf85	1351.19	4488.333	5099.841	3511.893	3445.933	4558.373	6069.767	4516.33	1380.606
FANCB	146.5883	1699.749	397.4236	1425.87	442.0497	654.5679	600.1048	806.2505	1297.08
DNAJC8	26961.27	27157.77	27895.91	29299.98	26996.71	30040.17	28490.89	27313.87	29796.98
TMEM100	424.9026	49.2836	109.3166	53.68813	74.14079	102.1302	69.26379	37.45603	746.5576
DBNL	20459.87	17614.19	18675.16	19755.81	15774.69	18533.09	19452.83	21366.72	19057.26
ABHD12B	96.82764	106.3481	58.5618	100.427	55.52768	68.92401	53.29144	65.94584	61.60017
CPM	76.2568	61.27836	52.88044	172.6776	67.75557	77.48741	50.50611	49.46679	122.8016
LGALS14	143.7508	103.8901	57.99038	91.47012	58.28404	73.54532	61.87026	63.77133	96.55899
AVPR2	487.0826	257.0083	145.9696	311.6512	142.7228	235.1526	154.2504	148.461	667.2797
NIT1	37.69343	63.09507	43.34256	51.2531	53.54512	74.38869	33.45306	38.07544	116.1477
C15orf53	37.96915	39.95229	264.5901	32.98627	208.5083	36.42185	67.34625	38.02813	2553.243
NHLRC3	649.1432	587.7649	511.1738	510.9049	280.7797	313.6892	300.921	166.1866	1415.34
CD84	1578.308	1427.758	853.707	854.6888	211.9337	826.1027	547.0161	500.9586	420.0147
NEK9	103.6721	50.83796	653.2	53.278	327.6832	57.37764	228.125	390.7999	45.14957
GNB5	35.36429	48.53923	36.11363	46.56395	45.40898	38.46027	68.18028	33.14535	41.94656
NFX1	64.5014	586.0892	324.0536	755.2614	58.13314	76.0021	183.5932	60.95179	1652.577
ERN1	1471.028	4268.143	4222.598	1914.5	4059.302	4422.381	3028.562	4996.231	95.69804
ORMDL1	216.9905	186.2535	318.4102	359.3761	173.3165	264.8034	311.6212	332.6128	120.1144
NCOR2	1578.422	1228.375	1265.342	1841.844	934.0427	1295.661	1063.534	1451.675	926.5121
MYST4	33.7196	273.5486	108.7203	53.29906	150.4886	182.9738	48.02639	123.7365	42.49317
PALM2	41.86795	33.74777	46.17508	35.08392	35.38472	43.08854	35.23514	32.97604	44.60034
SYAP1	38.96044	274.6714	632.6027	485.062	921.2852	682.9328	1003.379	828.481	59.43845
MLL3	59.66536	49.31398	72.67634	62.22679	57.78873	72.25027	45.24914	46.62952	71.36234
SNAPC2	5905.219	6045.084	8707.961	8029.959	8600.949	8580.944	8584.68	9670.147	5145.038
RHOV	120.2992	61.20014	167.1365	314.6325	218.397	207.154	118.3679	126.5009	76.05215
SDHD	43.83131	44.56888	107.6228	36.2542	138.9411	163.3804	126.7339	80.00748	63.86476
MBD3	32.3776	284.9847	199.069	42.73655	444.9195	62.23155	304.7472	302.5471	663.0652
ST8SIA6	63.42506	53.04314	51.07465	62.28475	39.02254	66.13263	41.31922	48.60872	82.66321
SORCS3	151.3003	103.342	60.71453	70.88155	55.13522	52.85335	57.12743	47.23734	61.88729
IDH3A	1858.53	712.7285	1009.545	51.43846	1019.014	54.6301	591.2412	325.2221	74.11953
HNF4A	136.7851	61.99673	55.84587	131.5984	66.0314	85.37303	65.15152	40.17946	170.3515

RCN1	41.91371	37.57964	36.40437	38.07855	39.7914	45.7046	38.23728	44.16323	34.11895
AACS	102.4848	118.1878	1604.339	69.68582	2007.539	1108.731	1374.001	785.2255	128.0907
KRTAP4-12	54.77365	97.05262	67.66453	84.82645	51.73732	63.76191	47.14101	47.02039	56.04162
TMEM43	321.5009	203.7316	93.05146	186.0827	98.4015	133.9321	90.90485	117.7207	439.7755
COG5	35.36442	44.36739	111.36	424.6937	57.17635	38.15567	55.07785	32.3299	47.44621
LIMK1	54.81292	50.8012	44.15035	45.51126	42.12751	41.95311	37.33479	52.18005	33.61604
NR1D2	41.99678	255.508	120.7009	297.6022	64.15802	202.6775	198.974	141.3162	54.26758
EFCAB7	428.1788	218.8329	111.0481	209.4805	99.03236	166.1692	74.20572	124.8196	530.6501
C4orf27	36.81648	472.4541	125.6825	51.8543	164.8199	34.46158	182.4218	258.0696	39.94489
ARL17P1	63.95563	682.4806	463.9795	452.0462	299.0559	2679.445	360.93	1424.67	1074.984
NID2	734.275	61.27254	464.0363	82.53558	510.8352	893.2727	1085.906	337.6001	62.44946
NHLRC2	50.7483	878.5477	574.8161	46.32068	300.6941	234.9991	337.9476	423.9931	1912.165
FRMD3	132.9383	167.537	72.39156	161.509	86.55229	104.6969	96.89919	183.3415	175.1315
SLC25A27	1466.91	1762.22	411.0363	595.3491	358.1067	106.3874	404.388	786.6569	372.6943
FGD4	114.171	124.868	81.90794	134.548	67.07782	75.22121	128.4848	99.72547	74.69846
SEC31B	48.45779	64.11443	39.4198	67.9365	46.46988	41.65694	127.7464	50.80293	2525.635
PALLD	53.35882	249.0575	556.4977	264.2159	1950.877	308.7799	1102.184	650.1173	70.24298
HSP90AB2I	87.52683	40.67638	53.72707	96.27627	37.60007	59.52645	34.87001	39.90877	43.23088
UNC45B	60.68692	54.60664	40.15833	48.90179	33.24031	50.11382	46.91108	38.63211	52.0471
CLRN1	427.1006	222.3348	125.3709	270.6941	113.257	191.3066	99.5966	94.4296	605.4864
NLRX1	45.5763	220.5505	537.6608	538.3507	578.465	312.5248	176.9812	664.416	133.3064
PRDM13	36.23513	36.89838	40.03929	41.05132	42.57035	40.24754	43.19455	34.29446	158.1197
B3GALT5	40.37938	47.92081	55.82034	68.96558	39.35767	40.712	35.79764	43.18677	44.69524
PYCARD	139.8522	89.91021	66.32749	84.83237	158.9413	72.16512	54.21112	83.42325	123.005
C5orf33	1760.732	565.9018	896.7723	1536.438	967.9814	1262.96	1116.99	583.4913	914.6928
ADCK1	228.6616	46.40295	41.6436	60.78518	36.77248	40.75167	38.55947	48.5101	54.54507
SLC8A2	62.01511	62.20562	50.15263	55.30627	919.4886	338.4535	48.14683	342.3717	74.79365
CHRNA2	174.6654	167.7892	83.9303	209.2884	85.31126	129.3208	70.16257	88.12524	252.0789
PTPRT	208.9012	148.9725	83.04006	165.3107	192.7504	100.6948	69.52624	81.8217	281.4669
OR5AS1	152.9245	96.36139	69.51769	75.38562	49.31408	60.88686	53.35888	68.36124	60.19397
NLRP9	41.08231	39.60984	49.15795	52.63765	41.94848	39.95578	44.30421	41.8868	119.8229
PDF	40.79305	38.45256	42.98692	53.22921	61.66002	525.5268	47.28152	54.35131	49.65717
CMTM7	61.44374	2072.391	3202.744	1718.548	1549.557	2947.688	3958.719	2792.014	1068.871
PWP2	133.5912	87.25664	170.4941	368.6992	225.1223	106.3735	189.0255	234.8363	309.2671
TNFRSF11A	721.7544	351.3123	308.286	244.9421	163.2941	410.4996	157.5307	371.7089	792.9133
BTN1A1	43.53354	54.85059	42.7928	48.84142	31.45957	43.58847	35.47237	46.67289	39.85255
ASXL2	542.6415	177.4472	169.6348	227.0462	273.431	183.2543	219.3041	138.2052	440.9886
DES	41.24454	418.3181	147.3243	38.48732	38.91027	558.8134	284.0056	358.9758	35.12256
SLC19A1	2596.785	1227.927	427.4497	619.1479	172.229	310.6887	106.3758	104.7002	2032.257
KCNJ11	34.49659	33.5028	43.19212	33.82233	36.19308	34.30688	43.92546	38.13646	40.39629
C21orf7	549.2484	819.3225	507.6972	1418.132	940.0801	500.3962	522.8218	652.236	1102.459
FLJ46010	46.69368	45.41957	37.88735	47.80988	40.64156	34.90811	94.69435	41.29046	34.7952
MAPKAPK5	659.9537	354.9341	186.9349	512.032	194.1008	214.8018	148.3014	323.0769	915.0114
C14orf118	219.3566	122.6295	62.61084	109.8867	69.7529	104.8431	75.93369	89.96827	283.8828
SOAT1	427.9509	56.50887	118.5467	219.6653	56.42111	55.98092	63.79445	75.38221	114.951
COPB1	1646.775	3612.672	3978.629	3261.331	3022.062	3708.115	2228.733	2760.323	1970.308
TMEM16B	172.2411	161.3022	82.96879	168.1423	92.89406	130.8174	104.2296	95.55614	360.615

CYP19A1	168.9615	78.08327	57.40639	128.2402	62.52254	51.50071	55.04605	68.76031	286.1399
ITK	117.1977	58.5494	1280.029	104.4972	381.5433	1408.394	914.3646	405.2867	183.3267
C5orf45	73.53216	316.4822	46.47718	90.93244	51.67997	45.01038	40.14286	115.6889	43.36217
AFG3L1	54.87132	1398.883	54.09101	51.77773	34.38897	44.35512	39.52562	61.7931	92.04929
C1orf146	41.62642	39.8633	43.32511	38.6959	45.26368	35.93972	39.53104	49.04673	75.27689
FHDC1	104.5263	99.39803	129.9329	419.8372	267.8635	74.95367	98.39466	123.1106	148.584
NAP1L1	447.0836	242.1057	112.5577	274.9301	146.3899	163.7786	126.9205	124.8821	536.9441
FLJ45721	41.23125	45.6435	40.22279	32.7756	34.08368	74.80145	34.01791	36.83924	45.85144
C1orf96	710.4546	2794.03	2796.756	3552.142	2610.661	2781.256	3186.541	2197.197	1336.723
UBR4	2904.635	2283.55	724.3937	2791.885	735.7634	137.7351	565.5992	597.7844	2327.797
MSMB	80.47184	42.95463	46.05969	68.99493	55.35658	43.07578	84.62857	67.25697	41.88173
ZNF649	74.7422	110.8671	336.372	106.7553	818.6865	50.59543	639.1938	358.7392	67.21199
TMTC1	118.1655	68.39684	200.3258	64.5847	156.2012	55.64044	41.42722	209.9025	165.2203
PLEKHH1	195.9037	108.0565	74.9641	124.9464	67.49543	133.0101	106.6499	57.92469	70.5709
TNFRSF10E	1818.55	2016.712	2213.051	2004.985	2377.62	2477.601	2237.086	2266.698	724.8654
C19orf75	87.28181	59.56096	36.32058	61.96976	43.61855	48.22074	38.03925	50.02332	59.33295
CNTN6	59.98947	88.58289	71.97225	78.00554	53.26348	69.2463	58.90988	74.9041	71.23167
LOC339524	38.47392	48.62619	51.40783	55.72558	61.4091	59.75267	49.39911	57.43175	63.02702
ZGPAT	75.02343	51.61516	54.49883	67.23564	48.02293	36.32731	49.90625	56.77587	108.4929
ZNF711	45.49306	37.69453	42.48097	37.09814	32.98158	54.19354	32.11114	36.81711	63.66774
EPHA2	3197.865	2254.409	1479.001	1025.145	1253.862	697.0546	538.39	835.7542	3732.676
SLC22A5	39.16503	37.79843	32.39101	33.88563	775.6481	492.3627	179.8553	193.1972	64.4157
IK	186.4898	133.3468	85.32082	51.8884	84.08655	70.40878	70.56113	86.55261	138.1175
SLC30A6	32.13807	37.61834	40.51985	34.26672	45.15053	42.34287	63.74159	43.73978	48.32985
PPP2R5D	867.9288	45.86577	142.0726	463.9659	30.38275	50.56386	39.69112	37.64001	51.49819
ANXA10	130.6707	77.25837	54.71393	88.04519	62.10869	66.79433	59.98882	59.69819	104.8443
ZNF146	442.2575	278.4846	164.012	266.1463	133.6401	185.1037	148.3832	131.5901	438.8959
ZBTB47	43.51409	368.5239	209.458	1340.364	1085.738	620.6622	1749.195	1029.044	38.52396
MGC35361	66.41051	129.8923	47.78229	66.37653	119.5782	68.9833	171.3181	58.05239	44.67308
ST3GAL3	4886.521	6877.171	4782.921	7458.611	3858.793	4728.093	4764.716	7651.87	5968.785
KIAA1967	291.4875	167.9477	116.4075	167.1071	63.93216	127.4345	70.45384	101.7409	405.6583
TUBB6	153.2427	317.819	1026.166	796.5584	1274.341	397.3828	1114.69	868.8149	58.08617
PHF21A	6023.331	7204.287	10549.36	7161.249	9506.316	8322.678	10072.15	8438.728	7105.35
PHF7	116.9776	36.09148	35.22585	64.95604	44.81858	34.17191	39.58967	44.96519	47.74213
SLC8A1	131.5838	1007.351	186.2365	141.0427	70.34012	627.5714	322.5748	92.61529	153.4267
C9orf135	55.26149	102.9804	46.30576	43.87122	40.55592	52.64752	44.278	37.48854	51.7157
PCSK7	336.3748	206.8858	1418.995	240.112	1596.874	645.5143	1492.083	1479.049	423.7223
PDZRN3	685.6877	55.73581	198.3897	250.7432	84.71413	154.4057	63.94353	52.21223	595.848
EFCAB1	38.22086	69.61974	44.46721	47.81398	685.2214	47.13396	780.7534	280.1929	2713.379
PYY2	48.21769	36.67292	41.29691	37.86259	101.5576	35.99837	41.19228	34.29667	50.79834
TPD52L2	7289.767	9724.613	11726.5	11783.4	10529.38	9789.762	9342.425	10797.64	9171.417
NRP1	489.7039	511.4751	487.8671	1122.891	403.8373	568.7236	387.4376	224.0509	1492.718
CDON	213.1848	85.64021	52.75441	116.7977	92.15826	88.13727	52.81168	120.736	67.52864
BHLHE22	49.43113	67.89059	40.67953	730.4546	37.67675	45.03289	314.6573	258.0866	36.05418
AP2M1	539.3235	320.5177	3071.462	340.7389	4476.096	261.0385	3233.805	6271.121	713.5732
FCRL3	91.25012	77.4223	44.32591	66.72193	63.11332	63.12091	48.64297	157.4857	125.683
MRPL12	38.46434	61.81214	52.41394	1151.045	75.68676	38.502	299.108	1142.463	2816.742

ASB3	1973.593	2565.655	1933.032	2113.431	1309.906	2086.493	693.6915	1102.388	4383.928
MMP25	165.7206	50.92998	49.24115	39.47659	30.05529	36.56683	36.73914	40.69651	67.38863
CCT6B	222.6127	201.5551	241.6899	176.8105	281.208	435.971	286.7214	501.3191	249.1925
FAM81A	595.2631	365.8885	212.9402	295.9538	154.8268	33.22942	189.7155	171.6499	35.08682
GATS	36.02433	1483.561	1111.498	36.45258	1699.107	1594.452	1120.39	1634.303	36.17191
RANBP3	133.1931	71.62845	69.02936	70.34937	76.45785	69.75179	66.98826	60.35244	194.7862
ACIN1	936.7484	441.7616	155.0539	492.9443	371.704	299.033	612.9402	95.19276	81.17093
APBB1	39.69204	34.30294	199.0568	35.96165	35.10639	39.67799	37.31583	43.04966	37.89361
IL6	53.39888	53.61825	59.71156	56.49507	39.29468	49.28551	208.2208	40.65501	221.4117
CLIC6	1363.363	979.4979	1696.441	641.533	2485.697	1529.554	1465.974	1510.317	97.22397
STRADA	51.48852	36.30428	351.6377	42.42614	41.35924	633.7025	181.9836	36.18372	66.37101
BLR1	63.58072	71.16921	42.47388	44.23086	39.03735	43.62611	40.14194	45.44398	40.15164
C14orf147	73.34571	56.25286	57.23262	41.99084	224.396	39.69149	283.1433	320.3881	40.72182
PBLD	532.2789	311.9952	138.1978	353.5385	115.077	215.347	115.0641	134.7197	691.7715
TNFRSF21	12679.06	9380.752	9539.869	11059.96	9659.77	10029.63	11274.24	7279.518	11589.44
ARCN1	65.25959	63.28281	136.0284	70.55521	424.6123	56.19122	246.1438	293.8293	92.48698
DEDD	60.71991	51.31886	163.6824	123.3032	341.9906	48.87241	305.9888	532.5449	54.80399
C2orf3	227.1064	126.8606	85.2295	139.5553	79.50777	110.2815	64.01321	99.73811	175.4439
BTD	288.7549	177.8643	101.8918	220.3936	102.8065	150.358	86.38509	121.6885	479.8305
FGF1	243.447	104.6497	66.88544	111.7293	79.26411	98.49808	63.9827	75.47382	102.8156
TRAP1	171.6137	128.8397	482.625	178.039	357.3748	311.4349	350.3118	168.082	260.7659
ABHD9	12141.48	7105.554	13185.19	9660.595	19007.65	12483.15	5116.866	11176.21	12766.93
PDE8A	196.8475	111.4962	110.0965	178.1919	68.84424	133.0714	65.48109	83.12065	632.863
CBLN4	82.393	40.34373	60.30729	64.88884	49.85016	65.89287	40.75605	55.81012	59.99157
RRAGD	361.7112	220.3208	374.9828	240.4524	238.1697	178.8763	226.0965	281.8669	376.1148
HRAS	205.4677	99.95049	57.56708	82.19147	57.02164	51.20921	40.54012	74.16972	50.67424
SP8	47.95576	65.36379	42.64455	83.17259	43.54527	42.27178	51.92107	57.33274	48.39865
CAPNS2	772.962	553.7515	2781.404	731.3054	5245.887	2473.743	3708.891	2503.16	504.7986
TLL1	101.9346	39.22731	56.48972	55.54129	89.58971	205.0958	57.4602	42.38786	63.91294
TNPO1	35.35508	37.70413	38.57361	57.10959	39.27621	45.35875	40.55689	39.56558	144.4498
CRTC1	35.86345	41.00322	223.5585	39.02951	33.98414	329.4335	31.49911	35.62899	54.3726
SCRN3	75.21889	60.27325	143.7759	60.48178	172.9702	242.2905	93.31849	47.18558	120.5126
CES8	4705.29	4155.342	4026.051	2495.44	3662.475	4084.943	2765.209	4657.341	3440.732
GRIN3B	86.66432	40.47608	373.0101	1217.385	199.4335	54.80387	111.9747	413.8202	44.00874
RALGDS	13967.86	10488.41	14968.85	9547.479	14557.17	10682.57	11809.38	12843.53	11080.41
GNPAT	2831.844	2240.468	1350.148	2268.203	805.1829	1228.242	1160.542	883.1133	3216.085
OR2M5	117.8035	75.95827	57.73158	114.7828	55.2099	77.48306	51.90771	50.68296	58.76963
ST14	1523.873	566.2519	614.9979	777.7232	648.892	479.825	332.1853	576.826	3833.52
C21orf29	48.52654	47.64378	57.23741	43.45647	45.28314	49.25895	44.87911	37.61967	42.39347
IL12RB1	37.13699	50.99399	42.05256	51.33287	43.54966	52.31774	39.32025	33.45832	49.10065
TGM5	46.90701	40.63777	322.8047	40.03065	1796.344	55.2669	510.8685	1743.112	128.7935
HCST	8352.596	8776.392	10390.26	7780.874	5570.909	8752.431	8015.843	11886.39	7826.728
FAM70A	189.6036	175.7625	81.79478	149.1633	68.81544	75.48693	63.76355	76.25378	120.6527
TSPAN18	221.545	338.5407	696.2259	446.0324	965.087	467.2129	627.8073	597.4929	110.1834
BNIPL	10755.92	7288.956	11162.08	6645.838	10736.01	10904.26	7114.208	9024.311	6303.403
LCK	454.7745	319.8966	956.452	264.5122	412.6907	665.5729	491.9238	564.0425	601.3276
TCTA	191.1206	118.2913	63.88416	94.74949	73.02564	94.67583	74.58759	73.19512	232.4577

UST	40.35999	48.94754	59.39792	36.73819	367.8195	51.76259	81.17355	54.38687	39.7512
C9orf156	197.2021	161.8425	292.9579	110.4914	425.0393	257.7843	512.7588	703.3945	132.7215
PRAME	207.523	95.04183	68.65115	149.1094	87.8018	98.81567	75.74454	70.22012	392.6435
C4orf6	95.16987	84.63292	70.48857	113.9202	71.92184	103.8176	69.49081	81.68678	80.96392
GRB14	48.80084	58.15778	43.09068	36.84604	34.19583	45.56537	41.53879	51.43432	52.80888
TLN2	1608.881	1938.436	1921.385	3643.927	826.7338	1770.654	1001.339	849.4387	2381.73
SAP130	53.13863	59.11277	127.3431	91.7056	942.9609	683.8454	444.0053	613.4208	213.8616
ALAS1	52.23319	48.02048	48.12162	35.78485	53.87993	35.03231	36.54849	38.32298	49.52994
SNORA62	937.4597	601.3055	313.6673	474.0308	448.0061	267.6356	360.3282	365.3265	363.7922
FST	41.47604	61.57196	1342.101	2148.161	1336.568	47.56994	3984.037	1829.977	32.63218
DOK1	351.1963	170.3788	104.9145	219.0296	103.9501	154.7201	89.4366	103.0074	200.7537
PTOV1	12098.12	12881.1	9401.882	12009.91	10013.02	10219.83	10445.43	11176.48	12988.25
ITGA10	2111.703	1254.086	3440.054	1206.543	2879.058	1540.996	2916.158	1730.42	38.90858
VPREB3	108.4022	77.26089	54.01334	119.8537	50.01076	57.60156	53.44672	74.11255	83.47355
LARS2	79.14964	35.11276	48.8606	46.28216	101.3751	39.6382	138.8368	110.9253	59.92209
C15orf44	401.2448	271.9814	102.2431	313.2494	105.5259	145.2888	93.93121	138.5998	594.7587
APLNR	1645.763	5175.033	3776.514	2254.787	3634.968	2562.039	7610.89	5171.182	5739.523
KCNH5	50.0312	51.99937	56.72467	75.07766	52.81636	50.94805	49.56964	50.20995	46.55846
TMEM135	49.38445	39.1699	1182.957	550.5347	765.3458	1510.308	1214.466	878.0659	41.38539
KIAA1984	6185.255	5719.553	3420.983	2347.008	2260.959	2800.191	1793.279	2075.38	8559.129
FBXO30	266.7519	209.6012	115.1044	316.9823	97.28391	171.897	190.0113	182.7766	151.0054
ZNF500	211.1811	118.4071	92.32569	97.25309	146.8629	73.3059	67.17862	53.98345	265.5685
MMD2	54.44893	31.81213	41.9707	50.14444	32.81739	48.97869	32.90235	54.56954	50.47576
WDR86	550.5693	300.2165	143.4138	265.9915	111.5607	245.9488	109.0036	107.7737	613.1347
C4orf32	411.2563	253.9957	178.2982	282.7456	152.8885	258.3677	126.7542	170.003	726.0256
QPRT	8610.137	13147.08	12489.49	15348.01	5732.413	10523.54	6371.527	6787.919	13466.13
C14orf100	45.66079	35.74269	44.84811	48.90973	43.51007	41.09321	58.52516	37.4394	37.9483
OCIAD2	805.2717	901.686	1917.614	2482.19	2551.779	2639.696	3808.311	3135.554	476.9638
SDCBP2	1239.174	372.5813	304.4203	35.92777	240.52	331.127	103.672	43.44522	152.3504
SCN3B	39.97993	55.75021	51.43999	45.17486	251.591	67.2282	102.1278	61.52592	81.62195
TROAP	104.9399	197.7208	552.6066	770.3005	508.4549	532.5083	801.2239	556.3467	46.13309
RNF41	32.94273	44.82998	34.74342	67.44495	46.369	39.00602	33.63198	35.9772	49.91392
ADCK4	150.7833	1464.294	40.81574	54.41352	503.6042	61.42115	348.3857	209.1225	42.57507
TRAF6	114.5945	507.6003	113.3436	326.0932	112.5082	61.92738	69.04804	105.6983	62.45089
ZWILCH	64.33472	440.9465	261.0736	44.37947	104.2137	269.3508	169.6459	137.5679	94.26478
SLC26A1	38.90629	46.92825	43.15575	48.81284	42.64801	39.54185	48.81846	42.77069	54.30214
ATP6V0A2	1378.694	543.2269	2905.123	805.7597	2819.772	2390.6	2884.231	2283.615	47.27319
KIAA1522	1928.876	1045.286	389.2875	1596.571	442.1231	641.3533	290.4576	876.115	2243.941
TRIM50	57.15405	36.61306	39.30455	71.08803	39.704	43.80774	126.3455	39.16394	103.0435
SLC22A14	45.8725	61.00499	151.3112	63.13647	41.77684	58.9963	246.9937	42.12413	34.53998
HSPC171	173.383	88.39593	142.8131	161.5921	314.0067	206.0352	386.8385	250.9854	287.6103
HLA-DOB	85.91483	806.7995	277.5999	106.9855	53.65939	751.8618	526.9948	305.1563	128.6535
ITPKB	214.9254	159.7742	660.2912	184.3977	498.5605	539.7405	911.274	384.1306	362.1907
AARSD1	478.3381	773.2454	374.8404	300.8541	483.0747	571.5035	353.4326	859.5286	566.3787
TOP3A	255.3686	162.6776	90.67812	197.8157	195.4319	93.92506	419.8156	76.51395	241.0287
CLASP1	78.91434	52.95704	49.93581	45.46976	53.10654	296.605	131.4733	52.41566	72.04268
HMGN4	91.58401	65.33219	521.2557	355.6526	1200.244	1013.222	1078.69	543.804	104.5503

OR51I2	52.73164	48.24264	48.39967	43.30745	49.51537	49.74948	52.05263	65.62898	69.75085
ELOVL6	70.31972	32.73476	39.40426	38.32433	51.10434	41.64076	59.90029	38.00277	75.43105
CHST9	36.26031	35.04359	98.63937	32.70592	99.89278	190.9565	64.48707	38.20586	35.88605
ZNF552	80.23201	268.6816	110.7889	88.03766	57.88004	187.4814	94.68809	94.8847	112.8724
ZNF750	836.8607	1649.083	1354.278	610.5575	2266.803	783.642	1110.415	1336.223	114.8651
IER2	432.1132	220.1059	142.0423	247.7347	138.056	185.4491	134.6645	148.6967	470.8907
C7orf34	35.92892	39.16854	182.2743	45.72923	42.99881	42.87621	36.05309	35.86582	46.0832
FAM18B2	315.3427	154.1368	123.4841	236.5216	139.2169	153.3992	95.89745	112.4403	449.4403
RBM14	8091.341	7830.889	8315.365	7481.935	7958.114	8101.649	8519.191	7574.951	7829.101
RNF43	55.80793	744.4244	35.66306	39.01163	516.1899	63.55652	409.6481	485.4528	59.1217
CDK10	7370.327	5069.811	2337.377	6151.344	2164.371	2914.335	1595.097	3774.995	9223.104
DDX24	119.8626	69.98772	1730.811	772.6422	3309.67	912.1223	2868.749	2411.458	75.65207
ABHD4	1371.769	129.2496	423.4117	213.4807	512.4459	483.4173	265.148	606.3252	3209.78
CHAD	61.52598	64.02211	67.54488	72.44704	47.12221	50.28195	87.2701	56.94849	90.5606
RNF135	237.3504	130.818	190.1989	128.9682	315.9591	157.3058	265.0478	133.0043	538.9211
ETV7	50.36642	62.17637	299.6054	322.1356	59.84959	247.4811	228.8567	369.5474	40.23988
CD99L2	1008.424	92.45988	98.95321	73.691	483.2945	94.96101	671.2853	893.4744	1353.758
VAT1	440.4867	192.3166	181.3427	274.6747	233.2987	175.6651	224.726	176.2076	522.3187
SLC44A1	1812.259	3582.292	5394.576	4075.447	9645.631	7977.776	8216.936	5908.364	2531.92
GADL1	52.36456	84.07349	48.09726	99.43849	55.26044	81.06067	53.35152	67.85163	138.9051
SOST	37.16628	213.783	38.87654	35.85603	36.30517	49.64889	40.61343	57.40944	35.47667
ST7OT1	82.75898	35.73803	45.19197	408.5892	41.61339	218.5402	44.63859	51.72289	43.05789
KRTCAP3	1314.034	2189.764	751.236	1567.766	870.61	98.00144	446.9266	654.9612	139.1563
KIAA1370	54.51337	72.51135	400.9409	46.45488	212.7898	188.3634	278.3137	184.6977	146.1418
RIMS1	125.1088	56.27898	49.25544	61.37873	47.17175	52.4306	112.6116	38.54692	85.0267
PBX4	68.6591	63.24249	205.4659	55.42619	45.4897	232.0075	125.6345	119.2979	57.7449
ZNF324B	472.5302	93.91012	338.3913	455.3498	284.7593	267.8012	287.4373	253.2507	252.7239
PSKH1	36.27192	32.70984	38.38396	40.7968	140.4444	37.70673	37.85294	37.63452	34.95794
PRUNE2	93.86063	1358.291	261.0015	852.2861	45.26718	395.0298	509.7258	1028.811	207.659
EHMT2	73.6066	60.28264	40.18247	54.77955	61.87026	69.64227	42.04798	60.08661	56.80793
KCNH2	271.9655	105.3322	68.26626	158.0289	94.81683	97.85171	65.58056	59.25458	340.2071
UBFD1	1293.829	595.5745	557.6214	764.5358	169.3246	567.6708	199.5623	488.9021	1956.2
DPP3	154.4058	374.3322	45.49849	720.9715	95.66388	70.13381	100.7443	51.92538	48.36584
SLC27A2	52.10813	70.90939	146.5941	88.10456	50.68539	56.00745	47.05174	49.21057	118.5008
SLK	51.2101	410.9647	498.3629	38.47151	347.9759	33.25706	394.7106	208.7083	40.67092
PMS1	44.90544	259.6118	221.185	297.2834	215.7303	392.9077	238.7029	308.6162	33.10922
TDRD5	46.50735	46.7313	36.23202	43.62574	38.38295	33.3271	40.98071	32.85757	38.60661
ENPP1	523.608	187.3854	108.5308	129.836	120.0334	108.5652	69.83625	88.82697	231.1108
PRR25	90.94014	51.81495	60.56235	95.55032	46.49404	71.48876	44.15574	66.02969	72.08131
SAP30	42.16115	368.3705	765.8041	818.0566	1347.278	1316.232	1242.745	299.2374	56.09169
BMI1	818.2712	1839.288	2220.888	1810.984	2710.583	2735.327	2456.94	1598.516	39.27526
ATG4C	395.098	200.9407	125.0946	235.2833	134.7928	165.5777	112.9985	99.17242	467.4359
HAS2	410.6643	224.2349	134.7581	177.051	94.46857	141.047	159.8205	88.54074	326.6221
TCTEX1D2	99.96041	60.63659	1355.308	73.03212	3332.005	1739.65	1668.266	2644.88	68.77662
C3orf59	407.7065	241.547	553.8005	259.3553	332.381	160.1813	397.3828	438.7357	553.5416
ENSA	3286.208	2237.357	1411.836	1817.042	1480.226	1950.11	1061.274	990.0245	1833.247
C6orf205	2150.435	37.27412	4168.759	34.95087	4363.898	2316.294	881.7006	46.07787	55.85735

ST7OT2	141.833	88.58595	64.82515	77.77277	57.10295	76.67092	56.15009	72.38612	76.74948
PRDM5	34.82364	35.74582	47.34792	44.07008	66.77399	39.97232	34.17743	49.60326	41.99189
KREMEN1	429.3939	1138.844	534.3737	1000.499	520.7548	678.5208	304.9164	547.7176	3321.002
HEXIM2	44.43106	1226.198	1708.428	1147.069	1649.184	1402.939	1955.344	2545.025	59.78154
PON3	3796.095	6080.646	2324.936	3236.977	4976.894	6383.064	3009.25	5656.834	5740.069
EFNA2	46.8698	35.0221	36.67809	39.07333	37.96822	34.49329	34.44556	61.47635	46.58167
SAA1	64.77599	262.6147	82.22894	60.07052	56.24752	85.98718	86.29003	52.39992	158.2719
EPHX1	203.2272	116.1884	93.78152	136.9677	151.3477	149.1743	81.19188	83.88223	57.47053
TUBG2	2316.948	5774.025	7112.633	6658.932	8566.027	9445.6	10291.82	9779.06	2369.469
RP5-1022P	45.00152	34.54515	1652.368	844.6152	2243.303	1970.079	2625.17	1593.535	1229.535
LOC44094	53.59527	41.06303	41.50575	41.20233	31.95511	38.59566	54.24556	33.53921	48.92447
GBP1	42.0792	252.0267	84.33154	279.0397	40.65649	48.13904	78.84404	32.36964	45.36036
BCL2	511.8052	261.4579	126.7128	221.0456	85.94667	151.8126	86.26569	110.8505	755.2416
CC2D1A	1025	426.7239	645.1628	192.9274	719.0314	306.5551	541.6815	873.1056	253.7746
C2orf49	603.3904	402.9322	312.2821	648.1563	412.1466	331.2134	311.3523	353.0915	612.3591
PTEN	1725.066	889.2711	603.8497	225.06	1140.237	285.4904	1326.793	309.8816	2243.541
HP1BP3	791.7092	965.5964	792.8987	1456.453	566.5296	468.2682	356.0782	451.0486	1161.804
BARX2	2445.978	431.1006	1491.608	1926.413	929.1289	678.3918	423.923	357.425	3420.388
KCNK6	9541.774	10104.29	11300.16	9465.786	12897.79	10491.33	11394.62	9573.609	9298.173
C2orf71	152.9869	79.39397	55.50094	111.485	50.77224	67.43272	54.5711	48.05405	98.20858
PLA2G7	661.5504	54.23989	373.4256	426.0516	90.44141	258.1192	177.6935	407.55	37.31292
VEGFC	98.49787	65.77049	64.72315	743.901	39.46455	41.14761	298.9757	41.58485	298.2182
PIP4K2B	1622.839	2775.854	2441.457	2446.241	1439.302	2197.917	1958.463	2599.076	3141.832
BANP	724.9489	185.3336	154.9363	165.2454	98.56169	234.9898	125.9487	177.3434	1053.438
PIWIL1	90.04925	100.8527	108.1094	81.93372	64.00819	93.98806	55.5728	61.16196	168.6329
SH2D1B	44.79256	48.87609	40.57187	74.60035	36.4286	40.03131	50.76155	48.02315	65.36475
C10orf2	33.10033	45.48986	397.0215	759.4619	1044.845	37.2887	525.696	156.0032	40.62541
ITFG2	262.9043	127.2954	158.2866	188.9938	76.05219	128.031	147.6637	636.7686	283.495
TREM2	258.8017	119.5554	114.95	184.1294	104.0076	80.4921	114.7267	191.2259	206.432
TSPYL6	334.8487	225.27	105.7582	237.9398	91.47099	172.5137	93.77303	85.57706	466.1955
ATP5C1	110.769	354.9841	1638.033	47.53146	404.3939	372.4741	467.7143	1038.281	44.58801
COX7A2	44.29356	657.1669	1753.926	692.4197	1340.62	1663.837	1251.468	1675.799	40.41417
COL1A1	11685.12	9262.828	13080.11	10303.76	10489.18	17673.81	21429.23	13168.42	8549.171
LOC39990(	218.5096	570.43	962.8095	277.7243	523.8239	163.8275	697.4695	621.6405	69.99988
KIAA0467	136.9305	128.3067	86.91669	176.1552	248.6954	110.6288	77.53982	119.5745	361.1476
UBE2M	16211.06	15940.51	12231.6	14944.49	10096.6	13377.84	9017.974	9891.691	15036.18
C9orf150	47.80882	235.492	39.80714	54.20796	40.64304	46.61711	46.72734	46.22935	93.93767
NUP35	33.7852	46.26221	56.87571	36.32875	112.4242	253.8698	145.8316	46.94501	33.98973
FBXW5	193.276	120.3705	164.3945	158.0038	196.4716	120.049	130.1804	194.3689	347.5048
MGC34821	57.81984	51.59097	43.83491	64.82349	55.36747	68.67658	43.24763	50.08458	51.5432
STRADB	766.9259	350.1794	530.8643	719.7498	577.0859	561.0789	938.558	526.7667	38.51327
TBX3	542.036	240.9037	82.12686	80.49514	87.61594	165.6462	52.14665	52.04958	639.4472
IKBIP	374.123	256.865	127.195	286.4478	161.5574	230.2385	189.1763	130.4089	520.1723
HIP1	94.39629	40.63562	40.76838	51.91374	39.38669	40.21578	44.11654	36.77743	36.18841
PIGQ	10739.37	9105.457	7616.413	9671.123	6787.059	9445.396	7771.309	8336.264	9684.641
RAP1B	2622.214	1720.846	1329.771	2414.768	1014.26	1976.825	1406.596	785.0022	1616.917
OR2L13	40.23551	76.58036	57.3508	69.3843	47.41084	54.337	49.13189	36.33928	42.84915

FBXO6	8493.654	11149.54	11973.68	10479.67	4216.568	9820.746	8960.735	9007.034	5457.833
TIMP3	71.71915	695.034	239.4649	1571.753	618.5235	1126.35	753.0253	185.8264	782.2311
ZNF776	243.6066	81.16304	53.59737	125.6575	89.46838	73.71549	62.94915	85.36362	214.2173
HGFAC	47.39968	47.32701	55.81104	58.81125	46.30867	40.00417	36.74797	61.84062	35.62115
POFUT2	418.45	202.611	398.9984	191.0714	118.9485	158.1332	118.4773	129.9725	480.3713
SYP	36.52055	54.27337	263.8724	32.22633	323.7614	368.8153	236.6903	404.4832	52.09726
TAOK2	104.5555	673.7935	1596.333	1473.185	1552.048	734.1373	1196.134	1821.576	233.8751
CD5L	54.20902	65.04552	70.08984	35.03335	47.58165	50.2494	45.38712	44.23962	86.78926
EHHADH	1102.032	595.1946	78.51218	135.9158	70.37268	341.5143	107.3425	327.8802	49.86126
KIAA0391	1529.754	1641.301	3270.381	3446.238	3595.737	3711.919	3242.555	3614.499	1184.23
KRTAP5-3	50.34353	40.57513	57.64786	51.91713	38.25623	36.57559	44.87548	41.88462	45.71679
ABCA6	38.61335	843.7932	286.2646	45.23398	266.8487	245.318	71.00882	101.3663	1350.697
WBSCR17	68.42957	68.81188	63.55042	61.39921	49.19558	68.37287	66.04543	44.56708	72.7344
MAP1A	46.98386	69.47497	155.8804	38.27678	170.3569	59.44381	486.8107	212.8578	39.89814
PKHD1	51.21608	98.16842	55.26641	45.35176	55.10851	58.93752	49.58052	42.26325	86.84604
UPK3B	127.3443	109.4651	74.71703	77.1031	70.46745	90.00074	52.13444	66.17042	279.1432
LAD1	42.40623	46.38071	572.2078	702.7813	1815.359	1027.265	531.8978	290.3318	2128.456
RPS20	981.2755	806.2715	661.4014	1944.465	409.8812	526.3267	509.6953	411.8163	1223.677
ALPL	5811.445	8298.529	7110.604	4208.125	7783.545	8702.695	10162.24	11908.85	4194.32
TBC1D8B	77.29151	48.22432	69.91782	93.9122	42.32969	40.4622	45.47377	50.46796	41.90046
RARRES3	37.53331	410.0292	801.2275	42.34211	489.7469	843.1986	838.0531	665.9194	64.68427
CDC73	125.8575	66.56034	73.97102	75.45231	48.86568	66.65524	52.63144	64.10194	182.1463
SCO2	2261.764	5184.229	8205.838	2711.993	4828.546	4941.936	6832.417	8083.915	1844.842
SULT4A1	65.99701	94.00153	66.5406	91.35254	48.36066	92.79219	46.51448	80.92451	102.7425
GDPD5	175.6478	1121.932	3114.72	62.61469	1749.27	1615.002	3197.979	2448.971	37.92165
PCDHGB6	364.3391	132.3088	102.3846	112.0397	144.5744	169.8148	85.95629	97.47181	509.4067
FLJ45537	63.0639	77.64338	68.87125	83.54971	58.54829	75.76253	57.82665	70.96767	84.00438
KLHL15	66.99521	39.98973	61.4188	45.96318	214.0783	44.87706	177.7379	41.91774	101.9993
ASCL1	50.38534	86.03021	58.90934	82.10267	57.35572	263.5465	51.69334	51.29962	46.06115
SLC24A6	4282.545	3966.015	6245.292	3992.244	6018.955	5920.671	7094.82	5340.681	4058.651
CYP7B1	1287.826	768.1729	708.6128	266.399	350.6246	579.8344	528.5806	645.6722	1050.488
SEC11C	63.62029	66.91297	356.6873	275.6201	844.9636	229.3222	524.9506	663.9357	47.07204
KLHL4	52.25647	90.72692	41.71365	57.46843	77.77364	39.79271	45.51007	46.67403	55.24885
BRD8	1364.893	50.67636	292.3767	45.01516	258.5338	641.8265	164.4968	334.5879	55.2054
C7orf20	41.94285	39.50664	263.6559	45.76296	184.1317	451.9545	125.1057	50.1131	35.46787
AKR1B1	481.5433	489.7139	179.2513	302.5791	661.2391	235.1211	259.595	360.541	615.8826
UQCRFS1	1502.399	724.3215	3438.989	724.0146	6181.188	2444.439	4794.293	6247.909	262.6645
PGLYRP2	73.75435	49.49763	46.40933	50.08229	52.71235	55.20313	53.32304	50.5466	78.60433
DCT	691.8405	381.1502	634.0773	87.42245	56.33035	219.1746	67.35738	112.3184	84.47875
CADM3	44.41841	43.97358	56.6343	77.25418	35.73515	38.26727	65.22898	36.83482	43.77296
RPL19	11754.54	11032.29	6871.289	11659.02	5868.847	7627.526	6711.471	5932.065	10571.49
KLHL2	424.8651	205.1615	90.18743	88.32601	85.87334	150.5396	67.29311	92.70313	55.54332
ZNF44	636.2315	264.6353	40.42551	41.41627	67.54808	40.36524	48.70908	35.26548	41.82618
GNG5	534.4919	251.5586	191.91	140.5749	134.8082	158.0646	125.0411	89.83173	54.16468
PPP1CC	51.28003	530.0424	1645.55	616.3098	1262.657	957.813	1630.974	1707.84	153.5637
FBXO22	44.97712	61.87026	207.2394	1254.358	37.05396	388.1773	242.7625	122.8542	175.8222
C9orf50	102.1694	40.73255	42.26698	43.98381	48.62757	45.57354	110.1289	167.611	46.42244

ZYX	5489.924	2474.393	1270.838	2434.767	769.338	3602.874	1017.902	1578.303	4244.174
AXL	122.7371	37.68218	164.0502	328.9526	141.1445	240.1911	366.3802	166.669	81.90863
LOC401137	47.88596	33.77992	40.35695	40.40889	45.79221	68.37439	40.4383	71.87103	53.2462
CD300C	43.55683	33.93312	697.174	48.11197	483.5914	848.0897	233.9074	200.5832	53.22124
C5orf20	299.1567	1274.953	3064.134	212.3827	676.2267	1970.846	2405.969	2436.197	328.7341
CD274	38.58609	498.4644	35.96777	34.29299	34.85147	42.72829	33.53763	35.98259	32.17823
KLHDC9	211.2775	88.07109	58.03716	60.32741	477.9933	84.46355	285.1149	48.60748	113.2364
SNCA	104.9325	51.86579	50.95115	68.37754	126.0781	329.9106	156.4154	248.37	299.5234
COL18A1	129.0728	75.54993	71.15276	108.6211	56.09457	74.868	48.30616	69.52876	94.07285
FAM73B	54.14137	84.06119	78.35725	115.0203	147.1721	48.61733	125.8447	210.8241	38.87297
GPR34	524.6121	46.45319	248.2301	40.99029	54.75107	42.85344	72.34703	55.73965	147.9555
DLG1	766.2	777.5054	403.457	811.0186	330.5519	389.0309	248.9942	334.3914	874.4273
SHC1	45.30227	43.42739	43.02443	715.7994	222.1074	366.5646	299.6485	265.8943	599.9711
SLC9A4	56.71836	48.18293	37.65367	51.83496	50.59183	41.32628	41.73843	52.53347	32.70189
SYNJ1	11521.95	10649.96	8544.273	9141.272	6569.146	8412.012	6344.132	7204.103	11599.74
GRID1	33.49936	52.89731	39.57454	42.59313	35.67363	45.72501	34.98376	38.51944	43.02956
SV2A	584.4269	53.55612	132.3844	433.943	202.1677	580.7549	256.4913	228.5194	33.89161
PLEKHG4B	42.4111	40.03782	42.32787	85.75977	46.15162	40.71321	281.1568	55.40717	84.74928
CNTROB	34.29984	741.6569	499.8712	50.96253	53.89837	54.56431	177.0668	491.6538	38.96672
ROPN1B	99.62219	105.1145	65.20348	115.7243	58.68248	59.22658	63.13504	77.19002	44.72682
FAM189B	55.34454	40.88599	33.10679	38.84989	36.74119	41.18304	34.99028	192.9376	48.5957
MFSD5	48.22118	94.52215	759.8204	52.48745	1413	296.8373	802.4765	1210.622	55.72236
RAG2	48.93241	38.38862	67.35692	72.47028	44.4281	66.15288	42.72553	50.66099	60.99092
ODF2L	235.7739	106.9047	78.60249	161.9758	154.9622	107.3948	411.4937	204.5605	203.2491
SPATA19	111.6003	65.1554	52.01955	69.03165	57.13757	66.38829	58.09189	49.78022	216.0813
EFS	70.58736	105.1477	1981.862	48.92852	4044.67	63.07996	1891.877	1428.255	122.9923
NDUFAF3	1426.271	72.14751	40.36213	65.28654	40.44593	50.99607	48.55801	60.56589	113.2383
LNX2	251.117	150.6146	109.8984	158.8134	148.78	111.0211	118.6468	117.8994	149.2252
HOXB7	46.74416	48.86625	41.99139	66.425	504.1047	37.80376	164.4155	34.82004	40.50368
SPRR2G	3707.988	1467.204	3960.292	966.2128	16863.03	720.0087	4571.7	9317.863	1334.744
RACGAP1	441.9138	273.1961	150.0123	270.9854	95.82142	170.7439	75.6763	123.3591	538.9374
CACNG4	147.4282	131.6098	83.95606	135.8493	79.95425	112.9601	59.41208	91.48804	156.4508
HSD17B12	112.0982	307.4709	870.9699	1511.847	439.4358	959.4972	275.3694	573.4658	62.64153
LPCAT1	465.0498	302.9032	191.0497	352.4085	209.2532	236.0605	235.6355	291.118	572.3805
ST7	1141.804	1150.244	2256.236	46.1667	2940.773	1047.887	1934.479	2866.071	130.6256
GLO1	301.8846	187.4163	117.1975	465.6317	318.7875	483.0547	307.5309	119.4021	507.5115
C9orf140	94.71448	46.72567	295.8074	54.7738	68.15856	68.59809	49.46837	220.4285	73.05907
FAM19A1	74.12437	36.40416	41.34902	39.7053	43.57356	37.554	33.72145	37.20171	30.61198
GLYCAM1	33.62468	38.83682	46.46926	46.02809	35.91771	50.79653	35.29115	34.14663	40.74745
PCDHA5	75.09731	60.36696	47.8122	86.52588	61.6756	78.7644	48.11463	46.348	104.9668
NEUROD2	77.15013	82.74741	69.008	56.76742	49.39979	67.39505	56.47247	90.42579	143.9637
POLI	44.89194	345.2044	378.6874	973.3497	264.6595	351.0072	377.4138	197.8514	60.79774
MCM8	109.1045	84.49332	59.34367	211.5209	47.85972	81.36301	51.82166	62.67806	239.3274
CECR6	110.2406	97.59627	285.8105	127.1626	157.9901	89.97484	128.3055	64.54218	165.4659
C1orf54	512.9356	1801.37	3426.505	1513.143	2220.687	2669.386	4582.826	3212.982	613.1547
FLJ45445	59.68695	45.93481	414.3607	49.66006	41.60395	40.92902	109.5711	36.20687	2233.412
TMEM68	98.72176	309.4625	414.2885	511.2063	579.5439	619.5714	491.3153	548.4391	93.83171

KCNT1	2236.334	4316.194	597.0072	689.8979	387.9442	1658.57	2373.306	4124.068	6340.814
HDHD2	7628.557	6636.542	5847.741	6516.781	5176.463	5956.036	4410.697	7030.722	9325.976
KIF3B	539.0751	346.8109	273.8299	586.7021	241.2672	421.6096	253.7238	389.1675	795.851
CARTPT	309.6831	169.7875	98.93385	231.2281	97.81126	167.1287	90.45588	110.5985	447.893
DOCK10	712.5252	1474.981	1636.918	455.44	771.6064	1663.254	1084.749	784.7969	160.5863
LGR5	151.9501	73.88756	85.5863	147.6116	65.61012	62.78537	152.6908	65.49174	159.6421
JAKMIP2	34.67506	49.69848	51.85939	53.79917	54.22602	273.6129	46.65246	42.40815	53.4258
DRP2	47.3647	42.68935	48.99469	32.73555	144.4319	57.67362	91.8568	33.99369	37.84536
MMP16	32.68308	32.12275	33.82334	41.04929	40.54353	40.86982	64.27591	127.3022	40.61712
SULF1	147.1293	44.56337	212.7339	466.7832	117.0148	555.4563	234.0493	141.3422	98.48229
BMPER	50.26755	56.95892	47.344	34.30994	40.25648	39.53598	36.39725	43.87563	144.2017
CST9	32.98804	35.82277	37.79479	41.58996	38.93878	34.7907	55.08807	34.88385	41.74978
CNGB1	5146.621	5042.684	1996.924	2748.468	849.2573	1290.941	1297.328	1111.397	3041.182
ZNF706	8821.157	8742.107	6249.518	8794.468	6693.105	7176.409	5697.326	6814.487	8727.684
LILRA4	240.5777	135.8233	125.2628	152.1849	95.28413	114.4505	93.98403	94.29926	297.7078
ZC3H3	14442.28	15393.97	10906.19	14123.12	9551.912	12721.39	11113.43	10699.02	14412.75
PACSIN2	1755.682	1621.244	6061.858	2800.352	8806.563	6229.922	8418.289	6539.031	2357.263
PKD1L3	161.2301	100.0679	65.82489	91.20049	61.30214	75.88525	47.0769	47.59469	194.052
SPIN1	2007.219	1050.694	808.2748	1929.906	478.3312	912.6835	464.4604	252.0205	1408.866
LRRC67	168.1423	118.1225	78.75836	93.69298	62.052	107.7474	59.68594	104.1004	95.45287
SLC35D3	32.4513	47.42152	41.06071	45.97699	105.2672	52.59423	39.66511	42.94165	33.95001
PCDHA9	290.0418	167.4438	100.9804	172.4651	87.39925	145.4594	81.29325	113.6639	333.1216
SOX30	297.8773	127.3891	84.43174	255.793	78.09431	127.6367	64.81453	85.57283	595.8885
DNMBP	63.9419	96.39645	59.30451	53.68747	51.33092	56.93222	52.57	49.79668	186.61
IGF2BP2	8794.198	11910.22	15063.49	20203.4	18814.7	14198.44	20615.03	15817.74	12984.76
HOXA9	35.0354	38.84916	32.89343	56.45741	34.69353	43.79938	38.49225	39.74297	98.02003
SGK	80.91063	1635.957	4264.443	1041.864	11065.28	2959.676	7623.536	4217.343	89.34706
TRIM63	211.4712	456.6041	153.7239	300.2904	97.38006	366.2651	534.0247	970.7079	267.0452
FTMT	34.8144	34.12367	48.90775	53.76719	34.82136	42.17762	36.70066	38.25641	85.90575
RPS6KA2	35.667	3309.253	4491.726	2271.733	8968.855	7878.163	8712.751	5841.547	43.01622
ALDH3A1	23750.9	9929.834	16216.08	9890.707	18590.18	17099.76	15398.12	12128.04	21840.16
C17orf48	51.73902	40.42228	562.2365	44.68959	517.2004	39.22503	548.8428	541.787	71.7982
CD244	191.8649	138.574	263.768	133.1461	155.741	130.9758	132.1453	301.6977	131.6292
TBC1D29	39.35551	33.67025	42.58713	30.40364	48.47575	39.95162	31.10373	35.77275	40.08169
CCDC55	68.96839	60.61654	93.65751	95.05672	74.28617	105.0274	67.61037	89.02496	88.68588
CALCA	32.36247	40.06765	43.73825	39.21796	41.91706	41.50807	35.36165	42.63969	36.50843
C10orf96	35.22906	44.50253	33.74814	48.5967	42.22432	47.80997	33.35622	64.2621	52.99839
WDR73	38.18526	646.4973	576.9102	694.3558	735.3129	730.6882	572.6847	561.0975	48.14423
KCNA3	203.2525	75.39352	74.10923	1103.449	377.7316	571.0187	252.3676	42.30952	2241.969
B3GNT5	55.3658	36.64798	112.3549	44.15844	64.70148	57.18977	48.42891	59.76818	60.5069
FLJ43987	54.05159	68.35054	58.14422	89.04256	54.57435	53.00471	55.5937	61.24907	78.27787
RETSAT	727.1667	128.0272	152.3726	321.2772	209.6279	206.2924	134.6121	130.0501	150.629
KLHDC10	1504.102	2063.451	2257.01	2089.919	1020.767	1306.576	991.3102	1421.76	109.0929
ETV3L	42.45671	50.52357	38.16787	42.70195	39.05926	42.3092	36.60436	41.98801	165.969
C17orf68	2912.605	2542.775	2254.1	2039.614	2572.607	2104.52	2206.275	2491.839	3801.028
SLC33A1	622.5962	47.33686	411.4829	386.9626	415.7597	301.6311	259.8768	235.6615	42.7268
MTRR	45.70336	49.20501	47.08311	42.38472	35.90247	36.72826	44.91138	60.53356	75.79692

TNFAIP3	106.02	71.84168	174.244	360.3035	148.2549	176.1893	208.4375	80.86645	57.51408
AFAR3	87.42616	224.7619	184.5703	304.8766	129.3513	154.0237	159.8331	174.7722	809.992
LSM11	5189.289	1485.143	3104.616	4414.833	1241.714	1669.751	1266.867	2128.094	6762.728
PPP2R3A	1352.907	564.7632	604.3091	2430.455	360.0346	648.5479	229.4162	431.8688	1498.908
MECP2	410.4839	181.992	34.48238	46.33185	38.86138	39.06587	47.46714	33.20393	56.18595
KLC3	37.30955	45.83529	35.69956	38.39861	504.6445	37.93265	219.7956	36.53126	57.14656
MGC39581	64.05317	49.9695	55.16204	87.05452	41.3168	39.28616	46.65953	39.98528	51.21173
IGFN1	45.91122	1735.06	208.249	45.54729	249.0533	456.8035	178.2135	913.9028	137.27
BMP15	114.6907	54.70526	63.54522	54.67149	59.15358	49.99274	48.44489	42.03873	50.84799
TMX4	89.19306	45.4208	61.37632	889.7596	312.1695	397.0302	346.8239	34.53099	44.83442
PGM5P2	46.87329	53.37693	54.69248	39.66047	101.8029	58.36851	47.84298	81.32385	181.9603
ZMYM1	3131.815	3940.809	1384.023	4088.724	1338.374	2262.83	2392.848	1132.794	4876.159
43529	891.1728	147.1295	73.79685	472.6329	48.19833	110.5394	145.3352	51.30109	191.3401
CYP26C1	149.5701	89.43851	71.13248	111.8863	76.39141	96.71543	68.56944	65.96699	410.8706
LRP5	2174.493	1553.94	1402.092	2911.158	999.9843	1901.636	1651.31	880.3785	1702.327
SEMA4A	302.1561	160.5894	357.715	181.1205	1619.533	133.3403	884.4689	776.5084	249.8413
KNDC1	503.4118	455.7218	133.0083	88.30047	85.21439	209.7605	68.44053	194.6018	85.90034
LOC541469	134.3106	129.618	76.76652	147.0548	104.3869	145.8195	81.38524	87.74718	140.7182
PDCL3	2324.936	2983.377	4305.693	2158.539	5642.859	3017.86	3972.891	3851.329	2323.605
OR4E2	43.47736	71.13985	48.52625	53.35524	38.47318	34.3582	41.84381	36.44456	46.83255
FLJ41649	53.43412	57.53961	66.07233	88.78728	49.19141	45.2194	54.33899	79.11691	88.57131
B3GNTL1	141.9303	36.61773	249.371	33.41357	35.95459	37.42588	38.26317	36.65554	45.51403
TTLL7	58.22465	53.58207	41.25953	57.26105	48.68912	44.30814	108.2422	59.69653	68.77962
NTRK2	170.8301	254.0244	108.8681	117.5726	115.0167	107.9263	71.85593	126.7745	389.2468
OR52W1	112.317	159.6258	61.50811	94.15171	58.91857	69.21789	69.14183	67.42431	223.4184
TAS2R45	112.0368	46.99652	42.23516	44.73207	80.09005	61.10728	82.8062	124.4586	76.39017
SH3BGRL	44.36241	70.63376	54.7198	71.6989	37.93707	49.68423	78.38382	54.53769	46.89523
PSMD1	10890.7	11084.36	9586.441	11437.21	9519.656	7583.708	9881.392	11951.48	13699.17
VNN3	45.12248	39.65345	36.14511	37.4837	37.56425	33.46245	41.89624	34.85498	52.18372
ADHFE1	81.25687	524.1078	403.9066	95.29872	582.206	874.4462	1163.512	883.6086	230.5831
FLJ22639	54.50051	56.47904	56.52453	74.55109	40.05432	67.87942	79.1206	48.31493	57.00398
OR2AK2	37.6496	47.80207	55.74675	47.5443	37.53376	64.18638	41.25079	51.30887	51.08752
PLEKHA7	207.7269	139.1809	222.0659	205.8601	233.0195	289.6019	185.1049	231.7201	699.0267
PKIG	428.7837	247.3926	172.8039	955.5761	108.4723	161.7164	158.8382	90.20048	1007.173
ADM2	235.0633	3992.805	2821.868	3440.622	5716.266	2258.114	1906.416	1814.575	4089.692
THUMPD1	45.36285	73.51761	153.114	63.12128	135.4862	61.62775	111.7983	104.4791	116.0675
PDE6H	161.3354	104.1756	90.93983	175.3591	78.78719	104.0467	78.3435	102.2049	314.2877
NLRP6	42.00228	40.68288	33.77367	36.90926	30.84962	32.66982	33.60355	38.14193	31.67903
GJA5	4857.321	2850.433	1319.844	1712.888	1037.679	1426.356	823.21	1506.965	4797.59
TMEM166	38.67743	40.26115	203.1468	48.37152	40.37027	46.74208	189.2555	31.84289	40.28361
MTCH1	8224.673	9684.102	8604.571	9881.491	12190.03	10662.93	11639.23	5651.509	7964.027
NOC4L	74.44974	45.73196	44.09644	32.31902	37.64358	34.46642	93.16512	215.494	125.8184
MAGEB10	79.40041	55.126	217.1826	118.9966	59.59881	70.30097	50.56749	47.51532	62.94324
WRNIP1	217.4949	112.942	79.87466	854.6397	173.0641	76.74114	105.5445	310.7097	973.2111
IDUA	2325.918	45.11328	45.92413	50.45325	229.2112	45.66076	170.5606	251.6252	34.48456
AKAP7	881.8407	1317.259	467.0586	57.2977	238.0983	751.7623	312.4976	451.3005	1230.577
SYT15	54.99625	63.76823	50.43271	39.94996	35.83398	53.30568	43.44165	56.33858	51.98524

CREB3	105.3165	129.7714	112.9419	136.4386	90.56415	138.9855	65.39324	101.0451	159.1577
CPD	34.44695	504.2803	1330.561	478.2718	2160.187	1520.093	1752.928	817.5905	33.92395
PYY	83.61523	46.95682	65.00671	269.0783	82.51785	65.76637	57.50658	93.98846	307.1228
NFS1	669.3552	315.6357	195.0059	421.491	141.5174	255.0368	211.1159	267.1299	554.8354
RECQL4	257.8414	98.48455	308.4962	235.0653	140.8723	76.76348	56.96771	196.5019	243.9847
UBE2L6	333.969	183.8032	906.3521	1180.054	265.8324	133.6977	153.4148	368.3064	426.4973
KCNE4	692.3439	37.9183	209.8349	66.15893	40.32621	31.72055	53.99724	46.50068	50.0197
BBS7	70.41947	48.86718	49.71843	59.93006	69.30338	43.68329	59.61507	48.87911	39.1286
C1orf200	108.0651	84.39901	63.66904	106.3556	55.7061	66.84577	59.06496	69.22146	82.21475
AXUD1	12461.13	9836.646	9822.333	9707.514	8623.39	10483.76	7866.166	7357.455	11984.14
LYRM2	49.14128	42.06253	118.1263	46.47963	36.95255	34.965	133.3446	45.7051	51.25233
NEDD4	343.4842	198.4878	658.0768	618.2586	84.40524	120.6294	174.8692	87.29514	303.5999
N-PAC	1212.443	71.25234	138.3148	108.8718	132.5429	80.89246	83.22263	54.93731	121.5815
SFI1	50.12759	76.00772	47.5499	44.44237	43.13713	38.61684	43.5568	203.9909	1656.323
SCG2	254.1564	413.8416	212.1973	156.1251	231.124	138.3622	599.3812	80.81602	204.1492
ETV3	169.4451	115.9194	169.0808	138.6251	77.24262	85.55994	193.3623	179.563	161.6351
GPR148	186.0048	217.8246	86.08393	207.3891	78.19665	96.80581	72.07182	92.91137	283.5164
FGG	35.81389	35.90864	42.01582	39.76039	51.66754	48.06233	35.73841	36.904	51.93368
PRKD3	459.8735	210.4164	110.1085	189.8869	103.6416	162.3952	98.60315	126.3118	429.3833
EDN3	39.31222	66.71171	44.07033	55.46359	59.13207	53.04116	33.56347	38.39486	90.08453
FRS2	33.72747	208.6123	223.4572	44.68182	111.3326	165.7423	153.6351	34.22501	38.14933
KTI12	61.91353	70.59317	412.1898	124.2218	455.0448	315.0982	333.0157	278.0905	156.4505
MAP1LC3B	517.3513	325.591	285.968	412.1184	387.7409	379.7957	321.1823	170.8797	91.7247
SBF1	2587.539	3929.137	5336.118	2470.239	7560.032	4023.382	5606.299	6083.405	2060.637
SP140	85.10061	54.77516	399.9075	54.99938	126.3464	275.0948	238.5727	45.20961	174.4488
CXorf57	33.84508	41.71702	36.77837	35.07224	312.8163	35.60885	46.92767	296.8847	46.07519
SPIRE2	141.3028	931.4696	700.8992	141.485	840.7284	889.5038	192.4517	328.6381	68.91823
C19orf6	1271.409	1004.017	1548.643	734.4099	1725.349	984.9089	1687.21	1694.803	94.49624
TWF2	535.1407	301.1419	294.3054	699.9188	614.9609	826.1161	573.9187	427.4444	661.6541
KLHL36	4173.053	4878.886	5528.047	5164.453	7190.339	5893.769	7027.074	4505.936	3568.074
PDSS1	54.83798	65.98413	295.8164	809.2999	276.6158	47.34738	168.2419	243.7266	120.6922
ARL6	36.89625	37.01119	37.93407	31.86515	32.57118	35.04729	42.6939	33.40552	35.60584
GPR44	38.06383	55.90027	46.91737	49.90195	59.9356	36.86685	40.29919	66.79209	40.3572
PSTPIP1	48.14905	43.65706	98.30366	47.83104	32.72213	44.32415	45.79734	35.80467	104.4432
FEM1C	554.6262	573.265	736.4688	547.9661	815.6133	794.4766	463.7991	862.5677	823.2347
CHRNE	185.1409	186.8065	70.01288	163.6045	78.0276	117.9912	67.53264	105.0022	260.6787
ZDHHC12	69.53692	50.40316	175.9861	1887.82	186.6639	40.72622	555.1396	270.0536	44.21843
NAV1	44.60576	47.58638	48.16272	267.3077	70.54665	36.81532	66.01668	66.7508	84.95089
VPS41	9671.457	8501.422	8349.692	8324.041	5129.507	6712.608	6837.7	7853.442	10176.62
FZD3	194.5482	762.5237	72.68703	101.7244	339.4236	1051.168	641.5393	309.47	139.5722
PARP3	80.2842	154.8913	85.85925	42.7679	94.91246	131.0033	67.29921	59.01429	160.9213
LATS2	44.9581	53.55481	50.41879	39.5275	167.7021	158.6461	263.5032	186.7873	41.13824
FLJ10374	862.3656	1333.25	1077.517	973.9358	868.4413	1169.482	1230.481	1198.159	652.1819
PLSCR3	11512.42	11160.71	14050.76	16627.78	15645.91	16343.22	17269.93	14056.14	10342.88
ZNF658	2097.113	1648.344	1064.091	1433.468	1590.453	2589.309	1407.236	1330.26	1098.83
ZNF593	4290.645	4232.508	3835.799	3712.609	3196.695	4270.028	4732.347	7813.726	3004.979
WNT9B	141.9904	85.56445	66.30691	80.78004	74.13747	155.5156	74.3151	78.8356	170.6751

TBX20	51.74015	47.89767	43.90182	36.60894	35.5694	41.3218	47.23676	46.15411	43.73076
ATF6	786.9752	2813.467	6101.173	3489.27	6953.42	6089.685	7409.793	4163.78	1678.503
POLK	474.2964	367.7451	316.7386	255.6414	241.1986	217.9488	121.2907	158.3955	44.24595
ARHGEF2	5891.964	5851.417	6403.91	5928.389	4781.107	5880.269	5949.567	5921.983	5239.335
LMO4	628.6053	777.8587	2886.284	1673.785	4007.678	2956.594	3322.238	3255.021	1363.993
HISPPD2A	34.92889	37.1791	47.99126	41.37627	38.69064	35.15985	47.16498	42.38366	40.58783
SRP68	276.3286	160.9133	1027.79	787.1739	1882.434	151.8199	1672.765	2573.585	473.6007
S1PR3	104.5366	68.33909	44.63811	103.21	431.901	292.0508	313.9767	47.9132	40.70867
AIF1	2826.225	1189.771	1251.381	1377.563	328.1803	570.2651	596.05	896.3238	2166.963
CSHL1	520.0594	356.5117	143.481	317.755	118.657	275.3828	169.7396	388.7805	747.3688
FHL3	42.71301	54.18051	651.7445	291.0851	372.0562	351.144	1139.613	1134.685	44.1167
DYNLRB1	83.82497	52.19541	188.138	53.15053	231.6052	84.4345	161.6869	115.8634	54.12119
GLTSCR2	7998.331	7791.023	9445.128	6326.295	8884.06	7427.865	7924.399	9673.082	9175.729
PCDH20	36.73162	60.41779	49.61289	43.13514	53.63239	56.93716	32.32243	40.90744	38.60863
DNAJC17	5406.702	4431.578	3149.561	4518.475	1903.931	4264.953	2690.865	2784.715	4776.503
BAIAP2L1	38.87777	1622.516	1741.571	2164.417	2115.085	1855.947	2073.373	1937.145	34.54905
AHSA1	59.73147	459.2828	240.353	68.69748	186.5715	96.34779	481.2725	480.8836	163.8011
PPIB	1574.571	1566.233	1420.681	2335.024	1024.839	1973.216	1032.994	1031.885	1573.061
EEF1D	32.30582	36.26616	34.70432	35.07983	35.76506	40.66699	32.80331	39.58044	38.22428
TMEM82	62.13735	46.07746	66.75166	80.71239	51.92695	44.56741	51.51873	42.49252	106.9002
TNIP1	668.6758	50.39174	914.1907	352.7318	572.8011	414.8957	751.8186	648.7437	889.2977
COG4	158.3328	91.92431	69.31007	99.23203	55.43797	55.84795	221.7498	245.662	81.1618
NAP1L2	198.4077	153.6998	91.19113	200.0928	84.23696	87.33442	65.29371	81.92992	224.7599
BRF1	115.1351	147.0009	370.3395	711.4109	241.8217	61.31317	49.87242	43.90451	115.0841
RASA2	105.0767	72.56704	44.14207	58.65684	53.90557	57.27103	47.27476	74.74597	66.78042
LANCL1	388.2387	210.6298	274.0553	232.6199	118.7123	136.1751	182.3394	129.0503	447.6151
KRTAP5-10	93.64584	41.83368	41.69353	73.02161	50.17679	65.07708	37.09397	36.95961	85.69566
SF3B14	126.9691	337.0465	81.20403	161.1575	212.3004	91.66574	116.9237	123.2415	84.76118
AATF	79.00521	1257.221	1366.041	3565.045	1639.863	1259.004	1208.03	3259.706	99.39051
OR5T3	50.60588	68.82505	50.48593	68.91983	60.72708	56.10012	55.52153	75.60677	102.0259
CEP192	95.60046	93.50429	182.8171	121.1145	330.714	69.72557	410.109	197.4494	71.64517
GPR12	49.26529	57.91449	41.30104	72.36747	47.92081	48.25475	45.89302	50.74941	42.9481
ATP4B	129.0509	147.0762	74.77725	155.9688	63.89193	99.12905	67.60787	90.65514	207.5845
ADAMTS3	121.1707	48.23555	78.308	50.64117	57.7855	74.83173	61.28974	64.73825	59.52009
UNC5D	36.62862	34.95141	37.19208	32.35043	41.1558	39.93014	41.21574	36.52301	49.5492
AWAT2	74.21849	37.14954	52.63892	40.91884	46.45593	57.36824	37.83513	35.69701	115.8973
C20orf186	36.19877	35.46014	32.39602	35.19536	39.59558	37.12336	40.72549	169.2538	77.64549
DYNC1LI1	352.208	223.0413	181.1102	232.3159	405.4663	296.7293	196.9165	315.5577	299.329
EFNB2	665.6923	640.9266	623.96	1483.502	839.866	1443.672	1026.769	967.9199	1899.969
SERPINB3	5220.458	7258.673	2480.682	6148.306	5244.277	5330.024	3391.589	5639.762	5342.198
SFTA2	68.65737	407.5345	684.002	58.78661	629.2122	476.6954	60.68284	59.53864	1474.31
SUPT5H	47.54861	49.08456	153.1018	526.8472	334.7937	52.95385	164.4196	294.4664	56.33475
OR9A2	100.5957	71.42976	78.00931	71.57532	49.74269	47.12983	49.10531	36.27862	243.1097
SLC16A2	217.4381	789.3939	435.2721	171.6849	119.3264	115.5776	430.9524	81.12703	219.2315
TBC1D9	2761.314	2247.894	1332.374	1749.788	1096.264	945.6378	739.2827	575.8538	3594.651
HJURP	561.6549	747.1852	730.3679	3596.035	518.7037	876.7191	997.0995	615.2512	421.9183
OR6C3	149.6925	198.0328	66.2497	157.3949	73.51313	112.5361	69.41623	98.19554	231.463

MKKS	56.84794	88.53613	453.6463	573.5987	465.8393	527.3241	414.6182	885.7921	90.83006
PTGER2	75.94519	59.70654	196.0161	45.52434	151.0134	59.56902	47.96259	55.43878	116.7938
ADORA2B	105.2448	53.22445	121.1329	75.23191	87.11898	48.16032	68.79301	87.17445	54.66615
KPNB1	4435.232	4186.226	4544.774	4544.225	5336.311	5311.874	6341.95	6374.14	5171.229
KIAA0251	8514.707	6675.803	3944.578	8010.441	3532.519	4535.005	3874.874	3215.67	8272.506
MAB21L1	68.98022	111.066	66.72961	93.8952	82.61047	66.94541	55.57384	71.6912	334.5047
TIGD6	303.0523	185.8098	97.27161	168.9537	83.54626	133.0615	74.37444	94.02187	306.8964
ARFRP1	221.065	142.5082	94.10188	78.42024	83.28566	64.78123	55.65703	60.60102	57.36256
IL1F6	4422.381	421.2791	1793.841	448.1504	886.4152	454.994	508.0011	546.1112	1682.065
SYNGAP1	39.42667	35.00014	94.47005	36.56994	51.36719	43.84789	43.5844	39.60775	79.89811
OGFOD2	127.4125	168.1791	44.38266	58.34123	53.22548	68.57351	55.65709	54.29882	73.00377
TMEM207	366.0178	160.3586	94.82054	210.4988	87.63327	159.2328	85.00236	81.0148	457.811
TMEM160	394.8157	226.8859	576.5287	243.0276	427.2863	161.4903	133.4174	283.1409	597.2877
LOC643905	218.8753	177.4927	93.57707	163.1448	81.19721	126.3616	78.67538	92.51917	455.4047
DEAF1	266.3708	158.2617	93.08951	158.3373	87.02518	94.61156	70.26366	89.28239	247.7173
HSPC111	2386.124	59.5261	1625.158	1998.549	2508.92	451.3048	1161.622	2084.067	90.69926
KCNH3	2437.799	1106.874	1645.651	1776.104	1629.472	2198.54	2141.779	1587.554	3276.576
ACTL8	154.6218	121.6425	59.60098	155.6131	64.47405	80.26646	58.5022	62.03221	227.034
RBM38	47.41644	976.3668	34.0388	38.94067	34.27016	34.18431	128.6534	33.78537	37.30207
CMAH	778.3159	131.2309	458.5379	113.3747	172.7472	357.4441	74.6132	193.1599	142.903
DGAT1	2581.661	688.7769	1263.673	1153.053	998.5809	1426.501	1497.687	2020.106	311.2597
HSF4	805.1621	64.44371	50.66695	59.87085	122.2072	494.3694	100.3915	171.0798	435.4968
DHRSX	113.9671	85.54321	51.26399	132.0907	87.07077	81.0376	55.31221	67.98924	71.77351
TNFRSF19	2390.186	1262.135	788.1354	745.5961	1381.259	664.218	804.9144	474.1107	646.9665
SULT1E1	106.1193	127.8544	76.27574	149.059	61.55564	106.0599	58.1966	82.89101	391.9704
ATP13A2	2276.405	663.9976	1375.255	1265.386	2086.603	1163.273	1659.842	1212.064	839.7649
AIFM3	180.4092	137.0455	86.51683	179.8532	84.23509	107.259	80.08542	83.36589	207.435
PSKH2	44.72503	35.68093	37.01101	92.24931	40.25836	53.22186	52.1694	71.22388	42.72942
FUK	2974.277	2653.55	3076.257	2132.358	2557.38	2659.135	1890.51	3248.12	2422.017
DSG2	97.81895	60.05225	48.25361	1091.395	41.14316	36.32819	114.8278	187.4277	110.0334
CNTNAP4	182.688	76.3719	64.18648	72.45367	65.39277	69.64967	59.19992	51.30506	104.5623
C1orf27	75.00149	67.5829	46.91072	56.15606	97.39999	70.8351	95.65852	69.96364	41.76126
C14orf126	95.60408	644.5259	67.91401	73.25362	155.1736	65.66108	46.75103	55.85489	146.8523
OR52A4	48.82082	37.11234	50.15041	36.3714	33.2497	34.13587	43.58808	54.67762	67.752
MYO18B	44.40216	33.04067	56.36264	44.75697	46.32959	52.77467	173.3786	750.5096	36.33051
C6orf106	258.494	1463.852	1410.487	995.815	2388.632	1542.367	2214.135	2362.955	282.4559
RANGRF	2458.243	2108.154	1014.894	2146.847	453.1883	1317.091	550.8261	566.1995	1834.656
FXYD5	59.25977	4237.428	8830.99	7697.555	7986.64	5209.146	11063.71	12011.82	2374.849
SPTLC2	111.7486	232.3661	118.7864	98.09196	205.2911	198.0997	117.8066	89.25107	77.09744
CDH3	52.1427	56.2141	202.5708	649.1309	360.2511	436.1358	760.1783	635.1129	68.55341
LOC220594	32.48384	48.53862	33.32208	37.39674	41.28685	41.24632	38.33661	35.12731	49.7564
NIP7	1235.15	38.76129	1102.396	1380.226	1426.348	1750.125	1684.18	1569.566	911.3893
IL2	39.2101	49.89557	38.06947	58.94995	35.5703	39.18924	38.26098	48.02784	39.47851
UBE3C	97.03699	501.7799	274.9609	82.99143	634.1051	308.1914	267.7582	673.3538	89.9575
WDR25	52.48436	935.5494	558.9682	41.4581	792.3715	34.22653	562.2292	1020.091	90.93562
IGSF3	122.5089	65.60894	535.5359	821.8386	1183.864	290.7663	811.459	568.8448	43.26877
LIMA1	6241.676	5332.125	3363.034	8495.885	1710.189	2650.053	2759.492	1667.897	4015.36

GLDN	5152.043	1581.181	3355.146	2118.264	3452.375	2433.11	1606.425	2088.255	39.17884
INCA1	56.06185	60.95551	49.6211	56.83776	35.2163	49.7467	60.71909	48.3643	108.3324
TRIM37	51.89251	477.876	254.1485	35.87316	41.67236	535.4462	33.72287	31.61959	67.87177
FZD9	6313.277	4194.32	2609.321	3636.865	2689.165	2521.769	1348.378	3065.13	5249.143
PSMD13	533.3958	227.6168	125.0682	323.8707	162.5903	196.2184	118.9211	269.454	798.5339
PLAA	64.44301	51.79174	58.78881	56.49706	42.82685	53.54542	54.84399	35.64768	94.51148
FOXD1	49.707	50.03274	39.26684	358.844	54.57968	39.97645	94.65079	42.56901	43.72017
ZNF556	481.7023	208.0096	110.761	124.6506	55.40132	110.5948	71.30556	75.96458	195.8911
SLC6A10P	57.18114	690.0969	56.19114	1917.789	1140.736	1067.951	893.1743	1532.675	114.2826
BCL9	1180.333	1308.031	884.0922	1270.02	779.1876	1625.792	843.0765	842.5884	1367.851
GPR20	273.3689	78.35595	95.03677	98.06246	66.86282	77.70231	60.35932	94.47495	54.52824
EID2	703.1633	59.88891	128.6691	481.4327	42.12251	39.21112	159.6121	159.6065	53.52711
CCDC153	39.32229	43.40283	51.31898	48.45998	37.02826	46.88792	93.97208	35.61215	37.7812
COX5A	571.489	1064.478	1893.459	505.778	1673.822	1915.099	2371.094	1750.084	367.1597
OR7A17	161.2129	120.7554	75.12111	119.4884	67.5653	120.9506	53.72563	69.45016	215.4091
KRT25	251.8922	92.01585	76.91401	105.227	44.43508	69.58869	59.90672	70.03419	154.7709
SERINC4	419.4459	229.1381	106.4169	213.0952	98.86806	161.4592	81.74479	134.4662	331.8231
PPIA	332.8909	217.3382	113.5254	220.5964	102.6701	148.5559	91.29374	135.1379	319.451
PTGR1	1468.245	706.9099	372.2799	39.06525	529.4523	47.51581	187.5051	531.2481	3616.702
PHGDH	15408.66	17271.63	16810.91	15242.86	25406.64	18974.69	16327.65	14571.14	15510.41
RPS6KB1	880.9429	1163.221	1011.796	1222.008	775.8528	1453.122	925.8158	1590.894	880.6023
FAM53B	3626.133	3605.155	2221.147	3144.771	992.2673	1515.411	1096.094	866.811	2689.019
OR10A5	31.50589	31.80357	35.20231	32.34335	36.77119	38.70199	34.49451	34.91117	44.93183
TP53AIP1	85.47752	77.92522	70.52428	62.44706	123.4828	258.2951	44.04609	63.68295	171.5111
ABCA13	49.10603	43.9097	106.2383	38.27366	31.66692	162.3912	47.02969	45.22081	46.55175
HYAL4	406.4233	191.0718	110.017	245.1208	117.0857	173.1299	103.2334	163.0031	551.4865
EFHB	279.3108	168.5887	96.96835	161.3873	73.44455	125.4057	65.26053	51.29552	234.9363
VPS29	2521.39	1279.239	2025.88	2206.087	1626.528	1072.394	1525.659	1363.485	41.05854
WDR44	73.37257	135.2711	146.0512	335.9058	143.4442	185.1334	95.76587	138.9921	88.6762
SLC1A7	219.128	105.2616	87.07076	560.485	160.7253	325.1499	260.4904	73.80571	202.7012
C4orf23	64.20177	34.92646	34.10538	38.03099	352.6237	33.71983	35.31505	37.94938	643.8123
PPP2R1B	34.24966	41.85105	362.4757	1123.318	371.1006	196.3486	158.8306	183.5607	36.48045
OBFC2B	46.79957	40.42228	34.88618	39.28765	36.17983	40.35934	45.89503	46.65108	42.27864
APOL4	87.73251	103.7545	75.2188	144.7575	89.9521	87.676	53.93703	60.41214	56.42877
LOC34440!	49.48794	81.84303	54.04693	357.9251	130.2645	52.38873	138.1528	191.3419	142.4221
CSNK1G1	81.50725	65.81036	77.40182	104.9743	94.79115	83.34142	91.88723	127.6754	63.54289
CD93	42.69176	49.53828	356.5381	44.08903	688.0461	39.59317	651.1981	202.7708	175.5395
PROKR1	32.58386	36.81358	33.5369	38.20165	49.39677	38.56186	36.02145	41.50986	34.31747
EPAS1	228.5523	835.5729	1517.036	1638.423	1588.61	788.0715	1977.555	644.2792	38.44258
ZSCAN22	85.85931	213.8257	226.3498	71.89427	192.1782	159.696	128.1717	111.522	71.481
C10orf125	69.48141	55.07209	69.95094	69.71794	65.85787	38.25441	40.38703	50.74727	62.69654
FADS6	101.1591	53.92154	51.1503	66.05698	43.35645	42.69037	37.55975	36.40401	72.51685
MYPOP	6420.935	7180.823	6783.87	5668.618	7296.842	6735.187	7420.292	7136.68	4496.454
MARK3	357.1557	539.4819	508.439	681.5543	233.5381	118.4208	322.3672	328.8377	2693.832
ARL4A	83.14208	897.6326	181.274	578.4978	571.5186	143.0388	469.9216	252.9581	46.66132
OR2A12	43.61048	38.21168	56.33705	51.03511	39.12632	56.76868	33.25348	38.26233	73.60017
MYO1A	185.3285	449.1852	319.8116	216.9236	85.79184	120.7758	196.5907	462.8209	203.0248

PGLYRP1	123.673	108.507	73.97771	55.37131	56.07198	64.40936	61.73344	69.9333	120.1617
TCEB1	2730.573	2876.494	1821.836	3320.804	1787.435	2161.443	1823.5	2749.487	3195.629
USP38	463.2946	798.3929	1172.507	578.8806	1599.393	1039.106	893.1194	1578.98	598.7975
PPCDC	74.72558	63.62641	91.42964	76.60158	42.49887	49.67015	191.1291	47.941	56.07504
GPN3	845.9607	668.921	532.2018	666.2443	499.563	876.8126	751.0485	528.8535	831.007
TRIM5	61.16475	60.30925	48.33697	371.7473	47.21506	293.6044	100.0385	53.99446	40.28233
SARDH	43.132	54.10594	33.95084	35.13932	48.63834	33.13197	42.90712	42.37561	36.22261
ERI3	121.6081	65.25589	55.44219	73.25213	54.10888	91.44848	59.89695	55.3247	163.6744
CYP27A1	299.2713	151.8073	84.67033	139.0679	107.2598	131.0796	79.43499	114.9354	75.68794
SEH1L	39.49687	31.68608	34.21728	46.73773	36.06383	56.30198	42.59923	34.84371	38.48816
FBXL7	134.4989	86.36611	61.12021	111.9515	89.66742	119.1239	84.16245	59.81186	201.4842
TMEM67	152.7941	56.83361	63.77824	53.66279	50.89338	58.77602	78.71902	64.73302	89.84222
NDEL1	8551.726	11741.71	11880.31	9482.535	17600.18	12697.39	12226.31	12755.87	7920.905
OR1J1	96.02595	60.6825	56.58846	54.47514	52.99591	57.36891	49.193	64.36449	89.37887
SLURP1	5731.266	1973.9	1499.862	1242.708	1414.454	45.10283	249.7042	277.4121	2457.911
FNDC3A	36.99676	40.60952	34.3243	31.86116	140.7076	47.17174	57.97425	41.92304	38.23122
NUMB	2719.459	3686.395	4919.894	4409.449	5098.929	4811.196	3497.982	4756.293	1713.795
C6orf85	56.10803	785.3828	249.3979	81.51109	1786.802	98.16023	1566.618	1172.851	149.3589
ULK1	12312.82	17469.28	18089.77	17985.74	24819.96	22295.63	20617.05	20963.36	9130.734
SF3A3	19075.6	18291.5	20671.03	17920.74	16737.71	20082.15	18036.88	16691.68	20920.22
NEFH	76.72917	78.46159	183.4772	142.6558	71.06696	286.0472	59.45452	251.5451	162.7888
SLC26A7	97.12484	69.49709	60.92342	94.19849	54.12273	88.13323	53.77602	59.44983	50.45
MPO	437.197	557.9138	225.2358	263.5541	237.0475	187.1777	98.84285	257.9627	556.7133
LPPR5	60.54496	56.12541	63.55196	52.98915	53.22606	36.91118	189.791	43.75579	119.9872
FOXC2	262.7345	204.5101	94.15233	212.9937	179.2672	134.5082	94.14487	131.9386	390.3425
GRASP	98.20559	67.28172	231.9899	47.83954	64.97563	58.66145	56.67062	74.32931	42.32991
TMBIM1	890.7371	1007.153	1091.328	1698.922	1483.463	1213.119	1278.945	1578.348	841.5264
TMEM110	58.09438	41.02131	324.1547	48.14296	283.5692	50.70302	49.88661	37.17345	47.92741
PRTG	41.67954	53.91087	151.7114	50.21336	42.80685	43.74111	46.11108	50.82307	40.83473
SLCO2A1	1580.478	1276.467	3258.93	3616.968	4403.705	2477.401	5504.822	3121.072	43.49669
TDRD7	413.434	314.3091	365.2125	350.2557	204.4445	554.896	317.7615	340.2335	348.0477
SERPINA7	52.13749	59.07065	46.30106	60.30966	46.25691	49.62281	50.86311	52.10275	83.47907
DNAH11	34.14259	46.35016	36.3089	32.52481	35.92339	46.66619	35.61916	36.68132	38.48048
KLK8	122.9553	84.43732	241.9241	58.17995	112.2502	64.53515	42.69638	54.16843	127.2016
PTPRD	32.71293	42.25032	36.44176	35.42626	92.32254	34.41725	42.20351	35.10461	30.94114
CAPN1	598.5045	500.8875	553.1086	539.5272	466.1607	484.8289	510.0601	649.2846	847.8948
RELA	61.01373	41.10021	36.80735	38.02895	45.72174	45.0305	36.06002	64.40409	40.4965
MYBPH	17931.4	18085.07	19683.77	17215.72	8248.72	20478.59	23038.73	24828.75	15163.25
TEAD4	7479.607	8171.076	9232.075	10698.13	8206.195	8690.305	12070.32	12816.4	9895.256
FCGRT	872.6743	1088.94	2606.535	455.9662	2117.812	1208.347	2909.658	1931.119	288.8863
VEZF1	3452.548	3218.924	5858.261	4425.936	8388.163	7637.051	8525.815	5866.503	1920.615
HRNBP3	88.96092	65.89117	36.72525	36.96585	61.58051	62.90017	48.20502	52.14655	64.04942
ZNHIT3	185.9368	105.5375	62.4352	109.0013	55.8549	79.62606	52.14619	80.0443	557.4886
SPATA21	152.8401	58.97734	47.81997	47.80259	42.66972	48.56808	40.78112	56.78381	64.73547
NBEA	41.04277	45.60072	48.10999	59.77573	49.52997	54.77143	49.48914	36.46532	64.89454
FUT9	228.203	53.4278	40.95417	36.21792	33.54576	40.35775	38.18447	66.0005	39.01639
BAT3	1954.276	2061.792	4791.488	2702.308	5102.235	4637.863	4381.384	5594.587	1791.987

THEG	79.70423	81.38148	42.45258	68.67503	62.38576	45.31036	60.11491	69.99222	173.7195
KLHL22	9029.744	8947.543	7821.409	9027.706	5694.969	8133.152	7709.048	8351.315	9780.789
PIK3CA	81.98929	66.11969	126.4366	57.01522	107.4476	279.7845	61.91214	67.22598	115.3021
PDK2	87.46724	181.6359	65.55783	111.2676	65.13172	199.4786	91.88622	161.8067	83.35641
CYP2S1	2578.248	984.9523	6418.203	1150.801	12883.95	3950.528	9731.73	6159.009	146.4109
C1orf213	70.25344	80.6189	181.2764	85.18539	85.4643	91.50017	63.33024	79.75661	100.9077
CBLL1	4486.422	6046.843	5101.414	7823.986	2371.901	3409.287	3356.185	2772.52	6166.985
TRIM58	169.9363	99.25848	62.68789	148.1974	58.9017	79.55215	141.3101	91.45042	140.2831
MRPL41	39.27357	843.8951	263.5326	904.6639	490.4106	783.8051	303.3589	542.1791	58.01542
FLJ30058	111.0614	47.26597	48.8674	56.95632	37.13082	44.60579	49.6592	54.7084	52.59535
RDH14	77.15541	56.00772	247.8853	625.3835	179.0655	105.9548	158.6818	219.8094	82.49668
CAMK1D	41.98142	346.933	40.78054	32.97345	92.7215	35.30467	34.11489	33.28918	53.55169
RBP3	97.60395	91.89082	64.20004	57.83771	54.90417	62.97043	40.78246	63.37587	51.39055
LOC401589	92.05446	37.62469	51.07096	71.9204	51.64115	45.58333	52.51648	45.83931	54.53578
OR56A4	121.14	62.05988	57.90036	52.29176	56.46955	69.93803	50.69661	70.58224	305.5377
KIAA0802	76.41323	45.65225	56.1297	77.29332	38.51536	44.90033	97.32976	41.57026	46.23856
H2AFY2	711.0583	268.1949	299.2991	406.1897	363.8116	668.3634	342.8036	154.7547	893.3873
MPP4	39.8803	33.04404	35.11917	49.60471	231.6855	44.21104	217.0459	155.5507	33.06696
NAT10	395.7849	690.445	1484.865	1081.486	1870.802	1577.677	1600.585	2126.976	406.116
KLHL17	133.4022	96.18683	53.5135	83.57945	209.1483	61.59825	48.06087	53.99971	147.0877
CFL1	18983.7	19882.04	21791.42	26240.06	19912.59	22608.05	23891.05	18835.26	18397.43
ACR	241.0611	155.6447	82.25848	199.8183	156.2821	117.2011	68.88578	92.20449	297.6163
BCAM	38.64477	95.18221	57.13719	68.84784	44.76259	54.52517	44.04698	60.25965	43.72635
FAM71C	62.09952	41.42125	49.62351	54.46886	38.07215	42.26977	45.1683	40.71803	138.0702
DMXL2	414.8955	850.6004	98.76163	211.5502	89.42206	147.9	216.5096	102.0682	391.0559
SULT2B1	383.6533	179.8825	97.49135	233.7514	152.5585	101.3581	84.79086	91.47187	369.9372
C1orf220	32.45223	30.94485	31.40981	34.31663	49.29941	34.7791	33.23455	141.411	42.97853
SAFB2	911.5525	2398.457	4792.822	1099.363	4832.692	3848.653	4183.175	3431.099	89.98134
LEPREL2	99.31976	97.85519	77.91475	124.813	273.4296	70.08569	301.0855	399.7819	114.1656
MEIS1	355.4808	233.2899	691.9819	225.26	588.6461	360.6659	367.3453	236.4479	334.8894
NRGN	92.94089	61.77012	121.8431	94.03613	55.13394	59.29812	119.9001	59.28785	231.8939
ADAM12	395.0411	207.5939	104.6745	202.2583	103.2773	94.51856	66.92458	71.60436	257.7618
COPS8	3450.565	1969.09	1371.055	2872.219	980.1949	1345.107	955.9344	816.0271	2970.134
KIAA0460	1493.297	36.17135	727.9181	848.2444	1329.651	1082.843	884.2066	375.2065	43.09365
C9orf131	188.0882	137.7677	88.93032	137.2329	95.61581	155.6662	62.50235	114.8552	303.6422
IMPDH2	235.6258	153.7168	1453.644	180.9336	2751.487	158.477	2533.839	1556.408	320.2428
FLJ10357	169.398	126.3968	100.2792	216.8439	267.9771	134.5998	78.95264	93.60046	70.81064
OR14I1	98.76337	59.22532	41.46165	48.21463	47.11732	52.56466	44.55621	49.35125	114.4144
LSG1	82.76945	50.92155	138.1988	137.3438	202.7193	111.6348	160.0709	109.8055	83.33693
C3orf26	162.1671	2465.736	725.6068	5946.415	1888.854	1351.136	1845.623	2005.805	4412.289
CCNB3	37.77072	55.27804	37.96823	65.83508	35.92436	46.06225	34.95447	37.70938	37.47322
SCT	148.1811	156.7458	69.96697	144.8721	59.74817	96.10945	58.68429	85.34356	227.2843
CRAT	117.0724	127.4401	69.80584	127.3709	59.56205	85.60786	65.79199	94.57217	252.3955
IL7	37.98632	34.73691	35.68962	40.24003	44.1651	51.62572	35.44187	141.4416	53.01026
TIGD5	44.35466	2558.578	2350.247	3043.673	3117.88	3324.24	3414.337	2478.75	52.06293
NXF1	40.22566	547.2668	45.46639	36.91194	634.3852	35.13586	725.8586	382.7655	39.16693
CFTR	224.6598	85.87089	77.40148	110.4432	86.38936	128.6924	68.78368	86.38998	295.1029

KRT80	4386.195	5354.836	2033.501	2690.832	8862.074	2286.04	2842.219	3064.704	3297.092
RICTOR	751.4036	563.1718	326.1591	563.0844	205.2781	270.1422	202.984	191.9168	824.9102
TTBK2	62.38482	76.38664	45.25143	50.17645	40.41987	250.8405	83.59717	46.00838	82.37881
CCDC122	164.5352	114.7299	77.50574	138.2349	78.67822	93.17861	65.65412	74.80906	176.9374
FAM104B	13491.43	10675.53	9771.566	7931.616	12731.51	10740.59	9706.208	9730.967	11188.78
SPR	54.07585	52.68433	40.05349	40.8867	49.42469	60.87759	134.5082	263.8001	55.6051
AP1M2	382.8147	845.8299	322.4772	581.4374	164.4626	301.8241	194.3222	129.3472	987.0812
XIRP2	4893.653	5923.444	1458.178	1128.185	1234.502	2698.238	3025.321	5611.195	3274.971
DDHD2	1257.161	2336.583	3018.268	1339.652	2707.166	3081.913	2850.84	2812.508	559.9575
SH2D1A	387.4154	539.1906	251.5201	256.7804	129.3419	177.4672	170.4461	151.3315	606.499
ATXN10	392.7454	216.8262	211.7781	565.9376	287.6323	141.3646	148.5396	129.5513	474.2758
C1orf138	1001.73	45.45526	41.95253	37.05658	116.2424	35.2563	68.3196	38.42635	36.20533
NUP153	83.8494	38.99686	47.89818	50.55312	51.22	38.72777	47.80418	50.27153	60.03777
FLJ42393	54.25949	86.43549	189.6788	53.97718	144.7681	56.25535	47.42571	190.5035	66.54005
SNRNP200	142.0202	1625.477	1326.957	79.3856	1635.465	793.5477	1450.795	1893.492	64.30228
LONRF1	44.8887	2494.093	1927.448	675.7864	1876.294	379.3453	613.6058	788.7335	3083.7
HNMT	547.6096	336.1309	144.3558	380.9563	191.5812	241.8076	165.4355	252.9781	750.1269
PIGA	1607.132	529.5676	1970.637	105.8613	2648.776	2663.987	1911	2219.975	195.4364
MAFK	179.805	70.36683	152.7711	136.9579	69.77024	74.63609	91.01296	86.9266	166.9582
FAM43A	13106.34	13966.33	7768.894	8895.629	10091.27	9552.674	6815.799	11562.79	12545.61
KANK1	741.712	824.1947	196.9241	511.4338	195.1616	34.06458	80.55334	49.68982	42.8628
PLD5	700.235	32.47898	249.2182	471.2575	354.9214	47.24817	35.12296	32.38028	49.79545
ENTPD8	118.1141	70.80024	67.15769	72.29074	70.46601	80.47218	57.14503	65.99707	81.83608
CS	3645.605	3743.746	6631.255	4594.791	8043.723	5930.822	7372.001	8451.62	2395.449
DEFB125	250.0128	193.5688	111.5142	209.8013	90.06356	148.1825	93.53574	111.1786	416.0811
HECTD1	829.0016	406.968	827.2218	483.3761	654.8994	60.72121	442.1978	411.2415	196.6687
C7orf31	34.38321	48.02856	59.52417	34.39537	44.69288	45.49147	45.11706	33.95014	82.1171
HN1L	358.216	580.4539	107.9771	232.7248	160.3929	150.3154	396.0361	137.8579	410.2305
PAGE2B	52.38492	40.09449	41.6413	49.5645	59.35349	56.03139	38.39336	44.89637	94.29697
PDCD2L	37.78654	34.1083	268.6623	38.69083	481.8511	37.71292	232.4082	66.83754	42.79723
ICK	662.1187	106.5102	70.02321	150.7953	69.97701	66.64358	60.64025	70.43616	697.2171
C12orf53	35.86193	70.877	45.08446	75.06841	230.281	50.91019	135.76	45.87393	127.602
TLR4	93.73884	62.03161	448.1685	461.5918	116.6732	90.2794	155.4981	393.4274	90.87444
CCT5	226.3359	149.1113	1453.01	1074.587	2030.374	1355.181	2751.825	1509.249	309.8689
HAGHL	212.0114	175.9989	74.87505	164.8343	72.4957	99.31249	77.0534	78.03619	288.7641
RNF157	158.2135	85.9161	78.93932	103.3004	58.07586	82.35054	53.00858	46.68832	69.62746
PTGDR	33.31023	46.69004	37.38563	40.91841	37.85484	35.66693	65.93101	37.15557	38.56367
HCCA2	1509.45	1177.322	1173.918	703.9992	1057.859	1342.762	1081.67	916.304	72.26668
EIF2C4	983.5511	852.519	310.246	404.5202	213.0914	507.1697	353.8601	281.1219	807.0311
C14orf112	5375.508	5701.806	6378.41	6430.649	7536.657	7060.002	8731.598	8115.469	3760.684
MRPL33	297.8983	164.7642	1029.94	598.4909	887.3324	376.0741	818.3734	841.3796	404.8917
SAMD12	162.1131	139.482	74.02245	154.4586	76.95222	75.56664	55.04798	63.58886	321.9586
ZNF746	47.8164	774.2177	2973.64	915.8645	4581.596	3106.799	2986.455	3039.054	36.71531
ABHD6	138.9078	929.3176	2779.672	3376.394	4665.628	3208.711	2848	2595.012	395.7037
TCTN2	429.0288	449.6889	387.3683	242.4245	364.5092	288.8709	332.549	365.4392	1191.698
RFX2	618.9884	40.31728	96.54777	325.2999	121.971	61.05162	60.79354	35.44	31.91797
OSBP	6029.137	5721.058	6162	5650.316	7658.835	7098.901	7138.093	7467.274	4803.34

UGT1A6	569.5031	44.02258	39.12701	36.97719	37.74081	32.3421	48.1437	39.37988	35.19258
CHMP6	38.13951	49.53525	48.92675	33.66737	34.99918	42.36306	44.13562	36.02556	69.63506
POU1F1	46.03715	42.97187	38.74508	45.42443	44.01583	54.53098	35.33682	37.59153	61.09399
ATP9A	108.4783	95.76102	81.56403	73.97915	63.9506	83.41478	53.38264	61.77078	63.27519
HUS1B	443.8451	248.6397	636.1855	255.6615	231.47	240.5753	139.9929	463.6287	611.3276
C1orf66	79.79893	41.8882	48.13215	49.10917	451.4166	491.9348	275.3583	34.68969	34.46158
DSTN	42.56799	189.2031	1923.453	56.08578	3550.84	1328.386	2117.716	2113.739	87.24762
TTC5	818.116	69.63506	73.08228	79.24763	287.4373	271.7371	247.0446	58.11201	126.5097
HOXA7	76.45191	93.89498	63.95097	87.9343	60.91619	63.87208	43.38969	60.70813	68.02386
BARHL2	136.0269	94.16518	61.56092	61.09492	66.42094	118.2922	67.93729	70.94936	167.4436
DSE	41.04365	207.1517	613.5409	579.0015	1031.822	813.5399	1610.439	537.8884	44.93862
EIF2AK2	19851.02	21130.36	19438.92	24243.39	10715.84	19604.98	20321.97	11359.45	15876.17
COL5A1	2445.878	1144.489	1877.203	3101.867	1476.464	3372.159	4969.996	1957.788	1598.187
ZNF454	232.8116	159.9036	93.1734	261.2333	104.313	79.8366	79.18797	115.7183	363.2096
TEF	287.5229	338.0244	107.3349	197.1456	97.73279	135.2793	68.07795	100.3446	386.0012
CSNK1G2	11482.97	11191.95	10221.76	12478.72	8252.862	10608.87	8467.39	8670.998	11025
SPINLW1	109.5165	108.9121	59.68501	145.9229	89.87847	80.02679	67.18192	41.47949	147.4557
KDM3B	552.7279	101.5947	197.8055	300.2889	209.3985	257.0656	153.2139	144.9696	747.7938
SPTBN4	45.87183	52.70892	36.50844	62.71356	35.51862	58.90916	34.36958	52.2399	77.03973
FLJ20628	101.5678	51.53544	61.69854	73.5045	232.029	104.1316	98.64535	38.66869	71.25299
SLC35D2	1032.232	55.92524	767.7409	518.5124	854.7909	1192.327	777.1876	578.4814	54.27611
SHBG	1203.003	1963.051	576.116	1005.227	345.8391	1149.111	838.7013	918.302	1396.908
APOL2	37.71622	46.70249	37.2295	66.30782	38.23729	37.41927	39.34269	53.67879	55.76827
DLAT	810.1957	548.4391	323.185	556.9188	325.1459	161.707	212.3827	259.9257	743.0527
MLLT1	38.21515	828.8445	486.5329	50.44905	358.3076	470.2352	133.6455	336.1875	73.98822
PPHLN1	43.01997	33.20926	39.62207	301.5103	69.90552	38.83025	74.80588	39.62063	58.62085
ACPL2	62.52169	32.77785	36.15176	40.87311	45.16327	42.75402	33.49712	35.49235	40.75549
PLEKHA9	2375.597	1367.938	2440.717	3403.35	1001.795	2552.101	2696.885	2481.012	2836.172
WFDC2	50.15092	57.29964	42.33138	35.93908	44.54562	46.67027	34.00344	57.22435	36.4805
ELANE	9834.834	6611.338	1242.557	2490.555	1525.037	3241.434	1076.2	2444.546	7643.036
COL6A3	4933.202	3169.667	3524.285	3661.589	2444.331	4666.218	4239.872	2638.026	3165.556
BDNF	294.8109	182.0898	92.64754	179.1951	99.53524	139.6357	66.09465	121.4719	273.1296
C6orf150	48.34028	38.12709	751.9286	40.53112	639.2126	1019.33	735.3414	432.8928	100.578
OR4N2	176.8072	114.2315	76.2291	114.6661	74.7813	82.68868	66.57381	74.52059	255.1732
OGG1	55.11872	64.46302	592.5239	1317.794	271.3436	651.6826	165.3353	480.4179	159.0911
HYI	56.62633	65.25838	54.331	624.2839	173.0879	50.81871	41.70087	37.54505	107.0253
FAM80A	45.658	56.80804	60.93781	48.93886	65.34621	49.62757	67.80333	76.73731	32.21322
TAS2R16	53.71047	83.14693	46.26237	69.40672	54.50891	48.30283	42.44088	36.1368	37.48711
MAPK1	104.7853	2598.094	1191.618	1288.38	2566.161	1761.458	1994.351	1457.684	48.93438
SCRT2	39.87978	35.13972	42.52591	41.90968	49.49656	58.84807	42.19036	35.8865	35.99841
KLHL5	150.9429	264.8725	254.88	317.3866	147.0688	462.3154	230.9204	219.637	697.3508
CCDC87	42.29497	54.30088	48.40439	49.49942	35.15956	50.27057	40.42262	42.60433	87.99063
WDR68	1589.878	152.4576	1606.93	1823.989	2110.601	595.9714	2063.305	1697.461	345.8232
CD200	355.2814	452.8152	82.38076	180.5953	78.20248	148.9991	138.564	96.55205	450.946
ZNF451	1342.677	1210.474	512.9374	1346.393	980.0735	1257.273	850.8334	849.608	277.3895
GOLPH3L	80.80912	56.09379	271.329	51.81397	501.5501	284.4889	328.6313	283.4586	75.42372
LIG1	57.26232	101.7111	58.90385	68.09344	210.3197	59.81263	83.48481	46.33721	44.57718

TAGLN	62.01576	59.44516	44.74413	41.51752	130.7949	79.74809	44.17771	46.08889	96.81425
H2AFV	250.7364	394.1917	249.8391	432.2381	121.0376	618.6149	306.7984	271.1086	315.0446
TRPM5	36.32005	34.96475	43.93291	41.95818	46.27667	47.20406	33.58828	47.19655	38.51996
SLC6A14	66.91411	53.35243	116.2622	50.36227	259.7082	339.9039	166.8516	36.20806	70.1897
SERP1	994.2459	838.6756	231.8133	778.4648	228.4455	314.0204	189.8923	331.821	1205.753
THADA	44.82994	362.1654	52.58754	44.47797	35.68713	40.5606	86.38602	54.39408	39.82537
PKN2	54.59936	63.35046	297.7475	57.17205	174.1165	209.617	256.6873	133.891	61.57439
TEKT5	44.19038	35.72699	48.66863	34.86949	37.57019	37.85419	33.9743	44.17962	35.75889
DEFB119	50.29662	38.66411	39.45775	38.52374	48.11641	44.11641	41.44738	40.83618	42.35196
TCERG1	899.1778	578.2969	426.877	245.6154	323.8606	170.0673	215.3015	333.6974	831.4229
OPHN1	39.76302	56.67163	528.2198	39.17511	261.0932	51.01276	97.6867	52.04717	135.2664
C22orf32	2115.404	4810.027	3443.645	1908.318	4099.634	3251.826	3521.233	2997.682	5888.556
RPL34	108.8098	57.44725	98.9698	51.65358	290.3298	60.5237	391.631	406.382	54.57174
C4orf42	236.3427	421.0643	117.8532	286.5207	130.771	145.8604	113.1227	154.4051	208.4228
HOXD13	39.40964	717.3956	40.41204	40.89554	364.7386	33.7124	35.07243	363.2461	42.48064
PSG11	76.047	57.12851	46.14835	45.04306	43.55154	43.99144	35.01461	43.91159	45.82784
CCDC136	5785.056	5503.475	4262.075	3458.941	2633.652	4525.927	2805.015	4327.291	5980.473
TIMM22	7600.73	7115.607	7706.495	8316.282	8132.566	7133.202	6269.161	9615.719	8237.93
KIAA1217	1221.103	2113.795	3935.176	1568.263	5001.982	4998.521	4543.522	6957.86	2012.877
ADAR	34.0308	44.95049	34.98343	57.0806	48.77506	40.34473	42.23753	41.99528	62.33922
ATF7	154.2357	137.2845	83.59232	127.2202	59.71154	88.07098	65.80517	67.0499	292.0473
BEND6	46.19218	33.20013	310.0744	43.21869	43.46996	38.52782	374.7964	283.0605	39.9087
ZNF271	38.97234	460.2208	111.219	44.54683	265.0601	45.83433	151.8839	149.7363	66.76818
NFATC1	374.0957	229.4733	152.7312	199.2946	123.491	180.8391	124.3099	129.2222	325.42
GRRP1	276.6377	148.0432	66.02274	89.74003	82.85213	112.3404	65.27567	83.06013	111.1714
REV1	49.82856	446.3384	521.2906	913.867	1916.315	874.8382	1438.387	1156.197	85.4148
SALL3	87.08173	44.01937	63.16498	63.93126	59.61557	62.43396	87.77065	59.3765	169.4982
MAGEE2	396.3304	214.6416	95.07403	178.3482	93.34705	139.9402	64.93166	113.1577	390.1627
PCOLCE	6018.376	2470.956	768.3256	1769.417	1429.979	1626.42	1016.944	2150.009	956.1478
SORT1	163.4828	88.77014	388.9503	159.7636	719.3409	296.4304	483.6268	684.6387	46.41421
UBE2D4	49.87653	2156.111	3010.185	2374.619	2780.463	3203.325	3298.296	2503.746	3306.83
DENND2D	3130.586	65.9599	3954.288	82.10806	3893.675	1288.661	2303.716	2960.053	226.8748
ZNF180	32.83982	39.90607	49.73272	195.3801	70.5463	66.77314	47.31054	68.62076	97.09685
DDX19B	47.13718	40.62501	77.55393	360.22	109.4685	148.6124	67.26762	108.7564	731.5828
C17orf97	7537.593	8910.615	4980.098	2986.909	5894.997	4460.646	7868.968	8692.971	10957.34
LRRFIP2	1351.642	708.9833	1450.324	695.8701	1389.606	430.4142	1176.994	913.1662	44.56003
AGMAT	280.0103	88.62476	385.3439	553.8382	44.319	331.7269	482.0416	769.5652	50.39606
HIST3H3	45.61668	49.43339	42.76087	39.12091	40.44701	48.27658	38.36861	46.74131	208.8106
WDR20	37.28406	45.40945	1314.097	548.7921	1062.155	1119.531	1413.68	1137.422	1383.053
B4GALNT2	50.76347	45.11481	39.3284	45.23653	46.43989	41.61179	34.36184	49.21753	56.08725
EBF3	143.6625	519.4673	67.14412	145.2899	427.8691	319.8867	396.059	229.6897	139.5498
HCCS	80.91679	118.2551	61.06028	132.3671	57.94343	68.74531	169.9102	362.9444	147.3676
PTBP2	1173.987	541.8414	186.6414	575.9896	53.24424	162.9069	333.4439	177.5285	136.5988
NBPF3	50.45222	112.8777	61.12397	307.8862	42.29127	42.48335	109.6852	55.76728	84.1709
HIST1H3J	198.0458	267.1117	92.78369	146.4448	70.60406	113.0816	81.46559	104.3713	297.0092
UCN	92.70173	124.3526	69.38569	82.63319	73.76551	113.35	65.1815	94.52963	209.8858
KRTAP10-2	49.54496	65.33659	54.31172	69.43147	53.54133	46.38126	45.36904	51.36275	74.96877

CCDC144A	539.9938	934.7155	305.3373	1000.597	394.7741	739.5115	314.0893	775.7901	1026.851
FLJ22662	212.8982	2318.972	3712.809	57.63971	5281.119	2405.945	4632.877	3085.586	2170.239
KLF1	360.6093	215.3061	110.8029	213.6659	103.7152	157.7536	86.85999	149.9007	460.9995
IKBKG	7358.896	7757.222	10083.57	7023.555	9649.85	9942.024	10660.89	9052.412	6898.707
CCDC54	194.9801	56.34705	56.79584	53.88641	50.90614	61.0889	46.39605	44.41311	33.80273
NFIL3	183.1391	58.12513	803.2292	1478.371	1179.599	245.6937	809.9152	703.9036	315.6079
PAX3	115.0666	52.08828	57.8738	57.74208	73.37448	58.44631	49.13956	54.48751	470.0782
TASP1	54.75962	44.31224	683.3978	77.63399	47.01794	55.62658	40.59782	59.06451	43.13562
ARPC4	1184.817	297.2916	213.6396	49.6368	189.9359	298.233	231.8034	101.0913	35.38915
BTG3	57.05618	35.56977	33.9791	32.90353	135.1748	260.1189	264.5213	57.81162	32.78898
LRIG1	74.68932	56.22662	47.2659	49.67281	47.74633	384.9166	296.6383	454.302	79.12931
TUSC1	565.4716	54.22646	59.50644	43.86849	32.24037	49.68207	57.44466	48.78248	33.69287
OR2D3	48.91396	37.67897	40.74398	39.75469	36.02127	53.77245	34.83925	34.24465	63.44307
CFL2	81.72304	76.30676	49.49918	71.92313	49.21104	51.8598	43.69422	67.38215	73.78329
OPRD1	314.2672	71.47549	100.4752	144.0686	88.72456	130.8422	83.55154	138.0271	447.7269
SLC2A7	40.27557	45.63279	42.42423	54.77882	50.20816	44.17034	47.70205	39.04314	78.23618
PRELP	148.4327	76.7761	57.86568	59.3565	112.7719	68.01907	59.61718	47.16871	64.40356
ESRRB	278.0179	133.6941	106.2451	93.66985	129.4833	161.0562	80.15426	126.5921	492.9045
CPLX4	34.84694	34.34923	39.11997	33.05045	36.25604	38.44002	41.05072	33.16941	37.49338
LOC38850З	190.1604	92.56927	62.08097	80.65305	62.54426	69.97285	59.1609	67.15524	322.3494
CRKRS	34.67242	35.05355	253.0037	36.15885	154.9897	35.74391	201.0455	125.7023	41.03274
TXNDC8	61.44825	39.74497	35.23877	54.50855	43.83469	54.38154	51.50162	51.70087	42.44952
UBE2E2	2179.346	2885.411	3277.16	2750.723	5364.97	4283.535	5066.101	4123.309	1675.599
BTBD15	1534.607	1587.851	1133.474	1597.71	875.3989	1884.791	950.4426	1383.841	1501.438
GEMIN6	1407.513	199.6835	1389.334	229.3994	1957.659	921.715	1698.792	1543.215	354.3101
C1orf144	6735.445	6158.843	10078.34	7364.19	8882.392	7727.741	9597.814	12987.22	4496.245
PGPEP1	53.94297	45.45232	44.30101	50.8807	35.06885	41.8656	122.2905	45.47852	44.67084
NKRF	223.8671	342.6544	246.3588	78.54654	257.3341	94.36768	458.5215	142.1895	258.8731
RAD18	659.1555	62.42185	45.08454	45.87305	48.5747	53.30069	55.15715	47.05088	57.34792
AFMID	49.58076	73.93062	199.3829	145.7314	43.2865	64.34182	221.7715	222.7655	94.29287
FAM59A	59.01859	127.6449	59.96144	75.9342	95.72054	75.44373	65.67633	88.17787	104.6724
GABRA5	41.78045	49.85698	36.57689	47.44464	41.88504	40.43025	45.2674	47.35527	43.37309
SLC46A2	64.23799	68.05217	147.5407	83.68174	313.7553	227.9413	175.8481	198.3435	131.5332
TNIP3	9122.307	7419.927	5980.473	5071.708	622.7538	6089.386	4234.078	9279.731	10398.69
RGN	56.78286	220.9628	34.11723	36.00997	44.65786	42.12467	37.71903	39.18863	47.27805
ZNF37A	3287.834	707.4948	972.9441	1921.225	546.0445	598.9615	407.5321	935.91	1904.689
WDTC1	152.5647	94.06016	76.40282	127.3885	114.0921	71.18531	67.64227	76.86453	656.2965
FIGF	134.0499	90.88238	48.01694	95.29012	71.94984	80.10901	67.17378	48.11851	134.6712
MED10	47.42487	47.94087	3321.366	1964.797	4198.262	2502.098	5014.044	4459.898	88.67224
CMC1	37.49624	310.9266	112.1366	773.0303	120.0631	176.6278	152.8555	85.6721	42.79945
MPDZ	97.27979	92.22868	184.2968	147.6175	184.3627	205.038	87.0552	67.40814	387.1776
TAS2R42	63.17366	45.71447	46.5244	98.24236	52.71163	42.67307	45.85307	37.2798	43.87308
CLDN16	40.57317	59.25443	615.6885	64.84014	40.30771	44.96762	42.6339	46.1726	77.48233
LOC73030Z	130.3927	81.28159	52.96109	56.30425	54.99631	61.28532	43.97931	68.28556	55.33833
IFIT1	2550.766	2613.287	6577.461	4160.762	1147.767	9008.709	13415	7614.226	34.53053
SLC6A13	55.87107	51.08374	47.21807	52.85046	44.68866	78.16344	53.83828	48.69794	34.73732
CIB4	60.90348	31.67021	33.83072	36.1784	37.89695	34.8562	38.00126	48.16573	44.14501

SLC1A5	155.746	230.3738	140.4106	289.8629	141.3493	253.5329	244.2356	149.1126	514.2827
TBC1D26	98.53595	59.76605	61.29416	67.40063	63.77404	43.6963	52.39042	51.84915	175.7651
PIM3	1909.309	524.3536	240.5194	467.1926	564.3904	209.7863	129.0516	150.4256	54.51295
OR4C15	39.6235	42.94109	65.79648	64.07976	48.31549	49.43939	34.93536	61.79779	41.51221
ZNF257	42.92141	88.48635	55.68574	49.52978	53.63423	60.27811	40.76862	52.04001	46.22435
C6orf165	34.411	46.43981	35.69555	40.7161	38.05138	29.94135	40.60534	35.14024	70.10703
CCDC72	5765.554	5825.071	4777.867	5929.342	3790.466	3263.011	4067.369	3242.688	6122.707
TAZ	35.53609	51.36135	160.9061	55.89084	166.3476	45.76077	45.07247	33.90421	31.95609
DGKK	58.21061	32.33587	35.25571	32.25529	41.69239	48.09368	44.49756	36.36437	104.17
RNF128	208.1029	120.4876	79.31324	98.35567	69.75209	103.5317	61.20589	94.70914	243.7432
RPS5	160.9978	387.3232	234.1989	429.3406	252.5986	70.29342	146.6327	216.8366	69.838
UGT1A5	53.82587	39.69522	36.76605	40.51865	40.86512	46.83457	35.44035	51.78228	38.74812
H2AFZ	37.22285	34.26548	2319.409	32.85259	4897.566	2801.785	3896.917	3926.545	39.23218
RUFY2	119.4249	110.1254	75.5996	101.023	62.02289	64.48545	200.6108	65.05291	174.7228
NMNAT2	60.46088	58.98485	40.70477	77.36234	48.65753	52.31593	51.19796	57.91142	133.6429
DHRS7B	63.69929	461.8952	196.2595	61.08018	630.5657	537.8224	456.0824	1163.992	49.5174
C19orf39	45.65978	52.21728	45.79437	88.22459	51.40922	56.49114	49.7936	51.75747	79.75758
HELB	75.56685	505.848	85.82486	792.9746	62.38509	65.7774	74.21589	275.8869	49.31006
AES	296.797	138.5269	107.9465	743.8575	69.9198	106.4761	64.93771	101.1908	2300.916
NBEAL1	80.42965	35.96716	34.05016	257.6512	66.07261	40.12261	36.92977	38.55914	46.10145
SERPIND1	40.92941	39.8102	43.63543	32.86546	42.69386	38.39674	43.95652	47.80273	38.53907
CARD17	42.34203	53.47876	314.4417	52.77457	42.75191	352.443	77.61559	235.831	38.85578
FOXR1	39.77187	218.0957	113.8755	161.3975	100.992	79.07372	96.57214	134.8168	300.5721
MICB	8380.126	9740.813	10118.33	10910.9	5755.724	8360.49	7008.939	7362.522	7652.081
LANCL2	47.44037	52.7926	789.728	60.99563	818.5154	1742.665	1029.098	1169.255	123.8186
LOC51149	39.66736	51.30708	47.56683	51.96971	39.75996	44.06302	47.28302	38.65567	41.22937
C20orf108	1191.911	579.3604	766.7507	543.5769	900.9355	125.6436	964.0545	889.9916	119.9779
LOC39970(	49.262	48.62294	36.10853	45.42022	33.75712	61.69891	48.72205	40.68931	41.13665
C9orf127	41.04465	2457.442	3378.311	2348.5	3283.284	2560.896	3397.009	2374.65	872.4122
PLEKHF1	1153.23	1979.304	5666.567	3317.759	4631.825	5126.024	6803.223	8269.475	3083.107
USP13	122.7375	263.5486	129.1133	75.43375	150.3484	70.91733	231.9128	172.6646	63.95845
MAP4	13373.66	11828.42	10362.42	14166.13	8125.113	6763.854	9716.726	11799.54	13844.77
DAXX	139.9088	149.4969	457.8951	830.8306	113.063	134.9612	247.7431	295.155	283.1465
LCLAT1	784.7805	108.9408	354.7689	697.7775	1068.426	715.468	1206.346	932.568	1111.376
ARHGEF5	49.34781	363.1425	1684.001	52.82359	2070.02	1171.455	959.4212	832.1628	38.12079
PANK2	6142.405	7157.034	9982.268	9669.951	8519.539	9580.305	8450.889	9453.65	6551.178
LIX1L	43.44666	56.81398	266.8325	882.499	235.203	404.6216	326.5319	48.19906	46.17383
LMNB2	536.5822	371.483	852.1508	443.6916	1092.28	841.2117	1623.821	1049.649	51.88345
GAN	122.8283	2556.58	331.6171	1966.355	796.9962	1208.913	377.6841	563.8922	4627.966
DPT	622.8873	936.8859	1171.274	799.0118	4452.954	2440.234	1264.044	2161.751	1799.769
TMEM144	3025.97	3355.364	2615.455	2180.965	1729.919	2964.01	2447.681	2593.837	1097.11
EHF	1496.704	370.2047	324.8241	216.7542	148.4975	148.458	127.2967	170.024	1093.165
BHLHB9	65.48618	62.2106	114.6095	60.45598	248.5175	66.3761	356.2831	164.2905	54.05016
FAM127A	46.99933	559.9931	918.4828	810.3627	1888.873	1907.663	2182.155	2275.452	68.21269
TBPL1	46.22437	419.0805	1444.027	848.817	1388.336	1265.777	1438.996	886.5086	38.25381
LAGE3	60.5569	39.74359	36.95574	54.23138	534.9861	36.49366	659.9244	192.7511	47.87094
BPIL2	54.7205	69.44857	51.17038	47.05527	41.69381	35.82614	44.23598	169.9866	51.29966

PRKCABP	128.5118	1475.706	1221.294	146.5552	937.5398	1078.148	1260.952	1330.595	194.8335
KPNA2	3295.149	2881.268	1175.88	3622.483	562.9607	1473.014	1121.177	1737.974	3192.459
EIF3A	18995.11	18203.44	15705.1	18814.9	14698.24	14656.84	14968.88	15183.83	18913.29
ODF1	69.41814	35.94239	48.52797	44.6547	36.95431	48.68583	35.39758	66.88892	43.17825
PZP	37.60295	41.60841	50.01204	36.20266	341.8797	386.408	372.7703	259.7383	42.15874
OTUD4	51.72814	116.1094	451.0635	34.44324	505.0408	943.4566	450.4613	433.8085	349.5197
COPG	37.55916	544.5076	800.3292	995.2208	1204.536	769.2601	1208.963	1464.35	2004.176
PAK6	68.28896	495.5175	36.25003	38.38448	202.8357	35.81296	119.2205	180.7261	44.36276
OR52E2	48.5256	39.05548	49.21889	48.18145	54.08398	40.70915	38.33334	49.35986	36.05587
LILRB1	233.2321	133.4637	376.3329	125.9334	186.4362	81.37454	208.773	80.20272	97.4814
NR1D1	4802.626	2128.052	1499.864	3268.05	343.2726	4704.149	1389.607	1194.88	1220.034
C10orf55	358.4075	194.6996	94.10905	203.0677	115.2812	187.1714	92.9836	114.575	494.9198
CYP4F22	37.01054	961.9033	1224.292	361.7842	5717.759	573.625	374.0788	1322.266	35.63572
C11orf16	105.616	169.823	89.16706	190.1302	78.84341	81.04604	70.29979	92.83162	382.4421
ALAS2	37.60649	35.04189	35.14037	57.72182	46.10148	55.10817	33.66545	35.4968	38.07899
RAB11FIP3	5220.745	4664.312	2614.842	3749.785	2234.035	3475.103	2682.484	3293.97	4958.25
DAPK1	235.7992	210.6496	156.3339	213.5248	91.95699	118.3057	169.6226	251.7905	471.9675
DNTTIP2	950.1404	1644.156	3369.021	2584.813	3399.01	3697.769	4061.573	3612.835	200.0373
ADAM8	53.748	71.49744	49.59606	51.06319	237.993	394.8944	202.6831	204.7889	2904.984
LOXL1	39.63192	48.54502	34.23511	38.56287	42.31426	55.53053	34.19234	38.13354	55.13692
EPS15L1	61.53928	60.99536	395.3829	42.79407	492.8128	50.04706	416.3957	205.8664	38.92588
IRF1	696.19	1269.141	1026.987	918.0012	273.175	1180.42	985.0049	662.4489	537.0495
C3orf75	5743.208	7265.654	7749.662	8049.722	7695.763	7042.485	7919.763	9926.657	5565.842
NR1H4	151.9001	108.8106	65.96027	95.42791	68.88731	78.93526	56.77235	74.14719	175.3657
SLC25A41	47.14362	47.61379	37.75212	36.90179	37.98366	44.85895	41.52771	47.0192	40.23721
GNL3L	9519.746	7837.218	6526.924	8178.885	6381.46	6362.856	6237.814	5919.355	8579.136
ARL6IP1	418.933	3209.97	3606.207	2541.971	4097.873	1300.086	2904.156	1703.734	594.478
KPNA3	275.7744	185.3675	105.4809	154.7103	69.92218	127.2266	62.10377	78.30404	73.66787
GPR150	45.6423	48.47297	44.47089	43.04832	48.04406	44.94904	43.21254	35.29752	35.09853
PLCB1	148.3054	104.7153	74.451	179.4959	50.38497	97.83011	57.12733	82.25625	103.4529
DSCR5	111.5159	71.09766	59.15545	52.91057	56.66846	62.08708	49.23947	42.00335	72.81434
BIN3	137.5613	48.01243	46.88211	52.89135	40.23597	39.36853	94.23817	37.06267	58.8318
CNN2	38.02775	38.05005	179.1397	706.9123	49.27817	38.52759	96.71049	55.59321	81.49847
BRSK2	244.3399	81.14172	66.35692	66.18042	107.0291	140.5198	111.7382	159.4842	133.716
AFF4	591.4786	397.6775	638.3336	396.2816	367.1211	471.758	316.822	340.7166	391.5164
PPM1E	39.10953	76.30176	46.41696	67.85198	58.12141	76.28378	184.9505	535.3079	39.12728
NFATC2IP	67.46198	41.32355	57.85399	86.12443	52.58156	52.75327	91.22339	64.41231	45.97258
PRTFDC1	152.4985	316.3972	47.25365	70.87302	57.01184	71.68119	68.29826	50.71973	86.23522
PAPPA2	85.5717	87.1725	43.47513	40.44835	43.06772	40.04268	37.03789	46.18272	75.6404
RBPJ	3565.638	3020.139	4010.065	2440.467	2837.17	2303.871	3007.916	3834.698	161.6013
PMCHL2	77.69321	67.64674	42.50354	57.85848	62.94234	63.47237	49.80084	52.02629	117.0119
PDZK1IP1	36.3372	861.6862	862.5187	1367.833	805.9699	1339.94	652.9922	1388.686	1784.628
ADCY5	64.45355	67.45191	48.32985	52.90447	52.55481	50.7257	47.71463	45.25812	183.7717
RDX	38.18587	31.42771	53.952	50.81142	87.16514	215.0358	100.0049	48.67926	43.16828
ZNF771	3896.055	5293.489	2044.752	2404.711	1438.505	1579.975	1004.958	1481.095	6811.382
RWDD4A	425.4872	179.7007	164.8728	262.8583	201.4526	232.9488	150.4644	161.5135	289.5042
ACAT2	54.76384	55.12176	574.5519	595.8147	303.5662	77.59604	123.1128	213.0053	41.05049

C1orf215	1086.279	2326.639	1489.317	5110.62	931.2503	2624.896	1771.162	743.7583	2704.921
RABGAP1L	995.0168	1745.135	1619.228	2073.892	809.8992	1578.619	849.4891	1279.58	1124.612
H2AFJ	144.2941	267.4676	321.3818	71.83864	287.6624	218.2733	433.8171	196.5715	43.79588
NT5DC2	65.7322	49.68365	935.7615	36.92395	1465.029	41.26477	1140.074	398.707	68.87454
OR6C4	47.12842	46.04851	34.29724	53.31177	33.2452	35.26411	39.23346	43.52217	52.66982
OR5B2	70.022	65.56369	61.23453	62.31579	45.69276	56.60396	50.84224	44.34388	67.33041
GNLY	87.87151	33.1658	47.22395	39.02989	38.21686	63.41347	42.79094	41.67221	57.4042
MFAP3	68.69053	41.94284	422.7251	687.6259	266.2906	199.7242	302.8811	223.4937	61.4057
RAD50	43.44093	49.55318	49.66488	41.94803	37.02589	44.92914	47.74569	31.81524	41.70114
CA6	112.579	106.3452	78.55556	182.7826	77.38696	91.27213	61.32238	76.94064	302.6986
EAF2	44.25424	64.11754	166.2881	47.98524	262.7688	176.9057	241.6487	128.5752	75.78478
NCOA3	4265.501	4391.051	3248.302	4188.603	2140.235	3408.495	2209.421	3514.541	5653.915
APCS	135.5015	52.95568	44.50095	63.89544	60.20757	46.8699	52.41394	50.66484	119.3074
GOLPH3	75.96109	285.9086	491.9283	141.3128	506.3801	83.69895	346.9094	284.3829	1251.536
MTNR1A	43.90688	45.22014	46.33445	43.58604	37.61896	38.56299	37.00704	42.28517	45.17636
CSNK1E	73.86562	49.39375	192.3633	47.57183	124.9491	344.2474	282.3644	195.5318	51.77164
LRRC3B	63.3791	43.46305	66.31354	85.55088	57.2164	44.42107	41.53295	65.43856	75.86419
RAB20	757.5095	387.1132	185.5236	98.88388	67.09271	133.4929	158.3064	82.26962	225.302
SEBOX	59.06496	60.72387	42.14403	63.89999	50.44894	59.66607	56.89785	47.96014	97.81306
SCFD2	183.4386	171.9298	87.45685	122.4242	130.4625	114.2785	132.612	75.26275	156.4249
TMEM161/	127.3792	132.7233	273.4093	73.22282	55.39114	75.25584	299.2367	66.86592	73.10284
MAGEA10	60.2282	37.56972	46.90992	58.65234	42.6112	41.647	41.15648	37.51581	50.27552
C2orf29	13040.55	12634.49	12063.93	13143.26	12249.83	10548.54	10326.8	11958.26	14460.71
MEIG1	646.5807	276.9674	342.2048	281.976	165.8973	391.6042	163.4411	337.0296	902.2562
PDZD4	94.72222	59.90874	46.39172	66.68797	381.7271	85.3279	101.5692	168.9596	46.30466
ABL1	1129.269	55.90318	218.2882	76.65857	43.73265	47.2198	277.0546	315.2652	62.14529
ADM	6125.99	7963.583	6976.009	12211.59	8490.717	8102.794	9459.114	9936.428	6874.362
EXOSC3	44.06178	1550.525	3013.945	2712.942	2625.676	3295.478	1951.777	1933.925	1100.244
FLJ44112	49.02117	129.3116	55.14522	55.47612	104.4147	53.21929	57.16866	47.01219	81.97054
VPS4A	107.724	58.16459	935.2382	547.5981	1424.534	454.7329	1273.568	1377.358	1000.257
GAR1	125.0479	115.6428	109.4476	368.1032	104.2332	95.7355	90.67223	131.376	66.04266
MYBPC1	92.15509	71.50197	155.4776	74.58237	57.34757	246.9229	140.625	169.4966	808.1924
OR10K2	35.06425	49.50085	43.82063	44.40947	35.40226	37.61201	42.16825	43.8645	41.55887
CASC1	40.65101	39.43776	181.9288	37.53254	130.2639	375.9714	95.05141	39.056	36.24953
HSD11B1L	176.0701	143.4144	290.6959	128.0893	59.33593	77.77283	84.18431	81.0872	234.4325
CCNY	530.7755	293.4538	186.8894	541.8801	157.4143	108.0828	103.0985	208.3356	706.6201
FLJ41046	165.8405	153.9028	65.53832	70.93456	53.27528	112.9837	55.76012	52.35134	92.55719
MYBL2	375.0974	117.3039	103.5936	162.2132	94.05864	155.7388	76.96143	103.8072	427.5088
C15orf51	81.4128	69.81424	220.831	670.2927	57.00344	68.03488	163.944	257.6665	2896.643
C6orf145	2008.419	1292.684	762.3328	868.7877	261.3186	581.0624	472.9624	108.309	3014.776
FYCO1	231.7482	71.21955	70.61646	94.18196	79.71828	108.1282	105.1349	72.08361	357.9369
C6orf10	211.7668	315.0767	73.19733	139.3068	123.1837	209.0105	104.7081	108.7868	149.6282
RPL13	88.12831	45.07193	35.85406	39.257	141.6379	306.4632	92.34026	37.1846	121.3583
RBM4	39.69722	551.5488	159.0913	39.1174	742.0714	321.8246	470.2991	359.4006	41.57062
C9orf46	349.6933	195.4773	108.1177	192.0124	80.97366	140.4218	100.3898	140.9582	1003.647
TGIF2	41.01504	60.68064	379.3892	90.39423	592.3192	414.0192	482.7283	586.7213	72.05389
FBXW8	33.29871	39.27957	468.7005	57.43918	255.6942	35.6137	549.627	247.0713	35.28701

SAMD11	119.6285	77.76373	68.57713	109.0111	124.3474	81.4567	47.8864	56.15387	116.1915
GSG2	32.9721	58.04237	187.6042	40.97272	152.3817	39.93555	150.6059	372.0982	32.97806
OXGR1	139.2268	56.85648	66.81553	104.6594	67.9837	67.84811	50.41305	74.71547	54.07509
ABCC13	34.17048	67.71098	44.26225	38.01807	39.06525	43.68015	41.37091	37.9064	47.81005
LIFR	109.6055	124.0165	253.4144	118.1857	62.96233	75.27966	108.6242	192.9175	203.1591
AKAP13	1332.945	101.2606	49.09731	75.30405	174.6911	333.7491	149.9382	65.11189	47.23821
CHM	62.94387	57.1547	47.57954	78.12093	43.79742	80.17911	49.30853	57.69723	63.53098
MAP3K7IP1	277.0179	1121.186	2609.354	1345.333	3573.238	2856.118	3788.947	3412.573	330.3698
ANO8	69.80401	213.8051	132.6663	233.5874	248.8058	188.1797	154.8608	90.98218	85.97819
ELP2	142.9394	134.6466	65.21555	114.7464	199.1627	107.8245	161.9418	158.9039	104.4211
PURA	37.49563	614.3698	1315.287	331.4062	1505.292	1654.021	1124.16	1080.713	121.9616
PRR16	297.107	161.91	81.7434	175.5766	137.0853	127.5246	262.9609	265.8637	176.2799
SHCBP1	58.80113	42.22456	67.56056	82.4123	42.35598	53.61437	67.22157	46.42068	294.751
HMHB1	175.8316	111.4174	277.3701	114.0781	66.14925	94.62318	102.7516	158.6963	139.7367
ITIH5L	36.47355	58.33062	55.605	96.19302	48.17388	55.76165	46.07203	56.14287	81.89402
GPR155	678.5146	451.1533	64.40075	136.551	77.7913	263.2546	93.26465	65.08344	349.6367
CLEC4A	47.16162	50.18861	48.66218	46.28546	159.9118	63.76914	41.91611	50.87234	42.61051
CCDC84	98.36053	72.54412	41.92683	53.50568	572.2649	40.17863	262.5569	57.63734	47.82034
F5	327.9214	175.4827	84.80612	184.0468	70.27045	108.782	75.71037	115.0072	266.9008
EPHX2	89.49465	496.2328	914.5463	530.0087	466.3572	625.4774	376.0345	144.2228	108.3685
DSCR6	289.6188	273.8064	168.084	393.4557	191.3436	284.1317	129.4463	121.9735	361.9782
RNF170	35.6492	42.08925	36.88512	36.96674	194.3226	35.02833	85.39246	135.4391	39.06525
MKRN3	35.41682	50.38509	42.61446	36.28617	45.79692	54.24625	119.6667	45.39602	51.71399
CDH12	38.50392	34.386	141.3077	40.90561	42.69727	48.05027	39.55476	37.37244	37.5618
CDCA8	367.5776	199.7099	127.1086	225.7906	135.8788	169.077	106.9759	134.3318	369.2579
IL5	136.7783	112.5534	76.15108	122.1782	68.55493	102.9845	67.70223	84.06146	243.2717
C13orf39	39.35325	46.91224	36.03146	33.46338	37.71662	35.63246	32.64523	39.83718	56.04486
TM9SF1	54.52058	39.83841	211.1741	2110.067	44.93182	42.44745	888.2806	99.76557	38.22814
ALDH1A1	365.8517	220.0167	551.4169	204.3994	270.6114	156.9869	150.8077	366.3852	517.5394
CXCL3	173.8917	96.02543	68.38074	72.43926	64.24524	100.733	53.57103	66.31599	116.491
FAM186B	85.26231	614.377	268.3055	477.4439	218.682	581.6107	294.468	439.3444	144.6332
PDCD10	47.91204	956.4503	328.8193	31.66812	329.3011	544.9557	262.7547	641.2933	42.92706
SLC25A34	12229.33	13038.1	9881.026	9816.514	9784.495	11686.23	8372.896	11773.08	13661.99
MASP2	40.20891	35.52333	37.95398	48.35582	44.20935	42.11811	45.45174	47.84686	44.24849
RIPPLY2	185.0347	68.18137	66.94345	115.3478	62.31323	59.24046	56.8687	66.61622	151.4506
VIT	53.70065	66.74796	288.3658	74.92286	48.11567	41.31174	50.56041	40.17722	96.31348
CHMP1A	1123.193	967.8014	1763.805	943.4789	1731.483	1484.983	1745.306	2144.234	965.3026
TFPT	1374.183	1965.393	2043.583	2483.313	1282.516	1019.88	2504.142	2866.349	3143.697
CNTN4	37.0658	41.70586	46.92288	51.61992	37.02729	35.82448	36.66531	39.2896	42.91624
FAM71A	141.8787	64.26909	45.10468	50.26725	51.33723	64.16849	45.42302	64.82663	43.82538
ALMS1	1643.489	292.1457	245.7516	57.18446	49.21106	47.22939	119.0436	47.67365	58.64014
YAP1	84.13453	85.53964	308.5847	607.574	913.3817	554.0664	1253.651	143.9774	51.63746
BLM	791.3173	339.8695	186.929	407.615	56.20721	55.90777	74.33327	50.12546	183.254
SRPX	45.64404	37.52759	184.4952	40.65662	221.7272	40.21093	82.12206	268.7608	51.59967
RTEL1	122.5599	47.61023	48.49238	44.09295	41.2821	35.41707	219.1205	218.0317	54.36535
PLEKHM1	51.34602	811.8202	3126.476	1508.297	5404.118	2851.25	4123.951	4322.211	2395.511
PRDX2	55.12345	40.0351	187.8716	610.7927	39.73103	39.04846	47.74796	182.3612	43.70966

PGM5	86.49604	41.29243	271.6527	32.65541	508.8916	54.11509	656.5801	1412.746	860.7188
EN1	77.81978	376.4839	60.95004	133.0725	56.08693	66.41624	45.97745	47.15399	44.12625
SNORA64	3543.005	1760.251	1113.147	1409.844	407.6414	681.6755	296.0583	1331.224	2846.42
IGF1	150.3812	79.90466	54.39503	56.6115	93.44122	60.78555	94.53934	156.1647	137.4776
MAPK7	223.5518	105.8219	1672.873	116.1316	3333.222	1014.693	2089.156	2037.907	220.2056
FAM14B	313.8058	186.6647	108.2406	203.2778	97.98873	155.9552	103.7628	117.6711	169.6983
CCDC107	70.34134	48.49697	50.68147	826.2037	49.66102	60.08651	155.992	55.49771	57.82862
CMBL	58.54234	58.26497	277.6094	38.2772	246.5675	405.1979	752.4681	646.2393	44.58042
SLC38A11	350.9102	185.8494	102.3355	236.9428	115.7688	145.1411	93.14809	95.79697	517.4016
TMEM151/	66.7315	64.59911	46.64576	52.01793	49.82347	33.03921	39.00599	44.38732	122.4483
GFPT2	46.26286	36.63383	52.72973	37.91739	395.3274	47.04086	36.47358	31.00398	52.08667
RASSF2	1322.433	1419.501	1420.426	693.9534	829.224	1430.271	1026.691	1367.493	1579.383
LCE2C	574.4431	3277.878	46.49268	833.2817	2803.32	752.2838	457.9017	1177.759	2608.074
LRRC15	1308.208	413.4091	455.1657	284.9249	367.8287	656.4373	212.3775	281.9157	787.8751
EDG7	86.33775	57.30277	209.1422	1180.668	281.8701	306.0649	309.5702	403.18	63.37204
FLJ16165	927.0204	3816.344	1631.502	1774.185	2673.869	705.7253	985.9592	1783.161	4129.088
TXNDC16	129.1517	54.29502	48.85269	34.25537	48.40715	40.42558	43.13651	42.71722	46.39666
EXOG	103.2414	543.5379	673.9127	55.88148	282.2072	367.6862	307.9209	570.2775	169.5102
SUSD1	3926.135	4177.897	5662.299	3689.11	3644.538	3307.999	4562.437	5909.325	4926.019
RAC2	586.0369	523.8188	2339.655	469.8707	709.6097	2162.522	1579.828	1619.633	176.2108
AGXT	60.1162	81.6385	262.5673	61.57853	56.13688	58.63281	40.00655	160.6127	111.9208
C6orf25	123.1845	69.82265	67.67593	84.05242	68.00719	143.9792	60.46358	53.2298	65.26575
POMZP3	61.80711	68.36813	39.89878	54.6752	131.533	55.78145	67.12639	145.0358	95.2072
NMRAL1	3627.895	3859.554	3450.427	4427.715	3402.312	3269.016	3531.199	4313.635	3692.091
SLC35A5	162.9302	292.4504	315.5635	224.1871	530.2593	631.8912	492.521	478.0932	219.453
AOAH	331.7564	366.1799	443.1717	161.8593	265.6689	245.5803	516.8629	296.7046	248.2356
PCDHGB1	101.3181	59.20901	79.48428	129.0621	73.53037	69.32058	129.1168	388.0278	156.3541
KCNJ13	184.7237	136.0526	94.33494	144.1812	73.2431	76.36762	56.55891	76.13983	152.8389
UNC45A	638.5988	40.49233	164.0279	162.2757	125.8296	402.9808	181.7915	235.605	1247.431
HES2	60.13189	47.72613	47.64948	68.2924	119.2658	38.71179	107.2372	149.8566	47.08522
TRIML1	237.3071	152.2419	81.33662	175.7562	84.33089	139.2124	71.26133	83.93961	413.7202
CLPX	57.61251	64.97731	162.8259	509.5513	798.19	261.8839	307.5085	145.2018	101.6125
B3GALT2	150.0556	59.89774	107.9948	79.14823	50.13635	73.45001	72.51218	128.4303	63.13881
PLP1	100.8044	67.78008	46.94576	52.51287	52.48661	62.31413	49.95431	41.64803	93.16512
LOC90624	91.39806	44.86303	48.66187	56.82325	44.54186	59.26584	33.17226	37.39812	54.05865
KCNN4	436.8633	251.6301	125.5615	267.8812	141.54	176.6072	147.9957	151.8634	577.8849
DONSON	46.91292	546.6962	248.5333	36.92229	364.3899	549.5248	141.3018	252.591	53.49646
PEX11G	2144.455	2114.473	1342.93	1698.346	886.3752	1500.443	892.567	1508.576	2640.706
RPL41	56.73949	35.58824	55.6953	54.2379	45.39698	60.11706	39.70025	47.49587	42.333
CD79B	96.39014	105.8372	75.38914	124.6615	58.42706	73.40551	67.63467	54.8498	43.7877
RNF133	66.06688	77.19656	55.21775	52.65459	75.22152	51.07403	39.76903	49.60634	188.9232
PNMT	41.13063	47.42382	38.69257	52.62412	40.29398	50.02808	146.8273	42.25347	40.21028
SDR16C5	161.3958	268.6026	103.6766	255.0168	205.3392	149.8626	93.23022	142.496	128.8024
ZBTB46	48.84917	981.4579	2222.651	42.42157	2147.977	3256.717	3076.798	4045.412	38.87627
CMTM8	37.476	1094.036	3690.866	1823.896	3970.828	3981.738	4675.121	1049.524	3425.692
HDHD1A	258.295	113.6249	219.8837	109.9649	594.6241	339.4028	142.3663	153.1202	168.1423
EED	51.25361	276.8513	382.2261	36.68106	296.2502	294.9148	396.8562	166.5969	58.48579

GJA9	32.91872	33.78878	55.57694	50.32631	293.1287	44.00445	33.97854	38.63596	56.45063
CCL17	56.32784	35.54814	37.76395	33.26637	31.5785	38.84268	32.3548	35.575	37.6032
TNFRSF4	174.6497	130.8818	75.57384	106.7061	66.63601	67.48312	61.94616	59.71562	342.9557
C10orf6	821.8748	1350.621	2349.633	1346.573	1974.214	2637.856	2355.697	1617.157	53.45677
MFSD6	374.6296	683.5886	1170.33	772.3839	2143.989	1393.854	1122.66	1116.458	1068.898
AVEN	802.0205	1004.713	698.4066	1380.598	653.6953	710.4546	1082.284	993.028	1149.763
MLANA	35.5847	49.58636	321.4684	41.78796	39.86222	45.6171	57.84899	34.94054	45.9219
CALCR	239.4095	91.26719	56.4153	151.3593	68.2511	88.93938	84.79062	84.15457	137.1205
GPR35	72.22285	42.03378	227.5225	40.68733	56.54148	293.4652	226.7377	32.69809	44.88869
OR1F1	40.8755	37.72551	39.89146	49.62407	60.60315	37.30133	38.25867	43.76075	85.78342
ZNF385D	3165.078	1608.94	1671.049	681.491	1661.729	1080.676	1551.303	1764	1142.068
TMC2	132.5118	94.16125	65.04729	94.6208	64.08134	98.75719	66.99901	77.6433	187.025
TUBB1	61.66124	100.6773	34.46158	69.94844	71.10108	40.03399	205.1646	38.42704	106.4891
HES1	2051.047	1102.885	1247.233	2053.087	1530.917	1024.394	1364.447	1182.504	55.50455
CNTN5	41.89047	48.05594	44.98383	58.14654	50.06344	50.83002	49.60986	44.99372	55.52039
TTC25	1170.879	544.3087	469.5732	131.5758	530.8708	330.6938	550.3533	672.3084	1819.408
TMEM31	135.9206	84.17342	73.08594	130.6474	152.0109	68.41936	94.69759	47.6198	74.14294
NOS1AP	1809.648	4375.602	394.7307	3255.177	1202.187	2830.312	342.2273	1495.607	3306.99
MCHR2	39.35685	54.29683	36.54954	33.18435	38.36952	50.79734	44.00736	44.10139	62.10351
SCEL	1506.081	63.32347	644.8398	481.8175	677.7967	278.0257	52.3451	216.1264	1353.001
CLDND1	38.0874	36.3511	608.4627	34.87933	1614.747	655.0748	1297.109	954.0775	431.8583
MYEF2	120.1072	70.19548	67.91379	70.04656	74.68213	66.75897	65.77047	46.18886	102.918
KIFC3	100.5288	53.48856	35.80872	71.24569	73.29648	61.98298	56.23294	75.45041	180.9568
CNTNAP5	423.6574	236.1776	118.6115	256.5749	131.352	199.854	126.0375	132.8477	598.4444
PSMC1	12581.43	11735.99	10109.88	11952.36	10314.79	9642.307	10863.55	14727.1	12242.29
LGR4	103.8768	91.62683	201.591	75.89749	678.6897	51.72314	156.9817	48.87913	88.86826
LOC73041:	206.0539	94.53783	52.77504	65.45889	280.8598	59.50341	56.56306	61.66072	127.7967
HS3ST6	113.3341	108.0569	58.60519	127.4385	64.43915	70.55092	62.57018	77.27605	121.4501
VPS37B	120.781	66.6702	1078.194	534.7042	1913.612	388.0575	1262.747	795.3245	163.1639
CDC2	60.60288	114.3208	67.87955	67.03362	61.47326	53.83974	47.96362	71.59881	230.4378
TBC1D10C	47.17143	1702.446	1733.851	39.11929	1041.614	1035.458	1242.128	1008.352	2330.167
RGMB	383.0638	502.4233	56.68732	151.9029	63.58333	291.2264	96.40565	74.88407	1135.06
APLP2	4639.889	3478.287	2469.114	5183.377	1916.638	4362.252	1512.167	2713.216	4533.948
PAPLN	1370.067	1758.171	2085.655	1346.428	2587.42	1581.743	2633.256	1851.913	67.85049
PFN2	53.01047	35.75148	40.04329	39.0326	38.59329	61.7728	43.23612	68.56861	40.3628
BCL3	177.5311	414.4022	2556.603	1456.573	2351.526	1015.413	2416.135	1506.609	1074.159
ATP8B1	38.66622	77.00115	48.6255	49.23197	49.4562	53.99451	48.38346	64.98658	40.97034
PARD3	159.9166	73.07435	491.3684	52.36968	621.2839	61.01008	300.4539	324.2578	54.26477
SLC39A3	619.8102	526.3112	166.7126	572.6622	55.78614	203.9346	93.37268	128.6335	84.5374
PAPD5	43.16653	49.65185	832.8171	403.0967	1011.651	163.1819	820.8681	888.4034	47.86163
PLA2G2E	132.052	98.41821	55.42854	92.4549	59.3007	64.94771	58.88791	43.55891	274.7574
SKI	245.1661	160.484	608.731	1369.457	618.6395	433.1002	617.3809	595.2043	216.8325
L3MBTL2	147.1177	55.91475	40.03463	284.8376	105.5674	42.78578	56.0704	53.0948	99.55147
C8A	53.70123	83.13916	64.0042	79.66555	55.91608	73.85878	47.13022	52.57605	43.18795
C17orf73	146.1475	50.77894	58.18652	121.1775	39.43355	113.904	53.14011	66.70879	144.9823
DUOXA1	13945.09	14263.62	12541.21	11018.68	14082.28	10250.1	11898.96	10982.65	13241.16
MARVELD3	1186.049	1911.461	1514.683	3170.624	2798.467	3573.727	5432.135	2155.689	973.7211

MTMR8	119.4425	57.80312	57.97026	88.64696	77.40809	122.9232	50.4272	103.6671	131.7271
LETM1	106.6584	347.593	148.6431	149.3165	292.6624	185.3017	221.7291	465.5352	97.04175
FBXO5	3198.09	1307.643	1407.273	3121.11	1643.176	1789.132	1097.169	1251.368	2141.876
SUOX	125.4264	53.41993	61.01643	77.08669	46.4586	46.52459	58.82687	46.23338	66.0332
MIPOL1	64.23443	68.07592	49.02236	94.12476	61.59816	49.29138	117.4825	171.3725	59.91741
BUD31	688.0028	561.7512	553.5696	497.5076	679.2402	773.6269	583.2084	708.2462	891.2642
ZNF474	35.38004	37.34176	44.6947	65.24461	91.24835	62.64269	49.33903	50.35043	88.28577
APOBEC3F	203.1156	1268.004	2298.476	1353.797	861.4219	1807.703	1840.572	1992.374	385.0865
GSX2	34.02399	46.13757	34.05326	33.50115	49.63969	33.94123	36.612	33.91486	37.22743
GZMK	42.59695	52.95855	166.924	80.76095	59.8045	42.61107	69.67648	48.51548	39.16549
RC3H2	385.985	1211.64	1758.211	803.7706	747.5659	1743.65	1592.437	2021.268	1235.851
C2	82.5363	124.6519	420.5539	70.75459	251.5806	645.6985	387.5897	242.7215	124.8099
NAPRT1	16960.15	17942.29	14464.56	17928.2	11803.89	11513.25	10266.28	14615.15	16492.29
ENO1	7308.115	8151.983	17645.76	11877.39	20585.29	11082.65	17641.67	15892.9	5526.961
RNF20	1929.907	2962.49	4411.502	2547.14	4671.789	4158.129	4506.854	5146.377	3127.664
MAPK8IP2	60.4744	59.05482	49.10361	52.43592	35.67614	45.93781	46.0158	57.36295	80.5181
ZNF673	498.7189	274.6614	165.263	301.6458	193.7205	237.3969	159.6969	142.5497	518.0201
PCSK1	40.70425	45.95129	50.55508	36.57367	38.07928	57.36767	34.85182	35.84546	38.21998
GATAD1	84.88818	128.3148	139.317	278.9927	260.7599	158.3033	200.5091	194.9727	94.09545
MAPT	64.86743	68.52805	281.3734	76.84106	52.09465	554.0333	35.68113	267.9356	50.44754
RNF145	53.26557	282.6882	202.3355	68.96754	98.57336	70.68649	80.01059	217.022	91.16341
SLAMF7	383.5969	166.8689	121.5267	165.0734	107.5464	115.7819	85.81184	113.2483	300.9064
KRTAP10-1	50.58109	42.96737	32.27798	55.99854	49.33026	41.64852	44.60986	39.7145	44.3995
IFI16	1002.982	344.2977	965.087	667.2847	399.9818	281.1945	1097.747	598.3139	54.02241
LOC283932	830.5714	48.65987	602.1174	534.8801	489.9346	78.01595	498.8575	676.6835	50.31601
TMEM97	6515.207	5694.494	4706.634	7450.75	4250.942	4768.11	4399.74	4400.403	6337.889
ANKRD39	162.2513	305.5459	358.2834	131.5483	316.9696	108.9751	443.6529	301.8241	83.50242
GLIS2	50.99948	41.65997	34.89404	1105.158	31.93781	508.8308	36.8148	54.98202	50.9626
C1orf111	63.42712	41.58473	56.50311	68.68569	53.50497	68.49179	51.58818	58.74513	55.30342
CDC25A	56.78941	42.4819	308.5919	36.07788	112.5778	45.89735	39.97305	52.46607	44.60429
CRYAA	281.9166	197.1838	96.49241	212.4743	85.51418	127.6679	72.0043	93.76118	367.6322
HIGD2B	51.68606	50.60458	36.79327	85.78108	46.83235	44.86343	48.46819	48.40829	57.91784
CDK5RAP1	39.41286	42.57722	1833.931	656.2277	2225.077	675.8291	2628.887	2127.375	1908.976
NRAP	62.14532	227.8613	70.04818	35.4793	57.22981	171.9577	115.9747	98.61479	42.4909
OPLAH	12858.84	16785.63	11755.12	22669.67	13869.78	13778.56	16983.56	9139.009	15119.36
IFITM5	37.0862	46.40898	35.83797	37.35221	35.46014	33.4366	37.14389	36.83425	56.79093
ZBTB17	170.0664	109.5382	167.1556	95.76302	70.86027	106.9392	237.1282	61.66248	304.6259
CACNA2D2	57.89144	37.59195	41.75578	49.47894	100.0463	60.86536	59.46709	108.6994	55.35642
DUX1	58.18354	62.35196	59.62214	61.10013	42.74108	61.04488	56.42645	69.54999	53.9638
FLJ25006	1605.878	785.4916	257.4436	1630.395	46.76812	45.94741	285.8448	38.64517	48.34122
MMP19	213.1763	521.3431	176.8868	171.6283	66.33392	109.3595	64.73615	81.13011	873.4079
C22orf42	72.5449	64.22958	62.84988	77.97813	55.55811	53.35255	43.56444	44.36516	44.10051
YPEL5	993.7938	275.5394	859.2165	580.6995	1774.654	1237.426	1241.392	1232.645	1502.154
DNAJC21	1926.038	1519.063	892.1396	58.38411	472.931	61.6003	156.3778	385.7704	1635.684
KCNB1	49.40404	427.82	129.4274	48.18864	204.897	36.23204	107.0357	305.2486	62.32466
B3GNT2	69.15075	363.506	408.6875	64.11709	443.4853	685.3124	375.9232	388.6184	62.25664
SLC9A10	32.7005	34.20588	35.28081	49.13942	35.81823	35.76229	102.3384	34.65072	34.65943

ARC	129.5517	223.2756	77.06786	148.645	227.6151	103.0331	125.7566	111.3595	437.9471
MARVELD2	42.57684	533.3029	2093.049	73.62743	2214.22	762.3299	1059.594	1736.121	63.04634
VPS37A	683.6196	336.9795	392.0616	332.3555	480.0125	237.9584	285.5345	461.7939	897.734
NOTCH4	47.37246	194.0701	198.9623	56.28436	210.3254	716.8724	384.1456	561.9722	76.38635
WDR8	63.76154	90.6487	64.2334	508.4541	216.4312	368.9913	222.9675	202.1077	51.76922
CREB3L4	42.30024	38.99108	39.72679	44.78412	38.36252	37.72493	35.99952	37.62419	37.23531
SPRR1B	10124.48	7981.58	4252.349	6168.253	4145.823	4149.333	2510.712	3841.306	10759.78
ZNF331	1854.162	989.2942	2940.804	1579.721	2177.092	2919.889	2690.333	1273.558	448.1297
C6orf15	8743.171	15914.9	872.5861	6715.275	11437.71	10277.55	2455.14	6164.098	4216.823
CELSR2	95.9541	82.38799	347.3593	370.8407	1181.319	247.8456	859.485	927.1033	386.5209
ATN1	59.73047	87.66547	35.47377	40.54135	36.48528	46.15637	45.6055	53.91278	33.49583
GMDS	1790.361	2617.392	4843.997	2870.049	4548.196	4954.601	4505.2	3334.001	1824.639
SCG3	53.38505	59.78708	50.29317	54.62566	43.77601	49.6354	44.80676	64.50583	117.9386
TRIM24	69.54688	252.5607	191.5284	49.60955	285.8428	55.41619	211.0065	174.4446	149.0866
WDR3	74.97827	51.74684	193.6215	279.4759	333.8211	238.3432	339.1706	288.492	148.7549
ASB5	38.03777	436.2561	476.6835	1839.175	333.7939	529.9531	863.374	994.8686	2192.41
SLC5A5	41.34528	74.21022	38.46863	37.65207	32.47074	37.26775	33.04146	44.84703	64.77601
PCDHGA9	158.1501	67.17816	75.68198	105.792	60.48058	50.3245	55.72947	60.8154	144.4797
RGS20	40.48715	56.1481	48.17836	39.5331	38.09109	54.01991	39.60741	38.66486	67.31087
GYS1	158.6332	111.5987	124.5582	247.2639	158.6067	176.9993	128.886	145.547	211.5123
PODXL	238.3888	142.6861	61.37223	68.9581	81.53326	65.81762	55.71537	50.2383	206.0182
GPM6A	126.9895	77.98965	59.97252	63.39326	50.09687	45.60612	48.76546	85.98747	45.85692
NUDT5	142.4192	150.7139	304.2636	173.693	411.1166	97.26385	362.8695	126.9376	181.7114
PHF8	336.9843	188.4043	93.85103	198.6389	88.96742	103.3836	86.76232	115.2833	292.7736
MAP1LC3C	194.5934	113.8811	89.39799	137.7292	70.55473	118.5759	104.7334	68.45653	363.2152
FSBP	98.4297	47.14214	37.40911	53.85291	44.4351	51.05907	57.12885	104.1951	53.0976
OR7A5	64.46935	34.10691	36.88678	39.74836	37.34745	45.72781	73.42513	38.31153	61.29885
ZNF233	116.4179	49.32743	45.25742	37.36916	52.13295	45.36961	74.90726	54.97947	44.87872
NRN1L	148.4526	70.25358	71.28439	106.7489	65.2188	79.14444	54.34359	94.12097	230.3393
ZNF319	77.81496	114.7281	295.6403	505.2378	198.8892	487.0866	421.5619	170.6487	118.9341
TRAPPC9	77.68332	86.19432	2021.602	1225.941	965.2399	566.6262	1321.291	1498.146	49.97403
ADAM11	45.19435	56.70709	32.2937	42.36703	34.13665	49.26673	37.8494	44.9785	37.0356
STK4	1036.137	2533.666	3405.99	2749.723	3003.447	3738.462	3451.185	2243.468	809.0951
ANXA4	52.89511	44.81653	67.26411	258.2567	62.72467	35.21238	55.9319	41.09118	45.63921
MRPS27	668.4801	437.3737	285.8506	495.1028	254.5849	347.3715	269.7295	368.7409	883.2673
PLEKHN1	2453.929	1709.858	1170.673	1491.271	828.4408	393.547	650.7985	676.541	3308.449
VN1R2	60.15249	43.13922	55.66027	96.01316	57.07459	43.62057	51.22432	54.37524	52.23344
EMILIN1	33.12134	57.15908	35.97229	35.55262	152.3253	35.56511	459.6195	51.26011	37.50114
UEVLD	207.5064	67.8582	211.4044	404.1785	487.3295	279.5785	293.8276	188.8819	625.8454
MNS1	38.98086	43.75066	241.8294	38.14252	144.4389	37.76125	50.59724	43.98112	34.03332
UBE2T	76.21523	68.21619	169.0033	546.2985	154.213	60.99454	537.3082	316.8763	73.01288
CCDC78	38.99546	54.10326	48.83698	65.24715	53.17838	51.47899	49.82259	38.40496	42.7029
ARHGAP26	55.9515	745.9487	45.80309	58.34389	44.95586	51.11234	60.80788	49.87551	88.55454
SYNPO	442.3688	82.0033	68.30444	234.3633	75.67626	106.3672	59.6045	57.34404	39.31046
PRAM1	47.04686	59.69637	50.75348	42.68676	34.14426	36.077	115.2593	202.7389	71.83603
COCH	69.89436	38.2187	36.66024	47.99592	178.0559	37.49427	113.2356	38.63611	62.6333
ATP6V1A	2738.233	3025.685	3333.548	2813.505	4206.978	3173.253	3237.27	2665.417	2582.438

OR6C6	70.08788	39.92129	49.75375	62.74435	50.00432	42.18382	43.22171	34.19247	45.16544
CHMP4C	98.69076	49.52253	68.99399	83.75974	218.2622	169.8953	156.5687	94.18205	138.184
C14orf169	1647.17	1797.882	1464.46	1694.54	1034.996	1667.375	923.4864	940.1452	1253.879
MS4A4A	37.193	48.45758	45.45775	37.84892	46.38264	46.63328	30.51224	38.80159	38.36227
OR7A10	59.54511	81.40059	40.47454	35.01461	37.5493	37.71056	37.35357	50.08315	41.48005
TIMP1	20132.68	13510.43	17930.41	17209.3	13869.4	19849.02	18913.07	17030.18	14472.88
CD96	43.51567	888.1345	1372.129	53.03025	216.9771	841.1026	596.7906	825.9083	51.09806
CSN2	122.6577	55.20336	49.77864	39.37318	60.9343	49.96781	44.94729	38.26403	63.92912
PRDX5	726.8676	832.7132	1921.697	602.3307	2756.225	1001.451	3204.64	2785.65	795.6635
SSBP2	713.9415	1116.429	1353.754	903.7075	947.517	1596.185	556.6837	917.4662	929.7044
GMFG	7438.445	6845.129	5801.206	4852.501	2979.962	5234.236	3351.551	3375.289	8101.632
KCNE1L	49.91402	326.5848	155.6891	56.69285	56.4357	44.64014	140.1091	44.56579	79.03402
ZP4	77.88852	57.47341	33.92048	41.62731	61.83046	42.74835	41.85486	44.95782	38.57219
SPATS1	88.94469	42.308	53.86861	38.75982	43.51225	42.78959	35.90552	62.10404	47.45435
MGC57359	46.0119	36.29901	51.17943	46.61156	40.43698	42.95887	39.60948	38.18801	50.8236
C16orf70	34.19802	883.8766	42.99921	43.69133	611.7598	45.40386	150.1822	338.6812	39.6992
GAS2L2	52.95227	103.6609	82.15781	86.57899	58.40049	69.52458	58.9897	47.99949	64.49157
C12orf42	40.09778	37.12142	101.4138	33.51582	34.34352	38.19851	32.09144	33.21041	67.02497
MGC50722	72.39351	75.57462	298.5732	96.6242	53.93071	70.26018	52.95525	56.47818	131.4005
ADCYAP1R	43.21695	52.29545	46.05847	49.21661	46.51849	51.36817	47.51581	48.40962	43.19218
PLTP	767.5212	118.9964	221.3853	113.3901	119.5926	252.7376	221.6122	112.0408	1020.13
FOXR2	60.01925	50.72307	36.34892	120.9992	42.02584	52.98796	48.50401	68.9931	59.9327
PSMD4	61.95153	40.36653	49.48194	68.33205	45.07442	52.35136	45.72978	39.80732	56.42509
SKP1A	2644.613	4103.485	5026.713	2535.576	5863.64	4391.8	5079.404	4870.798	4604.46
NFE2	9730.516	6188.783	6354.586	10844.73	2675.485	7983.45	8176.85	6304.65	7341.992
KEAP1	40.3475	529.8136	172.1421	34.23658	40.78896	39.73408	102.6404	266.1604	35.84753
DEFB135	37.74646	38.72242	40.73246	32.92063	37.4362	34.48734	35.68234	34.67627	55.76457
GPBP1	166.313	455.9674	215.1142	79.90347	131.0855	63.05428	135.0592	160.8868	69.4522
C9orf40	56.15886	79.52469	688.7264	47.23134	916.3587	953.9576	972.858	1043.7	90.22141
PIWIL2	50.35551	61.2197	35.87778	973.407	40.78471	44.47636	39.01973	34.36277	67.6832
C17orf70	19345.22	19482.81	16663.65	18081.22	13754.03	18834.76	16122.01	18179.71	21576.93
SLC24A2	66.12519	69.26625	53.48178	57.48424	46.06863	63.60709	49.00816	72.49037	165.743
ANKRD10	40.74281	33.68179	206.9774	53.39597	240.1476	249.1824	154.3144	55.44175	95.10123
MED24	84.54781	80.18131	710.369	510.6682	3224.779	51.28662	1935.671	3547.619	42.63643
RNF7	2937.446	4194.714	8026.953	5522.728	12138.92	8715.549	11011.75	12060.39	3667.855
ENOPH1	37.51886	35.36783	596.9087	39.87856	1111.86	35.50859	1226.217	417.4185	48.50922
ALG3	2275.971	3699.936	4641.952	3308.587	4253.235	4086.823	5105.326	7106.141	2925.989
COBL	94.20198	85.80272	65.26517	118.3164	95.27996	67.87064	81.25536	45.09409	357.467
MYB	710.1204	897.6422	2432.383	354.1218	1262.665	1990.557	1319.347	1224.856	1316.243
ANXA2	152.1858	1257.679	77.19342	1367.004	474.6666	880.8881	60.71457	199.6984	75.33708
MXD3	43.44297	60.17913	33.32484	38.68993	44.11443	49.82584	51.17861	34.75629	46.14851
EDEM2	529.6457	454.0378	757.4608	813.4689	736.0387	300.2836	823.2676	638.8865	1278.093
SCYL2	1857.094	2824.218	3171.531	3047.656	3781.716	3489.856	3550.232	2635.93	1193.339
FLJ20920	49.64415	66.49922	580.9812	751.8074	269.2652	364.5853	393.6958	189.1364	47.66923
OR5AN1	77.11266	61.17223	42.74096	49.48472	48.95464	53.66489	40.51903	41.93473	77.12726
MAP1LC3A	38.72849	41.04365	44.62085	55.77513	41.42324	63.65337	46.24038	42.04822	52.75137
KCNK12	3506.198	1935.017	1011.392	491.084	692.8633	651.2048	508.549	684.1152	1586.468

ANKRD17	108.7913	1026.647	1584.417	1251.363	2456.916	2923.677	2470.968	741.6843	193.644
RSBN1L	609.1302	38.98646	104.0984	39.64871	59.35807	36.51243	114.7469	42.6157	38.54077
TMEM115	20039.91	19207.99	15526.08	19612.02	17062.99	16517.26	20312.96	14028.72	19085.63
CPXM1	673.0282	455.6403	280.9643	544.6905	203.2374	349.0935	286.3361	390.015	838.3098
FAM19A5	1773.544	46.41191	40.04081	37.69497	160.4336	46.97508	46.84874	49.48561	62.44452
EXPH5	68.67798	165.2451	117.9991	251.7352	144.7545	130.2783	73.76304	101.9188	121.4011
CCDC135	402.3858	231.8205	121.7595	238.2173	106.5046	166.6179	103.847	146.4211	540.9005
GCNT3	2245.032	1131.959	3315.502	34.34027	2789.959	5355.899	2143.624	2296.144	2918.913
FOXO4	11767.29	10927.16	11131.36	8103.421	10415.1	11347.52	9775.73	9857.591	11269.21
RCAN1	163.0359	109.4634	144.6797	434.3135	299.7989	112.9782	87.30208	89.93002	1532.262
LRRC8B	50.65436	53.97526	49.55437	70.62875	54.9859	65.76996	87.23013	65.29945	208.2807
LIPK	65.61229	56.55609	51.31189	65.07262	59.77025	49.07489	50.19406	56.58628	74.57256
AVPR1A	34.42928	34.25759	161.3139	57.37592	59.86887	36.60106	124.767	64.72919	39.559
TMPRSS11/	556.2118	51.60477	134.6902	46.78223	249.2841	220.1366	116.0244	46.85091	58.1871
HIST1H3C	37.83556	795.7302	226.3388	41.60841	178.3275	33.97055	100.6787	34.12059	36.90268
KCNMB3	293.5496	476.9125	78.47717	794.6177	489.0787	95.74815	119.5248	259.3783	327.5367
SH3YL1	9686.388	8466.8	7782.805	9353.03	6697.047	9256.298	6912.797	6753.997	10073.03
PPP1R3B	38.3152	36.9113	361.5172	43.56388	35.41352	35.75942	34.46021	45.22784	50.37401
RPL10	50.56826	71.74617	34.8889	37.05863	37.58183	31.60887	38.32764	34.25421	34.51413
ZNF605	99.70147	55.54877	75.62099	64.68063	94.71246	52.52243	100.1012	193.2458	281.4366
WNT10B	47.01215	43.16088	36.50899	41.8165	247.0984	38.06103	152.1835	36.11718	49.57522
ANKRD46	55.59299	36.4213	255.6077	63.38744	526.8884	45.23367	434.4789	413.1587	73.57367
CYBRD1	55.55358	582.4926	255.7319	89.63113	331.2965	75.85471	215.2362	295.6944	209.7389
RAB11A	220.3862	1814.473	2379.923	1692.795	3347.966	2981.43	3002.989	1743.786	155.9194
DUSP9	104.4868	38.73789	42.90733	43.68051	42.78268	45.16845	36.92761	46.77015	60.48767
DDX50	3370.109	5191.875	4267.522	7357.462	3061.512	3609.033	3053.669	4152.535	7999.995
DOCK3	216.0302	66.4009	111.5335	269.3694	130.6026	220.0434	79.2738	46.3534	102.1112
NCAPH2	34.25443	50.86406	318.1432	587.0134	114.2642	805.0366	272.8834	37.00142	58.03099
CKLF	45.53861	48.04704	157.0247	908.295	156.9462	38.3243	209.5789	303.3969	62.48501
CUTA	229.285	174.7643	2259.956	1510.795	2460.041	1325.749	3187.427	3150.506	401.7964
GJD2	105.2439	98.55132	66.74636	109.3324	69.95254	93.65378	64.78951	61.12135	121.0306
RPL39L	278.6734	560.1327	84.67305	108.4861	67.27105	122.569	64.2059	189.8698	224.8916
DCDC2	43.32779	39.43823	205.2846	57.9148	43.45449	37.43437	38.71879	35.74001	32.37476
SKIP	157.9242	89.8037	72.5276	125.2701	75.94828	114.3761	63.32901	83.14891	253.0805
FAM113B	11075.01	12181.13	11475.98	12500.02	7216.007	10527.09	8657.482	9181.737	11657.54
PSMD2	509.3988	327.2106	1178.024	326.9456	1929.761	679.7852	1627.515	1700.984	994.8569
KIF20B	4136.116	3286.379	3163.179	4119.132	1625.021	3929.903	2283.046	3099.813	2144.552
LRRC52	53.00606	35.09158	36.24798	37.37596	44.88127	47.19007	31.91602	37.24826	33.46471
DKFZp434I	47.93805	55.23858	47.49945	60.30467	53.93775	59.48094	55.03524	48.38601	100.4479
PDC	39.41428	53.27867	51.69628	42.50731	42.53119	44.5867	51.05849	42.5909	52.44466
CBLN1	45.917	69.93273	161.6267	53.91941	41.55693	670.5647	132.1509	45.15743	58.53125
GOLGA8A	1866.111	983.8323	517.7238	74.26231	599.7726	517.6277	465.9724	226.3588	69.93634
HS3ST2	80.00054	75.12843	43.44418	62.81924	53.43737	59.91747	51.64944	39.39428	152.9148
LGTN	59.42803	52.21854	3039.156	1165.075	3782.046	1763.502	3986.874	2759.578	39.99644
KDELC1	125.3222	52.75326	147.7214	205.8857	127.2575	193.9706	188.6216	150.6568	238.7507
ZNF696	1104.872	1306.144	1019.548	1462.802	1682.791	1718.504	1935.095	2356.641	1765.662
CEACAM6	8499.377	7108.769	10115.18	5393.591	7880.061	9479.632	9696.59	5549.185	8977.864

MAP6	54.85373	69.90382	40.58734	34.18296	35.27028	34.4838	35.5268	39.19917	52.12316
AKAP1	606.137	1481.285	1406.547	1251.152	976.1469	806.1902	577.1818	999.5925	975.4772
CARD9	6400.982	4153.934	5444.108	2795.781	2642.945	5658.907	4685.799	7741.441	4737.607
NODAL	729.6824	656.9366	450.0021	57.72675	504.8037	669.2429	52.50333	152.9684	75.24194
S100A3	13036.7	11965.86	9691.287	17840.32	6554.603	11638.07	10909.98	12365.29	15339.5
ZW10	1184.068	1083.049	1532.847	1506.15	1715.785	1425.836	1211.293	1509.91	37.06438
SF1	36.94987	833.9569	40.52648	859.254	188.0979	473.3878	108.7613	37.66775	45.153
FAM174A	464.2873	284.1347	420.1682	790.1799	429.7067	208.2472	277.3468	200.4012	2090.783
METRN	69.2326	36.73151	41.09013	32.92459	62.27028	36.13466	32.6468	39.10131	39.81972
ZNF829	147.8698	436.1229	62.43128	144.1631	64.10835	226.7515	99.50571	75.14555	117.5171
BFSP1	59.72216	279.4846	58.53098	286.9191	45.94614	46.40294	52.67854	120.0877	41.28954
PNLIPRP2	44.06799	34.34767	36.37945	33.38366	38.02887	36.66832	36.19669	38.63985	38.11369
C6orf81	105.091	89.3811	176.0065	102.0964	144.8067	51.26775	156.6602	104.7916	48.55123
XDH	337.4371	341.0684	277.5087	221.6315	181.2163	312.1725	272.9019	395.5271	598.2618
TIA1	1333.9	905.5496	756.446	1594.912	700.4954	949.9761	757.9921	859.4045	1423.621
NVL	67.40931	373.3845	1261.367	408.1112	1169.656	929.9874	1364.336	1424.453	58.05956
AIFM2	191.952	126.5972	75.46195	104.5587	89.10687	85.24916	90.60165	86.38185	103.1748
FALZ	1294.871	61.78677	45.60439	43.56055	182.6278	41.14721	37.5849	55.20794	67.6308
PDZD7	34.23536	38.28833	39.82838	37.06546	44.62216	34.7485	48.05019	32.61911	35.71969
SASH1	968.6747	53.88937	47.50278	38.45212	180.1152	48.35292	45.66526	41.90682	1487.367
RPS27L	98.51129	99.40473	1568.941	840.9629	2104.536	1269.182	3064.929	2215.644	93.2246
RAB40C	12954.59	10836.27	8491.255	11554.36	10032.31	9928.85	8263.6	11207.18	12127.99
ABHD8	5934.134	4400.309	1993.822	7448.426	1637.653	3208.349	2134.832	2499.945	5008.216
TRIM47	52.74701	1649.509	2111.518	815.2135	3683.918	1590.365	3855.118	2615.278	1197.516
BPNT1	77.45141	142.5942	1435.638	1038.104	410.9646	2119.949	886.4388	1585.711	40.22303
MAP7	83.12725	289.3911	882.2268	492.0283	1123.658	578.2623	718.3229	878.4872	68.97713
GAST	56.58348	40.2896	49.27858	51.37099	44.42167	51.35213	50.40029	43.16534	37.97008
DKK4	39.76497	31.09752	430.3621	35.814	250.932	35.1796	438.3952	168.573	50.02837
C14orf129	102.7744	69.08549	128.9979	392.4552	130.8856	53.32001	79.42643	53.90792	152.024
HADHA	13493.02	15059.53	10782.34	15612.38	9490.059	10618.38	9655.486	9795.528	15428.32
LRRC41	733.2861	710.1787	790.8901	880.0071	600.0256	797.9687	773.6467	618.5828	659.1458
SBSN	11495.63	8992.781	25459.4	9580.227	29082.72	12121.79	25600.58	20059.2	7432.095
ERGIC2	50.3471	885.814	2467.663	1805.927	3189.077	1928.967	3324.91	3142.169	120.1551
IRS4	348.4024	189.3382	100.2324	199.2905	91.5056	144.2803	86.29406	111.7691	429.1388
KCNK7	94.75301	70.31001	46.80863	95.16502	54.77849	99.50079	43.35593	55.98219	63.08813
AHCYL2	35.87035	35.85321	37.09002	37.51783	175.819	41.61203	159.5728	266.791	42.37049
ZNF608	102.8539	83.84087	165.0749	296.6115	270.462	394.6128	269.8043	122.112	76.12753
CCNB1	5776.065	6204.285	4541.925	6869.241	3333.696	3946.202	2467.631	4481.77	5393.565
AP1S1	547.4811	239.4243	165.8647	233.878	132.5008	171.5786	149.4574	177.5745	637.6198
HIST1H4G	35.4759	32.5584	35.44656	40.3375	39.7225	32.84987	32.91731	36.85426	32.2858
C6orf27	99.98684	70.21387	51.79847	76.11773	42.45668	41.80359	46.4318	56.72875	55.862
RNASE7	18365.46	19947.65	15061.86	15035	22253.24	11686.52	14681.31	13909.99	13276.67
SHE	37.04384	34.46158	35.71997	40.85644	37.98614	36.23838	41.11697	44.74668	48.51762
SLC10A3	236.0913	1629.321	3221.775	282.2644	7979.37	2239.065	5789.667	4081.998	3312.674
HSPH1	48.78745	939.8458	1739.274	1765.909	2780.298	1956.225	2170.304	2773.653	1685.514
FUT1	278.3773	177.1246	112.6116	208.9868	85.78535	125.9641	75.29102	124.8671	345.4218
UNC5A	36.21211	34.65979	575.6719	37.65411	39.51844	1005.931	927.5247	41.84032	75.12899

MGC45800	70.12724	275.1282	185.1674	399.7061	50.56923	178.4781	307.7404	54.50251	50.03041
NQO2	457.5161	223.4499	159.2801	246.4187	282.1181	177.3414	199.5696	210.2591	562.131
DNAJB12	38.87344	57.55027	247.173	67.84152	328.8604	82.67507	737.8223	849.0686	51.85962
CCK	39.14505	32.08414	41.02162	43.0743	99.70573	31.51188	110.5239	34.9096	39.74895
DYNC2LI1	36.29256	33.99962	44.43973	35.61895	36.56052	34.37502	36.61033	31.47205	35.81218
DUSP2	419.78	235.5787	271.3057	241.2566	245.8885	208.9091	191.275	193.7961	563.1711
YIF1A	23710.85	25706.44	31644.04	32527.14	30598.62	30962.43	39781.11	33138.63	22624.53
CORIN	58.87923	93.63192	56.04755	68.49056	43.42379	34.46096	48.63807	47.67397	61.88142
CDC34	891.0365	112.1195	201.5281	616.9016	152.299	218.4135	163.2435	131.2952	882.8847
ABCC11	38.28961	88.96935	54.99048	46.37317	54.96804	74.40832	46.87037	37.92596	74.67024
PCDHA7	37.01183	38.89144	40.19484	31.50418	33.13501	32.29057	30.16291	32.47598	33.77629
OPN4	59.17671	53.84863	38.05027	45.51035	40.46062	50.87519	35.24026	33.80886	69.24854
PGCP	251.5771	862.1053	201.0234	99.54109	123.0619	270.0696	112.7478	105.6364	964.8204
PFKL	1640.384	1453.086	3598.609	2235.703	4294.039	2388.262	3558.04	3246.619	46.98581
PSTPIP2	370.4688	315.5653	505.1892	435.12	337.5434	628.8353	496.5021	284.5421	756.6125
RPL27A	48.99768	107.3504	356.0211	498.091	130.7291	507.3805	337.0077	175.9761	1257.861
MSI2	1742.739	2634.414	2419.396	1406.117	1292.67	1995.941	1918.006	2430.383	3202.304
AKR1C3	14135.37	9183.967	11549.2	9882.129	17792.02	12525.9	14721.6	12079.78	10298.94
TTF1	592.0891	2734.948	1629.569	2789.711	1197.099	928.3615	1258.205	977.0761	1774.83
DLGAP2	42.22356	35.4466	59.31332	39.53895	37.92385	36.66953	35.85768	46.28692	33.29179
MRPL3	103.4229	1075.996	879.5912	631.8457	2999.663	2055.413	1873.445	2166.561	155.4896
MCRS1	77.90651	46.0916	169.6839	79.08778	93.3334	63.98508	159.0967	379.963	79.98798
MSL3L1	348.9433	532.6213	1843.734	998.2951	965.4496	1561.82	1138.47	1916.89	1576.367
USP16	57.98794	65.10792	50.53139	233.5884	68.30354	46.0755	49.38865	74.25037	41.55527
TCP11L1	1615.965	2504.359	2669.132	1993.137	982.4942	2488.637	1741.645	2410.193	277.8587
FAM116A	493.1541	388.4207	594.5374	698.1858	671.4753	822.5703	932.9281	433.3122	43.99948
C12orf41	5115.73	7564.106	7874.891	7646.23	8601.214	8222.244	7565.969	6292.886	3047.603
MGC12965	7870.918	4629.413	1952.7	4314.375	1136.51	6053.427	3132.608	1490.268	4723.486
SLC28A1	94.57491	67.44069	43.67106	65.81665	59.58232	58.41209	50.18671	73.49426	78.66157
FSCN2	1101.374	1109.863	574.1633	793.4615	361.6081	735.0415	163.3352	299.9013	2088.01
PHF19	1246.049	1110.34	1145.461	945.1955	942.4383	1387.699	913.5198	1007.646	1599.269
FAM46C	2184.754	10031	6238.508	2073.802	8737.318	6428.188	4851.409	3629.248	4292.574
KRT74	251.6059	106.1947	88.28694	104.0006	163.1383	111.8858	60.18961	53.15403	117.1827
OTOS	52.97979	40.11573	42.50281	35.85445	41.25216	44.01126	33.96394	36.30683	51.75331
NAPA	234.7493	165.5792	77.1407	157.4916	90.88495	111.2926	72.82767	64.8586	148.4253
DGCR2	143.9559	850.6365	181.512	565.4423	385.8249	60.02592	436.374	556.8414	1238.646
CYP20A1	39.745	40.59117	94.29337	56.02306	52.80383	128.0165	51.76215	63.24243	45.68172
CHL1	86.15094	63.39882	51.54597	103.0622	148.5341	48.89906	107.1392	32.89524	38.67684
SATL1	64.3123	54.38158	583.0965	47.25797	44.40652	40.62754	100.6733	186.2606	63.68991
RPL13A	1121.86	1723.95	1285.917	446.0513	1036.111	893.7174	1041.223	1811.273	794.9569
EIF2B2	2413.364	56.77815	1464.502	748.7758	1195.847	1174.471	1168.462	1274.571	40.46168
PHYHIP	205.4886	261.6691	66.88998	80.40649	106.3192	53.61259	84.37798	99.9563	61.56869
NCCRP1	5148.358	6225.102	10934.63	4530.616	16566	8290.722	8023.759	10008.54	4790.04
FAM160A2	64.18079	146.7502	2443.694	764.8232	2884.78	373.1383	1498.635	1531.215	37.11572
ST18	43.36102	34.32806	34.1957	32.68053	32.96421	36.93317	33.96642	47.73426	54.12239
MYL6B	2247.019	3760.976	3574.163	3335.105	3448.459	3063.297	5613.619	4681.803	1457.156
ZNF671	44.34684	451.9008	247.5884	46.57143	456.3421	51.54673	352.1009	140.5801	40.06233

HBZ	51.40358	894.5757	85.09731	376.4435	180.398	247.4019	117.101	157.5986	701.2496
AQP8	50.35862	46.60551	288.4288	62.36956	50.56907	382.1525	71.11187	158.3284	74.62234
ENOX1	60.47544	54.3536	48.39655	45.43725	63.7253	50.90827	46.35516	72.60227	141.8187
CWC15	51.71618	334.766	776.3646	49.70655	1101.65	761.9819	1376.089	932.2742	70.77578
RNF25	62.15405	46.1392	54.27809	48.59817	48.01696	56.38853	90.22289	40.43145	37.48144
VAMP2	35.84314	1479.62	459.5715	1199.655	442.1784	285.0597	522.8543	282.1466	1380.479
ST3GAL5	48.17756	381.3319	160.0098	523.644	351.5519	486.6079	417.7714	272.1796	43.62052
WISP1	444.9072	61.88107	100.0294	43.64894	77.71786	200.0299	119.732	34.21529	44.60265
NPC1	44.01778	76.6101	828.2007	265.262	889.4183	700.4719	611.2046	653.8856	915.5618
ZNF205	113.5182	67.87064	254.9342	51.54273	70.21413	58.10672	210.9399	215.3867	69.83679
KBTBD12	46.55228	2058.546	845.3543	40.20901	509.5929	762.0625	2657.149	1910.345	2155.992
SEMA6A	928.9211	3031.293	1778.457	2070.38	3076.271	2168.723	2397.241	2414.588	234.1406
TKTL2	60.61176	37.6689	45.37095	32.62471	38.72311	36.18494	32.91969	34.28991	36.06366
LDB3	59.96201	55.99389	62.15258	75.0179	58.73444	61.71059	48.47349	48.40901	114.1889
C20orf103	2335.392	1899.446	7473.842	2824.183	2783.946	4971.779	6642.729	5002.299	85.29784
OR14J1	37.43548	56.87822	42.35522	67.2954	46.85493	48.87227	42.13009	35.73612	113.977
PRKCB1	213.404	185.4335	41.84383	39.7379	38.30191	31.29449	39.00546	45.19986	44.86384
RPAP1	516.4432	220.4257	178.3614	218.6187	102.869	174.5027	145.6861	134.9777	130.7959
ATM	47.53071	51.3536	44.19123	715.3128	44.85452	412.9537	36.73047	202.8252	33.42607
S100A6	1680.195	1934.756	1800.354	2850.904	2126.294	2163.792	2421.292	2886.298	1590.284
TSNAX	3118.464	1226.502	1134.467	570.3193	684.2899	1171.015	955.4449	1065.793	1496.461
MREG	1374.495	80.77819	128.5362	116.1274	200.2847	103.2704	134.1375	284.2372	957.3661
IL34	107.8375	43.16502	37.96325	45.10533	34.81571	45.94304	150.2138	59.56958	41.97899
DHX32	4845.291	3266.35	5790.004	4494.027	9721.103	4759.194	9074.71	6201.213	2785.47
ZNF786	3179.105	2394.482	834.6464	1285.603	931.7055	1415.062	671.145	794.8166	2452.432
KRTAP17-1	44.75301	50.02961	37.75509	48.29707	38.77657	55.84857	150.9263	43.94275	90.462
ALOX12	72.09513	41.18631	142.2609	53.57929	759.9037	307.5846	84.79121	38.33978	40.36598
CORO1A	50.43616	39.83552	752.5218	421.6849	41.00958	432.3999	51.14526	733.8643	1932.27
HPGDS	106.6498	335.6191	164.7836	347.066	181.4511	52.58067	166.2256	260.5932	133.9812
GLUD1	42.52083	1412.665	3386.4	2696.489	4736.982	3031.044	3619.321	3664.126	2192.673
OPRL1	48.82338	62.84057	61.17734	60.11291	68.41682	47.47375	45.80101	54.27728	65.08655
UBE2J1	7334.655	8682.626	7757.992	5666.567	8883.341	6401.657	6384.038	6267.426	6706.364
MAN1C1	50.76101	34.16493	36.12199	40.82452	45.63104	249.0857	82.28555	37.05976	94.90228
KGFLP1	992.591	896.9652	304.043	363.549	182.5345	288.4523	269.7057	257.2066	875.1587
PTGES	278.2867	202.8266	60.85207	172.7008	73.46844	97.61543	137.0853	82.51957	245.7983
SNORD15B	1665.741	1696.999	1492.576	3177.129	455.5984	1716.786	1858.743	2275.203	131.5744
ENHO	59.61899	62.10024	65.29923	70.6881	88.54136	84.42884	67.15518	60.76613	274.3141
LNX1	35.51756	35.49307	35.19133	30.78916	60.91987	37.51129	42.45025	34.01635	63.31419
PMFBP1	68.66592	46.17223	35.999	49.53105	53.36739	59.84761	61.67682	49.26448	71.0719
NME1	500.1897	299.6016	150.8773	320.1656	139.4182	219.6615	141.9392	277.9567	695.6729
C16orf42	4407.458	3157.381	2654.897	3870.886	1817.262	2343.648	1942.957	3005.285	4991.884
C4orf34	4166.919	3706.068	4416.822	3391.905	4183.146	4442.153	4546.677	4758.169	3551.962
KDELR3	99.67899	70.05395	57.59413	74.68597	286.3405	54.93075	121.1093	258.998	71.34374
CYTH3	456.5358	253.3553	137.9437	300.0631	157.1941	185.2887	248.0577	218.1663	606.8845
AMTN	36.28065	48.19165	47.11715	1537.074	43.89059	59.06752	807.2636	40.17911	81.61367
LITAF	40.07259	46.81358	140.6542	70.28246	154.6156	58.59245	205.6405	60.9235	96.2017
C3orf33	3855.405	3535.731	1819.341	3245.397	3803.897	3827.196	3209.848	3616.817	64.7226

C1orf106	46.82044	45.13776	1099.096	686.1478	2302.727	618.9819	1100.32	1419.307	39.27392
MRPL52	7669.855	8455.816	8737.794	8602.717	8162.187	7381.718	7574.917	9378.654	6932.244
SDF2	132.1244	1005.825	1227.299	1214.631	2061.649	1683.229	2311.081	1547.839	319.6482
MAF1	123.6849	66.0216	41.58088	53.205	476.5738	41.88601	125.3717	58.85241	96.09052
TRIP6	535.3944	1141.615	1052.828	312.3333	2632.266	228.5004	1421.135	2007.034	731.4617
UBAC2	875.1814	838.4345	1041.135	1128.355	1087.93	1401.376	1068.531	1092.205	1090.392
SCNN1A	22516.29	16668.16	25536.9	15324.94	27821.41	18978.97	18383.04	21562.34	18924.84
NXPH3	44.58187	70.36842	49.25331	62.15387	49.50954	56.75077	47.37315	80.7541	60.28231
KRTAP4-4	59.62547	39.82936	48.73539	57.25157	40.5802	36.96899	46.58536	35.78157	43.34161
SLC9A5	298.5952	173.6102	110.234	149.9729	88.00997	74.14703	72.72376	93.47311	272.4564
KRTAP12-3	50.97665	65.54518	60.22725	53.66436	69.67157	66.77258	48.6599	55.91936	272.1705
PLCB3	1318.16	814.3772	1307.894	1519.814	529.434	555.5614	915.4474	1159.644	1329.224
FCN2	34.83405	47.61137	38.34426	47.66672	36.15628	37.64196	33.04164	49.0835	40.43635
CMAS	185.4747	111.1657	258.9586	115.7605	625.7181	86.60448	217.7977	191.0147	131.2502
KLHL8	831.0054	612.3412	881.3155	49.12011	504.8875	447.6855	630.9435	283.0401	743.6974
DGKH	216.7521	155.9131	164.8783	497.3349	104.6326	301.4823	128.2323	102.0337	129.9463
RMND5A	70.55168	68.97884	143.8051	44.91042	161.284	55.8054	183.7452	58.66906	101.1096
TP53INP1	473.5787	1057.208	687.0808	894.346	849.7734	829.5817	601.7119	495.1056	643.5132
CD164L2	1873.848	49.90678	4036.549	957.6035	4058.986	2737.08	1619.497	3261.727	85.13691
PRMT8	70.45391	46.99849	37.10121	284.9575	547.2816	254.8905	63.096	43.72221	48.45204
C9orf90	1294.941	48.7873	1600.461	44.14114	2406.038	921.8661	1838.294	1780.252	1917.5
GALE	33.2963	44.13533	35.89781	50.39716	42.94954	35.08999	35.21704	44.08916	35.66928
PON2	450.8911	249.0545	131.9929	824.5385	289.2167	496.8425	272.2809	271.3403	619.358
LUZP1	7470.059	5477.371	5412.472	9314.517	3980.864	6969.794	5374.685	7039.515	8322.126
ST3GAL2	11267.62	11787.57	7370.625	10140.82	4298.396	7305.515	6002.628	6994.176	11527.97
CYB5A	563.5574	671.2086	517.9041	41.42189	1634.063	917.554	993.0084	628.4766	81.24556
SPSB1	36.16265	620.1281	667.8791	682.8862	1218.428	789.5791	888.9253	804.8419	36.18172
MEGF8	214.0184	153.1677	104.3404	164.2366	135.3209	176.2915	96.55654	130.0133	338.5231
TSC2	4465.887	3474.683	334.436	986.2401	1245.336	313.3134	1134.591	1160.156	5440.776
C1orf105	283.6159	129.3686	62.5658	160.8265	62.85277	84.46054	96.28544	125.4471	407.9201
LETM2	162.2741	71.56168	46.33414	61.66303	56.45438	40.81038	147.6998	57.16448	52.74523
SLC16A13	259.8513	673.0514	69.95806	145.8394	66.90869	122.6794	65.91865	78.61537	151.0798
AGRP	76.57454	56.58585	43.45242	43.82079	46.59919	300.8777	40.50803	47.79531	145.8665
FAM19A3	37.15947	53.07607	57.99791	46.01616	46.09478	53.29749	44.99456	48.89749	51.1568
C9orf30	8029.63	3979.554	2991.689	6926.813	1177.604	2713.546	2434.106	3331.376	6066.999
DNAH2	2690.191	832.2509	1795.398	1682.887	6517.653	1160.643	759.6403	1734.179	2988.951
SETD1A	325.8387	526.5442	988.248	566.8746	1178.57	1052.811	1377.245	1217.811	259.4038
RDH10	47.34082	41.79622	450.2085	752.6487	315.7791	180.7109	218.267	128.4477	36.18113
GLCE	41.67315	687.4742	901.905	54.96281	486.413	682.7539	651.2745	763.5787	142.5589
DMWD	1049.393	2937.2	2204.009	1412.105	2367.264	2339.699	2441.353	3284.364	158.8467
ITSN2	216.8486	81.29255	55.31797	69.57935	49.86216	60.56017	44.88914	263.4248	49.22718
PCCA	358.8949	184.7932	316.9071	224.1989	479.6145	166.0357	528.5981	123.5635	401.7184
CCNB1IP1	36.42567	39.25249	34.36963	41.88995	32.03437	38.85176	116.6252	48.08129	35.44895
CRYL1	206.2715	117.7136	172.769	112.539	109.1821	147.8961	106.4934	95.80755	337.9136
SNX27	2389.437	351.7737	1428.947	816.8715	2913.39	2149.039	2215.597	1654.802	2323.573
ZCCHC2	89.23801	61.06148	1015.247	1027.761	321.5055	57.2943	375.796	48.01475	3160.503
PSEN2	1324.711	2700.76	3552.614	1653.665	2385.144	2832.073	3314.606	2102.719	41.8412

FAM101B	1915.267	909.2552	287.3713	1024.613	189.0809	544.119	156.1243	64.01902	184.8482
PTCD3	69.35741	58.72923	409.3778	540.4531	254.4925	42.85349	213.039	114.4796	55.66615
FBXO17	94.31335	75.47889	141.4683	54.65988	44.16953	47.78528	90.59935	54.12052	56.26867
MARCKSL1	1345.73	1353.292	929.6672	804.4957	548.8192	1691.064	1284.285	1079.237	253.2756
MUTYH	1459.062	95.00135	671.3898	499.3056	1265.892	863.431	1108.844	652.9658	87.10371
KLF12	65.13835	43.03524	43.92358	50.29606	50.79061	48.73591	38.68957	54.2863	58.74204
SLC6A9	4679.411	7967.528	7032.729	7891.803	14340.07	8064.839	12778.66	10505.79	5917.132
PLG	48.52224	34.96106	33.54791	34.59945	36.51972	39.77913	39.66129	49.42089	78.50674
OR5D18	234.793	182.5895	95.10997	145.1143	66.68192	95.67494	61.11861	85.40405	468.1284
RNF14	245.4134	210.6966	190.5402	226.8354	187.4106	252.3828	153.217	301.6724	295.5279
ZNF436	600.696	240.3535	169.4044	348.407	135.7841	177.3448	194.3336	238.6456	480.2505
OCM	43.79877	61.44335	40.25057	34.08528	44.6977	40.81798	41.11519	40.79673	56.92309
GAL3ST2	73.48156	42.27946	44.29007	36.08406	36.41928	42.34893	36.5425	40.43705	44.4269
CIB2	192.9964	868.9737	606.3684	1114.182	310.3385	279.9913	544.8711	546.7437	1740.82
PARK2	41.49831	107.182	39.23796	40.18013	36.26758	48.21934	39.051	40.5674	55.16541
MRPS12	69.09785	567.4688	2152.334	49.22759	2535.156	344.2627	2767.908	2764.607	55.69208
CLMN	309.5934	109.1531	99.16438	195.3416	297.773	131.4392	278.6718	364.6313	494.3096
VAMP3	251.2576	76.61742	1509.602	206.0535	1914.579	484.9416	1311.519	2259.06	138.3886
SCGB1D2	48.91069	43.2495	40.46217	35.05505	34.8611	42.60663	60.02767	32.56	61.1602
AHNAK	1166.177	1184.663	2414.874	2003.366	4180.263	2639.475	3380.182	2405.92	1020.344
PCTK1	38.326	594.9559	144.7652	524.5869	94.1797	35.21312	62.70451	108.6349	1089.366
OGDHL	37.45489	41.84861	57.43024	55.67579	49.56526	39.71247	44.99632	45.75686	38.43727
VGLL3	75.84154	33.88807	31.86655	57.4166	37.44954	31.34801	32.29365	37.4641	38.90607
CDA	6552.296	3109.349	8751.321	6055.624	10324.27	5892.638	5236.792	5324.35	2683.848
CAMLG	17503.28	16511.45	14944.27	17259.49	15348.92	15884.53	14830.24	18334.38	17475.22
NAV3	78.76768	36.34697	45.38269	66.37131	46.45616	41.19438	48.09868	53.85587	92.72935
TNRC6A	60.31252	236.6881	159.9431	335.2555	121.5098	188.5408	57.72929	78.46197	45.67985
TOMM22	3421.575	3853.857	6434.8	4777.751	7934.606	5062.97	6864.854	8603.013	3741.319
MGAM	187.2463	126.5106	67.44384	106.5237	94.72989	106.2871	74.06264	94.8101	118.3123
RASGRP4	62.18293	81.06463	45.12317	116.914	56.53689	40.86884	47.31312	63.39014	81.89584
HOXD10	51.13122	49.15359	268.6273	46.0593	48.19207	420.3595	137.9248	441.6159	48.7086
TRIB2	1287.878	707.5544	1273.246	1524.596	1901.101	1892.677	1233.355	1209.913	1994.542
OLFM1	949.6813	38.42965	579.3785	44.09462	558.6595	829.3749	380.3431	387.4376	95.48993
CCDC120	104.572	313.7835	338.1534	480.5445	454.9688	677.2084	435.7597	369.4641	125.1152
TMEM34	105.3362	109.6913	60.51585	48.08513	61.06543	49.78069	50.22709	63.96127	161.0164
CXorf59	242.6783	75.14593	89.15977	103.95	68.40908	104.3098	61.85777	54.6496	473.1962
UBE2C	131.2375	68.42943	91.86939	126.6587	54.51971	57.02634	58.82488	60.45799	140.9923
ASF1A	75.95923	57.17066	50.47631	120.5949	50.37683	44.17274	59.81557	54.94775	93.68046
PHF6	64.39434	44.94992	36.4118	47.23646	41.76669	61.04475	42.77071	51.06148	47.83339
YIPF5	1021.843	1355.227	2365.131	1878.203	2426.952	2425.709	2157.07	1338.499	1224.482
CNIH4	7024.699	7912.427	8013.135	10151.73	8831.71	8459.806	7538.707	7850.271	6763.006
OPRK1	322.2019	170.3221	83.06437	182.6216	69.29295	106.5947	78.33848	98.69682	317.2097
ZNF3	349.17	284.8491	173.7065	388.7496	91.79922	175.5482	62.64966	94.28853	48.93381
RPIA	75.12597	77.59178	128.0878	76.05061	149.3974	184.7542	158.1644	108.0473	76.82512
OR6K6	49.71672	45.04124	34.69645	33.13429	50.47424	46.35252	41.83804	40.24377	72.75607
ITGB1	2666.48	1273.071	667.6503	3180.912	498.1299	1207.484	755.7504	354.6884	204.6314
OR4D9	74.22768	43.59219	57.7418	39.22199	36.07512	37.60606	40.07899	56.51339	92.78263

ZNF354A	53.18837	57.5718	75.11507	162.4587	88.17945	140.9864	69.68536	80.18448	89.47187
PCDHGC4	38.50731	492.4874	33.12319	34.86065	123.1926	37.67289	40.93662	146.7676	34.57871
CD68	13883.17	12812.19	9875.698	12409.79	6361.116	8632.098	8857.59	10121.11	14109.74
LIMS3	57.83945	43.751	38.24208	43.45233	43.77154	44.61593	37.66664	37.06913	37.49097
GHRHR	34.8981	56.08123	38.14313	69.99685	51.89312	51.56975	33.84597	35.60817	41.41636
OR2A14	102.9347	147.2711	78.5822	109.7194	65.49187	65.53719	69.62723	80.96472	174.57
SART3	301.8947	105.1938	97.45576	100.643	97.80565	131.2173	74.2556	69.80325	306.342
CCDC142	43.99239	48.36633	150.1406	55.32524	55.61235	44.62086	71.46728	44.60188	42.19787
ITPKC	110.9577	108.268	93.72097	151.5995	75.6986	96.439	54.1989	59.07072	100.7997
ZNF703	647.7982	420.8376	132.8665	459.5679	151.8403	245.6452	148.075	144.7303	701.7534
PLA2G12A	54.59229	63.80978	138.2119	45.48988	137.3413	118.2659	104.509	162.796	40.83529
IQCH	59.73729	62.98437	60.79734	44.33935	46.23851	56.6409	44.76658	61.54865	69.05073
NFKB1	2045.605	1520.889	2411.029	1992.862	1995.882	2066.365	1768.379	1712.406	1552.157
MRGPRD	79.15303	81.99791	74.08491	95.21081	58.66443	67.77397	61.5754	75.66128	126.2262
TRIM54	41.23545	47.51581	59.84861	39.83467	36.08222	49.87873	37.76745	40.2274	40.41506
HOXD8	79.22819	61.74398	89.65462	84.83456	46.49719	57.12038	85.42583	97.08077	38.15347
DBT	4053.325	4624.857	8349.892	4626.556	6856.759	4385.003	7135.436	4961.42	6943.037
ERMAP	285.1958	120.7273	115.6985	92.86623	200.714	83.56589	242.5019	124.8894	371.0263
AQP9	722.2046	62.15527	161.8309	96.38004	114.2827	95.78041	275.9638	149.5463	1016.463
TANK	597.2265	397.8	225.7908	383.8404	219.2115	317.6551	223.3786	359.3761	788.9909
KCNA7	222.703	85.84515	62.00926	110.3459	107.7303	56.6294	147.4391	137.0996	348.3778
C6orf168	165.2979	133.875	74.2777	139.7213	74.66657	82.29948	154.862	89.59383	249.2472
MTMR1	31.57588	50.82659	35.94348	36.05984	34.30803	51.62529	35.95396	40.22562	47.59174
HARS2	10061.49	9451.018	8437.513	9240.79	7640.993	8892.681	6967.545	10090	8927.665
HAAO	179.492	162.084	80.05906	115.4019	74.07899	110.9092	75.51789	80.51724	212.5638
CCR7	1208.025	3200.264	7773.913	1209.136	3081.566	5824.396	4502.797	3264.311	3035.481
IVNS1ABP	48.17232	49.79412	832.6537	1420.128	334.8934	857.2902	507.0858	246.7049	52.16214
HLA-DRB1	498.0012	242.485	2186.101	1869.086	105.9258	174.3699	101.6744	91.68055	416.936
RPS6KA1	2720.662	2809.313	2642.714	3494.354	1710.831	2603.913	2511.823	2613.922	2840.384
MET	492.9485	675.6468	853.1802	1553.657	774.2303	863.9587	1080.383	781.5304	1512.698
SNRPA1	234.8896	231.6577	691.3871	630.2241	698.7193	749.5799	970.0365	765.0386	121.0005
USP8	73.07234	58.73236	132.4285	44.95238	191.8222	39.82277	116.9844	37.80849	210.5545
ACE	96.13477	91.78748	59.3363	89.56703	61.00803	51.26296	46.10339	62.61367	65.66933
DNAH7	66.05772	68.26852	44.58969	59.68713	51.64433	63.52346	48.12928	150.8816	54.56162
PTPN7	177.9477	131.2154	414.3669	214.3775	169.8144	293.9818	304.4008	483.4145	260.6914
MAGI2	43.43605	49.2658	142.2227	46.3483	169.5814	248.8308	43.36305	57.91445	39.41237
OR6V1	208.84	159.8092	94.16296	211.9796	97.80576	152.8329	83.33221	99.49286	354.1019
PICALM	55.86297	48.83022	214.2866	697.0251	96.27843	225.2981	125.5275	189.4994	46.60523
FXYD2	36.44395	44.92957	42.1214	51.60427	42.90559	34.3669	33.42115	37.10052	54.27005
HSD17B8	100.7303	99.29187	193.5744	159.5916	176.8264	106.4585	164.8383	142.6859	219.5493
TTC21A	69.66312	69.57285	880.0259	57.55987	337.1176	47.66703	323.5088	66.95222	120.2267
SCYE1	60.74636	308.282	222.1295	69.91119	218.2408	212.2596	178.5495	184.2752	66.71967
CAMKK1	4501.247	1872.185	1352.639	3156.729	1922.124	1808.453	1313.355	1433.185	3468.928
INSRR	358.8748	216.9601	101.8361	217.7229	90.1569	153.8378	92.1162	112.2602	259.5142
B3GNT6	35.14605	41.12423	630.6381	1286.111	529.5608	1040.43	440.2971	453.049	2222.651
GPR111	73.30385	40.41147	40.9321	52.33177	46.41411	59.69861	43.93734	61.52478	42.52925
PRPF3	255.483	529.2053	2513.502	555.6385	2906.256	2315.014	3025.317	1985.327	151.7819

MAGEA2B	41.02354	45.7668	40.58279	44.30603	41.17827	47.49883	32.50991	47.08891	34.31908
MT1G	5349.612	4022.45	5158.458	5931.951	6050.295	4127.192	5106.597	3253.484	5754.986
GPR174	44.65318	46.16656	214.9318	36.70908	36.53652	377.2608	70.65797	46.4614	43.64648
NUP188	269.2462	1796.376	2431.306	168.6504	3115.371	954.4523	2739.376	3458.943	212.913
SELP	7854.687	6838.824	7085.024	3926.274	9174.138	4767.296	7169.212	8393.396	3146.402
KLHL10	37.0314	51.18212	49.17065	85.42302	41.16187	41.75282	46.33132	39.28839	40.29031
CASC5	86.49787	441.1214	1024.319	650.0508	725.8173	813.662	849.586	685.137	122.7915
FREM1	46.28091	32.12208	50.72969	35.88083	43.14435	387.1283	34.55725	34.0638	38.65432
SOSTDC1	159.9293	183.9498	92.34521	212.5865	72.91904	150.2923	103.0211	118.7471	318.4227
ULK2	120.8821	139.6956	338.8192	328.0214	246.9234	123.4325	585.2051	259.6367	139.234
HTR2A	4140.322	2531.42	464.729	1521.632	294.4904	888.0013	1044.179	1227.875	6079.193
TTC6	42.48033	40.24245	50.32772	68.21603	40.44704	37.04017	40.22864	53.81354	38.89325
NNMT	2598.266	1555.166	612.4807	817.1576	153.2627	379.3771	287.3201	664.761	2903.57
ZDHHC21	63.85336	439.7722	899.3434	400.7615	1063.439	1240.304	403.9866	525.2436	1467.536
TRAIP	304.8775	205.1234	110.3354	215.5767	98.08773	146.4713	90.25601	117.9327	458.727
ACOT1	56.68529	45.66616	42.35213	48.19166	42.77835	38.91748	45.54934	44.34887	64.87861
GABRA3	106.631	69.5836	51.86849	55.80204	61.55471	73.76835	44.84141	58.91304	40.30634
DBP	569.381	754.9714	459.3684	389.643	97.45081	264.1019	221.3824	193.544	851.9324
C12orf50	65.66663	39.40081	55.20336	41.30693	48.30959	34.01869	41.4665	44.18527	53.81096
CCDC53	514.8491	782.0081	1389.117	1039.261	1748.868	1176.168	1970.873	1983.39	2442.333
ZC3H11A	4514.685	5748.415	5224.29	6649.725	3315.87	4065.681	4067.595	4719.871	4836.287
BOP1	2811.244	2894.647	1234.794	4092.134	694.661	1821.361	1260.081	2132.371	3030.635
SOCS5	46.47482	38.26	39.70649	32.22593	34.94528	58.48425	240.3078	38.56068	56.34427
IL23A	44.11211	635.1605	307.1793	53.36601	538.9414	385.7283	293.3082	425.8315	1891.54
CHMP4A	2241.839	1385.823	579.3421	1242.203	302.4422	1177.887	561.9745	506.4458	1780.699
OTOP2	59.98859	65.10128	53.45243	60.75045	53.32808	46.70444	44.46247	53.60163	87.91975
PPIL5	46.72348	49.75539	119.7737	43.91457	257.1161	155.0376	261.3012	216.7759	40.67012
C20orf94	883.2064	1497.572	1271.625	1638.961	1174.115	1596.825	1059.697	1213.123	577.5943
ATP6V0E1	2142.195	2182.895	1623.745	1968.455	890.9293	523.4852	1046.757	1145.782	248.705
EIF3B	82.06908	145.22	319.5446	51.28704	559.2078	67.82382	608.3579	158.6377	222.1253
VANGL2	193.2715	168.421	51.40552	356.1163	250.6869	61.02903	211.8806	116.2002	50.02283
C9orf71	46.68257	52.41089	51.74865	79.14326	41.60841	53.07183	47.06439	66.93398	83.16966
HIPK4	65.73126	61.90523	40.20393	111.5527	39.93373	61.56458	112.5377	219.1447	43.6049
TFAM	1083.002	1897.37	3438.102	2587.305	2862.328	3815.193	3621.102	2204.024	982.0793
CCDC80	1397.347	2034.899	370.553	1321.647	1937.983	555.2897	959.6707	1151.845	34.47262
TP53I11	94.00896	92.33675	38.318	54.78214	38.02068	476.8233	102.9888	45.00144	50.82097
HPCAL1	36.50538	59.78577	37.38522	36.7609	40.72569	33.50538	31.16648	43.18633	33.55901
ITPK1	64.13505	55.90754	303.3478	59.29848	47.49906	53.02385	221.9989	250.3972	97.29762
NEIL2	40.85575	47.26619	58.60049	43.38883	45.72977	50.33073	164.3431	51.49095	37.11009
S100G	75.75374	35.08693	40.05101	33.18568	38.84689	43.1281	38.06272	58.57112	33.01436
MGC71993	708.7606	466.2732	3078.394	83.78825	2945.774	1270.007	2627.46	2647.926	1454.823
DALRD3	45.67284	68.95941	50.37069	41.08879	47.31585	40.41488	49.45763	38.55443	46.60207
SSNA1	337.978	157.419	95.05921	205.7905	336.4833	136.8672	291.7125	431.0733	454.2708
CEBPA	8307.282	9352.126	4855.376	4776.688	6725.743	4434.177	3086.822	4846.584	8275.686
ZNF784	487.5008	394.4286	262.104	320.237	253.1432	367.7306	262.3246	333.8769	722.2993
MCART6	34.52139	35.39837	213.8404	46.48553	37.43178	240.3215	41.21059	37.30021	81.55361
FLNC	21204.28	24630.35	18155.28	20221.89	12843.18	17788.38	20196.07	24828.84	22189.4

LGALS9	52.05838	462.6871	36.51337	71.72583	142.0597	54.53288	224.2304	236.9069	43.98061
MMRN2	135.9981	91.00863	61.2024	93.88808	106.4186	90.21249	76.85036	84.16992	94.97367
DOCK1	65.59198	635.1038	387.4637	1459.247	37.76194	43.80148	619.2755	408.4413	71.16552
PITPNA	44.45143	38.07322	168.7216	40.60155	1430.759	40.05313	878.0168	704.0849	35.31403
OGFRL1	68.89629	860.6549	1255.246	2525.958	349.6557	846.2815	693.73	619.2806	40.115
LMAN1	738.403	452.3035	339.7125	410.6594	157.8311	321.9323	207.5654	134.0581	838.797
CYFIP1	90.67128	249.0765	61.92933	297.5069	55.95179	95.15904	47.59404	67.87601	125.7436
FBXO16	48.57407	57.35723	44.57521	46.49298	52.62847	101.4243	41.72735	59.75645	88.28639
PIK3R4	84.35453	61.91318	215.561	143.4271	310.2544	66.2366	55.78264	60.02517	86.81448
CRYBB1	3180.896	2270.453	728.9597	839.2172	200.4269	479.8635	374.6954	414.6143	1884.58
PMVK	690.1466	332.2909	347.2745	387.4637	477.3553	277.7711	242.2034	331.8045	213.4325
FBXL17	111.7792	64.22095	89.25589	54.93645	65.63849	64.18311	56.67045	51.54077	95.55016
DUX4C	75.96314	57.78661	39.36477	39.4212	49.42341	49.65253	42.41498	43.42215	55.64902
DSN1	136.7664	66.12392	41.13575	576.51	214.5541	75.81751	242.8856	186.052	115.6229
TAS2R60	45.01852	49.52674	41.64639	57.95098	41.37829	39.38509	68.39722	46.11448	65.46552
VSTM2L	50.5943	69.98088	49.4679	60.11296	271.9209	52.14285	108.5173	51.30153	72.37158
MLL4	890.0521	1179.959	222.693	78.33342	1265.549	94.72136	996.0193	86.05152	161.9471
SLC25A37	33.92737	33.851	43.81362	35.23766	46.18667	32.9418	36.41583	43.16521	33.40902
SLC22A2	80.27586	69.73445	46.35117	90.28162	65.0955	90.95492	51.96292	71.79504	198.2428
GRM6	165.6101	129.9914	78.87196	85.97906	41.14463	74.73356	63.97144	56.05832	231.977
GREB1	47.30802	39.28226	42.05359	55.89513	34.5039	37.57997	41.93921	166.105	46.54345
SDPR	617.2148	285.2348	96.65881	242.0853	324.0389	273.8999	150.9054	202.8889	123.2705
HGS	34655.71	33947.71	36956.79	35337.37	34077.71	33999.14	33275.65	35744.36	36803.39
NSUN4	463.3315	355.8077	306.1592	354.8514	232.641	380.1964	298.1737	177.0307	305.6781
C18orf54	100.552	77.20718	64.24188	72.26235	141.7503	78.03848	87.90781	72.97279	202.6621
RHBDL3	107.4269	77.4653	138.8453	86.60332	324.2904	211.5015	149.8262	124.0829	91.53582
ARMCX6	2393.917	50.39904	40.55958	58.46219	34.39507	35.07729	178.4828	59.23429	50.85718
LMF2	1927.001	1184.45	886.3715	1038.938	711.232	428.5457	1081.154	1256.65	1340.936
CAPN2	6140.199	4261.963	3121.265	6152.88	3656.578	3970.894	2147.218	2446.612	4232.992
OR5H14	44.53614	50.52643	40.61175	42.84307	44.18512	45.44176	41.23076	42.08176	70.32764
ARGFX	43.72952	74.51102	62.37094	75.3578	45.94611	56.21002	55.76282	56.28117	70.87091
SOX1	53.20069	39.30796	33.66363	44.27484	35.34367	46.36774	34.3623	35.27213	58.01722
PAQR3	41.85765	44.001	164.0481	457.578	590.4179	48.46622	253.3295	283.5175	58.52883
CBL	166.0853	39.35935	339.7608	1310.727	531.7172	354.582	238.5928	719.9955	51.15331
KANK2	35.22351	36.91103	81.65804	33.73688	37.07209	34.07457	65.0781	40.0412	50.36433
KRT10	1591.651	6712.02	1616.088	3588.355	7799.272	5089.949	1438.091	1094.141	2361.648
NLRP12	64.15936	45.74644	229.7314	44.15947	39.39423	49.29817	33.37278	42.64991	43.06493
KRT71	48.29058	43.78574	33.26974	41.05026	35.46384	37.39991	35.72456	39.81415	49.57291
UBAP2	88.57684	38.19481	230.9775	57.22043	56.37324	42.01227	46.83921	47.38074	79.27378
HIST1H2BG	52.63185	1061.601	599.897	403.1048	638.1081	666.4909	285.1923	705.8134	2195.354
STARD4	553.4659	372.6454	203.9495	528.7968	84.07879	283.784	107.5276	160.4455	617.3024
ACOT2	38.62967	39.69681	49.65845	62.53061	41.06818	48.50327	37.75306	45.99301	58.83931
FRZB	344.9176	1097.775	555.9305	394.3408	1502.392	1203.256	745.7999	867.0049	477.492
FIBIN	60.84538	70.65075	53.81152	45.68764	47.89334	94.5239	49.38787	52.89592	37.45069
OR1K1	58.85381	80.49125	51.07751	98.79269	55.34097	88.62599	47.96143	53.79984	52.60378
PROK1	201.3838	88.37746	64.93655	131.6106	67.13931	56.26417	115.7545	78.71841	139.7723
ANKRD1	4836.321	4431.008	5297.932	7979.942	1998.191	6691.782	7164.589	8523.204	4796.968

PGM2L1	69.5383	39.96975	326.4124	66.0715	562.3798	51.53059	342.1922	151.2795	47.8803
KBTBD8	729.271	846.2327	1087.303	625.6743	610.3534	948.0695	603.6478	673.6519	776.7893
LYNX1	37.98342	46.09924	36.70107	49.57714	44.66956	53.04345	101.1094	44.15467	68.16454
CENPE	312.9019	216.2105	320.1151	204.9046	635.18	149.4733	311.2399	119.9582	464.4193
LOC731102	83.09158	148.2064	61.88043	125.3332	65.21975	104.2024	64.21445	106.6414	169.104
EGFL6	107.667	41.99222	120.5621	299.7167	167.8197	196.4643	231.314	312.9725	871.3971
P2RXL1	70.01719	69.03199	40.00073	61.80166	54.33065	34.7276	51.95393	55.63838	66.44849
CKAP5	54.14552	48.60255	43.29199	55.99712	45.88392	33.57718	334.0598	41.208	42.44532
CCDC132	47.44549	1773.776	4013.467	2986.64	3893.267	3038.966	3305.787	3282.003	3306.542
SLC19A3	9881.371	7616.436	4041.32	4393.714	3204.76	4058.877	3179.374	2784.407	10040.38
TNS4	45.79887	42.19961	521.3904	73.14464	554.1802	35.43752	594.2586	798.195	59.48336
HMGCLL1	440.4768	180.301	63.0399	74.02778	66.35797	130.6983	62.48789	73.12882	542.7047
LOC387882	593.5652	305.9154	484.2841	349.8204	626.1719	836.2819	1148.342	449.4862	80.43689
ATL2	4760.325	4142.163	3762.997	3823.622	3294.862	4339.969	2647.023	3403.679	4604.938
LAYN	684.9097	129.631	538.7744	127.3773	378.6019	216.28	353.4373	483.8835	850.9871
FOXM1	115.9825	1364.879	55.91323	722.9971	290.184	64.99835	173.0947	65.38822	67.95748
ADRA1D	80.11974	88.466	64.66711	68.54216	57.25572	63.41833	68.35926	75.64832	212.1344
ATF7IP	414.4985	287.2697	105.3336	132.9164	68.44217	134.9972	109.9886	111.2554	603.2103
SNORD4A	695.997	564.6861	142.0485	33.70426	58.62338	34.21252	62.35591	181.8613	36.65783
BCL10	50.43348	323.4366	218.4657	408.1108	347.0544	325.1027	362.996	519.158	48.2133
OR10A3	66.8336	59.54261	47.01191	64.56842	52.64963	44.78774	46.06382	51.65675	74.00601
LGI4	112.7127	129.9668	60.17607	93.86385	56.67899	81.43561	50.20004	92.92793	88.18688
RBP7	984.6813	80.05382	973.2418	43.87621	825.8937	51.40753	593.2821	258.6617	447.6403
LOC152195	65.81732	56.91121	54.10945	65.84017	37.37276	58.38008	76.5232	150.3551	76.34562
NANOS1	140.1091	81.22295	81.58188	152.6757	59.90082	81.36088	59.90328	61.95963	71.85909
LCE1F	63.7783	66.62183	58.24588	56.94459	206.5261	86.31711	50.29047	53.10686	77.12881
ACD	90.82179	39.64732	291.5011	47.27049	1922.896	718.5255	1226.621	501.4637	134.8368
PACS1	86.56398	50.34755	56.71307	49.5449	549.4409	33.80249	396.0407	39.42035	70.04376
MGC70870	49.36281	61.78104	40.7876	72.29285	49.8864	60.9745	44.56743	41.72529	64.45552
RAB7A	3306.353	6815.174	10158.58	5894.942	14530.87	10237.63	12531.06	13195.21	3971.504
NAGK	191.8591	291.9533	353.2049	196.1512	186.2713	208.398	198.3519	142.1652	439.2682
ENAH	218.2628	128.2162	259.01	128.8483	1293.29	1778.227	528.3192	551.0296	359.5305
KIF23	2653.388	3137.125	763.0927	3013.088	521.9247	1034.055	639.0162	723.6529	3560.365
INTU	36.56707	714.6524	435.2523	87.63325	437.9711	65.44389	110.225	63.21679	112.5083
NME7	636.302	645.3175	37.92731	570.2776	174.4917	37.17446	209.9462	48.06624	37.18708
SPRR1A	29825.08	27156.63	33631.89	24809.81	31025.16	27471.99	26783.83	29785.13	26486.32
ABCG5	138.3107	71.69239	81.46505	74.83662	84.29049	99.75462	61.39001	71.9771	64.87617
NSMAF	667.5773	1725.183	2392.251	1638.4	2675.109	3111.277	2688.717	1208.3	51.32612
NGF	486.5248	281.1198	155.2433	306.1161	176.373	268.0744	170.0316	259.6034	737.8978
ZNF543	2866.341	2633.256	2187.672	951.8291	1152.846	1867.213	1444.797	1311.571	40.60851
VWA2	79.42	117.9284	42.3693	139.4941	66.59795	86.1596	61.18663	61.30441	319.3079
NPC2	20738.21	21001.02	23635.45	23025.14	21600.27	22152.07	22011.6	21496.13	17101.24
C21orf88	117.5969	88.48002	73.57359	72.7243	61.96081	92.55627	62.71199	73.96819	183.3387
NLRC5	53.43997	78.83637	62.34179	559.1683	64.66411	77.78154	98.43557	43.90483	126.9756
C6orf64	61.11119	1348.732	2674.731	1822.953	2746.559	2090.78	3245.704	3157.319	54.40057
FAM44B	6380.049	9428.025	11822.02	8865.136	12441.19	10200.12	11443.23	12535.13	6599.436
MYOM2	144.0984	137.237	301.6408	1151.748	347.7786	336.5823	740.0331	670.8986	99.09634

CD80	107.3445	129.2379	169.732	104.5272	142.9014	46.04771	113.5969	131.9659	64.77329
PRKAG3	107.5577	43.85595	242.6856	37.11317	34.84472	45.33664	115.5676	52.32628	79.97846
CEBPG	484.7608	277.5074	177.2736	268.1916	557.8055	184.4503	297.1287	260.5975	81.60983
APBB2	85.82571	1168.601	105.8175	1225.736	40.85476	321.706	322.5802	235.6575	81.08328
CD302	45.4208	47.00584	117.3928	59.28341	79.56133	41.09493	44.49283	36.32967	37.92223
RALYL	51.43541	37.67873	35.55117	37.8618	46.17604	31.49437	38.39078	31.7342	82.34628
TMEM87A	2801.325	2243.332	2745.44	3128.948	4151.891	4799.675	4577.652	3903.37	1630.042
GORASP1	60.00153	36.56511	322.594	38.25467	1122.647	672.5741	736.4863	207.5789	86.48019
ACSF3	77.0507	49.23312	343.9205	87.26357	36.15203	791.2212	411.1525	56.46407	66.73805
C21orf56	89.87189	831.0601	51.57825	92.87648	218.9009	61.69027	53.08503	47.91948	2199.068
ELOVL2	529.8502	317.2812	338.0679	340.067	384.59	297.922	260.1602	274.1054	769.5939
TDO2	746.4366	347.6026	147.8397	37.95481	37.49646	69.70683	82.69329	37.70725	59.3868
PPM1B	11516.13	12010.63	12596.41	10833.46	11812.85	12403.18	11670.12	12243.93	9280.561
DNAH9	40.90387	40.04363	37.54213	59.28351	45.49676	34.8278	40.8498	38.30775	53.5022
CTNND2	61.491	62.05491	50.14629	67.76383	50.16463	49.90076	61.60712	47.95065	82.51279
RPA3	62.96558	58.77019	247.0091	64.15127	410.224	312.733	675.8991	239.5537	38.22379
FLJ20254	297.4724	141.4382	403.7396	477.996	578.1583	240.269	431.3145	301.6118	894.7637
ADAMDEC:	114.6079	64.81551	83.76554	148.6037	59.75859	118.7055	73.67339	53.79272	215.984
TRAPPC1	17490.41	17553.32	21710.75	20146.43	20502.58	24869.13	24675.42	26422.82	18150.72
DHX16	41.66016	2031.232	3302.693	1295.516	3116.88	2116.17	3308.656	3492.178	3061.943
FSIP1	36.35215	53.13083	141.289	34.75386	45.88634	40.3973	71.64674	35.67882	33.24968
FBXO10	49.44044	55.18251	52.35888	61.62608	38.47666	47.94664	73.95941	111.1386	36.73563
C1QL1	97.86461	111.4339	74.99825	93.6151	63.73648	73.25462	52.10478	83.38024	49.67869
RBMY1B	32.59718	36.98052	46.00614	42.61642	43.95925	44.40743	39.22475	44.63427	67.58788
RNASE8	40.65421	53.94611	63.79496	67.33479	44.66207	42.59044	40.22376	57.26849	44.1105
POLM	70.49384	114.6934	291.2338	89.83473	388.2491	585.5403	298.803	372.0163	239.9699
LILRA2	38.2455	799.9385	1622.671	830.9004	790.8262	1554.265	1602.602	1191.751	42.90265
ZSCAN1	38.14342	45.1267	51.55993	59.90151	51.12248	41.9653	36.73003	40.92001	54.53916
C2orf86	52.83906	42.4444	50.63168	42.37449	45.28109	429.7258	39.45031	37.55931	57.89373
NBN	31.1988	43.97048	44.7621	36.53754	32.25166	36.68238	37.63047	36.18907	65.0027
FLJ33590	5158.154	7336.635	4519.589	3588.981	618.2297	1829.22	1336.773	1953.912	6038.741
MOBKL2B	49.55674	243.6402	106.6754	43.14695	34.33307	40.80268	47.29942	51.01743	33.56601
ZNF561	1707.967	2339.766	1869.785	1182.061	933.3589	1924.754	1177.682	1816.184	2549.767
STARD10	188.5265	240.9644	38.75208	307.7337	48.07932	57.92212	45.34834	108.8464	44.6178
TXNRD1	42.89989	32.20033	40.20459	552.3333	120.5417	256.3886	69.52676	293.5209	34.01134
WDR65	133.4187	267.1287	69.13902	147.9373	83.35838	57.08945	93.93909	114.5084	166.1082
DNAJC16	132.3375	69.51599	54.17949	97.24627	54.32423	286.32	54.40601	59.48618	1822.934
DSCR3	36.01143	42.19668	540.7096	31.53175	430.6657	230.8738	318.302	228.9822	34.00442
MINK1	1146.836	151.7057	130.1546	246.0634	85.78054	161.5704	227.582	219.3092	1469.477
OR10A6	146.1642	79.15224	54.79733	67.6963	70.54964	52.8144	53.17702	67.53455	145.1393
CLSTN1	891.4226	1089.332	280.3051	1871.733	584.2697	572.2863	495.2918	651.3038	40.35223
AQR	2223.159	921.7923	2063.731	1930.476	1495.429	1335.853	2322.461	804.9755	2589.703
F13A1	34.32541	38.41281	497.5676	38.05237	46.43745	35.21333	46.53433	41.68449	1872.128
IGBP1	1152.677	571.5905	548.8754	607.3084	393.2753	367.7664	423.3693	612.3522	33.64445
ECT2	5913.746	5751.35	4587.025	7490.203	4615.18	5270.259	4523.917	5872.279	5312.289
MTMR6	1857.274	762.8411	2262.23	749.2161	2660.067	2699.674	1657.823	1714.873	66.00925
FAM128B	54.86916	55.47372	60.26725	42.5446	57.58984	48.33635	38.17245	45.63811	38.08015

MBD2	69.48492	71.46114	191.2837	74.65959	65.4265	60.72205	55.69737	65.38003	118.0803
CD3G	1214.052	663.9402	742.9429	134.695	313.7896	237.999	249.9116	768.0416	354.0777
OLIG3	134.7459	105.2798	62.96823	126.286	64.33583	117.4685	68.00362	71.91862	261.2696
C7orf70	1206.686	1195.193	681.8275	1843.078	354.2899	672.3255	539.0264	889.784	1609.131
C9orf21	533.7977	301.6069	200.2609	737.5255	227.4787	247.9913	265.4804	143.9703	605.6396
C9orf66	46.49927	39.05446	141.2755	47.96583	108.9595	48.95697	244.7382	104.9642	51.04494
FBXL21	342.2689	178.3531	97.80499	183.4662	91.30287	145.2248	81.44274	104.1897	385.3472
HLA-DRB3	3155.684	2460.574	1847.835	2334.75	1568.263	1984.059	1180.762	2141.832	3028.57
PROCR	1003.599	843.4418	743.0004	1794.191	515.5298	1728.676	891.1561	1700.008	1212.222
FLJ37078	1906.294	1314.271	148.1877	432.5514	191.5903	285.9306	109.6484	210.4198	167.0032
RLN3	42.87146	63.1729	44.68325	50.1895	48.29787	46.13149	43.96548	49.74088	47.42344
PTPN3	756.4506	339.6739	485.1297	559.7372	561.5241	381.3981	402.6457	459.6053	515.8901
C6orf122	61.52382	76.14926	64.50201	94.06245	55.70393	46.7883	58.6715	62.57162	138.4456
NFKBIE	2163.595	3270.782	4422.027	2749.069	1959.503	3367.843	2735.87	3095.664	4049.416
C16orf52	165.4141	79.01749	193.5377	72.08412	114.2844	59.05282	58.94384	54.80044	62.32555
SLC29A3	46.39048	41.63707	42.02501	37.00272	41.22137	56.84663	39.40142	46.74711	47.82383
VAV1	154.3521	133.4623	176.0262	112.8562	101.8731	233.9258	72.48421	88.203	169.9256
43531	35.66851	49.92796	127.8818	42.82849	305.8416	256.4531	227.147	127.7799	548.5293
MDFIC	417.0905	220.7936	125.6014	239.2768	134.8692	220.3304	118.8471	162.553	584.7858
C22orf34	1234.528	1481.061	2748.89	1031.054	1280.377	1487.819	821.2908	1067.426	35.27043
HOXD1	43.23154	42.13422	33.44394	39.71595	59.90115	46.6463	159.9998	153.8286	35.26127
PTCHD1	32.88869	41.15183	36.92585	58.44404	34.94424	31.97781	45.51133	40.32274	45.93946
UBE2W	369.1486	198.1073	111.2034	232.7628	99.5448	155.0625	90.68534	93.65952	525.769
DFNB31	62.35821	111.2987	210.4272	49.92689	51.21169	47.74991	170.6156	57.30307	131.3203
PH-4	63.44535	42.59534	44.18843	34.24277	36.47042	35.44667	39.71099	36.26278	51.02664
NPVF	51.16739	46.47852	40.7182	40.55957	47.74362	51.62755	46.9113	47.51922	178.2535
RPL6	5148.868	3427.277	1146.887	3701.494	822.9668	1417.913	993.2761	795.9631	5791.456
GHSR	39.61886	37.76594	33.04938	52.93355	33.61403	42.88247	41.82343	44.10491	42.9576
CNTNAP3	83.08941	492.317	152.1038	53.4866	56.97436	91.13794	50.81874	43.78505	95.00547
LECT2	39.8268	39.65128	35.4363	40.66727	32.74963	33.88071	34.35067	31.78231	32.15333
LOC49719(	83.68095	77.90338	59.19579	104.4908	52.79487	77.15688	53.25841	69.20097	90.0103
PGA5	40.89943	54.41203	40.66333	73.98967	40.62204	564.5098	393.0238	619.9094	74.34587
C17orf64	324.749	115.8371	63.85595	136.1005	76.06588	117.7585	70.75787	77.75118	158.0791
DKFZp761E	568.5728	300.2466	73.18452	302.0285	106.1144	122.2532	85.45409	76.63943	126.1362
CNTN3	60.43332	87.78281	58.48562	124.9824	57.46986	59.15956	56.76426	37.8544	104.3063
RGMA	3071.177	2430.788	1654.536	816.134	2171.247	2398.509	1635.247	1809.393	3602.309
SIK1	254.2293	942.7108	518.8448	687.8325	288.2357	189.3272	239.1568	120.6351	834.079
PEPD	3125.859	1505.046	2998.078	959.7016	2328.023	1178.713	3231.903	2701.67	133.7008
ZFHX2	40.52856	37.11835	38.16567	39.74843	46.44378	43.40798	38.15404	42.76357	46.49978
IGFL4	41.16703	64.25819	38.92497	35.8577	46.9011	43.67928	37.69267	48.09373	122.4764
KCNK17	191.8314	111.8124	79.66359	139.8078	75.4109	94.38064	67.16491	88.22329	317.2815
PLK2	116.4849	125.742	110.4228	197.9762	123.1878	175.3335	111.3644	131.3454	108.3298
PSTK	4811.697	1738.412	1199.123	1188.16	397.865	1151.722	1022.548	1542.85	502.7152
SLC26A5	106.6694	310.1485	63.38669	67.5225	57.70484	310.2634	69.8885	129.4219	84.98998
CDKN2A	268.2104	125.8975	103.4044	218.0853	117.9748	71.06961	50.55673	131.0739	76.28586
PDZK3	78.27046	430.2557	48.73624	704.1069	122.0515	85.44253	131.6324	152.7941	133.1904
FLJ43752	160.6161	102.1663	79.8148	139.0304	71.52823	101.2923	290.3714	92.27499	117.3768

FKBP7	32.97856	46.23058	42.83441	44.93711	34.50916	228.2931	34.26283	45.2597	46.17727
TIAL1	54.11706	36.10758	244.8421	734.2375	167.5907	100.9013	245.5875	391.4474	35.77773
CALML4	5492.142	4710.281	2177.561	4971.072	1871.731	3753.447	2112.762	1394.471	4911.173
NUDT11	88.11558	43.4581	52.81944	947.9664	186.5952	100.8943	294.6998	43.22083	42.91098
VPS11	831.9573	1179.096	1591.497	589.5241	1153.166	1193.434	925.0264	1301.793	1058.247
A26C3	54.37441	48.6444	33.06941	51.67553	42.25504	37.42463	37.24581	48.89634	66.36221
C19orf33	1225.181	2929.018	4312.448	4143.859	4883.108	2839.344	7338.933	4550.82	4193.832
PRKCE	51.66453	37.34948	35.74807	43.60371	41.09821	33.80947	37.8331	38.45622	34.82362
CDK5R2	329.8785	209.8313	106.3735	185.4006	83.78734	142.821	69.56456	93.16434	517.464
RNF24	43.90163	243.3483	106.7956	153.7499	86.37379	39.46733	61.46394	78.75654	53.77541
EIF1AD	626.5892	460.6253	958.1873	381.2986	927.9764	1282.519	1698.629	1124.576	36.29059
USH1G	657.0071	449.9097	683.9674	450.8849	1046.041	328.8419	213.2816	292.9376	834.0667
DMGDH	72.27094	46.61946	57.06447	65.85879	37.78693	41.87392	39.20495	44.77294	39.1823
TRMT11	33.23059	311.7356	180.3637	272.9637	100.4743	230.3403	111.3317	93.9842	37.48565
PFDN1	45.4219	1296.488	1105.332	1075.336	593.8748	702.0479	554.9286	745.9039	245.3983
CCT8	2704.025	4066.904	4264.809	5490.007	3445.61	3257.364	3107.721	2420.077	6157.796
PKHD1L1	63.6699	93.50518	72.82303	94.9734	81.66549	129.1028	66.30836	91.29736	149.4116
GOLGA7B	52.72776	40.85049	43.66704	59.76626	278.8354	58.45601	226.8196	190.6095	51.41027
ACTL7A	75.49522	52.70168	46.21689	53.33162	53.52913	55.48706	45.74144	38.93622	70.11179
ERBB2	232.8839	135.3455	90.64242	107.0341	65.44189	95.96808	64.44309	74.4554	219.9246
SORBS3	135.8287	56.85038	52.58693	42.23457	157.0052	46.29876	47.48293	192.3493	54.75501
TMEM16A	94.02851	1650.929	1085.638	2984.953	1026.269	2076.813	2457.207	1741.146	88.04975
SIGMAR1	630.6232	377.6183	281.9399	188.4431	155.8356	252.0841	212.6577	136.8218	73.95848
SYT11	8440.4	7858.968	9926.221	5157.361	9804.242	7836.733	8427.372	9984.558	5856.552
SPTA1	218.5177	102.9745	88.82986	165.4327	83.58112	128.2351	95.75835	88.52473	282.8136
DGKE	81.23939	374.6179	976.3165	688.3788	575.1996	577.1168	705.88	1165.278	31.84898
PCDHGA1	43.13657	64.99782	48.63676	50.94735	47.03793	43.07335	33.38979	63.96024	51.46609
TMEM1	200.8777	1306.286	418.6179	449.0742	438.1011	244.7808	259.5494	430.0692	179.2458
CYHR1	178.1065	934.7764	318.1653	1852.342	60.94703	51.90474	528.2187	447.9457	82.35444
BZW1	2785.598	2502.018	1720.805	3089.808	1610.034	2173.799	1860.73	1810.501	1878.584
CALD1	11119.36	8544.885	5314.894	10980.66	4597.531	7015.519	4823.858	7785.995	11818.38
CPSF3L	810.7209	77.73878	788.4158	2167.343	1129.344	1625.224	1047.005	1203.315	132.9544
PFN3	96.80985	138.0166	59.49627	65.20969	73.92355	89.98309	53.81274	59.27886	90.39715
APEH	35.25399	42.08025	1854.042	1707.92	2716.542	629.1124	3198.061	3126.577	35.85277
ZNF549	415.7271	231.1744	252.7527	392.6818	143.8786	474.8701	201.7293	260.3271	513.9612
ELAC2	100.077	60.64969	429.8856	848.3294	915.918	52.98788	577.5316	736.7204	44.70298
ARNT2	47.82109	59.70392	50.8604	32.25718	47.91591	75.25054	37.22725	40.48786	61.51939
OTUD7A	149.9656	104.4534	64.90099	99.73055	62.41125	70.06857	48.0491	80.79286	141.6719
LIPA	698.3612	83.56242	121.9437	76.46742	132.7644	196.9756	141.0311	118.8531	733.9672
MIS12	344.6478	162.0055	120.0785	251.7478	139.0032	183.5659	160.2998	171.343	119.1564
CASP9	85.90833	122.772	635.6276	39.09692	337.642	70.55084	503.6614	249.7999	69.20524
AGTPBP1	510.9218	253.0661	269.8415	125.0043	203.6025	260.204	198.9641	213.7112	124.4873
HOXB1	98.2914	134.0397	75.24984	140.648	75.30444	92.89153	59.60381	83.30164	269.1674
ALG1	104.3278	506.243	60.32268	58.74646	55.27686	251.1167	44.18316	36.52055	58.0434
OTOR	232.4769	133.2636	67.14732	120.4299	74.30003	107.0193	65.70006	58.02303	251.4808
MAP2K5	53.80555	55.59859	1601.096	39.20848	838.5091	941.5567	1605.979	2571.085	2147.291
ECHS1	86.2656	842.7037	2771.781	63.97871	3323.205	1629.919	2772.696	4263.096	73.49706

TRIM42	100.6568	128.0997	67.85157	118.0212	71.13291	119.022	70.57135	75.69705	230.8125
DEPDC7	64.41253	63.40929	174.8291	82.07709	53.00558	191.3648	69.37525	113.3779	38.81666
MED26	1110.262	101.159	53.33477	116.7145	51.54101	43.09523	49.26576	59.96561	297.8351
FSIP2	79.84084	76.05354	53.31079	101.2448	44.77318	67.70014	62.01767	114.0656	105.5924
PCDH24	262.3261	198.9002	106.3394	209.2731	83.78664	130.2056	87.37263	130.4366	383.2706
MYLK3	1667.685	414.1516	43.62893	54.14644	120.8927	173.7884	129.4833	112.0396	1308.844
NPY5R	64.25885	52.39251	49.04501	47.3466	56.85413	51.60927	39.40633	31.37805	59.8389
KRTAP4-7	43.93103	1129.744	52.52368	48.23321	2853.532	439.1331	1062.084	229.1783	35.44816
KIAA0100	4305.8	2483.173	2816.651	2368.087	2201.762	2879.355	2509.707	3250.979	6116.527
SETDB1	49.18657	2116.836	2791.787	892.6791	2967.552	2220.936	3757.546	2098.348	3938.692
OR13H1	67.88956	112.1343	51.09645	85.97604	63.05439	103.4918	61.6772	56.72739	235.9948
CFHR5	301.701	190.7156	98.34293	215.5709	73.16092	126.172	75.27905	111.3944	418.9609
TAS2R3	121.5785	70.38964	52.2818	101.6496	48.23334	68.51907	45.01431	60.51305	133.0043
ATG5	96.76676	262.8983	160.9561	377.9388	112.2113	205.16	122.4439	135.8359	67.38809
DGKI	39.52502	55.18434	159.086	65.99266	37.22209	326.2472	55.8461	165.2708	52.40078
GJB4	129.7785	125.7844	82.35957	390.8047	109.7853	115.2519	73.06315	84.77747	103.3369
MTIF3	607.5572	528.0112	1818.083	908.2194	1356.33	2112.206	1382.149	2343.874	1460.881
HOXC8	133.3738	90.50191	49.07031	71.85235	40.03043	53.48865	54.15199	51.91812	115.2087
STAC2	212.9768	174.9442	108.6266	169.4455	82.29694	128.17	83.91447	110.3788	259.1446
EML4	328.0527	231.1977	422.14	210.6037	500.0633	368.0654	457.7233	570.9659	265.1466
PDE6G	822.0924	813.4105	863.0393	448.4232	164.4705	1095.602	397.8907	519.1332	832.3722
PVRL2	209.25	186.4533	790.3241	701.7833	175.8627	1596.958	219.9134	1079.925	2401.349
COX15	44.24335	2893.289	5439.339	1908.516	2551.563	3498.187	3121.693	3887.224	39.49333
ITPA	1646.36	1511.372	1391.755	2081.001	1283	1601.84	1222.329	1351.562	1188.05
C7orf50	10269.28	12726.12	12268.21	12140.32	9719.175	11277.11	13604.07	10287.87	11222.6
INTS7	611.9501	509.1554	564.8541	423.265	500.9974	675.9374	526.2247	449.2772	94.53753
LOC38891!	38.12709	34.23539	33.08315	32.31473	37.37006	53.59388	42.23732	35.91557	46.26402
LOR	12873.41	17742.34	3135.501	11086.06	23695.83	5312.003	1483.687	4948.479	9498.19
LCE3D	1181.143	1826.158	1117.045	727.9601	8129.366	895.527	2293.107	5784.441	43.56303
DOC2B	2599.968	908.8235	1293.846	37.70632	749.6349	38.28928	246.3803	220.0425	2401.716
LCE1D	96.05579	93.83978	42.13898	97.77946	57.19996	107.819	40.45718	34.88407	267.766
MRPS28	193.2406	146.3349	72.75517	137.9952	107.2672	94.12922	206.4078	135.4113	143.1121
FAM69B	194.7409	104.5015	74.34531	86.50154	75.13452	92.60827	55.9281	79.12594	187.3949
MAGEB18	34.66877	57.30463	35.1395	37.07267	41.66877	70.96843	38.43309	40.44568	42.77729
NSUN5C	108.2665	66.36392	59.06431	116.4258	55.51079	61.3333	52.40834	48.09589	108.0323
UPK3A	570.7261	361.7516	247.7169	379.7798	283.318	394.3718	213.2835	424.3923	893.5474
MMEL1	62.65615	39.81533	52.54297	43.28653	46.48507	57.40005	35.47338	56.2444	34.99902
PSG6	53.06547	42.51107	100.836	509.7965	55.89393	73.69188	380.9868	60.43317	99.73854
SGCZ	100.4533	74.7114	70.81064	101.2496	52.5979	71.22237	56.55712	83.70229	45.762
C10orf59	163.2206	538.2875	774.8272	566.5882	576.4957	1768.21	1224.941	1504.719	245.4642
ITIH4	39.43523	38.42949	44.46984	789.1927	41.71123	727.1523	1125.124	659.4813	60.17371
HPS6	36.36442	893.2408	1558.956	995.6326	1068.92	447.49	1613.769	1830.555	53.31534
CXCL13	606.8299	299.5739	952.0726	286.5211	36.02448	461.8495	214.8298	150.8974	833.9926
PARP12	2132.371	1135.584	5211.77	995.9391	2145.154	1095.824	4211.332	2829.664	68.66559
CDKN1C	67.53847	57.21708	56.9291	39.97879	113.0774	49.22961	40.47844	51.98077	85.94832
OR4C6	60.65193	49.84918	52.1824	39.92131	43.41498	53.69868	35.05444	58.4395	58.78876
CYP4F8	41.57225	255.8256	45.06613	249.7316	36.0741	33.34145	38.72081	34.64655	46.32632

TSPAN4	14485.64	14751.07	10369.1	16427.74	8447.17	11189.43	9138.925	6709.677	15869.08
ARHGAP17	3735.845	5455.822	6359.676	4114.025	8858.646	7734.363	8579.287	7815.394	2184.825
TRIM11	920.0039	1077.163	1823.505	1326.708	1676.585	2004.381	1494.119	2001.468	1573.704
TSPAN32	6313.77	6123.386	5204.407	1356.579	1245.698	3333.47	1249.109	1866.517	4917.854
NRIP1	314.7086	207.435	200.46	232.6907	187.3162	257.5357	126.5103	149.3575	370.8804
LRP5L	1484.187	1397.533	3028.007	2373.531	1491.751	2395.17	1314.488	1029.248	935.1852
MMP15	202.0123	137.5467	193.4611	175.043	97.29176	76.51647	73.18833	101.4347	271.4507
ZBTB26	68.85816	56.86012	51.62181	372.8528	70.60365	55.64116	77.27562	34.27494	37.9965
GJB6	672.8109	615.5057	256.0629	583.804	563.0925	797.3776	564.6869	844.7034	940.7068
NHP2	798.5043	108.6571	298.0367	341.3172	1144.038	884.5876	347.1308	339.0679	258.3372
USP19	56.56504	48.6598	36.46949	35.78329	37.81341	40.56108	38.26985	67.93918	66.9887
TPPP	95.6582	121.6376	65.48283	70.00827	73.22317	84.52087	60.64722	75.19112	128.3779
ERAL1	15433.17	19573	22603.47	18640.07	21321.98	23454.59	26071.45	15384.29	14143.6
C20orf177	103.3648	103.4537	38.54887	45.56923	52.73888	56.5091	79.68726	72.18789	82.91954
KLK5	1195.386	507.9959	1696.266	1583.629	4264.026	1158.006	1932.396	1422.554	1564.276
MLLT6	8504.94	10867.48	11369.39	8723.685	11456.65	9951.157	12166	13472.42	8161.483
CD207	54.31129	41.90766	1055.376	39.73972	1286.039	1143.32	545.1331	1716.997	39.37238
MT1F	1887.599	1781.534	2290.249	3481.72	1738.715	3540.087	3946.396	1755.964	2584.606
FLJ36070	59.06134	8252.17	2408.779	1871.493	4615.162	2629.044	3098.064	7553.576	4389.831
CCDC24	52.97435	50.57511	216.1245	610.1651	41.63649	390.088	257.6903	224.874	31.92613
EXTL1	126.3307	99.30486	71.98917	151.9665	124.0376	107.6505	92.82801	84.93818	334.3947
XCL1	38.74173	43.66496	33.4135	36.61432	33.48405	108.3491	38.55617	41.61705	36.43189
SMC3	1547.804	249.7304	129.6519	265.4552	339.6982	935.2372	247.9069	811.1844	1957.631
AKAP10	399.2195	559.3159	349.3528	87.66762	215.6833	695.6813	255.6817	274.0042	1501.076
CNKSR1	1035.818	534.1957	1107.387	206.9471	799.0479	1694.318	1034.922	1419.706	1319.636
SLTM	57.25539	50.73609	119.1057	34.55769	90.18954	31.37044	75.49997	61.24042	745.7571
TMEM57	1623.293	746.0761	1144.39	576.9178	1640.887	1016.236	753.8769	1563.673	78.73875
SPHAR	93.66806	44.83822	400.4604	370.0555	383.5455	475.8098	428.8401	281.0331	100.9563
QRFP	38.01154	32.55583	39.37442	40.17247	41.47317	36.66642	39.82148	50.68424	34.60778
PLCL1	36.96309	74.70361	47.80418	46.03924	45.78618	48.3978	45.81437	33.99384	34.06013
UQCRC2	542.7608	252.7446	121.4915	249.5989	135.3277	144.5054	108.2067	157.1302	532.3389
KCNK1	111.6393	44.92125	207.793	293.7129	341.6095	189.7275	202.7182	157.8842	68.4285
C5orf46	141.6194	40.22919	41.4514	58.95555	572.1149	78.53824	113.1393	245.776	42.46801
FSHB	285.9281	104.2297	72.59969	150.7961	74.98714	101.6272	61.25992	66.41849	263.5051
MACF1	1613.833	1512.921	608.8223	1329.764	627.649	1066.122	814.6208	556.6434	1164.894
CNTN2	49.52022	34.63525	32.3705	46.39267	31.54721	238.7512	39.33883	45.83144	44.17168
HOXC9	1047.216	732.4171	324.9442	2576.654	420.0903	899.4714	545.084	441.0628	38.97555
KCTD2	251.5558	220.0758	113.6592	67.35025	97.03977	198.1749	94.38523	148.9704	156.3368
TSHZ1	38.72385	42.83571	47.06173	62.32993	117.7705	34.03197	149.0336	36.64946	152.7601
SOX5	144.8576	84.9502	48.36401	90.40332	57.44116	53.27029	61.56431	67.7866	150.0135
ETFA	522.6284	300.06	342.2994	312.2413	253.4152	328.8363	255.6192	267.3292	637.0024
C14orf49	1144.83	166.2596	83.97926	193.9019	75.53298	116.4575	69.39999	89.02835	417.0914
CRYBB3	70.15861	37.04425	534.5429	39.60205	42.00337	44.38868	111.2792	384.8949	35.15678
ADAM10	321.8768	630.3375	506.2115	698.243	304.6386	1160.472	464.9447	613.9658	325.0057
SLC39A12	99.3838	87.11892	57.51102	68.4285	69.07246	69.0742	50.80375	60.28375	62.0384
ZNF433	1499.477	42.29675	1013.992	337.8968	499.7089	447.11	594.4903	237.0479	52.58433
SERPINB8	50.59599	78.78201	293.0443	69.64736	524.1661	472.6804	314.834	600.7866	69.63467

BNIP3L	210.7467	143.9797	260.9665	382.1474	115.285	130.142	264.1138	116.5791	181.4728
TTC7B	67.8085	51.95673	49.94059	112.9061	47.2361	49.85771	48.99629	57.1685	55.62265
MFSD3	6145.632	8729.922	12017.42	14320.96	10660.63	10355.46	13445.05	16031.85	8365.831
GADD45B	4793.806	5512.177	6252.935	6423.12	3939.915	4444.71	5565.082	3843.935	2794.914
BEST1	43.28055	103.0327	88.70847	108.1048	79.19462	75.2678	62.17006	62.43587	173.2362
FCGBP	276.0939	280.1644	217.9258	154.2485	219.9487	461.068	101.5135	328.6548	415.8001
DAZAP2	44.98613	1064.601	999.4175	539.2511	2701.765	596.921	2720.608	2064.635	47.87228
NFRKB	43.18671	64.7164	42.18087	75.27921	481.4155	38.8198	307.4582	790.7028	46.58068
ARL4D	165.4143	84.8538	1110.177	63.35435	1156.446	606.9427	939.7751	1306.763	370.2114
PATE1	357.3399	145.2985	83.56559	170.2954	98.64802	127.3018	70.29448	88.97731	348.6169
UBE2G1	49.05625	38.48702	290.277	1278.192	1642.432	359.5364	809.3447	944.0654	35.28968
KCTD10	88.51704	645.8996	1754.799	523.3433	2920.309	1242.772	1617.932	2411.416	1113.673
FOXN4	42.79066	82.77022	47.88105	46.87959	49.97409	59.31265	45.81279	45.47704	144.0549
OR56A3	89.7785	31.80323	40.49015	40.33321	36.94601	41.3342	35.86514	33.47097	67.00744
KLRC4	208.1183	108.8577	69.10668	115.1081	70.20998	90.40144	55.64449	83.94984	69.50303
C18orf8	61.45003	113.2745	213.904	119.6152	117.9413	201.0427	91.14149	134.9632	99.45412
UBE2CBP	195.1578	109.8411	73.82777	594.2565	117.3976	110.7178	58.90351	91.79017	167.3694
KRTAP20-1	42.67907	79.45758	42.07698	60.91659	38.72271	34.7919	51.95515	35.1744	60.89931
CRX	198.2623	167.0561	85.63231	176.7642	82.72608	89.78449	73.77958	92.43176	262.2111
APOBEC2	57.69581	362.4754	163.2846	54.65469	371.5145	399.2413	682.5708	861.6111	42.16725
CTBP1	72.29654	306.5666	648.1468	63.63227	691.3239	602.7966	1039.319	936.6885	531.9689
ZNF557	3455.248	3286.457	2475.097	3252.127	1077.357	1928.449	1365.343	1935.486	4051.289
BRMS1	149.8398	1130.585	518.8315	65.59489	50.9202	1105.47	261.0453	362.2027	1505.51
WBSCR27	67.86954	44.32708	49.8467	62.25711	284.5235	61.64036	340.4953	60.48888	40.79922
ACOT7	49.47872	47.43642	57.991	528.6715	38.16211	47.0075	41.07534	42.46349	147.596
CCDC9	311.1889	143.4421	58.57664	129.5004	105.5829	48.67802	73.70274	75.52627	219.8682
IBSP	311.7195	139.1508	70.26659	151.7326	67.74999	105.8476	65.91798	78.76376	345.7769
TULP3	245.3304	113.3732	72.84116	134.6023	54.61803	78.89947	134.4701	205.7939	115.7109
PRPF31	62.08479	69.23225	310.2487	120.4857	713.4937	489.1993	794.9138	841.7419	43.54119
FBL	11544.52	12700.08	16367.32	14611.95	17573.36	14179.12	17608.27	17052.99	12136.76
EDARADD	49.23193	38.2136	33.646	43.03127	32.66154	34.22918	40.30994	35.72581	58.77412
ZNF616	64.87973	744.4029	43.50798	89.9408	612.6963	406.2473	700.0423	397.9341	80.27673
C17orf45	5132.959	4388.427	9427.117	7081.604	10124.71	9076.136	10522.47	12091.93	4150.56
DGCR14	76.04206	65.37434	39.18877	35.36819	118.0379	34.91561	74.89804	156.2811	57.54829
CDH5	1066.127	735.4413	388.0766	933.8132	622.3666	552.2468	920.8076	509.6528	35.67104
OR8B3	58.54765	34.05663	45.51662	54.65551	39.7223	43.75351	39.72535	54.35771	42.79024
SLC44A4	191.122	74.90673	72.34078	157.8	65.05293	79.81717	51.74366	66.85046	177.7619
TRIM9	389.9332	462.873	223.2154	271.7339	109.1215	327.1703	186.9211	276.9006	1215.154
C9orf163	204.8387	147.0819	88.09135	158.4232	61.0012	86.47832	72.40849	89.55201	163.7303
HEATR7B2	56.74172	39.61833	49.21938	32.84556	52.90922	85.78903	46.76545	50.77554	86.03293
CTSA	3839.095	1707.829	1254.411	2182.239	874.5054	1598.568	1084.047	1019.669	2439.872
OR51E2	116.7245	46.57965	51.12913	57.78545	51.56707	54.11101	44.07588	66.61065	103.9465
LRRC39	69.85698	188.6371	84.39478	126.2422	59.90766	82.02401	69.84039	77.30503	79.68777
TMED3	18726.68	17886.63	18574.1	19187.7	18784.47	19425.33	18482.85	18452.82	17945.51
WDR26	160.8842	560.6809	145.2615	350.8896	178.9292	107.2927	183.6701	113.943	94.94268
LOC73082(	200.828	62.55391	92.27387	90.0223	146.8634	93.6105	245.1387	164.4476	52.53352
TCF21	259.3815	113.2521	79.74079	66.0713	80.7286	142.0356	68.93804	82.36208	133.7397

FH	16646.31	17209.67	18514.59	18385.96	18241.96	19659.23	17671.05	17209.59	15773.83
TRHR	116.6476	59.19647	62.05844	63.79978	46.64085	70.89721	38.934	47.20338	83.7023
CD1D	85.45439	96.2066	53.80192	66.81809	53.74905	61.10604	45.29258	51.52402	133.8385
DCLK2	60.30096	43.94546	46.43421	59.7663	661.1882	509.8707	34.25729	41.7276	35.05103
CRIP3	229.7862	117.0766	78.07754	168.6738	127.8964	93.39785	69.43639	98.12904	151.2109
EPM2A	750.8382	459.9482	236.0367	391.0325	228.7204	290.345	248.2485	322.2671	867.1876
MSH3	67.44867	316.3683	324.4478	144.9104	199.875	513.5862	334.7087	688.1263	82.8084
ABHD7	301.1329	147.8154	65.96838	175.1447	87.01729	104.5293	97.02268	78.84258	547.2935
CTTNBP2N	61.03103	57.74729	122.5656	63.45155	100.3051	156.9833	120.9379	99.8353	48.04191
ARF4	513.1081	383.9645	462.5884	337.5108	1150.859	746.3738	1010.308	452.4542	697.4247
PCDHGB4	38.51006	47.78064	57.08503	59.38485	69.04559	58.66707	34.99916	46.78897	53.20069
MAF	321.3064	1186.422	2589.528	252.3996	5437.041	2817.373	6145.264	2598.114	2775.879
INHBA	3781.33	1175.62	463.6676	4161.175	74.90678	898.4573	592.068	662.15	1771.382
BICC1	32.84405	58.28316	150.9674	35.05211	36.99492	53.33885	101.4013	57.92911	90.06934
C10orf67	37.24905	432.5628	323.1484	456.0898	534.9534	672.2883	1071.058	799.7717	1237.922
MYOM3	69.58064	4353.656	4547.34	2945.682	5580.57	6935.112	13550.05	12627.28	3134.991
SYTL4	4627.531	4703.554	4105.205	2133.223	3634.7	4335.971	2568.2	3076.017	5178.244
CCT6A	37.23109	213.6035	111.7018	287.4717	104.0575	127.5402	139.7929	114.6873	34.32101
DNASE1	65.80215	38.6828	276.7386	49.75157	257.1638	46.44456	186.9171	439.3301	54.758
RABEP1	35.83295	35.98821	49.08718	55.34718	122.3885	67.6301	34.77473	42.98463	68.34547
SLC27A3	4897.352	6221.899	6220.057	5348.162	3990.624	5161.114	4683.044	4366.391	4137.602
SMOC2	108.8363	73.34667	64.29759	92.27384	307.0135	106.1462	217.5733	217.1166	145.1737
ZIK1	58.67123	50.35372	205.7953	46.94647	42.48891	47.81986	41.3497	59.94318	46.88097
TMEM63A	63.27689	65.75275	40.66272	56.26861	212.1737	55.14282	122.272	257.1939	77.07745
MRPL38	56.59449	1698.7	3796.269	849.0635	3894.509	3034.633	3527.399	7415.546	65.92085
SEPX1	3393.477	4015.129	6676.353	4707.435	4296.567	6026.812	6968.098	8351.69	3587.053
PTPN21	881.4096	549.8121	216.1091	814.3943	109.2726	45.13291	191.5941	200.9104	57.80813
MOBKL2C	105.6954	49.60492	38.97991	69.08073	47.37971	46.59805	40.6572	53.73385	50.79899
NFKBIZ	11518.14	18331.19	11506.42	11331.47	11672.17	8134.494	8709.326	8013.61	9912.872
PLD1	2401.282	1925.061	2289.422	1850.36	2039.392	2470.515	1444.835	2188.749	2033.559
PTGES3	41.41826	380.0018	1094.495	1359.656	1666.789	661.6563	899.3019	961.5314	970.3077
VGLL1	264.0872	104.23	94.92832	167.569	98.29443	137.0125	91.68252	79.70158	140.105
FBN3	76.17963	506.3576	49.59488	75.58168	138.5124	65.58569	43.85585	70.38002	61.75169
TNC	4360.74	2725.739	1974.393	1794.855	2522.32	1220.539	3443.376	2548.749	1528.612
MORG1	1466.191	1876.656	1521.893	1100.615	969.2931	1409.583	1409.462	1646.696	452.7316
UCK1	57.85062	58.42191	43.58617	57.7358	36.08871	70.03368	48.24063	48.46245	66.60376
PHLDB2	744.1332	527.8795	194.4487	974.2091	246.3671	372.4716	350.2385	354.2509	821.6971
MESDC2	74.64098	1904.974	2451.872	2177.184	2723.331	2217.791	2633.268	3636.411	186.8271
REM1	65.90092	2246.122	1060.104	861.4053	1301.296	580.5475	1214.704	875.3829	2488.727
LOC44195(	41.16463	46.22568	32.65275	44.71239	41.11248	37.69177	40.51604	58.18875	38.31593
OSCP1	83.90382	91.42439	45.65844	73.4543	118.9368	56.33696	46.42968	43.34344	69.59354
OR3A2	101.8035	57.31064	52.11312	59.3079	57.39912	46.62005	43.91386	72.4773	88.54481
LOC38843{	48.20168	40.84471	36.95066	34.91489	41.56992	33.81216	44.18814	42.46037	31.71456
GRIPAP1	3682.46	46.9021	573.3438	50.90813	46.76242	614.9117	41.30251	299.8552	35.48249
PACS2	1834.17	134.0688	62.86594	1036.363	376.1891	69.84691	75.80441	266.4708	44.39452
RNF13	83.86046	474.2161	168.0037	513.7686	76.43443	92.22936	102.9729	188.7715	112.8024
FAM72D	2273.925	2194.581	3045.57	3633.115	2935.906	3321.708	3883.631	3179.776	2650.827

KIAA1804	86.64066	42.94961	51.96577	34.10378	42.04012	40.84211	33.81846	41.41275	78.68427
CYP4F11	6512.943	2507.123	3485.777	2288.145	5912.732	2054.61	1541.715	5018.988	4784.355
TLR10	35.00573	51.88355	35.51022	34.44485	42.04942	36.37681	33.87257	49.53179	38.70413
HADH	77.11648	84.94751	263.2338	67.37801	461.2727	395.1189	339.7832	241.9241	81.14512
KLHL13	41.76565	67.79562	77.47337	35.78608	245.8069	58.34356	39.22128	46.12945	876.9178
FOXE3	36.82858	39.41692	58.6126	71.23817	33.67019	41.14767	41.12508	46.87244	41.73826
FGF11	3061.823	3758.299	4774.775	7216.749	5037.352	3012.38	5014.744	4511.408	4200.571
PLXNA3	1067.492	670.9845	744.9943	594.2612	1406.315	953.6053	585.158	822.5997	59.67835
HOXC4	666.5189	1898.857	340.9628	2233.023	185.398	876.396	232.6797	230.5448	1038.888
FEM1A	14438.76	16458.45	15914.9	13932.9	14502.49	15887.58	16535.77	17937.46	17257.32
PIK3IP1	909.6907	784.4427	2184.038	515.2291	1330.653	1975.962	1722.967	1210.841	1164.656
HAL	81.89165	59.77327	45.72864	64.05907	604.6844	135.6494	94.13921	220.6487	35.12009
LOC440295	3577.778	2069.616	1982.452	1692.329	1623.637	2515.568	1384.028	1482.496	1451.187
C9orf45	1425.775	2841.647	2967.044	1025.983	3649.462	3058.709	2104.508	2889.695	90.19411
ZNF23	168.4473	1207.807	101.4361	74.4809	221.6209	69.45232	352.7037	87.93044	117.8531
CCL2	2774.059	1193.106	1493.737	792.8911	948.9418	1377.18	1474.99	490.5243	1856.574
ZFAND3	240.3345	54.61833	109.6975	185.2441	94.24126	150.5125	95.48166	119.877	121.7563
HMBOX1	293.3756	164.9668	491.1854	192.6143	485.1515	384.3748	564.022	487.3937	891.6046
EHD4	43.56686	59.743	62.13027	60.93781	255.8745	68.06534	126.0038	57.65004	39.96203
SFRS12	885.7613	863.7145	1230.932	824.3776	1025.279	1238.371	825.924	1117.133	1014.882
PCP2	57.59497	98.31186	76.47567	95.58365	64.60514	443.1973	44.55997	68.50482	173.7957
PTPRO	7320.455	5306.127	4442.452	4845.515	973.5873	3995.917	2523.803	3264.399	6177.473
GMNN	46.91362	32.98999	366.7728	38.45426	390.0322	46.62449	453.1231	563.9001	40.66002
RCC2	6384.439	7818.933	9672.915	7633.066	11203.35	9026.879	11613.15	11022.01	6535.131
CABP2	99.17481	64.42822	52.10215	90.63855	50.30972	85.07468	42.06531	51.7082	127.6144
ETS2	76.33083	48.85852	46.94682	102.1431	208.5012	258.42	175.1258	48.30115	47.82034
ZNF117	55.50624	42.08648	48.61838	56.38823	42.41196	59.79966	58.3453	51.15794	48.22534
ARVP6125	105.2974	106.588	86.60353	160.5606	72.3924	103.9351	65.31417	67.66965	156.5703
GMPPB	84.70795	70.11923	45.93011	93.32301	64.29681	77.25758	52.52332	72.28537	174.0939
TNNT3	3804.668	6958.276	3201.366	4561.558	708.7623	2615.188	1684.747	5513.075	4808.395
CCDC49	35.04663	47.04941	1087.684	36.88923	1377.124	989.3087	1088.317	1101.349	41.58909
ADSSL1	63.79418	1787.907	1379.818	60.67349	1014.612	564.8456	1023.787	2122.192	1904.764
TRIP12	1928.368	67.65742	660.1495	68.12949	949.3669	483.2541	846.2421	606.6809	151.655
IL25	581.7639	408.4209	336.4904	384.1701	235.6649	358.1513	272.9027	439.175	751.5372
SPANXN2	58.98874	48.65625	35.50171	40.63213	39.8061	57.38185	34.92949	33.83478	31.67913
LOC91431	1763.049	2830.981	1751.456	3478.941	1107.052	1145.535	1494.891	2184.127	4951.804
TARSL2	123.2325	662.3544	659.6301	1132.367	420.7902	840.9564	1317.473	1237.057	98.77525
CDR2	436.095	246.4896	691.3687	846.8035	1059.458	1341.035	1435.135	558.9495	1015.325
C2orf53	68.05748	88.14178	59.12554	94.67023	54.78435	74.75491	49.54	44.20808	104.001
NQO1	8794.102	5750.991	8416.497	5448.321	14340.43	11049.24	13298.17	14020.46	5589.109
E4F1	10609.49	13704.07	8204.885	10963.02	9484.134	9809.823	11171.93	8596.598	13368.32
OSTM1	35.62339	33.8321	157.0107	42.1401	118.0216	43.63992	101.2722	41.55571	34.85008
SLC25A46	625.7337	496.8511	499.009	322.6299	593.7644	332.84	414.3088	427.2472	41.08608
PTS	71.76109	272.5095	50.48376	63.43181	89.76781	66.62834	85.80336	231.534	53.92558
SLC7A5	9223.648	11152.65	14262.77	20346.2	19189.69	12430.28	21535.2	15202.38	11656.81
LOC653602	59.85774	64.49415	68.55202	40.38515	50.32703	63.91695	61.49947	52.2274	76.25639
HOXB9	106.7861	54.36292	52.92705	41.87142	51.48835	54.25413	48.25463	52.03399	54.80801

G3BP2	464.3713	302.162	353.213	585.1495	199.2336	248.4787	223.5807	150.2379	93.31539
PPDPF	3498.14	56.87941	821.2433	1762.229	711.2479	276.7345	460.0938	408.9991	4262.891
ZNF420	46.57256	31.29218	185.1173	32.82998	153.2899	212.9485	142.709	33.1109	34.321
FAM3D	1557.144	710.0334	3054.414	48.68304	4168.442	1678.64	952.8798	1494.442	3447.847
HELQ	112.8024	139.3013	66.14581	125.4587	57.0246	59.91018	113.1888	68.65327	66.45444
NCBP2	1206.899	401.6909	629.1999	563.6431	340.0722	504.9073	298.1112	709.6533	828.9672
SLC17A4	40.4757	40.35608	36.17352	37.73269	43.99729	38.93988	34.05176	36.22685	46.74821
PHF2	1642.032	58.9822	721.6751	859.028	584.5303	65.42561	409.8292	88.44189	118.8397
CEND1	76.4225	110.1709	85.88116	192.1371	55.12374	63.42668	52.11869	187.0716	137.3839
CST9L	76.95519	36.10397	49.67479	59.62154	38.38852	53.55716	34.44159	65.06232	55.30849
GUCY1A2	50.71637	74.97609	281.8876	439.6005	70.51915	68.6759	233.9972	134.5082	84.76926
TMEM120I	377.5425	410.5176	107.1044	235.8746	365.6027	136.8118	250.8854	449.5032	446.0484
LRRN1	120.02	67.68948	59.09152	86.89581	54.47126	274.2484	53.71048	87.72043	64.23794
SETBP1	10222.28	10834.76	7357.978	10363.58	7289.267	8712.909	6167.913	7778.268	10225.22
CENPN	55.50403	804.6567	3620.202	4104.87	3584.883	3865.511	6091.457	2367.91	73.07919
INSL4	189.8819	81.27295	58.80796	129.6881	84.7275	95.08959	55.26636	56.01609	103.4806
POLR2I	34.69298	667.4133	423.631	57.63455	902.9827	417.8239	641.0101	239.8114	38.92566
PRAF2	1444.002	465.0849	1082.438	915.291	957.2128	827.4279	1056.636	1370.34	515.4927
MDGA1	6074.966	4325.964	1355.855	84.21077	2456.671	2106.753	1710.045	2729.226	4213.479
ARSG	250.3092	211.8559	206.2244	179.4819	79.65685	364.0182	136.8857	366.9989	390.416
LRCH2	75.61256	128.2713	76.61385	130.9171	62.43023	82.94256	163.6393	76.2064	205.6972
OR2T6	78.21526	101.0659	80.78617	96.91072	60.12526	62.52961	56.16098	54.9183	41.35564
SYTL3	47.25204	867.7316	446.8159	46.83249	667.2759	53.77713	442.5825	543.4423	126.2485
MAPK14	307.7962	166.6907	82.58521	189.5087	84.66531	126.0202	68.17886	73.16683	251.8871
OR8B8	54.24103	55.93649	51.93867	62.41439	57.46376	52.67514	54.52065	68.27379	90.83914
LOC73083	37.17601	51.93022	281.3984	40.10361	91.28982	42.56298	36.97101	59.08489	32.78502
SLC25A3	1127.619	404.8703	160.7185	554.9873	200.3603	649.1747	77.83062	135.5642	136.4643
CSNK1D	10835.84	13384.67	19261.63	15867.48	20107.13	14377.25	20602.77	17769.98	10174.25
HSPB3	4110.053	6094.658	3964.75	5140.715	2182.749	5429.773	5105.677	6766.472	5117.625
TRIM72	223.8712	320.3926	95.38588	383.7044	86.42756	102.5622	93.47192	203.1487	266.291
IFT122	306.1249	190.8425	86.70831	162.3744	114.5081	133.05	96.74841	118.8022	152.9301
C10orf71	478.1688	293.6251	108.5022	57.11291	83.81535	95.09164	169.8449	262.9207	634.9576
OR5AK2	54.66759	65.13142	53.54221	87.70073	55.98392	47.13515	53.38064	37.01435	152.2319
STX11	33.8496	35.57907	439.9538	33.80967	344.1368	334.6317	248.6955	126.4628	34.95841
SNORD4B	50.05492	70.38242	399.9841	59.08782	371.0394	629.7052	310.9475	130.6406	36.89556
TECR	93.87443	75.17726	53.16908	54.42517	776.7004	81.4239	534.7061	1039.783	58.84536
ZFP36	530.4595	117.0537	131.324	151.5916	206.7556	160.7696	126.7768	96.40168	87.78366
C2orf48	128.1671	623.4966	76.07263	310.4096	54.94734	71.61745	50.74008	210.0345	98.61797
DECR1	2517.762	2787.45	2941.317	2491.656	2773.014	3074.867	2906.17	3650.676	1968.685
SSX2IP	37.69542	39.87201	39.27987	41.41062	121.3817	66.11065	40.98516	34.16779	44.98841
PYGM	133.0885	904.9421	176.2821	107.5983	937.2872	545.2742	1357.953	2259.476	259.75
BAZ2B	2940.509	3191.114	3242.464	2423.393	2728.565	3372.284	2735.962	2202.343	3962.166
OSBPL7	384.4859	640.1616	471.3391	229.5393	817.9717	783.7891	983.8521	1229.092	478.4771
C10orf62	36.15379	65.93729	54.13958	69.57484	49.1307	54.42454	44.49647	46.50496	50.20581
SETD7	617.4736	300.668	55.43946	53.69473	91.88322	44.34178	130.7879	92.8503	31.03048
PSMD5	1966.816	2406.577	3561.087	1641.029	3883.293	4453.91	4186.632	3203.237	2237.304
FKSG2	49.74985	42.7395	43.99368	34.78973	40.71614	44.9375	33.81019	48.22619	54.80865

CMTM4	1701.142	755.3237	1157.537	2884.708	321.0866	1033.848	985.7831	1227.186	2266.228
VCY1B	34.55982	61.68148	33.40313	59.99079	38.92285	55.12181	42.08488	35.73839	53.63507
OR13C5	42.7985	33.29581	37.27526	38.02704	43.41197	37.71449	35.6411	40.19832	33.61115
TULP4	64.67741	540.1862	243.0406	434.4445	594.2843	56.01609	637.3241	135.7268	110.8641
PITX2	49.01306	35.54438	35.81563	54.66238	39.09745	33.38006	34.32264	32.28495	42.65483
SPINK7	3288.891	451.2516	1108.546	33.95423	4221.608	795.0056	344.1775	441.6383	1357.472
ABI2	272.3491	751.6356	391.1441	198.541	161.0506	661.8751	179.6243	298.6432	406.8373
SLC12A2	44.71167	450.074	193.4726	77.96875	380.1348	42.52781	130.0417	36.23691	34.94488
CA12	11159.56	11340.45	12068.4	15009.02	16941.22	18003.71	13788.4	11607.9	9146.872
DUT	91.70151	78.67152	50.32413	51.15231	54.88616	61.65546	51.6376	51.87306	109.2944
LCN1	119.4961	106.2837	69.61847	248.2754	51.82351	71.71969	62.0605	98.05242	274.2171
SRFBP1	37.47188	41.10455	322.652	594.0356	149.0402	685.8717	120.8126	153.0393	66.76677
SAMHD1	67.3259	54.4799	891.7014	56.37061	443.8228	365.0793	299.045	65.57912	35.57191
RHEBL1	248.5754	84.57685	132.8315	144.4712	104.2338	107.228	184.5006	160.1826	192.4118
ZNF227	418.8083	245.0865	206.8812	317.3121	76.39447	176.1444	122.9938	119.8354	122.1538
MS4A6A	69.97458	641.4519	1068.607	55.58509	996.3625	402.7449	1359.142	1800.375	32.12007
PTMS	9189.422	4840.199	8407.959	9265.007	11771.48	5978.321	8393.359	6413.424	7615.092
C17orf54	86.88812	93.47518	52.36754	73.70274	68.74254	57.48769	56.87571	49.7337	83.55512
SP4	45.52538	52.98383	49.50582	33.96382	48.7767	39.5093	60.93221	133.5092	62.43935
MS4A15	48.67173	69.13159	51.90981	41.16565	37.62277	45.71372	38.69461	46.54482	32.52147
TAF10	5863.017	5341.334	8763.573	7716.259	7594.75	7532.352	7602.257	7796.011	6637.504
C14orf159	1119.859	578.4963	381.0512	334.291	450.8997	448.012	609.9015	544.3611	796.1237
USP10	43.64378	33.26437	127.1571	399.5988	86.05728	44.41246	44.72578	116.529	67.12048
HIST1H3D	550.3949	230.2309	209.4553	287.9213	135.8777	278.06	178.0108	194.6652	506.3473
TOMM34	2858.858	3556.687	4533.158	4464.345	4828.152	4749.884	4206.057	4887.734	898.2472
NEK4	89.51369	727.846	424.0637	55.65523	50.45305	76.01409	172.765	45.00415	69.87984
DKKL1	321.8197	190.2906	102.4698	214.5074	99.56031	169.7471	84.69563	110.816	442.4096
TGM1	1747.725	3190.396	7830.925	2742.312	7457.827	3421.879	5682.676	6067.219	4945.104
H6PD	832.0577	1057.325	437.7549	1231.959	320.5867	340.7376	317.1747	491.6355	909.5439
GRPEL2	1021.047	539.811	1255.043	475.3717	1029.235	832.7781	725.1651	1068.846	790.7927
HTR5A	39.57988	51.66417	37.75979	43.19698	36.21324	37.13886	37.26646	35.32573	51.86545
MYT1	51.09454	53.10925	53.78283	46.6413	171.3983	39.55045	34.55877	59.74322	64.54205
COL11A2	65.25734	72.30276	52.31603	53.08502	52.90607	49.295	39.73324	80.29589	227.6456
KRT13	12329.94	61.72225	14414.09	3265.837	21833.22	6980.511	3292.739	2411.458	13127.31
C20orf4	1754.162	50.91512	82.4426	39.36074	522.9376	59.28032	534.0128	596.1202	61.34945
PSCD2	54.35144	49.84127	398.3111	37.11827	1201.668	1223.954	834.0975	621.5276	57.19994
KRT75	43.44703	45.04445	1500.461	997.5989	2209.939	505.986	3253.883	696.468	76.25704
PPP3R2	42.11742	52.45494	41.8272	52.34404	30.78373	59.37738	43.733	37.88208	84.12581
HSPB6	13819.29	22010.54	10871.58	12185.35	15640.95	18099.77	15863.39	23168.13	20623.82
TNFRSF10A	106.5924	57.55754	36.42268	56.49103	38.18242	37.19096	36.92438	46.96261	388.3853
CERK	112.0743	58.90202	62.22932	292.089	57.99938	104.3253	70.39015	54.00936	62.26511
MRPS18B	133.4295	74.11859	73.44932	108.2747	55.65741	67.16673	109.4837	177.485	200.0858
C22orf23	83.10172	43.68796	43.99323	39.46589	55.94141	44.65767	63.31374	103.4822	40.77122
ZNF276	272.08	183.2645	97.1357	140.7763	248.6523	136.9688	148.6586	114.1028	84.04702
MOBKL2A	10851.33	8358.579	5549.044	11031.9	2126.429	6881.759	4843.981	3132.982	12397.24
MAGT1	184.2962	226.1812	142.4926	191.3567	118.4913	164.0891	113.4053	102.1656	717.4599
KRT6B	28845.36	31077.95	36451.57	32015.38	36263.47	29426.29	33317.32	36377.18	25648.27

FBXO27	37.25139	38.15369	37.75094	34.65775	94.55408	39.23143	62.81979	55.57123	32.90513
ERAS	44.03922	42.34995	54.94432	85.95137	51.52899	38.02337	39.99656	45.02536	72.28381
ATP6V0B	52.71843	3587.8	7170.421	3304.546	9475.86	3782.255	12332.56	13676.87	160.651
PARP10	267.8456	992.5894	492.0918	139.7256	59.70897	516.3405	69.05397	271.6743	1825.628
HS6ST3	38.86426	48.28334	52.82524	57.65461	41.1805	57.85155	45.79268	43.91018	50.79487
CHX10	89.8769	94.38223	67.87482	87.77547	64.43449	69.0358	49.37436	60.93781	78.53284
C21orf123	48.37226	35.94353	37.16894	38.75797	37.08296	46.23345	38.85704	59.54404	47.93053
FERD3L	320.32	156.3499	83.83155	185.9437	93.41367	125.852	76.83951	103.8055	337.7148
ADAMTSL2	7576.128	5361.807	4280.967	6152.288	3158.733	5146.518	4909.477	4119.469	7193.89
B3GNT7	42.03722	858.9432	42.69729	44.20908	43.65474	38.50191	35.63978	35.15987	106.3654
NWD1	36.1337	37.73085	42.12792	36.63586	35.27851	212.4788	136.8957	35.13903	34.63692
WIF1	198.7237	134.0648	73.12632	135.2473	69.20554	112.5059	61.20846	83.7054	287.0545
PLA2G1B	39.71944	32.97862	32.88974	47.46944	35.93536	50.16562	37.92054	37.05114	48.92786
FLJ44313	59.83091	161.7925	58.19509	90.04231	49.87516	55.58101	52.26095	41.08551	195.2303
FABP6	99.28918	2196.678	333.8852	144.6371	293.6398	76.7326	50.60535	482.7604	149.8023
TMEM127	10757.8	9388.147	9588.053	9734.437	10005.93	11860.85	10095.74	9688.938	11092.48
C3orf24	41.57783	38.76518	43.90751	43.95152	46.28577	54.75465	40.69706	40.23436	72.45448
VPS18	60.89551	996.1635	1589.595	846.9741	790.8226	2373.018	772.2447	2441.552	4125.745
OR5P2	64.06187	52.36708	39.93812	95.67409	37.86008	40.48235	42.04238	47.65942	85.88941
TBX15	48.69278	49.59652	47.36366	38.50543	42.95704	63.11526	62.43546	51.14951	59.91256
C19orf63	48.98225	40.86697	33.24186	43.7955	33.06691	42.70293	33.80878	40.05261	49.49052
SMYD2	146.9226	135.6441	148.6071	75.32926	152.8727	193.3515	79.15912	107.7198	109.6175
NKTR	1742.473	1357.652	1627.499	1856.626	2141.643	1395.584	1598.012	745.7805	1187.629
APOO	818.728	1019.18	598.4362	1206.675	739.158	860.4839	446.3624	1021.085	328.3122
ARID5B	586.5784	410.2873	360.6651	383.4344	478.2718	520.328	308.0065	304.8727	824.2289
C19orf73	35.39774	42.77934	203.5685	38.48458	1068.934	330.6212	918.8654	469.0237	45.43075
ZNF654	61.34815	64.92812	36.36916	83.87497	52.9581	73.21042	89.76811	39.05155	45.72829
P2RY4	342.8262	160.2823	95.95148	168.8828	97.43285	149.2975	65.82622	96.89196	490.8686
TSC22D4	48.38921	42.64542	50.32204	55.99672	130.032	52.4006	119.7447	40.51728	70.09463
RSU1	851.1922	850.4117	781.5142	477.1039	857.9711	806.6526	561.8085	618.9615	88.00961
IDH3B	4573.742	5670.353	7213.697	6086.584	7363.504	6986.689	7101.085	8662.027	4561.772
GPR171	468.6193	72.40334	116.5524	42.58429	75.66898	174.7265	65.4715	72.92787	49.5004
STRA8	36.9148	47.34232	37.26838	49.94692	40.66858	31.83014	37.15226	34.74723	43.07771
PNN	5211.82	5822.451	4411.792	5747.891	3353.776	3839.647	3643.697	3248.169	7073.586
FNTA	42.10915	1683.996	1374.317	544.2144	1017.811	540.4554	810.8493	830.283	1498.518
EXOSC9	150.9607	162.1848	67.34647	169.6865	75.89651	111.1303	66.15324	78.03648	374.1595
USP6	44.62295	54.84201	50.15458	39.22704	40.67262	37.28745	46.07338	36.69786	45.32013
PPARBP	741.6868	395.2376	568.5209	745.4721	563.4454	384.2813	412.0098	554.2698	677.0488
GSDMC	61.8448	60.77506	1871.803	1666.189	2300.908	2390.833	3365.26	1529.843	52.24188
ID4	477.8861	449.5709	150.9106	405.8486	575.3613	195.1619	202.9441	120.3778	39.24398
IL18RAP	1572.619	2582.554	2764.532	600.8003	745.6256	2527.62	1651.679	908.9099	1956.225
FBLN2	35.7682	39.91076	52.7337	35.35064	157.7948	34.37914	34.19889	36.92999	37.12677
PAPPA	2267.931	45.22181	261.6165	1172.763	365.3923	416.6484	246.5837	309.3039	1189.32
MPP7	813.9335	89.71761	331.8792	88.56501	275.4502	74.27338	258.4625	202.6189	169.41
GDAP1	58.71531	81.79565	117.2341	66.63951	54.89852	165.5328	83.0827	143.5446	179.5333
BTN2A3	42.19017	52.39172	49.91912	41.3266	39.41585	45.65345	39.80955	60.48732	54.74742
IER5	42.5359	1211.356	403.6309	977.2015	434.854	722.8105	460.2221	347.074	1107.925

RYBP	36.85008	512.3868	1112.812	1829.024	1193.081	827.67	1392.114	1500.516	34.25492
ZNF660	51.62261	40.53479	43.79953	846.3495	44.76287	48.37651	264.8584	207.7725	52.70699
TIMP2	2255.914	1645.906	1366.773	1140.874	1398.372	2019.47	1811.719	889.443	2063.848
ITGA2B	71.94183	42.88127	64.19705	63.3713	57.71985	80.31191	47.11004	45.11296	199.0168
CTNNAL1	281.5522	299.979	383.9204	2413.036	739.7809	1188.346	492.768	331.8792	1279.063
LOC389151	40.58738	35.22847	38.21392	34.75316	34.46292	42.75631	37.38689	37.84452	37.56128
BLMH	695.6272	347.7911	264.4798	543.2656	358.1822	385.7119	351.5797	335.9029	786.4963
NAB2	55.99951	79.75121	107.165	97.7717	101.6429	131.7409	70.65414	81.70018	78.61993
UGT2A1	151.1318	62.69917	67.83677	84.67831	69.69759	97.55429	59.40505	58.60569	110.9249
NOVA1	92.82508	86.35326	56.1559	69.13866	194.1758	58.44707	129.7029	322.022	61.85101
ARD1A	3062.526	5019.313	7692.323	6967.763	8521.358	6793.588	9260.566	8961.025	6337.094
CARD11	2301.947	2079.09	1645.083	1315.996	491.4301	1386.442	776.4547	703.923	1741.616
HPS4	32.31115	32.32722	33.87867	750.1048	38.13848	39.11197	41.24501	37.87578	37.5854
LY6G5C	43.46069	63.35294	56.56551	49.09319	51.49195	49.46174	47.16492	38.38044	60.24433
MAGEE1	78.22456	139.146	82.11824	111.5006	74.13162	130.7881	74.03123	95.22904	233.3208
CPEB4	73.39304	2393.628	257.1483	833.7133	353.2166	422.6303	170.824	286.5338	248.8241
KIAA1586	37.80645	40.88353	159.0983	54.55514	34.53629	32.97257	88.7874	47.4453	75.4142
PEX5L	122.8844	36.02167	59.77011	38.54718	49.50253	38.90654	40.70227	43.63979	32.02958
SAFB	807.6055	2501.796	4086.163	2284.789	5669.115	4172.334	6268.771	3061.896	124.6504
ARMC1	999.3777	1460.775	1107.522	1294.838	651.4202	939.4969	883.4209	1276.665	1207.733
APOC3	62.95155	63.70196	45.79671	50.42601	48.17245	46.15944	50.64582	40.96539	49.16265
MLXIPL	172.7529	61.4614	71.53288	142.5439	244.4565	98.93365	153.6456	409.5171	96.49907
RNF217	39.27349	37.86791	146.4841	56.26536	112.9351	34.25737	172.9118	267.386	39.99255
TP53TG5	223.2865	88.72866	71.67805	67.17557	59.8699	60.51205	49.18732	63.32915	51.84681
ANAPC11	2638.041	594.4442	3019.818	725.2795	4581.198	2591.225	4212.958	3160.273	2282.916
CNGA3	58.33788	42.26889	90.49961	42.64972	49.61854	63.62342	40.18143	44.93411	52.55166
EIF6	213.9641	104.1581	76.29836	76.88796	69.3632	144.7345	65.5187	81.66558	65.15346
RWDD2A	36.56209	1159.841	1840.07	1621.43	1549.013	2540.285	2351.298	1115.22	1558.06
CASP5	47.88364	614.357	336.451	36.21202	33.82584	379.209	247.7883	349.9278	1554.428
C2orf56	2189.308	1998.049	4247.579	2567.95	3104.272	4235.579	3766.441	3470.636	58.00995
PRPF40B	35.69326	40.69997	34.18775	43.8562	34.441	46.6693	39.13409	134.0617	58.93035
TCTE3	107.025	705.7682	41.38617	52.37724	60.24665	49.88074	49.82743	162.856	89.58495
MBOAT7	239.8296	123.8491	176.0791	132.0329	50.57786	100.1492	132.1811	77.86587	90.84282
MRGPRX4	73.71511	49.69392	190.8372	58.42742	53.60814	35.43996	45.60007	51.16588	52.0514
TIMM8A	2922.127	1756.371	2439.81	3417.93	2400.224	1932.289	2168.863	2716.542	1996.298
PAFAH1B1	116.7417	159.2918	112.5107	163.0281	304.0507	72.66673	350.2572	178.2043	96.11649
IL17REL	102.457	89.56603	59.79356	86.39974	54.12754	65.95672	58.45895	80.01242	111.0897
ABCA5	33.98331	48.95479	37.46885	34.60827	258.7593	602.3168	114.0149	84.56235	32.06269
LPPR3	83.44539	76.75815	40.50615	104.2766	47.89732	52.24313	45.20726	54.78882	81.78458
AVL9	51.73662	43.87343	183.5776	38.14998	322.7204	35.54162	326.6491	332.8067	52.85292
CPNE8	57.13284	50.49987	37.54105	47.61393	311.5629	36.81461	161.6038	39.42472	26.26047
ZNF48	63.48258	62.69796	47.21319	50.5727	47.18164	60.108	66.64254	149.4003	52.80415
ENDOG	69.19572	115.4768	46.6828	70.267	56.24716	48.68359	113.8578	54.32119	73.24897
CASQ2	114.5349	88.93563	63.89296	126.4132	110.4201	705.1762	473.6821	514.3997	76.38219
GK2	126.641	42.85491	54.07178	47.87194	54.58599	41.39711	41.76874	46.5557	52.78677
C6orf108	102.7046	101.4957	69.83576	138.6323	72.86431	73.31622	67.79826	75.12836	178.4776
IMPG1	38.8744	37.12308	42.857	39.50384	35.60632	43.92019	43.07654	34.55162	35.76887

C9orf126	39.11127	53.83912	198.1265	46.29799	189.7017	207.5385	172.132	143.0775	84.21088
CHRAC1	129.0415	122.5077	65.87003	183.7465	57.74044	60.15535	140.1577	88.65399	58.73004
HRH2	48.12444	42.46381	34.97962	35.50494	54.94832	68.18059	66.40464	36.4523	34.08468
SHPRH	37.62858	948.2162	331.9899	38.2871	700.5546	486.4767	120.5829	219.9599	46.75901
OR7D2	34.58204	45.56704	34.97946	47.40215	35.87053	1020.591	41.56703	44.04327	60.7365
MGAT4B	42.44193	40.47076	39.97636	33.40225	31.97971	51.03269	167.8943	456.3872	40.94578
MC4R	43.86285	35.10668	37.45629	43.67348	50.17188	44.58111	37.95721	41.038	65.10405
CCDC60	78.94782	74.58928	65.37054	46.30982	56.55354	59.47464	51.13634	39.83743	203.4898
NSUN5B	117.3092	420.487	133.4678	107.4776	183.2204	284.2324	170.6011	146.9073	285.5681
TFF1	81.41654	49.58597	65.44542	41.28872	54.69321	55.51163	44.42989	38.30007	44.29851
APRT	1564.551	1411.946	1327.43	1492.89	1015.643	361.1328	767.3857	622.5596	4970.856
DUSP19	450.962	335.1616	169.2548	260.617	117.6525	128.2046	104.7584	150.1558	593.5863
OR4L1	35.25211	52.21338	48.96751	65.19883	34.31945	57.70861	38.30618	45.05159	70.02448
DEFB105A	68.08074	43.07196	46.11291	57.18083	45.01574	52.1824	38.86599	60.95285	50.48948
OR8U8	44.20149	37.76476	34.98975	43.31646	32.58169	34.26687	39.24819	37.45507	41.06144
CCNT2	84.10024	1222.294	1155.428	720.1749	1533.163	1270.713	1108.764	806.6809	139.988
GALNT14	81.77175	103.9667	111.5664	111.1086	61.39976	69.82273	125.4209	127.1511	107.4514
LECT1	58.50674	38.79342	34.59814	40.79293	58.73602	59.62259	50.47929	41.15396	42.63805
JMJD1A	61.28077	247.8855	202.0509	294.6435	380.2703	44.15799	294.1554	196.2302	34.29316
DEFB123	203.9936	60.87004	94.55005	143.011	54.94249	106.788	49.3686	55.41118	85.32754
DCD	234.3137	137.1092	90.20295	111.2599	78.2551	80.66444	57.07913	75.10036	164.3722
NEUROD6	116.9818	75.44158	62.5066	128.3186	74.16426	97.40321	62.90692	84.1191	88.46703
COX4NB	91.18471	58.46811	172.809	56.02305	34.81785	69.46262	219.6954	42.94105	222.5113
CDX4	137.0853	56.51248	40.9365	47.42044	40.52821	60.93248	46.54708	44.39655	56.04411
CD163	1528.367	78.5257	394.7171	150.1046	61.44324	64.2361	359.8818	63.15847	154.6641
FLJ41170	58.30285	42.68287	41.12555	51.94371	160.8103	49.63796	38.67693	46.86007	113.7644
NUCB1	25116.32	25231.16	25644.13	24850.66	25022.88	24609.6	22999.66	24766.37	25706.98
REEP3	51.53986	50.09018	47.36377	40.38745	37.29172	43.34014	37.8618	34.50724	46.17331
SF3B4	5849.296	6113.541	7751.12	8009.241	8725.317	8290.283	8490.156	6685.237	5341.139
RAD23B	53.17648	368.3736	156.671	53.43235	177.0839	198.5291	182.4114	169.7543	115.384
PCDHB4	37.51814	52.75783	40.02163	58.94325	129.504	39.22635	33.46888	44.99554	343.3679
MGC3196	222.459	162.9656	78.88111	166.7336	79.54284	128.1406	69.77309	94.04417	414.0088
ALG2	1430.096	1959.273	1293.82	1223.176	842.6653	2215.183	1277.133	878.0209	1206.953
RCHY1	43.41269	46.46418	59.18783	100.5045	45.39163	42.81324	82.03958	209.7519	68.58572
SLC17A1	123.6681	152.3783	108.9286	252.887	75.57935	159.1878	103.636	239.8816	53.53312
BMP2	187.4046	336.9639	136.1658	371.6888	518.6005	242.8686	258.8493	303.2494	94.93423
RICS	85.17905	52.69263	49.30088	67.37291	262.9532	51.92507	84.96516	46.91462	52.2473
ROGDI	82.1924	52.35931	171.0813	55.61028	376.0859	108.4659	173.5174	305.7702	813.5489
SCLY	230.4563	86.78603	362.9315	83.09138	932.2241	198.6374	824.7063	706.4111	120.5122
KRTAP6-2	51.19908	68.16876	50.33653	58.82277	31.77408	47.65345	34.33032	40.29445	40.67682
LDHD	63.73347	54.60906	52.7268	94.2236	45.53564	45.31985	41.71166	54.05438	94.35445
VPS72	138.7501	95.58357	77.08765	164.6963	53.96937	542.2772	405.6084	1399.943	163.3469
EGR3	55.84076	375.3843	191.1812	62.8982	121.1283	41.20707	91.09702	153.4289	60.99955
C1orf49	42.4905	50.22248	66.91467	41.83285	38.54926	48.99318	41.08485	46.31693	51.37734
PLCD3	1324.598	2640.74	907.9944	2353.106	811.8455	1044.936	610.9172	1337.571	3459.391
NRCAM	154.3341	80.71758	113.843	163.455	129.5864	67.35323	112.6989	62.47144	111.704
CDKN1B	790.7275	294.6754	757.1188	674.8958	977.5714	284.8372	442.4475	268.3503	873.3748

FLJ36701	37.93974	45.65504	49.16697	52.10183	45.5708	43.33657	93.6824	58.56067	42.47237
GIPC3	38.90572	34.86217	37.37376	48.18935	36.52259	41.73135	34.15773	43.78651	70.27921
HLF	1480.104	120.9563	843.9801	113.235	825.454	702.8839	327.8745	527.9427	204.659
RGS12	26687.92	24559.39	25812.63	22215.24	24410.31	26900.87	28456.32	27941.84	24871.5
MRPL46	72.41041	60.98629	61.77219	72.52148	669.479	82.05348	508.6239	928.4196	163.3086
RAB3B	61.75354	88.62895	62.8213	72.38388	67.26552	89.11765	54.38152	50.39627	193.1935
UNC13A	50.7864	58.52834	53.6816	75.51251	56.20335	52.30848	54.84037	40.56506	55.2319
VGLL2	163.7374	91.86702	58.08666	110.6655	64.26839	80.212	56.48561	71.13273	99.44721
VEGFB	1675.263	1900.453	1653.405	1563.259	1606.54	921.5412	1894.72	2202.429	2189.508
PPP2CA	18454.75	17176.71	14334.13	15551	14355.97	15582.02	13292.98	16847.26	17203.77
CCDC36	116.8468	69.3469	58.49385	50.75283	60.40148	82.92976	46.95045	58.65805	193.3212
PDZD3	48.53591	43.98476	46.27851	55.5843	59.0291	38.72115	37.9558	51.53121	58.55872
MYH9	12244.16	11257.54	14516.63	15418.26	17029.32	15358.79	16164.94	13961.45	10259.7
C12orf40	41.43909	43.69225	38.67249	55.43206	40.61066	42.99223	37.11307	45.59734	68.16566
SFRP2	38.09026	42.48672	40.94786	44.7186	68.61822	235.0582	60.27723	39.02744	39.83896
EIF4A1	10124.23	8094.874	6119.846	9040.777	6851.698	4833.962	5265.183	4778.86	8257.45
MRPS11	71.20205	1645.774	4917.776	2317.477	3654.009	1536.186	5620.973	6119.355	116.9749
SRGAP3	36.05765	42.00044	36.6727	37.36959	37.94828	193.4325	37.94976	33.87864	42.09932
USP47	3566.684	2393.322	3483.768	2274.699	3483.267	4397.748	3170.553	4309.973	45.43306
TLR5	48.75611	40.78112	764.4709	385.8407	590.4614	366.4734	418.7973	493.3296	63.42385
CD72	242.2613	195.039	188.6505	172.1365	76.62148	136.3592	73.57944	83.2244	369.3909
ZCCHC9	1003.291	538.4004	898.5588	1149.539	784.8425	220.5457	754.8131	831.0708	2483.85
ICMT	131.6834	75.61285	446.6399	103.1232	2079.352	835.402	1682.366	1836.952	125.1627
OLR1	59.08046	87.92735	55.7583	93.06111	64.83366	219.2035	194.8051	58.64036	107.6494
CLDN3	53.68992	95.07459	41.11771	59.83575	102.0477	310.1808	73.73379	61.77484	36.04163
GJC2	305.7113	697.4173	846.647	1322.907	760.6865	941.074	767.5589	592.5239	2082.914
CRB3	70.81812	503.6726	2298.686	45.48809	2846.716	1383.407	2507.743	2220.661	98.03741
HIST1H4A	1114.398	547.9329	75.74069	37.19066	43.46746	316.4705	32.29485	135.0594	52.22206
WFDC13	362.2329	45.83884	48.3699	125.767	41.40385	36.84933	43.1725	51.39188	36.75003
FAM10A6	44.70991	70.42484	39.35276	33.17494	39.4357	36.66578	36.61979	39.17676	149.0542
CLEC4F	31.73422	33.69456	970.0146	34.21023	373.5128	36.34993	420.2339	337.0834	42.12225
IPO9	150.3056	642.2726	285.2505	180.6908	411.6759	263.1552	266.3531	178.0714	155.4242
LOC650695	62.07131	642.0299	46.97386	33.55584	42.42559	46.62843	43.39903	151.7767	59.80473
GPD2	424.6969	74.89854	108.4553	284.0971	67.68825	37.39226	76.59115	65.89909	762.4902
TMEM121	36.97004	35.54223	34.26108	33.88218	35.60763	35.85011	35.19997	40.6953	35.5309
ANAPC5	629.4882	524.0587	777.1503	187.2648	1008.884	726.5469	1174.921	777.0959	480.7632
ELOF1	275.7759	236.64	116.3989	280.047	121.3839	121.0521	80.65467	106.8612	347.8877
L2HGDH	93.89398	137.8813	1785.554	626.226	1241.205	707.0268	1254.846	1954.368	85.21088
SFRS3	36.69543	611.3837	108.1585	40.49986	101.3368	142.7462	108.1696	33.70648	659.2677
FGF3	47.47669	43.94368	43.88283	54.5713	47.93292	47.41666	45.47972	36.96551	86.77159
PDE5A	35.51794	52.40885	152.0465	34.06839	241.726	34.50933	34.06681	37.74327	32.20451
DUS3L	99.58121	78.66504	31.05625	50.79453	114.0598	70.69617	343.0051	58.60564	58.41127
ZNF222	454.8609	43.38131	70.57916	50.56436	96.31173	57.55938	78.02662	66.2122	37.10209
C3orf42	61.87026	48.5862	61.45691	59.31004	89.72173	47.25854	41.83443	50.44676	52.36171
C1orf137	118.2961	152.4675	76.51451	139.5309	68.4285	70.33127	55.97305	98.12653	308.245
ETV5	516.8806	247.3739	83.57878	106.9906	96.27658	151.3492	75.5566	103.1701	394.0082
GPR55	1458.736	1471.164	802.8242	232.8727	384.4731	781.7955	360.9461	514.423	1162.189

SPINT4	49.7827	40.45258	34.82573	47.56693	44.29992	41.97015	33.99939	46.14442	63.07467
SLC6A16	1540.002	2231.766	1429.41	1381.646	801.1809	1570	891.1334	1043.293	2066.445
OR5T2	70.7013	41.58381	38.04274	60.06581	35.39369	63.09652	42.86586	43.17246	44.02808
HCG4	165.6452	138.168	94.47379	154.9858	81.77435	113.1751	78.32117	80.84715	422.7001
SCGB2A2	44.36555	57.52754	52.90052	43.738	44.37169	64.64368	38.73223	52.41542	55.14803
FAM122A	231.1324	122.2158	60.98619	187.6268	64.29343	107.2452	102.8717	56.41195	50.17661
TSSK4	33.40685	42.0491	43.53619	53.34755	361.4209	47.84042	39.76403	36.05088	64.55028
KRT26	48.42252	58.17089	42.11203	45.79951	38.89451	32.77935	38.71889	49.50156	47.89094
ASCL2	8011.144	8511.376	5970.42	7918.799	2340.356	7536.12	2318.491	5198.531	7863.495
HACL1	4315.591	3070.866	3322.516	2557.011	3341.509	3912.154	3661.202	2814.448	1863.872
CEL	1421.569	2324.936	2471.689	755.1713	2040.026	4011.633	1928.11	2272.649	72.67221
PFKFB2	65.54373	59.83777	44.05993	63.26361	223.1379	47.00097	35.32539	45.25013	65.79446
ZYG11A	56.12223	32.41975	46.02652	35.97399	37.04723	36.03479	38.99458	37.94381	37.2189
LRP2	46.94455	44.10701	47.30563	35.24842	39.33686	40.08002	44.77287	39.41839	60.56548
BST2	494.945	500.1219	496.1896	609.9232	120.3449	467.335	318.6833	316.8894	592.5411
CHST3	399.4612	1165.058	1462.771	225.8302	1918.922	1094.148	2492.392	3263.742	545.3824
HK2	125.194	325.3623	81.01679	131.546	68.30439	120.1149	107.0364	136.1072	241.5084
SIRT2	159.9278	66.35819	74.01357	99.80228	119.3642	115.0698	133.6175	57.03185	100.6569
CD1A	1824.468	108.9798	2750.847	173.401	1707.796	4877.045	1684.533	2553.879	152.5967
AJAP1	45.85871	47.09941	35.64294	439.8225	39.10752	39.04268	143.3757	251.7012	36.7887
APOLD1	49.38259	54.7206	234.7609	35.97356	277.7022	46.74236	244.403	225.6632	36.40523
USP33	356.07	38.54718	301.1169	37.21066	214.4949	245.1888	378.3801	243.4866	94.05891
ZNF137	68.63126	53.5324	260.2013	69.20661	192.1916	42.81772	216.6324	42.10856	79.16193
C1orf118	46.08242	66.30779	40.19431	54.19769	110.9622	55.32144	41.25284	49.28834	65.28873
C1orf227	42.75858	41.10939	52.96648	60.52631	34.99327	43.49722	73.88152	44.74235	281.5266
MAP4K2	15047.37	15576.66	14452.8	18023.68	11383.72	13814.93	18609.45	11933.55	14837.64
APBA2	36.07298	56.74383	61.77265	45.81651	36.14149	62.33409	101.9091	38.22728	37.61271
PIGC	517.7604	329.4147	146.4729	311.7266	219.5972	234.5957	101.7109	184.3259	751.9116
PKLR	199.6511	88.41049	56.36422	57.12876	46.16437	36.90712	49.72985	47.27363	61.23738
ABHD2	481.2308	261.8795	328.6424	278.4839	233.6883	404.0687	408.8051	545.5831	565.6036
NKAIN3	174.9126	132.3333	73.58834	74.2436	76.23381	88.5878	57.65404	68.67159	100.7411
PRPH	3341.914	3759.334	863.4068	852.5856	4076.725	1974.188	1546.066	3883.924	63.95899
DARC	199.1416	107.8197	173.1284	74.18502	469.2856	81.4739	296.6035	232.7847	137.9614
PCDHB10	41.74658	38.09091	39.22878	43.18425	34.9916	32.64041	39.10673	57.0718	65.62777
GUCY2F	178.0072	112.8542	110.9496	175.64	78.49776	102.7999	70.04733	70.91573	289.5579
GRAMD4	1106.073	387.7152	513.0419	39.58016	1956.225	1208.094	1334.432	661.3564	46.7975
C9orf116	35.72089	49.0451	140.1699	406.5009	227.0195	41.58776	191.9642	137.0274	54.82496
RSL1D1	941.7706	43.94876	373.8713	69.84514	61.90586	654.8161	171.9515	250.1794	1339.105
ROM1	514.8815	381.8585	301.5168	327.5021	477.0385	256.4621	280.6911	485.3335	664.4208
PALB2	195.8709	111.115	277.0786	111.5109	309.564	69.02496	360.7637	57.98581	295.7429
FLJ32011	100.4859	139.0344	43.66286	86.14266	45.10024	57.90516	52.94976	39.82773	124.8448
SLC6A17	126.2332	130.122	76.99483	146.421	83.53255	97.04749	62.38879	63.94751	335.3176
CSNK2A2	33.24656	42.52808	366.4373	653.7251	864.2997	47.41323	809.1186	638.0145	2879.553
CILP	4994.148	3894.501	471.257	1685.109	3075.554	1813.519	673.2278	711.9639	3370.66
OTUB1	309.2483	157.299	168.4678	220.56	775.1382	373.456	703.1268	271.0331	432.5224
MRPS6	15787.51	16698.13	16570.69	20707.81	17161.79	17384.35	16059.76	15843.09	17938.45
ZFP2	50.99336	658.9479	551.4081	51.80247	860.159	1029.984	248.2478	541.6254	64.95548

GTF3C5	71.23079	40.97769	42.86951	36.36263	429.2856	34.33708	446.5208	808.884	83.644
LOC402176	36.82983	490.8946	200.7045	35.92892	41.76324	38.58216	82.88334	49.52194	32.70565
WIT-1	44.08614	65.43288	40.15236	40.84525	38.47153	39.62423	41.06384	35.90625	98.10753
IFI44L	47.19885	2580.896	4016.914	1025.599	172.4768	4400.179	3876.239	1935.566	40.47659
C10orf30	54.89001	54.44515	35.43004	53.27515	48.51435	42.63043	48.0977	72.13204	96.2117
TARP	107.9485	189.3846	64.67723	107.194	56.65903	76.78398	58.17458	45.93035	114.3056
LXN	829.7207	734.6131	481.6775	105.4002	572.4292	497.2943	395.0876	256.6527	125.2452
SLC29A1	2222.458	1406.71	940.3287	2942.942	732.4188	1401.664	965.087	830.736	1184.417
LGALS1	2085.93	2556.627	1636.569	4706.482	1636.314	1987.262	2046.227	1674.742	55.04875
SETD6	7590.379	6450.517	5727.396	7176.861	4283.322	4480.233	4219.446	3785.347	7930.932
SLC6A19	70.48344	46.71591	47.78168	74.24238	60.56903	73.41107	46.10038	60.38739	171.6126
ANKRD57	16487.36	15785.53	23695.22	19610.16	25917.02	21026.95	24330.91	19981.35	15697.1
ZNF519	34.92136	39.09578	81.51604	33.27247	35.16777	87.19672	57.09432	35.48589	44.25725
GAS2L1	34.29182	35.46119	808.7361	897.8406	1699.326	42.04113	974.2897	38.4653	42.67422
LIN54	603.2238	578.0408	659.1198	609.2242	567.2294	585.665	412.0233	530.0716	244.368
PLCB2	48.13729	35.34881	883.691	624.1589	514.7224	329.2027	685.8796	517.9318	38.06873
MAP3K14	81.10172	76.7645	163.3324	42.70099	125.3276	290.5192	120.9353	34.84007	49.84753
TNK1	57.56533	54.6956	48.45976	38.00484	52.08783	37.52466	116.6461	40.68794	38.94262
KAT5	162.1072	68.60861	65.79476	100.118	60.62577	54.99115	60.138	59.13552	65.46434
TMEM123	418.6963	681.6356	1415.29	6019.49	499.5278	901.3501	2073.815	558.9698	525.9564
CBX4	919.6988	403.1163	296.7796	510.5366	602.9095	484.9798	425.393	220.4641	71.55431
ORAI1	516.4923	594.7695	697.2748	590.2559	177.2377	604.4727	344.7784	312.4369	557.9603
OCLN	468.662	296.0761	125.4865	271.9401	75.20246	190.3074	105.4939	200.7514	609.6498
ZNF93	1356.529	40.37087	126.6598	51.54465	36.79026	48.73862	38.15712	52.11295	33.50885
ARX	195.551	149.4145	61.22682	124.7167	69.67165	100.054	69.71684	338.1385	230.2228
OR56B1	298.4014	135.2065	89.67684	189.1872	69.05931	123.0477	74.45234	74.81452	254.3126
IL15	72.67548	756.8885	519.4825	91.00632	604.6378	944.0659	586.1403	627.2008	57.51324
CDKL1	118.9394	65.38116	69.35137	140.282	63.99009	97.16865	57.17161	65.91251	155.3344
NPEPL1	4528.083	7494.079	7425.976	6073.415	7290.396	7857.136	6781.062	5905.269	7665.858
IVD	106.7626	54.09826	44.08028	90.83996	217.2694	45.85431	231.3536	83.3378	94.72434
GPRC5C	279.553	123.186	64.10069	150.8931	64.7685	75.92341	52.02339	51.93692	194.4381
PAGE2	65.98434	38.09556	47.71252	35.8674	43.99958	49.13571	38.14648	38.93721	38.35749
FUT7	126.2249	96.12386	280.168	46.18781	222.6539	302.2977	248.2358	290.5422	221.7196
GRN	86.14253	637.4266	546.5388	80.54114	189.3089	52.43575	273.616	387.7399	54.9621
INSL6	48.89567	41.92155	36.24495	54.31821	45.5717	43.51551	35.54002	40.61428	50.01652
PART1	55.06018	40.01347	50.51962	33.05574	176.8911	35.77825	33.18696	52.95354	46.84598
LYPD2	249.0475	60.5065	216.861	330.5146	672.6138	328.886	134.9128	359.1754	1361.121
LOC401296	250.8323	147.8135	77.15968	167.9524	75.47029	110.283	75.23841	86.1227	490.6421
KDELR2	609.3792	392.4328	866.8615	594.2015	1306.779	199.846	1317.372	758.6284	57.31144
MASP1	132.7847	118.6054	148.5723	67.90776	113.5462	59.06496	54.3599	49.07913	179.8072
NLE1	86.49607	64.28503	66.51945	143.9881	66.47656	84.66032	89.03562	78.1391	143.6092
CACNG1	1652.37	6589.763	4425.556	3256.857	2461.988	3756.432	7198.187	11918.25	3287.818
LMTK2	710.59	62.23206	341.4938	319.3236	302.9974	181.186	259.1647	255.3786	107.679
TOR1B	61.45148	35.26008	54.38887	37.47654	202.7767	51.43102	96.32076	365.3654	63.56974
CXorf45	54.63227	42.95887	32.46518	47.74355	122.0496	42.45676	39.87104	34.51177	37.65054
IPMK	352.1962	145.5744	83.09167	194.4344	95.03839	143.603	80.77985	104.9907	457.4654
RGS3	192.0355	154.535	70.85257	145.548	55.05749	72.78178	61.0172	77.21633	327.3896

SV2C	36.49448	51.80563	44.31123	40.28644	40.66624	49.48315	36.07717	44.60483	60.44475
MFRP	58.57319	56.0258	38.8241	46.06396	30.24394	33.73116	34.33648	38.35389	90.17343
NDUFB1	619.2754	801.5195	237.6772	325.1698	166.7468	270.236	171.7828	177.5422	1205.051
SIRT3	128.6524	117.8736	86.64492	84.97642	70.17909	124.4489	73.29326	83.28072	193.1557
MUC17	47.79845	45.02505	40.16759	54.22711	44.76226	51.56588	47.71825	49.57005	86.80596
C2orf30	165.7376	531.6079	327.7897	89.34667	1243.605	444.2536	874.1858	621.8015	169.8768
PCDHGA7	38.99669	34.18289	32.76925	35.00246	34.2785	35.62265	34.23209	33.98404	58.22931
LOC642293	56.13475	33.35152	34.68906	42.34927	35.94178	32.69707	31.73568	40.88818	46.23371
GCG	44.04284	37.1479	38.50393	100.8298	45.20536	33.512	37.37281	35.51196	88.43574
INO80E	248.7024	133.539	200.0139	140.2024	75.51309	131.6595	149.068	225.2213	249.3679
TACC3	63.68887	66.51311	3138.432	1086.18	2410.951	1947.278	3413.542	2870.71	65.87329
USP46	59.6031	64.01028	64.28195	66.26551	54.64576	65.46677	92.5205	59.94041	106.6447
SELL	41.86977	1591.174	1107.075	50.33707	235.5434	46.09806	341.4387	40.28303	684.4566
PRRT2	554.8201	305.6079	136.2248	310.5381	187.982	223.6088	95.73993	174.8427	647.5875
OR6N2	65.33794	58.08321	36.93881	64.03182	42.48586	54.34189	47.50001	37.21452	77.06857
GCAT	46.1182	45.00103	38.61213	593.1902	607.9311	35.15072	440.6525	47.87539	41.89985
CARD16	207.0591	123.9072	146.5796	173.2952	114.7462	148.2572	102.4058	129.8949	53.75439
NR2C2	72.18913	100.2694	142.3867	54.06694	267.7224	56.77427	202.9396	168.6994	41.48521
AXIN2	36.54943	41.97435	246.7624	33.18946	1151.732	1152.017	692.1011	151.0121	49.31706
RFC4	61.30847	107.9477	1689.494	1603.981	1659.42	1894.628	2208.339	3520.285	64.72682
PSPC1	1272.013	2296.281	1193.186	1425.763	752.5251	789.2183	480.8996	1099.449	1954.645
LYSMD2	48.1483	34.25161	222.7234	33.06726	207.8587	51.61524	263.9655	150.2863	33.70111
SESTD1	855.9685	764.4683	497.26	897.4395	306.0014	827.6863	340.0071	284.6263	219.7802
DBX2	57.87479	35.48004	39.43089	38.27542	44.21169	39.8528	35.03288	51.81327	44.21567
C10orf93	46.6284	38.62775	45.20642	31.28272	40.2897	34.88707	37.01274	34.70187	38.17514
LY96	16207.06	15860.72	16958.46	13833.27	13483.3	18892.6	16484.06	15185.59	14583.61
CART1	54.47688	35.85919	35.01476	48.04017	33.67196	36.31995	35.68892	41.83404	35.24645
PPP4R1	3289.473	3012.978	4415.105	4780.741	4424.914	4643.089	4780.319	3688.483	3094.984
EDC3	38.08768	273.0377	118.9624	335.2802	91.02528	202.3342	154.0032	128.5657	676.0778
SLC2A8	4595.206	5212.157	7186.651	4207.416	4201.151	5447.637	6145.432	9281.585	2922.655
SLC25A35	41.81896	52.08863	218.1352	89.21577	34.19193	384.191	450.4151	245.5138	45.35664
BNIP3	700.9705	1608.25	2288.399	2996.114	2312.82	2445.304	1902.987	1341.681	161.7771
CIZ1	104.2928	73.46709	140.7516	56.06111	299.703	100.6956	101.7838	243.1313	81.14092
OR5B21	45.55604	56.79539	287.6223	59.53569	45.90862	49.78719	38.61069	57.51617	133.4015
CLDN1	35.36434	41.41656	615.0433	1608.056	870.7108	33.60435	1445.804	599.2795	67.13248
ACAD8	1044.771	1776.476	368.1978	939.4716	319.7543	505.5598	580.1122	633.1817	2207.693
RIOK3	94.79215	68.08908	67.68271	113.6321	280.5257	66.02948	125.4091	61.36538	60.97235
RAB33A	37.53098	54.70613	146.1054	43.08781	32.91219	45.95948	38.48093	36.19144	69.5733
MAPK11	33.11087	497.5127	155.7926	307.8534	316.7392	32.2431	240.3818	161.0203	54.68323
NCK2	7725.944	6207.292	6206.601	7248.795	6387.067	7093.201	5378.032	6957.344	7667.605
UQCC	46.25604	38.08379	37.4958	124.5712	51.69315	48.7055	96.06068	212.7943	75.02518
TFR2	1420.035	76.21257	208.5634	50.00647	378.4417	156.546	53.56194	49.30566	97.27795
NIN	83.76605	2473.423	2384.492	2420.708	1565.174	1729.543	1667.692	1404.314	1950.429
MPP3	84.19986	790.051	90.57766	91.77982	73.74915	177.6667	125.9309	382.9849	144.2294
SYT3	622.394	187.0747	97.44208	186.2649	102.4779	168.2903	85.02207	120.6322	424.6936
CD59	117.8246	37.79193	745.556	945.8622	984.7197	728.9169	817.1943	373.8303	165.7577
PLA2G2F	110.5045	677.8259	80.3353	56.54273	172.0313	52.23247	54.40512	51.58993	197.8488

MUPCDH	58.64023	44.79372	46.63462	65.13934	38.56588	50.94214	48.47996	57.52842	57.29854
RAPSN	351.5168	196.7333	102.5716	203.5361	91.63665	124.1673	234.5189	273.791	540.5118
PARN	2176.871	1875.963	1593.816	495.6257	1598.937	2371.191	2075.182	1113.666	1179.522
LOC39974	790.3817	85.64413	67.09406	47.95592	49.40193	52.28409	51.67942	48.3701	183.4409
PCBP2	545.1111	486.5394	1815.342	562.9359	1991.639	1188.528	2291.256	1516.795	550.0229
OVOL2	44.21596	935.8879	898.0228	1056.581	2413.209	941.3976	1717.957	1435.686	48.26627
TICAM1	1943.162	1580.523	1920.082	1866.059	2561.065	2187.284	2049.81	1776.281	1259.504
OR10T2	47.08789	54.21451	49.19989	89.24765	44.47942	54.7438	52.46946	49.74181	50.17918
SNORD22	784.3481	453.8609	154.5734	378.9524	101.0529	204.1369	127.9795	161.3532	907.8392
KCTD4	31.39523	33.09573	35.91784	49.39063	33.72899	34.32504	40.49732	39.46873	45.26352
C14orf93	5215.141	4723.298	4201.959	5318.121	2718.48	4888.361	3846.875	4939.204	5522.969
SLC22A18	91.56246	67.0611	66.96077	98.57042	51.34689	61.87026	61.17315	83.93439	88.3268
NUDT3	5321.461	4863.863	4478.083	5761.922	3581.013	5718.5	5105.633	5595.591	1616.352
C6orf191	112.3514	114.9794	59.80577	132.9619	60.43188	101.5786	63.93639	64.72131	189.1966
SPINT1	98.34678	73.83414	111.9612	45.56473	154.2011	82.89968	99.14046	197.2915	67.79018
LOC73237	56.10635	98.10503	58.83262	40.92487	62.37091	70.7335	47.79058	67.38628	85.3101
EIF2AK3	342.2535	192.2392	150.2927	230.0364	78.56317	135.0137	79.56628	93.58128	226.9926
WDR53	454.0971	428.937	138.0706	278.6309	180.3934	250.5981	166.1316	305.8163	593.2841
COL4A2	2015.232	906.129	572.5489	1302.529	314.3557	334.6198	411.054	601.57	2869.742
TMPRSS6	52.66772	57.27515	44.10172	52.31616	39.74067	40.99413	36.50064	36.19478	44.78523
LHFPL1	183.7214	89.56247	84.00237	119.362	75.18928	72.1936	57.54226	97.05898	57.35702
ADAL	46.66821	96.29919	85.8344	51.34281	69.16001	79.90147	70.47792	72.07732	66.03975
CCDC12	66.39642	42.99968	58.50394	73.11687	41.85461	38.55149	38.59138	35.38408	41.11927
DPY19L3	36.09725	40.60294	34.75264	180.9949	39.44973	71.12311	94.94405	59.02049	56.70658
TSPAN31	4130.062	4175.905	5170.623	3584.728	5135.441	4653.801	4269.309	5097.901	3362.693
CTHRC1	864.3816	334.4093	1439.077	700.0544	1128.538	2899.276	2814.122	1741.108	867.2431
RTN4RL2	48.07932	61.15502	41.87706	73.56336	38.30132	47.19121	53.4844	41.73052	74.82532
EEF1E1	37.7014	55.54662	360.6266	55.23052	720.5842	44.6366	616.9792	587.2161	69.44295
SNX25	580.912	791.8433	500.5499	1054.918	151.8621	249.9315	190.0948	216.9377	930.407
XPO1	41.36476	34.80423	380.0004	356.3921	551.3293	300.5724	507.779	234.6452	33.78039
FAM50A	28770.46	26646.65	25378.56	25582.88	22716.95	23985.96	23335.37	26498.96	29141.51
CA9	96.75337	78.77644	64.9483	2138.412	66.83578	1138.605	169.4002	48.95847	65.15914
POLA	263.3231	129.2814	89.03606	144.8791	74.61533	70.50826	63.91076	84.99748	262.4329
POLN	82.43802	74.12546	591.3978	49.61185	206.3801	455.9802	450.4772	300.6989	74.31409
INTS12	192.817	633.3111	1164.181	660.6138	1398.629	860.3573	1339.828	2407.681	105.6388
FOSL2	3338.437	2538.672	2658.204	3785.071	1753.399	2038.513	1989.452	1950.058	4117.993
TBCE	49.35789	398.2643	433.9814	92.46189	532.7657	340.7097	353.7941	392.1945	73.11711
PITPNM2	162.7646	142.4985	59.86894	568.8519	142.5291	305.5126	95.35363	217.7725	306.6591
PRKCI	286.8415	143.5824	236.8143	162.5642	608.5921	399.0626	353.5618	287.2382	279.4039
IGFBP2	856.7148	363.4149	579.18	813.5749	2058.447	64.06771	1586.141	637.2285	35.54867
GSTK1	116.473	953.3027	5689.039	1323.963	5927.709	4261.935	5747.398	4750.227	248.6475
COG8	895.083	141.8186	94.65553	207.386	70.73655	138.3821	59.78652	76.9115	364.9061
GTF2A1	3563.857	4482.112	2087.432	4218.082	1831.505	2758.499	2509.992	1104.349	6479.652
CCDC50	446.7509	337.7094	128.944	363.5873	66.99127	146.9451	78.61308	117.6707	248.6627
CDC25C	3043.446	955.443	1559.143	3484.621	684.6183	2209.006	1422.289	1829.894	3791.968
GPR3	118.4143	76.91804	68.51966	142.045	75.6711	96.70544	69.73006	75.81486	372.6738
C4orf7	44.64734	38.81051	131.7618	40.61967	32.99353	446.5553	117.0354	186.7166	35.99475

GCGR	1591.549	2198.155	726.5756	440.6185	1636.472	1541.979	1041.575	821.8694	4007.574
RASA1	2290.239	3489.689	4672.34	3753.606	5596.07	6962.221	6129.549	5827.152	2199.656
GBP3	1207.197	1549.985	2221.383	1456.68	778.2	2654.35	2361.438	1827.43	752.4404
SIGLEC1	68.09911	63.87314	52.82233	73.76333	48.65886	589.6022	138.3578	39.23458	137.4534
CLDN23	4125.488	3515.302	3331.854	2522.963	5250.97	5869.311	4149.753	3353.336	1943.301
SNAPC1	40.18528	47.82331	37.39813	39.10625	48.0166	45.82484	34.2028	35.16441	39.91462
SLC27A5	261.2917	119.0436	84.68598	134.5901	65.33153	73.13033	62.00951	83.58317	263.2895
TMEM200/	675.0933	326.1883	154.9476	359.727	189.9689	344.2584	679.9456	247.2805	196.6106
ABCC6	186.4126	155.695	101.7072	193.0042	96.41107	141.4006	95.88604	104.1098	394.3006
NELL2	96.55212	48.11111	116.0627	46.60708	71.93993	228.5583	105.2522	92.54176	59.35303
B2M	580.7622	2416.383	2640.905	1584.442	1498.051	2557.845	2424.766	2638.45	1016.285
GK	2340.646	47.99882	1231.71	62.0943	1643.763	381.5591	670.4631	480.5299	1919.682
RGS17	3068.907	2175.793	1166.65	1559.483	826.1126	1782.546	760.5412	1227.039	1541.65
KCNAB2	103.9002	37.15847	109.3246	45.28621	48.24277	319.8301	89.52248	41.81889	53.92463
DHCR24	266.3833	100.3225	214.9768	226.7266	198.4139	167.6509	155.9462	271.7414	308.5341
ANGPTL6	71.81712	39.73884	59.96504	35.75173	43.04541	45.34773	53.39629	41.83003	100.8525
CCL11	38.05404	33.18445	380.5431	53.80451	172.0918	1592.172	876.2554	1450.191	34.39928
LOC400657	65.80679	75.17265	47.90852	51.10788	129.7073	43.7821	209.3896	274.8842	55.03756
ACTN4	7525.453	9612.621	11186.6	10905.93	15505.44	10873.29	16112.55	11029.31	8713.812
DNAJC5G	218.9605	182.8185	84.34594	208.302	88.00355	118.5059	113.6368	95.78611	358.7736
FAM188A	638.5609	566.2255	799.899	568.1199	422.4375	802.9668	728.054	452.2647	61.24638
FXYD4	35.79103	30.15738	51.40302	39.14646	36.93589	36.51049	45.52752	33.05988	38.65969
GGNBP2	42.82263	46.02814	34.27959	37.59457	282.4688	320.0023	247.3828	33.45245	66.3504
SUMO3	86.01691	663.8854	2720.793	2778.949	4666.813	2746.562	5521.658	3821.178	66.97822
RAB41	64.35519	78.27362	53.3578	83.77884	46.84171	64.59582	51.09911	94.5478	152.3458
GTF2H3	182.3287	216.032	449.9853	402.244	562.4899	880.4841	765.1498	696.5026	114.3852
GFRA1	89.19396	282.2289	183.1745	86.56398	142.1221	72.59341	68.07085	99.74167	92.11814
ZNF596	33.90209	35.54568	34.27432	35.09735	34.04928	35.57718	33.48577	39.52002	36.76019
LRRC1	406.2852	229.8891	108.8205	249.2854	147.8941	171.4409	130.7062	126.8542	511.682
FUT10	71.97861	47.90442	119.4768	241.111	189.4294	32.62706	187.7094	38.56022	47.22901
OR52M1	42.88701	52.85717	44.87441	63.4875	42.25703	59.60911	34.42985	39.07661	41.02071
SLC25A30	1744.714	44.8152	257.1065	1707.022	176.9174	484.861	229.3265	331.8853	51.46906
ATXN3	2743.337	2308.531	1181.856	2423.535	648.3455	942.2341	850.5171	510.4777	2739.671
MOV10	281.5119	578.9453	3283.511	209.9643	2256.768	2025.892	3407.948	3580.321	238.6473
CLPP	91.24136	55.47887	334.5049	99.87499	203.4382	502.0513	556.1437	67.7422	2590.938
ATG16L1	2488.529	123.1506	6052.103	3258.665	8127.76	3702.006	5032.616	6790.625	416.6661
ZSCAN4	37.22492	53.16917	36.98389	56.27856	52.29407	52.72889	134.7243	166.3851	64.17227
DHRS2	393.0691	221.402	107.7338	241.6924	104.9023	212.0517	258.4463	132.3007	558.9005
MKNK1	1639.356	1085.601	166.293	848.301	422.04	782.7908	472.8852	621.5576	5970.215
TNRC6B	54.48602	85.72475	59.46317	74.43712	45.70868	270.4819	220.6227	163.1583	64.80566
YTHDC2	2470.75	1779.646	2328.097	2178.431	1461.899	1018.864	1186.582	911.7955	46.40438
POU3F2	52.45725	50.87242	41.96188	40.36058	44.98763	50.60452	34.77838	43.11846	37.46147
DNAJC12	38.57999	42.75393	40.32949	38.1234	589.6461	49.58492	137.9441	240.4164	34.28009
RFX1	606.3087	302.6743	374.7433	57.35162	584.6235	444.9464	444.6874	349.991	62.51348
PTPLA	61.53023	646.6031	360.6693	49.70798	183.0815	545.5461	490.6377	574.758	117.4081
GABRG3	45.60645	96.80949	49.18843	80.31398	38.36377	58.21095	39.3818	55.0861	63.49659
IL24	37.95308	43.86488	38.91036	40.0034	34.39847	49.21751	35.94674	53.92367	43.38437

PLAC2	87.17276	70.14535	41.89951	54.24134	50.41031	61.73642	115.8298	44.14886	63.73355
IFLTD1	51.47674	31.34529	39.14767	32.23498	39.10945	41.14372	36.29823	39.29233	39.83208
S100A2	1277.968	5703.492	7743.46	6533.239	9769.245	6954.3	10027	11500.27	1994.045
USP3	53.1339	44.40989	835.6611	628.2645	482.8273	36.07247	388.9841	175.6715	37.84134
UBE2U	314.5886	188.7575	93.51241	143.5332	141.1996	164.7664	73.62394	135.5988	399.2615
AFF2	83.69422	63.67725	134.1246	52.40157	39.64829	48.1364	137.7275	88.92355	168.6333
AMIGO2	356.6721	187.8896	158.8154	239.022	106.1918	154.9701	220.7515	210.0769	507.4333
HOXC10	40.8713	54.12219	44.16347	442.9599	60.34098	62.9985	45.75607	65.05817	84.61061
CNTFR	75.78648	437.9739	54.94296	54.9458	56.72362	42.95493	46.65727	41.64598	114.5346
KLK12	12433.45	6440.049	15481.37	4830.88	14658.61	12503.22	9318.449	13845.23	8700.573
RPP14	199.9739	114.4211	94.40995	117.9008	73.63344	108.2196	69.32144	108.2198	185.6911
TMEM81	52.13454	87.49506	75.09837	57.31591	62.35766	107.919	57.99215	68.32277	51.89283
RAPGEF6	735.3693	590.5802	1277.582	532.6258	1725.988	786.2424	732.5798	1393.296	1166.447
C1orf14	36.34762	38.81075	37.39525	48.69122	40.02689	50.52678	38.31689	32.81174	40.91601
ASXL1	1960.597	1381.304	1073.553	2145.252	870.5107	1457.494	1095.689	812.24	213.8408
GPX7	37.49824	38.30493	37.27129	31.5002	240.4527	785.5067	531.8657	465.4295	41.59318
FLJ27465	975.311	78.55713	61.03773	102.3697	183.3622	68.35065	132.9494	63.16444	206.8602
C13orf26	34.26675	57.05745	36.65454	40.96187	38.53491	33.44679	44.15844	39.50381	66.97748
PAK1IP1	61.78338	37.78699	1154.146	592.0284	686.4633	317.668	1065.781	968.7355	1227.062
C11orf51	65.81414	254.6959	442.713	579.4077	205.4702	221.6591	552.9083	336.7721	846.647
LASS2	409.1483	213.7687	121.8212	243.8681	161.6656	211.7369	133.9932	151.0137	500.8913
SLC45A3	527.3833	56.63289	44.65438	60.74782	233.7056	57.66541	179.8763	141.1135	51.27095
NEDD9	142.1741	74.44994	131.6221	76.19901	108.3278	133.8149	83.43303	73.29498	229.0869
EDF1	3181.022	4093.439	3033.209	2412.428	2051.426	1353.251	2287.268	3578.819	6085.438
ITGAX	887.3726	748.7008	460.5882	417.6168	108.6781	695.0038	259.8337	706.2287	2272.239
C19orf52	259.4666	323.2881	107.0592	90.86758	247.0713	79.5679	137.1043	203.1295	890.389
MS4A14	176.8457	88.05656	119.8184	122.4834	117.6482	85.53962	86.22684	65.24871	267.5226
CTSL3	255.278	190.8887	92.3433	183.4153	81.95181	122.6175	83.19501	95.38428	173.324
SCRG1	46.4651	48.64816	40.62099	42.62204	41.0928	62.11594	36.44542	47.73363	57.37351
GPIHBP1	95.18675	61.95402	56.94942	86.08533	57.33684	71.43298	47.21218	77.50381	199.1415
FLOT2	33360.91	33974.27	33950.02	36356.31	31434.65	31554.29	32411.3	28311.47	35351.75
SERPINI2	40.40995	50.20957	51.84079	39.26469	556.6865	39.13271	38.88343	33.10959	39.24281
CD1B	33.64476	39.35271	62.6023	42.02816	38.07856	37.06705	38.6941	76.59328	44.21762
TBCA	6840.95	5426.874	6682.136	8293.764	7301.955	4520.805	6408.061	6839.768	3780.63
TGFBR2	46.60498	60.25548	205.4423	37.95918	154.4386	37.46623	217.0676	38.38615	54.25386
ADAMTS16	133.4882	135.4739	57.51151	84.06707	75.85366	75.75974	60.33772	79.46365	213.5848
CPAMD8	46.04761	118.796	68.36812	90.29361	76.58657	95.71608	49.31284	81.99686	35.0879
SSX7	33.37497	39.86571	34.61386	36.07661	41.05836	44.11893	37.69781	34.41258	39.53993
ATP6V1G3	127.0644	110.2386	58.37641	108.1582	53.88317	56.07308	66.53884	63.14744	118.0666
VPS13B	32.26313	69.72189	32.49701	41.88593	47.48943	35.84763	37.58276	35.43229	39.13114
TMEM106B	60.76854	55.89252	37.67826	64.85217	37.68405	52.47229	41.04963	48.75689	120.296
C12orf35	655.4016	660.9941	276.6176	92.24406	131.845	270.5787	210.3926	309.2963	70.8166
CSTL1	52.55115	490.1244	850.4526	40.26307	259.2965	744.4505	344.8647	358.0034	36.17612
RALGAPA2	266.5038	112.8057	242.558	180.6589	130.7637	91.02453	97.98518	77.05814	100.172
BIN2	80.35379	239.6122	105.832	66.72094	44.09088	58.2112	77.5784	117.7868	187.9869
THSD4	2295.54	112.0496	1473.449	2124.263	440.0791	2051.817	391.068	340.4127	2089.773
NECAP1	838.6971	400.3269	455.6376	46.94845	631.7391	260.0714	565.5397	993.9014	31.68584

SNX9	557.4747	306.7002	137.2566	310.0545	79.94629	238.0739	140.147	182.4572	670.9183
ZNF135	4311.29	3960.649	2596.399	1597.692	3209.758	3463.005	2326.671	2469.141	2394.963
NF1	909.2415	610.0109	324.6596	1074.719	60.55156	353.4359	255.6682	340.7914	141.3522
ACBD7	68.76291	341.3049	306.155	312.0479	145.0455	508.9246	160.9716	279.9539	579.8105
MRPL39	2028.708	1676.32	1098.069	2058.185	682.2976	1083.818	526.9485	1306.829	2735.721
BSND	255.9693	174.3271	85.31964	170.3073	96.82645	143.3431	73.93667	94.08741	348.4384
METTL9	615.5139	608.1348	530.3051	59.26168	418.5209	203.0079	178.2557	257.7652	69.56536
EFCAB3	234.5083	193.068	81.49368	68.39624	69.31402	51.91231	74.63091	90.31425	90.08387
MMP1	6868.622	656.4027	6809.513	4266.03	263.1638	10040.9	19601.6	8128.563	1613.375
NPAS4	34.9985	33.26782	356.2465	56.56737	261.1851	280.0717	89.97855	52.04794	38.44911
RPL14	3314.203	3894.854	7729.316	4747.956	7462.964	4092.917	7340.351	6808.475	880.3768
HDHD3	17468.9	16125.17	16184.68	12404.37	13506.31	20640.36	13824.78	20581.21	17106.26
SEL1L	37.25504	633.0424	40.32994	85.7282	423.958	40.32357	327.4472	40.18766	222.5114
GOLGA2L1	127.2319	132.2945	68.74378	160.4369	68.41186	110.7868	70.07666	67.67178	805.6222
TDP1	3932.911	4295.463	4897.567	4009.932	3974.146	6727.04	4786.814	3595.906	7701.243
OR10G3	51.78868	71.31841	51.10412	64.90078	47.18184	44.41409	47.82877	56.17173	44.4932
LILRA5	78.0142	67.46428	74.76949	96.37653	58.07244	83.07047	63.35182	95.83562	55.45654
MAP3K5	475.8077	309.7811	340.7612	300.1503	243.5482	228.5018	357.5043	215.3905	576.6004
GDPD2	243.9886	82.35725	57.48952	139.5118	72.99181	79.42668	53.58511	54.39088	268.3766
IREB2	36.36406	43.29898	370.4884	37.6257	290.5841	218.8469	254.6024	167.8983	52.56644
PIK3R2	3951.368	2375.311	3141.043	4954.603	4090.282	4748.334	4110.56	3024.476	2680.615
CHODL	85.53402	84.73662	83.76686	121.6657	114.8818	95.40249	65.24526	60.81497	234.9814
CD248	2745.246	2195.031	3071.53	1589.397	2531.392	2465.508	3342.425	3468.164	1145.116
FAM65B	160.2716	79.00081	607.087	113.4538	381.8653	411.3114	596.5149	397.3828	163.7159
PPEF2	71.38638	62.64082	38.68811	36.05016	35.34148	60.22968	46.94982	51.35014	59.14937
ATP5I	37001.99	35160.89	32406.95	34839.26	34076.01	34122.22	32921.01	33445.7	37257.89
ZNF182	1770.417	64.35635	125.2326	74.22441	48.90731	787.8454	175.1412	167.7623	48.03443
YPEL4	51.25792	37.15826	410.923	58.15418	221.2777	43.69937	312.6652	450.9476	40.47194
C7orf66	93.06469	125.2632	51.14755	70.70576	52.15972	61.25953	52.01433	86.001	243.3917
THOP1	50.44386	63.44617	1449.852	64.13496	1756.573	1069.651	1398.276	2108.018	105.9944
GPX3	34706.23	30211.44	31680.09	27092.73	31919.13	25657.67	27202.21	28558.16	32986.82
GTF2H1	561.9183	36.83389	251.6785	38.14182	371.4601	469.8824	256.9381	433.3341	59.76976
PSME1	214.5305	126.0174	142.1393	152.7063	86.08263	115.5651	95.53342	81.99341	247.3244
RSC1A1	43.52871	50.12837	41.38196	58.76732	115.6518	48.85185	159.5263	221.9233	62.59808
PENK	44.87976	57.47118	42.3224	58.30062	1251.552	1956.935	100.7931	339.2516	51.85138
NUSAP1	8442.197	8060.674	7804.571	11664.06	7435.41	11195.32	7221.221	7712.098	7134.67
C2orf76	754.4614	691.0245	149.213	52.41954	372.7234	360.0341	213.1867	281.9733	123.9707
MRPS30	80.23592	61.91522	138.8819	69.76254	354.625	54.02383	432.8364	54.6835	73.02785
TMIE	52.97897	44.75216	41.61353	53.24301	38.23418	58.08764	40.13064	62.06478	41.07035
APTX	181.1668	216.017	111.0176	219.5722	102.5466	162.6069	93.24558	154.5389	389.3215
COPS4	112.7388	127.4286	261.4272	825.5476	69.16718	437.323	75.60085	66.24783	107.0434
CNDP2	1614.454	4162.031	8654.662	5483.139	8044.904	8771.143	11053.84	8927.796	1303.698
RNASEH1	50.78968	56.96088	562.8126	1099.073	869.2894	326.6292	708.0195	443.502	46.28591
UBXN10	90.47036	107.9055	60.84095	59.82207	41.5275	42.13076	47.52682	47.55307	186.5333
STX2	225.5098	81.65006	84.28606	90.24456	49.94706	76.96075	53.10606	36.94691	92.58282
BHLHB3	10731.14	9032.722	5312.245	2969.618	5515.063	9073.623	5491.732	5771.012	4408.107
HIST1H3G	516.5792	437.4682	198.0183	515.5745	180.6658	585.9867	425.8428	141.4997	96.65939

DCUN1D5	47.12964	44.1899	48.03454	46.82938	130.6574	46.26325	842.3982	167.6436	40.12687
B3GALNT1	161.5619	230.0611	133.7025	222.3993	160.7826	178.293	136.4245	165.354	411.4131
TYW3	815.4663	491.7119	522.3741	534.916	261.0106	285.61	277.1262	479.6402	823.5168
TPCN2	2287.83	2747.34	1974.798	3284.11	1722.753	1947.254	1795.493	2410.753	3215.669
PRR15	286.9266	174.6001	64.9451	171.7053	81.38235	139.3384	92.69939	63.61517	478.6783
EPN3	554.2509	507.3595	506.1454	385.8994	2089.532	599.9819	589.0822	892.4139	683.1949
AKT1S1	61.98184	44.40806	43.49147	61.03196	248.4602	51.31966	100.2834	48.77235	35.82707
SYNGR4	693.645	57.01645	129.4058	46.14838	41.75214	330.0025	94.98519	54.68136	159.0679
TCEA3	716.9755	1137.831	575.7402	660.2326	633.1282	1218.308	1330.155	1195.167	950.2968
C5orf44	649.1748	38.61249	41.09927	255.2406	62.08179	103.5701	116.4465	197.2437	35.82418
FLJ12331	35.18114	41.29745	49.46609	40.10058	34.46779	32.598	85.56812	36.26162	784.5035
TWSG1	773.155	128.3164	337.5337	122.434	70.06435	119.0155	59.78172	188.3395	959.6406
OR51S1	37.40531	35.69953	40.20558	63.21247	36.36758	36.17082	35.92056	40.25169	55.64014
C1orf86	10494.61	9658.499	7944.565	10120.88	10164.44	9152.682	6724.053	8727.326	9664.614
DOM3Z	162.863	63.93496	274.3988	129.3856	73.49773	89.54278	56.01165	66.87498	135.088
DDX46	68.55217	42.15311	132.5818	60.5389	148.6973	227.9099	171.5296	177.6641	49.27785
SFTPB	157.7251	69.82059	78.0839	67.4189	54.36497	99.91777	50.70624	54.17192	171.041
AMMECR1	2169.855	871.9258	1739.459	1542.258	1936.699	3167.555	1847.398	2422.12	861.2525
ARTN	97.11429	41.48858	42.62075	76.90723	41.71051	53.20728	85.26797	43.88593	65.53796
GLT25D1	1234.229	248.0195	1692.079	666.5658	1878.181	1452.35	1909.922	2638.713	656.8746
RASGRP3	1103.195	498.4194	1840.555	598.8107	2289.852	1937.88	2083.494	1040.574	40.69921
PPFIA1	56.64471	35.86132	1246.991	2623.807	1444.159	1447.891	1586.104	776.1452	57.56819
DDX19A	694.9819	1420.577	409.8963	456.2079	596.8019	892.936	488.2694	746.8455	985.6426
C12orf48	792.8702	1142.698	1568.263	874.0258	1664.877	1473.748	1342.606	2032.052	67.25536
FLJ42289	45.92476	65.10591	37.62593	78.85949	40.92036	39.6953	46.24613	41.10199	60.94831
MEA1	632.3845	468.5763	321.213	408.2509	252.3653	325.0061	251.5241	269.9978	802.9322
C17orf37	75.43643	67.18928	249.1901	223.5589	303.0376	187.3842	249.4995	451.8447	105.2471
MYO6	523.9304	1038.787	2484.82	1998.059	2344.29	1117.576	1913.003	1760.144	703.0201
GJA1	1297.615	1838.929	885.8734	2679.058	1820.942	1615.286	1828.803	1441.996	82.58516
ACMSD	35.04709	46.3689	39.11571	35.31568	36.32322	42.15673	34.16082	39.96677	38.43845
GPR158	301.8241	432.7708	104.9267	169.8713	64.40551	117.4645	66.51785	209.0652	623.903
PPP1R16B	108.2016	286.6565	386.4247	309.3853	260.8675	236.4645	274.657	279.7263	70.88354
LUZP2	239.3605	182.8125	102.5334	187.8869	174.2239	123.1256	71.53314	95.4376	421.0118
REST	172.7594	669.1525	522.368	112.6185	254.9807	1031.079	476.0012	500.7025	1613.283
KLHDC5	4273.26	2600.042	2807.358	3981.695	2119.561	2241.763	2029.359	2640.907	3760.157
FIBCD1	551.2963	105.5457	126.6663	75.04373	93.66947	93.34553	89.38546	62.93253	190.9159
IKBKB	2722.949	1625.335	1081.833	1718.385	796.5148	979.1938	745.0187	384.0212	2337.233
BIRC8	44.1577	45.95279	40.1087	44.70449	49.36041	58.13552	32.89749	32.22277	41.10618
DHRS9	103.2569	68.55404	46.13416	101.762	48.60296	44.36555	44.57069	39.74802	57.12694
ARSI	37.51258	44.99765	114.4527	52.03623	130.5665	266.3771	73.46526	119.0289	35.99492
SMARCD1	77.44012	240.5247	252.8934	78.95096	39.95628	44.42833	186.3136	252.4283	231.4916
C11orf92	35.83337	38.05531	105.7266	303.9043	52.59633	39.87831	52.78772	37.26576	33.53773
ZNF763	51.01173	438.6841	438.2088	68.12617	300.7813	397.8545	197.0225	173.4814	43.92838
WDR36	76.54678	49.90002	300.6446	273.6408	454.4526	36.34485	316.7354	239.0159	99.10214
CALU	1842.802	1507.952	1134.511	1949.467	1671.928	690.3979	2273.518	1456.988	1496.226
GLTPD2	33.2952	39.3047	37.14867	35.97369	38.41179	45.39842	40.64949	39.08815	75.14252
SLCO4A1	193.0224	160.962	89.92849	536.261	102.5872	119.7021	114.0824	103.5098	305.9376

ZBTB6	266.8904	162.5027	90.60054	130.4055	102.9611	111.9852	83.01865	101.0665	411.6669
CRNN	17188.61	2363.331	24234.02	7334.728	20950.06	16510.89	11077.68	6054.884	12239.71
INF2	2367.062	3659.649	1216.743	2973.429	1393.38	606.3037	2191.561	2783.559	5383.93
FAM21C	48.05481	54.90728	35.79264	54.46121	52.14566	52.28198	39.1592	36.21737	85.46362
ACCN2	45.68576	53.12815	54.29504	43.35421	45.24306	37.44596	42.59865	43.01227	48.5972
AMELY	219.7227	116.7874	78.30944	59.31435	80.91399	145.0996	67.70508	88.39359	68.9968
WARS	57.34063	929.8318	2265.762	57.93538	1264.637	1802.051	2640.626	5672.79	40.17249
CCT8L2	50.44912	41.44431	40.01357	57.20542	34.42646	33.37024	40.31462	40.53507	48.6118
TAF6L	11596.4	8423.912	6655.041	6860.427	4324.279	9248.402	7063.291	7322.488	10218.17
CD4	58.62951	45.13417	67.58395	46.11839	87.09544	134.8352	75.23883	107.3408	77.37926
PHF20L1	645.0023	985.8315	1753.77	1340.417	1840.449	1652.05	1712.122	1890.597	52.61912
EVPL	25876.73	25387.9	25248.27	25672.65	25787.2	24094.86	21607.58	27066.17	27706.86
ZNF444	77.63271	171.1977	233.8806	580.2448	485.062	120.1276	381.6169	572.2103	79.30548
FAM82A2	1245.705	243.6203	534.8924	268.7608	988.8024	987.9818	829.0551	730.816	612.3659
MUC4	345.1872	125.8325	83.03632	187.6127	93.13983	145.4066	76.12574	111.0661	454.0883
DENND1A	42.07043	35.25196	169.0314	73.2755	43.25854	36.77747	95.69755	159.8599	34.07609
OR4S2	33.70214	39.75466	37.81503	42.94049	35.98783	35.61151	37.42679	52.7436	49.42873
MAOB	163.1542	80.32883	105.5609	47.01399	81.62605	67.75077	51.94339	80.14967	133.123
LEMD3	2694.311	2181.156	1934.607	2942.28	1683.643	1787.51	1814.15	1691.206	2557.564
CDC16	7239.616	8730.504	9719.651	8006.312	8451.12	9279.096	7147.035	8363.708	7930.928
ATPAF2	533.9043	1058.899	1031.417	650.5562	1418.96	792.9021	1194.578	1830.197	50.53226
FBXW10	206.5857	148.5744	88.97593	120.0707	96.91873	136.5859	125.8664	118.8262	329.9019
HCLS1	1099.033	1130.384	393.044	439.1789	2102.645	2263.007	3461.667	2156.802	1545.329
ERG	120.4017	236.1097	72.84433	65.99247	70.82944	65.58531	76.53114	62.46555	158.2353
KCND3	225.2632	149.7274	81.72177	133.7957	74.78461	93.33186	73.09347	83.42813	370.6815
C16orf53	517.5101	312.1063	163.3317	369.0884	282.2769	250.5099	220.1098	260.5295	1466.142
HDAC1	3889.552	5560.613	7788.069	6021.657	8753.002	9519.746	9743.412	6635.059	3246.304
ORC1L	384.1916	203.7969	79.44806	228.3118	81.29404	127.1225	169.9312	90.33099	465.1776
ARSE	34.26785	33.37908	30.78589	38.44363	38.46564	49.13716	118.3123	41.50938	45.07401
KCNMB4	5548.438	2627.932	1344.023	1799.56	1707.873	1956.787	2299.911	1172.282	1553.672
TPRG1L	7227.088	8501.062	9580.394	8034.255	10477.6	8286.661	10087.09	11472.35	8189.884
MYO3B	100.4409	52.91087	44.07368	36.81313	46.43777	52.02322	54.06812	53.81775	38.62194
L3MBTL3	1641.302	41.06568	2133.997	503.9456	1371.395	1725.831	1662.064	1555.083	43.69457
CCDC158	45.62489	35.50235	49.19855	44.3055	34.42234	35.89956	40.14233	37.94404	42.80595
ADAM9	362.9259	191.6928	342.6466	194.4285	888.4751	169.0086	604.8304	817.1438	1464.829
KIAA1407	98.05897	89.10869	58.963	55.9179	54.89427	869.0427	42.2468	316.6572	47.87996
OLFML3	281.1782	1614.258	773.4545	746.3176	766.6961	135.0708	1061.529	773.2847	3624.455
C1orf112	246.6904	163.4433	536.3543	169.5505	423.8616	100.478	441.4461	247.7821	322.3488
C5orf54	7211.313	5959.365	3360.168	5881.514	3582.71	5882.297	3214.301	3649.805	6589.199
MRPL27	184.6552	131.5841	66.76241	93.97809	61.18184	57.96924	61.31933	63.78656	159.5081
OLIG2	107.4328	51.17696	55.60982	91.24328	52.47434	59.60722	49.67585	62.9432	51.44409
ZNF827	12601.65	14153.21	12966.57	12421.06	12022.28	14533.93	13303.51	16398.66	9887.638
GLI2	39.48103	35.67459	55.40314	846.3513	256.4912	475.8185	204.4745	326.7308	51.24376
C6orf107	151.1549	62.74185	57.6271	56.72564	55.97274	55.97988	50.45334	46.88428	177.5363
FRK	4765.994	3599.345	2096.992	3989.933	1644.698	4108.935	2756.011	4419.138	6025.361
C12orf59	78.18782	65.59579	47.45314	51.32861	43.56035	37.83113	86.4191	66.31287	65.03175
XRN2	1053.328	94.25816	548.0547	44.842	138.2386	62.7516	224.986	327.4823	256.6357

C8orf38	39.13664	356.0267	175.7855	43.01301	82.97067	261.1403	163.5986	72.77585	914.1165
PVRL3	91.63124	49.03073	223.819	104.4639	49.47127	60.93316	43.68341	369.9816	67.25978
MOGS	64.38215	1484.973	263.4052	957.5312	240.4815	50.44238	114.2697	34.00581	33.53061
ZNF155	127.9178	63.59047	59.30241	83.94004	63.39866	64.35992	129.6234	75.59473	102.6239
FLJ44674	321.3918	89.56297	52.04237	135.2566	69.53627	102.9658	67.04807	93.9056	284.4273
SSH1	45.24894	41.23866	169.5596	42.76555	259.9841	344.106	106.9265	46.30395	58.79321
TNFAIP2	1082.965	1631.369	697.6398	1390.024	484.184	477.7856	302.5487	549.3346	1686.714
TRIT1	575.8937	551.7715	369.1887	387.7471	373.2673	263.8683	382.5471	293.5616	765.1852
GPBAR1	830.0093	369.636	478.113	117.2858	487.7211	578.3353	521.5268	350.3501	118.5427
TEKT4	42.6309	61.48758	41.75684	35.43637	46.04013	61.40013	49.06646	57.76859	37.06191
RAB33B	7333.704	5813.938	2842.589	5102.208	1950.213	3751.162	1434.547	2237.4	5789.392
SYNCRIP	6855.342	6733.838	6839.403	7557.637	6177.972	5922.224	5610.972	6047.173	7628.706
LDHC	52.79679	35.01911	37.81335	42.80102	33.03294	33.19651	40.65299	35.01832	61.57787
IDI2	35.16648	49.46098	39.49093	38.51224	47.64167	31.98072	34.50487	45.39338	71.16492
GMEB1	155.2019	156.9973	67.74596	193.7028	356.0915	66.23618	151.0916	89.46031	2226.97
CASP1	36.24583	36.69132	59.08544	39.74943	33.84722	40.76509	48.91414	57.64982	39.80853
NTNG1	45.05537	392.8011	32.79081	42.10205	122.931	37.04704	35.03491	32.07461	35.14333
RNF12	94.80921	147.9076	221.402	168.4033	58.55114	76.21095	75.25584	116.134	201.2437
MOSPD1	588.416	376.6716	407.6016	329.284	384.6059	452.6461	326.9281	255.4787	786.7831
STK35	100.0778	352.1884	135.2005	37.93011	34.72563	32.78186	81.65188	117.711	68.9435
BRP44	148.2677	177.9865	303.3558	220.4141	474.494	332.0501	242.2447	129.1349	386.9853
CFI	1143.351	1685.526	636.9117	783.3983	247.1343	387.582	477.3107	440.4397	982.8751
RAD51L1	146.7034	272.9445	98.95129	116.6043	101.1821	114.0938	90.98473	120.1067	413.823
KIF19	55.21375	45.97084	35.43215	40.91343	40.7886	38.83813	36.86548	33.02646	36.29667
SLC35B1	62.75887	107.2425	398.2057	708.0977	1458.689	611.2684	1365.783	1021.191	53.65401
KCNC4	197.1762	126.3201	75.51266	154.4972	100.4015	141.1432	72.68876	103.2538	197.7381
FPGT	69.31391	35.9301	37.63085	38.38108	110.5939	49.50315	36.97757	40.17077	61.69567
CDSN	1243.996	5035.537	782.7462	2031.224	6260.481	1686.179	1603.989	3565.876	4777.114
C6orf188	47.94494	50.50039	47.68298	37.96818	40.28845	58.61865	36.25943	32.47115	43.54772
ZNF140	646.6978	516.0946	380.9856	509.4726	292.938	545.1499	360.4461	438.9097	688.0952
ZNF764	95.38585	40.32232	316.0115	61.47631	48.17691	374.4841	154.9941	37.17342	157.7485
ALDH3B1	67.55428	48.39817	50.64644	35.51377	163.4998	57.11628	33.34966	34.4232	34.34364
ONECUT1	92.17329	78.06145	47.36711	49.54515	57.59367	68.3252	42.99467	60.15611	69.172
SNTG1	44.88843	37.3695	38.92822	36.42341	46.3433	39.82065	49.1767	37.41673	49.533
FEZ1	1633.206	470.2163	1113.126	4942.566	334.7933	496.2682	573.6067	692.9843	3509.766
BAG4	2202.399	1431.981	1910.65	2242.938	1349.147	1527.022	1446.07	1047.97	2082.861
SFRS12IP1	33.23133	38.24883	33.76521	32.69681	46.64816	35.43102	99.96354	44.46338	66.23245
TMEM171	35.56085	40.51143	147.1966	431.3032	37.73741	285.6374	170.0828	319.3098	891.4111
AKR1A1	36.85926	36.97567	1470.628	44.716	1814.285	1002.898	1574.553	1285.285	50.48139
LOC15816(	9970.137	8772.913	8893.401	7808.683	9944.987	10013.21	8297.045	3426.651	7345.829
S100A16	2680.132	942.0034	1625.473	1669.246	1260.427	1167.387	975.4087	1267.535	2383.324
ASTL	180.2901	103.0955	60.53682	110.0569	68.8907	86.68276	57.47097	61.97812	180.0179
C6orf57	119.2107	288.4129	145.3434	235.6922	167.135	170.6829	166.0194	126.1688	345.8248
TLX2	46.17888	37.49111	35.95462	59.70222	33.63744	37.9635	35.97315	42.43672	35.56088
OR2F1	43.80717	52.07635	44.17151	43.47057	39.24783	50.45975	38.49767	43.18825	35.78571
PHAX	568.3764	923.0762	1310.185	1353.177	1623.763	1448.011	1311.864	1464.213	318.3394
EIF4EBP2	696.4427	1859.662	1057.897	699.9861	329.4968	695.0556	565.3432	511.5374	1818.443

CCDC75	438.8489	220.7126	102.5025	206.1472	71.88656	64.2931	79.47189	90.99817	442.9127
TNF	68.8809	45.32417	99.9149	40.0398	40.96881	139.7013	189.8979	86.0239	42.80213
GNG7	304.454	839.9106	1211.333	216.1535	1140.262	463.0786	731.7053	546.9592	359.7999
NKX2-1	44.25022	63.1484	42.17912	46.64347	41.85765	48.36163	119.4731	59.71024	44.83935
OR10R2	45.04095	38.19561	41.78308	54.88035	34.32423	35.36121	48.74986	37.81591	46.02652
LDHB	614.9399	569.1309	811.3216	852.0008	1377.559	846.9095	1527.563	1273.511	238.1683
OVCH2	174.5154	41.16114	35.86186	63.1768	35.77112	51.73606	53.93706	59.40546	41.59232
C20orf45	106.1577	106.3229	633.2556	827.8555	1685.443	915.0095	169.6335	663.1769	2963.316
CROP	60.50053	64.8138	52.37169	56.4283	58.71528	58.3878	46.12885	53.59082	57.74898
DMRT2	51.77988	68.54917	57.45261	62.60823	62.66372	74.98357	53.53113	62.6152	132.5378
ANP32D	35.15089	41.11894	43.44991	51.43468	54.21257	50.15176	48.49728	46.01178	111.1138
CLDN5	5310.311	7934.957	8335.594	6412.616	10051.6	8277.08	12656.83	12568.12	9359.712
POLE	2347.62	401.5359	1387.089	1465.964	1364.388	780.7631	759.8679	1372.679	41.52554
TMEM22	64.09617	367.035	280.3017	458.6075	544.4352	304.6403	166.2051	374.4793	1086.653
OGFR	121.928	1173.954	618.7262	1230.791	1127.825	79.6703	1709.937	1380.594	78.72503
KIAA1524	77.96108	73.52879	126.3676	34.54919	187.1952	70.43804	126.8907	177.9034	44.70238
ZNF548	716.4677	961.8846	593.631	1511.312	275.9272	818.3645	702.1741	442.5825	1121.552
ZNF30	157.2125	76.22495	166.7123	119.1697	182.6662	246.4762	180.5713	237.0892	398.3242
SLFN12	50.24602	62.43902	51.97076	38.66409	50.31146	50.18001	124.6965	58.11674	80.43155
LALBA	233.7632	127.0319	81.38186	104.0185	66.03121	85.33967	58.60431	90.52228	262.0387
SPAG9	157.2003	90.91713	127.3147	109.279	691.126	76.29214	227.7597	420.1109	86.65014
SAA2	9407.697	15088.81	9108.348	11063.71	1420.683	15105.61	11946.88	17789.2	11046.17
C14orf177	376.2701	200.1516	112.6175	213.6706	94.65197	183.4389	107.5045	136.2155	422.7405
SBK1	210.2273	352.8645	677.3295	365.1497	975.771	1438.608	475.7096	745.1243	174.5112
GAD2	79.02393	71.70881	51.0078	86.09264	52.09677	55.26778	49.36222	64.82026	62.1689
PUM2	59.49677	90.48348	51.76781	51.18117	494.1816	59.52532	286.9991	37.86876	76.46477
ACPT	276.4684	102.0808	85.1272	185.595	78.98537	132.7825	59.50073	80.13942	403.1608
LOC340527	38.62553	46.7954	152.3059	51.85689	195.2963	58.09924	129.475	166.1331	1185.191
LOC400499	51.16526	72.40147	49.18096	54.96151	52.94008	45.0303	44.96567	39.81332	47.59605
TMEM63B	244.2639	200.7831	92.49315	1036.035	74.40734	117.0686	80.4967	377.4913	244.0214
EYA3	45.41894	43.58098	36.51763	45.18649	39.4352	40.74828	35.43922	34.42661	70.35374
MED29	301.6281	353.6288	250.034	363.4447	301.7158	230.5819	349.2985	164.0104	168.1115
CLCN4	40.14128	882.5382	43.89184	41.5064	32.27732	42.19574	37.19036	710.559	35.48199
PHOSPHO2	769.4775	296.4596	359.3331	34.97258	230.8993	282.5099	173.1499	231.6378	51.37099
C21orf119	166.9388	186.1786	86.56398	143.4182	219.8871	126.0842	78.39932	69.77841	227.3531
INPPL1	316.4414	1437.991	2246.303	600.4736	2081.71	828.4539	4941.192	3273.485	385.9013
C12orf45	418.3589	495.4612	308.268	522.319	264.5256	171.2219	174.1839	88.18472	468.4424
KRTAP6-3	75.09093	63.53785	37.49371	41.80075	44.06444	55.30277	48.60339	40.13274	75.6124
IGFBP1	70.49464	51.27354	102.348	85.99268	68.4787	63.84626	81.99791	100.6194	80.9102
GCM2	38.55606	42.2018	38.25219	50.10661	35.74522	38.9557	42.79809	34.99786	57.3846
GHITM	6341.323	8156.439	9399.892	7544.903	14659.27	8689.222	13344.71	12049.79	6247.505
ARFGEF1	1093.527	457.3106	643.0534	1258.527	397.6571	516.3759	490.0967	487.8867	569.9782
SYT9	80.86013	59.28152	64.06934	36.2248	62.4934	73.51276	47.806	59.74992	63.76341
CHRNA9	93.66028	72.50614	66.22398	87.12066	68.00872	77.37626	74.03037	51.60173	95.9812
YWHAH	14482.63	13903.02	16075.37	15626.82	14953.74	14677.35	14274.22	15422.7	12007.3
BAG5	1873.607	2425.062	2173.599	2640.239	1567.447	1929.139	1416.066	1410.49	1677.3
FNTB	78.26703	2486.523	3272.5	943.7629	4016.118	2593.919	5043.574	3198.085	75.64436

HDAC7A	2223.007	1594.588	331.0397	274.1485	424.9795	396.5183	298.6609	533.3214	3278.481
ZNF655	41.55404	33.21479	33.28945	45.06076	34.1791	49.48512	112.4185	159.8387	35.61255
COPZ1	37.23688	674.3307	219.2334	36.54034	157.6531	33.15578	104.593	59.76513	48.5758
FUNDC1	1434.098	482.4562	215.3893	888.0557	560.11	168.7849	578.8907	146.2841	518.3004
DOK5	42.72662	54.84837	57.58136	129.1638	75.85964	113.4969	62.8984	70.32836	88.06722
C3orf18	250.9646	745.9889	75.67271	120.1251	60.54727	401.2581	247.2135	456.191	2248.419
TTC3	351.2397	1401.975	2276.708	1223.368	2951.222	1609.9	2498.726	1377.32	457.3498
WDR5	1668.219	73.47202	166.3051	58.39592	309.257	62.17351	346.8702	216.6133	35.69445
LCE3C	41.41129	353.8846	44.86213	61.90036	47.72778	42.68002	74.77493	148.4787	83.88655
PMPCB	550.897	766.118	1656.97	1156.63	2284.596	1694.308	1580.522	1132.016	746.3416
IL17RD	63.26939	37.16285	172.9457	50.43322	46.33649	45.05411	42.62248	37.80615	57.2664
KIAA0196	175.4033	155.2622	92.39894	116.771	90.27305	97.59816	108.8069	62.26468	76.04034
PELP1	2508.195	4215.764	6946.836	4043.219	7755.202	5533.212	8158.908	8949.651	1091.005
GDE1	35.05861	59.07831	282.1828	71.12623	479.4583	36.90289	498.8258	514.3727	38.15954
SPTY2D1	3002.499	377.5154	246.8937	55.3011	193.5941	473.0462	132.9385	47.90982	131.3269
ZNF816A	7567.226	4697.411	7111.032	3805.664	4743.961	7099.74	6915.704	4525.83	6882.394
SSSCA1	81.19089	87.1348	69.26241	112.8019	56.42048	58.0918	52.15538	50.56292	256.7326
IL17RA	3111.092	1626.339	591.0972	2051.017	558.194	423.451	956.699	843.1702	2086.112
STXBP1	913.7136	991.3441	536.3394	446.3519	800.3574	300.8879	971.8529	717.0448	1331.535
NGLY1	46.08115	348.9698	579.8093	47.30304	601.9617	314.3449	650.3423	424.3383	33.59476
GPR116	5076.46	4863.75	2548.38	2783.797	2685.027	3180.135	2813.949	3314.116	6384.304
AGR3	108.5081	90.33339	70.19954	159.4205	72.55226	96.31763	65.90604	122.0764	159.5523
FBP1	67.25315	76.6412	66.0407	69.90881	352.9837	697.7759	344.6509	1008.583	112.9083
SDCCAG8	67.70992	62.77161	439.9434	52.10026	599.2527	511.6911	496.795	820.2336	4562.951
HMP19	125.4214	337.9272	85.6522	176.5914	136.5838	113.3191	174.1642	108.8309	66.5901
NDUFS1	97.00123	314.7885	37.83874	259.339	107.1463	31.86689	67.24072	35.71222	571.945
GRK5	183.2678	142.3854	76.96019	163.9957	72.81715	110.4013	57.17827	67.31625	302.4789
PTPRN2	106.8832	218.0104	61.24052	247.8195	50.05489	83.78123	56.97218	94.46713	641.6973
DDX3X	461.8177	2552.305	1666.503	1420.566	1945.017	385.8384	1123.477	1408.106	2220.208
PAK4	143.1217	188.2919	79.51775	171.0549	94.22451	97.81594	73.61635	86.3018	182.3447
OR4A5	39.18258	35.49244	48.76548	36.78562	38.59422	38.89028	40.34007	33.68141	46.68797
SCAMP1	4877.867	4930.042	6455.594	7398.288	7336.313	7057.71	6930.032	6944.013	2733.855
PRUNE	2856.909	89.41325	334.4802	95.59617	617.0192	61.65841	490.6512	525.9722	882.3465
F2RL2	48.59345	41.3802	166.2698	50.13105	133.0137	755.6568	188.7705	124.174	31.60097
PRC1	1042.662	452.7281	1212.648	525.0072	1901.799	1482.358	1562.547	1067.038	331.3569
ANKRD5	72.7135	51.167	48.47687	51.85141	46.23365	49.43991	44.04626	199.4583	34.75184
C11orf57	284.8925	228.3648	414.8731	301.6316	351.5237	338.5016	308.0372	207.2429	108.9154
FAM64A	41.36568	48.71367	63.4245	78.89241	73.52775	58.35084	140.1495	44.08988	76.44569
SCO1	294.0223	1694.674	1050.359	1290.151	830.554	1193.576	761.4168	2102.258	177.8115
NR2C1	80.55353	585.6222	1634.48	1099.955	1684.259	1039.841	1879.341	1086.243	76.31745
PA2G4	168.2947	1723.436	2360.721	2873.279	2144.541	2208.737	2599.805	3235.378	2361.384
Septin 7	1004.947	916.6329	712.4097	1820.379	710.5784	675.0977	695.9855	448.4403	1019.285
OR7D4	89.43146	84.87505	53.45097	69.56858	56.80316	40.28472	47.87431	56.60834	141.4941
RPL9	493.6744	1278.56	309.326	548.3467	231.3197	439.7875	202.8974	491.5709	655.3166
MSH6	418.0815	2759.977	4721.661	4171.049	5847.826	5691.629	5584.02	4632.89	1278.197
LOC730347	52.75014	35.13858	36.95627	543.8641	36.60831	42.13007	98.26952	175.0305	50.45577
PTH2R	46.59719	89.23	38.82875	57.89107	35.19654	39.21486	38.97918	49.70463	39.12296

PDZD2	947.7416	1353.05	985.7623	818.991	127.4939	650.6079	505.2721	421.3674	2058.879
CHPT1	65.46719	51.53176	209.0656	580.4974	83.04447	59.95687	68.77299	102.3602	181.9059
COL5A2	762.4075	1409.356	2194.701	2149.201	2770.177	4089.446	4622.746	2661.516	985.584
C7orf42	712.4186	35.95219	128.0934	66.53886	119.2997	30.15742	102.1043	32.8317	40.7995
CENPV	39.24921	46.63247	44.49274	50.32805	173.7269	55.35707	108.9498	230.9984	61.7366
EBAG9	34.53553	34.44135	275.4223	603.4452	33.36404	31.72469	86.08839	32.00319	31.83987
NOTCH1	1634.316	635.0571	1194.359	529.5517	1030.205	1897.105	1210.372	784.9109	759.4613
TCEAL6	34.02879	38.99601	41.58222	35.57745	36.20274	42.3812	40.0983	51.71751	62.55172
EFTUD1	39.3337	808.9838	272.3856	34.25845	238.8669	547.2001	315.6213	513.8166	45.52855
GPA33	361.6241	219.944	104.5765	231.8568	109.9425	163.7712	98.54553	130.0176	543.9399
SPATA5L1	145.9202	71.51944	62.58455	79.90039	88.21881	71.35755	61.99193	82.10062	64.57599
GATM	40.45697	34.66098	301.1646	37.38625	92.01682	42.50148	144.4241	125.7406	43.28493
HAMP	86.36338	54.12064	355.9578	92.66586	37.95073	55.38525	185.3263	532.1597	54.75295
CYP2C19	42.35543	37.26485	49.00748	46.72076	239.4228	48.99365	35.50575	41.22285	73.96223
PDILT	68.30181	64.07891	48.35118	37.93578	48.87826	52.12802	41.27763	37.98855	47.56912
NGFRAP1	990.6524	744.6898	833.4157	1042.176	753.8209	996.5607	1072.006	927.7552	227.2295
GRM8	202.8442	392.52	145.6027	99.29704	113.6949	424.1721	345.0471	121.5284	111.1628
CRYAB	1082.862	2343.826	4517.607	605.6971	5243.053	3642.366	5307.591	6207.477	1721.346
FAM131A	2845.711	1729.318	2472.92	2894.635	3760.01	2973.286	2558.162	2091.586	1910.733
SORCS2	61.78978	448.6404	55.82608	48.97614	114.0482	305.9544	298.8474	266.4139	41.88546
TPP1	4744.202	1626.47	6086.997	3016.277	5988.683	4221.791	5663.961	4967.815	3205.701
CYB5R1	6648.666	9154.465	5038.237	11684.43	3094.138	7365.86	8206.566	6280.441	10959.42
MYH4	44.27042	35.05696	33.02071	39.17812	39.5553	40.69152	38.0447	148.2906	50.97314
DIP2A	749.7247	356.6062	456.2281	387.8018	342.7459	269.4965	191.8361	200.0919	1155.738
SPHK2	5735.339	5662.096	4261.657	4270.668	2991.69	4916.542	3527.523	2977.358	5249.218
NMUR1	59.40804	38.43503	101.0422	73.45344	35.7126	44.67157	42.55964	51.89267	96.78075
RNF130	503.0052	243.9341	145.7979	350.0917	232.1042	229.3481	205.1971	157.217	569.5877
PRG2	357.024	204.694	106.4254	248.8868	97.67422	161.6862	136.2996	107.5182	339.3496
TOR1AIP1	133.1803	107.9594	1250.374	1106.882	1973.581	1392.469	1265.161	1306.219	124.5923
USP7	232.8666	154.998	519.6791	178.131	1137.401	633.3745	709.3123	1136.896	273.6741
ALOX15	75.30821	48.83878	53.7967	60.14211	56.98508	67.70199	52.62212	38.40966	53.03767
SLC7A5P1	46.13331	31.30102	93.9974	45.73831	36.78794	33.07638	39.3871	36.58575	34.85334
FZD8	51.85389	648.298	159.8954	1338.908	509.098	764.0369	537.6537	411.712	64.43918
CHRM2	68.32195	122.9926	56.95009	41.85895	55.29365	76.89835	54.21493	61.35315	210.6603
GNE	230.0081	188.2655	126.649	263.2425	174.6667	187.3295	150.9817	132.8294	378.753
LIPH	223.8515	363.8977	143.215	154.9897	156.462	247.6578	102.58	359.8846	1045.959
C16orf62	41.96461	99.64891	160.0965	45.7478	505.7451	716.8231	320.9575	464.9756	48.21875
TSPAN9	18175.74	20279.03	17315.48	22148.47	20835.21	24618.25	22581.92	22586.33	15757.59
EML1	34.23987	198.8752	244.0922	484.4535	256.1071	258.1483	254.5083	339.8292	40.22055
PROL1	107.6737	131.0274	74.9687	108.7462	67.13177	95.31536	60.01735	62.35905	181.0428
KLK15	97.03113	44.76226	37.41137	40.44194	39.76435	47.4416	44.0251	50.48736	35.86241
GMPR	272.1032	123.0053	102.7723	169.0122	158.2398	150.3893	100.3041	98.69803	531.7077
TRIM41	1474.588	669.8594	787.7537	1026.337	845.4652	769.3792	785.199	955.5043	1148.587
TMEFF1	63.80782	91.71517	56.57108	52.97335	72.23857	41.30759	38.46694	71.11053	53.04929
PIN4	2387.093	1372.428	1073.295	1769.389	849.4856	1822.835	1218.258	1078.455	1350.204
BAI1	416.8358	238.0439	99.3722	160.2973	132.6996	193.214	82.12056	146.8122	438.2686
APOA1BP	159.3192	2346.135	3429.329	670.4986	4528.169	2365.751	4859.037	3989.982	1893.595

WBSCR23	126.629	99.86672	83.22411	119.3694	77.7989	141.6752	65.02424	66.48126	76.00236
C19orf54	4832.702	4932.454	3296.521	3586.89	2766.277	4840.352	2827.557	3973.056	5740.247
UGDH	5004.332	3965.929	4153.727	3862.909	3292.417	4334.595	6526.075	3065.408	4216.233
GDPD1	336.3402	250.063	89.36076	192.2355	91.42767	124.0593	72.03683	102.1359	390.094
REEP4	1780.086	440.7306	459.6322	539.9194	600.2849	1072.375	595.8666	1328.774	48.88484
OLFM3	36.73347	37.74536	42.42228	34.24923	35.19424	45.86523	34.25357	40.54833	61.10219
CLDN20	368.1372	234.4398	127.8393	242.4824	345.1901	180.5755	126.8065	136.3987	368.9296
ESRP2	868.8607	39.23342	229.5201	51.52423	226.9323	48.4468	63.07141	57.00028	1025.542
BBC3	818.9957	348.5739	199.5295	306.2441	159.8906	233.5653	262.9865	189.7869	651.0204
PEX7	41.89795	1643.624	1678.52	1157.965	1233.495	1736.121	1373.154	1910.595	56.70485
SLC16A6	181.3216	65.10042	46.07043	62.4523	40.29889	66.08134	35.0654	47.24293	46.58014
PEF1	117.3036	67.6235	813.0901	144.9393	1348.268	83.47906	926.952	1446.116	198.8363
HERC5	4317.238	8758.163	10730.64	9554.884	2337.298	11307.28	13033.43	4857.562	3851.588
TARBP2	3916.206	5533.742	6040.38	4242.428	4999.281	3380.352	6667.944	7711.851	4996.453
OPA1	1433.408	935.1719	315.964	1442.134	492.0384	734.5083	698.2784	599.6579	1528.395
SGK3	44.63094	34.86525	39.76376	32.10421	33.07296	38.34612	36.72066	36.91364	34.14332
S100A8	5110.503	5914.709	11189.6	5816.751	10318.28	7826.182	9412.634	12215.56	5871.668
VDAC3	9462.234	9578.747	14866.97	12508.21	17710.21	14356.64	18265.06	14084.01	6657.645
KIAA1530	78.3205	394.9523	421.5571	62.03408	129.8432	234.0078	113.0709	69.67023	1117.595
LOC653994	112.1818	472.0522	119.6696	331.8395	67.37553	95.97398	141.989	96.77076	216.2811
AANAT	44.1148	46.40785	35.37021	39.60513	42.34446	36.96342	35.32248	40.23466	128.4341
C11orf42	55.36079	45.62692	63.80336	63.32634	40.08049	41.96308	36.53659	43.98409	59.06544
ODAM	77.50348	48.33261	44.27507	57.41845	42.4847	50.90655	49.45986	45.47554	39.78033
CHRNA4	113.5278	120.7583	67.57993	57.33975	50.40255	60.42788	56.59757	78.66369	138.7223
PBOV1	35.39447	36.16228	36.7989	38.8507	38.48575	54.48935	33.5279	37.98324	51.83207
SPRYD4	1380.477	4789.669	6061.369	4915.24	5265.54	6034.514	6545.938	5993.989	1819.93
OR7C1	39.66689	33.57553	37.7503	43.94941	37.19967	33.04474	33.64442	36.31534	32.58133
ABCA7	35.80049	47.07501	119.8267	62.68033	39.03923	33.35816	65.77162	34.85727	42.79187
GAL3ST1	53.17204	62.00914	43.61866	34.19958	147.1397	37.94231	42.9259	41.64635	43.54413
PILRA	3213.228	79.93454	892.2866	95.1301	571.0228	579.3215	562.0144	645.2676	45.82843
GPR78	126.1298	80.6638	399.7283	158.1001	68.48263	236.5963	150.1607	311.8212	294.7262
DCDC5	34.28948	42.10673	32.6882	43.80746	38.38497	39.22431	109.4443	40.56948	92.65005
LOC347487	111.0549	47.30243	43.95212	44.91933	38.51538	61.75093	49.63275	49.86931	46.56409
ZNF828	185.39	617.7859	2841.027	1193.016	4023.828	2995.203	4068.761	2274.782	67.53403
RNF216	1052.066	556.801	845.5707	959.8195	520.6374	442.5742	718.8088	585.6593	44.7254
ACSBG2	151.7095	76.6221	163.1234	127.5907	408.3601	101.8826	171.2344	229.2467	1310.317
RFP	6332.848	6071.928	6469.112	5777.462	5554.999	6754.742	5772.798	7313.719	6468.257
MPZL2	3134.088	1635.399	1198.494	1217.669	860.7589	890.4495	759.7105	1066.282	3416.984
BRD1	493.9113	316.8014	240.2451	274.8358	248.1006	125.8467	158.8733	163.2445	203.5567
ACSL6	42.01064	59.35765	45.91742	40.59938	41.93975	49.46476	42.52956	39.65619	49.61446
MFSD9	69.9658	63.6755	46.46702	90.00374	37.86077	55.42428	37.62983	56.04686	46.36814
BOK	149.3713	140.0594	255.3283	130.6442	183.74	106.4345	276.4731	113.0233	226.1479
IGSF21	1873.369	42.72663	449.4608	43.6995	525.4831	511.2363	877.5962	309.4545	39.56304
C10orf141	299.865	37.1371	133.0282	71.80658	83.11213	207.435	65.53721	116.2781	51.03319
C7orf26	94.42163	267.123	835.2427	521.8809	1562.236	335.0723	1176.928	897.5521	47.36322
GSTT1	58.68077	434.2104	41.79721	395.0555	827.322	302.2085	415.8257	470.1734	48.93251
RPUSD2	8329.496	10368.98	10247.21	11699.77	8800.97	12705.49	10291.75	11487.84	9601.338

RPUSD4	16809.84	15931.96	13669.98	15029.79	12291.19	14761.2	11757.98	15919.93	16232.05
COMT	792.1265	1444.281	1622.864	1385.444	2511.21	1969.093	2611.994	2045.058	233.3258
LCORL	1782.055	92.35568	479.3969	48.48299	167.2808	274.6167	234.6492	125.6313	68.64917
GSPT2	362.5366	193.46	251.3676	219.8442	119.0168	141.5055	208.6651	250.9095	484.3122
TMC5	125.5457	97.32938	182.0809	98.45657	578.3743	86.24335	193.4446	149.2628	103.6213
ZNF19	56.43507	58.60282	33.01144	35.9357	40.28867	49.14277	36.16809	58.59332	37.7771
MAGEF1	38.89652	50.48223	149.0295	48.48092	119.3477	70.08775	51.87274	42.88679	1227.218
OR5A1	132.6051	111.855	54.9028	99.45881	56.94111	56.54873	52.01302	68.5858	184.6398
RNF39	1946.615	5442.909	7556.282	7774.128	9347.584	6573.357	7121.512	5505.808	38.81096
CGNL1	4669.854	3700.277	7029.533	1422.872	7750.281	4731.431	3686.273	3222.036	1835.851
GNG13	59.33755	37.33453	32.81341	37.5758	35.65548	45.67545	43.5	56.38373	40.4297
N6AMT2	2789.707	1737.025	1750.542	1805.477	1543.213	2359.442	1339.896	1527.608	1805.631
DSC1	39.03724	322.9505	138.6754	40.92582	1760.678	824.5818	442.5598	420.714	46.89179
INO80C	147.9059	266.103	138.1451	179.8017	130.9572	132.9853	131.6362	134.7718	159.1019
SIGLEC8	67.62086	313.0449	53.91003	86.1366	63.24738	450.6495	63.88512	59.30807	64.31501
MED16	42.50435	70.09955	44.38863	46.93845	43.8379	49.15427	111.0524	45.82985	76.6423
CRSP6	45.46375	42.75132	40.2312	44.1545	32.76198	42.99417	34.94822	34.47105	44.21196
CASP7	33.07783	36.3508	233.0372	31.3539	32.928	52.00826	33.74864	31.24572	33.47257
CCDC115	285.5842	209.7707	109.3739	802.895	501.6002	150.257	87.41489	130.728	183.4316
FLJ35767	288.0154	350.3185	143.8639	165.1413	91.38337	99.73847	136.9076	84.1161	316.0005
FGFR4	114.7755	75.15046	41.77953	76.84142	63.56783	120.2626	53.65294	53.52253	230.436
MRPS18A	2697.637	3148.633	2279.749	2241.379	1532.591	2195.665	1796.643	2696.467	3158.917
CYCS	148.6545	91.2853	61.39921	110.4509	240.8641	72.93687	121.8404	55.7907	86.6783
IGSF8	293.2796	184.3359	118.0467	265.7654	147.0142	223.5253	155.4579	119.0144	133.4525
CD2AP	142.0573	182.6887	166.2051	193.1089	68.84895	233.0677	111.3341	85.10762	299.5546
OR52K2	49.25312	56.30963	37.31441	63.7527	41.37081	63.07634	45.72923	44.92806	163.5488
DEFB108B	218.7742	73.67664	61.8088	96.08565	63.54328	87.71675	67.0399	73.00497	203.3762
FOLR3	2700.248	525.3205	3078.753	5485.398	425.8877	1584.852	6872.732	1060.901	3387.475
DNAL1	639.9554	414.807	561.3143	583.8451	312.3232	754.5363	542.2781	518.76	96.45849
TAS2R43	147.4866	114.9306	69.85461	115.9999	79.32788	96.87514	60.28158	70.0976	256.912
FMNL2	630.2617	39.45911	114.847	369.8503	124.6085	48.37322	66.3206	76.60087	731.937
PROP1	343.0807	197.999	112.3197	243.2498	109.8181	134.8364	89.70921	125.9586	547.7032
TRIM39	59.24946	37.67733	264.3063	40.81683	170.3314	49.06721	93.49443	46.5815	76.01173
AGPAT4	56.9436	83.65146	1399.552	1226.715	3034.26	1614.78	2213.655	2457.956	81.16883
TMEM133	58.06654	45.78095	45.56572	57.95332	46.20006	52.56408	51.29992	37.33736	140.3147
HLA-DPB2	53.50828	67.49369	41.186	47.02702	53.72314	52.47795	54.3395	32.61575	42.17421
FAM195A	122.4456	1791.412	179.2739	164.5809	227.2069	122.7856	79.48087	514.574	2035.539
NPHP3	46.17721	50.73962	222.58	68.30384	57.33743	55.64673	175.0195	43.92104	51.49892
GNAO1	35.5341	44.46759	32.65934	50.48712	34.821	42.59837	102.9606	49.40106	58.3184
OTUD6A	147.9393	103.4526	73.27441	117.8347	70.08621	106.8159	61.50605	99.87546	232.548
ZDHHC7	317.0788	807.0631	1932.577	2350.787	2616.146	2460.447	2635.644	2339.357	359.5783
GCLC	509.613	184.0992	135.3231	302.674	197.1007	200.5983	173.9471	167.3848	305.89
FAM194A	67.15015	51.85552	36.5035	55.64109	52.09491	57.47386	40.70505	49.61926	119.5742
ARRB1	816.057	183.8874	618.9169	186.5134	411.8734	297.6612	330.5251	1072.994	216.2122
EVL	26366.48	27618.79	29830.98	25735.78	22508.03	27471.32	25011.66	27343.97	28313.51
AHSG	218.9081	171.0074	98.42999	178.4679	131.4499	143.6795	77.63479	112.3483	570.9403
RNASE2	40.32378	98.28693	72.03314	97.7417	53.11257	54.96487	130.9434	60.90578	96.90589

CXorf56	55.86551	1470.293	729.4162	619.6645	613.2626	849.3638	988.5029	1719.968	1712.254
C2orf44	234.5877	1865.272	1192.769	1527.863	1621.553	384.8014	1712.721	1579.883	1747.302
SLC22A15	71.93107	336.9341	564.2049	52.16863	386.1073	299.0525	491.0937	475.6579	99.38582
TMEFF2	56.83072	42.02543	55.23942	63.36436	1915.947	45.79561	161.6067	46.02462	69.08061
YTHDF2	78.77608	62.27129	70.99469	61.39584	137.0404	62.63249	282.2411	225.4722	187.9679
GALIG	74.5955	59.65123	360.1001	71.49114	56.67851	82.56137	188.417	305.6698	1675.158
FSTL3	151.493	501.2034	168.903	139.0257	362.8477	80.34093	485.8661	61.6046	264.9151
MRPL16	821.6296	613.7716	363.8514	374.2887	518.0974	782.1406	606.94	697.9265	570.1425
ZNF167	59.8173	68.61861	38.86732	50.38676	56.78121	45.31076	52.50836	45.45689	47.70808
BSG	195.6212	1321.077	79.23825	124.9973	67.54984	117.9182	57.24508	104.4962	302.4103
ADORA3	47.05328	35.07727	43.38332	37.83073	35.0042	35.75314	48.74216	58.63376	42.05233
OR2B2	326.3095	187.6785	93.29587	191.826	83.30843	148.1221	81.2357	109.6214	177.1221
C1orf75	67.95565	1094.936	318.4939	37.84554	43.52899	35.25633	38.79	398.4998	135.0052
ERLIN1	81.61835	48.78393	120.8734	252.9685	202.2112	154.4226	221.5354	139.5309	189.2847
TMEM205	12844.62	16671.59	16760.27	16014.02	16078.17	17272.11	17578.7	20022.88	14424.6
VSIG8	29928.88	17059.45	28675.11	13575.31	30705.5	22562.74	17474.18	18391.25	25793.63
CCDC73	70.86182	82.44915	54.65668	70.3707	53.13798	50.27161	45.94474	46.73131	183.5354
KRT19	70.71035	45.33324	57.0313	96.7389	35.0541	344.959	135.7877	54.93909	193.9563
SERTAD4	736.0587	856.7064	1259.98	778.7045	2395.864	1637.87	1665.78	837.0566	63.43151
PHF12	1087.034	464.2146	904.135	1530.302	460.909	372.3704	904.4897	516.6979	79.48698
SLC29A2	407.5864	243.8694	422.2064	634.0883	338.7393	218.1375	671.2646	1260.685	458.8096
PLN	479.2521	263.5482	42.85368	245.4631	87.66992	168.6918	86.62416	127.3396	568.8558
C19orf30	78.76399	65.73014	50.36745	57.01435	36.09652	45.10136	46.14346	56.57425	70.94155
ZNF512	103.8704	49.07504	67.21441	1003.589	374.9477	60.92145	57.43569	50.61024	156.0437
SGOL1	51.73645	51.3747	216.7093	33.49304	43.55685	430.4766	38.40726	36.76786	53.40335
LOC257358	254.4379	122.4029	82.99426	86.28624	89.68103	50.96783	65.13288	78.23751	241.7802
KRT18	74.81438	52.77297	58.89365	44.42221	48.46245	41.84214	47.17419	46.55201	100.0161
PDE6C	279.0539	131.7952	64.65658	199.7808	53.3827	131.3996	62.56657	97.18562	339.8764
AZU1	47.29602	41.48081	35.78852	46.69723	46.99814	63.43605	40.56311	43.52483	39.06693
ZNF680	338.7957	1570.954	1107.6	1363.523	1852.047	2140.809	1880.484	1719.682	151.5874
FILIP1L	144.0398	100.0477	268.9219	461.0609	564.1296	86.53105	237.1918	91.94672	218.6413
ZFAND1	2630.254	4623.316	2788.896	4738.123	1984.101	1676.858	2589.486	1636.324	3295.194
KMO	100.1635	108.1112	64.18839	69.34524	52.2472	182.2107	90.49133	129.1659	212.0376
CDK5R1	166.7647	92.99518	902.5651	91.45792	1401.977	1045.874	1230.16	1504.313	69.73799
KIAA0652	46.22351	36.48499	48.3077	43.4129	149.6653	60.04679	100.1082	46.77522	1570.115
RUFY3	3879.219	1944.717	1197.026	1764.322	1021.2	1330.745	981.0302	553.6428	3505.104
SLC39A13	316.7654	146.6715	91.28159	165.9336	87.34933	128.2514	70.8509	100.7003	464.3762
KLHDC3	15914.9	14908.63	16648.11	15787.84	16358.73	18692.54	14882.93	15227.18	13073.25
C19orf4	79.69386	39.90151	43.06318	49.54678	175.9537	208.5291	147.6487	132.7231	41.27891
CLEC18C	283.8256	194.9235	105.7754	210.8138	97.03448	146.0778	85.23902	134.0463	215.7309
OR4C13	125.4622	128.2789	90.85872	168.8303	69.62426	117.013	70.17102	86.25655	267.9873
MALT1	41.83949	310.9688	1063.991	351.127	1124.445	1481.926	1070.547	1194.133	40.45124
AFAP1L2	119.9239	396.761	221.0157	1192.467	1334.818	1256.294	1230.616	1200.975	41.49476
TSPAN6	41.03311	40.04839	127.8018	298.4162	73.83302	165.8579	95.69586	72.70569	45.72575
TPR	40.65456	1505.597	1310.521	99.52428	2833.599	1551.765	2115.892	1436.222	73.49297
ERICH1	776.6205	139.5907	143.1444	178.8424	250.1478	71.31533	117.4911	81.14204	304.2565
HEMK1	272.4905	970.6495	885.1616	579.6598	1456.423	1935.191	1639.258	509.8283	152.8146

DYX1C1	80.23029	49.83125	45.11782	38.19746	41.35796	60.42236	69.77671	61.38943	58.19395
TRA2A	68.05289	931.9757	749.1712	1421.532	309.9384	292.8347	388.2715	335.2699	1048.504
KCTD14	80.71465	40.21076	46.74288	54.92369	51.95217	39.21513	118.7209	37.25787	82.91786
TLR1	628.3146	295.5933	170.0634	268.1863	97.69807	247.0357	116.2626	134.9102	625.5122
MGAT5	31.7539	33.33552	32.751	31.02962	42.24846	37.82507	33.3384	32.01572	32.41988
NPY	57.34708	47.14297	45.04275	74.08822	39.99297	65.0745	49.2342	60.00949	52.55197
CABC1	44.61614	30.49726	178.1447	38.50462	508.0801	56.92712	813.8615	916.3295	35.18534
ORC6L	838.2554	977.4155	1339.138	2158.065	574.7983	1177.276	924.2119	1852.876	1108.333
ISL2	56.57813	61.46544	66.20531	127.2964	57.9797	77.92243	65.69463	58.98473	96.07966
USH2A	64.03284	70.892	66.80085	57.19589	43.90054	62.45031	42.10058	57.03144	94.85199
CCDC92	8438.145	6635.482	7361.517	8184.072	5823.323	9280.857	9204.357	6733.448	6880.909
ZBTB43	5163.109	4814.777	2939.328	3427.229	1818.677	3095.287	1661.713	2598.727	5582.229
RAB11B	70.01172	930.1649	583.8854	982.3212	820.051	967.7219	743.7269	304.752	2927.716
TXNDC9	614.502	1093.171	2241.023	1664.36	2309.654	2171.658	2409.692	1756.009	1311.809
MYOZ1	10860.8	18072.26	12826.22	9124.446	11104.03	15072.86	15407.54	20345.37	12028.63
SLC16A8	61.83568	41.3257	50.16034	38.47811	254.1505	49.43696	95.22665	79.44965	40.18888
ARHGAP28	48.7956	51.20213	49.09383	52.44154	53.55245	65.4839	99.35324	47.362	42.75129
EXOC2	142.4949	90.63766	71.42361	118.4044	60.65001	61.12469	61.40364	69.74409	464.5807
TEX2	39.872	32.99547	2456.916	40.13568	4162.141	2790.433	4024.015	3684.443	46.18851
MELK	100.0652	68.94185	65.4802	76.77823	50.09335	50.63195	57.55676	83.74964	115.8762
BCL2L12	1777.436	46.95562	83.74064	46.31351	72.83486	68.70123	55.74388	57.75637	44.02692
STRAP	1001.932	785.782	1024.685	580.6193	1422.018	478.2718	941.929	1230.386	202.9863
NKD2	17963.63	15964.84	16842.83	11474.93	20527.22	24231.14	15632.22	17598.05	19492.37
TECTB	89.0844	90.84851	51.63647	53.07745	51.96942	58.74363	36.97277	69.65565	62.31447
MYBPHL	1466.955	794.5541	2193.819	3044.064	953.5739	2414.919	3347.219	474.726	43.35631
PARP9	966.6081	148.2262	917.2192	82.97391	218.1885	942.5975	1291.914	1215.728	295.4731
GZMH	42.8566	1671.55	1935.792	40.75877	1146.675	1620.907	735.9874	1435.026	37.5838
CLSTN2	324.6125	176.0166	96.49584	161.3138	76.255	557.6352	216.2594	82.10269	301.7736
FGF7	38.81371	58.57277	35.52519	32.64837	150.1808	57.24253	33.96479	40.97412	43.52848
DPEP3	558.692	2495.524	2286.073	2622.092	937.6057	2548.188	1641.589	916.8958	94.56748
ARFGAP3	51.12684	46.56395	173.3329	510.8976	36.36453	36.24517	36.76534	43.65918	35.68763
CA5A	55.10641	34.83845	38.3709	36.91346	37.29041	39.13264	36.85134	34.39604	33.13034
C8B	114.1037	36.93845	37.04055	37.32471	34.73259	45.55864	39.44838	45.8932	64.08881
GPR179	61.31957	78.24622	41.89706	44.58075	58.29737	327.3965	40.69112	57.20896	103.5622
PML	41.86913	65.23781	46.94579	53.67161	41.72683	72.62589	45.58694	67.22079	76.98028
OR10H3	47.6324	33.04918	48.92187	36.73675	33.34461	43.44648	36.25609	50.31092	40.39402
FLJ46210	130.4867	133.553	53.70823	84.46355	67.56807	91.35535	63.60494	89.54482	55.93917
LDLRAD1	98.03545	69.56612	56.12909	89.50572	47.43454	46.52665	51.40414	39.55851	253.3142
HOXA13	42.63477	38.55893	34.96447	34.93324	33.05034	36.34307	44.19558	39.87909	44.78531
CHPF	173.1423	569.2281	1433.613	326.8337	2033.037	773.9845	3222.914	1261.067	222.1191
PARL	1360.623	2260.624	3872.745	52.52214	4815.883	3433.578	4631.437	5832.517	50.1965
ZNF532	1399.931	2900.049	4171.249	3849.692	6694.174	9180.167	7287.996	4917.588	3818.398
CPE	1259.469	471.5274	752.4532	588.8865	3077.511	2165.297	2209.573	2104.131	172.8382
STMN4	142.9714	57.34163	58.67814	118.1568	65.64989	51.64379	53.76376	51.08969	166.1183
UBTD2	58.53393	91.56246	476.4843	1180.078	749.4728	366.9118	456.5453	635.1266	37.83694
ABP1	1913.323	1519.13	927.4413	866.1033	1542.941	3961.126	704.2251	1164.64	4372.072
COL11A1	646.134	128.3072	69.48602	395.4218	90.13515	258.6815	80.7021	83.59901	247.6619

ARNTL	195.658	131.536	432.7858	855.7379	439.2239	340.3335	349.0395	624.3204	995.2689
GPR156	377.4913	135.6687	90.36642	177.1551	101.7325	139.4815	74.31454	99.73476	420.3978
MARCO	970.0198	395.1436	74.49673	83.10992	97.25329	123.0004	202.2955	148.674	420.7839
PAX9	196.9868	171.1619	990.5084	356.3666	810.8403	786.2304	276.2111	90.83254	411.5676
A2BP1	35.80017	45.84337	127.8469	34.83259	34.38728	42.92334	40.12647	125.2586	36.98094
GJA3	52.89027	43.02241	35.40407	38.74297	39.87284	34.49942	33.93605	36.45692	43.15031
PLD6	89.01849	82.60763	59.88829	90.05239	42.08806	48.80025	44.67528	58.29357	41.54727
GJB3	53.26216	33.24571	54.62819	880.3043	39.78773	455.1559	53.32246	55.82457	125.4672
MS4A12	62.76368	39.06525	41.46079	53.47794	56.65059	40.23524	41.27746	43.11216	43.76678
RPL26	597.2748	728.1842	845.2153	1645.39	618.3766	662.6881	730.9889	332.9739	1092.332
GIF	107.6885	64.80039	66.88399	85.17095	66.09253	74.3945	50.38015	59.14307	89.54813
ROBO3	298.1917	348.3431	281.9514	161.8329	670.3447	336.2514	438.5345	326.8613	438.8384
RAB3D	66.96817	825.7365	1181.729	1602.809	756.7675	211.2209	547.7682	881.6305	73.90118
PTPRJ	63.68233	78.81503	118.9727	72.38463	69.21326	59.61197	92.4212	68.58146	130.3279
AP3S2	48.41776	2574.093	967.9995	1242.292	267.0208	624.7615	1023.767	592.9359	1492.589
DEFB118	170.3864	177.7293	53.8503	88.90055	44.52087	50.21503	48.60151	55.75323	151.2132
UCHL5	3705.54	2457.133	5447.566	6187.113	5612.737	5169.771	5743.473	3747.012	2618.574
DCLK1	59.2957	38.28539	39.20966	701.0042	53.43095	54.7153	48.41269	64.11327	78.02068
DAD1	2042.055	956.3068	2116.188	2371.733	3370.199	1800.389	2577.057	2422.814	48.10728
RXFP3	57.54876	56.51182	68.89882	58.60329	60.22681	72.44537	49.89329	41.80984	67.10105
CASP3	98.55046	96.53841	63.43049	116.9464	183.5808	83.71619	53.9351	64.84865	63.55371
MGC39900	36.18683	34.24434	43.74081	37.98737	57.97498	56.31023	126.2435	52.50279	38.05887
SETDB2	8506.256	8250.256	6648.29	6568.675	5564.67	8303.558	5613.854	4209.268	8379.797
FOXJ2	4787.482	5801.249	7065.807	5781.989	7204.723	7334.655	7486.268	8284.938	4330.627
ZNF641	432.3071	191.0012	142.2746	105.4179	155.7688	184.0475	136.2083	86.86649	80.94227
KLB	272.7509	103.0901	80.0746	159.5153	62.22101	126.3699	71.43666	76.31407	113.6333
RAB15	1668.997	197.8236	69.30694	61.70509	239.9045	250.9525	97.16314	92.99878	41.62374
OR10G8	31.57883	38.51758	37.17595	34.84993	38.48137	36.30815	31.34509	68.42449	33.0081
POLR3H	207.7215	405.4052	184.5313	111.6688	68.33201	121.6523	70.18566	181.6794	2110.995
PDRG1	411.3846	219.8476	379.6313	274.8586	446.9761	197.2639	553.1228	334.0891	461.4642
RIN3	1710.593	36.75877	44.11416	41.78257	103.2066	254.3285	47.99355	251.8539	65.71009
TNFRSF13C	57.17388	80.6429	819.4179	91.60596	349.0393	394.6265	558.9429	423.0217	40.63208
NINJ2	8443.464	8863.274	7214.535	10027.36	6284.335	10746.68	9796.365	7465.297	5915.328
NTRK1	60.40263	53.19473	464.9646	92.7582	338.6616	418.1364	428.1213	158.8153	99.97177
ATP1A1	3160.905	2931.503	1548.533	3406.312	1302.982	2110.962	1300.601	1823.987	2022.919
ORM2	43.04742	32.23043	39.9242	32.757	40.02568	38.89104	34.93325	61.26364	44.32526
LOC606724	71.28597	61.11712	89.97627	54.09174	36.45359	61.54219	50.10674	48.27814	61.82256
OXA1L	1905.232	997.8377	1794.408	1846.58	1983.697	1342.84	1488.667	1493.166	1585.5
GDPD3	53.11036	66.21131	42.89353	80.96766	59.32637	99.03117	45.97601	48.28643	110.2471
UBXN8	1018.061	796.6522	987.0045	301.1927	791.6684	713.3901	657.4724	839.0458	34.98451
FLJ90231	44.76065	39.46339	38.48569	38.08568	36.13046	49.46728	34.80484	46.28148	41.6866
SCN4A	96.42506	62.86483	49.538	59.76928	54.22357	111.1707	819.3226	1038.28	175.946
LY9	61.66582	749.6115	381.9196	42.23012	157.8593	34.99082	118.8106	54.21048	42.76889
APOM	54.59344	85.53947	46.54567	51.4719	187.5388	82.3857	171.3804	240.4546	139.2773
PCK1	60.84814	35.3324	32.87516	48.47429	55.98951	42.11588	40.92884	40.91645	145.7212
SYT10	49.36155	35.66067	35.80054	36.50078	38.87867	34.81855	34.27792	53.34611	33.0163
HMGN3	1616.429	1703.898	1184.231	1634.333	775.8721	677.0986	867.8319	945.5905	1812.468

SCYL1	54.47424	816.9252	389.5427	67.41766	784.4926	83.52051	1307.367	966.7556	31.2612
PRDM7	34.08678	35.97755	34.41475	60.49466	37.01443	63.83215	85.87825	37.36442	41.60156
OR2A5	135.6378	49.73262	49.46073	49.19995	45.86183	60.96656	43.98322	36.84472	57.33784
HPSE2	55.1172	44.53009	40.59368	35.00303	46.05337	47.04725	34.3961	47.31891	45.72061
DDX3Y	67.51532	54.20452	50.3276	73.14654	36.40861	64.96602	53.62928	73.49915	151.0625
DPM2	138.0656	183.0548	89.21046	211.4422	75.86698	588.2751	104.6115	199.0565	174.387
SNRNP70	23638.94	26117.15	24345.47	23658.62	25326.76	22665.99	22639.77	22611.23	24983.56
ARHGAP1	630.3108	2659.574	5657.374	3413.712	9136.314	4886.472	12601.15	5076.236	124.189
CKMT1A	63.95729	66.35876	136.9435	89.39417	80.1208	102.0707	94.60896	61.20295	64.83437
HIST2H2BE	436.8392	275.743	117.6908	239.8867	159.0537	168.8616	114.1492	112.9861	109.8222
MRI1	9258.194	11707.99	12900.31	9854.96	13545.43	14005.83	9811.785	9238.469	8885.022
RBKS	1220.98	980.73	752.5334	505.0219	454.532	787.2208	570.301	490.8228	871.4188
PPP1R8	34.07977	34.79457	381.2745	34.35182	286.5655	39.47572	38.33652	44.50736	56.62924
FLJ42957	5581.142	7121.48	7732.94	5335.495	7879.628	5976.79	4413.258	4092.994	3117.045
AQP2	59.8053	73.12602	57.61367	70.1964	125.3634	48.10756	82.50151	55.89182	46.21907
KIAA0101	205.9545	485.3597	358.8783	2122.68	151.5908	322.7565	133.9372	218.8392	66.13868
COL6A1	2541.342	1629.052	1468.002	1860.036	3538.877	2439.042	3307.444	2414.136	1835.448
FMO2	4422.705	3240.084	5592.214	197.3414	2427.021	4001.95	2294.343	2997.654	2671.939
SDHAP3	42.47576	36.86991	58.28228	39.95473	41.3219	69.94841	39.62891	66.93253	34.29612
TMEM143	275.5435	105.8301	895.6592	108.5653	996.5851	127.1198	1126.184	1259.504	2574.122
SYCE1	75.04311	71.31293	45.62937	50.19699	33.87815	36.6495	37.04434	57.725	35.00261
MN1	185.4121	173.9931	70.15435	150.0065	74.04178	160.0714	74.904	94.95816	217.9394
NARS	4932.508	4763.598	6213.152	5613.369	6076.444	7427.022	5548.075	5834.521	4100.172
OR10G9	45.62396	62.68454	45.44588	49.51643	51.77083	47.33994	43.9481	47.50657	64.60145
GP6	68.20706	70.90864	329.5425	53.37609	50.56398	48.06066	75.31587	40.33504	57.64547
BTBD9	158.6083	134.6021	61.06689	88.42899	72.4045	99.61785	63.36341	102.5655	285.494
ID3	35.43295	980.3846	409.5784	42.59126	424.4415	522.2537	373.2538	645.6652	45.47944
TAS2R9	53.27798	41.78271	36.30492	45.9445	54.8101	50.54512	50.35734	36.87619	47.62339
PTK2B	1163.502	834.5562	154.3884	452.3016	195.9297	242.2352	140.5938	120.8866	1297.577
PADI1	105.8569	77.98466	247.1679	45.85107	51.94039	55.425	55.29445	51.66094	128.5525
LOC646200	4943.454	3387.99	3551.096	5342.026	3281.269	2633.256	2954.463	3334.853	3751.88
SIK3	1095.888	481.3425	3230.107	1190.379	4030.725	2881	2865.71	3469.113	47.42737
C17orf41	329.4734	162.2367	76.82417	259.9865	56.90742	79.80798	53.20904	44.4906	183.3583
EEFSEC	35.71326	352.2413	688.3647	414.0164	459.3879	502.8622	640.6838	833.0991	41.95282
SCGB2A1	53.347	40.57662	265.0643	1058.033	864.999	961.9269	316.9963	35.27987	32.95676
C2orf15	33.04809	38.78705	395.6114	33.17178	532.01	34.51693	108.022	43.75269	52.1092
SLC16A9	9196.785	10909.73	7787.99	9588.02	11508.55	6232.053	7496.392	9862.412	11106.77
CRHR1	308.3431	149.7967	75.39737	206.8347	91.3439	137.2426	95.76942	133.8297	495.4518
MAPKAPK3	12520.99	13736.25	19783.58	13244.34	20799.2	15780.69	20890.36	16215.6	12173.8
OR2AG1	68.24842	74.62041	52.60054	50.20736	63.24131	68.83456	50.04723	50.04658	37.19572
HSPA4L	1768.574	508.5009	443.027	617.53	828.7123	659.5601	354.1443	765.961	107.2556
STYX	99.57211	775.0189	1476.539	2613.506	882.1614	72.02715	835.39	1556.542	2500.273
OFCC1	94.15497	106.07	53.33792	76.55742	53.95485	73.3381	51.67411	61.30615	90.26064
LRRC40	149.5517	109.1393	81.33585	101.3081	59.94507	77.24869	95.69283	147.9076	69.95036
KNTC1	732.9443	817.6999	2113.666	1067.742	1888.876	2153.192	1985.335	1339.156	54.92373
LBP	68.34738	41.72599	41.79151	57.42756	34.78836	50.96227	35.97731	38.74801	65.95468
KLHL23	33.91911	44.86617	33.77485	38.65191	32.17057	79.06464	52.23302	107.1272	47.53788

ZNF346	38.10256	1566.396	1017.464	43.09747	1431.833	723.6216	966.8241	858.0438	49.46033
B4GALT6	37.82027	249.748	109.9783	329.9636	66.12161	38.55993	103.1035	59.43779	34.05029
TMTC3	33.54213	50.0116	76.27296	40.46785	37.58208	41.86988	46.74136	40.75002	605.3452
SEC14L1	562.121	47.26995	48.22131	50.75187	77.40813	146.5847	45.61377	91.97906	85.81397
TMEM216	1532.883	2276.134	2326.539	2134.281	2626.237	2596.444	3037.013	2525.773	1652.452
FLJ45455	90.01476	101.5298	52.65031	65.26529	53.9468	66.03712	48.74088	65.59161	103.8025
KIAA1539	65.756	1712.855	2503.461	1229.173	2687.096	2469.419	2953.904	2753.915	47.74475
SHB	243.8503	170.0864	254.2865	372.9757	250.4077	136.3909	275.5098	172.1518	315.5647
CRISP1	80.84985	48.00402	41.45099	40.1456	47.90281	41.62723	41.67296	33.56925	69.88161
KERA	32.13839	35.77687	35.92892	38.51167	49.07941	34.80923	35.93517	31.66184	33.18673
HCFC1	10211.55	10780.4	13619.37	10152.21	10470.18	11468.19	10106.06	12668.45	9445.816
FAM71E1	104.6397	72.94658	64.67279	142.3544	61.08451	83.51116	57.11646	77.04289	150.3237
LENG9	763.8615	605.0882	628.052	1050.756	261.056	325.8985	295.1199	372.399	98.20416
TRNAU1AP	89.11838	55.09425	65.64806	90.75404	150.2309	79.7221	53.5913	213.2621	90.36642
SAPS3	42.33808	891.8394	1393.45	648.5273	1337.456	661.8478	1147.881	1576.99	51.32537
C11orf66	106.3777	109.2277	63.14558	56.38924	52.38855	80.8857	59.48455	64.32359	103.617
CHCHD8	403.2898	1335.785	489.3289	1170.965	330.7461	369.6252	494.5929	575.3657	1056.598
PSMD9	38.45554	45.52865	48.29843	33.63696	149.6906	305.126	341.4906	69.91472	44.17122
PPME1	1036.822	1187.97	2000.648	2307.723	2345.877	2152.244	2370.29	2871.856	930.3616
SCN2A	735.0371	361.8444	131.7742	58.26698	146.4296	42.65177	297.2176	212.6935	2556.114
GPRC6A	80.83659	62.4974	55.46093	112.173	51.73074	68.15579	53.21207	55.95163	227.9417
ALG9	53.14613	648.9893	781.274	90.78707	924.5061	692.7534	1311.071	977.9515	2833.134
SNX17	68.11175	63.33773	1924.58	874.22	2682.394	1320.747	2583.553	1810.859	87.01175
ILK	35.0002	38.2249	41.04659	42.09746	39.87691	38.85714	38.4211	37.33619	41.64675
CHST7	1838.047	977.9458	2810.406	2329.192	5014.448	2160.958	2965.195	5931.951	37.38335
FLJ26850	390.4264	238.4597	108.2775	238.6703	110.3324	164.9223	113.8501	138.2689	426.1901
IPPK	40.51422	48.73878	41.31889	46.29248	34.52242	32.98697	36.02943	40.51022	62.311
BTBD12	885.0684	1951.791	2765.03	2374.119	2619.935	3262.859	3326.021	3358.203	1168.623
OR5H1	33.94772	35.95303	46.09476	48.06767	38.16593	60.16985	35.76902	55.9899	36.82862
LRP6	517.6277	293.3341	176.5685	308.9666	241.0478	282.8162	162.812	250.9507	724.6462
LILRB5	267.6097	228.624	110.1069	177.4691	125.1848	144.6908	162.3914	157.8047	705.8237
ITGB8	127.6835	89.00898	332.6186	60.43277	137.861	285.4229	253.9616	125.5117	65.58865
SIPA1L2	8470.335	10044.11	10347.21	8853.068	14404.28	10776.91	10707.71	9141.142	4431.106
FGF14	39.78773	39.53672	39.90956	34.97247	43.58281	38.2687	38.8061	52.63772	175.7214
ZBTB32	685.5581	2073.671	1568.432	150.0418	339.9696	2226.521	3212.561	1123.269	229.5375
BCAS1	3189.835	1011.288	1260.081	644.5385	538.4562	3204.941	1336.807	645.3943	46.16195
RAB36	53.33388	43.43229	34.1476	42.22231	407.7691	32.85291	294.3326	307.4002	34.60138
ANAPC2	41.73971	37.57038	35.87177	41.25261	176.7019	33.49744	43.48169	40.21727	46.5929
LOC28590ε	85.30366	99.37452	373.1171	68.0073	1347.092	1232.125	1075.935	462.7457	200.7453
RASSF7	11944.55	11925.68	11964.91	14245.36	10005.84	11207.61	7226.001	9760.64	13697.01
RALGAPB	251.4686	1963.541	1440.683	733.6902	851.7968	931.9393	488.9211	647.3092	3498.677
TFAP2A	243.0899	116.346	90.5519	192.9267	83.45543	112.1218	188.539	97.95626	226.625
IFNA16	97.16431	56.02291	39.4276	68.68322	35.21361	36.91019	37.74449	52.90848	80.75877
GIT1	341.9389	197.4361	250.2379	218.7418	1058.508	114.8112	995.4395	605.653	451.5668
C9orf164	2103.973	1333.587	545.511	82.27317	915.8399	525.2347	36.9506	275.4889	53.88613
NRBP2	5235.396	6463.738	4184.805	6648.103	5038.954	7571.887	3986.996	6053.294	5372.823
GNB4	55.56289	63.68744	47.43593	282.4903	94.7908	57.09003	49.7853	46.11897	67.05016

WDR93	36.78465	34.88924	43.91169	39.70092	36.33356	43.5528	35.08832	41.5621	45.16003
PSMD8	818.0602	1310.389	1031.568	1539.218	1130.872	1322.034	1174.198	1434.093	969.4029
CYSLTR1	776.9962	439.2081	447.1272	301.6323	187.3844	383.8281	217.2999	160.8989	735.0106
C1RL	650.9543	464.9327	259.0609	412.2628	79.37688	269.4777	271.2047	228.0864	711.0301
ARS2	155.6015	103.1934	62.08707	100.4448	44.75353	61.84316	49.77276	38.89092	163.4869
C1orf158	52.50291	73.97227	45.2396	65.70097	58.30984	69.37073	76.66983	60.33788	73.01827
EIF2A	1884.821	4303.791	2555.711	2538.616	1866.075	2144.239	2025.929	3179.659	175.1311
PIGB	33.00792	49.5676	107.4836	50.69563	90.44108	46.39952	84.3484	45.46009	43.63773
TEX9	166.4776	48.51888	229.6149	57.22806	117.6806	266.6827	263.8327	275.537	42.28703
ITGB3	148.66	83.96661	206.8096	892.3127	587.2667	400.9958	464.8342	354.4516	113.1971
CALML3	7810.995	9950.328	17402.31	8551.668	19802.78	8039.282	14618.82	18716.79	3685.765
SH3BGR	248.9036	116.3783	76.10105	176.8786	71.28825	85.64629	73.16687	94.55392	348.681
PREI3	95.4932	339.4937	141.4253	84.90878	313.2799	101.8061	242.8959	145.4538	927.9172
QRSL1	102.7124	105.8546	62.69171	57.29718	77.83827	231.1431	56.43924	47.56337	95.06063
MAST1	31.93881	39.38792	37.14645	42.22456	41.03946	43.18114	46.88558	39.3623	33.99391
FASTKD5	202.7511	154.6064	73.96116	198.2831	249.039	109.0234	86.4943	160.8148	139.9859
CSF1	39.63121	833.0587	45.20937	70.19915	50.95147	57.12737	56.35605	50.05177	63.14043
QTRTD1	126.3871	166.498	98.00576	186.0054	83.72621	148.4389	69.51853	73.62494	353.2054
MFAP5	10549.48	7274.357	2674.375	15061.43	7095.039	5459.732	10179.59	6805.469	7878.59
CCNE1	43.71603	49.99117	38.59009	70.86113	43.35686	35.43717	37.42181	38.79578	66.73116
PRKCQ	3406.343	3553.481	2268.126	2764.704	608.0398	1839.752	1139.577	2065.91	3633.96
RNASE11	169.8703	160.853	95.76547	130.6344	97.55358	126.8585	72.74344	52.5472	294.6964
SULT1B1	48.45963	48.07629	46.43626	46.70351	50.62487	51.42916	39.10314	41.80523	34.49672
HSD3B2	85.56116	52.59369	53.19805	97.23313	61.80526	103.0243	50.18591	49.91814	311.1348
GOLGA3	4370.989	3830.569	5175.321	4113.583	6724.821	4893.06	6266.063	5810.938	3230.62
LOXL3	134.7426	150.6125	76.87358	126.3866	62.57167	74.42002	65.62213	65.70041	108.5474
HMGB3	196.6349	126.227	53.25616	140.9572	70.75225	112.7874	174.4177	74.33342	216.6496
C20orf134	58.12613	37.40024	214.1974	49.34375	38.79974	54.25265	109.2533	35.60495	61.92016
ZNF366	223.0951	146.6419	116.0274	175.6308	126.7016	97.89961	68.36559	186.0014	352.5902
GPR123	65.60317	41.78615	31.9805	32.82164	35.2168	39.91164	30.09226	44.22	37.23559
UCP3	78.55923	413.4479	61.41284	543.6744	55.70906	69.7785	165.6589	252.0941	162.1026
LOC10000{	27426.25	24453.9	27446.92	26672.45	26393.86	27796.55	20171.91	23464.79	29403.84
TFB1M	67.11048	67.88038	1228.955	358.7787	1583.156	653.3339	1302.993	1253.884	41.82076
GPLD1	33.54979	33.44972	42.9043	33.41905	36.64139	34.63303	34.46869	44.76193	44.46652
ART5	35.78339	46.56356	37.88979	39.23153	36.92533	34.57407	43.54383	34.92439	38.57155
AKT3	44.34227	47.35355	116.3643	47.88503	46.94278	40.52124	40.42449	60.34566	38.92596
NDUFC1	1788.47	34.70716	474.0305	60.95932	2389.612	460.3092	1261.147	1252.231	36.19715
MTF2	4516.359	4609.012	3360.151	6324.884	1463.022	2705.341	2389.439	1950.986	4815.866
TFEB	45.92506	36.82941	46.86371	38.68434	41.13786	35.744	34.47095	151.737	36.71615
NEDD4L	337.8182	201.1247	167.546	387.3978	187.7694	328.2933	99.7223	223.2607	477.622
SNAP91	978.0687	842.3738	233.8748	315.5674	219.5018	284.8057	218.605	218.8092	2194.049
CXorf40B	111.7707	64.52271	126.855	90.09456	61.94149	63.11671	43.009	74.20312	61.39024
PLDN	1419.357	2155.167	778.1583	125.611	259.6947	746.861	286.3253	66.16385	94.62814
DHX35	117.3434	107.9115	58.76789	91.41024	65.31512	80.69476	103.6408	54.56663	98.88905
CDH18	54.60488	56.02685	162.1631	36.64357	37.18813	34.2522	40.33587	40.30225	51.03105
MPP1	238.7787	152.5851	251.8671	831.4504	372.8423	269.3763	440.1775	336.37	346.3276
AZI2	930.9422	340.5199	500.1853	150.0171	506.2595	302.3323	362.9721	131.1028	209.7989

HIST1H1T	49.96193	66.85305	62.74593	51.71152	64.11599	68.74991	42.226	47.18491	50.47001
IMP3	576.3975	573.5197	3822.293	1639.879	4097.364	3208.587	3692.689	4422.381	513.9088
TRMU	1982.883	53.87709	285.0291	39.01926	59.46661	59.26379	106.5052	67.1448	65.07966
ANKRD45	134.2676	49.51353	216.3488	63.09647	45.1	50.30954	51.11853	67.47343	144.1958
PAGE3	187.3338	94.19724	92.65753	133.4934	95.38979	154.0439	72.73669	81.30973	142.8239
REEP1	42.50829	46.12513	208.4729	37.65096	1359.258	480.8924	417.8417	168.8505	59.26274
COL12A1	353.059	68.58601	182.932	96.68983	80.29434	162.9202	169.95	62.35583	174.2829
LOC374395	35302.25	31038.49	30759.28	29055.67	25998.92	33664.89	36839.88	22587.52	30409.06
MT1B	65.5079	91.57377	59.33252	49.6716	54.9299	78.58699	49.2176	58.38265	167.6447
RNF190	69.24323	47.71675	46.25386	50.6168	40.82827	34.182	37.90818	36.30977	51.31205
FAM195B	60.76315	35.40125	39.22486	39.55461	206.3796	44.17324	187.664	484.3311	45.91151
PCAF	59.1998	51.69491	86.97563	67.82453	47.52559	51.56368	49.20341	56.81054	124.0282
IGF2BP1	136.6097	78.8107	67.45245	101.963	79.23164	91.85499	60.68546	87.04055	199.4568
JUP	2198.659	39.8572	1455.576	2348.936	969.3419	1111.374	575.3739	989.4858	3211.567
PGBD5	77.35487	833.367	47.21164	61.79464	763.2313	49.47592	278.3506	216.235	72.62001
TCF7L1	47.39706	40.30556	36.42039	331.8792	66.7858	43.87537	102.9959	56.98877	821.6796
XKR9	56.72277	91.27387	44.15567	55.31443	51.99743	53.47889	52.95848	46.1549	51.09033
GALR2	46.7677	38.41603	344.7184	50.23816	958.4799	726.8698	605.8444	1233.912	51.03479
DACT3	1540.49	1554.256	639.9169	585.277	751.9476	953.0872	556.1509	757.0701	1156.641
DCAF15	43.12767	64.1471	51.44087	662.6196	53.36259	61.09565	109.7683	225.5207	66.62671
SYNPR	39.10419	42.89116	47.02938	64.87286	48.77632	52.04346	52.31485	54.29976	46.87597
FAM100A	69.83366	70.29633	46.54274	45.23914	192.0436	36.98689	41.76657	44.82259	51.53076
SLC35F4	151.1642	130.3939	62.55787	121.6537	83.3919	99.90827	57.50361	66.8358	274.9724
OGDH	73.86291	194.0379	1734.793	265.1754	1310.791	2226.39	1153.837	2348.036	61.20858
SH2D2A	307.8705	218.7779	347.2162	211.236	167.4584	272.2468	284.2027	202.0953	302.2508
RUNDC1	898.9225	961.4597	937.2786	776.4547	828.3656	785.761	660.5323	550.5303	1787.07
C20orf114	66.53288	63.31655	44.98262	56.57523	55.03924	76.89536	46.32358	39.86752	69.9155
TCEB2	3023.243	4713.987	8741.717	7412.907	10769.82	6610.799	10947.04	13105.33	2739.754
DNMT3B	38.15163	107.349	65.79804	328.7466	55.67046	63.70887	97.29922	56.67989	35.15916
PREB	776.2901	487.0453	728.5454	543.9175	943.0426	390.1158	738.0521	819.4982	809.2424
PDCD6	2247.613	3352.391	1956.225	3847.924	2137.977	1434.229	2337.055	898.3292	3977.027
SNUPN	903.1479	452.6959	613.0031	1241.71	750.8233	287.6599	507.3229	401.1295	75.96636
MMD	68.12695	60.83572	50.79851	298.0598	81.34407	62.0196	70.11393	146.7304	110.7493
FKBP8	304.8899	175.2483	170.4358	648.4344	242.1273	229.9945	234.6458	211.6233	277.9601
IARS	47.90932	33.27438	129.8025	38.9963	119.7504	53.86793	35.4982	51.66029	39.77008
ADRA2C	227.5701	173.7171	84.45961	136.4242	74.6757	127.3684	65.91122	88.73626	251.4674
GTF3C4	1969.014	69.33266	546.9995	1099.219	44.20074	910.7961	35.32801	115.0818	37.99682
BAG1	145.4936	141.9911	65.69192	114.3318	68.49335	72.0751	54.41848	74.09907	63.95862
SIRPB1	48.62416	56.30315	53.61084	117.0715	334.1863	81.01151	148.4923	896.1111	49.12782
C10orf118	96.10326	159.784	93.4372	134.8504	67.66219	120.8349	64.88274	74.20021	428.3925
CLSTN3	115.2926	1249.281	357.3556	86.80663	289.4922	118.5083	65.00371	291.5991	237.3078
DCLRE1A	40.84648	41.84499	36.63269	35.54502	40.34283	51.96929	38.85122	36.68668	44.93855
LDLRAD2	67.25961	47.44149	43.78567	56.53266	35.59102	37.8802	43.48023	54.42088	47.87778
TATDN2	3253.288	2073.345	1135.85	1983.286	832.0037	1214.099	990.37	1068.308	2297.338
GDI1	1023.211	2513.588	2118.352	2185.429	1909.371	2871.401	2269.254	3513.358	240.4701
CGA	251.3157	147.6614	79.91769	204.3433	75.84485	69.38347	65.0211	82.53379	265.1935
P76	64.07047	651.7892	1570.531	675.6785	1408.106	736.7356	1808.951	2345.138	67.44251

TCEAL3	6154.103	6670.611	8448.68	9519.746	10857.72	9748.794	12959.07	8321.026	4778.304
RNGTT	2754.925	3368.058	4246.626	3390.476	3286.474	3774.719	3815.488	4245.214	3125.086
SPPL2A	1302.563	45.47165	670.1771	41.04577	788.6117	44.83163	737.0522	965.9563	1899.788
DDOST	3946.335	4041.928	3047.009	3043.099	1864.639	3168.661	2362.723	2726.88	4424.507
ZNF770	256.4394	81.77819	284.1057	52.63925	429.6657	323.3531	717.0052	294.9596	60.80324
DUSP18	874.5148	60.152	458.0728	91.33329	1359.783	253.2811	742.9974	745.7421	44.40199
MAPRE3	5717.016	5903.854	6397.296	5732.417	7813.254	5870.552	7386.769	6961.392	77.19319
OR52E4	46.50567	44.75783	63.86842	59.15211	35.52621	106.7368	44.44445	37.52494	58.32651
GCN1L1	7415.402	6262.02	5709.966	7187.492	4812.916	4552.958	4891.245	7521.122	8027.849
YME1L1	529.7474	355.3754	355.4092	607.4807	240.3096	348.0848	271.6462	255.2403	808.5502
DPF2	289.2192	150.4234	199.4408	179.4458	178.1536	177.0361	206.023	226.7899	279.9582
C19orf36	33.27054	36.72714	43.49783	37.17482	41.74069	47.93073	39.01468	38.46223	84.07465
TPH2	58.19056	67.1629	45.92517	67.15192	47.96342	65.6025	38.18542	37.6954	49.97454
GPX4	87.41874	228.2528	99.38928	81.3443	127.7682	76.51588	202.7104	129.8214	636.2235
PAX2	74.96904	128.0823	53.32695	135.485	55.68476	85.50436	51.38831	66.1223	237.2375
DPF3	198.732	95.86773	65.58024	130.4045	68.68199	110.6013	59.28509	300.5302	136.6381
BNIP1	67.75422	41.46523	38.62201	44.44393	149.8516	33.30734	59.64257	35.32517	42.05678
C14orf133	152.5556	70.00336	692.6772	484.4456	238.928	506.7863	661.6042	77.17957	184.722
LOC91461	162.2328	131.96	70.51588	427.0493	128.6641	111.8131	89.00956	76.80842	244.6702
PEBP4	254.0868	138.1869	76.48239	112.7111	80.1097	112.8797	63.66233	100.88	263.7535
KIF2A	111.4743	107.4413	240.2174	555.4194	68.77811	232.1287	107.1578	282.7222	140.2642
PIP5K1B	72.54095	66.52122	54.39293	65.20259	57.40036	62.18001	51.5122	65.90394	217.3949
ACCN3	358.3664	220.0017	101.673	247.074	234.9873	143.785	93.27347	107.4475	555.5284
BSDC1	3692.063	3955.765	3842.871	3205.735	4353.71	3611.797	3726.076	4382.668	5287.221
C1orf19	710.6325	1097.296	484.6606	919.783	359.121	367.3209	567.2657	339.569	698.9943
ARID1A	40.17179	41.79177	38.84134	40.19594	32.54071	45.96932	44.39668	35.74352	53.78431
OVGP1	4231.474	3523.782	1760.971	3061.616	1889.989	2698.242	1420.589	1297.647	3688.796
SLC9A3	57.86158	241.9741	115.5062	46.87796	57.44462	136.2792	40.89495	44.19136	159.3445
SORD	70.23429	41.72351	48.59603	40.32397	43.33188	38.72411	43.74044	34.26933	46.84654
TC2N	34.79797	36.35925	35.12717	31.24426	63.66197	37.67453	45.26329	33.94385	43.27688
SRRM2	13853.3	13170.35	15810.82	13285.06	17066.85	17918.19	15801.44	16057.89	13428.2
ZNF77	34.7894	35.00417	168.0577	51.30494	34.71231	37.64339	116.0347	51.09968	38.47214
BDKRB1	50.79593	62.7348	36.27016	68.34766	430.6169	59.47205	46.65163	57.41084	53.75637
ALPK3	52.24831	47.76387	34.72727	32.81684	54.71313	41.84051	41.45293	70.00784	46.09131
SPAG4L	207.0991	110.8093	75.31253	137.4151	64.00834	126.5497	71.4354	42.61939	335.3218
FNDC1	1293.67	50.61398	46.84592	60.05231	257.9496	66.03099	444.3401	414.9253	48.05469
ATG9B	53.44072	740.6031	51.5448	101.4939	828.0306	37.31027	193.8481	50.50257	69.01437
SLC5A12	49.19122	65.97861	45.23623	55.53344	42.33619	65.68768	46.83658	49.92375	76.9166
OR9I1	54.57638	54.18206	41.8387	73.31618	51.43869	48.65619	39.54361	43.79323	51.96835
MEP1B	202.8173	115.5697	69.57333	145.239	70.96422	95.77072	62.67695	94.97736	159.2459
FRG1	9794.037	9912.695	11909.32	10354.82	10016.22	11085.98	10130.89	15902.65	9693.295
MANEA	60.95173	1073.525	284.5968	45.84682	346.9296	825.7267	317.6028	51.98701	1601.146
CHST13	2617.673	1204.443	2564.949	1710.681	1170.422	2248.645	942.1427	1731.777	1902.612
VPS16	35.55036	739.8956	35.00112	34.7126	176.2265	34.24434	121.039	32.12208	45.60814
GPR65	753.2664	880.4135	888.5718	406.4536	113.336	714.3615	368.3009	227.1609	999.928
ARHGDIA	991.4066	1796.801	1593.759	1376.001	837.608	1110.172	1731.202	1205.857	742.5767
CDH19	50.02224	38.36967	33.36362	33.07166	38.24926	39.12282	91.82777	46.73211	90.74961

LILRA1	48.65908	38.94606	38.27895	36.555	44.80872	55.29031	39.3222	56.20143	50.42335
C6orf182	514.2941	374.7098	261.5753	304.4163	160.8177	297.6425	215.0304	144.0665	625.9973
PRSS1	153.203	110.8242	57.42136	146.4596	45.08469	79.73979	52.69962	51.50179	53.99186
IFNE1	59.38286	61.241	35.7451	58.21037	54.57218	54.84306	43.68042	49.30825	100.7626
ZMYND11	1105.762	1926.919	907.9051	1509.051	1064.093	1197.402	530.7872	1109.461	35.54157
ATP2B2	38.1937	44.91248	42.98271	65.79494	37.17015	47.46136	40.76221	39.21213	63.32673
AKAP4	110.1514	107.8872	58.30934	120.2014	78.77593	94.62737	61.09164	68.50418	123.3625
HSPA4	594.5366	286.8926	415.3172	372.4655	367.1853	380.2893	279.2754	449.7248	53.98362
NUP88	1367.845	661.4445	1720.382	2459.671	3137.781	1489.564	2797.877	3332.807	896.029
MKI67	93.10197	1531.039	332.3314	1243.164	225.7387	1081.587	426.9193	1410.848	362.6249
MTHFR	102.3199	40.36895	184.7891	38.79704	63.3362	49.59246	142.2728	314.9853	188.1826
SELPLG	277.9589	541.2528	893.5552	342.8756	271.4244	365.2224	227.3284	265.6329	1111.043
SFT2D1	78.21857	94.74848	427.578	65.70663	698.6057	56.73011	527.0673	305.1509	75.82043
SLC4A4	36.98124	39.98362	41.88654	44.38702	42.17693	42.33157	36.32778	54.64345	43.18541
CUZD1	67.31414	63.81002	54.80267	47.73601	151.9847	54.06218	98.67173	71.60828	67.21732
C1orf156	79.9045	57.6954	54.68512	67.56389	43.4067	50.72528	50.94609	38.8329	526.4432
BBOX1	1556.382	456.4387	342.849	75.02508	846.1965	709.3876	236.4619	677.8828	1111.38
IL23R	38.58211	54.01334	47.23525	40.7802	36.74159	75.35406	41.81138	78.61654	40.7924
GSC2	50.99726	33.34761	45.59334	36.05983	37.20734	33.23923	34.1137	38.9533	33.82479
C4orf43	797.0073	51.77684	171.5501	49.36779	313.4311	201.0244	210.9739	292.2386	752.426
ATG2B	207.0368	119.3157	132.17	96.68404	144.1685	220.8505	115.9117	124.8255	241.7817
KLF6	33.88895	325.4069	1469.53	746.186	2453.069	1509.757	1700.969	1194.578	40.39302
OR4N4	72.75295	37.28809	47.12404	45.75507	42.76107	41.48764	42.20169	47.41874	50.50591
A4GALT	3272.505	2246.247	2832.942	3361.499	2399.584	3431.734	2670.237	1692.718	3713.349
CHST12	822.5232	3196.211	3106.535	1697.888	900.3749	1251.324	1714.144	2252.042	1393.14
KCNG2	35.47292	57.81019	52.50771	55.46053	36.81517	58.13731	47.97954	40.62387	54.26041
KIAA1841	36.51271	41.09563	33.22752	43.87562	40.56866	45.15704	39.75384	54.16967	35.01574
TIMELESS	1730.911	3551.913	3313.251	3569.937	3421.775	1959.664	3388.354	4015.517	4185.457
ANKS6	3568.009	2893.931	1142.551	3826.307	745.9226	1874.131	1105.65	1303.243	3212.7
OXT	828.2543	53.9422	532.717	34.48642	554.1298	494.5496	195.7426	539.8518	32.82201
C1orf38	174.9914	103.3219	65.89919	146.2493	60.78716	77.29635	73.48164	69.75113	167.688
EIF5A2	6713.128	5920.397	3552.219	8517.274	3988.067	4764.95	4246.036	5408.253	6588.11
GLB1	50.22761	38.08706	445.0416	35.37019	138.5635	225.4114	420.6429	301.4995	40.18365
SLC4A11	250.2499	156.8048	129.8314	76.87194	498.0094	77.546	219.8465	232.7122	307.5152
GALNT12	327.5808	174.2095	99.04079	219.0711	81.97825	139.4068	169.1269	198.6113	283.0455
SUV420H2	415.7751	203.3228	118.7598	203.9193	131.564	182.2102	103.0721	154.1497	516.9222
LOC151121	40.98254	37.6335	46.17688	63.82617	55.29036	42.43396	41.86997	58.01639	42.26244
NCSTN	475.4523	2384.482	2143.509	1950.381	3428.382	2837.79	3874.372	3154.907	2305.411
COLEC11	234.1898	164.5222	72.70461	168.706	195.4853	416.5898	258.3293	55.10059	216.519
CIAPIN1	658.0849	37.94034	371.6381	71.87708	506.7548	42.96552	394.3263	280.8781	68.59193
IL22	953.4386	46.15718	54.73136	82.68212	50.00632	44.6619	40.50626	36.97373	123.2405
ZSWIM4	406.6986	203.6821	108.2217	257.3991	98.13834	157.3525	131.8853	118.0337	365.2906
WDSUB1	75.60742	338.5701	171.2075	69.664	209.5361	314.2007	237.8866	168.6942	64.93738
OR13C3	36.00948	43.49092	39.62891	54.21653	36.01869	39.01323	37.76113	35.49185	34.0858
ENKUR	111.3328	70.24696	56.85489	72.62858	54.44967	74.69341	42.1163	56.93405	95.43622
MCEE	42.59438	842.6522	1391.295	1068.832	1266.88	1437.485	1121.784	609.9232	1163.385
IRX1	495.4142	270.279	163.0045	318.4134	134.7184	243.0914	151.0662	177.0235	665.6826

SDAD1	12279.54	11490.83	9294.954	11656.66	7540.409	11514.49	8263.87	9563.379	10755.93
ANKFY1	2940.759	1831.716	666.4859	886.5325	780.938	1191.769	551.1786	48.04544	105.69
GJB5	434.8066	397.9849	412.8111	564.6343	487.6873	571.496	464.776	756.7188	637.386
SLC22A11	52.38061	42.7513	278.3558	58.32531	543.9578	54.41869	46.05317	723.6816	44.50598
WDR22	8123.17	8822.091	7981.179	6106.922	4656.077	7638.64	6032.328	6610.911	7154.442
NUDT16L1	266.2231	74.5684	150.2935	93.58398	301.8382	202.2021	342.2422	390.4958	419.0294
ISCU	46.46313	429.3765	926.6324	63.15107	977.6238	429.3412	1017.394	1203.975	43.83406
FTHL17	39.79748	57.26717	54.98362	40.29558	34.65132	43.26836	39.54902	49.30379	40.73378
SLC7A9	63.29586	572.4149	980.2953	34.89931	649.9251	935.0651	1010.964	468.6303	48.73896
FAM98A	22141.71	21592.22	20363.39	21423.87	21864.32	22335.18	19825.71	23772.34	21194.6
DDX21	47.35088	103.4621	41.08	63.80876	100.7363	204.084	94.02666	95.81188	39.99746
EXD3	48.03776	68.69136	494.565	46.84598	708.3829	60.85479	179.039	65.10574	62.61761
FRRS1	199.4856	176.7649	91.69717	215.4515	671.1704	122.0943	151.6956	230.1908	311.5432
F13B	34.21956	33.79339	39.03481	32.193	33.12549	56.90425	46.12227	32.50259	55.87608
TBC1D8	80.598	69.90678	198.9617	77.98208	198.0838	82.2345	149.8336	60.36047	203.7725
DCBLD2	59.74328	37.45883	154.6985	491.7556	264.2116	61.42891	724.0222	210.8272	38.98201
FAM181B	3011.043	2070.189	923.2655	5039.947	3276.387	35.86133	7316.417	799.5858	42.02668
PKD1L2	43.75502	43.01907	44.58471	34.89559	37.30867	39.64661	44.52425	52.08115	60.41721
HIVEP1	72.85628	53.58533	36.66909	66.05106	39.63735	523.75	118.7442	57.42004	53.94049
MAP3K9	87.57233	125.8469	85.95014	178.2188	76.54758	89.6749	60.05109	82.67191	288.0187
HOPX	58.32961	56.64136	117.5	78.52002	116.9899	52.2907	47.34689	43.13657	183.365
DPH1	40.71838	43.56652	46.95066	45.21077	47.86959	59.76929	37.05275	62.76387	42.69297
GUCA2B	131.6921	81.36525	59.96012	55.07221	43.44698	73.92206	57.21251	63.191	186.5282
ST8SIA5	1813.806	108.1485	1199.706	2052.493	700.218	2457.144	2560.948	3361.165	174.0855
AGAP2	69.62083	57.92335	131.4038	55.24141	53.77803	56.12238	34.984	57.86756	113.6082
CDC42SE2	812.4817	596.2006	856.433	764.3403	379.9111	493.4618	433.2343	461.8252	1020.034
C19orf47	229.9816	150.025	79.67768	158.1073	229.8574	76.2204	76.33792	321.4258	102.3721
FBXW11	209.8093	115.5227	94.81461	216.6364	79.15099	67.86082	70.47647	62.38191	48.25094
DHX38	100.8279	76.82767	35.61162	80.63307	48.96577	80.58978	59.01879	51.34497	46.23065
TMEM30A	421.3652	216.7225	174.2765	227.6202	172.9845	240.9233	142.4017	176.7419	378.9103
PIWIL3	123.7599	97.61591	74.94685	97.02708	62.49123	63.79259	66.64524	84.76022	160.1988
NMBR	49.06679	40.7199	45.51284	128.8173	45.98168	48.4585	36.19132	39.07615	55.85366
KRT1	792.8041	4838.118	898.7517	5669.7	18131.7	4316.693	1927.231	3426.376	69.83233
KATNB1	2629.328	1928.474	3469.521	2199.842	6796.567	3433.016	4831.886	4243.107	2363.63
SLC11A1	49.57052	49.13099	44.83197	50.4699	47.8037	46.52236	67.50077	37.94241	69.11573
FBLN7	175.2923	258.7111	105.68	61.30306	67.2177	89.50106	75.53839	92.92702	128.6039
OSTN	74.43167	37.15561	34.48401	39.24335	35.54552	39.77114	39.03804	45.53244	56.09024
NPNT	77.40365	90.36811	91.49298	228.7626	84.91756	68.09903	60.51828	101.1775	355.8989
HTR2B	104.5063	104.4704	61.85416	85.81186	59.81111	68.86039	63.1386	67.40097	59.97195
RAB14	69.73283	65.38835	214.4639	320.2917	126.3184	239.7845	303.7615	148.4904	787.4136
KCNJ8	51.01434	46.45207	1097.668	44.80138	1151.287	416.1708	660.7556	972.4847	75.00848
TRIOBP	236.3849	178.5538	1738.798	202.0111	1717.453	587.7523	1639.563	1191.185	277.7647
OSBPL3	1425.898	1517.77	1079.775	1714.796	959.6184	1913.943	1063.093	691.1066	1229.648
CYP4A11	75.45031	74.31191	55.45523	102.9873	56.44333	57.62048	55.26365	44.30062	104.0066
TTC30A	1053.123	456.4901	747.796	1057.619	1138.506	1330.681	795.8513	1165.701	584.2899
NISCH	3285.75	3772.952	4847.912	3063.471	4943.094	2801.133	5304.512	4315.963	2130.568
OR2T3	46.03373	35.73946	39.28583	50.40059	36.10341	40.30134	37.95161	32.81487	38.20215

UTP15	504.254	498.8745	376.8561	429.0424	322.2729	314.8393	350.1443	448.4866	109.5035
VMO1	371.2878	177.8827	237.0186	189.6811	98.13441	147.3412	143.5093	105.8133	472.2493
KLF2	9817.482	9513.846	7772.025	8685.494	8203.136	7319.91	6967.844	10723.21	10520.76
METTL2B	867.7415	618.4823	259.5154	390.1932	187.9606	356.2079	187.6364	198.3387	977.9446
CNOT4	1932.372	1209.491	757.1474	1312.548	587.3663	1100.967	344.5595	857.5518	2424.881
BCKDHA	11835.4	12778.36	9834.791	10414.86	8205.753	9636.754	8173.791	11397.71	13281.32
NOX4	3206.233	3134.386	2166.863	3332.382	1393.614	4974.237	5130.944	2819.585	3599.705
COPS5	801.9335	612.674	674.7679	660.2739	1192.1	714.3399	1288.242	1563.302	1356.537
CIB3	54.73205	75.35189	62.03669	76.50363	58.04838	72.86777	58.46423	44.76709	90.55746
MMP8	37.73399	43.17487	33.36026	35.30032	31.67236	33.02739	55.41083	36.7928	42.03193
ME1	500.7469	281.068	166.1055	271.6137	256.3548	126.6987	128.4478	148.3962	132.7002
ELL	184.9239	852.8699	810.7759	1594.171	1151.427	1284.102	1531.029	1232.861	995.954
SUB1	68.91814	574.6816	2467.558	442.1997	1972.939	1769.492	2841.646	2591.851	57.45372
DEFB106A	81.71535	72.12307	42.9191	48.52559	51.53119	60.75723	36.9464	39.36689	51.95352
GLT6D1	65.36475	66.19096	67.72067	104.0244	70.70554	74.29057	58.17217	62.82916	265.3412
CNOT8	58.65229	55.72174	111.9393	212.1344	104.4285	39.02255	60.76382	57.8154	67.25139
LIAS	175.0802	85.67848	103.2302	253.566	59.60529	84.47929	92.19854	68.99873	591.3113
THBS4	37.91428	70.37674	41.96261	48.0935	481.7473	492.4255	579.3223	171.4867	48.29634
SNX33	215.7191	119.7445	89.06207	134.122	58.64074	69.34731	85.9069	68.43924	60.52282
DLL3	2110.192	33.56341	43.01364	39.04496	279.0804	39.17722	150.9302	40.87222	51.5443
GLIS1	38.96211	331.1133	38.84465	43.11026	39.06087	38.69492	43.30502	39.26821	33.43099
ITPR2	41.75344	1520.767	1382.422	761.6584	249.165	411.2407	574.6041	699.0528	1306.244
ARHGAP10	1675.091	778.3176	1355.413	218.7532	2135.146	495.0797	2246.721	2280.151	443.7428
KIAA0773	38.15683	64.77971	142.5791	80.55677	212.3088	262.353	112.5257	96.48415	109.3891
C18orf22	1622.35	1676.595	1336.771	3739.249	735.6164	920.5675	695.9379	1497.999	5099.812
TYW1	184.8967	161.8112	97.04137	96.16943	57.64026	64.26353	105.414	142.1182	132.6343
OR1L1	34.90466	44.14024	37.72362	34.21461	39.49367	44.5927	36.21677	41.29048	61.0737
ProSAPiP1	41.37234	36.0279	43.4298	47.72006	1120.892	48.262	764.2434	45.92353	39.13721
GC	113.2418	69.41702	58.44049	55.31612	59.04344	62.06723	58.8798	64.80285	219.0799
PLK1	513.0214	313.6587	195.6688	520.2827	222.154	388.8918	379.9199	194.4017	708.1069
AEBP1	1672.716	1395.547	2108.024	1184.311	1770.148	1010.659	2369.831	2302.682	1645
FRAT1	57.07232	46.25937	41.27805	46.37207	137.4553	47.6084	40.69439	47.76971	62.24715
GSTM3	7336.256	5533.682	1593.533	3010.067	5526.152	4266.382	2771.951	4802.375	6571.968
CHRNB3	106.6924	53.35804	58.65194	87.49989	61.65136	64.94389	57.61377	48.8575	226.4224
C1orf77	63.85355	1874.33	2678.124	1883.986	4160.258	3306.216	4102.46	3488.514	39.71358
GNMT	102.5758	82.942	74.88059	85.45122	59.10205	76.53809	166.2789	46.94718	85.32027
PLEKHG4	43.35123	1271.928	1268.666	2727.847	2912.702	730.631	1253.324	372.074	49.44523
CALR	196.6001	187.9073	139.3841	52.08328	264.2474	169.8227	172.1034	229.8137	130.9379
ZNF555	576.9924	297.4699	54.6813	55.86153	44.14241	281.1627	91.08558	117.4006	40.87014
RFT1	217.0072	161.0744	84.26881	116.39	141.7938	129.1369	143.4244	95.52724	291.1635
KCNV1	74.21692	58.67642	48.63097	48.10114	40.90503	47.3914	41.75775	69.63887	80.11559
POU2F2	36.45005	30.97442	37.4926	32.70919	33.28939	45.1202	34.97496	32.89304	38.3681
NALCN	41.42818	53.18434	54.18138	82.23258	53.82882	54.14941	87.70224	39.11937	101.3254
CDC42EP3	95.69849	101.7299	560.0394	6038.993	654.5945	72.36875	1062.489	861.9349	73.74123
ANGEL2	12486.33	13553.36	11902.96	11735.97	8853.875	12031.88	9978.554	8313.534	14009.67
AMHR2	3402.304	3540.534	692.8884	51.18805	907.9997	1680.061	729.1886	1843.849	1993.972
PLCB4	76.58586	117.9093	67.78306	130.7932	63.96636	61.57247	53.5887	69.97807	215.9902

PDXP	97.95385	61.05128	782.0375	1508.967	1303.708	977.0466	1186.245	1841.013	90.05721
LENEP	36.13737	37.00367	40.33429	39.73077	37.17211	49.50567	34.51411	46.71092	63.92021
VWC2	181.6473	80.57104	74.29418	117.0911	69.20789	76.08606	60.29291	76.11472	199.6133
RCVRN	4491.965	1104.832	2164.025	1168.986	775.7524	1729.896	496.4749	1181.511	2969.77
SECISBP2	636.8731	2884.957	660.4376	900.2131	836.7241	1092.319	940.2025	1425.714	176.4788
GNB2L1	21264.69	22636.86	20250.26	22026.41	18288.73	21687.07	16918.15	19847.31	21179.72
FOXN2	10800.43	10886.34	12251.57	12189.7	10102.56	13431.99	11794.54	7896.132	12056.1
TFCP2L1	46.7301	1227.735	355.9116	1164.382	724.547	1391.971	909.1176	972.0034	46.77747
RND2	7912.875	5285.963	2730.81	2974.419	3785.94	4379.523	2337.426	3225.868	8407.895
TPRX1	49.50703	82.28177	49.41286	55.78278	45.01197	57.49579	51.96814	42.87102	239.6845
SLC16A14	169.4662	69.28161	64.84538	136.1514	54.14561	43.35075	61.38136	61.06609	58.35665
PHACTR4	1033.288	625.3394	1272.797	501.3722	1719.069	665.0484	1475.717	679.9547	47.88533
ZNF280D	51.84396	95.05961	792.8903	1615.083	1130.11	1844.71	1053.062	805.2723	106.8521
CD74	11792.79	13701.76	13274.33	9630.455	8745.451	8203.097	10394.89	11641.31	14394.85
CASP2	53.85696	970.9713	306.0929	32.49812	1597.782	473.4115	802.8773	620.7411	49.0229
TRIM28	839.3662	1318.254	1243.992	1710.669	1351.317	1779.689	792.8682	637.2618	3380.651
ZNF688	1004.185	1532.844	1604.586	1181.511	1613.366	1683.526	1939.495	1129.005	925.4973
MCF2L	39.83158	35.35274	33.95678	32.16893	36.19933	60.90003	36.7943	40.7538	58.85524
TMED10	63.22836	49.15051	424.7102	60.9646	512.3174	344.7893	313.3698	229.9492	257.1176
TKT	53.42723	1231.754	802.2531	58.4981	2277.652	371.6583	1278.23	1142.471	2142.292
PHOX2B	118.1603	75.13974	56.70341	152.2245	56.48617	88.91045	47.43843	80.08189	101.4565
NCKIPSD	4198.852	5883.107	6486.049	5839.895	7179.286	5583.309	7618.386	7347.475	5546.088
ORAI3	64.13074	59.47445	122.2372	37.85561	103.7055	46.19066	248.4035	247.2284	48.73758
JMJD2A	481.5646	184.6549	104.4932	192.7002	84.39928	106.0638	58.67206	69.51369	270.8748
LCE1B	96.31253	149.6731	69.8934	474.3705	2084.929	103.1232	179.2396	163.1132	152.2977
PPP1R3C	42.64399	486.4349	405.1821	350.4213	260.8903	285.8257	125.9702	120.525	110.2682
SPSB4	137.0236	135.6361	77.34873	160.9747	77.08074	148.0995	70.97171	80.21796	259.5135
DIXDC1	37.24115	34.02885	38.96236	44.2849	35.62795	43.1615	40.60604	38.04771	40.56287
SPIN2A	103.8214	48.26937	42.81747	69.31792	44.13045	57.1347	44.63945	44.56266	66.1676
LRRC16	1162.324	36.93901	470.5811	483.6617	480.5522	35.82067	379.5411	356.5358	46.99583
RASSF8	229.7369	208.1439	118.4008	200.8324	91.514	142.4271	104.5726	164.4335	383.8718
GLT25D2	53.02046	43.39832	39.76043	45.16582	39.3253	35.70894	38.83482	41.28459	41.40027
MARK2	38.97987	36.08655	43.93313	48.01036	48.61494	46.37548	37.29387	33.76748	36.48745
VPS26B	431.8669	1217.184	550.3718	775.9745	480.8851	410.4332	158.4013	577.0479	564.9278
COMTD1	176.9662	398.3823	636.3966	145.5951	1083.1	341.9827	753.2028	649.8274	448.2109
GNPTAB	176.1673	77.75737	55.49807	54.14705	61.29755	62.44567	65.88786	77.39668	62.72105
RB1CC1	326.1584	209.5033	438.3349	341.0597	1333.556	1075.581	1009.896	788.7578	447.499
CLNS1A	36.45542	934.6713	1692.17	605.0279	2247.921	1788.228	2754.089	1713.373	65.77103
C13orf3	838.8999	917.4915	373.9467	1019.4	362.6291	459.836	395.942	709.3458	96.46626
HIST1H2BN	3601.76	2485.105	2160.431	4913.754	1716.984	1850.996	1927.004	1389.28	35.13758
CHST10	53.4345	63.64697	977.1917	43.59474	408.384	54.68347	465.0498	332.3087	46.44617
MDM2	72.86761	1204.029	462.8228	863.4976	234.0164	308.9271	426.4962	421.7607	36.1133
PRSS33	66.58334	54.31739	37.31831	71.57201	42.43503	53.69793	39.94847	44.98522	38.25826
SKIL	6284.875	5081.89	3022.28	5139.177	1892.617	3551.948	2659.224	3677.517	7484.882
LOC39989E	63.48335	43.44335	43.22375	90.29016	41.6163	52.21278	50.57786	49.77877	35.45029
TMEM140	1171.919	94.00005	679.7844	94.31214	395.4533	664.9006	723.765	691.4085	373.1082
SAR1B	2825.277	3378.691	2824.353	3164.089	2688.778	3240.553	2643.195	3657.178	2434.988

ZDHHC23	518.4574	233.1816	266.9531	78.61601	413.7865	214.9757	219.1395	185.6114	668.1291
TMOD1	2773.749	3350.136	1867.867	2072.462	1878.631	3935.432	4221.19	3631.416	3719.345
PLB1	181.6979	142.0916	170.8103	137.3959	144.5414	101.3005	165.5325	101.0426	195.0031
UGCG	947.7813	1858.737	2142.218	2162.5	2095.4	1639.171	1788.87	2060.198	1692.71
HPRT1	110.0598	41.06318	42.18223	33.62845	179.5917	33.31	151.8524	57.74189	64.71834
CEP97	77.39152	60.43427	42.70708	49.91455	42.2467	35.72296	54.99332	110.708	77.77111
ZBTB7B	1112.29	1100.694	820.1835	137.0195	1060.594	359.9406	817.978	557.9667	182.4145
SIGIRR	154.0992	86.18103	61.81398	201.3707	75.66055	82.21162	70.06249	247.4233	179.4559
SLITRK3	79.59805	59.06496	52.17353	57.50511	50.37736	46.19488	44.08181	41.89185	55.62037
GKAP1	237.7578	295.9752	140.1444	128.1964	156.8715	191.7645	112.7409	170.0317	103.1206
SOX12	111.9581	164.7229	70.56047	112.7566	125.0454	96.43346	166.6438	98.06049	117.0261
HPCAL4	142.7162	88.4157	626.05	1356.85	529.9706	901.4082	371.4962	958.3138	90.10519
BRD4	77.22871	58.36942	74.38405	69.52224	59.31124	78.71153	54.53333	80.97066	427.5194
PHACTR1	121.0376	151.3804	310.1074	137.4055	75.48184	566.4814	395.3508	90.12706	249.5809
C12orf66	1266.288	1217.998	2145.335	1631.965	1169.507	1926.857	1502.42	2063.612	1615.359
C19orf61	1184.054	53.71681	204.1665	91.74812	54.62295	94.07954	154.8248	359.7816	1683.221
GOLM1	34.95026	52.23024	38.47466	41.5411	215.8877	35.62223	36.20984	33.16168	90.81277
RPL39	3366.117	3861.546	5151.523	6744.999	8403.621	2940.264	6699.982	5944.812	3019.269
PLCL2	2013.091	3786.585	3709.589	2167.815	3262.802	4446.146	3580.787	2388.338	2738.839
GGT7	146.8711	243.0083	77.24728	64.32675	62.78602	93.94922	51.21912	89.1496	175.0022
HABP2	58.48388	44.50338	36.02266	39.2735	34.1158	50.69761	36.02245	63.2996	48.31468
TTLL1	57.7779	48.7914	76.67329	40.11861	51.07195	41.76609	51.34552	41.1912	52.34015
HHLA2	36.887	52.70471	44.03924	44.04657	37.69996	48.69353	42.56481	33.15308	46.6792
TXLNA	4546.641	4375.622	3965.857	4181.741	3027.781	4740.347	3484.218	4671.708	4731.344
CAPN5	11904.67	8681.939	9510.66	7536.609	7060.971	10283.48	6394.817	7204.46	10963.66
LTBP3	144.8659	75.44648	65.72746	125.1942	57.82791	84.70557	58.85074	65.12092	285.0991
DKFZp564N	45.0294	46.88145	33.36721	48.18975	46.94535	47.63753	45.95383	47.94342	57.27824
OR6C75	95.18786	41.58935	59.54268	384.809	50.93673	70.67086	40.54006	50.32691	63.76388
TRAM1	1837.117	1000.482	3949.64	2714.09	8224.637	4614.521	7696.493	5255.258	54.7327
DAO	244.3143	155.2736	80.41471	190.1207	91.47171	152.6106	77.19122	80.28705	383.2413
GRAMD1A	4400.554	4366.663	4444.425	4288.177	2482.662	3626.389	2808.724	2633.516	4482.208
LGI3	51.03526	46.97578	51.62635	37.60815	44.45078	48.52114	35.20465	45.08157	111.3669
SH3BGRL3	2262.291	1814.712	1831.956	3079.578	1385.536	2161.076	1612.647	2254.545	1955.465
THTPA	2630	1516.373	279.5505	65.03999	414.6203	740.6049	88.30352	600.5842	3208.777
FLII	55.92052	58.86868	41.62573	54.11994	46.79889	70.03709	42.48047	168.4089	84.46355
CDCA7	1459.811	209.7051	1818.193	211.8103	3598.966	1782.025	3040.417	1034.581	458.3781
CENPK	35.26756	38.32903	119.1254	58.76789	41.76175	42.49136	104.7831	124.484	887.5152
MCM4	1161.826	57.89911	533.903	1828.426	717.6696	916.9376	1033.427	530.2389	102.084
NTF5	283.5458	50.52456	522.7663	94.20202	352.7529	711.9069	735.3045	624.5333	90.82973
ZNF585A	1032.627	2297.331	2476.773	691.6114	1419.337	2236.857	2189.853	1778.934	1710.176
C8G	93.79875	78.53736	52.38879	70.33868	241.9769	73.78429	49.79752	97.55092	72.31889
C22orf36	41.80253	163.368	69.03561	239.1773	71.99821	158.9316	116.2611	104.1523	47.31687
TTC16	142.2688	100.6392	168.6331	106.1027	219.1494	93.72507	98.20137	62.13187	292.5446
MAT2A	3249.695	6633.731	10548.72	6573.743	12200.44	8971.296	12284.75	11299.74	1492.672
GLRX	229.2021	776.6572	1566.909	885.3207	2042.111	1597.714	1469.922	1278.286	286.4618
SLC25A17	75.41953	56.5892	453.4146	76.39822	528.0237	352.9879	511.722	48.93932	74.61335
BCL6	3594.424	2680.307	3599.9	1924.913	3324.608	3408.99	3813.763	2806.578	85.45052

ZDHHC15	41.11266	44.55825	51.63103	54.34917	118.0187	35.39787	38.25532	39.76612	37.5561
SLC10A5	55.74081	33.86903	38.4138	38.81712	45.95613	40.59641	63.89167	40.03056	35.41043
FNIP1	59.3695	53.25697	197.4205	70.13712	160.7466	60.22018	102.0118	215.8605	92.84455
DDX41	75.02933	1273.468	2371.353	51.79355	2410.184	1173.184	1927.454	3717.699	49.60646
PSMD14	46.24933	973.8143	1462.907	1676.055	1953.991	1767.291	1896.271	1310.604	49.56943
MSH2	208.4611	120.4088	92.94589	89.05535	62.53179	83.25295	65.31907	67.12367	68.68202
PGBD3	62.27564	55.52549	134.4958	46.97802	118.9936	39.72488	66.80875	199.7827	35.49087
PCMTD1	3436.615	3032.336	2407.649	2405.646	3146.367	3376.194	2898.176	2649.835	4046.604
RARG	725.5991	824.5385	261.1049	258.1667	264.3735	178.7254	138.9232	210.3374	1592.357
FOXD3	65.14704	57.11507	49.0835	46.5804	42.50827	51.57787	42.83463	47.13759	94.25659
OXR1	4196.509	6242.458	5916.362	6256.202	7478.908	9566.893	7106.228	6001.979	4971.779
ABHD11	35.33801	33.21612	136.0693	37.63045	32.77477	33.89389	34.08865	31.7505	41.71467
RTN4R	50.42343	89.50873	69.06965	43.05267	60.10067	51.9782	39.30901	63.40272	69.9895
MYADML	281.8533	191.2521	94.4965	198.7105	101.4169	141.7193	83.75613	83.39731	324.7993
KYNU	118.3953	89.97002	177.222	173.9765	53.53335	73.30685	106.3638	108.6915	51.2061
RPL29	68.20071	51.22277	174.5732	51.73514	253.7846	37.96135	107.4704	34.36087	34.55547
TAF4	415.1365	1585.412	1112.315	1920.951	1359.017	2421.005	1276.903	740.7693	1995.794
LOC88523	9376.413	9676.641	10896.15	6789.507	7649.785	12130.08	9390.105	9902.468	10288.7
RS1	44.9781	538.2343	53.27344	54.45449	56.484	48.8257	42.24512	48.7373	94.5841
PPPDE2	120.3307	56.01591	482.8443	63.61814	508.5808	324.654	295.5474	628.9032	83.77364
VARS2	16059.57	15826.29	17788.16	14355.97	19246.54	16687.49	19860.18	21242.37	15428.53
FLJ10986	247.6209	179.417	381.3458	173.3643	83.00456	614.1382	285.2184	108.1186	2839.341
VAMP1	1327.707	2537.12	2780.808	1955.427	3583.725	3815.893	2941.559	1616.794	526.5449
ANXA13	37.07672	36.92728	40.78125	41.73362	42.41484	38.13942	39.92159	42.33589	55.48928
C15orf55	85.92933	75.06353	39.98556	79.7739	61.17017	58.11395	49.52128	50.78516	65.49155
ADAM20	38.4135	41.7892	117.4766	32.02685	34.37038	32.3527	50.11441	35.25785	39.69132
RAD1	540.1218	254.9648	186.1339	294.9326	141.4636	277.8596	171.8222	127.0702	65.33279
CSAG1	68.66933	805.1527	1077.072	105.9683	54.95485	52.71603	247.3952	660.9251	322.9128
TMEM219	2865.419	2662.779	4496.261	3265.763	6195.056	4937.448	8398.367	8103.751	30.94066
DIO2	4686.646	148.0624	886.8265	865.832	2307.6	873.6475	856.5139	1088.416	193.1755
CDC40	3275.399	2746.914	1499.311	2574.709	1324.167	2123.422	1239.075	893.3568	1185.247
KRT78	26366.66	15448.74	22674.39	13117.22	18547.54	16418.81	9824.951	9362.931	23930.83
BARHL1	64.91856	55.15953	42.71783	75.80916	48.90577	60.40505	48.99574	59.33859	70.85942
C7orf13	363.1445	226.7999	89.43703	1075.576	81.22077	143.7674	156.8332	90.58828	423.4804
NEK10	60.95754	68.07758	66.96149	67.82622	52.81034	70.36625	61.87125	67.41312	118.9864
CRLS1	67.43179	1271.298	63.0639	1292.69	313.3523	57.12764	553.5003	153.5565	108.0882
PDGFB	142.5636	131.8963	70.74546	195.0342	497.3983	624.8853	61.3017	419.7069	227.1149
RNF126P1	66.37346	60.32011	57.92307	56.2142	48.49247	59.86256	49.17116	47.7675	44.47062
RPL10L	471.1396	264.4488	334.6807	266.905	96.5173	183.8628	88.82542	576.9111	625.54
CDCA3	374.5052	1394.516	827.1328	4154.273	439.1865	767.9225	579.2677	1355.426	47.21947
NDUFA12	13436.9	18014.71	15504.59	16529.15	11377.45	13248.91	14558.1	16297.53	15487.85
AKAP2	818.9545	276.4029	32.82435	430.63	108.6993	39.56334	123.9345	167.8072	45.08816
TPCN1	3003.361	4093.399	1232.602	3878.752	864.2421	1762.171	778.5671	817.2257	4073.842
CCL25	68.55603	662.79	320.2014	52.27934	770.4734	54.1165	149.1021	56.83081	803.9789
C12orf43	4526.467	4858.062	2563.214	3132.147	2000.914	3417.529	2888.972	2400.832	3208.36
C7orf16	40.5948	49.42064	52.61633	49.58734	49.05097	40.27272	43.35637	52.04448	78.03641
RMND5B	7033.171	3660.297	2695.619	3367.316	1699.983	2257.037	1252.115	3347.5	6626.266

GTF2B	64.89999	59.8475	204.1021	112.3073	264.0205	398.6302	312.9216	270.0224	2847.215
ZNF70	40.63148	82.45925	40.56652	51.83937	37.84081	38.46395	40.16361	46.44466	39.99402
PSMB4	456.7804	419.459	851.6448	38.35873	1148.654	405.3277	1698.361	1228.695	839.6719
C1QA	762.3051	331.9053	120.4154	183	124.6479	128.1775	98.76474	145.6837	459.4576
THPO	42.07095	39.37952	169.0995	33.15942	112.7019	48.57567	113.3768	102.557	897.7698
GALNS	396.0847	239.4926	241.554	240.9425	230.0972	275.9523	207.0455	174.33	565.5432
MKS1	353.5041	216.8491	103.6092	236.4084	113.9657	176.8988	95.16221	123.4841	484.2368
DCAF12L2	102.3507	34.94204	41.53976	40.05591	39.32031	49.53181	43.03753	42.7532	62.69517
HIATL1	2141.621	586.2622	838.6668	1756.733	571.0362	1093.054	726.2046	320.5402	890.1292
ELOVL4	14970.79	15308.55	8728.48	16688.09	19623.11	9440.658	12370.29	15803.47	14137.52
TSPYL5	35.952	44.64589	45.90047	54.50223	48.83943	49.37154	64.8465	54.7236	43.20483
FSTL5	39.10302	881.9394	39.24531	482.3242	37.68042	40.19022	183.1589	397.4203	48.75239
PRNP	3119.901	6364.396	12215.74	13055.88	16029.74	17313.63	18446.21	15914.9	929.3895
ERBB3	344.2901	192.7184	391.9509	233.2142	308.9362	158.8803	268.5602	247.507	357.1603
POU3F4	130.3921	115.208	63.93416	128.323	67.66375	67.71246	58.19834	56.65976	127.0123
CTNS	32.82204	224.913	33.9116	34.7728	157.9787	37.21122	32.20064	58.3677	34.09653
BRCA1	190.2978	1419.047	2058.516	1641.804	1528.135	1604.263	2062.74	2304.715	1169.619
OR2T33	156.8674	124.4082	52.11509	110.4073	73.72796	101.2523	52.70138	76.66691	312.6892
CPNE6	111.0445	74.69907	70.17959	81.72398	50.8005	73.20231	59.04551	55.48809	125.761
TMEM118	58.79808	704.3788	2798.787	91.99931	912.0598	1596.523	3745.881	1391.618	45.31583
PER2	3419.399	3683.677	6678.344	3232.567	9389.392	7262.184	6359.696	5910.234	3266.829
C2orf34	65.34505	96.27815	482.9842	46.41745	483.1053	1105.121	480.5652	329.5185	115.5235
TTLL13	83.30545	57.12737	59.10256	68.95175	63.37442	82.66859	57.69209	57.38295	179.4086
COPE	46.27481	1467.107	471.0715	566.937	1389.339	775.1829	1322.949	1769.804	57.87258
TMEM119	33.17932	56.31314	555.8932	42.41753	541.665	754.5157	285.4205	592.4622	45.44054
ZNF772	290.9874	157.7392	111.2623	69.30804	63.12921	107.1715	82.89473	76.14904	91.69749
HOXA11	35.04662	48.64446	34.43454	38.51486	38.28604	41.11925	110.6983	37.22229	41.4863
NES	77.07921	122.6152	601.1502	120.3188	890.1279	71.48542	1189.04	634.7159	124.7723
SUMO2	4365.697	4801.982	4993.472	5795.324	4477.74	6932.347	4876.577	7035.094	4117.646
METTL4	127.1599	112.4626	74.78644	121.0376	69.18111	93.18732	61.61414	92.47681	249.9649
FOXP1	2957.918	1412.103	2064.56	1789.087	2072.806	1597.138	1802.27	1947.608	460.7382
CISH	59.8695	44.88573	221.5279	656.8421	70.39588	43.45997	136.5299	34.19907	61.3325
GALNTL5	51.66608	37.04869	46.19371	34.74197	40.1503	44.87449	39.38012	41.56519	35.77739
C6orf208	54.03865	98.80304	56.73656	73.60098	50.99138	236.8043	44.51811	39.37911	55.36773
NDRG3	4546.525	3108.988	2461.418	3810.762	2219.984	2087.908	2127.598	2955.764	4332.746
AMIGO3	34.80643	34.38605	39.52529	38.66662	49.70783	40.83331	42.54234	43.53594	53.94764
HS1BP3	126.0021	72.85772	56.47887	68.21083	116.5295	56.85904	55.91903	49.62626	143.4272
RBM6	2822.857	3935.428	5883.318	2431.095	5419.766	4606.366	6248.858	5729.912	49.34476
OR10A7	59.11871	86.48959	40.36518	70.9486	50.34236	57.24776	54.59214	59.28641	113.6062
DRD3	243.6717	111.0388	70.25908	143.1507	73.17163	83.98524	58.05832	65.51163	381.6204
PCDHB7	35.24551	42.59547	53.03466	36.19558	35.9298	59.87846	41.89327	44.72103	41.45797
PIGU	12942.62	14159.77	12233.19	14336.51	11870.42	12027.15	11740.11	13849.99	15565.57
IHPK3	2307.974	2393.74	1136.981	2191.016	68.66817	1237.463	855.2602	1376.119	3919.367
DHX36	162.7138	76.68989	106.9622	51.72083	219.086	442.9727	241.6598	112.8563	108.592
TMEM185/	425.0349	240.6316	205.2904	267.9185	406.8453	181.5233	257.2066	199.4837	486.3456
NOX3	47.587	49.46415	37.73968	59.95192	33.60026	37.45693	41.97757	48.23205	39.09236
SLC4A8	2172.033	4468.534	2010.169	2114.231	585.6158	1852.136	2140.55	998.0577	3443.559

SHROOM1	51.13367	67.13636	46.22203	50.60217	51.39061	42.53393	54.9705	67.46354	52.82105
EIF4E2	1770.924	2087.51	1741.162	1220.119	990.3702	1509.262	863.9778	931.905	1029.762
TNFSF18	153.7712	149.3381	63.28983	154.0255	59.92647	175.8536	70.24328	118.2471	201.8111
METT5D1	558.7352	384.1456	230.0168	372.327	235.0992	262.9538	232.2157	299.9466	671.8215
EFHA1	539.5849	642.068	1889.693	1058.477	2340.287	2130.561	1422.942	1357.281	82.81957
RNPEP	1438.218	2118.286	3481.35	2812.401	3911.331	3734.839	4130.329	3079.478	962.0068
RNF19A	90.2165	58.1605	184.687	273.2866	149.6341	36.76345	43.52956	126.8866	42.63873
TUFM	8017.196	7821.409	5987.678	7670.902	6783.032	5114.797	6195.41	7593.404	10647.24
EEF2	6251.408	7812.758	7546.249	7753.635	9027.656	6790.827	8635.974	7825.128	6535.667
FOXC1	1457.123	3167.383	3968.378	3970.383	3931.074	4116.674	4913.702	3329.429	1969.428
ULBP1	57.91463	63.77773	133.5454	383.7654	60.56066	219.8974	91.01994	38.94787	75.25993
G6PC	90.39471	53.72417	41.27855	40.0412	49.66108	40.40313	48.16811	44.36327	34.39896
SPEF1	162.5186	124.8004	88.56478	139.1966	82.37164	140.2831	83.13455	101.8312	390.0488
TMEM201	43.72153	48.35367	35.89407	45.73291	43.90007	54.93835	49.18729	34.03646	69.48172
MAST4	1079.244	495.8005	649.3053	882.1276	832.7327	593.7452	707.552	768.7538	1442.569
STIM2	480.8484	282.3642	224.1916	250.2889	229.9225	195.6709	206.6638	478.6096	504.8654
KEL	44.43066	35.07638	455.3499	41.51732	435.5188	32.96312	127.6557	42.43634	54.54724
CEP27	10742.34	10143.98	6007.764	7702.952	2280.966	3455.051	2321.438	3617.202	9910.209
AQP7	44.57605	53.2318	386.5648	58.6249	378.0609	488.1008	131.231	879.5593	102.3954
FCRL4	32.9765	31.85075	34.22208	32.5231	34.61656	40.19899	35.96689	33.13148	31.43177
MYD88	66.61344	60.36337	185.1604	149.4792	131.0618	100.9744	160.575	191.4862	67.32347
BCS1L	54.18605	1029.301	4874.676	58.07716	5700.403	1292.063	4422.381	3956.604	3355.205
ATAD3A	991.9974	1001.27	1245.535	1515.596	970.6635	567.8118	1137.642	1257.632	192.7958
VPREB1	49.36249	33.00823	87.88994	38.6986	35.08813	328.8482	43.8762	37.99509	172.8556
SLC1A3	884.0638	691.799	680.377	1730.458	614.3958	1595.52	1620.978	534.6775	176.4682
USP48	36.68784	207.2887	100.4351	39.79523	127.6465	85.25864	93.53951	42.71897	35.05101
VHL	325.6483	163.3973	65.64137	161.7813	451.8385	113.5533	575.5178	86.86434	432.2493
NR2E1	314.364	144.93	86.00979	178.2517	90.6864	137.9624	68.98516	98.85308	297.0855
ADH5	195.9113	231.8523	109.3137	221.1232	85.54801	124.336	80.52725	90.56139	346.4002
NDNL2	521.6978	252.5338	119.6975	323.8093	133.7475	180.395	139.3943	115.2698	747.5296
LOC338797	65.94422	35.04457	49.82722	106.7547	39.67406	56.21026	52.44781	57.81524	40.27596
PRKAR1A	463.3367	370.3697	195.5112	373.7945	228.4692	200.3184	306.9529	397.0528	604.5113
CMPK1	985.7558	81.2143	586.5846	86.83546	230.9153	378.765	277.2567	599.1458	1217.442
LY6K	59.10176	52.58196	176.3008	532.2894	57.62755	61.20216	121.0002	51.80405	54.08437
GLOD4	3782.046	2749.229	6330.753	3419.85	7471.542	5952.851	6079.497	6772.291	1372.743
SATB2	34.36006	30.47618	35.67594	47.69704	460.1414	374.895	120.1462	34.26402	38.92899
LOC731085	46.65019	37.05928	35.41307	43.90149	101.9546	35.10836	64.70452	45.39883	38.64999
PRPF8	269.473	3026.426	3676.278	3663.478	4917.533	3158.529	3797.805	3435.91	2791.348
PLEKHG5	46.7156	1186.628	48.71095	747.3167	49.98645	474.4484	92.74316	65.6384	1236.426
NUP62	2336.846	2040.919	6058.496	1787.735	7449.673	4021.084	6923.7	3848.715	591.4645
NRXN2	189.7926	980.3794	749.4017	1254.863	1532.094	2423.568	2431.224	710.6612	393.6604
POU4F1	48.99125	628.363	606.8261	47.49344	388.9147	52.3477	200.8897	325.6394	65.30186
FXR2	157.4282	462.1039	1094.561	468.0495	987.2812	101.5345	815.424	1226.279	2283.875
OR4N5	43.50835	56.20764	52.78023	44.72501	41.66028	59.93733	38.08387	38.50465	45.0232
TRIM26	1524.558	2201.251	2216.6	1354.867	1874.508	792.116	2281.974	1748.891	1906.731
GPR180	2926.47	2737.46	3313.342	4419.661	3201.775	3889.521	2327.781	3083.519	2444.909
CYB561D2	83.09901	231.7041	1080.896	60.119	1125.288	327.2425	1345.661	662.4994	127.4625

LIF	320.2434	183.5877	308.9656	203.1399	99.33576	144.9636	109.1465	99.93778	2049.788
SNX4	185.6412	137.0514	80.22885	133.3198	117.5664	191.3708	142.9808	124.2395	205.1056
PLAT	435.468	89.38547	1072.767	2857.11	1279.717	523.9468	1487.204	281.4288	1889.219
TFCP2	33.49622	40.12351	454.6168	35.07258	245.3221	30.28404	296.8334	112.1937	37.37606
PIGT	1559.529	54.9399	106.8983	46.68096	460.455	58.28251	607.9854	94.36376	46.14985
PLP2	691.1999	190.5433	108.3066	359.1272	103.189	133.0239	120.6252	112.0302	113.2425
HRC	80.84741	63.49727	487.7811	91.04742	782.3505	114.5903	2287.077	3399.365	141.96
GDF9	101.9049	95.57298	72.79559	57.35737	57.60888	58.37066	50.48679	61.30226	45.12033
PPP2R5A	89.0602	787.1526	1423.24	829.3824	3419.404	794.1766	1858.516	1965.189	111.0954
ZNF71	36.4383	39.75937	37.29305	44.43586	71.24679	226.5902	150.4866	36.5085	34.03603
FOXO1	102.7816	118.3138	479.0099	141.3065	852.3371	69.4307	661.9335	384.7885	43.60042
RAG1	319.8724	204.7818	168.9741	293.5055	105.5063	61.25794	154.7129	143.2032	94.75105
EFTUD2	101.8441	473.2386	647.0059	91.10619	783.6392	977.3349	1055.169	589.1683	1338.967
GOLGA2	1226.511	2229.357	989.414	1863.662	822.9951	502.4319	713.7158	1386.103	2744.398
HIC1	122.2305	88.3748	81.26624	90.34514	55.97969	84.02624	159.556	371.741	91.07434
VN1R4	190.3768	107.2204	86.94157	181.8376	83.96472	110.7116	62.64692	71.33896	450.0556
SACS	37.3849	39.1573	60.06253	43.85553	46.26473	55.42545	33.15698	77.6841	35.36576
TSSK2	55.6508	69.0216	52.7392	57.09877	48.45609	49.62787	44.11895	46.83964	94.89655
SLC9A2	242.2509	785.1801	257.2066	197.2863	1417.97	299.7825	295.7633	739.2522	1459.422
DEXI	107.4421	1136.132	2276.751	1574.308	2974.455	2444.182	3296.466	3373.705	1066.127
LOC440836	211.9302	351.5881	63.62141	111.8797	56.83547	90.34261	77.91212	191.971	165.7454
GALNT13	31.90306	44.32354	41.39889	38.79127	41.88779	42.48443	63.54091	128.4872	58.90482
WDR40B	78.133	63.26853	50.02994	79.93639	49.24188	51.80223	41.64721	41.39569	41.53734
TROVE2	699.7377	632.8339	507.1032	823.5614	280.4617	285.7898	231.6306	173.2859	820.4709
ACAA1	1768.553	150.167	193.4214	893.3734	235.6679	321.8424	246.1514	203.8648	60.2594
TIMM8B	2526.46	2433.668	2113.178	2022.328	1757.177	1901.432	1471.449	2546.083	2620.874
C10orf104	1213.097	2280.515	1934.939	2047.723	1618.616	2716.658	2088.728	1747.058	2230.355
SYCN	237.6297	131.5025	77.62739	162.0836	77.91459	104.3128	65.98678	105.2062	154.6132
USP54	904.9714	3394.079	2596.792	2019.588	1338.298	1610.994	1552.323	1238.059	3128.977
SPATA5	158.6943	63.45745	67.68299	90.85355	50.5109	104.3085	66.44131	57.49883	170.7335
HS2ST1	51.46078	43.34764	89.44475	34.86437	176.1995	203.8799	167.9227	151.1225	50.89442
GOLSYN	1116.908	35.09145	322.0472	37.08035	116.0047	334.0055	218.0178	34.58418	41.72602
MNDA	65.78455	50.89827	654.8185	88.90194	89.3619	91.54145	329.0421	210.3264	1551.156
PIK3C2G	55.53492	64.07132	145.8084	39.81689	161.7245	73.04012	41.93018	53.58938	69.16323
TACSTD2	16527.86	15699.01	22089.29	22139.35	24513.7	23564.71	23970.29	24483.22	12940.15
ATG9A	489.3073	274.7148	1302.742	1694.875	1643.276	1426.591	1939.775	3114.932	611.8305
TMEM70	69.82275	44.58452	125.9667	53.8185	157.6001	62.56571	176.328	134.393	47.49641
PIPOX	82.45662	59.60824	45.59552	52.48294	47.55011	49.48408	44.70931	55.63576	39.90484
CCDC121	403.8105	233.0736	103.0588	266.9894	110.41	188.0825	108.8795	128.1803	548
HIST1H2BC	77.8582	37.29479	244.3465	1013.714	34.14326	138.3147	180.6497	34.91581	38.19736
ABI1	100.2508	97.05908	50.96731	125.2971	65.69627	59.37094	59.30584	62.23619	80.47784
SIRT1	37.16627	46.65358	113.7267	42.52979	208.4459	240.5015	322.6631	40.00413	43.77212
SNORD43	60.93411	873.9387	1022.783	1368.061	196.3389	483.9245	400.6841	390.4261	1820.78
MPZL1	3030.646	1291.756	383.3605	1434.396	489.0672	39.48896	373.6685	48.50768	52.85735
C8orf45	49.67466	44.72898	42.04376	46.5388	35.34013	47.26021	34.59813	33.02962	36.67343
OR1N1	40.72139	56.84912	44.24534	88.65556	51.49295	51.46857	48.794	73.16624	61.39295
SI	72.37349	109.6721	64.23834	99.88042	64.66914	96.73827	54.40718	52.2944	126.092

MGAT2	82.72849	76.79362	197.2909	73.74708	75.88551	75.8142	66.84839	44.30954	2330.64
LSAMP	262.8688	157.4596	491.0422	182.5571	650.5577	557.4597	259.3241	199.3166	307.9959
OR9A4	486.0013	264.9534	134.8015	294.0965	118.0159	213.086	115.1003	153.2067	626.9303
DOPEY1	104.645	64.26141	1035.831	75.84436	332.1053	534.752	492.1961	296.0995	44.75377
CALML6	80.33967	784.842	38.23048	107.1383	178.1774	82.83661	83.27266	38.71652	931.011
OR2T10	32.0934	33.38156	32.18791	39.90005	46.25787	38.1869	33.29176	48.75788	53.09478
TM4SF18	1787.636	33.97349	41.54922	46.91193	165.0715	58.52165	41.25468	206.694	55.98254
TBXA2R	57.03166	74.68428	45.40081	38.03476	40.20556	47.00057	32.77743	45.13611	35.22501
C20orf79	226.8279	57.06847	51.56905	110.3263	57.50405	63.4401	49.02116	57.62759	260.5269
FAM39DP	837.6544	380.6722	294.1794	41.05322	763.3652	52.09905	264.0525	194.1777	1950.858
NSL1	4716.447	5430.158	4971.484	6383.537	4105.632	4601.464	4384.967	4107.736	5997.8
TMPRSS5	56.88796	81.38257	49.44669	70.81815	169.0539	39.44324	112.3989	49.1642	57.71325
OMG	36.83123	74.85695	46.85044	52.8229	42.94827	51.03891	77.43411	39.30166	36.76591
ZNF668	71.78204	109.6041	1177.084	1993.874	868.989	85.97156	2115.135	2421.084	136.1104
L1CAM	51.52795	101.6102	62.65494	95.64185	98.96114	68.3414	52.27596	49.12291	108.3212
CHAC1	160.7465	86.62164	65.07714	63.32757	555.2999	82.14192	187.0957	228.2032	127.8031
BAHD1	334.6405	161.325	92.40974	73.65002	98.04931	149.7332	72.79376	108.4061	255.8967
EIF3E	15922.81	15926.18	16042.19	17022.47	15831.75	15156.32	14376.11	17044.24	18635.2
UBE4A	81.60242	86.34714	464.1104	33.59562	412.0044	67.9375	306.6962	201.007	74.92793
PDHB	3584.161	2873.225	3511.214	2789.472	5093.581	3435.986	3811.612	3223.007	1403.326
HTR3C	34.39291	75.12262	61.15133	61.83958	188.9861	408.5479	54.35305	68.52357	78.20355
SLC6A5	138.2153	70.31079	52.69829	71.9949	46.26658	43.46534	44.1856	68.04922	47.53816
GALNT4	187.2648	147.2027	74.75414	171.4776	85.65958	448.6761	217.5015	87.23026	2278.756
C11orf48	1824.446	1273.195	1103.106	1747.555	388.0578	831.4045	1129.599	1293.304	38.74215
TBR1	51.86342	33.25272	41.76591	37.42271	50.85543	40.90258	43.25265	56.61305	34.97647
ZNF498	328.2383	174.2741	75.17712	135.3604	94.16925	118.9682	88.97209	95.88368	332.1209
NKAIN1	46.75135	56.09663	51.0879	52.92814	54.39086	42.04266	59.87327	39.72714	61.5566
LACRT	42.18184	37.29094	37.90025	55.22886	47.24465	90.46802	34.97884	50.59586	70.41135
NLRP13	57.02227	60.93668	59.98833	53.0072	37.92253	47.26051	40.05979	35.87976	46.3191
SELE	364.5149	436.0755	106.9394	211.5805	135.5434	150.8849	127.6466	117.4566	508.9608
GLMN	258.7066	152.9091	1052.897	390.7821	1076.333	1267.615	1323.698	840.4692	74.23506
CCR8	586.332	59.50315	115.0651	284.1783	42.65616	38.42167	44.87791	66.08224	84.53925
RN7SK	14361.97	15022.63	8505.986	13013.3	6330.844	8389.572	5900.687	6814.979	17753.09
IFIT2	94.8765	378.126	1218.264	447.8142	276.9524	1887.264	4680.974	504.6386	42.57879
HS6ST2	66.45451	117.5725	104.3741	69.51935	94.95097	50.61121	47.48344	68.12504	40.86727
GANAB	394.5724	217.3915	124.3461	237.0489	119.2226	160.8068	113.328	107.3643	284.2893
NFE2L1	40.59268	2222.8	1214.869	48.03637	1657.653	516.5247	2238.743	1632.336	45.44264
USP34	110.9629	108.2226	232.029	1303.984	295.202	76.66563	105.4164	233.9601	79.49725
PRRX1	1494.335	52.2998	1046.131	60.57106	1821.56	776.453	1766.457	807.1475	79.73254
GTF2H5	1617.767	1111.436	448.4245	523.1871	362.6657	761.7581	355.5258	352.4069	1279.172
CASP14	43.64472	2152.097	45.3866	4422.416	37.40165	1054.171	182.5159	44.42721	46.85378
NLGN1	1233.243	404.6103	373.8318	160.673	870.5766	661.3825	350.9886	974.9885	289.0431
NMI	485.7811	209.2634	249.4817	247.9271	289.886	282.2154	422.8912	315.7585	455.067
FAM187B	46.17258	40.41169	40.81073	47.95119	38.35796	47.48737	33.21162	44.29406	68.66968
CYB5R2	81.55745	377.6628	433.5331	591.3203	1051.899	654.9453	660.4492	2092.309	1089.6
FEZ2	1618.616	1872.868	3490.751	1814.075	4933.214	4826.968	4494.301	4557.668	708.7493
ACOT11	9085.509	9850.193	11068.47	13310.46	7061.551	11142.88	8059.137	9033.937	11719.41

MSI1	57.29485	52.26298	57.50306	47.01714	182.2904	54.33653	185.2061	46.76873	71.70804
LYPD3	1002.411	713.6265	1754.737	741.2382	2607.096	2525.562	1674.901	806.6848	443.2995
ZNF408	1590.951	251.3862	135.237	270.4198	749.2538	209.1058	1247.506	1277.059	639.9132
PPP1R12A	45.59456	50.95959	112.364	76.93912	85.8176	173.3374	64.43555	88.54685	89.74917
FRMPD2L2	40.48616	47.25826	38.92329	56.04693	35.78299	47.88611	33.17682	43.54961	38.71758
DCHS1	442.4631	226.8262	117.2029	243.0684	144.996	187.3516	113.4716	138.9509	522.2204
ANLN	600.1092	323.1919	247.9111	1282.731	194.9154	281.9818	251.3293	144.3837	789.3942
TAS2R7	33.99649	35.05658	33.29446	33.3377	32.34554	38.11208	39.80255	33.01552	32.55583
ZDHHC6	247.5999	709.9582	1483.344	1433.038	1771.761	2302.218	3088.155	2749.581	410.5619
JAK3	132.463	74.20769	126.8707	74.02714	56.4301	214.6236	47.39867	126.9824	111.8139
LRRC6	45.54621	59.50793	122.1537	61.71402	183.036	50.3179	145.1661	68.35979	51.12185
NPFFR2	77.72318	51.53436	49.75419	45.60068	41.03442	39.98711	33.21268	36.82172	83.24792
SCUBE1	227.8357	50.8433	63.15051	44.46257	49.04781	73.46073	42.01809	50.09832	59.41396
JSRP1	15686.34	19738.46	8854.275	12586	7238.475	13211.79	10125.31	8407.255	19676.9
B3GALT6	1314.32	146.7704	398.1334	193.7456	233.0744	135.6577	448.0173	107.5334	375.8495
DKK3	290.1047	169.8831	93.39803	109.5256	117.455	141.216	76.61505	92.66737	500.5219
OR13C4	77.32254	104.6144	60.45852	95.87848	67.03174	74.65202	60.18608	44.72022	78.98506
CRSP9	1240.461	1096.844	676.2805	1479.943	673.8397	717.0789	786.3791	965.5086	1773.687
RHOG	597.7224	550.9589	1694.525	1150.266	1990.109	1115.323	1876.745	2193.251	835.2758
CD3E	497.524	249.5964	331.1345	155.8784	160.1228	262.4532	238.1872	161.5862	217.7039
C7orf30	212.1344	144.9239	1518.308	799.3876	2031.586	824.9554	2330.115	3053.681	218.6698
NDE1	1521.551	1295.181	1957.594	2395.241	2083.397	1669.668	1852.03	2065.446	887.7802
RAI1	82.04963	33.23471	45.7119	39.38663	102.682	47.41844	42.41297	35.1806	36.71983
PRKCG	232.0614	116.3741	87.56428	99.21169	73.73067	90.78061	70.46056	96.93227	170.8129
ZNF547	44.47043	43.19949	35.70162	35.68447	94.50378	43.31525	78.94431	35.12842	60.9219
EIF2S1	1584.439	1832.313	1368.338	1393.59	835.9763	1407.713	1321.563	1963.391	1098.035
DGAT2L6	49.14025	110.5621	47.65003	114.6126	47.19058	64.60636	40.5406	43.88519	98.5118
LRRC8A	75.83724	905.4688	3458.227	1857.1	8134.932	5789.011	7427.16	3957.592	4372.498
EIF3D	3810.872	7248.286	8913.804	9217.896	9630.065	6673.766	9992.108	10318.81	5453.915
C15orf29	2962.055	3466.282	3108.053	2802.814	2797.789	3014.27	3691.733	3302.908	3512.794
ADAMTS18	253.8131	150.0613	110.702	133.4529	85.83546	124.5371	80.77162	118.9263	312.3445
RAB3GAP1	5833.898	8848.418	11906.15	9718.998	13342	11455.12	12203.32	12399.76	2544.997
SDR9C7	174.0693	65.47283	63.85115	188.5669	62.82922	108.0802	58.44824	96.11818	144.5756
SLC41A3	1009.1	895.3069	1940.9	3560.971	3992.219	3364.646	3795.503	2697.476	33.00094
ZNF618	41.33056	322.4047	102.0994	32.56054	48.44021	43.23091	96.85024	69.56602	39.78133
FAM117B	11285.73	8155.326	9339.358	7292.038	8752.016	8567.47	6358.942	7902.298	7880.105
SBNO1	1585.706	804.5634	271.848	879.7756	225.7046	88.08968	177.1865	243.7308	252.183
VKORC1	12607.05	13771.25	17202.99	15113.89	19413.93	17847.04	17831.31	18867.94	14281.68
TBK1	116.9837	594.5698	572.855	530.3994	689.6504	553.7049	587.7522	237.1432	133.403
SERPINF2	74.15106	63.61012	2824.223	108.4195	1104.829	1110.867	2353.053	1789.233	47.83323
PCNX	19866.1	15218.06	17221.84	18696.52	14610.54	17146.08	15669.51	16548.18	19110.55
CD14	76.41005	87.02423	62.34368	51.42482	38.26762	42.89215	49.46016	50.06931	55.46557
PRB1	109.8316	113.3363	50.61403	77.69917	37.38915	52.15017	52.66326	60.82719	50.31734
NAALAD2	286.2602	289.9024	118.7163	125.9301	142.0218	137.0323	146.2451	101.6484	208.2407
OR51D1	103.8838	82.02175	67.87654	64.53413	60.65998	77.55526	56.2596	48.31567	162.7782
POU4F2	50.6086	62.05894	64.44563	41.22833	65.86071	57.96697	55.3681	86.52819	53.88165
TRIM16	321.9763	143.4239	78.18475	193.4974	102.8136	317.0009	88.81864	118.8999	401.285

ESR2	55.04002	69.55961	39.6396	40.40658	44.98307	55.84925	44.45097	51.3867	48.58517
MYOD1	38.67134	41.18393	48.10942	58.46383	39.94305	240.4636	91.1666	35.31451	49.35603
SLC16A12	220.1385	153.56	76.32386	97.15444	54.20326	105.9884	71.73312	49.17374	249.1437
ORC4L	273.7181	289.5968	127.0946	65.17118	116.0151	46.09386	78.62151	48.42664	52.70225
BEND4	305.2928	192.3134	107.2056	175.5583	106.1537	133.0173	69.22234	119.1101	477.7342
HOXC6	286.2747	191.7249	79.2485	130.6792	92.30884	111.3118	67.6841	60.10638	98.33755
GPR83	59.58346	64.76257	41.30822	51.75432	53.01758	44.7245	47.26566	67.39301	96.49013
COL9A3	620.9531	130.1809	85.88357	192.7928	68.53451	112.1918	64.14847	81.72814	404.5865
PLCG1	496.0395	296.8203	1532.808	553.5063	1489.5	1252.332	1213.394	1334.478	662.855
ANKRD26	74.27894	54.84611	45.43787	50.35508	51.15262	61.21791	135.0135	323.2236	52.50346
C14orf102	11453.35	12402.1	11672.89	10760.09	11758.42	10957.1	11812.32	9878.965	12374.95
TYRO3	74.47293	43.67453	1532.27	63.48176	4883.407	2291.25	1817.682	2587.677	51.20114
CHCHD10	46.95952	396.459	695.4371	37.09954	1699.722	53.1738	1021.639	803.2635	46.1401
DUSP11	313.9945	184.934	338.0751	223.4014	564.288	252.5511	181.3035	151.3328	461.5261
SNX3	72.2795	818.0283	3117.634	1167.993	4060.126	2228.116	3560.58	2580.461	52.13275
TPMT	833.4128	100.9532	2813.939	1338.659	3895.087	1460.584	2508.619	2491.007	2218.093
ELOVL7	41.66019	202.7219	129.3161	63.78513	248.9355	50.67338	68.40122	133.4339	65.25876
ERCC3	51.56598	47.41041	51.82955	40.52219	302.1409	44.5045	489.0741	40.86917	109.6598
HACE1	517.0381	275.3155	186.0048	301.3843	167.1849	233.9628	156.6234	168.3318	766.5792
UGT1A10	165.4182	52.95002	56.17255	69.68555	70.98086	67.50188	42.68002	64.87627	57.20633
GSTO2	10539.13	6336.808	11989.39	11349.01	10508.43	10701.33	14146.8	12797.77	7429.983
KRTAP20-2	45.74821	51.75149	36.29445	36.9131	42.26925	44.64521	37.89023	40.03828	47.08753
POLQ	518.5528	762.8951	321.4204	405.2937	306.8073	795.5508	419.7518	441.6593	50.24582
PANK1	57.1175	34.75197	42.51561	38.10183	36.90853	39.56469	37.6012	38.00747	40.06281
MAP1D	146.3048	107.2984	68.51578	76.34222	73.39372	75.36147	62.85513	69.78637	71.05309
IFI27L2	5304.303	7163.082	7461.498	7552.114	5913.686	7095.646	6374.266	10368.96	6492.508
LOC642782	48.626	96.37836	67.6502	113.9417	60.14744	80.59225	152.6971	75.57738	49.52029
ZNF473	100.4863	466.9891	57.9999	85.15386	58.13772	98.6696	55.11621	54.96428	201.7129
THYN1	32.47341	32.37942	102.6553	32.6763	37.8223	40.51668	53.46148	32.76585	38.53942
KIAA0174	2588.348	2382.539	3539.855	2692.663	3924.942	3913.132	3067.305	3461.721	1617.228
NRL	56.48862	68.63005	45.62727	78.33153	38.60525	53.90578	38.89386	53.54806	42.42775
CEP164	175.6934	846.072	1025.067	757.209	1259.603	1442.54	887.9014	925.9064	338.6817
RAVER1	212.8715	292.376	258.6186	299.6849	417.5218	217.4162	694.5694	553.4774	344.5101
HAO2	1957.498	645.1186	37.02879	44.12501	43.27716	360.5789	46.43906	38.39098	2157.499
CHSY3	1559.929	465.4482	947.5779	483.9156	1092.96	1390.512	1288.662	739.4727	64.96195
OR8G5	42.19156	91.21989	58.99006	79.47272	50.93683	60.49566	51.59621	61.48645	124.8265
USP51	40.49449	44.35463	52.82095	58.60028	36.05096	49.97363	45.77739	42.77903	55.51556
CYP27B1	40.37581	52.52877	118.7914	1211.707	279.4984	679.8126	902.1855	293.1015	44.94756
CATSPER4	94.37795	62.49981	66.13514	60.71258	58.04218	65.94193	61.13461	58.03385	147.3596
RASD1	128.586	134.2558	158.9513	151.4447	136.2679	84.92347	205.2908	94.01254	334.6432
FTL	4972.798	3821.214	2019.325	4687.516	482.5359	2145.449	1490.767	2276.244	4205.824
SLC2A4RG	2934.519	3559.568	3212.564	2385.905	4143.764	3413.542	3372.167	3205.856	357.8086
CCRL1	44.15861	56.31055	39.36758	47.02462	50.41042	53.76603	45.80554	38.08005	972.9323
CYB5R3	979.265	1171.918	632.0817	1246.418	731.3919	379.2242	703.7798	280.5366	2609.597
LOC220416	37.13018	40.51215	46.07676	45.32026	56.3452	49.75759	42.48984	39.99813	80.76173
DLGAP1	54.11748	36.52903	36.65908	49.15009	39.6109	65.00367	42.77849	43.82138	46.11641
FAM46A	11776.34	16500.97	17210.72	14511.73	13044.07	16822.78	16774.62	13912.8	12844.56

FABP2	66.70634	77.15745	47.99097	95.60334	56.80167	60.28658	51.8705	63.2879	254.633
ZFP82	34.20125	61.95761	243.7244	49.48189	61.54994	278.1902	225.0084	199.7815	59.16246
PCDHA11	154.7696	73.91029	70.85857	116.9427	61.97102	81.55277	68.60279	78.64886	112.5106
TSPYL4	44.98134	693.2081	574.746	775.4911	45.23571	636.4764	398.1682	418.7782	151.3167
CLEC4GP1	41.38697	1727.777	1162.492	43.00832	1497.707	55.4563	637.7661	1479.038	47.75968
SGMS1	189.3349	161.5166	42.20736	140.3708	54.88491	59.69025	49.39125	46.93503	173.7519
KCNMA1	82.89408	38.61345	65.88532	57.58542	52.53381	61.44459	100.9745	49.52103	167.3484
KTN1	33.30608	36.24283	53.50346	34.34447	419.6564	35.01565	325.5042	887.147	37.69256
NEU3	61.34662	50.11715	52.09563	745.2554	52.22101	52.7615	251.9334	372.585	45.80743
ASB15	65.0477	287.6342	60.79807	57.65693	54.37211	84.83799	43.90464	59.03682	77.56165
SNAP25	7065.844	6904.94	4934.939	5743.037	3081.746	5824.955	4030.411	4924.979	8328.882
F7	2872.05	1046.678	755.0314	956.6802	799.6343	2038.281	703.5812	855.8424	1937.356
TCN2	58.58339	43.64387	57.80734	60.772	76.89645	47.43487	82.2074	58.94962	98.0221
NOD3	90.54923	448.913	117.0301	96.86411	83.33548	69.14949	54.40317	68.57391	234.2221
UMOD	313.8136	137.7972	98.92304	185.4182	109.7362	128.5536	75.77677	93.97879	357.7049
PLA2G2A	12631.56	9984.549	4968.742	4097.192	9373.759	5648.437	7072.326	10304.98	10786.9
RPS11	2757.771	2697.023	1149.877	2554.169	1370.187	1161.757	1338.828	884.4266	2444.554
CDKN2D	42.97931	44.33621	61.5102	35.59998	59.70108	69.77147	47.94455	50.49103	50.06786
RAB11FIP5	4228.671	3601.015	5243.739	6873.524	7394.525	5660.455	7148.106	6565.89	3091.905
RTKN2	33.5991	35.28923	201.3982	300.7363	33.33152	35.57403	63.62229	40.16533	35.38216
TRPM3	46.5082	34.10603	34.81706	40.61842	49.60431	51.42012	36.83107	58.53698	57.50165
PCDH8	57.13149	70.03259	42.06903	51.28062	43.55291	41.01883	37.98643	47.06772	80.206
TIAM2	86.42757	69.03146	61.58765	98.34841	49.26401	63.84324	43.03339	66.96974	140.5452
PRF1	40.36195	46.26242	307.9916	56.63702	44.6394	38.64214	91.04058	209.18	46.68891
GRIN2D	60.00106	89.95212	121.2262	66.55746	44.05208	56.8444	46.68944	52.48368	915.9905
ATOH1	98.96095	118.3237	57.52016	69.64385	62.08035	61.02216	62.0935	60.59866	55.92519
SLC22A25	77.90173	42.89744	43.76488	56.32607	55.42831	53.37911	96.73891	47.24593	31.86992
PMP2	38.28997	38.24104	241.0661	50.39949	892.3232	56.80246	234.3584	59.8502	58.47172
RBM26	344.6062	212.7021	154.289	327.8665	131.0617	257.5519	162.5105	109.5391	75.60888
ADH1C	49.45692	43.82146	38.25364	53.27379	45.29994	56.42072	38.59536	43.76986	50.42921
SMTNL2	69.66689	48.21901	114.734	48.46245	79.14402	54.04987	95.96999	245.1841	53.85582
DCPS	85.0724	42.0747	39.74256	66.22282	60.00594	49.2275	45.36791	52.27144	167.444
UROD	48.14529	463.3812	1555.182	51.0411	2080.236	1373.57	2643.914	2714.696	68.96844
RPL7A	393.9848	238.6071	128.6419	249.8785	128.3967	184.2035	130.429	138.5728	504.5066
AGPAT6	350.2866	218.5412	312.2797	757.118	196.9657	162.5642	240.3231	128.1047	447.9865
MAMDC4	51.96789	67.78707	945.2241	2453.553	827.4707	987.0573	681.0292	1138.912	46.48234
GRB7	55.83925	90.19584	1376.986	119.4465	2368.881	1179.573	2369.181	1724.446	56.30534
CHD3	219.8213	52.71365	84.72185	63.87633	84.71215	129.6451	76.25732	86.46445	67.91972
APBB3	962.4763	517.0317	1334.688	744.7487	1607.438	1507.519	1660.382	1030.391	1072.658
OR5B12	130.9422	39.73839	43.92209	39.84706	44.65535	46.06908	34.59089	35.25597	38.04277
KRT37	328.9835	195.1935	111.7957	216.4073	178.2326	130.714	81.57807	112.0849	274.279
OR4C11	94.32747	101.4154	63.08624	45.82222	59.66739	72.11086	58.34467	61.98381	103.0075
CERKL	34.01418	37.90821	35.65728	43.49481	32.44446	61.91782	31.00266	35.13822	40.30299
SALL2	87.71491	68.7718	223.4642	56.55998	501.9084	45.26818	236.4352	309.4867	45.86979
PCDHB15	37.0433	43.98833	41.17192	99.68137	49.43594	31.91658	39.15306	41.67069	54.9795
DTWD1	620.0899	56.993	151.1139	36.9933	76.30744	194.9717	105.6077	190.2106	61.56743
TREML2	213.3567	107.4345	72.15859	120.08	61.49683	85.7157	107.4117	55.35786	129.1619

FGF5	47.42324	88.2577	60.95944	370.3103	60.21357	66.41457	90.72564	84.49972	121.2866
FPR2	56.5689	54.45648	60.76808	77.3175	56.46295	61.51252	49.97533	36.7796	47.57702
C14orf148	82.25038	49.35899	45.22661	46.15508	44.88176	39.59098	39.85995	55.97991	74.63772
C7orf49	5365.283	4160.402	6770.292	3251.998	5849.179	4965.541	5177.183	7294.894	3564.52
SPATA3	214.8996	145.9626	92.43173	160.9073	88.23708	126.8854	69.67072	101.8971	252.9523
SCNN1B	241.9241	158.7825	264.0953	153.0224	1118.558	111.2272	121.9011	260.0461	98.1269
ACADSB	67.55426	267.1092	158.9316	73.44408	95.73354	244.4361	113.8219	144.2213	123.8005
C20orf54	68.31348	125.9488	49.34613	51.86171	172.977	53.29498	194.2715	422.6111	176.987
LYRM4	90.34091	53.56616	513.8499	42.36472	569.0948	815.3925	626.7618	572.7272	55.97997
APOE	32.52862	43.55562	831.8428	50.32304	175.4033	34.14046	46.89894	464.9217	57.01338
LRRC19	100.4492	95.19227	65.58261	64.88972	58.11146	75.98193	60.48542	82.4389	148.9141
MACROD1	39.04141	43.48858	45.56819	39.55605	209.2802	44.28301	131.9917	199.2678	43.89981
NONO	5831.894	4539.587	3752.055	5094.016	2174.621	3200.739	2134.63	2788.116	6095.342
GPX6	116.6276	94.92933	64.65688	146.2462	66.00647	101.0665	61.83144	74.91051	243.3289
CA3	71.89587	47.47297	111.6421	53.20069	94.85429	221.4008	178.2456	113.118	76.10516
ANXA5	45.08454	484.4931	49.57655	59.83301	443.5217	40.79864	621.3748	207.1232	47.26797
RG9MTD2	168.2658	232.8694	97.86588	112.3906	79.80434	73.44276	85.2156	61.93257	60.87394
SGCA	167.5686	1168.777	667.1714	142.2715	806.2521	533.4117	1929.893	4474.946	149.7191
SLC28A3	50.83372	56.77157	38.07692	58.14291	334.2975	49.45345	121.6691	82.91237	43.6926
CH25H	99.03571	44.33768	285.8391	326.1858	59.57789	374.4352	119.7344	166.892	89.83289
HERC3	113.5767	58.70596	74.4554	68.95438	61.10496	71.92795	51.22017	53.44979	46.87672
SLC5A6	1757.579	892.514	2603.083	956.5103	3011.829	1807.96	2508.416	2325.915	2428.081
NUP210L	84.41319	58.00171	38.26954	53.64075	49.32999	64.91426	36.96807	38.68586	49.52737
CXXC6	81.55789	505.1645	41.9571	569.3825	42.95536	36.90165	36.58322	283.095	216.5956
SCN1A	39.45972	65.91327	52.25685	33.6114	51.32061	44.40998	49.0256	41.66219	40.00055
FDFT1	908.8094	1256.798	864.4291	1466.49	2174.402	441.8822	770.1879	950.8213	1225.965
CHRNB1	32.94493	1146.86	287.4774	569.1838	399.7024	479.0674	647.1349	430.3857	50.71156
TXK	67.91784	41.67354	42.68006	48.72411	35.87597	181.3547	42.20111	44.65569	43.71148
ZNF41	186.8107	95.42747	62.17935	715.3392	57.76708	49.41674	60.48346	75.70708	182.5604
ARMCX5	965.782	82.36394	323.7886	121.2978	156.4996	108.0046	159.1256	377.2881	272.7922
FLJ43980	45.6283	48.09794	48.68405	43.15614	43.5757	42.87995	39.74972	66.52048	45.49887
MAP3K8	48.56681	110.8361	414.416	49.59169	377.5175	768.062	433.4205	479.8856	75.047
TM2D2	37.31179	452.0158	455.6157	36.59142	429.0111	228.0818	290.7396	324.2121	49.22244
RAB11FIP2	53.79937	76.7124	48.58727	382.0494	50.50329	67.29395	76.30653	51.27667	37.03438
CHMP2B	492.9268	278.5965	622.9205	828.7198	1020.403	351.0545	809.8293	201.1789	589.3267
HEXDC	1707.447	2364.945	1717.502	1321.587	906.0891	1916.825	1096.65	2259.633	2339.503
RFX5	92.87217	730.4703	1832.214	41.03277	736.6786	1974.151	1384.831	710.5676	3187.394
KLHDC2	3832.575	4439.092	6329.742	2779.319	7380.425	7592.88	7672.406	6072.895	886.8958
NXPH4	44.48225	60.75541	50.3975	64.219	77.34603	182.4056	44.13287	45.26431	40.5986
PRR8	2264.487	1960.068	938.9768	1214.709	1111.402	482.3437	589.452	999.2231	2171.942
UBR5	7597.234	10300.87	10620.06	11946.43	8123.944	8064.376	9614.104	10169.43	8941.052
MDN1	54.12267	47.92316	40.17056	49.64169	54.05059	60.38952	46.45861	50.38571	52.93639
MRPS33	1488.348	1770.446	1664.29	462.9581	1591.127	2067.189	1363.905	1909.613	2015.553
CRYBA2	60.15783	63.81603	55.31164	97.63531	82.21077	60.35863	108.9259	171.8868	38.72548
RNF175	78.90807	67.74458	287.6208	83.49361	407.9976	95.14915	236.8376	379.8758	320.4641
C22orf30	55.70749	38.17802	165.9257	59.90399	335.8864	457.371	162.3346	189.3924	43.9501
ZNF782	43.35983	319.9019	164.4379	299.2774	207.7662	211.0777	80.6424	91.42812	247.0125

MUDENG	200.7824	169.5112	299.6119	93.72182	840.426	1091.073	654.5756	892.2278	2202.677
IKZF4	3166.762	1869.725	1371.585	783.3066	1445.422	396.8492	1267.621	1889.262	3200.262
EPHB3	178.8629	439.7158	64.96665	145.5023	267.3951	88.81853	131.109	77.60376	148.5495
CEP70	1410.482	1162.526	791.7428	37.1038	1076.753	1853.675	825.1073	952.1108	34.27097
PRICKLE1	11016.8	10157.35	9026.021	6179.499	9866.517	12771.66	8757.109	9833.946	9691.842
TMEM8	1481.138	41.65709	33.85201	763.815	166.7474	349.5347	107.2379	36.64967	59.99694
B3GNT8	910.6456	453.7488	280.3419	477.4264	104.9356	155.1405	182.4143	257.6628	424.8365
RALGAPA1	113.5987	68.95226	127.7108	106.2298	231.2088	52.24292	128.5936	152.8863	117.3548
KIAA0406	455.4337	180.1093	205.4803	276.5993	240.2049	168.735	196.7981	161.8351	623.0081
MSC	2402.209	58.45199	52.80082	73.78431	39.96618	42.98498	50.24977	60.89083	50.44798
HOOK1	96.26105	63.81842	91.90831	169.3564	79.79043	76.62984	60.77981	70.64609	286.6147
AOC2	105.048	84.09731	79.96063	43.24035	61.36264	49.55589	53.92559	59.23163	114.9409
SLC35A1	2061.227	1995.185	2991.145	2742.354	3867.833	3463.058	2935.58	1931.666	1072.627
USP30	757.2711	1007.329	1273.5	1290.392	867.8685	800.5185	736.5109	1651.656	49.43567
HOOK3	566.5831	747.3876	1140.409	1604.552	1156.255	685.4042	980.1298	996.7108	1024.979
PTPRH	98.01102	61.8065	67.98199	76.29758	60.23009	81.57059	168.2501	69.04454	1988.979
C7orf47	1685.992	173.4468	88.95226	170.3174	165.1539	455.7298	180.9387	91.89467	346.6568
SMAD4	770.1481	2006.904	1640.909	2212.714	681.5907	1212.322	1221.664	1327.15	983.1337
HPS1	82.42558	959.4858	461.2199	1047.131	578.2849	837.5646	731.2631	1049.925	296.1129
ZNHIT1	1267.309	591.5073	551.0835	1614.034	871.7414	637.406	566.0844	710.4169	1855.949
SLC2A13	46.78644	38.32498	41.59253	55.84921	45.11091	39.31672	37.86718	60.0881	39.93562
KRT38	67.23573	37.35592	37.6818	53.72495	35.46046	46.19052	45.11345	38.34137	89.28085
RSHL1	72.45895	56.35289	39.06903	103.6415	50.4342	55.60551	36.67739	69.00335	53.75901
DCTN4	486.6786	1167.377	652.9875	548.6872	485.3711	997.5967	722.7877	1404.97	778.0452
BMP7	927.8237	717.7275	417.7268	291.033	654.7763	584.3734	467.2874	323.6591	804.7385
AKAP8	109.4909	50.3078	837.5411	415.6466	1100.132	386.486	1132.933	751.4666	36.15461
CNR2	68.46416	62.48038	38.87439	60.79196	54.7038	45.21568	47.28785	44.54832	182.8172
SFMBT1	145.0948	139.5988	366.0438	152.1532	382.6932	217.2893	223.5835	282.0373	105.8001
SLC27A6	188.3998	54.36644	46.25815	66.58892	57.67162	77.74924	57.88189	93.144	140.8031
CLCN6	837.8824	43.64594	419.2037	40.16279	839.76	351.8228	1057.642	632.8484	83.51749
MEMO1	495.2099	89.94709	103.5025	189.9787	112.2002	135.7854	97.70992	109.6526	266.2301
PFKFB1	1521.434	1309.832	517.2473	59.85511	408.8378	738.3231	789.3217	949.8411	49.90771
DBC1	44.59999	75.28393	144.7492	37.02049	390.3616	58.63868	443.1708	338.9072	105.1433
CSAG2	42.3839	42.35166	48.75332	37.62699	34.92794	42.04469	36.24588	47.94516	133.075
SERINC1	478.1694	260.1091	130.6422	194.2002	323.947	221.5825	309.2342	206.3752	64.72592
SCRT1	197.5911	126.5796	65.83565	116.904	73.19736	112.4544	62.11425	64.77919	268.4817
RAB3IL1	14926	14808.4	12458.11	9456.308	11092.24	12556.12	13273.92	11732.39	13032.17
ZNF675	73.12341	56.82138	95.78415	63.75233	86.36916	89.43248	73.2586	34.60274	60.62057
HLA-DQB2	6159.495	7422.025	11695.55	4330.381	4050.618	16812.6	4404.588	6979.927	6674.062
PPP1R9A	41.26036	40.49854	35.61484	58.04393	106.479	180.0833	97.55755	112.5644	50.91292
SMPD1	3253.509	2818.619	1918.156	1905.045	2110.184	2398.044	964.185	2177.278	3350.098
CPXM2	268.8408	172.3216	731.1353	160.265	753.7061	252.9334	497.5539	443.7708	319.6028
FOXL1	185.5929	73.40681	58.42499	115.9281	61.33541	58.30181	59.46964	50.73251	85.24935
DOPEY2	1184.797	597.6011	342.9805	64.79017	60.83299	633.0872	123.473	151.55	54.15779
TEX13A	270.5591	188.7795	58.79796	111.4464	70.17316	88.69848	59.90485	89.20701	377.4913
QPCT	329.721	221.0659	100.1033	205.8566	191.205	145.239	148.6028	373.9145	359.1897
DDX28	36.18275	486.2308	627.5208	513.9256	507.0078	614.7734	597.6017	537.7242	416.5407

DUSP28	2693.549	2803.814	2644.639	2630.408	2915.01	3003.74	2930.081	2712.517	48.33969
CDC7	151.7645	224.8777	143.1605	287.293	296.6694	241.4518	282.7505	254.0341	90.14659
KIAA1429	77.95531	39.73159	37.28834	50.65855	40.06714	296.4404	36.16454	45.33171	49.34698
RD3	309.6129	149.4718	100.456	146.6945	100.2926	141.6178	77.63716	120.2569	347.977
MMP10	8015.252	1174.568	2128.783	4234.859	50.98741	2681.524	12714.49	1894.28	34.54928
TTPA	62.03473	43.8801	36.12825	73.73045	37.16852	49.42343	40.66954	41.62119	77.96737
WDR47	68.22916	61.2328	40.60281	35.35041	50.63026	41.33504	120.9553	43.84648	160.6069
OSCAR	92.00558	85.80325	52.72776	138.1156	64.35529	91.67397	59.55602	66.27012	132.4024
OR9Q1	40.84792	37.59793	38.79227	41.77958	43.86117	45.08454	37.3405	51.99886	56.33414
STX16	1061.17	1592.905	2357.853	1641.276	1773.933	2252.524	2219.904	1564.116	779.5961
SNORD38B	406.5404	317.4428	161.985	216.3733	134.7533	206.9744	89.28185	131.2452	582.3184
SMYD3	65.27893	440.9551	644.6996	1062.207	927.273	977.2185	1024.026	940.9648	41.87266
RABEPK	7176.263	7081.981	6848.253	7323.554	7299.25	7486.012	7712.295	8324.963	7620.421
DGKG	86.93332	75.25584	43.07287	53.61369	252.1827	46.89425	53.8923	119.9176	129.8257
KIAA1161	346.3921	188.024	105.4373	397.7368	176.6011	210.1395	107.6812	157.6485	428.6938
SMARCAD1	45.84875	57.50279	127.8151	68.85015	55.73836	37.17739	66.06124	36.77567	90.83452
BBS10	1067.533	615.7811	195.546	801.9202	84.74791	218.6099	128.021	242.0982	1064.222
CD160	143.7056	535.3642	255.6908	121.9232	62.25014	745.0136	145.814	168.1645	92.31973
RHPN1	291.6304	172.3196	78.57341	80.18184	61.54965	92.01188	61.58121	50.61043	273.0605
C3orf30	83.14372	57.49538	43.28775	57.88597	53.81461	51.55001	48.13839	59.25965	57.25886
IFRD2	66.81583	63.21992	48.62672	46.28822	48.29528	41.67884	120.2903	273.766	181.7361
GDPD4	313.8789	190.7676	107.3546	181.1112	102.8161	150.4964	70.04311	107.6487	229.0226
GNAI1	869.541	700.1636	635.4196	2179.941	404.6522	968.1921	327.9388	585.3923	48.14167
VN1R1	34.23568	45.3377	39.28765	34.14511	32.04929	41.8791	36.62887	37.19849	54.41687
SERPINB9	693.621	456.3969	129.5641	331.4296	108.9449	221.62	124.3047	128.7088	202.751
RP1L1	127.6862	44.17418	41.21511	533.5665	148.3135	45.95536	148.4556	38.87484	47.44986
PRAMEF10	77.90968	37.29381	39.94806	54.54961	44.73206	75.26195	39.41098	42.92933	79.0745
ADCY1	45.18906	35.8891	62.11776	37.51971	42.57089	38.52025	34.21962	37.00887	43.08028
C8orf76	65.23727	43.65604	2865.377	43.53254	4644.011	2516.857	4574.878	4174.027	36.30138
NLN	223.4237	76.86837	63.95144	273.5771	86.03961	111.2532	67.96073	58.01262	144.4663
SSX9	35.92755	32.49057	34.64656	44.83335	38.04746	31.93637	38.28614	35.96695	34.19371
DNAJC19	37.47797	447.206	247.6137	40.60912	37.41313	44.50951	183.3834	184.3777	35.65216
RNASE12	35.9941	37.9676	36.84611	40.00406	38.33944	41.29923	36.72696	35.54966	33.57063
H2AFB1	47.68351	39.69619	39.56304	43.07049	37.69312	42.65083	32.62306	41.65485	49.2131
KIAA1128	593.0187	350.9421	55.90785	126.8245	117.1296	117.8288	76.17869	98.09758	189.8955
SPAG16	4709.757	3190.579	2282.019	1156.544	1365.821	2551.542	1450.914	862.2252	4345.605
HIST1H2AD	67.97291	314.7858	171.5111	880.8173	86.42744	183.8124	83.65719	211.0993	43.53746
ESPL1	110.396	75.78911	64.58441	59.96447	196.2944	84.92257	106.7526	281.9794	76.91294
UBL4A	113.3474	1052.013	3501.739	49.75949	4242.182	2602.908	3110.665	5381.909	43.48615
NRG2	38.98089	37.11453	36.41459	55.38536	37.31115	50.41366	43.98657	51.38811	46.38738
GRWD1	59.91871	54.59942	182.0024	51.61873	154.8013	67.00378	219.883	177.2801	36.24347
TUBA1C	2113.873	3754.888	2975.472	4448.958	2120.742	1706.181	2099.255	2885.541	1572.776
FNDC7	143.1739	66.65388	60.29633	50.49892	50.74448	34.33612	44.27524	47.93219	93.81228
MAN1A1	83.85483	275.8833	59.59626	51.49643	85.28242	51.11594	65.31484	66.97153	100.0732
GABRE	56.29876	45.24842	44.9541	36.41653	41.26298	66.28836	36.84916	52.25948	61.66375
DYDC1	35.94478	33.63889	36.99981	33.41156	32.93744	43.72303	34.87255	38.61634	56.73467
SET	75.99208	580.6613	722.902	364.1707	1481.5	775.8933	1363.148	1359.75	197.6263

CCNH	259.7057	121.6279	650.3846	154.925	193.7024	360.6594	154.5496	78.47496	280.6448
SSFA2	77.31227	70.62621	583.438	623.6223	645.2805	64.86926	431.0318	365.2124	48.06138
AZI1	3096.555	74.92253	1005.512	997.2981	357.9939	501.215	97.30086	371.9441	209.7482
REN	133.8412	108.1765	67.79609	118.5141	61.10532	89.24552	53.81258	63.41578	104.7989
DKFZP564(	603.079	279.8402	133.7809	324.3007	101.1669	60.86534	86.80224	117.6402	795.2175
ZNF620	61.52287	77.45947	76.27886	64.06691	54.151	83.31555	56.59296	68.68457	42.58073
RNF121	200.3893	711.4935	237.9191	804.2582	389.9388	422.8976	454.2176	552.4472	2033.355
C1orf9	3632.797	4861.383	4255.798	4232.733	3910.658	4737.156	5040.904	4889.792	4248.765
PLXNB1	37.59213	1056.874	5965.29	2678.035	8686.781	3020.014	7615.049	4883.736	43.34467
KCTD12	55.5213	84.58257	268.6544	101.6372	177.0037	45.477	156.2133	42.44157	68.51684
CA14	42.31056	39.08704	67.84875	81.68749	46.66973	45.36013	45.6924	39.3795	36.23539
TBX5	74.18389	36.41811	44.87349	33.22645	43.78727	38.35864	34.66935	36.43531	36.93512
CCDC52	87.06522	59.86876	240.4205	45.45112	39.48977	82.41478	34.52087	33.24077	112.0167
RIMS3	1369.301	1639.322	1812.097	2928.559	3038.042	1236.792	1390.632	1829.461	52.3978
FRMD1	37.54512	33.43956	47.41772	35.10874	41.09358	36.46724	47.78471	43.27076	34.16135
CEP350	87.29742	83.22207	150.7239	92.5129	161.6299	215.0888	67.58087	51.76835	99.75989
POLR1D	134.1808	83.56059	73.27795	85.38698	290.2343	110.0246	505.4435	173.9832	148.8548
COBRA1	817.1217	597.1748	191.533	198.8292	816.576	137.3754	428.7606	850.2778	376.1427
UGT2B10	42.09553	42.37219	38.18586	34.70613	37.60443	39.27425	45.51567	40.41658	40.05048
TAPBP	666.3008	344.7178	348.3024	335.6768	282.9588	192.4003	298.6108	353.3392	812.0918
OSTF1	61.55033	841.1641	1055.275	56.77174	1010.259	1033.168	956.9955	766.4476	69.14103
UAP1	478.2718	339.9039	357.9484	382.8033	442.4438	357.5585	434.3477	288.7433	43.88589
C6orf138	481.8493	651.1399	156.214	297.9767	156.1899	212.5196	151.7577	239.2723	649.1747
GRB2	67.90202	33.72839	47.54302	37.612	42.77065	32.95205	46.05725	43.89664	33.05084
COL4A5	446.5114	317.9318	778.7554	1605.952	1284.721	2162.438	2644.573	768.3593	252.6904
ZNF518A	394.8835	221.1956	129.2115	234.6782	116.75	157.1171	156.0408	108.0551	717.3149
MEF2C	34.28715	768.7429	522.7946	38.4854	711.722	657.0239	1273.201	705.7924	45.61171
GFI1B	228.2215	95.61894	54.27216	118.2051	63.80808	76.87296	55.48152	67.40063	63.96597
MLNR	58.69044	94.03041	43.64405	45.54538	40.43068	50.38237	46.32417	65.90198	63.76101
PAPD1	331.6106	174.1171	376.3512	161.1185	354.0017	398.759	340.5897	246.4549	198.7396
GTPBP4	5120.772	4439.664	5902.18	4273.982	5096.586	5855.89	4735.74	4870.338	3950.914
MKLN1	50.5715	47.62518	2444.648	1509.807	5403.616	3393.071	2578.157	3239.755	1810.301
TMPRSS11I	110.517	134.9093	261.5468	94.85458	113.4023	173.1762	83.22164	169.0474	162.8216
CR1	37.52624	40.25618	40.7639	36.12457	33.7349	35.52311	34.05199	136.0345	36.14086
C1orf52	2541.013	3381.458	3457.893	3707.094	2853.403	3818.22	3186.493	3108.639	2776.535
XAF1	21999.81	26273.17	25928.02	24490.4	15659.08	25111.4	23934.95	24178.81	20819.95
LGSN	38.33963	39.91789	40.836	35.22472	37.13278	36.61152	31.88758	36.17466	37.05653
HOXB13	34.21315	39.13064	37.2969	34.19827	36.90879	41.17344	39.25216	35.30189	35.99635
SLC39A11	44.7442	54.2739	50.56093	56.71432	183.6593	40.35285	111.1516	44.04664	46.62219
SLC35A2	153.1549	161.9976	76.16004	159.1111	95.25766	100.4844	82.86234	96.17461	382.369
C1orf190	1956.496	79.02569	70.15436	90.99751	574.4985	50.18065	219.0342	265.863	201.0769
OR8D1	46.15247	63.34071	58.52956	63.80544	48.58722	54.58467	48.66748	63.26736	45.52534
UPP1	139.7905	98.28614	61.2077	82.21091	68.23311	73.99732	59.16632	58.30975	195.2629
ZNRF1	174.7132	125.325	97.6431	137.2956	80.78693	115.853	65.29947	85.27994	314.5648
CENPL	76.39558	113.3682	55.26753	320.772	41.93826	53.42637	67.83839	66.91782	77.06952
KIRREL	161.6631	1034.886	64.1968	91.31306	59.75132	67.4031	232.6308	310.9119	147.7099
BAT4	150.0096	54.37202	50.28023	56.07454	354.8737	68.37312	336.9982	338.6674	81.58376

BHLHB4	40.09283	49.89254	48.24438	36.89858	46.06663	34.34103	36.02882	39.14748	36.288
LPXN	2254.214	2027.039	4207.445	4223.061	1538.097	4071.42	5326.733	2969.581	365.1985
SFRS10	5301.589	5644.583	3370.765	4749.506	2465.373	4832.15	2665.375	2681.021	5791.263
TMEM167/	687.652	390.238	323.4413	312.5138	251.619	474.7584	170.4939	388.7592	260.3823
SPATA13	75.56093	83.27982	285.2803	40.54698	54.6745	78.47385	51.20161	290.2217	104.5115
EXOC4	770.3058	608.4819	744.3726	442.2092	610.7565	490.8946	653.8447	792.512	1070.717
DIRAS2	160.4393	81.80753	70.37574	92.65186	49.63536	81.60268	54.28914	61.5513	71.55302
F2R	1476.354	1929.827	1463.154	35.55736	604.6622	861.3407	1000.476	631.5156	1601.036
UBA2	851.246	390.9954	836.6392	673.7931	691.6255	256.3467	429.2838	616.6508	252.2334
SYT1	67.55748	65.39031	39.07276	40.35804	147.8305	43.5573	47.15483	38.08328	38.9924
WDR77	55.83331	49.20947	57.7543	53.58662	39.75798	58.04251	56.28771	72.95674	62.96729
NXNL1	124.5704	63.54046	38.6067	51.28577	47.73943	67.39741	120.4203	164.5638	884.0188
ZNF584	37.66273	43.71898	185.7008	45.67419	239.249	52.14527	168.1435	33.98146	32.42432
HESX1	46.79809	54.59053	41.14493	42.37848	37.05153	36.1547	43.5492	85.97151	42.10718
ZNF33B	189.8907	282.0029	138.5033	271.0575	123.3728	187.8212	85.41219	142.3544	548.6586
SEC14L4	57.31708	48.12698	50.96714	62.0136	66.81251	47.87309	128.9716	35.89701	1065.057
CLEC6A	136.0717	93.35624	83.60964	97.91739	74.88395	158.1683	66.30271	79.59089	160.5069
LOC44117	55.48405	51.00234	41.01046	60.46554	42.6813	41.04013	38.01663	46.78591	63.68702
ZFP30	241.4103	225.3275	114.6086	230.0529	184.3712	267.5432	456.7166	159.9406	316.1289
IPF1	120.1813	99.62335	61.58257	73.00286	51.34188	64.17593	53.19955	90.63927	154.5167
CAMKK2	248.7529	278.0244	81.87162	172.2206	99.32511	144.0127	84.25179	149.092	186.7799
CTSS	2752.826	1106.002	2584.453	1186.742	1175.578	1551.783	1560.229	1362.456	2788.092
SERPINA13	50.72112	45.74263	49.94684	82.40059	33.67086	56.12245	45.31825	39.63416	47.95853
PWP1	51.83564	79.51205	567.6181	189.3163	1487.21	753.1064	1588.823	1189.417	1613.177
ZNF701	47.86345	51.96417	235.0272	326.9161	128.1693	173.7739	143.8688	137.2246	31.88316
MGC20983	52.02428	73.44215	52.97545	57.6595	50.08868	46.56814	46.4549	36.78245	76.96569
TNFRSF18	74.01479	47.03092	43.48975	52.77595	43.33337	56.95935	51.96075	45.09402	94.1984
SH3PXD2A	1573.829	2850.37	3871.314	5009.671	4980.121	5236.05	5539.738	4553.423	2310.366
RBM4B	41.40782	55.39273	595.1329	49.28411	2258.501	1991.599	2222.651	829.4401	35.71297
PTGFRN	108.7724	66.39394	313.7068	694.0245	140.9131	46.39701	249.0151	224.994	98.11706
TOX4	392.2536	218.085	115.4562	189.3087	117.3649	167.0727	121.3769	146.9201	488.6195
BAT2D1	78.92216	41.73316	158.8098	667.1342	58.40738	261.172	151.2567	146.9593	62.92432
LASS3	4624.81	4902.839	4991.438	4666.62	5803.478	6009.964	3969.821	5839.159	3564.694
ERAP2	346.4167	836.0013	869.3472	174.9101	196.4713	1147.713	321.2853	259.7635	339.0937
C6orf120	34.00504	33.0524	51.72202	35.92395	35.23927	34.53321	33.95436	41.62294	41.17356
EEA1	893.1926	41.88511	161.0986	51.59356	440.7911	59.27038	391.5262	331.0602	56.63548
EGR2	82.78603	83.87895	65.31471	63.66915	63.89315	69.12032	66.65026	53.00437	109.5465
OR51G2	48.64669	39.18143	39.89091	58.80538	40.72141	33.42943	39.32274	37.6322	50.69596
DUSP8	67.69956	1408.985	287.0989	872.1774	994.1052	67.80704	516.5261	388.5876	112.4804
NEDD1	123.9946	81.46166	73.3999	788.3594	258.2161	582.972	115.7196	353.2271	1207.495
CA11	49.87472	286.3167	174.91	72.495	148.5639	64.13299	153.7988	40.91905	40.02314
OR52I1	55.14803	47.71602	43.35646	38.44023	35.77166	45.10612	45.31684	35.70194	52.94571
MICALCL	35.02211	167.2947	625.0032	146.7949	1039.173	71.6202	643.9341	319.1245	93.16501
GP9	45.8192	392.7987	41.02848	56.25281	79.49393	93.61668	81.84554	45.45827	50.33864
LCE5A	962.6063	95.60159	65.69972	85.86635	141.4441	68.90364	105.9307	255.6144	83.06899
PXMP3	57.86176	40.83436	35.1768	47.92924	32.49307	38.25679	135.5625	36.6479	44.24062
TTC1	60.10918	418.5119	532.9746	36.1863	825.6854	434.5178	1140.786	1112.279	48.30451

FAM109A	259.1938	107.9068	101.5348	121.7213	159.5409	132.9925	81.12421	74.86346	209.7695
PAFAH1B2	91.34609	56.02412	111.0265	327.9723	98.69865	54.78919	117.6706	150.7171	58.33436
RRH	35.33484	37.04678	36.28547	44.26739	34.11342	31.90025	36.32281	45.47932	33.78416
ADIG	177.9614	165.8642	55.93043	140.9984	74.22906	104.9121	62.99488	70.2894	285.8193
CHST14	1344.721	290.1801	1038.07	319.2259	2680.965	2042.703	2426.037	1478.524	1127.778
AKT2	605.9822	1160.335	812.6891	2325.823	676.9721	629.8769	103.7743	1253.261	118.0601
TBCC	40.70257	49.84152	124.9484	71.73877	39.75437	46.60768	53.47618	33.55029	40.2808
XPNPEP2	187.2751	373.2172	297.1636	311.8789	274.5678	235.3296	263.488	442.2718	893.9585
FZD4	59.1753	58.3779	39.57707	58.67747	434.3471	51.52563	784.9898	1062.267	67.2915
ABHD1	157.994	78.84226	54.55584	74.81258	75.61012	90.23974	57.6966	63.84798	136.1773
DLL1	917.3428	81.33659	179.191	487.247	428.5445	46.76834	477.4094	131.5393	41.454
HNF4G	343.8526	220.7626	94.74104	209.8273	49.87405	138.2715	63.26302	87.85101	544.527
ACOX3	53.1677	44.10662	37.26558	48.50569	41.15429	43.06867	36.46616	37.24983	52.50765
PIGW	442.7152	260.0085	113.5233	246.2975	102.7161	189.7774	157.5199	161.4661	510.9076
SIX4	106.6803	2120.096	388.2641	761.3965	743.8303	961.8741	1630.729	1283.732	64.8492
SIAE	40.38439	52.25237	150.2032	48.37893	32.68512	46.17521	37.93998	46.98523	43.47072
RCOR1	51.84465	37.90839	45.95251	39.12875	174.1043	35.25261	115.8806	137.3906	1150.07
DGUOK	12937.42	13442.93	14780.35	15972.18	14606.77	17107.18	17649.25	14875.54	11817.35
FAM107A	1031.275	1012.248	1276.46	400.2242	2006.664	980.326	965.2227	859.2713	56.87571
ZCCHC14	42.53899	34.44979	285.5879	1609.154	241.4111	32.10679	183.237	43.18118	48.52903
TSPAN16	46.344	43.95476	49.16116	37.62067	32.4217	48.97407	41.81326	40.56554	90.01266
POLDIP2	2139.586	3035.672	1565.77	2313.643	1678.143	3357.35	2823.024	1514.015	4234.588
VPS24	1260.648	1109.816	755.8251	1961.542	1013.158	1068.601	746.3581	714.812	1213.753
PIP5K3	36.70919	42.39191	345.328	1111.731	230.4734	32.01501	428.9956	371.294	47.16161
MLLT10	51.55719	54.53961	407.3772	894.2167	733.4462	494.6228	389.5882	257.1019	75.72047
CEACAM3	40.03211	63.0845	52.30301	50.28232	41.27468	50.73273	50.96896	37.25253	61.92167
NUDT22	492.8449	509.0264	324.9195	309.7472	392.6782	257.2899	354.7758	302.5855	1482.808
C17orf69	240.0246	122.8421	414.1664	613.3056	522.1152	487.8178	554.4725	671.6968	132.9369
ALG14	88.24956	72.76456	53.38759	42.49196	49.84331	50.47451	199.2705	47.85379	44.87418
ZXDC	3815.314	4399.131	4397.075	3658.982	4305.507	5330.964	5031.34	2872.584	1803.12
VASP	382.3977	180.1018	108.498	194.3226	125.8297	186.065	90.65561	149.782	521.7594
OR2A25	98.26865	134.6234	93.22788	151.8646	80.83922	112.7157	64.22655	68.22771	277.3381
TMED9	1173.526	211.3858	723.0394	659.1354	514.8962	156.5376	847.8509	1643.661	430.8533
KBTBD3	159.8607	165.0516	170.8675	437.3289	60.02126	94.7409	68.44363	60.92142	108.6842
POMT1	395.5761	878.9566	2599.589	346.2813	2114.047	2333.88	2627.908	3371.555	2606.652
HNRNPU	5080.65	4802.707	4221.914	4892.685	4222.12	4559.416	2647.123	4720.276	7394.593
ACOXL	44.66422	45.11863	36.14238	57.00623	48.19556	56.00295	41.96157	49.37624	50.87279
CARD10	561.1845	49.65913	112.2422	434.688	312.2961	39.69931	257.6956	145.8388	265.2139
FLG2	60.86961	56.68183	49.41421	546.035	55.28176	233.6292	43.00119	56.38232	41.90392
RUSC1	44.48161	80.64616	105.159	65.21214	87.65741	77.72496	68.17535	76.76646	65.52288
LOC44115(	164.1362	76.82143	55.19903	95.31602	54.56369	75.68378	48.4877	64.96978	145.304
TJAP1	8748.04	7401.863	6392.761	8672.343	5346.698	9947.085	6117.679	4093.298	6325.897
ZNF446	835.3958	1397.924	1282.938	687.3178	1631.723	798.5274	1486.223	952.612	59.12778
SLC25A14	10154.46	9014.16	8745.151	8772.783	9405.074	9282.526	9008.723	8057.908	9934.175
CCDC43	140.9402	126.6466	75.29592	106.4903	68.9396	91.5026	64.57268	61.08087	189.4506
SHMT1	59.60349	1540.005	304.0135	48.81921	370.4275	547.3345	38.47415	759.2725	155.7014
MGC26647	31.2194	32.84927	32.38555	40.16146	38.35429	36.43181	39.41824	36.65851	36.87047

MIF4GD	544.4933	279.0698	498.3259	393.9502	454.6242	548.4391	504.3116	706.2422	661.6666
ATXN2	7179.582	9747.863	11820.53	11084.67	12209.88	10121.6	11554.75	8152.772	8642.805
ACAP3	1008.713	891.2538	360.2932	1640.896	242.2922	34.10751	77.91234	142.0911	46.1366
PANK4	36.02957	49.63741	114.1095	57.13721	371.936	47.74894	257.7749	324.287	55.63
SLC25A39	24867.71	25798.7	30792.3	28931.57	30753.04	27706.86	32336.32	29356.28	24543.05
TSHZ3	3284.492	3366.242	2468.938	4160.69	2806.67	3248.995	4455.768	2188.278	2097.966
C5orf24	757.9661	352.1594	188.0306	58.24487	90.92101	216.0177	173.0303	163.4937	1085.482
IGFL1	2918.125	552.5507	1821.762	4152.925	1894.431	4502.242	10746.04	1293.019	1522.612
BCL9L	59.96118	340.8964	122.929	132.7574	242.0566	238.5901	231.6711	391.109	104.3282
TRPM2	65.66196	38.09127	41.46443	41.94038	40.39673	45.09905	36.49843	49.45772	50.13317
DCP1B	156.1578	1653.287	587.6345	93.58539	57.19171	1090.189	84.59369	47.72081	104.7899
ALG12	378.2375	188.5518	107.913	229.4438	97.24964	149.5037	157.8618	121.1096	494.9221
SLC44A5	40.92804	52.14833	48.42307	35.94651	40.70914	39.06525	39.52656	40.02911	35.17102
GFOD2	9188.77	8964.604	5872.516	7763.909	7389.691	6773.535	4294.097	6313.601	11172
MGC18216	415.3142	189.9013	108.3638	272.8288	105.6125	210.097	97.69138	101.4616	633.4315
OR51A4	48.81058	52.39795	37.6957	75.92936	45.1379	50.3957	37.68199	41.76867	79.71078
GPR27	144.6603	50.69221	72.12273	87.24004	60.96983	58.92346	48.47971	72.91865	199.8379
TAAR1	127.9243	100.5899	61.73962	129.9128	64.08898	71.73809	49.37742	63.34653	161.8853
DCTN6	2026.492	1823.285	1413.659	786.6122	2104.435	1237.004	1616.588	1504.464	3631.214
ELP3	1211.248	2059.773	4013.317	1929.479	3667.539	3266.267	4208.189	3747.926	751.1757
C1orf127	100.5832	66.4669	62.67855	53.79743	141.4239	68.24133	89.39042	178.2671	349.1916
IMP5	165.9458	178.939	81.4696	174.894	66.34275	84.78957	64.20321	69.05272	471.8464
TNFAIP8L2	37.66119	308.0542	145.6732	34.0689	65.62748	250.4098	106.5418	80.88313	726.7217
ASTN2	39.33451	876.2649	329.0538	412.986	1103.718	355.5951	447.401	704.0073	101.5268
CHCHD7	207.671	188.9557	424.2639	436.2015	138.9601	140.6359	153.5286	199.1331	72.18245
PTTG2	57.44318	336.4363	135.2527	40.0333	135.9219	34.20533	43.61565	127.7369	65.89965
CEBPZ	3424.261	3669.462	3822.315	3962.66	3641.374	3925.379	3529.666	4007.786	3562.328
SRMS	94.54787	108.886	55.70063	113.6263	46.0065	61.91695	39.37995	71.97389	157.4722
PLA2G4F	2873.402	12206.92	6698.165	5134.781	13066.79	9222.981	11406.63	12485.28	6441.571
INPP5D	299.2798	320.0761	984.0353	388.2813	466.2868	596.1249	662.5432	712.1193	147.0334
KLF17	43.60946	44.17068	41.30878	46.40162	42.71381	54.98705	40.54353	51.21764	64.31554
C20orf106	66.17857	39.78816	46.66892	54.30835	51.20174	54.45697	48.4582	49.18736	53.66248
FEM1B	1763.567	464.0027	1924.568	1460.473	1324.925	830.6945	1022.224	1091.514	441.6684
WDR67	59.06249	50.35143	736.1724	50.05849	759.5164	736.3071	992.9368	583.8502	43.27319
LOC729264	3053.388	178.3083	2289.415	1456.344	1515.107	741.5411	804.4654	584.4026	59.67674
OR2D2	49.5109	54.03433	43.60785	103.6871	39.54465	40.08651	39.6679	39.39796	58.35334
DHX33	365.6215	210.1501	376.0382	210.785	837.6695	402.4228	814.1523	536.5411	440.3337
TATDN3	74.46985	41.42505	65.11223	607.5737	68.2475	53.19921	141.6351	187.9117	33.92316
SERPINE1	719.9086	279.6085	146.2484	1577.713	147.9165	303.0892	217.8391	133.5487	297.2449
KRTAP5-4	43.79144	48.55412	41.68833	36.66162	37.04781	58.69554	40.06678	32.20837	34.56551
KCNIP1	99.00294	66.92054	163.7336	68.7969	49.99396	62.32542	183.4272	55.2871	68.51525
ZSCAN10	1744.068	41.13236	267.903	536.1282	127.1932	281.9598	244.9949	185.7336	82.54713
KCNIP3	2909.176	2134.037	1241.265	2441.486	1486.992	1393.789	1028.832	900.9355	2001.541
OR2C1	50.34303	41.00537	50.02647	38.42661	40.31678	41.19098	44.97951	58.71432	77.58293
COX8C	200.685	164.8738	68.24359	183.9255	68.28123	81.10472	65.34452	77.28071	360.4059
TTPAL	1459.88	2383.965	1489.127	3037.237	800.6336	1939.533	1667.789	2085.253	269.5413
CYP2W1	116.2426	88.11804	61.92905	49.47388	43.05537	54.81297	50.42211	57.30744	174.7148

C1QTNF5	49.1764	850.3157	486.6239	39.72505	264.6073	552.796	742.1769	501.9007	54.56853
FAM53C	9380.629	8726.885	8554.262	8153.171	7024.359	8591.131	6643.202	8653.262	9053.978
GNS	7950.443	9518.735	14268.07	9549.791	13411.57	11212.3	14079.31	13187.61	7383.645
LYRM5	696.0306	2825.998	3696.697	1868.835	5641.008	3912.836	4128.063	4447.018	2330.58
ZNRF4	35.71131	35.74852	60.89196	35.96027	36.78065	35.77763	40.57327	48.72387	48.46969
TRUB1	129.7683	97.29467	215.8671	75.55573	58.61414	240.7633	102.4337	58.54551	122.3366
CYBA	55.32875	53.4835	60.37538	47.20111	46.48062	64.15055	160.959	36.58809	81.25809
WDSOF1	395.4331	263.3314	302.2222	319.0162	324.5628	343.0415	339.9178	389.665	91.05058
PLEKHG6	64.35052	55.9608	420.45	34.29198	577.1006	56.42268	218.2889	50.38651	40.70351
FDX1L	94.51141	54.47725	905.9741	641.0049	1231.732	1935.329	1463.515	1501.578	1694.834
IGDCC3	44.81902	285.4063	326.9858	33.60228	286.7387	51.98353	33.90796	34.06805	36.40627
CAND1	1069.898	711.4175	844.8881	585.0804	1108.831	856.4025	1067.913	943.8951	291.8985
PSMB5	651.9283	1608.219	1710.099	1673.456	3196.185	69.36422	2476.706	2252.367	5260.216
EP300	775.8163	870.4349	418.3694	1451.758	515.7133	349.3394	452.549	146.5975	99.56343
RNF112	444.8112	220.0948	107.2394	252.0365	107.085	203.757	112.7579	142.5653	621.2435
FGF2	45.37979	65.31696	57.52265	87.54621	48.86052	54.56275	53.86779	43.94683	80.6367
RGSL2	44.1417	287.2019	44.20609	44.05036	38.59786	50.58017	39.60675	45.95491	36.77977
DLG5	340.0486	230.2563	130.5326	197.807	146.2339	177.2708	123.6537	128.7141	502.7414
PARK7	2665.025	1307.213	2313.118	1611.784	3472.309	2192.469	3526.914	2645.942	259.3281
BTBD6	237.0651	1886.565	2245.024	2397.747	2888.46	1393.045	2427.155	2067.679	2693.029
DISC2	47.18759	47.24605	51.67955	40.03351	34.11644	44.74986	45.46373	38.18483	55.63756
KRT4	4823.641	113.4549	4499.997	425.0625	4529.659	2136.989	724.2676	595.6607	7172.225
TECTA	258.8901	214.9717	91.9174	236.0575	101.869	138.407	95.03188	238.5555	388.0679
USP26	196.045	146.3636	89.55403	134.1664	80.67661	107.6222	71.69242	101.0167	214.0069
TBL1X	18134.71	17777.15	16087.62	14668.86	17201.28	14603.62	13853.85	15221.85	17488.77
CPB2	56.57686	71.02901	43.01204	81.23167	52.09642	92.15735	44.44554	53.37178	78.12886
UGT1A1	46.56395	59.87291	43.95215	130.7687	148.6489	47.94605	51.13731	50.80922	123.1411
BECN1	652.2495	141.5116	179.701	134.7486	174.3294	207.5464	262.6717	248.6348	196.1597
MRPL21	33.07008	967.3456	498.2484	59.76391	1049.317	35.05984	2031.168	2412.289	35.01081
PCDHB1	35.26068	52.97593	42.82447	30.36987	40.26611	34.11614	32.00534	37.4048	38.44549
OR6T1	35.18096	58.53958	37.06776	38.05201	46.32941	40.28371	40.52176	33.45092	33.88387
RLN2	111.9639	112.7077	388.3495	69.39685	52.36787	39.38121	231.694	58.19317	240.8893
KCNN3	101.5287	153.9761	47.2995	641.1737	211.3902	46.27261	107.3348	36.97746	50.42464
CTTN	156.2316	65.92903	53.05492	66.08662	318.5211	129.1626	294.1643	173.3902	59.73288
FUCA2	260.1377	57.87805	104.2329	149.9796	154.3495	84.91348	105.6904	117.7095	65.67081
WFDC5	191.0258	1430.697	82.84494	1174.428	293.2521	97.70702	67.89466	181.7963	2134.702
PTGER1	86.50039	1184.319	173.9125	140.3781	306.7381	65.13633	287.5943	333.8223	1532.615
PTPRM	35.20513	1780.449	451.1119	315.8295	476.1842	478.8867	546.636	518.268	2285.405
TCOF1	55.89615	44.68575	57.42324	89.77075	69.15746	544.9487	57.86852	70.50823	67.2439
RHOBTB3	96.48392	42.68044	45.25879	51.03799	38.27311	41.5172	46.93694	33.33667	47.09472
ERO1L	9390.741	5384.046	6369.384	9807.651	3574.361	7694.533	4465.627	5007.459	8660.482
ZCCHC7	1454.688	2136.493	4772.457	2063.384	4895.51	5813.118	4753.402	3957.587	1944.488
RGSL1	61.74808	41.72982	45.13844	54.01334	37.95124	55.3675	40.80466	33.71338	34.31789
CXorf41	58.39642	57.52593	35.13696	61.36808	37.17041	41.51105	42.89982	44.70987	34.81221
SLC30A1	772.8419	819.2437	370.2339	697.3453	367.7744	641.6096	428.9318	301.993	1255.229
SLC11A2	10691.84	8026.339	10612.44	9546.218	13301.08	13047.56	11964.93	8821.157	8829.583
TAS2R4	48.40904	275.3515	45.78777	81.01147	46.0968	85.18408	46.67476	53.10701	107.1944

TCF15	105.0646	40.58714	322.0446	64.45332	352.6825	54.83589	554.1547	1168.234	62.37549
UQCR	485.8086	2750.554	774.2552	2547.419	531.1454	715.5166	491.892	320.2796	3657.448
LOC541473	41.86143	44.30712	42.30373	47.46294	55.37651	57.20045	59.57639	47.82198	37.23399
RPLP0	45.51472	37.81007	39.82755	1580.165	262.7099	41.16712	335.1423	33.5319	102.0014
CLINT1	123.1928	259.774	174.7715	339.7292	181.216	245.6996	175.3336	205.2179	105.2439
MIA	393.5607	218.744	123.1991	281.4409	117.5793	190.3756	182.9147	148.8001	483.2638
TRIM2	438.0746	397.9256	936.3331	531.0592	2133.665	2300.944	1584.367	999.3259	39.71336
CLEC1B	72.10093	51.6892	42.94476	37.92271	40.07932	57.54507	46.4915	56.8851	68.19187
ADAD2	1170.57	112.4695	217.6448	88.97941	246.3762	250.0117	129.3867	147.8988	862.892
LAPTM4A	539.4698	309.2993	190.3804	414.3534	173.8513	235.0878	197.8677	192.8899	671.5451
FAM102A	133.5168	149.8594	236.7874	887.9924	525.8121	1031.364	134.044	107.7697	444.3466
ABHD12	2940.496	1745.434	1978.099	1008.357	2491.648	1223.707	1816.891	1085.8	2564.845
C7orf11	245.2294	148.5259	163.8737	645.0006	65.49376	63.80636	264.8783	52.02765	92.34035
GDF11	318.9905	227.0269	115.1491	243.7554	104.6938	138.6868	114.0051	119.9021	344.7576
TMEM16D	34.94938	48.61155	223.4667	45.20481	39.94979	41.59896	34.98237	39.77803	49.40806
ZNFX1	42.23566	42.56449	47.61174	141.909	102.4365	39.66527	115.1848	151.2526	74.66869
SHANK2	37.11552	101.2697	62.00275	65.14612	54.41501	53.17603	49.73022	37.28257	46.35662
PARD6B	131.7763	181.1425	87.40942	154.6833	145.1842	86.88955	61.07777	55.04101	184.2991
ILF2	2804.308	3598.875	2640.003	575.7614	3583.045	2170.586	3378.762	3292.05	5601.569
PCDHB3	521.9823	258.2609	121.7612	266.2881	111.4452	57.848	60.60376	44.98457	726.9388
FLJ10081	35.48872	784.6838	565.8155	44.68296	840.6149	494.9858	1202.092	810.5863	2450.135
CDC42EP4	39.54323	3001.092	2589.504	3135.711	4423.001	2525.958	5137.121	4233.599	3787.89
PLSCR1	40.51764	337.5522	140.769	607.7207	57.25762	45.53953	220.2371	85.83713	43.58627
CXXC5	105.3522	70.5724	126.0153	63.06123	128.4461	223.6662	149.1238	227.4331	186.3103
REM2	75.30599	38.66363	31.96076	34.11335	35.09347	36.99812	60.44814	46.4688	54.26219
EXOSC4	60.18671	48.63316	250.9438	56.58998	140.1486	56.60744	68.67815	166.727	66.39177
RAGE	83.93218	1605.564	3565.491	3585.872	3630.788	2051.046	3173.471	3646.126	187.9615
FABP7	313.1832	208.0675	95.47727	192.7205	101.2157	147.3512	85.78412	95.50505	481.0315
CREM	418.8018	125.4715	188.7502	140.6126	109.1596	223.5952	129.004	102.0264	42.93993
PRR14	572.9838	389.2709	377.2357	486.8614	215.469	290.9649	244.1612	179.2747	772.264
RNF151	50.7743	54.02101	39.23837	41.89228	51.74187	66.47604	40.9342	40.20158	34.51764
PPP5C	66.22635	114.2831	94.60061	83.24568	80.33008	66.98162	56.74538	95.27724	185.9434
ZMAT2	12396.98	10071.86	8003.658	8449.358	6190.3	10296.68	5372.775	9008.192	9893.53
MT4	43.49334	59.00253	50.22679	34.46158	44.43769	50.60687	42.44724	54.50792	37.36774
CXCL9	30.40026	33.6341	49.821	39.43047	33.04564	44.57748	34.09536	37.43878	43.19701
PKNOX1	61.76554	83.8369	161.6932	73.1698	91.45953	90.76244	149.7403	107.7481	68.11514
TMEM50A	509.6656	235.5988	127.5485	138.7498	126.5687	256.1792	206.7175	166.8136	67.08803
IFI35	8422.133	11457.82	13239.67	8514.212	5308.498	12532.82	13244.82	10163.13	7104.805
HNRPM	413.3139	3199.997	4152.567	1543.192	6834.285	2772.162	5784.951	4476.383	566.3409
KIAA1328	95.83183	550.5688	803.7675	124.7121	574.2139	1462.553	410.2865	707.6296	218.7608
CTCFL	42.66791	51.69964	32.32615	37.95528	45.20065	46.63306	34.6878	43.65596	67.35942
OAS3	2161.979	2051.532	2484.677	4450.186	402.0249	2179.944	1799.337	1244.229	2006.18
PCDH15	84.43412	119.6548	83.8624	106.8003	50.24281	63.98531	59.2431	46.02652	200.8614
LOC340529	230.4438	171.0253	70.24166	117.6212	72.79615	75.19126	60.33341	66.32112	280.2791
BBX	1068.219	1185.917	644.719	602.0369	491.3358	1024.063	631.317	846.808	159.9042
ZMAT5	44.58755	51.13366	708.3551	44.83627	119.5208	46.97801	238.1127	703.634	34.36557
UTP3	1794.875	3050.323	4924.369	3128.17	4166.809	4135.918	4346.971	4673.744	1219.154

HIVEP2	884.3583	793.9023	439.2379	815.1887	446.6573	392.0995	436.1272	495.2727	1066.292
IRF9	81.3038	2131.333	4050.753	2698.334	2398.924	2628.884	3612.968	3537.607	46.50332
UNC5CL	77.02359	36.89515	55.74801	49.32961	51.22737	34.21704	38.76068	31.72066	63.56192
C12orf26	50.68312	55.04372	117.0509	75.80882	98.2505	257.6843	124.3874	40.85389	37.51577
DNALI1	3043.236	226.359	1210.454	221.3744	1669.894	665.4142	969.5487	605.6377	431.2392
MCAT	59.42118	48.79262	43.5071	45.79292	468.0841	39.32482	38.80908	154.5602	108.2085
PF4V1	63.24378	45.72059	57.64311	62.48924	59.23339	56.1647	54.18499	41.91425	138.0221
KCNH1	67.6339	253.7604	85.34869	164.697	84.47743	140.0249	67.94308	136.2151	51.35569
FAM117A	42.79203	407.8032	157.3064	31.72298	77.36373	216.588	99.62704	32.21995	881.2394
GNAT2	42.09779	46.96652	44.94063	60.99748	47.79265	59.01392	49.32206	59.60013	107.0845
KLK7	18829.54	20447	24415.74	17491.58	23296.6	20721.16	22093.19	23205.02	18974.54
ADAD1	41.57721	43.72235	34.40531	40.69003	38.58454	40.15951	40.47941	48.98657	39.9032
EXOC1	201.6009	127.4007	830.3331	144.269	1114.144	821.8066	921.7971	695.598	2540.504
A2M	10597.74	12573.08	12892.47	8788.88	13138.85	12459.45	13808.22	17757.38	11018.12
KRCC1	3463.595	3266.745	2313.15	2897.889	1426.945	1933.131	1416.873	2927.679	5111.372
EFCAB4A	3791.442	6099.091	2539.311	6215.187	2529.089	5540.287	2784.969	4749.556	6454.402
OR1Q1	33.8289	33.20106	39.85083	35.2942	38.53873	57.14465	37.21177	37.19995	44.31555
MFHAS1	1043.42	508.5651	518.5081	949.8823	255.7076	315.0892	288.5327	354.4328	40.34471
HPD	37.47863	55.03794	39.741	48.09193	46.79283	43.07978	42.34491	43.26624	58.50861
MGRN1	2264.566	1597.996	1969.714	1559.208	1553.756	1787.385	1196.313	1020.162	3011.294
METTL2A	162.9089	173.6577	83.04582	48.3791	41.20649	41.79998	37.29722	52.37885	49.0626
CORO2A	1152.117	158.2551	66.21006	86.57159	78.09529	85.04808	101.8268	95.8842	1655.842
C9orf96	43.66949	102.8913	40.19518	49.15792	39.37755	332.3093	41.57412	34.49814	52.60776
CAGE1	151.9124	159.6789	62.10331	100.5494	68.06382	55.33932	51.96835	70.54955	120.5742
DIS3L2	4737.141	2538.232	1664.171	3023.158	991.1783	1865.478	753.9984	1050.435	4764.711
FAM62C	123.2104	397.3537	37.38836	41.91474	383.6603	50.29842	119.3363	294.6772	165.287
RLBP1L1	52.3444	49.80071	44.2501	55.53741	46.18766	63.67544	38.04235	39.29284	70.40546
ASCC3	187.5867	84.96168	62.89877	107.4549	185.4806	79.1996	90.21527	150.9979	163.4533
ZNF324	37.49936	63.76788	48.62011	44.77766	437.2712	660.6224	165.4075	51.29176	50.91524
NR2F2	480.094	281.5037	79.33503	104.2056	117.5819	81.00643	73.27563	110.0782	79.39292
POLL	62.25558	45.57349	53.99552	41.23645	143.642	53.05222	195.3217	354.5429	61.74115
CX3CR1	292.1074	141.4765	115.3859	191.1505	83.32294	130.5467	75.27009	81.25893	228.2858
GTF2F2	674.4449	327.4334	592.4116	615.3506	802.8196	824.4235	547.4074	644.2556	587.5154
OR2K2	32.57728	33.35032	38.36158	35.84198	35.73534	34.79017	35.24252	34.30457	34.37151
TMEM62	48.77486	45.47808	931.3316	35.38711	469.8615	375.1576	991.1835	527.0145	36.69781
TDRD3	325.5833	43.50742	64.63557	53.78084	46.79676	117.0121	55.38092	47.96127	67.87064
KIAA1279	94.18588	88.58028	193.5901	58.32994	198.6434	67.06322	192.4936	218.3402	80.43204
NELL1	58.50791	105.397	61.7205	63.28695	769.1494	76.5451	513.0794	312.0462	76.62389
TBX4	53.45717	50.49572	46.47523	51.98328	44.47242	59.75685	42.40386	39.97269	50.02162
ENTPD5	179.08	163.8475	810.4362	639.6061	536.9802	337.8288	575.9616	675.2289	261.0964
FLJ40852	378.2727	275.4649	265.4079	230.4465	310.6183	246.7235	151.0692	280.4316	545.2504
DNAI1	103.1045	41.82545	50.08538	329.7301	92.30109	199.3863	81.49895	48.79862	849.7672
LOC401410	95.74711	57.85597	53.0163	64.32671	39.23379	60.47055	44.3514	54.36809	39.29386
POLR2F	3847.615	2662.541	5257.86	4012.416	7079.692	4654.638	6788.586	4973.622	64.50558
FBXO21	3180.125	3239.372	2523.199	2485.699	2858.287	3190.717	2530.013	2341.772	2452.243
CCDC6	159.8485	57.40944	4754.272	1506.298	6462.455	3333.695	5897.97	3151.115	78.22907
TGFBR1	680.9771	392.868	219.6036	783.4319	246.1228	284.058	308.7489	418.1253	801.2896

OR8D2	55.79853	63.04963	50.14857	60.87989	54.26581	41.44405	49.1816	46.15326	56.50934
CCDC5	73.75898	138.9093	120.1224	108.8076	58.62671	90.66393	75.09431	73.77164	70.01152
CEACAM21	150.9618	86.82628	86.21649	119.9895	79.71973	76.53709	59.35419	55.12739	225.5741
CORO1C	89.7193	368.4589	354.8679	549.8169	746.5388	323.2413	689.3864	697.3145	50.38877
FOLR2	134.4862	118.5021	381.2115	135.8791	681.9453	465.0498	508.4246	568.5047	314.1294
IRAK2	3827.638	3457.257	3145.5	3284.82	2475.234	3698.759	2544.765	2365.881	4606.61
TRAF4	118.7413	39.1506	77.64065	88.17676	68.55261	143.9462	54.35012	92.93856	90.62131
NUDT12	252.9469	302.7121	137.6231	342.0365	183.7899	364.5642	185.5643	210.0612	513.9991
GNRH1	1146.769	1158.386	498.1215	214.6404	905.5812	698.728	548.1432	117.9029	460.8473
C8orf30A	55.51616	63.01543	36.44643	39.57059	45.85767	44.96203	42.49866	50.42588	44.34046
MBD6	489.6283	314.7148	408.3784	329.3947	501.5462	459.3949	477.8937	441.9058	777.0672
SPOCK1	115.0283	150.3439	163.4827	154.1762	54.04121	72.81374	279.1474	48.19226	215.7238
MDM4	1374.965	534.3562	417.8113	49.54956	91.41777	377.5799	289.4102	65.80445	54.96253
SPATS2	1225.807	1295.883	274.735	1580.095	554.3999	680.8226	438.4208	451.1053	1019.46
RIMS4	327.2697	191.1042	93.46029	201.9225	89.66187	151.914	89.22503	125.5458	431.7901
IGFBP3	87.00539	39.62196	298.0395	60.92226	1071.31	631.483	1171.83	441.0155	49.78752
CPO	113.4833	63.1948	44.14011	53.49609	36.18539	42.45167	37.05296	54.0188	1138.047
STT3A	51.30022	48.89513	42.59422	421.2896	143.0539	245.5901	350.7473	210.0441	120.0321
OCA2	52.85045	59.72259	34.44235	51.40176	128.5316	39.76469	41.30052	51.63367	50.58517
ACSM2B	39.27158	39.57193	39.61391	35.24485	31.79562	35.30242	32.142	46.19754	106.8177
PHF23	2633.256	1663.283	2932.791	2831.292	2409.553	2771.358	2601.735	2790.269	2796.038
RLTPR	42.70429	764.6069	2087.257	919.7849	562.6625	1175.94	714.224	624.6437	44.06373
LRRC33	76.77673	81.69805	176.0233	72.8027	37.48	377.4913	229.6286	305.8065	71.09517
HOXD11	165.9416	214.1695	88.62769	223.7881	78.68427	93.29302	63.46268	123.2684	175.7434
SCD5	1156.139	36.06191	213.3335	1515.988	406.8335	378.7298	205.9412	321.7948	41.00332
TNFSF4	45.77379	46.68799	58.90736	51.82692	51.13535	61.91523	42.13476	45.16747	32.95259
DUSP21	54.52787	58.23592	57.08183	39.89772	42.82325	38.168	41.32169	52.20918	214.028
ABCC9	445.8502	167.9914	81.12211	49.51005	75.0052	73.91825	66.98922	55.07185	106.7457
SNX20	55.15833	47.30228	38.8374	37.92973	42.6561	46.14309	33.58071	34.18618	39.56884
DBH	47.68649	62.81225	31.61766	43.9313	35.1196	41.95202	149.8498	47.60378	44.63469
KITLG	2646.235	3602.365	1578.248	1965.241	199.7496	2271.079	1496.354	1264.018	40.85186
TES	573.9237	253.4966	136.1734	415.255	96.41349	170.8678	114.931	117.6124	514.6846
DAZAP1	98.68705	113.862	99.86111	141.7854	63.07427	108.5429	72.81884	51.06028	465.3867
SARM1	1137.463	884.1864	1000.31	1071.975	935.2328	1020.209	1117.314	1593.501	1651.303
EPC1	1170.573	1664.671	167.9537	45.5051	228.9211	338.4992	97.59072	42.23793	53.3933
MLLT4	220.957	121.8587	102.8399	94.41344	87.96651	74.94228	83.13931	102.915	297.1024
LOC389791	32.14709	30.24095	38.29385	40.27555	29.98545	34.95807	37.89487	47.99883	38.83715
GALP	69.84332	62.99499	50.0508	68.5796	44.03163	65.75227	50.67463	46.52126	307.3513
RERG	792.0264	47.46178	32.69862	42.96407	138.4546	38.46933	37.51928	35.56744	32.71629
SELT	2864.516	2430.564	1890.23	2627.043	1434.974	2357.239	1214.177	2808.875	2072.684
LOC64298(	243.0267	159.4835	109.782	163.9125	80.34982	104.1806	69.58315	233.7744	218.8986
GHDC	1496.719	837.7685	398.0575	99.37302	289.5579	407.9592	552.9683	260.6646	33.39807
HHLA1	172.4317	59.13244	89.64713	79.50011	45.23719	57.63121	56.08403	58.58329	177.538
TSEN2	317.4393	1247.381	1134.578	701.3777	1378.257	998.5809	1379.903	353.3213	424.4886
MIDN	936.384	1316.947	1204.526	789.3866	1142.225	1012.292	1150.069	1027.646	1149.779
TOMM70A	143.6424	254.0661	255.6078	252.3887	392.4283	369.4214	423.4425	390.7678	555.1172
HIST1H1E	54.35468	160.1883	75.29321	108.5095	58.1087	71.17287	66.17715	64.85955	342.5765

HCG27	472.3463	1935.315	1033.068	106.0277	378.42	1627.905	957.8286	319.2762	366.7188
LOC727752	151.3126	75.96802	55.4209	62.61886	64.90374	91.13101	49.51534	66.17685	84.1007
GTF3C2	355.7885	146.7321	1401.862	598.3029	2948.946	1348.867	1989.031	1390.086	237.6507
FJX1	198.3976	554.1061	838.0732	924.2346	1200.895	902.0027	1331.784	1407.086	400.4135
RABL4	230.2999	172.2816	151.1169	204.3196	143.7389	107.7966	145.6913	100.9505	458.524
DNTT	144.165	79.62351	57.10589	70.77348	57.97052	276.3604	48.1424	45.18377	1397.031
RPS25	14790.19	12834.93	10344.38	11615.93	6557.25	7235.97	5127.084	6225.377	13338.55
CDC27	108.4425	120.8233	60.26692	141.0716	61.57899	91.30061	52.71167	64.44032	146.5149
BCL2L2	2959.781	4235.994	4355.611	4823.828	4820.598	4383.979	3899.059	3811.002	3825.896
CD6	173.0215	293.1379	1603.802	78.58478	832.2871	349.7027	825.7706	1434.203	71.67116
UBE2O	684.5238	1176.494	1633.579	1614.396	1820.849	1268.168	1483.765	1725.703	1697.799
TALDO1	44.77951	65.12585	509.3252	53.53022	806.8748	510.1032	405.4637	198.6092	35.76043
SCNM1	175.5433	523.0501	77.49263	128.757	146.4578	267.1517	244.8807	444.6469	51.6798
IL6ST	45.65416	711.6642	32.41644	38.31637	54.09085	50.51474	80.22497	163.4633	42.21491
PAQR7	87.58819	406.6953	714.0768	577.9294	586.4588	643.3199	644.2457	754.2468	90.88384
C9orf25	41.68819	260.642	313.6931	463.9669	384.2188	513.9695	340.8217	257.0212	60.873
CHRM5	62.99923	58.29849	129.9301	68.68687	43.98489	42.36908	164.2204	57.03099	40.00935
UNC93A	51.31361	392.9758	403.381	76.15852	132.6288	352.3094	89.25328	47.64848	181.3676
RORB	669.7786	1016.682	172.8709	43.71557	373.4448	150.8477	149.1816	117.497	731.6431
WSB2	1482.669	1911.899	2475.024	2149.402	2603.254	2857.003	2452.578	1707.224	1520.627
C18orf26	7779.959	467.6136	7388.122	499.3137	5382.305	2869.774	1952.83	374.2354	5025.456
CLDN8	70.99036	73.09346	65.67709	101.9362	88.83909	82.67984	64.25594	55.13085	286.5471
C1orf107	280.9033	381.5591	164.3896	149.4886	82.43699	123.6123	102.6122	82.43304	289.0457
POLR1C	5093.653	5012.573	8127.635	5539.353	8100.889	7023.261	8363.85	7646.724	3872.315
SGOL2	66.01799	47.43176	204.6051	33.97674	147.5084	42.99982	101.2837	219.885	45.698
TRIM8	5024.418	4999.681	6686.249	4886.044	7407.633	7694.697	11351.34	6179.339	81.63412
PCDHGB3	58.44772	48.18725	38.07102	63.98056	41.81324	54.51331	46.19082	55.21858	89.8996
TTC39C	42.93561	775.3229	1636.539	69.27618	746.9644	1777.644	828.8218	1670.919	62.81813
ECM2	50.87069	33.36422	35.32182	32.0425	31.88516	37.3703	58.68387	158.3365	42.75477
PLA2G3	81.95654	4588.893	7357.875	2017.921	17024.85	4235.27	4842.481	7919.192	3063.009
SLAIN1	53.38859	51.64706	493.192	64.7159	470.5701	564.3444	409.8209	290.2103	70.64761
CGN	444.5035	2613.542	4039.651	3307.854	5406.072	4277.426	4051.052	3135.478	3510.592
C5	256.8278	995.3004	1814.931	1119.842	1719.227	3745.574	2098.116	1310.665	206.8824
NLRP4	118.9784	99.98684	156.8764	68.04572	63.86714	81.34662	59.46031	70.21724	162.7196
C16orf65	69.26787	67.07096	44.5208	85.61209	58.04357	62.49557	51.90493	76.13752	140.5617
ZNF114	83.02847	315.035	114.9704	345.8092	67.87064	55.33582	225.6715	49.63797	172.9687
CEP290	2687.048	2348.058	1086.923	2474.657	671.0361	1014.407	889.0197	1014.152	2469.288
PTAFR	678.7321	137.5412	232.1628	162.9948	89.41662	123.2317	186.7919	138.7338	316.6598
TTLL4	44.78618	45.52861	36.36456	70.04186	80.24883	71.08281	260.0951	51.51341	2276.394
RAB21	1620.693	2572.882	1081.941	2721.276	1278.3	952.5375	1008.899	2137.584	4190.508
PSCDBP	2187.183	2672.091	2431.057	1225.159	878.2106	1816.095	1053.464	1106.054	2781.779
TNRC6C	84.37372	69.09377	60.23574	73.84827	53.01593	63.69507	98.25015	52.66037	97.79299
SAT2	58.31693	333.3788	1661.183	49.22217	2675.927	912.9228	2830.764	2313.869	2052.776
PCOLCE2	50.75078	634.1625	49.94308	66.78799	159.4624	55.45841	190.9261	476.1591	79.7739
ZNF610	37.94799	34.82894	113.4089	38.30803	69.64635	32.99884	42.03036	44.99564	57.62431
TMPRSS11	3182.719	4149.834	10928.6	3056.622	16125.93	7750.107	8932.671	7578.687	3405.27
DNAH17	49.9271	516.328	158.3131	472.0816	41.67094	39.02666	91.67635	142.0665	42.51702

HIPK3	4108.13	3071.114	2114.063	3413.599	2694.595	1988.964	1246.328	1422.5	3650.116
CSMD1	292.804	178.5915	96.99268	196.0975	90.53526	148.044	81.02001	106.1319	456.4734
CCDC11	121.1476	138.4899	73.91828	79.47928	191.6441	392.1991	90.33987	281.2096	890.4376
PHF1	242.6388	185.567	754.9812	657.4056	693.943	407.0067	830.7723	98.23698	1656.35
CEP55	82.02728	259.8427	428.1985	1152.356	882.545	394.7458	1072.642	396.7004	99.81939
PDYN	323.9268	158.1181	95.06149	194.1189	94.51755	130.7445	73.93085	86.38183	343.4941
PTGIR	39.56649	43.50773	47.36787	60.58632	200.9814	39.76835	37.88057	39.92513	61.88566
ITGA4	455.8234	247.0713	165.7786	267.9874	162.2413	191.6045	147.5629	201.0599	462.9905
TRAPPC2L	3845.585	6616.049	8549.987	6876.042	6861.2	6072.669	7942.353	11282.4	6143.091
NPAS1	627.3517	1931.68	874.4163	685.5991	650.0892	752.2843	628.0869	876.8875	2114.818
HYLS1	148.4704	110.998	262.8659	348.3745	115.239	91.97277	106.2727	122.8625	182.019
FLJ42177	43.87664	58.9105	242.5087	52.11604	278.0827	850.7125	312.5469	381.8246	1250.143
LUC7L2	308.7489	194.2375	92.97774	182.1177	95.38913	154.9132	89.63352	79.32337	410.9576
CAPS2	137.4814	446.5427	107.0317	57.69723	134.1706	40.32147	77.52539	177.4772	90.122
CASP8AP2	36.29679	48.03377	50.54605	46.06808	63.83974	38.29054	61.32285	38.38094	87.66518
TSG101	257.4631	131.8337	124.5131	155.0519	284.1107	105.2789	109.1029	159.7157	176.7525
DDX47	368.7316	206.5038	98.76582	831.3177	77.4986	120.6657	133.2191	116.7349	545.2608
PUS7L	88.90731	64.22169	437.5557	54.40713	565.439	42.61386	313.1486	259.9892	57.78362
RNF125	56.2706	50.45004	47.54602	80.59355	83.18723	43.9149	57.94526	120.9654	69.86848
PSMB8	3361.823	7991.756	13467.88	7957.5	10605.74	10259.53	13659.07	9471.071	4795.56
SLC41A2	46.85182	605.873	59.71951	58.53655	97.80453	77.69891	57.13938	46.63539	59.05393
HTRA2	946.5468	434.2	531.6353	468.5659	446.1427	448.7947	315.9866	355.4474	41.17404
JDP2	550.1104	388.8988	173.656	395.2342	174.0547	285.4436	162.391	221.9135	683.2762
EYA1	102.9634	44.35462	54.37244	44.21889	43.52134	44.55953	54.53575	43.63592	110.9369
C15orf41	649.9747	525.8679	281.9912	785.2043	112.1096	187.5421	431.2476	211.1384	1013.536
ADORA1	1314.527	1332.247	959.7303	467.8974	617.1689	1012.406	779.9535	1239.035	2889.06
IWS1	40.83131	47.88438	115.2821	278.279	82.28861	38.15284	94.79079	130.5438	32.17291
CCNG2	182.4834	74.32287	86.62548	93.64716	84.01877	102.5009	53.25174	70.44877	106.3001
LTB4R	3266.708	1606.354	3528.052	1182.544	2824.367	2051.569	2794.534	4822.561	36.84477
ATP1B2	317.2538	68.02963	111.8292	129.4349	92.82677	116.5314	63.87118	86.63979	309.3042
ZNF648	52.3409	48.33824	44.14727	40.54536	48.73072	37.77058	80.7477	41.35776	174.8301
EVI5L	10374.98	11463.89	13366.29	11449.39	13986.25	14075.28	12796.22	14439.26	10681.03
DDX39	3083.497	4913.507	7832.904	4442.632	7389.169	5741.329	6462.725	7103.47	3625.15
GIMAP8	327.6249	461.8785	960.5521	471.5576	751.2311	756.1895	905.9244	571.0789	647.757
INADL	3387.253	790.1092	902.0161	457.1706	415.7876	592.0597	378.6928	451.0106	1686.309
IL3RA	287.8649	196.5994	214.5998	180.6974	218.904	161.9606	185.0511	234.9758	408.0268
KIF27	451.0874	241.0591	207.0665	295.1444	183.5667	244.6886	192.4994	244.1272	668.1807
SLC24A1	48.36769	58.77713	333.6084	575.73	289.3533	40.68279	586.2476	347.7515	62.97574
SCML4	38.93587	51.9517	211.9193	69.83963	41.60582	41.08838	36.68634	47.6132	66.10369
PLCXD1	73.64205	1620.551	1650.258	696.2378	4327.511	449.1134	1844.888	1090.56	43.11227
TMEM184I	79.85052	68.81877	70.51501	99.48296	218.7848	83.66583	94.43476	66.85626	278.4111
EMID1	34.14293	700.358	39.92605	32.51574	257.1471	41.21712	51.40096	40.15407	52.397
AHCTF1	35.44685	39.92916	203.3171	201.0122	33.76934	43.54548	225.9433	165.7589	33.34539
DHX34	1012.107	416.9506	819.4774	497.9946	625.7116	334.1219	527.8445	576.9682	92.26623
TSPAN1	214.6458	51.51233	71.64335	120.079	117.269	104.1285	102.5928	95.56281	79.73361
CDK9	2865.801	1862.737	1113.839	1494.552	730.6483	283.2969	539.8129	1550.345	50.82266
C3orf65	35.323	39.10678	39.85153	33.09468	43.91747	42.4978	33.03865	36.89015	35.31794

TG	43.37239	49.46882	54.24657	56.19778	129.8655	39.08397	41.99938	46.87319	56.6659
HIST2H2AE	57.71479	60.27799	49.2684	66.31841	54.68219	37.30492	136.4234	44.45769	48.54259
PPP1R11	6035.12	6156.24	14086	6152.178	16815.23	10976.65	14291.21	11742.31	1845.688
KCNC3	76.48007	88.01756	236.1655	82.40689	61.25194	76.89859	53.9164	74.12002	2010.548
CD33	37.57224	35.05143	40.49687	42.9582	124.0474	44.07582	71.14428	133.7481	36.17124
DEK	7996.113	7927.682	7680.407	9142.14	7118.32	6727.385	7606.149	5691.65	8420.575
BMP6	66.59615	299.7189	114.8985	46.43781	186.0048	139.1039	125.032	62.36712	102.0714
CCDC74B	38.65133	42.5399	44.11354	46.48922	42.98552	38.05245	37.20269	38.4837	50.05177
CHML	483.1069	255.3265	189.1874	266.1814	208.0698	249.8132	156.908	247.1687	668.1714
ALDOB	122.48	139.4808	77.48245	154.8338	84.47376	104.5346	62.41544	81.93622	185.659
LHFPL2	87.37708	136.6459	241.9198	67.11364	57.85013	296.8239	96.72215	63.82962	151.4567
SHANK1	42.66298	38.82513	41.50863	34.91653	35.36498	43.25623	36.84632	36.61982	55.70851
LOC73074	59.88879	51.44978	208.8905	51.10605	701.5026	373.8758	552.1466	456.6411	70.22562
CYS1	53.74738	40.39582	46.287	38.97294	49.3203	39.92024	42.71891	39.05262	101.0059
LCNL1	159.6824	69.88102	74.40967	73.14249	69.60291	94.6875	264.4931	82.1216	249.4412
ZNF85	3237.913	2527.629	2724.731	2214.737	964.821	2664.152	1684.411	743.1309	1645.824
LYZL4	51.6238	51.48888	38.68487	37.03732	36.35593	43.5543	37.27747	41.30648	158.9766
SMARCE1	1117.715	2032.04	3323.323	2917.836	4837.655	4372.838	3453.689	4998.698	907.574
CD3EAP	533.0145	277.6062	145.8336	364.8263	150.7862	327.6153	257.653	219.7506	628.701
NOS2A	71.33937	38.19933	46.14317	54.5372	36.4183	48.94562	37.59236	41.56484	218.6346
DNAJC11	87.03062	139.8514	70.88801	60.54554	65.49025	70.27778	183.7578	76.07532	95.66823
ZSWIM2	36.20581	47.95942	49.48038	43.71404	36.2534	44.38374	39.92931	49.95366	33.69151
TMPRSS7	111.1768	36.55058	50.25906	57.28053	53.19201	45.27628	38.57626	53.4883	70.39555
ISYNA1	535.8743	292.9896	118.4631	291.8671	108.2948	184.3728	102.6574	104.1399	698.186
GOLGB1	51.49304	435.8445	1249.191	468.1196	2509.712	1251.039	1562.394	1174.919	542.02
ZNF695	52.01875	1353.829	645.132	1086.475	340.3507	866.5057	247.3127	46.12001	46.29511
UTS2R	114.1245	57.85469	52.51682	51.49375	40.0289	90.34901	53.83942	62.85567	190.5159
RBM33	137.7437	329.1089	633.5076	331.5342	1278.785	1063.676	839.2867	757.1291	342.2264
PAEP	54.71428	400.8401	92.07996	119.4209	53.58525	95.45898	147.3422	347.0418	99.94431
GORASP2	10469.98	11499.6	12924.63	11800.54	15156.33	12836.68	13742.19	9251.367	11445.47
OR10W1	48.28522	43.48676	48.28237	53.13526	31.95564	39.47283	36.73328	46.28352	44.64441
PGAM5	66.98003	36.04555	50.71132	547.1291	45.98896	38.02948	36.39425	214.7362	57.57575
KLHDC4	5133.3	3355.522	3346.037	3581.567	2974.167	3534.798	3870.119	5398.669	3988.421
LASS4	168.8342	143.6989	74.50058	136.8167	84.94823	88.99338	60.29698	73.2372	477.3806
RASGRF1	117.1514	149.5834	66.59553	86.83069	64.17175	67.05492	58.94334	56.3373	127.8091
SLC2A11	347.5514	198.3387	112.2589	189.869	97.5529	152.8921	113.5851	113.7386	384.4314
F8A3	342.6716	154.9687	87.58273	124.9101	86.80159	127.3755	73.4137	86.89339	350.7246
PAFAH1B3	13659.21	12133.01	9986.689	16028.38	7870.064	12293.3	8593.24	8197.163	13495.81
CACNG5	107.3635	80.92725	56.35923	37.66191	50.45815	80.47919	46.49618	57.41524	35.52279
PEX1	826.8814	367.5232	449.9099	782.485	401.3222	245.9581	177.4647	217.0998	59.12938
GEMIN8	3607.299	3671.981	902.7233	693.976	2749.723	2511.01	1781.513	401.3223	3178.788
KLHL14	49.57096	54.39896	48.66153	38.12595	36.89866	47.59975	39.28519	35.46384	50.19838
MTO1	1624.68	1415.242	999.3082	930.1107	892.9277	1217.037	816.031	827.041	1179.525
SAMD7	401.2523	232.7768	113.0237	199.2192	112.9813	193.7187	96.76336	142.1832	502.0168
KY	48.10722	40.00895	43.3488	42.49003	39.39235	35.90382	125.6627	116.7855	35.76466
SRY	385.9452	249.019	127.7578	257.1521	105.2649	182.5348	106.9965	109.9044	439.0791
RTBDN	68.89055	418.606	57.99422	502.7537	45.64554	54.97518	54.59203	646.8582	139.3151

C20orf160	38.32292	1462.774	934.9471	631.8267	1926.3	768.0728	1818.358	1010.982	67.0378
PADI6	157.936	157.7232	54.43275	171.283	64.84068	68.14587	67.53722	80.70061	152.7395
CENPP	36.57429	38.35188	358.6714	40.68707	264.048	45.59145	210.9014	299.6119	63.00249
LRRTM2	154.9131	77.34592	62.15225	309.696	98.24606	180.1264	89.47427	85.13113	212.957
ZNF714	42.63619	58.9754	44.85122	66.90477	44.24588	100.2216	37.00611	38.14273	80.88313
ATF2	55.82284	45.07874	1245.415	50.34741	59.86557	814.6382	661.0698	276.0844	1872.244
MAPK10	56.01312	49.68293	39.87352	87.41557	320.1886	288.1114	154.278	77.7531	48.92743
PIM2	61.42728	302.5562	275.5881	83.79043	195.3724	176.6396	177.1581	129.1905	150.4537
FLJ13614	320.9263	256.6213	146.5652	281.4699	96.4515	172.7965	82.90424	124.211	401.2892
C1orf55	40.1755	57.68399	203.1987	38.53921	183.6996	40.56303	179.5932	42.49166	40.29545
CXCL14	7687.284	8611.339	7620.082	8243.741	9938.465	10461.36	8852.577	9454.454	6417.027
GPR85	43.88852	47.68343	37.71827	40.00697	41.4864	50.57628	44.11728	40.42461	51.08489
OR10G2	45.73642	81.94627	47.78964	90.02399	42.8096	52.62806	52.78126	58.88793	51.52132
CTSL2	783.7958	1549.204	582.7243	948.858	430.4101	1941.794	833.7933	1351.606	424.5915
MBNL1	59.62041	31.38392	51.98581	33.3186	31.44923	36.20414	36.55614	269.1473	35.01042
SLC5A9	76.1746	85.27379	202.1898	76.59512	55.73711	57.71987	46.74343	45.6048	137.5425
NPHS2	81.7512	49.1087	38.73915	61.33182	41.13138	57.52649	46.49409	51.20089	56.33527
43533	2846.147	809.3547	1738.347	850.7923	1065.708	841.8823	944.7891	1124.987	67.23957
ZNF239	204.7282	97.7691	248.5396	141.1971	146.8548	113.4655	147.7917	181.2476	162.7502
POLR2G	3058.587	3590.292	2683.716	3545.199	2507.712	2459.464	2810.82	3525.94	3958.985
TAL1	186.2177	104.9404	68.15536	85.46884	75.69408	74.37775	66.54187	42.95541	105.9859
GIMAP4	15352.84	14061.39	15289.46	11139.87	12642.5	14888.94	12726.73	12670.93	14877.48
NOP58	1011.715	964.7496	2202.219	553.2662	599.0743	714.7012	1192.214	1266.954	740.4717
TMEM199	70.14574	48.20577	822.7323	77.49986	721.3405	817.3539	610.8213	603.5112	156.2769
TXNL4B	45.98851	46.89394	39.29629	38.79895	116.3991	41.45572	105.9001	151.3609	109.6425
LRRC2	49.44482	113.029	51.78405	95.65717	63.60709	75.84229	44.40295	51.94128	51.69244
ALDH1A2	200.6603	132.116	268.3717	161.9801	304.1134	112.7167	455.5019	101.846	432.9498
STRC	319.6615	195.2307	93.03848	156.9384	87.79166	103.1018	84.1574	79.38551	225.8735
LOC85391	155.4144	58.51072	61.7459	96.76745	76.58607	123.8078	47.64178	58.86019	226.8682
RAD21	836.0969	2790.938	3027.997	3783.473	2849.401	2197.69	3388.797	3150.053	1548.681
ODC1	168.3143	3532.277	1197.309	816.8193	4135.623	1266.441	1770.712	3160.016	1394.453
NOBOX	59.25753	82.80978	41.39664	58.88913	44.3765	57.41533	48.1715	74.52586	48.29675
RELT	246.3143	228.7946	119.7152	381.8931	95.20131	94.26915	69.6	88.48731	706.485
GTF2F1	146.7114	470.7408	705.9938	55.60652	132.017	272.9969	330.1104	859.1478	104.5429
TCEAL4	8987.771	7497.012	9285.006	9729.529	9485.395	10271.71	10316.24	11752.82	5852.58
MORN5	50.41894	42.45433	40.13898	51.11557	39.48573	40.78506	40.914	35.15114	42.35752
MRPL45	2682.307	4249.022	3526.222	4764.376	4398.805	3948.442	4765.643	5745.766	4039.884
PSMB3	4999.923	10538.51	16334.45	10740.21	15229.63	11797.48	21358.8	23984.69	2439.598
AKT1	304.3179	211.5872	101.8274	187.9192	109.1979	107.2449	76.17669	129.7029	306.1229
TMPRSS11I	2189.354	267.023	1567.471	480.1403	1210.532	1371.065	456.2444	173.6585	1276.139
TRIM31	893.5208	1027.213	754.4388	721.0519	463.3771	1452.644	1061.817	2016.472	592.5239
NHSL2	186.3704	863.8621	507.4497	50.17287	210.5287	475.0164	304.3161	58.93196	57.12774
LIN28B	50.36987	60.52657	57.87904	53.50051	40.95274	49.39722	92.22898	41.76273	38.58776
CRELD2	58.51567	324.5715	386.4209	52.82644	440.262	323.907	289.3887	393.7712	94.02278
SEC16B	49.11779	99.40365	50.84178	54.24384	59.37781	81.09177	52.18833	54.79781	142.3544
CTRC	76.25408	2138.474	1103.922	68.08265	324.1638	936.8035	745.2193	1226.219	63.01073
NLF2	6792.533	3032.595	2828.19	3818.87	942.7346	2658.925	1899.3	920.1536	6695.185

KCND2	37.86668	41.65579	37.50605	43.47686	47.36791	113.2723	65.89479	47.14412	35.05313
SLC4A10	47.14996	58.72746	54.73174	115.9324	40.27486	40.6815	38.13534	44.06586	93.40309
ZDHHC9	48.94356	59.01462	68.04981	45.25878	57.56902	36.82989	51.09921	55.39389	69.4604
TMEM130	54.76155	39.59633	55.43383	57.26386	37.84234	43.94887	36.31897	53.01846	110.2462
LOC37492(	472.2212	243.8299	113.485	279.5219	81.61047	144.4599	78.5246	113.9646	585.9252
PPP1R13B	14697.04	16484.27	9751.296	13934.2	10442.83	15291.91	10258.32	11450	16762.21
FUZ	60.75037	50.82425	110.399	64.06091	54.11395	63.63297	64.56735	51.56421	72.77517
CALB2	259.0543	610.4399	1372.233	1085.615	2077.692	1975.017	3256.572	2083.131	1491.116
CNKSR3	637.0771	603.6237	527.7462	449.9185	1735.297	499.2723	1069.644	519.6398	751.178
KRTAP19-7	349.4814	176.8984	111.2695	195.4155	103.9497	171.7942	92.67818	125.1352	354.9033
MRAS	67.97304	583.4272	190.6753	42.16667	43.53756	356.7966	38.28421	43.34773	37.97021
TOB2	225.0214	158.7899	120.1161	132.9277	102.3278	112.3315	117.2544	135.2047	539.5625
GPR176	234.9338	75.19347	130.371	388.677	56.75486	76.76063	104.0399	232.998	71.34472
TMED6	44.67533	35.59466	38.22959	33.40895	38.59555	39.71184	130.4275	47.04229	33.03463
LIN28	39.7333	40.63264	37.40031	31.70014	36.70657	43.43444	34.58915	33.47012	45.26931
UBQLNL	72.73639	65.42244	910.1674	75.62554	481.0866	358.8859	174.5824	566.6218	51.72441
PROSC	39.29766	46.02652	579.296	38.38929	808.1453	386.655	986.5354	679.5427	56.35969
S100A12	624.588	385.7307	906.8427	100.0155	2558.92	1481.106	1073.831	3863.153	46.08448
C18orf55	747.0516	247.6911	276.2029	245.684	201.0232	310.181	176.0585	725.9794	953.9841
SMC2	423.3747	225.9711	105.8856	236.484	109.0008	149.0881	92.01241	115.6507	510.7229
TMEM209	2726.999	1135.493	1084.538	2446.278	1006.579	873.3521	698.1847	1362.357	1574.335
PRR15L	310.3401	197.0774	101.9694	228.9368	79.96281	151.9658	115.1907	110.2711	272.0887
SLC4A9	45.68312	49.59584	46.50421	70.57836	46.38018	48.0123	40.8876	49.43519	58.10455
RCSD1	979.9808	63.68553	409.8307	81.4064	471.2704	700.9476	219.9346	268.4233	144.6188
REXO2	37.69029	1737.686	3870.653	1483.979	4934.459	3232.861	5093.576	2843.037	2929.889
NECAP2	130.643	124.2818	66.18008	59.6448	878.2133	859.8955	646.3328	540.5359	114.655
ACLY	412.0757	268.007	236.0264	292.5572	183.9287	269.9973	136.018	179.9278	548.615
LCT	57.55863	68.23322	62.81407	49.11928	50.07846	55.3413	47.40241	42.78674	51.70269
CREB3L1	186.6922	695.9202	65.65675	62.67933	52.73335	414.174	323.4168	400.8185	114.8279
CALN1	297.9817	179.6759	79.67396	163.4485	86.7645	107.6906	60.70438	100.3257	282.9988
PVRL4	40.77625	45.58151	257.3233	855.7053	584.1005	41.53938	299.6123	458.1029	35.73453
C8orf74	42.74323	51.38016	60.9671	61.25185	48.86283	48.85041	47.95464	38.4177	88.87344
HLA-DPB1	86.64153	39.75835	274.4422	327.9027	201.2592	179.7689	171.9126	243.2297	139.3755
CNIH3	219.6147	156.5389	78.73454	111.8042	79.68812	124.0262	77.76822	69.61488	299.135
TCEAL5	228.6266	135.6647	68.83255	142.9623	90.16022	77.58739	72.51459	68.63745	48.11018
RIC3	56.29708	80.14089	68.85844	75.36685	49.92873	64.19519	42.68467	50.12563	76.99292
AUTS2	7922.698	7541.99	4875.731	8121.389	5126.089	7363.149	5345.945	3627.198	7489.407
FAM27L	70.92942	78.02279	34.17671	40.46494	41.61536	41.46619	35.61929	44.73193	37.161
BCL2L10	42.68666	60.1905	50.12747	54.68761	49.10233	54.58292	51.27618	34.06682	124.6241
H1FOO	46.36238	52.41122	40.20953	83.95924	55.3308	54.3981	50.34056	61.41755	58.03894
PSAP	13846.04	14227.95	15901.06	15725.01	14188.88	15428.13	15332.34	16430.85	14138.38
OTC	49.34457	46.97924	37.54862	61.36283	321.2258	34.94201	120.5995	38.29598	44.03472
CALML5	908.3992	3302.437	660.2188	1418.251	2512.515	1061.153	1250.476	773.6077	1269.69
ENPP3	161.0287	56.01062	53.83815	39.42702	49.49152	58.58537	50.17205	46.07803	99.93777
RXFP1	48.68016	59.68125	39.33607	40.35155	42.99852	33.8097	44.58754	42.1427	50.44796
OR2AE1	53.46696	41.16237	35.79995	42.30618	36.4603	48.79242	41.35897	45.67497	54.42957
RBM35A	1702.975	107.1688	561.7957	1495.833	493.8029	82.30973	181.2989	91.82631	90.41502

BUD13	409.0842	345.0298	430.539	569.2944	757.9929	332.597	567.6594	849.2723	617.3547
SDF2L1	23245.66	25521.84	22140.19	23554.17	25066.04	23117.91	23444.01	25750.47	23895.88
TOPORS	1071.478	44.58029	538.5194	50.41861	585.3156	449.7855	549.3824	355.1988	53.52747
MGC34774	272.4308	178.3537	68.33179	113.2603	103.675	349.7818	65.86325	65.5589	351.1305
BANF1	161.7881	452.7573	256.0103	103.6223	138.9025	158.3991	168.2739	407.5368	36.95868
LYRM1	8182.798	8229.692	7994.743	6434.461	10063.31	7769.035	8072.233	10785.34	9506.895
TUBD1	1940.096	1741.209	2303.838	1304.042	2240.768	2039.135	1981.609	1851.344	2122.622
TLR6	388.6815	222.6943	101.3106	216.6754	225.8899	170.5346	96.23636	103.8997	94.3704
ACTL6A	4244.472	6113.708	3443.855	7333.151	3179.767	3337.245	2941.838	6136.575	5795.277
C1orf222	151.5977	134.3968	73.88596	112.165	57.10075	72.37074	63.75483	60.39397	335.8427
DPYS	53.23387	72.77741	42.64904	53.16515	47.19751	49.34972	44.97771	33.96489	47.44332
MAPK13	245.9847	2431.698	8476.91	2054.653	13448.3	4679.782	8409.84	7273.38	426.7881
ANKRD29	124.0206	58.04338	128.2912	78.65246	218.297	247.6671	305.9216	51.26042	78.68598
LOC554226	59.32169	49.82137	47.25459	41.35662	39.37218	46.3554	40.47955	59.44327	35.50306
ZNF322A	52.03233	58.00609	66.36933	47.52819	49.21321	44.62495	48.74306	64.92116	43.31409
PLXNA4B	41.07543	34.7826	38.07289	33.54604	38.60639	36.25294	45.71078	271.2799	31.61119
STX5	12472.12	13212.49	13207.02	11677.91	13093.12	13176.86	13988.96	15479.47	13438.4
CD28	44.61883	712.0096	339.0476	411.6116	102.367	461.2856	156.2338	312.2709	43.05316
HIST1H3H	51.83416	1371.81	58.61646	47.90229	266.7869	40.64156	47.65894	346.135	156.745
GGT5	212.8296	100.6051	76.90856	114.9087	195.5128	102.9565	401.6622	82.14125	233.9444
TNFAIP8	246.116	72.68344	86.04862	81.44769	80.08392	126.6623	70.27906	89.59158	319.2976
LSR	391.3214	1127.377	712.1103	2316.908	627.4346	572.6329	580.3177	1031.047	1604.907
WFDC11	62.17309	54.00147	47.69576	43.58593	46.33398	36.12036	48.94083	48.89367	57.42688
TSPAN33	9829.199	8950.164	9990.735	4174.28	4731.844	8074.268	5780.5	9615.531	9985.998
KIAA1191	374.5796	216.4734	317.9959	308.3941	285.4246	254.3044	192.1327	184.9428	526.8838
KCNJ1	52.0569	54.85692	97.0442	55.5163	56.2395	156.54	45.48439	76.50821	47.11199
MRPL32	864.4771	997.043	949.1766	698.0488	1242.341	988.3408	1060.275	1105.877	626.5682
RPS9	13479.62	9761.661	5688.852	10295.79	4280.355	5135.033	3692.958	4707.114	13487.37
VAT1L	402.9894	221.6161	112.7094	247.4322	111.4634	179.8513	108.1656	139.9854	434.7662
ADARB2	1279.243	185.5394	176.6578	234.392	242.536	264.0171	155.2986	290.0873	374.1056
ZNF215	2088.134	6226.624	807.5875	2273.761	2089.61	1966.104	1072.94	676.4213	1562.911
CELA3B	111.9474	135.0977	68.94997	102.5387	62.37784	54.5216	53.70852	87.81986	130.5769
TMEM225	96.90352	82.79636	54.092	42.08673	39.35168	88.89645	47.46426	68.5745	49.36602
SLC35E3	1460.776	2345.09	2012.311	3920.391	2211.667	4019.285	3526.509	2549.972	3577.072
CYP7A1	35.96259	276.9605	46.50212	36.09059	39.15154	34.90117	60.37591	34.82091	42.58998
KIAA1285	998.5192	760.5459	483.1912	1005.35	547.9291	747.9493	559.5291	479.489	967.0215
ITGAL	7346.028	10120	12136.49	4527.981	5529.553	9821.291	9087.448	8898.279	6765.567
ZNF778	78.79369	63.62352	55.02329	106.7462	55.15833	78.01126	78.36742	55.91862	61.81195
CUL1	457.4925	257.3368	321.6354	292.6382	582.0338	589.9871	610.9752	417.2007	550.2343
PLRG1	278.1212	171.8164	491.4176	194.1996	768.5627	544.3069	332.2494	344.3565	231.7977
DNAJC22	143.6343	99.51045	56.96137	112.3395	61.40306	57.8746	49.62603	55.4118	53.3274
PDE4DIP	3923.013	5389.375	1992.628	1838.885	708.2275	1684.736	2038.996	3039.147	4239.983
CPXCR1	43.65712	40.50639	62.26565	68.8845	42.68044	48.79328	34.64812	51.87913	139.7913
GPX1	1489.162	231.814	1020.636	751.092	994.3273	174.0314	1032.896	929.0395	439.3291
OBSCN	158.5607	64.75726	751.9174	45.85359	1172.205	56.40041	1463.26	2290.167	264.6326
WNT8B	72.09465	38.52976	33.7345	54.29437	36.84386	56.82266	35.31461	38.54737	55.99615
KIAA1704	1682.954	1466.526	1288.102	1361.433	870.4322	850.8745	731.0726	1370.491	466.7196

NDUFB11	792.1026	377.056	851.7664	638.9725	1401.458	513.23	1536.617	1365.685	833.8624
RNF213	93.62511	101.6779	66.79038	78.28601	137.37	52.28162	125.6151	153.7308	132.6945
NECAB3	39.90034	46.19926	33.46463	51.78791	236.8143	36.31408	116.6215	37.11149	46.89323
SH2D6	44.02671	46.23122	42.69688	52.22187	38.72982	39.04695	37.93922	58.96324	46.88304
NOLA1	155.5962	158.2719	377.2176	1341.837	60.37525	106.49	126.2711	212.5941	78.11311
HIST1H1D	515.0206	328.0361	130.8779	482.6171	116.195	178.539	129.5556	161.8502	167.0772
NSBP1	274.672	66.00295	159.7336	97.58197	49.63341	43.95082	79.84902	63.27365	157.4325
ZNF813	771.3588	310.9775	200.1241	306.6762	172.7419	200.3803	275.1529	42.79716	1094.56
MED28	614.9624	847.9206	1250.823	1642.161	880.4933	1711.428	1278.713	1082.812	794.4752
NT5C1B	46.74226	48.10945	34.39329	50.67514	41.18066	50.307	35.84442	35.06646	33.04575
DLGAP5	75.77246	113.5297	60.62971	57.99951	94.31698	184.2712	114.0853	173.2421	69.98857
OMD	950.2814	367.7769	159.0878	159.6082	159.5921	84.81864	90.20348	95.68409	927.7424
SLC35F1	153.0888	169.9174	85.82566	205.4938	102.0261	152.1849	82.74991	115.409	458.9714
GATA4	51.00402	45.72321	35.42077	983.2957	34.97139	685.1872	55.20336	37.56921	39.67879
FAM171A1	375.3091	229.0225	195.2091	238.4058	581.4197	173.3946	375.0767	155.7251	506.17
FAM124B	42.38948	708.2165	1363.3	704.4739	2419.301	1553.197	1827.965	1960.251	186.4639
STRN	1103.743	1279.27	511.763	1393.014	451.2044	830.7859	280.2054	443.5342	1494.26
EGLN1	105.7278	776.4547	221.0598	58.65501	328.7129	60.43188	126.2158	68.48618	2421.698
C1orf63	3137.825	3160.621	2835.51	2510.367	2029.304	2745.738	1836.471	2609.276	3196.949
TMEM48	110.6979	101.2843	160.7582	53.29099	51.71049	52.37241	121.7273	114.1704	184.9585
DLGAP4	79.05399	800.0362	247.6952	63.55738	201.4239	40.96798	112.4148	49.68655	38.36412
FAM78B	156.2365	330.3433	104.6246	130.6154	109.399	76.95867	159.9826	120.2571	300.3503
NCAM2	43.77629	65.4032	43.51376	52.13217	40.93628	55.41066	39.63309	108.7488	95.75535
CCDC70	279.5898	197.1338	97.62366	218.488	82.03645	134.8778	83.44555	113.7842	394.4852
LOC440313	248.3022	120.8993	67.78273	109.7867	49.9625	73.16756	60.48705	68.33884	94.76982
KRTAP5-11	73.11095	68.62014	62.45567	80.09489	59.08243	87.09805	53.08743	65.49119	72.9499
CD200R1	187.2086	1571.052	873.9045	177.809	448.1018	454.1957	474.6793	447.7614	368.4128
SUPV3L1	60.2539	99.10083	55.72422	46.95509	384.428	61.81108	512.6949	692.0598	64.82094
EXOC7	233.0216	262.0127	81.28716	248.7649	79.68889	109.3303	79.6562	110.6873	615.61
GABPA	1066.747	1162.017	1596.194	693.8313	787.7352	395.2245	925.1246	907.4049	2958.7
FLJ39827	2941.104	3323.342	5240.458	3682.807	4398.716	4168.528	4086.492	2706.313	34.62009
NOP14	10770.02	9188.797	8006.249	8970.315	8256.713	8989.248	8408.647	9585.713	10251.2
PPARGC1A	43.11868	1157.937	1562.037	324.6794	1448.868	2382.011	1963.249	1708.107	74.87466
DMTF1	2372.903	2418.486	1564.622	3319.502	1108.524	1895.972	968.8451	847.8663	2500.896
PURB	2204.156	1654.779	1610.568	1407.932	3493.683	1718.077	2801.814	1686.035	1726.836
VRK2	3019.45	3235.361	1831.184	2805.522	475.4173	1395.823	1186.74	1150.881	4267.822
CLDN19	348.6043	214.6843	107.8506	234.5928	118.2731	172.5813	96.80369	144.4788	453.4284
TUBB4Q	51.95581	34.14691	35.0099	49.2769	36.3752	125.2398	58.23006	41.56613	36.16228
S100A11	21449.99	21261.27	20097.37	22413.29	21640.88	21567.84	21935.15	24054.42	23594.49
EME1	237.94	148.9766	77.04307	164.7605	124.7043	375.4996	143.2629	282.9093	185.3064
GSPT1	591.661	372.9861	289.8707	793.0337	286.911	232.6278	368.6745	442.855	475.4203
C3orf19	1889.262	1623.946	1945.551	2236.81	3072.051	4712.281	3463.608	2528.006	196.3184
YWHAG	75.6796	242.508	120.5676	237.4268	174.7665	125.6077	137.0562	137.8568	485.5308
OR52A1	41.43216	36.31357	40.37517	37.31773	40.20274	35.16948	38.559	34.30434	34.63929
FASTKD3	66.55561	48.3975	157.9488	45.67627	42.31145	41.11544	75.40397	42.95505	45.2248
PRR3	561.7032	380.9178	333.7282	500.6833	288.1884	353.4024	538.9536	385.3558	1231.398
HIST1H2BB	572.668	303.0536	126.3143	419.6542	94.89439	138.6938	81.21983	79.65131	38.59543

TESC	152.431	161.6495	143.3228	67.91564	204.4998	121.5206	112.8746	174.9034	296.6383
MYCT1	54.92339	33.61692	40.09344	45.1239	37.07051	35.98617	33.87722	49.57658	53.58765
PRSS2	272.8799	77.83855	60.73443	76.28725	63.57515	86.52715	59.42308	56.19261	56.65102
PTPN14	51.20831	48.51468	38.75932	39.01931	129.2594	35.14083	105.9231	53.76126	52.80473
ZBTB1	1144.341	666.1773	540.7354	706.7308	270.7987	638.4147	278.2512	189.1244	985.5581
ABCA12	203.7119	403.9261	113.9341	351.6815	342.4086	174.6405	75.98722	170.7313	67.83596
ALS2	1273.954	1526.925	2589.196	1611.902	2727.275	2949.784	3164.542	2114.221	1020.402
MOBP	75.42311	56.29758	40.79263	50.52391	37.71508	39.62086	35.77308	45.44018	41.1781
ADAM7	171.3015	144.2014	83.96462	181.9714	94.19189	95.17406	73.65176	107.902	367.9949
ACCN5	55.96372	49.03569	41.87482	70.15845	45.67633	45.63971	35.85089	35.93586	34.11411
OSTbeta	43.01308	38.23625	41.04552	33.9863	37.8618	38.81619	42.55139	40.44862	32.65282
IGFBP5	2200.016	3600.586	6889.96	2466.314	10240.12	7996.122	12740.01	11355.2	3178.023
USP20	32.03989	34.04076	47.79913	42.33546	49.99004	54.23657	45.89781	43.81879	45.08454
OR51E1	36.96786	476.2281	572.5112	44.88419	456.4756	789.0275	518.0965	478.6379	41.98194
PNMA5	268.0133	61.7244	73.2861	100.5096	56.31142	66.87064	51.50431	58.80401	239.7166
C10orf47	82.90199	46.02452	258.5087	33.49575	39.98288	34.69105	35.05508	42.5648	35.22582
C20orf185	45.38503	36.13503	45.11892	61.77524	42.59729	57.41241	48.64444	50.15366	44.964
KAL1	58.38147	40.91322	32.19687	41.02405	40.98927	41.84809	57.01208	52.8207	76.73474
PCDHB17	60.65588	70.92033	60.17882	97.10818	60.68023	98.07407	57.42116	63.26481	105.3869
NPPA	63.39185	56.21828	35.04411	84.72384	42.95916	32.84553	43.23378	47.51552	68.7009
SLC39A6	107.9478	51.43468	120.7946	61.39926	164.0759	129.706	128.3222	364.6928	66.11862
C19orf66	16614.84	17071.51	18257.17	16395.52	10689.75	20436.29	15657.24	16477.26	15860.13
C17orf62	152.9846	160.4679	381.4706	174.7551	644.5728	453.2462	400.9446	380.7959	224.8164
C17orf42	59.13618	566.3295	304.0677	511.6775	115.0065	138.5362	156.6498	80.25814	793.8605
RAB9A	577.8019	453.3546	1427.089	676.4883	1758.958	1712.545	1376.216	987.4541	141.0665
ANKRD36	36.589	38.21471	48.68654	38.70568	38.81751	44.85075	31.87812	42.56543	41.74179
UCN3	219.0829	103.5404	71.62538	132.7718	61.26725	94.00285	67.81398	66.5388	73.85413
ZBTB16	338.8888	327.3099	199.5675	150.4258	91.51861	119.2309	100.6402	201.0878	338.3368
GPR109A	532.6279	506.8528	107.8064	642.9379	91.57161	267.8197	86.79547	102.6338	740.5462
OR9G9	57.85618	61.39327	34.75982	38.42745	41.9759	51.02626	37.00227	39.86286	91.0835
NT5C3L	1441.684	1096.122	1059.043	2101.161	1774.398	1265.83	2130.231	1123.317	352.2032
PI4K2A	4720.564	4031.168	2254.279	3739.003	1838.165	2379.258	2000.06	2940.018	5195.509
CERCAM	141.1872	129.522	147.3566	128.9016	84.69138	106.8944	100.6508	85.60288	55.39045
CLUAP1	113.5617	126.0578	261.5265	784.9457	253.4067	257.7001	191.4013	69.88086	81.50607
ATP2B1	103.9307	87.00112	49.49033	64.27641	115.6608	72.41138	50.4895	68.13905	46.7363
RXRG	923.5454	735.0371	223.229	174.9189	286.4032	371.6935	258.507	288.6754	693.6908
RSPO4	115.4574	80.9297	56.82552	99.09794	61.979	47.49264	51.08189	68.91445	42.50176
RPS26L	385.0202	251.3292	122.2831	251.6675	65.40857	109.84	75.57598	69.65039	140.7324
SRCAP	252.6131	98.18372	79.9324	50.69744	110.3221	110.1328	84.89883	60.45853	90.17421
HAX1	5184.62	3903.821	1686.057	2909.538	1971.907	1343.255	1643.078	1550.799	3240.952
FBXW9	609.1462	578.1712	766.7416	675.9215	533.6019	763.3803	670.611	768.7786	1198.414
SDSL	140.8502	60.81054	1653.641	124.3286	821.9126	1619.599	1882.769	2110.556	95.39906
TIMD4	39.2856	31.90763	189.9869	67.91111	39.13703	38.3449	36.76904	38.39158	56.99082
MPP5	137.3824	148.5966	55.99913	157.544	78.79681	100.116	89.62801	40.79176	133.5927
ARFIP2	183.1076	152.2636	98.83067	145.3348	86.4609	109.8587	67.97849	92.36189	233.8389
SSR1	413.5819	216.2203	123.8335	163.0298	234.2046	238.4775	199.4103	210.4033	373.3347
FEZF2	134.5876	93.59035	74.38544	53.86072	75.35313	103.204	54.03744	60.38883	113.3181

MYO19	38.37404	1247.573	595.3607	843.6153	160.9579	35.06842	915.5026	977.1378	60.27009
TNRC15	134.8747	126.3302	49.40047	77.23346	247.1144	40.66296	219.6035	42.46351	152.7287
RPS7	458.5062	252.1578	129.7029	273.1591	95.03152	173.3221	99.37723	129.9032	609.733
NCR1	251.2285	581.6312	204.9047	145.5603	75.66979	111.6988	75.1994	95.87556	170.2387
MAGEA5	146.8724	93.91867	67.48112	127.0374	64.98264	98.88821	61.50667	40.87372	406.1562
SYTL1	69.33544	56.15516	164.7072	75.56973	64.28532	51.05672	119.0394	57.58319	57.29473
GSTM5	53.74797	55.67454	36.71861	53.05253	222.4055	44.81396	79.21234	40.1673	50.43069
VAC14	60.63832	512.1416	414.6298	55.43085	53.86602	44.47874	163.1808	54.58355	50.87902
ACO1	5417.475	4229.378	3764.115	5717.354	3963.018	4618.481	3728.432	4973.701	2913.474
ARHGAP30	1830.422	1318.026	3636.48	46.09668	4387.409	1809.905	3272.933	3518.791	36.2354
KLK14	45.43744	1190.305	34.75026	70.25297	37.3894	492.3449	38.19723	301.78	65.69337
CNGB3	125.8898	40.52726	54.20784	44.50903	35.01461	37.36357	37.49767	42.2201	62.06847
SLC4A1AP	63.92003	51.27675	105.9764	66.8676	170.2048	211.4923	126.7826	157.7508	63.45221
AMN1	42.68051	47.40905	38.03902	45.75017	40.16331	39.23891	52.60031	35.31893	62.6756
STX17	81.18162	98.43188	59.30278	76.90213	136.8034	49.18376	219.6243	303.0424	75.89401
LYZL6	103.082	76.11076	66.6964	58.53584	59.56387	86.59144	49.04913	66.56662	116.3447
STXBP6	229.606	124.9515	77.12756	89.86914	103.3647	93.78938	60.7781	219.1554	247.8528
SLITRK1	91.03042	50.12896	59.21577	43.94889	42.31598	47.97634	40.00444	47.04436	60.70482
DLX4	107.8855	61.22968	230.8735	80.79674	186.0645	64.48882	95.25607	58.41755	85.95209
TM6SF1	104.2523	78.30824	252.2212	76.00733	41.10822	55.32864	144.7652	61.68201	64.66467
ZKSCAN3	305.9733	576.3759	1568.965	700.2778	1012.382	147.2982	843.1774	741.0591	228.2689
C9orf10OS	42.46175	50.79485	141.087	221.3588	63.10312	237.7899	74.16574	49.35627	50.17645
S100A9	9638.456	9412.732	11327.16	8541.416	13601.57	8298.737	7358.399	11768.49	11532.26
SLC25A26	260.9355	194.8318	92.4875	143.3632	92.05305	126.003	114.9189	423.5365	147.9293
LONRF2	76.39806	95.9211	73.67308	98.44197	75.02974	84.62258	121.3644	98.25323	392.7628
PACSIN1	291.5197	419.7683	1593.465	94.30779	125.9624	670.9829	755.9077	420.759	284.7598
SERPINH1	1226.683	546.8425	1001.413	1355.886	923.1982	480.5478	1673.624	743.6784	1004.891
C20orf86	107.6002	77.97055	66.10787	94.33595	63.28396	89.17953	52.42301	59.45927	214.8539
C7orf25	51.56353	43.6343	195.1527	399.8324	198.5577	469.0444	279.1733	113.186	46.32134
SCARA5	38.13443	52.24841	578.7702	59.31532	2275.602	1576.305	830.9185	994.77	56.64945
C9orf11	56.3633	50.40195	45.95243	68.28078	36.99878	54.03665	53.1902	48.79055	63.18869
SRPRB	461.0103	36.0793	380.5889	343.2309	357.2793	410.3518	341.0115	888.3972	48.57363
SETD4	2324.182	45.3204	94.16353	83.4846	189.1388	80.51144	246.6673	229.7701	97.76907
TAP1	4282.033	9274.563	18244.48	12843.19	9838.728	16202.92	18725.44	15042.81	1573.859
FSD2	52.24301	4730.896	828.2938	68.68825	57.87444	1223.306	710.0431	2766.35	116.795
ATL3	5273.084	4755.425	7776.399	5792.747	6163.732	6041.657	8897.213	8157.835	2473.306
AMBN	61.52556	41.50687	68.61431	44.1921	46.01267	59.73987	44.42382	59.356	67.57041
DUSP5P	51.85485	38.58301	37.84201	39.32799	43.46309	36.20116	35.33096	58.32532	55.42453
CD300E	221.8754	88.27368	70.35196	91.41013	61.39964	75.90128	56.52972	70.65346	143.3722
CREB3L2	411.4853	251.3818	334.966	264.0395	516.9844	173.2977	457.1314	143.6354	568.4188
CCDC48	5356.923	4009.89	2010.136	2391.043	1561.557	2919.729	1591.222	1627.008	5907.934
HIST1H4H	3113.217	2730.128	1731.445	2806.46	723.785	1399.273	896.8013	830.2961	2745.05
ZFP42	308.2607	155.5375	68.04011	276.6077	52.31603	83.39519	55.35243	42.93548	129.6374
C7orf23	112.906	81.03377	119.7201	411.9409	52.71858	75.73103	87.69728	136.4259	77.7953
SENP3	287.7769	193.1555	130.1624	177.8589	131.6098	206.2914	117.2314	173.7604	421.4998
COL7A1	112.605	64.08926	2852.152	2758.881	4982.876	2716.635	8785.852	4911.934	710.7789
ADAMTS1C	146.4587	87.41412	58.27523	50.05236	59.96105	68.16431	54.47602	88.07417	96.65517

IRF2BP1	86.39911	80.85131	162.0459	64.4231	37.76076	69.72254	46.8517	58.11913	100.979
C20orf46	8838.23	7203.886	6055.981	6023.473	9400.268	5738.937	7094.152	8349.101	9144.994
C1GALT1C1	102.6082	162.6532	117.8349	301.8035	146.6017	212.0327	133.2461	102.1458	140.513
NDUFA4	6328.146	9008.411	12590.95	8736.22	12484.03	11524.57	11810.94	11977.06	6429.941
CCDC126	37.98717	44.65282	56.58075	37.34791	305.7639	45.02166	178.8864	909.7256	46.2057
KLHL32	68.63402	90.15511	48.55075	69.77046	63.46332	55.61306	46.21334	40.69244	102.0328
DERPC	1606.261	898.4185	323.0011	760.6646	37.88932	53.43017	483.7742	317.8204	54.17596
FAM83H	37.06784	463.0655	716.8376	777.0897	2493.089	746.3915	1849.518	1492.178	3960.875
RBCK1	12021.42	14999.33	16848.7	14807.89	15072.54	18010.2	14589.02	15551.39	12112.08
NUP133	108.4777	120.8854	285.3537	120.7867	626.563	129.0536	961.2729	499.9354	163.0805
FASTKD1	68.39287	91.44731	124.1546	151.969	369.5047	64.83206	217.244	233.9384	92.32024
HNRNPD	3382.923	2896.417	1577.98	4144.602	4093.568	1920.36	3091.348	4100.78	4654.686
ENAM	47.58497	43.7556	43.14378	43.82056	45.04867	35.8394	46.50082	55.31414	82.13845
RARS	3009.726	4278.882	6484.943	4855.211	6394.703	6013.146	6205.818	6429.263	5301.589
ZNF684	69.20458	46.43056	549.5198	385.0389	674.5933	409.0869	502.676	397.447	64.9943
KRI1	1285.49	625.5677	110.2488	231.468	251.6176	647.9601	104.1759	249.6164	533.8654
SKAP1	2475.399	3415.464	3518.439	1517.427	969.4451	2103.968	1219.954	3059.654	2984.858
ERMP1	2044.509	1923.389	2703.356	2355.009	2489.727	3037.989	2164.394	2335.349	2095.206
WIBG	2300.752	2213.925	4467.616	3180.783	5461.455	3694.762	4835.618	4659.323	906.7509
OR9K2	65.8455	39.22157	40.08088	38.24299	34.23969	42.80864	33.54424	36.53398	44.3796
EMX2	181.5251	72.47423	51.50394	93.67865	68.43542	68.78129	68.2534	55.29974	237.7594
NCAPG	111.4757	125.9176	1516.221	838.64	1626.144	642.9564	2229.864	1979.511	219.0916
PPY	87.53716	50.93251	564.3204	1021.058	62.92875	60.37976	281.4352	326.6272	67.64548
NDFIP1	10399.5	10792.74	8028.195	9606.171	8443.076	9811.88	6407.311	10882.6	6538.21
FEV	40.306	56.39489	38.79919	65.72734	52.17038	49.26246	51.43597	67.82899	107.0015
TNFSF9	961.6093	741.4354	390.631	1561.306	235.0677	931.7345	302.0543	814.8824	1225.04
ARHGAP29	45.32035	59.6403	116.0973	233.9659	89.33164	184.576	202.8617	129.7004	638.5313
ADAM15	204.808	164.7754	79.37987	1016.91	868.5997	90.505	146.2015	51.78673	3113.841
GADD45GI	647.7706	55.4076	845.512	37.79424	818.9321	406.7113	933.3395	1405.209	46.6778
C16orf30	32.79697	61.89324	69.71059	35.24841	50.32103	34.73639	41.54	39.47777	112.7107
C1orf173	51.68848	79.46722	48.83281	72.96321	55.29544	64.52726	53.16398	58.24464	127.6479
RAD23A	974.5895	2503.442	2951.458	1385.602	3434.571	3185.825	4260.045	4148.061	975.845
TLE1	1190.156	150.2881	250.3432	296.4332	889.2419	374.1325	782.8702	595.7929	1063.591
TEK	37.63574	61.93179	44.98051	117.1576	139.5793	40.25243	149.9176	42.99456	64.11951
NUDCD2	53.0126	178.0634	112.1034	34.03589	57.07933	43.34249	51.1573	50.8409	97.99756
HIVEP3	708.0912	335.6494	154.2343	80.53065	138.2943	99.42459	144.954	89.95804	173.308
SESN2	83.08839	45.98547	188.3326	50.2154	62.48937	69.04436	143.993	387.5218	57.34337
FOSB	15852.57	13595.64	10134.25	8251.24	12480.83	15775.83	6474.437	8275.154	19908.25
COQ10B	523.5576	319.1372	478.2589	333.4345	486.9667	611.5104	599.6254	601.8515	758.5255
TRAPPC3	15298.94	15920.16	19121.9	19670.02	19038	20878.42	20005.32	21656.54	16694.36
TDRD10	79.24782	47.88742	35.95293	79.31027	50.91304	72.69705	36.68184	49.00477	68.28782
PITPNM3	47.502	69.50458	40.08998	35.8648	33.45687	38.33891	44.38	42.63098	46.41576
ANKS3	277.3047	94.40736	55.82037	766.8508	51.35282	95.8648	65.58456	78.70444	106.4189
TAS2R38	34.05021	41.1074	35.83188	71.45605	32.65427	37.26299	41.71381	39.16572	33.82893
OR3A4	57.85604	69.5496	46.36374	40.46844	41.48607	54.17293	51.62859	49.41644	71.01262
GPSM1	40.15036	34.63335	42.31665	554.6427	39.77377	62.84309	35.54814	141.5208	66.72888
CHRM1	58.99841	54.20463	56.90443	55.46821	51.54049	42.59285	39.96629	60.70319	49.13377

ZNF17	3721.48	3978.885	4066.239	4284.067	2490.638	5675.173	3781.886	3913.447	3667.304
ITM2B	944.5635	878.6361	961.1223	1541.732	869.7822	962.1683	423.0016	448.8363	47.10667
XRCC1	84.71562	43.43325	1108.79	69.71218	524.885	1135.414	895.6652	1662.38	48.82266
ANKRD41	98.21662	68.93905	54.70665	80.78882	42.50687	250.1782	81.99693	57.94428	52.79438
IDH2	4076.59	6027.623	2493.896	3921.677	1244.561	728.3282	1357.583	2381.04	5691.416
CES3	147.4473	49.2648	39.98369	57.07308	42.73199	55.53663	35.55248	50.54413	49.56981
BCAS2	258.0544	835.4463	342.8029	485.0565	251.6252	214.131	296.2438	268.9435	289.8071
POLD3	1164.855	591.9524	199.5635	78.76345	76.06532	328.8482	75.4364	88.12813	153.4831
CKAP2L	2400.177	2413.364	2389.008	4154.877	3679.163	4217.902	4501.216	3553.29	48.67784
LSM10	102.9755	274.0602	107.5572	48.66722	56.53246	48.42304	153.4546	42.4346	78.70174
MCTS1	612.8834	311.8425	695.2894	64.0979	1217.056	238.5478	1485.714	1586.119	36.29355
RUNX3	272.4785	163.8881	1159.396	130.3651	95.20618	1475.267	179.7224	1022.102	3348.557
LRRTM3	49.70422	52.70343	45.73388	62.49244	39.56437	37.39286	37.50204	56.60907	35.20135
TCTEX1D1	89.51741	89.79211	62.10796	106.3478	320.5476	76.71358	97.05805	162.7435	257.2949
STK11	385.2556	216.9092	112.4446	213.7867	93.79845	181.3352	92.08636	124.6525	486.3645
GPR64	113.7255	71.33064	70.34824	87.74251	54.86898	94.3971	56.06734	69.89178	100.4309
REC8	937.2758	4082.75	5265.179	3364.925	2315.063	4250.131	6742.931	3519.899	2177.215
PREP	43.44889	41.56948	33.65061	33.70853	29.91217	33.21041	32.76299	44.92668	32.93695
FLJ32569	54.5183	58.71693	41.78374	45.27347	50.97668	46.64704	43.55503	47.47239	57.83965
C12orf4	126.7283	76.06899	111.5149	45.80268	129.338	144.2368	169.896	111.7677	56.23835
TMEM163	49.37222	50.65864	166.8948	480.0146	50.85867	44.04318	149.0191	61.37838	53.93587
MGST3	2386.331	1143.234	2343.257	1631.975	2576.242	633.0449	3417.289	2462.026	2688.83
NSUN5	5765.259	5972.964	5174.419	3433.37	3004.969	3215.927	2630.003	4430.964	8697.139
ZCWPW1	1194.808	2130.892	2300.441	2449.199	1541.483	2624.886	1563.598	2147.14	3819.409
ALOX15B	36.28744	34.45685	139.3529	35.1218	35.48539	33.21458	60.18924	131.8235	31.75901
DUSP27	219.2902	127.157	95.87499	159.898	77.6775	74.82536	57.42299	87.97919	183.1164
RLBP1	58.12318	40.83263	53.41574	41.88391	56.56779	53.65443	48.05607	35.08082	41.89687
PRKAG1	55.67461	60.25439	46.6425	56.32983	187.5133	46.99843	247.4587	263.08	162.3845
UBE3B	1463.202	1366.406	1158.431	1394.262	1217.308	893.0433	1118.189	1028.751	1447.922
SH2B3	403.1927	232.029	112.2212	247.6071	149.1054	166.2057	85.5589	152.6272	572.8853
ZC4H2	145.4225	137.0838	90.04597	103.0375	62.66275	117.308	55.82441	66.08894	117.9357
CYP11B2	95.89972	80.63437	40.28601	46.24673	55.30366	44.76793	47.60584	51.13375	57.34784
SMARCAL1	35.26548	1181.511	2351.639	438.5005	2623.834	1864.126	3024.059	2476.322	50.14164
TMEM55A	89.42999	47.96354	39.42616	70.9425	68.77679	44.38754	95.73646	75.5722	85.33163
LOC284751	102.5213	62.16908	37.86198	35.52858	43.69223	40.8589	34.57765	35.87374	36.95527
ERAP1	253.9458	168.9547	86.18108	215.7551	78.55763	126.1665	69.08165	124.0095	375.9616
MS4A5	44.37579	54.90274	43.36377	51.61797	51.32869	47.36047	38.84851	40.30255	45.6053
HCG4P6	46.28756	37.05583	49.7622	47.6734	35.39056	44.84527	53.32199	54.99508	48.94772
PDDC1	65.52187	43.08349	1909.528	1053.464	1758.189	888.7164	1560.081	3382.949	155.3868
LHPP	7023.134	8186.354	8485.848	8157.381	9859.657	9423.19	11483.05	14009.44	8549.852
TMEM60	234.1454	112.1609	307.4933	144.3247	370.8498	401.2348	261.4723	318.7334	310.6096
UGT2B17	44.23188	41.12362	55.27939	58.46551	39.72736	50.35461	37.08342	34.34979	52.33991
OR11H6	71.96826	68.33811	50.59653	63.4381	46.68839	46.36977	49.71082	36.29395	45.95625
P2RY11	11108.34	10454.13	6925.685	5866.161	4463.62	9501.437	4033.06	7268.573	10629.21
TIMM50	60.7812	47.36031	35.30326	36.32479	40.19904	39.15038	34.26654	40.52239	59.19545
FAM38A	11041.28	11230.58	12915.07	12685.91	19331.55	14523.2	19935.46	12490.19	9925.496
ACSS1	60.50402	32.33612	106.8298	45.89224	412.5489	505.1311	525.4094	1014.878	35.61469

SLC25A40	231.881	163.2728	68.01823	125.7745	61.79201	100.8386	72.43994	63.56196	291.6815
KIAA1143	78.23606	50.71736	365.4915	67.02347	88.73829	58.16475	145.6109	51.51093	126.8316
DNAJC13	60.18629	54.11333	154.0474	41.71967	51.2978	46.03658	180.8111	77.89526	65.72585
ITCH	7458.795	7555.301	5279.653	7520.468	4337.061	6408.464	4131.422	3831.509	7409.87
RPL4	3434.046	1769.347	1184.404	2416.691	1072.469	1324.724	813.9091	894.294	3342.884
RAB6B	54.48928	69.98467	44.49029	40.79116	181.3905	35.38904	75.12524	777.9645	77.75655
YY1AP1	17292.24	15558.86	14479.77	15529.45	13640.78	13639.7	12489.53	15069.84	17518.2
FLT1	668.4374	35.53862	46.63595	41.93375	50.16085	218.6682	34.94889	36.02546	32.70798
SLC35E1	748.1665	35.76317	285.9443	568.5012	241.1348	565.4359	340.113	417.5238	75.68025
CEACAM19	294.9148	181.6256	87.2775	187.8401	72.866	113.918	74.5521	73.09865	271.3032
ARF5	90.42629	77.25764	589.3745	74.31773	1733.892	93.12533	1128.546	833.2206	184.5805
FAM13C1	66.82898	56.80235	53.5689	78.51035	288.9461	60.53366	160.031	68.90431	1605.036
ATOH7	38.5248	40.21843	39.06977	35.19986	43.74295	34.1044	38.91793	36.47138	75.81567
CBFA2T2	41.46511	45.00842	43.65158	61.52537	49.91035	38.2786	39.47684	35.29881	93.33094
KCNC1	52.43361	38.23298	38.71259	48.1468	39.12864	37.29764	36.95935	36.79986	35.41561
KLHL11	59.1049	67.5051	206.9657	67.99449	231.617	244.4688	45.26839	117.569	114.9383
CTSH	338.9308	846.0164	176.44	358.0847	247.6888	197.8145	184.9517	136.2977	1436.39
CST4	270.3487	126.4545	84.43848	95.09473	95.42511	116.913	81.05675	97.44408	261.8498
DOCK5	48.289	474.1898	170.0439	85.73837	152.3933	56.5872	279.9788	458.9262	48.84558
DDX4	41.65321	41.85471	39.03472	36.58358	43.98479	55.85522	42.42197	54.5228	40.24232
C6orf134	622.7221	129.3968	418.5079	874.4521	383.6051	1217.887	530.5435	803.2028	68.75867
DHRS3	477.8794	1032.115	1202.586	276.7935	1396.999	1385.775	1531.623	1955.743	654.1436
COX6A2	34.97881	34.12608	36.73231	36.81854	35.65377	48.60368	92.10731	37.758	32.87561
HKDC1	107.7563	47.84602	437.1915	62.49567	196.5324	83.6649	137.9503	298.7354	49.95938
FAM13A	54.61843	41.69455	35.83372	55.85207	41.65375	254.3844	133.5582	33.12921	49.98671
SLFN11	1148.736	338.8568	400.2638	154.5588	466.2423	448.8929	459.9176	475.0157	72.12097
RNF144B	48.95092	46.27678	365.0152	37.56971	260.5913	36.76767	497.7984	46.77066	44.53281
KRTDAP	12279.08	20272.28	19413.53	18749.81	30295.49	21462.12	25246.28	29482.73	13878.43
NEU2	49.416	52.41896	38.5784	39.86555	40.37735	46.14779	43.17448	46.11794	66.41107
FBS1	1719.941	571.2084	773.4775	158.5959	400.7283	583.2966	456.8693	329.0107	2044.92
USP42	48.23969	67.43864	46.04435	81.28977	47.85727	55.99217	113.6298	49.81041	49.29987
ATP6V0A1	9900.916	11831.94	13145.18	11924.27	17874.89	13034.43	15741.34	16413.89	9705.096
RAB5B	1151.717	1026.628	1523.977	1352.753	2195.361	1672.369	1588.639	1952.435	1017.752
TMEM183A	1073.495	1012.353	760.0612	933.4182	646.3825	952.2086	678.5544	766.7021	910.0645
LEPROTL1	81.31216	849.8485	2466.31	89.69025	4306.409	1681.227	3255.527	883.9374	240.0971
PROX1	61.78311	38.69251	417.912	59.57994	137.8775	541.1519	351.8256	115.6995	36.84659
CGGBP1	1106.339	378.6115	526.1895	1571.101	873.1898	430.1467	656.1292	322.2704	1107.845
KLRC1	201.274	149.1041	87.15153	150.425	93.41694	308.4598	80.06791	74.19367	416.9807
NGFR	120.318	81.36665	68.97081	145.004	67.96277	100.7654	57.39725	50.07383	255.9985
ELMO3	62.63298	47.51638	49.83125	52.87848	159.7112	52.01812	236.5019	439.0398	74.0414
DAAM2	435.9922	215.6518	116.8597	246.1783	226.217	175.9314	119.1138	134.7637	508.4843
ALPI	94.23323	134.5091	84.09876	90.04336	63.39999	63.678	61.79365	65.70627	222.9234
NPBWR1	47.6515	51.86618	49.43696	67.94823	45.23303	31.8567	35.74831	32.94304	67.72361
FUCA1	45.38281	49.18713	634.506	397.2046	534.2664	614.6506	652.0583	754.1223	54.12278
TRIM23	482.3426	251.5428	218.8463	304.8896	212.7715	308.5204	252.7561	265.7314	308.9349
C20orf71	93.74777	133.4317	58.25364	104.908	58.14993	129.9784	49.2024	56.58445	117.59
MCF2	50.50157	58.63821	71.31967	80.75204	108.4859	68.7396	68.50079	120.0004	46.81247

VWF	33581.89	32747.21	32145.63	28176.89	33990.13	29020.87	29516.3	32208.17	35012.74
BTN2A2	2899.35	80.57463	2049.447	634.0609	558.7543	1625.261	1171.688	930.4585	1690.507
SAP18	85.7099	66.22792	214.49	68.80626	179.3871	54.02408	90.69631	156.3522	67.81415
CLPTM1L	42.82289	41.99634	390.9142	35.04851	150.8608	33.07012	253.0297	228.7664	39.21797
ARL8B	199.7412	159.6553	490.0923	159.8724	1086.036	1083.364	923.9017	629.4745	331.1226
GRM4	367.741	176.8081	84.94021	184.2995	79.07391	133.0313	76.31561	103.221	406.3926
TAS2R5	58.78198	50.37521	62.26987	112.9667	37.71007	48.6954	125.4843	39.33852	70.31349
AADACL2	87.45225	57.02028	89.66778	66.91319	185.6064	33.98335	42.69103	44.60932	52.95288
STX8	80.18298	53.97443	220.02	45.45522	46.94476	388.087	275.8714	187.5299	133.5867
CDGAP	32.52078	34.9248	38.00911	40.08154	36.56289	37.72403	70.01006	40.7534	35.81678
IFNAR1	1543.672	579.5079	2520.22	1444.352	1951.006	1893.655	2276.477	3055.017	2676.183
KIAA1600	1284.375	1166.028	687.2493	822.4613	490.5574	767.8052	744.0837	503.8749	817.5291
TMEM30B	524.4012	306.23	350.3117	367.9632	746.1245	815.2003	1023.366	655.1763	44.51159
C17orf79	186.9912	167.91	312.7294	197.0473	637.9575	226.6653	717.5334	565.1998	468.8227
MMP28	149.2375	85.7831	65.43985	91.10841	77.6916	471.3058	57.21202	76.34782	406.404
GREM1	82.02455	50.69168	118.2927	113.0198	284.2898	228.7865	201.6772	124.4465	136.5507
LAMB2	70.45543	1066.864	475.7415	799.2228	2070.334	1522.701	1300.065	1487.009	59.17501
RAB2B	3031.975	3248.416	5556.176	4932.04	7287.444	7533.844	7728.133	5419.26	3791.563
RELN	62.73468	52.90474	43.48661	65.97803	61.26355	37.9946	50.38303	55.19641	53.68126
TACR2	124.0326	126.4656	71.74034	80.77377	68.57796	88.23378	56.33081	69.40178	119.4249
REXO1	18382.64	15429.72	13009.91	15397.01	9149.409	15281.44	9900.617	9323.91	16969.68
PET112L	15070.32	13602.2	15169.6	12658.26	13552.69	14870.89	12905.01	13350.09	12871.04
RBL1	33.21047	55.62894	52.58809	64.32076	52.60034	58.11306	42.93039	34.73343	59.57746
MRPL35	5922.28	5340.855	2979.018	6045.448	1223.081	2291.96	1199.987	2046.823	4684.308
LPPR1	119.3592	128.5983	70.18278	93.355	71.52967	64.52034	71.61003	85.52724	165.3952
KIF15	220.616	95.14226	1100.958	1249.092	887.895	774.675	573.595	856.4274	114.8665
RANBP17	36.07445	63.05855	47.50078	35.97064	44.56366	35.53679	36.26421	62.02481	63.30751
VTCN1	75.01222	49.37939	319.7292	63.48351	61.04679	2811.499	625.6762	508.8292	39.36889
CD5	41.99522	53.46718	34.28883	36.75843	48.54571	42.37112	51.44525	34.98697	66.63262
MCPH1	2568.806	150.4116	471.117	2181.274	2052.549	1526.324	1348.796	1386.134	5541.883
SLC9A11	78.62662	94.6516	58.73056	57.61034	54.11823	83.7637	50.27425	56.75765	112.2328
SPATA16	54.29879	46.74097	52.91113	36.51759	49.06911	48.7644	48.14167	45.41068	56.96733
ALK	291.7895	403.6746	168.1098	119.3144	138.993	286.7469	159.1364	180.2419	419.5074
LOC12422(	96.8852	55.05041	42.85554	105.925	51.94753	60.52027	150.166	54.36958	221.221
RHPN2	3005.454	1473.709	1408.106	6532.871	597.9617	1708.548	2484.633	774.2827	2088.989
IPO8	633.2194	50.35759	110.6388	52.17504	230.634	35.45877	164.1141	248.9331	45.66094
WNK1	1212.022	63.41759	580.1257	130.3782	181.3205	436.1649	60.13848	422.886	47.93254
COL20A1	132.4034	84.08462	91.09591	143.5372	58.6868	115.7733	66.34708	294.7644	99.79063
C9orf102	41.83183	563.944	490.8828	38.96542	1072.806	1477.436	865.5396	256.1516	68.25764
FZR1	583.2708	274.1912	85.98385	375.6656	138.5037	111.6952	117.4613	150.4551	983.4241
SNTA1	64.47422	48.71755	234.7403	51.70502	47.67842	297.5819	180.9988	54.6987	54.24731
DNAJB11	542.8594	891.7755	914.6339	679.8094	1024.539	1120.503	899.9644	1158.824	915.2859
DDX10	64.59715	42.09112	173.937	60.27102	352.6826	41.33716	251.3333	128.5686	54.37323
SLC12A7	302.5934	152.7241	96.93427	82.63148	95.01331	179.1285	74.79557	77.97572	289.4802
C12orf76	395.7083	209.9318	193.2922	240.0615	102.1381	158.4195	97.7397	129.3197	509.8044
B4GALNT3	77.42586	62.94315	243.4004	510.7737	182.0123	192.8164	90.14557	41.26046	67.6293
HNRNPA0	94.79705	41.59346	51.04452	79.0156	131.3618	58.87959	120.4651	121.2949	139.6764

PLXDC2	2447.039	911.2634	1811.957	711.7845	2533.401	2167.642	1859.739	1552.481	4039.768
PTCH2	81.59	50.15906	59.92156	91.67911	55.59777	73.22749	56.22059	46.61134	50.91487
SAT1	7538.015	6857.279	6674.022	5839.684	4376.624	5641.675	6802.239	7444.228	6745.253
SCAMP5	54.43305	1136.153	57.71191	40.54442	248.6719	47.08885	155.9821	329.8712	49.74123
DEFA4	196.4513	151.4381	85.2954	146.533	92.62103	112.4789	75.3668	108.5111	247.9747
SLBP	94.29836	51.83688	489.027	49.99614	55.02451	49.57423	47.558	39.57093	150.4146
ABTB2	44.21284	33.91185	42.48616	51.2805	33.81592	35.00125	39.50645	126.4886	36.99126
RASGRF2	35.72685	97.71788	54.52095	34.75325	46.19432	42.3339	161.7081	32.60753	52.61826
ABCA10	58.87054	53.505	30.2894	44.34982	107.0956	41.88012	70.01028	108.3826	44.29804
OSGEP	1541.183	2035.374	4307.415	1350.649	2419.555	2828.229	2443.204	2210.263	1268.34
LRRC23	63.26978	90.70887	56.54345	92.62096	246.224	56.07964	51.27561	501.2544	112.203
KIAA0556	6799.179	5391.218	5181.028	5857.739	6213.345	5638.659	5724.122	4148.908	6865.015
DCAF16	238.1824	143.1044	80.65735	153.6721	82.45343	116.9136	83.38207	58.54906	322.2503
CST1	33.38098	41.88515	48.63386	307.9014	34.91201	106.9318	146.1529	270.9062	84.79006
PKP3	39.79537	53.73307	35.35469	48.36326	121.1621	36.99919	96.85552	174.2878	65.76341
SAMD4B	55.21122	78.1673	446.6856	69.92957	1250.094	34.90375	1251.719	578.4012	58.95361
CD63	449.336	239.4488	212.3316	282.655	270.5687	228.2099	280.4466	341.9902	558.165
SUMF1	45.58817	771.9571	1556.364	1949.796	2684.556	2644.83	2041.435	1103.439	95.49695
FAM86C	794.0446	321.9686	400.0855	374.8053	302.0438	114.3337	229.3907	377.8497	78.19333
COASY	22702.63	23580.87	23608.54	22986.75	22623.46	20768.17	24193.31	20664.31	24493.43
ARHGAP12	76.99488	81.44816	52.36332	115.8104	55.19299	78.35246	113.9744	73.25414	212.1322
SRR	89.4891	300.49	158.3649	464.5951	673.0626	571.9975	535.963	196.539	658.3129
SDC3	54.60776	44.73528	326.0748	1192.863	233.9827	657.2872	360.9834	517.3202	3174.235
HIST1H2AE	78.00169	164.7437	167.6129	518.5837	113.0203	200.1491	219.1427	287.4373	64.24436
TCEAL7	70.14194	41.58536	54.40848	47.02668	45.23866	35.9229	40.30134	49.32838	54.06472
STAT5A	383.4531	223.1663	163.005	396.1823	209.6947	141.0077	150.1231	232.3591	991.9996
LRRC34	73.77608	128.5492	60.12382	409.485	47.59707	71.23766	81.65057	49.64853	162.3012
GP1BA	450.4852	248.319	122.9459	250.4024	123.9462	208.0305	111.9202	183.8248	636.9727
RPA2	4980.529	6944.23	5639.358	6795.628	4722.56	6307.287	5352.979	3931.156	7422.532
KRT8	57.30987	54.84104	127.4929	960.2487	780.9398	30.3409	1509.904	195.7701	52.55223
CDH22	68.20408	133.0277	54.66828	86.10844	106.2046	92.51024	59.98625	63.58475	109.6631
FKRP	209.1317	88.22791	113.9224	117.571	101.834	161.9917	140.6847	143.297	251.4351
PNO1	4775.495	4765.327	4742.002	5138.72	3885.497	4868.275	4335.425	3349.769	3294.021
HOXA5	41.60268	38.24111	36.51577	34.36683	608.1335	540.9388	313.167	38.89666	42.56972
DOLK	238.2053	188.1596	94.87083	212.2002	302.4141	495.0438	352.4393	546.546	267.9297
MTAP	80.95458	2354.999	2746.243	4069.97	1243.529	2368.454	1264.016	1229.064	3016.421
SPRYD5	68.90582	134.2507	70.39137	119.7626	67.44265	113.3447	66.07879	65.26807	200.3462
C6orf118	36.45143	52.11934	54.52779	47.55067	234.483	45.58095	38.644	60.57574	58.29702
PRR4	41.19359	39.35809	311.9895	33.73408	366.246	583.9122	449.4035	35.20099	40.56994
GPR161	100.1791	102.5477	53.54038	78.22771	54.3067	67.87682	51.53511	61.73606	148.4412
SOCS7	107.3671	55.97051	400.8989	669.8154	363.3356	279.243	334.3551	698.4553	877.0931
STX10	195.1458	1002.945	560.022	923.4323	198.8015	396.577	232.2563	181.1958	2163.045
EIF2B1	259.7084	1301.076	1855.617	1064.082	784.3334	544.5941	981.7288	1107.394	2748.378
GTF2A2	981.7535	611.9871	832.2943	609.371	880.0701	849.7875	916.05	762.694	1533.069
KNG1	65.87227	113.1383	50.48642	59.90298	54.4497	63.5723	56.78753	59.85128	53.94097
ASB14	43.56058	41.12997	52.49986	53.81371	39.06384	44.38532	44.04165	51.15694	50.27473
HDDC2	14617.14	14220.16	12988.25	14408.45	12632.01	12966.3	12930.51	15714.43	14495.45

IL27	35.82934	38.4152	34.45225	38.64493	34.40987	65.81857	28.72138	37.85516	40.44053
ZNF266	3516.81	6209.715	8383.705	6357.305	5952.012	6911.474	7604.985	8753.618	3592.972
TNN	50.28723	32.21465	1186.149	64.54981	3004.858	1754	298.953	461.5456	49.68322
ZNF259	76.39565	70.87193	227.4096	88.4703	128.6537	90.98764	57.05653	173.9084	223.995
SOCS4	464.244	318.5256	175.041	311.2789	116.3083	247.2149	278.2201	306.8937	606.3958
PRKD2	533.959	263.1793	95.28085	223.1589	102.4242	131.1043	71.33073	100.887	126.4854
IL17D	2173.575	1155.475	534.9801	1887.343	1217.686	1339.552	1630.07	884.7291	128.0365
ETV4	85.4001	90.24879	159.2971	101.579	426.1338	53.4629	69.58265	130.3629	110.3879
SRP54	1050.463	1705.482	1314.807	1177.058	1079.088	1400.261	1265.396	1323.864	2906.513
OR13J1	55.70591	36.11695	40.6116	48.30624	40.26842	52.34561	53.39606	60.56734	111.6445
YTHDF1	368.13	201.7207	546.7113	155.3401	974.4354	85.44217	487.3456	367.0681	453.9178
CRISPLD1	52.11012	51.20024	48.14671	46.84029	40.02686	37.69749	101.4498	36.70127	63.66694
CDO1	207.5457	138.1133	77.90418	175.1125	74.39001	109.6631	78.61842	105.2755	1041.903
FAM150A	44.21945	96.6225	57.71418	73.5084	52.19463	51.93092	45.56361	45.57093	32.50592
OLFM4	59.66472	69.77612	70.32305	44.7639	51.00554	79.30198	48.51321	63.56378	57.37497
LOC619207	50.47629	60.93984	57.53297	76.90302	47.87667	54.0042	51.10325	56.2954	35.88408
NPTN	242.3359	138.7072	220.3045	717.1101	53.2903	104.5936	68.3742	323.3006	1896.618
IL1F8	85.84302	98.31516	57.30053	76.90132	61.78371	49.85336	46.24734	57.59595	69.97887
KRTAP10-1	158.8255	57.21126	41.84433	36.75918	38.2562	46.82291	33.33737	37.63343	52.9357
C4orf21	45.2132	38.80873	42.61838	57.18027	34.92072	42.60261	42.86894	41.95841	107.8059
FRMPD4	264.9413	174.0246	67.33851	185.3134	71.25644	94.16644	75.21301	91.27798	452.7656
FANCL	490.5446	271.8712	128.5319	289.4111	135.3115	289.9302	132.1818	192.3873	715.2896
KBTBD5	33.14933	31.55434	32.92715	37.07511	687.7482	85.02722	899.0262	1266.154	107.1801
SLC34A3	35.01919	46.01625	38.06711	63.76756	401.1005	292.704	84.43732	178.6795	70.23477
APOA5	179.7455	148.5645	70.54758	80.40975	68.55484	113.7798	57.91565	74.67763	316.3318
BEND3	262.6587	189.63	84.17285	107.7245	86.17362	135.836	67.86083	108.8556	208.2472
CACNA2D3	5927.182	6173.732	5067.795	2951.563	7167.613	7533.134	5545.138	4192.46	7302.222
CABP7	300.7206	184.4848	109.7977	166.0314	127.6774	178.8079	94.77374	125.8517	436.9329
CLDN11	1503.776	2325.138	784.3099	765.033	954.3141	2131.522	1033.545	916.4769	952.4242
CSK	138.5497	4031.897	6986.632	3584.116	6508.011	4742.343	5835.749	7130.655	99.0447
TRIM52	40.24411	608.1501	35.29961	36.15295	54.37753	37.75404	182.9341	42.03094	34.68708
FLJ45079	235.3476	124.9266	78.60399	97.32518	83.35361	137.854	71.46615	84.14675	87.58469
CFHR1	318.6612	114.867	96.81506	116.7831	92.14722	142.4232	76.93417	103.3293	382.3368
CAPRIN1	645.0325	36.12162	212.4094	49.242	38.18915	209.9941	89.88322	162.9669	37.1493
LETMD1	34.3011	35.71686	428.7307	760.5098	910.0091	710.0368	515.9081	1511.825	35.53783
DDX53	162.0936	138.9677	80.29817	100.8525	73.2611	75.50506	54.10651	54.6554	104.2828
GDI2	9291.292	8699.719	8102.844	8355.55	5692.505	6161.46	4864.002	5234.422	9011.435
AP1G2	627.2838	133.4288	125.6345	434.9849	208.2033	218.9846	247.0642	129.2941	258.8774
DMRTB1	162.7688	75.11806	54.30387	100.3868	68.68894	109.9587	60.33492	67.28772	211.2634
RPL31	7971.183	6612.709	3989.502	5995.678	3799.422	3909.374	4111.952	3952.322	9818.429
BCL7B	86.40764	400.4853	384.5316	51.66145	186.2562	702.235	167.8016	506.861	46.9122
PLAC1	260.5309	175.8347	106.6582	203.3288	117.1667	135.5197	158.4537	107.6315	346.5126
SF3A2	8381.948	7395.42	4878.523	6325.043	3502.406	5929.488	2300.302	4081.876	9618.816
SLC35C2	565.5735	1008.616	361.0189	343.6237	703.7225	286.0877	1024.586	256.2369	770.0513
C16orf73	64.92968	50.20948	76.91004	51.52402	48.09559	393.1908	49.66212	42.26163	64.48076
MYH8	486.3748	472.7216	837.8591	292.4873	476.3397	1557.357	588.4392	584.5969	43.26651
SLC3A1	86.07706	68.91267	46.56501	74.15883	57.46956	76.89053	54.30711	73.12101	125.6246

LHX9	48.23373	34.71178	49.87434	44.61435	51.44881	39.74102	53.33596	51.27769	69.28362
GINS3	39.68698	1901.499	1887.068	39.98479	2228.335	2584.597	2175.846	2674.297	51.59298
RPL30	4718.182	3600.465	2475.746	5077.914	2138.49	2360.72	1969.631	3217.457	5281.908
SNRNP48	35.20607	43.91877	35.62444	39.12551	162.6305	97.35211	136.0755	212.9071	76.68304
KLHDC7A	58.08632	72.22771	51.14764	94.94748	66.10521	77.67871	46.49404	69.57503	128.0346
MMAA	73.36487	302.0938	256.6707	264.2581	193.7915	421.9038	202.1775	141.3866	61.26037
CPA1	46.2291	43.97337	40.17269	63.76751	43.5806	32.22052	42.60074	50.72228	93.82766
SPAG8	738.5072	40.02632	56.04251	34.07551	299.2686	45.7221	81.51225	232.1472	87.34412
C12orf72	230.442	161.6366	75.40088	90.97729	67.41402	71.587	63.6041	80.48685	429.2125
NDUFAF1	108.6293	98.12473	62.23968	85.84689	63.75639	79.15004	49.67705	61.73169	98.91534
ADCY8	354.6747	205.728	114.4043	230.8503	107.6754	186.3217	102.6961	145.239	488.1335
RGS9	33.03912	38.92318	34.98636	49.44241	38.33772	43.38086	34.58312	38.81583	39.4784
FBXL20	66.28072	4858.154	6426.695	4026.605	10030.65	6859.198	6935.924	6647.201	45.76488
IYD	41.98058	42.72831	38.36829	60.57999	36.56059	38.02225	46.3161	54.13117	39.40536
IRF4	144.2128	333.0378	117.2279	135.4041	352.5411	200.2784	141.7819	73.2178	112.04
TAAR5	93.61449	93.14687	61.2765	74.56104	81.035	101.2025	40.81698	72.42283	84.87598
LOC12387(	84.02722	43.02887	37.99678	55.35593	46.93938	37.15404	39.59009	45.4005	57.97816
SMAD2	61.24033	50.15516	37.04101	46.0691	33.53214	34.60716	40.43042	48.14562	64.11554
AP4E1	163.8977	169.9603	63.39449	106.0077	62.91797	55.65645	71.16641	59.7136	108.953
C10orf58	406.3001	519.7391	404.9834	256.1929	406.9417	274.0973	192.3394	246.4459	546.5879
MYCL1	282.0677	92.04483	85.9042	158.61	83.3783	102.9075	61.28598	75.25584	231.1188
R3HDM1	339.9039	268.7791	145.6143	264.7907	97.55647	159.9977	107.1323	95.82572	103.8288
MED19	629.4745	334.4869	294.049	349.1344	253.786	486.7285	181.7303	181.3713	711.7043
SVOPL	40.95666	122.4528	45.47814	61.60192	42.11929	34.54371	31.17998	44.11228	42.12792
LRRC10	379.8746	199.6066	102.8095	231.2316	74.96547	126.3639	65.95179	90.76347	485.6951
ARHGEF17	2636.898	1252.271	1130.603	1312.808	1921.321	90.34712	3239.197	2351.927	198.3387
KCTD8	242.133	185.2149	94.01094	159.4659	78.40912	112.3236	64.46514	76.07671	250.938
KRT6C	15442.38	12671.7	16475.06	12438.92	20706.42	8878.459	14515.16	13007.12	9894.029
FRMPD1	40.01363	33.52915	49.77856	46.63613	328.3915	43.46495	32.7618	51.62537	40.83654
FNBP1L	40.7927	207.3818	214.2518	263.9152	373.1406	279.0594	489.2373	164.8005	65.50551
TTLL5	9573.275	9576.995	9345.009	10108.55	8853.877	9798.699	9454.854	11930.8	8537.664
TCP10L	452.824	248.0366	256.4387	186.5568	440.1655	843.4184	238.9361	118.2518	414.0696
ZCCHC24	392.1069	227.6873	120.1634	265.3167	169.623	179.8604	109.6726	130.4065	425.7458
HAS1	85.6448	42.46764	38.94461	77.08834	34.26537	40.37467	114.6161	41.52113	47.9471
GPRASP2	180.467	128.0554	78.76418	78.05947	333.9044	91.79573	373.9001	52.21285	132.1277
AP1M1	13112.19	10177.26	7035.422	10886.36	5346.317	10270.77	7709.57	7490.556	12029.6
SCNN1D	23197.67	28150.13	19749.09	25191.51	23565.08	23593.9	22490	20987.7	26844.82
FAT1	488.17	454.4765	550.5037	571.1209	757.054	1371.199	1233.128	824.1879	598.1389
KSR1	40.27972	72.33515	508.959	56.18054	430.6061	302.4739	299.5624	578.0176	997.8002
LHX5	223.6171	166.006	77.59379	158.8956	62.79939	123.8954	57.43491	63.05468	122.2736
LRRC45	67.97672	271.7747	161.2606	199.0047	433.4059	178.2177	182.0896	644.1108	3100.537
DHX15	63.1086	73.52929	134.2719	46.70118	100.6467	51.29717	68.3084	44.76291	68.14369
OR2L3	123.289	74.17011	81.26598	107.9956	64.75699	101.3793	106.726	83.6965	154.3456
SLC25A18	116.3448	74.50184	48.60866	76.39339	393.2975	103.9481	51.4125	61.62254	94.77436
LOC40045!	40.62589	324.1249	230.7788	412.5943	634.7417	199.3849	415.0462	377.939	52.50978
TSC1	65.90154	84.53783	53.50901	53.95283	89.04326	53.10238	33.49168	43.08395	61.68526
HERC2P2	92.15902	79.44864	277.762	69.28291	101.3468	555.6642	414.0525	303.3719	5113.61

C1orf162	742.4284	302.4507	603.5547	357.054	327.3812	636.7112	514.2132	433.4398	236.3912
WDR88	151.9415	50.05287	62.25768	125.916	78.98561	59.94389	58.57185	52.61592	110.492
C6orf58	40.44716	51.88445	38.75541	46.00563	33.94717	43.03361	35.79942	43.90422	46.37239
FAM43B	1492.57	112.682	383.749	114.6857	664.9266	701.8	198.7485	69.73583	137.5207
OR6N1	43.60898	44.50346	49.07971	36.1268	44.496	33.44237	34.21716	39.71656	51.72806
MYO1E	365.3946	173.8054	215.2439	423.8501	184.7849	205.7784	192.3686	182.535	265.9037
TBC1D1	217.9161	70.61853	63.90315	59.29332	131.7896	67.69863	146.8647	75.15173	52.31108
LMCD1	42.32149	183.9835	199.5805	278.2649	341.9413	314.1976	346.6311	288.4724	126.7009
N6AMT1	69.18266	59.88887	64.07588	43.22709	59.15686	62.96186	47.68367	58.0434	116.0021
SNORD27	1342.179	982.1757	812.09	1181.936	259.8649	388.4984	353.0605	459.3977	949.7487
SLC26A10	124.4485	40.31693	60.1073	77.99921	52.67354	64.55029	52.92075	45.00364	135.5232
IKBKE	62.92866	57.89642	829.8502	69.63755	1070.761	485.6115	776.9066	872.3177	64.40022
NLRP8	56.39714	45.13195	35.85209	40.2031	48.94578	51.01985	37.99075	34.64175	125.2516
C7orf51	182.8648	97.31475	70.12102	99.70135	74.61371	105.4642	53.56182	72.07203	157.0487
ANKRD44	52.14345	36.35854	42.69702	34.87191	40.05788	44.88455	89.93187	49.6032	49.56632
EFHC1	47.25922	42.36034	46.66548	49.75489	268.7362	39.7319	128.6602	42.49861	1037.643
IDI1	322.4804	218.6913	170.5926	299.5083	153.0484	180.7573	181.7442	256.3962	362.1956
HEBP2	2346.744	1819.334	4429.8	2822.672	4459.009	3653.991	4073.953	4188.476	1493.31
C6orf199	39.07024	35.55741	47.72541	37.47137	48.38422	46.78187	34.66456	50.61988	66.96844
EIF2AK4	5486.649	9158.602	5959.63	6000.131	5580.272	5132.483	5375.037	5234.473	8051.542
RPL23	4505.068	3775.61	5514.11	5223.808	5277.167	4003.75	5551.415	4701.99	3627.133
MAN1A2	174.3782	578.5211	816.1155	430.0618	469.2566	503.1889	600.6487	746.9272	66.73702
SPIC	63.69273	95.09209	69.83099	62.34641	75.60715	56.78415	61.3918	99.49652	127.7108
NEK8	3443.458	3570.853	2104.095	2566.358	877.3851	2130.388	1541.054	1104.99	4159.821
PLEKHA4	179.7547	598.671	100.8967	100.3375	175.416	163.6218	80.62511	70.14597	43.97828
RNF180	642.4789	806.3715	563.7738	479.8033	133.6784	48.30437	259.0668	167.8355	986.0184
CUBN	35.38828	42.00036	38.37994	37.37285	39.19265	67.38335	39.10923	36.52853	62.04119
SAMD5	58.47959	35.8843	262.7969	59.21058	505.7099	48.20653	167.1863	35.98352	43.09224
LMAN2L	50.98003	1522.845	612.796	844.7592	2692.496	423.3627	2374.312	2303.377	36.05957
C9orf82	6028.992	4267.321	2890.726	4163.536	1312.97	3848.379	998.3252	2643.381	4448.518
TMEM102	42.96705	37.71977	42.15303	46.01886	37.78701	346.4957	81.58633	35.13796	43.0919
XPO6	399.5307	219.828	110.259	225.0908	102.2745	170.1624	95.11951	138.6278	554.2374
ITIH1	446.1005	294.0819	161.6288	259.7893	133.4898	173.3032	107.5759	175.9497	493.3549
FBXO15	165.9248	168.3863	74.31066	118.2444	92.6881	114.7304	60.35559	367.4574	265.3063
CEP170	572.4381	564.4272	289.5016	947.4392	105.835	306.7199	168.5918	116.5519	847.1458
ZNF217	42.61842	40.96518	232.9289	38.08994	361.1064	436.9712	422.9318	727.6028	53.86138
HIST1H2AJ	1128.143	512.9932	421.6417	1313.488	308.7925	452.1708	377.2261	552.8934	830.1741
AAAS	54.38965	320.5178	287.0665	343.5828	171.722	437.3032	312.9867	346.9947	962.6506
C19orf45	53.69816	41.04042	42.57193	65.6657	40.04206	35.87971	38.28842	40.1709	39.46138
TUB	57.1589	36.90542	38.92956	59.69798	132.1556	216.9739	73.68614	89.93492	43.02551
YBX2	2982.151	1204.415	838.8467	604.2281	797.3381	1974.251	558.2262	1269.59	4117.2
RALA	8066.184	8468.781	13611.24	10332.92	16132.54	12609.88	12965.44	12373.07	6186.434
RAB3A	176.2361	122.9555	144.4274	89.67471	74.30903	96.18611	68.15005	103.8898	240.5766
ARL2BP	576.7205	36.8055	176.6593	336.4659	348.8078	158.9304	195.402	259.7759	100.3148
HERV-FRD	66.84762	46.25593	35.40865	53.68803	46.36377	53.41075	36.29526	60.50565	90.43781
FRAG1	2497.941	1875.742	2394.052	2447.2	2456.181	2559.619	1806.926	2982.751	1420.417
RBMS2	4953.278	3809.829	3180.475	4557.968	1063.393	2163.988	3959.536	2868.056	4262.446

C12orf36	61.02917	49.91128	40.30335	65.71922	42.99829	40.32919	175.5361	43.98047	54.61239
EIF1AX	72.99716	75.79939	60.84965	190.6779	67.58094	79.35114	59.14662	99.66692	171.7619
CALCOCO2	199.6603	666.1522	290.3042	169.615	381.3477	474.6377	432.7256	469.9839	223.094
KCNAB3	83.65907	65.49766	97.86751	280.1393	54.15049	52.0971	69.95709	38.86628	734.8032
PFDN5	2589.211	233.2379	4803.994	1492.233	5288.336	2255.091	5716.627	5879.07	84.63586
EIF1AY	84.25701	298.2328	54.37716	520.0831	53.06501	89.39853	1276.63	1309.813	170.4341
NKX2-8	220.8347	74.42972	69.40375	102.2157	76.65184	127.7724	67.46425	82.65693	201.68
HK1	44.34309	46.74885	45.59515	61.27544	45.87709	49.54355	38.76853	58.06569	49.17491
SYT5	84.27921	56.20384	64.76366	71.50983	54.16313	70.98035	53.65197	65.80633	149.9918
PCSK1N	93.2291	138.0105	72.2522	114.6237	65.66084	73.95486	254.8464	278.8269	325.3594
ANKRD47	294.3865	113.2967	84.11727	120.3273	72.6825	123.6126	192.166	69.31942	235.923
DIP2B	67.38193	846.1986	40.07055	52.30032	184.2046	66.89813	372.3396	58.12306	50.46334
IER5L	195.5911	150.5114	91.50081	96.79435	63.90952	76.59783	161.1551	90.5218	318.5536
TAF7L	32.3127	42.89291	44.96048	41.14974	46.78938	205.1872	38.77425	114.4552	36.41817
MGAT4A	56.57614	506.3613	1300.968	63.97683	597.2936	999.792	599.6879	672.3794	51.35888
SLC16A5	41.29267	3974.164	3586.89	3962.006	7445.4	4704.764	7236.593	3353.756	3254.807
RASIP1	2083.052	2896.786	2603.315	513.2685	6444.156	2516.413	4806.366	5811.604	1697.13
ABI3	3614.314	2171.181	2011.045	1456.205	276.0519	1810.76	865.3828	1521.049	3180.39
RPS26	67.86139	55.04355	52.71983	35.01701	42.99866	42.53623	44.02076	38.58361	54.01334
FAM12A	100.0536	41.14859	58.60947	81.40862	56.87108	66.67596	113.5758	55.51622	83.11953
FAM158A	7703.431	8417.014	4071.522	7439.828	2980.597	5396.382	4058.181	4056.091	8544.077
PRB4	110.4822	76.04402	61.84922	59.88925	57.26263	44.57138	50.69814	79.40143	42.60693
SLC2A2	46.78698	69.91767	46.18713	82.65671	36.06954	42.52977	37.54364	34.42523	44.18936
KDM5B	75.00031	682.9102	676.8307	428.3245	1473.602	1242.907	1443.581	811.8784	96.94099
ARMC9	557.6805	263.9919	122.3626	119.9477	140.2419	154.3148	106.1678	136.0848	276.7046
RPLP2	31132.52	31763.66	32463.32	34065.3	29798.16	30251.15	27181.67	30739.74	31546.14
TRABD	13850.48	14505.86	11671.92	12925.66	9251.271	14143.55	11577.16	11876.61	13379.09
RIF1	37.92023	96.1441	504.8873	43.6666	428.6656	306.9945	194.3226	183.0924	39.54334
OR8I2	63.859	59.81545	72.28374	65.14405	54.59066	57.8288	97.93401	66.49577	118.2145
KRT76	732.3588	56.34589	60.84652	80.23347	100.3326	52.19922	46.15872	48.10422	103.711
MEI1	64.78123	51.92522	31.43536	40.07682	40.20704	38.61299	41.39783	39.51884	40.00245
STK38L	2258.314	1056.886	1315.593	299.7805	1367.908	1402.274	1005.228	1088.145	2766.034
BEST2	53.85023	78.11436	51.74378	37.46642	224.5194	72.43831	37.07275	54.15031	106.8962
BGLAP	205.5055	636.0858	94.29535	148.4088	81.66924	110.5678	93.83648	97.98151	1878.37
SYDE1	687.7983	496.9908	434.955	378.2327	375.4126	248.2333	559.9192	347.3834	1018.457
FEN1	384.6147	391.3779	261.2913	395.1171	84.66573	147.8575	175.6062	179.93	460.2561
ZNF350	59.44708	47.16277	43.44206	38.12051	45.1241	38.1441	74.23064	37.20003	134.6379
PFAS	3422.603	3373.803	4354.902	3968.74	3016.669	3482.789	4137.753	2442.159	4608.822
OR51T1	143.4254	160.1508	69.51462	118.3766	62.48571	72.44953	55.8927	93.80087	311.3978
EMR3	58.91429	74.33113	56.34074	82.43424	52.1318	69.59181	51.40527	69.126	39.59547
C13orf7	1293.807	903.2428	1030.575	1472.375	471.3326	840.8477	379.3592	876.6251	55.52763
C20orf11	10951.98	10474.48	10808.97	12108.84	9677.326	10851.04	9349.807	10803.36	9479.53
LOC158381	220.0771	183.0026	82.73518	136.4671	76.46933	133.8484	76.6491	107.601	354.6711
DBI	306.8598	148.5275	87.4062	144.0843	198.2933	104.203	73.56669	439.0715	204.8721
GPR120	130.0045	93.31013	54.33289	107.0686	76.68772	86.0499	59.0677	143.6618	52.66345
CPEB3	2079.89	2851.563	4461.252	1698.154	4225.76	3938.376	4051.377	3449.932	62.5703
ALDOC	9327.103	10077.19	14852.31	13870.31	12287.33	12547.86	10455.96	10884.88	9846.669

ATP6AP1L	236.6534	93.49513	75.24584	128.942	77.24246	122.9615	63.65332	73.28731	201.0293
ATG2A	999.4187	2441.556	2317.98	1408.851	3640.618	1845.63	3029.251	2315.349	1512.799
HIST1H2AN	33.0189	51.59161	202.2021	39.48359	73.56091	261.2689	77.75326	195.9474	599.4592
OR4B1	162.3328	90.5144	77.86658	85.92133	55.21724	77.04549	49.75632	70.7307	53.5772
MED31	121.0353	53.12732	54.01297	53.44559	54.77129	75.24779	38.99382	73.04849	241.2695
NT5DC3	384.0607	221.4142	260.6498	688.2206	565.467	392.1876	855.741	487.901	460.3903
NOL4	54.56044	36.38768	36.48452	35.99953	49.77686	43.08607	46.65111	43.88955	36.63149
INSC	58.28717	37.00198	50.673	76.2402	50.25545	66.09054	43.96758	52.43589	160.5644
MFGE8	25955.4	23916.94	22330.29	25421.14	23236.88	25335.43	25684.33	21559.92	25803.46
WASF1	42.65577	34.103	138.1551	40.60156	33.63317	38.61806	180.399	283.4373	46.14917
INTS3	125.3	831.5834	175.1088	114.4444	295.9085	64.40277	481.5687	546.2853	107.1071
RFX3	34.94204	46.02568	48.11938	38.61565	35.07083	49.49457	98.19784	197.021	83.24294
LARP7	1542.928	490.559	953.2786	1770.811	308.9166	301.9234	550.7802	602.345	1079.67
H1FNT	70.53336	54.1111	42.17924	65.82667	41.63541	66.68125	39.46749	56.84228	39.75741
MFSD11	206.6872	237.8344	399.8766	159.0171	319.3326	47.91066	125.584	76.50727	212.3133
EXOSC8	484.1143	452.076	596.3088	322.1939	798.4014	440.6547	674.3577	877.9929	577.1932
BRS3	158.7256	58.79462	70.52387	108.8265	60.08072	67.11609	62.39032	63.78293	68.4393
RPS6KA4	602.0466	71.87476	577.3786	314.2362	621.4565	304.7583	746.4662	453.9658	78.39433
OR6X1	102.806	86.51933	51.91438	48.48745	52.86935	67.58051	56.69821	46.67682	45.72546
MOV10L1	208.3027	76.96645	53.13759	78.24277	53.96666	80.83532	48.05256	55.75846	174.6666
DDX18	50.32775	32.50124	2620.338	1014.451	3496.631	2218.779	3654.798	2674.174	36.24344
C1QL2	519.2603	303.561	159.8092	297.2594	135.2554	216.398	114.4672	177.6443	761.2736
TMEM217	117.7073	497.9189	49.71125	114.1565	135.5142	62.62052	272.8441	186.2289	95.23879
NKX6-1	67.00334	64.39886	72.37222	84.97564	101.5431	97.37627	68.13704	85.35492	56.51037
GPR143	367.2585	136.2942	120.9801	202.2595	202.163	184.9485	109.3131	162.7195	289.6758
FFAR1	145.4353	131.1192	71.26775	79.40766	65.91632	89.35595	56.95248	70.80404	167.4393
PPP1R12B	1411.461	1152.51	180.0758	1198.444	265.8038	340.4626	352.6663	483.4488	1761.942
UFSP2	56.96669	1608.39	2450.568	1734.943	2393.7	2368.714	2085.634	3273.701	72.2998
SEMA7A	1833.674	2487.564	1655.058	2699.128	757.9523	1353.877	742.2823	1096.141	2085.691
CKS2	444.4311	255.708	152.4778	262.8546	144.6966	209.8819	128.9975	174.7052	586.7461
MAPBPIP	67.12882	51.54853	185.6157	38.74109	149.9436	287.2985	167.169	35.42208	596.9734
NDUFS3	15945.71	19637.48	22274.29	16915.14	22348.6	22993.46	23878.58	20724.03	16374.06
FBXO36	1087.167	554.4387	510.1513	1502.485	1124.564	1620.907	1101.624	700.4458	1501.68
RPL35A	339.118	171.5472	92.12225	191.1786	90.10406	128.6969	69.76088	99.62786	471.8257
WNK3	59.68594	49.35491	68.72793	47.1967	54.79272	56.99024	41.63818	40.83374	81.52172
SYCP1	52.74898	50.68171	37.30348	62.24473	119.6795	50.28984	45.45286	136.4143	37.83657
TMEM177	419.2227	220.7304	109.6557	216.4249	201.6934	195.9646	188.452	156.6077	593.4839
PARVA	56.00302	60.77719	71.72068	103.333	59.63153	52.54564	80.78419	74.40189	47.56833
ANKRD37	41.39796	635.6655	526.7516	954.7639	46.35403	71.7701	139.8577	54.1185	54.40704
SNX21	84.93572	642.9075	189.0049	93.81014	506.3641	388.7593	246.2086	294.4896	94.0575
SCRN1	61.96416	1516.629	1889.318	2986.386	3620.877	3177.492	5749.894	2928.25	38.82999
INHA	46.24124	197.3545	65.73132	213.285	93.2179	265.0289	87.94517	69.27145	95.03034
PMF1	33.23357	43.49456	35.90778	44.63459	422.8687	248.9314	197.1423	296.9689	61.63023
IL5RA	385.5824	232.3061	236.0627	214.2385	550.6647	177.5708	241.0168	182.0949	532.9782
GAMT	3240.407	11218.68	5575.899	1913.538	5725.511	5789.231	5125.97	9913.995	7253.89
ISX	33.87572	38.57598	38.74427	49.89277	38.75417	39.7899	40.51785	48.96729	34.76198
CYorf15B	116.697	421.3691	58.09138	106.0425	54.13246	86.07455	117.5727	313.3638	108.048

LCE3A	2977.297	2578.758	543.451	1143.12	7610.183	511.3958	1697.921	2087.683	1996.644
KLHL20	1118.822	1291.334	365.1102	47.87022	519.8065	1075.77	620.6327	1003.049	42.57942
MXD4	22864.16	22392.95	18121.41	21062.45	17949.15	20933.96	17594.47	13859.9	20205.62
TBC1D17	128.8836	45.37622	42.32817	51.33567	57.02978	42.11195	45.80804	46.11436	126.1617
NUP62CL	1784.557	2667.111	2069.945	1579.914	1811.109	2273.691	1829.598	1920.946	913.7612
COQ2	43.88947	302.431	1379.176	1194.531	1139.04	1304.817	1098.338	1509.955	38.47942
NDUFA4L2	3364.897	2221.104	698.8061	1781.524	891.0563	108.5333	390.5869	824.5385	3605.832
CHRNA6	264.0708	132.7402	90.34991	152.986	78.59795	126.9227	66.39713	141.2262	245.8925
SYS1	38.75825	48.75665	209.4873	64.27435	611.2214	714.4853	471.4229	469.1679	38.46157
SH3TC1	2226.875	36.69145	872.6653	1724.019	480.0652	47.1323	1159.596	35.27189	35.8555
STK32A	73.33931	38.15984	49.20106	38.1036	45.10956	55.47086	33.12264	43.35223	53.21013
SMURF1	210.5311	106.5501	97.64383	114.1101	66.99384	97.32034	64.09966	69.91808	333.6159
ZNF526	839.9314	1974.11	1051.766	989.0759	798.9207	670.2926	746.2419	950.2686	1267.654
POGK	1119.281	2457.133	4505.497	1060.572	5742.083	3055.537	4946.483	3945.867	1005.84
LRRC18	174.6499	48.31424	54.3882	53.88824	131.2453	89.7489	92.25903	54.0438	70.15861
DEPDC6	55.40249	58.02602	55.52043	48.76201	47.9013	61.56579	171.5111	349.2534	94.30034
SYN3	58.68443	36.51055	50.3299	59.77306	38.06115	42.76098	94.61945	47.45991	72.78649
DHRS11	6636.281	5135.648	4283.891	5048.743	4916.841	5422.978	4013.871	5239.962	7051.502
CHTF18	222.4645	217.722	259.4072	135.3505	163.8318	111.4789	280.666	409.1301	285.6473
RUNDC3A	45.29946	42.722	189.781	34.41978	91.79346	347.899	173.929	31.60906	44.13116
FSCB	40.93518	59.52054	40.56556	49.85101	45.84943	44.51844	37.08759	41.93494	136.8694
DPP7	95.81178	69.01431	78.63969	82.42191	66.08076	91.17023	64.30736	85.03889	75.22348
MYO15A	79.38042	75.42111	65.32008	47.24845	98.89405	61.29681	67.42641	87.30679	154.3828
LINGO1	65.16058	35.44708	260.1875	37.39842	219.9293	37.35221	240.3572	442.6631	48.8281
EIF5B	3044.168	1887.077	2407.529	2661.703	2948.31	4568.644	2281.05	2898.031	1679.659
CLTC	192.1487	108.5975	107.9234	104.7219	114.6637	133.2461	146.3039	132.2532	337.6716
FREQ	1627.287	1117.601	4498.954	4134.708	6131.029	4994.848	6370.639	7847.107	376.5196
POLS	79.63255	568.381	1696.401	481.2843	2395.28	1500.795	2486.222	1591.271	152.1114
MCL1	1572.6	1348.264	946.1548	1825.688	764.2421	781.24	695.305	905.4581	2821.654
GPR62	59.55498	38.02255	39.19296	45.17679	45.89922	43.76603	38.93985	50.91747	46.04602
C1orf103	65.16981	60.7758	84.74217	83.95654	71.66068	47.38263	50.38036	45.1006	70.84949
MORN2	147.2512	146.2872	142.4234	112.6435	116.3362	92.72201	89.42741	147.1146	212.9679
HIST1H2AE	42.52456	2033.669	1550.114	1074.318	682.7123	1230.664	949.6224	1175.801	49.7836
TXNL1	3736.266	1027.618	2044.701	2866.562	1893.842	2507.372	1056.854	2051.952	3827.496
APOL3	507.6368	327.6016	252.1628	278.5526	221.5522	224.3808	260.2952	279.4517	53.01635
ATE1	887.0174	70.56445	51.87997	45.17408	161.3609	39.87451	96.83462	100.1802	64.30824
FBXO42	356.7124	201.3701	374.6369	493.7051	390.2444	158.3323	329.7462	305.244	260.0132
C14orf179	360.7363	216.0457	170.4282	173.5321	454.802	164.3255	176.1809	150.3076	406.7572
SLC39A9	2716.87	41.08934	757.1125	1412.176	184.8715	425.8456	515.2837	930.7917	63.99124
RIBC1	115.6405	86.19439	238.4924	766.6843	52.7085	76.42737	63.08239	58.20092	91.06405
COQ9	162.1704	663.6989	146.8945	411.2798	454.9419	255.853	314.1768	238.3432	264.78
PLA2G15	50.78661	78.73219	567.1105	47.19802	541.8489	287.5775	1048.325	661.1724	56.02181
DNAI2	39.01667	53.53456	51.53539	32.06566	51.35276	37.16129	38.27221	46.43271	36.78554
RTN1	3104.099	676.2581	1403.137	712.0702	751.4573	987.8144	895.5412	951.5084	68.90265
OSBPL6	142.4519	38.43671	471.7111	61.73773	1506.723	35.68891	620.0706	508.2279	51.50243
C12orf69	70.58034	45.01916	50.33639	66.48988	49.70656	48.06549	42.10457	30.92472	52.59455
GBP6	2414.766	830.0259	2876.5	337.2411	4168.02	1849.894	1654.391	1206.884	1883.907

SLIT1	170.4088	85.22677	52.27481	105.8974	66.59013	49.65982	49.91544	49.9695	118.0526
CLIC1	3828.811	2044.594	1899.103	3190.798	2286.147	1759.814	1732.296	2331.331	1927.234
EPHA8	115.8273	84.90078	74.00388	105.8533	73.90814	82.88142	55.22482	78.12482	107.3741
RBL2	631.4114	557.2933	503.568	520.0467	668.3634	666.6278	519.5604	719.0732	110.3227
LY75	3504.445	3257.833	1988.719	3811.38	673.5558	2939.391	1227.42	943.2792	1678.821
NTN4	52.1759	51.76364	38.13695	42.48246	41.05576	34.44807	39.24458	43.34785	35.105
CDH8	65.01144	37.32282	38.6469	49.68015	45.14355	42.72205	70.57282	39.81921	37.9695
AQP12A	61.34439	92.90926	47.44822	48.35972	50.93044	75.86472	43.43541	72.12064	48.60529
SLC16A1	313.2373	179.4771	133.0901	151.5929	248.7264	120.857	236.4801	136.1617	463.4109
GNG10	37.50841	43.84547	34.67163	35.48637	37.48822	384.3714	140.8443	59.67793	2565.593
SYN2	331.7406	393.5761	97.00427	222.0108	93.34642	161.516	94.84708	110.5217	466.1821
NUMA1	99.81219	95.90823	50.40392	135.0096	71.41726	167.6615	66.15508	91.3864	690.0909
C10orf81	48.51483	925.4492	204.0535	66.40709	116.5777	944.1255	861.7863	57.94038	79.06373
43530	12773.8	15599.66	13511.28	15967.57	16411.62	14010.36	15914.9	14596.04	16875.44
C22orf31	46.47625	43.77703	47.74585	48.14843	41.54832	39.17072	35.35382	40.94191	62.45663
KCNQ1DN	134.3873	39.92836	46.76356	79.72746	61.20448	75.45461	46.27229	67.45271	114.5683
TMEM88	67.68642	1370.398	489.7354	66.88609	672.7521	1260.081	608.1824	783.7156	58.09236
SMC5	47.96906	45.18731	41.33221	36.54961	38.68187	32.19804	41.4009	43.83965	68.3627
MPV17L2	45.17814	69.2066	298.568	109.9335	55.73854	52.40206	150.9773	55.12218	195.1192
C4orf38	94.84561	259.6653	148.0182	100.1508	75.43336	102.1814	63.17583	70.5858	387.3219
MAGEC2	179.7567	154.6766	87.19685	111.7845	87.32279	154.6221	89.86866	113.0008	325.2557
OR5W2	47.2828	54.71435	52.83487	96.65342	40.47625	52.42762	42.35959	40.83815	245.3445
LMBR1L	256.7742	165.4744	116.6364	169.1644	122.5555	154.3353	98.96799	127.4661	190.4982
WDR49	157.1566	124.3671	70.175	117.8117	88.7868	94.42942	75.26833	71.10089	190.24
GIMAP1	65.24518	346.9233	1228.715	421.7928	985.207	983.6419	1248.673	487.8881	182.9183
PLAC1L	56.96048	37.68064	57.91226	66.02116	43.95579	42.67482	50.54475	54.93737	53.32584
ZNF250	57.28236	50.20937	111.8878	36.33065	165.2694	47.68559	103.8917	82.26167	47.05285
DDEF2	877.7109	1073.616	914.5334	2067.836	1354.892	106.7721	1887.786	690.4518	2279.123
OR8B4	102.454	57.77721	65.09996	127.1376	40.2841	76.79606	51.10603	77.90636	161.1376
ZBTB5	179.5225	132.6307	451.8354	192.6011	377.4913	105.9975	161.9671	352.1544	68.62634
DBN1	34.98456	394.2231	38.61493	40.63956	39.61044	39.86733	35.91295	259.1616	57.11586
THAP1	58.26497	508.3011	188.4935	46.06316	40.80976	39.82955	275.3172	59.95647	47.30618
MAPK6	66.21132	84.12599	1727.288	938.4608	2904.072	2115.371	1561.582	2639.89	85.70388
KLHDC8A	316.0358	159.6215	79.06805	167.2278	77.2857	81.78026	66.18204	49.54726	335.9208
PRSS42	512.1617	282.2268	194.2506	328.6691	206.1804	240.8318	157.0863	269.0103	734.1998
PCDHA8	72.43414	92.55585	46.43346	64.96127	60.51184	86.31969	50.38984	76.96362	134.2765
LARS	734.3444	1417.518	1240.095	1443.186	1020.219	922.8161	1160.975	1441.409	1007.434
LRCH3	56.26088	832.1722	1079.465	837.597	36.5176	486.4971	117.2706	259.3413	48.85292
PLSCR4	274.0331	164.5669	98.91862	282.2685	94.64067	128.3145	107.6686	114.6292	265.1895
PABPC4	2951.873	2125.473	1631.756	3475.03	1311.053	1131.895	1793.351	1997.342	1501.438
S100A7	4186.673	3306.14	2635.37	2721.462	3978.514	2616.904	2076.678	2989.482	3630.546
NCF4	166.5839	151.3476	359.1806	184.1259	180.8908	125.4168	90.86113	95.33311	274.5219
OPN1SW	61.27069	53.98997	42.33092	70.0451	49.95356	51.55375	54.43016	52.78363	50.87654
RAN	8848.168	7972.877	7557.472	8846.357	6785.554	7147.64	7448.723	7263.703	8520.373
UCHL1	1669.751	134.5082	1119.533	998.3682	454.3838	682.7824	799.8039	658.4999	1545.405
OCEL1	1031.472	754.2661	2334.347	1150.521	1998.465	1795.365	2235.174	2063.444	1769.223
HIST1H1C	1399.396	879.7422	2157.249	1967.306	2205.934	1920.615	3687.984	3725.502	1560.928

ZNF256	8526.939	7084.476	7039.995	5037.513	4114.683	4821.082	6159.169	2918.649	7103.314
TTLL9	42.5635	144.9223	67.86239	94.53831	60.0216	69.32481	52.46548	59.26694	195.397
C1orf163	121.512	1818.937	2787.623	2197.612	2173.952	1407.07	3242.959	1550.538	1635.136
UMPS	57.00726	61.65618	46.36144	51.97894	154.0403	45.14989	44.88828	155.4876	131.3796
GDF6	42.31091	179.1926	43.48353	70.54562	43.33512	56.98217	42.41165	49.97466	73.02528
ITIH3	277.9483	36.55598	77.72935	65.41092	63.5069	88.6527	54.35214	69.4085	124.0092
H1F0	2762.585	3595.331	4577.028	6532.642	6063.56	4622.519	7165.554	3149.191	1658.09
VGF	89.40161	97.46256	223.3581	81.37707	50.08084	58.07858	188.4722	244.3774	192.8687
REG3A	212.6785	100.8344	45.4528	100.6548	58.42036	89.38877	50.23149	89.48691	54.26179
ELN	150.0168	100.6775	84.85121	164.0516	77.04662	97.63353	63.06937	93.19923	147.9887
CKM	1135.513	3878.2	2803.752	1340.531	4763.383	2638.841	8891.928	10076.3	1100.834
SNPH	55.99822	105.8485	56.13284	98.94162	41.17042	82.28257	53.28525	46.91346	50.38119
USP40	381.0591	157.6647	101.8846	216.0782	84.57419	104.3641	69.97746	108.5	372.7724
OR1D2	185.7182	132.2595	89.94977	171.8549	80.88011	91.16059	64.45152	66.58928	204.0716
PTRF	6769.322	6973.29	6003.971	10120.27	7734.637	7504.756	8771.205	7073.241	6379.013
MPG	40.76786	34.87004	39.57697	37.88212	36.03509	45.25109	35.41051	42.11763	71.34994
GSK3A	436.3681	165.1351	84.61958	151.1064	85.81708	120.0326	60.32844	104.7431	139.5309
TPM2	78.2573	3237.819	3183.265	2369.909	5764.715	4361.665	7857.439	8425.614	61.51745
ARID3C	46.79093	66.18652	49.73423	66.09148	55.9715	56.80867	47.72548	48.71529	41.78393
ZMYND19	2164.13	2639.615	4453.601	4523.896	3243.657	4627.54	4309.396	2886.104	176.1929
GJC3	325.4001	137.3998	103.6651	91.93685	91.60397	128.8408	66.70703	82.63266	254.7073
DBNDD1	44.92023	56.09291	2318.221	35.36848	5937.175	993.9567	1645.66	1953.187	38.12648
C5orf35	45.91768	188.4689	58.68123	41.06559	60.22706	38.00582	41.7245	47.51581	42.5262
UFM1	626.8088	594.2092	392.8932	426.0292	123.4337	592.8972	220.5537	291.7157	65.76231
CAPS	928.9485	609.4399	343.0696	384.2699	308.014	660.3051	344.3912	310.2764	1233.739
NEIL3	42.89967	36.9323	37.3115	48.41681	38.2105	32.03041	45.32745	35.12114	55.24228
TTC27	2227.496	452.9305	2279.559	2723.535	3293.512	2826.503	3271.093	2749.723	1184.872
OSGIN2	76.22552	49.00269	197.244	62.01194	44.78919	40.3815	193.3187	38.44334	63.23594
IRX5	112.6728	1387.084	183.5995	1393.274	1251.141	945.0545	690.2935	55.33597	42.80198
ZRANB1	6823.741	4537.632	3494.755	3840.909	5426.139	4034.065	4746.893	6476.924	52.30111
LOC55423!	73.72364	91.16132	58.49807	56.02294	58.54338	93.13503	57.13514	49.07388	184.2308
SUDS3	585.9957	197.95	109.2937	240.8869	89.05796	118.586	83.65018	99.9399	578.4011
PLCZ1	85.72348	79.22694	55.76215	71.74673	76.93093	60.95242	87.90077	49.90434	76.34856
SAP30L	228.7994	507.5685	1404.473	1153.7	1253.328	1661.952	1349.243	1429.165	949.3271
GIMAP6	148.607	71.53823	52.38766	52.15961	87.27755	47.82253	42.5886	64.21806	78.69263
BRWD3	3530.895	3379.221	2449.497	3442.593	1615.902	2606.195	1865.257	1819.77	1317.801
OR52K1	108.1	119.9122	56.17593	92.0498	64.33675	109.2782	69.76199	71.33324	185.2567
AGXT2L1	198.9382	67.45118	44.49447	59.79136	58.18215	39.69909	55.48043	34.30684	200.4816
ZNF230	76.26904	82.80288	44.94791	93.12933	39.21863	102.1846	44.42302	34.79955	54.49937
HGF	158.1879	130.8385	82.54183	153.9867	80.62254	109.1191	64.88527	97.81274	303.6595
SPIRE1	7745.22	9916.136	7645.74	10177.26	10268.8	8582.334	8960.896	10944.99	7866.542
C11orf68	311.3517	210.544	229.1881	199.1497	142.4106	286.7273	211.6591	270.2711	305.8028
HCFC1R1	4989.112	6712.841	5366.671	6061.02	6184.124	5492.505	5896.539	8647.887	4761.975
PPP1R16A	6989.933	7577.397	7740.888	8060.532	9705.377	8552.303	7642.14	9556.865	8324.971
ARSH	54.86322	43.30745	53.24715	43.38611	43.09378	40.76618	43.68884	38.5043	39.86471
HIP1R	44.17367	49.64915	46.78215	40.55689	36.5699	60.11153	41.16285	43.82836	51.09237
C19orf56	5695.039	5906.134	4629.647	4970.042	3845.448	4747.335	3530.382	4968.313	5797.146

PCGF1	632.6263	49.65605	323.8416	627.1158	628.0135	585.7269	498.2114	527.8333	836.8239
FLJ20309	68.38804	37.96935	230.5473	2139.323	211.4208	894.6758	749.8148	524.1653	213.3831
PARP2	64.93685	55.28559	582.8523	398.2356	890.2267	609.9232	903.5863	458.4299	106.459
ODF3L1	77.41347	89.20285	51.16933	83.10696	348.1909	91.18607	63.95379	426.9841	102.7737
ETFDH	47.34701	46.24487	1077.364	68.06654	702.0583	633.3555	474.4097	694.8851	1853.655
ALKBH1	74.42792	81.4453	71.67082	100.0866	143.6071	89.12518	57.45233	156.4415	107.8598
KRTAP13-2	45.5897	55.77772	53.08989	78.62901	49.62486	57.77041	50.24213	42.79181	54.81673
NFIA	826.5366	39.21516	198.4438	39.15685	201.4646	318.5479	108.6535	49.17863	42.5505
YIF1B	43.95485	496.8372	581.4071	34.60259	652.8229	590.923	943.506	782.1672	36.74406
SELK	40.68685	35.94168	35.01425	41.81895	43.99652	54.20307	39.18874	35.01596	92.11174
TOR1AIP2	1601.138	49.18539	60.93542	38.76688	39.51169	39.13945	368.9288	51.82924	100.2006
RGS4	98.02986	224.4925	333.3342	52.41275	208.2681	62.68135	324.8437	37.46935	151.3313
SNAI1	38.22387	34.61395	36.498	49.39363	37.16692	41.27702	38.35554	52.12547	38.39946
TAOK1	78.45183	1669.207	1439.22	864.1577	2197.774	1329.582	1862.928	1631.115	103.4409
BCCIP	139.1586	67.15183	86.01425	63.85932	82.07009	88.12865	51.51848	70.72475	80.30236
C14orf101	961.0557	948.0748	225.1429	537.3639	155.9052	365.1093	264.9771	113.2778	573.0195
GALR3	41.07928	47.93892	44.1979	45.49959	48.75053	51.75657	44.23774	49.08738	124.5798
LOC64385З	273.1028	203.7891	105.5591	223.602	85.76229	125.7198	79.18135	64.35036	381.1018
PRPS2	343.2896	179.0996	142.6792	257.6895	192.841	191.3934	196.1609	147.5897	488.0867
ZNF791	57.41505	70.59795	62.75914	284.8213	67.32954	51.08522	56.50486	72.45942	53.88748
PIBF1	51.54658	49.99636	173.4514	40.20646	44.88847	54.83418	90.43934	62.58994	43.14712
CYGB	4337.378	4076.571	4556.588	7053.559	4471.579	5624.546	5799.176	5735.256	2919.858
OR6F1	54.29935	55.02333	48.06115	73.38816	74.96455	70.59035	43.19986	64.88543	109.3597
SIRPA	3515.098	3162.331	5420.076	4289.235	5815.823	5740.861	6892.103	5146.517	1997.603
STC1	119.1534	450.2436	608.1648	1265.801	802.5541	275.956	528.2684	515.2114	98.50694
TMEM99	432.7711	322.3186	473.7787	287.6912	640.805	409.27	908.4191	606.7355	545.2186
GPAM	36.99619	51.45386	41.86745	46.76036	38.28243	38.84668	36.19381	35.15712	37.30079
RARS2	798.3187	1553.231	1655.783	1296.615	1833.795	1969.162	1099.385	1705.973	3323.073
QSOX1	849.3378	1319.012	534.0491	1466.562	664.0247	822.3335	311.6269	587.043	3040.55
GGCX	1152.198	42.41951	224.1661	42.11733	439.2057	376.2446	724.2546	555.1725	53.01787
SCAND3	106.8996	97.04287	52.75681	63.47717	65.22269	67.83724	54.94767	54.67231	107.1061
TRAFD1	295.7621	687.0471	62.55645	85.13466	145.8228	99.89037	95.12429	453.5534	289.8825
PRLH	93.24036	46.56279	46.63991	53.68646	53.82588	47.11759	38.44959	52.00945	57.06308
MYPN	44.0446	1364.053	228.9498	38.2702	49.26265	53.17351	44.19834	275.974	89.30481
CALCRL	61.43513	66.27606	131.0846	70.37168	115.5005	55.87866	108.8909	53.37786	47.66273
NET1	923.9229	1322.205	1110.355	983.025	1704.163	1208.972	1054.464	946.2896	375.8835
SLC9A9	2697.978	892.2329	852.2578	505.15	1085.082	599.0209	296.643	415.1789	2082.448
ACPP	67.57047	36.52773	444.2676	56.01488	786.4256	47.74665	47.02703	215.9789	48.08932
SLCO5A1	73.87365	63.6095	50.98119	55.34362	49.04427	42.85027	53.84454	38.98014	152.3266
HMGXB4	243.3458	153.3384	94.55147	158.0541	92.64341	148.4081	76.17651	82.95354	196.2427
ZNF213	6045.983	4942.989	6781.654	4515.535	7075.914	7161.408	8701.032	7749.416	3500.803
OR2W5	180.4993	135.4025	72.07272	72.4708	74.79434	93.67627	59.71741	95.35857	251.6252
IL1R2	191.3226	81.60034	72.10712	82.14235	86.64746	42.33394	54.77223	59.36539	120.1388
GUK1	781.7181	640.8179	374.4791	918.9946	478.5135	554.1212	457.049	359.081	1172.352
GRIN3A	62.0783	1067.254	175.0273	65.5088	585.5052	534.6649	276.1357	321.0334	73.45438
TFAP2B	1188.594	62.26259	42.22923	40.48504	185.2442	40.63753	54.30094	50.9241	105.2407
COPS7A	2119.629	2289.22	2674.4	1692.78	2500.964	2552.14	2750.108	2250.197	1720.39

TNFSF13B	244.2617	176.1509	1582.637	948.6224	350.2024	1213.621	902.7297	1097.688	263.0249
ANGPT4	43.38983	37.27578	43.04925	46.02234	41.74764	34.19404	34.21086	34.53777	39.27169
TBC1D16	3512.773	3414.468	2670.62	4442.394	1626.963	2559.192	2333.768	1991.693	3572.597
ST8SIA2	44.38513	37.19893	51.263	40.54301	566.3353	1016.641	248.6655	38.25212	40.08496
TTYH1	38.43292	44.85396	53.65867	41.03462	37.97243	68.00336	37.75298	231.3556	45.87041
ABCA3	2618.865	1702.178	1137.646	1318.692	1647.3	1463.762	926.8093	1565.526	2490.933
P2RX1	124.5353	114.2332	1636.256	113.3409	1190.96	1073.648	961.7047	1608.712	80.30376
C12orf12	34.41355	31.18207	35.48389	40.4546	33.64326	35.09559	36.0856	39.30821	35.7636
BMP1	2347.783	1168.385	207.173	2312.627	553.3473	1936.123	1147.95	1149.434	6078.345
CNIH	75.62412	49.56224	297.6983	532.1105	260.3931	517.1724	405.209	649.2695	153.2001
NEK7	787.554	268.8761	320.6189	937.8347	241.7423	243.1359	235.0927	209.5942	241.1326
NAGS	1875.539	1862.575	198.3387	2221.367	102.2549	233.4133	248.6724	499.2113	1467.738
FAM163A	38.80549	56.14904	48.5701	768.2088	38.23287	34.22179	101.5097	44.87324	39.37772
RAPGEFL1	5266.971	5661.273	4358.67	4374.387	6190.676	7061.907	3777.695	7150.093	7239.201
SAG	60.01992	52.18264	48.71786	35.57375	34.72548	48.60415	43.3608	39.18103	40.10146
IDS	4004.594	5804.395	8916.29	6467.073	10949.85	7659.861	9353.559	5176.211	167.3528
CCDC130	17556.44	17733.92	13974.19	15579.49	13820.62	14558.42	14172.16	10177.26	18170.32
FLJ90757	72.64423	56.02793	231.1435	251.977	48.04765	46.88343	99.82669	238.7399	72.91928
FADD	782.9905	363.2671	1091.763	330.1779	1362.907	409.3866	1517.525	955.665	1062.841
LNPEP	33.79661	37.41825	36.61285	37.90922	35.50965	36.99664	51.04986	34.20387	45.0523
OR5AR1	128.7513	124.8844	68.06213	139.7552	59.56721	91.89629	63.4585	74.46493	126.2345
FAM107B	3596.957	3332.131	3089.069	2587.466	2306.834	2612.608	1961.038	1195.486	3177.458
TMEM93	4355.731	4731.344	5895.872	4887.731	6694.929	5914.296	6404.488	7497.229	3924.987
TMEM168	58.04871	37.98368	45.4815	39.29811	393.9644	445.0115	357.0818	671.8729	76.00569
CTNNA3	41.44122	72.06248	31.36933	46.80292	38.21537	43.59294	35.5424	41.70404	37.98739
IRX6	8057.63	5996.235	3333.994	2035.106	1421.734	3313.145	1719.343	2335.859	7635.458
SUPT6H	199.7621	175.3468	86.70632	132.1585	75.30431	139.869	153.3474	110.9809	236.1029
FANCG	4097.629	5460.809	11363.42	7237.599	9048.887	9865.504	9174.43	6008.623	5355.309
CYP2J2	125.025	93.38671	661.0175	72.78735	434.9544	957.6735	1366.056	832.9097	222.8728
EARS2	197.207	1251.422	996.5569	101.2185	618.0785	95.94857	665.6019	658.6667	127.0115
CEP110	38.97144	65.18839	258.6862	37.67494	46.84953	36.02577	123.2377	541.6458	141.2819
TSGA13	72.78617	65.91868	63.59925	98.23356	46.98707	46.11558	43.77185	54.00356	94.10022
C21orf2	9701.103	10295.62	5915.387	5594.46	5515.322	5291.056	3165.193	5265.072	10848.08
IMPA1	446.1317	374.6349	326.4325	413.0249	218.9651	431.0511	293.9662	182.5187	646.6289
LDHAL6A	153.2052	135.2269	66.08694	114.1894	76.76952	68.41958	58.60583	90.65368	64.80566
UGT8	48.78612	52.16908	45.4823	36.33512	176.6825	44.98616	41.20852	39.05568	88.40638
SMARCB1	1346.528	418.7685	823.6631	794.4799	1200.347	516.8184	1018.982	779.3798	116.2196
ABLIM2	194.028	81.41424	960.4419	610.7371	687.8743	75.87064	2398.39	1880.168	112.7719
RTN4IP1	147.3074	440.245	58.49749	78.33006	130.2135	78.94231	139.2314	160.0932	65.25484
JPH4	40.37517	112.2747	44.83427	85.88436	44.1228	64.07537	47.92545	51.23582	117.1548
RNF31	58.38152	827.1873	1512.935	1268.439	1942.512	595.1261	1848.083	2361.392	80.55778
ANKRD40	1644.012	755.2492	373.6707	1891.804	417.6778	1018.115	421.783	750.5203	707.3571
ENTPD7	612.4284	558.3727	621.4969	519.6002	467.7303	486.8791	730.7711	553.2632	801.0724
DAB1	55.16026	46.84598	37.01981	34.39043	50.02511	49.3098	38.32714	54.8677	62.35046
PADI3	279.9056	129.8076	295.4895	183.9641	73.19946	91.22218	184.2447	584.1332	344.3217
CTSK	52.88494	47.93361	2407.567	44.56469	3008.352	522.5097	2785.514	3034.157	83.11696
GNA13	516.5635	212.6899	226.3356	424.454	82.28093	197.9547	142.3926	119.4145	676.4131

PDCD2	549.3979	373.1219	227.6637	375.6402	237.9315	215.2649	172.5603	195.4277	567.1111
BRD2	15430.63	15419.59	15393.65	14326.86	12133.41	16684.79	11981.88	11728.65	13493.32
LRFN2	32.32049	37.15539	39.74483	38.56802	42.75783	39.16656	36.07542	187.1535	87.32291
SLC31A2	577.1686	411.1108	169.7688	377.9929	150.1785	331.1413	286.1917	370.407	486.0187
RIPK2	131.2573	49.15054	1012.566	1083.395	444.707	800.5898	621.7675	379.1146	110.0274
REEP5	11601.45	13195.16	16316.24	11996.61	17625.53	17235.53	18558.64	16363.81	9951.993
FAM5C	109.4327	64.98328	54.15191	105.9031	54.39992	49.69524	42.69534	38.78726	170.0491
NADK	63.65069	70.70781	70.85252	53.34718	68.75761	75.77851	59.9049	70.69467	61.74168
OTOP1	131.6861	95.84295	56.43119	122.2067	57.80391	69.75719	60.99634	79.46153	104.0578
SNX12	2838.027	532.5701	400.981	565.1706	225.0603	526.9884	157.0762	383.8007	51.3259
ZNF689	3546.532	3486.671	3742.055	3308.93	3255.326	2783.206	3118.522	3249.862	3617.09
ZNF333	113.6579	69.70226	59.77982	107.517	85.91758	71.15305	60.6471	127.8401	133.3864
WFS1	4125.137	9416.489	5301.589	5750.676	4218.22	6061.193	5786.576	8317.668	6600.621
MAP4K5	8642.8	7423.812	5903.858	8537.661	5747.544	9506.398	5816.762	7071.496	10327.07
TNFRSF13E	1288.375	7472.667	5944.793	708.6738	5567.292	3584.994	3276.38	2764.154	4763.083
INTS8	5450.181	5814.16	5856.798	5610.556	5804.722	6180.262	7532.516	5235.886	6121.806
OR10V1	62.98964	61.63591	38.05711	56.50948	40.79147	62.85801	39.38099	46.62847	76.62273
DEFA6	43.65346	49.4272	38.26275	43.13913	39.31981	44.57993	31.41818	32.01346	56.95981
CXorf40A	48.49351	49.68111	59.57871	54.26439	45.97576	70.26728	41.92968	46.56628	48.24937
MLLT11	42.0548	48.34925	200.9229	746.1552	449.2419	42.82454	1342.65	1072.352	33.36269
XK	89.0956	68.48364	52.45313	62.83233	37.91096	62.55785	113.5723	206.1799	75.59677
IL1F5	45.05093	40.76399	41.47931	42.34092	36.88522	47.5351	43.91273	45.16762	41.84305
PABPN1	39.14762	59.52122	208.528	147.5782	65.1722	373.0519	195.1021	58.11217	113.364
IFIT1L	44.05811	39.60023	40.37451	51.97645	41.4814	32.68486	39.47064	36.04902	85.8876
DUSP10	2653.117	530.7018	331.7542	1271.894	54.43318	1093.793	596.1195	405.2337	219.5274
MGC16291	48.48494	56.0294	34.35139	56.23551	38.6509	31.18695	37.53223	42.98638	58.6568
PLS1	43.78786	43.02452	174.5788	38.78466	36.93418	45.75975	34.25956	34.39986	36.56451
IRS1	68.48342	131.2984	117.8483	754.3888	533.787	652.7875	838.7584	731.7847	35.3754
OR10P1	41.79107	40.89829	38.34415	46.52372	36.07092	37.39489	41.06734	62.64519	36.21408
TAP2	35.13587	66.38035	290.0605	560.8517	54.40939	59.49746	196.2811	221.402	84.21786
ADD1	17289.34	17342.94	16310.51	17949.15	13852.57	15626.85	14986.5	14880.58	16204.58
C16orf55	41.02667	31.19318	35.91863	46.24311	37.03429	49.07076	94.9028	42.13662	45.11167
OIP5	95.15899	1096.087	1198.947	1266.099	756.5571	655.7385	613.9575	1215.259	94.04501
AMPD1	2270.147	4855.781	1833.342	635.431	1625.542	2862.373	3500.042	4298.639	2930.367
C16orf91	208.2659	162.9456	87.34923	155.0853	249.0917	94.38458	292.2583	210.1802	398.5197
P2RX7	174.4361	87.73885	53.62979	78.046	59.75832	58.58105	42.83704	59.17297	270.3521
KIF1A	4433.166	3452.805	718.5269	423.777	2079.939	1436.51	1141.017	2155.476	2351.383
MAN2B1	2811.475	800.2003	818.421	389.4568	111.5683	653.8129	590.389	1084.507	2149.835
LOC72827€	46.15551	63.47048	34.87989	54.72617	52.16884	55.64644	57.667	62.29729	98.06586
NNT	797.654	610.2015	154.1001	520.2974	100.0707	301.2922	215.1039	161.6281	745.6395
C16orf79	86.38692	64.97805	738.9959	110.5194	638.4985	213.8945	743.7773	461.7997	114.4077
ILVBL	544.8307	55.58307	66.08313	61.85917	44.54145	53.04771	55.00913	56.95551	59.90738
ZFP95	89.384	66.11603	69.81688	92.64966	60.70833	72.32538	55.14566	81.76046	1079.387
NOSIP	707.1879	509.3072	951.1353	1844.714	1099.452	746.2798	1510.37	1514.097	2799.184
TAF7	1274.674	2405.484	2143.18	621.7525	1468.785	2064.646	1356.862	2438.375	135.3146
TAS2R39	278.0565	199.913	119.3341	232.0651	125.4377	178.7309	121.5452	106.1524	362.5467
C13orf35	65.40553	37.94254	33.19705	38.08768	35.45146	34.51733	40.42371	42.06529	37.28463

XPO5	3315.549	1872.253	647.0416	2800.564	883.5264	1163.762	672.1501	1013.095	4099.872
TNFSF15	249.1529	120.7449	74.03767	239.8632	80.62928	145.2301	78.43725	103.6964	289.4604
RND3	43.17148	72.66825	109.3611	58.68879	614.594	137.5853	349.3308	150.4694	66.03987
OR1E1	66.29926	70.6655	43.6618	37.01424	39.95213	37.64826	50.38158	39.72216	41.53904
SRGAP2	449.172	317.9736	243.3119	1100.119	353.6101	227.9685	336.148	185.799	709.8268
NRIP3	892.5994	379.3303	221.5254	1467.12	241.5463	582.6935	208.3581	382.3593	1396.922
CHEK2	69.07995	93.80585	57.79633	74.30329	115.334	56.20639	74.07446	153.9495	82.50763
C22orf29	69.05031	59.47728	326.8221	81.21552	561.7625	52.3052	50.91659	68.7429	60.45898
DLX5	1469.589	723.0213	59.26069	392.7018	1108.175	810.1544	618.2625	631.361	51.23666
PNMA6A	56.16114	36.96978	55.60122	35.46014	180.1836	49.3381	39.61247	48.41395	56.64053
SERPINA4	105.5143	99.74295	73.39252	113.9502	64.64838	91.5197	58.07446	96.10495	199.959
C1orf61	30.66807	39.05187	35.31454	37.60843	96.43987	34.46005	36.22515	38.0748	45.38184
AMACR	217.3391	157.2162	90.21001	149.5076	81.73893	99.63255	186.3241	86.0331	304.6706
HOXC13	383.247	169.3634	110.9551	210.2443	371.3806	156.5679	151.6166	218.9581	521.7546
FAM81B	90.72947	106.3639	61.95847	132.9441	56.6318	70.79763	56.46223	79.53629	213.5181
LOC73136€	49.37436	42.60768	148.8648	41.28471	61.47839	49.22788	144.3625	49.99562	175.1546
NLGN3	47.94926	35.24169	40.14994	51.77349	158.9316	41.68095	107.5649	56.20591	37.96517
NDUFA8	3726.777	5087.925	7119.693	5631.266	7456.208	8156.651	10446.08	7603.521	1432.711
SLC38A10	1222.008	3373.315	2775.671	2006.767	2079.872	2089.641	1233.841	1038.274	45.30318
IGSF22	52.73243	56.34118	41.95074	67.99561	101.0308	43.3984	47.75602	49.28168	48.87463
CTXN1	3550.506	2768.384	1152.958	5002.409	2707.645	3498.047	1761.959	850.1479	5343.94
LRRC56	4100.185	2753.911	2369.977	3264.125	1250.449	3245.335	1723.661	2411.456	3135.512
THBD	51.26059	56.14454	282.7566	41.22481	1955.978	1230.16	836.0453	1351.054	39.87512
CLIP4	94.9528	54.43681	63.43929	57.96418	46.86967	51.03584	48.75976	72.75826	76.04815
DPP8	131.4986	64.19703	107.9945	81.84043	159.4146	61.01339	103.6776	51.01298	183.9877
SORL1	35.53011	63.56279	78.36952	271.6471	46.79261	85.52221	48.19527	49.51408	536.5656
HARBI1	242.5675	38.37671	35.05462	103.6369	191.2123	44.04724	254.9504	321.1318	37.33662
RTDR1	60.38592	563.2875	36.72104	32.91221	41.24485	42.72602	34.95574	36.60954	40.10829
SPRN	55.20039	43.02923	42.40458	54.49699	35.49791	46.02189	43.04453	61.09483	53.30964
SERPINA5	520.4939	251.3653	149.5751	160.0887	120.6004	107.6044	160.8689	151.8749	531.9235
ARPC1A	1385.492	711.7172	595.9	474.8586	1350.128	506.2434	1246.616	646.676	726.7648
CTTNBP2	52.0295	34.63435	38.4879	33.45658	41.59425	36.60001	44.19681	46.11926	34.91956
MS4A6E	52.18356	52.29747	51.72886	91.43517	53.14291	45.5109	42.21901	49.43626	51.30573
RNF5P1	951.6303	151.8271	242.8652	894.1295	79.15603	111.1715	62.60208	90.72253	377.2033
PSMA5	1409.26	1112.035	4147.826	1763.665	4603.858	2515.762	4543.501	5827.12	1285.053
PTMA	1938.256	2886	7744.747	4105.84	10393.94	7254.984	8041.64	8911.789	1092.763
MYLPF	658.009	1037.992	169.1088	51.55052	301.4177	397.9929	614.1799	730.0645	34.02184
NDUFA5	2760.145	3782.817	2037.793	2297.461	1263.923	2265.584	1462.155	2222.596	3005.786
DR1	426.791	193.5864	184.039	297.5804	153.2737	254.3473	128.2932	127.6449	545.1396
KCTD1	2827.708	2128.988	1624.478	2402.231	1553.012	1931.777	1160.607	2398.74	3068.139
HS6ST1	166.2997	143.0236	79.23449	159.5502	81.21148	87.2754	171.3148	74.91408	200.5255
MGC3731	8896.503	5362.458	9106.159	4658.62	12378.9	9712.943	10040.31	8675.089	5991.653
COMMD6	342.1664	164.7652	65.43886	182.6542	65.44574	155.5125	64.81197	45.77293	218.1979
WAS	10794.53	9516.271	12253.17	7679.074	6183.434	10399.2	8587.148	8776.463	9183.354
HOXC12	35.02718	48.18886	34.84359	43.63449	46.08043	47.78387	38.04845	34.92168	46.39722
PPIL3	188.7039	84.29227	215.3328	120.3783	140.5242	140.7527	110.3138	56.24733	1732.658
VAX2	51.65553	100.8868	132.7819	1074.156	390.3485	242.9938	844.0407	502.2982	88.82925

SERGEF	100.5227	52.86333	43.74079	58.50283	43.54835	72.38675	134.4554	49.70465	34.37073
IFI44	4941.357	4447.896	4536.478	4894.668	558.2545	3664.686	4080.218	3156.735	1430.808
OR2T11	206.3418	142.2166	85.70937	200.462	92.52145	148.4667	81.62157	107.756	122.7517
SLC26A3	55.42326	41.36397	42.3722	40.0689	96.13832	36.36546	36.02386	42.6568	34.96258
CCKBR	33.46582	36.73412	46.67444	46.81239	44.47002	48.43048	35.24379	37.21271	64.00241
GKN1	71.25526	90.77029	42.57727	54.2399	40.21312	42.09155	46.42507	38.42715	82.87431
SLAMF6	1615.565	799.5858	877.0688	119.0778	233.6949	459.2799	240.5108	163.4947	2101.692
RRAGC	233.1086	812.8807	2120.767	1568.85	2475.941	488.6642	3072.145	2836.237	444.359
ADRM1	8614.099	9394.463	8300.78	8870.002	7210.251	7182.373	7643.581	8606.254	10853.88
GATA5	41.00308	67.60384	51.27411	56.62948	38.66426	59.41464	43.19884	50.08811	43.22216
STX1A	333.157	199.9135	95.76878	161.0441	209.2604	133.7833	79.53889	111.03	340.6992
MCCC1	2108.177	1795.796	732.2343	1628.549	1740.88	1241.9	1010.146	1668.368	3302.734
MID2	35.5603	42.38869	56.58276	53.12761	170.861	45.83073	239.1233	137.5178	210.6897
CALB1	35.55698	69.54229	43.051	80.58117	46.76074	50.24319	134.7844	36.03884	51.4577
DNMT1	1969.202	4055.46	12096.06	5860.435	12402.42	10659.95	12880.63	9129.537	2304.884
GDAP1L1	120.6792	129.1883	63.61546	187.8378	71.36807	72.53457	107.5217	71.83377	318.1027
RNF111	493.4039	253.1424	77.62164	65.87253	91.69363	166.2776	71.13683	89.60225	286.5398
TMEM42	388.463	761.6209	1360.204	246.0963	1974.812	892.3438	2275.206	863.8562	499.1929
BSN	3330.209	3044.16	1477.34	152.0342	1308.883	1570.765	1032.383	1485.208	3118.865
CD70	211.861	152.7686	63.09373	127.8702	79.95443	98.50394	77.54009	67.54304	86.74752
RBM22	8163.673	10678.63	13332.72	13235.21	13105.99	14172.93	14559.5	17008.34	11599.8
NXT1	4893.058	5281.769	8285.799	8397.431	9831.063	8995.108	10216.25	7459.49	5221.314
ZNF321	58.09843	374.6523	200.4047	277.8021	165.5565	237.2705	342.52	188.2103	113.2269
OR4K13	79.45	119.8571	57.80873	137.3914	57.81689	73.83382	62.09596	88.22889	249.6485
ZNF417	114.9946	55.46966	81.1292	67.75195	184.9725	404.4823	132.4854	36.71465	1995.488
CCDC8	189.1091	543.6248	97.62664	880.5886	78.24088	125.4615	201.3772	111.2863	423.2977
VWCE	63.19167	64.46989	827.9389	52.06484	353.2672	51.83025	329.4525	943.6954	36.01225
GNB1	8989.155	9823.129	13061.48	12197.67	11261.71	11463.51	11639.89	12124.07	8068.221
PIGL	1149.993	1241.021	1158.057	2132.371	1160.139	1042.748	1462.555	1638.189	2591.114
ADPRHL1	38.31449	67.33585	39.65572	45.50621	36.65497	46.59559	216.4408	51.9539	56.33637
ZNF774	809.5463	312.5635	109.0231	305.6794	96.80181	209.0636	150.4924	85.20827	634.0673
SRGN	4452.241	1974.447	3844.952	3507.701	1614.842	2700.679	1900.658	1605.956	127.8846
C2orf52	408.3838	138.7215	86.48096	194.3244	82.91361	120.7308	68.30869	94.19434	595.0732
RNF160	900.0787	2006.491	505.3289	1854.157	496.9126	259.241	582.599	378.8926	104.7634
DNASE2B	36.4719	54.07131	41.49637	51.14985	41.19715	38.56023	45.0993	46.12754	147.0155
GDF2	194.5316	61.64591	47.61757	33.49695	42.42813	49.99081	48.66157	38.9888	39.20868
TSSK3	35.29058	57.58265	44.80299	54.3803	46.2875	44.22323	44.44421	59.39372	41.3308
CD8A	178.9749	107.4406	123.0004	127.999	78.84343	232.8689	99.3287	141.4979	143.7011
C8orf41	37.71238	259.6026	137.8435	54.31956	388.3149	242.5542	330.0803	294.9148	47.28415
SHISA2	149.3049	44.91273	48.56158	41.77483	47.7824	57.17041	217.3378	154.3093	33.41805
AADAC	76.51657	88.68443	51.78485	118.2678	227.0846	59.66167	62.28894	77.14234	52.26286
GABRR1	50.65924	33.37822	37.62308	43.87482	85.57746	42.29493	34.54509	65.54202	37.45626
LOC38993€	63.90315	66.44212	59.44196	94.42529	60.9416	51.66675	54.34194	43.9001	95.91803
MRPS5	44.34875	64.74831	48.22857	63.83408	266.5976	70.41503	83.04428	117.9484	66.38078
AP4S1	308.8883	168.6254	92.71224	160.9504	82.48824	111.5819	69.47569	196.4663	375.0955
MOS	253.0236	87.35457	78.23858	154.5198	74.37437	171.3918	70.57935	94.6725	146.9658
ACVR1	3202.712	1746.347	1391.247	1476.327	2494.13	1699.114	2427.248	1650.909	1274.772

LIMS2	818.7506	558.102	425.0226	422.2701	837.4597	213.0036	730.5411	801.4247	195.8242
ZBBX	381.4285	220.9756	123.3799	251.0734	126.544	181.5856	108.2238	136.6404	520.5645
HSD17B7P2	80.21708	43.1637	48.76147	43.63367	36.3583	38.87858	35.96206	54.67973	37.54599
EOMES	2567.853	8077.112	6139.564	4349.877	3045.014	4965.864	4232.921	6403.678	1956.121
DYDC2	58.4727	83.81841	80.56792	77.97705	666.7227	78.02731	58.43343	317.1367	64.69068
CYP17A1	35.71076	35.7465	59.62235	64.01653	50.69975	56.472	35.59659	43.42957	72.88391
BAT2	664.8163	593.1828	275.4502	456.5625	120.417	215.9362	181.2428	222.2573	828.3204
CT47A7	146.3004	59.20633	55.74269	86.30897	66.27531	87.87959	58.68991	73.07587	152.518
RAI2	148.667	70.12218	76.78725	96.21977	82.3145	81.75513	77.00826	73.55727	43.94174
PTP4A1	104.9993	83.07766	69.65153	55.39054	57.71278	66.38226	60.27575	90.40313	76.65704
CRY1	67.38286	1587.673	932.5342	1538.416	776.1175	317.1883	528.344	941.5964	69.35007
C14orf1	252.8537	102.0355	79.79803	149.9547	86.19135	88.18515	75.53492	81.99129	252.7738
IFT80	313.6293	220.0822	103.2091	241.3137	90.98492	129.0815	77.95269	103.6727	150.4794
TMEM33	3765.201	2357.642	2483.252	1040.637	2316.895	4141.918	2903.857	2937.635	1831.347
C9orf86	588.0248	239.3967	340.3556	246.8438	144.0338	160.5572	80.75838	195.1855	1332.276
POLR3GL	2951.202	3055.494	3240.552	2136.976	2089.021	3363.546	2259.857	1971.363	1552.024
MORF4L1	745.2593	225.2426	310.5709	188.2557	480.8309	134.7916	509.3228	423.375	460.9603
STAG3L1	112.1974	104.1933	62.55955	1597.047	48.85213	46.81435	48.11917	81.70003	105.5133
SFRS9	45.3775	421.5978	579.0292	709.1209	3146.527	1841.025	2087.688	1511.416	74.64346
SAMD14	115.3237	84.39685	542.386	790.5082	184.0945	762.098	484.6357	581.1838	64.28542
POF1B	261.4988	187.002	98.09554	239.2197	171.5591	174.56	89.69598	122.771	482.0787
CRYBB2	36.82644	40.53467	551.6014	49.19748	179.632	31.55093	129.2216	33.42173	36.03321
RPS3	8266.552	6590.291	4273.668	10595.31	3215.837	3247.992	3523.333	3729.021	8957.546
ZMIZ2	864.8552	2118.091	2554.588	514.5634	2629.955	1850.044	2380.473	2630.539	1210.95
KIRREL2	39.43965	56.24077	47.83573	39.50385	42.65841	32.20592	42.1062	35.48883	36.16277
TAS2R40	67.01347	111.0388	53.76352	42.66444	53.57379	47.6414	58.43045	57.31764	124.6598
NDUFA9	40.2355	39.61519	430.643	46.69131	1433.229	283.1741	756.8719	1066.579	42.6141
BRIP1	497.4341	292.994	156.2091	316.343	124.4505	162.1692	129.8817	121.1639	754.7422
ENG	3716.37	119.9882	798.5215	743.8643	639.7529	94.00858	920.6187	1202.568	85.82579
RYR2	158.8633	145.6729	62.40145	128.5181	54.49878	60.77752	61.75631	76.3978	163.8999
NETO2	48.69624	837.2733	1671.606	1500.577	2459.151	2220.868	2839.521	1042.052	758.3175
OCRL	1688.061	796.837	353.7801	512.4639	740.1315	782.5176	678.1346	344.9398	72.31596
RAB8A	64.27579	114.2329	2852.596	87.52534	6438.583	2571.284	4492.651	3742.676	42.92963
C9orf4	63.37263	52.92141	37.45196	40.84887	44.34026	57.33537	35.20517	40.46254	40.53487
ALS2CR8	92.26871	57.79944	59.77185	51.4266	36.64511	62.24895	38.03514	53.79014	46.50318
SEPN1	3650.088	3554.2	4840.527	5520.631	6525.602	5352.943	8430.268	7446.924	2413.364
FGF19	124.7507	242.2609	100.6301	102.3251	84.15733	71.30918	64.48728	44.20338	91.55437
GIT2	1836.973	842.765	1601.71	826.8279	580.8615	859.2481	498.3973	1172.786	2489.018
SLC23A1	219.0091	612.4282	145.3631	133.36	62.83897	144.8118	65.96183	196.1303	414.8808
DENR	5414.481	3587.819	5499.416	5340.475	4218.271	5129.225	4388.298	4622.515	3138.773
C1orf100	79.43289	80.10467	48.97338	115.9114	55.91579	57.47559	51.17428	72.54154	177.2044
HFE2	40.28743	40.62364	34.59322	68.51138	43.37129	48.88796	49.13406	41.9252	40.61044
SH3GL3	57.34245	85.03973	324.5079	60.64191	2187.26	991.9687	955.6648	67.11769	68.04052
ACER2	228.4037	71.72381	57.38521	50.38734	53.66815	54.22561	53.28265	50.73612	115.5994
PCCB	79.54102	96.30327	1530.854	821.9998	3169.298	1113.818	2450.901	4100.899	56.08768
SPZ1	45.35203	89.6357	72.59312	109.1562	53.21845	123.9751	47.01996	43.52394	71.54002
PTPDC1	986.4341	531.5425	247.6114	709.6494	261.6075	563.9175	542.5662	1027.229	871.9236

OR5M9	70.16746	67.64799	60.77353	69.43278	56.37197	61.48677	51.6127	41.74995	89.13066
GNB1L	328.1385	94.89999	76.8497	135.5674	65.08097	101.5682	89.51288	160.3068	226.6124
EIF2C3	70.70863	573.3671	465.3383	838.0731	347.6483	504.4786	439.7351	363.4427	72.73468
ACAD11	1703.618	2190.559	2596.474	3000.761	4295.056	3557.731	4519.977	3203.375	1604.597
PAX7	40.72082	59.55061	61.53552	67.03564	43.08915	44.44728	47.97691	52.74305	52.12554
UVRAG	196.1684	342.0031	125.2258	353.0006	91.35873	53.51772	121.4227	71.81572	444.2599
HNRNPA3	2826.309	3125.125	1614.245	2854.461	1489.854	1494.109	1077.398	2302.248	3869.566
FNDC5	48.62948	224.3148	132.3887	89.07612	423.3554	343.8734	593.9238	845.0859	71.71769
VPS54	41.77872	42.53397	44.09182	35.01727	85.50008	33.45811	42.53037	35.20526	40.92356
B3GAT1	103.4424	65.96436	62.88232	38.01419	40.45073	38.20835	39.83117	41.51389	41.68336
CCDC112	98.69425	94.1664	125.2166	84.3893	101.6106	105.2747	54.77664	114.8217	83.37199
PORCN	283.647	98.44597	89.12479	136.0315	95.00729	91.91651	65.51748	102.442	173.0355
GNL2	4715.784	7303.923	8343.653	6790.224	7961.314	6395.995	10189.49	7711.815	3929.54
KRTAP13-4	97.10454	84.31571	50.4253	75.4536	43.4809	49.70113	47.87541	65.34534	75.20769
CSAG3B	35.69859	274.9273	219.6929	82.61043	33.50878	40.29564	40.3447	70.43665	39.58948
PECI	635.5457	1975.969	3192.422	2720.908	3058.067	2814.617	4194.585	4340.49	961.9786
C9orf142	46.67361	2587.113	3600.176	688.6245	3167.963	2749.46	2804.615	4715.667	55.05342
GABPB2	137.286	1416.402	362.9746	1494.952	273.6948	613.1951	350.2525	594.6671	2721.076
ESYT1	5111.478	4644.564	8980.121	4712.928	9627.846	6987.638	9525.349	8522.556	1897.501
HS3ST3B1	51.61516	36.59646	38.98507	47.55857	38.83026	55.62725	104.3135	44.97154	49.81852
RPS6KB2	104.1712	662.1633	63.54523	793.5618	54.20417	67.48172	44.54695	237.2683	79.02854
NOL9	37.50795	34.80486	48.29391	38.47835	40.83605	47.18428	43.30835	38.70591	40.76991
SLCO2B1	739.7781	382.7973	436.8405	304.112	223.6526	124.6429	170.1486	195.1062	945.7814
PALMD	294.0137	194.2241	87.01377	94.78588	87.83685	97.24957	59.90226	96.81889	207.8327
LOC155100	37.85745	42.5023	35.23352	38.73395	35.01186	31.28427	33.94003	36.77049	41.7761
COMMD1	17514.03	16837.67	16315.51	15825.41	16001.3	15489.01	14844.41	17406.79	14822.12
PIR	295.502	341.1417	138.9547	185.162	200.7876	320.5402	161.2356	188.9914	251.6965
COBLL1	11742.32	12381.06	13204.91	11667.28	19739.98	14464.18	16498.87	13553.75	13449.73
MPI	682.0124	77.98475	61.3412	42.45401	43.61528	59.29673	72.26494	42.12792	73.00054
BMP8A	41.33697	44.51406	45.54921	59.16441	42.70708	50.23073	39.16105	49.70451	85.60421
FUT6	138.9154	78.46379	68.09576	97.82234	95.9287	99.84238	127.9044	62.98139	92.30331
C1orf88	92.58918	98.11603	63.71452	118.6397	78.94095	94.01064	59.81572	63.24433	398.5885
OR4K15	133.3536	93.7627	124.5958	135.9692	135.6008	192.5309	106.869	183.7597	45.52689
ADCK5	163.3667	79.72997	251.9393	140.7991	54.88266	95.20113	321.9415	299.1646	210.3571
CRHBP	109.936	71.88335	61.19344	76.59153	55.79687	77.61707	46.67561	49.63782	174.3444
FHOD3	40.07134	36.89468	257.7183	45.19912	33.67704	36.54061	212.4948	39.75032	44.27362
ARID4B	100.1813	605.4821	69.21181	77.85754	245.6207	260.1251	261.8245	344.3842	139.602
ZNF530	50.45858	46.63084	51.29075	36.22947	52.2756	69.9146	136.8991	64.75634	68.80772
VAMP4	45.11769	151.153	97.18008	274.8604	105.8428	193.7378	86.56675	52.60507	610.868
NCR2	50.04474	78.01516	89.29694	96.72967	67.30589	66.60333	56.46654	54.87134	119.604
DAB2IP	54.29456	41.74685	38.84209	36.17925	50.45951	40.60389	39.41562	34.84664	51.3586
C10orf119	4736.667	4118.307	4069.937	5625.354	2627.863	3014.933	3193.258	3183.608	4352.987
OLFML2A	41.7906	93.07111	222.9398	83.08474	1407.125	441.6514	1618.839	434.894	78.97432
WDR24	35.46006	37.10006	44.56816	34.20535	43.00317	49.14108	38.93075	48.53348	46.62951
DHRS4L2	44.89176	68.4455	47.91199	81.57327	125.9199	60.41841	153.8806	136.6355	61.12527
ALG13	108.8683	168.8632	369.1584	74.6999	684.309	222.5544	436.0026	290.7581	397.3347
EFHD2	20513.08	20233.41	22134.96	29546.25	16073.47	27832.46	26215.01	18853.65	21759.81

ZC3HC1	9694.778	9168.299	8230.018	8568.222	7707.29	9807.78	8638.461	5354.389	8296.191
C13orf34	336.5525	225.0365	90.50735	202.0123	80.8784	158.0053	65.20038	99.82043	295.5645
ZNF75D	1301.384	1043.44	656.4397	608.4266	376.6096	371.3779	407.2036	711.755	2240.342
APOH	54.20828	47.09449	43.6514	35.76624	46.86913	48.47358	56.04654	46.96544	56.81645
ALDH3B2	115.4187	425.465	125.5022	346.2638	133.0597	203.4982	58.58201	80.56838	288.1754
EIF4G2	44.17178	40.36174	440.9496	517.6277	916.3627	318.981	473.3484	218.6454	38.94313
FAM131C	50.16075	39.72472	48.34844	64.07364	47.60045	61.38566	42.06649	33.68608	142.2771
SGPL1	2069.713	2364.854	1545.737	2970.661	2222.651	2848.101	2570.177	2491.12	3092.026
SEMA3F	163.4758	136.9897	239.4349	118.1961	288.2635	92.69276	102.5922	83.67809	92.73531
FAT4	324.0979	771.6483	155.7658	141.8163	212.6925	114.3816	168.0633	118.0452	390.0608
PHF13	2491.966	1675.698	1022.422	2019.128	1038.594	1517.472	993.559	697.432	4178.567
C5orf32	42.75562	710.4546	432.0093	882.6948	762.1816	1051.316	888.9968	1006.784	53.9276
STEAP3	123.7156	520.7021	52.13432	59.38775	51.89307	69.42372	49.83436	81.40238	38.12694
FAM35A	172.5767	106.2485	128.9499	125.6157	106.2928	117.8702	81.17751	103.2391	195.8069
PRDM9	293.0721	188.0156	105.0769	176.7514	92.93065	133.3267	73.51942	103.1054	402.8033
CNTD1	57.36222	46.87603	43.20524	50.50137	39.07594	38.94262	46.56455	34.87922	36.51721
CYP2F1	53.09422	43.48692	55.97174	63.29673	38.3305	51.69181	42.28955	50.56071	53.65911
WNK2	111.7682	106.167	95.56017	65.14291	80.94285	83.58043	67.63359	269.7548	2245.213
SLCO6A1	77.67848	52.68634	38.05421	43.11365	34.76787	58.81216	86.14178	42.26574	35.87273
VPS35	1439.952	1002.919	1664.309	2764.234	3036.591	2179.01	2080.025	1745.5	1085.469
ZNF579	275.1937	165.2411	490.301	152.3578	262.3122	413.975	718.235	862.2574	1362.269
HLA-DMB	16446.04	14787.69	15252.52	11145.9	10928.01	13911.33	11937.46	14767.3	17089.99
ALKBH7	68.53088	154.8053	303.0703	101.7058	70.23656	103.0109	166.267	409.1535	186.6957
GPR25	58.76967	35.88035	41.60534	59.44512	40.80945	66.33263	45.94668	38.0226	52.06521
NUP155	1501.748	112.0402	41.59731	50.51935	156.23	352.0439	327.1517	282.675	84.43418
TNFSF8	235.9526	137.5723	91.21485	204.4463	78.31847	140.5184	78.33332	86.35178	184.0696
RAMP3	122.7949	126.6731	90.56983	134.8321	99.28687	103.6172	79.07558	84.04207	84.63937
CA7	40.68037	38.21543	44.05883	46.25965	38.46676	45.78734	41.4562	52.18181	58.66105
CSTB	6215.085	3436.067	4403.125	3407.903	3926.617	2427.269	1947.778	2342.924	6152.821
ALDH9A1	17734.69	16211.52	20854.12	15328.47	21501.57	19604	18383.11	15953.54	15864.02
MCFD2	1799.813	587.7202	845.9189	2595.882	598.325	405.2717	624.197	486.4333	1260.547
E2F4	744.8852	1322.895	2285.978	1877.873	3308.193	3070.539	3723.307	2114.716	189.4723
SLC40A1	182.0512	159.1307	149.7464	90.23109	219.4104	193.6284	218.0779	107.0873	205.4234
ZCCHC17	243.1163	631.3568	275.7771	633.9865	496.3728	75.86333	498.6895	66.4271	69.23876
CACNA1A	50.08254	47.57162	36.20467	45.17373	35.90366	43.93028	35.33108	40.25567	35.14822
STOML3	51.906	59.63037	51.80687	65.12385	159.4062	71.56931	52.84084	59.95978	134.2176
C3orf14	52.80257	47.56709	46.6013	36.57778	44.41974	64.46781	164.325	42.16861	52.7429
KCNS1	2048.176	4636.008	939.7717	7993.358	1889.996	531.8134	436.3215	3100.217	3438.574
SCYL1BP1	1686.951	2984.957	1675.221	856.9313	533.0412	712.6334	582.1193	975.9305	489.4076
MGAT1	381.7384	167.9984	3370.471	178.3083	3352.291	1672.063	3791.168	4463.02	506.2761
LCN8	49.00064	33.30422	34.57962	41.21563	33.2416	33.02079	37.52278	40.76678	37.03873
IL12RB2	64.49058	47.89648	41.65897	747.1408	147.6127	503.027	1390.549	781.5245	75.06512
TIPIN	864.5555	656.3148	1095.47	434.4008	474.5878	759.4731	727.1389	415.6909	50.42739
FGFBP2	39.4483	38.79589	46.09536	53.15367	42.11529	34.89925	45.88767	39.67411	45.34741
PROM2	8202.647	7262.873	5636.896	6460.02	4925.541	6348.146	4739.976	6687.621	7064.883
CCDC90A	40.74457	52.14928	399.7744	38.10628	1339.117	483.8807	758.9463	383.1163	68.07616
C4orf29	52.08998	45.9768	181.4135	56.74171	42.85375	42.40974	50.58353	98.66972	43.7933

ZNF200	40.50775	41.53894	35.50208	52.25883	38.23603	41.11667	39.13552	42.95119	56.67356
PPP1R14A	31539.69	26635.95	22217.01	26783.8	24779.84	24965.71	25389.51	18786.58	27209.65
DERL2	106.1276	52.85139	49.36259	303.5482	124.4079	74.81187	164.6546	185.0021	917.4863
FAM96A	47.63062	45.53436	213.1589	68.35683	299.5295	83.39534	329.1378	34.53102	100.3715
OR9G4	63.84447	38.92724	34.48071	35.54095	38.76056	37.3246	41.02028	32.81038	34.25836
RASGEF1A	60.46582	53.73196	110.0577	281.8606	113.0415	64.90281	67.75023	177.9598	120.2361
SFRS5	11968.86	6253.847	8315.345	5495.106	7216.271	6334.254	5448.367	5735.684	8953.873
C1orf168	103.7988	40.50652	43.38746	46.15994	37.29798	57.14409	41.0091	43.92624	102.5951
CSTF3	489.1888	346.8268	125.6391	273.6803	89.67077	129.2097	95.03382	93.48909	36.99865
SEC62	497.0901	257.4947	179.974	274.2248	294.3889	270.83	211.6453	137.7811	140.7428
FLJ32867	72.06728	49.41495	65.45819	96.77811	47.60057	81.99791	51.07417	81.19703	76.49081
TRIM48	45.61417	68.75184	45.44962	60.72845	38.98573	47.08539	39.33117	41.21554	59.90159
TAAR9	142.5816	120.5007	86.79128	85.78257	56.04182	100.1614	55.55252	89.48694	202.732
NAPEPLD	64.73189	80.47059	42.26677	84.10015	131.4341	50.39616	49.92763	51.10502	88.46475
ZNF551	154.4762	135.5219	96.90733	180.8168	94.05901	123.777	94.64671	107.1018	277.7769
C17orf80	237.0533	170.2547	549.3004	147.2889	389.6207	127.751	439.2531	554.2266	286.7141
JMY	389.26	3244.315	723.3996	2151.063	764.5437	1091.619	635.005	599.2239	4876.025
OR1L6	73.23805	90.85054	37.32279	60.64281	45.86424	35.94926	51.15545	35.6986	74.22526
CATSPERB	34.15598	40.09565	41.641	46.24073	39.06019	33.15764	55.31437	33.92699	48.17006
ABCC12	63.83287	54.15441	47.58027	64.28323	39.97171	149.2	83.4967	114.0561	50.83011
TMTC4	41.04437	81.78326	61.56353	47.34441	67.40757	134.4312	75.08676	69.47158	47.30708
ZNF747	63.31087	70.46109	63.27713	56.24129	51.18005	55.13	112.0018	50.54124	93.87951
WDR48	105.9719	77.41547	340.8371	53.9199	1719.384	418.5791	623.4854	337.5517	64.43578
MMP2	1619.641	768.2426	1088.691	351.7437	226.1965	965.9752	698.6185	280.7198	3358.32
CRYZL1	61.33354	52.07438	46.88927	854.9982	272.1749	67.04337	118.3116	214.1958	46.34361
ECHDC1	576.7187	288.375	111.6907	260.7721	94.62187	162.4429	83.61141	140.0395	607.97
CRB1	254.9391	134.8713	87.54477	171.3274	89.03855	127.8313	76.37204	93.65147	411.8759
FBXO40	57.807	45.13705	47.17143	58.044	39.02141	44.8282	36.76442	41.53637	36.31044
KIF9	50.46473	55.78561	45.2097	52.44593	46.10285	41.70907	41.25188	34.05735	41.33378
C1orf150	753.9014	627.1509	342.7203	461.4001	210.2113	320.949	177.4194	338.0418	813.8329
HTR3B	84.88467	141.0118	94.76905	123.4481	199.9306	123.6859	61.98073	80.38555	223.0275
HPS3	404.4695	301.5875	150.4222	291.0006	79.79785	165.8251	93.47617	90.46942	536.8904
DDX49	50.75341	46.57828	116.1565	338.2925	66.78706	42.64715	69.38136	58.86679	94.1603
ZEB1	51.91154	134.6515	102.8161	304.3022	80.11626	41.41214	72.2494	61.57828	651.4201
EFCAB5	44.56741	51.78657	42.21127	35.33497	36.67355	43.4497	91.30591	69.63946	59.19114
DCTN3	1736.121	2468.97	2152.887	985.6346	2982.038	549.9451	1891.492	1554.512	2905.354
ASPRV1	48.4768	42.12502	809.1728	623.678	4594.577	940.4063	1433.343	2209.717	37.02956
LRRC24	74.1197	50.18004	33.47472	58.46391	44.74279	42.62705	41.15183	49.00044	95.73756
FOXRED1	3699.641	5857.164	6482.454	4390.016	6354.509	5890.596	6510.126	5138.227	3075.634
C1D	8182.572	7773.63	5776.999	7939.279	4798.562	7847.977	4759.884	7640.942	7509.28
7A5	584.8643	388.2856	983.3906	549.1625	651.8834	342.11	504.3913	688.7769	95.7298
OR52R1	53.50694	68.86019	52.41515	70.72655	43.18159	34.76513	49.50179	45.32081	81.86005
CABLES2	60.07795	39.86849	995.9557	37.62817	1399.551	1261.537	1104.208	736.6802	49.45571
PDCL2	36.2131	34.50269	38.75291	38.12075	40.27903	39.49574	38.25982	35.11975	34.42065
UBB	2194.886	1523.201	910.7345	1404.477	972.0384	707.8912	773.4205	825.2564	2418.401
ME3	56.65998	80.02381	60.18153	102.8995	71.77454	63.67318	53.31705	81.05898	96.22062
MBL2	33.24548	55.18209	42.55196	33.24468	33.48738	44.22042	49.1128	39.46728	43.23964

BBS4	5473.518	3268.178	3189.703	3930.879	2463.4	3125.193	2740.424	2694.917	2980.779
SMC1B	62.8864	97.40778	51.75819	96.41293	55.81176	81.60117	55.56717	60.20724	65.70288
SLC12A8	154.8207	125.7822	247.9133	138.8711	196.4502	544.296	155.8357	121.9701	901.4612
FGF9	133.6094	133.1596	768.0668	613.145	516.1786	761.3025	640.4261	789.2097	135.1116
CXorf22	35.06545	34.90438	37.4797	35.82484	33.99117	34.98581	32.58302	51.90453	31.61949
LOC158572	218.3233	107.8645	64.5799	154.6424	63.41872	118.3054	74.6861	50.3497	303.0668
ORAOV1	153.5978	134.2538	1324.506	259.6746	1788.699	2254.916	2195.394	1756.564	419.7005
PLA2G10	1595.096	701.7346	222.785	2497.313	2195.223	783.4164	952.6785	1083.648	2227.161
ARNT	174.9397	208.2589	100.7408	207.3417	105.7221	137.5304	77.06369	115.2512	305.3171
NOD2	1154.515	44.81207	583.4509	36.72873	755.7738	33.40997	488.8741	428.2408	36.80867
SERPINC1	39.58431	44.56913	38.96761	46.218	129.2844	39.83493	105.1374	38.82841	44.46668
AQP11	58.87813	34.07915	48.13138	84.75456	63.47684	37.17284	88.54198	50.9161	70.24436
FAM175B	49.83846	36.97278	930.3152	464.0417	1167.626	1712.401	1149.025	943.324	33.63337
FAM111B	705.3271	313.2004	233.3955	427.6605	121.4292	210.8152	118.9659	161.1851	312.6104
ANO9	373.2213	209.5382	108.8025	236.2782	159.9262	169.1485	93.51025	118.9107	529.9637
OR10K1	42.67698	39.24405	44.88692	43.84711	42.21358	38.41701	38.22349	57.60357	33.15338
MED11	281.4052	88.55953	100.6194	182.5508	112.5728	89.17934	105.3583	141.9248	181.7632
C20orf70	68.17023	49.9711	46.90342	55.22792	51.34568	42.86932	48.85224	74.30672	94.3993
NMT1	546.7454	768.59	852.7156	600.6664	787.0082	789.9875	719.8568	1012.451	636.6951
FAM181A	43.92686	99.43504	50.54116	60.20417	177.7441	470.1396	164.2001	1078.968	111.115
KRTAP10-4	62.05549	64.16238	52.06216	68.14802	43.97319	45.73217	51.20977	36.05965	48.41545
HHEX	153.4819	102.9569	235.215	154.0943	460.3814	296.8755	410.3015	352.7062	172.1686
C19orf59	174.9349	74.78788	52.30659	44.47793	51.02823	58.75129	177.77	147.1531	140.6179
EIF3L	337.6264	537.0507	653.627	273.8284	1745.827	657.3556	1219.63	955.6865	512.2236
EVC2	79.23603	57.14081	41.05833	52.77489	35.98877	37.63295	40.0575	44.34336	111.5054
RPL15	808.9044	636.3179	491.2116	681.3669	447.1588	282.35	359.5788	338.7629	752.587
SH3BGRL2	4947.879	3311.294	3194.005	1693.905	4248.025	4141.634	1846.227	1107.831	3528.842
RNF26	116.5128	129.6682	61.1475	175.0396	493.4436	86.22575	337.6562	203.2423	172.562
MAGED1	102.713	70.12841	274.1083	206.3656	108.377	165.1955	97.50207	79.24574	462.087
CNIH2	51.3696	56.50384	48.95509	60.57579	42.41951	38.54639	44.84222	49.32797	42.94315
RFXDC2	148.8043	116.3032	229.6244	74.64185	262.0961	364.136	323.0523	508.4804	173.3606
IL1RL2	75.28343	77.22781	62.87359	106.8906	187.5991	415.3105	117.2815	60.07946	79.22919
CANX	1194.083	3168.29	1910.94	1805.777	929.5604	1349.007	924.4813	1302.109	287.2619
HELZ	35.58662	47.80466	131.1942	49.29652	143.8775	233.912	318.6631	160.5536	75.4469
FAM163B	253.3121	131.2609	94.28181	144.7801	70.81805	121.7989	61.65863	80.82398	284.7689
HLA-F	6337.057	7586.383	5527.774	7906.396	2272.633	5178.744	3660.495	6809.064	8005.202
RNASE13	48.12998	39.2624	52.23132	64.28358	54.04902	37.40181	37.93504	52.93346	64.11176
DSCR4	111.4341	102.9405	61.1502	67.20136	60.41628	84.57832	57.45361	71.88451	135.7572
DCAF4L1	253.7245	134.0178	94.27404	154.4219	77.74809	118.4926	68.72291	96.77466	246.8969
ECE2	648.1922	516.3679	346.0557	409.3015	421.3473	432.001	360.3735	879.9766	751.7239
FAM119A	412.2256	478.2459	628.1606	244.1545	592.7883	345.8903	591.5768	634.5038	902.2809
GPR113	106.18	116.2896	70.6002	150.1514	56.03786	78.16428	49.93784	63.34645	68.55736
EGR1	1637.447	1876.389	1864.84	1545.13	2544.992	1532.903	922.1066	1614.962	3319.88
CHD9	15609.82	15273.22	13573.58	16192.61	12900.44	13826.82	10561.58	11480.91	15260.42
KIAA0141	260.4107	161.5514	322.1789	167.4442	1073.45	688.8536	702.0008	354.936	489.4887
PIGV	1427.358	1233.611	882.1906	1010.064	689.263	1149.863	743.7058	1058.105	1212.309
ADAP2	2259.64	1424.119	2245.416	1610.757	2393.418	1283.484	1501.953	1623.485	1408.922

HIAT1	79.66798	104.4586	241.9447	105.3671	135.0341	100.6794	143.9612	278.5774	253.5368
C7orf10	133.8305	88.55133	222.5165	743.1398	490.7973	576.4869	2046.114	440.5659	109.1521
QARS	13480.5	14429.84	14256.2	14553.58	14948.06	12620.37	13116.98	14676.43	14534.58
C14orf43	352.2067	184.3384	548.4391	233.185	573.3553	800.2281	483.6735	209.1663	413.9597
MCCD1	109.7126	158.4532	71.62004	129.7223	64.8945	72.93275	67.87405	85.21847	108.2922
CCBP2	11492.86	9138.839	9645.825	9253.397	12628.94	8923.576	7685.665	6527.964	8908.576
DCTPP1	100.3245	327.4375	599.4144	137.2329	1120.055	355.9226	984.8905	877.171	81.74808
NEU1	366.1172	580.6485	887.7464	1703.867	982.3124	1494.831	864.8355	1252.3	395.5565
NDUFB6	33.97875	45.33366	48.43195	37.49488	170.6341	35.53367	54.96445	46.07888	38.59996
DPH2	195.0554	156.4635	85.56681	177.773	87.16489	117.1179	67.50309	73.79941	351.2185
RNF148	45.56928	34.85139	36.92385	32.93185	39.23963	34.48304	37.15808	43.68361	35.33035
WDR72	2608.894	9818.783	4600.374	1248.737	1788.321	7231.266	2954.54	461.4549	37.31996
STAT6	10242.55	10205.12	13616.62	10948.55	14636.96	14355.97	12856.2	11688.83	10697.91
DZIP3	50.39068	60.03221	50.10802	62.91433	46.79397	48.69132	39.20405	39.86872	54.98145
BATF3	2036.823	2208.503	4490.153	49.29594	3230.308	2249.765	3210.723	2178.472	55.50658
ZNF10	38.15088	50.11418	43.12168	529.8002	56.13079	495.3101	190.3434	46.39505	49.32508
MEF2D	302.6036	321.9283	990.9111	536.8138	1138.011	1406.625	1266.769	802.8677	361.0407
TTBK1	60.54763	60.17178	57.31971	63.47904	38.71558	40.94138	50.25275	52.33154	63.46313
MRPL2	40.0716	49.44622	407.1298	84.70822	259.6706	46.26667	164.9022	521.2504	161.0718
CHMP1B	696.9304	41.34912	213.973	916.0167	276.9568	711.5934	511.294	400.2544	54.17371
ZFYVE28	45.4442	245.3152	39.67931	38.82403	37.21132	36.04532	269.2305	34.46455	43.33864
TNKS2	197.1886	56.09592	59.14512	53.58391	80.31776	79.66449	54.50338	71.85983	160.0786
PLSCR2	68.90952	32.7555	240.0452	42.67416	42.38422	34.16185	105.8702	244.5682	37.17171
GALR1	38.61199	50.81357	47.56549	44.06538	47.28272	75.98997	48.6547	46.50641	44.67947
C12orf39	86.89321	63.19444	118.7418	77.85587	43.97865	183.9859	73.01382	152.4063	822.6772
GZF1	56.23178	54.14476	67.81023	49.76214	43.99393	374.1718	47.78163	234.7486	39.86712
ADCYAP1	43.92134	49.28531	35.24216	36.0557	32.61222	36.06046	32.89013	36.97187	37.63255
PIK3CB	71.88451	77.99869	79.50148	52.37963	349.4274	54.13926	287.0298	37.73723	40.51443
WHAMM	93.75355	887.3599	979.6377	458.9387	880.1436	985.8556	1163.472	1312.225	93.30753
NPPB	56.61964	49.17392	54.68009	51.97097	39.16979	37.89552	42.9751	57.25308	50.3702
BID	54.19885	70.89767	1200.825	42.40828	1647.453	72.19885	1365.153	1787.504	164.0375
ISL1	684.9667	322.7315	656.1141	439.8248	71.15209	256.0136	333.8185	65.64259	793.6691
CLEC17A	432.9901	227.2495	86.39795	242.5031	80.92871	136.2072	66.27589	72.24066	489.4534
C21orf58	57.7002	439.2039	74.18466	85.67535	147.233	51.98189	47.40546	49.46075	51.72381
CCBL2	100.9487	102.5065	54.04609	104.8796	59.57234	58.9683	69.03436	58.01108	104.6745
LRRC8D	94.31487	79.33654	57.28313	58.22451	323.1928	75.07253	222.7494	310.498	146.2978
SPHKAP	62.50071	63.41677	60.4154	49.98519	43.25451	43.63877	46.15841	41.38113	99.36464
TOP1	61.47676	282.5082	122.2131	68.35577	93.88906	71.99051	78.34015	108.6841	54.24076
OR6C65	94.3448	67.98024	40.81525	74.49315	43.70102	56.30795	44.90937	58.92936	44.70042
SETX	736.3798	398.0002	273.8203	133.7106	298.733	75.33897	273.5413	174.67	1007.698
CHIC1	141.6876	141.726	190.5436	115.4221	227.6136	92.92563	70.17141	87.44941	333.1144
KIAA1202	43.76341	38.46972	37.83303	39.27527	38.76874	34.93097	106.4449	33.59896	55.34744
EVI2B	35.40902	293.3957	146.4855	33.2667	97.82648	206.0404	74.04939	69.78598	562.8098
VSTM1	228.3401	66.60093	58.0683	169.5189	68.13105	108.4776	58.53205	91.2533	271.6786
SLC25A42	41.22634	555.0297	39.00955	57.9403	34.7562	42.76919	102.0269	42.03806	42.21156
LOC65287(	58.5392	52.40285	37.46514	49.43975	38.9819	43.30745	39.93869	52.25053	57.77794
CTLA4	259.2764	956.7446	3161.776	137.1269	914.9568	2360.775	1490.977	2427.928	2422.961

CKMT1B	36.1604	712.014	648.3651	523.3886	1165.925	281.4699	509.4365	254.8642	1415.741
GPR82	47.52172	85.17616	58.4349	64.5686	39.87674	81.63531	32.92799	55.12241	45.90471
SPINK2	264.7737	106.2421	84.443	113.774	63.15072	108.1999	45.19445	55.92509	99.78307
HARS	13876.54	13507.35	11756.05	13572.64	11111.17	12528.61	11176.5	12269.64	14851.85
LZTS2	81.76964	47.6396	263.8395	53.92627	280.8813	50.20163	420.4996	142.5245	62.2883
LCTL	66.61214	54.37165	49.62375	62.99345	52.37427	53.30812	54.91738	45.94463	120.2277
IER3IP1	752.428	50.05485	500.9261	759.0062	814.0977	725.9969	560.7079	357.0005	1468.857
PSME4	1329.452	1804.413	4946.898	2256.591	8908.603	3305.379	8085.658	6954.754	188.5913
NFKBID	242.9489	114.4624	72.90784	111.2927	112.3041	114.2682	72.73209	84.06545	260.9547
TNMD	217.0781	158.7655	87.04137	113.3181	568.1597	108.3633	59.48666	71.25458	227.9759
ZNF423	36.18555	733.9274	36.04704	48.39706	495.355	33.82961	36.89388	37.41709	34.22961
FAM177A1	37.71154	695.4828	43.20715	35.3307	181.8695	36.42319	157.515	33.53411	33.00824
CACNB1	1152.213	1632.108	398.2786	803.7593	247.1569	637.5816	612.4325	1182.666	329.1067
DIDO1	78.1292	339.9355	112.8108	54.633	341.6293	305.0766	325.4472	207.152	45.85817
SLC22A16	47.39759	44.09549	38.39565	45.51096	36.79005	212.3767	74.00632	53.47148	34.86497
TMEM132I	32.7673	42.41341	119.466	43.6757	45.08178	38.91904	115.0513	66.12304	59.76499
PEX14	35.85094	37.57668	386.1872	58.50944	200.1806	426.0372	237.5149	163.6342	39.98118
BCAR3	56.89168	60.90717	47.90448	156.7905	37.11964	34.49802	166.6197	33.4937	34.3417
ZNF614	1913.769	423.8408	178.2975	1134.382	646.0479	283.3569	797.621	330.1085	81.83894
DENND1B	39.27394	37.80345	519.99	381.0035	96.41719	421.1855	142.9336	184.2063	37.63946
CXXC4	244.7735	94.02764	68.80119	91.03419	65.6775	76.52855	48.2552	66.54086	117.5006
TM2D1	452.8788	304.5487	135.22	342.4033	117.9411	194.0942	126.0939	176.0856	597.8029
C17orf49	222.8423	1403.753	2755.452	650.1306	2645.454	1253.168	2749.723	3344.276	1606.436
RSPRY1	9196.091	9733.688	16756.88	10103.98	16517.72	12251.84	15835.82	14524.05	6460.821
C9orf24	547.859	292.8408	124.0271	168.1259	528.5067	366.8562	175.9295	152.7578	637.0428
RGS11	7056.73	4019.802	2513.75	1165.609	2375.462	3063.449	1808.405	2103.606	5700.22
PTDSS2	67.80375	446.4007	1313.524	515.1361	1807.47	280.9737	1385.36	1353.651	1901.777
OR6C68	48.24491	34.2763	43.09832	42.20373	36.66923	39.60897	46.16759	33.9621	35.14484
APPL1	556.0374	416.4428	482.8143	519.5771	504.7138	552.7001	457.1166	366.6336	680.9065
KIAA0853	108.5083	34.90444	41.74226	38.54404	38.89345	40.04599	35.84695	47.62172	88.71575
SOLH	69.70868	51.69173	39.86609	50.0316	224.4527	393.8646	301.7737	41.05454	35.89114
FLJ14166	101.3885	106.8176	63.09951	83.10884	75.12173	124.0267	64.50234	57.98364	118.4589
GUCA2A	192.2969	256.8062	84.97478	234.4724	100.3818	68.96244	82.80125	108.4141	311.9302
NPTX1	40.42134	33.55822	45.83985	43.57803	33.05047	36.47089	31.67981	37.54657	53.62621
RIPK3	3397.089	6884.465	6958.978	4171.123	6311.866	5810.054	7431.576	5278.055	4242.796
DNAH8	194.4994	135.2245	80.11164	77.34982	67.12441	136.9819	58.17855	86.99023	119.9528
PPM1L	1453.789	48.8254	53.34653	32.62045	556.4662	38.01532	275.4884	246.9444	67.97431
MTF1	94.72645	81.13922	112.6404	94.99857	482.7862	58.20143	217.0147	48.93346	75.19826
APLP1	269.9474	484.9573	318.6238	155.1067	537.197	652.7686	378.9285	167.2054	268.6855
ANPEP	325.8187	160.6832	430.3565	237.238	635.5866	125.5786	515.4269	539.9457	338.2313
PTRH1	208.5581	136.5851	67.47482	141.1512	117.6593	415.0204	104.6055	78.96393	350.2485
CAPSL	59.13427	58.60538	65.37154	77.99948	69.44706	68.45045	51.65549	74.52352	198.383
ACACB	12595.01	15981.02	7932.421	8465.425	8414.194	11030.73	9657.454	8582.825	14489.33
ABCF1	7251.678	9171.447	12034.67	9339.918	11097.47	9827.277	12587.64	14456.52	7188.841
CDADC1	125.2222	56.46272	71.20524	74.149	60.01421	68.44084	61.23978	128.2978	49.48775
KRTAP19-3	43.54782	50.82854	35.0149	52.02528	35.1134	37.10059	52.50518	42.61018	87.61436
Magmas	1893.139	1575.227	1531.504	879.5737	2782.375	2250.423	2191.107	2279.482	37.78691

FLJ35258	1641.231	2639.199	868.332	1167.682	1882.959	2352.338	2230.342	1255.956	2755.761
C6orf173	44.56289	440.5194	1226.141	1564.917	1635.998	1027.514	1932.734	1568.092	33.84418
ACVR1B	68.61693	43.86695	36.18538	35.63836	37.6739	46.97183	38.57808	46.09588	46.86757
LRRC49	60.7739	40.87632	67.43013	343.602	391.5776	218.6721	305.1386	213.3619	56.84777
FLJ44005	124.147	52.08378	62.92492	88.18135	61.02814	73.28429	55.83911	59.46986	127.871
SEC63	1946.472	2370.289	1176.807	2559.011	1162.193	1346.67	830.4894	1472.981	2031.086
FXYD1	402.8393	229.802	122.792	254.0368	183.6963	181.9165	94.40495	127.3631	1301.635
FLJ40448	55.39864	55.33095	60.18584	68.65891	59.18924	65.48654	47.01372	55.90171	129.817
XCR1	144.1476	275.174	78.96107	127.1781	65.72768	91.11964	59.23915	62.14011	451.0912
P2RY14	47.23534	35.50659	66.31165	34.11499	37.04349	55.1112	38.64752	34.31491	81.36543
TTL	762.1849	359.4007	234.6681	287.8989	169.488	196.1428	188.0115	228.7653	840.796
EGR4	66.56034	65.35139	67.17628	130.5604	76.60596	72.67909	60.77363	48.84421	188.0808
F11	158.6616	92.58641	73.09093	176.3227	67.64383	53.01071	56.28946	67.09902	71.25747
R3HDM2	62.90615	52.14263	233.8425	55.39301	208.0526	74.43685	124.8254	185.8079	47.47932
CCBL1	4074.243	2732.996	2388.188	3623.756	1632.436	3078.696	2049.788	2049.004	2813.918
IGF2AS	227.4505	209.042	55.67569	83.46009	57.20817	106.7778	57.22128	159.2269	188.355
NPHP1	91.24623	317.6665	199.5891	373.7135	223.3186	457.4455	424.9087	357.467	87.41476
NLGN2	2658.978	2686.896	1708.471	2861.013	2114.793	3099.039	2606.559	1366.182	786.9494
TRIM65	299.7664	122.1327	80.52012	259.5289	188.3989	137.3523	220.7677	84.8855	382.6825
STXBP3	47.82989	1082.536	794.0802	2120.06	527.3739	634.2642	492.5816	313.0031	98.98432
GPD1	79.70693	63.95872	59.75006	38.4961	54.03276	61.65353	38.72053	60.63766	58.90522
JARID2	36.96239	39.48358	1461.626	1175.613	1925.527	1114.57	1718.891	1156.377	34.81467
CDC37L1	1057.433	3215.132	1042.229	1411.782	799.7267	1527.433	797.0029	762.4543	1076.113
MAP2	56.05261	1315.461	2638.123	1190.569	3013.862	1631.479	1595.033	603.3981	59.9503
RPRD1A	387.2195	164.414	97.94264	198.8491	87.95966	135.6073	100.5187	105.8755	323.6281
OR5F1	163.1906	78.74136	57.68178	110.7301	60.86626	77.21391	50.49812	70.89265	64.95158
DAB2	17259.78	14234.77	14781.12	12466.29	10638.19	13592.34	13488.27	11992.63	15914.9
GNPTG	347.4616	664.5045	88.20636	169.9594	144.4664	113.2224	115.2378	156.3835	464.4226
PMPCA	5998.26	6429.858	5433.28	5345.623	5765.537	6082.99	6524.264	5189.272	7020.601
CKMT2	947.6082	1046.205	943.7079	101.0911	542.1609	1885.463	2486.25	3491.41	1481.386
NXPH1	150.3992	123.0444	65.21146	106.8407	78.92797	125.2189	64.7001	77.84588	256.0447
ARF6	460.8174	292.3317	142.2149	305.5825	128.2007	209.8856	132.6357	184.3479	629.4745
ADH1A	70.75084	56.22061	51.54998	56.29223	45.57533	38.67164	44.32259	74.01494	51.94869
FGD1	49.21608	801.1265	1850.644	3108.486	2181.311	2668.038	3124.887	2040.905	36.76929
C8orf58	217.2312	93.72531	151.2714	481.7777	465.6181	102.1893	465.6985	154.9105	245.6722
ACOT4	243.9151	72.00329	279.7301	208.6943	174.8329	217.3722	475.2966	81.44465	233.6577
DUSP16	198.6445	197.8075	92.06374	121.6621	68.311	129.8102	73.3491	82.91119	374.8467
LYAR	2304.705	2254.949	2193.397	4143.61	1717.303	1629.033	1699.605	2953.14	4436.408
GLTP	1818.545	2870.148	5349.1	1726.356	8520.998	3839.792	4087.351	6535.343	1080.704
MCOLN1	37.67536	789.8413	207.5287	41.83945	569.2896	419.7333	149.9826	39.17323	66.15421
HDAC4	1125.505	1370.932	1841.777	1109.64	2226.587	1931.472	1518.575	1548.567	1349.922
TUBE1	88.96836	90.92954	61.00402	95.78178	92.46992	63.66158	45.74191	77.27876	842.7004
FAM12B	89.25487	141.2088	62.68571	71.37951	56.84262	74.28183	54.65856	42.25992	260.8429
C22orf13	53.24266	1085.225	3317.436	1910.056	4451.329	1757.981	4812.485	4025.629	2903.275
SOS2	126.0694	1023.223	330.3792	650.7707	166.6749	590.3276	196.4017	54.51184	1653.898
COPS6	35.49331	39.53596	593.1915	516.7245	1281.602	227.9507	1092.875	1081.179	71.52277
TECPR2	44.89352	51.85665	34.73901	41.5819	38.51352	38.03326	31.98717	41.26969	2804.587

TBL2	239.8926	166.5406	76.06853	147.529	73.81923	84.44067	153.3864	91.81087	279.4558
ATP5A1	427.8987	533.9451	3866.323	1544.636	5998.058	4052.151	4919.691	4994.341	516.1461
C3orf10	3310.093	4123.909	4064.576	4196.708	5558.164	5439.014	5323.708	3451.133	3857.068
PTK6	2987.003	46.87659	1689.879	820.5947	1643.848	730.5574	894.4649	949.1571	44.95752
OR4X1	62.09452	42.23162	44.34068	44.38465	49.48829	43.53562	49.81062	42.51529	55.70838
LMBR1	31.29962	418.1662	954.5412	50.14943	1311.307	1316.131	971.8251	731.7143	49.72811
FIT1	3803.318	6091.946	2338.984	2271.177	748.4031	3416.518	2666.934	2950.022	5039.231
MFNG	961.3353	1680.301	3096.336	1159.972	2179.362	2507.357	2298.569	2354.291	1624.383
FGF6	94.28875	87.06458	63.63019	159.2883	68.61008	68.85692	57.30796	90.7113	55.42788
UGT1A7	875.9474	60.58881	60.2085	68.66949	106.101	251.673	42.54287	58.07227	99.57235
OR52E8	38.09202	48.79957	43.36837	54.90923	44.17831	35.84761	34.6796	63.88688	48.09933
GPATCH8	40.43436	313.4882	112.3017	334.9455	125.8285	34.07561	199.5152	177.1804	167.4817
FLJ45244	432.0554	249.0241	159.3501	254.2441	242.4295	188.244	391.5808	526.5063	575.4096
LYPLA2	133.4488	66.39226	60.35575	494.7314	127.7411	227.9654	76.01383	170.1005	276.5035
SLC39A14	131.3769	909.4078	56.18047	703.0457	443.2567	618.0012	442.1865	296.3521	107.1457
LOC20245§	157.5439	216.6891	89.58732	189.7886	73.53607	90.21828	69.39499	75.79557	76.7207
NTS	60.66588	51.10719	43.4986	53.75008	376.9015	46.0638	67.95434	136.5282	73.57371
ZFP14	458.0775	273.58	502.902	273.2799	557.666	1186.126	333.7418	268.4653	550.6202
MAFB	2670.347	1504.324	3313.081	1367.452	7923.872	3542.46	3983.913	2347.436	63.35426
LOC55908	437.9526	246.5276	132.278	245.8434	165.1378	224.4614	138.6533	221.9797	578.9566
RPL8	3913.007	4630.48	2700.693	3175.354	2955.966	2918.938	2055.446	2224.085	6239.762
SH3PXD2B	65.77304	57.43725	156.1461	55.47451	329.0368	179.8798	281.4699	218.0461	102.2902
LIMD1	39.27426	41.00483	36.9222	50.64223	34.35445	39.59748	48.21495	48.29286	48.85425
RAMP1	37.70489	826.4496	599.6115	39.8862	292.6892	785.5204	1453.871	1782.301	34.9931
MUM1	671.623	899.3268	908.4153	467.899	1010.47	1014.84	873.594	982.3193	747.5156
MGC15763	41.19388	38.02314	218.271	41.95271	37.82393	213.9735	80.88768	121.3769	50.34488
NASP	40.78112	52.1092	50.5462	49.42797	54.78596	41.12029	45.01311	38.39521	85.71698
SVOP	70.71246	142.3544	61.96276	102.8902	43.3565	74.11762	50.42838	252.9174	165.047
TNFRSF1B	7370.691	6525.318	11003.51	5957.3	7130.297	7365.556	8504.038	9842.541	8431.703
PSMB2	80.79291	993.1064	2029.382	3558.407	3570.798	2888.342	3334.341	4206.411	7095.646
FMN2	69.98223	37.40966	39.515	43.034	47.21607	59.6038	45.91706	44.79201	32.5176
GLRA1	65.87506	82.14767	59.27331	123.5109	50.95154	84.39941	45.58882	45.45232	74.65298
SMU1	1026.227	764.3874	386.1903	655.2682	225.8814	419.8348	247.8637	351.556	1052.597
B3GALTL	83.87518	420.2565	326.3208	54.6945	700.0887	608.887	144.7321	586.2745	50.45684
TFE3	411.5739	238.5505	135.5509	249.386	169.922	238.6569	225.3244	134.355	564.7087
TP53RK	264.6445	174.4747	84.56738	133.0804	94.51792	127.8121	85.59972	110.2564	448.7466
ZNF761	77.35776	48.61606	221.8567	363.2909	127.9268	58.19213	202.6521	111.1059	36.05082
ISOC1	39.09264	38.092	36.29834	41.39243	122.6075	48.01588	114.7498	40.49932	57.25745
LOC649431	48.44389	37.70699	37.4097	34.43122	112.3507	32.40136	35.13375	32.78651	38.33156
PIGP	1189.306	1714.932	1239.257	647.9171	759.4388	1065.497	1184.819	641.9449	1541.331
ELAVL1	584.1807	86.21071	355.2597	482.358	406.2517	609.5668	410.8493	209.3049	184.8933
C20orf75	114.875	108.4966	155.8963	471.3089	187.763	59.48828	1387.788	121.4985	46.02509
ZNF384	144.9254	66.69749	63.78483	56.02565	61.11493	70.59222	68.43378	49.41744	105.7243
CDC42EP1	1025.177	904.276	863.4169	1384.68	1545.349	1581.194	1688.602	1045	1188.133
AP3D1	250.808	143.6848	141.4395	145.5391	429.4795	67.98942	356.542	191.4913	2210.519
FAM154A	41.23684	36.72406	40.28146	52.99531	35.35312	40.26725	43.77779	39.13348	220.705
IFNGR2	252.0065	890.0591	2813.818	79.80378	3374.538	2926.585	4048.375	2196.356	1398.667

JMJD1C	55.95788	614.5403	77.71819	104.4694	1030.664	399.651	714.3287	345.95	152.8824
SERPINA1	66.62706	120.7191	73.05463	132.3509	63.138	85.28965	196.0235	40.22298	193.0394
HIST3H2BB	49.68572	38.58367	40.93919	794.0421	36.1595	47.00895	35.41352	32.32923	70.7035
ZSCAN20	46.39904	40.9599	197.9895	43.00389	37.80836	55.39424	111.8646	40.16539	47.92829
NAT6	42.37843	40.04659	947.9705	529.5005	958.5236	291.6973	580.3275	162.5852	36.52258
FOXB2	105.5269	74.90407	67.21204	85.86179	62.04049	92.87283	46.81626	77.06028	187.5533
COL6A2	280.6746	201.2083	225.7099	221.6146	81.27032	152.3912	82.89106	113.4744	376.0587
SACM1L	2468.709	2692.225	3856.767	2755.538	3643.125	3173.982	3308.026	2918.875	1175.536
TOP3B	8815.237	9231.159	10653.21	8271.643	9349.496	8732.258	7659.726	6585.532	5642.05
PATE2	70.17423	63.64484	46.16355	55.9572	59.42456	56.5902	55.62444	66.20444	47.88801
RPL38	94.73542	2152.771	1849.913	1667.291	3415.928	1431.923	3630.546	5706.531	2854.023
CUTC	33.16314	42.70747	45.34258	34.44876	41.9075	35.14639	144.9354	39.53176	79.20605
FBXO34	658.3434	43.06384	599.5781	46.13164	361.8829	341.4874	518.5717	63.46892	37.80761
OXER1	131.8806	87.34406	63.48434	53.90587	149.2378	71.1354	119.3876	61.21593	114.5766
KRT85	162.5446	69.6744	50.72316	63.59123	48.34303	44.27857	52.99631	54.08418	75.34454
APPL2	33.95979	34.20163	1222.621	947.9335	5220.54	2169.923	1936.226	1386.787	2387.078
RBM10	1497.944	94.34595	526.2027	166.0408	1797.907	446.8953	992.9202	998.2817	302.0882
TRMT6	472.8163	149.6925	92.49261	217.7135	99.03585	140.1488	104.6745	111.9215	601.2929
ANKHD1	983.0174	66.81096	151.1822	33.21307	241.8164	198.6372	129.6378	89.66148	60.89576
CST8	73.46154	316.1928	76.42591	93.71935	65.30879	90.63586	79.20306	83.03728	153.7567
TNFRSF25	55.74817	1675.211	1885.422	49.29633	3979.723	1395.184	3376.555	1888.464	42.04277
PAOX	197.9704	83.45127	76.21046	172.1886	85.34321	106.0736	67.71393	80.62092	201.4136
MAP3K2	1467.707	2480.691	3308.097	3000.458	3345.668	1505.083	2380.757	3503.523	3501.276
IFNAR2	731.0859	735.1506	388.0371	618.3875	247.5228	460.1476	264.3371	364.6698	1428.925
ELMOD2	781.1736	1563.787	1541.724	1027.005	693.8472	2349.738	1241.84	1306.286	943.4637
MBNL3	45.23899	430.4101	116.157	67.82844	121.9142	60.82159	59.90149	53.03316	58.75817
LHB	43.80339	38.6177	40.71165	49.70043	49.32108	41.04383	40.54686	39.3519	35.96444
HEATR4	58.59365	67.69527	51.28944	45.06783	39.69624	40.4461	35.93449	35.95964	126.1939
ST13	5316.679	5031.332	6096.934	6481.887	6200.574	7138.333	8017.882	6550.316	4110.048
PRDM11	45.65676	62.22333	43.29723	53.34848	39.957	48.42242	49.52276	41.30324	87.60822
CARS2	102.6542	1779.843	3448.419	1273.123	2904.443	1618.616	2267.9	3115.334	143.0967
NEUROG3	46.60969	67.55522	48.73479	69.44988	44.425	44.68065	47.59719	44.17147	96.60242
CINP	83.33943	93.16512	39.55661	75.58378	53.84805	82.89189	156.3242	56.81963	299.9556
HLA-E	20064.31	22072.68	22118.52	18757.72	19808.72	21793.58	18933.63	22173.91	17087.43
CAPN11	4848.154	2739.119	2228.149	2611.764	2762.121	3198.685	2354.583	3741.74	226.0551
MGC35440	158.6487	517.4857	134.3642	431.6636	80.82998	99.87215	69.31508	157.7075	170.2054
PRIMA1	67.77197	41.869	63.89821	50.83501	50.97419	64.71346	49.20888	70.52579	100.9034
TCEAL2	5530.268	2807.557	1225.189	49.49188	2209.396	48.71337	706.265	569.505	2620.614
TIMM10	6994.303	11024.42	10684.28	9989.742	9393.355	13727.48	10142.71	14411.23	8745.927
NOX1	92.27721	79.63113	53.52982	75.14921	42.35225	72.79157	41.78118	54.27815	42.39303
DSC3	6239.06	3599.776	3514.354	7287.248	3611.612	3632.698	2107.636	2869.936	5610.24
LUC7L	5178.464	6090.893	4947.271	5520.532	4529.569	4788.216	3891.148	2601.71	4913.093
C20orf77	55.63025	86.50777	68.75155	82.28205	52.31397	66.64714	61.97559	67.50375	122.7789
RRN3	220.2189	259.2776	162.1086	277.5403	218.5588	260.9771	363.7232	210.0369	161.1126
STAM	38.25776	634.6962	485.8181	1281.302	368.6361	479.6335	498.7301	502.2492	56.49851
RBBP9	373.775	208.8939	139.9736	285.75	112.7263	195.7215	113.2044	154.1333	676.4025
TSGA10IP	74.08432	81.14233	36.17693	65.93317	61.26923	66.09192	46.18527	50.84731	63.82722

OR51Q1	249.8686	189.0602	81.60847	185.0461	74.99648	109.6292	69.39114	89.5008	70.08141
EMP3	3035.5	2314.513	847.4006	1188.403	391.93	169.1909	710.4715	1132.46	622.6119
AMIGO1	124.912	138.9061	95.54444	116.6537	88.74517	105.0682	75.25101	93.69981	147.7368
SHKBP1	566.6508	1270.5	1391.564	1824.284	1644.702	1048.057	1761.318	1692.772	2271.412
NDUFV3	74.97997	682.518	37.89771	37.17373	126.7351	300.6996	144.1647	36.21032	1075.813
RNASEH2C	62.03503	425.9212	365.1118	382.6047	113.083	248.6469	240.0563	340.7735	1319.078
MTIF2	1061.746	307.8925	1541.819	1250.555	1281.504	1086.3	1434.506	1380.122	37.13857
PPM1G	43.98282	32.9236	34.65991	34.2726	42.52535	66.70973	34.84359	47.958	36.45712
TOLLIP	40.13418	45.28966	39.77448	33.96179	127.7108	38.66996	79.18623	46.38977	42.58675
RDBP	14291.71	15101.16	7902.121	14492.12	6386.937	8821.157	8293.701	11552.49	16052.01
IRF2	3521.366	1716.698	888.2933	1118.263	788.8443	1310.308	843.8398	1215.168	2778.002
HOMEZ	144.0864	54.05261	57.96823	95.8247	56.61424	72.29837	52.13534	61.97838	67.17159
OLFML2B	2147.61	1879.556	5361.555	1090.412	6399.783	4282.578	6754.097	6480.842	2927.612
NPAS2	126.5839	113.9539	195.0318	102.4363	133.9852	252.666	62.94408	81.75595	179.3794
TIPARP	153.5929	117.1253	179.9743	152.213	252.1391	201.7475	174.9067	219.8273	179.6795
TMEM38A	251.211	331.1119	83.70815	59.29213	87.44576	284.2036	167.3603	473.4156	269.5124
APOC4	74.00822	45.19218	38.10406	58.58073	40.60037	47.95716	41.06643	35.30119	75.58517
SLC6A2	368.9029	368.6551	115.955	1384.544	219.3504	406.5183	505.9666	406.8983	490.6938
OR52L1	75.40809	93.31666	68.58108	65.7515	48.74392	54.95329	51.17767	63.60709	165.6091
OR1B1	112.0502	75.89204	72.37482	80.83695	50.66093	74.58562	55.47942	51.64804	128.7885
VSTM2	48.18535	54.44226	35.15584	73.56354	35.39771	35.78273	40.26601	64.88084	48.82944
HDGF	77.72148	74.46954	69.90484	56.74045	401.7381	62.22024	315.5177	237.1693	74.14767
FLJ22184	63.91303	43.82643	35.06828	53.2966	55.94333	39.13599	46.46629	48.15897	37.527
SEMA3A	183.3015	119.3829	548.5941	58.44838	74.94379	70.98848	324.4165	86.94485	336.6746
COMMD8	984.9467	454.6168	1283.415	219.6341	870.5965	969.7383	1121.98	791.1913	533.1768
C17orf50	43.00544	41.75288	35.93366	68.32288	43.71494	47.75982	48.14326	39.98371	45.33814
IRF3	1481.485	1841.913	1974.301	1376.856	2113.903	1791.349	2078.642	2997.318	141.0646
IL20RB	276.6528	559.9832	591.126	788.1517	611.2697	219.2648	552.273	1483.281	1025.662
ALS2CR12	469.3707	189.8952	149.0642	48.29284	108.5556	228.1558	109.8655	157.8248	30.60214
C19orf70	174.0563	607.5309	328.9512	88.05399	1077.894	494.9717	543.2761	786.1802	144.118
MAML2	746.8752	346.2921	140.9175	118.0831	171.6804	187.0321	205.9518	51.32209	97.92863
SFT2D3	76.86302	47.10289	336.925	80.76147	375.6752	48.71182	498.4288	202.9359	48.29982
SYCP2	54.91156	109.9153	84.13268	43.98569	79.39919	118.2937	58.21526	65.52502	80.38594
MYO3A	55.55972	58.32936	61.37185	92.10531	47.98187	40.18488	43.63062	43.57151	43.88685
ADAM17	2620.782	1841.437	746.9521	2439.817	377.3788	1112.179	695.5593	1053.468	2394.941
C9orf79	239.4673	173.9889	83.68826	180.9826	86.13718	117.2441	77.17603	104.1996	264.4077
ENO2	504.3635	353.2125	594.8779	783.1293	705.2538	539.7088	521.2184	366.9672	96.60215
GAA	48.41056	1035.659	589.8991	33.56956	559.1721	998.3079	608.3326	1227.797	36.4794
WDR23	137.3411	65.49624	57.17504	159.075	144.209	115.7018	50.43671	36.91116	112.0889
NUFIP2	3227.65	2282.028	3918.829	1869.393	3094.17	2981.141	2394.735	2219.392	95.14111
SLC9A3R2	497.8412	206.829	147.1796	339.3998	256.6265	61.84605	165.853	64.78481	858.527
TRADD	122.0076	608.736	70.07199	684.343	624.8433	64.9523	344.67	58.08405	158.3861
CAB39	1561.309	875.4847	1092.707	4449.532	2121.539	94.11723	1284.475	827.5283	414.2345
DOCK11	1346.958	535.7595	666.9014	321.1606	888.5376	774.7123	917.0831	423.8634	540.5607
SIPA1L3	343.6293	171.1307	95.60777	206.765	96.32528	154.8094	82.56592	79.98636	494.6104
ARHGAP24	87.58307	50.53907	147.8586	62.21204	180.4039	251.3443	140.6625	43.6824	81.97321
C16orf57	546.3851	327.5481	591.8418	535.9105	693.8706	584.7331	915.0588	717.3318	622.5023

C17orf101	568.9832	329.8568	107.7693	356.5782	116.5902	175.9184	88.05429	155.3083	986.697
HSD17B3	147.3838	44.33147	327.5659	55.49136	484.4645	308.1827	777.9722	233.406	54.35607
CHGB	600.8398	127.9256	123.6147	519.3425	65.30982	220.187	143.5346	178.2344	175.6883
MOGAT1	68.15179	70.66681	49.06508	47.38382	49.78953	57.82029	112.2088	51.80887	91.08912
INHBC	41.55294	42.90522	56.75525	47.95428	40.4512	45.31062	36.99435	37.90087	70.46259
DMBX1	49.49038	66.97993	68.5353	48.8556	48.10894	48.21629	43.99276	72.53367	76.25352
ZBP1	101.9867	53.7029	290.2998	49.75051	58.65928	808.6818	140.6118	48.55541	1569.216
PM20D2	45.32509	73.50293	54.31119	77.23605	67.76981	129.6764	57.45561	44.77221	86.24146
YARS	8729.152	8710.196	7073.47	8968.431	5458.351	6324.881	6181.488	7736.893	8668.015
C11orf88	53.4204	136.3952	59.48051	49.20118	42.23059	65.04813	54.60237	60.95173	80.64117
SH3GLB1	72.07207	415.8199	653.2701	1029.098	609.486	268.9014	723.3764	794.9616	1240.267
ABCA1	9216.336	8395.046	9777.634	7296.226	7306.506	11535.23	10995.08	10409.26	7303.676
SULT1C2	127.1199	145.7253	78.49216	108.6613	65.78097	91.69612	69.2756	73.96349	249.216
REL	884.5908	40.86197	158.9795	232.9467	103.1052	50.37557	229.9619	134.5769	70.08206
OR2G6	147.025	74.15586	62.62815	146.8208	70.04267	108.9875	71.51174	96.22234	89.52473
MOSPD2	1327.786	2632.978	3949.697	2055.255	3669.006	3212.133	3635.404	2991.192	76.84136
KCNK18	389.8756	214.1712	114.8631	243.8417	89.03368	152.074	98.11026	118.5034	442.5794
PLEKHF2	711.1283	556.9235	396.0901	997.6658	439.4434	944.2358	455.3282	580.2232	773.062
FHIT	1061.299	2425.06	1736.121	938.1121	1199.719	1427.17	822.0327	1238.286	1465.467
LONP1	36.58062	50.01846	759.5426	42.80611	916.4952	34.27614	283.4634	604.1451	1899.127
THAP3	33.46101	37.97002	35.60893	28.18303	31.05842	36.12214	41.19122	74.58738	35.58261
MRVI1	33.72073	37.45636	33.9187	45.1778	113.664	37.7327	61.84104	140.9115	966.0418
MIER2	412.0248	238.4362	108.0843	149.1612	124.4536	168.06	127.4283	95.02399	193.9084
BCAT1	1187.693	147.7109	147.6285	1428.429	143.7278	410.9088	316.6436	139.6179	985.3855
SNF8	4114.347	2814.77	2576.172	2863.25	2107.796	1862.833	1180.723	2697.31	4027.811
RAP1A	55.04676	44.77912	90.35733	53.92253	63.58928	128.1611	69.91969	37.65457	129.8001
C22orf43	73.20863	52.90384	58.34552	44.63929	47.10163	54.42919	43.98631	151.9884	51.27735
GNAZ	425.4459	240.2535	100.8178	264.0629	131.0207	170.6071	117.4938	132.1407	472.2823
ZNF558	38.88354	161.8502	240.3104	257.919	198.9443	284.5157	202.6743	189.3611	45.86885
CALHM2	410.7166	234.6406	149.6512	195.1539	189.7451	155.5076	135.4014	180.3404	335.3201
GPHA2	203.9328	119.0157	94.8283	128.8341	74.29386	91.40189	62.96626	78.85419	251.282
B4GALT1	1248.922	383.7933	753.6971	1344.899	485.1691	334.594	1006.088	322.6247	121.8511
LARP4B	1056.493	183.0998	408.2509	227.3758	319.4089	137.9692	456.0396	493.7916	485.0105
SUPT4H1	48.81288	42.9284	702.7662	40.17551	569.0834	512.1154	1163.462	1266.711	38.85101
ZBTB2	4387.996	4157.575	2391.451	3261.986	1878.206	2870.462	1923.416	2742.729	5168.726
SLC2A12	34.95005	36.71791	42.02195	37.68754	137.969	856.848	761.0841	271.3004	49.89516
PI16	1972.677	80.69182	307.4259	90.64803	707.5354	204.4147	188.5715	566.3195	876.2554
OR51M1	114.4624	53.36551	56.28883	75.65128	54.68296	73.45872	57.66975	64.75742	41.09254
PIK3CD	260.8243	1538.313	651.4093	130.8176	570.1816	601.4114	890.3166	621.1663	483.0211
ANKRD54	3279.243	2145.028	1498.429	1091.869	1600.758	1136.303	1166.784	1256.016	4842.024
IL3	43.08081	32.47502	31.98229	34.32287	35.32468	33.48787	38.38658	39.89834	44.4409
ZNF512B	1547.411	1553.499	517.7538	801.4673	369.4398	850.1022	502.078	426.227	1446.416
GATAD2B	829.0289	1474.224	462.3752	1634.46	259.9569	544.0422	345.5497	486.3488	1870.669
FLT3	137.1028	56.2291	332.2187	458.8993	125.2862	289.5135	175.2357	55.89979	210.2468
ICA1L	41.31174	41.05625	76.01915	49.94898	42.15736	81.35902	35.5021	37.08436	68.37003
PHLDB3	37.4212	54.95298	138.4715	54.30102	51.90155	36.77163	52.16031	56.23796	1479.527
PCDHB6	59.80574	35.26495	58.5814	66.11028	46.27938	66.95687	39.08149	37.41111	85.74136

OR1M1	53.64542	47.87314	39.54008	68.44431	35.05461	57.55973	35.84983	42.9464	50.42265
CBFB	52.45402	36.01744	38.80809	35.0467	44.6861	42.3026	38.50883	47.32563	55.24421
AP1S2	885.8488	130.6569	146.4341	106.7971	294.4059	63.55645	61.20992	420.7248	413.9688
TMEM95	85.51637	55.3882	42.17285	69.42899	43.85607	62.15694	48.22961	49.61424	154.0678
WBP11	80.80144	284.4445	362.8644	298.3789	404.5484	372.5157	465.0163	582.3796	215.8338
OR5H6	42.58916	40.98993	44.23604	36.2476	36.59475	50.80109	43.77629	41.79282	41.16072
MGAT3	44.67355	41.33098	46.08149	47.19842	38.61477	68.71703	127.0942	45.94194	73.54529
LRRC25	200.9436	142.8117	78.54604	123.5905	85.50948	100.8124	80.26147	102.7819	341.9241
HSPA2	3828.87	3421.829	719.2	7268.904	1753.553	3054.299	3098.906	1798.364	4391.19
COMMD10	952.2604	416.9391	552.1843	523.8906	318.4037	434.0421	577.4445	577.5949	67.1194
SLC25A36	52.4188	70.96101	443.5298	53.29137	64.93939	63.34155	294.3779	209.5425	50.67629
BCL2L11	52.4614	69.21916	212.3149	76.83025	58.96637	401.5374	112.9011	62.52892	1921.319
ZFP28	133.3775	63.06897	45.13525	58.40409	37.63693	59.46585	38.98708	34.06171	39.00498
CPA3	65.92153	322.0716	48.5845	68.71897	53.28706	47.63354	130.2947	188.693	103.0532
ANKHD1-EI	53.17889	37.03416	189.8235	59.85253	267.4303	45.26974	47.75963	52.31494	33.04931
KRTAP5-9	102.9711	96.23949	56.57623	85.62184	58.20615	66.41647	52.71368	49.63372	302.935
TERC	46.58182	49.61095	184.0886	378.8277	321.8257	236.12	126.3674	63.47041	36.11562
PHRF1	2525.631	2141.89	727.2985	1647.558	620.4841	1010.528	446.4433	815.9545	3850.416
ZNF121	50.8582	31.81617	128.6525	422.7332	116.6297	38.06962	130.5868	32.57603	1014.456
GLA	40.07185	251.6212	3093.246	1248.461	4345.143	1821.011	4166.256	1875.962	1877.59
FOXA1	58.47877	56.60737	357.8863	49.48917	196.7574	50.42953	137.8639	182.4925	35.66634
MOCS3	56.07464	40.43538	147.9131	45.65393	119.3205	123.696	79.17533	177.5695	64.54272
PKMYT1	82.57786	53.93245	42.17455	75.16684	43.41829	63.35602	44.15377	62.47615	146.8827
CDH6	103.2878	69.26145	656.968	1063.008	759.2865	494.7635	756.4102	263.3225	152.3377
OR5T1	83.19548	46.28975	36.59267	41.37096	42.7564	33.05844	42.48799	34.47232	47.96635
IL17B	46.50777	61.50058	102.4563	880.0375	47.02094	50.77534	363.0338	423.9643	84.77711
LYSMD3	170.3405	261.558	102.4566	326.8031	120.1439	183.5131	91.1841	156.4772	404.9129
WNT9A	245.9132	93.52187	88.49474	96.24101	104.0211	149.8338	76.29014	99.21935	440.9639
BPI	95.58284	88.90636	56.10017	81.99791	37.07304	81.15942	55.45465	42.67138	264.5834
SNX31	89.27515	129.8535	86.57586	108.0187	63.66641	76.30795	50.50647	54.82209	166.3727
CCS	2677.511	3602.961	3008.624	4084.305	2777.659	2380.103	2641.913	3593.176	3288.977
CENTB2	33.75484	45.9508	471.0988	797.8104	179.1056	324.892	118.039	178.3978	37.59445
TUBGCP6	41.3459	1674.109	2084.367	41.37079	3131.768	1800.424	2768.019	3477.073	47.16717
TK2	108.5475	69.08226	72.28194	57.82687	54.44501	53.34891	65.38712	54.92603	59.0502
TMEM126I	1098.228	335.3043	557.5587	1034.032	285.2433	1010.914	516.5302	174.0883	58.71916
MSL2	462.584	273.6644	182.2249	286.7417	120.5369	285.186	177.0516	285.4211	527.7819
EVX1	80.88313	77.46494	60.99558	149.5247	67.83958	89.94793	64.83797	61.0923	95.30625
ZDHHC19	61.23384	43.57588	988.546	65.67731	436.9435	1990.458	745.3589	1079.971	144.2031
MFAP4	9767.627	10898.32	10882.95	5296.201	20979.38	14031.94	9943.771	9957.257	8356.661
RPAP3	343.2213	236.1358	77.81641	213.6906	75.36115	121.2061	69.29579	105.9885	441.3646
KCNK5	180.4748	227.3079	99.38492	159.8282	73.44937	135.1435	74.51974	114.7252	164.512
CDH13	47.20197	38.89882	447.4486	568.1279	1063.248	754.0098	1560.478	683.7177	1651.036
PCDHB13	1438.894	1006.635	685.3743	465.5996	455.5746	239.2424	229.0432	523.5119	194.6834
MSX2	413.8522	251.8473	102.8895	257.7756	114.4188	137.2732	90.91962	89.91693	215.3846
VCAM1	560.7088	656.7058	409.6992	510.3802	102.1362	577.9663	212.782	174.6514	86.22176
UNC50	78.44161	2384.652	3784.69	515.5652	5071.673	2733.98	4181.542	4098.272	2234.558
DIRC1	112.0321	102.2372	46.4013	60.90433	49.418	44.1595	50.55825	46.85344	54.44901

ABRA	64.49857	132.9402	64.0951	110.7489	69.73873	65.75362	56.51304	66.02235	223.5639
BCORL1	57.34153	103.0109	40.32047	57.70127	73.43002	34.54596	69.38565	126.8309	109.7514
DENND1C	1849.276	1500.543	2472.462	909.9935	509.065	1868.434	1457.418	2291.968	3578.784
DDI1	237.8743	198.9548	113.6118	194.2498	123.2719	158.5022	96.08508	143.6028	469.1255
PF4	33.8922	35.5896	42.38233	37.13243	32.54134	29.08649	38.88826	38.2738	41.46201
SMAD6	2861.384	3128.628	1272.806	1477.631	1838.56	2228.995	1429.179	1128.828	3320.858
VN1R5	75.36687	151.1239	63.5339	95.19462	75.43271	97.66575	57.81241	81.54264	210.718
MYH13	87.87653	56.58553	984.0611	708.1707	46.81274	2029.837	285.0528	48.29456	57.89881
ANO10	3085.435	1105.705	832.67	572.9896	842.097	136.8011	1296.046	921.8752	3070.311
AOX1	40.10796	36.19218	122.9925	35.33933	88.92175	212.4129	128.2644	112.2953	36.64471
ABCC8	224.2419	135.6909	255.4075	57.44108	279.4282	741.6466	308.3675	506.6909	270.1365
SOS1	56.39952	587.8785	447.0539	765.0029	187.6013	604.6481	273.4418	317.378	59.00332
NDUFA13	578.6445	164.9216	479.7251	171.9438	628.5549	390.2787	732.5486	788.4659	425.4639
CAMK4	45.26016	46.18457	369.3662	38.96199	50.54258	44.18102	166.8675	124.2697	44.26403
EDA2R	35.73462	49.19857	78.63855	46.24817	67.64361	41.04951	90.95249	35.90568	45.73705
OR13A1	81.36179	55.18097	53.72625	67.7048	201.8739	47.33774	41.5945	50.23655	1684.905
RRP12	51.9369	36.23804	608.2778	34.92879	2070.184	46.87525	1376.729	1688.556	45.57541
CCDC138	143.6717	97.27112	50.44366	74.17971	56.02866	45.20238	47.76406	57.97522	114.4633
PPBP	394.7002	321.3206	105.9745	208.165	283.7838	200.1574	182.4745	406.6743	806.4278
LOC400965	205.3119	120.5171	75.00269	166.835	70.71677	97.03429	62.62123	67.25536	286.6079
CXorf20	47.91648	55.31601	33.52667	36.94304	35.42579	33.59882	38.693	34.16075	33.1294
EDAR	35.74401	40.51678	44.89925	56.26232	143.6914	46.97973	42.60985	39.57127	51.80254
EIF4EBP1	298.9189	104.516	85.76946	667.7153	112.8106	130.8997	216.4404	106.3956	214.9433
SFXN5	50.54906	43.27145	35.47218	36.38115	341.3984	645.9033	299.4927	218.6078	52.40421
NTN5	38.95114	35.84047	256.4783	30.08933	835.3765	508.9355	225.1541	709.4644	35.57076
ABCB4	40.29555	32.50485	132.2776	43.34336	36.80214	169.1965	147.45	183.2929	35.09103
IL10RB	75.5611	39.99217	40.23498	36.77189	37.54831	37.68826	88.87089	147.325	67.87559
ADAMTS6	52.41344	94.22833	160.9845	396.4768	106.007	48.71381	155.6262	33.46685	48.55174
PAK1	185.489	533.9589	952.2038	1262.064	1517.784	1236.159	1116.666	1957.122	1609.515
LOC153561	207.5362	101.6496	96.75037	95.20076	76.03847	163.4972	60.68517	85.65028	119.2175
GRIA3	319.6753	127.9484	64.92471	148.1787	60.08445	97.51405	63.65455	76.46143	462.973
IL1RAPL2	45.19996	43.88947	45.93568	72.41922	37.05487	46.38619	55.76752	52.37789	103.6051
CDK3	239.7733	153.7613	72.89815	152.1086	204.7666	113.211	124.3046	64.05968	174.4995
CES4	40.94485	35.03976	50.77459	43.52753	37.02312	40.90757	47.95146	35.14592	34.7919
TBC1D5	2062.207	944.9207	265.8306	33.27257	40.84834	549.2376	409.0511	37.91155	36.96802
FBXL14	45.60834	46.89924	36.77098	42.45395	38.84445	48.10382	42.27134	41.8796	36.3592
RNF150	7698.238	10372.25	5987.503	3188.407	6321.78	8403.856	4649.946	7580.951	9795.324
LHFP	1567.789	2111.924	784.3179	2642.901	876.3516	1203.633	563.608	87.5576	282.2606
POLR3B	12281.53	11938.45	9941.146	10426.43	10112.72	10676.48	9389.786	11052.21	11957.63
SCPEP1	210.4804	524.3154	1028.09	502.9628	839.9102	834.5159	966.6198	1668.407	1484.364
XRCC6BP1	57.66465	53.35705	55.48096	57.50776	155.8132	63.18157	44.27299	53.68293	135.798
IL17RB	39.04054	39.40287	46.06882	35.20153	66.62814	125.9456	54.04153	72.43114	45.4182
ARAP2	34.66102	37.79688	47.53414	95.05018	157.2992	55.0749	223.0113	126.5886	59.68784
THAP10	41.86819	43.77231	43.17073	46.15179	37.00945	36.4295	66.30785	48.72179	51.2805
C10orf10	3801.618	2284.043	1480.634	3034.047	938.5552	1081.001	829.0197	1453.395	2944.942
KRR1	59.82543	47.29735	450.3559	56.2879	39.70839	44.06277	38.67953	40.53674	113.6
C21orf94	78.33147	68.33743	46.23529	61.96048	45.45137	68.72352	42.82512	57.20843	48.21241

BBS2	44.03582	74.18803	92.73163	42.1099	90.39927	51.52369	59.22064	87.91956	37.35199
C11orf46	58.63124	109.2121	52.73861	62.79548	163.598	238.1	73.0191	53.83022	78.87277
TECPR1	188.3643	1355.37	1879.718	2282.46	889.9973	264.7403	559.0454	856.7044	2610.152
MAT1A	44.38778	37.8203	53.77826	41.02461	43.25568	44.14836	42.21172	59.52298	40.92485
RRS1	369.6161	324.1245	100.1911	417.7168	150.115	197.8203	231.3609	146.1692	36.66162
LASS6	862.7571	277.9211	856.9047	450.6808	999.0738	720.6547	973.942	625.9602	42.15037
LOC49386	5319.563	2712.083	4289.085	5977.685	2393.039	4928.56	4242.698	3905.491	3319.799
SCN1B	209.8381	63.91293	51.53231	61.95987	52.07459	51.14686	40.08458	54.47329	59.91797
SERPINB7	35.65635	58.71568	37.40094	279.9803	67.67549	56.95975	50.45214	56.39201	755.587
SENP8	39.60798	39.17694	281.7663	42.11427	102.2012	194.5606	175.4761	105.8497	57.18718
CASR	47.91345	50.86611	46.02581	46.30217	36.86795	49.83944	38.60909	35.9337	46.10778
AFTPH	74.76013	44.38261	50.08316	53.47036	59.61734	54.2468	52.59829	54.62344	53.29704
HDAC2	5666.567	4178.623	4255.369	7290.705	3969.59	3909.027	3210.378	3076.89	5512.529
SAMD10	186.2095	66.85016	66.86664	159.184	175.0816	68.63784	226.1046	156.1717	387.2601
ZNF416	64.92562	51.44738	380.446	83.41985	358.4068	448.4567	267.474	46.14618	35.69241
MAML1	73.20187	592.1864	47.03889	57.86525	37.44718	52.61304	54.54463	39.52378	101.6812
APH1B	38.45758	45.60374	41.68987	40.98797	39.21707	33.97103	49.83019	33.62803	37.99488
GRK7	39.46373	36.51295	35.03301	39.44609	36.66393	34.07671	39.15293	36.87874	47.33512
C10orf120	36.845	39.69697	33.3135	51.34916	38.8815	45.89101	47.05089	38.80909	35.69305
C9	39.07069	43.88662	54.85865	68.13142	32.03236	43.93929	45.49293	66.23186	44.58724
GOLT1B	153.0395	77.47311	74.25402	36.38591	79.90038	51.2496	65.49129	56.35749	33.96636
SLC17A8	38.16265	51.32478	38.57131	54.2012	42.97569	43.56173	43.79275	49.18966	49.14356
PCMTD2	33.69004	44.63287	45.74476	50.57549	36.09312	43.40742	141.7194	45.60436	39.58963
SNORD15A	896.2184	425.3932	168.4333	828.9698	169.8762	278.382	173.002	325.4923	34.40164
DUSP12	279.5096	220.5213	363.4633	224.5864	317.8651	273.5977	366.1722	379.9442	444.6652
POLB	951.0756	1881.966	2645.219	3331.19	4812.58	3734.155	4139.544	2494.617	49.93828
LGI2	294.9272	125.1061	103.5009	152.9866	102.5051	163.4585	131.9765	84.3371	442.0104
PCSK5	282.9286	631.1072	469.3657	215.6322	806.1777	794.6574	960.8495	282.3976	613.6866
NSUN7	48.20437	70.19462	46.97484	56.84946	48.62169	65.23038	119.1781	51.52226	115.3921
SLC12A6	776.8756	375.7891	403.9274	76.90335	432.3717	480.6331	716.5737	726.8314	220.0571
C2orf57	134.8457	70.15872	69.26167	63.61901	60.92859	67.31511	55.58996	59.6395	79.75499
TNFRSF17	48.87963	55.01205	38.51081	52.87341	148.9115	50.62377	33.69255	44.15782	39.48014
HAUS8	233.1236	369.9997	506.4726	135.5712	511.0162	249.2157	797.295	530.2834	1037.036
DEDD2	1098.383	1423.756	2260.617	1997.712	2436.584	2005.565	2216.599	3235.112	366.4453
MMP12	364.2333	206.5986	106.9707	257.6793	90.16677	168.0469	97.3254	107.578	561.9622
FOXP3	193.1086	101.9117	110.7563	79.19848	86.28791	148.5413	70.93468	119.3672	170.5559
SCOC	1464.226	978.8857	1624.486	1882.065	888.8542	1598.284	768.7528	552.8881	928.8014
GFER	113.3325	119.8302	63.95132	81.81057	60.90549	74.53529	68.17924	82.29826	152.3538
ADK	37.30331	34.79681	159.8181	38.64094	33.16187	29.84936	45.42829	34.30026	58.3714
OR52I2	38.12447	34.57725	32.79946	56.4539	31.87086	32.23697	32.36962	33.16328	33.95577
C2orf50	262.0972	82.03605	83.28566	179.7409	119.4231	96.07568	89.25029	118.3693	400.7359
RPGR	1288.906	719.3891	741.5948	972.663	921.8303	530.002	757.7426	514.768	52.27143
SCGB1C1	49.68243	591.0039	41.03366	47.41336	49.29597	39.44769	106.7055	35.60758	163.109
TSPAN12	367.0812	477.5612	425.3264	259.5152	463.2703	116.1096	258.4241	174.3089	140.0325
RBM25	7590.18	9456.077	8326.886	7909.347	6427.864	9383.232	6500.401	7760.394	6812.38
C3orf48	57.06216	50.15362	42.80183	51.64908	47.67633	70.06754	45.6806	44.05264	72.92002
EBNA1BP2	1998.575	1481.085	3101.413	3746.991	2984.431	2107.113	3708.928	4575.018	1057.303

RNU12	46.02773	71.58867	38.35196	38.85201	38.54718	44.88172	34.43933	42.26842	49.17301
OR8H3	192.1508	120.8648	93.77223	146.8737	60.65488	114.488	74.96095	78.00147	348.5671
CXorf26	3824.038	3818.319	6246.963	3230.031	7493.546	5151.45	6502.018	5892.775	31.14252
TOR3A	180.6003	471.2087	236.6007	669.6046	456.2842	120.9894	286.2209	297.8936	1153.61
ROD1	5247.103	5977.03	10118.32	5162.553	8880.307	7178.026	6943.779	8518.639	2812.973
GAS6	56.18436	40.92482	72.9936	341.2464	38.84894	37.23827	54.12134	40.70024	32.90595
PKD2L2	73.21498	73.55335	64.21134	114.1571	47.92842	95.55824	49.43199	66.64396	195.5566
SMAP1	49.20681	39.54923	38.27597	35.25149	339.2935	42.79874	279.4267	205.5257	35.51785
POP1	50.74534	55.52781	36.05856	43.27546	39.54535	45.58455	157.4145	36.9664	47.6973
SLC22A10	143.2344	62.06274	59.27023	90.91119	49.34551	61.56911	46.25211	45.50695	70.76064
NME6	69.07877	74.24133	44.4854	61.90139	117.0537	43.16156	51.0199	39.94056	43.80961
P2RY8	59.52599	68.69884	254.6362	56.57833	348.8325	74.06131	70.11468	394.6929	60.84093
C10orf31	39.26266	91.94433	56.19466	99.65871	49.15073	76.67027	58.25453	64.17237	114.7705
BAP1	693.202	849.9324	669.4917	38.62663	1224.519	736.5685	919.6666	833.3894	816.6354
ARL4C	80.93853	58.34391	89.53989	137.5035	89.30853	121.0159	84.38087	74.53339	222.3113
SNRPD1	73.95314	263.548	110.7114	243.9003	89.48335	193.118	109.0889	116.671	153.3368
ARPC5	5305.236	4699.105	7902.727	6002.977	9324.498	8380.5	7979.054	8317.615	5035.166
AURKAIP1	20038.51	20819.1	19147.79	21476.4	14513.11	15909.97	15080.44	14143.73	21529.49
ARFIP1	1700.824	65.33797	45.60098	39.56304	42.70723	58.17089	44.37574	41.19177	48.63409
TF	35.08151	33.20188	891.7997	38.08171	1632.422	1397.296	377.4913	704.3575	1377.766
FLJ46156	114.6937	38.2829	38.42601	37.25238	36.83614	35.09638	37.91981	45.63185	47.79902
CCDC71	125.1793	87.56212	60.65143	116.9716	264.3854	88.5252	584.4808	255.3205	228.7987
PRPSAP1	1912.55	2010.453	3697.936	2099.144	5304.04	3022.881	2937.91	2400.705	64.37979
WDR21C	91.24897	75.19332	50.45998	81.89288	40.46639	65.31852	61.99939	54.41018	93.40116
TCN1	40.95654	51.577	395.4644	33.42401	157.3197	69.54347	471.3349	291.7992	31.0407
VGLL4	43.69527	354.9843	1246.99	889.6812	1219.084	1297.875	1417.638	1077.245	40.28785
KCNK3	331.1128	199.5298	103.2	219.8459	77.69778	152.0499	88.08994	93.15017	534.1658
TERF2	38.74547	50.60357	856.3254	58.30584	484.7673	502.6146	526.0534	523.3781	90.79099
IL13RA1	33.83884	184.0335	2427.197	49.5082	4237.556	2278.055	4143.815	2796.839	71.94497
MAPK15	179.5171	169.492	88.14861	891.4227	350.9849	110.9127	634.8743	114.011	390.0042
MAN2B2	6016.292	5994.995	5935.479	4875.711	5045.406	5649.257	5033.436	6975.927	7335.775
CACNA2D1	44.17188	44.65031	450.5694	53.87388	173.1982	38.33319	105.4755	38.35543	35.65271
BHLHB2	4084.667	2906.453	10973.8	8141.509	11685.82	8115.528	11726.77	5376.456	3478.91
CASKIN2	123.6631	163.6145	83.87154	112.6195	74.92889	131.5428	81.6824	111.2377	161.2784
OR10S1	282.5741	140.2127	76.11626	162.4379	77.41025	125.422	65.23507	77.81241	376.1743
SFTPD	1197.272	58.61151	634.1561	783.7417	624.3771	637.7123	711.0812	793.5215	64.46812
DLG4	63.25055	57.26062	39.68054	49.12395	102.906	51.75758	41.28953	41.38063	72.81715
LAIR1	233.4814	124.6897	109.1983	150.4101	78.02359	96.81796	66.03221	68.66074	261.8345
AP1S3	33.52369	31.42106	44.88998	36.96424	41.8246	39.11689	42.95887	53.58942	38.43744
CETN1	44.78916	36.85506	51.34135	56.96605	43.46215	33.76489	46.5214	37.39492	55.67155
TNNI3	122.7122	85.53949	104.8766	98.28115	59.86129	64.80204	73.03148	44.24253	121.3098
CRNKL1	34.91183	41.32817	35.20931	36.61792	209.111	33.76714	126.2691	34.37479	41.04365
UCK2	1206.267	594.1923	70.10279	72.2977	347.2816	577.4438	483.39	779.4234	1687.68
SYT4	456.1436	78.68427	41.80855	54.5434	175.3049	63.71696	393.3067	650.9675	64.9155
RDH11	9067.619	6788.56	5840.776	8809.02	5281.736	7192.825	4708.375	8104.928	9266.12
DPPA4	119.0789	327.8034	492.4868	337.2805	48.90429	239.4203	155.2094	181.705	101.5228
FIP1L1	3068.455	1558.148	2268.312	1122.59	1364.11	896.8687	1339.817	2218.118	446.4126

MTA3	234.3613	108.8732	558.1633	85.68581	397.0386	292.467	333.7835	292.9907	396.4977
SCGBL	170.347	133.8943	83.53035	101.8728	61.95917	101.1193	64.2932	73.24342	240.7969
CACNG3	101.3924	99.80411	63.9413	85.27668	57.00592	70.41865	53.51849	72.08771	107.5603
DEGS2	72.18826	55.98227	284.3426	63.78703	672.5544	38.75339	181.5336	53.0429	58.56017
EGLN2	225.2331	600.8456	591.9382	157.3512	860.5892	369.0959	689.1987	883.0886	477.8995
PSMC2	48.93599	1562.947	4844.114	2428.201	7019.623	4835.238	6480.02	6146.986	48.61009
UTF1	48.2555	73.94027	47.76565	33.93714	35.94871	37.98559	37.79922	35.66421	90.69216
RNF113B	284.9758	125.2761	84.21297	174.0484	74.92793	125.5395	75.40354	60.96754	424.7708
ZNF134	82.48828	163.7174	69.80217	95.20568	63.2725	73.66109	45.67092	59.82974	106.3257
FXYD6	33.52156	978.2598	603.5947	387.0461	1090.409	658.4282	567.118	456.183	729.6259
KLHL6	40.22607	509.2213	161.5648	40.37311	122.5578	57.75968	41.74069	45.58868	52.99301
LRRC46	227.2274	82.07724	72.81715	171.6768	74.94463	201.1189	67.54365	96.38308	257.2021
KIR3DX1	53.62313	65.96572	44.70009	48.74216	46.86499	39.93406	44.54417	43.39721	134.0982
CYP11A1	1341.161	775.2836	395.249	1360.592	357.1921	913.7629	543.1411	818.6932	538.6455
ST7OT4	97.71533	53.54474	43.36027	42.63322	49.28885	50.12017	41.20312	40.205	103.2798
DNAJB8	419.3895	236.8143	114.3009	263.8219	180.5761	211.5474	128.9297	162.186	610.6321
C15orf37	312.735	209.8285	100.4465	181.5984	62.15536	96.85709	58.82695	77.61441	504.867
CHAT	106.1351	57.9925	40.52377	42.15071	37.24805	41.65372	44.62087	45.08244	50.50097
AP3B1	71.6412	92.19688	233.2873	145.4761	637.13	379.7653	769.0875	525.3661	57.42818
CAPZA2	2942.304	5878.589	9778.418	7234.906	12759.25	9733.216	10476.82	8791.399	5334.306
CCDC7	128.9451	125.8689	482.4887	163.4651	249.6904	646.7486	64.25529	64.6517	139.1556
OR8G1	54.17367	37.60299	43.2132	60.26948	46.56395	53.56919	39.91286	44.78624	172.6514
IL28B	52.10227	42.96683	45.74023	47.49036	34.53952	32.43361	35.44247	43.12773	35.39777
TSN	4937.104	5194.28	4409.139	5001.023	4312.521	5398.046	4103.953	6335.803	4292.638
FLJ35880	190.9568	88.11711	75.77497	121.9416	136.4875	184.987	66.68241	52.5773	280.6302
MGC6210C	54.97808	48.39983	82.46522	43.29605	69.79202	112.9441	84.06358	42.44238	37.51801
C1QTNF3	45.41581	47.67087	234.9023	37.04969	125.8276	670.7167	264.473	406.6379	60.27239
U2AF2	14435.64	13123.94	13342.9	13155.75	11197.75	16388.6	11905.09	8143.866	11958.26
COL15A1	3910.283	2355.746	2649.659	1240.393	2159.85	1730.222	2161.356	2733.724	3147.858
KIAA1377	159.4069	297.9894	88.86002	146.9108	263.7708	127.0855	553.4019	302.1055	327.7108
SDC1	336.0125	224.3449	94.16395	318.2028	86.03817	115.9437	73.65519	90.60357	505.2495
RASGRP1	717.3675	100.5387	659.5806	85.75221	416.817	232.3645	382.9625	507.6574	220.5306
ATG4A	32.53178	216.3733	79.17151	298.627	33.42392	36.97214	38.33145	34.71379	499.2694
AKAP8L	999.3248	3131.777	676.7813	744.7247	1599.608	1047.265	832.0742	838.6573	1981.353
LBR	79.56465	88.69637	59.95301	49.58918	58.62452	91.09194	56.91598	75.30623	41.46944
HIST2H2AC	1781.362	1682.295	1007.441	1245.529	650.9124	1410.722	1189.57	658.8829	1321.355
GLIPR1L1	335.2392	193.6279	89.15674	199.6228	113.9314	177.0258	83.91245	133.3227	423.7705
C20orf173	35.03999	38.25935	36.50357	41.61584	38.19019	38.39175	35.59251	33.03474	33.40868
BAGE	247.2022	166.9571	85.51551	206.608	95.95296	100.6148	78.52744	104.347	359.0831
C8orf79	553.6849	308.8427	148.3886	273.5026	148.4273	103.1758	101.4523	117.1351	544.0884
ASGR1	808.391	390.873	475.5317	638.7264	303.4149	563.4481	467.802	414.7419	606.9392
GTPBP5	491.2257	261.1875	142.64	269.606	98.96072	168.61	80.31965	128.8599	647.6917
PCSK4	608.8568	280.4495	485.067	742.6541	288.6243	573.8489	269.5018	394.3935	1170.658
CDC42SE1	1440.947	1176.378	734.7315	638.1241	366.34	722.8136	387.813	467.4569	2083.237
FRAP1	53.58693	55.73315	58.14378	43.25996	49.58196	56.79423	45.62545	149.8074	126.3686
GLYATL1	75.66714	90.97058	70.8473	58.93697	65.29427	79.47287	52.70626	71.20667	131.8955
NCOA2	44.51186	610.4017	38.57109	38.19056	39.11146	350.4325	314.476	39.8679	68.90555

XKRX	185.9325	62.88646	74.84052	102.2287	235.3194	83.96043	154.6804	113.2344	93.87719
TXNDC5	5044.354	12262.42	1863.635	5723.281	3150.527	3455.144	2395.005	1738.426	10551.88
PRCP	16116.91	16493.95	21266.71	18087.83	18020.19	17880.89	20491.4	19382.95	15792.68
FAM176B	257.6103	166.2822	558.9434	226.2921	305.1428	116.8007	823.8757	1090.057	222.1732
ZNF253	575.0637	383.7159	124.7958	304.0916	134.8105	315.9411	90.11777	146.3231	73.11693
MAFG	87.25807	79.91396	66.95657	74.30668	93.7479	105.646	50.58551	66.45841	102.2645
ZCCHC10	223.9006	139.0649	90.94778	145.0955	86.50609	111.6034	68.30875	74.04504	335.2422
C2orf58	97.36528	223.3008	105.8604	85.08684	89.33537	62.70371	69.34627	52.84427	90.11486
LCN12	71.26199	64.95583	442.7301	107.6996	252.3103	594.9518	149.4509	182.872	52.033
Septin 9	17684.68	21115.82	23332.62	23563.28	24153.76	23968.66	23809.99	26143.45	16107.18
CCL19	12606.34	8901.395	7059.088	2746.251	3298.233	3244.52	2095.854	1833.618	12006.22
KIAA1958	1348.427	90.85611	221.3466	165.9229	74.07846	705.8513	392.0092	259.8155	302.486
KCNE2	65.34756	72.78123	68.11484	75.0109	54.09922	69.71009	53.38723	56.85699	66.23254
YIPF7	73.12617	52.07964	237.0214	87.1567	44.28531	47.70551	128.6482	57.51167	58.19562
THAP5	77.47559	357.0386	243.7629	502.5064	100.9594	338.381	165.625	174.903	54.3832
CYP2C8	80.37016	90.19369	65.07249	96.14343	57.2083	62.74804	55.38505	57.18856	193.3974
ISOC2	1427.852	1038.669	1727.919	484.5993	1496.637	545.2754	1329.203	1719.525	993.5379
ARL10	85.66605	649.7497	55.13251	49.61669	105.1468	39.04521	89.04378	54.72653	106.2219
GNAS	8143.956	4897.909	4976.367	7600.848	6282.241	4084.069	5271.415	3309.214	5560.839
ERC2	42.89046	32.33395	131.2936	46.02318	85.79099	34.34435	41.55923	40.52552	36.77087
ELF3	3742.383	2801.382	6656.657	1931.9	7205.009	3573.148	4942.197	3569.604	6155.262
KCTD7	65.02375	378.3622	44.75581	668.3634	162.9185	36.09864	126.5555	56.20519	66.41183
TPSG1	95.76092	95.83806	259.5087	70.99979	237.8543	70.3515	60.73468	107.8811	103.7671
BCL2A1	383.9393	232.0508	144.1024	252.8113	116.5816	179.2183	106.3169	160.0907	519.5626
GLIPR1	40.38334	38.651	40.73276	32.62032	34.49304	41.21988	70.83844	170.8881	45.35032
ARFGAP2	662.0867	278.0698	262.319	222.6841	248.4426	792.4801	363.3825	522.7775	1017.466
MCCC2	34.99469	385.9195	505.5885	412.8951	121.155	345.9374	185.7677	394.0677	44.72027
FRMD6	50.7866	79.02829	163.1675	103.1457	138.5673	535.4709	251.6252	68.59879	1525.284
TTC39B	121.0279	86.63132	1538.982	54.197	1629.764	1327.614	672.4866	767.777	91.36249
MTMR11	46.45277	2031.254	2993.553	2172.873	5614.332	4180.913	5480.961	3354.441	55.10114
CNBP	1651.177	2763.144	1732.752	1944.337	1731.304	1457.035	1370.2	519.2798	2349.78
NPBWR2	120.2049	115.2536	57.21518	70.3437	64.77176	76.35004	53.24204	50.35204	210.8305
ELAVL2	177.4214	117.1772	40.27193	74.42104	102.6635	41.21653	41.51875	51.87871	56.63094
OR2M2	55.38241	98.36356	50.2289	52.22863	36.49923	53.14344	45.81082	50.52617	56.54241
GIMAP5	305.3129	396.4806	456.6115	214.1289	643.2143	573.6918	974.0836	468.3157	447.2304
GJB2	4629.576	4321.804	5875.094	5977.096	9374.867	5335.32	5424.466	8070.799	3110.473
ABHD15	52.31603	557.4615	1189.538	550.8269	844.0725	609.6685	646.9709	698.9774	121.6078
PLCD1	477.7585	516.9196	133.5311	443.8362	334.4078	361.7237	222.114	118.4088	435.7456
C9orf123	101.1472	2250.099	1617.85	89.04141	2000.254	3630.783	2611.74	2831	172.6371
IQGAP2	328.7107	484.7286	712.5322	369.6683	571.751	1042.122	518.6633	545.9319	58.53611
MBLAC2	136.9122	410.1027	452.7427	107.4876	236.8244	458.9103	139.3008	356.4546	1192.608
PIH1D2	39.30943	35.18341	34.07458	40.87785	46.32598	41.54588	40.07125	35.02739	53.66666
SPIN2B	314.8867	44.02923	300.5486	975.7439	83.03442	116.5861	216.0522	250.0007	3228.873
SETD2	75.56765	1776.891	1968.389	848.3907	1466.013	1079.247	1447.768	905.2405	56.36521
CACNG2	73.65797	112.648	34.11382	46.10631	47.39241	64.89024	38.93034	48.87703	88.69478
ZNRF2	5708.838	1706.142	3658.867	3360.341	2365.179	3467.417	2539.339	2068.555	2672.971
PIGX	494.0485	615.0536	573.4407	616.4781	344.1049	445.6285	528.8098	988.5936	882.2432

PRKDC	11958.26	12769.3	17041.04	18891.63	19770.52	17379.26	17916.48	17183.67	9708.438
C9orf139	58.79404	32.4623	31.56211	48.81625	43.57171	49.0919	36.10662	69.69569	44.17144
FLJ43276	484.9178	290.9231	162.8817	380.4011	119.6782	200.282	132.9831	152.8353	673.2265
PRPF19	41.85119	54.74083	50.48949	94.99682	62.99368	94.80362	51.36916	48.91058	73.74216
RIN2	43.22166	1242.078	2405.816	1770.06	4865.221	3416.623	5914.667	2593.768	56.95438
SLFN5	1038.631	628.4266	639.184	917.0787	248.5647	448.2034	486.0932	200.6118	1921.175
C5orf23	39.94064	34.3979	39.58523	32.85203	111.2216	39.03932	37.02532	34.0947	35.39256
GLIPR1L2	60.4779	37.43745	46.77708	38.50445	35.01978	37.11039	43.19907	43.1064	43.00432
AFARP1	77.19547	62.78276	56.35472	110.341	53.25537	56.6362	43.51578	50.85956	114.7866
ATG3	791.7949	48.07972	567.4639	554.3707	1042.671	854.0204	623.9822	1084.532	41.03281
JAM3	6006.513	5217.718	5942.437	4574.477	11710.9	10080.43	8662.175	10671	4281.797
PRSS35	43.29939	39.66964	43.29324	61.56742	91.22033	46.55651	44.83621	57.28275	38.2509
MGC16169	69.76563	81.31127	277.8275	107.0906	252.0435	750.0425	241.6175	57.41838	164.7545
PIWIL4	68.99602	35.14429	34.61777	41.31656	38.99387	33.76895	85.97083	46.10744	51.74855
FLRT2	170.28	73.7014	272.0065	521.7845	64.9531	264.3895	254.6708	69.5449	81.31168
CTSO	501.3062	114.697	667.2196	497.9779	1045.412	699.1478	1084.903	515.6317	239.4883
STRA13	7682.866	8311.93	9334.717	8265.224	10659.5	9411.468	10245.88	6677.836	8050.835
RNMT	62.21207	417.2436	1012.423	743.0786	1113.667	813.0188	1070.512	1036.176	67.34251
LYSMD4	168.7103	126.2838	85.35805	116.8219	84.16234	96.33381	68.49508	112.6259	208.7669
RAB5C	49.13649	210.6296	316.0545	362.0858	453.3755	451.7931	754.3143	491.343	40.02864
GPAA1	54.46746	51.62074	58.12885	31.95829	50.75153	209.264	40.8411	55.86441	48.91407
STAU2	55.72368	339.9491	82.80506	91.63938	367.8438	69.14736	306.6115	310.2367	36.00634
SP6	182.1049	147.1742	85.8774	185.5335	66.84057	119.2724	83.52587	84.50675	287.5685
NT5C3	150.1066	102.892	250.9607	318.9777	137.1111	91.0386	221.5098	126.0955	146.269
PIGG	305.901	197.2971	100.0101	155.7889	77.70764	145.8897	60.98354	108.58	287.6648
C14orf104	40.01506	70.73456	36.67833	51.93685	34.21251	130.6982	79.22106	48.43921	45.19292
SERPINB13	16480.61	12413.74	14163.36	12976.69	15969.07	14494.7	12117.97	17548.71	15604.84
CDKL4	85.02139	43.53665	39.95457	66.71586	44.15844	37.66178	44.00463	45.57415	45.69686
PTP4A3	40.57704	2289.633	215.8407	1083.926	167.6479	504.5276	101.7219	766.0745	2055.688
LAX1	208.3362	615.1298	215.8346	149.9982	71.99467	113.3493	79.81483	81.46159	138.6998
ZDHHC2	89.27082	104.6653	71.63177	306.1543	86.76273	85.45679	57.08515	59.37363	238.1181
SELO	53.0981	796.7532	1921.583	38.58277	4128.404	832.887	1918.344	2703.209	37.78123
C6orf142	42.42982	48.98626	45.73626	58.7014	48.83886	53.1394	70.76345	123.7697	40.11181
ARL6IP5	16922.8	15266.63	14214.25	15160.19	11589.39	13411.72	13552.87	12525.37	15779.51
L1TD1	515.4773	175.2322	568.0187	105.3537	213.3391	84.50811	278.0122	326.195	301.7801
HORMAD1	38.55821	46.83593	35.36877	51.52534	33.64281	58.10021	34.13272	60.8466	33.89365
PCDHB9	523.1458	268.6423	45.80759	64.1777	90.57505	83.32871	54.01334	51.6371	50.94558
PIP5K1A	385.8433	216.4087	142.8242	181.7351	148.5433	190.4247	111.9093	140.1593	428.7436
TBC1D2	650.7098	321.8829	2721.203	1211.762	2564.41	2953.541	2825.208	1944.441	108.7173
IDE	50.02558	39.47709	38.5786	36.30562	1001.582	37.97653	259.8537	299.3209	35.30041
SIRPD	129.2191	111.2615	84.84627	160.4723	76.95231	123.7709	67.09633	65.17093	262.6993
CDH4	104.4909	94.97021	50.42337	68.01253	56.23759	86.08363	118.9657	66.1075	128.1745
ULBP3	160.1406	79.60569	68.90881	70.93388	154.3833	95.09673	54.05096	268.5552	198.7524
SLC38A1	119.5566	159.1669	164.8812	509.2433	458.5695	197.1948	417.1096	385.2454	756.4096
OR4K14	82.02985	75.55621	55.64434	60.81829	38.38268	68.00484	45.57489	49.95402	94.37551
C2CD4A	625.2645	351.8427	83.22146	280.641	37.98699	283.0619	128.5968	70.29985	111.7124
SFPQ	687.0641	491.6449	374.9418	443.8976	344.3069	346.3799	374.5121	475.5019	816.9915

GPS1	645.2482	328.7638	146.1414	330.9191	133.5174	209.7609	147.188	173.5506	666.2588
DPF1	165.2779	76.17612	64.15127	65.47388	126.7315	81.72249	101.4515	69.0954	85.19963
IFNA10	108.8057	42.95906	35.38107	56.60097	38.46923	51.77132	46.56395	43.48591	125.3134
LCN9	63.47247	89.39231	71.30213	122.4927	42.97594	38.67928	38.52155	53.83688	43.21619
PTPN18	1266.349	1028.941	228.6774	835.569	175.1618	561.2932	293.0032	204.1907	1968.171
KCNQ4	256.9813	176.5146	80.33787	192.4558	84.42386	97.94654	77.06795	98.23042	212.6536
KRT33B	108.8968	138.4356	346.5954	81.60975	3405.832	56.30853	1375.386	1010.089	96.02724
C7orf43	93.45584	74.29021	684.8504	366.2302	688.7769	416.5444	506.7543	450.1133	99.457
ABHD10	2429.748	700.0641	1288.558	213.8965	1704.429	493.9216	1207.935	751.0676	264.5972
NCOA5	67.07786	47.92529	303.6388	96.77054	458.4206	346.6378	188.1365	285.0214	296.018
CHRNA5	41.55499	52.76465	57.62963	52.59209	49.24131	61.15196	45.94416	56.21501	45.6018
DPH5	50.3704	335.3189	139.8874	294.3156	222.8431	51.77928	186.0613	61.75817	32.1824
RBBP7	33.82901	100.6673	133.2334	292.658	165.8075	196.8434	170.4846	129.9528	474.0081
TACC1	1318.573	2089.608	4010.893	2373.704	9321.442	3796.626	6266.559	2437.45	38.38842
BTG2	642.42	1619.143	832.5412	829.018	725.9234	757.0887	466.2819	533.0938	1424.806
MOCS2	65.2421	62.03445	44.00136	46.95938	162.4244	53.19313	38.85333	233.7158	50.69061
FLJ21687	2082.786	55.25875	57.33323	59.71754	460.2546	349.7753	274.5406	457.2506	117.4496
DNAJA2	3214.131	4000.154	4941.314	3490.703	5058.737	5012.439	5216.249	5855.567	2247.725
C10orf113	58.75944	1882.199	290.4659	43.14903	44.7606	44.96307	34.27073	47.56002	41.06661
BST1	84.37224	50.32858	50.62938	54.59649	158.2668	46.97423	66.56034	39.50033	114.6112
ZNF669	139.877	166.2598	76.26748	94.83561	86.8905	75.99284	84.07446	91.56246	90.30284
GEMIN4	33.58761	245.3106	838.0372	375.9539	1338.162	993.3794	1281.596	761.8554	90.8729
KRT34	37.38895	44.4852	39.85048	45.08704	172.9384	461.3171	544.278	274.6517	49.2569
CBX1	613.4547	423.3705	126.7773	581.8474	137.4467	162.4517	112.798	165.0285	209.723
EPB41L5	417.0944	310.7719	176.1444	144.0377	155.6379	303.6715	190.2991	305.8339	556.2593
ZNF354B	636.4462	795.6765	414.6578	680.3008	342.7393	660.1916	340.0106	239.1268	704.1965
KLF5	2632.616	3356.213	5897.534	4889.243	10066.44	5565.975	6125.085	5175.434	1297.658
OR51B2	51.64652	55.66032	51.8121	56.42114	47.71814	49.12782	46.2107	52.91855	52.9797
RABGGTA	245.1602	857.5872	105.0274	220.8819	124.7951	175.3178	239.4648	345.9605	294.3488
TM4SF1	20124.24	21406.95	18897.39	20331.79	22080.29	22019.42	16977.39	21807.12	17477.87
RPAIN	86.71695	98.6223	304.6665	125.1065	1364.847	51.05339	585.039	1371.838	74.62621
INE1	112.5159	31.6162	122.1169	54.57243	97.35971	39.42439	44.10529	39.86013	78.02523
POLRMT	239.8017	111.6736	78.44791	90.40245	64.39417	107.0514	61.33075	69.58607	159.7664
TMEM39A	59.73013	74.35421	206.2663	94.05479	55.33276	66.87741	56.13526	67.242	111.7893
ANAPC10	34.60111	52.55505	48.70634	39.81618	37.02185	198.2181	109.7771	308.1501	49.20734
FLJ46838	37.12508	80.78146	61.53305	70.78599	60.83462	79.79602	44.19124	73.63609	162.8516
TMPRSS9	123.0482	44.9439	57.04578	57.17367	37.40296	60.16335	45.13897	68.49023	39.2461
VPS39	41.40412	44.62172	196.4163	37.08269	41.76395	47.81702	214.1434	167.1929	65.9495
C10orf107	34.12209	38.84101	41.11441	37.66941	64.2862	41.32566	40.50202	33.68497	102.2082
ZNF385C	36.11373	51.46186	46.61902	37.98001	36.55683	46.75749	54.41297	58.01094	43.71331
AHCYL1	695.0024	994.7496	1797.563	490.4778	2627.09	1673.941	2210.023	1630.491	139.7193
DEFA5	122.7464	122.5561	72.67385	70.07762	68.15758	89.76534	58.96587	51.27323	108.9731
CDIPT	1892.862	845.6036	286.7739	235.2522	558.1408	505.1531	359.1469	1090.522	3412.074
PIK3CG	504.1531	660.8204	206.7683	66.08006	118.7909	151.5928	147.5257	43.25914	66.48045
OR52N1	33.29818	37.58959	39.64233	48.44009	35.16339	32.80484	37.81535	41.85711	32.23733
KLF16	59.98945	56.02561	37.02826	46.57017	39.45509	38.83398	94.72292	165.5497	70.33353
AGRN	430.2065	640.0651	476.2058	861.5046	452.1347	183.3763	1085.792	728.4577	554.0543

ATP5G3	35.71415	722.9058	414.155	42.11176	897.5206	702.0726	645.4434	593.5588	44.83055
GAGE7	46.82753	38.44689	9281.2	88.31781	72.96266	169.454	93.69743	127.4054	40.68483
LMO7	2426.388	4072.52	2497.25	4018.787	2267.004	4069.53	2786.463	4809.125	3977.993
CCDC37	49.60926	57.27187	54.14366	76.11595	58.84502	47.09685	46.47792	93.08611	81.5885
DQX1	94.36768	120.7158	67.23782	82.32633	133.775	69.33274	61.36929	83.7282	62.57272
BIK	37.20401	380.4244	143.2048	34.6559	121.4124	36.58599	96.40982	43.82235	32.54227
UBE2V2	296.893	161.1686	116.801	377.0155	386.1342	129.3597	199.4918	421.6178	1247.502
CLASP2	75.83882	100.9165	43.84895	40.09671	41.94139	39.88805	47.96334	56.37401	55.98396
MORF4	53.46969	63.37708	38.6047	55.20336	52.25607	36.19082	36.232	52.0274	37.74544
FAM177B	43.93044	42.26432	184.6237	38.66057	40.19956	38.71544	64.37997	36.7941	46.15587
RHCE	501.8142	1259.953	227.7482	661.0719	148.9344	456.1673	176.9772	241.2792	948.0477
MERTK	284.339	147.5999	67.27113	133.8634	67.44727	103.1508	63.53312	88.20602	145.506
KRT15	3439.18	3102.462	8707.554	3558.078	7360.238	8132.438	4798.726	847.9042	2009.703
FAM58A	48.32439	404.9546	327.405	1069.852	597.0069	840.567	1300.752	1349.124	1438.235
SLC17A7	155.4296	76.36319	73.66792	120.8655	311.7933	263.6031	170.8159	150.974	312.2404
ZNF665	51.17041	46.68608	33.86736	41.99673	38.35035	56.07276	45.06932	40.2569	31.50573
SOX2	1770.995	668.4485	491.7384	751.0964	913.2008	711.1065	364.9066	463.5277	1823.401
KLHL3	1449.429	2480.771	4199.387	2623.004	4085.058	4713.524	5140.221	3314.984	58.69977
MAL2	40.84528	322.8903	131.1214	104.4501	198.2819	258.8475	252.6722	102.058	39.76437
KRT24	269.82	172.6771	545.6052	208.342	630.8968	105.6603	1169.244	1861.072	3441.13
HECA	559.0784	61.12556	249.6918	237.506	305.9572	238.5229	216.2619	186.337	561.1508
AKNA	48.09603	865.8414	171.6865	57.10833	714.0634	37.596	217.0879	322.5126	70.01875
GLP2R	41.03557	53.15614	49.57407	45.47206	36.48277	35.76086	39.35838	33.35255	36.3885
TEP1	3275.401	3159.509	2819.909	3034.481	1152.779	1068.373	1350.028	2123.553	2371.247
CDKL3	138.7941	94.67836	113.7127	143.5203	107.0267	103.6505	97.70618	132.052	70.02142
WDR12	112.8931	159.0883	79.4484	170.346	195.6855	115.1039	226.0833	88.71135	367.6319
OR52H1	39.15451	54.12532	45.56788	48.14505	52.42426	57.25791	42.99929	69.25539	49.80537
BTNL8	117.7989	37.91931	42.18655	44.64557	32.31804	34.87944	35.87751	36.05357	44.23259
KIAA0664	563.0075	413.6435	428.912	367.0743	271.6393	248.0922	198.4162	331.5538	748.5758
AIF1L	740.5415	435.5102	767.6032	48.6587	616.7755	414.3586	539.4155	467.6598	824.5385
COPS3	2906.38	1286.083	4069.224	3072.942	4305.048	2698.579	4045.466	7216.897	108.7261
RIC8A	765.7466	82.21273	592.8944	421.2385	715.8409	389.5802	682.7071	472.448	217.7469
TMEM55B	55.02099	46.26648	445.0606	1678.92	31.39386	40.34216	39.88052	50.38341	76.44382
AKAP6	71.34978	65.37725	47.04811	67.58453	48.02521	39.08474	93.30993	43.47626	39.2641
MGC52282	35.28532	65.3157	38.54504	44.20825	31.55112	340.2148	89.07783	39.67993	38.31133
SLAMF1	36.54969	585.5911	291.6561	45.48786	126.6058	326.5932	198.0299	37.83996	36.46613
CMA1	59.52048	82.80701	60.08733	112.9723	55.54594	83.702	54.78096	38.03188	115.8212
DDAH2	70.76026	132.8195	51.07313	114.5029	83.86546	75.04012	66.1322	123.5493	109.6014
LOC44055[illegible]	41.24109	45.31195	50.49878	56.97233	40.03489	40.20649	33.49681	47.85158	36.53069
PODN	2161.32	2703.335	1776.721	1931.734	1707.368	2809.176	1939.15	2790.781	3070.519
BNIP2	1603.757	1872.678	1209.431	1762.381	916.4974	919.5831	775.8235	287.2476	1676.749
PELO	754.7086	282.8914	272.7389	224.1826	408.6162	763.0267	754.3844	843.4459	975.3158
ARHGAP4	474.2801	363.8496	419.3745	285.2484	330.352	253.5814	451.3049	810.4966	645.2687
PNMA1	50.42699	57.76257	219.3162	46.60175	174.5352	36.0437	74.83144	150.0809	41.97923
SYT2	249.6579	77.62338	45.56637	85.2678	63.20071	84.13609	59.81248	56.45332	50.6499
TRIM46	37.96238	50.22227	433.002	41.95779	214.8932	52.64872	177.6924	341.5895	36.4775
YY1	4194.32	4350.602	8492.661	6796.388	7481.747	7775.083	8081.434	8245.174	3736.586

SHC3	116.091	79.94382	77.40677	145.0541	59.35143	101.2706	214.7565	74.12093	125.6164
NRSN2	10596.37	9586.725	9814.837	12432.15	7868.403	12091.57	8302.595	12436.45	11579.08
TMEM45A	80.66145	84.81744	59.97992	44.97577	137.2434	300.3495	50.63218	135.816	124.3129
COQ5	388.6028	236.2958	987.2878	235.705	2725.195	188.6407	1934.269	2082.392	409.3759
PAK7	97.70562	132.0252	57.91761	64.81964	56.5042	65.8536	51.8582	67.47148	257.3223
CDH1	121.4242	100.918	222.0538	295.2208	204.0823	54.27203	198.5984	147.4693	115.4408
NAALADL1	795.5459	2520.361	3145.513	553.6208	1378.505	2004.028	2251.259	1984.642	1672.917
TMEM134	83.73317	55.21891	1162.667	73.339	750.8423	66.42739	448.6962	973.0316	76.43099
TMEM83	59.86593	58.69056	57.48146	53.68051	52.27609	39.29568	44.00378	61.3364	113.8957
HAUS4	2058.012	430.3118	1169.466	491.5258	1116.908	540.786	1216.984	964.9605	239.5176
C18orf20	97.95298	74.91505	53.7714	58.84155	47.87223	37.47169	44.86438	48.09058	183.0682
MTMR4	732.586	428.0806	260.9831	418.2593	453.1989	290.9087	316.7224	325.112	301.8241
GARS	1096.453	1889.532	1353.071	1183.276	1524.132	887.7316	956.2454	1267.049	1247.121
GTSE1	121.0623	48.53797	1139.928	478.9675	1399.666	662.4954	1004.075	1542.53	44.79925
XAB2	4700.177	5839.948	4863.278	5878.436	4545.297	5758.979	5193.405	5639.227	5745.267
VAX1	175.5771	50.32209	41.6036	163.4807	60.01044	63.71225	52.37362	87.55376	163.864
SNRPD2	381.1081	397.0816	111.974	244.8111	163.2894	163.9267	98.71182	130.6981	425.8109
ZNF414	62.52365	760.171	57.53141	86.1581	182.7586	42.36678	113.7224	89.24501	4188.52
LOC73154(	65.48062	50.94805	35.61553	63.72552	54.02663	59.51958	46.5659	57.34894	134.1877
SLCO1B1	305.0305	149.7644	84.06145	201.8562	78.07598	131.6255	72.65835	84.84795	362.0708
TMEM45B	9051.271	7485.332	7111.263	7368.901	9351.561	3505.402	3222.047	7478.831	12662.7
ZKSCAN2	773.869	617.1645	403.0265	637.0256	598.085	483.1185	444.1703	663.1063	1168.767
FBXO31	6517.774	5068.181	7486.187	6375.749	8114.64	6699.737	7517.57	8802.14	5933.72
MRPL19	547.7737	343.8982	356.1464	432.4569	444.5887	495.797	470.176	331.0873	688.0068
KRTAP10-1	311.1689	210.4331	90.41777	37.64344	67.59436	134.4084	55.05816	63.64132	119.0436
EME2	168.0915	106.6401	814.7578	140.5434	61.5182	98.48965	301.5894	86.2024	239.4715
OR2T8	70.02228	68.70321	49.8802	56.14029	148.3908	46.15548	45.08454	49.59802	124.4754
PADI2	305.3744	143.5005	82.3725	202.7657	89.91579	132.9696	82.36558	129.4576	350.1776
SKA1	210.2841	143.0518	114.8789	140.9731	89.83018	79.9407	80.19167	125.0548	274.0316
CHMP4B	134.0481	58.6663	499.1616	88.33311	741.3737	900.8147	958.8439	376.4048	46.61636
B3GNT4	68.64646	61.78151	65.75565	68.76279	202.2021	51.78294	99.9566	56.67237	164.28
SYNC1	2896.173	4139.355	2434.798	4208.678	1227.029	3481.68	2604.453	3801.285	2700.736
ELL3	186.1787	61.10366	125.7861	78.93084	254.6417	67.84056	239.1338	43.44715	63.09357
BICD2	1842.778	40.85564	283.7803	1374.508	53.20069	519.518	118.5651	404.2803	2732.107
BAAT	33.57452	35.13099	55.58587	56.85307	52.70307	39.60638	45.65462	42.47863	37.89936
CD47	36.72816	38.93871	306.7931	386.5167	740.4317	820.6075	1111.703	427.078	38.31654
EML5	153.6315	103.0812	68.02621	131.5431	55.00663	60.83852	57.4387	135.1098	79.96155
ACN9	657.6287	388.4084	173.8265	430.6909	124.1646	189.7642	147.5781	144.516	67.40916
PNPLA5	163.1707	79.55444	80.70091	107.458	78.66705	119.1364	64.51311	56.51443	154.3774
PEA15	18352.98	16256.06	20298.25	21500	19785.28	20719.75	22178.8	17793.86	16374.21
GBP4	476.8013	256.2913	126.1031	275.5017	69.02421	179.4677	73.74117	145.7668	174.1995
ALG6	50.52609	37.48486	245.2032	46.35758	308.8852	375.7048	220.1784	337.5377	44.88168
ZNF833	114.2406	111.2484	78.39222	122.0205	79.98618	89.33843	64.29326	71.18462	79.69763
TMIGD1	119.5964	112.8972	56.62116	130.3233	134.2642	57.85857	51.44292	55.59991	51.10575
KIAA1324	40.54221	47.55365	388.1101	54.61819	345.6955	903.0864	532.6631	1003.887	53.55613
OR12D2	156.9092	155.9368	54.71999	140.8658	68.88432	124.6104	61.56131	97.56867	266.7756
C5orf56	809.5204	2041.096	705.7484	609.6747	304.417	422.3263	293.2919	590.5447	1347.535

SLC36A3	48.07866	37.40909	34.39724	34.95581	34.89679	39.25958	41.10244	33.1319	35.65044
PSCA	142.4754	95.28307	219.1301	99.55558	66.4223	766.7833	330.1223	75.47095	139.2447
FMO9P	41.2425	54.66051	1303.771	39.55203	121.8527	1380.448	189.2904	420.6255	40.1146
RRM1	5242.583	4294.877	5925.57	5744.407	8183.741	5184.926	6033.061	9473.561	3732.581
PLOD2	3990.285	741.9004	3789.589	8682.425	2724.649	2869.655	2962.65	1839.356	2458.766
UFSP1	77.29995	38.47121	43.17611	84.20023	278.3524	64.1501	330.6297	152.1328	69.26961
DRD1IP	2434.846	3251.346	1003.021	38.67268	725.7274	1393.424	481.0385	892.5305	3689.93
ZMIZ1	3122.342	3821.527	5753.043	2721.253	7448.233	6810.46	5872.358	6027.007	2121.043
C14orf124	53.89396	32.55529	50.57786	39.99979	34.08994	50.4345	33.75068	34.86625	76.71822
MON1B	106.7218	113.7472	192.3475	117.4936	284.655	73.37817	133.1283	64.1293	2848.667
BAIAP2L2	8261.164	13090.42	10532.83	15665.12	11416.08	11475.15	11541.71	12682	13160.1
CLEC4E	122.5636	129.6376	72.64903	94.48273	78.33414	94.60572	63.30155	74.85958	401.3711
RAB7B	56.94527	1656.818	3878.506	1363.046	4606.953	5424.663	3266.879	4099.322	1770.858
EXD2	37.53671	899.5666	1120.333	534.0768	1066.118	676.2581	1015.655	862.2569	1027.514
RCBTB2	53.64039	47.89223	41.7468	48.11747	33.8359	160.331	41.15524	38.79145	59.11362
VRK3	53.12069	2979.182	3640.583	669.1854	1916.34	2743.414	1908.569	2931.245	5305.561
S100B	56.54968	70.34748	62.14499	51.84868	37.9963	46.09409	43.70749	39.12526	60.25229
AS3MT	277.6257	325.2595	148.4981	203.0111	124.8568	75.97613	306.0965	115.5626	312.3928
ZNF708	789.9308	365.558	296.6562	393.7528	323.606	584.2781	240.3907	207.435	39.62282
PRKCA	643.9528	313.1486	356.3482	106.877	433.912	629.778	502.3563	567.686	158.8236
C20orf43	6869.083	7629.953	11988.58	7569.858	12543.13	10997.04	13985.26	10830.74	7727.703
DNAJC30	1147.056	977.1282	222.4976	1040.929	751.5206	362.4463	406.9594	362.1247	2000.031
NUP54	60.64354	52.16843	34.42965	39.45985	52.51341	44.83885	43.66359	37.56119	33.87381
FOSL1	792.4594	2414.3	1701.863	3165.174	654.2659	1457.345	777.1096	1527.124	2331.809
ZNF575	37.38343	68.08644	114.0207	59.91235	83.11547	198.6473	84.21505	43.16389	137.6806
TXNDC11	170.2004	69.65982	89.52332	114.3016	219.1213	68.38821	85.83829	101.7213	126.6173
FAM98C	2015.815	1182.395	1084.37	789.2719	777.7653	1700.634	635.7995	1439.62	3311.505
ZNF45	140.2751	540.4158	194.8404	113.2483	133.7691	324.3399	247.1772	50.99701	1351.377
NUP37	1378.814	50.73441	3248.128	2792.315	3974.081	3081.84	4556.461	3363.682	57.52212
HSCB	56.79937	51.06678	102.3913	47.30383	71.42622	50.00925	49.9315	94.29155	75.30939
MAN1B1	60.21815	372.9622	959.242	650.275	382.3558	895.3865	923.8593	1421.561	109.9959
GLI3	238.2917	143.6436	196.1358	169.4279	100.844	136.8674	217.5518	101.2312	254.1244
GSTA4	42.387	340.346	503.7341	55.15794	809.5712	344.1823	708.7404	279.8253	851.7223
SLC44A2	16634.6	14725.53	13391.73	16290.25	11593.19	11855.18	11837.19	11959.8	16497.54
BRAP	177.788	41.7304	66.83179	46.69	63.39443	79.49229	47.84941	42.09825	52.75251
STEAP4	115.8572	36.09823	129.0423	349.1979	196.2421	247.1779	230.782	175.7747	52.11435
LOC54103	470.1358	134.0224	176.8997	330.1777	128.6175	398.9664	142.4469	135.6338	48.13211
OR2F2	60.89686	44.08052	44.144	57.06413	48.90039	87.34138	43.4389	45.14813	101.5945
C19orf51	50.15946	37.55722	746.0939	960.803	501.289	1181.511	818.5933	1232.803	51.45147
TAS2R49	48.74627	43.1778	54.18562	54.46467	235.9661	50.44185	97.56633	151.0374	1849.235
U2AF1	34.4944	41.17572	465.0195	722.5645	281.2385	35.79261	495.5355	56.59565	42.06075
ATP5B	9852.964	12778.94	14481.25	12433.76	14073.83	13811.3	12231.69	14838.01	9981.676
SULT1C4	87.02567	91.16515	43.23505	58.03445	102.6506	46.81211	40.55103	65.10571	194.9953
ZNF536	304.557	210.9361	104.6467	236.625	104.2151	141.1578	98.51328	156.3895	454.6168
ERP44	38.50356	54.60176	43.12914	51.18478	45.10806	39.01715	72.64545	37.02805	32.02841
PRKY	703.2504	116.6363	62.06435	61.97701	73.73931	85.93903	53.47305	77.21764	62.36067
OR2G2	41.77365	38.07101	37.76256	46.61539	36.20533	56.48215	33.46038	56.99783	49.41787

ABCD1	38.02867	34.19986	33.79073	34.21471	39.54839	43.92509	110.0564	35.41943	37.5826
CYP3A4	77.91758	99.16196	42.10738	110.9945	57.26062	58.24196	45.90215	49.40256	71.73502
CCDC88B	94.25378	351.6647	51.75702	34.43841	108.0095	64.36131	69.79991	218.2439	46.90132
CRYBA1	43.76717	37.64021	34.32279	45.84341	45.95963	47.63611	34.94378	39.51939	47.28189
HLA-DPA1	5253.239	3778.758	4907.392	3539.094	1056.622	1790.207	2112.924	3055.127	3615.426
KCNJ5	51.78009	60.57619	46.52986	84.4682	220.2126	46.00121	206.4561	268.5037	56.69167
CENTD2	469.049	352.3647	169.7465	249.3372	195.4085	164.41	154.4881	132.6758	607.4193
SQLE	89.50819	114.5852	442.6873	81.27603	454.8048	368.7942	120.6619	321.4473	129.6507
TRPC5	301.434	173.3808	97.91071	171.1617	90.95944	129.7657	81.34249	122.5445	286.8798
GLG1	10713.74	13705.09	15660.34	14563.89	16668.59	15106.18	18886.55	17892.94	11904.18
TANC1	63.97902	52.40607	49.17273	70.80283	35.89903	62.34851	89.77927	127.4369	54.62062
CHI3L1	63.51362	54.3531	47.65408	53.22983	39.36053	54.04638	38.44506	166.7274	47.97493
MTHFS	42.74477	36.21831	36.71245	85.54408	664.6127	51.69229	585.1927	297.5094	51.80792
BARX1	128.4448	159.7944	71.03467	114.8499	71.21155	113.6394	60.61079	94.29828	267.6221
UNC84A	189.0047	126.0333	88.88559	528.9937	525.4966	89.35301	467.9381	238.9252	88.82542
LAPTM5	196.178	76.22318	243.5058	66.18147	436.3788	62.3655	593.3412	563.7823	57.69723
PDHA1	910.3088	959.547	778.0479	1150.191	729.1174	913.8392	698.6881	998.8152	959.4494
ARHGEF1	2839.36	1724.99	687.6627	71.49233	311.4242	418.0552	241.7859	709.0291	127.8768
ITPRIPL1	101.7189	99.08691	67.59837	81.75697	57.393	62.62369	53.60614	46.6852	179.9615
TLR2	31.83654	37.32579	194.5863	41.01405	234.1139	36.66948	265.6693	346.1977	59.11717
FAHD2A	57.94825	511.3143	38.78683	43.55077	36.76059	50.37395	36.66162	42.61618	46.00196
C12orf34	114.4419	119.0617	884.1846	88.59778	1390.997	487.4297	596.2667	880.4244	163.8839
HDAC3	111.097	365.3688	1879.768	1536.369	2516.929	62.71207	2055.094	3144.203	219.4583
SPAG6	73.14877	54.93814	42.00163	61.7216	48.37536	58.5802	46.62984	42.49385	66.97978
RPS23	813.7274	113.5808	1584.053	1680.048	178.3217	1954.845	1428.051	2098.308	371.3795
SH2D3A	185.9402	122.5166	100.782	1184.867	95.31242	127.3188	82.36653	113.1523	93.97903
SDCCAG1	298.7615	89.05302	425.4794	845.7253	143.7535	102.5613	95.20626	591.602	112.0651
TMEM132I	60.30441	44.01125	54.58544	48.09209	50.71046	66.18284	42.76294	43.00517	50.7743
MTHFD1L	296.7204	898.2575	296.8879	151.9899	383.3379	327.3884	509.354	69.23841	218.5314
ZFP161	313.2613	171.5999	1125.89	2617.069	942.2533	1431.53	1390.975	738.0995	324.056
TAAR8	326.7366	151.6495	91.28079	194.74	77.03956	112.288	68.59254	69.99815	409.0987
C12orf44	395.2129	1958.487	2299.511	1461.81	3446.499	1015.724	2831.956	3480.082	1699.189
SCTR	369.9039	201.7124	884.1463	206.9899	166.5743	149.0766	198.1191	117.5164	401.7353
ATMIN	612.7231	705.1614	1799.716	2443.314	1036.699	324.3421	455.0164	714.8273	3277.215
C21orf69	75.23335	1766.118	635.3077	692.0136	275.5315	1609.825	1116.984	1466.408	96.78043
POTEB	49.30721	76.22098	38.71248	51.31758	43.60383	43.88374	48.78711	52.41775	104.6053
C6orf97	83.17259	93.19932	46.20374	43.30252	40.45788	39.86063	48.40983	74.31705	50.43017
FRS3	63.13196	1417.867	535.2936	51.49061	440.4278	763.2307	746.5699	471.6346	201.355
ANGPTL2	4255.397	4081.496	6781.473	766.2962	6503.706	5207.51	6368.749	6112.884	4613.673
D4S234E	99.39888	1199.366	450.32	117.3641	1594.362	588.7712	863.7524	1555.402	177.4261
EPX	233.7173	77.13384	76.22052	104.6128	81.15509	115.0691	55.98819	106.1832	303.9563
EML3	28135.16	24265.92	23011.11	22796.81	20163.79	21160.71	19258.8	20268.48	27534.53
AGK	58.12186	90.59848	537.1587	76.88362	666.8953	1055.96	625.2859	463.7271	45.60027
CNOT2	711.0207	393.3622	151.8231	504.1749	342.0163	256.4267	367.6506	87.12744	429.2115
ANKRD53	146.7008	120.8358	72.70777	83.9755	63.86404	60.69669	60.47103	63.88857	168.229
P2RY1	3517.694	6042.267	3916.999	1212.052	6938.596	3536.342	2105.655	6534.688	3832.732
IL21	118.15	130.991	70.39618	127.1179	63.70951	69.52093	56.73562	56.80884	191.4177

FGD3	13235.83	15704.26	15101.2	10869.6	9697.833	11408.31	11751.27	13134.15	13941.25
TMEM18	114.691	116.2526	142.6827	99.01231	212.8882	82.96318	99.04905	81.22445	427.913
SFRS2B	374.402	209.1989	112.952	252.0955	290.5553	160.9633	194.9435	159.823	487.2785
OTUD6B	52.63115	46.12284	55.97884	67.73605	66.85647	79.65889	41.58294	60.59038	771.9996
C10orf32	572.0336	356.5106	193.0939	354.0577	192.1807	275.3554	173.119	309.4535	844.4787
BMS1	2945.633	5797.979	8335.645	3995.917	8682.674	5998.189	8650.758	7103.407	2870.629
GPN1	2034.473	975.5839	2370.412	1259.185	3342.489	2236.332	3382.352	3988.172	859.7083
GPR75	41.63391	42.1925	40.38183	47.76276	32.15919	46.0117	34.99591	40.54155	41.06824
HAGH	2218.385	3482.437	3748.309	3172.551	4853.7	3312.088	4773.969	3380.734	1147.276
LOC143188	43.61154	60.20325	61.18227	84.34926	51.90892	44.33249	138.0005	69.12531	1665.746
PIGK	280.693	160.6913	99.33915	269.4686	113.8503	131.7201	76.98721	105.4023	369.0428
TCP11L2	826.3513	377.4913	151.2297	406.342	225.8092	202.3849	99.78157	182.5818	1024.309
KIF16B	72.8548	64.348	56.52805	42.59148	51.72985	64.77715	75.52293	43.93521	47.79323
KIR2DS4	272.2454	130.4588	71.1116	103.5472	71.38338	92.96067	69.92827	63.25188	238.4356
FGF21	35.79486	32.63044	48.74877	33.87024	39.83313	35.4608	34.73368	34.76633	39.2641
C1orf161	182.122	155.408	88.05429	131.9648	67.94662	77.81034	95.58347	64.28469	339.0651
DYNLL2	1581.831	1822.324	1249.998	64.43352	839.0792	51.22435	911.0905	1496.503	111.1022
ARPP19	1105.08	1159.2	587.4293	1336.28	336.6751	983.2823	325.5796	470.9735	1020.197
SLC4A1	48.56859	48.09494	50.71738	53.94418	45.42982	43.32911	36.00774	41.9381	44.98127
NLK	650.8643	299.9722	1061.588	108.3921	1235.349	1180.659	1530.133	957.2974	896.4586
LPIN3	284.2382	198.3104	102.5926	200.9971	109.1803	151.1971	138.228	118.9567	360.3316
SNRNP35	1181.276	1320.618	1190.997	1534.105	886.0497	1379.612	1049.375	1582.553	1709.787
FLJ46257	51.35188	35.71118	46.79189	38.05698	40.5918	47.85818	44.51698	44.19926	32.5874
ZNF395	48.99548	36.11777	1194.26	33.2287	1932.507	51.01553	1014.135	243.2948	57.54217
OR2T1	492.2884	273.5576	141.1036	318.3838	177.4251	224.0037	146.8764	196.1952	739.8051
TMEM27	89.95956	78.526	417.1674	53.48305	50.47688	53.34888	325.7331	49.98584	41.08667
GAS1	184.381	142.9939	54.04419	56.59986	59.93706	201.287	182.6216	74.2699	41.80219
UBE2NL	50.13881	38.63018	61.10172	32.56479	42.55792	42.73535	39.1873	40.2206	40.9561
CXorf23	129.7381	90.03746	74.21912	95.21439	62.8521	81.57874	65.97703	63.72815	210.7072
STRN3	104.0906	125.1442	77.52364	139.6352	101.0408	86.18696	102.3193	135.8856	147.9646
ST6GALNAC	41.2781	33.6423	43.15001	52.13116	46.85123	41.2606	36.57458	48.11237	49.01006
FOXRED2	49.27171	978.2098	269.9781	54.63385	318.6304	37.0299	401.7451	411.1791	1160.932
PDCD7	1151.133	2660.832	1684.068	1769.626	1393.823	1480.803	819.8085	1088.186	3279.237
CCL8	239.8938	346.3758	2139.725	392.8029	1104.092	1652.226	1918.48	975.2291	264.5304
BTNL9	47.12553	38.73688	301.7396	50.43135	230.2635	46.37186	244.3769	109.4566	44.736
EPN1	24880.54	23716.29	22520.55	26414.01	24426.41	25813.4	26413.89	27360.17	25677.77
CHPF2	54.2103	67.62988	51.48561	91.50208	63.64351	79.41972	67.06129	65.41892	82.80079
LOC730417	41.5919	51.21061	518.3268	438.2144	843.4315	643.8478	561.8406	212.5803	1066.302
FABP4	610.6531	320.6917	110.849	228.8004	126.0376	208.2034	118.3005	354.1462	705.2776
C14orf73	1629.039	2780.587	533.3958	690.313	454.7458	505.148	252.5735	2165.086	2659.773
CUEDC2	470.1553	76.02297	83.47806	117.8615	114.1895	185.6724	141.4661	126.8471	190.3558
SGEF	68.87599	53.18962	38.70462	38.32903	291.6046	39.7253	37.50882	41.22598	43.15318
EFHA2	177.2982	75.55846	57.09694	120.0095	59.46165	69.81192	43.62716	59.53505	62.33359
GPR124	47.86281	1046.24	1705.689	2449.765	1200.318	1843.829	1481.259	1864.518	38.02294
CCDC63	140.1571	100.7817	81.51697	84.81486	57.82448	66.87132	63.67899	65.07301	179.462
CDKN2AIP	901.3533	411.7412	822.2726	1163.774	686.4609	1258.706	504.3635	1279.364	63.98228
PHYH	8377.541	9389.496	3851.029	5693.717	2448.355	4963.996	3282.24	4267.982	8438.366

COL3A1	6311.631	3889.334	2897.454	3458.209	1346.707	2631.792	1985.307	2192.805	4376.326
OAZ1	23236.21	20169.92	21245.74	21966.38	20638.26	19813.92	18701.18	22426.85	22902.88
CYP21A2	118.2312	113.1976	70.30959	156.8307	72.0694	84.41094	87.28699	62.74852	74.30434
LOC28375!	51.18731	61.73655	63.07159	34.16929	38.6677	35.89135	40.35371	35.21582	66.20851
BAI2	60.72931	47.44476	39.32062	534.014	38.12959	38.14791	34.10452	43.84988	35.56041
GOLGA8E	33.89935	34.47008	55.814	32.41353	34.72863	39.54098	64.85732	50.43994	30.15351
CNTF	107.6012	60.65316	42.84124	42.47515	62.0996	50.19622	51.6527	48.37852	73.88773
CCNF	1470.467	3592.851	1951.061	4981.595	3602.971	2477.352	3775.455	2296.728	2228.454
ENTPD1	149.6063	137.4417	949.744	205.1602	1240.475	747.996	482.058	239.1838	196.0293
FANK1	286.1986	226.8855	134.6601	265.6775	88.72485	177.2614	108.7083	113.5267	206.517
C11orf61	728.5048	66.12997	272.5973	477.7208	77.78304	309.8244	121.2391	239.8646	44.63449
NFKBIA	13075.12	15514.3	17178.76	15883.65	16282.49	14952.7	13400.65	17078.62	14034.19
MRGPRX1	50.03553	52.69808	34.49058	35.66888	36.26862	41.00483	42.64821	38.97396	48.84532
C19orf34	56.77222	35.80006	33.08512	49.84529	44.74029	47.00041	38.85856	35.83844	39.20041
CP110	777.6271	405.3872	2145.2	1007.988	2952.047	2887.766	2077.176	1785.58	123.2246
CYSLTR2	365.6368	199.0206	104.3234	236.8316	111.5753	147.0645	89.63515	111.6311	435.52
DRD1	156.495	90.8008	49.62343	98.80856	56.04124	92.26354	54.86637	61.85632	111.6819
DLST	1219.156	674.923	1153.855	1072.168	2453.638	1490.567	2019.863	1063.044	42.23707
ACER1	219.0106	130.4979	98.28287	159.3214	90.25902	147.6256	75.70211	132.5045	326.63
ZNF410	605.0818	452.0005	1113.235	2124.791	440.014	812.0928	713.1983	725.9368	91.49271
ESM1	451.2591	305.5714	221.0887	1776.236	296.3843	1139.677	877.4022	461.6953	808.3297
SCN7A	295.4749	168.9495	98.4941	219.9481	88.73282	139.6312	76.28532	92.34876	401.725
OR8D4	52.12184	45.01281	43.42295	41.48946	39.21469	44.52262	37.56005	47.01425	63.81962
NAP1L5	50.46102	59.53463	161.7802	61.65722	100.5846	44.39319	77.70418	326.0224	161.1149
FUT5	138.2383	171.5111	91.03842	181.5885	71.29795	106.951	68.353	76.01945	269.9308
USMG5	69.83707	78.05871	52.90556	83.65553	62.92725	52.92736	44.68475	42.42104	106.1313
TMCO2	46.16423	43.79704	34.7995	55.22688	147.7332	51.63766	202.0912	49.59116	49.126
DKK1	70.8131	55.47218	84.04531	542.6915	82.363	217.5067	287.4199	49.57311	769.5297
CCL5	2384.204	2155.974	1908.652	2529.375	338.466	1287.658	1448.887	860.8835	2409.635
CIITA	2222.651	1758.273	3081.402	776.7094	605.0054	1476.005	861.0764	1263.998	1689.142
MRPL48	46.098	63.2203	39.61993	48.91866	869.6647	47.60881	1123.498	543.1271	2327.474
UNC13D	1066.329	1222.794	522.1481	1186.651	87.38563	131.0411	169.3899	211.0504	1613.56
C13orf18	154.4369	76.49409	127.7614	170.8289	117.2538	76.51862	90.91663	51.52413	197.6748
RDH5	3172.207	3768.332	3053.906	1968.518	2927.546	3598.016	2457.99	4287.671	4807.799
C16orf72	105.6092	140.531	1159.907	658.7312	2762.254	1073.328	2355.14	2914.77	143.137
DOCK9	47.27731	45.40498	375.6463	311.8868	39.77533	48.4086	140.4358	323.3953	79.45297
SOX11	66.58607	1408.102	492.0011	2203.054	448.5362	1258.562	1244.194	527.4365	159.6058
UCMA	178.9127	107.6854	55.7311	113.1228	51.61423	69.9984	54.31427	70.36082	401.2233
USP4	53.91175	1765.188	582.136	269.3117	200.8871	57.80534	225.6607	37.53047	45.27552
MRGPRX3	367.143	201.5036	214.1489	245.2598	181.2914	147.567	97.36359	105.756	497.7725
RAP2C	1006.756	1624.773	1072.445	1715.311	676.0682	1101.882	807.9834	918.5518	1296.909
LAMA5	12017.34	12430.96	13134.92	16662.01	15569.33	18125.11	16660.53	13554.73	11227.54
CPNE3	744.5162	309.7138	529.1602	439.7541	667.2584	329.3561	564.9314	470.5091	57.17516
SLC17A2	96.98625	103.5714	79.83958	101.6417	65.82944	89.40554	119.7341	40.61427	171.5497
POU2F3	220.0559	73.10536	57.60821	97.75822	48.93732	97.22205	56.70461	69.42063	235.8165
SMARCC2	2535.086	3084.069	3524.174	2150.742	5863.011	4297.645	5285.449	2772.346	4058.114
HSD17B1	50.42026	83.5316	412.6531	43.56356	692.0042	45.90074	382.621	459.7798	46.60598

SLC38A9	3501.551	2348.529	3202.563	3173.787	2726.035	5100.111	2913.472	2469.584	40.24073
LRRN3	110.4176	50.08931	310.357	42.41234	50.22087	64.50253	79.20523	121.4732	88.05429
CLCA3	76.90877	36.55917	114.1977	36.20541	35.78115	170.6168	75.90011	90.60936	35.02149
MRPL9	62.33255	90.38408	949.0712	1484.089	1253.476	1081.305	512.0117	1123.539	79.9528
FLJ44635	161.206	101.9386	86.23694	100.8695	68.82767	70.76299	58.58815	97.69111	285.2122
GOLGA5	58.91265	85.11714	81.08376	46.34461	546.1862	92.51785	535.1191	649.9171	1540.968
DNAJB9	1293.089	1608.842	1632.541	2282.502	3473.629	2265.733	2094.587	1500.837	170.679
PAICS	34.46922	388.8871	608.5641	47.67537	1468.707	447.3961	837.7438	565.8913	47.43485
MTA2	2910.293	2521.023	2938.053	3592.665	3105.683	5132.051	3344.654	2930.193	5800.662
FAM173A	111.5127	711.9998	630.187	756.0735	1147.827	779.6677	808.6516	998.5809	158.5068
TGFB1I1	65.05225	37.66239	44.22578	50.02462	162.1453	48.80782	37.31594	38.27988	49.07386
FOXP4	68.91515	134.8025	45.33413	116.6302	259.7625	81.70396	66.59361	475.7008	298.9099
MUSTN1	7798.945	9764.494	6959.326	7265.228	7526.844	11394.39	16268.18	17455.31	7865.045
ZNF585B	96.96436	151.1573	62.19008	128.0455	82.09237	101.5292	66.43327	85.83465	112.9384
DYNC1I2	49.5349	1800.054	610.618	1215.461	798.5843	246.8866	667.8363	689.8563	2498.016
C15orf32	60.46141	40.3876	43.27346	42.45977	34.56487	51.14558	42.03135	31.53431	88.2843
ATP6V0C	3623.402	2851.637	1590.184	1416.53	1015.337	1534.073	815.3173	1139.198	3621.062
FXC1	3284.769	3940.224	2527.674	3778.902	1635.639	1911.466	1448.835	3007.336	5031.163
CPT1C	40.50418	61.56475	1596.63	1210.217	2324.936	2156.827	2472.194	3293.329	67.01096
HSPA5	10393.68	9773.849	10290.5	12274.61	12352.54	11524.1	9310.348	11276.12	9911.674
U2AF1L2	7788.223	8288.063	6907.927	5476.383	5551.43	6639.504	5616.377	4234.842	7846.278
HNRNPCL1	50.52811	45.05015	37.0677	33.73036	39.7472	41.29646	36.58919	49.37436	48.55563
DMAP1	64.25961	287.6558	168.9901	47.46831	280.7423	200.1602	393.0934	281.9504	63.26458
MED21	1949.664	1851.316	1608.549	1989.873	1387.625	2121.278	1819.791	1290.434	1087.175
STX12	922.1582	1027.292	1179.583	727.1507	1515.814	1295.879	1248.583	1576.872	489.396
NCOA4	8197.354	8243.534	10079.99	8397.401	11957.52	11122.86	9987.145	10631.69	7757.284
POU6F2	53.49308	71.48145	41.38713	47.20712	41.77741	41.66533	41.02425	59.98204	56.63706
C12orf61	35.54681	40.24068	46.80567	46.80409	33.2744	36.97191	34.22488	39.24086	42.43554
TMEM165	1047.023	99.11098	466.6545	992.9069	551.3589	92.0678	686.2574	51.0008	261.1886
ST6GALNA(	38.54051	38.27226	283.6546	40.88723	35.97438	42.85168	90.46162	36.63985	62.37942
GNA15	36.82044	1010.253	4326.536	1884.077	6999.843	2933.726	5973.098	4641.267	82.74821
H1FX	194.578	113.0525	742.2094	86.01379	1600.51	76.99144	775.8082	949.9473	154.0575
SERBP1	886.7484	888.2982	782.8464	1152.566	665.2703	1493.214	766.1348	811.1541	1218.789
ZIC1	76.65441	62.0833	55.88909	86.13834	42.07476	58.87963	53.98028	73.81166	177.5161
MTFR1	37.1931	473.346	403.377	726.6494	281.4404	227.2147	420.7957	460.1777	41.4784
SLC25A5	1121.897	277.8325	2002.973	663.4214	3504.094	1544.42	2711.305	2249.103	1553.823
TMEM141	10929.28	10746.86	12265.05	10563.9	9444.057	11449.46	9594.382	12688.04	12287.5
CD177	121.9494	95.69919	89.58006	79.05792	127.1826	71.24948	54.56179	77.70231	64.54499
RXRB	10695.66	9798.555	8305.396	8855.868	6597.467	8245.323	6552.59	7777.25	9713.364
GBP2	13732.83	14475.12	14502.28	9468.318	7275.715	13804.29	10258.96	12635.48	11553.7
TRIAP1	729.1859	520.2095	695.1324	712.4559	643.0905	666.3542	728.6545	829.6284	785.9916
CBX3	72.88369	85.89442	42.24491	58.46976	52.61993	61.84499	49.11918	46.05466	60.89704
FECH	5509.282	4792.675	4435.796	2733.201	3806.825	5026.995	4943.946	3328.498	2095.021
CHN2	1243.558	67.19798	219.0342	39.74784	36.3513	410.7225	55.16186	57.07911	44.87962
STAC	121.0135	90.41134	84.31205	117.6286	330.9246	107.5914	188.8212	151.7486	337.8576
SPHK1	36.49772	53.46261	187.1345	46.64782	35.58352	48.96674	35.00394	208.7256	76.49023
ARL15	620.299	343.7841	255.3224	446.3951	264.4094	359.8922	265.3951	435.9775	750.8211

URM1	387.5885	1723.911	3548.447	897.5382	3018.358	2735.189	3995.917	5057.41	1810.656
ANGPT2	73.28345	65.36993	180.68	392.1758	170.9866	735.3229	734.8682	323.1539	65.89558
B3GAT2	51.96835	47.81654	43.70147	40.7629	56.07977	55.42291	35.87507	36.71009	67.52212
FLJ45032	631.131	39.71291	524.9796	41.87361	125.0361	49.37724	107.06	44.99742	59.54334
C2orf51	36.88686	45.98381	44.16869	47.86194	43.99575	40.88835	39.25843	40.51777	51.6263
SLC7A4	76.78432	74.39616	277.5432	67.87064	56.64569	64.00426	261.1752	54.82921	79.91074
R3HDML	125.2536	38.87072	34.95594	38.60189	44.99702	44.01859	46.88768	60.79042	53.38103
PLUNC	114.2843	56.23886	56.33992	93.71831	42.82674	65.92851	49.17859	48.3585	50.20938
PRSS21	54.20771	56.01609	50.2154	32.84194	47.62133	61.78057	42.35096	51.07842	156.7881
FAM63B	197.9077	612.9532	254.2514	172.8802	191.1438	146.0855	347.35	173.1429	378.4004
DOK4	159.3082	119.5349	62.98848	144.4411	60.95205	119.4688	57.78186	91.82461	48.64917
C2orf82	50.15913	35.05458	106.8072	55.42975	264.0004	46.50123	170.7176	127.6469	70.63861
ESRRA	52.91981	500.5405	561.6425	80.6433	524.9379	76.15498	203.8357	303.1858	43.13555
CFB	42.27468	459.1587	396.6235	301.8241	156.5158	547.817	639.8669	495.8548	45.68437
WSCD1	247.0555	173.5928	74.98539	174.6671	70.18252	112.2366	60.86612	79.7739	168.9339
HIGD2A	3996.857	4269.106	5767.415	4760.327	4870.358	5857.49	4268.873	5281.86	3420.326
FLJ45831	52.01107	67.60966	37.08715	233.6667	40.32338	39.65031	59.50604	47.3717	55.66851
POLE2	61.62294	56.97401	54.83609	434.7971	56.28974	51.2187	48.63989	46.5265	70.67389
MYOZ3	2218.337	4201.563	1740.295	499.2042	2398.495	2987.691	5667.019	9912.741	97.41663
LRDD	330.1296	248.6093	115.5653	196.5336	126.3932	166.6035	119.2724	161.9405	448.9991
LOC20042(	69.6177	111.4135	78.85252	70.19613	53.49285	59.80074	84.50176	83.48317	108.7738
NIPAL2	1424.089	98.62396	1197.575	1938.955	1560.799	1403.074	1135.669	351.3976	2271.601
ZHX3	1874.818	1946.753	1177.834	2324.936	1368.315	1776.613	1592.505	1002.808	2731.643
LRRC36	86.41425	47.61186	79.82381	51.93772	52.74455	99.52303	76.73544	49.61576	61.5776
HSPD1	66.36391	60.31924	51.58738	73.92224	46.005	46.48016	49.32166	68.40379	157.0046
CALCOCO1	75.46423	790.7985	754.0098	885.0354	818.0045	612.3441	644.4708	711.7067	1111.142
DTD1	153.9032	73.56384	571.0281	101.8856	578.7084	70.35487	465.8608	356.5828	251.3428
DCST1	130.2474	152.3972	608.6478	138.0375	430.1886	90.89432	465.5743	356.8331	428.9255
KLC2	54.40774	48.52086	39.28857	564.8053	38.57944	38.894	66.85556	36.53764	63.16391
KAAG1	57.06352	96.09002	66.09716	233.5116	60.22412	54.22886	50.71756	57.13072	144.7468
C2orf28	55.61997	673.0154	277.774	70.7677	2390.802	1050.259	1053.387	1171.248	1986.21
IQCC	3546.969	3413.542	2013.362	3218.865	1456.497	2023.371	1678.459	2560.967	4746.906
BSCL2	3519.6	4019.197	3047.782	2994.011	2901.805	3638.231	2829.426	3027.874	3286.506
NAB1	80.54697	279.7803	347.2471	312.4819	1164.777	587.7585	704.7648	346.1895	104.6772
SNRPB	23272.73	25829.27	29501.92	28356.93	29854.3	30632.07	29726.15	30519.91	26083.17
TUSC3	36.22069	39.01168	131.8263	37.09164	175.656	551.7899	185.6753	175.8602	33.25832
TCEB3B	38.6278	42.03305	303.7093	41.48039	40.01146	35.83832	43.79787	53.36562	178.2969
ORMDL2	601.8542	399.0718	194.9319	444.5372	166.1202	268.0387	234.4562	191.1523	710.4546
OR5P3	53.18756	38.62423	54.84391	57.67219	59.67999	46.2313	43.88947	54.11977	112.1456
CGREF1	91.86801	152.0351	70.7821	122.5762	63.76941	108.0384	60.51299	75.41936	181.9084
SOCS1	56.38662	44.76852	1418.976	461.6095	559.6027	386.983	1137.558	507.3354	146.3018
DDB1	555.1763	200.3323	542.8022	563.0702	516.9612	549.8051	649.8359	495.389	746.67
MMP14	99.55752	129.8483	73.86786	112.1914	54.95583	77.01373	65.36839	69.68169	95.76883
MTTP	898.0549	592.5239	160.8018	429.5214	60.08792	259.9539	224.4811	497.6313	633.436
SSTR3	111.8582	126.8368	66.49212	80.49231	67.93175	80.48447	57.87497	59.16795	131.3163
ZNF496	155.7888	133.4038	121.7842	327.2894	56.77554	213.1954	155.0263	212.345	229.3357
CEACAM2(	37.32668	48.21076	41.94289	46.15974	34.43571	34.70753	35.87063	42.47058	48.4724

KTELC1	34.0901	34.78645	379.4428	37.87593	650.5536	190.0988	642.2585	175.2433	36.1145
SLC9A7	108.6022	127.8478	91.97801	131.3663	85.95255	148.0832	80.55514	220.0121	529.5635
FXN	32.82765	30.3757	221.0822	31.702	34.20996	30.90841	38.73747	34.78117	37.64058
VEZT	5645.444	5357.265	8103.167	8646.409	7651.374	6979.383	5787.666	7424.383	6591.892
PLOD3	41.73103	3271.528	4980.702	4934.949	4603.108	4374.576	5837.635	5714.935	32.12176
RBM19	139.0488	1319.06	263.2891	537.5251	74.41193	555.662	276.4349	256.2749	233
UTP23	623.8604	390.9063	216.5694	328.1176	193.4282	241.1918	177.15	231.5143	684.2994
SLC30A8	63.97274	70.58896	58.33861	83.84964	44.58248	359.0437	47.60911	69.43352	85.51097
C8orf49	40.46819	45.12276	39.56021	37.57429	35.81689	44.05867	36.67659	38.29677	50.40913
LAS1L	3709.896	3936.67	4489.329	4067.499	5413.714	4955.568	5904.496	4754.548	3179.458
NRG3	59.5477	62.86426	42.04142	61.18706	55.94293	73.86697	48.05363	106.1673	170.4377
CPEB2	663.0437	358.0704	177.1634	286.8801	155.0439	224.9952	174.349	271.954	745.8915
SNORD21	1694.538	2432.55	453.0997	1933.521	298.9217	649.9536	446.6184	261.5042	263.3239
CDC42EP5	9795.032	4856.605	2588.337	8654.564	1765.379	4306.661	2526.686	1804.637	9567.149
IL17A	49.3818	72.25747	42.10679	122.4731	44.64154	83.89713	60.56411	96.16469	150.4921
C16orf13	832.1649	785.4188	1566.054	195.7759	1805.066	1308.204	1690.601	1201.853	386.1061
KCNQ3	158.1947	114.2829	62.42237	83.48981	128.755	59.79811	52.86497	51.49438	86.67619
NCAPD2	1820.165	3928.127	4027.14	5704.268	3205.612	3525.179	3518.41	3447.677	5348.673
NOL10	155.8846	264.3951	157.8207	293.2041	98.99683	96.84583	88.7681	104.0855	327.3997
SEC22A	5548.313	7951.382	8228.268	6947.778	8528.02	7979.792	7782.167	8852.184	8276.382
WDR69	40.047	32.27671	40.57883	40.24761	45.5798	36.97169	44.26757	45.84073	44.71448
ADIPOQ	311.9662	211.3652	80.50945	171.7475	59.41497	104.9587	58.68311	83.78071	482.3306
DIRAS1	189.1487	100.9348	94.63709	137.6202	77.12956	135.6746	61.82738	63.3489	229.7623
CSNK1A1	186.6041	187.8955	109.2804	155.8204	101.3402	146.8997	81.13211	105.624	461.7599
LOC165186	369.6178	194.1591	98.48347	187.1141	81.33284	126.4619	87.94564	127.6119	201.7236
ZNF273	40.92723	50.36727	42.29786	32.33022	83.29054	41.10535	70.49273	38.5249	40.23487
TUBGCP2	478.4809	405.4702	724.7579	342.0906	734.4874	428.2144	732.9879	516.7317	548.5272
ZNF232	74.50854	50.38379	39.96869	41.28501	258.0383	62.80832	138.7415	314.1575	56.00249
SHOX	224.3016	140.639	61.23215	77.14574	53.94923	58.24761	56.90635	50.06195	99.70033
SND1	321.2941	534.0972	335.0089	2655.063	959.1752	148.9009	513.6465	223.9725	488.9272
ENTPD3	54.03689	504.5576	154.2161	45.70429	129.9453	39.70856	94.15893	161.5621	75.06527
C14orf162	265.6631	121.5232	93.07751	147.2675	86.21792	131.5096	77.8606	112.2791	497.6766
C3orf45	55.74342	577.5847	36.88366	37.51484	190.1863	48.86862	135.274	50.44812	72.17553
ZIM2	65.8047	71.72681	41.1433	53.86578	49.86518	57.82524	56.01088	51.2205	115.2854
PDE1A	37.53217	35.56476	38.33403	36.70705	40.0969	32.74533	40.15499	35.66889	43.57625
MT1X	5626.05	2074.79	2083.364	6004.778	912.7367	2542.5	1221.843	1678.286	3310.83
WIPF2	359.5589	171.4921	103.2671	212.6088	100.6942	165.1639	94.52114	124.5721	439.5421
RPL3L	58.26939	52.72289	136.5404	57.69576	42.36477	76.3431	116.7901	449.9932	80.59808
GSR	8253.5	7268.66	6463.105	4203.334	5115.638	5040.504	3455.467	5919.069	9780.59
CBX8	159.4594	555.9207	282.3201	82.33387	672.1895	117.1984	625.9313	326.2136	1514.942
BCLAF1	54.14911	375.1768	779.4144	313.3738	1605.688	1289.332	1284.385	837.9028	78.73863
ERO1LB	58.93173	91.93917	157.7528	81.48035	89.03963	121.1189	63.80618	127.5116	93.51618
NOS3	1872.428	3183.271	1559.921	1520.415	3177.835	2736.8	2686.377	2671.333	1531.693
FAM9C	95.52123	46.07934	37.59621	33.75814	134.5082	37.72442	33.1054	42.16418	41.31411
PROK2	207.4915	182.6473	106.6976	211.2867	126.4618	104.0237	100.017	84.80159	458.2862
HECW2	577.3087	272.132	473.3788	386.7011	499.6461	308.3193	426.3224	285.8407	639.9733
EID1	1483.245	333.6891	362.982	193.7533	442.0529	529.6164	321.9879	487.2398	61.17241

ATP13A5	58.02117	50.57053	42.18563	34.7625	42.77398	35.72922	39.94893	36.63571	34.22833
NOX5	278.591	121.1331	87.22906	164.7721	88.85558	124.1939	69.66597	93.87307	298.793
DNAJB1	93.53797	81.52355	412.3683	874.0234	597.9101	476.6284	448.0734	495.8586	388.3811
MTG1	5478.26	3760.779	4713.558	5799.381	3107.217	3311.23	2990.568	3815.338	2863.436
C15orf52	18104.61	19017.7	7911.953	24642.41	14103.85	16769.6	17745.35	19119.51	11147.08
SFRS13B	52.65906	54.38161	34.97716	53.73286	39.2389	54.7083	46.02652	48.01823	39.35892
KCNH8	143.6925	135.4489	147.5714	198.1644	82.9408	124.7378	63.96654	96.46932	439.1778
RUNDC2A	93.51009	63.77773	43.68781	67.09634	43.59125	66.78194	98.25596	185.8458	93.88972
LTB4R2	41.38212	48.58652	45.87768	48.64779	225.6794	48.48066	170.6837	37.01094	58.44554
CMTM5	195.9919	104.3841	59.66827	101.5769	62.08415	90.45284	54.16378	67.46162	126.1745
SMOX	3586.352	6356.701	2887.096	5643.101	3299.912	3810.476	2084.697	4287.738	4058.929
SPACA3	231.2043	320.8468	122.4338	194.7313	119.1712	122.345	122.9474	82.69027	186.1939
C10orf88	448.2155	230.8908	463.8065	387.6216	242.9516	510.9087	410.7121	268.5424	460.8241
CSMD2	3150.353	1636.695	1592.621	2029.351	422.1145	2538.041	1287.758	1141.107	313.7595
SSPN	4102.179	6599.936	4798.23	3150.053	6609.024	6826.78	5257.009	5714.669	4184.8
FAM100B	912.9836	53.20069	32.92255	55.25085	478.9525	235.0712	247.6076	368.3013	148.3912
IL1B	2522.657	95.636	1172.104	3292.625	581.3696	392.6571	1489.673	1356.535	5117.31
CYLD	141.4651	94.03111	61.86662	112.6964	54.74778	90.83877	55.25637	59.59687	202.8832
CCDC113	114.206	152.9139	64.26707	63.81791	58.28635	95.51669	64.05481	86.45328	94.65257
TRIM69	326.3968	176.4733	104.0059	243.6395	87.92029	133.6953	76.55441	100.6781	395.6121
FAIM3	615.2053	897.7411	958.6652	1335.43	271.2042	1144.228	611.8299	997.1563	1507.836
PRSS27	19049.42	11809.58	23772.07	11349.52	25634.12	19074.04	17379.09	10981.12	17178.95
CRISP2	192.5546	81.12516	73.98939	177.2832	58.91924	93.70207	61.88104	70.56424	346.4787
DPY19L4	68.02628	45.53302	38.28936	68.3868	147.7888	198.8294	194.8365	118.9322	65.3146
ACAD9	1895.983	4302.052	4573.761	5241.253	4110.404	2811.034	3901.444	5759.05	150.2601
STX19	11555.02	11649.54	10314.35	8024.13	11959.62	10809.48	8484.189	9791.302	11896.25
TSP50	47.57016	43.63744	66.7657	69.1263	49.16174	52.5359	43.31558	45.54042	238.8454
AGTR2	164.7997	88.65727	69.36488	154.1007	66.09125	118.5439	58.38558	70.81064	293.2968
L3MBTL4	39.60033	39.63459	39.46632	41.86657	35.85036	35.12314	39.62743	55.83747	43.58773
PRNT	51.65473	73.95863	58.93755	78.188	47.01906	37.0419	47.09845	35.67543	127.5172
TYSND1	5810.943	4721.861	5891.643	6590.124	4763.813	5301.64	5641.772	8295.748	8326.661
C20orf91	193.3139	108.0601	75.26141	126.7357	73.70975	87.30494	70.15861	86.47955	155.3391
EIF4G3	1781.705	1313.669	1115.133	1095.368	1018.98	1661.243	1165.927	1936.631	485.2181
TRAPPC6A	570.6541	352.1501	253.3719	312.224	206.0571	242.7016	232.5219	452.7623	632.3251
SLC2A3	7022.012	6807.918	11935.1	12994.3	7002.736	9376.733	9989.38	7930.21	3957.563
GOLGA8G	36.02048	35.5757	34.72717	35.21217	34.45219	35.52965	149.5462	36.55422	34.187
NPM2	58.39437	79.82671	39.78853	61.05862	223.1297	56.87877	42.26904	55.93335	100.0714
CDH20	372.0133	311.4955	133.1443	209.0008	105.4	169.3966	83.87734	146.988	477.9623
BTG1	2327.438	3181.35	3478.284	2828.57	3299.625	3132.957	3464.874	3291.595	2366.645
MRPS16	6587.042	6319.902	5366.919	8111.965	7532.729	4568.51	8878.139	5775.864	4889.937
FBXW2	39.24437	447.8642	581.3335	38.64602	322.2416	106.175	564.1792	541.0953	35.98682
NRAS	468.8631	226.4341	181.4144	333.5917	133.1422	245.0622	248.0943	108.7129	59.10517
TSR1	89.68393	338.1718	213.1625	421.1699	101.0965	181.5137	104.1646	32.82886	37.17694
GNAI2	38.73503	419.8278	679.9575	403.3406	1799.254	590.5626	1436.129	911.488	56.37957
GHR	6145.83	6853.783	3450.186	3925.972	4194.008	5167.302	3647.554	2782.777	2988.893
PERP	1002.974	1739.506	4217.092	2681.783	7102.927	4498.955	4176.055	4982.078	838.7274
TM4SF20	83.85066	181.0044	87.71434	154.7795	99.5573	94.38854	67.80261	81.5913	366.4265

LOC727811	170.3458	119.9976	68.6794	137.5103	50.11598	90.74689	55.76345	55.57486	206.6677
DDI2	552.7914	341.2696	337.7878	309.4641	199.1677	240.7428	211.261	313.7035	746.7592
GPX5	40.75749	54.69697	47.6143	63.56743	38.32561	44.94457	43.82726	49.45075	131.5694
KRTAP19-2	137.7658	63.74532	56.22988	42.35383	50.34112	69.36074	57.25365	68.22815	276.3932
IVL	441.4519	268.6659	144.0846	256.2863	227.8762	198.5562	201.1812	225.6117	566.2071
PHF5A	179.3741	185.7452	397.8503	121.6331	150.9633	76.99165	260.9767	274.1013	1128.066
HIST1H2BI	55.29476	35.92192	37.8618	41.45786	41.46708	34.93486	34.38049	57.76794	51.07508
SPATA6	87.83768	104.9295	262.298	95.44367	306.8544	676.2838	133.1189	45.36897	52.85549
PNLIPRP1	70.9482	64.01362	40.61034	93.74591	54.49737	62.72849	56.1873	38.89712	174.7736
EPHA3	44.11904	50.25428	333.8576	42.00272	285.8784	351.5602	83.28566	64.02664	50.01017
SF3B2	1235.682	1121.978	684.1802	2160.086	296.5208	523.176	608.0661	550.6443	1011.563
BRMS1L	40.71362	42.56054	42.41985	40.78112	36.56905	37.1051	40.4226	36.57211	36.49003
CAND2	67.0696	33.34498	33.20777	54.17986	38.84678	36.46987	35.93296	41.29006	47.02069
SP5	63.04638	59.55163	40.31744	45.32461	84.86649	48.92609	48.00272	33.39828	62.84857
MCOLN3	150.8238	77.33906	53.55629	94.43378	50.72557	58.74225	128.0722	62.35307	101.3335
EID3	299.6888	79.86329	39.98817	43.82074	55.45512	58.47105	40.91894	46.12933	486.0612
OSTC	59.74738	61.68837	197.606	515.2644	137.0928	60.99183	136.2658	39.40783	40.77533
EIF4H	620.1477	1363.733	313.8484	1100.934	396.954	426.8235	486.8615	605.4151	811.9778
UBXN2A	1906.534	1666.74	819.2056	1624.079	902.695	1249.359	1146.251	1038.798	1570.94
CCT4	3425.632	2736.06	2024.57	1901.905	3184.002	1806.902	2338.773	2141.304	2879.743
POFUT1	2545.255	2475.887	1280.084	3405.68	808.0015	1459.247	1715.676	1238.551	1496.949
CACNG8	33.51832	41.17538	31.86451	34.27818	33.41264	34.53655	37.24352	37.53354	44.49337
GTPBP6	26393.69	28771.15	31098.7	28839.7	31910.21	29456.34	28630.53	32651.64	26098.21
NAT9	805.8795	1216.393	1618.616	1212.83	1504.455	1928.343	1642.54	2341.347	836.0901
PPCS	11102.29	11682.57	15304.76	13381.52	14595.96	17253.98	16913.53	15858.86	10352.36
PSMD10	51.09744	36.87797	41.8568	579.4509	211.7205	49.61845	210.2082	54.64967	37.54128
NDUFS5	39.02412	35.88309	478.5104	378.0993	571.7472	1384.45	630.9892	659.2646	49.77343
PSPH	54.8648	48.31966	70.56801	47.49028	48.45475	51.25598	41.90184	42.02381	112.1318
ZNF622	5330.558	4798.437	4807.847	5407.381	4071.891	4656.353	5301.342	4712.74	4486.366
SLC10A1	38.17312	43.78601	43.28722	37.3498	33.69804	40.59625	31.29704	33.80258	40.20829
GSDMA	49.46791	960.8266	162.7281	452.3005	1546.823	269.7462	624.4072	936.896	1198.515
ABCC10	81.54795	623.7499	812.2721	561.9673	741.6265	378.3938	547.9406	422.6022	204.5502
MGC42367	76.07574	44.28884	765.9202	77.05338	2651.821	972.1246	1830.518	391.6494	112.2085
C2orf62	75.06639	105.2761	53.1772	90.83709	77.03876	56.30545	54.40891	52.74327	74.97427
RTP2	228.2875	170.9508	95.20332	172.5944	74.45657	109.2468	68.36742	106.9719	259.4565
OLFML1	4361.992	3880.033	3001.426	1613.169	3455.204	4159.1	2217.647	3915.621	4344.594
AGGF1	2328.215	891.8745	279.3932	715.9666	601.4523	595.967	688.9596	814.7513	1652.638
COG3	44.58773	30.67195	191.9189	34.81881	141.3004	31.85918	134.0865	33.05935	36.82785
Septin 4	14358.94	16305.09	13855.76	11835.57	14685.54	14290.62	13843.99	13250.2	14585.87
ZNF396	57.51166	39.77866	37.76094	42.25557	90.64581	33.89495	39.11858	85.43279	109.5975
PSMD7	855.0902	429.0078	267.3465	456.6583	241.3728	70.54178	510.6615	675.7772	1283.017
TMSB4Y	592.9104	307.6759	32.04682	35.52391	34.75605	39.84751	145.2918	306.8074	836.4507
TGM6	170.4836	354.1621	80.07611	148.5595	168.2715	81.24855	60.65332	50.8379	249.4147
TMEM174	267.6088	69.64326	83.55035	124.3753	81.02546	128.9309	68.15131	69.94283	232.6799
CRYGB	124.3818	83.77311	68.61224	113.2954	65.37496	90.07841	56.17628	67.15252	401.7348
CLN6	195.0162	349.6179	296.6476	391.43	112.4865	268.1276	112.1999	154.0733	178.0051
LGALS12	146.7324	84.85559	57.17772	149.7826	131.9036	180.2772	58.32246	181.0123	2123.834

GOLGA4	544.4798	75.26934	174.1718	440.2059	213.3436	277.0923	105.257	142.3243	1225.524
CHRNA10	1536.651	1345.99	1503.301	829.5417	755.0694	892.2557	730.7663	992.3523	1890.669
UBE2Q1	77.21693	630.3884	1184.432	1084.294	1184.943	2173.722	1227.773	1094.964	1112.41
TBCEL	171.7014	56.7109	44.06529	58.34323	80.0835	41.38597	93.60181	150.0571	68.81688
UXS1	293.2706	170.4216	91.582	174.1047	88.41203	148.7501	79.45004	112.4103	129.3244
MUTED	1189.661	1668.252	2476.698	2126.513	2328.369	2980.05	2816.323	3287.914	1736.121
C8ORFK36	38.99336	47.34443	36.46851	51.91048	48.05002	46.9552	35.60973	51.19973	36.15485
PIN1	11184.42	12876.18	8413.26	12941.23	12767.33	11360.39	15370.12	4510.896	12035.01
ARMC4	40.43738	47.10384	39.63246	69.80716	34.25573	40.30928	40.88295	42.3002	48.08852
PAIP1	40.95298	36.3349	48.01515	33.87217	45.86788	35.36633	46.94729	41.33976	34.24278
KRTAP10-1	79.73546	61.18948	72.0109	64.17998	65.386	76.93446	60.53719	57.65219	117.2979
STK17A	54.01497	250.6356	108.1502	47.40992	43.50717	59.74831	48.56203	47.5561	62.06718
GPRIN3	1248.807	435.0372	124.9185	488.2746	101.2246	202.7185	153.053	163.5871	818.2396
ARPM2	58.51424	45.25228	46.61129	61.99315	41.53772	41.17424	36.42735	35.78173	214.5425
PARP4	127.0796	318.4529	2358.885	1859.213	2667.382	2212.891	2705.549	1581.312	59.30187
TADA1L	63.56755	70.886	57.01391	356.682	76.18392	69.74516	71.54163	64.8858	780.8141
C17orf53	9097.132	9498.814	9998.021	11053.29	7458.111	8825.42	7881.61	10816.85	10516.14
MPPE1	609.2326	1635.987	1068.563	52.32352	798.0699	1051.572	1569.857	1710.784	3339.309
ITPKA	2423.571	1447.712	646.8858	1242.139	606.4771	1781.814	1001.457	705.5567	1735.03
RAP1GAP	8903.342	10143.53	4157.622	9665.875	8153.256	9510.001	7067.3	6361.116	8272.811
RAG1AP1	3710.492	4104.494	1680.14	2325.943	1333.076	1731.534	1081.735	1233.024	3472.41
FUBP1	1140.703	584.2606	984.7432	1116.321	1077.764	1163.061	714.5413	1168.755	36.19112
C1QL3	114.65	70.80129	56.66754	107.7712	54.04296	68.18689	57.24236	45.34443	301.1289
DSCC1	1027.378	1342.029	906.0182	1374.763	748.6951	946.6897	824.5385	1228.249	1051.164
PDK3	2877.746	1364.426	1937.939	2160.409	1709.951	1787.13	1528.944	1730.577	50.32213
METTL11A	169.0472	112.7137	89.98792	124.3953	73.8415	94.45677	68.6686	67.76167	108.6323
RNASE4	1403.12	1252	376.9602	641.3974	1120.753	836.8535	566.2592	662.498	38.84293
KIAA1862	240.6682	161.9855	106.1999	209.1985	112.7049	139.1061	102.8535	126.9212	414.6859
MPL	2387.171	4500.948	1696.404	3380.344	2077.173	2433.236	1981.266	919.5827	1976.083
LRRK2	216.2362	80.96722	112.5156	106.3367	75.43975	90.88248	95.33134	128.9321	397.2112
SCML1	49.48095	240.2617	117.2157	353.4565	89.62997	42.04889	85.09383	32.52098	56.18676
GRIK3	58.81349	52.89867	121.4979	64.34842	46.71578	49.99554	81.07435	79.66032	75.58224
SAC3D1	1085.507	2077.034	1827.522	2393.083	1744.537	865.1614	1690.273	2342.146	2822.764
GIMAP7	30.91771	36.72883	221.0748	303.2592	71.45713	184.8213	168.9279	170.6516	32.92973
PHF11	2287.013	4255.425	7536.82	3441.713	5411.052	8158.151	5975.752	5792.081	1937.491
LYPD6	161.9565	169.5044	88.27543	114.1047	169.0918	286.1858	171.1588	71.60617	237.8245
PARP15	97.43589	395.4865	464.1972	57.33381	121.2866	319.5239	282.735	70.13345	64.50329
C5orf41	48.37661	954.1562	761.2411	104.6421	280.7848	525.742	319.5584	152.3049	68.80718
DKFZp451A	32.29591	1430.685	1112.422	278.344	629.9729	2505.111	1678.877	2985.335	42.7639
HIST1H3A	158.1245	281.6721	363.1593	1545.078	157.729	51.98712	95.61649	148.8217	94.71432
OR52B4	39.95437	47.78366	34.13668	49.03773	33.54979	42.23658	50.73771	43.3335	51.526
TUSC5	311.4037	210.0764	90.83501	172.0718	82.05047	89.88533	71.0755	100.1199	382.0681
DUS4L	740.1834	576.8895	301.4529	1435.437	560.2367	1195.538	518.826	276.8022	767.1873
HAP1	101.0471	60.95002	43.36454	69.90816	38.81418	57.182	508.7047	33.10712	36.8525
HMGN2	473.0483	302.7241	129.4682	307.1895	112.1861	218.429	97.10096	113.6171	464.0401
RHOXF1	46.20474	37.60628	40.5724	35.91758	43.07496	65.9381	38.32151	40.75639	38.91074
FAM23B	66.02606	255.7521	60.13468	81.35616	62.07843	57.62333	89.35783	47.42066	60.69232

PRSS22	180.0729	682.775	351.9843	127.4994	77.72948	99.04939	778.8055	69.45499	236.202
ANKRD38	5917.418	2786.115	514.3232	6866.255	881.4218	1171.858	3407.62	1314.79	1632.19
LMAN2	496.32	295.2675	207.6464	277.1313	305.2009	312.1295	266.2092	286.4292	983.0425
LRRC50	61.42406	97.80315	69.70207	66.82782	66.1184	77.59412	53.94325	62.04002	147.9948
COG2	4294.46	3311.757	3328.656	3882.063	2876.17	2364.865	3007.478	3760.114	2897.759
DNAJA4	3047.258	42.34741	154.4	35.87328	1037.471	479.91	1171.976	846.5836	2380.675
CTH	68.12743	38.68998	56.08693	93.38149	53.69698	64.30828	48.05482	63.98909	63.61263
NIF3L1	1702.093	3308.304	6399.86	4741.896	9975.327	7360.31	9116.056	6031.102	4375.116
OR5H2	34.81731	39.45136	40.58773	45.61733	49.33761	46.19513	40.01052	43.85817	36.59052
LRRC57	40.45161	609.9232	593.0888	60.3348	1204.972	620.7142	814.5338	492.5038	79.62066
NEURL2	46.53453	67.48743	45.96409	38.06469	119.6942	227.3822	154.1465	43.15657	174.8644
INTS9	3745.664	3991.718	5036.987	3059.059	4716.304	5062.531	4901.802	4200.134	552.5121
C16orf56	72.29458	81.91691	489.7396	75.77673	1568.314	71.57088	533.3958	405.6154	47.9732
MAK16	2622.448	2660.259	3444.596	2267.139	3715.429	2892.787	3490.341	1858.247	2207.788
AGC1	68.26697	68.79162	35.42597	50.93561	49.20063	34.60457	57.95113	39.67342	51.97267
CFD	27818.1	26501.18	19499.6	22304.4	18558.46	19520.73	16820.97	23921.19	28286.79
ZNF157	415.026	70.4203	60.63756	176.3388	62.10833	109.3916	49.49189	61.08942	178.7338
GRP	143.051	51.95745	57.3381	137.0853	56.8842	63.44672	58.37362	61.00056	94.90366
C2orf67	333.1456	71.06985	70.91484	49.88275	63.69024	55.85198	51.93305	31.85361	145.0319
VCP	41.88609	47.69098	35.20799	37.99	40.5626	70.22237	46.00604	42.72437	94.15621
E2F2	7840.427	8124.763	8609.224	6482.064	8715.014	9495.053	5572.022	6394.424	7662.706
ATXN7L1	428.9044	320.876	164.4454	287.8963	124.451	215.7865	128.9384	153.8935	616.0717
FAM3C	44.47441	638.7017	174.7426	1590.771	153.3786	84.29659	335.8926	250.5832	39.74288
MEOX2	44.26031	1786.09	459.7171	343.7034	2179.774	712.2679	1143.524	739.437	50.23718
ARL13B	33.10743	37.3192	36.16557	31.33286	141.9385	38.01532	82.20498	274.839	32.73296
CNPY4	117.4892	922.1362	184.5731	620.0779	143.9201	454.2056	195.6386	177.16	59.30363
C9orf64	314.8742	229.3445	95.97924	519.3937	55.782	75.8178	202.6163	294.7845	137.416
FAM91A1	55.20115	295.9499	119.0201	307.5711	259.1964	194.9789	278.6904	315.2159	46.96004
DDX6	853.784	640.9657	754.331	440.8798	310.9727	351.0426	412.3877	141.3902	741.0828
PREX1	75.2523	69.45049	37.88382	57.72022	50.95601	53.36942	49.92046	36.38699	194.9276
RPS13	711.0893	442.1555	1174.882	683.8195	1662.178	752.2762	1091.242	900.0563	491.4899
TTC35	216.501	226.8519	117.7949	198.2911	111.0165	190.2312	109.7737	123.7458	384.7839
MSX1	4189.214	5548.876	4160.875	1066.127	4313.444	3064.192	4916.37	6335.895	3261.42
ATP1A2	33.22091	1944.335	306.9012	35.71009	133.0617	353.791	344.1707	632.8239	872.1137
GAGE1	306.4139	154.4428	69.15253	148.9799	55.56472	102.9797	53.40817	58.30499	347.787
C1orf177	412.306	212.6682	137.5423	453.1142	80.30442	314.6719	86.19611	102.1611	479.6935
TCL6	110.0485	91.20092	72.8161	65.96748	55.19578	77.19004	50.17486	58.28541	143.1832
IFT74	920.6691	982.4562	1338.565	1789.706	1235.459	2450.29	1091.318	690.2839	1618.616
SPDYC	175.1246	116.1595	73.63519	132.1425	66.44388	70.87811	52.34489	88.30847	78.53669
C9orf106	50.21758	43.61553	46.76812	34.79613	49.61259	41.19367	290.74	51.08284	99.21252
CDYL2	407.0063	235.6963	96.18902	251.0148	136.5317	172.6331	84.85916	109.8248	631.0257
APOOL	42.75695	45.84581	1134.23	1131.168	1275.057	502.5106	1660.827	1618.334	38.80078
ZNF839	198.8216	80.49322	168.1179	353.4218	63.7644	70.09399	65.18462	58.30724	238.1162
KCTD11	1009.821	1143.086	628.8935	2381.252	710.4546	979.1397	565.9251	660.92	846.1939
ITGB7	164.3512	144.7812	115.8104	112.8727	64.99752	54.0031	77.53001	125.6602	140.9596
GLUD2	80.38755	151.716	74.20844	169.9257	74.49798	66.13735	66.1443	68.09102	123.1311
MED20	49.27445	1602.527	2686.238	2268.31	4158.796	3251.615	4680.694	3226.412	934.8851

NCBP1	10113.17	10504.79	10976.8	11474.57	8047.07	13271.84	9412.899	11705.65	10322.68
KLK6	401.5028	220.3166	122.4216	259.1255	189.9161	215.7449	136.1823	186.6708	512.5258
ARL13A	110.7212	52.45012	33.62721	48.09177	41.98234	42.53872	40.94165	39.88386	45.92807
ESF1	873.4579	1403.683	585.3168	509.7727	392.8596	534.0961	379.8562	325.3012	1449.214
SOAT2	58.93335	47.42719	44.8393	50.31677	43.75943	36.44264	35.44154	40.36674	58.56182
TIGD1	80.09909	528.3946	493.3331	420.6133	586.2086	579.7106	463.1855	408.9019	159.8524
KARS	52.0089	46.56888	556.5482	70.85987	671.5309	410.8197	1056.129	474.8477	58.36383
C10orf65	45.59921	400.783	723.3143	49.39431	1097.18	1668.29	1416.698	2614.361	48.22445
SLFNL1	44.7815	805.2402	133.68	369.4751	69.97453	35.74065	176.9499	193.4998	973.443
OR2A2	256.8384	109.6426	61.06385	82.36347	69.63506	102.6442	62.91182	64.79707	125.5671
SLC22A9	46.63516	46.80389	36.96424	42.58332	35.47502	36.48511	35.85191	30.96821	51.49608
SCARF1	69.31706	73.57713	50.37865	70.72783	46.03028	61.44992	47.45523	69.88399	39.57571
RNF144	199.6514	177.387	178.2529	198.6904	440.6986	582.5315	475.3763	170.9575	397.7645
TOP2B	1437.053	1289.305	1066.947	1321.112	756.3314	1224.768	558.1483	353.4213	258.5032
SLC22A13	47.79927	61.55804	54.95075	46.05575	112.1675	38.76685	41.5038	35.72063	65.64866
ARL9	68.8026	50.94033	183.2369	1224.586	535.2007	811.5671	480.935	1096.936	76.27962
OR4C12	165.5864	85.68317	88.83897	119.8218	78.81292	98.61218	74.43656	89.15961	83.5262
C14orf121	215.7383	112.9155	75.20224	178.461	189.2517	109.6005	163.7262	89.34667	380.9106
MAPRE1	430.4101	249.7687	1318.463	1790.725	2002.976	1576.222	1578.081	1006.425	2776.187
LSM14B	330.9187	282.7676	1801.124	2873.696	1278.612	1631.774	1539.227	1671.857	1806.1
C8orf55	7585.368	7945.765	10420.74	10145.73	12352.49	10249.85	11783.9	8797.474	6473.94
FAM133B	40.94007	344.0126	697.9985	430.3441	823.5225	491.6285	629.8919	951.3892	40.8622
LOC40172(	4174.682	3842.562	1775.892	2768.652	1779.245	2801.777	2735.72	2136.722	4086.641
C12orf10	21509.99	20241.04	17731.74	20454.72	17113.16	20912.71	18544.03	18334.83	21714.21
LOC65398(	36.78706	45.51056	34.19037	45.99098	38.50204	39.47599	32.63221	35.06396	36.52094
PRKCSH	7500.097	5940.964	4065.318	6201.024	3552.211	6197.753	4245.204	4479.284	7128.118
AFM	30.35012	41.95233	34.37555	40.67771	41.43928	38.7315	41.37553	38.63523	38.30302
URG4	1723.057	3848.654	4756.449	4941.989	4820.425	3912.94	3789.313	3564.622	4145.789
NOTCH2	55.09723	42.50645	104.2925	60.60181	94.21856	161.177	62.07065	56.20316	46.64355
MBOAT2	66.31454	45.9451	51.49118	61.46	47.46717	59.67689	58.81768	140.0854	58.15845
TSPAN17	14643.91	14396.39	11324.79	15891.16	11448.98	13320.76	12489.08	8680.356	14484.22
LRRC37B	7903.898	7568.597	7769.464	8520.258	5981.976	8986.479	7008.504	5670.738	7260.867
RPRC1	17636.82	17432.57	15812.57	17946.75	21519.43	18706.68	21488.88	19619.68	18588.9
CDK8	53.71734	41.76258	48.77841	64.23906	38.58891	38.34166	43.65172	208.714	58.47716
STX6	201.0997	132.052	104.2424	198.125	317.5528	149.9099	229.8465	109.7517	188.8393
DSCR10	300.3776	210.1887	95.60075	232.8826	103.0058	127.6193	93.53522	131.687	525.3065
MORN3	101.6719	67.02316	112.8673	68.12086	61.2691	65.92731	49.47968	68.70521	158.7763
SPRR4	2490.897	3171.251	247.5377	1335.953	1066.127	892.1123	445.4811	1637.843	741.4951
C1orf114	54.98125	82.65818	76.52241	63.33475	45.35093	66.77756	57.77481	62.76661	75.25584
USF1	238.6019	926.8052	1320.189	940.3968	937.7515	645.4957	1103.062	1352.753	261.6458
IL32	8131.624	6974.656	3627.992	5085.12	1583.745	2875.214	2978.416	4827.398	4566.934
SURF6	10744.48	10402.89	9595.989	9950.578	8524.783	9531.482	9188.171	11778.04	10260.89
FAM9A	36.17386	33.46784	44.83563	34.86593	47.952	43.68174	37.08315	37.12188	33.03526
SLC39A7	288.1351	108.2936	60.9234	75.90708	66.67255	119.7182	61.74768	82.44557	178.1759
FICD	48.24899	59.8879	863.4033	77.56593	2171.539	873.2451	1527.345	920.481	88.93143
ACADM	123.9572	894.757	1204.4	63.26263	860.2479	1191.708	777.4843	828.2269	111.9935
GMCL1L	39.61859	56.98898	43.13062	54.33245	40.63195	53.0173	40.22309	63.20692	59.59966

C2CD2L	39.72405	60.85153	53.63314	51.32309	43.19921	41.06046	36.48593	274.6788	45.57153
ZMYND15	804.2204	3105.826	3872.153	3103.642	2096.423	2949.947	2658.541	3502.397	2231.713
GRAP2	1308.352	271.6188	229.2056	317.9357	78.70883	433.2857	147.7978	317.476	52.49199
FLJ20489	264.3444	1826.149	619.374	1013.834	1017.5	1231.019	1057.637	971.4764	1178.542
FIGNL1	99.46799	49.1447	57.89396	45.73953	36.75694	36.18637	52.17576	44.57145	78.68977
DNAJC25	35.80596	889.4091	1451.287	483.6345	1601.356	1939.275	1538.927	1093.09	33.27252
ELMO2	37.10814	69.73952	314.623	48.6202	45.39546	38.43943	133.365	262.5865	54.75468
AK3	6256.218	8105.315	5094.026	6034.218	5311.804	6969.374	3582.783	4907.29	6694.283
TARS2	59.68518	455.3874	165.4602	508.0733	45.16519	308.6274	249.3752	172.1789	633.236
TP73	52.78217	40.56396	34.16166	1915.023	294.728	47.29393	57.56368	31.28325	2044.072
CLN5	42.16951	888.9399	3484.697	3223.596	4493.005	3225.573	3532.909	3855.434	2641.915
STK33	44.47383	1605.023	478.997	589.4608	160.3926	258.5253	139.9618	439.9117	1014.134
EXTL3	76.9764	90.19016	65.60986	104.6561	98.7944	71.11526	53.39498	61.56599	72.60266
PTPN11	32.95392	548.431	186.4802	395.8752	426.0411	226.2364	553.4296	216.5634	46.75031
UTP6	90.17071	557.4871	1699.792	1977.955	2595.484	1855.946	2410.49	658.5548	100.6175
GFOD1	4404.082	7421.328	9048.065	9237.729	11380.85	7797.666	10617.86	11031.15	6072.516
CALM1	3356.224	3672.704	2653.325	3160.471	2792.861	2259.737	2474.968	1929.321	2225.271
FARSA	36.05932	37.25991	341.2352	35.07114	834.7414	351.4191	736.6553	106.1707	39.75044
RGS6	123.0588	424.5373	70.27978	98.59848	70.05674	125.3924	53.24193	83.28566	315.9723
HTR3E	53.56264	37.05145	40.37859	45.09006	41.76978	38.80053	49.98938	36.78842	53.76672
SENP7	172.9248	108.2935	64.44936	119.5536	157.2064	108.4206	58.60221	59.49282	79.98338
COL4A4	36.48018	31.79301	142.7486	339.7024	246.8239	255.8107	432.8086	147.7869	101.1682
NOM1	322.5665	166.1905	97.89056	81.83204	287.6981	98.09838	142.6783	59.4371	163.6071
FLJ20674	204.3986	150.9425	76.52563	84.06799	92.00075	94.58717	67.06325	95.24079	76.25226
DMKN	50.79431	823.5073	1095.348	1827.926	2651.196	531.7793	1033.245	1987.918	54.63855
SLC2A6	12914.71	13690.05	16134.34	16271.65	9082.858	12840.96	12913.15	12552.12	13138.2
PCDHB14	518.2409	269.603	112.9991	272.4493	128.0326	207.6164	106.6048	166.7944	257.408
MMP24	86.47881	49.51594	61.04199	56.35786	58.15922	48.64453	54.65173	53.72026	71.20988
NUDT2	1160.681	66.81643	1359.619	1139.132	1991.226	1540.437	1656.643	1594.555	89.78383
DNMT3L	58.71876	73.08118	74.37693	62.13539	53.42328	61.54091	60.01061	57.05287	51.3933
ATP8A1	2197.867	1963.626	557.332	48.84437	46.76108	42.25638	142.3331	831.5989	42.47415
PYGB	51.61307	49.99837	63.67534	45.55743	143.4528	69.16694	121.3414	104.3832	51.60271
C17orf102	83.44044	53.92106	56.12054	55.70235	50.64038	45.61269	44.61264	43.73756	38.13018
HLA-B	6535.458	5554.754	5781.294	5743.791	3982.737	6328.703	5863.056	4328.339	4055.77
DPP10	70.37319	59.9868	49.25283	80.56355	47.60415	65.65401	45.74596	51.03666	55.06384
FKBP5	598.344	3012.427	3428.076	4365.054	824.5748	1875.964	965.6649	1599.558	2713.008
IL18	13795.08	11873.59	11001.96	16426.91	7277.782	11437.4	7055.639	10339.16	12021.84
CCDC28B	67.68849	1649.299	1446.63	631.7577	786.8653	1124.166	997.2579	1529.169	42.08512
WDR41	1197.511	1681.313	1883.749	1966.531	1305.371	1944.625	1231.687	1751.435	888.8086
LOC40098[illegible]	287.691	130.6397	76.42591	38.75057	82.35474	51.09991	71.6384	36.15241	58.84034
MC2R	39.63906	37.02328	36.14149	39.91632	157.9718	53.75206	123.0964	39.03292	85.19112
KLF9	68.60555	44.70891	140.1381	59.22862	235.1894	181.9907	191.0173	175.6814	51.91115
BTLA	158.0474	112.378	128.0213	141.2728	62.18597	54.868	68.50877	63.10229	684.1463
DUXA	41.8764	91.02226	69.87672	61.84469	45.33487	55.98558	46.50722	54.66634	92.00294
CDC2L5	2639.968	2355.578	3136.826	1643.348	3216.255	2751.401	2721.768	2082.347	2160.975
WEE1	542.7557	52.19087	348.1931	485.594	318.5148	492.1822	349.0682	518.0612	67.08918
WNT7A	106.3735	55.17348	408.007	69.9032	47.39809	66.37766	374.9666	49.39723	161.4705

SLN	11513.9	16738.38	13053.69	11891.15	11331.34	18383.85	18975.53	23655.64	12863.97
GBAS	409.14	128.9454	142.5389	245.2168	219.931	217.9454	270.1131	225.2699	259.8476
TAF9	66.82662	214.3475	127.7741	67.58089	98.71599	246.3964	61.71084	106.0537	370.1642
PAGE1	41.15124	33.65147	41.04755	61.25971	33.56615	37.5285	45.30492	39.43935	86.57029
CD44	744.6252	368.3196	540.1474	828.8837	429.1833	363.6596	343.3346	284.1249	64.15733
DEFB137	49.8157	35.99318	35.28535	44.69937	52.00832	84.10487	38.30014	32.31865	42.14574
VILL	75.67448	45.00784	33.22506	55.38968	37.47136	44.09068	39.74105	39.75312	75.54303
LOC73009‹	55.91696	48.21991	49.28095	398.4176	100.6445	345.8941	77.48315	119.6979	40.08957
GOLGA7	1556.929	913.3257	886.4554	1268.147	691.3856	692.0636	651.4186	796.2667	1438.505
MRS2	41.40893	51.88976	118.4626	63.86684	35.99314	51.84639	88.64815	37.01237	61.05978
EBF1	35.99511	675.4269	220.9229	46.51417	963.3803	59.87203	597.2512	216.7681	104.6024
RTN4RL1	226.9922	128.5681	91.66009	151.7857	76.72791	113.8089	73.64635	158.9561	315.4879
RHCG	736.1208	851.3138	4281.692	1246.157	4210.567	1616.465	2145.367	3730.385	261.3518
DBF4	226.8164	186.6303	83.57651	151.7553	81.80506	103.4309	71.57393	102.0654	183.501
AMICA1	138.9691	505.2114	1327.7	149.2523	993.011	645.0875	797.2404	900.4006	242.3411
GOT1L1	107.9833	86.56261	60.98267	127.4943	85.70518	76.99746	65.43251	68.26852	229.7388
SMPX	41.5717	283.6792	116.2963	237.0254	64.56425	226.1815	156.475	240.2599	162.5707
TRIL	65.46072	485.2611	942.2616	523.9806	749.6312	1628.893	1006.643	1403.656	84.28329
AMOTL2	418.1964	242.816	131.0305	259.2324	132.3396	170.9175	103.6978	144.5215	538.2408
ADSS	302.6994	197.875	136.398	169.2834	147.7267	168.0918	95.05565	159.7838	176.0731
ZFAND6	69.65423	285.7338	199.4542	480.5634	127.2098	271.7549	115.5413	159.9754	132.1107
NTSR1	5494.01	1069.11	452.8438	10408.76	231.3254	1891.318	1902.133	472.8167	2951.486
SFRS7	142.0763	107.9234	188.3798	126.9073	335.4345	240.4518	233.4711	145.8625	203.0381
FAM5B	124.5612	117.0389	64.27426	152.9072	58.22713	75.01963	56.6919	77.08289	165.4884
FAM111A	57.50871	74.2555	1152.974	2441.404	835.5403	52.65731	849.1619	300.277	59.45381
FAM179B	3633.948	4207.712	3901.255	4207.749	3855.38	4986.305	4303.267	2752.416	1846.309
ZNF345	64.38059	88.23978	56.47292	68.25106	72.03418	98.9453	138.8304	76.75351	185.8765
TBC1D21	267.5049	176.2105	93.30897	210.23	98.40841	138.2382	89.37688	107.0501	312.1358
CDK7	3455.309	1909.906	2676.051	3395.418	2798.055	1276.483	2377.375	2319.877	1695.222
C6orf203	207.9738	185.524	116.9832	231.7818	100.0391	160.3228	86.28775	104.7148	286.8929
STS	36.97499	822.3073	536.2771	38.16016	1008.471	1240.149	494.203	729.8425	1123.89
CLIC5	39.69949	38.09264	43.22857	46.89698	76.63971	37.79422	121.5619	238.9328	32.6715
TYROBP	2255.87	1651.906	564.6119	1033.117	52.29578	37.15976	166.8293	43.7883	40.14084
PRMT10	49.83265	451.7771	887.3385	52.2973	1436.987	917.2222	1351.466	1271.528	1238.151
NFIB	112.8314	1834.993	2839.464	840.5565	4618.934	2759.808	3270.741	3325.573	2201.931
MAP3K3	2041.218	921.39	330.1752	44.19633	340.1955	213.7405	224.864	549.838	44.28971
TSPYL1	4483.062	4343.583	3330.263	4001.723	2542.904	2642.953	2116.085	3242.907	4463.958
ZNF467	12974.87	14331.48	12681.41	8865.254	12649.42	12695.54	12688.28	8824.82	15393.16
ZPLD1	36.32373	38.60088	36.91833	30.76805	31.63244	32.54015	37.93191	45.25957	37.03774
NADSYN1	41.63542	41.49668	702.8765	64.68076	585.1674	379.2138	668.6261	226.5049	44.58545
INO80B	6566.569	3959.243	1948.016	4328.324	952.7114	3534.11	2178.098	1502.611	5236.37
CACNG6	2298.718	7800.191	3230.225	3249.437	3309.318	5310.764	6147.122	12126.84	2868.01
ST6GALNA‹	49.58092	40.39888	55.59092	41.28555	153.0323	302.2427	131.7355	332.0357	41.56561
HINT3	80.13255	73.94157	276.6094	136.5117	58.71166	118.7467	71.8035	292.4293	373.6384
OR4D11	85.93638	91.64268	48.51014	52.36633	57.02394	55.24378	49.55176	49.6287	74.49635
CEBPE	253.1788	109.7615	87.72612	89.6881	68.90749	100.9984	58.82844	67.39303	206.7336
ENC1	176.1585	264.7698	240.8407	247.3509	264.9274	287.6872	325.7278	270.3621	91.00891

KIF18A	42.74276	43.05149	40.58768	37.65233	45.2303	35.03874	31.38704	32.72563	43.91853
C11orf67	1836.948	864.3944	166.4526	336.5922	145.0939	220.192	148.4541	217.9386	687.0308
SGSM2	7152.539	7450.111	8234.617	8519.742	8530.456	10032.96	8141.645	5522.136	8495.262
U2AF1L4	657.5975	325.8653	380.7464	410.3781	178.9585	408.8035	234.5124	244.686	747.7344
CYTSA	7722.188	9140.884	12042.92	8805.481	13210.73	11412.14	9989.957	10109.49	6463.094
ALKBH8	42.21196	39.57638	286.3715	39.60316	375.2006	465.5762	331.761	248.8007	73.71011
TFG	46.89317	51.51921	40.56184	40.7737	55.44347	47.43358	41.60344	40.55029	56.21962
C7orf33	40.23103	34.63806	35.89839	34.08781	38.91856	40.65087	38.05293	39.79507	36.9453
CDH2	509.9013	236.5681	125.3152	2177.113	109.8895	162.1547	113.3438	117.6856	620.5416
CXCL6	163.6234	108.4247	69.85805	85.16031	69.30138	92.86443	68.63953	82.75022	194.3226
TRIM22	944.3558	1757.208	1207.16	835.8656	142.5332	553.1525	477.5208	402.4884	717.1438
ARMC2	61.96735	1197.135	490.5596	66.54189	162.3243	187.0004	229.3863	280.7354	1638.895
CD40	34.78543	2376.91	572.6853	769.5143	202.7981	952.8152	512.547	1112.179	2289.34
C14orf32	40.42072	59.79288	288.7922	454.3828	330.5154	281.5766	419.2639	248.4517	61.71311
CRYGA	221.6765	111.675	87.8682	149.0033	89.24765	133.8516	68.48686	75.73462	216.223
GZMA	1447.529	127.3983	1955.881	404.0417	811.6467	1639.069	1690.786	1335.34	325.9285
ZNF630	42.4799	39.02155	231.4044	41.75791	442.8974	220.4513	308.1179	155.1764	32.57799
SNCB	56.0704	48.27712	54.42643	786.0148	53.44109	59.58101	44.03856	43.58541	42.18254
GARNL3	36.6692	40.33455	34.49056	45.46334	84.19884	161.7713	32.90045	34.89393	31.45055
PUS7	1121.801	1039.109	2173.785	2684.336	3478.875	3143.323	2643.625	2728.633	284.3439
TP53	1006.617	438.147	318.3356	35.86724	229.1794	669.0531	312.753	129.6871	1400.662
ARHGEF9	132.567	155.256	72.04946	61.93338	62.30338	114.4022	59.75177	56.89967	218.3582
HIST1H2BF	92.25948	366.2676	44.60979	597.1062	80.11854	256.1686	128.1915	245.9574	62.20642
GSK3B	5156.513	7263.458	11011.01	10557.85	12294.9	8148.204	10892.22	12634.38	5981.739
SEC61A2	1132.712	419.8916	438.5451	329.3025	340.4925	843.3021	429.4701	282.5448	41.17982
SNRPA	131.7519	77.6995	225.5452	79.88528	295.7517	93.08407	471.1634	229.509	225.2151
CHP	1806.909	1922.282	1397.187	1727.559	1684.626	1197.812	1179.706	1550.229	1803.333
PUF60	7259.61	10432.78	14334.34	12988.25	16738.22	12411.97	17887.03	17019.4	8143.973
OR2Z1	130.5064	91.12675	61.25989	147.9076	61.02909	85.32576	54.94827	68.98792	111.9849
OR2AT4	51.94182	58.12511	37.26489	43.83383	45.64291	57.05287	51.7951	34.42224	143.9611
SCUBE2	1344.502	705.2032	1138.46	379.556	2717.696	2286.481	735.0371	2037.803	1575.352
MRPS14	177.7542	1538.946	1484.365	1033.801	595.5363	1560.542	916.6978	661.8934	2504.96
EFHD1	89.37539	115.0777	63.46955	76.66127	418.5813	472.6156	952.3493	245.4696	55.15488
ABCG4	4414.168	8294.05	2844.224	5817.673	13708.74	7607.739	3213.869	3660.485	2806.693
VHLL	50.67229	55.81649	38.98925	42.52686	39.54318	48.40952	38.13777	51.46824	59.3619
FAM126B	1062.577	80.9042	198.0238	124.7275	230.1332	113.4952	190.105	285.3461	237.2891
MC5R	624.0684	168.5752	68.60567	269.0972	46.53551	48.93478	59.44641	46.09047	137.7757
AP4M1	47.87194	50.62422	34.9254	58.59045	41.73908	41.31816	100.7476	44.37869	35.01461
CSAD	68.83779	748.0856	218.0663	67.99399	52.96455	68.33271	120.6091	76.74014	191.1452
RASEF	49.07945	1062.978	910.9618	54.84848	1032.458	936.3133	1114.571	518.8678	1267.509
ZNF347	39.45464	38.96161	264.5694	34.31556	97.43333	38.20488	235.4307	36.57649	35.01958
CREB3L3	916.219	273.9443	168.1423	54.77516	70.62093	305.1093	198.4905	201.0088	319.6716
COG6	1550.651	823.5436	331.9037	457.8806	360.3175	256.3661	289.558	315.0296	193.1179
NCRNA001	2599.327	3399.739	2889.94	5822.065	1748.702	4290.881	3036.657	4296.993	3208.852
OR10J5	56.89087	89.48353	41.55907	55.16845	54.41182	38.29728	55.92992	34.69482	139.0382
UTP18	219.204	133.7507	85.89117	162.7125	88.58308	147.1065	84.05034	85.05238	330.1064
CIDEC	414.0386	177.4128	97.73824	177.1841	88.71381	133.6568	77.7821	116.2987	396.2532

RIPK5	333.5242	151.9973	219.965	215.5065	70.03036	665.0737	66.74304	471.3796	124.8247
SUCLG1	13581.34	14034.18	12930.92	14614.22	12999.7	10742.47	11231.42	13352.03	14736.04
PSMD6	2132.167	179.5877	995.5291	1619.036	969.351	771.5767	706.6246	934.3962	508.1245
IRAK1BP1	1189.324	79.20959	181.5986	124.2082	214.4593	447.5463	362.807	212.4953	144.2566
HIST3H2A	434.7145	282.8789	295.3962	827.3768	126.8263	203.8163	164.6693	125.621	560.1744
TRMT2B	57.35	49.88708	192.9621	78.644	185.6842	229.6382	173.9064	41.87341	173.4138
A1CF	39.10699	48.14603	65.30679	83.71442	54.26814	63.94281	58.21718	57.24575	64.53114
PTPRG	120.8005	65.36475	282.3403	79.77077	274.7223	61.78782	608.7957	281.3833	132.2994
ZCCHC6	10173.27	11517.44	8389.557	8925.684	4961.339	9778.396	5904.299	7633.851	10913.66
ACCN1	31.77604	39.26614	39.41345	32.46203	195.9994	34.21785	358.8884	267.4898	52.66386
LOC154872	79.65881	54.00101	55.61167	51.41615	49.63287	61.22949	48.59899	39.31806	51.08316
TP53BP2	38.92113	35.43828	175.4651	42.14259	371.5938	54.92649	451.7162	43.95714	1729.14
DTL	39.79255	360.4415	389.7066	376.1689	220.1018	402.1497	426.5171	102.7636	35.23853
C7orf59	215.1777	857.9567	589.0223	1674.235	739.3914	102.7054	847.7966	679.8131	207.8474
GABRD	178.4323	36.37848	62.43737	50.56073	244.1765	73.84485	57.15145	51.9219	140.3381
SYCP2L	70.83936	79.39899	48.46245	99.48801	91.39545	48.61471	166.1821	116.057	99.32918
C7orf55	630.9527	654.3212	485.2091	400.0333	505.6565	678.99	583.1671	418.756	826.6422
OR51B4	102.9098	54.64239	49.75657	42.64293	51.40114	73.7459	56.48036	55.97525	106.9927
PRR19	390.4946	190.2321	255.2907	239.1534	116.1246	157.7375	97.60876	127.3353	496.8002
CHTF8	89.36349	78.25553	61.56718	87.81946	82.79985	84.80639	57.76413	108.7539	66.77799
TCP10L2	59.52249	39.51152	38.52735	42.90221	51.85128	48.42229	43.01435	54.27894	89.22766
MARK1	77.25361	85.02888	284.9948	720.4413	590.1313	586.6758	612.7294	327.476	189.0103
FAM96B	3768.338	6377.973	9794.842	4575.95	10458.95	5908.539	9309.702	8173.708	725.0306
EDEM1	231.0877	149.9156	248.6329	709.5833	81.42611	126.3733	133.6751	88.22013	344.6169
ARL6IP4	65.07091	53.90712	34.48794	38.77568	43.75714	40.44351	43.78414	40.03463	45.78249
CARD6	574.2941	449.2357	536.4623	786.7958	373.6109	958.0436	746.7319	253.6551	757.3829
HSD17B6	235.6855	194.3269	77.49261	1571.295	54.77123	95.79266	128.9337	54.17539	104.8486
KLF4	1655.592	1494.811	1194.786	1084.313	1148.186	895.335	609.2877	945.4681	2117.052
FAM47A	78.0583	75.88138	58.40022	116.7597	56.2186	52.83335	42.80113	60.99327	139.0754
THY1	84.9335	386.5378	460.6736	52.2289	430.5597	808.9955	1074.511	654.2191	773.0487
ANKRD43	93.48965	75.58511	79.59413	152.2229	64.15715	73.68897	61.87147	56.93067	133.5002
TBC1D14	78.16424	73.77151	210.7554	1359.875	686.0869	459.5894	541.2982	677.2764	87.41991
NPR1	380.1945	245.2624	104.7416	171.9303	91.79375	121.8025	93.0892	109.7437	171.673
INPP5F	130.7392	80.13782	80.57274	124.4597	73.89297	124.5945	75.39794	81.06456	291.315
ANKRD7	58.08477	46.78887	63.33889	50.43279	45.65739	53.16849	50.87531	61.07001	97.59075
AK1	61.40754	66.09245	54.55306	50.3344	37.08552	53.35546	45.33039	60.26851	78.32689
GLI1	60.29157	47.84496	43.25021	66.78118	548.7597	64.04124	118.6605	46.30624	156.3426
OR4C3	57.29187	102.4211	66.37525	50.26184	49.73018	67.88944	44.47694	46.01748	44.48323
RENBP	1664.551	36.95695	548.9094	35.89622	829.7192	515.7208	834.8732	761.119	40.51697
FCN3	13992.34	12203.64	9444.187	13612.85	8412.277	12440.77	9754.377	13616.84	15863.42
ZNF781	42.55595	51.65993	167.3174	34.48218	33.3705	45.46004	35.83633	35.46014	42.33804
PELI2	32.09045	63.27607	36.3635	42.32769	48.36477	42.7701	37.76917	40.68323	38.75088
TMEM66	43.85867	800.7502	3413.542	1567.629	7081.527	3072.872	5754.098	4105.034	39.47586
QTRT1	44.57303	341.8752	182.9705	70.71938	43.1575	68.83442	95.5863	249.4858	81.26802
APOD	1490.455	784.9341	485.1485	40.47603	959.2263	747.525	329.3121	446.8011	1917.117
C20orf195	41.07579	33.88342	56.9681	66.80422	119.4098	43.44452	269.8772	62.59672	39.12548
B3GALNT2	789.6976	154.8521	307.3214	62.57511	242.6203	228.5213	302.7132	233.2332	59.60162

PSMC5	3602.991	5297.255	5439.084	5680.212	5690.209	7042.836	7663.707	6134.913	4031.899
TBC1D24	1440.88	34.35604	45.18575	45.84287	44.57753	54.09221	50.68419	49.96026	48.20829
SLC6A7	213.136	153.1332	79.21757	135.5629	69.1055	94.30166	62.96797	64.54018	117.6895
PMM1	5921.667	6098.885	10338.36	6606.276	12230.37	8252.255	10402.23	10772.87	4744.243
C1orf56	92.42358	68.40212	47.50274	238.5786	47.37642	52.06496	48.87626	51.07117	68.06155
C20orf201	31.44337	33.30837	218.27	39.30167	45.08454	47.58271	108.2521	37.95243	45.09159
FAM86A	1406.995	620.5747	692.4858	1232.997	1815.403	700.704	1416.095	852.7077	225.6728
LOC92154	39.14896	35.11113	43.20618	38.92531	35.63517	44.3521	33.22456	33.89533	51.2317
DYNC1H1	366.5826	778.5673	486.6107	380.6634	664.3549	152.6905	557.8168	483.353	75.72708
DDHD1	50.48772	40.39212	105.3531	63.27073	95.62276	186.5395	85.52716	120.5886	40.30312
PUSL1	46.96464	35.91233	32.3141	37.28522	80.1334	33.96348	34.18732	37.00745	41.83939
KRTAP4-1	39.00052	35.85769	41.27504	44.94905	34.37432	40.98472	37.20508	35.30445	36.93356
KCNJ2	41.58224	252.9826	161.6042	42.63964	313.7475	334.706	289.2478	172.7141	42.07824
C20orf132	193.1465	167.3384	90.25282	196.9623	77.11746	104.2514	75.31976	95.15411	352.1188
PRRT1	261.8461	192.6573	101.5074	178.0673	86.23409	107.6292	79.32254	105.8849	364.8903
GPR18	45.39006	43.87971	44.00983	53.20251	43.16208	40.78112	48.85559	48.5538	46.62758
TRAF3IP3	34.79179	35.22658	234.1784	35.19678	415.4355	44.18646	204.3445	604.1733	32.25684
C21orf86	417.5214	241.8403	116.2129	90.0387	87.86007	161.0726	106.6293	152.8656	230.886
KRTAP13-3	41.94572	70.33981	35.33625	42.33835	42.02389	41.35246	51.61516	52.10538	47.06676
SLC25A21	47.24072	34.33346	34.21847	38.6195	37.72002	48.13046	99.60451	47.12318	83.8309
KRTAP12-4	59.49097	92.19077	55.93427	47.25899	58.49212	65.57587	49.1417	93.74308	113.5559
ARL3	104.1574	63.79137	35.09826	50.49643	48.57472	533.0204	405.6383	51.13223	193.5699
C9orf41	43.73008	459.3483	332.5933	493.021	280.3752	571.9009	161.1342	286.2306	896.701
CLEC5A	3701.745	2719.214	1654.76	3286.235	724.3799	2773.507	1536.085	1896.175	1885.109
ACHE	150.4337	50.57694	41.81567	69.53264	46.76335	39.36862	42.77404	37.39785	350.1033
C19orf26	248.1296	108.026	84.54618	94.97126	71.84283	139.2472	51.87984	83.14159	280.2657
MSTN	59.66339	48.484	36.97418	73.85114	35.34449	43.73872	47.26282	66.20424	41.50409
RNASE6	39.18166	851.9288	366.1608	564.2089	405.6976	754.2236	362.9717	373.3263	54.45849
OSM	90.60298	47.48122	227.7482	49.31295	128.513	62.2171	302.3362	238.3709	214.9615
MYO18A	10131.74	9986.868	4646.51	9052.24	2780.281	5024.135	2880.946	4961.695	11072.95
CRP	194.2796	120.6818	137.1148	72.62914	219.6545	69.6203	412.2318	478.2718	158.9316
CD83	435.919	247.2152	246.4196	274.9765	239.0609	198.2607	207.691	166.6065	556.8994
PIAS1	769.7698	2370.248	2062.153	1389.423	1320.019	2317.42	821.502	658.9476	3957.539
EMR1	43.13393	56.21763	42.80924	48.10806	37.66628	48.16979	32.77975	142.7221	38.10159
TMEM192	351.3145	149.3654	438.0983	193.8161	162.3976	212.5122	540.7726	365.4301	263.2431
ACCSL	100.3754	151.3124	74.86152	169.7591	88.99399	120.8577	75.27804	77.62232	407.5245
KPRP	68.35628	1745.335	2311.862	90.75305	16767.08	1013.83	2199.307	3960.727	57.0434
KLHL30	38.18454	54.66095	165.5001	95.29424	44.48432	278.9146	189.8955	698.6083	38.74924
WDR51A	5689.757	5853.172	4864.875	8514.51	3840.679	5111.855	5022.736	3518.469	7397.543
PLEKHG3	5019.236	4717.709	4705.208	6389.861	3388.306	6345.85	4958.457	3929.625	4215.732
HSD3B1	70.87498	70.30132	46.38123	61.97992	45.75337	113.1471	41.39	54.00697	79.67191
ZFYVE19	358.0568	888.5449	591.3976	583.5265	670.1367	317.954	895.2303	1168.681	311.2919
THAP11	681.3035	952.5626	1618.617	458.881	2477.866	1288.219	1494.23	2017.923	909.6283
RBM23	415.854	2363.056	3287.649	1921.321	4457.653	2754.021	2971.851	3628.301	550.9622
POLR1A	189.924	122.051	277.7845	84.81535	515.6658	84.00407	399.3582	202.7999	2528.966
OR9Q2	56.32063	57.21835	43.3411	49.283	39.41533	66.37066	42.52147	56.93855	1276.966
TUBB2C	166.1347	368.7964	128.7639	266.7773	97.48435	87.90842	94.27987	120.0472	110.2657

ASAH2	68.24786	49.31965	68.10161	54.52127	58.1212	51.30365	46.31667	60.5296	67.22721
DPPA2	86.26611	112.8538	103.8286	167.1232	64.23978	86.34053	59.08659	58.58785	131.8311
ENPP2	1338.875	438.1837	1051.452	91.22481	493.7922	528.7961	300.0137	285.5219	114.628
ANKRD36B	37.51369	334.9052	43.77358	65.91267	127.3483	60.05902	95.799	90.197	37.24095
PCTK2	378.9377	231.334	105.8914	302.333	140.6946	200.1172	98.27865	97.11657	548.4391
RRAS	1898.862	2407.285	4567.75	4717.355	4963.546	5019.439	6048.335	5630.282	1008.849
EPS8L2	68.17249	58.17176	140.6499	459.9146	57.89385	276.3804	64.23654	53.88967	1478.312
SLC6A12	96.44237	114.3831	79.11417	124.163	69.20409	96.5883	70.03181	57.23723	160.8421
C2orf16	73.78403	118.7558	147.3383	53.52497	46.48023	50.81208	111.9169	58.66519	116.9774
RANBP6	878.1807	593.8322	74.2392	105.1287	76.62798	391.6102	110.3089	70.08047	106.4031
PRKCH	1898.65	250.1272	1443.372	960.8742	2313.488	596.1083	1142.446	648.7365	616.3981
PPP2R5C	44.13592	712.8946	294.8546	456.9796	97.55838	251.3927	151.188	241.27	1210.611
SLC43A1	235.432	86.15655	94.50704	72.57071	92.49158	167.4828	86.96629	128.6106	129.0904
MUS81	668.3634	944.3164	1306.771	538.0621	1476.987	1361.131	2047.672	891.1765	980.0012
OR5AP2	90.70913	53.741	55.48462	68.10675	52.34618	75.95226	54.6856	53.41619	49.6496
RFXANK	54.95323	42.35951	41.14808	41.33267	45.14681	44.76907	41.69242	182.0837	88.1671
DND1	38.6493	52.21908	39.23018	48.56291	39.81868	40.1252	36.46545	42.02364	40.46102
ETAA1	441.0471	62.94745	90.37823	233.3683	49.14923	146.1893	85.66136	101.68	70.40006
SOCS3	68.4727	211.475	147.4645	89.26405	37.56482	75.05724	102.8473	58.17703	935.2283
ARHGEF3	1261.801	2101.077	8627.327	3980.724	8317.772	6377.605	8370.574	8052.82	2176.095
CPN2	467.0534	259.5585	132.9842	276.0396	108.136	191.9066	136.2738	162.1731	616.3639
OR6K3	71.49056	109.7213	61.21897	70.84466	64.01925	70.1597	59.87674	76.31702	88.48065
TSNARE1	1277.73	504.4935	432.2155	488.8184	1213.638	819.1932	347.3985	1053.488	1374.018
ZMYM5	42.85223	339.3521	196.5841	41.18897	105.4055	285.6114	129.6895	183.5291	892.5904
VPS26	47.24479	310.3836	836.9453	320.0323	1535.188	526.8911	1324.734	1021.221	87.57299
IFNW1	230.9774	134.8864	84.47107	171.6638	96.25297	124.226	63.47222	99.13213	237.0038
PTPN5	164.2959	67.06994	78.828	117.4277	89.25237	114.6822	64.2987	80.27879	335.7269
CEP120	533.8246	246.7313	259.6938	341.1554	93.36841	186.4958	107.1025	106.2752	733.72
BRDG1	842.5783	1834.592	2412.994	676.1981	783.6802	1586.472	1075.192	627.7042	1495.537
UBXN4	5834.524	8238.441	9545.076	7358.138	10554.01	11704.82	10223	7874.379	4778.196
C14orf138	37.77944	838.1341	39.62568	41.36285	529.1488	36.95897	165.3891	520.7917	59.47252
MAP2K7	84.46355	94.85053	69.24858	111.9853	58.53097	77.02515	48.79233	62.4759	128.3028
TWISTNB	2157.872	876.2554	939.2891	1138.802	916.3659	992.6352	830.9639	701.4692	1275.17
STAM2	62.65577	51.61456	195.9635	55.06636	211.2372	62.0477	154.5067	171.2196	117.8883
GPRC5D	57.25966	42.87984	44.03186	52.5486	47.00367	55.88607	46.4429	37.45437	64.82652
ASB10	1651.895	4254.779	1165.285	1006.076	130.7648	1665.344	1311.771	3389.665	3795.929
INMT	1704.956	2399.606	691.5056	40.66765	1916.99	465.6455	1446.596	3060.262	50.57786
LYN	84.43405	799.8895	4898.757	3786.837	2813.649	1468.115	5822.397	2610.748	98.70728
KIAA1009	51.91832	48.79085	50.75587	93.7192	63.65403	57.9465	323.1293	73.70274	73.92516
MYBPC3	123.8319	119.7104	73.29141	407.339	68.21208	89.12834	104.0743	126.2451	192.9056
PPP1R3D	1203.813	50.98094	212.0019	44.19172	281.5265	57.12306	155.6569	349.8315	47.30339
FLJ35801	66.82206	44.61863	53.52404	47.73406	47.45869	48.40614	54.67518	42.70109	53.60875
SLC25A38	51.07948	36.27995	521.6227	2025.931	474.4638	35.43005	1303.116	174.9263	49.89642
CCDC22	53.16544	41.13333	48.95549	67.84184	66.03862	51.03063	44.92226	36.73117	64.99879
IGLL3	327.8876	187.0629	100.5382	131.3263	108.4922	136.5396	88.70191	117.8347	456.4929
LEFTY2	96.38331	129.3701	67.02388	75.05391	111.6502	204.8782	51.26394	47.91634	227.8003
GH1	55.28956	49.00089	48.15302	59.96157	45.61935	58.32342	50.38918	63.1681	93.25918

TMCO4	186.3536	1190.563	676.612	212.3072	648.8931	1182.85	408.888	629.6154	201.7257
SRD5A2L2	49.75771	86.52874	63.13432	90.01499	50.38509	55.15746	102.2786	44.01341	105.1292
EPPB9	41.53668	66.47315	38.74384	57.97088	256.4814	48.29605	35.94209	505.8268	62.6249
DUSP1	86.55241	1520.847	3265.111	2051.579	4376.235	530.3912	1487.431	1428.517	383.1578
RHOA	2091.969	3136.787	7869.833	2825.685	10222.54	6067.579	9251.044	7727.921	757.5491
RAD52	49.44643	694.3501	841.5214	35.40881	569.3497	46.27362	778.4096	832.9222	2565.692
MPHOSPH(	1029.515	532.9091	356.0587	814.9293	218.6742	356.7996	257.0995	264.3323	1517.858
IFIH1	142.5864	281.7218	289.4866	75.42812	108.3626	651.9542	827.0125	346.7107	159.614
TUFT1	53.53834	2987.352	4638.735	2691.24	8664.297	4901.917	7702.158	5954.362	85.65903
NFKBIL2	420.0667	250.1845	65.07867	261.7787	54.70462	141.587	76.68904	76.55666	559.2915
WIPI1	1118.133	70.61922	713.4806	916.6669	2364.886	1571.149	1530.671	1401.626	42.38554
PGLYRP4	1250.43	744.9099	287.026	570.0479	357.0983	1120.081	678.7984	394.3619	802.3894
FGF17	186.3752	146.7472	83.29927	145.6485	704.8485	109.8883	218.1732	104.1196	255.0333
GLCCI1	68.25323	599.6637	469.6566	64.25909	511.1331	68.35829	574.4	57.87702	87.18161
TIPRL	213.0188	140.4815	75.82972	140.5823	83.63573	524.1663	206.5636	162.7995	326.289
OPTC	58.62403	65.34415	47.76046	59.63934	58.20871	55.66573	48.82246	68.618	153.6477
SMAP2	248.4855	634.4581	315.112	118.3008	116.8868	87.31849	91.56246	88.51295	304.9453
CDC42BPA	374.8778	602.7825	602.3824	797.1317	1612.988	747.6233	1996.202	2900.489	351.8477
SUV39H1	5360.328	4997.947	4095.759	5680.204	2509.729	4446.929	3706.142	5770.938	5109.221
PCDH17	7964.612	7349.764	4730.646	8070.82	1320.001	3499.835	5014.919	5044.728	8939.85
SUPT16H	70.94226	440.9378	453.9539	78.16611	1459.447	469.1523	1069.498	872.0586	43.43668
LIPF	119.7771	120.054	67.93666	105.2094	85.69126	94.11168	80.49529	97.25075	178.7129
FAH	53.23605	990.8822	53.94751	275.1082	234.766	283.27	258.436	138.5949	95.12972
KDM1B	903.7131	348.178	291.1737	373.0681	257.8816	385.1588	144.896	248.0994	901.8448
ST3GAL6	709.2355	1611.319	1148.571	720.5945	999.1594	2766.04	1149.421	1487.558	726.0238
MESDC1	33.3802	42.23599	34.63277	42.89913	46.73069	38.33062	41.70609	42.14556	45.35287
BRI3	632.9106	264.6503	251.9615	394.2597	71.18362	46.18229	214.1998	293.1166	38.5705
TEX264	39.62222	37.45092	48.86554	45.76319	40.89904	56.89936	187.2316	325.9319	33.61255
SURF2	147.3126	100.6381	177.516	132.083	115.2704	263.2353	120.0964	205.5423	166.0024
VWA1	221.4151	127.8027	236.6405	1298.406	299.9904	91.05237	337.4373	98.87998	223.632
HDDC3	3652.402	656.8112	1763.396	864.3601	1382.091	1159.807	1391.942	2660.739	2348.605
SCGB1D1	90.52435	42.65605	50.39635	48.21881	45.85515	36.68395	40.43193	34.92391	40.54353
PTH	35.48414	44.50022	41.26534	45.03428	43.43639	48.88271	48.9796	45.5685	50.60825
DAP3	77.49012	37.17134	510.7415	43.42178	1379.238	50.5589	1023.551	1214.238	57.7944
TRAK2	2610.55	756.0807	964.5355	34.09474	1569.116	1112.612	2010.476	1703.645	2501.365
IL18R1	1323.134	1285.227	791.0686	82.11317	65.30006	271.7029	452.5398	429.2684	1857.54
ZNF720	44.91455	35.63292	49.60084	48.22089	48.75665	36.46012	41.98613	43.94086	59.38877
FAM133A	41.77534	48.60819	402.8583	79.70205	187.2558	41.74604	325.4909	44.93072	48.33965
C9orf152	115.3045	81.89354	49.54708	48.86516	397.4519	184.3279	106.3795	74.49171	133.6672
CLEC16A	7880.341	6282.439	8258.635	8318.541	8807.319	6958.95	7466.955	6386.602	8831.786
ENPP4	38.01082	258.8075	39.6184	43.81432	137.0749	237.3177	180.8276	76.80135	69.74965
HNRNPH2	5060.371	5625.522	4141.927	5379.89	2452.296	3617.451	2932.321	3098.173	5435.489
C2orf27B	50.14882	41.89614	44.50648	41.86484	35.76895	41.04365	47.39529	37.80035	34.58591
PRRG1	37.76402	64.51484	457.9176	55.25878	456.7166	54.96391	353.5819	77.29478	71.4594
ERH	259.7999	540.7668	251.2566	247.3362	371.7993	195.2638	353.8825	356.6157	832.7902
FIZ1	10334.83	8758.371	5184.12	7241.438	3561.979	6438.882	3676.765	4047.441	10551.67
CHD6	296.4161	69.55097	86.35889	114.7105	118.7621	133.4745	75.83177	154.7972	226.9839

PAPSS1	305.5834	1430.37	1046.572	926.9671	1087.347	812.7267	414.7073	835.8214	1942.333
FASLG	59.52595	48.87516	107.1821	64.0813	57.76587	180.1917	50.74041	39.08777	84.76901
UGT2B28	102.1588	56.7306	43.12811	48.18843	41.75163	39.15059	37.98755	61.24413	44.87508
RBM8A	643.1448	370.0584	93.70518	411.427	56.71087	106.3574	84.5144	136.8754	607.3392
KRT12	189.4981	133.9874	75.5274	115.0693	62.51514	92.30042	63.16227	52.65828	334.7508
LOC44117‹	70.78712	65.91839	50.72894	77.85922	54.84893	46.69978	45.54061	41.14659	123.1656
RBM17	38.65656	3496.196	5623.037	3395.188	6950.196	5017.086	5870.2	5451.606	40.22914
HMMR	84.98435	57.10683	63.64135	52.46809	53.44218	59.91113	56.23225	46.31546	53.20832
FAM83D	35.26538	60.42346	183.5873	46.83274	179.7758	41.63259	81.58702	33.14572	36.17825
SCN8A	34.5347	43.77584	37.22175	35.54881	35.53291	35.12532	32.52547	40.76586	43.36035
OSBP2	114.1951	1341.126	454.2528	1401.771	603.951	750.5034	436.9307	606.1716	61.96032
AP1B1	6452.249	4971.779	5271.543	4122.798	5288.868	4915.582	4917.664	5236.011	5196.381
UCRC	1235.296	148.1879	251.2177	364.9918	868.9427	97.79199	427.4546	593.1335	232.2111
CDK5	85.36785	52.08513	56.27687	79.24055	139.2849	65.3665	66.56271	48.79616	59.99425
NUDT16	48.61152	40.70614	297.6876	40.35627	954.8928	52.88108	1146.206	358.9812	43.74272
BAPX1	38.67248	38.48129	256.2083	89.84055	283.0046	1713.165	720.8187	595.6605	82.8818
FCRLB	825.2451	1089.754	196.4309	60.00318	488.837	753.3189	794.0016	346.4225	56.20337
ZNF707	146.5323	389.5027	260.5231	382.099	156.3001	54.81117	201.4274	215.684	171.0002
ASH1L	39.15532	55.70369	104.219	39.28839	50.7281	49.96644	37.30547	36.28	36.96571
ZXDA	69.04625	928.9501	1216.397	651.4599	664.7054	1254.496	999.6904	395.6694	96.63663
FKBP4	1539.216	1606.759	3097.109	2152.55	3242.511	2902.277	1625.125	4233.671	1202.156
CEBPD	1461.044	1747.054	2295.773	1576.186	3020.46	2274.473	2372.005	1510.795	2083.23
ATP8B2	45.32359	281.0697	192.0065	35.31443	178.5531	189.5899	90.36642	114.3117	46.16714
TRAK1	158.8396	495.2242	1635.615	1366.641	2422.148	1660.009	2026.348	1345.154	182.0005
NPL	1080.341	2463.166	1826.822	69.2859	1324.447	3003.223	1901.095	2972.363	1533.442
ZFP3	154.9208	70.5159	1604.698	510.4353	2679.256	1362.464	1656.819	1205.085	49.10668
ATPBD4	78.17173	375.333	137.6303	60.75673	104.9427	42.56272	76.3508	69.13689	80.712
AOC3	550.4232	337.0832	182.6168	262.0003	260.2863	257.5746	183.31	323.9081	593.1058
PAIP2	438.9673	266.1166	167.0182	502.9785	187.3671	218.3965	192.5964	313.4793	553.2598
C20orf55	332.3811	165.9536	791.5882	244.9349	1510.795	1255.529	1302.96	2168.341	408.9566
SMO	131.3414	102.1306	109.0703	115.7688	199.3113	176.2306	95.58479	130.1806	320.42
ZNF295	67.17767	43.53504	53.11568	45.85756	36.46159	46.8732	32.35618	40.29523	51.69019
ZBED4	37.6791	50.94509	269.0381	43.30801	528.684	462.1337	429.6893	347.1216	1466.107
AFP	115.7083	59.66879	56.68594	61.86787	56.11753	51.53088	56.4793	46.10151	42.14559
KIAA1033	49.69254	304.1881	123.102	47.97867	219.803	157.1604	203.9922	120.7386	32.64075
ASZ1	59.25208	45.55428	32.57711	37.16994	32.71583	54.2483	34.72234	40.84224	54.01966
KAT2A	3046.946	4046.51	3067.379	3631.764	3396.136	2810.143	2889.186	2680.746	3747.943
N4BP2	502.1904	345.5287	136.3813	264.0998	98.38702	138.0538	107.9234	96.68022	659.3038
NYX	249.7295	174.2007	97.52798	169.4798	75.86335	109.9939	71.55241	85.90655	146.5046
ZNF382	69.00013	286.864	46.28877	41.71323	33.87701	35.56117	51.42282	104.605	76.86372
LAMP1	862.3091	1963.094	378.177	738.2552	1026.679	1171.381	631.3522	1075.528	500.1976
TRPC4	134.491	491.72	150.7616	116.7279	152.8442	121.0295	133.1472	70.09496	215.2225
NR4A2	699.8007	1021.782	861.6353	503.6105	1347.185	1035.821	539.4321	540.4858	1328.945
HIST1H4L	555.1991	34.34166	143.4037	40.11489	139.1044	48.2764	80.70345	41.39183	56.10798
VENTX	1545.965	761.7941	1268.453	657.7661	517.6819	454.6028	1069.214	1191.165	2834.264
LOC44035‹	85.60876	526.1043	3270.275	1556.396	5765.178	2008.565	3125.451	3202.709	2541.134
FLJ38723	46.78167	40.86986	47.32611	49.43058	34.86218	43.18864	108.8651	40.94415	123.7401

POLR2A	49.01213	1615.995	2095.832	1042.005	2426.432	2130.825	3140.828	2546.427	167.9458
PRR11	97.69748	70.02636	58.71264	535.4963	58.99771	43.76231	201.596	57.99213	64.98836
IRX2	82.64274	68.81957	1209.129	2118.961	1365.535	83.2715	3953.846	696.789	39.07903
GNG11	584.9515	446.9725	226.5343	357.6665	234.0225	248.1713	333.5258	266.9571	827.9626
TMEM56	38.83183	33.63452	35.72068	32.14801	34.26857	45.57038	129.9659	40.58281	36.1988
CNN3	10092.56	11099.67	12970.97	10645.32	17894	13897.17	15646.26	12286.77	6894.684
CDKL2	56.25964	41.52225	51.86868	42.62683	46.61674	56.11394	36.4659	35.94184	78.13702
YIPF4	1249.361	915.7819	907.3787	859.4344	1025.88	510.5647	755.0051	943.4641	98.23354
OSBPL8	788.2479	1185.804	2625.039	2381.971	2881.427	3536.56	3483.801	2034.791	934.4796
RNASEH2B	4829.561	6671.863	8366.183	7899.024	6196.315	8420.837	6902.96	8585.97	7159.973
MTERFD1	1718.048	1461.185	592.0433	37.83243	387.2698	933.3342	811.9297	1120.276	36.01608
RG9MTD3	1677.32	1942.214	604.2143	169.4701	115.3068	159.5792	208.4003	111.1794	216.2908
VIM	5430.067	5668.943	4468.539	5111.334	4606.879	2498.932	3131.58	3273.16	4779.177
SLC2A4	122.038	95.37373	84.14461	112.953	84.12054	106.8927	69.18226	65.43891	243.9104
E2F5	148.7153	110.3256	203.5178	69.93913	62.18864	110.7715	84.81532	222.3893	81.467
XG	44.15844	35.58044	53.08349	39.94114	87.23811	31.02408	38.76581	32.92035	40.078
GUCY1B3	43.24323	33.01712	33.50883	40.81872	41.72738	44.44845	205.8381	47.58924	51.35514
IMMT	1188.793	68.41753	2412.685	2117.244	3111.572	1899.741	2399.476	2269.66	1568.154
DUSP7	106.9238	68.73916	63.68412	74.10509	587.239	46.3924	332.4832	36.95866	37.47025
STAG2	38.6084	46.96875	39.22698	64.28289	42.32356	235.1535	70.18174	45.67962	47.36201
C9orf16	41.81636	43.8043	48.62156	1338.688	1027.575	38.78059	1660.306	1376.914	55.10125
RPS12	4607.31	4789.04	5393.607	4514.977	6431.933	2731.889	5795.817	3432.502	6997.771
BOLA2	65.03267	656.2923	119.4497	529.3661	149.1266	33.90866	175.3978	501.9143	107.088
YARS2	954.02	33.32532	216.7071	532.536	140.2495	226.4569	304.5811	140.9317	38.76238
NLRP14	50.18466	53.24969	48.67407	38.20125	36.76927	34.96465	34.99667	41.4188	37.40694
AHRR	47.24893	34.88138	40.59493	31.63376	45.36913	44.79267	104.8925	45.57865	121.6568
THNSL1	47.51581	73.60408	35.69395	36.59784	46.41263	45.46025	43.49702	41.28965	45.52119
LOC14962(	210.6586	137.6336	92.90121	119.3562	68.56642	119.2722	71.62389	70.83848	307.4763
CD19	84.24252	1103.616	2664.409	50.63008	950.2714	1317.965	1807.974	1735.521	83.24543
SERPINB4	63.78466	643.0313	1193.203	103.2469	2432.437	1929.184	703.8277	2462.224	91.72548
CLECL1	34.94059	34.79128	248.1112	42.52745	112.6491	54.31356	95.4639	35.81639	34.90005
CD300LB	41.62872	58.34334	309.8099	35.7592	44.10086	546.6283	231.8326	47.79334	74.11227
FMNL1	473.9736	278.8985	123.192	215.8277	101.4234	146.9605	93.45847	112.255	661.5862
FLRT3	151.9853	82.4887	57.09463	259.5536	112.7377	108.7744	105.7164	122.2689	177.4454
GLI4	39.24906	36.68566	40.25478	34.50846	44.14791	45.363	115.3455	58.19643	37.76276
PAGE4	84.24335	67.04536	58.32048	59.49288	56.83766	56.43542	54.77516	68.40615	49.63558
ARHGAP20	53.46769	104.8597	147.679	47.61507	110.8139	52.00157	54.46018	49.02127	70.55455
CSDA	4034.72	10310.57	14363.63	10917.58	20417.91	12711.1	25769.61	19912.66	8233.081
SMPD4	302.8509	158.7483	69.37652	281.095	74.1101	105.794	79.02213	78.72781	406.0623
MDFI	301.685	191.4295	95.4328	199.669	86.67184	160.7273	69.92034	115.7499	446.3103
ARPM1	164.2596	66.33225	130.1216	298.2478	360.3902	369.0923	263.3114	442.7425	78.7946
DHFR	61.95874	408.2637	311.0342	740.9332	528.0812	588.5884	403.4749	929.1725	1727.84
GLTSCR1	97.6169	504.4219	1528.061	612.8126	1709.737	1930.253	1625.307	1244.053	125.8914
C10orf82	524.5582	42.71651	38.81124	41.72093	58.2961	39.63081	36.58183	44.79092	47.02924
FAM83B	66.09262	52.76852	139.972	42.69899	264.338	53.57623	152.1673	108.0984	50.25089
DLEU7	53.20841	57.6477	46.92666	46.70715	72.74787	64.25329	42.07764	65.65407	88.40809
PDE4B	229.9914	128.0648	253.6255	60.67931	65.71303	155.8531	210.7129	556.195	263.386

SLC16A10	5462.819	4672.765	7017.104	7930.688	2337.675	4382.394	4097.735	6601.271	5244.902
GPR37L1	2648.705	1393.858	541.0634	1053.097	759.6237	1241.13	617.8156	1303.578	1157.763
HIP2	87.6637	57.75649	99.45783	70.48991	97.14621	366.0048	171.0623	204.6899	50.97583
PDE10A	55.25317	42.62981	109.3206	304.0553	91.79858	39.56304	65.74833	49.94583	69.3427
UBA52	9097.334	11812.87	29958.21	15814.85	34485.42	24434.64	35729.87	35248.33	8571.259
GALNT8	251.9721	113.7987	78.0779	176.2635	80.96629	110.3548	73.14007	102.7102	178.3022
DHODH	53.9491	40.05028	34.22778	39.41536	33.88402	31.80802	35.08287	120.1517	32.78107
C5orf51	56.92186	40.40947	182.9302	66.5623	215.6343	276.4836	360.3763	169.6362	80.92187
C6orf167	462.6881	255.9555	160.1922	278.375	175.005	213.233	162.0652	226.4259	583.7844
SLMAP	39.94465	553.243	1613.844	432.2146	2244.749	1786.709	2313.112	1724	66.30159
SAMD13	49.68057	88.65996	58.15044	59.95964	38.0927	37.37473	52.29467	49.85668	41.36818
RPS4Y1	471.8403	264.2385	140.2448	293.661	142.0108	200.0752	533.9424	1156.117	622.9921
LY6G6F	148.6126	75.77869	60.29056	96.71213	53.18998	111.8663	66.75753	73.95821	149.3335
FBN1	2288.476	1097.681	998.7134	860.5883	628.0899	1344.285	826.9702	1181.794	1225.91
SOX21	67.20628	429.2426	132.8685	282.6272	1623.204	859.3372	782.8593	578.14	897.5308
OR51F2	47.61706	37.95381	44.23485	34.95558	35.43105	41.6696	35.21564	58.81028	40.17235
SSR2	901.7991	62.03711	615.8017	3019.286	707.8336	812.1757	879.1413	510.8815	2339.852
PLEKHB1	375.0123	193.5998	439.2939	230.9978	209.8347	194.4203	175.2749	352.0434	395.2692
C10orf11	1087.157	167.5621	179.5929	185.3098	69.38985	135.7551	75.302	107.4116	314.9512
NEXN	114.6473	540.334	532.2434	346.928	280.5186	462.2201	626.8693	651.4702	131.526
CTPS	66.76986	67.12088	55.55399	76.22266	55.59174	45.28209	34.37354	44.04355	61.17977
S100A14	1080.934	698.1393	596.2266	756.1448	451.6886	530.661	457.9399	736.5684	1591.179
HSP90AB4I	317.5092	71.76434	59.31629	203.1773	35.55629	75.21512	94.05922	47.17143	61.03896
ZACN	60.04939	57.61201	47.44164	39.05009	50.81307	42.57745	39.20166	38.54718	59.74526
C16orf61	6834.365	7020.32	8329.634	8274.223	8808.025	10395.95	8255.146	7385.018	7358.828
NPFFR1	57.93744	43.66007	36.46665	47.06077	42.2338	39.69644	46.38895	47.25705	85.78415
DECR2	50.80529	49.5693	46.95687	74.94799	224.0047	40.46089	196.3925	167.8804	68.47897
DCDC1	44.71439	33.95399	38.23639	33.5724	46.07845	51.62221	52.03469	45.11237	69.18092
BTN2A1	42.73514	36.87117	108.5553	36.97201	43.52558	38.41325	58.95763	37.59215	64.65185
PUM1	2166.898	2199.105	1583.435	2641.691	3810.85	2003.636	3050.242	3207.987	34.80386
DVL1	50.94651	46.32013	55.08265	74.34391	212.3209	49.54092	35.98563	38.66494	65.70749
DYNC1LI2	10414.59	11958.26	14142.45	12181.93	14884.11	13468.69	13818.29	14523.82	12663.4
LOC642197	138.0984	90.73711	49.01049	134.8319	52.64568	72.17435	120.062	47.87436	52.48681
HOXD12	49.05574	48.61999	39.93446	58.21668	37.38834	46.6507	38.98819	48.19345	52.51019
CARKD	243.7651	100.7998	554.0257	148.7677	646.3683	121.4604	435.178	240.4801	431.8549
TBX19	41.0625	451.4196	65.27348	100.4097	47.82958	44.25127	42.3041	58.12885	146.1299
LMAN1L	490.2222	158.2252	133.7668	140.155	281.6139	210.586	129.2871	176.6873	583.5833
TMEM17	32.74759	34.26107	37.99966	36.04743	35.94623	36.04449	47.98982	143.405	35.96643
CCL13	2318.534	2070.835	2757.484	180.0587	3113.585	4378.937	2523.439	2343.005	1885.799
MLL5	83.42505	1228.611	1089.238	49.72233	801.6459	47.98953	383.5595	422.034	60.01033
HELLS	458.3773	210.4074	126.9623	387.7701	168.1423	182.1792	129.4819	172.4494	444.5097
CMPK2	35.76074	44.09438	207.3813	43.58492	43.52134	43.34824	105.3688	44.71135	44.52548
COX6A1	4032.144	2985.035	1444.655	2687.029	3104.369	1289.413	2712.154	2953.853	5809.971
OR8K1	44.09971	39.26305	35.53439	38.17702	36.70205	42.39949	35.42043	39.33246	56.35101
C17orf38	92.44492	103.2914	68.55751	166.1145	58.22208	51.06898	54.50928	55.57347	73.28557
ODF4	92.40835	128.2088	253.9984	85.79441	58.07292	101.3644	70.01058	76.29897	159.5614
PCDHB12	57.13343	40.83165	361.3897	38.10505	435.3506	352.3006	240.7253	179.8556	37.61752

SLC30A10	248.277	145.4531	89.44093	125.1502	70.07259	88.80641	59.09945	70.0394	190.0772
ASB8	70.88933	121.2027	219.8271	109.4859	60.77696	293.4126	229.5408	128.0338	72.03299
CCDC15	1735.39	1938.222	3859.624	2620.609	2864.51	2730.982	3374.142	2779.835	55.88946
MRPL34	2481.164	714.2744	458.7634	229.6123	410.8513	336.2443	673.7739	291.8284	147.3419
C17orf82	3044.109	3136.851	753.68	3110.517	1216.368	1859.639	1443.332	2005.904	2820.667
EPRS	230.8884	276.3929	400.8087	330.995	753.713	581.2256	623.2504	347.055	531.7723
MORC3	40.22366	61.63418	44.74662	566.4454	41.8264	35.99745	122.1011	37.779	55.46547
CYTSB	700.3446	117.2679	129.7858	121.565	75.71551	189.55	85.48602	81.52781	280.9505
RRAGA	60.08414	529.1265	1872.712	688.5644	1995.294	880.9608	1554.595	1275.869	59.22196
LOC400506	40.92147	874.8206	288.6877	35.14672	165.3949	199.0306	397.5159	254.7973	42.72665
TBRG4	96.19057	77.36192	556.5995	46.5701	497.4176	46.39793	768.1647	530.6218	45.60151
FLJ41423	36.34954	33.53724	703.6928	1135.804	168.3483	1768.471	738.735	1626.458	51.90724
TXNDC12	37.39275	1991.349	3135.464	35.06895	3423.385	2449.596	3803.248	2132.847	55.62496
SLC22A8	40.15042	40.08219	45.42599	38.46278	35.07863	43.50248	34.31765	36.41529	36.66957
GAS8	107.796	66.58495	2588.046	745.7964	4366.659	1882.797	2388.693	1518.024	57.67212
PNMA3	2733.656	4350.054	4042.841	3330.905	1442.538	3317.108	2327.272	1591.979	3685.313
COG7	111.6396	119.1832	235.2666	92.20993	66.47079	292.7497	60.83564	242.6807	234.4826
GAGE6	56.29839	63.83079	125.9381	55.61604	48.8112	56.72511	48.17374	37.18354	43.98602
FLJ42986	46.71508	34.47735	33.99563	33.90948	34.61951	37.64557	61.53912	37.9889	68.35574
MIIP	98.07793	991.2147	1329.277	2061.664	584.3944	52.4436	354.7428	1095.134	316.433
FCHSD1	162.466	69.90413	57.42033	124.8592	77.16467	90.33397	58.16246	63.52769	96.74789
CD164	44.52908	509.4758	1270.459	1804.797	2427.501	1556.997	1892.979	1098.757	40.82244
ZIC5	143.5371	92.31714	65.25168	646.8745	312.0453	94.55844	251.3056	70.94314	247.2829
PRKAA1	69.59833	66.87264	459.1735	104.2323	654.0696	75.82716	584.5024	379.1973	402.8932
LUZP4	53.91977	40.11646	35.92471	52.83319	32.60955	35.41153	46.41807	50.72309	38.75471
B3GNT9	44.56483	258.4729	73.08663	96.20458	38.35883	168.484	90.1612	94.16512	814.067
MRC2	3151.147	43.4416	494.9226	1637.629	841.079	259.7409	491.4332	1622.342	2900.481
ATP8B4	171.5111	781.6558	802.8799	879.1682	579.0145	889.9826	568.7533	896.2279	891.6729
AGXT2	35.11915	40.22403	48.64114	44.61544	34.73158	40.03357	41.89491	42.95733	70.61784
FAM24A	47.95185	73.6597	45.68622	46.31357	48.57187	58.69588	50.46243	40.13449	36.61215
TMEM105	479.5575	181.2368	121.5537	501.2378	115.0413	165.9345	137.8905	190.8808	719.2498
DEM1	543.1415	248.9518	1774.106	811.8683	1163.195	1250.816	1426.54	1459.699	746.1179
STARD5	3853.19	3101.078	2095.58	3146.913	1273.948	4141.368	1949.032	3811.941	47.1961
LOC644615	42.05773	64.24388	49.23692	34.09108	58.76147	465.0813	285.7484	228.6622	42.30451
TIMM44	40.43669	62.91759	589.2777	39.62637	349.4689	489.7192	50.2969	342.2294	1412.14
CDH15	10645.95	14400.35	9316.892	11905.26	2542.547	10466.63	10062.08	12513.88	10227.95
ZBTB40	761.1312	683.1321	440.0391	361.7346	303.4995	185.1735	372.6623	364.1992	664.5694
NAPSA	74.43942	90.27271	59.42095	82.37438	60.93704	67.86101	54.20081	55.53903	40.67506
IGDCC4	41.45803	49.04665	120.8117	43.97273	35.14004	44.47	37.56911	66.53615	39.99603
VPS13A	42.97749	67.56967	104.8088	62.61235	90.68758	129.601	62.08117	93.50967	69.82538
SLC38A7	795.365	382.7008	560.4183	98.54778	1158.75	613.6469	1220.148	1417.292	243.2274
NAT14	368.0968	178.3348	157.8165	221.3811	496.8015	798.4999	649.1747	337.9146	1980.489
EPN2	88.22575	38.98377	39.18958	36.25513	37.26014	47.09318	36.94376	38.5342	36.33941
JAKMIP1	50.19024	49.52233	33.86623	42.77331	39.04366	101.4569	37.02458	34.12882	64.34815
FKTN	38.31324	43.55749	41.7311	38.61069	44.28927	43.66138	33.93969	31.22367	47.64943
IFT172	124.235	69.16704	62.49762	78.9637	55.62151	49.27619	49.88501	459.9332	109.5899
ACRBP	118.8401	57.45797	49.63654	63.47316	53.30833	54.2007	85.68034	75.12293	54.46949

C1orf128	38.53288	45.79003	938.5021	49.46655	1651.178	416.8541	965.3495	1862.849	145.239
KRTAP19-6	270.9865	209.5414	105.8797	217.077	81.22678	119.5705	83.47551	94.62115	347.9982
SOX15	5291.773	6859.266	8174.462	9418.385	10483.02	8763.91	12287.33	8946.705	4209.41
DDX23	77.48338	113.944	55.01855	672.9792	403.0704	403.3358	336.6869	246.7743	175.9779
TRIM43	186.3531	152.8339	84.83755	165.4966	67.93134	112.8628	66.04613	62.14944	136.0044
SEC22B	601.1845	418.7614	211.4292	371.8774	381.9152	301.9486	308.0541	213.7344	52.32769
HDGFRP3	5601.727	4765.547	3911.67	7159.57	3480.483	5033.585	4876.507	5959.473	4598.404
TNFSF13	109.1998	48.11371	55.18673	70.30318	58.24371	64.5146	44.02361	42.36993	70.70842
AASDH	71.41225	695.2068	1064.166	51.16891	1347.859	1133.182	1388.059	917.3543	177.7954
CD1E	690.0441	491.1087	142.882	179.4021	130.0304	140.4663	72.38893	92.91686	343.5069
NDUFA1	1810.522	2293.161	2123.866	2562.319	1604.645	1748.685	1588.933	2220.065	3714.587
TGFBR3	615.2536	243.4564	479.321	105.5232	1158.947	355.3872	571.7919	415.4869	66.63477
ANKMY2	614.4174	68.37138	105.3679	40.05548	193.3203	55.58271	164.6336	98.17543	170.3222
CPA4	51.86051	1052.98	820.305	1843.666	3185.041	387.0143	1181.217	1220.29	50.66294
C19orf43	7490.664	6946.581	2682.636	6929.092	1859.747	3354.142	2651.485	2262.547	7326.241
TUBA4A	13146.95	12408.88	11897.82	13378.86	13634.96	13175.62	11302.91	13224.37	12694.97
LIPJ	70.10674	75.54223	288.997	51.93344	60.70668	275.933	101.7145	77.73006	276.4352
SNORD25	681.7373	528.2723	198.528	940.508	51.5924	588.0129	244.3224	84.96843	48.16906
SECISBP2L	44.59671	442.5825	934.4349	504.5966	930.9063	1026.577	697.579	521.955	46.65034
TTLL11	49.18278	49.44809	80.5881	57.23858	54.11849	132.2753	110.8228	98.77306	36.27959
MYOG	222.515	242.9211	80.39059	139.764	59.51923	89.8033	64.09859	84.00836	342.8688
DTX3L	231.1877	577.3158	635.4379	501.5663	228.2997	208.0207	374.9334	409.6203	298.3195
C8orf48	667.0584	264.8083	515.8572	390.1247	424.8595	788.7603	493.9417	412.0856	511.2338
UBP1	2051.708	1721.526	3242.375	2940.722	3884.978	3361.873	3193.171	2440.764	1557.475
MYH7B	44.70633	89.84953	51.60459	54.78912	213.093	48.79003	610.8096	363.9483	56.28656
KIAA1688	14643.23	13312.98	9264.074	16278.99	9967.82	13356.03	10130.28	8175.845	16526.12
PLCE1	143.5499	96.91452	121.7432	88.5912	127.0787	84.93751	86.41434	103.3831	592.4338
MMP3	11208.95	2866.248	10925.13	8734.663	652.3148	11515.3	19907.1	4156.659	5909.202
NUP210	67.58262	55.72722	57.05788	65.53838	121.5232	66.91771	60.95102	60.74809	41.25061
LOC401286	1392.31	116.6125	50.88142	99.05309	52.45618	93.38817	46.21056	62.77977	80.16339
MYH6	6468.131	10768.37	3941.638	6861.935	1531.615	8191.997	6766.732	11455.8	9246.072
TTC18	58.43189	65.28474	53.28404	56.93828	44.02228	45.51007	46.57067	51.55861	197.5912
ATP6V1G1	2685.75	4490.918	7311.538	4367.115	7905.864	8989.006	7704.266	8557.316	2730.659
COL22A1	4080.096	948.0466	804.5204	1830.234	836.301	5211.981	1036.292	3652.156	42.71087
SELS	451.2674	358.1986	177.8553	325.8083	273.4967	255.282	188.3307	169.2764	282.3625
KBTBD2	4585.009	4155.591	5145.009	4533.389	5030.981	5964.617	5448.731	5331.883	5468.099
LOC728343	198.7626	74.09746	87.36321	59.03359	65.95924	94.69644	67.36642	114.1843	243.612
SDC2	451.8633	242.6813	377.0939	177.6024	779.7968	153.9583	464.0574	311.0633	573.7794
ZNF12	140.876	54.09971	46.42474	42.474	54.94303	47.71569	54.51141	48.5675	38.97656
LRRC37A3	1199.521	353.6472	309.4983	42.97041	87.71112	395.8105	150.3689	380.3494	996.6697
MDS1	101.2222	66.98454	61.60201	126.4892	76.42301	103.7768	51.28572	60.59742	81.06213
RINT1	152.8972	238.2511	207.8417	140.1212	177.1715	247.7154	197.5241	73.80901	957.2438
SBF2	896.3029	1100.795	803.5529	1346.273	643.4296	1224.124	627.8644	866.6279	1528.399
CDKN1A	11751.77	15347.55	19021.71	16891.24	20840.36	18706.45	19441.97	19501.22	10947.97
CLEC2B	563.4708	159.3949	610.1863	214.0447	501.4759	1090.625	1226.981	1273.308	285.0621
KBTBD11	61.65026	1574.976	2861.936	759.14	2324.266	2675.292	3145.264	2795.86	2036.651
CHRNG	543.2458	37.88321	47.84035	37.691	43.89574	36.97469	38.54835	38.83824	43.63454

NDUFA3	42.68052	1129.246	311.3167	43.34978	688.7271	337.6174	595.5762	593.5318	37.67971
NAGA	49.20275	41.30604	39.65046	43.46859	36.73369	44.54231	105.8768	393.1819	128.5576
TLCD1	36.96989	39.9406	558.9332	72.7609	1307.382	1341.639	1306.286	682.5162	74.33762
TMEM2	54.45181	349.0148	432.9611	36.3972	405.1738	222.9564	659.8812	359.9112	49.63448
PBX1	3236.562	5162.613	4804.282	3508.264	7614.546	3507.293	4219.861	3638.896	8281.903
GNPDA1	1768.94	2257.796	3803.688	909.164	3859.867	4043.766	4727.7	5067.215	71.62072
RPS19	28078.51	27082.82	31554.59	27688.24	27706.86	25560.03	27992.42	27940.63	29068.03
S1PR4	75.2646	68.9355	253.4406	102.7398	225.7748	74.15116	70.03012	69.28668	233.3804
MCM3AP	2586.671	2621.678	3088.822	2753.86	3287.147	3098.802	2812.446	1705.784	2665.211
P2RY10	3582.167	3658.7	1945.259	1225.235	665.4549	1141.703	861.275	1651.818	4458.232
CIC	122.6229	48.92549	67.53619	82.81184	83.52505	96.26936	110.2322	67.14315	139.4652
NME5	1394.747	43.45284	202.4854	43.35035	154.633	830.8848	648.7335	177.1572	77.01155
COPS7B	65.80488	996.8945	2184.462	504.7394	2360.265	2198.563	2795.866	2707.161	62.04538
GRIN2B	111.8697	91.80245	54.15515	113.1246	46.85456	65.81445	41.2667	38.35592	72.21713
TRIM59	743.7806	410.0229	418.7898	457.7969	279.6257	40.67543	228.0624	304.3682	46.56395
ODZ1	36.70782	40.86727	35.71672	36.33896	36.09395	30.48335	36.4971	138.7767	32.42853
PPP1R3F	822.5518	1219.679	1497.958	1587.972	1115.19	1679.536	1154.891	1241.786	1193.243
OR52E6	101.1918	134.5751	63.48161	68.4482	57.92657	86.83441	56.19944	68.60172	231.7785
CREBZF	37.58048	38.77174	73.70274	38.08755	81.78075	36.90542	60.20329	40.49524	56.76603
CES7	92.75111	94.53877	73.31216	88.85212	56.84238	71.30592	62.48716	67.59704	159.0337
FASN	24784.71	26970.35	22977.6	27706.86	21259.81	21934.01	18570.58	22893.09	24649.3
LILRB3	14287.83	8860.621	11262.21	7921.451	5959.473	8274.895	8481.207	8403.103	10946.49
TAC3	70.94658	49.40243	49.08084	49.73907	44.81467	35.88172	44.78146	47.47663	66.44593
ORC3L	112.2222	649.1405	417.5223	98.44482	390.4556	395.1628	239.5844	310.1809	273.0607
SPOCD1	970.7307	1869.454	510.7804	1711.657	369.6478	2226.04	1681.495	2556.056	174.6871
EPB41L4B	91.59387	136.5906	286.3173	138.9746	839.6602	685.0845	509.5316	525.9338	188.4743
DOHH	522.9634	264.0307	114.7199	254.9365	130.7153	190.5994	122.4331	122.6888	569.2661
PRRT3	528.2996	49.35466	317.2155	332.8546	174.79	397.7518	213.5822	183.0269	58.81036
TSPAN14	164.015	113.6284	58.46395	287.3853	70.32342	73.27595	100.2181	133.3454	169.6029
EPHB2	75.24628	50.23608	34.60412	55.289	49.38437	55.42578	55.70212	48.26473	47.8786
ATP6V0E2	10136.01	9302.959	7936.651	5521.212	5583.975	8684.883	5364.383	7144.488	10033.7
NXT2	6635.641	6136.26	6920.886	6018.093	4982.64	5509.661	4460.388	5733.673	6644.416
MUT	799.0067	149.9605	120.457	295.4627	100.104	336.97	102.032	111.3986	337.4157
MAGEB3	41.99265	47.59072	40.30548	78.47794	44.30361	42.06831	55.68223	42.65664	46.939
MICAL2	78.15105	58.69278	47.73709	70.96103	57.98681	89.51114	51.53066	65.3946	151.8125
HERPUD2	58.26055	134.0614	148.3827	57.1886	81.5076	60.00573	176.6205	235.3712	1042.383
CHCHD6	32.30752	55.57283	48.66482	51.18446	34.04367	41.14782	40.13629	34.48755	54.58715
ARMCX2	55.23716	319.313	170.4908	290.3682	264.7402	56.2102	140.4033	151.3372	39.74977
SWAP70	309.2047	958.9138	1177.315	1725.07	2414.652	1517.951	1723.204	1615.816	168.0778
DNAJC14	62.49001	56.13454	50.96001	88.19374	45.14584	50.20151	50.95049	36.90017	59.44022
UBE2G2	9471.997	8128.144	8315.093	7802.369	7842.423	7442.65	6535.409	8251.007	7309.143
TMEM14A	73.11285	32.82057	128.2204	268.1311	65.70871	48.88357	103.8042	79.14918	41.73528
DBX1	116.7275	57.22736	45.46891	68.81041	54.26982	54.71636	39.04505	57.86124	171.0451
POLE3	691.3217	772.2302	1785.46	1122.958	1628.2	2005.686	3176.1	2983.724	952.7815
STAT4	38.72728	110.5648	217.7725	35.47962	150.4285	299.4941	283.2967	153.2352	54.21188
MTCH2	517.4661	256.4096	230.6521	333.32	553.4028	372.7334	581.8324	336.6375	535.977
SLC26A6	8959.722	8193.762	7155.732	9807.601	5876.759	6809.963	8200.424	6014.401	8831.01

PLS3	60.17905	292.108	97.29288	819.049	283.984	312.9411	124.6772	42.27833	41.18309
OFD1	477.4939	441.8464	1547.906	289.0748	1409.172	1187.299	1453.977	1446.27	591.5242
CD58	50.85857	57.74685	126.6537	90.364	98.50913	51.54129	65.03416	45.27477	56.44611
COL1A2	3912.77	2943.7	1489.445	1846.711	1154.417	2525.549	1699.254	1472.726	2516.058
ALDH6A1	1726.852	1017.301	2959.622	1250.271	1919.311	2265.051	1644.87	2298.927	2830.654
ABHD13	132.904	73.49482	58.56002	81.31732	56.73523	101.4135	58.39684	68.94605	61.03054
DOK6	49.30084	53.35837	36.45712	60.20549	40.90251	56.91525	38.41412	45.15061	40.79576
RAB19	220.8198	72.63771	91.82902	175.7621	142.3402	94.81134	56.55152	63.6819	321.8408
MTHFD1	39.83204	60.97468	1213.093	1909.08	1184.13	456.5377	1072.538	920.1182	1748.206
SH3BP4	1919.857	551.8973	1122.547	2405.823	1593.127	2015.494	2470.694	1326.224	925.9072
PMM2	105.4007	2036.436	4293.13	1928.835	6548.167	3128.602	6222.568	5505.527	1278.028
GPR84	651.2912	595.5649	428.6497	934.6955	341.1409	554.2994	374.8687	528.1351	883.2698
DENND5A	1798.612	35.90843	1683.551	40.98939	1101.346	1153.578	1508.959	1225.191	34.65519
ZNF8	675.5294	399.9087	425.79	528.5239	498.7438	584.2581	456.2705	661.8709	721.0666
SLC1A2	85.71681	51.73863	59.70213	51.74716	59.44589	84.10143	53.24008	67.56574	173.0094
FGFBP1	1001.704	4104.556	9705.309	8944.002	12820.98	8915.634	7431.003	16293.08	4234.398
SLC17A9	133.3048	311.6583	59.11089	101.4516	54.15678	256.6368	63.83222	69.26318	204.1611
NAP1L3	33.80743	253.5378	42.88073	47.56378	37.72369	51.4475	35.61465	47.7865	80.91756
ATP2C2	39.04812	44.54781	43.691	42.72099	35.31904	53.60149	149.4103	39.0568	108.922
LEMD1	98.1039	55.17303	44.79568	59.72575	160.3289	59.74929	44.1702	64.33728	94.00934
ACOT12	59.0188	40.02981	52.029	58.09667	47.9919	62.02175	52.22028	53.44092	69.69878
C20orf165	1087.941	72.79513	172.9081	67.82481	162.3835	46.09928	89.35501	172.9536	1457.505
HISPPD1	48.1715	54.38522	46.93808	63.24924	42.88736	66.51527	52.01604	39.6733	74.09315
LY6G5B	41.94485	50.82682	61.76516	737.8928	380.6373	434.4974	237.9778	207.5755	35.45824
SLC30A2	43.4731	46.26335	42.96637	49.17838	43.81057	48.44841	35.44734	33.57226	37.12088
FGF16	639.5714	41.66838	32.19466	61.51488	46.45805	46.83636	35.62333	57.99702	85.09769
SUSD3	63.84026	52.37661	48.30056	70.03249	61.64384	75.59988	58.12885	60.39082	219.3597
SMTN	42.94581	41.93495	41.96461	824.5991	57.81393	45.15705	359.4593	228.8959	55.26674
BRD3	1559.433	53.65517	52.76355	94.06181	59.72393	52.94724	147.5806	569.5627	97.72113
C20orf29	276.1176	66.87077	289.4951	65.74593	51.34132	105.5337	303.3705	299.4241	44.97458
OR11L1	34.14832	38.53979	41.00859	48.17521	44.58643	36.4388	42.25537	34.29449	41.86202
HMGB4	323.2722	141.6444	75.12056	113.5167	75.42432	79.04659	69.89308	94.16912	410.8634
SRPR	133.0656	1322.696	1398.946	702.5458	1914.117	1112.274	1402.511	1040.228	329.8913
PTHLH	3081.083	2968.124	1326.679	7681.482	3640.493	1320.891	6991.792	4405.56	3031.197
LMOD3	40.22496	61.50132	45.43291	69.60035	57.87563	34.75627	54.42816	77.38473	59.92354
NEDD8	304.5131	151.719	240.8711	973.2394	293.1469	294.7154	377.4801	315.273	103.6477
TNFAIP6	141.406	231.8781	403.2611	621.618	709.4341	351.8295	1119.7	712.1528	60.51713
AMZ1	74.43026	46.61423	45.53246	48.98012	56.73035	53.08648	59.64082	56.70336	39.81218
LOC731314	413.4796	218.4519	234.4843	191.8338	576.8611	131.7165	280.7	277.8758	452.6866
TM9SF2	8303.053	9038.546	8068.819	8540.902	7593.661	8453.977	6091.965	8706.577	7720.541
PDE4A	907.0901	692.0515	433.547	652.149	416.8976	582.0801	421.4411	737.975	1155.865
METAP1	104.9928	54.53481	358.0788	54.70857	651.6202	232.3875	322.9936	354.913	41.13747
TTRAP	547.8055	123.0798	112.2854	263.1692	97.06499	75.38206	87.42925	107.5827	171.7759
OBFC2A	2684.228	1315.278	4256.077	2314.119	3594.154	3300.988	3572.359	3034.901	4161.605
PRKD1	44.73513	304.2765	151.3241	291.8394	712.6821	449.3124	367.1845	150.079	423.7935
DSCR9	73.77174	50.38508	51.87107	54.91362	38.08141	38.11676	38.48853	50.39765	58.65659
C1QC	3385.069	64.93818	544.34	65.35448	62.48798	1647.779	771.2052	631.3005	34.6296

CDC6	126.4221	94.96127	72.86506	87.547	173.7043	1013.243	429.5728	282.4822	137.3882
LOC38896	172.0252	127.7245	58.19271	65.60024	61.09712	54.12494	55.83705	59.09611	90.69175
ZNF621	1320.977	3244.514	5369.186	3651.434	5774.767	3456.817	6245.627	3437.2	2103.006
C19orf18	211.38	2571.639	534.0235	776.0552	597.7037	1165.505	1519.591	768.8024	1024.746
C2orf60	39.13602	489.8674	218.3879	50.30153	103.2188	48.63157	71.44971	35.56382	35.77516
ZBTB37	2318.527	1392.018	505.4326	1096.65	344.0422	345.1266	535.0424	430.9449	1480.596
ZCCHC16	45.55487	33.85393	35.47839	38.23927	42.66674	33.66338	37.51186	37.41406	49.37215
CTCF	998.5809	35.28289	214.2791	41.54045	128.2615	40.86424	43.33599	33.35864	60.06922
CHD1L	805.3199	54.55654	734.0041	700.4933	1273.856	1097.073	1234.162	431.6264	41.71383
PRKAB2	1158.042	1203.416	897.5239	2821.169	937.4427	1291.083	1457.337	823.5009	2916.65
GPRC5B	551.1396	443.2076	290.8918	319.89	291.3048	321.8123	246.6525	291.6457	716.6915
GPATCH2	35.86106	593.4578	145.3661	36.13092	316.7153	33.91895	297.4158	170.5284	39.10563
ASB9	557.2672	43.08322	336.7824	34.41986	648.7603	207.955	395.607	247.4245	46.069
SLC24A5	40.535	42.05948	37.93469	48.07796	39.36042	39.1998	35.27674	34.37291	34.95658
UHRF1	97.15644	726.6147	432.2392	377.4913	286.2215	670.3983	544.6525	451.0975	1240.276
MEPE	57.39052	70.41563	75.82883	52.09955	56.65475	66.38499	58.91913	46.04094	93.99001
ZNF471	81.73424	58.17821	50.38293	51.12448	249.4467	76.30431	262.823	52.87362	112.6508
PCBD1	979.9763	451.0262	992.2159	410.1551	919.4217	525.2099	808.7465	766.8714	623.0219
TUT1	54.71347	57.57865	476.1566	105.4443	503.9135	616.3306	528.769	212.3375	96.87537
FXYD7	74.38793	114.1891	282.9462	66.6642	218.239	69.63902	51.60339	56.98277	64.45217
CCNA2	2351.211	2119.868	1039.324	2882.662	853.0269	1148.633	901.517	1566.948	1666.794
LOC40046	44.45159	35.14416	144.5517	41.78061	264.3439	176.7514	159.87	135.0129	37.87769
CD37	46.11892	42.57287	432.7271	46.22239	190.9167	63.14836	36.2064	220.7679	62.46071
MKI67IP	447.9832	71.12591	156.7067	253.0419	164.727	219.3022	183.7878	122.0774	113.4305
PRKACA	1089.125	431.8154	293.7528	1358.683	337.5963	203.3325	124.048	239.8803	855.0195
NR2F1	2289.107	3732.983	2886.983	1171.239	2997.354	3489.793	1672.727	2294.382	3223.372
HERC1	108.1965	70.3707	375.2376	1499.677	896.4252	2056.71	617.0092	1073.349	45.91634
LRRC59	510.766	66.0168	116.6547	278.4029	160.9182	247.4659	126.0097	250.142	707.4658
OR6B3	111.5776	83.02146	56.59735	82.46272	66.42001	50.45098	55.89972	60.94505	82.54108
C1orf122	53.56043	68.11015	229.7804	1029.268	395.043	280.179	424.2111	424.6857	47.9373
PAPOLG	1439.783	1165.383	435.2245	141.2622	63.1994	804.5253	612.4253	706.7632	1630.575
FAM149A	46.55521	511.2913	181.3323	47.80737	44.2887	50.84112	100.7	37.18965	1491.711
ZSCAN12	90.94191	68.48269	36.94464	70.69317	63.61548	96.80593	48.86808	62.82018	69.37789
NIPSNAP3E	745.6115	1083.511	492.3822	457.4157	186.9623	632.1564	306.9957	631.5321	1554.974
MVK	236.6387	108.3468	65.21419	139.7178	590.2027	320.024	132.6397	83.90805	172.0426
KCTD15	7812.819	8125.455	5994.277	7553.356	7089.605	5386.909	7531.881	3543.84	5798.995
WDR40A	43.97906	54.31723	476.4537	654.6125	1191.335	363.7543	506.7113	414.5671	46.78478
DUOX2	40.64236	33.87002	40.38317	38.43743	121.4095	99.73303	82.10172	34.81193	69.22478
CNGA1	38.40466	34.10727	196.8785	49.85146	242.2755	1047.1	160.6223	400.2468	50.07915
RDM1	295.0149	188.017	91.50544	171.0674	91.89356	142.7131	74.92819	106.9386	235.0127
SYNGR3	49.93517	51.89296	30.98969	63.60044	36.81911	290.8466	40.26055	48.46581	54.71857
AQP6	51.62658	55.79671	47.42862	459.6845	46.67657	41.58021	42.20594	66.76922	61.38015
RNF113A	2036.47	2501.424	2129.399	2428.064	1777.222	2695.126	1906.733	2323.053	2477.06
ZZEF1	74.14191	66.49355	541.7943	46.83571	984.3248	608.129	851.7021	594.6178	40.46918
TMEM149	201.2489	156.0119	78.63047	153.752	76.79882	108.2344	109.1755	240.2049	214.3889
P2RY5	1579.49	364.4849	1524.174	407.5174	1145.347	834.5005	832.8842	1003.651	81.29302
LPL	277.226	282.1124	94.72837	115.9387	139.512	114.3207	126.2462	115.562	458.8138

C1orf74	583.0101	396.2856	553.699	1431.191	408.8786	739.6044	744.4147	668.153	275.5619
PRSS38	41.53818	50.60505	44.76047	41.42633	36.23522	45.73872	42.11893	54.67151	41.34159
IQCF1	58.67163	43.34929	36.52585	42.69102	37.09613	41.75943	44.68983	41.70715	33.52661
COL14A1	445.3803	185.4767	140.4677	274.5174	142.6052	184.5061	105.8211	134.5119	749.9326
PLAGL2	1266.509	72.77015	1075.5	729.6794	1207.098	706.4259	668.3634	876.9171	2170.115
PSME2	569.8726	492.9807	470.5482	396.8846	370.7939	402.0937	475.0941	668.4015	151.8533
SIX6	41.08234	51.49978	36.67476	43.44772	40.82078	59.33939	41.47243	34.93063	51.61368
PRX	76.98345	105.6627	70.59015	52.76171	57.66636	60.82865	47.27337	56.58546	88.84588
BOLA3	1945.946	2069.699	5142.122	2381.64	4423.152	4472.836	4394.756	3758.663	897.6124
MTRF1L	288.9635	446.3787	129.5274	158.1066	81.80424	280.9441	87.40016	152.8845	448.7229
DEADC1	96.74571	72.31455	170.3506	90.14374	51.50632	75.22295	53.06468	138.8434	89.95448
NDUFV1	3992.585	3328.366	1669.751	3564.051	1252.025	1678.676	1798.156	2023.203	4810.757
PROM1	1602.498	1916.439	805.4957	586.7913	839.8845	2463.074	2819.1	1945.969	2389.473
ZNF385A	2525.023	1521.605	696.9412	1681	1367.948	1154.366	1326.91	1431.935	3816.429
C11orf10	3880.861	7653.829	17203.43	5695.145	22006.98	11427.58	18117.27	16440.07	5768.935
SNTB2	60.86629	877.0596	394.7611	401.5624	143.3891	44.60097	189.3902	244.9199	91.84837
LOC38764€	77.18279	91.58688	55.87709	59.83478	50	68.7026	43.20955	60.77987	90.33067
SLC18A1	330.2559	175.9903	103.9076	216.6455	100.9921	167.7838	96.79121	116.2296	377.4031
FAM102B	214.8033	134.3728	111.8287	57.08503	177.3387	137.8175	139.1663	129.1158	93.39359
KIR2DL1	747.7843	1710.565	484.8572	538.4392	46.88253	340.0159	225.4805	770.8816	949.8995
ICAM2	49.54287	54.90751	59.96652	59.98471	791.9683	66.16025	435.5598	42.87034	83.51866
PRKX	96.78242	75.14284	59.82517	94.68507	48.07237	69.11702	60.49171	53.93826	75.13014
KCTD13	1457.732	765.1166	1942.075	150.766	1446.906	1696.915	1733.452	1329.715	822.364
TGM3	22026.29	12480.15	27326.38	8050.282	32930.55	17441.12	22256.87	14434.76	18043.04
ZCCHC3	43.20419	36.9418	33.70616	45.09189	45.42877	41.59456	57.00499	37.75259	93.60998
C11orf31	65.9015	461.6396	200.1614	64.15228	126.4077	62.23494	54.32914	72.58911	50.17613
ERCC6	137.7688	540.2538	71.09367	659.5266	294.1381	137.9002	138.945	224.0048	1590.192
RBM5	57.91131	48.72449	4253.46	54.81199	7109.499	2019.525	5852.902	2548.122	2628.053
KRTAP26-1	34.58858	35.88814	59.35906	35.09241	33.35995	41.85765	36.40693	32.06955	32.67255
JARID1A	38.7611	1201.291	1855.449	833.4131	1455.087	2112.163	2328.86	2275.614	44.43856
OR52B2	48.34106	35.82898	55.46043	45.19178	52.41012	49.86011	46.42867	42.77662	37.40845
ATRNL1	118.8287	60.73734	54.47757	77.04729	54.01334	48.3903	56.89397	43.86481	116.111
SARS	1449.901	1092.897	886.5918	1966.223	637.8296	890.2377	606.5842	778.8345	1357.146
RAB22A	54.08451	43.02671	44.70147	46.65894	607.8745	339.5838	448.9265	403.9059	89.4094
NKX6-2	57.64805	46.13795	38.87647	69.44303	40.83926	57.59694	36.32577	45.701	45.97324
NHLH2	56.6809	69.43697	58.25306	62.14003	42.9148	38.41085	89.10954	42.05742	94.5208
SEC61G	44.0096	40.64718	58.50475	38.68004	173.5379	828.8827	57.20996	46.4966	50.48161
LOC38919!	66.48482	298.3178	134.762	71.79504	108.0848	74.19083	69.45616	128.4175	137.8608
GLRA3	82.37559	39.69424	39.83088	42.35145	51.05944	36.85669	42.86729	46.08189	94.7403
CLK2	45.377	42.54943	219.9438	35.26909	337.4702	50.71267	235.272	125.951	33.37417
WWTR1	51.65321	50.3228	37.68256	48.21838	150.7784	41.73999	149.25	56.38359	51.49941
SLC43A2	1257.438	1776.157	2589.809	1627.956	1074.09	2690.288	2786.811	2402.264	1238.349
MEGF11	119.3004	46.59308	49.49387	83.11135	47.33012	50.32399	49.64113	76.13206	47.05923
NOV	61.14332	75.00343	46.73611	39.55556	172.7762	56.66806	76.56804	107.2983	140.3248
NRP2	723.6896	401.9396	295.9468	368.7621	161.2523	430.6502	145.0859	155.9503	75.0701
C12orf28	96.05201	132.1787	54.09852	107.7872	60.43343	66.8453	97.59843	69.02542	158.1705
DAAM1	2685.608	743.0963	526.8322	510.476	735.5186	988.7344	559.3199	4994.44	1668.732

HCN3	6433.542	4872.548	4082.745	5562.986	5214.759	4489.902	2712.497	4183.577	6112.204
GLP1R	41.31191	31.98757	36.528	38.31768	33.72469	48.61765	38.41244	50.78574	64.81793
RNF212	116.3315	72.4991	75.44717	80.52182	56.22512	84.43407	51.95816	62.25265	91.30129
LCE3B	45.14492	37.30825	40.19665	45.27575	33.95114	32.62953	45.94336	37.2122	44.20659
ZNF503	8139.98	7964.43	7070.676	7088.524	6367.457	7754.094	5834.699	6887.346	6465.735
SPINK1	77.90305	41.1246	41.14397	49.62757	49.42305	50.89643	43.56076	55.08664	66.39327
STAG3	1050.968	312.8916	192.2568	555.9221	139.991	289.8094	105.5779	150.4408	1588.53
C1orf135	798.3415	1301.907	960.1675	2712.793	575.6499	1195.542	1433.554	1531.489	1551.064
LOC15038[illegible]	79.29404	106.0485	54.17693	81.08351	71.6875	419.8765	56.76572	60.24791	105.5672
CDC20B	77.24736	73.27346	63.32831	104.5833	45.2943	52.44012	46.39352	48.63677	94.46327
PIP4K2C	176.4226	308.8183	801.8728	335.4911	722.4572	921.5976	775.9752	580.0239	103.0321
C16orf48	17703.07	17949.15	12643.9	16365.59	15561.06	14422.11	13789.46	14470.26	17580.37
SNORA65	9402.732	9349.035	7037.628	8245.154	4998.675	5880.077	4066.469	3510.321	8116.877
OR8G2	416.4607	240.94	155.9047	290.0573	269.0955	265.8185	177.9969	285.3175	534.5919
FKBP11	2377.021	4386.552	3857.578	3060.264	3871.366	5384.17	3920.728	3005.539	3925.86
GUCA1C	162.5726	73.30937	70.32531	103.6775	65.8344	97.7913	56.35257	76.32787	155.763
ZNF335	91.15464	57.2962	184.4166	44.93218	225.2481	43.5615	111.5378	151.6587	50.01652
RETNLB	315.098	118.3811	79.24803	210.4232	75.45575	122.095	70.12382	98.62318	462.1211
ADAMTS1[illegible]	142.5407	81.96353	64.33863	76.52237	59.18063	77.90246	55.99183	69.7253	140.5878
NUDT17	74.34438	271.4853	48.62151	61.95033	47.63868	93.81893	51.28758	61.71442	82.90906
SNX16	35.6305	60.02488	584.0848	786.6599	990.8038	753.6472	903.1755	637.0089	797.464
OR10H1	73.7462	33.02937	38.97639	39.19573	40.30134	49.20631	100.2414	38.83452	61.61744
CCDC17	64.37889	55.04266	277.3432	48.99727	484.3397	69.68439	547.6033	47.29313	55.12715
CENPO	44.04964	45.28623	155.6278	574.0422	160.1322	58.08138	382.1083	364.7323	94.87961
PRIC285	37.53291	5725.023	11019.79	6476.282	6388.463	13496.04	13135.46	8417.753	5682.364
THUMPD2	103.2353	540.3513	307.1083	606.3798	887.145	499.6621	783.3233	1043.837	305.5937
ACTR3B	59.58525	43.88926	160.6902	37.30243	52.98826	46.94194	38.68209	61.87026	40.09701
CAPN12	1364.294	232.4558	471.3021	2033.293	1128.539	544.7607	416.9585	1100.386	1907.419
GSN	38.85693	71.76212	35.46001	37.61998	50.69838	37.57821	43.78321	39.74299	70.42631
MGC13057	308.3206	171.8478	104.5104	174.1553	69.24614	130.2834	227.8112	88.85825	409.7746
C3AR1	485.7394	137.7453	259.9324	250.5368	202.3482	269.8017	268.5839	181.3474	490.2968
ZNF177	48.11407	59.37596	101.4161	43.67471	102.8157	156.4678	190.3526	49.53563	82.54228
SPSB2	65.80181	42.97561	35.89835	37.5465	43.78672	43.78464	70.09449	44.82633	37.41978
HSPG2	52.12401	102.2321	53.71585	99.02051	52.63705	54.07723	59.49648	58.30661	65.8271
NPC1L1	90.41833	57.14625	48.23263	58.33504	42.48235	49.27626	45.13861	35.86078	52.12708
CLCNKA	705.4939	417.4794	166.9721	271.62	141.3228	245.1178	138.4034	235.4465	743.2797
CTAG2	96.28899	82.04784	44.85313	49.11893	45.8642	69.46523	52.76448	42.77477	261.1392
XPA	400.1983	199.437	121.1029	247.4072	124.7314	172.4583	101.9147	150.6057	327.7773
GPRASP1	257.2066	173.6015	101.5428	142.401	83.34109	132.1465	70.51391	91.04967	269.0684
CLDN12	58.93055	386.3248	331.4836	425.5785	499.2774	328.1885	456.1173	300.3395	94.28204
MTMR7	101.2623	122.2038	63.77058	85.02312	160.7373	343.2176	133.7259	237.391	225.76
PLLP	273.1254	170.2463	89.19982	194.1445	98.47041	139.6232	73.20486	91.7797	260.5571
CITED4	39.20254	2645.962	1181.663	2366.761	859.3711	309.1122	1489.635	1127.803	2916.804
ATP6V1B2	755.7193	1376.195	4583.602	494.2231	7909.058	3568.398	5450.04	2821.54	1201.994
SLC27A1	40.48053	61.73279	1141.994	57.61652	1988.896	766.316	1789.074	1915.793	93.46687
HEATR1	44.1557	646.5778	2181.647	677.1157	2394.906	691.2809	2127.501	901.0085	66.59096
CEP63	2507.038	3385.449	2232.308	3945.166	1609.044	2128.483	1825.425	2599.199	3271.431

SLC5A3	342.8367	1513.21	1437.637	2160.615	1210.956	1842.876	855.7446	2048.269	2681.442
TRIB3	114.4035	369.9879	937.4489	445.5513	655.7064	680.0743	618.9233	266.9891	190.9828
ODF3L2	1854.982	468.4839	33.6132	525.4635	37.45534	805.3978	611.9904	1285.766	1370.67
HSDL2	965.087	57.39302	643.6366	496.258	737.2256	400.1073	981.8745	505.9794	1928.866
C19orf10	242.4232	127.9312	72.25769	137.2043	303.363	117.2492	78.14079	223.8905	274.815
OR5AC2	36.36526	38.28264	34.89948	36.08195	43.25452	32.66185	36.68416	50.10186	35.41433
ARL2	18828.54	17148.74	13485.08	16048.31	13084.01	12500.49	12937.93	14520.55	17479.93
NUDT18	56.98056	59.21114	956.1799	908.0796	1654.373	1563.588	2221.93	3427.694	39.08825
CXCL1	1987.306	2073.708	756.3582	2267.804	509.2843	984.3428	668.3897	710.0109	4595.793
PNPLA2	146.017	567.1086	363.061	116.0189	375.6984	53.80307	376.3299	192.0313	281.4699
LOC389517	3021.954	3357.918	2327.613	3529.137	1261.8	1349.839	1181.394	2703.331	4460.048
RPS15A	2198.866	863.3712	1097.285	1309.212	837.9495	767.8918	848.1494	622.0063	2013.288
RABL5	407.7114	166.2987	107.7931	219.5894	94.97495	156.3456	78.41795	138.7017	117.9486
UBE2D1	97.9231	79.72934	63.10028	119.4331	64.61389	74.40534	60.90207	77.40153	64.13899
HSH2D	38.08567	406.3063	143.9077	36.61577	38.77417	38.01779	44.58039	39.00262	65.68118
LAMA2	4095.977	4786.292	1536.245	2230.461	1524.941	1690.69	1374.072	1500.183	1483.873
CDKAL1	35.02265	652.0766	1162.425	45.53554	383.4913	391.0376	709.999	703.3594	39.73359
PCID2	58.79989	552.7618	1726.767	1047.547	2509.707	2417.421	1837.714	1741.396	1452.082
SLC25A12	49.7246	74.88451	808.6118	46.27668	700.4327	419.5757	956.8675	536.166	114.9267
PCGF5	55.91396	35.49243	31.23206	34.877	56.91748	33.11425	98.94949	71.431	36.68284
Septin 12	216.6953	123.3788	71.84991	152.7941	68.13803	118.9822	73.70701	81.17349	247.756
SH3GL1	10914.86	11093.33	14191.37	12719.04	14930.47	12416	13653.72	15816.9	12090.58
SPRYD3	6115.885	5723.487	4896.801	6785.431	5893.641	5762.549	6032.767	5112.658	6136.024
LHCGR	36.65968	55.91549	45.28988	42.5168	39.33739	44.81288	84.70862	37.00727	49.42236
LSM4	851.3885	748.2726	636.8522	1788.205	1128.357	441.7601	1463.349	1155.688	1146.612
IL11	389.1155	217.9116	109.0204	244.1488	104.0507	130.1399	155.5481	118.5236	411.6563
IL17C	95.78005	463.9156	152.6842	100.3357	259.7336	50.60156	169.5054	73.59432	385.124
SLC23A3	44.12129	39.9871	849.0681	586.1316	835.0457	962.7923	659.8601	662.7131	36.09243
GSTA5	42.23357	36.64863	41.84864	34.32043	37.87841	52.72326	37.07248	33.03965	33.31334
APITD1	3463.828	1270.585	455.1147	46.08986	639.7009	419.2037	257.1495	313.4258	1509.061
NEK11	36.89407	943.1332	1304.459	70.25445	1875.039	1310.875	1804.405	500.2092	43.91831
KIF5B	1162.128	2057.434	1299.553	1950.394	1586.001	1340.779	856.2208	1292.283	44.10652
TTK	493.0614	309.0939	380.9602	479.5745	221.7241	296.8825	268.115	252.9213	672.0826
RAB13	3195.402	2192.89	1377.504	2288.007	881.8372	1577.305	1014.523	1527.257	3414.759
CIDEA	9053.197	8193.543	1311.725	4304.285	5999.051	4642.224	2398.184	5638.957	11607.82
ARMCX1	1102.123	587.3278	34.36491	50.61156	44.90936	77.94389	82.70686	41.23777	38.55076
HEY1	71.04998	351.8318	388.2072	60.80252	616.5126	635.5706	804.1115	730.9875	148.1424
SNX19	687.6102	359.7634	296.7125	568.951	218.8343	450.4504	183.4674	292.5296	1431.753
FRAT2	95.96027	57.14467	1978.655	896.738	4825.93	2314.237	4027.182	2186.662	73.80715
RSPH3	1358.708	2621.459	2787.302	2573.675	3507.137	3127.285	2755.48	1729.875	121.0348
FGF20	94.85654	107.6981	86.36256	105.8796	64.7316	88.13876	61.63647	62.20436	912.6454
AMDHD1	1042.077	123.0605	201.7864	132.0954	349.3575	340.3019	230.3484	249.7921	1342.932
LDOC1	1262.875	788.2408	49.28353	66.89639	167.831	60.78391	278.0748	68.38194	161.9934
SNN	427.2609	241.3855	201.3456	218.6365	245.1176	240.189	230.2763	263.1637	191.3286
PPP1R14D	82.67313	63.6232	52.94627	64.30412	41.07382	44.6074	44.67316	45.82066	125.8576
NAPG	2644.361	2324.209	1232.339	3288.246	1367.835	1642.302	1021.399	1647.974	3289.396
COTL1	537.9443	305.6239	147.1706	394.8213	90.01252	205.6795	100.9934	105.7269	760.2948

LHFPL5	112.6699	106.4241	59.84628	54.41812	47.55521	53.82294	58.97893	69.3779	125.5827
SIN3B	53.90983	43.02586	693.2611	89.34563	979.4261	364.6783	1481.173	816.714	91.7937
KIAA1737	99.26292	33.31788	219.0437	51.31823	120.8647	215.6987	89.06773	41.5095	55.54731
MLH1	206.7888	204.501	341.739	189.2234	684.5136	41.63465	543.4182	555.345	87.97002
COL27A1	49.32072	76.8478	46.30542	107.1774	45.04099	486.3489	199.4583	252.005	79.26118
C9orf5	42.4944	42.62674	53.72197	39.71494	97.74505	144.6373	190.9737	161.9922	711.5734
UGCGL2	6713.948	5034.54	4107.874	5990.948	4998.843	5243.652	5536.43	3560.481	4080.349
NOVA2	417.7957	1479.899	120.3786	746.2655	207.0591	150.4585	86.06406	122.0912	559.2823
TTLL6	73.50441	426.551	39.64282	64.0914	149.1101	46.00922	35.71058	44.8408	67.91074
KIAA2022	50.93416	37.84074	35.04359	35.46832	33.62233	36.80581	32.96609	38.31847	44.27961
KCNK15	56.62805	64.80025	150.9132	47.21815	223.7047	50.02073	222.9425	79.75573	39.39644
ABCC4	33.87306	33.39479	47.66614	36.51872	33.45685	32.47495	31.87485	40.55505	38.70637
ARGLU1	3347.078	2745.008	6182.768	2829.687	7242.127	5335.6	5118.534	3489.777	1275.837
HIST1H3F	896.6566	347.3259	202.8936	206.884	76.58383	583.787	234.5534	258.2472	1215.612
MED18	218.0321	46.79219	53.50639	90.68757	60.39539	75.63215	54.70564	55.38047	105.172
SIT1	63.88491	77.9634	46.62513	42.60424	44.08753	73.20689	52.23082	55.52222	67.23824
NCALD	3087.947	5526.381	5181.38	3499.125	5593.752	4284.159	4855.451	4190.208	5387.528
GCLM	3189.549	966.6117	1899.884	2400.787	847.6047	1825.063	1675.597	1173.737	56.9677
WBP2	11909.96	14697.59	17401.05	14140	17469.54	18150.97	19052.88	16972.43	11051.52
RPL23A	34.22141	814.6375	2124.129	996.6469	2361.164	1132.874	1985.598	1642.158	38.56601
EXOC5	891.8488	43.4878	257.3264	43.01991	288.434	57.18868	132.6394	48.99443	1519.601
CCDC46	70.34573	83.23215	103.5966	62.76713	117.198	212.3808	188.8238	212.2169	157.0913
GPHB5	175.3741	149.1401	89.82863	148.7983	65.76752	128.5099	66.12395	86.8299	209.1587
CRLF2	40.59481	87.69387	232.8055	39.17136	42.3204	60.40464	111.3034	41.09426	40.01199
PHIP	33.28644	2136.905	5206.504	38.1286	6566.461	3378.725	5634.912	3984.99	48.75116
ZNF710	916.405	440.0662	392.9985	449.4149	168.804	45.59101	118.7044	113.6753	41.18429
HSN2	53.84698	434.5975	295.4855	48.47102	120.5315	39.58956	226.6594	310.9607	49.91324
LGI1	74.65849	44.523	60.77341	63.13167	67.21889	90.23948	43.22443	41.57707	51.75729
TCF23	252.9781	159.676	77.80749	183.1415	71.12454	97.99821	69.86269	88.8818	216.132
C11orf36	64.17148	71.1455	53.87533	80.3102	49.31673	49.3575	48.70615	49.7608	47.90243
BPGM	38.45078	38.9907	155.4457	55.27718	202.0609	36.07184	35.85554	46.94009	59.68948
MAS1	55.26561	76.03813	57.46771	52.01631	48.82525	43.89956	46.29599	44.58411	34.32141
TYR	52.02151	33.56718	43.14735	40.39264	43.81259	33.37888	38.32549	48.68305	82.11144
Septin 11	11426.09	11097.12	11287.59	14893.83	12420.89	13012.06	11163.62	8940.963	9596.237
TRAM2	34.79319	334.5374	74.34696	344.5229	99.98684	158.3228	202.9531	118.5873	41.54868
GLRA2	84.0486	42.18623	54.35003	37.46201	47.82377	65.08208	40.48439	67.89041	107.7522
CHRNA7	192.7203	58.73625	91.13091	117.2527	70.32676	86.19025	61.36689	68.48554	184.7911
KLF8	36.93061	41.14822	442.5825	39.53968	722.8008	44.05008	244.5546	666.3099	32.92273
GCH1	136.5711	152.8848	69.97955	146.4059	82.3517	123.2459	56.95943	75.82034	138.8761
FAM71F2	69.17262	77.42522	58.83054	72.24841	49.82604	74.18027	48.93061	168.8134	42.63377
C12orf30	2922.276	3040.527	2058.64	2449.289	1563.751	2447.469	1500.177	2085.247	2847.75
PLA2G2D	215.5666	113.9078	62.93036	117.6431	72.75009	87.84487	70.28125	87.22246	244.8378
RASL10B	163.1664	39.75814	184.6687	52.59907	37.53337	321.0057	72.47708	618.9974	42.06054
TET2	127.6718	59.93003	100.4938	87.43822	46.77332	76.92301	52.59398	59.63984	92.43869
MIB1	42.46262	43.25911	54.30847	52.3651	150.6432	44.42081	58.64442	38.47465	46.37691
TCL1A	35.84943	53.38059	139.9741	35.33614	45.72445	33.60503	66.46629	41.87486	49.8744
OR11H4	85.92587	59.64992	41.57368	51.61516	44.03778	35.53786	33.31128	53.21969	64.72439

SERPINA3	750.3565	2332.066	854.564	1240.736	819.5682	1791.528	1843.278	1374.236	38.95535
MORN4	182.0857	67.1534	56.92435	67.83166	56.32887	68.97955	48.96118	43.59358	187.0247
TOX	59.24914	52.18838	472.1344	52.33694	57.60272	914.42	245.4657	50.91531	74.5302
SRD5A2	232.5644	150.0174	118.8829	183.5899	69.14922	198.9468	62.93395	72.72212	207.6186
ABCB9	57.99924	46.00386	38.0764	49.99385	37.10745	37.37955	36.87391	33.99491	42.8714
DOCK8	722.915	61.44776	373.783	41.01745	378.063	245.3179	287.6674	273.165	74.03088
GGT6	201.6357	117.3675	380.0591	113.7389	714.8522	269.6969	129.2324	184.0359	185.7636
PDLIM1	503.4128	319.9678	201.986	369.5493	207.0182	251.9533	364.117	481.1236	539.6927
PPP2R2A	3330.989	3552.597	5999.613	3757.035	6229.839	2747.402	3027.306	3869.894	4896.215
SLC35B2	93.16512	97.34955	504.2102	71.76494	685.8815	270.2259	580.4361	290.2237	314.6712
APOC1	946.8235	920.4888	1414.993	1006.311	313.2848	1565.516	529.9129	1120.143	905.633
SYNGR2	608.1298	40.29123	198.8362	50.2669	279.8154	50.33662	186.6223	68.48861	50.0443
SLITRK5	50.37661	40.44371	46.51997	50.35519	184.3811	50.28333	107.2496	46.55357	70.63649
C1orf188	53.57587	45.78527	39.22904	53.97226	37.77108	41.25856	82.56044	52.06867	42.09192
GDAP2	93.2634	1819.854	1373.024	1175.852	754.3683	613.3254	941.7385	1160.243	108.1598
HSPA1A	1231.053	2752.571	4142.054	3028.648	8583.014	6081.65	7316.104	6982.044	1350.587
DCTN1	277.8067	141.7218	104.2443	232.883	112.2664	154.3011	80.95019	113.0551	397.5096
NINL	56.87571	950.8699	445.1066	446.196	277.2417	286.3013	283.4582	210.5853	43.33962
BMPR2	1810.15	491.3323	61.44005	55.5336	275.0164	279.5998	203.9448	342.4099	56.77608
OSTalpha	89.89032	61.15321	51.5826	65.01152	1030.316	484.1443	270.5304	48.73584	126.5963
IPP	270.9105	128.9728	89.74758	159.7547	128.46	66.12782	69.7316	66.24099	144.1001
TMEM145	3249.38	2500.153	844.7505	5653.093	854.4671	1929.841	1332.925	1634.016	1490.373
PLK4	212.1104	133.712	329.4687	143.4756	500.8258	242.1472	268.6626	138.3461	274.6208
SERF2	6173.42	4201.094	2763.023	3806.575	1595.515	2251.377	1924.098	2173.318	4629.672
C1orf113	138.6512	103.0336	74.64285	79.67972	72.34196	72.91276	64.74393	54.0124	171.4587
MAB21L2	131.612	45.40347	39.30696	37.09711	33.09018	31.70241	35.56406	33.17642	55.03787
SHD	7918.76	4753.003	9956.614	5068.566	462.2051	3458.503	2814.184	2753.012	8715.839
EEF2K	3436.095	2526.843	2163.629	1766.255	3409.68	3040.768	2373.455	2330.396	32.82886
PSMB1	3252.935	3640.7	3226.053	2782.627	3346.723	2875.178	3314.961	5144.096	4876.123
OBFC1	481.0322	248.8417	1022.913	237.9881	1173.11	196.4177	863.596	690.4207	471.0378
MAP9	538.6069	275.9329	333.5674	575.188	202.6983	239.203	176.0876	281.4699	69.03567
VPS37C	19940.58	19583.98	22103.22	22312.15	20978.48	20477.47	20629.57	20573.1	20451.38
MLST8	87.12907	1112.478	953.0669	1551.875	441.5605	995.5883	1324.622	1284.631	36.36507
VPS33B	35.18426	70.71071	165.3694	51.03928	399.7387	47.62426	382.1041	229.2999	93.41044
SOX8	4891.377	5711.765	2203.284	2293.474	3888.947	2770.692	1901.467	2999.933	3308.761
CCDC25	3769.021	2435.799	3665.138	2637.478	3910.869	2672.375	3152.451	3802.455	3243.503
C12orf67	110.6511	75.93452	69.69841	99.06709	77.55721	83.52261	66.48959	59.13824	233.6938
NDUFS2	277.0261	2806.308	5068.081	2418.256	5291.651	4021.211	4727.026	6018.187	264.5345
UBQLN4	9884.601	9267.275	8664.339	9978.991	8688.415	8666.087	10462.2	11115.57	11252.28
SUV39H2	343.8238	152.1414	94.87107	135.7869	89.27963	92.50797	78.37778	107.4567	412.9915
DLX3	360.1355	1105.537	495.5519	983.291	3064.255	1143.274	1081.089	755.0025	285.9703
ZNF628	40.29508	33.6657	36.89316	33.96981	239.5392	42.76728	49.35089	92.96744	43.17752
PKD2L1	36.73795	52.02483	43.78556	40.52014	39.56555	44.53479	97.10266	43.47601	50.16356
ZNF789	106.811	442.1298	497.0363	46.8022	510.7834	375.5186	381.7628	412.2925	35.11759
QSOX2	310.6263	214.5862	614.7776	174.9377	735.3661	753.7662	1140.384	1224.328	216.0761
KCNE3	72.4945	76.48678	207.7956	35.06814	155.7056	36.15939	95.11952	192.4234	36.67864
CATSPER1	72.38656	560.1743	305.7975	53.49348	224.445	251.6252	405.0313	405.6678	48.37107

FOXD2	52.33078	199.1001	117.2248	50.44917	167.2997	279.2335	91.75021	226.7089	38.04053
C22orf25	54.32594	712.6019	856.5174	44.94042	1391.003	48.97079	1168.92	1451.158	44.90655
GSTT2	83.82542	76.80681	69.60626	68.94855	60.97503	105.3183	50.81563	43.09076	150.5356
EXOC3	86.563	65.30102	419.7262	122.5111	272.7195	82.14418	588.7247	287.2575	115.59
C1orf218	8987.64	8767.619	6126.069	6566.195	4061.89	6214.912	5049.012	5545.562	8702.329
IRGC	68.48312	44.57183	48.54127	75.70155	51.75237	51.70627	40.42295	49.76758	82.1143
GNL1	8298.529	9047.37	8013.664	8588.975	7936.571	8661.464	8680.51	9461.966	8640.993
SERINC5	34.1412	40.54353	32.01286	36.05514	184.8456	34.26276	90.82847	36.5278	34.50572
VMAC	567.21	348.0689	81.48264	158.598	129.6329	356.2764	107.1088	143.9689	543.3212
ZDHHC8	22326.16	23679.25	21359.22	25138.14	23703.61	22029.87	22096.22	16653.18	21401.97
KIAA1875	3960.597	5191.986	3195.793	4127.623	1421.974	3857.834	2365.39	1669.751	4648.284
GPC4	43.43295	35.02335	38.77643	35.94945	170.2206	42.36525	151.9937	51.3538	45.02795
ITPR1	48.48547	55.49971	273.3456	44.19979	291.0003	84.71087	39.81349	35.87962	48.16335
GTF3C3	85.88895	237.9417	1064.174	81.38476	1009.906	854.235	1122.915	733.705	229.4724
STIP1	46.41538	74.39454	702.9687	834.0436	1567.792	73.18153	1361.785	825.6355	37.34507
FLJ42102	32.91232	35.34189	36.9871	37.13536	32.60609	47.92831	37.62961	210.8475	41.31231
DHDDS	1029.098	890.9814	842.8738	260.7556	821.2655	603.8707	597.1509	750.9722	634.9949
FAM164A	58.12885	33.52932	47.22124	43.87017	46.09237	45.40185	89.31571	113.2338	42.03414
ETV6	9911.472	10325.93	12782.29	10768.72	11793.62	11316.25	13122.43	13560.69	11950.91
NBL1	4398.855	2370.603	3371.175	2160.301	7821.536	3095.902	5801.669	9092.704	39.87342
NUPL2	232.3172	148.0451	264.1072	166.7225	275.6025	504.823	404.2895	206.4957	262.7623
FER	58.12336	71.60167	69.30992	95.85104	481.2003	385.5758	327.6557	263.2707	2255.855
MFSD2	41.80999	40.31077	34.42821	708.0479	37.15059	44.02421	35.14247	34.64291	38.1897
FMOD	248.9718	193.5677	545.9069	169.7441	792.0538	256.6893	585.2229	475.5769	112.2473
ABCG2	35.21497	385.4918	45.17307	44.38733	42.71397	41.7828	91.17482	39.3829	65.97903
DNAJC5	326.9436	197.5111	100.0707	225.8347	99.36538	160.7783	163.4192	121.1146	517.6277
SCGN	59.56652	65.23784	41.84671	52.14547	42.57977	54.30911	39.32856	39.11446	69.65498
OR7G1	147.5788	160.4462	83.27087	124.8224	90.90852	84.57006	68.5248	101.4261	167.3048
AOX2P	97.77763	74.82801	68.09623	60.96351	66.98602	64.94846	66.06898	77.92368	198.7324
XKR7	136.3535	103.361	62.12033	71.36395	69.01826	50.92842	56.193	61.29659	55.72771
GPR21	85.98462	112.5429	118.8415	93.42217	69.06742	61.39921	91.45226	67.9054	158.3764
GBE1	52.34876	318.3395	455.07	52.9619	589.5103	337.0173	457.1608	238.4067	57.81813
TMEM189	47.12589	40.44849	42.61649	58.64264	58.39184	168.4682	42.62599	42.32957	41.25744
CLIP3	17241.9	15894.67	13153.34	12340.25	11875.07	14552.6	12028.3	13509.91	17973.08
MFSD1	3383.526	2835.774	7301.798	4349.091	8941.033	7272.04	7313.393	5719.178	1619.816
WDR74	146.9742	857.7992	525.9058	91.46259	342.7714	484.5555	261.5693	65.73013	2577.657
NUAK2	679.1444	76.24649	465.3311	60.95146	210.2779	48.23992	165.6357	61.07238	259.9919
C11orf37	112.2778	52.25926	33.57946	43.52529	45.24725	61.87097	42.28915	41.48428	60.81432
OR51B6	42.57388	41.61425	51.45292	52.70047	36.77446	53.44857	44.03518	46.71769	36.40401
CFLAR	14562.2	15340.38	12231.96	10587.37	9433.535	10515.92	7990.669	9763.454	14695.73
GTF3C1	81.15938	40.92263	49.98351	50.58715	50.84341	50.44318	175.1736	381.9692	77.78947
ITGB6	2161.127	3151.685	2836.768	4146.918	476.5243	3140.584	2386.956	2501.043	43.64388
WDR35	54.30435	683.426	193.5394	64.24062	148.1615	58.45935	44.60748	39.042	98.65965
PSPN	41.73985	46.10505	49.88926	47.88686	41.33393	34.27954	42.68914	30.68667	70.73572
PAX6	104.2873	74.64091	51.33485	83.56687	89.71559	78.72227	59.91591	79.35739	83.63061
TBC1D9B	39.23256	603.7507	302.3831	33.21041	923.1719	325.9503	343.8494	283.8091	1700.055
C20orf166	2376.811	4005.669	1199.517	2399.669	240.6849	597.2083	1027.082	2515.957	5101.598

PNRC1	1293.695	1038.188	791.5206	167.6354	162.43	60.138	449.7424	151.0032	86.01263
ARFGEF2	1281.807	1566.214	1289.426	1781.482	932.0753	372.1102	802.7455	902.907	40.76362
43528	41.40313	59.62993	34.1691	37.36182	38.47154	34.27069	220.3747	38.74454	41.91961
MGC39606	98.44838	70.84293	41.10371	72.94117	55.35335	55.22268	51.0711	47.81421	85.74406
SLC30A7	12301.91	12043.4	9457.012	11306.19	8267.84	9667.652	8890.93	7878.199	13444.4
CYP2U1	196.2563	510.2575	280.4174	132.1361	285.4034	271.145	258.2535	234.0517	185.0992
METRNL	328.971	241.9241	79.49774	256.3499	94.69285	121.2207	68.94118	75.45405	461.246
SFRP5	36.78068	42.84548	44.51068	36.8296	35.98481	44.63865	35.43964	44.88496	43.98984
NXF4	73.88972	176.313	63.95182	106.5528	93.57701	146.0479	69.75867	97.65176	321.5967
FAM105B	1890.995	5016.2	4703.971	4242.167	3981.928	3633.811	5196.732	4006.717	3477.016
C2orf61	219.8956	136.6318	64.57929	70.26459	83.84067	89.09529	62.17985	54.00423	69.40996
C11orf82	101.3948	57.07596	176.0447	76.14688	148.8747	37.45601	94.51022	112.8353	86.47761
SUMO1	70.32166	41.12188	41.45316	61.52454	53.76752	54.45019	44.6385	41.15403	138.8214
THAP8	45.09396	35.43354	106.7476	40.45844	40.25484	39.85986	37.85627	38.12223	35.24951
C20orf20	18549.97	16427.48	14419.97	23242.23	11812.69	23623.44	12879.14	20466.01	19289.38
PHF21B	203.1523	141.4249	67.53239	175.0317	76.18607	96.51142	68.80056	96.95451	256.7399
SULT1A2	56.11059	45.87624	35.76385	41.02998	53.49302	44.10177	39.92357	33.08867	37.98354
KIT	264.5883	203.1822	95.66426	124.5452	101.8289	141.071	160.9073	74.68148	75.97601
C1orf35	177.1815	224.1674	529.4333	243.8887	562.2451	154.7752	230.6531	113.2812	520.663
TXNL4A	89.18494	86.01962	42.95279	84.19427	164.5978	53.33154	45.57719	51.66558	58.17549
IGFL2	36.37194	44.63505	34.25588	34.56155	34.29211	35.46068	408.5323	45.72923	49.90613
FURIN	1610.748	2173.605	799.055	1473.399	412.1916	707.8333	480.8965	941.9262	2316.349
ZNF181	37.37968	76.22472	51.32273	64.73591	34.84835	55.60931	44.68915	56.14261	42.82021
OR5D13	86.71454	103.012	44.47071	89.80915	41.69297	57.9192	37.90298	54.92462	175.3618
F8A1	1034.154	836.8665	1148.998	1032.398	1027.32	1683.682	1741.385	848.569	136.5928
KRT36	138.0675	66.66179	71.78799	88.17398	62.02388	69.96809	40.26595	45.18828	221.2956
TAC1	40.43095	36.8847	51.4659	42.03917	41.94194	45.92381	40.49176	41.59202	35.95323
SEC23A	51.42919	39.98504	37.74164	48.79974	469.2079	460.9603	241.2582	506.7793	85.75823
C7orf63	2413.94	784.1309	875.1818	1617.388	1925.897	734.3718	784.475	974.4978	1256.01
RAB26	180.9313	133.0416	321.2178	145.6046	947.4185	91.68825	552.8739	809.1304	107.2882
DDO	47.98569	41.59118	40.42621	34.82407	35.11517	39.06969	38.91007	57.70965	60.81287
PRRX2	5127.145	4109.532	2281.589	2531.954	3492.717	4453.677	1850.594	2863.366	3231.223
TPRG1	75.63818	43.96058	416.918	50.47569	63.87534	55.27161	114.7248	42.79745	67.1752
PIGZ	990.031	812.1122	429.7345	859.6199	37.45113	45.14442	228.4626	35.5412	50.44974
RABGGTB	489.6977	661.6769	444.0566	636.9224	411.3365	322.3734	453.2269	358.3496	830.545
RNASEN	670.3929	1211.584	951.8607	961.6046	971.2852	1399.182	1492.842	1535.103	931.9753
ZNF574	61.07368	48.98375	40.45961	81.73833	115.8279	58.53562	89.36581	102.2802	83.10173
C6orf125	101.3445	954.676	1079.837	702.9694	2114.481	986.6271	2189.24	3417.448	57.09975
SLC25A28	11170.41	11949.01	15512.71	10217.62	11069.92	14406.81	13599.97	12260.29	11209.07
KCNA5	183.7428	306.2378	53.95296	131.8683	127.35	75.69891	107.9864	115.8344	218.2811
PHF16	389.5963	416.4537	278.1593	249.9925	226.9039	456.2695	239.277	387.4667	481.0858
LUM	4088.428	59.09684	744.725	35.1148	680.9831	1472.408	1020.057	776.7184	45.63681
PHACTR2	59.62533	70.07568	250.4571	79.54211	322.9276	193.7283	151.6308	293.2166	45.81406
C16orf75	212.6951	128.8096	70.19638	79.02367	143.2436	92.55383	171.158	96.51161	164.2885
CCDC146	38.38355	38.22997	35.70495	37.22745	41.75859	53.68079	36.75043	48.38382	37.83696
IL1F10	48.57575	33.56138	40.08475	756.5416	515.444	65.90146	93.21151	278.0868	50.25259
METTL13	86.66315	54.55193	59.78517	372.0507	97.31348	54.94442	43.59951	67.02895	105.2583

SLC35F2	80.27136	62.01247	234.5154	65.68775	328.6743	402.925	125.485	311.0511	62.86694
TFAP2E	55.95873	50.34035	33.28598	50.37711	227.5139	39.99474	324.2904	33.93884	36.93169
ATPAF1	1075.038	417.5357	1985.362	817.6517	2007.077	1617.914	2440.344	2394.356	135.2354
C11orf80	6877.892	7828.529	2188.405	9678.024	1693.329	3015.273	2271.968	1081.812	9496.868
HECW1	295.1788	183.9273	86.6244	188.7494	83.2832	124.7338	78.03942	87.95726	400.8525
C16orf63	221.9108	82.8152	742.6931	112.8082	1075.792	1132.575	927.6043	734.6061	327.2641
GNL3	2124.225	1697.694	308.2715	105.1199	236.9015	609.9611	252.4693	56.09461	3519.458
PDE6D	168.9037	55.71194	170.0379	98.62415	240.6727	217.2487	253.0371	171.9978	180.5507
CRISPLD2	33.99592	57.97182	731.8344	39.69533	1075.261	46.09345	766.6544	142.3308	38.65526
OR10C1	57.24334	38.85649	34.09493	39.69245	40.47861	41.86869	41.25438	47.71189	37.99257
C1orf189	499.3832	329.7138	221.571	345.4959	210.3542	289.6512	185.705	272.2878	473.5307
OR5AU1	35.72921	42.25004	39.56474	38.49737	40.3339	46.99022	38.03774	36.8512	58.80165
C8orf31	71.97616	62.1266	52.43371	350.0201	93.14111	51.57619	48.03502	43.64826	40.90718
ADSL	1258.506	325.7367	643.5682	402.1569	790.2083	1020.929	1511.26	1367.64	1101.419
POSTN	3361.265	964.7505	751.863	1528.247	248.1286	1700.44	968.7664	703.7311	1185.415
PNMA2	102.2527	84.55738	64.99692	42.74254	103.1809	103.0967	57.88569	58.74429	181.3078
DHRS13	5642.504	6716.965	4322.871	6542.142	3477.93	3746.667	3419.752	3347.281	7345.636
VMD2L3	54.37938	130.0923	64.14477	69.89577	52.72344	52.03277	55.53075	59.35092	57.01356
VPS28	83.28893	56.30328	2847.946	195.9814	4728.151	847.867	4747.846	4224.705	58.20018
XPC	107.9234	49.90519	2118.692	266.1573	2850.119	1716.136	2306.4	1686.565	79.16189
HCN4	98.18481	145.3177	63.13214	53.79542	53.83552	70.32388	51.17254	60.97099	344.4206
NLRP11	53.3106	96.31307	46.69353	43.71384	50.40847	60.65457	41.98523	46.29114	65.45754
PLAC9	2548.356	1864.184	281.6353	50.93898	965.204	40.79058	187.2586	515.3076	4097.461
CKAP4	157.5319	88.97172	59.26738	90.29837	60.41662	70.31264	59.7408	140.6015	139.2536
LOC284009	48.70888	35.209	138.5908	51.7897	44.91	92.94343	34.82768	71.218	46.60944
NFE2L2	2683.822	2205.681	1448.866	1690.66	1136.878	1080.168	552.9391	704.6074	2288.808
HOM-TES-1	36.07418	918.5388	660.1991	37.41962	330.7897	46.42244	334.19	39.57475	31.24827
ZNF236	46.47517	53.64349	275.1596	34.52552	293.4269	53.45851	98.15073	35.73233	41.65707
LOC653216	34.94255	40.59337	35.81758	79.80827	55.64648	39.21382	39.38309	48.61757	38.55044
C19orf50	39.20354	475.964	2046.264	937.1392	3047.667	939.3563	2880.161	2276.344	55.67682
TBCB	843.1483	1766.87	2688.187	1465.786	2742.012	2524.463	3615.448	1854.849	1048.406
RINL	42.87976	49.26808	130.4091	513.3582	179.4131	39.06653	192.0157	134.0407	49.8173
CDS2	33.97728	63.83297	489.3587	48.74031	780.2985	1179.758	667.3572	207.7826	83.97896
ATG7	9220.552	8703.981	9783.983	8804.982	7467.934	9907.91	9351.508	9960.502	8992.009
dJ341D10.1	330.2512	198.8741	170.7987	336.1182	146.8841	212.2767	157.7371	286.0447	668.3634
HUWE1	1652.209	1541.963	520.094	2061.386	1010.845	1708.918	952.8486	1001.299	1840.324
OR5K1	53.52922	35.68221	54.52765	58.62817	35.57812	44.15026	37.46799	32.80615	39.64494
AHI1	45.57333	466.059	1070.47	51.35398	1166.772	308.3407	1053.159	827.0709	79.5996
NEUROG1	45.39919	80.00943	40.58463	82.64019	40.32745	35.65943	45.26537	55.78419	71.7442
RTF1	46.99834	36.14887	59.49356	106.3041	134.9555	32.73108	76.15857	214.1903	60.58521
FKBPL	61.29555	64.40863	78.55048	77.5896	112.332	35.79913	43.18929	41.30961	63.86882
TESK1	150.4692	78.7211	122.3417	50.56398	131.1034	67.20931	142.3457	66.04178	102.7776
ZNF513	286.2855	177.2417	214.8406	190.0434	405.5322	117.6747	117.2829	76.58756	329.0903
SPRY3	34.15947	835.9495	39.48669	62.90867	137.7679	305.6439	110.9034	42.06641	45.96076
SERPINA11	81.48436	34.23073	41.3701	108.9152	55.2148	49.88369	46.24923	377.0288	36.11821
RSBN1	603.1415	81.78699	438.4818	638.9169	719.0513	828.1229	596.2737	750.1802	241.7691
TRIM6-TRII	36.62836	85.41794	83.27574	46.65592	90.20904	103.7638	87.92451	79.40872	47.77168

HEATR6	66.52045	60.57114	896.7482	305.1517	2106.05	1750.049	1992.76	1434.003	93.06648
TMPRSS2	77.5556	47.29831	32.09002	37.35962	325.5344	36.36741	114.6096	44.45045	39.79088
YPEL3	96.83022	67.80627	57.17041	95.90363	308.4452	1574.255	144.9574	290.2538	176.3388
MYEOV2	254.7567	141.3311	54.31409	164.6198	75.82077	113.3181	79.22538	101.0482	308.8124
ARHGAP18	2054.8	2852.573	3057.094	2890.955	1625.567	3034.371	1877.563	2024.449	1538.623
MUSK	98.3833	63.93166	57.82651	199.7364	73.20499	115.3877	69.93922	54.74533	179.5732
SMOC1	32.56928	33.12454	41.37242	38.60274	38.76657	35.65918	31.47755	67.63766	33.8158
ZNF490	280.0689	300.2543	126.4211	51.52372	35.14674	179.0676	104.3321	69.86324	38.10756
DOK2	57.34708	278.6234	563.4345	57.15055	396.5888	349.6978	351.0746	734.2232	219.0751
FAM40A	273.48	176.7725	171.202	158.6541	192.1422	161.5883	174.8292	221.0691	230.1719
GLYCTK	2423.416	567.6224	296.8335	133.5676	274.1079	83.76598	122.1963	151.5293	1875.762
NXN	1014.283	919.7629	1941.262	2371.134	4343.662	2382.931	4918.063	6102.333	390.2731
SPRED2	483.466	241.112	208.7596	294.9148	261.4685	379.9278	282.3461	251.1916	662.8984
FBXL13	41.097	44.7123	38.0323	40.23243	39.28973	54.92158	40.13516	110.7044	56.02522
CDH10	75.03123	37.2847	64.14602	43.88947	33.26901	61.17358	32.9608	54.55202	50.75867
HSPB8	6006.5	6257.416	6391.406	4080.179	9882.044	5471.824	8284.816	8340.971	6909.632
EFHC2	46.41925	293.7382	152.9343	237.3412	572.4781	346.0634	148.4488	98.75797	907.489
C9orf103	184.7944	1167.284	859.6304	121.6052	657.3563	628.3931	617.658	953.5487	209.112
STK39	252.9869	697.3077	1518.997	436.7187	1507.491	1450.273	1388.459	942.2706	163.184
ATP5O	56.51122	53.43124	125.8257	55.90508	129.3892	59.15434	95.54578	91.39254	57.34414
RCOR2	76.14964	359.0545	266.5808	1363.099	291.5945	334.6332	935.7352	250.3954	41.0686
STAG3L2	45.16379	1008.299	1423.266	244.8783	1564.881	818.2795	911.3542	1077.052	1181.755
C15orf43	183.8753	57.82343	70.28687	109.6846	73.52825	82.25503	57.94554	73.43652	235.8142
ZNF787	4754.535	4689.551	2311.71	3702.067	1388.496	2424.641	1623.401	2808.831	5094.781
ITGA7	56.80832	179.0386	67.10408	86.64809	63.35479	124.4166	62.78144	60.76897	135.5103
LOC40105	1002.035	977.7365	364.9708	183.8373	1188.849	1922.253	650.1962	1029.233	60.99467
EZH1	397.3828	223.4143	108.8534	226.2069	91.61758	161.8338	87.23892	94.65773	495.1199
MBLAC1	60.91306	59.98496	456.5435	41.68843	312.0384	40.30679	191.9375	477.8922	67.797
HMGB1	10232.34	10001.16	7288.642	9954.338	7821.409	7809.669	6273.355	9240.433	10058.43
PDE4D	76.33218	1190.804	42.86234	54.53846	155.8637	53.71468	142.0993	223.0406	1737.91
CHRFAM7	141.4887	89.83301	64.14767	63.66717	134.5069	104.8671	59.28452	55.44513	232.2812
TMEM176	1563.528	1387.166	356.3279	63.54771	292.8835	727.5245	312.2556	676.6078	2570.699
ZNF497	101.5679	108.2071	55.18294	87.33031	79.51499	59.53569	60.82944	84.34953	63.22563
CPA5	137.618	114.711	79.71474	111.1786	56.29153	74.85755	57.80085	69.1963	325.8015
FA2H	42.76481	37.55029	213.4584	47.42747	37.04752	37.54916	34.84356	33.85785	34.61857
KRT23	1973.915	4755.47	959.3829	2625.08	1146.741	959.2002	553.5573	475.09	4491.893
SPAST	39.06084	160.0479	148.777	89.20243	116.5414	156.0081	98.81018	85.87605	34.96909
CEP57	52.83331	67.9245	102.1485	133.966	92.36983	157.5706	121.9713	104.0388	89.57964
PLXNA1	88.99471	178.6031	1089.93	728.285	1638.023	2376.995	1643.928	1603.33	1139.875
HNRPLL	39.78439	33.96074	432.7567	1792.46	1869.041	1799.266	1813.808	1180.606	919.6877
CHSY1	11045.47	10112.99	8499.94	8996.14	6653.913	9518.59	6952.313	7774.661	10830.8
OR2Y1	39.76028	50.41914	64.87352	40.16393	38.34455	35.98604	39.03155	35.92949	52.84626
DENND5B	44.32647	55.76232	44.12402	57.69661	52.23588	58.19131	42.7375	96.67787	55.20336
TSSK6	150.7856	81.34052	82.55333	121.9373	93.28628	85.94354	124.6923	58.42652	255.6126
ASIP	3094.21	3786.844	2922.181	36.90254	925.5682	1586.744	1272.766	718.6127	4786.354
C5orf36	75.66559	33.51068	54.98561	42.96195	40.15992	44.83662	39.89291	35.98163	37.18876
TRPM1	34.79955	34.69186	111.0639	49.91171	33.20566	36.92523	33.67751	35.44566	41.14486

C10orf137	74.13303	48.79037	556.6918	43.67221	1118.041	536.8635	1068.342	387.0296	59.10408
TAT	54.00041	51.8504	57.63028	65.09875	53.81674	63.83574	104.5263	68.07115	43.78591
DNER	35.72755	36.91064	32.1877	33.46318	33.75937	39.71561	33.02409	35.07767	32.63556
BMP10	36.63923	34.95253	33.13028	39.16071	38.38651	35.70847	36.08746	36.38075	53.58444
DUOXA2	638.2596	74.87432	108.9097	66.74081	127.1362	203.8335	137.9046	103.5477	1327.954
KRTAP21-2	110.8275	40.69717	43.59921	54.94171	35.3174	43.59084	31.90755	57.54186	65.27233
TUBB3	9984.492	6017.514	15236.4	15914.9	20199.14	13152.92	22402.64	11785.24	3445.696
CXCL10	1570.708	1445.785	1831.588	2920.681	89.75443	1044.151	1998.596	2363.899	2052.577
HCK	241.072	155.9023	3684.77	1085.631	1996.729	1636.755	4259.899	2725.368	2225.775
OR1A2	165.2818	69.52324	59.3462	131.2332	55.04354	85.71581	53.18818	77.10172	83.82443
KRTAP5-7	83.20617	60.00291	49.43947	62.09244	140.4651	65.76469	49.65524	55.5252	54.96448
C12orf5	33.9265	607.9812	1419.711	1065.986	743.2401	572.4893	493.72	392.1571	60.04455
ZNF768	978.2874	138.6186	61.99552	429.7766	132.1761	122.2893	80.70621	101.8249	236.5168
RAB25	7658.298	6720.077	11567.05	6235.812	13636.12	11976.47	9624.205	7591.636	836.5746
PCDHAC1	124.1995	92.7606	56.44987	91.38192	42.64577	85.51902	54.82644	58.17925	156.9828
LYG1	38.45592	55.50574	219.9534	50.58353	60.96928	56.44331	150.8505	43.3273	37.16833
RALBP1	2888.651	1481.706	1928.39	3161.611	2473.844	3512.376	3118.353	1990.23	1245.147
DAPK2	43.84171	41.43984	41.46939	52.70686	180.0116	791.3908	108.4114	42.28632	2180.365
EPB41L4A	433.3103	1052.485	137.3258	265.6573	345.5535	395.0631	117.0116	121.1508	537.4326
COG1	41.22416	35.14305	44.46279	615.2233	134.2029	50.08087	96.29588	254.6376	40.35846
SNHG11	4864.229	5015.061	6035.17	5550.344	4666.381	7324.671	6124.595	4782.157	6665.117
SPON1	61.08506	43.92285	1370.585	64.00241	2395.392	1342.919	2385.573	1713.961	175.597
CCDC103	50.10478	258.4183	205.7568	133.5318	178.7086	390.141	290.1497	92.56517	98.22179
SIDT2	9068.917	7935.653	9508.069	7893.251	9657.941	9398.053	7632.237	9766.1	8657.387
NFAM1	442.4562	249.1132	114.3512	168.5669	102.9863	202.4306	118.0642	106.0768	289.2998
CCDC82	3521.277	4643.055	4194.32	5624.854	3546.216	4798.821	5319.073	3660.254	3113.025
PGBD1	82.58154	58.76638	361.1009	36.39732	175.4653	42.11187	470.0721	36.66723	45.08942
KRTAP13-1	72.37383	51.1606	52.36589	75.4258	33.52193	76.5757	52.82505	57.18365	96.88704
C20orf200	42.98232	44.28852	54.27091	37.02533	37.69497	35.25723	40.38826	40.14872	52.752
FAM70B	514.3359	257.6582	162.036	289.4519	251.2059	253.3186	169.4481	240.1281	686.1877
LCAP	145.9618	96.32352	66.1512	70.20303	58.18063	69.00951	59.57364	60.67242	201.1499
LRP2BP	235.9618	181.8385	89.96259	136.7188	222.5857	100.0679	68.90554	91.03276	378.9716
FUT4	38.26762	59.30213	159.4521	543.2122	36.49257	59.44162	128.3702	38.59995	58.32028
CD22	250.1314	102.5748	147.0219	162.3113	75.99284	127.7108	133.8022	108.8043	163.6176
RNF114	2539.997	2744.491	4760.805	2711.908	3671.127	4232.006	4620.688	4253.446	1127.071
C1orf101	207.4998	115.7839	73.67683	132.0975	60.47978	105.3311	63.89714	95.26086	379.7097
ZCCHC8	2339.665	70.52554	919.647	1243.84	149.1041	381.7223	379.3797	359.9914	2063.818
TMEM63C	272.1389	179.3804	71.971	187.4602	873.7469	114.4741	188.3468	185.1821	408.9592
C10orf76	119.6167	51.09527	257.4705	39.2308	1488.972	445.2042	855.8864	315.539	49.42832
KCNA2	36.01723	208.0485	51.44984	40.50086	33.00348	46.72863	40.42383	42.46957	48.1796
TCL1B	53.25418	50.40782	43.33512	68.88112	41.98844	48.06121	53.43201	43.56175	54.52705
DEFB126	59.66546	63.12708	46.07867	53.88026	32.8469	34.01465	37.61451	40.07453	37.39079
TUBGCP4	1927.037	1721.775	1397.442	2018.523	866.0962	1547.816	1079.896	884.1674	1061.121
RDH12	93.58199	59.82902	685.5438	63.36258	2867.894	53.8605	421.9698	730.318	187.0371
KCNK16	162.207	65.08163	69.5874	62.36371	46.73951	80.85426	43.71411	66.85272	68.48514
MLX	594.2688	363.0607	1077.774	396.2214	541.8798	526.2966	510.3319	634.9125	48.37452
C8orf44	47.97462	48.73111	45.4208	598.9605	172.9664	65.29996	115.3775	225.8988	102.2388

OR5M11	51.16849	34.87276	45.16958	40.31051	41.55701	34.26068	38.29455	33.08176	37.01995
TBX2	644.7467	281.0804	165.957	334.8245	680.7677	307.3584	473.7693	516.5341	49.52798
TRIM4	45.0347	74.36046	32.52727	59.34583	63.97802	55.5298	47.19413	50.7248	40.37291
C10orf46	59.65841	44.1098	992.9527	51.4756	49.89917	55.9031	140.1785	413.7013	76.01036
CCDC149	63.36103	472.5559	326.6196	45.22723	539.6844	799.5858	583.85	458.9511	69.07961
RPP30	34.63369	48.07939	63.02648	37.30689	53.28222	112.4176	96.47988	119.035	34.97527
CCDC83	36.59011	44.8028	34.73376	40.37372	33.71067	36.38201	35.74914	41.55264	36.58126
LAMB4	449.8145	247.2666	67.96839	176.2405	98.07497	138.6649	64.98682	73.55836	347.4252
ZNF318	555.9109	590.7278	1022.049	2063.327	953.6633	1309.462	1415.55	655.896	61.77419
ACTR1A	12735.57	11541.26	10082.37	12172.49	7237	9684.151	8249.173	10147.12	11979.7
SLCO1C1	269.5739	235.0828	112.4601	246.8069	105.208	98.68786	101.0397	116.6544	471.7087
HDC	12003.42	12867.93	10310.91	6410.251	10096.1	9219.28	8298.529	9237.073	12349.38
PHC3	61.38437	45.91183	36.44038	829.3127	42.61219	316.343	131.0999	41.96882	38.03009
DPYSL4	3397.047	1684.269	700.6141	1017.79	320.2587	1012.758	606.5491	1051.427	2210.099
KLK4	34.53328	691.0087	137.4622	734.6368	162.521	878.3378	196.8778	935.2707	70.00934
PFN4	128.2958	93.82974	59.73577	109.3952	52.23511	85.79527	136.9905	65.47475	64.29211
HABP4	397.5812	1913.997	1354.39	878.8174	605.1293	881.1978	1180.599	1192.271	312.3258
RNF165	5783.438	3467.659	2475.378	4381.166	3732.206	2640.425	771.2119	36.98511	2552.156
CSN3	77.30009	52.81321	43.6591	45.9066	37.30215	34.93875	39.78246	53.53736	37.82609
TM4SF4	1161.157	70.3995	216.2173	33.69732	151.6293	42.8519	80.90601	233.9157	42.20383
AMOT	89.28581	95.9866	57.59853	87.67695	94.83151	182.1992	84.37918	65.0009	61.15073
C3orf39	43.39694	32.65632	36.62366	42.46164	1133.79	595.8859	474.3611	577.1657	34.08918
FRMD7	50.33628	36.62141	68.29646	30.84315	62.50132	62.02349	41.61528	75.86391	40.98126
KIAA1920	63.49249	61.38745	51.62755	64.73003	44.16195	50.35579	49.6137	58.63772	112.7909
STAG1	580.3502	308.7678	203.8871	342.5784	242.4328	284.7378	145.3089	146.6321	50.84118
LOC34817	60.84578	44.98957	48.33328	46.78749	40.56704	54.1035	46.30305	56.59023	41.6048
ALS2CR11	38.59395	126.4082	41.89924	64.01247	37.40613	33.71584	114.7533	50.94805	1417.22
TMEM158	12585.01	11030.5	8018.408	16157.94	13072.53	12040.86	14664.11	8256.381	10025.74
CCDC104	1269.44	880.9905	219.2045	1021.12	396.1612	332.9547	159.9689	507.9156	419.2597
ARPC2	5407.976	6361.116	14094.22	8133.271	14789.59	10194.78	15219.05	13366.45	4061.352
HRB	1692.792	65.02055	388.5934	744.8244	842.4749	484.6835	659.1175	784.7925	76.39989
PLA1A	42.12797	43.59289	48.02187	41.28777	35.68554	44.66487	40.53308	34.24939	44.97518
TMEM59	5677.806	6229.1	7551.982	7357.303	9969.777	9225.846	11102.22	8457.969	3585.029
STX7	1208.177	887.0151	1831.541	288.3315	1298.16	1084.715	1018.265	1059.382	695.681
CD7	13810.52	12528.62	12220.69	7920.242	3150.053	12621.66	6951.015	9287.445	14155.01
C1orf175	2083.614	1222.507	1237.723	561.1777	1178.716	1441.786	746.6235	1538.05	36.45508
KRT9	8442.844	13944.42	3857.017	8229.03	7567.025	7006.868	7264.864	3975.899	6492.129
ARHGEF15	996.8602	168.5815	88.13094	151.2759	145.2699	107.6901	84.23007	214.279	1370.971
VPS4B	37.30911	746.3173	1919.768	1544.39	2341.066	3042.814	1383.244	1370.55	164.8591
PMCHL1	354.0012	166.5045	88.8598	203.0294	93.51611	128.638	81.13837	100.8698	449.9507
CRYGS	108.5543	83.48759	117.4425	81.52456	289.5124	91.37291	160.051	315.5233	119.9778
OBP2A	90.59001	59.06942	70.54964	93.00117	65.46101	85.75089	52.47528	36.36605	247.624
CAMSAP1L	1084.222	2008.843	3517.786	3359.408	5766.988	3832.107	3932.878	4354.792	572.2016
HLCS	134.5344	638.9195	167.6187	65.80243	345.1681	86.24045	94.36768	208.6192	211.1863
C12orf29	184.5649	75.93269	141.3347	128.1252	255.6821	177.6686	150.1796	131.6182	49.80916
POLR2D	72.86443	88.56016	187.6454	88.17551	208.159	81.96758	337.6491	257.2949	65.1021
ITGA5	18749.38	12339.15	14433.44	21949.13	11655.9	16695.38	16525.16	12057.95	14666.38

CCDC86	1875.063	2480.557	4031.128	3339.556	4313.907	3689.961	4906.292	4122.585	2567.286
MCTP2	57.24808	49.50721	36.92108	45.17961	41.60249	39.61916	38.38945	33.10714	41.30953
HIST1H2AC	35.17616	39.91312	39.20998	741.6077	154.3225	40.47742	77.74822	196.0843	47.7271
DAPK3	145.3261	106.1874	317.9916	823.2495	53.38708	68.27648	221.7162	83.94988	83.88761
LOC201725	43.01043	55.1773	51.42081	34.82063	949.1299	691.4427	451.1991	388.4928	42.98194
TDRKH	62.86337	60.98672	55.56266	56.67729	65.99866	59.87155	55.34092	43.64663	77.07245
FOXA3	54.84856	52.96302	48.64769	41.74498	115.2442	39.75527	41.57977	164.7606	55.48906
COL21A1	42.84301	78.53436	33.13361	47.44862	39.82712	32.97145	37.48678	35.18793	39.35609
SLC13A2	271.1468	169.2348	90.85515	137.5789	66.98405	112.6871	70.98301	91.31441	193.4341
CPS1	131.3962	77.57263	129.5666	107.8097	145.5227	56.54757	198.8549	37.8166	143.1491
LCN6	64.28387	32.48523	46.87454	34.20852	36.79189	35.44443	44.89079	41.79767	56.54645
TMC1	36.45206	46.50377	49.54108	53.80003	38.67642	40.04784	39.63272	38.8343	49.60223
NOC2L	4619.666	3739.663	2041.26	3880.313	1413.264	1556.272	1263.174	2479.258	5087.807
ARIH2	205.2154	989.6261	2456.775	1106.947	3688.524	1723.467	2487.094	1723.097	272.7934
CIAO1	67.76772	46.22877	174.4901	61.99194	43.19635	42.13581	46.2094	43.68731	57.78803
MNT	8651.616	8337.928	9842.863	7714.844	11407.7	8755.807	10176.54	7267.401	2810.487
SOD2	11990.9	7117.071	7468.515	6999.096	2511.826	5209.032	5011.088	3883.674	8180.643
LYSMD1	654.5817	91.58809	52.87802	188.1925	168.6151	194.3226	91.2326	128.9862	99.36118
SLC10A6	45.51126	34.94266	37.5106	37.73732	61.31466	28.89988	40.55852	60.40594	43.87988
ESCO2	464.7881	240.1885	118.538	181.2263	119.7685	95.30713	113.6776	74.36662	571.8795
ZHX2	50.51256	139.6295	219.8918	97.82646	329.4558	101.7633	274.838	96.99499	82.78365
IRGQ	41.97325	36.60583	49.19349	48.32062	41.07609	34.39289	36.84259	316.5328	86.63061
D21S2056E	230.3456	75.78817	59.90151	70.31958	66.19921	117.2274	45.48951	59.17735	190.0475
ZNF439	56.17209	462.8222	1342.393	63.52375	756.8817	898.3585	1352.753	662.0826	67.92574
RFC1	318.1323	178.9812	175.1727	227.8694	248.459	232.1349	161.5727	150.4596	535.0396
ZNF202	963.4073	430.9604	175.8858	51.37123	363.2076	51.14911	201.5418	186.2953	1327.004
C20orf196	317.8963	1149.654	2033.207	758.2921	1408.474	2220.887	1713.392	1465.212	116.0658
DGKQ	872.0657	1248.066	1903.283	503.878	1451.047	881.6933	1250.974	1285.683	701.5789
OSAP	39.82975	46.54793	42.92967	58.47689	57.75156	66.16937	51.52671	56.19095	71.10529
RBM28	480.9522	291.0161	449.2795	313.8374	1236.146	223.9619	568.1843	781.195	651.1296
ADAMTSL5	334.8186	156.103	92.79967	186.1647	131.4952	228.3895	112.6002	78.02971	440.726
PAX4	125.6188	81.47556	75.29496	115.2816	63.64042	74.35237	59.70803	76.47812	261.5298
TAX1BP1	12944.27	9979.18	13793.95	11962.55	13214.72	12620.54	13608.96	13312.69	11100.92
DCST2	31.57711	42.12792	39.26733	35.87171	125.9605	43.33435	34.60657	36.05685	34.7642
OR2T4	52.98966	72.7957	51.55085	50.02696	39.85285	48.69484	47.37991	41.65148	102.8385
SLC36A2	2089.608	8643.883	1860.065	1127.393	1771.061	4116.874	5570.9	8310.577	3187.486
SMCR7L	6681.437	9877.426	13044.37	7821.409	13238.09	11078.5	11734.85	13631.79	4312.799
SRD5A1	61.12855	44.98694	209.5295	47.43649	302.5968	59.99035	678.7281	176.8713	114.5443
C1orf94	279.7055	163.1267	80.77305	144.1999	71.4017	119.5391	64.60043	157.7893	232.3616
ETHE1	146.8191	87.42793	186.5679	305.3696	188.5894	374.1635	164.3093	149.8805	70.92926
WDR60	926.4304	1936.961	1606.805	799.2468	1670.248	1843.492	1621.532	1272.149	139.662
KIAA1529	44.42362	34.90191	41.04066	33.46584	33.66126	36.98928	40.51237	34.82295	43.41228
MOBKL1A	898.4901	1561.623	744.4684	1048.367	405.7815	673.2097	547.6745	553.5948	1232.896
LOC643842	45.76686	45.80213	45.99402	39.29063	38.69225	66.82013	57.0696	64.95813	53.59074
KIAA0430	2734.801	417.115	1266.126	1891.244	784.0032	1518.057	1358.897	931.4035	54.87101
KCTD16	59.75417	150.4328	70.74934	70.42907	43.07445	53.70467	46.46668	69.49059	50.3248
JAK1	1623.455	35.92353	249.8036	800.7316	203.4789	843.7245	405.1913	40.01228	61.86735

MED12	1791.523	1334.071	927.4167	910.5327	2245.76	69.41458	1195.364	1596.255	55.97622
OR2G3	47.07264	41.45331	299.4258	45.49166	40.37284	48.44816	44.65112	43.35561	36.83702
SNTB1	2156.678	1746.13	1491.168	1459.145	735.7291	2132.371	1228.135	1911.978	1528.927
RAB10	3718.831	2888.6	3360.204	2675.669	3863.44	3945.354	2507.692	3003.302	2288.116
PRKCDBP	12498.46	11277.79	10454.52	16562.33	10050.74	12886.45	12931.24	12726.28	15438.59
C7orf36	64.78255	465.3582	467.3519	74.33105	708.0629	54.64497	463.3309	275.4502	1108.808
RPTOR	101.1089	94.79825	292.6975	98.61957	77.70231	77.15468	54.1183	48.66813	956.8757
RASAL1	1869.187	1510.163	3278.781	565.2727	2300.634	1424.269	1964.712	2437.816	547.8788
PRKAB1	1069.48	35.10667	1904.542	36.72908	3240.786	2045.076	3479.156	2815.627	39.43778
GABRQ	98.06136	41.86363	68.4892	64.99077	169.0854	59.08913	50.98716	52.58694	52.682
LOC389031	125.7396	62.78154	58.98247	70.275	57.77173	57.04253	64.31155	88.71404	103.8848
MUC15	108.6456	85.23243	540.3724	62.60806	711.5359	507.8743	244.2448	195.4233	217.5123
C1orf51	35.88012	41.31502	38.11446	34.13101	141.7186	36.28239	283.0678	37.32797	39.78773
PTPN4	751.952	1331.24	758.1266	1543.916	597.4085	1131.322	1039.361	717.4401	829.0917
C6orf204	32.90742	39.98615	42.42763	45.3378	60.79844	40.43348	38.65902	38.07966	39.16982
C11orf72	47.69918	61.31836	50.19624	89.41894	37.75574	40.75965	34.36981	33.17649	52.56975
ZFP37	1583.906	1250.861	691.1268	618.9398	1032.188	867.7419	850.4026	736.5636	999.0615
RNF38	306.3762	151.7093	439.4452	543.7267	593.375	766.8668	772.5344	211.9076	184.5401
ZFYVE27	75.57787	67.94989	71.16815	77.98225	72.66154	89.48277	67.42204	63.10093	87.01367
SERPINA10	303.6295	163.8241	93.7914	107.9786	85.1963	126.627	82.47562	87.968	309.8322
SLC4A3	50.50805	62.72096	55.73576	934.9471	123.3102	214.7084	110.4891	156.9621	832.8809
TMEM222	174.6823	633.8178	905.1992	738.556	584.3875	495.8415	659.8399	1221.817	97.4086
MRM1	535.6129	36.35543	42.68026	49.37436	40.91259	45.84587	56.9084	32.58403	29.72105
UBE2N	1120.975	633.4954	1566.602	1065.28	1331.806	1849.139	1330.189	921.285	789.7941
TUBA8	76.39094	518.736	104.7977	102.5375	39.20052	54.85945	47.80098	42.69574	67.23989
SIX5	3980.953	5182.132	5269.497	5882.268	5248.042	5666.567	7760.565	5355.988	4077.996
ZNF766	1102.429	1203.268	380.5536	494.3391	458.1735	732.803	487.1136	258.3244	1075.422
SCAMP3	11874.81	12604.63	14841.18	12568.2	13756.98	11970.76	15780.25	17525.03	10781.94
KRTAP10-8	35.18865	40.05517	34.77086	43.23118	52.57095	39.97411	35.94126	43.67867	32.53878
C3orf31	45.03813	56.20705	268.1888	42.9526	526.9927	117.0202	185.0682	242.9533	49.59863
C15orf39	103.4804	49.61787	113.787	48.67337	189.5861	65.09868	588.1898	145.6456	70.42484
TM7SF4	35.92792	39.69539	40.23517	45.56661	43.49266	40.43887	45.22161	42.92928	36.78936
FARS2	141.4046	1377.504	275.1644	152.1731	536.7506	88.81876	771.4496	1116.266	124.468
DIRC2	6486.917	6606.638	5468.858	6345.578	5675.447	6407.497	5685.926	6516.803	6462.749
DNAJB6	53.18671	48.10176	167.4756	42.44727	1152.154	46.14721	461.7659	252.2911	40.80938
TMCO1	4937.958	6592.394	7246.443	6683.185	8755.281	8233.421	10229.54	8598.081	3848.117
ZNF542	548.7187	329.2603	243.6502	307.3648	106.7203	170.2567	119.447	186.9599	662.1938
INTS1	43.50038	284.2982	37.38121	53.7363	107.8687	190.9326	66.14618	132.4323	41.62958
ALDH2	911.8453	1841.096	1229.623	41.43568	1670.808	1060.309	859.9315	1453.533	971.15
SERPINB5	1454.632	1090.07	2475.87	3247.08	3478.339	2313.024	2682.363	4088.531	1265
ANAPC13	219.6036	387.4474	546.9492	388.7658	1309.82	1288.906	1603.748	919.6155	768.222
KCNT2	38.88805	49.19361	44.54375	48.3636	147.1098	34.33199	81.09875	70.59515	38.09016
PISD	61.36992	70.91798	191.38	53.14893	158.2019	44.90694	157.5209	51.36449	44.8596
ADPGK	394.68	228.5431	235.0944	439.1849	720.3205	176.1475	455.5392	331.7309	527.959
OR8K5	129.091	109.8462	66.857	134.6577	67.04186	67.34467	57.7416	69.66225	70.33486
PCDHGA8	50.94805	103.3839	45.00832	54.15816	43.90296	305.8106	38.64214	43.84094	57.58277
BOLA1	542.3861	55.56666	93.23247	48.08462	86.58966	149.0523	44.64244	43.3686	88.43393

ID2	57.11049	341.4821	492.3124	337.8072	295.4864	592.8044	243.8605	37.4798	54.31972
CHST2	373.2932	277.3474	123.9394	296.9166	142.9202	194.0603	120.0021	360.0583	484.3185
SERTAD1	178.478	567.6048	1878.381	1032.769	3143.564	1462.414	2765.335	3839.004	154.166
FAM49A	299.8835	126.8299	242.9889	129.971	141.2136	151.1271	167.2106	107.1116	236.933
MTX2	1128.039	274.6035	176.8516	587.641	443.1736	242.6538	313.9986	403.4571	400.2523
ANKRD27	62.47966	42.50079	49.50999	40.47987	40.89511	142.1597	50.35427	146.1616	39.0965
ANKRD13B	43.39035	41.16058	56.68232	66.06406	52.47868	48.47039	75.48669	55.02199	78.17134
SAPS1	2252.772	1163.002	991.7797	1184.95	1763.289	650.3273	2105.924	3042.919	249.1483
HTR1E	298.7148	208.7998	86.69928	195.8312	92.53567	143.7285	73.61749	100.169	461.7855
MED9	221.402	205.3261	152.6454	181.4486	222.9318	196.2791	133.3175	193.9317	312.2825
C3orf56	55.7983	47.54707	43.65845	65.02246	36.75601	42.12792	40.2741	36.31736	41.08533
GAPDHS	50.63374	69.65302	90.96934	48.06876	38.91831	49.68657	38.19435	44.20137	43.3561
ZNF79	35.716	31.90816	562.4833	32.80654	168.6053	34.48981	282.5884	754.1963	32.02793
LOC38960	827.9937	337.0015	655.4919	519.7868	108.22	50.04065	137.9258	380.3244	707.2402
NDUFS8	11618.62	12753.88	13787.89	16249.55	13395.19	14355.78	14626.63	15316.92	11251.7
SMCR8	1726.899	96.65346	591.8723	364.8862	349.9141	770.2214	191.9883	770.873	133.3815
LOC37532	107.2476	92.37888	44.31264	112.1073	42.76624	77.96674	47.92525	49.37651	247.4201
TMOD4	316.343	386.6211	182.371	207.752	286.107	1206.383	1276.331	1593.638	263.9906
PGD	94.56358	194.8204	381.8518	60.80899	281.4699	53.80487	119.196	151.2088	54.12909
FBXL3	35.47202	35.8199	39.65668	38.23457	59.69621	33.92725	44.49516	33.67547	38.42495
CCPG1	3686.245	4588.198	2547.421	1500.734	3077.396	4102.735	3968.556	4960.323	3310.463
KCNJ12	35.50612	330.5856	31.18598	32.2561	43.9913	37.28078	58.11446	61.45013	47.87194
ABHD14A	43.63005	44.2469	967.1638	69.89182	1076.46	743.7832	1136.984	935.9634	58.38592
SCAND1	198.26	93.02594	73.30138	114.1834	208.8888	136.1515	103.489	122.3733	113.3092
C4orf26	84.71455	49.27679	185.3523	54.97006	49.27478	43.26958	40.22326	52.15878	56.37188
NDUFS6	86.60445	43.50121	64.55769	54.12613	36.25374	42.6837	34.3151	48.51631	46.42335
USP28	5323.162	4388.614	3797.92	5082.922	2529.283	4160.816	2581.413	3764.403	6764.691
POU2F1	170.0561	59.1697	466.7623	103.1327	206.5029	464.985	541.0398	654.8486	51.73481
XYLB	757.8572	59.19928	115.1125	69.9293	97.72541	166.9514	161.4889	69.76505	632.6117
ZIM3	179.0905	85.65847	72.37276	105.6731	66.99076	66.89375	64.2073	75.24765	129.4729
CTSW	1371.723	299.5579	409.928	328.9652	111.1516	1018.208	217.0998	398.074	1119.736
SEMA5B	47.14635	44.47983	47.7064	45.14257	33.92466	36.31874	65.87012	37.60728	57.85418
GHRH	52.4336	57.28876	54.39132	46.64303	42.19439	71.25961	40.46107	49.01363	41.88077
IL2RG	589.3555	37.23804	580.8088	53.70115	42.22417	396.5354	130.7732	270.9747	737.509
SNRK	1633.096	774.4199	821.1584	1307.268	461.2002	275.1981	510.0006	127.1632	1093.458
QPCTL	2888.714	1941.735	265.1447	2169.627	234.8717	65.74548	264.4475	735.0371	504.0379
FLJ10781	269.8144	136.2796	64.25033	138.6058	151.144	105.7494	453.1997	138.2638	216.5303
SHFM1	347.1213	1620.126	107.4383	200.8149	562.4058	139.0332	192.6808	608.7422	500.8248
ADPRH	257.083	132.2097	82.74238	116.5883	68.51968	116.4328	72.47749	53.18839	174.234
KSR2	64.13867	59.12106	54.74318	51.38419	47.57651	54.80611	45.19553	42.6811	89.36244
EIF5A	1315.02	136.2783	652.0159	457.9534	1235.064	865.8189	1413.492	784.309	244.3069
CCDC81	147.047	80.06616	154.5263	40.56192	185.0326	52.35558	192.6456	49.54991	125.2789
KIF11	51.88444	66.31087	45.61955	61.15226	62.10135	66.90513	115.3606	186.0319	111.7393
T	61.49102	36.51532	35.21145	45.85671	54.79047	42.74796	44.14745	42.50992	75.93513
GAP43	2586.999	641.5181	655.8311	2526.894	2232.635	2112.822	1721.155	2183.805	62.52386
MRGPRX2	45.80639	35.06023	45.44819	37.85084	43.88947	42.0598	34.82469	45.09098	35.56859
RASGRP2	249.8921	218.3368	108.52	178.9488	129.2777	173.8396	102.5842	106.7108	313.0234

SCHIP1	87.50269	908.3361	268.3744	66.16984	207.435	46.49951	522.0739	340.3028	57.23569
SNRPD3	71.48774	424.8112	39.2451	526.0513	201.0143	32.65102	107.6777	281.9962	41.23976
UBA7	16366.36	17772.44	16197.36	15157.68	9909.901	15395.55	13348.83	12630.11	18572.62
VIP	3274.146	1146.565	1147.557	1261.631	1326.505	1465.84	1527.556	2900.447	1532.324
FUT2	35.29597	39.47594	33.63225	35.39503	42.39881	38.05593	31.51797	33.86718	60.52575
DSG1	896.0532	520.4954	307.2591	718.3915	751.8701	454.4483	282.4903	542.9703	912.4083
TSSK1B	420.7181	285.2401	126.097	231.2279	112.037	198.7705	110.1081	159.7408	629.6241
C9orf47	38.60273	51.39318	50.90381	762.7586	46.60929	45.01047	38.13857	60.47984	48.966
TMC6	2204.131	6219.79	8663.862	5711.86	7360.65	8114.457	8993.413	9132.361	4681.898
IL9R	123.364	53.78012	194.0132	56.05881	34.85517	54.54055	41.45218	41.67057	34.98494
PRR10	48.84973	33.21676	35.17506	33.92316	36.45499	36.08706	38.36282	38.25678	34.55401
ATHL1	2803.275	3209.093	2004.254	2887.583	1094.119	2115.048	1108.842	1063.704	2230.087
RNF167	1581.89	2091.628	1841.78	1468.196	1672.875	1869.414	1493.624	2333.318	1748.926
KRT20	130.3062	94.2077	79.87093	144.1256	60.48449	117.1336	59.76596	67.31015	163.3136
FLT4	49.94373	55.53841	61.28945	107.6266	55.02532	53.23399	52.93601	69.26814	45.97907
HES5	597.6652	2360.961	2185.466	873.0197	4389.286	1656.263	2801.1	3335.719	1484.476
OR52N5	226.9629	134.6665	82.17033	108.6592	70.62309	113.1495	59.64718	72.64668	208.4872
SHARPIN	833.5976	121.8239	130.2903	154.9525	79.51143	203.5994	100.0966	137.0853	104.3158
VAV2	352.5485	789.1167	152.6911	396.1067	151.7398	325.8287	383.6322	278.3842	909.9684
NDP	2673.204	1273.42	578.1531	1958.815	1039.28	3495.486	814.0663	919.3434	52.83015
CHRNA3	95.74879	42.69159	37.52655	55.06918	84.79096	129.3795	57.51046	37.23027	47.86071
LHX8	35.42982	35.90893	48.91082	36.05101	111.4878	35.87081	89.90226	40.05974	42.41782
ZNF626	54.50566	76.10607	36.86206	57.70539	44.13633	241.5659	107.514	39.59959	45.61513
C6orf117	49.11533	43.28664	39.78292	37.36673	46.85183	35.70155	34.58855	43.40448	40.51408
LGALS13	42.4015	36.32484	46.58608	34.52971	33.10363	32.98071	34.5771	36.2714	34.04789
THRSP	48.72062	1223.601	30.46449	45.64044	1352.243	309.9353	219.7961	224.9342	80.16834
LRRC4	112.2384	115.943	86.62978	86.25678	70.97005	80.94157	63.48862	74.81698	52.0745
CAMK2N1	1494.556	2004.168	2024.317	3784.862	715.8678	1417.179	2743.585	628.8365	1789.15
AGPAT9	589.4202	2164.353	995.9664	571.6251	1537.425	1035.849	609.9232	1190.031	717.3726
FRMD5	177.9508	96.38824	50.69421	130.6046	62.16608	102.7195	102.1866	116.8962	145.1242
SLC38A4	291.2237	75.83988	96.30307	356.8025	108.4273	279.6118	366.921	113.41	579.6451
UBTD1	108.1474	49.39099	41.43003	36.46844	36.58641	32.68126	32.96639	294.3134	36.15501
MS4A10	55.674	55.61861	51.1326	40.10333	89.63247	85.70719	47.63282	53.89717	231.4776
LOC90925	107.8473	74.64678	54.77516	74.07378	97.03963	60.5383	100.0318	57.8702	67.23248
DSCR1L1	214.8508	94.44874	80.55806	88.60589	76.57077	57.11057	51.8138	81.68678	353.7752
RGAG1	64.1162	74.13212	52.69529	1127.909	54.50805	51.28617	44.63207	57.47206	56.59684
SLC39A5	534.0088	410.834	157.6327	403.5951	93.80041	127.9671	128.7663	74.82475	954.7676
BBS9	1025.22	1358.642	169.785	49.21325	227.0833	297.9366	207.2815	158.0217	58.18987
SLC35F3	72.34206	49.55749	35.41979	70.22433	53.11301	37.53849	380.6447	40.01147	75.60711
ABCA4	79.97814	514.2792	815.89	382.623	330.6404	2251.722	1305.983	684.7741	58.17463
EIF4ENIF1	286.5717	185.724	150.5262	346.9669	316.2089	231.841	183.143	464.8359	360.2952
LRRC28	1101.398	2126.268	4041.007	3112.354	3290.708	2542.416	3598	5235.604	151.424
NUDT4P1	34.57716	37.85264	50.80592	1335.965	47.83043	601.924	55.33588	42.80547	63.7073
C2orf47	36.65971	42.7472	33.03059	51.28063	411.9253	40.75077	189.8822	164.0392	37.8083
OR7C2	266.7021	172.8813	84.06948	209.2735	99.62759	132.5683	98.42462	106.2188	428.2165
LRRC3	394.6022	85.5362	67.97942	97.2323	71.16052	100.2373	63.63791	65.91632	259.4583
ZADH2	94.91812	46.84575	415.4682	58.76653	1181.511	89.25236	203.5231	65.95129	63.50535

CCDC116	57.40591	45.2439	42.08683	40.72988	34.86837	40.72723	37.89479	46.72475	66.68326
ACADS	53.38522	59.0636	60.9158	88.76417	57.69723	65.80022	53.10347	54.24495	70.55361
PSG8	39.47876	42.78418	39.87545	48.04883	40.3156	33.23668	35.65825	32.23148	59.81483
SIDT1	38.17775	48.83424	35.01705	735.5475	42.94957	49.9327	37.98766	58.02516	65.65668
MYH3	2128.764	4836.307	7992.815	3619.5	5300.813	7959.399	14834.8	5028.955	35.0038
C13orf23	80.92393	771.9883	42.03532	41.03939	178.3083	68.18504	42.89446	57.75387	52.20863
S100Z	35.3202	35.45724	48.75608	41.44184	89.62981	32.54999	37.01828	32.26242	30.81735
FGR	1157.399	1853.07	1947.256	560.1743	860.5115	986.2278	1971.729	1675.414	1436.585
PTH1R	1681.722	1700.567	799.3854	1138.941	1078.934	1149.384	708.538	949.3987	415.7662
SLITRK2	2041.812	2185.581	886.3766	780.0884	1034.223	546.6938	540.8887	513.9905	1685.782
OR5C1	241.6109	109.282	79.1925	150.2779	71.69543	116.1865	69.06222	73.47043	263.1638
DDX60	1956.225	7056.657	5110.035	6965.046	233.4549	3846.718	3141.848	5112.798	43.77642
RNASE1	3434.019	2094.19	7012.397	1613.033	7258.067	4198.54	7497.962	8621.964	3954.812
CLDN2	102.5038	96.87869	71.3075	73.60838	55.42463	76.04598	54.08888	69.25746	255.6251
DHX30	65.9749	106.9217	211.3782	94.07466	213.7616	102.042	66.81764	52.32872	92.69569
ZFAND5	5183.789	3053.193	3157.277	3823.967	3110.457	4206.568	4133.429	3749.495	4527.453
SIGLEC9	88.4015	54.86531	53.40944	103.2005	148.8391	76.3624	100.0385	53.3826	61.22194
ARID2	64.61282	50.98642	224.7213	60.62703	38.01721	59.43037	34.25666	46.41492	39.65095
TNP2	35.02237	37.64434	49.46305	40.11553	35.78591	36.50496	34.67905	35.05786	53.57014
SPARCL1	948.2684	742.9247	442.4904	1103.844	693.3598	56.03018	695.9058	385.8867	79.44067
GPT	50.20677	46.54206	46.88844	52.70844	41.00174	58.14519	47.24052	364.1307	40.84378
EMILIN3	81.42874	72.87384	40.58989	45.97418	101.0972	55.37465	44.02676	37.80278	61.99472
USP53	686.665	93.38537	52.76322	541.2128	124.9252	99.88966	172.2522	254.111	118.2676
PHF15	49.3158	327.3276	252.8786	48.252	156.4859	262.0922	251.1583	242.0558	60.8914
C6orf47	1372.275	2391.819	2106.234	2042.413	1967.032	2321.395	2067.121	2138.846	961.6093
FLJ41603	614.5832	1073.358	1114.279	1736.121	1444.027	2389.108	1813.945	3789.048	74.05935
ACTG2	20695.98	12971.78	8705.358	10623.74	14859.75	11010.23	17966.18	7334.655	11912.17
YPEL1	197.1218	119.5909	371.3776	117.1953	198.8077	712.9127	228.8719	90.36047	179.049
SFRS1	3455.599	3033.009	4088.345	3731.6	3199.178	2631.126	2693.541	3893.11	4289.984
MLPH	139.7975	1255.743	954.5418	131.1815	1554.968	1643.109	1101.882	1186.205	108.9301
GOLPH4	50.33562	64.02468	1054.01	939.4016	2099.113	1069.439	1768.776	2203.463	44.58886
SPRR2A	9175.403	5965.901	7693.603	4427.766	11489.2	3344.315	4540.062	7564.497	8910.722
RPUSD3	268.2978	906.9849	2275.957	136.6647	2374.552	3209.854	2985.097	2397.201	2732.157
INSM2	1022.528	392.4399	472.5631	60.3227	135.5924	661.8354	250.3862	459.7148	42.52849
SPOP	1309.825	43.20655	186.0759	34.60426	467.9305	358.1852	546.4309	342.4707	39.22722
C2orf24	72.27831	127.6697	273.7516	1005.49	408.2509	68.19333	444.1837	50.19685	257.5184
WDR59	1558.454	1677.247	2522.404	543.8989	3444.234	2172.254	3080.478	2399.519	471.2095
KLRA1	31.1231	34.82673	59.61207	37.49016	56.89223	46.76601	51.55769	36.7037	42.0324
ZNF264	860.7956	107.074	745.7062	106.122	812.7531	570.3597	627.6943	505.2708	155.8761
LAMA4	51.31067	46.17646	38.63544	41.0075	289.2279	40.81024	181.8131	160.8608	49.31348
OR2B6	81.44602	73.6097	45.40004	60.09104	41.07898	60.49781	37.66011	55.23323	52.85148
CPSF6	48.08474	363.9576	259.5837	408.2951	184.334	42.04982	67.80246	227.9433	60.47248
MORC4	247.0021	175.3796	96.11491	153.4344	95.51861	125.856	84.60187	95.51591	328.9239
LTA	8943.909	8357.751	6733.387	3896.604	1831.841	5055.918	3325.72	5851.818	9871.63
PGC	85.27189	50.38269	47.6955	35.43745	32.57633	44.65868	43.51876	35.01504	37.69942
ERCC5	3241.955	4175.443	3983.708	3378.644	2785.515	3338.046	3058.118	4478.812	2956.286
MAML3	2011.746	1280.784	801.032	557.4648	441.8002	270.6319	337.3123	359.8214	2616.379

MCC	585.8774	60.251	106.2722	69.63645	78.25001	60.50387	54.07262	132.0771	276.7488
C3orf25	95.72835	83.65031	83.4481	144.2	85.13352	111.7687	81.26173	93.69279	272.9798
C11orf84	240.0445	272.4514	145.5767	95.83584	147.1364	174.9442	184.7131	131.7385	408.5797
CHRD	512.7565	250.6766	332.6091	48.49842	491.1185	585.3877	408.1616	382.6292	151.0811
RAD51	34.7974	38.75798	382.1245	1246.697	197.16	785.6528	34.51434	44.71986	34.90648
MGMT	5386.499	5023.136	9588.229	10015.95	6794.159	7915.263	8151.402	7938.653	4506.513
C16orf82	33.04663	55.75555	35.72815	43.81894	38.03981	39.19572	35.0092	42.91656	39.29515
SNX24	51.44118	36.98534	56.3678	61.39819	49.63062	48.86283	45.69521	60.40092	97.86253
C9orf173	48.05027	40.5359	36.82853	45.77673	62.74362	42.38044	41.60692	37.9015	44.43206
POLR3C	2376.71	2754.734	4485.843	3782.862	3703.477	4303.111	4662.308	6038.19	2019.815
STMN2	138.2138	111.809	73.31196	68.58942	59.35072	74.32262	53.89579	37.57587	54.02073
KIAA0232	60.45263	62.04072	52.61271	43.34661	103.1349	254.9244	85.01726	51.24006	44.14503
WNT6	85.57732	94.51981	49.09667	58.39705	67.73891	90.23282	69.75337	44.35939	73.38327
LCE2B	815.2355	5876.503	134.9473	1905.159	3344.067	1578.443	577.0802	1099.814	2796.143
DPYD	752.024	409.5619	266.1713	423.5514	94.22563	233.5611	117.2653	173.3518	699.2175
MPZL3	67.32517	1324.186	774.6556	573.0544	990.8079	709.0107	239.9036	551.9758	3218.479
CLIC2	312.1859	203.3799	375.7433	457.7862	436.5781	143.7478	218.081	523.4026	349.1371
CNKSR2	115.7106	75.99284	79.52183	103.211	68.16168	69.82554	71.21323	102.3842	97.95733
PLEK	2107.362	2254.675	3573.698	1899.764	1302.889	3781.202	2243.806	3061.433	3100.894
P4HA2	46.25063	70.8116	214.923	52.12875	96.26633	296.8231	205.6334	615.7028	3398.039
HBXIP	45.54572	176.6214	55.68652	76.52203	59.49234	76.64912	58.54457	76.93433	55.61948
ASB2	406.9611	285.2091	137.1123	306.835	107.3055	204.057	130.4888	203.2771	512.2859
DIS3L	4476.459	4588.293	3177.652	3931.063	2187.53	3667.728	2918.458	2652.354	4687.336
ATP6V0D1	133.2026	555.3574	197.3911	592.2141	650.8642	68.36461	444.866	385.4883	105.2742
C4orf19	72.90392	85.46983	288.0945	87.31569	262.0425	245.8797	111.4009	58.48026	69.96556
SCCPDH	428.8772	250.4513	147.4764	275.5089	197.013	252.98	215.0813	269.7802	293.1529
GUCY2D	49.91304	490.8055	63.55791	69.67959	66.43016	75.86678	50.40554	71.03475	76.57908
COL19A1	50.86407	1886.317	512.5706	443.9031	124.5011	51.2805	179.2848	722.5557	65.88473
CHST8	61.83226	52.2561	75.30581	95.56534	60.43527	78.03712	55.18838	62.30578	94.28927
FGFRL1	283.0139	481.9887	356.2805	166.6099	435.4796	473.8309	346.6103	342.7068	333.5769
UGT1A9	82.77235	56.88392	61.78938	54.65509	251.4122	111.576	47.26595	40.63188	64.54362
TMEM167I	121.2993	168.4115	91.94617	176.864	228.9176	101.3801	230.6003	103.4532	150.8453
ZBTB4	473.6685	176.6412	147.455	274.515	212.5232	182.3808	176.8448	212.3561	579.2715
TTC37	233.9156	241.5402	399.8575	241.7791	263.2497	90.48083	345.3849	201.744	575.2981
CENTG3	41.6209	37.26818	46.51035	40.77046	56.67393	44.36414	52.80454	47.96836	562.9297
OR2M4	107.2992	42.31866	37.3851	78.57751	49.20401	91.76461	42.86702	46.44415	69.92709
CCDC16	1753.031	2807.108	1857.577	2291.59	1311.288	1747.674	1723.173	1512.099	2421.397
PTGER4	150.4729	676.5251	1093.381	72.96262	1329.424	1431.41	998.5433	618.6779	164.255
CHMP7	94.9307	382.4325	176.7912	102.1649	103.3289	78.02199	75.27086	123.4303	2324.513
C3orf36	2891.622	963.6287	1159.718	1071.516	1929.257	780.9964	1071.233	1167.145	253.2504
VIL2	6809.287	12051.87	19630.09	16526.11	24468.65	15468.91	26502.76	22364.11	9966.98
CORO7	272.0022	170.4479	94.38308	129.433	60.43621	67.13364	70.47823	94.56409	148.0838
OKL38	17620.19	10724.9	8393.559	10262.21	8108.71	9869.135	6891.715	12238	16580.34
UBR1	39.60042	36.52267	32.97734	45.40838	124.9842	34.92911	76.66393	107.0782	33.12725
SFXN2	53.01058	39.97073	38.66117	264.3559	57.59525	127.7146	84.695	37.13554	40.12969
C15orf33	35.12534	38.48915	42.69125	48.34612	38.13663	36.7051	40.32097	54.34216	33.27138
C22orf28	2455.01	1155.139	652.5779	889.1277	1246.107	655.8062	1304.802	1577.671	113.1521

TERT	44.50398	42.2994	47.54563	49.31335	34.7633	40.60399	35.65611	40.1346	46.10805
NFATC4	47.38214	1325.997	337.5279	51.06478	204.1342	337.6922	337.4285	249.7356	705.7866
ANKRD30A	38.36171	40.2318	38.90619	35.72906	36.84274	267.4317	70.8523	37.6581	39.39937
TRIM44	36.59069	252.0279	114.5674	299.2595	162.6732	133.3041	160.696	64.99842	37.10286
CBLN2	37.23489	35.43587	35.81507	31.62093	33.37534	38.38257	37.83165	38.24171	67.0844
C18orf56	9552.539	5714.055	5403.5	10320.4	3725.509	7913.432	9006.123	4947.11	7158.808
ACTL7B	35.76823	38.03883	34.30705	38.385	42.07312	44.96766	44.90799	53.46076	62.25249
NKX2-3	63.81813	67.06086	73.82459	70.26445	47.14914	47.61818	53.56053	34.97061	119.5701
PRSS37	54.33949	54.83396	54.5666	56.12903	50.02002	52.39542	60.46465	63.60982	88.48048
KIF13A	52.19536	44.0402	173.0248	480.2531	180.6041	36.01402	172.5817	35.89724	41.14083
BCDIN3D	185.1322	404.0114	1647.242	66.30923	2746.145	1792.889	2388.726	2584.851	102.4642
ZUFSP	298.3277	185.9627	246.0044	219.7306	220.5915	140.236	305.7341	147.4137	207.9056
EIF4E3	1293.934	406.9291	198.1269	629.2516	171.6003	34.97157	98.10327	38.74419	3210.421
GLRX5	10173.54	10021.18	8518.41	9449.255	6712.749	8163.743	9006.461	10300.95	10127.18
GIPC1	5246.911	3424.479	7640.165	4188.857	10297.48	6043.454	7334.655	9162.292	3366.545
HIST1H2BK	2727.78	3210.262	2068.732	2391.836	1419.749	3142.904	2667.435	2858.228	3026.2
CRY2	904.9551	900.189	747.4053	413.5882	514.7512	804.8111	660.113	556.1794	894.1498
PPRC1	1112.389	3078.517	3066.458	2095.223	3869.553	4135.646	3816.085	2695.768	1808.166
VASH1	1600.376	3234.29	9431.684	718.5967	9373.307	6654.332	12904.95	6893.094	52.51493
FGFR1	46.23364	38.60391	159.5265	35.61231	236.6853	35.22228	82.64114	171.7195	67.43867
CIRH1A	7943.307	8295.571	12165.4	11008.09	12016.6	10877.93	12653	12990.21	9013.126
KIAA1012	317.6242	37.24624	623.2313	175.8753	618.3383	419.5035	437.2616	264.6709	36.44064
CD2BP2	1638.181	2866.917	2180.219	3084.832	2513.949	2547.996	2588.038	2461.27	1270.91
DNASE2	1272.36	1643.959	2777.867	2329.958	1990.642	2509.698	2631.993	3111.617	812.3002
CRTAM	53.64588	636.5056	209.6721	58.62788	52.00298	238.5641	111.8606	56.4357	1094.166
CSRP3	4637.432	3628.669	1292.535	1280.083	960.8619	3007.874	2414.798	2355.924	6704.813
MYF5	176.4053	162.2646	78.07201	162.0466	68.31691	123.6848	59.82439	84.00528	130.319
COL5A3	57.95874	404.1304	141.3766	35.04709	179.6327	52.31603	438.3515	406.7165	40.52037
MMP9	35.93323	36.01517	1285.706	2022.702	558.4346	1150.538	1641.186	4253.263	36.67586
LPCAT2	381.3172	286.107	114.7626	326.5117	135.8286	210.9469	127.7108	112.6212	235.7805
MAP1S	43.58384	71.50143	357.3667	56.15441	240.8585	42.33297	662.8606	874.5508	55.10286
CXCL17	9305.875	5114.257	7350.064	6303.384	5829.349	9074.373	3423.292	5232.868	10098.9
SMPDL3A	754.3888	385.2993	197.984	40.20964	129.7029	226.5663	279.6034	103.8328	1124.391
C14orf37	53.64395	76.74946	138.5976	55.54871	48.63153	49.91328	46.42513	37.96671	65.24087
CISD2	31.0232	593.4479	339.4066	36.382	1864.153	660.6953	1606.824	1949.164	30.27834
SS18L2	17061.91	16839.49	19762.66	17900.16	17478.87	18944.34	18635.32	15377.6	15487.2
C1QB	1412.086	1273.119	4096.253	1646.137	1984.241	1365.591	2691.557	2607.674	2564.942
TBCCD1	561.8825	382.6966	127.3403	309.5689	135.9708	181.1861	96.71386	168.837	376.398
ASNS	68.46743	5102.211	6676.373	2789.174	9840.595	3996.883	4456.544	5981.869	49.64416
CLIC3	2679.004	6172.492	8839.836	3203.201	10958.45	8326.53	6025.309	10108.27	6326.597
PSMD3	54.97798	61.23833	95.69115	234.0324	216.7724	120.4464	235.7343	197.9062	275.5011
GPR101	191.3061	206.6395	105.2436	230.0612	100.0366	163.8851	102.7408	157.8503	473.7725
EFCAB4B	197.5679	69.53562	41.41034	62.51217	100.0502	62.01226	55.18489	69.90935	359.0447
RYR3	2086.482	973.2118	549.7225	940.0878	742.1849	565.2977	1992.287	210.967	745.85
GABARAPL	277.9059	1450.052	2324.764	858.5672	4923.355	2068.167	2375.069	3033.219	2084.549
PRSS8	1687.07	1694.204	651.4034	1631.034	737.7257	1123.075	596.5366	805.4236	2601.919
WDR37	71.958	72.90313	398.4672	655.9778	227.1378	70.02142	250.9421	54.63035	191.8044

GDF5	99.34863	58.97334	60.58747	64.60114	57.04691	55.62555	45.03684	45.46033	54.67841
GPR6	251.8761	150.3382	87.39507	192.809	71.08389	90.22051	64.95199	76.39465	298.8378
GRINA	1783.83	1395.506	907.3493	1383.359	281.9109	750.5101	340.6977	186.8309	84.22335
PIP3-E	565.8683	257.8525	211.7348	282.0605	148.4206	213.8127	139.2345	211.1821	713.6605
C9orf43	94.12647	97.17033	61.89408	69.11879	279.9899	101.651	243.4943	185.5948	1150.418
FYB	36.93901	41.70403	36.21816	36.9762	151.2197	155.3735	75.4511	187.8838	36.95703
HFM1	48.10433	50.32021	38.23448	47.37064	33.13517	39.05304	41.77851	33.58465	34.65048
TRIP13	1358.213	1634.881	1918.421	2521.804	3867.619	1435.293	3494.882	1898.978	34.28259
C5orf22	76.12801	250.0703	51.1552	83.74446	55.21505	127.1192	76.24851	81.24117	536.0864
LOC64322(	50.36785	74.21664	47.11845	87.5724	63.05856	80.61722	49.39811	61.26007	59.993
ADAMTS2	239.6562	598.7296	521.9073	663.6646	92.67884	68.25082	176.7859	225.9056	1469.279
EDNRA	17034.24	15253.56	16535.83	16508.13	12647.37	16772.82	15302.97	16185.47	17618.76
UBQLN3	590.0006	412.6283	154.4646	356.5653	156.2959	205.2602	158.4042	170.5573	700.0217
NCF2	95.03198	108.4379	66.16346	101.282	254.9427	354.6428	650.5594	565.9513	145.2047
B4GALNT4	86.20723	456.5691	85.99865	721.0932	147.864	101.606	107.4706	115.6282	45.82259
PSEN1	500.7119	227.2523	109.8675	487.9576	124.312	233.2661	97.18671	69.1748	412.1571
RIT2	36.74077	40.84493	39.71868	45.41743	38.0723	45.19915	38.43114	50.64583	51.73343
RASGEF1B	66.34656	59.91929	48.56181	85.41136	49.67362	53.79379	45.32685	50.02143	136.1495
HBG1	65.28899	1128.057	34.84886	51.48892	302.9628	370.8676	36.26096	415.2763	39.30193
BEST4	48.76631	36.99205	39.35984	65.28953	34.64541	48.17222	236.3885	59.35684	59.15504
PHC1	73.41178	121.0656	412.971	101.4351	560.6119	48.81144	391.9495	844.0636	58.30124
S100A10	4458.954	4032.333	8008.466	7270.966	11159.4	8321.363	10245.85	8260.35	1951.958
SPARC	35.14369	43.46056	267.7037	34.8507	2455.722	1222.017	1500.586	1028.596	36.88844
NHLRC1	32.79433	37.36597	39.74382	36.33008	32.39518	38.2014	140.1602	33.47249	36.00051
KIF1C	83.76602	284.6403	367.3436	428.0706	730.0241	260.4582	585.8459	834.6357	105.2053
KIAA1274	2068.729	943.2021	774.4587	661.238	331.1333	559.3787	658.4846	554.5068	1637.784
CCDC102A	3764.732	2721.745	1948.612	3567.881	2435.176	3693.874	1610.514	2789.214	4106.846
BTBD8	118.8276	76.46925	66.36349	43.5153	63.63745	100.9975	151.0417	85.85219	114.4426
RLN1	33.03669	40.75731	36.04493	41.68956	45.67778	42.30875	33.87647	42.77335	76.84612
LOC39074{	58.73843	61.45061	56.01609	47.18668	50.41161	42.81184	43.38726	41.47743	185.679
PCDHA3	55.28494	51.85024	49.80506	37.11576	43.30357	34.21534	49.30796	79.10335	129.9522
MGC2752	173.8754	128.3695	96.34029	117.4159	196.6527	114.0101	85.66542	91.69302	296.2957
ABCA8	3770.19	2655.208	1712.367	125.0181	2423.176	1478.494	1269.603	1583.764	4927.358
RWDD3	77.59261	83.29647	73.37529	102.1455	172.83	303.05	119.5615	294.3946	48.07978
DCAF8L1	33.41987	41.26534	60.92051	46.24343	50.53761	44.1655	38.10996	43.04388	42.77514
NUDCD3	258.6267	188.6904	724.7156	152.316	1234.826	926.5549	1000.27	1666.752	268.9404
LOC81691	35.52978	32.52094	40.67088	31.1696	31.19834	35.70639	33.74231	32.29903	31.62268
CXADR	47.09752	36.65049	155.2974	45.9552	151.4218	42.78996	172.3741	93.08218	64.40887
PABPC5	442.9334	243.9287	127.512	292.3051	151.4928	273.9723	126.2133	128.1624	587.4813
ZNF449	172.8047	51.5974	59.45573	65.67625	47.94059	57.55467	103.9632	41.03316	71.99789
BPIL1	45.3982	36.24027	43.14669	51.88152	40.2993	44.32163	33.04084	45.19619	59.03929
TPX2	52.38417	122.2917	96.30314	528.103	62.24586	179.4034	201.6215	301.6611	43.39623
LRRC55	40.69083	57.08019	34.15538	46.21014	41.93331	38.85387	36.44221	37.599	41.59858
PPP3R1	304.5433	246.132	123.1336	180.0843	125.8483	129.969	145.8698	127.8337	301.105
INTS5	176.9368	1793.903	1176.55	189.223	1672.022	613.0203	2073.131	3782.046	247.208
POU4F3	51.25717	68.8188	41.1025	47.40078	36.66097	36.63866	35.26716	43.47422	38.38284
NLRC4	285.8518	130.6201	81.93843	118.4712	88.89078	140.2324	83.00053	97.4804	198.2098

DDX58	145.7442	301.5786	272.3753	100.1125	166.3202	455.7788	326.3416	238.902	300.3647
PGLS	2689.867	851.6982	1318.589	1629.004	1768.036	161.9193	949.3443	2053.988	60.31882
TMEM86A	913.897	1727.861	945.6418	1097.934	741.9229	1208.49	617.3342	1180.859	1852.066
HSPBAP1	6839.403	8008.967	5935.638	6802.03	4507.242	7712.331	6553.265	5397.817	6183.643
MTHFSD	34.91126	33.95514	34.86206	54.80649	523.9187	38.3444	282.5695	33.11998	35.19128
LIN52	505.6274	319.916	191.3136	337.8104	137.2394	206.9164	214.3897	181.5039	698.2668
TAF5L	602.0311	35.76939	38.41024	313.8918	153.9091	37.13297	80.64971	168.7136	35.31647
TRHDE	1196.666	35.42873	45.72486	60.02408	42.10139	40.15816	34.96943	40.39167	40.87074
EMG1	793.5256	1121.524	1753.408	502.0142	1956.58	1303.385	1872.419	2770.078	102.604
SNX13	68.47556	44.79905	96.68029	309.8993	96.00311	187.3891	79.02125	81.6253	55.09896
ST6GAL2	38.6123	51.88034	59.71575	39.38052	33.9954	52.71587	40.85488	38.13356	46.72457
C10orf95	139.585	127.3317	68.36966	126.0286	63.02842	74.26443	63.72408	68.54739	822.4369
SCLT1	76.91656	42.02846	49.4929	35.08778	52.30939	41.55924	89.60711	40.00862	38.31912
FAM45B	592.5374	316.4988	212.1344	261.3188	222.6398	241.5431	126.9269	245.7051	833.1396
AZGP1	175.9872	603.1893	103.3188	276.1793	519.496	159.044	170.3556	257.6442	552.8676
C17orf75	41.08623	57.92743	260.012	59.60956	151.9081	288.0604	102.9253	46.44156	54.00093
OR4A47	56.22237	49.17919	39.05957	76.11461	49.14013	57.00025	35.42026	45.82793	85.86681
LOC93622	95.60214	69.53442	130.3143	226.779	112.3506	164.155	109.2761	147.4214	77.08518
PKN3	1560.376	824.7323	36.65212	38.52285	44.91984	35.53932	374.6056	271.7038	32.49785
MAP3K12	336.4565	251.6252	165.852	219.5817	147.7269	159.5218	154.2992	147.9797	511.6228
KIAA0040	176.7943	139.271	92.17862	586.0567	308.6465	475.4814	313.6848	251.4612	130.4232
WDR64	94.90619	89.69244	62.4352	70.93662	59.64936	60.88282	44.41957	58.60745	132.0477
TSGA14	69.44995	95.91604	59.59311	113.4024	63.75126	271.0099	60.98798	50.57853	193.1854
CLCA1	90.97984	31.84532	41.88505	70.67046	38.03894	63.78522	35.94911	38.01341	89.67519
GATA1	46.95871	43.35175	49.71523	59.11115	52.47272	69.28369	45.87016	44.6293	49.53748
KIF3C	36.27126	40.81274	37.37519	38.58478	159.0522	38.50281	327.2639	164.3735	34.98133
SIGLEC15	73.7217	53.96545	47.38389	41.96891	36.97161	51.37703	44.74609	37.33079	110.9052
PASD1	51.05318	35.16841	635.0301	36.21793	42.67353	310.1636	37.87438	45.5717	58.46355
ARMC7	49.39531	248.72	195.5661	85.96798	89.35911	297.0899	236.4514	243.4861	162.8839
C1orf183	115.3975	76.99891	145.8172	398.3506	234.1764	61.94234	91.53329	134.1482	68.43214
BUB1B	371.0289	189.102	98.05573	264.6317	99.57961	152.9233	80.76345	104.4626	381.9382
ATP6V1D	317.9399	2685.002	8972.556	3035.336	15105.01	7941.678	11500.41	10358.81	504.4443
HSPA14	2615.997	42.98453	1062.165	607.2325	1341.871	374.0993	755.9291	960.416	1770.266
GRID2	64.47198	116.0991	53.99836	39.08657	48.34785	48.07008	50.3677	41.49876	98.14426
NARS2	101.0975	341.7805	934.5908	775.6078	852.3782	694.2134	1028.027	518.4554	110.7818
FNBP1	6679.593	10256.75	15652.74	8565.724	14685.4	15223.4	15324.33	9413.015	8873.842
C3orf58	33.471	391.3193	328.449	488.4003	643.5596	35.47359	316.0034	226.9629	36.2975
RBM43	38.98509	37.77827	38.91974	55.38918	36.14315	34.14298	52.03942	59.81433	41.35012
UROS	9964.375	10347.85	9358.925	9356.381	7237.814	9270.239	9478.176	7981.974	11355.91
SLC22A7	51.7416	46.29713	44.49828	48.14184	49.08354	53.72009	29.87148	49.29258	56.21832
OR10Z1	45.27189	54.30268	41.91368	38.16339	37.62582	69.85484	45.7726	50.41864	54.12997
NKAPL	69.23241	55.99342	46.47843	88.42274	48.3636	59.19178	42.48888	67.9373	47.74698
DDX55	222.2297	173.7186	489.124	150.6855	258.9311	473.777	399.6609	540.4279	232.898
AMAC1	267.0162	84.99641	99.98461	142.8533	108.035	113.6411	75.6713	99.01469	289.1407
C13orf30	39.83826	39.08207	39.95309	30.49899	38.27834	32.34014	35.56713	51.07688	37.8937
GCDH	1305.274	115.447	183.905	1479.648	417.7824	480.7246	329.167	202.0276	1793.088
DUSP3	15251.73	13942.26	14068.17	14167.12	12086.96	11368.28	12003.5	15843.97	13409.59

LOC40012(	253.519	97.52814	72.82478	109.1029	85.55796	109.7382	60.02633	47.11715	267.0074
CHCHD5	48.47253	79.69476	249.2251	73.57606	1114.819	55.50379	391.2784	509.6472	52.28958
PRKAG2	1586.021	52.14414	734.4609	826.7943	654.4203	847.8738	875.2177	1789.927	37.37872
FBXO47	63.14354	58.78038	45.47225	41.90859	33.47228	48.50471	39.76818	32.38676	38.73306
LOC648148	54.77959	89.94414	56.21697	98.37859	70.4492	106.0752	50.47851	57.24963	120.3262
ZNF189	58.67678	56.00869	48.14671	85.43008	43.02802	46.46399	51.3002	46.23869	44.7317
PLEKHA6	587.2743	46.53484	220.243	59.74536	549.3278	985.0544	279.9615	582.7698	39.05751
SNAP47	581.1196	347.0098	275.2801	342.2546	280.9586	260.4708	235.6875	496.7206	797.2082
PLEKHA3	1283.077	38.11845	41.28307	48.18675	169.399	46.13432	100.8907	229.8629	37.8618
C2orf73	133.3884	50.51154	57.78627	47.62436	48.0651	77.49148	44.86713	38.50927	38.16829
CASP10	582.0587	266.2589	141.8563	285.484	250.3162	426.7072	237.7999	188.8416	734.2235
MYO1B	554.7958	427.7535	405.3788	1144.228	246.953	495.7666	367.6412	485.4565	636.9332
CAP1	3239.122	90.65789	3666.395	1017.468	9504.202	2401.408	8769.543	3980.529	203.9977
OR51A2	74.97337	36.79655	33.4366	47.58504	38.45698	38.46854	36.99508	34.4392	44.93953
NXNL2	57.60006	65.43916	60.89319	62.79374	65.80935	57.14339	56.942	62.88043	63.92318
INSM1	53.49197	72.89507	61.57206	56.39413	52.12592	72.02442	67.90108	65.04669	84.41347
BRI3BP	4975.251	4218.586	2844.179	5808.013	2137.583	3074.844	2913.325	3265.424	5818.062
RBM12	2022.04	4063.375	2622.423	4443.392	1922.033	4309.775	2449.916	3304.233	1068.398
HMGCS1	6969.078	8886.134	10829.8	12188.79	12234.76	8468.285	8458.166	10251.18	11178.92
C1orf21	1331.7	47.92857	276.8517	422.7711	1063.478	725.3364	609.9185	796.8901	39.22256
RCCD1	45.03305	715.4548	186.2248	36.19049	183.1615	56.96489	119.7387	34.88237	95.07076
PRSS23	3557.74	3954.525	1806.112	4596.75	1998.549	3630.033	4658.02	1849.045	3883.51
MAPK12	95.70679	51.39567	41.57751	122.3658	91.34575	53.71949	65.93877	64.69904	108.7432
BLVRB	9087.768	6830.316	5438.409	5519.646	3186.722	3956.869	2853.965	4890.833	8696.763
AMY1A	45.2328	38.8188	34.20099	39.72197	34.47138	59.02568	41.43557	36.7029	43.49655
OR1N2	130.5746	42.81211	54.32495	94.77254	56.39821	45.75555	44.1705	42.07039	124.0788
BACE1	323.413	140.4542	90.50082	197.2112	162.1591	184.5823	90.5321	133.9394	119.552
PRH2	254.4058	126.3403	88.47557	163.5999	56.78864	104.1432	65.18806	97.96407	393.8026
SUZ12	16204.11	16015.77	15158.73	17555.43	15247.71	14721.13	15321.79	10633.7	14965.93
SEL1L3	2857.117	5154.042	4290.475	2743.984	2570.149	3267.772	4771.619	1652.478	5301.345
DDRGK1	4429.931	3861.346	5769.92	4375.042	3723.641	3522.107	4720.653	5595.643	4082.757
ANKK1	63.24406	377.9114	38.23956	70.15794	41.75883	49.66103	93.02506	82.76195	42.41951
ALOX5AP	939.4067	50.55736	2132.823	54.89555	1012.741	604.7867	1498.784	2209.992	35.30712
OR7G3	152.7179	44.90531	69.86529	108.8692	68.34485	68.51586	54.3748	50.34929	120.6655
SCN11A	905.0986	395.2504	355.1696	31.8876	120.9673	285.7476	127.2658	123.8422	841.1917
PFAAP5	2897.967	1291.809	727.9836	663.252	1218.552	1328.123	1037.579	989.3188	79.8133
FFAR2	142.8695	51.21237	73.97636	64.76998	40.87858	73.2927	171.675	47.17082	278.271
ZNF544	6576.141	5808.835	8005.854	6234.535	6458.435	6420.783	5961.653	8907.519	5923.614
ADH4	36.34979	35.42453	37.11066	64.73827	34.74623	57.12641	39.00315	34.63822	74.7972
ROBO2	56.56631	42.79235	40.70451	41.70277	114.2518	35.07097	47.57441	39.14666	39.40801
MICA	52.46783	495.3033	47.97358	1163.213	51.02017	56.66567	48.69257	218.1632	38.498
MRPL17	4781.066	3737.893	3487.9	4407.961	2208.486	4476.423	2278.445	3448.299	4654.956
EXTL2	54.00295	49.22319	287.2243	76.28963	294.9148	50.46264	142.6598	182.9309	57.37471
ARL6IP6	426.8666	367.0774	215.4485	336.8224	261.0244	111.3177	179.6318	198.5807	31.91992
PDP2	83.36868	487.105	353.3443	81.17231	435.152	324.4887	335.912	256.7712	216.627
C2orf65	54.07312	46.30563	43.41508	47.11433	39.79195	49.36797	31.74438	41.73051	73.68564
AIG1	69.98294	739.2661	510.8002	841.0959	649.8126	381.2122	919.5065	644.8486	53.1505

CXCR3	135.9043	107.0445	496.1387	147.4382	118.267	372.7776	218.4046	234.943	115.1097
KLRD1	49.46098	36.28381	41.01354	39.22131	39.49662	36.66605	43.93515	103.0768	44.87677
PSG1	45.91521	55.40972	135.4489	45.85598	39.94814	35.77375	333.3413	138.7978	45.71066
ANKRD35	18411.52	14027.08	15709.93	8886.456	18736.35	18133.66	13005.29	10810.23	18508.81
EIF2S2	73.02363	45.73015	45.83263	71.29919	43.71578	72.02111	36.9707	46.25243	123.7104
XPR1	518.8672	75.24255	186.125	549.7745	148.8456	145.0116	259.0557	230.9792	49.10518
LOC401498	41.57406	46.57234	51.20165	34.25152	44.28486	51.32817	37.76521	37.09336	61.80526
ADAM32	36.27719	70.53632	60.02934	40.77337	42.93706	52.78956	44.58901	40.00789	76.92515
Septin 14	36.78378	32.33979	35.12101	37.09003	33.74891	34.67499	32.01476	33.35752	44.04037
ZFYVE26	161.8502	136.8683	469.4535	185.5928	579.1312	1039.133	539.8353	1025.99	318.7623
ARNTL2	476.5644	354.2983	267.7346	469.7661	349.9203	330.7589	284.3698	354.108	662.8932
HOXD3	240.627	281.2393	105.8176	297.4223	134.208	103.597	100.5373	88.6553	294.4116
PTPRF	359.1928	194.7374	327.0317	1156.837	263.9111	536.4589	354.336	273.7566	362.4498
CHERP	1901.328	557.4849	330.9323	737.5167	223.5708	609.572	320.0903	423.3495	1913.777
EID2B	50.36848	42.26469	477.9307	485.1106	207.0068	258.6667	415.9098	184.7024	101.4304
GRIN2A	197.4215	148.89	84.79033	173.2223	74.98164	122.3619	75.8981	97.83678	346.7607
C6orf211	328.412	187.3696	125.3342	264.2971	100.8243	166.2754	100.2378	157.6104	362.2763
BPY2	40.52427	36.27762	38.61172	50.83571	35.68251	48.01166	40.03865	65.72903	221.3267
ELOVL1	147.1157	543.455	207.4277	1268.605	809.4261	113.7504	662.0977	442.6888	1412.032
UPF3B	4151.002	3910.111	4804.162	3984.048	4148.865	4790.685	4267.133	4484.413	2916.37
PHF3	3884.287	4158.625	3421.004	3881.092	3249.736	4588.111	2904.296	4194.32	1822.75
IPO4	41.86607	72.83215	267.7998	48.55821	285.2353	53.42901	233.4563	232.3304	45.77759
CHURC1	1653.644	792.9226	1154.111	1500.533	891.787	1544.418	1960.778	899.1143	933.7281
MYST2	249.5579	178.8618	117.8249	174.293	119.9541	154.9469	107.745	118.8279	227.1272
COX7C	70.85915	1958.579	769.8362	1086.627	2111.118	683.9948	1038.777	1268.895	49.41936
KRTAP11-1	76.27061	132.6603	63.90972	79.60435	50.26678	63.72101	58.19374	85.33981	42.17051
E2F7	68.58904	71.23682	176.5524	97.0533	69.92892	59.60083	86.04622	71.12423	45.18296
LLGL1	63.39901	106.9866	636.9802	126.2242	204.7536	42.21616	736.8706	232.0525	69.30121
JARID1C	55.27465	87.58206	203.527	63.5817	158.0222	451.9696	196.3408	221.141	103.0367
PSMB9	445.2601	178.5441	171.1334	228.852	117.0631	150.8211	186.9202	208.2424	47.35884
ACTR10	442.7494	256.4116	122.8894	281.5625	124.5293	198.3387	99.59211	117.5391	127.8282
C11orf75	57.26657	38.66982	1029.098	315.0166	917.8536	1456.64	1236.084	864.8391	77.51753
AHSA2	1378.104	710.5377	1305.814	367.1805	1280.013	1481.365	1819.611	1608.803	216.0181
MBTPS2	37.21752	33.41766	135.7829	36.23956	39.40742	45.83484	68.45982	48.67459	39.32857
PAQR9	50.33014	74.5444	60.43301	53.21856	51.62953	36.56886	42.69341	65.14745	49.18876
KIAA1468	69.77248	111.3327	311.1526	112.9299	501.2516	267.7957	238.4931	276.4742	837.7108
PAPOLA	4892.053	6525.989	7172.175	5974.557	9193.809	9894.24	9807.552	7552.895	4159.086
CXCR7	3201.83	3477.659	3861.208	2800.436	3777.993	5794.251	8207.198	4838.785	38.04681
NTM	45.88773	48.15445	193.6151	57.09858	358.1456	35.77589	354.9091	315.3474	44.84284
PIGH	56.69148	319.4762	128.7494	371.625	163.3141	43.52947	41.84584	45.74279	34.71107
PPAN	766.04	40.65187	211.6196	2156.381	483.9008	1247.17	431.9522	699.8346	1575.36
FRYL	1764.936	4062.91	4898.909	4824.883	2591.301	3208.719	3440.119	4601.509	4132.751
CSF2RB	2321.542	35.6581	619.9186	43.89425	464.611	401.9413	98.46259	197.719	3925.768
ATP6V1E1	1810.635	3351.728	7302.688	2590.439	12246.97	6557.933	11986.77	11248.7	2588.704
GPR144	45.81358	36.2887	36.27428	33.55592	36.06468	35.70472	31.84772	38.69344	60.72506
HOXB8	48.56252	58.83887	54.07333	49.71569	48.02912	44.24141	57.3363	49.23105	68.12836
ZNF22	6130.155	5827.654	4441	5792.14	2228.176	4865.319	3770.67	4489.404	5379.565

OR3A1	256.5293	178.1242	82.24242	176.451	81.90309	110.3279	79.38322	81.45019	353.6524
SMR3A	51.51738	61.07494	37.70683	43.23133	34.16445	48.26256	35.91751	35.21108	33.35093
STK17B	111.3445	2778.731	100.2773	1470.183	422.0862	550.1959	586.5601	846.647	122.1553
HDAC6	1001.68	485.0901	377.9578	227.953	458.6972	325.8818	596.335	434.9606	424.8356
FAM71D	51.33481	51.12925	36.49326	67.76286	52.79963	38.52044	38.08135	43.59207	76.29971
SLC6A11	1667.189	50.325	409.1008	1634.163	340.1935	839.5967	369.3644	150.8604	70.27029
PCF11	235.8719	123.5803	69.06966	132.3091	74.17326	89.29932	68.5685	98.43394	180.0772
SOHLH2	105.1042	71.52912	60.07381	126.9254	57.20185	78.41299	59.43649	135.2994	58.02778
KCTD17	369.191	236.7092	140.3498	285.7015	137.295	194.9099	119.2731	129.3758	564.1232
PQLC1	5935.883	5521.699	10729.55	8091.545	13736.51	10696.85	12484.86	13984.01	4053.944
MOCS1	50.35382	35.45005	40.59571	970.161	35.16578	39.98619	179.5971	61.17535	49.55925
RPL32	33.24355	35.7151	60.8981	47.76621	38.50761	50.90189	60.63789	55.35737	38.70902
MCM6	951.1343	855.0388	3633.603	965.3552	3549.498	3193.916	4561.946	2939.888	80.94511
TBL1XR1	47.97184	212.3204	154.4682	249.3064	113.5353	161.1956	111.0827	95.95918	36.48204
CCDC28A	1195.859	2074.809	3789.145	672.3058	3639.574	5087.11	4516.906	3415.08	1268.815
GABRA6	65.29442	51.58588	42.23859	61.3054	41.20076	52.39129	38.58867	39.47645	42.88655
ADAMTSL3	283.4471	101.2118	75.64692	171.2485	65.08237	131.7869	71.42081	75.95479	185.062
CHRNA1	2142.458	45.47128	276.7818	253.2414	59.77381	216.3812	510.5322	74.56503	63.68809
EBI2	96.7793	70.85166	496.0481	37.61311	527.2356	38.5022	326.257	628.9506	92.18745
C19orf2	6737.891	7335.688	11561.84	8899.661	12776.22	10573.49	12928.78	13020.83	5676.959
CLCNKB	52.57214	38.51625	37.60635	53.0689	43.69511	51.79966	40.8587	67.82768	115.8719
SRPK1	939.9213	1009.775	2995.893	1735.099	4008.423	3016.096	2790.835	1688.201	1012.811
ZAR1	47.30965	34.913	48.68345	39.15333	42.48685	42.29582	38.00512	55.97572	39.33035
FOXH1	240.1404	81.42796	80.83675	73.84144	112.9113	98.20616	79.37814	82.60732	296.5013
GOSR1	133.6557	791.9449	275.5175	106.9407	58.34586	63.42724	50.98632	64.37054	65.49008
SDR42E1	55.01772	58.44467	185.2058	587.2245	141.6513	53.34159	117.3574	40.97472	45.26748
DTNB	166.9606	1166.101	461.5254	2407.832	238.8564	115.0393	430.8775	556.0592	4192.668
SRBD1	115.5416	72.42182	417.4638	79.02888	358.7712	204.349	419.3214	213.9627	38.80253
PLCXD3	55.55219	44.75551	53.42796	49.01691	36.5653	42.56563	67.22688	64.8187	71.05967
RASSF9	48.808	47.76009	231.6356	51.56205	695.3361	266.1454	160.1872	196.7097	50.9736
CAMTA2	52.46611	40.5489	164.9604	64.20364	362.8161	44.06546	517.7176	485.9537	35.61031
RBPJL	92.4325	183.6284	101.8341	343.4387	102.7915	129.7767	82.21745	112.6693	295.3809
TMEM85	308.985	168.3633	564.6638	150.0805	570.6042	280.9757	667.5482	459.0262	139.8101
RPA4	58.97486	42.6688	34.91728	48.96262	165.3912	48.3974	131.9059	43.91829	1734.579
NUDT21	49.31466	76.13349	77.92104	81.80681	68.96316	112.0338	51.35881	66.35374	94.79315
C1orf65	36.96349	38.2759	35.35025	71.91506	43.53341	41.62564	36.68278	49.91421	66.90616
MYH1	763.5579	48.00666	306.7038	84.67675	155.323	356.9881	463.1932	997.5213	62.25055
SOCS2	55.14481	55.35279	49.78484	32.61703	51.84209	293.6181	117.3859	47.85977	45.04451
MUC7	106.0813	59.71861	56.73706	48.2535	49.03709	68.60046	41.69389	40.48547	66.49195
FLJ11795	63.86343	50.81566	38.63683	70.26096	48.15387	34.11457	49.2501	59.69393	51.15626
C12orf47	9873.26	10701.68	11741.32	10255.23	9421.789	12309.12	10511.32	10618.97	8929.066
FZD5	46.56398	94.49585	60.61205	149.6091	53.72062	55.34034	37.89669	56.34135	92.85707
ZNF560	104.0871	69.70834	53.65046	69.79876	52.74794	51.43282	46.36202	56.12831	121.235
GPR97	44.71476	76.19787	41.11896	35.19975	39.41243	54.70984	42.38005	36.29945	59.2101
CAPZB	14853.25	14011.58	12484.11	13640.19	9596.414	11971.3	8651.264	12913.32	17525.99
MYL3	7977.237	9647.83	7387.98	6476.063	5260.716	7344.711	15436.45	16396.8	13808.69
CEP250	63.44687	35.53045	139.6879	61.10184	364.9517	65.22038	242.6718	46.7621	50.99893

PCDH18	96.10771	66.6171	61.25111	76.36877	38.15153	48.13008	43.8029	49.4202	74.00089
TSPAN5	100.0558	140.646	125.4543	60.77222	149.6749	76.70762	78.99674	87.80035	70.3341
CMTM2	36.40401	41.25809	37.14502	41.19744	36.17812	39.70872	41.96142	35.07073	31.34529
NFKB2	3448.905	3899.514	2942.197	3811.843	1276.794	2261.351	1147.304	2396.904	3729.723
TRY1	216.534	158.103	81.55394	186.1987	72.03998	97.188	74.42181	98.05817	312.2935
GRIK4	95.53342	86.77505	65.13134	92.5957	41.10308	92.9822	48.63492	44.23764	49.57234
SIX3	120.4357	487.2305	71.15929	77.12153	152.8111	237.7098	323.5681	413.2215	78.42275
ZNF609	707.1913	529.1278	439.5641	685.4538	662.7482	889.2678	526.0712	508.3441	60.43923
XKR5	34.50165	37.94746	133.5065	35.51171	41.76164	56.37581	46.63945	38.98121	32.54294
TEKT3	43.88509	33.54978	36.66011	51.50706	32.12447	39.45106	36.89819	34.16923	44.48934
C16orf38	304.1982	169.8926	94.22311	156.583	95.01255	149.3489	70.51042	86.02034	403.2326
MOSC1	73.37408	33.94044	33.86121	42.91729	600.7053	33.56828	151.0771	331.0215	36.3784
CCDC41	35.93274	326.0078	296.0572	363.2612	384.6505	422.2949	441.77	162.634	132.0209
SLC30A9	340.4214	1478.449	3408.819	2493.489	3401.796	2979.066	3189.914	4731.344	1204.817
TNNI3K	79.82134	85.30283	62.37333	77.31773	51.91021	61.69673	51.2235	47.78085	135.8662
PPM1J	90.53224	74.01714	183.5216	47.73423	186.8744	65.12347	123.3441	75.9239	123.5736
PPIL4	117.0023	43.95498	208.3772	39.25263	144.3045	362.2158	163.0035	246.8431	47.23208
C10orf110	34.95675	34.86744	36.90744	40.66613	38.92387	55.99379	36.74847	35.3579	52.9691
CSGALNAC	38.34112	44.21196	29.37738	45.08097	35.23935	37.92406	31.37457	41.23799	35.19555
OR6C1	34.86201	36.83585	36.0303	34.57103	39.51364	42.73802	36.8846	37.92718	34.46838
IGSF2	73.72259	70.32068	212.6735	437.4382	159.1313	62.64875	169.9042	144.815	65.06741
OR2B11	61.4536	56.77584	48.20551	108.289	47.06458	81.39799	40.56611	40.63828	46.98732
MRPL55	224.2637	157.201	93.19722	174.8257	94.18421	102.3262	78.21633	102.1825	366.2978
PRPF38B	57.55479	62.76528	35.23293	779.9197	323.1674	533.0717	130.2742	203.6329	53.78439
C9orf37	8756.097	7662.398	3645.894	6357.897	2593.416	6826.699	3895.242	2842.272	8118.427
OR10X1	38.46858	33.4137	35.24068	38.95233	41.867	35.64956	39.77017	37.40091	47.08631
TTLL10	63.27466	40.66288	920.4026	1291.584	712.1554	470.5778	39.19495	44.46759	64.2778
LOC65375S	50.95333	350.8247	63.21138	62.44549	42.17303	49.17438	35.02888	48.73913	1290.543
PCDHA6	53.66037	45.28709	33.28587	46.97872	38.76783	38.78281	47.84821	45.43251	39.65133
TXNDC14	50.84909	505.2198	159.6515	55.39009	377.6004	35.23246	607.5168	639.1433	84.00886
FBXL6	11555.03	12663.75	10831.63	15068.12	7994.597	14281.97	11230.34	9390.236	12527.31
SAMD9L	128.4545	556.8743	2736.281	660.2061	783.8518	2116.285	1796.905	1595.167	163.0962
N4BP2L1	110.9103	57.69723	172.0808	50.63768	444.4782	529.4293	291.3015	59.22046	35.86446
ZNF383	870.1516	645.2028	360.7028	422.5236	439.9065	130.7022	526.4384	344.3367	45.41618
MS4A13	89.37881	84.12144	57.12472	56.29539	47.33128	52.07425	51.61179	50.96439	55.94304
DNAJC9	45.24883	3304.8	5206.582	2711.308	7339.836	7944.708	7155.74	5719.189	1899.884
ZNF441	35.9557	327.8044	38.65373	36.16248	52.76335	246.1656	107.5244	48.40567	49.83511
GJB1	243.3968	172.3973	83.4073	241.112	87.07458	140.3599	82.39637	112.0213	477.3717
CCDC51	75.93005	68.3477	68.59275	44.15662	49.57196	78.93673	196.3722	48.82474	42.72844
NOXA1	40.76443	983.5067	297.9893	36.80399	501.1706	1155.928	433.7862	717.9572	61.97642
STARD8	6399.777	5260.781	4016.784	4037.771	3205.86	3341.686	2989.337	5037.756	7107.601
OCIAD1	2460.778	700.8956	808.5433	2109.847	929.8694	1670.553	1245.74	765.9633	1541.922
PNPLA6	11288.12	9211.654	7022.948	8835.313	5868.211	8753.548	6348.566	6729.571	11073.07
KIF4A	49.12916	33.88318	35.69561	34.94323	176.6636	54.22678	31.86572	44.08425	89.66331
PSORS1C1	273.3549	122.9104	67.87064	195.2868	805.9403	1180.005	598.5394	531.2053	425.9618
CROCC	511.9176	312.6295	273.1124	442.8143	233.6001	406.9865	149.5812	154.9283	959.6454
CDC23	1192.781	1246.794	1912.235	1127.845	1618.997	2449.929	1791.907	1573.229	55.03289

GYPA	41.2449	78.68354	64.04503	64.59905	41.01042	80.17521	53.22659	42.42561	51.55784
CCDC96	337.9183	171.8467	102.5029	220.1592	107.6221	148.8721	94.3461	127.0161	220.4456
ZNF398	1725.068	1261.333	358.5966	279.2316	135.3718	214.3235	228.4705	558.3085	606.8916
DOCK2	413.2513	633.9103	1246.657	250.5325	728.3212	1229.846	1039.757	735.8843	505.3219
FOXB1	35.85045	34.21529	34.21306	34.58916	35.49231	40.12413	31.60583	35.09638	33.81713
MYBPC2	19836.95	23770.57	21535.2	20865.85	11685.9	18501.93	19554.13	24009.72	25193.42
GALC	49.49994	72.30687	165.0948	56.87571	414.3506	256.8444	584.6832	300.457	951.3571
DIS3	3693.773	1098.179	625.528	1054.2	492.6525	695.3715	549.6711	575.4752	69.66261
WDR57	45.22284	1352.501	627.6962	412.2672	252.6192	254.4899	455.7304	348.6119	59.15303
XYLT2	2854.952	2863.096	4167.922	4387.737	2878.122	4879.553	4708.105	3741.853	1669.373
MARS	157.5801	778.9398	331.5129	125.2203	550.6998	218.5012	321.823	445.3419	897.1453
HOXA2	240.3581	193.8116	351.7593	208.4709	400.6554	608.103	327.7341	1328.378	351.9918
ANKRD22	102.3974	559.7106	223.3258	78.65292	143.3187	233.8058	139.5348	133.2215	699.7753
PIGM	6802.899	6578.871	3950.43	5597.049	2941.839	4983.137	3659.09	3707.716	5741.634
FLJ30851	47.09235	55.16939	46.42658	41.79458	41.76597	38.08382	36.83889	41.01776	40.62634
ATP6V0D2	118.4686	400.6353	60.69868	110.6219	54.03263	764.6358	125.9247	399.396	94.06283
ZFYVE20	46.67332	1858.426	3133.048	2112.979	3631.101	2390.21	3438.994	3076.157	2387.814
GALNTL1	434.301	189.838	44.04331	71.08652	52.12485	81.10017	61.37416	59.48486	619.9632
TAF1L	1085.919	1070.247	781.7897	1026.525	255.1134	1209.968	712.2557	552.6117	860.6276
OR4F15	82.98821	62.11568	38.41721	60.4367	46.20588	70.15915	49.47842	41.66769	137.0552
SNAPIN	808.1404	653.0246	1028.65	921.4843	776.4039	977.2366	733.4231	896.8456	862.1258
LOC22011[illegible]	50.02808	43.23609	54.66167	67.25536	37.1617	41.12949	31.55119	44.78355	50.32005
DHH	224.3379	140.8316	56.26313	96.23753	65.10234	99.66122	59.7034	60.19195	94.60016
LRRCC1	56.24575	306.1882	40.0763	44.95603	38.70324	206.0414	135.7519	147.8476	71.32403
SRCRB4D	321.2029	167.9262	72.78235	170.8848	86.76799	94.21054	65.50823	110.0237	466.6884
ACAT1	261.4349	1452.999	719.1081	592.4327	551.2343	331.9768	414.9639	509.1905	135.9341
C19orf15	102.9513	122.8616	47.58238	47.63981	287.9676	51.74239	105.245	198.9004	213.2018
NKX2-5	43.24985	34.37903	37.47292	47.97233	39.24755	46.58045	39.74959	38.53208	43.97847
LMO2	34.23661	45.25101	125.2303	409.8867	118.9888	68.38039	97.21806	62.64106	71.26462
B3GNT3	259.8357	152.0512	88.37539	205.5655	78.27709	86.5813	70.66048	76.17976	652.8533
HHIPL2	130.2361	121.0423	123.0753	85.6804	127.7182	79.24771	97.31503	126.1447	104.4126
ETNK2	84.27512	32.58491	47.95579	54.33598	95.85251	45.05193	45.07573	42.43373	61.97546
KIAA1271	3337.533	2451.753	1572.836	3788.08	1133.465	2519.729	1107.249	1038.134	3952.126
NR0B1	208.2503	172.3969	72.18311	187.7115	83.85658	111.0627	76.54204	101.7367	354.0773
RILP	58.36329	42.95926	36.52126	34.36546	38.97572	56.63226	38.74606	45.18971	51.94661
MCMDC1	151.5508	107.3848	80.11028	919.3506	85.96121	129.4807	156.9695	174.4192	200.1663
OR6C2	33.28119	34.18937	33.27064	36.97522	37.85685	41.72901	34.91097	34.95013	44.23071
MRPL36	107.2539	1376.271	2961.651	2775.244	3602.039	2149.722	3688.172	3972.932	2015.504
KCTD3	606.2732	65.1422	58.8292	65.91632	67.52512	36.45453	53.25833	52.05101	59.911
FLJ35409	42.39212	668.8394	55.62766	41.03007	36.99414	411.3546	265.5071	51.83804	2878.399
TUBA1B	1558.259	1460.159	3076.038	3026.274	2667.835	2722.204	2373.808	2725.396	964.5182
PPFIA3	285.6543	164.6231	94.04599	173.9538	90.45679	147.0101	75.40391	82.49461	328.6887
GABRG2	35.2334	38.39488	117.5264	34.96804	47.3942	33.22994	36.31371	53.23286	48.00233
LRTM1	137.3977	565.1172	76.72404	92.10751	71.35275	126.8898	57.06991	552.7136	195.0018
GTF2H4	93.15622	93.67471	47.38396	44.97118	392.7994	54.55276	46.75733	153.8206	43.74968
HSF2	221.2412	116.9526	56.38467	136.2637	106.6221	80.55545	113.4251	51.65483	112.1452
WNT5A	2929.511	1219.762	902.5859	1480.223	400.2431	865.9764	456.453	585.1149	1785.172

PTCH1	99.17991	64.68223	46.44019	37.16399	167.1795	56.12445	35.8125	43.83038	159.1978
NGB	98.38741	673.4337	63.89852	130.9	59.66064	411.8759	327.7726	901.8932	316.5968
WDR66	34.81543	61.45396	143.3459	37.28497	365.6341	86.60708	468.6808	260.9762	47.43266
CST3	1697.628	75.09283	607.8218	836.0248	846.647	696.6979	845.3477	388.7225	1360.95
ZNF511	4152.833	5601.229	4812.037	6421.408	5056.841	4973.035	4706.953	4181.654	5162.651
KCNH4	2519.311	831.7009	323.8554	701.762	332.5376	680.8334	754.395	579.856	910.7175
RNF214	39.29793	45.16244	37.39474	52.11686	79.95808	55.20336	38.63745	35.22797	78.0839
SLC9A1	1163.794	2221.717	3498.667	4247.762	5379.545	3406.504	4801.098	5053.844	270.8664
CSNK2B	332.7903	1376.024	3591.928	1587.328	6188.081	3684.416	5518.529	4817.256	2442.014
KLKB1	65.37833	98.90018	130.4069	113.5558	169.8106	64.06207	93.85451	94.45057	210.5357
HMOX1	2072.408	162.1412	423.1705	40.26188	1208.006	620.4595	273.3477	473.0476	45.71368
CENTG2	2729.588	1744.84	1156.864	1779.142	1059.637	990.5482	978.7559	608.081	1381.955
CD55	1160.657	890.8221	378.4968	724.6625	336.3808	469.9684	309.1595	419.7544	2100.357
RPS21	38.58792	71.38587	38.703	77.69097	55.53855	43.47359	41.56562	405.4075	56.71078
BBS1	329.6443	179.7741	738.2883	223.7103	1774.877	847.4397	1629.141	806.6003	190.796
OR2C3	50.77247	88.99361	49.55072	65.53474	58.41211	50.28065	45.77392	48.28515	76.58183
GCA	232.6319	159.2234	251.0677	375.1718	349.9751	387.9453	390.4679	281.9924	1311.783
CEP72	46.79906	44.42581	38.66387	38.47895	71.86389	34.24316	38.19712	40.05223	61.4744
VBP1	43.95904	525.7169	47.19151	56.29736	287.8818	43.90522	287.53	223.6748	46.16312
SESN1	94.98103	60.53983	474.9135	106.1603	374.1459	295.4755	441.3056	58.42167	153.8554
NME4	10415.98	10443.12	8584.233	14241.31	8741.212	8787.766	10275.3	9102.797	10355.75
PGM1	357.5115	771.0733	352.7949	220.8061	1501.885	181.306	921.4388	471.9804	405.7308
LDB2	1060.282	1082.135	1656.786	200.8387	1899.164	1715.367	2305.052	2377.404	129.2733
MBD5	246.9701	172.3953	109.6433	142.7677	74.38484	99.60397	63.98021	65.46502	361.74
MANBA	43.79391	51.95282	333.6103	39.92314	676.6204	57.99576	358.2646	231.3968	43.85602
SPINT2	142.6861	81.04179	609.0096	81.14773	1580.133	90.3111	1451.534	962.0874	50.52003
ZNF692	231.2008	1736.013	3449.858	213.1794	3515.588	766.1443	3465.001	3514.01	218.1442
RFTN1	10172.71	11063.85	11318.89	11687.7	9742.466	9469.371	11488.77	11471.04	9019.082
LOC34026(	161.8181	43.38639	76.27813	91.12642	141.5911	125.2054	74.4554	104.2104	88.82978
CFDP1	873.4474	491.7218	452.8807	886.1403	271.7692	376.5416	304.0596	374.7062	821.0545
SPATA12	50.23513	38.31797	54.15055	48.26134	139.7094	42.11357	46.50062	41.15942	50.5509
SCAMP4	57.89634	45.13402	187.1859	382.4621	325.0252	220.7744	283.3698	295.4032	139.2746
PPTC7	52.51765	38.9334	622.404	578.4521	960.3775	300.7006	745.2288	432.9089	38.2025
SEZ6L2	85.28113	56.6707	55.92688	74.61739	49.37282	52.40786	45.09295	49.93719	108.1237
MRO	49.17542	49.57529	149.3347	34.96716	60.01337	41.21962	80.53593	38.43879	59.33769
FIG4	111.9328	90.64438	1243.695	77.10936	1478.747	1039.373	1272.276	1188.376	123.4907
NRD1	67.90234	89.04963	274.7007	122.287	436.3209	61.98771	407.2641	402.3498	94.23909
ARG2	260.4413	659.1631	342.7596	178.7907	1612.423	434.0649	1029.55	1153.112	388.5545
ZFP106	48.80431	254.2311	135.1505	272.8431	93.16512	142.5235	119.226	87.15334	553.814
ARV1	566.1271	1104.886	616.429	506.7708	546.6433	951.7903	479.1777	431.3546	1164.805
KCTD6	365.7648	421.2279	99.17065	181.7049	188.8693	277.3835	119.2742	153.6803	473.9973
ATXN1	3220.258	3314.704	1589.573	3095.786	987.0427	1277.711	1183.849	1946.872	2548.716
PSMD11	251.4198	123.4999	96.60883	136.5269	74.10762	104.2872	60.25389	85.56537	339.3397
ALS2CR4	85.79319	50.14021	165.1628	504.2651	831.4214	309.4783	642.2612	276.6077	60.23127
PSME3	64.60343	484.0287	684.2109	37.64086	2481.217	1616.546	2088.407	1791.573	33.60031
HTR6	113.7775	107.5016	56.3305	135.3247	72.44897	70.39929	59.55583	98.4906	280.8213
DOCK4	40.95332	36.95633	49.07431	270.6533	31.33474	71.41232	35.79517	45.87397	749.8735

PCDHB8	38.36077	35.07898	33.05471	38.08016	42.64711	33.97999	31.50994	37.86446	44.09343
C15orf2	97.2074	64.06999	45.15878	84.60912	53.92783	65.85161	56.34103	82.99214	85.92828
DACT1	46.30056	58.75727	35.13838	38.4333	44.19841	50.76011	37.34072	144.4481	108.97
PLIN2	175.0358	869.8796	585.2332	1130.29	162.6521	75.80217	166.3648	375.9544	79.10541
KRT222	404.2337	294.3446	98.68191	41.31349	88.92754	107.0009	58.45269	120.7528	86.09207
ZBTB48	21447.41	20238.99	12324.64	16422.63	12333.6	15462.48	12978.27	16747.58	23065.26
LOC642316	116.191	152.355	69.80663	162.7789	54.15002	75.55584	53.79709	71.32681	105.6999
C16orf33	58.31227	54.36843	864.3329	41.49001	1441.06	63.00811	1181.648	591.794	57.43542
SENP2	47.85699	40.88892	41.07973	41.31084	158.441	32.55975	94.75247	36.2934	56.466
KIAA1826	50.64561	70.08995	245.7069	53.53369	687.27	32.40059	165.3362	414.1953	50.23663
STOML1	1001.33	78.64821	1313.986	52.51858	435.8953	477.0821	693.53	417.9038	59.70052
KRTAP19-1	83.07192	55.46302	45.13428	99.20216	34.97021	45.4208	39.96151	48.07357	59.85308
LOXHD1	220.8119	174.3076	101.0293	143.3352	100.5275	154.1402	98.52909	111.4909	441.2895
MMP17	50.25161	44.88658	35.90162	66.60345	49.65939	40.00021	33.7168	37.05247	74.10452
RLF	166.8709	58.19057	67.93636	90.8168	119.0727	121.4613	75.79121	94.36768	136.9457
CLIP2	111.4734	80.12637	74.39769	70.12482	69.60664	72.14188	66.40977	76.6802	51.13271
GPNMB	34.49928	39.32032	31.52686	34.6995	103.7796	48.90063	103.1477	45.78966	37.94058
S100P	7553.432	8787.564	10325.73	14120.22	14930.39	18982.41	16520.12	11155.55	13687.95
WTIP	612.2773	569.1913	302.3669	803.7285	286.8905	447.7035	359.6807	275.1369	699.1312
DOCK6	173.3875	783.693	273.2151	1015.197	1593.88	1025.144	1215.364	1073.674	2666.639
SPG20	122.5429	1200.575	663.2094	554.8558	984.3921	1325.651	706.8696	712.5048	83.8933
OR5D14	72.7224	62.18062	50.38473	68.63111	56.6222	60.19543	40.92522	58.56002	104.4943
IFITM1	13937.96	13094.62	15897.48	16037.29	5063.884	18933.91	13598.14	10206.89	10422.63
CENPA	48.12421	85.16305	165.5336	346.4133	256.5623	44.556	181.0471	94.13158	36.59172
FAM78A	214.664	93.97377	156.8413	38.02452	121.9683	255.6706	186.7445	57.64208	90.11351
TNFSF10	815.3259	39.49129	157.9099	371.3416	89.67733	436.1709	191.2124	269.8589	42.59433
SIAH2	354.5941	380.4931	788.1891	1700.995	897.129	613.551	485.3928	994.9255	1029.098
DNHD2	47.3649	39.37108	44.38176	39.14158	34.19609	56.93874	38.32903	42.49767	78.16205
GOT2	1195.18	1092.654	3179.021	678.6653	4967.554	934.4428	3594.067	4291.084	2067.274
TAS2R14	57.41863	36.82164	53.63824	43.14301	51.83907	42.19484	39.28542	50.93701	48.01829
DNASE1L2	35.31164	36.61956	36.50057	35.72007	282.3889	34.14058	32.27856	35.24979	39.02379
COQ3	37.12295	123.8596	256.1901	72.64503	170.3012	190.7043	283.6154	232.4944	51.60481
SMG1	49.36498	560.2139	560.407	1101.858	403.2359	226.0717	267.6897	195.3848	1071.025
YEATS4	88.02746	62.74916	80.25863	41.14862	199.4021	74.06656	209.8549	181.7884	95.54953
DCUN1D4	4493.823	3527.119	4193.329	4886.333	3325.327	4797.463	3925.654	3957.025	3801.939
GABRA4	116.4451	61.45382	61.29306	47.26547	38.52787	51.74244	42.80064	40.90598	55.94764
EMD	4985.456	5649.15	7560.485	4769.851	7178.583	5987.067	6344.228	5771.159	3277.085
KRT77	43.25055	65.47652	40.42876	37.42344	37.21014	37.17733	40.20597	190.9767	39.80418
RFTN2	38.77006	41.08363	80.92862	50.95893	207.5063	38.71863	110.0804	107.8162	62.95674
KRT81	66.76746	99.48516	346.0043	108.3896	44.39209	49.84094	487.1711	56.02997	70.07105
TSKU	17817.71	15320.47	14182.25	18332.4	13026.61	15759.86	14752.09	12484.38	16069.95
GJB7	46.84598	48.5677	50.26587	55.38824	36.15348	59.89476	47.91818	51.77541	44.44575
CDS1	14029.47	13383.27	12751.56	14724.37	13453.81	14028.51	11010.61	12370.21	15042.53
SLC26A2	838.2203	590.0873	425.4616	564.025	351.6241	527.061	263.207	395.259	825.4745
GPC1	67.82083	351.471	136.3562	139.9196	294.8942	70.36369	247.6004	407.8912	50.44525
LEO1	1017.522	375.2143	647.6096	1179.21	304.3403	669.4417	380.7046	714.3752	2501.939
C19orf44	7917.669	8436.371	6094.21	6297.056	5825.106	8246.751	5895.894	6409.656	8526.363

CXCR4	5679.817	4939.238	8060.612	3991.805	2704.392	2751.829	2021.094	3228.317	7442.326
FN3K	174.4276	105.3323	82.85683	98.12981	76.12773	90.0754	63.04008	78.43187	407.8759
CCDC97	354.2924	690.1956	111.0802	296.4444	121.7757	163.3744	199.6601	429.7086	355.755
BIRC6	1363.789	2288.923	1999.155	1900.284	1674.693	2700.99	1914.19	2072.896	2031.548
AMZ2	58.09632	691.5024	806.559	35.84701	781.2344	1347.86	776.4808	1535.527	39.6786
EMP1	9530.899	4948.665	5690.967	5392.999	4939.421	3392.051	2772.066	2628.619	8219.842
KRT5	8285.138	8165.339	14160.73	11565.91	17270.73	13366.11	15211.76	16345.99	7915.124
C20orf52	4883.861	4475.494	5048.047	5836.168	4681.145	5841.478	5875.108	5858.516	4419.751
BMPR1B	48.68379	37.18304	41.18872	35.45041	46.88415	61.40892	48.43622	32.57598	39.04953
POLR3K	511.081	633.0105	657.2458	543.3026	565.8046	418.4789	472.7609	768.2206	1119.502
SCN10A	41.77256	36.18008	38.3898	46.68094	53.21134	39.28602	39.65771	60.90261	70.94978
C19orf46	2549.95	2471.211	2252.217	1306.286	2084.423	2160.542	1692.345	1790.579	1433.865
CPLX1	75.14229	1027.921	35.29035	32.05811	544.0261	43.48714	614.0474	367.4139	41.77255
APBB1IP	452.3805	205.0521	311.2161	311.9568	313.5105	409.0336	298.5504	567.9371	230.9331
C8orf86	159.0417	79.4639	72.10466	159.7192	65.87355	97.47759	59.62943	45.41014	124.4615
FCAMR	66.82964	48.39286	43.10555	44.99035	52.6645	66.06484	50.91941	57.27444	54.37923
OR2S2	60.67127	33.04603	39.04527	43.64726	36.77593	49.0429	33.90434	32.8154	34.82994
PFDN4	914.0637	321.0781	370.3032	515.9662	425.3095	593.5568	688.3503	455.0224	1776.974
CCDC109B	660.1602	660.8745	2951.709	1632.485	2524.308	3176.922	2719.438	3161.545	340.7618
ABHD3	47.28292	924.063	40.87351	52.09544	44.58106	38.06786	64.86958	40.57232	40.2942
NDST3	80.13401	48.13356	52.32678	93.11203	59.98975	444.177	48.39285	46.88069	115.9341
PSG9	40.49311	33.11793	41.90191	43.73752	37.56708	35.16738	49.25171	44.61958	37.77217
MPPED1	65.48434	57.11288	58.05642	56.80571	61.96048	69.25726	51.26057	54.91211	104.8356
PRAC	55.00064	54.8367	55.32693	47.42849	38.59838	42.32836	51.33519	39.44178	50.10629
PTX3	649.3837	54.51657	54.32818	180.931	38.2453	41.4877	91.40311	34.28016	68.28247
DNAL4	4189.19	5553.165	5939.93	4933.862	7830.764	7273.1	6190.447	5730.153	2653.635
ALPPL2	197.9811	81.4373	72.71954	111.0451	65.9414	61.48222	62.53426	75.33282	172.0136
SEC61B	1473.376	2598.952	3692.249	2847.074	3758.24	3801.278	4169.597	4547.346	2737.092
DPY19L2	68.49031	571.3763	132.8027	44.91842	145.977	203.6918	279.4885	44.72279	47.3382
WDR75	1051.636	827.9045	1611.615	1356.648	1195.519	2222.651	1532.271	1790.057	965.6885
SLC28A2	99.30972	59.85579	61.80494	45.28404	43.35946	50.31747	44.4628	41.14106	54.27683
MYO16	95.46773	120.1143	66.8445	130.3118	161.6896	65.5013	68.83485	80.84554	208.357
HDX	366.3347	205.5329	96.37331	204.107	107.6966	152.2478	73.48394	65.90083	541.8382
ATG4D	82.26797	57.22594	298.0123	67.92899	44.02059	49.76633	90.93441	63.28013	110.5465
IGHMBP2	141.9178	74.50928	58.7738	106.7668	110.6435	59.76437	218.1049	326.0618	73.4545
PPIH	518.4574	86.55408	519.4617	284.7448	581.499	253.96	578.1088	505.5632	675.7791
SH3BP2	36.83092	49.86107	91.21746	46.20741	36.26834	48.53672	43.62037	87.76992	45.95398
ATF1	652.3654	281.025	273.9671	614.7241	150.1316	256.9836	187.734	170.39	738.9911
FIGLA	44.84183	75.33715	49.45574	58.60275	50.02468	52.19056	52.33945	47.13898	94.10497
SERTAD3	61.65953	104.4641	96.61423	522.3282	267.0583	68.62573	333.6973	257.7237	115.0399
CCNB2	54.95229	62.49691	941.207	34.14544	1081.345	134.9115	1102.042	834.4666	69.50986
C1orf50	5307.783	4624.288	4839.379	5091.926	2526.899	5559.757	4059.826	6290.015	4730.955
NAV2	188.1957	141.0231	67.53792	127.067	69.97148	101.0168	79.87651	86.2976	206.0791
IHH	64.32563	63.42896	54.78978	65.01131	41.07132	81.8481	51.97208	52.89067	185.9107
OR3A3	82.2673	61.57454	49.33057	47.3896	47.98637	50.4121	38.38304	39.47616	47.63072
MGST2	158.846	655.1121	4855.007	709.237	6547.182	3538.352	5101.831	4306.142	124.5713
CTAGE1	40.86243	41.36721	30.44352	37.38308	38.06684	35.90192	41.67413	33.71325	135.489

ZNF91	478.7229	379.0889	192.614	562.7173	177.321	367.3758	188.469	302.9581	569.7007
PRDM8	2182.193	1121.915	1053.009	2139.868	351.198	1006.984	651.8219	673.8918	114.3514
SYMPK	46.05593	1417.109	1636.721	1651.441	1190.333	759.6119	1368.361	2260.999	2394.912
ZNF677	208.423	465.6069	63.08301	134.0736	218.1614	281.7146	109.1244	76.89885	277.5852
ZNF280A	47.25681	47.71888	40.93722	47.18233	54.13979	46.65713	86.15766	34.44352	38.42448
ABTB1	4051.774	3995.917	2212.305	4325.261	1520.277	2405.176	1526.342	2825.002	5601.064
SCGB1D4	37.5534	34.2519	41.72951	43.73605	32.36044	37.9982	37.26104	43.91974	69.87998
STYK1	34.23235	34.98578	41.59666	32.00943	92.89065	302.8097	54.57832	112.1428	38.8279
APOBEC3G	118.5172	61.22076	145.5531	65.4062	60.41768	64.69722	262.7533	294.2618	43.37358
SLC13A1	125.9605	67.35816	76.14111	76.84363	62.11966	88.28826	51.60247	70.36309	220.9276
ZNF74	130.1616	101.5585	154.4042	122.7412	65.18714	96.58681	57.07779	76.32746	103.2947
OSBPL5	35.86254	59.59544	354.9363	1086.414	638.4957	34.40287	779.6087	439.6373	32.26122
THOC1	2228.63	2617.071	2084.611	33.08818	1728.724	1413.745	1906.573	2222.651	3344.718
XIRP1	34.90991	32.49504	39.19015	39.06687	135.6093	42.44516	126.9277	174.4123	36.14124
ALDH1L1	35.35334	33.10845	94.27097	40.37895	34.98699	40.53589	30.39685	38.95932	33.07901
WT1	231.1784	112.4775	66.86819	91.22573	69.04082	81.06667	62.69709	82.71623	137.0263
FOXJ1	291.0389	90.97387	71.20534	135.505	155.9194	93.56007	59.2411	59.1577	254.3904
C10orf99	6018.409	5007.516	6263.66	1950.637	10590.99	2134.173	6130.73	5353.016	2008.771
RHOB	313.0014	160.6573	847.398	1755.476	2184.288	1612.621	2176.832	1238.265	1322.253
PTTG1IP	78.66913	62.35102	53.45507	504.3964	56.46133	72.16917	161.2407	120.1481	44.43949
GAPDH	6167.543	7753.313	11050.42	9097.882	11677.44	8423.193	11402	13924.3	7646.625
PCTP	47.1746	46.35137	142.0127	63.80706	50.31134	46.90284	101.9248	68.86351	64.80833
WDFY2	507.6855	457.9954	627.2212	318.2286	285.3976	234.687	257.4084	238.1339	686.0283
SMPD3	109.066	245.8472	116.6766	97.94365	134.6771	64.28362	57.96595	85.83138	590.0067
HHLA3	119.3544	55.67488	41.55203	36.7488	162.0708	411.127	42.24906	45.56346	42.51619
FTSJ1	65.30148	499.5397	125.873	308.7679	128.3201	76.17994	62.87554	91.09628	113.6068
RBM18	1706.702	1384.391	797.1783	2250.942	660.1724	1563.975	702.6007	876.2554	1387.749
KRTAP5-2	32.68464	32.16687	32.65251	34.91607	674.8862	44.96793	179.0676	270.2991	34.87747
IQCG	323.6849	612.2083	435.1388	234.9203	526.2032	560.3333	420.2359	739.5175	411.0388
RERE	49.55615	56.26019	613.6132	56.84171	958.3806	44.23311	1225.712	821.931	59.7274
GNA11	406.6437	48.595	183.8522	43.40578	412.915	36.58039	382.9797	270.4771	62.91122
ABCC1	77.22676	803.4701	66.87828	903.3862	361.7063	52.07726	388.9675	335.6421	120.0713
ITGA1	44.99503	33.51058	905.3045	39.81254	681.8281	352.7992	1116.705	561.5179	33.7996
CA13	164.5184	134.5637	105.1846	144.1341	114.7438	163.446	71.36476	72.95463	184.4814
REG1A	55.92291	69.74191	56.91258	83.06978	48.19681	60.1069	42.21736	50.13265	155.5117
DCTD	55.84194	89.29044	1652.048	73.40063	1685.123	1053.328	1458.313	1027.56	112.0054
AGTRAP	58.40098	98.93015	53.50666	73.25625	170.5004	47.74812	43.83194	65.19768	73.24487
VPS36	13190.89	13886.45	12824.78	12369.71	10777.43	13106.06	9386.436	10255.32	12459.21
HS3ST5	73.37257	96.09904	223.2984	127.1923	63.77581	73.35839	50.90084	58.45259	154.9388
ICAM5	121.2286	42.90387	202.9214	805.3665	50.41284	47.06769	33.05272	54.37236	37.76542
WDR16	62.57732	107.5733	61.94611	82.99235	59.31144	63.25985	59.75776	64.1551	123.1844
MRPS24	325.4654	212.9633	216.1045	205.3911	664.0427	149.3297	629.4778	426.1471	449.9424
HEBP1	9365.072	7850.943	7894.318	9415.045	8114.487	6340.816	8079.84	5889.45	9826.577
SPEN	217.7721	179.3197	3424.218	241.9689	4452.008	2940.956	4577.723	2093.366	70.13617
CDK6	57.47682	291.3058	457.8191	306.5499	199.2832	44.51688	225.9553	144.093	46.11049
PSMG3	147.8838	252.8773	299.6988	342.5754	283.7865	163.9305	433.0607	467.9993	260.2691
PPAPDC2	50.33678	93.34628	42.7956	50.30825	166.1142	57.02441	94.7234	45.62811	985.8026

AGA	8462.357	7408.952	5035.432	4573.71	7161.188	7906.15	5188.203	3272.019	7556.559
GABRA1	39.21271	35.96274	37.29203	35.42156	51.43081	48.89209	46.79886	42.58968	40.95793
TOM1L1	52.61096	36.20205	53.11816	44.25045	90.19565	37.99583	42.24786	34.38995	48.27224
EPHB6	61.48255	62.77964	1003.753	48.78524	2273.782	61.25359	465.5509	1979.113	50.968
C11orf58	507.6497	129.9236	230.9102	321.7482	180.3718	195.9945	147.8397	185.7512	656.6545
EBI3	90.40279	58.63503	45.99869	37.06086	274.6815	57.48395	137.0942	344.5692	91.38258
GLT8D2	67.51817	80.0182	422.5511	73.39298	252.3904	606.789	562.9532	226.9373	56.6572
PPP3CB	2526.706	3400.762	5974.549	2865.959	5970.974	5749.641	7509.123	6624.981	46.44378
PACRG	49.33796	36.7275	51.22955	281.6924	166.3425	43.94164	32.49372	39.47483	45.34402
GULP1	37.09255	62.38492	61.55287	45.57822	41.01838	43.51526	43.27137	45.97271	45.506
WDR52	73.35443	50.57576	124.5446	44.27851	136.5707	51.31923	159.1797	55.35098	42.7149
LOC650137	43.08637	46.80154	51.90385	45.70106	37.58774	46.99897	35.95991	45.71093	63.11156
TAS2R50	602.2862	365.9214	107.8939	231.6358	35.34081	179.9888	53.67256	102.6706	797.1949
FFAR3	178.9657	108.5528	58.76137	113.111	67.42632	87.41187	56.0279	70.5174	301.8195
MLL2	1198.502	2802.055	1077.054	2637.974	520.4403	1100.958	496.9707	875.6755	1637.428
TSSC4	6485.91	5980.473	3105.337	5594.996	2577.563	4137.179	2614.285	3925.409	7557.117
C22orf15	49.62757	662.0758	783.1675	469.0754	201.0687	74.38016	150.3299	546.4935	36.14173
M160	34.34536	44.45933	918.382	968.85	1427.986	1048.912	998.9471	1780.5	44.23955
DEPDC1	37.35221	48.54874	37.3175	34.28399	67.8137	48.80671	61.66611	51.02594	35.4095
TCP10	38.89618	38.48197	39.5357	35.10751	39.56911	43.36053	34.29255	41.57767	33.66784
MTMR12	39.16658	89.78572	67.53964	75.0795	35.79202	70.13797	48.97795	42.55409	40.88563
PRCC	91.10842	60.51326	229.1921	248.9104	324.3898	149.4315	458.6793	291.5716	699.0301
ZNF185	119.1078	71.80871	1111.429	332.0046	1224.119	73.99636	614.4748	476.9143	71.90121
VKORC1L1	1793.264	1841.604	1993.235	3317.471	3191.44	3108.841	3132.949	2160.21	2543.458
LOC728116	49.53025	80.10815	44.92432	65.26405	49.47874	37.88717	51.86543	50.9202	125.0689
TRPV3	68.85646	1256.719	59.02518	147.6031	238.4496	340.1666	65.17363	175.1	1465.351
PIAS4	18684.5	21446.96	20715.39	18768.8	19278.02	18813.18	22574.59	21405.59	19440.52
APOC2	337.5953	126.9417	81.99791	112.8756	79.34715	596.0313	252.1612	563.477	329.6228
CDCA1	63.40556	57.03679	160.2046	51.18085	52.31167	60.38334	44.50252	63.1488	53.54388
FAM160B2	178.1811	470.9163	297.2471	154.626	676.4967	318.7497	427.7108	390.7851	334.248
ANAPC4	100.089	2324.154	4222.2	1745.53	4935.613	4915.079	7035.326	4385.597	213.0105
ZNF572	39.67711	35.90864	33.51387	44.54705	36.00552	46.54834	33.96181	36.12688	67.72775
MMACHC	55.65084	47.01883	54.20774	436.8153	38.39631	42.45584	44.64658	40.53547	56.31281
C11orf44	66.38332	58.07133	57.5751	35.96157	47.15379	43.93302	38.24755	34.83481	65.84419
KIFC2	2613.634	1780.749	1502.233	2433.439	2378.999	1889.262	1637.747	2509.707	2660.457
NOS1	69.01836	48.66615	374.6208	36.96019	549.2881	340.2619	85.92567	391.2086	43.44791
TARBP1	41.47478	40.21062	39.07225	35.1062	1008.07	624.8959	1048.254	39.10339	44.00521
TUBGCP5	196.1308	138.5175	735.1231	174.4647	840.5255	1278.01	763.7342	997.8684	146.6754
PDGFD	95.31941	93.46852	65.17843	49.80016	51.41315	62.41544	54.43569	87.97002	279.8508
SF3B3	1418.737	2281.559	1452.813	2217.405	1596.886	1635.597	1287.303	2311.962	2689.942
BIRC5	64.33161	46.74054	66.16374	79.05017	45.63982	76.7375	178.3083	254.6595	86.11643
RAB4B	6628.297	4111.257	1459.517	3143.104	456.7698	2197.938	1119.462	1138.318	6512.394
SYVN1	109.8043	100.9163	250.2182	65.87199	298.9868	71.31179	260.9615	140.2843	56.27398
PRDX6	1729.166	2446.408	6687.726	2024.006	8045.603	5608.51	7329.775	8004.442	61.7103
WDR76	46.52968	603.6405	312.4724	38.79158	235.0103	43.69434	108.4195	182.9234	40.50132
GALK1	15718.54	13752.14	11942.53	15073.89	10893.7	11211.92	11138.15	14151.73	17113.67
RAB3IP	76.71203	332.339	304.2315	733.6249	623.0986	297.9757	780.4165	495.2682	61.70366

FBXO2	295.6783	603.555	421.2121	470.479	694.2974	189.1451	1392.337	583.2166	112.8758
MXRA5	5706.262	4264.721	7260.364	4960.763	7766.456	9433.519	9180.438	6801.691	2649.255
KIAA0514	67.78411	51.08697	189.7172	641.5024	177.4949	42.18602	230.4087	441.8825	43.30745
GPR139	199.8795	69.89268	68.90511	97.55747	58.21435	69.61559	61.99232	79.44958	185.3799
TMC4	576.5025	1061.238	722.5026	381.5496	1356.921	1974.998	993.4104	829.0428	794.7292
PODNL1	97.37519	90.91106	58.3321	48.19911	43.76746	77.30643	58.14463	45.95609	333.8778
CLCA2	4745.438	6586.144	9711.841	8398.673	12439.12	10298.83	13460.34	10140.54	5386.455
KIAA2010	2956.483	2129.05	2613.566	2562.838	1017.322	1059.03	1259.554	1996.084	2214.765
STOX2	13924.78	15941.67	15816.52	12362.1	18239.25	13772.66	12723.39	14975.77	16368.24
MVP	99.07272	98.87846	2089.354	77.03205	2357.083	468.8494	2651.22	2847.357	188.0516
TMED5	42.11517	846.1218	280.4858	419.2037	385.0905	228.9613	191.4298	126.9127	55.89756
OR4C45	225.9527	71.5616	71.10932	70.81064	63.57754	111.7947	50.70729	51.95039	105.6977
PCDHGB8P	40.26663	34.04079	33.27851	32.16654	38.52836	33.09566	34.86359	35.22517	32.87587
C14orf68	73.07055	61.96216	64.56852	80.02563	49.6185	69.42281	50.55298	50.76139	47.47167
DIMT1L	14774.21	12416.97	10025.67	13356.62	9604.375	12701.58	9348.262	7517.208	11835.16
C9orf114	6818.408	8127.791	11866.67	6051.437	9395.849	9927.036	11163.53	13543.89	8548.353
PPFIA2	2326.17	694.2542	47.05802	56.0141	46.13017	390.8385	39.71112	236.4707	77.42175
EHBP1	184.666	148.5108	314.7312	98.8629	508.3135	102.0919	421.6671	101.1822	306.035
FLJ40125	156.9976	129.1237	89.73224	132.574	79.23482	94.5545	73.18585	69.39387	281.2376
EVI1	93.40515	293.0204	299.3767	65.95587	590.1903	633.9216	756.2305	383.979	43.1152
AEBP2	1784.128	1260.652	478.5718	1330.099	332.0332	781.4403	390.9752	517.6277	1692.435
ALX4	35.64027	34.95265	47.68381	40.04148	32.2695	50.85282	45.8879	53.13583	40.66405
TAX1BP3	54.17268	60.05408	117.0076	63.05579	370.7327	490.6428	714.7469	157.028	73.79816
SRF	323.0909	1803.899	4755.565	4120.014	8027.181	4638.212	7708.302	7956.631	364.5266
ARHGEF6	3494.145	3462.206	3930.025	1618.616	2369.185	3758.808	2747.765	2417.019	3113.953
CRCT1	7099.769	7130.034	9663.265	4311.066	13032.59	5870.934	9662.245	9227.316	4886.248
PCGF3	72.30706	73.61944	65.05547	75.11471	54.3501	57.7879	52.41982	59.52461	104.1729
PHTF1	1284.043	115.5317	1161.534	147.0851	509.8513	648.2877	619.0076	223.7012	85.50517
KCTD20	40.61347	79.0213	143.7337	47.03544	79.54102	346.7393	78.58268	175.0631	87.8199
KIAA2013	358.8066	154.0344	1192.911	384.0332	1467.258	818.5132	1568.645	1841.375	1382.125
LRRC31	45.9146	35.02191	39.77265	54.63731	35.64372	247.6739	40.25314	48.08796	43.48021
LAT2	57.06019	1096.062	2635.576	63.63969	2084.146	1958.609	2318.213	2244.761	161.9469
PLA2G4A	62.79569	507.4655	353.6392	695.8777	1226.452	44.0542	564.502	239.3974	36.21976
YIPF1	3676.939	3359.531	2099.878	3064.755	1483.589	2508.22	1780.768	1791.039	4280.809
ZFC3H1	154.2868	279.2816	172.2049	246.7274	71.58041	158.9091	168.4847	101.5611	73.41219
WNT3	236.1055	362.8379	568.1211	171.5964	327.8691	725.3857	575.9867	399.1587	148.7856
XKR3	36.68661	34.6114	39.13522	33.74838	39.60723	32.99549	46.72024	30.03717	33.24227
OR13G1	46.93908	77.75894	65.4481	48.90591	40.51278	35.17887	38.09121	53.75135	40.1616
TMEM147	108.8933	54.69428	940.0533	678.4807	1621.612	44.99512	959.1885	755.6088	64.06967
EPHA7	41.34599	44.52632	47.19006	48.69199	39.01257	42.36979	32.69603	44.44627	33.1262
FAM162A	664.7014	382.8988	1712.248	1642.262	2555.882	1529.64	1468.448	1623.267	79.14461
C9orf91	37.97773	1847.826	2042.827	1084.928	1548.738	2918.76	2253.486	2955.585	36.62418
ENOSF1	60.03691	993.3212	1715.299	832.5043	2787.894	1996.271	2036.048	1899.607	1621.144
ADA	113.5065	86.56398	664.9083	40.80501	1197.886	68.29629	921.0887	1366.025	140.2159
MMP20	39.71944	41.21263	54.64421	60.62587	37.59015	47.92144	107.1603	42.59997	37.07682
SLC35E4	45.18885	37.02408	44.63991	39.38546	34.22914	40.52205	34.82602	54.58229	57.13263
RPGRIP1	34.37066	45.93056	41.59505	41.2693	117.828	41.72249	50.20638	37.91601	86.56398

SLC5A7	36.94786	36.40295	33.65264	39.35214	41.73655	58.47939	32.95578	36.79185	33.94643
POLD4	2243.096	44.19773	46.93161	47.5807	50.3591	606.2807	135.1818	60.50464	94.99051
MAG	40.1728	1537.108	282.4667	42.77592	216.9784	45.95525	603.1143	337.6305	1635.728
AP2A2	62.48964	53.37064	45.20915	59.62939	301.0991	53.77475	39.6759	49.35825	71.16277
LGALS3	9192.299	7068.381	5427.889	7800.773	2954.806	5659.828	4230.951	4079.066	7306.464
TEX12	32.08477	34.38205	35.30601	33.5223	42.48153	39.90562	37.81109	35.93469	47.69458
RRBP1	11279.58	13382.36	11170.88	16550.08	12286.74	11875.84	12996.55	8477.715	9753.902
TRIB1	33.23287	4546.977	8694.225	5804.579	12371.27	9254.695	12235.59	9669.672	2630.382
ASTE1	74.54438	281.5642	125.8678	75.05046	168.0253	53.01118	247.718	122.4499	64.96741
IQGAP1	128.7609	426.8596	907.6401	119.164	1437.758	960.9199	669.3936	1559.759	1501.708
CNFN	11101.67	7421.207	7083.316	5027.144	12767.72	7270.598	6813.766	11807.57	9829.495
EIF1	1484.105	793.7278	1630.231	1216.118	3227.044	1686.141	2781.524	1339.654	1241.335
TSPAN15	261.7452	120.524	82.53108	138.2926	64.46129	66.70703	63.71945	85.71831	68.72294
NOL7	4929.818	3057.097	8019.586	4781.732	10042.97	7597.27	10329	8912.226	1649.72
FAIM2	1603.362	701.2519	1262.227	39.0917	1301.069	906.1924	517.0229	272.7125	65.05121
DKFZP586I	76.67702	114.1917	221.9887	68.84959	170.8161	57.43011	114.2204	73.34304	47.46394
LCN15	37.09032	46.51814	54.18895	36.61153	38.45574	97.44064	33.57113	34.53979	35.08016
RHO	36.92831	39.60975	41.14057	34.91288	40.813	42.51014	39.65058	34.75696	35.02652
OR5M8	71.77822	97.42667	54.07122	72.73415	51.21558	51.47036	59.98482	80.22945	130.5672
GDF7	67.75703	75.97818	68.20045	109.6787	51.09824	55.28755	53.91713	78.22472	93.95918
ETV2	464.3859	231.2859	161.8475	229.8751	143.7956	184.8243	115.1619	156.3342	533.72
HOXC5	38.90451	63.02816	38.66768	84.12113	45.37125	40.9888	41.6982	51.47828	86.73338
IIP45	40.54385	35.05159	44.48402	33.69607	33.29957	42.60456	36.54612	38.42058	32.24098
YIPF6	1067.021	1182.178	1198.117	32.6256	2211.616	886.3912	1921.071	1070.043	41.10985
TBC1D15	42.70165	773.6006	3236.912	65.20568	2912.722	1825.891	3668.744	3090.121	46.30886
HCP5	16232.15	16883.73	17809.82	18853.16	10300.53	17419.46	17818.83	15022	12272.89
BOC	5956.803	3464.422	1144.949	2648.902	1329.043	1094.168	725.3773	446.6735	3305.897
GALNT1	102.1721	60.95444	120.9781	92.24937	304.3617	67.71711	258.2482	153.1765	101.2096
LOC730243	118.7216	160.0397	75.99284	54.08169	69.7477	98.45086	47.66267	64.89703	1194.495
MAK	40.26873	40.92809	39.94009	57.4258	37.63464	43.43249	30.143	52.01221	51.1958
TM7SF3	2911.594	1002.193	1885.319	1857.114	950.1952	635.8115	536.6108	403.9676	2384.55
SDCCAG3	270.1926	322.2997	645.1072	369.7599	1177.22	1034.249	1201.862	743.2785	384.9277
FBP2	55.20691	41.08221	34.1693	40.52457	49.23455	34.99598	116.7773	30.9299	2335.896
SNX8	268.7657	216.6489	104.6355	199.2238	255.3558	539.5903	562.2883	649.1747	433.3521
SLC25A10	844.9196	685.4966	745.3479	641.8618	520.8768	568.4308	497.9743	674.2402	940.1927
FAM89B	183.1881	70.72118	2001.779	64.96455	3757.832	1251.3	4372.345	2888.128	101.2253
TMED4	339.8046	172.512	745.9107	889.1456	962.0205	677.0381	740.3925	462.3323	446.7935
HIBADH	636.2854	503.9003	903.0632	443.353	1443.434	1074.156	1168.997	1038.44	1099.388
LOC653686	42.33619	40.30974	62.91972	41.7132	36.77122	39.57284	36.29044	33.99586	56.44947
LOC284912	110.8951	118.1791	77.29835	163.6474	62.75044	94.8625	65.50594	54.69281	200.9986
ADRB1	102.8866	122.2384	68.62257	150.3076	56.03028	70.33469	61.47206	64.75327	176.994
ZNF76	63.68694	70.39483	277.676	92.49838	41.25677	57.87922	424.5787	285.9941	100.1627
ZNF835	45.52346	48.00705	51.23347	55.65696	41.46786	53.75713	42.41597	34.72773	55.22334
SPRED1	14838.91	11955.3	10870.31	14840.89	8748.491	13207.94	10746.84	7144.274	11508.87
TCAP	3516.292	4241.51	1773.99	4299.464	1650.875	2659.627	4291.304	8261.341	6574.006
NAP1L4	58.85917	1058.621	642.8195	68.52914	1367.522	843.8732	942.2282	1847.972	92.94888
WWP1	12237.7	12877.56	10962.21	13141.96	9761.291	10788.31	9736.875	11414.46	13041.05

TUSC2	1195.031	62.88206	402.1742	75.54569	197.2822	317.4893	464.884	310.4707	2507.13
GPR137	22492.05	22105.36	20054.3	21709.78	20019.93	17679.77	18278	21783.67	23651.56
CAMK1G	254.3511	162.4924	77.28945	117.6765	56.86642	92.00566	71.80065	67.09951	155.0267
FZD7	35.4069	46.1426	48.06109	49.67719	255.6616	38.31343	107.0685	119.7324	34.26347
GNG4	89.09203	101.4891	61.13618	138.5242	53.70836	55.28096	53.44239	61.44815	77.60654
SUFU	75.69881	40.93073	69.43919	89.06464	66.21275	90.34537	39.78088	63.39594	148.974
ASMTL	54.04252	1232.031	514.5655	622.6517	58.82008	37.34635	102.4713	263.4401	1591.488
C14orf4	34.69077	48.53672	511.5875	500.4764	583.5056	249.4143	976.567	249.7908	39.51764
LRMP	116.8002	860.1645	868.3058	152.0421	457.0494	65.43512	929.8673	287.884	1823.107
AREG	51.62858	77.63576	33.10399	59.29699	39.00802	59.6722	51.67564	59.40855	43.28458
PDIA2	48.73621	44.43351	42.63885	46.45116	42.87049	46.00051	35.39646	41.21806	151.9879
ATF7IP2	240.4909	109.8509	263.6064	152.6856	326.2693	85.71371	294.9148	225.8706	324.5076
FAM135A	99.08781	713.4174	200.8448	39.5903	451.6509	338.0199	143.0797	284.6569	87.28468
LOC441931	134.7814	36.18205	41.88184	38.95233	39.72882	41.788	41.46922	36.91165	107.3512
MYH14	1068.955	67.3829	1229.532	61.11398	3121.922	798.5937	1293.516	1282.565	73.90018
MRPL18	52.03113	35.18256	234.9166	55.84137	168.4179	381.2329	200.2787	620.7514	62.54892
PRKRIP1	3083.619	3419.197	3528.985	3018.275	3123.239	3191.724	2629.766	3999.391	2324.936
KLHL25	147.7616	95.93389	93.93186	116.8869	84.58911	102.0405	63.09487	85.1002	280.1656
C7orf27	6949.043	7898.51	5264.258	6350.094	5150.13	6026.473	5543.227	6532.635	7257.245
DPYSL5	229.1665	149.3392	80.37973	142.4822	86.42933	116.3505	63.29614	68.46689	257.8437
CCDC47	425.6692	251.53	146.6905	278.3663	134.6364	189.7372	113.847	179.6221	581.2266
CLDN6	33.22015	33.85449	34.27751	37.61851	48.50912	60.11967	39.46421	44.96359	35.95555
SLC31A1	1186.834	1059.344	868.7836	832.9336	301.8069	308.9421	250.4303	646.162	2258.09
HOMER1	71.39587	284.2249	47.77404	58.05164	139.5177	226.461	134.5166	200.8633	46.2819
ZKSCAN4	74.04869	828.7122	886.9058	1539.944	737.9753	841.5298	835.5433	1172.227	1441.532
HEPACAM2	45.93329	113.1963	51.84537	84.08451	47.5878	36.06541	37.64551	51.17226	103.9517
C2orf37	556.6573	64.65855	159.1016	253.5059	117.2313	164.1217	178.9446	147.1768	39.60186
C12orf23	469.4678	261.5233	246.3062	437.4389	222.7418	293.2871	280.6389	169.5703	427.8122
TPK1	51.83713	52.55444	279.0156	41.5364	219.3146	258.3121	99.32933	40.71527	116.3725
SMUG1	2822.589	3660.568	4049.077	5251.107	3740.965	4238.42	5133.33	3505.573	2607.459
PLEKHH3	847.2343	1517.002	2879.854	1938.548	3580.291	1550.767	2324.049	1588.93	1107.565
C12orf62	650.5555	477.6297	491.6681	49.88876	351.2736	210.2419	560.104	682.1361	51.29809
KALRN	320.7579	102.3045	82.32675	106.1427	94.5913	140.2523	69.23811	84.14262	1836.677
NRG4	37.411	59.37084	392.2671	50.90955	167.2182	947.0319	373.6678	214.9686	89.59447
THBS3	12524.88	10190.69	8261.712	9830.251	9504.967	8835.41	7101.322	8621.262	11526.07
RBMXL2	56.80693	40.40657	41.70345	45.91056	47.72189	33.45171	40.05807	44.84632	39.40224
SLC16A4	72.16634	51.07742	42.91279	67.58983	87.85909	33.84831	38.73791	36.92422	90.51533
MYST1	259.8513	967.4809	1420.609	575.6727	1820.619	1653.479	1590.054	1952.414	35.26176
OR2J3	35.72036	54.71808	33.11237	42.74706	37.91657	32.73008	37.03438	66.35554	37.05185
CBS	10929.13	10373.26	4229.456	6976.755	12834.2	14191.9	5691.081	14122.25	6307.588
RAVER2	47.30855	49.79322	138.7198	52.96307	165.5611	34.31065	412.2112	148.7805	1034.168
MEFV	82.274	107.7955	62.9694	83.80821	65.16158	64.31962	55.40619	51.15899	130.2545
MAS1L	155.2514	137.7237	70.64155	51.96044	70.3239	77.75117	52.02171	59.33541	112.1197
CLVS2	61.31278	51.04431	46.34361	50.08926	53.79572	52.82262	56.03789	51.88664	56.21559
BEXL1	896.1648	1354.583	2472.742	970.0157	2831.789	2711.644	1912.271	1088.995	968.9976
ANAPC1	36.81411	34.52076	624.4202	38.9235	1172.112	825.4331	1045.544	907.9674	55.72881
ZNF431	90.06664	727.9493	1222.042	1057.5	761.947	619.0344	640.0424	556.1671	958.095

SRXN1	1006.247	59.39194	1077.692	1262.291	1145.776	55.73376	947.9872	1329.715	69.15817
LOC645039	85.40018	78.36099	60.78963	75.73399	44.16367	53.63427	46.52252	40.55169	151.7344
C6orf130	141.1571	148.632	185.0708	542.2888	668.8168	93.75751	555.0718	400.4518	193.0556
OR4P4	58.236	47.98135	43.44994	43.67137	37.33564	48.24408	48.70772	54.53959	151.7178
MRPS17	504.4093	328.0908	537.9539	506.8027	561.3323	1424.236	601.3802	556.9478	507.8352
CTNNA1	2718.973	3212.584	5280.586	3661.871	6616.262	4321.033	5317.144	6441.765	2087.325
PRKG2	88.67879	58.31949	59.54327	401.2803	40.2613	72.0354	91.76379	49.46919	58.0167
C22orf39	29.0002	476.9055	36.44948	419.5927	130.6454	53.47048	173.5414	47.98922	35.30917
HTR1D	283.8373	188.5092	106.885	197.9953	100.3228	149.8857	74.22293	97.86123	303.2291
C7orf53	39.30613	43.9787	55.33295	40.95805	96.72578	51.2865	54.18731	150.1485	46.98252
OR1F2P	59.89777	67.58999	46.58973	83.75632	42.19123	46.53392	38.28233	48.50922	47.53852
OXTR	386.525	187.7668	296.0611	200.3983	330.8846	352.1879	613.8599	163.3622	139.9009
GPR175	626.0251	314.7365	247.0713	220.2779	342.2252	209.4744	213.0863	299.4547	418.4813
ZNF667	1140.789	134.2625	173.1832	130.6767	54.4367	76.29141	100.0644	72.19392	154.5702
CCDC18	2529.523	171.3819	245.0803	168.077	333.3993	126.5941	208.5761	73.63444	187.4623
CTSF	136.5448	307.6656	121.9795	122.9337	147.9889	67.47772	91.38524	198.4624	167.8226
CAV3	86.62497	101.2547	57.3354	65.13904	53.38824	99.15958	59.40443	46.9155	126.3194
MOAP1	139.6676	97.25673	50.6483	97.73656	209.8839	225.9785	163.7336	192.8843	46.91857
FAM178B	88.3412	874.98	3043.916	5383.168	648.6904	1682.868	704.7653	718.9438	213.9911
SEMA4F	144.6372	53.19889	49.29194	120.0553	646.0027	60.74836	534.4201	61.28517	122.1984
FLJ35816	53.64493	45.54978	45.59	40.70304	44.238	40.91185	33.01735	40.43428	35.74552
ERBB2IP	1076.301	1345.011	409.6055	155.123	164.0222	355.8326	238.0358	430.9432	126.6816
THOC7	324.7304	190.2282	105.5371	222.7834	372.3034	532.1182	496.9686	382.503	1405.51
OR51V1	1639.084	40.16568	41.31879	38.11754	44.71448	49.37906	45.0678	41.62292	46.25667
PDE1B	1031.514	40.07681	387.3689	42.40093	234.5448	47.1814	498.248	344.8629	43.63234
LOC653620	139.87	97.38242	56.34223	202.8232	60.33651	45.09368	54.53641	71.9616	59.18484
C17orf90	416.563	188.2227	104.6815	152.7901	89.4268	150.4751	74.2752	84.95692	511.2287
PCDH19	142.3713	153.3171	74.62555	229.6238	191.6452	145.4845	102.8135	88.00178	383.1617
C2orf63	647.9308	1523.39	1914.161	1175.02	1483.151	2427.838	1202.319	1839.692	1072.671
LZTR1	16291.96	20456.32	22587.5	17783.1	20643.86	19685.56	22044.3	17320.13	13087.17
TGFA	1339.49	743.662	634.8154	804.2252	445.7124	255.2979	559.0784	625.0204	58.90606
KIAA1772	55.93328	84.9193	49.54686	93.82601	48.7999	41.57189	126.8982	42.98959	164.8964
ITPRIP	827.2626	1035.119	1925.93	770.5435	2228.366	1515.281	2516.452	1644.747	59.72203
TAS1R1	84.79233	46.8282	51.42043	57.09496	51.10924	49.92258	50.62881	57.22044	41.72537
DNM1	1547.477	483.6742	1569.114	41.10984	2132.952	332.3186	2302.202	2225.691	55.8092
GPC6	1378.784	289.9451	158.0836	333.8859	352.8671	525.3282	316.4342	502.8898	686.6232
LPCAT3	1348.324	1087.976	2107.662	982.9446	2387.889	1851.643	2017.714	1317.749	1796.637
LRG1	385.3456	318.7039	298.3792	291.7233	235.0985	312.809	193.7629	342.3928	373.3817
AP1GBP1	104.499	100.9527	185.8595	91.82766	58.63692	46.56395	94.63857	55.8536	169.9849
RAB39	98.90272	65.52349	60.75048	40.04441	52.84811	36.72312	51.41639	57.2718	86.32984
ZNF721	1022.818	2957.893	1902.443	1948.508	3237.864	3905.28	3270.721	3335.749	67.839
KLHL26	188.1912	121.1859	72.32118	103.1544	49.45774	64.33174	52.72498	72.09633	168.0069
DDX5	1622.898	1140.527	2420.149	848.2993	4798.492	2423.964	3490.298	2048.734	1446.289
MOSPD3	439.411	258.0679	231.8698	286.2987	162.5224	182.7453	125.2533	275.1464	582.4889
KCNJ6	80.95607	63.19171	61.18093	106.6837	59.62188	61.87185	225.403	157.4881	88.92451
LOC168474	69.79465	85.01302	59.74476	46.96149	48.06018	47.13662	44.7828	39.06525	52.75326
PDE3A	191.9907	95.00668	183.8202	512.8755	188.8084	219.947	116.9912	186.2296	198.4915

EPB41L2	197.5547	242.0507	109.7628	439.8579	72.25994	70.38907	65.20232	67.37326	76.66756
TAPBPL	2825.015	4496.414	2638.13	2401.123	935.4639	3465.567	2280.521	1377.736	3254.19
NHLH1	45.2919	56.71264	152.7157	60.13575	192.4137	550.731	91.88193	244.1219	83.7266
SOX9	41.42757	43.20185	46.15158	52.18526	40.11399	49.0552	34.61802	44.49853	34.71667
KRTAP19-5	63.89302	54.8808	36.78732	34.1521	48.51544	46.88601	64.24753	45.4155	32.88884
C1orf131	36.90628	43.87196	615.818	42.22545	1090.288	428.6778	836.95	42.85562	36.29202
C20orf135	51.08912	36.91383	37.04536	48.1242	45.88668	42.43891	37.11927	46.28214	57.41676
WDR13	46.68971	312.9674	2874.275	730.96	4968.542	2430.145	4787.353	4771.255	76.76646
CD24	936.3733	872.1531	892.5162	997.7227	939.3387	124.1585	508.4211	652.145	1385.7
KLHL31	166.8867	93.84107	74.28162	80.67087	70.55553	117.5921	54.92825	101.7324	111.1879
PPP4R1L	37.32786	54.47571	238.1263	42.24812	123.3723	33.72245	36.53874	33.45592	40.13635
ADAMTS9	46.30634	50.31648	46.46432	71.22631	39.76844	54.12413	58.24004	77.13432	37.2941
SLC14A1	88.57095	108.4258	190.8032	94.88446	174.1364	56.98218	126.9103	71.8488	113.0532
LSM8	32.19759	41.47703	311.0636	49.13973	105.6046	33.49266	67.38332	150.7039	40.67507
C10orf79	55.52972	56.72175	33.53254	38.77782	49.54387	39.30264	43.06301	36.33186	97.21404
PLEKHG1	3782.685	2625.69	1614.988	1520.358	1691.233	1181.805	883.5748	1070.195	1289.374
LIPI	58.82764	37.43645	41.5913	45.26474	35.36873	40.46075	35.18505	36.67046	63.27239
SNIP	38.14333	46.23957	39.731	86.78029	34.60073	51.34822	38.1231	332.2496	35.46014
PGAP3	51.07769	946.1569	1802.008	151.3532	2868.999	2364.073	2480.378	2795.501	83.82408
RILPL1	15044.83	15390.02	11743.77	15159.15	10228.14	14328.14	12259.88	13584.17	15384.71
TMTC2	81.52193	46.38765	51.85218	93.29771	44.6448	46.1701	44.83524	45.12456	68.73373
LTV1	399.6057	1390.279	1475.072	774.6664	2452.59	1884.67	1910.916	2075.315	391.1156
ALKBH4	2499.925	3284.673	1765.56	4208.08	1848.478	3369.425	1861.174	2610.219	4411.063
GMIP	36.5347	36.93029	429.0815	47.10088	195.184	43.88947	333.1736	167.9193	82.51035
C6orf170	61.49762	40.70975	136.3535	401.0926	103.6459	38.88193	195.2183	59.63747	50.14433
B4GALNT1	239.5085	1414.032	278.4497	769.2709	395.8772	741.4943	507.4911	1428.316	768.5737
C4orf17	43.28144	72.05402	50.51632	96.09946	42.40933	52.05372	52.51229	46.00911	101.5891
PFKP	40.43789	48.07748	2283.952	2272.511	2810.44	1288.308	1697.998	1819.999	37.22702
DHX37	129.8353	353.6398	70.06427	119.5167	59.98228	74.03106	48.79538	46.52819	149.5775
IFITM3	8156.85	9297.091	15262.63	11829.88	12547.36	13317.3	14770.74	14207.04	5251.335
CMYA5	81.06662	599.9742	1205.106	472.2165	1501.013	2410.344	3577.433	5944.355	1369.158
POU6F1	562.3347	355.1336	172.0191	289.3702	158.6544	228.2379	122.6904	152.8299	680.6692
ART4	46.84867	42.81087	48.73362	48.36044	38.33372	46.11118	49.01259	36.30368	39.16827
RBM12B	1079.119	1577.302	1782.788	2185.563	1714.188	2142.793	1569.957	1767.763	249.3503
TRIO	562.1309	185.0349	98.7346	268.4384	144.5502	63.94503	315.6852	144.0435	88.76716
FLYWCH2	891.1417	47.85449	629.4745	37.12589	709.6389	544.4053	1306.268	1317.917	45.25005
RAB5A	1305.227	614.9453	940.8449	947.8009	769.0009	776.4567	587.0159	97.07735	1222.805
MYF6	1343.891	74.17965	342.7538	74.68311	53.83649	630.5607	244.3485	231.3458	197.1592
SLC30A3	570.7217	253.2104	237.767	323.7441	131.7059	268.3022	113.0179	150.9146	236.8554
TOR1A	126.4467	95.80704	294.7701	564.006	194.1364	260.3993	648.3911	146.5848	309.4499
BANF2	119.0789	91.46048	76.13321	139.6534	72.75615	87.48611	56.38776	67.27864	214.5803
BIVM	56.77673	42.94366	195.4907	70.05318	211.2858	296.7781	168.7135	216.0295	44.91568
PPA1	58.78848	199.9339	1336.365	2012.197	931.7704	1349.281	1238.368	1162.884	37.83101
VRK1	88.19843	66.23725	219.8576	782.889	308.4216	530.0916	807.4794	584.3475	87.12291
FHL1	593.3741	309.0138	154.3717	368.9453	514.1639	389.4204	542.8905	359.7263	85.8602
WDR81	45.49016	301.9399	209.1983	48.46101	103.5033	53.92102	68.37856	51.36253	69.78516
ELA1	47.64994	58.15251	39.24381	34.30509	35.10688	256.6353	41.29745	58.17133	53.21011

MIER3	69.65841	79.56909	231.3884	324.6806	201.2768	81.45982	127.7247	171.4922	90.60424
NIPSNAP1	41.53592	36.90181	330.3063	36.32545	119.3628	46.24819	218.4266	50.23619	67.25973
C6orf163	816.2587	425.5986	384.3684	327.3132	67.91408	355.77	190.3259	216.7741	954.7268
GPR19	51.74106	46.85625	40.54752	39.91018	96.33839	46.87978	37.04221	39.79507	975.6157
GAB3	35.38873	34.31066	129.1833	52.6734	41.97557	42.26668	42.10783	33.86706	38.78406
ANKRD32	59.5203	33.92316	59.33633	67.24338	132.2096	87.41933	66.80347	125.7106	57.67886
BAK1	340.5791	200.5744	97.85909	155.0883	91.42137	184.5511	82.84488	60.2362	524.2907
KIAA1598	1289.601	1056.672	1018.221	1910.882	1280.211	1640.393	1618.736	1276.722	59.31136
C4orf37	65.70145	87.32428	73.20725	66.63068	69.57555	58.22295	89.01484	60.77223	124.4043
SIN3A	54.13457	39.70057	799.9766	1823.432	617.8514	991.2564	758.8427	687.5133	66.41822
C18orf21	39.02758	418.2746	680.0992	638.1411	719.7106	256.5496	819.4019	1048.91	35.88006
MYO1D	75.74942	63.40382	49.15819	72.21768	252.1146	39.42028	172.9693	34.96511	67.45892
CUL9	3676.268	2852.131	1460.194	1458.967	658.6484	1037.924	848.8174	1312.295	2823.742
JUN	5872.037	8739.484	8856.242	11958.26	10663.8	10278.21	14849.22	7670.39	6056.601
PPL	116.1481	394.2209	424.2795	294.4801	1474.315	664.9152	440.0255	407.4899	56.35828
TK1	136.7782	87.69809	460.41	85.63625	1131.175	82.06254	836.4626	372.1956	63.49522
TYMS	743.9761	161.3698	199.2739	285.3046	210.5141	233.7203	200.6078	247.1583	409.3081
CEACAM8	76.5547	76.49026	57.99697	57.57465	39.75113	63.53716	53.94326	45.85415	45.90152
SPRR2B	15053.55	13048.91	20573.27	6138.603	33864.83	14197	21243.88	28288.25	8652.5
IFRG15	930.2656	433.1468	561.6753	441.7259	450.8117	964.5778	439.7447	251.9515	102.2716
RXFP4	122.7513	78.50896	60.74333	73.35812	67.06053	107.2835	63.32117	65.17164	201.7121
LOC65362:	190.9252	76.86263	64.01213	77.16232	60.07239	86.01962	58.39827	59.72702	220.6778
CHD8	2767.421	3354.695	4429.413	2941.109	3124.362	3046.101	3188.569	5200.847	3251.038
MRPL23	62.61988	60.42195	51.0404	919.008	207.0757	45.75827	52.13873	61.85276	50.03413
GNG3	53.66602	52.83042	249.8074	105.3796	48.9981	81.19463	56.58781	60.9041	68.88221
C16orf74	31.31324	43.27441	36.52454	61.08986	149.8443	60.7318	86.44178	35.33882	43.69321
C16orf3	103.9104	136.3598	80.14272	139.1369	91.53554	113.2176	56.87246	80.83063	241.5531
VSIG1	67.40091	142.4408	117.7494	287.6749	73.284	218.5201	151.6711	82.64793	609.1409
PSD	47.49641	86.74187	99.08932	65.36543	87.01938	66.15718	107.8204	102.3309	55.5649
MGC27121	71.44099	67.4416	46.89383	51.23255	50.61433	39.79777	40.52177	40.25045	68.7336
ADAT3	60.57515	431.6742	166.1135	57.3058	220.3678	54.21362	276.5722	154.1035	74.10153
KRT84	103.8683	73.7068	66.81631	99.14391	51.36652	52.43036	48.38936	48.17889	88.04742
LIN7A	57.05275	129.7029	160.4829	92.12869	36.42403	43.59124	48.00795	59.30408	50.14439
ENTPD4	134.5248	125.4461	389.2705	89.33658	61.80766	63.50352	58.85986	72.83553	392.1677
FLJ23356	42.86337	40.91535	45.02517	42.93367	172.434	48.92189	172.988	51.93314	1374.052
SV2B	261.2284	108.7493	62.78219	174.131	142.0928	113.6357	156.5393	166.7352	105.2414
C20orf30	368.9826	218.4548	192.8312	355.1667	283.6879	258.8746	487.3823	158.2734	479.3738
RBMY1E	52.07212	50.96831	55.71712	43.09343	34.83105	32.37722	33.4012	40.57163	60.97453
PDIK1L	746.9305	915.8015	440.1013	275.5375	263.7549	553.8239	315.6878	158.7083	469.0275
C17orf78	194.6038	164.8864	92.50916	152.3015	71.73893	99.94342	70.58253	88.64806	299.2163
ZNF793	682.1011	75.82211	52.20787	70.80108	150.2085	98.72649	162.2574	44.17214	39.20992
TLX3	79.11137	46.57247	52.48321	70.18181	49.6896	48.46566	55.34542	58.38942	46.34645
SLC6A8	187.3919	137.579	88.01255	76.09707	413.0019	95.42886	68.45038	253.4772	333.3653
FAM55D	50.52502	49.70735	41.32118	60.53835	41.32507	40.35994	42.44358	43.83154	69.32579
UBL4B	120.2697	513.1866	56.8792	132.9254	64.30061	119.3529	65.23445	55.46306	247.3622
RASL12	1021.778	2789.042	3521.579	1051.999	5650.704	2856.711	4317.627	2473.478	427.2474
ACADVL	9304.396	9222.028	13095.57	8407.55	15295.62	12030.25	14612.11	8946.507	6274.568

NT5C	14798.41	19338.83	17911.91	19494.99	16456.86	17175.62	16533.17	17705.52	17491.85
NTNG2	48.91947	53.39455	344.8069	711.1705	45.30191	53.159	125.2347	46.16195	56.371
C14orf45	90.47028	37.88997	32.31009	36.34935	58.64738	54.37524	46.16654	40.41677	36.39461
T-SP1	236.3398	180.2819	97.88403	190.1743	80.18285	121.4114	67.45097	89.04066	374.1371
BEX5	1028.376	68.55666	156.4776	499.6662	112.8249	54.83914	512.9493	39.42813	48.57037
FAM109B	77.36433	43.81394	42.73631	58.23184	51.49184	37.40224	57.59073	50.52576	57.29576
CCDC108	157.071	87.62075	86.9967	78.0329	74.06564	60.91683	54.16428	70.36695	277.3716
TNR	82.63873	34.03455	38.52881	40.02533	37.55716	37.19057	39.09403	46.24307	54.93658
CPNE5	59.49374	39.6987	68.23377	56.29023	42.9681	46.5011	137.403	36.08045	40.81063
ZNF165	364.1992	1248.877	1714.166	1657.978	2716.993	1324.558	2220.754	1870.623	41.73915
VPS53	40.44246	37.77929	493.5924	49.28559	397.3734	667.5068	299.9018	231.6115	63.05711
C5orf48	40.36064	49.4485	40.10308	36.47205	38.78726	47.16181	33.59428	51.06786	83.37556
PDE7B	52.31295	846.5497	808.7571	44.57242	1763.193	233.0546	1651.809	1159.171	41.17911
NR1I2	39.62175	41.70724	35.75895	33.21317	49.47545	39.321	37.18772	36.89787	39.32938
GRSF1	1665.527	1311.765	1923.52	1422.881	1863.607	1633.834	2206.092	2545.015	2458.51
MESP1	62.82833	64.3396	150.3076	40.05359	184.8989	94.99201	145.5022	389.2129	75.95914
SFN	6329.016	7412.869	13604.68	8739.087	17591.23	8414.587	18892.78	21202.88	5509.99
C1orf140	49.35732	42.84288	48.17406	52.06018	47.04238	53.8478	44.6181	44.84695	61.42865
HEATR2	1101.882	54.84161	34.17869	36.34639	37.41123	47.39097	81.39631	34.98994	52.10414
PARP8	342.9146	1720.525	1489.522	303.437	154.8835	431.3038	493.4442	868.3697	567.1661
C16orf85	231.9017	124.6685	74.29959	107.0749	57.32081	73.56696	60.96578	65.74506	186.8692
FRAS1	47.05082	39.94328	46.6477	49.65566	132.8417	42.59373	92.43732	43.26933	42.22989
LOC730862	55.96204	58.84036	53.00268	65.72942	50.28463	78.37968	49.88928	72.57005	73.11436
HEXB	1873.4	1953.365	1598.648	1109.161	3188.683	1385.663	3554.746	3693.127	352.0702
ESPN	12607.43	10177.53	7729.938	13726.59	3829.282	13536.87	7264.122	8627.706	9826.732
ZNF18	38.24376	260.8375	910.2693	81.56323	646.244	722.0299	667.6752	1085.993	68.75694
MS4A2	475.8519	383.5208	215.6587	273.2044	244.6438	422.7538	247.0431	268.6548	674.2733
PDCD1	35.85277	49.90444	556.2448	62.54969	189.4199	42.65685	34.14365	121.5709	36.36473
TRAF3IP1	36.26141	34.70544	31.60466	50.84412	38.54451	49.95427	42.5857	40.56776	60.50281
SPACA4	62.7142	53.14637	52.78081	82.65047	58.71198	60.42945	51.42319	49.40886	62.8094
PLEKHH2	45.91204	419.9474	338.1477	50.52597	791.2385	784.437	324.5067	393.0496	35.99996
GLDC	59.35754	58.93018	152.4319	47.13488	137.7633	48.07591	43.64199	49.51955	51.25732
MYBBP1A	45.36607	49.60548	65.53203	41.85597	61.39921	72.65944	52.46387	51.2211	82.48573
UBE2E1	20268.84	20220.97	19746.66	19649.09	19260.62	19514.01	16950.65	19447.46	18681.25
ZNF235	64.86936	64.71522	60.87537	59.0433	44.036	37.46333	92.50409	49.19454	49.5737
CSDC2	5768.316	4374.154	1985.255	3559.839	682.3217	3215.792	2342.779	2618.585	3940.889
OR4C46	48.50726	72.63554	67.15226	66.31362	52.00341	59.16839	51.30097	39.36497	102.2688
TMEM182	685.9019	249.2776	44.77063	42.61514	59.53052	154.9992	85.3316	222.6368	120.7352
USP14	1749.865	545.9655	406.0529	1476.545	279.9998	647.7976	420.2581	827.4792	586.0803
MCM3	57.93996	89.70303	53.02499	449.2669	118.5813	261.142	74.78557	150.6201	192.1113
C14orf145	39.71995	37.87914	98.32322	40.5971	133.6844	197.9625	83.48906	33.24516	67.99205
SSR3	480.7241	130.3391	120.8594	142.722	117.9335	153.3185	85.10305	121.2546	491.3455
PAWR	2720.218	2175.625	2945.229	3828.679	2959.04	2534.505	5337.677	2590.427	1905.185
FATE1	56.66521	53.12646	72.1391	89.33501	75.01013	205.8409	90.16637	160.0324	57.90531
C19orf62	43.16795	48.35424	56.51728	58.43891	91.6133	43.17271	181.6816	177.8402	94.37964
UBTF	1909.644	496.5659	357.5185	68.01803	133.481	39.95398	251.6295	576.0131	60.5755
ATAD5	200.2272	281.15	77.85535	260.975	66.40081	147.5288	70.472	70.17598	440.8395

C1orf182	63.79395	125.5064	55.60551	65.03892	55.97415	59.87254	56.10664	54.28555	115.1208
B9D2	2845.893	2808.405	2431.791	1789.485	1943.708	1908.381	1877.847	2142.074	2682.027
LHX2	32.74213	35.90345	33.52381	39.22542	36.98354	33.92628	31.37401	76.70978	77.97517
C14orf28	61.22038	282.5056	80.51215	36.8623	157.9702	195.5677	161.9971	161.1756	84.6673
AQP5	45.55561	47.85278	37.79639	65.94882	47.47453	50.47027	39.06522	46.68837	56.03296
MARCKS	905.8895	360.9177	1853.075	1374.257	1748.281	1346.046	2177.827	1886.064	1394.238
UBAC1	8701.231	10064.61	15384.22	6742.835	15588.43	15380.38	17525.82	19257.6	7653.52
SHC4	231.908	149.2172	80.84875	160.8706	57.59399	79.9565	121.0597	78.35703	126.366
C20orf118	95.11331	111.3986	74.86298	126.0214	68.37801	89.28207	63.23609	91.25546	245.858
MIF	1730.72	1304.044	2742.611	1441.712	2906.212	1463.805	2344.048	2669.624	108.8592
TTC33	59.84777	54.17421	51.5484	34.48513	148.5959	34.15934	153.441	37.1744	46.44519
MRPS36	4613.949	4511.557	3194.88	3281.223	2673.173	3373.504	1718.175	2693.523	4793.54
LDHA	1547.484	393.4525	2224.488	2867.835	3842.269	1457.283	2511.161	1860.905	122.1294
SPACA1	430.9402	246.8673	234.0739	266.5573	418.5293	320.9279	197.6805	347.7228	525.6126
RC3H1	123.3882	66.19753	69.81747	119.197	58.57669	116.1536	57.82735	68.12016	120.456
GOLGA1	94.32224	66.54589	70.22897	126.8257	191.4654	66.5456	53.63985	439.9394	73.90302
TAS2R8	54.68483	41.37854	36.38234	34.48121	44.06461	47.49339	41.02276	44.45605	50.75737
TINAGL1	49.96056	2032.306	1038.956	6743.259	404.95	1250.15	1065.968	1840.006	34.00439
PLA2G5	71.17607	874.6149	49.45924	51.66282	34.90102	55.59102	44.55177	46.77668	86.02149
KIAA1797	131.6577	52.05452	54.92231	75.26768	133.6695	51.43522	50.28789	154.6696	72.51399
FAM104A	12121.76	10897.92	13184.05	12242.26	11517.18	14095.15	13232.06	16689.82	10365.86
DHRS1	5283.188	4456.928	5652.523	5478.466	4864.617	5167.481	4349.893	6095.595	4145.813
NICN1	12358.09	12214.39	10118.2	12015.1	8711.529	10756.76	9163.433	10765.57	10222.51
USP44	38.02024	42.41158	33.45192	32.13262	37.34777	32.53417	37.24487	33.39696	43.15836
PRPF18	53.59222	93.92426	128.3333	112.4067	124.335	177.8187	105.6052	89.00564	518.1725
STGC3	337.5458	218.2846	98.77548	222.2212	121.8	174.534	91.93883	131.2264	485.9438
KRT32	51.40996	80.85769	48.92223	58.31618	55.0938	89.8912	45.44311	44.50392	65.34286
ZNF682	54.15512	226.1408	49.38105	55.34081	61.97968	55.01519	62.1179	93.73942	53.33393
PRSS48	397.4958	218.6899	113.0919	205.3696	71.1195	140.0853	73.24001	114.2235	464.4785
IFI30	1573.398	55.62368	1815.44	74.71505	1148.678	986.4393	2103.313	1575.574	46.44407
NDST1	639.5864	1431.606	820.8901	1840.438	575.8806	254.0187	312.8584	480.388	913.4279
CYP1A1	123.7653	101.3314	78.93573	73.06386	75.80278	114.2607	55.25256	75.57378	264.0254
PKD2	129.267	157.4839	72.881	157.5886	76.68288	115.9549	53.18154	63.84413	227.258
BLID	50.92416	93.09678	46.79238	59.25344	45.50167	56.74718	51.42067	59.34614	181.3078
HRG	88.05429	49.90151	50.39841	60.35428	54.4854	69.51612	46.35952	37.8621	58.21193
CLEC11A	37.89592	58.3463	54.93811	46.99679	48.86553	37.67514	107.479	45.74105	40.70971
ADORA2A	89.06174	56.65559	50.61316	70.04928	55.0727	57.29886	160.4847	58.30112	122.3793
NDUFB5	414.421	2805.833	2901.489	1587.796	3329.459	2514.944	2501.718	5254.54	1798.164
IGF2BP3	91.0092	104.4624	140.212	153.4359	329.0397	66.98863	422.6247	76.88993	1131.451
IKZF2	197.924	112.8001	76.23186	145.4879	59.17505	59.37191	64.07929	59.576	83.64873
ALDH1A3	77.70231	1942.438	1070.26	42.5994	803.4015	1187.529	1774.551	1006.123	40.37992
FAM8A1	116.3072	136.2519	71.13956	138.7881	70.78718	74.99716	54.6803	47.59277	211.3677
DHRS7	2359.566	1690.951	1045.602	2109.057	659.5945	1289.556	1017.476	832.7042	2566.021
FLJ45139	95.18851	48.16938	46.10739	50.92996	49.53852	38.5538	37.03095	39.7131	37.3525
LAIR2	65.70291	618.0094	1276.795	226.88	260.2075	801.9587	882.1328	739.8046	63.25573
ACVRL1	41.44382	34.96035	649.1747	38.59864	710.6694	49.92299	499.6251	688.713	60.41319
GINS4	141.8313	69.78551	65.69737	128.0046	47.4451	52.74392	57.59287	63.05515	126.3865

CPA2	95.59148	41.99956	57.7868	41.89432	50.12902	41.87282	40.71134	50.34752	43.48669
CSTF2T	34.68536	53.15485	185.2655	37.18275	215.4619	445.1809	257.6724	136.3164	33.5886
CNTD2	257.7792	113.6869	93.6219	195.2995	67.86657	95.37994	76.56351	115.3634	314.8135
PADI4	1547.109	48.28267	1659.575	1292.946	220.0589	1645.95	842.0253	578.1612	1393.135
C17orf71	456.0088	234.1517	132.5241	288.4765	242.1819	208.6022	192.7367	262.7195	569.7631
CBARA1	1567.07	379.9362	315.796	370.3083	578.3834	348.4947	695.3091	482.2015	749.6378
TMEM161I	49.60499	60.97847	56.31906	65.63286	62.86518	77.67162	57.42	57.55704	99.89871
UBAP1	36.92614	38.55708	844.2415	40.43239	2282.399	43.01395	1759.296	253.3293	52.66394
NPLOC4	43.32117	105.0709	938.3943	1142.846	1703.879	661.7306	1676.819	1458.409	50.34501
SPTBN5	39.3748	51.50553	227.8965	43.51474	39.18664	43.0439	309.3975	236.8456	36.35785
PRPH2	146.4513	68.33389	61.39869	82.19305	168.6541	111.7293	68.36409	87.44926	153.6833
PPIG	9684.745	10270.74	7036.78	9598.56	5843.955	6836.714	6494.491	9282.25	10938.15
HRSP12	34.29949	33.01189	38.80607	35.32391	33.02956	31.32272	73.39587	35.78385	60.92385
SPOPL	254.5401	295.1318	100.9631	329.7308	97.7194	137.4073	84.07346	91.48581	303.7377
STRA6	64.76529	43.3917	227.7852	59.06261	50.20243	51.33863	50.94726	46.02478	38.75089
IGF2R	7273.809	9407.481	12888	11508.18	14966.7	11325.38	16515.83	12740	4908.946
C14orf105	226.044	193.1381	107.6858	89.52931	157.611	159.4349	104.7206	107.9125	309.2688
PKNOX2	1698.963	485.4699	67.71625	613.0767	320.6009	304.8109	254.9728	868.1412	104.7132
HINT1	1545.461	847.1592	283.7643	436.2779	497.6327	223.5537	248.2566	352.2444	239.0942
ESX1	51.43711	37.78769	58.84505	50.82152	41.52657	38.46851	43.55273	36.62837	51.32374
SFRS17A	1655.345	726.4603	259.1073	100.6575	185.7416	65.1193	264.2141	680.3862	48.01309
GYS2	63.51933	76.49117	42.61324	53.19417	45.89145	57.26062	48.4961	49.94805	45.18293
TAS2R31	36.31404	44.02977	46.17446	47.70619	42.59753	40.32705	37.48034	34.12144	54.70954
KIAA0182	482.7022	336.7434	366.9794	390.9894	475.3874	489.7778	299.8228	200.8003	715.4807
PFTK2	34.23851	40.02311	40.2196	36.16154	36.54953	38.2893	41.43583	53.47664	60.8533
SKA2	494.2128	292.8264	200.9868	345.3713	195.3414	261.6286	217.6378	315.3433	670.3159
LY86	639.1423	341.9956	1232.572	511.4434	191.2515	830.7629	1082.297	803.6818	499.7414
ART1	404.442	224.2877	117.1348	210.8622	105.5052	143.7709	102.5027	146.1487	501.3081
TNS1	162.1281	161.6422	79.26985	141.718	60.53473	95.75714	178.1685	264.1259	421.8625
OR13D1	36.34705	32.3855	42.71686	35.07281	37.9801	32.56997	40.94315	28.31016	94.58782
GNG2	898.1541	746.0242	315.156	35.01632	103.3677	40.52692	146.5597	95.25783	39.13302
ANP32A	418.863	307.1594	289.6483	413.7215	159.4109	276.2786	198.7528	335.5695	781.6817
RPS6KA6	41.88338	41.13115	37.29621	37.431	145.3163	45.58523	45.38665	34.4085	42.77129
SNCAIP	8202.575	9260.504	8165.513	7693.234	8621.438	8656.79	7800.192	7980.447	8346.214
MICAL1	1422.911	774.861	2165.977	758.7009	2330.406	453.1917	1999.826	1841.244	65.37459
PCNP	2098.633	104.8399	1306.568	2381.138	4664.589	1484.563	3173.691	1658.403	2846.565
FAM46B	200.7347	724.0433	2735.794	777.4792	4585.748	1458.982	2112.886	2640.413	116.8071
FLJ45121	98.23678	76.14801	53.86095	78.0608	54.12574	68.24604	50.00668	46.4151	103.516
RPL10A	1776.481	70.04131	236.7302	965.8878	901.4867	882.1259	338.1425	416.1254	2789.46
LOC550631	39.5073	41.72856	39.91377	39.77672	87.10858	59.49469	119.5329	186.3705	33.99918
BCAS4	194.558	375.9136	486.7155	546.6429	562.7485	444.0303	1082.313	329.8056	235.0726
KIF14	320.2601	395.4604	86.05032	1550.095	213.7727	249.9494	457.8385	361.2254	1038.255
GPR128	55.61294	48.84755	40.13413	47.8007	37.1103	36.65478	48.17623	38.94414	46.41465
MMP23B	49.79647	39.60378	41.93373	43.28984	47.99193	53.63481	48.94588	54.23079	90.12927
TCEB3	583.2589	645.8455	1338.375	1429.915	1166.511	1773.555	1481.919	1350.15	559.7656
LYVE1	1941.444	1551.668	213.0674	33.56305	528.8181	579.2051	472.5966	463.1227	1148.457
ATP7A	58.19025	54.03395	171.6341	60.04184	58.44067	45.37791	51.25774	52.5249	1171.818

C1QL4	78.7793	63.71756	197.4567	59.752	52.62273	36.68941	219.5838	55.21565	50.34715
FAS	397.2497	50.20185	138.2192	175.0697	117.5827	57.29308	87.02917	43.02869	98.30564
RUNDC3B	6221.501	7804.341	6968.809	5223.938	5015.96	4747.634	4532.324	4786.242	6531.54
C11orf24	285.1469	73.20004	143.0208	134.9994	125.9652	112.8247	193.1811	188.9924	1099.515
GSTO1	62.0168	76.41885	145.848	504.9705	128.1437	188.9543	230.9843	195.5089	158.3234
C19orf22	181.7546	732.1196	1171.063	307.3896	1279.834	430.0858	1417.175	1341.538	259.115
RGS8	47.6995	52.25074	38.81158	48.34138	50.78025	49.8645	47.86837	54.34938	74.78788
LCE3E	12838.56	13244.09	5441.867	6909.403	16622.09	6472.857	8326.88	12992.9	11163.04
RGS16	38.81397	50.42059	183.4716	45.52834	52.27509	40.7579	127.6773	57.79002	94.44482
RBPMS2	9338.135	10507.12	5881.848	4959.592	6824.038	6053.867	5948.598	8375.092	7469.31
PNPLA1	34.97053	35.58916	66.0647	41.14239	346.5314	154.9934	130.1769	129.2447	623.1495
RPESP	3721.262	2087.683	829.5037	573.2698	2354.634	1826.809	1164.301	788.7829	2898.185
MS4A8B	68.22759	76.34189	53.63427	54.0276	48.53205	62.06576	49.0241	60.62628	80.90591
OR8K3	63.14931	39.15438	36.36631	31.00024	43.1314	47.15063	39.69349	33.26756	57.53251
KCNJ10	173.6733	169.5985	235.6521	81.2395	151.2455	203.3483	79.63892	76.61936	835.4173
SMCR7	190.6671	123.7465	64.57097	108.9084	69.27589	93.65421	58.14839	72.64236	84.8604
IQSEC1	373.7314	215.3298	113.0863	240.1093	267.453	193.0417	203.1288	170.5872	545.6887
RTCD1	35.41536	219.8215	473.4741	357.3373	451.3661	291.0451	331.1495	480.8494	57.36216
TM4SF19	68.12449	74.25083	890.3342	328.9945	39.61983	433.3225	958.5803	1648.283	70.05247
PPP1R7	1573.269	1373.211	56.05257	142.2858	299.2884	403.0581	285.6431	218.8521	59.22975
FAM3A	6495.618	7947.824	7709.508	7313.584	8156.751	7017.546	6839.543	7100.812	8483.911
PRKACG	46.59469	40.60328	41.98771	50.6301	42.31495	42.71812	35.11655	57.12457	36.60856
PLCD4	12232.28	13183.92	9145.143	12683.15	10607.94	13205.93	8334.706	14528.03	12274.63
ARL11	5378.39	3218.875	2314.21	3598.692	1352.483	2889.531	1709.769	2272.158	7596.233
FOXI2	93.63961	91.73626	59.43349	50.9429	285.5337	56.20858	50.60115	61.24045	80.91837
DUSP4	38.83256	42.74008	31.7231	45.84715	33.40201	35.69453	40.24889	52.96437	33.92222
ACAP1	86.04826	2145.998	741.1527	75.72208	821.2234	74.2664	697.4668	522.0382	77.27415
RHBDF1	8415.703	9970.688	11571.91	12691.56	15965.39	13957.64	13092.61	9965.815	9128.416
HLA-DQA1	46.05893	40.28514	577.1956	932.5397	317.832	720.8318	227.8149	246.285	45.20256
NPHP4	89.40269	45.97161	57.75019	56.73124	59.6289	67.75985	50.15126	50.43409	58.81178
MAGEB4	44.66476	50.91531	48.61026	63.5245	45.11607	45.86664	43.00021	44.15844	58.18964
ZNF92	44.88414	46.43393	109.1994	48.14904	99.48749	38.28033	41.88821	49.62004	111.0619
TBX18	240.4961	174.7174	84.60826	188.1232	87.89759	154.6809	64.32688	88.28664	230.9726
SPANXN4	39.69785	32.6132	33.6979	34.8089	31.77023	36.19019	37.19993	57.00722	50.74392
PLA2G12B	143.0362	428.9165	88.45956	139.6413	70.09228	68.95695	57.5989	90.12506	278.3734
ITIH2	132.6714	71.14797	76.75694	68.798	68.71616	71.53503	55.36103	73.83566	144.3114
CEP68	1262.324	36.39609	44.31167	51.77775	37.37968	375.9735	35.00139	198.1054	36.91391
TPSD1	50.91616	40.99195	37.34632	64.70757	34.27639	40.20974	37.10398	37.36872	48.59168
LRRC66	37.48275	40.88668	45.32112	49.96649	111.9385	42.43821	169.1532	31.97591	44.32615
TMEM180	3485.28	2618.135	1651.998	2927.989	1028.298	2136.06	935.9871	1577.982	3078.566
PYCR2	349.8713	165.2822	128.7549	243.9826	163.414	188.9904	121.0376	165.1247	470.0344
FAM36A	55.45753	1018.303	190.2426	496.3014	1309.994	49.11999	1161.129	752.0206	296.7128
ZFHX3	56.83952	44.5244	34.96231	90.25737	445.2162	56.50871	747.5488	345.6387	52.19535
GUCY1A3	55.54897	675.191	886.3361	652.0872	517.7439	684.6385	691.934	1006.791	69.53651
APLF	78.71935	290.0888	107.4391	384.2226	112.6687	55.98378	63.26559	70.48458	69.73305
C21orf67	364.3236	234.9059	107.333	275.8272	116.4949	168.2872	75.09856	107.7468	439.9295
C20orf96	44.86539	50.42549	54.85229	61.73363	301.2913	51.31918	498.184	37.03552	43.29929

TMEM77	44.6186	36.16949	640.274	403.158	206.6256	220.2683	286.8746	189.0372	50.3142
CYP39A1	41.08661	48.45661	65.49039	62.87729	43.57962	36.24337	48.07469	35.46437	108.5582
FMO3	52.99212	71.55507	44.75108	44.20367	282.8045	55.97885	47.76024	45.75287	36.95797
CPN1	273.3489	223.5577	94.26519	223.867	56.59155	138.1411	76.90348	74.3139	474.5524
OR4A16	68.69806	67.80998	59.90757	71.17671	53.04817	51.54048	54.49445	63.53259	59.07162
FSCN1	16913.85	17261.94	17860.3	19473.18	16644.95	17280.11	18849.41	21045.63	15159.53
RNF40	1383.997	876.6371	249.6985	1420.582	601.8564	231.7771	390.7313	603.3322	148.7811
HSD17B2	61.39921	43.30741	457.49	44.1194	133.1682	36.3998	407.5981	120.9898	39.66692
LBX2	320.4221	175.7733	104.1908	134.9176	96.45888	152.4004	87.30466	85.15849	380.6046
ZNF25	110.24	330.0013	779.5589	738.3062	813.9385	363.1869	553.2646	557.2888	151.2758
ROCK1	35.19886	38.11441	293.1593	974.4211	349.1916	316.0661	407.8553	316.3309	43.89049
C1orf97	391.475	227.5658	991.9978	206.0458	771.1885	345.2532	2001.64	1614.882	406.2281
EHD2	774.0236	439.4971	427.2261	849.2982	526.3894	394.9037	791.3688	1295.481	1053.006
STRBP	50.41817	56.83992	720.2863	579.8445	1595.536	779.8923	1405.844	912.925	45.23515
MYO5C	39.88058	983.0699	291.8663	55.25852	2304.785	51.87009	500.0583	332.7848	51.98227
ALKBH6	219.022	135.5177	60.61195	140.049	82.27348	80.75681	63.45831	58.73085	110.9364
NAALADL2	144.2081	162.4174	76.6853	172.1146	63.23272	146.2852	68.76761	61.64543	238.4903
SAC	192.1572	152.5507	61.87026	95.575	60.86654	115.5388	55.74561	85.7047	159.3602
SBNO2	87.16924	266.4732	562.2225	154.6775	487.4583	318.5103	385.7649	446.4223	828.7971
COMMD9	43.93393	39.82298	113.2761	48.85044	117.9286	51.9707	164.0224	41.62883	38.25262
TBKBP1	69.55433	65.55717	44.23769	45.54233	46.41815	40.06758	40.41676	47.30294	65.09946
LOC65380	38.07038	1029.44	34.87268	33.11969	41.93625	33.06117	144.6947	45.20965	39.22955
NPR2	48.29906	406.0972	40.36153	50.20467	36.30488	51.72538	235.3582	267.118	1385.566
AKAP3	101.0744	160.2922	66.73957	66.74323	48.85728	64.69859	49.09944	50.43337	70.84284
HIST1H2BN	58.78895	828.9911	325.6774	1055.397	240.5	751.248	507.0408	314.4636	55.15953
C11orf85	94.40865	36.33348	35.75129	36.60596	44.11462	44.58686	38.63668	36.66735	35.99614
RPS6	4114.702	2730.902	3586.656	5091.044	2792.93	3471.046	2245.704	2179.939	3151.76
POLR2J4	32.30482	41.89679	35.16211	45.87788	36.36473	49.75602	82.92934	42.58897	37.3387
C15orf27	57.56436	45.16814	36.46709	34.92495	45.13865	65.45163	40.51844	40.82947	74.40425
MORC1	70.07552	65.7253	50.41539	60.51357	41.99922	46.58847	44.6357	46.66482	59.0646
CLEC4D	351.4819	226.3016	103.1348	233.9225	88.12811	128.9481	79.63363	94.45399	347.5204
ARHGEF19	149.4584	95.64922	1502.55	110.1386	2220.024	1909.49	1938.979	2042.464	95.53757
CBR1	2427.53	1850.826	811.8959	1401.758	946.8426	463.3451	959.4445	726.3705	3174.619
PSMC3	7741.058	7674.488	6075.315	7460.772	5828.878	5918.523	6216.479	7771.729	7940.912
MYL6	9824.329	12949.97	20479.51	14027.98	21000.62	17828.22	23097.03	22471.69	7771.939
ATP5SL	119.6612	129.4551	453.2058	119.7868	1052.042	59.06966	695.1214	399.3918	270.6762
GATC	47.84541	34.97863	469.5911	933.1528	291.0624	273.3691	370.2449	543.3203	46.19287
LDLRAP1	2569.361	737.4055	927.4786	50.48109	1311.424	375.4672	991.7209	450.6872	49.51345
THAP9	35.10382	288.9532	59.55546	33.44538	37.76977	51.26008	53.84272	48.79758	51.41544
RFPL4B	53.66285	45.72498	41.59348	56.57464	37.15316	62.71933	37.58393	40.13014	54.35711
XRN1	7670.174	9409.318	9665.215	7612.759	8275.977	10064.09	8957.669	10864.64	6137.356
TCERG1L	47.12354	38.86432	34.60407	42.64515	36.11308	36.73963	40.41491	49.50362	37.95291
TAS2R1	160.1418	62.30204	54.81298	126.6156	61.06102	58.9271	60.72726	82.38178	199.6616
IL33	136.9401	122.1743	192.6004	68.36618	55.43861	97.62759	58.92001	66.86371	105.8785
STEAP2	808.03	453.3323	222.883	46.00237	276.2326	279.1883	213.2908	291.8267	721.1185
ING4	1477.642	1530.786	992.9253	1246.136	850.3724	1322.553	769.5291	1170.354	1579.435
HRIHFB212	40.04457	34.97865	42.80016	834.0029	173.5565	58.6694	112.2783	236.532	33.66301

FAM118A	10154.54	6013.461	4339.363	8159.192	3202.001	3169.797	3588.573	7204.629	9749.544
OR6C74	89.23778	43.35863	39.32298	50.23645	43.64827	41.65632	39.04667	48.34161	90.00663
LOC729021	1881.634	194.876	84.27376	194.6469	81.57356	110.9212	159.5731	115.6012	388.225
FER1L4	44.66275	55.98961	155.0177	40.57194	50.54554	35.25421	74.64499	31.72332	45.73449
KIAA1543	1613.998	36.37183	312.2773	1972.004	264.5767	53.81781	49.50954	49.05693	540.3845
PITPNB	1185.688	901.8735	521.4944	787.7081	433.2644	269.7112	295.6172	429.7517	1026.099
HRASLS2	377.8756	186.4183	88.03465	162.3323	78.53451	142.5426	91.91129	84.58784	310.1823
CLCC1	290.4117	69.63075	59.57098	69.97823	442.0571	52.00443	258.2086	800.2181	99.87214
DCP1A	571.6733	38.33413	110.6144	35.87643	171.5111	142.3544	58.96482	36.22557	39.66157
TSPAN7	33.44095	48.05964	190.0989	44.4053	48.95135	54.13546	258.3872	42.46156	186.5106
IMMP2L	71.31893	502.4978	778.0507	123.0251	652.84	1042.729	563.5969	1236.222	159.6525
PRAMEF12	36.35328	55.82561	45.71748	64.72958	48.1403	63.8993	40.06942	49.07487	52.55765
DNAJB7	56.1326	69.50635	49.52954	42.49182	50.90635	59.14251	39.01172	40.60479	75.72653
APOF	50.33494	31.61266	42.51137	43.62064	49.56023	46.12683	33.33313	35.84195	41.60236
C9orf125	35.94052	35.56361	139.9892	36.42557	204.1472	60.28982	196.3891	127.0956	33.69527
CC2D2B	56.25687	79.16171	188.4283	88.59071	226.4151	66.07026	178.0218	45.46757	57.38145
BTBD10	255.4456	138.338	430.7343	673.0559	219.5098	334.553	541.3481	242.7213	118.5049
PRL	54.01334	46.60602	38.08768	37.94573	34.2068	34.04854	39.59416	35.20661	55.27857
NTF3	47.55361	51.0546	52.47262	151.3967	875.0665	68.94989	180.6514	54.47536	74.43243
CYP4F12	44.65631	1155.825	2329.477	36.36364	1842.982	1862.165	713.3586	35.66596	39.62539
FAM49B	93.83072	100.9938	89.10854	112.55	122.5614	183.3973	89.05471	117.8839	31.34173
LRFN4	95.24289	45.87693	35.14858	406.139	89.48186	44.39259	70.81064	176.1334	60.03902
XBP1	1031.439	2243.452	1066.127	556.1869	1441.969	1597.917	1160.602	763.5215	1451.176
RHBDD2	66.33705	63.47731	131.6306	65.74799	321.3774	138.4139	278.6096	264.7505	63.53904
C1orf157	142.4207	89.66214	55.5488	87.73817	66.05042	61.51841	51.24696	74.5972	216.5195
VAMP5	274.6079	150.0874	108.1151	218.1322	220.4042	166.0167	209.4211	156.3457	256.1299
CARHSP1	29902.49	30069.54	31486.5	31782.91	30275.57	29783.71	29774.91	28992.47	32043.38
SSBP1	170.067	670.1946	1062.055	558.8448	948.3329	549.1002	657.3491	1197.115	301.576
HSD17B14	45.14292	46.10525	44.60914	44.8173	31.61366	38.61356	50.16481	36.77277	41.89683
NCKAP5	61.31689	35.03632	38.75397	37.39411	37.15828	104.5699	46.89451	38.55848	39.47921
CCDC144N	262.9431	108.3025	90.95408	134.1126	106.6129	118.9035	76.59026	79.66571	322.0874
COX8A	254.3794	122.1361	246.4718	109.2988	403.4671	65.1028	428.5448	767.3077	84.14438
COPG2	1230.131	39.99037	197.2523	58.0204	301.7469	377.3937	142.3544	171.4749	62.39318
OR10A4	52.14677	47.43462	33.34124	48.07135	45.78598	48.46696	38.47964	53.4538	50.42815
NACC2	600.8329	380.3027	993.8992	813.164	296.7015	2134.989	1478.521	1298.488	1679.479
TCEAL1	76.5714	53.83129	117.9721	56.59071	49.85698	48.91042	41.21233	41.93377	41.2398
ZSCAN16	76.44649	69.16402	582.1128	84.04668	542.4634	333.6743	501.899	251.3738	66.96409
VPS13C	42.02724	1352.753	472.5529	82.47259	368.5099	747.6941	281.8512	405.4838	67.73929
CREBBP	828.7098	904.6801	271.1241	623.4804	570.5507	115.6278	258.1061	85.38573	1225.601
GINS2	4249.375	4271.656	7316.933	5697.822	5952.772	7092.118	7277.913	7175.541	6323.703
WFIKKN1	65.67528	38.73673	33.72998	36.08599	35.08768	44.55245	89.46365	43.46518	45.12
PPIF	10818.35	5937.423	3538.385	6413.653	3703.884	3051.936	1942.934	3191.841	11651.96
C7orf45	116.4004	62.29478	50.51542	80.40794	57.95813	53.63905	49.0835	63.45349	47.33261
NGEF	510.522	1613.391	1222.008	639.2467	6895.119	3509.454	1723.291	1551.446	41.55039
TMEM26	139.7751	91.57839	46.96909	52.86753	107.6496	58.12359	83.7069	208.7601	168.945
LOC440145	92.09223	328.2049	151.5337	380.5171	180.6715	397.0513	219.5928	279.0571	61.45856
CAMK2G	318.6864	1223.662	2725.153	1915.741	2541.955	2639.988	2693.922	2713.179	1649.448

KCTD21	69.66122	58.82059	56.29619	48.00985	323.431	524.2518	130.7855	171.0475	52.93596
KLHL1	85.86009	57.42021	59.75019	51.10596	52.3125	51.32101	47.74496	58.73629	171.9704
PGF	53.96601	2498.104	1605.784	1883.114	2157.809	701.1277	2803.526	3029.6	2702.006
FLJ23834	37.37917	43.07353	35.82076	33.90473	40.15657	38.52839	37.99141	40.53016	37.8863
TNNC1	71.08712	559.1229	1485.745	990.7206	2367.249	2157.115	6798.754	4882.514	3414.523
RCN3	483.8048	997.7141	902.7954	1313.69	209.6226	1010.88	945.8798	1117.817	1340.69
ZRANB3	293.7847	141.1571	94.82194	178.1661	107.6689	148.7938	78.3953	113.7977	249.1003
PCGF2	152.4891	2179.505	905.6931	59.38599	2478.131	1087.137	1511.725	1493.673	49.40214
CCDC134	591.9713	249.9495	48.01408	88.18057	92.85522	69.03781	72.48633	56.83584	169.633
CCDC117	55.71254	68.70802	47.75829	37.87256	52.7021	397.8606	42.57872	66.4557	47.39948
IKBKAP	36.76749	34.98553	36.5873	46.50272	256.4344	43.9673	112.8667	32.91514	40.96149
TMEM117	454.4861	324.4615	195.2657	389.383	389.439	257.4365	328.5775	566.9668	245.6694
LYPD4	251.7729	153.0822	80.98912	167.4037	96.50256	98.70495	69.57178	74.09174	324.5386
NDRG1	7999.402	8994.565	9879.13	15301.54	8752.098	9601.393	7542.029	9521.361	6221.919
TRMT12	3225.553	3787.26	4055.199	3369.96	3982.921	4194.675	3534.66	7223.542	128.589
TMEM155	157.7971	111.7772	91.82562	216.9759	79.96788	89.24419	76.93603	77.18994	422.5193
AADAT	40.77942	38.20859	37.05053	43.56022	37.23674	39.57896	34.95842	37.69661	42.52705
RSAD1	33.75996	36.16567	277.7632	503.406	647.4787	151.9775	827.2265	484.0668	33.0562
OR10J1	310.2093	197.542	104.1775	164.456	72.90182	146.9437	76.93675	90.36642	493.7894
RDH16	35.03085	685.976	709.3414	37.29348	926.908	1078.799	1709.617	1468.193	69.11001
OR10G4	34.71384	38.57912	46.62779	55.30351	46.66721	44.48323	41.65953	62.09492	57.33997
SLC39A2	66.00816	41.88654	54.01032	50.04752	46.15409	39.82687	357.1227	431.9127	49.25627
RUSC2	616.5863	285.2859	249.8859	110.1772	492.382	458.7129	454.1582	260.1679	113.1295
NLRP1	1096.075	613.0758	398.5683	323.2475	118.1766	356.5412	199.412	415.8581	1491.369
SLC16A7	53.88058	64.74764	79.34317	53.93498	43.81499	52.47069	42.38969	90.34893	64.17757
CNN1	4067.114	64.41363	35.10291	56.5886	298.1262	38.29926	360.6943	42.82887	57.08316
ITFG3	51.10126	57.70311	403.5133	41.80034	1061.131	65.69683	246.5878	1028.915	35.70495
ABCF3	1341.476	40.99479	196.4892	40.82075	270.3668	52.67043	359.4055	227.7522	53.49715
TMEM179	39.38924	61.55155	58.26971	58.32618	50.54977	60.98909	51.45761	64.89134	200.4808
TSPAN2	43.33173	33.18811	35.1913	36.14318	34.82337	36.18086	74.46828	34.88519	37.37355
PBX3	121.8955	370.0833	459.066	396.0189	585.9949	533.4149	531.011	528.6428	716.71
C17orf74	414.5886	218.3655	127.3132	251.855	114.5634	189.7144	112.3257	164.2086	436.6609
INPP5B	83.38184	107.3073	185.9917	585.9027	290.5807	70.23274	125.5949	49.93923	1478.126
FAM123A	59.73465	106.4263	50.86677	52.85394	49.38385	39.64269	47.77411	42.11246	145.129
ZNF524	171.9247	780.9831	1075.444	868.7527	912.5457	1085.811	811.5065	2047.076	276.5071
FLJ90650	3290.853	4802.812	1923.56	2218.52	2917.94	1561.993	3693.113	5310.174	6067.549
SFRP4	4286.848	57.38602	875.2062	85.14655	49.04385	1011.233	151.1599	887.8305	4008.624
MAP3K7	49.64138	1415.996	2548.546	1605.376	2332.588	2793.082	2672.132	1280.591	3925.699
PLA2G4C	1732.335	5779.749	6540.262	1586.765	6196.834	4124.275	6738.14	9684.042	3605.642
ASRGL1	33.96058	34.59209	30.19867	33.93677	35.56763	35.37352	43.83192	47.49931	48.33567
DACH2	140.0616	80.4289	46.4146	78.71255	51.75879	32.88911	50.11674	36.80713	156.7308
JUND	33400.28	31367.91	29891.86	32250.83	32444.62	32560.69	30720.9	28901.91	35219.55
TTF2	36.22355	36.47715	831.0673	43.74655	2009.173	1480.136	1636.989	742.9888	57.06197
ATP6V1E2	71.58858	2112.832	54.72754	847.689	491.33	867.4188	579.1741	595.1011	89.08078
DNHD1	333.968	129.1888	686.9287	112.4141	1043.523	517.8339	792.0896	784.0226	337.7633
HOXB2	34.50375	58.06311	164.6563	41.99162	134.6676	69.87376	140.3725	48.27905	46.24603
SLC7A7	376.3505	334.2975	230.8997	357.467	292.5917	124.4049	151.2138	206.5028	573.3906

FER1L3	1033.509	1110.066	1221.203	2058.652	1582.442	1956.46	1827.036	868.949	1289.976
TTTY7	128.9221	71.58462	46.14401	40.34279	38.59734	67.92516	50.98655	49.35065	53.93616
LELP1	109.5769	56.94335	43.38267	42.16664	50.56578	37.14713	47.51436	50.44594	43.47353
SGCB	364.5461	214.0005	122.8462	229.6942	124.4694	174.4643	94.98352	126.4429	381.2595
COMMD7	45.12722	38.90541	2243.062	77.37602	2715.925	1950.064	3458.354	2812.605	33.02512
TMEM156	41.20787	33.99418	132.7421	61.28398	146.3109	46.28305	35.76258	43.88936	44.44469
C11orf87	90.51076	62.44589	64.4921	71.73312	126.3807	2033.244	47.7523	359.8221	38.65671
ATR	39.4672	36.01867	228.1712	53.78894	181.335	389.3296	94.10249	129.9886	49.1479
ZNHIT2	47.92521	34.97706	63.81723	248.2334	47.30075	37.15103	52.12365	68.53801	678.6546
DYSF	259.4469	972.1438	101.7443	229.9557	234.1687	159.3537	263.7295	356.6708	279.2153
RPP40	13101.85	11082.72	11891.44	12344.3	10117.83	10988.33	9012.491	11379.03	11202.61
HRH4	261.437	188.8076	85.64272	167.7784	61.25227	71.061	63.00293	88.19069	452.6908
DNAJC27	753.8475	34.92379	640.1156	50.60595	563.5051	451.6781	537.8118	200.9685	39.72609
PPP2R1A	98.67094	1518.354	1967.128	513.6845	1676.206	534.1859	1751.533	2871.712	3119.86
RAB34	245.5462	129.4474	457.4577	176.7134	1510.017	133.4364	1078.624	424.1595	168.0222
NFYC	588.0363	403.7546	829.1157	481.4446	1076.016	304.2472	1205.242	1140.89	785.9476
SLC25A44	851.0928	1451.942	2179.647	1414.807	1895.145	1821.089	2111.285	3003.361	1671.872
DRG2	79.24046	694.0853	1353.043	61.2098	2220.633	892.0723	1963.156	3111.934	41.36636
SDK1	1515.636	842.1918	259.2839	69.33958	46.07964	56.79566	125.0507	497.5543	40.66382
BAIAP3	42.53997	64.45559	43.50366	40.89261	49.04042	39.19656	49.96021	60.73807	54.87224
RHOQ	9700.178	9163.699	9952.25	8428.286	9638.42	8245.077	9234.155	9429.921	10960.75
P8	453.9939	965.4402	739.6269	799.4744	693.3317	422.5474	301.7106	344.8638	1134.95
IL1R1	442.7131	284.0982	138.7287	262.6017	95.74326	132.6006	73.52056	120.7433	631.4664
TCEB3C	37.30429	47.55056	43.65337	33.14591	35.78863	50.57979	38.6864	32.51215	44.26985
PUS10	55.09914	43.73733	102.9952	45.5034	135.9369	47.92891	39.41593	42.23222	46.09543
LRRC7	38.36055	37.59474	37.23666	41.92008	39.77877	54.31885	38.16569	37.74129	58.67494
ELMOD1	173.0391	78.99528	55.58517	67.90517	48.52344	60.39864	48.98034	56.77914	88.5858
PNPT1	106.1396	64.45005	200.304	39.67103	47.17446	35.33306	212.9807	282.4546	44.35109
KIAA0367	50.3762	66.57967	329.4422	46.9687	284.1716	381.4596	704.8798	522.7719	77.57582
REXO1L1	33.81678	36.11302	42.48549	49.25633	40.70216	33.92331	37.6288	36.55324	40.52937
F9	194.7875	192.1922	84.87288	153.6622	92.93273	95.12197	77.43335	95.25233	199.3876
LMBRD2	149.2227	115.1633	78.44995	113.3777	76.5688	124.3194	238.1943	67.77937	57.26964
TINAG	171.2277	123.1881	96.1527	163.44	96.44899	129.8204	112.9197	117.3309	357.2582
ADCY2	100.8742	99.2023	57.29053	175.4305	62.23349	70.13176	56.89864	65.03201	195.1358
ARHGEF12	5010.12	4115.923	3154.414	4545.892	2721.086	3328.55	2005.75	3412.811	4466.26
GFRA3	39.40083	37.03096	40.87882	46.0368	38.62703	49.20926	47.13195	37.29915	42.48437
DCUN1D1	43.76394	34.99733	49.49583	38.63498	113.63	49.93861	88.45786	40.84154	39.06479
FAM129A	230.6332	1180.755	2336.648	695.2554	2904.445	478.1358	2553.392	1208.734	120.5661
SLC25A45	213.4222	1008.457	681.1479	171.8875	740.7407	1159.35	917.7046	1659.746	1577.293
UBL3	113.6104	71.01276	55.96985	39.01209	195.2122	281.097	249.241	256.1393	65.10587
C4BPA	43.32854	39.71873	41.9453	45.63939	40.5097	37.35011	50.34354	36.91058	60.03878
C2orf25	92.4579	85.65021	411.7096	948.4939	496.9269	636.6995	473.2353	489.0639	1283.484
SNORD42B	84.14887	77.34967	62.77027	171.9841	48.00061	56.44123	45.05386	73.00623	106.4148
RNF11	5803.23	6224.517	4482.541	4359.17	5651.882	4452.684	3400.503	4243.267	4290.188
WASF3	56.53839	44.07109	48.18571	43.36596	39.27614	397.9433	176.4643	41.08246	53.20084
HEATR5B	173.8199	114.8134	96.54024	260.9216	97.81029	156.563	56.3551	113.4392	585.9749
MAK10	329.5895	203.169	133.5454	211.1665	81.94647	92.29736	97.3076	80.05399	921.3428

ZNF343	204.4913	87.75848	71.25687	160.2433	207.9246	121.4011	187.4856	201.0317	146.5079
SPRR3	23651.04	10246	26043.34	8528.584	25245.04	17777.44	13846.67	15183.76	23879.48
MTDH	422.0209	268.724	594.9283	447.4085	784.8542	489.9097	813.2587	456.7076	567.9361
RARRES2	562.9731	986.6084	2403.078	450.8095	4065.078	579.5048	1989.45	3287.245	827.4256
LAMC1	424.6713	331.0317	263.92	565.0579	457.7224	506.8877	474.0792	355.4269	702.6808
C15orf58	2731.347	1439.967	533.7304	983.2601	523.5281	1225.266	618.5488	701.3723	2441.942
HDAC5	54.74655	86.35576	250.285	160.098	203.701	117.2054	54.68546	89.94439	402.3054
S100A5	132.9136	97.47923	54.95142	87.94827	61.78992	69.79967	50.31839	49.34228	169.9143
ASB16	79.67769	60.60403	155.4174	57.64791	38.562	46.16589	91.28564	113.9642	55.42798
ZNF629	589.2747	303.917	146.3716	74.18102	140.9245	192.985	135.7695	142.097	80.3192
RNASEH2A	63.55633	655.0032	945.0435	1179.586	879.4641	1259.718	1247.522	694.7084	195.6846
FAAH	226.9418	155.3487	62.14272	210.2932	97.56715	87.48578	75.71732	91.45478	195.5574
PCNT	3785.371	5263.521	10156.78	5332.748	10177.26	9402.87	12748.38	14333.77	1299.544
MGC45491	127.951	91.23939	85.04206	75.13329	70.73168	127.5586	73.33479	97.06805	73.28283
MPZ	143.9406	111.8572	67.7879	80.93758	75.102	126.851	71.25269	50.52467	210.5765
CYP3A5	10058.25	7192.51	9263.163	6599.038	7629.848	9330.584	3948.879	3327.245	12427.3
PTPRK	36.11726	431.9117	261.4061	498.3378	253.0755	197.933	691.0723	181.3494	35.04596
MUCL1	105.2913	170.4904	1070.013	951.1061	782.957	4843.247	1107.032	1023.273	90.5937
CAD	563.2424	345.4036	222.5053	340.2015	185.7005	255.8668	259.0677	262.159	790.8818
COL24A1	268.007	128.5369	86.40927	149.9927	84.87408	119.8644	70.44482	112.1377	214.738
E2F8	2459.091	501.5652	163.3715	48.88344	43.27282	284.476	139.2939	758.0751	1145.319
PGR	53.04343	43.91211	49.53968	90.45808	99.31609	53.37973	54.59327	39.59597	57.63262
HMX1	30.84638	63.49417	44.66089	37.42363	49.41923	69.39303	42.16719	58.64576	48.61906
TLK1	8296.905	8681.076	5795.215	8741.405	5871.05	7429.964	6650.935	1828.944	8439.23
GPR151	48.75665	61.03131	37.8159	41.92197	49.72753	40.49263	43.38737	45.82689	102.0485
GPR107	84.93187	82.23497	45.03928	50.95051	147.7047	39.07416	334.7491	55.7056	107.3971
POU2AF1	105.4851	1271.201	76.5132	103.3618	1376.055	58.4811	554.6772	54.32933	93.01631
CCDC88A	988.7249	2249.304	2371.136	1696.31	2074.911	2905.435	2215.263	1972.698	1435.017
CABYR	47.05043	37.9219	39.86265	46.82839	50.05023	39.2097	38.62782	38.59642	155.6391
ZNF645	52.60116	47.57298	55.84034	38.68834	53.86461	46.35155	38.35395	46.19444	38.60146
PCDHGB5	36.47325	35.91325	170.0224	33.1181	52.78313	274.0192	195.913	232.0525	100.4264
TDRD9	101.3243	47.29234	58.55769	50.68922	46.42289	49.11917	120.9067	280.9046	34.11747
SDS	1652.753	1170.113	1615.813	1785.417	208.9654	1798.82	1356.576	831.6946	1454.229
CXorf39	78.98103	66.93171	48.54173	55.50512	219.7102	185.6141	136.4369	126.9547	733.4151
BMP2K	392.9892	212.771	173.2537	242.0591	115.4385	196.2115	121.036	147.7431	132.6853
CCDC27	178.4636	151.6636	46.22972	90.49876	71.27998	88.68832	58.39712	73.71886	221.912
GPR132	57.66467	1045.573	51.75817	1250.123	47.29822	38.04065	138.1422	57.41059	65.36584
SCAPER	260.4926	2249.285	2433.228	1918.473	1923.641	1644.921	1573.877	2812.137	335.3491
ZNF211	48.50633	53.26203	556.4749	40.08071	773.9455	578.636	770.1719	46.24922	39.31788
SLC5A8	3348.773	4425.428	2963.563	2000.172	1598.721	1527.171	1612.969	2424.44	3917.22
ROS1	38.70648	88.84347	36.59229	56.22178	48.6981	62.99754	100.6361	52.38415	39.61908
ALPK2	4027.921	7422.691	4832.729	7280.687	1563.128	5772.61	3428.756	6126.765	5985.763
ANKFN1	49.82529	43.70177	51.47908	45.74894	36.30763	35.37178	38.22596	43.57133	39.615
RELB	98.55247	163.5733	207.9299	341.997	240.8696	78.47403	86.84364	171.5811	121.7523
KRIT1	83.54355	100.2708	140.5492	49.91977	159.6416	57.10549	106.927	107.5924	51.30134
C15orf57	56.54732	1318.565	2161.608	1234.91	1380.917	1962.951	2063.548	2086.967	1644.235
TRIM13	1653.094	610.8031	461.9462	835.6899	204.9047	627.665	111.5598	350.493	1022.649

RNF186	158.2497	107.8678	58.29548	71.82439	60.67319	78.42226	55.66089	40.15143	69.88407
INPP5E	82.84693	103.2731	1374.996	46.48658	1274.592	699.5615	1146.581	46.11709	97.41576
SLIT3	46.84765	50.12887	185.0931	47.68719	552.4757	47.34063	355.5397	357.0784	35.41569
PANX3	182.0709	94.6676	68.87651	83.63192	57.46463	63.19841	54.88036	78.6526	112.9335
FAM134B	3150.812	283.9036	476.4154	34.40343	208.7189	547.3641	633.1605	674.1997	60.70672
LDB1	161.9038	145.3623	94.25017	168.6513	85.88637	108.1162	69.2842	105.7555	127.4604
LOC65374(	259.7702	72.65195	82.41644	114.7884	61.87405	88.19208	46.16602	76.64365	139.4412
HSD11B1	83.88313	82.72446	56.49168	85.7914	55.40243	84.69422	49.41866	64.56011	85.90332
FAM20B	560.8409	231.0313	362.5604	226.4577	501.7986	279.8394	400.7743	497.3441	315.7754
GTPBP1	69.67638	328.1046	45.01926	63.22396	40.55113	52.54955	49.56893	53.39648	101.1704
KCNA10	59.08013	52.82153	41.15077	36.7862	33.12314	42.49853	36.13629	37.07544	70.27629
TFF3	6858.128	6863.779	2857.899	4422.381	2361.977	3548.513	2140.427	2852.809	7574.634
HMHA1	367.3947	215.0528	252.2401	227.9831	248.7383	166.2593	184.1918	286.0765	410.1362
RHAG	69.71545	41.73731	34.16227	50.31639	40.68616	51.17787	44.61567	33.93983	51.16666
C11orf9	36.84777	32.61301	109.3545	40.57508	226.9629	37.62858	33.39808	38.58737	40.15701
AVP	38.87377	68.62959	47.91596	64.31709	75.76855	105.7986	57.52573	54.10319	282.7039
FAM151B	73.23872	78.40801	51.18708	54.07643	59.83114	72.05393	60.25581	101.1987	67.444
IRX3	15415.78	19594.99	11063.71	13823.21	15914.9	18958.96	10590.6	13180.57	16404.75
PLEK2	127.2209	341.6995	301.6579	446.5031	163.5695	633.631	277.9084	178.3265	213.4291
SPINK6	60.71416	348.7625	36.82424	36.16228	102.8748	56.07139	35.53475	35.31328	92.02135
ADPRHL2	619.5542	552.2996	417.5988	555.5371	435.8174	251.0743	383.1263	317.6362	852.5999
OXSR1	39.93969	40.60915	620.3189	56.15726	1087.099	37.53939	520.9518	230.2916	37.31139
LBH	84.69561	520.8585	48.93811	49.04755	61.13482	49.54267	143.6956	200.7444	40.04688
DLG3	37.03258	2303.153	552.0004	291.4492	538.6261	432.112	181.1061	441.7119	35.63152
DERA	1939.726	850.3192	5791.047	2145.336	7317.094	3273.74	7277.305	4792.788	88.23034
SF4	4410.636	5131.601	6547.667	4179.03	7013.025	5606.455	6322.372	7573.038	4222.021
TAS2R46	112.6769	51.3044	48.77864	39.77905	45.64941	74.76972	39.94979	46.54033	66.12177
MAPK8IP3	1319.151	3596.14	3456.123	1674.704	3412.677	3474.449	3080.966	3493.358	1753.209
HOMER3	180.0277	67.32977	261.6838	161.7787	87.44247	98.68469	258.4503	142.4174	243.9411
ARHGAP21	6153.846	5372.44	3330.889	6636.47	3188.922	4805.829	2898.052	3772.357	5941.674
NIPAL1	826.0615	395.1943	553.7291	452.3543	663.7357	645.0163	280.1727	178.9753	61.71392
PCYT1B	80.04252	60.2722	50.06697	126.1831	111.1793	53.57783	81.12637	58.69967	43.6406
ADH1B	3661.417	2188.614	693.4971	554.316	542.2864	294.4273	114.8185	1212.43	1520.66
OR52B6	181.4052	123.9243	64.95037	79.30378	65.64571	77.16961	62.0522	75.60772	128.3489
FLJ14107	49.87354	312.8764	168.7255	38.18455	39.26086	36.07358	37.41465	38.88963	81.70296
COPA	1230.866	1043.853	1536.691	508.7053	3012.157	1789.185	2425.331	1616.595	1125.87
EEPD1	45.91779	46.95098	42.28881	54.84324	388.0491	49.57988	239.8115	43.30745	37.89746
FMO5	236.8412	153.8944	54.1504	112.0008	187.8776	69.28957	57.09789	182.9182	1352.091
IFT57	7432.655	3906.884	1547.881	1101.752	3203.292	4517.1	1901.95	1810.656	4243.616
DCAKD	14608.19	13341.89	13230.82	14151.64	11757.1	13565.2	10638.69	13279.54	16389.69
DUS2L	2184.849	1912.45	1034.639	1842.137	857.8449	1846.341	1233.184	585.7705	2443.408
MTNR1B	287.9006	65.55201	69.1305	107.9958	64.41852	112.4682	62.3606	95.48777	194.4919
CYLN2	111.6125	56.72921	46.77827	64.65109	115.7646	189.5073	93.98525	63.45222	134.4286
FTSJ3	368.1006	172.4845	105.3125	258.5063	119.3017	138.6049	112.5776	121.998	353.5478
FDXR	117.8945	78.36516	54.77296	59.88539	83.57417	85.74707	69.09106	172.7871	67.14319
MYO7A	126.6984	103.804	343.5121	109.0352	252.0342	91.11929	314.8374	776.4755	130.5779
CBLC	47.08708	43.13019	730.0425	813.9847	956.2955	656.6291	801.7572	1411.941	68.81267

C8orf22	35.53435	46.12455	95.48183	46.41315	40.31596	165.7189	54.32706	163.4712	58.86948
ENTHD1	75.29471	119.9737	49.31038	86.30685	51.2916	48.78596	49.51665	49.34683	41.57122
NDUFB10	3796.438	3866.963	4804.806	4147.019	5956.918	4382.192	5500.395	6494.329	2684.945
ZNF192	55.17264	290.1532	164.992	322.091	49.43307	52.97973	60.4305	122.8453	47.9309
CNP	45.93939	40.02495	52.46802	43.27736	124.9908	43.32978	40.39818	37.15139	46.96769
INHBE	419.0448	155.3217	88.38968	211.3553	91.02719	132.9999	190.5851	291.2782	101.4939
CCDC124	296.1524	183.1719	162.1469	113.3828	245.8604	134.4545	120.5572	135.7254	310.5942
CCDC102B	51.1134	58.68607	210.4615	60.89497	249.4395	45.6991	191.6469	148.9942	35.29063
RNF139	50.45889	77.99915	53.97602	123.7727	60.14858	61.33944	37.37913	53.59542	174.9442
FOXS1	58.6725	283.1967	361.4996	43.6105	331.0261	249.3195	743.425	415.7591	48.2896
TAS2R10	247.2053	185.2484	92.29315	205.4544	101.2768	147.5484	95.97833	74.34449	407.369
SRRM4	37.5079	35.78814	34.38367	37.42251	43.4241	41.81707	34.97812	44.17172	43.35377
MED25	41.33272	934.9471	1608.339	1919.234	2543.041	1496.656	2003.582	1729.339	41.75119
SLC12A5	111.4775	74.1044	48.08357	93.6311	62.91824	65.69774	57.95502	56.5776	50.58147
RAB39B	45.65179	40.63112	38.96732	42.12792	40.38752	34.45851	57.50901	52.83774	64.84969
CSRP1	2513.196	2189.91	1451.943	2393.334	1523.424	1296.628	978.564	1727.744	2955.244
OR2W3	418.8853	217.9953	107.5065	243.6686	108.0496	177.1673	104.7085	120.4638	557.7227
PRMT5	208.9383	365.9436	877.6649	399.8965	1160.939	781.1055	1023.782	1404.189	965.5931
CAMSAP1	36.03843	259.8442	276.3362	247.1511	344.7736	355.0154	400.798	124.813	43.83114
MYCBP2	43.43285	833.4893	2246.398	649.2668	3060.535	2183.823	2186.64	1575.232	67.94776
FANCM	182.3287	43.269	48.66088	67.94963	46.24202	50.04262	48.83635	91.21114	71.22658
ZNF364	7977.071	7001.6	7811.446	7561.56	5358.686	8584.124	6382.516	7197.051	7501.442
PALM	14355.97	13755.98	12608.61	9655.302	14468.27	11734.28	10405.84	13851.27	11917.45
RER1	519.114	77.64104	89.23546	67.15804	103.8551	86.71503	55.84212	92.64413	149.0343
ABCB1	133.8432	150.4175	59.29884	141.3098	267.8582	375.8031	117.096	210.6894	114.4889
ATP6V1B1	3357.838	3205.87	1686.241	8786.008	5928.172	8468.945	8136.424	3200.515	4230.263
PARP16	76.77779	46.74716	193.2766	44.77029	521.4421	364.7489	309.1053	318.9034	80.8
FAM20A	157.0544	452.3018	429.8485	112.634	365.5475	77.36974	587.6165	42.79111	81.73223
UTS2D	41.26986	48.33491	38.53686	47.78646	48.4115	65.2852	48.28098	59.04651	49.01842
CADM4	1457.131	363.5542	1355.384	1186.677	1887.822	1140.517	701.0278	1959.293	1173.393
ADAM21	142.9265	112.4884	74.52834	130.0163	65.90957	81.41118	65.98304	80.139	179.2692
ZNF570	75.05363	448.1136	287.9901	85.38466	183.1377	51.48555	245.6671	144.6059	166.1939
C11orf30	133.5604	72.51783	83.24197	120.9865	89.03459	68.02023	62.44367	69.04671	175.8533
SERAC1	274.2028	88.40786	226.4957	178.6656	394.5984	84.56825	66.00025	58.36376	172.517
C12orf57	49.50229	509.5262	118.4946	402.9448	179.1495	40.48367	40.69349	102.8139	35.33207
LOC730412	44.63101	36.4843	43.44339	57.34212	53.29389	48.5617	38.89675	57.22911	55.00734
ICAM1	171.8552	176.738	88.45479	92.50322	81.90865	107.1755	76.02312	90.65228	98.98209
C15orf54	184.1655	161.3616	86.1005	127.5769	80.54945	109.0074	52.01062	64.77174	166.4265
KIF5A	45.71696	59.03262	62.7787	51.88848	58.5153	50.63927	53.228	60.14557	54.54221
GANC	39.46467	326.4163	115.0575	41.1856	37.34116	49.3271	37.50298	37.72355	53.44243
DDR1	300.8392	141.8072	325.547	384.1513	473.1353	135.2818	614.5578	281.8908	188.2122
EHMT1	68.4264	65.40714	200.0502	43.20786	151.1258	40.74207	96.88982	40.89835	55.25281
ARG1	1368.869	4206.452	738.7059	2908.761	7872.046	1350.264	827.283	1835.649	2512.972
CYP26B1	156.6403	73.70274	71.41292	84.96536	451.4053	75.99796	427.3528	52.16959	304.1824
FAM82A1	58.51239	44.78906	182.1934	38.48423	95.44153	321.1547	184.2384	41.52814	141.8457
NDUFS4	92.91576	234.5272	43.84223	282.319	95.1758	60.76091	123.2226	63.59483	35.11524
TBL1Y	159.5964	143.7051	86.64825	191.661	88.83978	124.6898	84.09977	95.72064	172.4475

CSPP1	71.4726	90.69037	70.09251	119.6404	71.55613	78.61021	53.51503	61.78173	145.4552
TMSB10	83.30959	1069.112	1331.709	1423.479	1340.628	1079.377	2574.245	1944.822	1952.158
BCAP31	4280.783	4990.94	6843.597	5440.431	8197.818	4961.168	8173.035	8323.086	2062.861
TIRAP	138.6962	67.88505	108.207	71.10452	101.7025	87.85162	56.31454	129.0926	52.40848
HP	87.8961	106.4185	75.99754	140.4767	63.91192	75.39749	70.07008	181.1174	192.7406
C17orf63	94.15696	80.85239	504.3635	219.0021	324.2454	301.8955	340.3322	48.89176	101.4813
PRPSAP2	415.7699	233.9313	168.6055	251.6607	154.4917	259.5493	132.5115	272.8818	494.3842
S1PR2	52.27432	90.6108	131.8294	795.6398	40.57534	31.74826	182.114	45.81272	43.62529
WISP2	156.5516	88.35451	221.1216	106.0214	60.67443	98.55251	702.1703	296.8624	2639.814
MLLT3	237.9215	167.7355	82.49957	175.2843	74.77335	131.6716	66.51003	51.10814	457.3206
DOC2A	40.22153	40.98242	59.28669	39.71455	52.44195	85.32796	37.89062	58.34124	66.48649
CCDC21	1430.421	50.09378	276.6968	70.87399	375.2247	873.398	459.0171	397.1808	194.4805
PHKA1	190.3742	100.6866	139.412	87.58315	239.1406	112.6908	83.421	68.37396	295.8314
FGF8	47.53785	44.42219	44.72181	45.0615	57.07479	59.11904	38.38259	61.97937	48.07027
TGFB2	79.46363	40.09458	37.69418	54.52773	49.18943	41.90936	82.32541	40.97421	48.4819
DMC1	34.42596	290.6832	34.16776	32.30331	45.02655	33.22565	51.25731	46.09192	33.69834
FLJ31568	59.56479	35.38791	33.14735	52.87247	36.25278	36.77031	32.55282	42.9411	45.99582
PHCA	1106.745	1632.708	1851.198	2759.928	1744.907	1236.767	1734.945	1589.729	44.85832
SLC7A6	1017.047	1468.84	1817.006	1707.836	1616.66	1863.313	1885.239	1362.876	767.695
ICA1	69.31661	101.3758	90.42538	90.2489	66.47663	88.58849	53.80917	81.97541	111.1436
RAB35	1051.373	1302.985	1680.015	1715.625	1460.009	1873.349	1667.039	1245.102	944.7643
YDJC	43.75701	45.61404	245.2135	37.89199	332.7412	279.4467	204.0949	166.0462	60.63218
43532	32.45459	35.58826	33.89345	33.4345	35.54576	37.13989	36.86275	42.41951	38.22437
MYO1C	43.92688	730.5858	398.187	1931.366	279.5633	32.36864	439.4044	202.8502	34.41704
ARAP3	7294.487	9519.746	6221.772	7900.111	8366.621	6256.896	6714.977	8714.313	7636.965
NEIL1	307.2226	196.7495	102.0235	187.543	139.5281	127.8969	97.04877	128.1201	365.888
ATP5L	2075.227	901.2922	2209.735	929.3157	3650.334	1126.075	2561.841	2087.682	510.5665
RGR	52.61936	41.85765	49.22622	66.97852	63.00122	45.51521	35.92605	37.25441	119.4103
DNAJC15	11530.79	10306.95	8473.674	13463.81	5445.721	6579.054	6442.039	9661.677	10667.48
HTRA3	128.7843	72.72869	56.72589	132.0709	72.1988	91.6051	56.79305	60.08802	123.4028
TMEM195	142.8417	120.6682	75.69607	102.004	69.13422	76.81194	53.60653	148.3867	277.8152
UPK1B	106.0742	60.83225	44.51165	61.45315	120.9463	51.24029	45.97714	51.61862	196.325
C2orf18	40.47937	961.7573	1217.62	52.3194	1168.121	754.3888	1271.727	1691.05	64.30756
LIPC	37.25084	41.52305	33.55604	36.35746	41.85877	34.74981	33.00903	32.75445	37.06316
C4orf33	1662.827	2271.157	1187.502	1393.6	842.9993	1087.484	964.3006	670.5324	2905.831
SIX1	40.22766	52.15101	49.83718	54.54187	50.84742	35.03859	46.38034	36.9501	35.6733
UBXN1	3986.121	5091.818	9452.211	5994.129	9711.314	8298.529	11331.87	8183.602	3022.886
FBXO28	69.01452	51.03676	268.2013	37.38211	231.5717	215.3209	173.1716	52.53941	55.20303
DSG4	61.41964	83.58179	55.03478	62.72604	54.82675	59.01418	42.41951	55.99924	104.1805
SST	70.54163	68.39877	40.55964	48.8872	63.28122	45.96676	51.96264	278.5332	84.15199
C8orf34	173.3488	85.94969	148.8085	93.65752	61.235	90.36178	83.13934	77.36323	57.09451
OR1L3	33.17208	40.40934	36.65842	40.84599	49.71013	58.61864	32.28948	33.84305	57.48169
ZSWIM1	10068.31	8475.967	8028.493	9692.597	5704.446	8917.258	8376.807	7563.591	9717.972
FOXK1	295.7504	56.86946	83.65331	36.06856	76.50211	78.15865	65.36015	83.83344	178.275
ZNF132	200.6198	180.1488	89.59895	111.1078	72.37566	96.8702	65.69138	107.743	405.9691
CYYR1	78.02296	428.1231	272.9454	84.18578	284.6099	79.41411	530.2242	187.2366	273.521
ANTXR2	804.2002	824.4621	553.5481	584.3216	385.7919	288.1623	423.3036	244.203	80.36954

TRMT1	6052.459	4744.235	3626.489	4802.804	3423.466	4194.32	3427.611	4801.048	7231.161
GPR88	46.38349	58.08068	50.59819	37.15247	108.3842	44.4497	39.57338	49.29002	105.5179
SOX18	2399.279	525.4983	3197.645	1784.215	5422.235	4533.285	5745.065	3054.568	112.441
RWDD1	58.0738	42.02337	52.7489	43.23975	163.5497	62.11377	105.7705	42.21905	92.3045
RRP1B	56.12416	54.39551	140.599	70.0401	69.72613	54.87888	76.93808	51.59417	67.88419
DDX54	1385.026	570.4793	818.234	616.4987	1214.914	398.5471	1676.445	841.7289	2231.807
OR2H2	267.6023	148.3746	90.17532	142.08	87.9407	85.86988	72.64332	110.3493	312.5372
PXT1	100.6323	46.71505	54.22828	80.92786	33.93856	42.75033	38.38342	36.56927	31.92996
VTA1	54.14241	56.22358	43.50968	36.24378	111.6225	180.9225	150.8674	102.3057	46.34567
DNAJB4	44.52557	47.80377	40.76952	183.5613	81.3026	43.89143	52.89432	40.76559	50.39047
CBX5	2263.087	1972.323	1337.014	2312.533	1007.571	1061.569	939.9355	1157.201	3477.446
BHMT2	1911.122	524.0344	43.71461	502.7352	145.9844	46.66318	197.2117	70.00648	38.51995
ASCL4	53.97768	37.21754	36.98806	41.9071	32.95169	36.44464	40.80299	32.50898	36.17785
ARL1	1544.692	1076.885	1127.225	1978.422	1304.098	48.75376	1142.164	614.7933	57.5523
KIAA1244	217.7259	114.5953	60.43188	72.43584	71.13374	94.00861	56.22375	52.65019	77.93743
GALNTL2	46.64129	45.484	39.84877	659.9837	38.79293	46.02568	47.9266	194.2742	62.90554
ITGA9	2492.981	1465.772	1409.663	2133.705	1213.92	2390.393	1751.781	1621.268	947.3585
MAP4K3	306.851	206.136	229.8283	616.8461	227.4171	286.1106	216.3677	169.8582	438.937
ANKDD1A	942.2963	1936.915	1384.982	720.8858	1068.348	1289.008	661.64	701.0363	173.8645
LPHN1	65.21024	44.78994	63.06808	49.93995	151.9441	56.45626	143.3105	59.8205	109.0544
SLITRK6	46.04212	45.67191	132.017	382.2333	117.7153	1019.217	408.5359	245.9786	60.55258
CP	3466.64	2295.841	296.9894	43.22749	1306.286	200.7034	692.4778	809.4409	52.175
MRPS9	1206.636	375.0001	840.6329	435.7651	756.6623	578.9078	1015.793	1097.331	1111.05
WSCD2	346.7986	192.6192	109.5183	162.2781	87.46918	142.8094	77.55923	118.0734	406.1319
C6orf136	51.2805	799.4002	2361.429	3144.054	3105.524	3244.448	2277.131	2612.158	118.7717
CRIPT	1188.132	1010.685	672.06	609.7708	814.92	945.387	994.9151	897.6971	627.3091
TIMM17B	15407.76	15909.36	19988.94	14927	16467.28	15701.22	15328.64	19953.72	15171.04
SUPT3H	323.0812	189.4143	103.9866	186.8678	88.93275	124.7899	64.34196	83.92521	380.8275
RDH8	129.1431	82.06058	56.43935	113.3564	63.36717	157.835	52.31603	62.77731	496.3978
C1QTNF2	59.2732	74.21522	74.47218	94.27869	61.51239	93.81754	54.78925	76.91808	163.7866
PDE11A	60.20576	42.29893	91.99017	53.24027	58.16555	70.41964	49.9795	50.61953	36.17334
LAG3	128.2384	673.2614	1460.87	109.4223	327.4701	393.4061	811.1061	985.0923	139.4892
PTGFR	577.7458	156.9191	67.12185	113.9008	102.1345	115.4788	80.71582	68.50391	419.9383
C20orf7	4229.925	6051.725	5685.432	6583.62	4912.07	5418.702	5086.854	5582.51	4596.003
HSD11B2	210.0454	92.85753	148.6421	162.7157	1252.514	245.0363	326.3399	167.2616	242.2547
ANGPTL7	88.76815	47.36015	59.03532	51.95625	141.8322	76.16692	68.85104	73.46413	67.29809
C19orf28	288.0732	201.1745	121.2992	171.2758	134.0328	169.8623	129.1074	137.5245	452.7151
TNFRSF9	593.369	299.6387	483.0177	148.2944	95.69685	449.9693	144.9296	295.3896	218.668
CORO6	46.54904	1030.301	521.6736	55.70882	234.3892	50.33697	771.6164	675.5353	40.79836
MRPS7	3322.924	1725.9	2297.967	1234.444	1909.499	2739.522	2062.575	3535.714	2456.924
OR1C1	42.06943	65.81717	39.60118	66.09922	54.36475	46.74451	46.59705	50.47779	107.935
CBR4	1371.289	1143.456	1534.034	596.7072	926.85	1089.709	770.3267	653.8705	41.19431
NAT15	46.86946	56.27927	262.5269	343.0439	279.086	256.6581	363.0742	306.3886	76.02239
ATP10A	249.784	85.54816	89.99947	179.1526	68.91599	115.9811	71.61889	76.56464	352.9071
RTP1	271.3404	141.2792	74.23408	185.9962	73.36736	98.71241	67.48206	71.76451	283.6422
HERC2	62.95704	987.0832	1135.907	38.69439	1397.096	199.4864	3175.646	701.5147	60.03835
RAF1	38.83947	872.1357	1461.149	46.3249	2832.238	1538.805	1679.718	512.4941	57.17721

SEMA6C	1445.944	1817.214	372.4709	49.50819	661.5367	418.3571	997.2347	1238.25	65.6379
DISP2	124.8396	153.2409	62.58022	188.6968	55.87216	99.66382	55.47742	57.05926	132.4273
CCBE1	40.91096	73.50481	53.68468	421.4393	45.72923	48.33461	42.5557	48.93208	185.8447
AGT	48.38748	52.03588	38.81871	54.66366	39.75169	52.19561	109.5423	284.8303	73.52101
OXCT1	227.4451	71.83526	149.7814	131.8436	86.03838	88.60751	80.93884	63.41601	378.5499
SCIN	42.14808	37.69438	36.90542	39.16007	37.53904	36.48286	39.91931	57.56911	36.86198
IL16	167.8612	194.2023	101.4131	220.1342	91.85331	142.3747	82.09302	106.2485	198.8375
CNR1	178.2271	121.3004	61.93054	148.9111	81.28551	100.7157	57.34099	71.42947	247.601
FEZF1	81.51223	50.63304	39.5351	31.42012	39.93957	43.99738	35.44672	37.02398	41.09484
NDUFB7	2262.879	4149.937	3293.735	2839.902	3166.21	1010.224	3289.522	3421.885	1880.774
TRIM25	191.325	588.3293	510.9032	660.1174	278.4292	294.2013	237.0358	393.244	1080.031
NKAIN4	35.76701	55.05762	44.21305	67.57984	33.13929	34.90368	39.14498	38.6496	57.99498
C9orf167	1972.994	678.2385	250.006	40.02032	204.685	43.03913	274.275	170.8297	1445.805
PHF20	161.4618	192.6841	82.98319	97.59512	66.36001	94.43901	61.50678	55.05246	102.5062
HIRA	519.8617	346.537	202.7668	541.0373	173.0294	304.7771	211.3321	252.5476	778.4487
ANKS1A	54.93941	723.4033	1251.936	761.6143	3245.102	1512.083	2084.488	1242.827	40.98565
SLC25A2	63.44619	53.89869	141.8191	34.87153	110.6051	51.02764	94.15848	447.0072	59.5157
SLC25A1	2200.906	1048.048	160.0352	1135.137	304.4879	108.392	250.9634	880.073	216.8974
MRPL53	2671.723	49.09631	810.093	1354.246	1218.177	48.46909	1235.745	1766.323	39.31791
RAB7L1	4185.875	3292.748	2725.649	4194.32	437.4047	1394.844	1004.604	1304.396	53.99999
GKN2	94.2186	64.80054	51.87623	56.52934	43.74432	55.12074	40.12492	51.28378	102.4746
HTR1A	58.28946	35.74646	38.24553	47.52595	41.54731	35.71242	35.2239	39.04482	34.57628
KIAA1199	490.9583	306.4447	441.0835	231.952	531.8177	354.4351	490.8414	624.7715	529.0341
KCNJ16	217.0275	166.0978	79.85803	128.4612	84.54307	109.9526	67.49871	85.2613	236.7026
PTN	231.6071	92.93268	268.9435	89.77171	192.211	156.155	106.3473	150.1942	353.6197
SLC20A2	2332.162	2273.01	4255.64	5048.357	6427.087	3861.622	5362.584	3096.885	3749.598
RCL1	1844.996	1981.693	332.0979	578.2575	295.6152	604.884	162.1781	188.1157	130.8611
GAK	24154.28	22286.98	21684.66	19211.63	20548.36	23345.42	20492.35	15978.94	23406.06
OR4D6	139.2063	70.01085	51.24888	92.49343	57.06225	79.51423	52.19196	71.27311	256.2843
TMEM169	43.63557	46.12691	232.7297	97.08449	48.07708	44.14557	164.993	45.1921	52.28691
NUFIP1	33.30079	37.76113	42.14286	43.32306	33.92456	50.22946	56.45848	39.77344	45.7966
NARFL	90.14314	897.6671	49.27068	952.2138	607.1687	54.01196	175.2204	232.9892	73.20131
PRMT6	139.9666	116.1843	216.5752	63.91678	167.6737	93.91854	173.4476	301.4314	101.8789
DARS	9145.837	10347.96	12351.49	11384.38	13072.7	10365.56	11382.92	11940.1	9013.658
C9orf9	65.49282	235.6757	168.934	34.4419	498.5809	38.54634	355.3407	137.6579	45.64962
AOF2	1420.739	402.5144	2633.018	2391.736	2954.761	2487.313	4020.283	3501.099	717.6653
P2RX4	192.7291	147.5765	63.00832	126.9148	139.6457	125.4834	61.13039	72.8658	170.0099
GRPR	76.64305	33.69285	35.52034	33.48139	74.06377	33.5164	66.42851	41.04044	78.07423
APOBEC1	205.7125	159.3632	93.42695	144.3398	95.6436	144.8519	74.9972	81.64949	312.5898
OR5B17	40.77814	32.69105	38.02121	37.33831	46.29887	40.15344	47.4063	36.38509	50.72908
DDX43	132.6249	44.6139	50.73206	40.59691	39.77877	32.78901	41.10875	48.76891	58.0003
HEXA	3230.755	1768.563	487.8841	1770.751	393.2809	753.2114	416.344	446.4151	1669.751
NOL3	69.46117	47.92316	185.9444	283.1873	350.3902	231.0243	234.4054	358.3887	33.9103
DNAH3	261.4646	95.21368	173.0921	123.7787	621.5751	513.5413	160.8575	161.854	239.2056
CHCHD4	6310.21	6321.739	8624.075	6760.065	8336.202	8188.84	8403.463	7337.262	4027.493
CASQ1	61.1562	489.1397	71.18718	124	139.4613	182.0818	147.6311	60.65298	185.4513
CEACAM7	1055.64	74.29323	582.2662	538.729	705.1063	302.1733	143.0091	88.64849	1382.547

SLC6A18	42.77057	79.10182	48.80791	88.84052	35.40312	49.54107	46.15392	51.1555	182.4648
ADI1	1110.877	1327.533	1649.8	1160.496	1965.424	962.8663	1783.543	1158.489	1278.362
DPPA5	47.92244	61.8089	53.68255	101.6594	51.43639	63.33878	39.8139	50.92095	64.14571
TEAD3	703.3134	1391.978	710.1385	1028.305	496.1433	1082.719	636.2584	495.8	1000.838
CDH17	41.24136	48.6453	37.56041	46.95061	107.2057	602.8828	248.9625	265.1315	47.36324
NR1H3	15395.66	15056.69	15579.82	12715.24	9753.2	13706.19	12126.04	15951.99	15200.94
CABIN1	2823.92	4590.207	5291.93	2966.427	5325.02	4157.508	5865.922	4521.464	5968.975
PHKA2	5263.513	7904.619	9069.493	8062.449	8356.529	9422.23	7579.416	8303.121	6874.451
ZFPM2	131.2205	323.6616	73.58153	68.46765	56.73893	101.6641	50.18421	38.97033	176.7186
C9orf169	21453.11	17940.97	16178.14	13400.22	13009.32	15330.28	11421.59	14533.69	20413.36
ZFR	2059.419	925.296	980.4578	1332.919	1032.247	843.1642	1059.226	671.8926	2085.371
PECR	41.0207	1131.151	571.3229	35.20105	545.8107	1492.026	408.1389	1241.162	34.31615
MDC1	1154.666	625.8561	2080.864	510.9471	3129.166	1748.707	2752.086	2940.939	222.4585
AGR2	258.852	184.4771	102.5686	200.1805	99.80215	145.5317	110.8499	108.7437	379.2882
PSMB7	708.6968	1494.909	650.8455	1375.592	1082.588	1075.516	1234.827	2038.061	757.6479
SSBP3	42.40492	88.82584	35.84666	103.6768	52.09363	48.2067	49.00087	49.50745	63.65488
SMARCA4	592.5865	506.7409	341.123	565.3779	253.1526	566.7061	329.261	321.1023	1193.753
TCF4	1385.538	1316.424	2104.033	1139.428	2959.46	2219.447	3150.053	1342.9	1296.168
HTR1B	35.12831	39.19862	33.05649	32.3231	38.86534	37.06601	47.16469	40.968	35.69775
PQLC3	93.44291	100.7094	257.3005	77.12183	63.36423	60.62612	218.4902	235.6965	117.569
SCNN1G	69.70242	74.6362	59.33765	60.1134	61.89563	72.24168	127.4605	84.70857	65.52325
MTCP1	4462.13	3399.385	2939.726	4338.671	2433.183	2589.085	2641.011	2755.897	2671.711
TCFL5	117.9609	674.5107	582.4175	516.2214	552.495	783.2529	741.2758	861.9047	2040.174
LY6D	13193.9	13341.17	17370.48	7841.993	20111.88	13164.04	19662.24	26145.98	12472.65
SPC25	148.2153	177.7861	271.3548	231.5033	588.1066	461.7632	325.6903	186.6248	380.3028
ISG20L1	1098.569	1375.391	1138.527	1239.674	965.6015	1473.625	1052.851	1572.381	1163.235
TRPM6	1246.603	957.871	2008.449	128.9931	1251.356	1222.488	952.4344	800.8123	947.6141
SAMD9	3702.288	4082.906	3842.709	4894.003	2864.176	5104.421	4705.725	5053.421	2077.621
CREBL1	337.3587	188.4559	101.0356	247.0713	114.204	150.8569	95.1655	114.759	553.299
PBXIP1	2426.671	1176.5	508.1756	74.07734	464.6964	673.5757	362.2782	700.4997	94.25279
AGTR1	55.21075	55.57952	40.69249	59.25603	52.6335	48.0654	48.9402	96.27953	68.93392
SERPINI1	148.8522	62.61075	226.9611	69.57988	62.8025	65.34496	286.9835	82.27356	137.6693
C21orf62	81.23029	84.09854	54.04543	99.78514	60.46549	75.26524	134.8659	58.60997	93.95934
EPB42	36.55652	32.49051	36.90174	39.12556	35.13888	57.65294	39.06238	39.50679	58.54977
WNT1	34.40668	47.04345	50.5158	43.63588	42.11684	42.34614	49.49244	36.86539	42.48102
RAMP2	542.1284	331.4862	257.2087	434.5389	279.7407	224.3256	380.1164	330.0595	690.6769
TRPS1	466.8859	220.1028	125.9126	240.3424	121.7225	175.494	84.46321	115.3043	562.0144
OR10G7	197.1357	142.2362	86.95161	194.19	87.73014	102.4897	73.28513	94.92114	348.6376
FTSJD1	523.5912	325.1901	545.6005	478.4688	381.3735	684.97	394.2334	396.4145	370.1279
CRISP3	38.23881	48.31982	180.625	35.70495	40.1572	200.2378	89.62774	63.30021	80.18867
LOC728037	71.64847	38.40674	46.67908	48.5485	38.32962	40.35686	45.41465	43.94685	55.92606
DEPDC1B	59.87138	74.38558	46.21595	41.38206	37.34171	53.60779	42.16614	170.2009	49.67311
GALNT2	105.4369	55.51283	57.51718	55.34676	66.67617	94.10626	58.37561	73.17681	69.29466
HCG9	38.64394	32.81901	89.66486	35.62358	115.3883	162.4495	78.29874	38.38963	31.49838
ZNF33A	99.01353	35.8681	84.99932	50.59611	48.64986	67.99373	55.13882	58.01353	93.32394
FLJ20125	512.7656	305.6798	147.0635	755.9993	132.2397	199.9613	210.635	212.1889	691.8482
ZC3H12B	185.2722	55.48484	139.5309	137.6946	170.8242	286.1895	62.80796	119.0044	145.1994

SMCP	247.7707	120.5463	95.0622	166.8528	90.76097	164.3215	84.09736	114.2477	475.3498
LOC389257	33.41842	45.99837	36.97595	37.15557	34.32563	37.01117	33.73228	39.12841	37.79875
TRAPPC4	51.46777	51.39459	685.7628	37.27253	822.0321	172.7237	688.7769	695.2068	36.2795
NALP5	104.5376	119.9062	74.1966	105.8929	61.59267	91.52875	58.43404	65.6097	68.22037
TFAP4	36.58907	43.95792	111.8975	49.58327	234.2035	197.546	249.028	260.7556	79.65175
PHLPP1	75.82899	49.1031	378.8797	38.0395	694.155	46.5622	388.836	1002.735	52.14296
BCMO1	961.196	2343.933	611.2076	363.7779	938.1662	1197.562	1478.125	463.4789	1635.939
ILDR2	149.9753	126.8862	70.99607	150.5724	66.58382	78.1843	57.59641	78.38203	286.728
PCDHB5	32.84377	34.47025	38.6171	33.60427	115.9328	32.81545	36.95534	34.26462	38.82862
RBP4	51.30151	63.35379	52.96168	83.01124	51.15284	69.0024	48.13137	39.19228	65.50839
SCARB2	39.82593	2149.675	2853.078	1616.704	2630.487	3447.692	4007.099	2128.999	69.03803
CPNE4	58.48255	59.75705	45.79229	49.70719	36.83605	41.86136	56.93492	32.58579	34.76349
UBAP2L	2280.847	1921.879	850.0032	953.9703	1274.017	617.3963	883.6993	1091.832	1776.478
BFAR	39.87695	36.18332	41.84229	61.38946	38.11945	148.4287	64.94766	53.95062	50.28635
C9orf7	346.8596	244.8843	117.1888	252.0559	115.4022	155.5156	85.94894	137.6602	483.3194
FBXO39	48.75528	38.67526	130.9825	34.73655	32.19607	219.9361	38.1372	142.2383	67.71558
TLE2	53.55964	2888.983	1580.726	49.64544	3014.754	1628.024	2022.323	1741.921	1986.975
FAM55C	92.09168	443.8064	196.499	325.9788	81.5108	291.9521	314.7543	348.5201	1918.516
PIAS3	841.8956	427.9853	226.7154	793.418	648.2678	38.39801	306.297	221.7313	1144.228
TCF25	5408.03	7319.909	8676.04	5902.47	9654.589	8530.468	9067.37	8313.476	6229.653
YY2	51.895	338.6784	60.36026	41.26458	53.98418	53.76007	78.96136	41.39041	49.40581
RNF141	1818.573	1440.537	835.9258	1948.237	681.7463	972.2214	642.5969	876.7761	1755.3
PIM1	1159.743	43.17022	644.1361	1326.092	597.2385	42.54391	249.8258	234.9939	119.417
SOX7	410.8921	560.5778	348.9386	218.2945	1177.223	1348.446	622.4554	442.8825	496.1579
HPCA	71.39255	36.2594	34.97873	49.30196	48.26199	68.60153	113.1885	59.07881	46.27894
C6orf153	10999.35	11285.79	12832.78	11566.36	11881.48	11063.71	10235.08	12701.42	10685.69
MRPS2	55.63614	39.15891	540.949	880.013	990.6409	65.6393	657.8946	751.3948	43.91542
API5	38.31784	1481.531	255.8717	957.9895	799.5858	329.42	383.8306	51.26959	55.24993
PDGFRB	2289.708	5038.665	7070.726	4781.689	8139.256	7412.623	9662.77	7369.01	3404.107
PXK	48.64528	93.40071	59.53569	81.61201	60.64046	93.81244	44.00038	45.87914	196.5239
WHSC2	474.4512	124.0037	92.97872	156.0864	114.6468	133.568	74.99438	98.24166	285.16
NINJ1	980.6327	1457.043	3521.759	2484.647	1898.316	3056.959	2666.849	2536.34	1839.846
OR6M1	79.65852	92.0831	47.71016	79.0803	61.02452	90.16824	51.30208	57.8742	65.88978
LCAT	18316.63	15783.74	10403.68	17047.87	8038.821	14614.65	11535.33	11646.08	18038.3
FLJ27365	163.6852	278.509	151.8425	256.0705	59.43671	184.6473	125.451	89.55795	61.92759
CYC1	34.76959	1512.657	3377.418	941.5394	2797.431	562.3044	2722.632	2401.319	41.82091
ZNF554	195.8849	94.69546	284.5927	175.1668	249.6467	77.02453	184.6469	95.8333	87.94392
PRDM14	51.19911	80.79608	52.37624	46.26599	59.59191	52.91328	44.18497	78.64941	62.63746
PLAGL1	65.23703	64.26134	55.77714	60.89641	121.7498	69.09779	167.4153	289.2572	68.39095
MICALL1	1197.391	45.56956	542.3273	1231.038	1610.604	928.9366	1709.944	1297.594	68.06838
PI4K2B	265.1708	444.6855	238.6787	350.957	368.8861	283.1048	459.5506	396.2461	619.0297
MAN2A2	1231.157	428.534	241.1156	239.149	168.5733	361.8201	295.7974	130.6453	518.3257
MYH10	8926.226	8006.68	6519.706	12070	8442.133	11742.76	11072.98	7339.12	6774.92
GPS2	92.84962	81.38955	79.95841	70.77601	281.3308	95.54132	75.61197	61.16448	254.8546
HVCN1	182.3175	715.1329	1688.376	872.6758	710.9154	990.9009	1295.787	566.1254	230.5516
MCM5	364.7933	199.1508	316.7118	212.0571	306.8557	193.6108	334.1605	196.5443	623.2453
ASPM	1438.879	1268.554	2192.482	2970.435	2146.133	3034.681	2195.935	1792.767	1236.669

HMGCS2	77.78317	81.51349	50.52683	90.28568	51.13083	52.75326	53.50407	57.78068	67.41817
LYZ	43.68021	591.1905	1026.677	39.85651	409.5813	2617.663	1403.675	1247.758	2859.934
OR51I1	55.24366	55.39613	50.02399	36.91351	54.18633	46.26041	47.90512	42.45469	46.75886
PDIA4	49.37611	40.18732	37.40381	36.51987	177.5048	44.69588	84.2356	35.44242	42.91361
RHBDD3	53.73507	1137.044	315.1965	54.07287	374.5213	54.71482	603.717	996.8859	56.21014
B3GALT3	151.4864	100.7208	62.30727	48.78268	60.9439	62.46672	53.03447	54.13345	107.8485
RPL7	425.9292	202.0032	130.3648	305.5958	161.3238	139.6966	133.959	125.9844	513.6002
EPGN	308.4893	364.5616	193.3651	65.4446	274.0155	89.21415	176.5031	104.9896	67.66082
NR5A1	55.10612	41.3125	39.37733	68.65607	45.78908	60.64291	52.77887	103.412	55.17915
CCDC68	60.96934	78.89275	64.99802	113.0683	57.06046	62.33337	52.25608	64.46488	159.8915
EIF3J	70.02353	573.5059	256.6542	702.3829	160.8349	477.9605	186.044	157.3832	1160.69
TMEM136	40.71126	34.35386	524.0873	650.8107	1433.318	971.3464	844.297	1134.343	54.34407
XIAP	495.963	255.4895	101.6496	251.8102	105.1932	158.2159	77.4155	107.5231	682.9684
FLJ21865	2370.379	1649.005	1259.131	2070.237	861.6565	1510.795	1341.979	853.1908	2284.933
SMAD9	69.62585	53.9151	48.05766	40.99954	141.8679	65.66141	108.5373	131.7197	54.66251
CLDN17	34.24918	84.08492	414.7698	58.73806	661.3631	54.1277	170.0702	407.8835	74.89286
ACTRT1	392.9923	190.052	100.7947	207.7194	98.93453	184.5109	99.27168	116.8876	551.6257
APOBEC4	41.52274	37.52068	32.40608	32.83713	36.85995	37.17833	32.67683	35.38583	35.13442
FKBP6	33.09989	35.82982	37.41951	32.39585	50.68841	42.78552	32.52423	36.87786	44.53516
NOP10	1896.661	2949.913	9365.047	4659.815	10132.36	7824.547	18057.7	10208.87	1646.531
NAT8	89.59616	90.90981	43.17268	68.21672	59.94045	67.54013	45.64416	45.51132	70.99606
ITPR3	6715.522	7540.289	5401.755	8425.601	6664.944	8082.354	7494.756	4971.779	6368.071
HBD	274.4612	112.9708	93.68641	504.9846	67.67876	139.5996	69.60764	135.7836	220.704
GCKR	69.54527	54.8371	48.98336	64.19817	50.17417	53.2869	49.36858	45.9199	44.18229
OR52D1	349.4026	206.718	106.2442	231.5934	86.53169	131.6003	80.43842	116.1725	509.5796
NTAN1	616.3582	234.3806	157.5953	256.6388	113.007	149.733	141.0181	193.1395	495.4732
PRAP1	62.41544	51.36004	55.17082	140.7227	43.7072	55.95926	49.99385	63.57664	49.52647
PLXDC1	134.7283	111.0114	108.3108	139.7771	81.51386	162.6481	93.11526	83.26052	126.4779
HOXA6	143.4139	161.0271	77.53818	153.4678	70.2804	90.66283	58.72428	90.79132	151.0069
ZNF223	9861.691	9128.553	5679.917	7558.86	2618.524	3709.15	2846.864	3260.027	9831.237
SYT12	69.61466	61.56049	59.31802	80.1925	57.86297	58.71552	49.8334	78.91982	165.4911
CDX1	44.47227	52.57663	825.3301	45.99997	162.6167	603.6718	1208.744	995.8473	37.47205
DHCR7	172.9839	137.2785	70.41649	93.04217	74.99043	108.5035	46.29921	67.43221	125.5338
COX4I2	56.13606	39.5345	156.4382	45.39736	43.23836	42.12633	50.67043	57.41925	55.76038
GIGYF1	683.8433	778.8674	528.2735	1163.048	296.8998	552.5526	338.4123	365.2608	754.5989
POU5F2	34.3927	49.89335	39.17325	44.2279	40.73042	35.72799	38.60367	48.42096	39.68749
TIE1	2658.851	1738.727	1230.307	912.1624	1161.454	2555.735	1308.474	1622.765	1518.347
WNT3A	1167.169	1915.56	656.0809	3575.585	1897.932	2725.588	1595.167	1889.363	3493.56
LZTFL1	43.52401	489.5621	1343.942	49.87502	1935.556	1135.916	1736.121	1347.658	42.03272
LMLN	104.8305	93.96257	251.8146	95.0788	55.02232	65.43978	115.0243	49.74282	66.90745
PLCG2	70.03351	486.5922	1455.782	381.0098	1770.536	700.6293	1583.962	839.1455	33.2069
FCHSD2	5193.565	7234.663	6077.201	6848.578	4304.322	5932.809	5930.898	5602.167	7652.647
SOX4	192.7425	182.6502	1184.807	507.72	1696.613	1016.054	1800.873	827.5718	1078.21
CTDP1	258.0355	75.74961	100.3455	76.96554	95.92049	67.63596	85.92662	53.16069	101.0482
TMEM190	430.4541	256.9043	131.5871	236.7206	108.2343	185.0839	129.2053	227.1541	696.9495
NT5C1A	48.56038	82.24621	65.36475	41.97469	43.34643	64.41146	41.55383	41.80408	117.187
C16orf5	74.51479	70.07633	56.86968	94.46911	779.3979	57.50979	615.9307	584.9131	52.38515

IGLL1	128.3173	123.4053	72.77592	86.289	52.23109	85.20885	51.69857	56.55954	153.5657
OR8B2	55.00002	54.84934	42.47701	57.04501	50.24632	51.17114	45.4027	54.90994	84.49824
ERCC2	131.6537	2020.883	427.7942	654.1766	465.8655	1038.522	1185.383	1331.192	47.4028
NANS	70.97134	654.1073	127.5075	858.8185	194.0307	63.29383	191.2546	161.0815	1288.607
C11orf59	4943.972	3976.201	8979.615	6712.833	8690.582	8175.59	10596.68	10747.27	327.5194
ZFP112	157.9197	90.83061	84.67992	114.5219	111.0551	102.6205	140.2709	149.9226	93.51573
NUP107	3076.3	2222.324	2742.953	3246.338	2292.592	2782.687	2465.199	2157.719	154.4331
CXorf67	36.84226	45.7319	40.63863	32.77769	38.21332	37.34421	34.38885	31.23416	35.35458
FTSJ2	335.358	406.1414	323.0759	334.1534	546.2188	251.8852	406.5213	455.3447	515.1978
TMEM184/	120.9508	239.819	397.3828	616.7456	398.2	387.3683	391.7282	350.0567	254.2924
PCYOX1L	108.095	287.5119	134.438	42.49387	46.02652	235.6075	192.2594	211.6051	102.801
TUBB2A	663.7084	757.9599	1105.866	96.38576	2159.754	774.2733	1082.509	1746.262	60.42122
ACTB	6466.057	6351.223	7811.309	9538.8	12411.32	5206.839	11300.52	8209.365	6735.968
CKS1B	93.34981	687.6052	2031.396	40.20368	2466.831	2010.885	3990.636	2330.44	60.42821
ANXA2P1	12074.49	10246.68	6753.523	16555.92	4339.64	8948.133	8478.521	6626.237	11110.51
DENND4A	2613.256	1300.969	2198.168	2120.105	1577.47	2092.782	1733.62	1631.618	287.5128
LOC15078€	49.24872	66.26257	75.47394	47.17301	45.88671	58.96024	49.41908	57.61572	70.75031
FLJ40504	651.4478	375.537	402.4394	1475.911	224.6434	253.173	885.535	750.401	1115.042
MRPL37	16257.14	15399.19	12913.79	15796.27	11627.21	12157.3	12396.93	13910.89	17439.46
RN7SL1	11244.76	13065.63	16023.62	11244.6	13612.5	8798.793	14201.68	14401.08	12315.6
GPR172A	820.3625	1726.191	2534.19	1963.021	2048.025	2116.261	3404.237	2152.864	91.39745
MYOZ2	73.97578	55.83568	54.55806	46.65117	46.84891	46.49062	56.90139	36.03962	47.65236
SNTG2	34.94787	43.30391	42.55606	46.31179	36.84902	39.69329	37.57544	32.96852	32.63278
MRPL42P5	713.1413	492.2437	95.0091	557.6692	119.2429	265.4065	199.8565	136.7623	559.5543
PTTG1	3992.967	3277.227	7090.403	5425.821	6357.237	6583.491	5274.315	7116.3	1495.462
C13orf16	90.28689	75.76737	45.05407	40.30668	175.1795	40.73353	88.67757	285.7917	61.46651
PLCH1	38.07836	46.30489	129.0784	34.33627	34.75783	32.67396	32.79465	33.41402	42.20642
ILDR1	59.00157	391.7693	413.4489	105.1878	303.978	880.5175	448.5658	136.59	355.8682
TOP1P2	71.0071	34.47842	41.26266	38.63815	34.35823	34.96434	39.32659	35.52615	37.43315
CSAG3A	182.0466	408.5419	340.9621	89.85016	52.17161	782.9265	54.69375	487.1451	226.6363
TEKT2	361.4626	115.8368	94.81894	194.4891	65.41099	82.59669	69.0533	86.13004	219.6021
FANCE	5644.611	6145.369	6740.691	10949.65	8080.045	7702.237	7550.779	8536.543	6538.673
PROKR2	101.5949	76.81975	70.99625	68.55721	56.37902	74.80088	54.27551	58.29081	182.6216
ZNF341	43.93304	57.8945	586.6376	59.82392	597.8528	37.10392	345.9873	87.58142	215.8087
PFKM	2385.687	5977.228	4648.935	1392.798	5147.999	5686.5	6505.866	6273.938	1845.483
ARPC5L	43.84349	517.3082	579.6219	1481.427	742.3574	983.3783	649.8934	1066.103	54.99866
IMPACT	47.77693	39.16402	41.14054	42.70285	33.80945	39.18249	39.62521	38.64728	60.38811
C20orf191	463.6061	279.8095	160.428	283.9651	309.058	355.1354	247.3018	535.8948	564.046
DEFB106B	57.43151	69.67071	68.72042	70.02306	64.55782	80.27999	56.42408	92.64066	284.4975
RQCD1	49.58846	90.21088	176.8674	68.55715	40.19298	68.11692	187.0786	442.4994	103.2818
KBTBD7	290.5536	158.4792	147.8953	341.2852	183.6936	463.5511	152.2309	253.7198	390.7215
LPAR5	36.42213	1272.077	4596.703	1581.563	5844.429	3651.79	3807.905	3301.071	2126.682
NCR3	60.24428	69.82503	45.35583	54.22039	43.9863	63.83031	49.24904	48.76246	95.53342
TBC1D20	563.4759	337.7102	223.3185	40.81701	256.9486	48.42365	96.9695	199.1432	768.9361
CGB1	1706.71	2382.043	529.7039	4392.271	387.6971	2920.15	1679.46	609.0939	2307.136
NOP56	48.25603	239.9027	115.8161	299.39	105.9695	156.4873	97.25402	111.107	207.7533
RNF5	69.40595	66.59904	40.4862	310.025	54.65108	51.9361	45.75026	65.33665	62.3922

ZNF691	96.07501	250.3763	176.7227	108.3283	198.9255	222.5384	238.4541	209.0561	104.679
PRAMEF6	49.94363	43.17669	42.12749	33.36172	42.033	34.64721	37.95585	54.73264	49.35603
CCL16	676.0924	468.1953	285.3947	100.5952	327.9851	477.7964	160.4554	371.6219	1333.976
EVX2	49.53927	59.21344	44.84299	49.61418	42.60408	47.09505	44.34129	46.89047	72.62068
CFHR2	303.6542	154.4568	97.61323	173.167	106.2022	117.127	80.80628	98.84638	470.0387
PPP1R2P3	48.67798	71.89903	42.60057	41.59377	44.5275	55.28075	42.92216	57.7443	61.9231
C11orf65	445.7714	1350.56	571.3254	792.7277	622.0438	914.1537	556.2527	817.5269	621.5868
SEPW1	1221.811	2060.737	2928.193	1815.824	4578.036	3165.724	3548.07	5196.521	842.6367
SNRPE	88.7345	62.29277	137.481	94.41843	113.3414	291.708	164.7209	127.271	68.93283
NFKBIL1	513.4573	269.8771	152.5868	325.674	113.5123	165.4358	148.7657	247.5836	618.5173
PGLYRP3	104.2874	414.8912	198.8509	339.8244	245.1244	249.5906	183.4363	430.8192	169.8456
LOC40101	35383.44	37506.31	32894.76	37670.19	31117.35	32503.51	30183.07	28421.51	39594.95
ESRRAP2	343.7881	372.1679	189.031	710.7939	135.6074	243.2895	375.0964	250.6894	322.4844
RP11-529I	94.30073	1040.21	849.4971	49.40922	2065.943	1085.991	1952.4	2330.556	68.25737
TMX3	55.6694	58.39985	50.49239	58.38689	114.2311	259.6235	76.19254	43.32083	86.22423
C1orf187	58.24142	59.27221	33.93401	45.33525	34.49685	41.64682	37.74447	36.13615	41.68917
CENTA1	15667.93	13385.56	11423.68	11417.71	8712.862	10867.64	9519.746	11250.21	15724.37
TMEM50B	521.6136	3522.169	4316.019	2783.618	7433.32	5629.146	7404.285	3400.026	175.3991
KCNE1	67.16442	43.56051	51.04225	56.97496	33.58115	38.68415	39.53839	38.47617	52.81207
DEFB4	2062.741	229.9403	4390.041	67.33987	15339.14	1063.63	4408.042	7327.216	1347.986
TTTY2	56.27417	98.75556	63.6356	71.42825	51.77747	61.21226	119.3885	54.27063	104.0068
RPS27A	8096.997	9436.387	12999.95	10323.22	12226.06	11174.95	12522.86	12593.02	7538.431
ZNF643	271.4225	224.5155	89.18288	279.4348	69.18354	129.3352	92.24842	122.2705	350.7159
UGT2B15	48.81244	43.37667	45.2406	45.16295	45.29359	57.0503	35.99561	44.74358	55.39879
SLC22A3	65.69233	39.09665	30.74166	47.39908	34.46896	52.40068	37.40612	34.88848	34.58627
SFTPA1B	68.97466	73.62422	40.6967	69.64561	65.25333	57.74829	51.68118	48.12435	64.13639
ATAD2	138.3236	124.8462	144.3882	91.59082	51.90273	190.5299	199.3845	60.29223	150.2135
NUBPL	9031.368	10573.83	9869.797	9759.431	7241.323	7125.881	8319.613	9515.33	11681.5
PTTG3P	88.49396	79.18801	558.5698	919.273	319.7157	553.9631	58.16826	853.1423	299.9973
DNASE1L3	1600.411	36.31487	4790.92	58.02065	2829.129	4287.063	2054.624	2951.219	45.98499
ARHGDIG	64.9963	54.50069	32.42941	76.08856	53.6662	35.99674	43.78513	36.09312	41.017
DCI	130.9207	209.9308	362.3011	300.0331	548.4391	539.6971	441.6624	498.2505	588.5037
LOC642947	27547.43	30632.86	29881.56	27930.19	28414.77	22879.47	25995.96	27046.98	28810.7
TMEM86B	53.31486	947.1834	1317.043	1016.41	692.788	1150.283	684.4283	814.3085	849.1782
TRPV2	36.61508	41.42066	534.2902	32.48974	298.8643	307.0332	447.7598	416.7359	49.03121
RPA1	656.1741	543.9368	2576.046	1846.013	4137.85	2384.679	3630.73	2626.576	51.84597
TMEM16C	35.60578	37.78131	32.05523	45.04942	33.30912	37.47846	33.05357	52.40866	39.07249
C11orf71	45.9203	55.47442	43.34522	77.85136	36.2468	47.73407	36.86847	33.76889	39.49556
C20orf111	34.65258	37.69161	819.1147	44.97844	1765.081	719.3835	1642.35	1404.788	88.44506
NFE2L3	73.50879	283.0676	431.5832	332.705	390.9432	340.1603	375.7176	327.1777	234.0153
HTN3	180.7583	106.4032	67.69743	107.1445	75.869	84.95245	53.73021	76.20213	130.3779
CCDC23	43.33239	41.93433	1719.913	37.50203	956.428	494.3659	1636.901	3542.783	37.33817
GABARAP	559.0863	349.449	196.2443	261.2051	140.4087	207.9423	157.1543	311.4391	639.3979
MYLK2	952.0035	3702.788	937.9874	86.11044	375.2726	1647.046	1889.795	3769.923	585.7931
UCKL1	44.76226	38.96946	38.19154	42.15837	347.1779	36.68725	281.4306	296.778	149.057
SAV1	162.7899	50.22866	46.4047	55.06442	42.72017	161.3317	57.82973	36.93422	63.53813
C11orf52	51.86202	44.79462	39.78446	49.29918	42.65702	45.80619	34.7919	31.35081	61.20964

SNHG8	13751.05	11615.18	11203.38	11262.25	11475.68	11249.59	10044.61	10613.9	12333.98
LST-3TM12	41.45502	53.0585	43.78987	66.53271	40.9671	36.17612	58.48649	53.66236	48.83928
SDHA	614.4483	1776.87	1035.45	103.0089	1105.7	64.12	3395.715	1231.293	2101.133
NEK2	3220.803	2642.698	1750.882	4730.714	1390.82	2953.59	2365.809	2723.763	2859.874
ALDH16A1	116.8644	1000.525	758.1119	105.7572	494.114	99.16841	159.1198	80.38346	204.1815
E2F1	70.60982	42.55211	54.39324	79.616	67.27908	77.92252	55.09489	40.0791	36.82811
DPRXP4	147.4898	64.63085	80.08291	39.69075	61.3626	115.3611	51.92603	69.88264	68.16997
RPL37A	3504.481	1930.545	2069.261	2493.283	1948.729	1975.923	2079.852	1936.732	2890.267
LOC401152	1070.06	40.25524	300.921	52.7146	121.1653	35.43972	115.3788	145.7426	34.3199
PTCD2	35.5643	45.55385	214.5115	691.8171	160.5209	51.80605	97.77765	37.7455	33.60339
GPC3	50.77852	62.98072	147.7904	56.57537	117.5441	41.75655	76.3931	52.91373	48.39615
LOH3CR2A	43.38711	70.2085	51.98114	58.95148	389.9108	49.70503	48.84695	48.81712	199.406
GBP7	48.25143	58.11684	105.8792	57.29733	53.06301	48.78207	40.78065	41.73052	68.21022
MYOC	39.53176	410.3233	117.1972	36.32441	42.23298	38.46322	45.00456	49.05672	47.55777
RAB17	1909.767	1446.75	2320.604	864.7312	2998.999	4137.693	3541.486	1588.566	4048.078
CYorf14	202.7466	127.7387	75.78561	102.0979	76.18389	91.96948	151.9654	192.3991	145.6104
GPR45	43.28384	43.98233	38.59902	54.43027	33.50985	31.89824	34.29934	34.13392	34.05608
IL13	47.89182	34.25169	35.0276	37.50885	35.75703	45.51492	31.37781	35.95242	37.69412
DUX2	145.9174	91.20782	73.90663	86.8567	64.65374	94.5452	54.89582	52.26553	183.156
LCA5	140.648	124.796	65.79203	100.7795	63.90735	75.17487	57.16778	74.19611	231.209
GRAMD1C	39.16314	33.60402	36.91979	35.99055	91.60068	38.17863	55.80136	35.59763	41.29037
TMEM116	214.1527	91.27304	62.76722	94.66771	231.7444	79.60389	252.4816	70.18409	278.4414
ACTG1	2064.052	2668.132	5545.765	3903.418	7584.314	4551.374	6585.408	6513.312	2384.535
FLJ16734	97.9271	62.08895	62.9746	75.73724	56.54821	70.9886	59.12291	48.83	69.54519
LDLR	4599.213	6321.823	7718.432	8709.702	10619.9	7978.941	9108.341	8447.125	5001.073
OR5L1	42.78396	35.86678	48.00011	52.27153	42.28928	48.12078	43.37975	43.493	40.59122
RHBDL2	441.4947	245.5221	122.1208	221.8616	107.4646	129.9478	169.7571	100.6891	483.0118
LOC653314	587.2803	294.9832	249.1081	341.4381	237.7093	249.9383	300.7023	359.2479	338.0355
FASTKD2	357.7574	255.721	86.62516	256.2331	82.63898	162.5573	95.40079	161.4892	192.5699
AURKAPS1	128.8137	78.2855	47.6476	97.62341	58.20855	77.35542	61.65379	91.42905	95.08051
CBLN3	5398.888	5654.583	5901.166	3156.831	2511.414	5636.794	4130.736	4335.879	4713.505
AQP7P2	51.95985	50.53297	52.21167	38.28685	37.13541	44.50084	40.73407	40.44314	68.64238
HIST4H4	43.71294	34.69859	39.62786	39.03308	45.28956	33.93411	31.83134	155.4761	58.26058
ISG15	125.3697	888.7861	3768.47	1020.617	501.1374	2014.103	8360.766	4653.456	48.81539
C6orf126	42.8386	50.11218	33.38396	32.99864	33.40992	38.54718	32.05516	32.55464	39.36946
MPHOSPH8	1604.043	1305.833	1899.69	1411.188	1388.497	2102.376	1336.222	1964.561	1568.263
SENP6	135.1656	573.9374	971.2422	248.3925	1156.642	1002.897	782.3101	797.4128	193.9466
GPR135	730.4089	65.94344	51.13079	49.3413	97.70164	39.36789	108.3428	225.1234	39.82705
LOC284274	66.60931	71.24397	57.03696	73.98542	46.64464	53.58054	57.41791	70.30258	71.94653
PPIL1	32.64068	36.4166	36.83356	33.68205	137.0109	52.82039	136.2286	176.9113	39.46128
ZCWPW2	48.04316	46.43589	49.02737	53.99572	182.379	36.99808	162.9826	179.0836	40.36195
PTCHD3	212.4173	166.0336	65.07259	167.8888	78.8378	142.9185	65.91632	90.91689	245.2919
BCAS3	64.728	43.36086	49.17927	41.62601	35.3627	46.73234	103.7464	59.86955	47.69841
DTWD2	25349.46	22579.13	15408.45	25453.43	14253.52	17179.58	15152.38	14470.36	24818.09
EP400NL	130.9072	112.2606	58.75564	94.72228	55.7439	529.5576	129.8594	64.46025	88.86993
CYP4V2	5931.236	6247.934	4966.394	4598.625	2320.48	4305.373	2211.495	2240.935	6093.602
TYRP1	67.24847	137.9256	68.51232	40.67875	50.46566	40.94647	59.94041	58.76853	36.25203

SUGT1	6207.49	6657.827	5321.585	7505.652	6342.059	7254.328	5124.154	6076.897	5829.561
ZNF407	35.59906	34.86989	37.16374	46.70065	42.09861	45.5462	44.03514	290.4255	66.1021
LOC389458	246.0867	102.6819	80.952	105.3263	93.82588	129.2843	68.67133	107.2745	227.4476
KLHDC1	424.3154	397.2966	103.4013	98.20604	89.99859	227.4475	86.70374	125.9979	586.6437
MRFAP1	9745.158	10362.67	8875.718	10844.19	8069.371	7607.515	8704.532	8857.188	9874.294
C14orf115	85.829	134.3218	78.28908	104.9309	66.37712	79.81847	67.68912	75.99284	45.59943
A1BG	263.2712	222.0062	97.3062	185.0644	100.5853	143.2862	84.71209	96.73403	459.1691
MBD4	338.3693	1617.955	1038.869	1236.886	1975.664	174.9396	1218.322	602.429	513.0099
KIF26B	38.21099	210.6443	32.78206	32.14499	32.83108	38.61613	37.24592	36.27537	44.58898
TTTY17A	328.102	222.6217	139.5991	249.0717	143.8742	180.1893	146.8618	148.3316	389.3603
EXOSC7	225.0368	181.2901	1259.396	188.7195	2596.093	729.9225	2270.687	1713.732	324.0202
CSGALNAC	54.10328	57.17055	177.3533	142.8243	205.3062	44.90224	186.1916	149.4623	939.2229
FLJ44060	66.19634	69.06682	48.80891	95.06864	47.62458	52.61671	38.45681	53.40379	83.14806
FARSB	124.7808	328.1367	68.32746	104.231	56.7593	71.78651	56.36121	77.19081	135.651
LOC723972	48.69996	84.27418	41.79276	45.51178	44.29385	39.47018	34.83086	44.3266	35.65578
CD99	417.078	178.2227	97.31628	105.2866	73.45467	93.16512	101.0652	67.96996	85.90761
RGPD2	1071.984	857.6699	642.5717	1438.89	211.8441	541.9753	588.5458	445.3389	87.77874
TXNL2	71.65316	60.87495	58.22644	56.57137	451.3651	1064.249	385.1263	53.86964	173.3774
RBMX2	563.5133	177.2908	559.5354	625.0157	467.3115	557.9063	508.9503	511.3064	779.2166
PPP1R1A	46.14923	41.89275	32.82923	68.12712	41.78856	40.2978	118.9934	165.5834	56.64718
C17orf56	56.01431	37.90701	440.5787	53.84324	752.769	38.51082	384.8642	301.7353	43.09816
MKRN2	1536.962	1800.095	2129.902	1492.644	3306.172	2900.557	2711.582	2413.364	1804.575
USP9Y	3846.786	5519.679	62.15027	4990.748	58.31248	59.04501	1453.032	1352.478	1856.934
LINGO4	821.4653	1835.304	340.0096	409.0579	219.1694	1188.412	684.1328	1182.857	38.14688
SPEM1	208.0278	186.7655	75.61182	166.9847	62.69722	71.32467	61.68314	85.19534	290.9229
TAF9B	589.658	305.4529	112.8472	286.9642	50.40107	164.1622	107.3559	46.69506	643.7801
PEX3	422.0861	366.6538	402.5347	410.6726	344.4318	408.298	367.446	317.2149	116.1389
PGP	38.26513	44.17534	79.58924	66.61018	49.52534	103.8805	151.4626	58.93101	65.83658
FAM62B	1712.656	3507.258	7020.899	3325.683	11572.21	4012.371	5850.625	4678.537	3048.359
SLC35D1	45.30914	39.80717	38.62998	48.36408	33.24969	60.48664	36.47293	37.87979	56.34565
RNU2-1	4795.524	4906.521	3438.683	6236.063	1715.09	2814.871	3398.936	4284.62	4622.82
PSMA2	577.3499	408.4589	383.1487	390.9223	440.3626	389.8345	324.2243	397.3402	54.40745
XGPY2	37.48521	55.70201	39.49318	40.13191	47.28607	38.17632	90.28849	41.39725	43.40205
IFI27	1349.251	3430.23	10240.29	5843.238	3079.72	7394.014	13310.04	8588.215	96.86165
LILRA6	1745.831	343.3665	161.7559	258.908	62.97036	99.24017	137.1586	72.38938	650.8474
KIAA0776	96.70776	97.46243	77.35776	86.89314	134.0503	352.0923	99.53457	79.53443	158.0696
KRTAP4-2	41.85439	39.42652	35.22794	43.5988	44.81078	34.46111	35.58257	52.18485	53.02463
KIAA0195	1887.436	51.03407	178.2553	1043.413	103.3369	58.57777	197.9106	557.5435	1601.286
KLRC2	60.51528	44.24435	135.4209	54.1311	49.99211	141.3573	77.06389	99.20054	121.9269
RAB40AL	58.5899	411.2913	138.0256	472.0067	45.14965	280.0964	118.0812	187.8393	38.8886
PPM1F	16379.51	15984.11	11964.53	14403.45	11768.64	11492.01	13475.69	11200.22	17939.4
SYK	105.2203	1242.15	504.0147	722.3585	600.1533	1266.585	417.2302	1159.203	174.1748
TMEM54	140.822	1653.482	4385.761	2934.035	8451.624	3198.832	7235.863	4771.583	36.48088
CYP4A22	114.2737	46.79651	39.95802	41.59113	40.9362	47.49109	49.48979	41.84296	56.41581
TTC38	836.2137	552.8827	941.6758	439.8068	936.3633	918.7738	1139.442	985.3253	167.8402
ADAM23	51.49069	569.1022	245.088	49.26481	481.5764	448.4804	165.7926	316.9745	60.99358
PPAN-P2RY	70.86523	55.03969	50.73682	66.73548	49.6964	50.887	50.53766	47.71304	165.8468

GRHL2	65.47634	79.64361	81.19176	64.61995	72.23069	319.9368	44.35488	77.21588	187.1269
MAGEB6	46.06411	49.70661	39.44805	60.48431	43.25552	43.85565	46.72469	34.96303	57.03187
INSL5	511.0985	309.6666	161.8502	296.0821	181.4242	239.6471	109.484	139.2342	811.202
TDGF3	72.69833	50.61496	47.68031	58.51962	50.35206	41.12864	36.98901	36.15435	38.76252
BET1L	264.4034	453.6673	72.60577	81.26389	80.38273	112.8588	65.53169	150.3798	211.6184
C6orf146	88.70502	46.24168	69.99291	83.60812	35.85384	41.07151	52.94712	48.50229	67.70918
PPP4R2	597.8528	307.2347	192.9997	287.6813	255.3592	245.5293	241.9241	150.0591	113.3254
CD48	78.41475	54.81278	1760.531	56.16091	547.943	1333.84	1229.844	1076.514	55.39338
MATN1	872.1982	704.2042	70.01442	127.2388	124.3119	461.0139	187.8982	106.8867	222.4897
SH2B1	69.04185	97.66415	258.748	118.7187	494.6042	54.67154	126.5406	517.6198	83.39286
TAF13	9910.485	8457.392	7042.357	9845.091	8023.04	6702.361	7357.939	9293.132	11022.35
CYCSL1	826.1134	914.112	484.4435	546.7929	266.6124	332.2395	297.2045	395.3203	939.9871
ORC2L	4385.187	4765.229	3185.494	4690.354	3215.784	4550.619	4234.201	2264.327	4649.096
UGT1A8	37.2113	51.41959	50.9157	54.04742	36.33469	39.55427	41.6944	49.57959	48.62828
PLCH2	6637.079	5628.488	5985.278	10953.71	5421.349	8110.642	7996.168	4473.763	7514.046
YRDC	263.3064	283.5903	276.2435	165.3097	382.9894	462.1925	299.0691	169.2062	198.4793
HES7	43.03295	39.17931	40.19938	35.54507	34.51385	32.70163	40.2778	37.87176	39.99188
RABGAP1	2874.738	2656.161	3814.879	3283.336	4109.833	2703.644	3972.752	2934.953	46.33798
LOC44009:	58.00793	73.74383	43.64875	62.93093	50.07708	48.0612	40.90048	37.97078	58.44749
ZC3H15	40.33014	39.4807	34.49451	39.75923	34.04155	125.3344	52.36864	66.68944	80.87223
IGFBP7	50.02014	49.6016	120.3854	45.58772	105.3103	418.032	463.8184	39.99734	53.73219
RBBP4	2260.841	2429.596	1720.946	2356.357	1094.61	1695.209	1387.026	969.6357	1181.511
FARP2	126.2778	127.7108	110.6924	78.50789	130.1846	171.0699	88.41812	88.11343	331.7551
SRA1	1195.996	230.4645	187.671	304.4981	273.6642	174.5195	173.6466	348.4366	555.9142
C11orf55	411.6021	268.7898	140.0236	252.3554	101.8435	164.4198	108.6665	185.9236	566.9518
TAF1D	946.9257	1511.064	935.2953	2268.688	974.4782	1295.898	1246.53	773.6013	765.744
MALL	4595.626	2037.03	3297.041	3485.307	3389.147	3294.471	3142.652	2802.562	4701.11
TSPY2	47.61113	54.77508	48.62865	61.74073	46.70899	40.0412	43.5891	56.8938	43.90926
CTNNBL1	174.4309	69.05135	289.3029	73.06367	350.6306	67.87881	273.8252	157.5955	159.3146
OR4M1	67.60657	43.46666	46.17096	45.00491	37.71693	58.15058	43.91327	46.93796	61.03199
PRRG2	3354.564	1236.647	970.5899	1962.874	1304.417	2008.887	1015.316	959.4752	3333.053
TBC1D2B	72.32063	59.40974	47.24311	48.89993	355.6411	461.3405	211.9222	56.60976	35.57127
STIM1	1336.539	449.6127	278.7936	336.2066	264.5451	350.6918	188.0511	144.3939	1068.452
METTL7B	1015.772	46.77122	44.71844	75.92004	149.8289	49.57516	162.595	556.4044	49.72749
LOC65349	1860.685	62.12898	52.27808	826.4666	132.8232	283.4804	43.29449	113.9743	128.5236
CCDC56	10470.91	10932.21	21362.01	12415.18	22280.71	14695.29	21300.36	22518.24	14060.05
HAND2	308.5972	112.4171	81.73039	125.9875	105.3853	136.8896	72.70386	264.7412	114.8967
KIAA0672	467.976	784.7083	277.7299	239.3061	1266.757	731.7526	1054.228	1023.331	39.06662
ELAVL4	73.39162	74.4724	51.64149	60.7065	45.57973	79.90591	42.89191	35.01461	127.2129
NPY6R	257.4414	335.8482	208.4161	361.0453	103.2975	377.123	158.5902	130.8891	736.5626
LOC16438	41.61134	54.21125	49.21808	41.78042	35.03811	51.88522	42.31227	42.33034	53.77782
TOMM40L	130.0656	2095.323	1003.537	135.143	1387.658	78.36568	1050.446	1914.999	144.122
GRAMD3	1848.809	1102.537	542.4634	1414.764	486.2339	896.7692	677.1503	537.3946	1074.516
SCG5	9896.711	6278.803	6361.116	11979.24	1434.31	9833.751	8773.906	7075.72	10078.61
MCM3APA	39.67622	104.4171	49.8809	70.54867	106.0582	37.98105	56.4357	30.92035	57.01026
HLA-DRB6	2167.786	1683.359	1228.074	1732.155	359.2983	1253.243	590.9878	872.8407	2526.741
C1orf64	75.64292	361.7792	65.28801	149.7115	64.47138	98.06086	50.25377	48.80372	263.9411

AFG3L2	4238.038	4057.802	4704.062	4731.344	5541.959	4901.653	5516.578	6659.432	3857.297
DKFZP686E	46.3879	57.80839	48.36653	74.91663	56.10891	61.44711	175.4169	208.9867	66.8228
TMEM74	43.68161	44.41138	39.16779	50.37094	45.53985	57.78387	35.77053	38.23755	113.6615
RRP8	341.6214	184.9836	216.091	208.3066	288.8034	291.7081	299.4685	420.429	909.2872
NCAPG2	38.68801	36.85004	32.45375	44.41285	32.80142	39.77216	40.40225	34.96557	34.91931
FAM26F	253.437	100.3836	94.3143	176.5464	77.25124	107.457	60.7729	93.74068	464.3141
POLR1B	43.6358	64.08264	59.93734	80.19886	108.3807	58.56128	82.68659	146.2283	167.6021
FLJ42953	109.9951	83.99415	53.39174	87.51024	54.11919	45.77423	52.60836	66.52559	90.18787
CLRN3	275.8438	155.6637	73.60272	200.2547	96.36656	126.5599	114.7682	115.7226	457.9603
PP8961	6361.116	5449.961	5378.492	4493.955	5060.674	4186.755	4057.256	6597.797	8896.833
ONECUT3	39.38399	39.67799	34.9075	57.25494	33.20592	43.05426	37.85701	35.32088	87.671
SNORD116	41.6652	43.69077	59.41568	39.44313	41.60806	40.86464	42.40115	43.48652	49.99809
KCNS2	135.069	64.27143	60.38811	90.95401	60.14229	80.79954	83.27814	62.09651	64.49127
LOC401431	118.2745	69.62076	64.26339	80.14745	40.09746	75.41737	103.3428	185.6881	143.7822
TFB2M	44.20037	359.8011	330.4472	972.2476	306.8798	319.1235	179.0563	384.7261	61.30326
OR1L4	57.42121	44.7384	59.21239	64.44444	48.16833	45.92149	48.1092	38.70442	78.7749
CXCL11	555.5366	325.1881	684.913	399.3146	74.51359	114.7722	554.9258	588.4753	337.8167
NEK5	50.96557	41.44941	42.7453	39.46305	34.44363	48.06679	40.41466	46.67941	53.17153
TFPI2	57.46914	77.406	47.97158	42.91474	45.90134	57.51833	43.22019	39.91661	225.3755
TMEM65	447.3674	218.4424	118.0196	183.1014	68.35369	70.30055	79.42472	39.82601	73.8105
MEF2B	130.3735	102.0114	64.53291	146.4829	61.63758	108.8763	67.95599	98.09124	260.3533
MXRA8	48.87476	35.74624	52.57093	34.83873	35.95378	35.10669	97.89107	33.75802	60.5177
ZNF700	1371.422	1759.419	2790.092	1020.261	2773.962	2333.479	1832.105	1698.555	917.2581
POU5F1P1	42.47553	84.93091	41.59212	81.2178	44.23827	78.24353	49.20388	68.7953	167.3149
TBC1D3G	257.962	87.08681	96.31689	156.1367	371.9146	132.9469	207.8899	70.38348	86.6095
DEFB109	116.6967	95.42317	58.79329	94.90993	55.2254	59.18576	42.33214	65.07407	174.0548
CAPZA3	43.84678	629.2275	208.3979	60.98343	246.4111	47.94691	42.09295	41.64358	36.39111
PVALB	51.21443	43.51719	41.8888	53.51136	41.6985	412.3015	42.43419	35.13163	53.7781
BAGE3	45.05828	41.69727	43.69886	49.99333	79.08492	45.71392	34.20769	48.57978	34.74239
SFRS2IP	94.81635	57.88772	182.0943	93.35676	310.9337	299.7631	242.2282	155.7159	80.17439
CHID1	137.5221	221.9182	66.44303	110.7606	65.99698	72.6321	67.27031	66.47972	152.6925
GTSF1	119.8401	83.06117	183.6683	99.81083	60.17568	106.0115	56.58581	58.92596	201.1541
MAGEA8	232.534	150.5137	91.63186	149.7183	89.14417	98.76757	74.9188	90.32779	361.8704
SNRNP27	62.60217	578.6209	1180.5	1106.576	1390.612	945.9577	1571.427	1192.995	282.6609
SLC18A2	87.81274	44.89917	42.32628	39.47681	40.17057	44.41026	39.69581	35.60696	56.95141
SLC25A15	49.32343	36.77489	268.8905	55.97462	559.4493	55.47924	263.2297	232.1031	47.93718
PPM1K	417.4288	257.7509	234.4123	265.9365	106.4111	143.6754	97.63899	114.1852	563.8931
LOC613037	2045.932	3100.998	1987.839	2900.709	2572.971	1345.761	2412.704	1310.82	3367.095
RNF219	560.7869	459.9325	568.7151	995.85	238.7833	424.2418	335.7443	395.627	623.9035
LSM2	8364.938	6111.465	5463.712	7327.085	4693.314	4619.229	3618.388	4983.719	7576.529
POLR3G	60.43188	79.39015	118.6189	61.35127	57.4415	75.23594	69.22993	62.69742	65.25477
TAF8	1227.062	907.5673	421.6776	335.4716	598.1234	785.4205	543.6589	738.8502	1841.678
DEFB128	157.1539	130.287	66.37806	125.6661	70.47347	111.9077	70.05305	84.91463	213.9725
ACSM5	357.6382	124.7312	89.77454	168.2659	85.76232	130.6997	82.5933	98.46414	310.1294
CORT	93.67096	67.97436	69.17593	84.60019	53.65273	75.37292	51.54326	59.61179	84.82816
FAM47C	185.6641	91.39337	76.35977	101.4167	76.16203	88.44262	64.12891	81.02864	144.8205
CFHR3	39.68478	32.39647	31.33405	36.26323	42.52322	51.54851	31.19826	44.59072	35.39519

RNF152	51.2886	51.00088	59.64704	50.57786	58.75947	517.7215	143.7166	216.1256	39.53374
ANKRD56	35.03267	44.1988	39.88556	39.08539	138.6581	39.71157	69.04092	43.20497	47.37091
HNRNPUL2	9304.501	8343.487	5659.33	8576.241	5171.894	6817.093	5814.962	7904.479	9663.506
C4orf49	43.50498	40.97173	35.21483	43.12811	40.3235	437.9913	105.721	39.08135	72.79021
BTF3L4	300.0585	189.953	271.4785	198.7958	175.3111	358.4953	272.9808	186.0711	316.1346
ECHDC3	81.96643	35.25019	42.10889	54.93945	137.1358	986.7252	666.4452	64.80057	54.15283
IRF6	1608.015	1513.717	877.7073	2707.041	495.6837	1234.012	1009.513	815.3706	2182.168
SNORD31	3409.331	4289.608	2302.49	3463.455	1102.296	3037.488	1703.206	1816.251	3152.153
OR11H1	191.0347	124.1108	55.6799	147.4186	66.12309	97.7072	71.89576	66.83866	200.0881
LOC401357	1644.115	1509.847	4889.339	207.5225	11121.09	4721.584	7419.947	4127.276	308.9352
MRPL14	1714.29	2425.026	6487.693	3099.332	8791.794	4405.807	9311.218	6500.938	2532.107
FLJ12684	50.80761	44.1667	214.7391	62.04731	322.8306	31.82218	459.9284	1116.594	44.60689
SUPT7L	57.52329	56.90811	66.73909	90.84062	182.1179	57.33597	58.70973	54.68456	96.96275
TMEM203	35.64741	32.28996	680.6069	502.1195	1510.935	582.5881	1810.656	1728.829	38.24608
AMY2B	748.5417	1025.127	599.1182	288.5968	364.402	829.6513	370.4301	394.1859	1049.385
S1PR5	1037.723	3154.263	2635.497	5049.877	2321.542	4962.73	4526.766	3470.913	1364.252
FTHL12	77.05996	69.81699	161.4822	77.82478	61.02691	45.2205	83.06739	74.99138	65.11678
C2orf21	183.22	108.5006	78.31998	137.619	75.12903	111.7506	60.99419	67.84041	217.9412
EBP	123.3669	108.4182	61.86966	102.692	63.52348	80.58752	109.3907	61.85671	223.3046
BARD1	2543.802	2150.7	1806.282	61.89798	2334.746	1687.984	2529.25	2156.899	37.94678
OR2A1	1353.879	70.60644	40.54055	50.86877	52.06693	59.30951	48.86026	66.53016	43.39165
GPR4	1833.212	73.60313	438.3933	649.3797	301.6412	70.68233	642.9271	42.98484	64.16177
FCRL6	49.94604	36.11098	33.05834	70.49385	33.92316	36.1578	37.82082	37.76936	44.85068
RGL3	243.0469	163.9104	90.35618	121.125	75.55938	121.6861	72.96503	80.14171	331.8792
GTF3C6	46.24257	44.57647	41.986	44.42876	46.48218	52.37594	34.63985	44.06647	51.26673
TREML2P	134.0574	65.20415	58.81533	95.98675	47.40683	62.82733	56.38698	41.86111	54.63041
PGS1	124.4332	97.24162	994.9406	492.404	1410.563	895.6686	1406.927	873.5779	109.6645
FADS2	2231.474	217.7495	77.88433	3589.939	410.3465	128.1315	82.25638	316.3383	455.212
DIO3	170.1262	118.905	86.94701	135.2367	67.79449	111.9097	64.41845	80.5461	95.7779
MAGEA9B	158.6481	82.51637	162.1817	101.6398	63.46995	89.02032	66.92279	68.32499	168.0344
HIST1H4C	3564.439	796.556	5764.213	6632.94	4165.768	3356.625	3537.246	3820.43	62.89016
PDIA3P	65.82601	326.4571	108.2186	49.43743	62.25683	267.0926	163.7045	113.9637	73.30435
MRGPRF	39.09144	40.58886	51.52417	36.40401	36.28904	34.79493	40.81094	41.27356	50.84531
ZNF284	37.96561	58.29146	44.89782	41.71067	152.4618	51.52721	48.36872	321.7529	65.03883
LRIG3	38.90081	44.65452	133.395	48.48771	90.01171	58.95863	86.71674	80.23315	54.40701
ZNF598	5950.448	7479.105	6415.291	7475.491	9688.639	6290.921	6708.913	8132.137	8970.18
ZC3H10	1223.629	58.24931	1323.077	1059.365	1241.894	833.934	957.5345	689.1025	102.0413
STK40	5175.125	3511.135	4880.341	5025.885	6147.105	2745.635	5128.191	5141.03	5799.371
DSCAML1	70.69974	34.80263	40.3581	36.93815	35.02948	46.17007	37.68331	33.32718	34.21671
C9orf117	264.2438	130.4951	84.12251	172.3564	69.77625	73.95823	59.23014	69.77936	81.22792
GALNT17	113.2429	48.58636	36.72965	54.51277	42.26539	44.58562	33.24202	49.30675	56.0709
ITM2A	239.5595	466.8874	1932.403	90.93433	3345.785	754.0272	2043.083	1515.778	1513.988
KHDRBS1	676.9346	609.0279	653.5052	579.0388	630.5312	336.1046	508.1234	492.613	1241.967
MRPL1	46.19231	484.0641	709.0529	40.19815	652.3906	59.48849	314.2299	772.0289	218.7632
MBD3L2	150.3076	64.08199	65.72067	74.78377	71.59078	96.48306	64.75297	79.60947	355.9954
C1orf149	51.47126	58.71749	64.02617	53.17042	48.14167	68.20101	61.03415	57.43478	109.3489
C11orf2	13237.39	12752.01	10221.15	11922.62	7305.083	9142.194	10260.59	5625.289	15152.5

RRAS2	827.0282	42.24105	226.1001	1357.436	272.2321	320.4601	431.3707	219.6306	33.53089
CLDND2	4275.056	5278.728	2195.721	2288.512	1133.429	2893.212	1400.574	2075.161	4464.273
C1orf109	148.4046	917.7282	55.91726	662.1632	372.7311	87.18086	297.1864	338.3961	148.8266
ZNF587	49.36615	44.39448	578.3298	45.34379	312.1873	40.87675	453.2899	200.6279	115.5621
CNOT10	39.7627	49.31747	225.1744	429.995	261.8815	43.04065	253.997	197.004	42.50496
SLC4A2	55.73072	36.75096	177.1773	35.71561	294.5781	332.6184	105.4635	33.53405	1627.527
ZNF765	1045.674	1474.806	966.491	1636.861	167.3693	404.0379	320.9167	341.723	1218.036
DZIP1L	309.5893	156.4901	94.52848	190.8681	130.2198	134.7656	79.49657	119.3554	440.5786
HSPC268	515.0073	400.6627	201.3763	344.4619	144.9123	187.6733	114.4694	179.8745	776.0824
C6orf105	37.47532	55.37872	266.0173	33.93403	32.38289	440.9081	115.6279	33.55735	48.17879
ACP5	55.09112	48.73842	35.84307	39.04834	32.88248	30.81986	40.70644	37.91317	126.7533
DHFRL1	50.57789	304.737	322.5582	334.2744	344.7923	705.7567	406.1974	153.1106	56.86451
ALPK1	56.08179	752.483	1241.066	40.61652	499.5096	33.14652	1103.269	1060.748	45.55288
C14orf153	691.2496	497.2301	259.174	629.424	135.6299	265.3496	174.2062	132.0963	77.22876
CSRNP3	2393.461	1686.569	1007.262	557.567	1773.068	1654.372	474.7657	459.665	32.10529
MDH2	13287.36	11895.3	10085.76	11865.99	8279.2	8411.475	7579.295	12628.86	14140.47
KRTAP4-11	41.54473	35.40869	36.20546	61.24837	45.43831	39.20128	33.99657	48.1668	40.42036
LOC650293	57.08569	53.34226	34.47729	59.73935	37.24821	54.64265	36.23825	37.81757	55.68307
RPL22	1147.922	98.19336	57.63853	110.1272	397.7247	92.44059	246.6671	244.4524	207.2655
ZNF204	33.13029	325.0956	421.7846	1100.288	771.8208	923.1459	590.3683	631.8375	32.92273
KLRB1	66.15801	50.8833	476.8476	43.3244	551.1777	114.0459	454.6168	302.1268	124.6376
LSM14A	9331.612	10611.21	12720.06	10342.65	15191.79	11757.96	12509.63	12708.77	9019.044
ERVWE1	70.86961	65.34771	51.60419	84.53415	51.51818	78.10041	50.81519	60.2225	59.16101
MAP7D2	48.45401	51.38602	70.29907	63.42566	47.45469	68.41264	54.5239	59.02076	41.71444
FAM10A7	924.7838	1032.604	402.1591	752.5602	219.9433	557.941	262.4629	560.9752	1050.225
TTTY13	105.1467	37.51512	56.9911	136.7495	53.03691	55.16151	48.2508	61.01237	63.15833
LGALS9C	65.94554	59.81895	113.9785	324.3441	202.4481	66.05161	129.5257	152.2696	66.01493
ZBTB44	818.405	1596.217	2441.458	1259.975	1599.17	2279.174	1362.9	3332.78	59.93543
ADNP2	3144.164	776.1313	1245.693	2588.01	1152.331	771.262	1141.553	590.2079	3543.768
SNX22	416.7639	908.5358	1302.072	255.8389	200.3662	180.5878	538.2731	232.2022	1827.059
BTC	95.30935	50.60337	138.1827	48.41671	43.23485	46.19847	67.60237	140.691	43.762
HSPA12A	17698.3	17112.88	12708.01	18257.32	14027.79	18913.56	17701.61	14679.46	17499.94
RAB12	622.6296	548.1616	165.7619	1315.749	218.7505	245.5611	337.4545	219.1468	879.9217
OR2A20P	62.24861	125.7927	46.93055	60.61691	49.8499	78.25282	54.56153	61.49374	52.2333
C7orf44	44.46235	1419.524	3034.223	2149.737	3003.946	2326.767	5049.976	3800.218	123.6044
PJCG6	54.01175	1503.482	290.2288	608.4912	245.6911	43.98879	223.5692	182.87	55.38476
NDUFB4	177.3768	225.2391	127.6363	239.7248	118.4786	195.3566	169.8414	172.3736	84.25041
GIPR	47.43639	51.25793	47.60124	41.46051	43.25297	39.37562	49.53226	216.9776	51.98981
ZNF214	397.0731	34.91716	61.94919	37.59845	43.31585	44.76226	56.40252	45.36223	37.74025
C1orf123	10161.8	8724.724	5547.206	7873.078	3156.328	5505.82	3682.488	6246.678	11318.02
C20orf100	3362.556	2631.572	4922.934	2468.017	4119.846	4547.103	4562.49	6294.691	3822.426
TOM1	15219.13	14363.4	14166.36	11838.77	17574.11	14699.37	14628.96	16484	15638.65
SLC36A4	3047.411	1984.565	1105.035	3196.844	725.6124	1067.108	782.7457	719.8842	3863.016
PRMT7	39.47879	45.09706	41.90395	62.38792	43.37455	56.97382	218.8677	253.318	38.03528
KIR3DL3	57.51476	81.56209	112.7698	73.12258	52.1402	145.8726	46.00888	52.14386	53.49824
SNORD68	1173.589	371.1978	184.4169	563.6296	160.3734	438.8602	216.6167	106.6701	103.0103
BRD7	14688.98	12815.87	9181.09	12726.44	8601.274	10309.46	8687.236	9492.373	15180.02

LOC440157	7934.416	11936.26	5707.465	12692.86	2330.726	6428.014	6495.037	3307.663	5913.799
OR5D16	77.10697	56.94289	44.15883	84.89333	49.45113	56.33598	42.04491	49.17156	38.07383
LOC284379	38.4097	42.11363	46.28591	37.908	49.69749	63.83933	35.93763	52.64841	32.97096
MNAT1	57.33671	408.4792	169.3674	549.4069	233.2634	191.7362	267.4612	162.8436	118.2432
PMS2L5	1570.525	127.5874	234.7009	476.0128	196.6174	267.7671	161.4129	167.3209	1165.621
IRS2	62.35787	74.25554	46.24994	123.491	60.31725	73.69501	59.93685	68.64746	349.7608
KIAA0415	64.15127	48.94791	53.19391	654.9885	54.49121	46.41891	59.43666	39.1536	85.41429
PHLPP2	60.22962	40.68141	46.26951	54.5906	153.746	352.2935	42.42302	61.28427	43.33136
CGB7	34.70232	61.88867	50.85249	38.99874	48.73516	44.8898	40.01635	40.04023	76.01301
TTC32	70.00562	39.78773	123.7519	289.927	129.3828	239.4582	125.0454	66.42812	56.1913
LOC400707	72.69436	53.54824	69.69875	103.1217	81.0886	78.41856	64.26099	72.79887	103.0455
EXOC8	45.77414	114.0898	115.7731	164.6105	108.1612	128.8851	87.5071	69.39058	56.9266
CNOT6L	41.3699	65.20142	983.7255	68.54606	611.3203	570.5396	413.9002	724.6352	35.74612
C6orf174	49.57458	43.77998	44.06503	54.85892	36.15664	44.09086	40.70966	34.78032	58.45608
SLC12A4	269.6588	88.67413	61.56896	343.9294	138.8726	74.62801	125.4432	143.6842	167.9944
M6PR	2309.486	4743.564	5188.654	3627.758	2853.104	4957.228	4819.644	3478.2	60.99214
ZNF595	456.3957	247.4608	298.445	215.5267	95.01152	113.9017	117.6236	141.2667	652.7235
PPP1R3A	51.51115	193.0353	38.57504	48.12882	44.83212	107.5435	58.88276	85.9596	43.09808
HBG2	350.2134	1481.086	111.2617	236.9119	333.9348	483.213	75.23969	375.5642	474.416
LOC440053	34.36655	49.26122	35.19665	45.52235	41.01471	35.63353	44.61255	34.80695	51.56417
TMEM176B	3520.079	1509.662	1138.524	1313.826	513.1108	947.1339	661.9318	983.4735	2127.758
OR4D10	229.413	82.33057	56.8055	115.7387	68.08494	55.82536	52.04016	61.64092	237.2788
FLJ35848	46.37182	42.54012	45.28876	55.83389	55.24297	35.52785	43.25431	41.10859	46.53054
FOXD4L1	51.79745	45.48751	97.99014	38.57663	65.18541	70.48265	58.31683	56.35152	39.60765
ZNF670	30.25838	61.79766	34.8697	39.13256	36.67757	32.35917	37.87303	48.1402	42.02534
SNORA32	669.2867	315.6113	175.3093	215.2848	112.9712	42.29979	76.67654	134.9654	728.3535
C12orf64	55.42031	330.507	37.22768	302.9829	208.9606	151.9874	152.306	126.7917	697.1841
ING2	289.3484	423.6014	1289.105	1101.722	1005.612	812.1766	419.1659	740.2303	73.50131
ARPC1B	553.664	917.0613	748.6214	935.4073	269.1561	144.4514	405.9115	622.7581	1569.841
GXYLT2	631.3417	46.83429	53.55203	40.74354	149.2305	69.38821	104.5298	63.5154	41.88717
SLC15A3	7058.922	7373.275	9763.513	7271.665	2734.139	8562.155	7923.363	8841.119	5702.669
SNRPC	9107.78	8366.481	5574.587	8263.277	4081.683	4757.078	4996.869	6019.108	10084.38
JUNB	1050.313	100.6109	496.403	568.0384	378.2708	306.63	410.0139	345.9175	171.9463
CCR4	848.3766	32.30706	302.4001	35.17765	195.9669	32.65134	140.6626	277.7833	55.91518
SMYD4	1710.133	277.5167	510.7525	957.5535	1349.885	1410.129	1639.38	747.4203	68.41108
NDUFV2	1659.137	2345.415	3911.087	1189.282	3642.263	2864.328	4150.946	5463.052	1260.573
DEFB112	48.14167	75.64536	35.49156	55.34703	48.55498	54.32418	46.73458	38.78303	41.73238
TOP1P1	41.54483	36.13962	47.64153	33.95473	41.14694	41.50116	39.48811	34.60081	55.97787
CCL1	84.89863	76.73626	51.36032	39.17451	49.59797	72.29167	46.17936	60.66785	43.79352
TOMM7	30593.41	31219.04	33247.84	32057.75	29953.49	29997.72	30615.02	33446.56	31924.32
RPL5	15966.09	14386.81	13310.86	16328.44	11716.56	14375.3	12190.22	11994.16	13831.97
XKR8	59.57585	43.30641	235.3555	69.3103	116.9241	63.06723	297.5688	190.6381	60.35567
CPSF3	3659.832	3637.468	2734.947	4416.15	2879.508	2203.89	3235.682	2512.945	4875.479
ZC3H4	50.45171	60.45912	184.8146	58.25488	334.5392	204.7906	264.1805	217.7746	96.67544
ECAT1	198.533	56.46349	65.33454	60.32088	70.19519	61.65301	56.7422	127.7796	109.8215
TTTY8	234.4308	77.59543	69.79198	127.2259	69.6351	71.90296	67.03997	74.33478	164.5614
CNGA4	52.78536	60.04149	45.99572	79.13695	46.56624	69.97395	48.74981	59.05377	197.9054

POLR2B	528.0568	309.6231	185.6481	294.6772	153.1581	247.0525	150.6185	222.7244	722.5849
GTF2IRD2P	5858.23	8628.662	6805.448	7991.118	7881.101	7818.189	6736.177	5422.601	10033.72
PSMG2	1665.624	1686.207	1533.427	2829.808	860.2842	613.7303	765.7738	1071.439	2522.541
CT45A1	45.87059	38.3023	36.06687	39.15596	35.35124	63.94716	38.125	33.5654	70.08287
CHR415SYT	42.86803	41.91718	38.43449	39.23109	59.99796	38.87617	32.32014	40.11361	38.48827
CSNK1A1L	63.64312	43.70579	34.29281	35.22601	42.45156	33.30821	36.96044	46.38766	106.1345
OTUD5	433.6612	278.4224	153.33	267.9899	151.6687	196.4318	118.6318	183.2843	490.8946
OR9G1	70.2929	78.10609	58.71157	59.64359	51.3205	71.14753	53.78542	48.26095	69.71311
RHOD	49.80511	38.24231	1144.679	839.0633	2098.56	288.2019	2306.017	1623.145	64.86209
SPRY2	7553.13	7425.304	4584.233	5721.581	4893.755	6334.516	4968.246	3701.155	7117.13
SASS6	1309.482	934.9999	406.4146	968.4672	215.427	177.5526	284.4254	112.9541	764.571
CYP4F3	44.93671	49.00254	37.61521	63.37694	42.32662	59.28979	55.7216	37.22457	41.13925
LOC285016	1068.176	174.9311	237.0196	167.8988	282.1723	96.99207	64.70086	88.82275	173.7735
NUS1	506.1115	277.7151	198.7661	289.9716	235.5037	175.9595	128.8002	150.2547	335.155
FKBP9L	64.0763	51.09491	400.7308	812.4789	1749.945	617.2355	1421.305	605.0937	34.20473
AQP10	3551.785	2463.941	1988.069	2745.652	1528.549	3464.175	1811.083	1490.098	1841.49
CDY2A	36.01404	34.02587	45.51026	34.34068	63.71067	48.05429	39.8021	39.48258	41.22429
KIAA0495	330.0114	149.7759	127.2254	178.3651	136.8569	172.8913	141.7723	182.3109	370.9461
ZFY	45.62172	38.17414	39.54713	43.52555	43.33036	44.92706	63.45661	43.66874	65.40646
OR7E156P	43.99253	61.57051	38.34463	39.78773	48.45143	49.51736	45.86048	50.64573	89.08681
ISG20L2	683.6062	476.9882	317.9242	939.9251	368.4568	434.3084	325.3742	529.743	887.597
FAM110C	1082.958	736.5322	770.4878	51.13326	207.9806	36.13899	162.8497	645.1718	1410.24
GCSH	341.7975	1067.866	105.5386	248.9115	95.27138	560.2287	793.888	125.153	442.8506
TMEM79	8419.712	8549.38	6012.403	6065.025	8397.63	6861.838	4853.813	4522.902	8490.539
NAG18	12555.6	12988.25	8961.474	10599.04	5151.73	6562.247	4869.234	7612.18	13962.48
GNGT1	48.38892	412.3451	47.02062	279.2176	43.75485	185.1936	67.68914	201.6069	39.29155
GOLGA2LY	76.31794	98.4274	50.90799	53.57827	46.41478	76.1431	46.98198	50.04819	175.5748
UNQ9433	148.1266	70.92929	44.79293	49.11912	40.54121	47.86767	59.62983	47.86624	59.08986
TULP2	42.0959	37.19398	33.17415	46.20547	34.76524	38.75664	37.97398	40.72065	40.24422
FLT3LG	33.08611	986.1058	648.5433	35.49681	593.0191	616.0537	610.8129	232.602	38.1237
HBM	43.36656	59.29625	41.72714	35.86891	38.54222	34.38318	37.44985	43.2947	41.9566
CYP2A6	35.55422	36.64668	38.33898	47.62091	37.13518	36.72336	41.86384	37.0508	41.91712
LOC123688	246.4607	144.2075	81.076	87.71895	74.96725	92.58318	67.64832	82.88011	216.507
LANCL3	43.11504	85.3363	37.55202	65.99759	316.8536	228.2372	42.42342	213.304	140.8322
FTHL2	110.1535	105.8815	63.45001	56.59023	62.98166	115.7262	74.95654	99.95351	192.8585
PTCRA	904.5825	1209.177	2763.338	595.4861	99.94748	1111.395	573.0493	781.45	1146.371
FREM2	282.1398	134.1401	250.95	147.2223	90.46687	121.3063	101.7	333.1186	283.2621
PIP	88.8938	75.40343	56.74786	86.83646	75.0347	66.45815	60.31857	60.87218	73.38165
FPR1	401.5445	234.3887	111.6703	271.5699	99.16789	164.0979	138.7163	130.9553	422.2945
TMEM92	83.30923	49.95626	55.14743	1630.342	233.9873	541.55	314.9238	180.8181	50.39669
LRP12	336.4782	112.1903	79.19683	142.7226	81.38013	120.5525	63.35785	82.86606	206.59
LOC392196	43.95838	39.75734	33.54604	34.5929	44.72106	40.87756	34.97719	37.40888	39.10694
HSP90B3P	819.1359	2004.104	334.3357	1268.677	145.4476	387.6247	339.4675	404.3796	1806.786
HSPE1	780.5502	578.119	358.8303	533.641	713.9358	491.1616	414.7282	612.6569	781.0042
TMEM109	957.2723	1170.163	514.5219	850.4708	290.9857	578.2238	365.1593	194.3056	62.65159
HPSE	54.91505	221.4825	103.8582	58.46934	108.4331	43.42254	60.95184	101.9936	38.24578
C20orf141	73.31255	81.86159	38.10497	56.42189	44.34761	61.43195	49.1101	73.01547	139.0796

ADAMTS4	36.1939	51.90675	114.6746	35.67551	50.04318	38.73338	61.53085	36.32927	52.08531
SSX6	69.07352	49.09326	41.64649	48.61912	48.95599	106.3855	36.86186	46.98161	76.95631
USP43	165.1251	302.8934	100.2795	228.4682	88.37264	134.5178	76.28513	86.22674	1393.849
HTR3D	52.32248	45.49185	39.67282	68.12647	30.61331	36.08039	41.92207	40.74609	43.53897
WBP1	1053.287	1429.842	1477.775	1213.773	1197.669	1326.454	1171.54	1447.146	1007.472
SHF	253.4282	105.1318	83.13765	155.8304	122.3354	130.5016	109.8881	159.968	207.3702
FOXD4L3	154.1465	76.28114	62.06384	65.35313	67.14442	87.90565	57.49855	76.07927	186.0048
GMFB	6632.686	7103.678	6411.969	6987.812	7455.924	7209.729	6273.97	7621.03	6384.16
THG1L	3180.885	1763.439	1740.275	65.93685	40.42134	1030.639	1606.843	1439.199	2544.986
ZNF571	68.25657	78.59579	49.69639	285.692	58.74052	62.74621	69.8402	39.73205	66.20049
IMPA2	1943.914	33.84844	943.5221	1551.701	2231.885	885.0934	1326.561	505.2251	36.62064
CHMP5	41.10533	35.76745	123.3966	44.5228	111.9167	34.82276	44.5885	37.59917	43.83298
AMY1B	132.239	89.52755	90.96969	166.4844	60.99024	79.57393	62.19204	75.92957	301.3081
ZDHHC1	11222.24	9829.26	5405.583	9714.745	14845.97	8452.755	9787.246	6948.206	13215.73
NUDT9P1	43.58469	445.48	33.89175	42.35164	40.08666	35.96228	44.36007	46.29495	40.44711
TRPC3	101.8733	62.89993	52.83862	62.11145	46.95238	60.07892	54.24627	39.35389	190.6772
LRRC8C	51.51902	38.22276	344.0191	313.4756	63.81935	163.4894	83.82193	78.82771	56.81624
PLGLB1	37.5351	42.96052	49.34824	43.54811	44.02631	35.5974	37.11536	41.55469	37.96179
TRIM60	52.32106	64.64818	53.10181	43.95265	42.10506	36.86772	36.76778	42.17265	68.15179
PRAMEF5	243.8874	175.4211	71.26991	127.2422	75.2906	95.79301	70.93016	93.52919	255.6749
TPT1	8421.995	8054.127	13252.19	11390.69	18610.9	10593.25	15800.85	11629.76	10467.92
VWA3B	207.6141	48.22029	49.56565	144.1381	53.61492	88.69756	54.0201	65.89696	77.07388
CT47A1	55.16457	53.09695	51.26302	51.27583	44.96614	49.29304	51.23236	65.5274	68.49044
PCDHB11	32.09383	32.12538	34.37628	33.86073	37.8682	33.72087	35.70539	44.72552	44.45612
SPTAN1	13456.5	12933.77	12520.71	13900.48	13915	12009.64	11732.75	12667.33	12989.37
ARGFXP2	51.56866	50.38758	58.99221	87.35162	51.84935	70.11742	40.68603	45.48859	44.65598
BMPR1A	69.32535	74.95284	68.10839	115.7619	65.42429	158.4529	85.32075	59.77279	47.17071
CDH16	102.5924	66.85896	303.0311	91.15228	242.0015	59.57926	841.3997	73.33533	226.6888
ANXA8	1680.961	447.2476	253.5817	1454.681	162.4479	197.5474	251.0242	91.49934	1344.612
FGF22	50.38254	95.5641	42.59851	44.68088	49.45026	75.85468	48.61889	53.09349	38.05091
ZNF678	72.12235	34.21343	45.42059	56.67361	58.41491	58.47406	47.43832	46.35446	49.90769
UTP14A	818.1448	408.0169	226.2346	473.0077	350.0753	826.0601	370.8719	175.9531	1653.797
MYST3	65.37726	813.5739	1715.606	51.3667	2759.078	472.0073	1445.854	1459.247	233.6221
P11	207.435	92.1503	121.3278	165.1413	149.8887	114.3352	65.64197	85.75064	134.4329
UHMK1	44.054	1055.685	568.101	733.7798	735.4767	316.4024	888.1626	274.2758	38.63614
LOC283392	259.7856	147.163	223.1617	905.2908	139.026	187.9466	103.8969	177.8554	63.74211
PLEKHA5	1537.697	1775.541	2279.213	1326.804	3230.164	3229.313	2808.814	1489.88	56.79497
HSP90B1	1773.765	2948.495	5065.672	3401.824	4774.845	4440.924	4902.147	2775.449	1440.742
AASDHPPT	39.7331	49.1431	74.7296	59.2043	90.58433	76.28078	74.85132	56.75249	63.44972
LOC260339	482.6878	307.6296	121.7911	279.6421	68.42958	144.134	76.12562	95.30209	781.9293
NIP30	78.2427	56.63234	41.25817	54.38211	50.14173	447.15	246.8438	41.05589	42.50859
COMMD4	103.962	872.6793	880.717	103.1768	2130.022	1101.486	1683.244	1378.331	159.878
TBC1D22A	48.38868	49.31205	124.6033	68.49794	86.9631	63.23609	44.50704	93.31519	71.97337
SNORA33	1083.021	2137.886	977.1083	1633.047	708.8496	994.9011	681.0608	956.2405	2087.683
CDY2B	193.0892	87.96871	87.22987	133.4586	78.9948	82.86875	58.13657	75.58656	175.4612
LOC401072	88.02403	92.1701	56.44118	102.5296	50.9739	62.25348	51.49471	47.04634	86.54482
MRPL51	996.1981	773.2667	464.9431	826.834	664.9753	618.6898	604.9923	947.6245	1102.955

NUDC	19273.99	19589.3	21077.35	22157.57	19734.37	21784.34	22526.16	22087.72	21766.7
TSFM	285.3679	170.3358	179.3158	204.3898	374.8652	133.4385	279.8656	206.0447	278.8474
FLJ10324	45.85672	49.07333	39.6326	48.0834	79.20917	50.16679	35.82013	38.68599	61.14462
C8orf33	4068.089	3054.007	3429.64	3746.48	3272.258	2744.377	3338.594	2836.374	3268.473
LOC65296‹	407.9711	196.9446	104.823	216.7437	216.5759	199.2498	166.0833	138.4728	519.9868
FAM10A4	60.66516	56.80525	99.97923	65.10704	56.20704	52.74113	48.26187	100.8577	103.6435
PMAIP1	1546.764	947.0189	2041.749	3483.244	842.9904	2749.723	2905.331	1304.143	1496.744
TTTY12	132.8503	37.93949	46.47681	64.69192	35.36406	35.52369	38.80452	42.52361	38.09162
C5orf39	6967.145	8397.431	6043.328	5698.45	4042.32	6349.977	7705.007	3486.291	6618.435
FADS3	238.1128	219.8602	189.2489	1274.348	251.5092	157.3701	683.5619	497.4179	401.6292
LOC44035(	880.8906	736.1906	255.2584	355.3019	109.9521	57.31563	222.733	145.2895	1194.629
THEM2	50.86021	51.47447	217.5103	46.74899	300.2748	342.0645	53.53409	215.7251	84.34412
LOC33879(	69.41671	63.33512	170.8999	51.28707	44.11914	43.08004	41.27396	48.49454	55.75912
MTERF	763.0723	409.1151	188.6356	480.2142	155.6234	244.3955	127.1135	162.6572	782.7535
MSTP9	33.34003	38.41373	32.48962	42.63089	130.6776	43.75217	36.91218	37.3622	40.03333
UNC84B	12674.09	11827.39	13428.9	12685.56	15715.31	16604.57	15851.8	12536.63	12451.87
C17orf108	1028.909	61.39505	144.5131	41.66404	32.68331	57.91058	47.86462	36.60743	49.36755
SRP9	164.6242	387.7427	547.9814	386.1834	969.8421	762.8828	1003.923	639.381	506.1754
SEMA3D	127.143	140.1059	143.4511	143.838	204.3911	168.6626	267.083	162.7641	38.23743
ATP6V1F	5759.636	6038.86	11864.37	7128.623	13796.53	9944.485	11131.09	11181.86	4898.263
TTTY19	93.45983	47.82705	44.06072	77.31831	55.24409	75.64541	52.19439	54.50932	72.72195
AGBL1	116.2876	49.789	51.9183	63.72135	38.0966	33.8043	46.06396	37.82137	104.1456
CCL4L1	828.6879	52.26486	728.4252	2684.174	145.7454	1222.323	563.4341	456.0093	1365.99
PCDHB16	335.2411	238.9365	97.8131	241.1358	91.73694	195.8339	105.4566	50.03968	124.7434
CCDC147	251.0012	176.5671	104.4245	252.8524	72.37751	124.5556	105.9463	69.10706	400.3385
HBB	2757.326	6353.878	1291.909	4199.12	4173.423	5023.169	3558.56	6371.726	1560.324
RFESD	200.7831	134.756	60.50914	95.05701	53.10914	69.99971	55.47559	56.03763	62.51552
CRYGD	78.25411	65.63596	31.77989	59.20528	56.735	44.23785	46.32358	63.52028	62.45528
MAP2K4	1684.515	3098.49	619.9022	1572.658	938.8765	543.5908	703.0645	1119.703	621.4916
TMEM9B	4907.148	4494.969	6255.5	5433.005	6483.007	6051.006	4859.019	5421.037	4208.882
ZNF528	146.0465	119.4355	49.45929	61.76735	57.22252	58.4149	43.84253	42.47372	42.12256
ADAM30	59.87766	60.48415	56.01341	85.80359	51.58481	48.70809	49.73856	66.8903	60.51381
C10orf129	142.6895	80.8731	76.01069	136.9418	88.29774	112.8561	65.75516	58.58435	239.7835
CTAG1B	241.0974	105.5981	52.01618	312.9648	40.23394	124.2152	50.49977	46.25023	345.8636
RGS18	264.3574	156.7105	146.4057	187.22	104.5287	210.5974	84.82356	139.4955	235.8651
OR11A1	75.69859	37.23459	50.87169	44.66395	48.0267	64.67921	43.38592	55.36872	37.34648
ANKRD24	377.6749	164.3446	68.4285	48.63887	49.3165	79.86226	60.94869	75.40182	161.3417
CCDC77	1360.264	1574.49	2280.098	2605.03	1840.143	2573.97	2248.547	2113.712	1078.147
ZNF625	128.55	274.6448	213.076	67.33884	120.5813	65.90421	80.98064	58.33138	127.6338
GGCT	2357.431	122.3926	1354.307	427.2493	934.9471	1229.44	967.0976	218.1712	111.4026
MBTD1	17329.13	18054.98	13479.85	14915.18	10770.6	11115.05	9063.585	12990.74	20340.82
C10orf84	2017.096	41.23665	354.4029	40.22879	626.7109	31.05886	490.3224	185.0458	51.40662
TUBB	1426.958	38.69582	5891.206	4800.833	11363.55	7451.956	11454.35	7580.869	1301.476
HNRPH1	8961.943	7984.411	6049.062	6714.302	4496.887	4947.672	3592.781	5285.588	9340.986
POLG	330.3228	162.8872	79.64307	190.7233	62.18593	124.7162	130.7233	98.14277	358.3017
ABCB8	431.7728	350.7975	186.6624	242.4449	70.00674	173.5704	86.39938	88.08262	753.4351
PGAM4	5917.36	4077.869	525.9988	5403.494	861.4432	1954.262	2189.253	2060.979	5802.914

UTP20	1393.373	1665.515	2317.64	2421.649	1755.554	711.3905	2479.334	1420.653	1376.993
GATA2	1055.863	934.0993	193.0352	410.4235	140.803	560.4679	288.8566	132.7132	674.1126
DEFA1B	41.89968	62.13776	56.11671	48.08488	47.27377	57.93629	50.77884	52.40801	48.78364
LOC28545З	252.8467	73.13211	63.51112	96.49623	73.00568	79.37127	67.50204	87.8071	202.4578
HDAC10	34.05852	47.19157	36.15284	47.16778	359.313	526.5696	142.5983	38.8455	47.05712
RBP5	422.5891	216.8403	112.4202	222.6654	132.3397	164.5103	115.8144	302.2692	551.7346
PDZD11	69.7608	104.7985	56.98856	125.1338	62.52317	80.2878	54.93835	65.38764	80.83975
TINP1	552.5219	35.25649	264.2257	447.3237	225.6172	38.44247	180.1326	213.101	36.80053
HMGB3L1	145.5639	58.75833	64.62583	60.11713	55.11054	57.85718	58.77949	34.63477	82.5406
ARHGAP27	705.2159	420.5934	124.6437	358.9621	122.6059	33.74755	104.1965	44.0298	51.06521
ZNF509	34.05574	52.21037	45.03771	38.15583	32.39602	46.51657	120.3757	40.8216	83.27048
ZNF234	280.1605	145.287	234.6637	158.0026	335.7174	125.1843	242.9897	400.1663	386.3562
GOLGA8B	6885	8648.909	7714.751	9381.688	5780.685	7504.094	5503.012	5915.687	5911.105
MORC2	37.91728	39.68234	44.41587	53.37292	39.4841	43.21835	105.5826	35.76885	2041.302
OR4F21	62.87848	72.10519	57.35594	59.83403	35.68184	49.34743	124.3523	47.91192	37.73882
PSG5	51.84664	32.63388	62.04932	682.6978	49.24525	40.64752	226.1295	58.798	35.59478
MRPL15	782.4189	990.405	1104.253	1596.323	1201.158	1567.847	1365.051	1213.196	851.1424
C6orf156	38.34218	34.70214	38.9288	65.0352	37.03929	39.68654	40.41555	35.94934	53.96571
OR10H4	40.22732	70.41267	34.31724	34.88583	42.1905	49.53342	38.1758	46.28142	51.93542
PIP5KL1	82.5393	285.9132	60.0847	86.51743	66.1574	49.37383	64.08126	115.1011	123.0752
AIP	8485.163	7159.154	7282.506	7548.829	8269.923	9451.298	9204.813	5445.149	4877.362
LRFN3	3912.376	4841.203	3645.945	3002.163	4422.381	4336.447	5569.752	5666.567	3635.939
FGD5	46.02558	894.3758	659.3202	45.88783	1281.934	1222.008	978.9824	1425.09	1041.792
RPL29P2	381.6302	207.7135	123.7185	255.9687	120.2823	166.5494	116.1776	114.1622	501.0618
GAGE8	38.95456	50.5471	43.95879	44.19954	36.87721	50.55241	39.17415	41.23206	47.42898
DEFB124	54.42901	252.4651	87.68467	106.2152	213.2806	70.70905	199.6833	276.2801	160.602
PRAMEF7	81.70037	60.82463	45.12807	44.97084	37.31556	106.9865	35.17119	54.80737	91.00866
ACP2	2254.383	143.2511	538.504	570.5763	514.0568	167.8581	796.7191	461.6798	59.32256
C21orf70	2029.124	2042.978	3490.391	2419.327	2499.609	2789.963	3062.41	4407.058	55.87902
ZNF443	34.99151	53.40339	842.3552	1666.461	422.1293	563.8911	625.6321	279.0659	1641.244
SAMD8	86.48631	90.2657	474.3996	361.093	644.5729	582.642	635.5856	324.4074	77.63373
PTGR2	236.7767	96.64735	71.1479	53.17965	55.69207	202.0661	47.41077	38.87154	46.56769
ZNF517	37.13996	50.33884	35.62852	39.59054	41.62014	36.97105	40.83292	44.40619	67.63353
IGJ	113.995	1267.351	1092.777	81.24367	2709.868	1298.798	1324.294	648.9684	1352.73
FZD10	304.9445	160.5145	85.4363	173.5357	95.14048	109.9718	70.03011	91.68521	329.7159
TMEM220	100.1587	1028.008	191.0857	916.259	879.8427	60.08604	377.1377	408.8317	63.89982
CCL3L3	39.41709	40.59944	43.35445	757.713	37.4964	442.104	365.5493	38.5214	61.96681
SLC6A3	125.7343	49.83294	58.91233	72.26591	76.21767	86.5531	56.19358	54.73341	169.3994
C12orf32	314.3727	1794.331	1556.215	797.4406	1072.546	809.7369	783.5568	871.1053	1811.135
KIF12	40.14221	46.18177	188.6873	34.13266	37.94139	54.44835	107.9303	36.29183	37.43402
SLC35A4	134.5443	51.36665	1188.889	814.1855	2621.87	739.5905	2043.451	2208.388	114.2138
XRCC5	67.52452	74.77911	46.67468	38.96461	233.7587	57.85881	98.37116	35.84437	53.64683
FAM127C	98.38832	41.77585	523.8044	50.40068	290.4811	56.52061	50.68568	44.63066	33.75692
KIAA2026	37.59912	511.9019	173.9395	48.11082	278.8643	337.5052	265.4743	297.9672	85.02637
H3F3A	649.6528	301.0396	842.2681	58.39199	2041.677	575.5463	1425.639	1055.421	248.068
DENND2C	96.7361	91.97889	72.86441	108.058	69.70512	90.93279	49.10112	45.95608	48.82528
BLZF1	49.07501	54.56889	61.40049	66.17	50.2352	67.2741	45.11773	41.82779	64.02952

NCF1B	192.2881	74.29824	63.71764	94.22137	58.64732	79.57023	55.2807	63.95285	71.57512
CHES1	3334.072	2224.603	5486.899	2464.138	9948.373	5389.587	8009.793	6178.975	59.57042
FLJ46347	39.16569	45.00157	34.25314	84.95601	34.60117	49.17368	45.57619	47.34426	412.9639
TXLNB	69.37629	66.17863	248.7789	48.88645	50.24776	224.9539	220.427	283.6506	1204.552
SNORD116	38.07304	238.4021	61.56065	39.85266	44.27718	41.31062	49.51127	38.94754	44.77385
OR1E2	36.84107	33.59484	35.52543	42.37278	33.49188	40.74269	33.47194	46.94885	34.2757
ZNF285A	50.75975	424.5292	246.2981	69.81992	182.3211	38.75425	70.16463	148.8652	49.38123
KRTAP3-2	43.57734	49.783	34.72566	52.00148	37.71445	46.26303	48.96724	45.83401	44.27786
TAS1R3	39.50566	38.60284	33.93788	32.50256	36.42397	37.40081	32.55305	36.78291	39.56996
LOC284441	125.33	52.81715	90.26364	216.9792	50.67711	73.81656	66.56188	37.89135	61.03339
THSD7B	68.48428	43.52034	48.06166	44.9264	45.9203	43.68238	36.18877	56.87078	67.82455
EEF1A2	20306.08	21599.53	14641.1	15577.19	11484.77	16470.28	16210.31	21774.48	23338.25
HIST1H2BA	40.59788	40.40701	33.7824	35.90361	34.36833	43.88347	38.22104	45.83183	51.55179
TYRO3P	44.67034	35.32345	149.1243	35.69899	33.03084	41.1314	83.85569	47.9957	36.05799
CLLU1	105.0664	62.06398	46.04464	41.66603	58.38891	70.25874	54.73658	42.71575	101.7336
FNBP4	1460.706	35.22853	508.0356	218.4722	1384.24	1062.787	1571.976	728.9139	2105.476
RANBP1	5637.901	6093.726	3774.277	7365.979	3995.917	3622.444	4568.757	4858.66	5750.441
EPB41L3	4990.925	772.8354	3356.817	1107.196	1807.714	2387.573	1532.579	1238.794	3391.505
SGTB	51.34423	63.31326	47.17874	99.42062	44.52081	48.67157	95.17537	33.43459	146.1085
CALCB	84.92924	84.37651	44.0423	58.63029	145.8741	39.07194	56.86921	47.44207	60.4744
NAPSB	39.98969	33.95609	744.8046	43.91753	414.8898	554.5854	794.7605	882.4041	32.19592
GZMB	5079.385	5375.943	12080.9	3782.046	4366.935	6947.066	8269.547	10113.91	5795.099
CSNK2A1P	82.28304	1175.338	188.2873	1162.728	145.0349	106.2266	197.6668	511.1032	57.83044
FLJ32679	31.34529	32.90847	215.7702	994.6706	70.82942	40.73151	548.4391	87.11586	37.3801
GOLGA6	66.75694	54.19874	49.14034	324.6275	50.6445	55.03248	53.74792	97.47977	60.53122
ZNF124	580.5848	333.7957	377.6736	448.1746	162.058	487.9216	147.4664	132.1001	936.0714
CFC1	38.05223	42.38077	38.39861	51.01941	43.02335	46.89333	44.06279	29.02319	42.5227
FGF10	80.4893	299.9485	117.1329	81.34534	215.9882	45.40126	92.45168	175.3917	167.6924
NPM3	778.4527	776.3028	2235.833	1905.427	3120.052	2105.394	3682.187	3452.847	1383.368
OLFM2	347.9729	173.6877	83.96548	787.8167	92.52737	119.464	75.83188	86.19324	414.3949
UFC1	1542.955	78.23359	2737.757	837.3514	2793.197	995.9979	1598.78	1500.398	102.1973
NKPD1	42.23561	37.362	51.63835	34.45808	39.0192	45.31743	35.61986	38.5016	36.64724
RPL26L1	47.81118	744.7573	169.8078	843.6866	396.9377	43.80033	567.3904	921.3336	73.72291
IFNA8	40.50787	44.71453	57.74206	60.68829	43.71467	40.51153	37.36834	34.33558	51.4216
LOC441743	33.99987	55.1657	58.83834	327.6028	53.95349	36.1339	71.91101	41.67597	33.29385
EBF2	43.24457	44.7176	38.18143	40.9643	40.05145	37.79147	36.69609	42.51217	76.40097
NME2P1	78.3372	82.99709	61.34187	65.284	59.18526	77.30109	58.25177	67.3611	117.7545
BZRAP1	280.3356	183.3417	164.512	169.0524	221.5647	136.5947	162.2426	158.0015	372.9213
NUDT10	483.0097	223.906	185.7685	260.439	279.545	323.5101	141.3379	96.57055	580.1652
LYZL1	49.87031	40.20152	54.81112	44.24012	49.89956	37.38694	38.4567	33.72501	164.6842
ATXN8OS	50.51111	85.11579	35.81157	45.60677	42.341	38.93752	46.2719	50.3835	42.67693
MAN2C1	38.40212	382.4688	35.09804	435.9081	189.3931	37.05368	236.305	32.88678	61.06857
SERINC2	43.95331	36.34301	884.5535	2912.745	45.58751	1095.432	662.6001	462.5359	35.96802
DNLZ	3369.491	4073.855	2965.497	3056.349	2858.055	3626.185	3403.017	4154.436	2929.362
DRAP1	7036.762	8794.525	9888.798	12297.74	10386.86	9747.394	10754.2	9882.066	7791.286
CD300LF	10956.73	8116.279	8399.647	4842.813	1866.398	7719.249	5355.879	5605.485	7735.238
PGAM1	724.4174	303.7763	259.782	559.2889	131.4907	225.2546	244.0639	370.8872	789.3307

MAD2L1	883.898	1630.331	423.9698	2476.362	402.5675	627.6619	355.6804	320.9069	1607.545
PAQR5	41.64849	54.01081	30.63371	44.01906	44.39445	49.10451	36.39234	41.2995	48.50844
DAP	4046.579	1016.884	288.8734	598.5544	796.0566	66.85677	814.0876	457.8745	1617.001
PRAMEF1	34.41491	50.4463	31.83072	44.37476	34.92359	39.14341	36.12951	40.2238	43.80236
FCGR3A	4289.485	3024.148	1564.308	2562.375	172.6466	1135.201	735.6064	976.1993	3554.769
C16orf80	734.194	247.8615	284.2782	469.5954	626.97	510.6668	472.7833	352.5418	548.9346
SVEP1	36.30876	436.6805	49.73091	346.9848	580.3765	293.234	512.174	427.8462	328.7421
FKSG30	49.709	52.40806	32.00611	35.57118	45.53664	38.89762	35.46014	56.54754	43.07232
P2RX6P	107.8604	107.3244	72.78776	161.1551	60.76031	80.67674	108.4669	62.65386	253.0158
HSDL1	871.7314	2293.312	1170.366	906.3069	631.8641	1367.349	653.4096	539.1602	2694.444
RPL36A	35.39909	48.04464	33.10186	33.03347	64.74834	46.58122	84.75552	45.89017	39.17129
ANKAR	6585.271	6065.075	2856.127	2787.53	2187.803	3708.564	2104.133	2374.103	6950.669
DRD4	51.42161	69.06197	1104.605	66.6174	189.6316	50.767	422.5951	72.83289	54.52402
KRTAP6-1	89.94013	62.26002	53.87511	39.55698	55.47743	50.34089	43.67275	46.31596	39.39447
NCF1C	349.8361	158.9492	124.6786	993.7105	200.0077	195.324	113.9623	173.8754	436.9334
C17orf39	471.4416	379.0244	357.9594	468.471	348.3409	731.132	437.3401	187.6024	58.1359
PHKG1	243.0792	149.4274	84.71222	162.2444	78.69283	123.3749	59.28045	55.17134	99.26415
RNASE3	33.40898	40.51044	34.45564	32.06775	32.37173	34.93689	34.37217	40.4429	34.79253
LGALS9B	2342.84	348.158	304.453	677.4738	219.6567	355.3977	286.2138	167.1003	69.97826
CRYGC	66.38551	57.79953	52.18475	56.91843	84.34897	63.91448	48.78832	35.86834	133.4904
GSTA1	58.84078	95.33273	52.79824	56.94908	204.8629	59.38878	45.85303	96.81534	184.1007
PCYOX1	281.2984	471.9859	148.8621	745.7853	277.2734	104.8463	88.73201	551.3459	302.7836
CCL27	84.00381	65.14951	212.4195	73.92719	58.60849	58.83608	95.9519	56.67098	64.03939
KRT39	49.3199	35.70897	43.38319	55.11415	44.13618	35.65995	35.86915	50.18594	59.75617
C1orf104	356.1231	148.8221	75.26022	178.151	95.23435	143.3237	78.7781	93.92249	478.2035
C14orf166I	103.8669	40.63121	50.68263	47.15468	36.46719	50.96172	40.09132	40.5778	51.99437
NOD1	1313.895	675.092	1345.843	72.24416	1566.234	366.4431	1517.556	569.5111	882.9446
GBP5	800.3434	1429.032	2185.583	79.73862	694.5547	1437.211	2230.633	815.6665	112.8244
SIAH3	268.3963	58.4493	64.83097	80.61008	89.00438	84.70678	59.96064	47.19822	131.7155
SLPI	6310.292	4178.327	3923.785	5914.313	6614.915	4841.141	3918.996	6381.357	5467.313
FLJ16124	42.9325	46.92372	42.20931	40.69518	48.64421	33.12776	37.23207	41.135	71.12457
FGD2	11490.34	11322.85	14244.37	6805.002	5907.557	14414.52	10927.2	11056.33	12259.1
OR1D4	103.1492	96.16507	69.8233	70.80067	56.29449	92.63393	60.5321	80.61909	50.24068
CTGF	578.5374	337.4024	348.4849	315.7188	994.7565	657.6313	555.3453	630.3159	660.6546
MSMP	48.93837	67.7009	54.22306	84.25868	39.87352	49.50102	46.96255	52.27391	56.35057
GNB3	36.50166	53.25517	59.02897	48.45492	47.94685	36.22853	41.11097	39.56067	34.32129
HIST2H4A	192.7124	104.1712	131.4515	344.3566	143.507	120.7699	134.6464	198.6182	303.8391
TNXA	301.1675	158.97	86.76696	198.3692	77.37147	111.3295	125.099	219.6749	265.572
SLC25A16	1074.932	1557.743	947.3766	2195.386	560.8189	1057.747	587.0013	811.5084	990.4856
RAB32	52.37908	55.58663	221.8778	51.48463	824.9955	365.5601	618.3594	52.00087	36.73357
LOC28503:	78.82747	84.54454	45.23618	43.91575	42.27387	51.68715	48.64798	39.86126	53.52227
NBPF20	3584.972	2124.118	3375.239	1056.229	2185.852	1100.136	1554.978	2215.552	1754.31
GPR112	359.6039	179.5525	99.64452	221.6534	91.76799	141.8421	97.51224	119.4466	429.3849
CD320	35.02305	34.05671	725.4353	36.94937	1082.997	50.006	1116.902	799.0796	39.74761
SEMA4C	358.1579	219.3277	78.69717	70.24387	118.6972	71.0693	100.5036	66.90112	178.1992
LRIT2	60.94316	54.24119	35.09422	34.78432	31.41055	32.94849	30.22515	31.94053	41.77656
SLC16A11	40.86598	30.3081	31.28198	47.108	40.06698	37.77983	35.01541	42.16295	47.35452

DUX5	99.40482	43.71949	42.69227	44.10981	72.67841	38.04232	35.07077	55.52832	36.86853
ESPNL	1854.041	1612.172	1668.653	567.3833	599.9987	573.6324	1016.972	275.7188	2638.573
ZNF394	89.11479	95.16908	52.00811	81.36658	45.6297	43.48261	38.03411	42.88874	38.02179
GSDM1	1356.291	3934.713	1028.826	1509.833	2160.598	1177.765	1634.843	3228.245	1397.938
TNNT1	272.6183	167.3186	81.38737	183.7941	103.5627	138.6564	89.09479	110.9967	320.988
TRUB2	786.9894	541.1426	280.3215	435.7819	250.1602	418.2761	276.8188	481.7123	937.3371
NPEPPS	1919.069	1701.748	1427.396	1785.876	2211.664	1387.907	1334.176	1507.813	2753.538
ZNF808	708.3373	369.0938	287.1134	286.6187	150.4822	130.9759	158.5191	146.5605	938.9144
RPL12	2388.412	2094.232	787.2396	2949.376	697.5552	1235.913	1140.134	500.5413	5106.402
LSM5	430.8716	325.3119	274.1418	363.1675	214.8517	308.0981	382.1243	294.8973	403.4073
GPR89A	100.7668	132.1019	61.90654	127.7259	52.18147	61.0643	43.11506	66.58483	93.00463
TFDP2	144.8447	178.4987	105.0155	188.2379	67.07099	130.9167	63.87794	75.80637	443.1925
SLC39A1	1383.446	806.3876	1742.565	1987.241	1447.075	756.2686	2093.589	1255.708	1351.948
RGPD1	38.30593	1045.067	256.0409	48.81425	445.8177	322.0106	136.0524	169.0407	40.55177
PIK3C2B	58.19274	600.6648	982.6149	548.4391	1005.828	287.3614	734.3393	760.0737	150.2675
NDUFB2	429.8055	262.5427	1957.762	368.0773	4293.389	894.0624	3071.945	3294.951	543.2364
DPPA3	41.20567	47.03765	32.62991	39.34411	41.91376	42.44095	41.48487	41.00431	35.04173
PDE4C	50.20037	1944.381	34.40015	968.8244	583.316	551.8282	539.0573	182.1887	34.96398
PLXNC1	62.35648	33.65025	113.7755	34.48621	36.5731	58.48246	39.34739	34.77605	57.89283
MGAT4C	33.8665	90.87872	41.45243	126.7086	47.48526	34.2931	49.59036	38.06486	43.44396
DUXAP3	2470.863	3739.362	3315.325	2391.224	1725.23	1670.394	1908.161	2493.062	2245.324
CSPG4LYP2	37.05006	35.57766	38.25352	44.86217	42.89473	38.7376	33.17562	40.94008	35.26056
ITLN2	65.2898	743.9501	393.9241	90.99461	257.4978	814.6195	207.9951	389.9295	2066.355
SPAG11A	57.76995	39.00282	38.31861	41.50314	49.89175	40.29535	34.15759	35.70495	34.55406
FAM120C	59.64739	60.49482	37.82261	40.33635	34.07853	53.73223	37.25667	29.3583	41.31642
CDCP2	67.69883	60.31071	61.04887	85.74912	49.31489	68.18164	65.94063	55.61049	156.9044
ERV3	57.03161	939.4778	158.676	57.17085	36.83462	907.1872	411.4824	277.2838	70.58488
ASAH2B	129.4053	867.6856	67.9618	69.04394	60.76005	82.02981	57.7112	74.85983	101.1421
RAB1A	452.0194	553.1842	856.2415	1141.549	1893.644	1014.757	1298.329	1174.255	91.50102
KIFAP3	1007.461	1197.204	1503.655	1286.431	3661.198	3166.569	2980.69	2395.717	37.88844
SAMD4A	60.72023	839.6158	166.6455	68.18896	189.3918	35.85165	165.9481	298.4961	104.2828
HIST1H4F	1333.477	260.3773	229.2474	36.66634	357.467	36.26161	37.15255	174.1852	836.9816
LOC143543	169.118	61.02213	46.45499	38.70556	56.12076	60.73161	50.61599	51.97739	146.3971
RIPK1	84.04802	64.47504	749.5411	489.4969	1165.847	183.9857	1010.006	762.5627	59.0275
TRY6	98.71383	76.31785	61.80325	44.12173	67.24483	83.35295	51.33369	51.25258	276.3042
CYP2R1	94.88384	64.83722	38.25583	53.75212	220.174	43.19699	43.77884	646.6414	58.82883
ANKRD34A	439.9201	233.1451	174.0231	246.0294	179.4059	245.3408	135.4237	178.0429	522.4296
NDUFB3	1658.93	1392.592	970.8913	1304.075	963.6547	1149.408	790.9137	1159.079	1718.696
FLJ13305	720.3353	603.495	382.7181	974.5781	403.9428	779.2039	563.8467	534.2657	717.2971
AGAP7	281.4811	186.3779	122.0447	301.3454	109.2475	177.8821	106.3062	129.1638	691.9007
RCAN2	2656.245	3445.242	2520.162	1354.227	3731.988	2080.786	2888.901	3121.957	1889.262
DEFB110	69.85471	68.95329	32.90751	63.26485	57.19164	70.63562	51.55194	51.6349	57.4152
RUVBL2	588.87	943.6397	412.1543	793.0662	419.9811	430.4101	430.2107	565.7993	831.4073
LOC729164	169.8972	134.4794	69.43327	99.44718	61.96745	82.43475	61.99751	68.03412	208.5113
WFDC10A	44.46069	42.03175	47.50422	48.643	31.80642	34.00182	61.90857	43.19784	39.85791
FLJ16331	51.95386	49.69142	35.65822	46.52568	35.7507	46.65227	41.47173	34.96374	46.18274
ZMYND17	529.2969	83.95785	128.785	121.1303	186.9479	175.9756	119.2897	198.9238	666.9039

HTATSF1	710.5165	552.2875	385.5794	584.2704	313.7059	448.6551	346.5654	560.4891	570.128
ZIC4	61.1418	86.99417	45.60403	69.13614	51.10975	54.33931	50.79657	39.8933	72.06751
TPRXL	35.40526	36.43849	44.4149	35.21557	41.75003	34.69517	39.13422	40.65463	60.75367
NPAT	37.44375	722.1967	332.0947	41.68826	392.6518	51.9765	229.3783	341.5063	2193.579
FAM125A	3485.275	4052.516	3488.253	3736.171	2959.097	3476.282	3489.297	3411.848	4100.117
HBEGF	297.5234	304.5451	629.6491	900.9355	1102.09	765.954	513.4772	832.3195	3716.725
GPR137B	483.9513	1870.66	2645.45	1657.171	1469.766	2919.282	2365.36	1626.153	636.1228
OC90	45.62838	45.92818	49.03296	60.90424	52.04192	46.74939	45.97485	48.43306	41.22501
OR2B3P	108.0412	107.5896	79.1667	155.9654	67.68802	92.10609	58.89228	68.98814	413.067
ZNF639	164.3837	129.2096	61.85809	68.34538	48.71618	69.49659	57.08177	49.15759	74.89195
HCG3	139.3329	112.0442	54.55518	98.26958	70.44002	59.86633	49.44483	75.37646	137.0528
FLJ38377	50.81711	67.715	63.53076	75.07754	54.505	62.9307	50.64964	44.00072	222.903
C12orf31	33.77213	37.01112	54.16619	184.5058	53.26752	34.19812	78.64941	60.78351	34.54481
HMGCL	4456.031	6542.511	9519.746	5171.228	8426.605	8596.873	10040.47	11949.52	2980.729
TTC22	84.73499	492.2059	245.4542	46.03038	47.62567	450.4579	45.12799	273.1475	85.36223
FAM83G	866.2654	559.9911	445.4115	1072.517	95.27122	206.6089	93.66548	43.12411	1114.959
CISD1	1324.408	438.8558	1443.969	1346.495	1221.119	96.86728	1482.542	1706.519	67.4801
MFSD8	47.26998	77.84849	594.7239	46.16684	890.2658	417.8195	1007.454	674.2018	105.8074
IL26	43.62482	36.56037	43.99827	70.00844	37.26653	64.95638	55.11423	41.69159	51.09532
LRAP	367.0684	242.7488	147.2608	196.7547	76.64188	101.7141	77.54346	75.59595	411.0019
OR4K5	60.27414	48.55388	70.41972	46.98634	47.21079	42.73045	43.69758	45.57004	43.49643
TMEM4	181.5996	167.4044	89.03487	141.123	95.48618	116.5025	91.4054	98.53649	275.9528
BMP8B	1863.251	3586.89	1120.213	2115.966	422.3665	259.0222	536.777	698.749	3555.871
FLJ43093	44.97671	42.37739	304.3778	423.5188	295.5058	256.0513	263.8316	296.6992	37.97879
LOC401387	65.44257	40.64579	38.52456	44.76613	37.27741	38.27411	38.2397	49.62757	111.2655
SYCP3	106.2901	118.5138	69.00851	68.77893	64.73084	64.71549	56.44687	58.42888	194.341
OR8B12	449.6634	258.9092	124.085	229.163	118.9373	214.027	142.1024	139.3377	525.4361
FAM73A	51.33505	43.48047	47.7735	44.95213	129.5426	52.55879	42.46667	59.66676	51.24471
FLJ43763	68.51978	47.69489	45.06284	49.56226	41.79094	66.22983	35.71883	42.82438	61.67865
LOC651503	68.29034	65.79428	50.57243	86.53693	58.43463	58.90214	46.26552	85.67084	184.9256
TBC1D22B	50.31934	49.54264	199.3638	742.1158	551.1749	48.42591	532.4313	42.19365	61.40895
FCER1G	6339.39	5802.253	4050.311	3100.145	903.9221	2794.534	1735.495	1940.119	4256.976
PPP2R5B	201.1962	644.0869	1363.417	156.8851	1926.658	636.9236	1967.564	803.0131	348.2874
CLLU1OS	144.2267	106.4724	92.69803	175.5841	77.75434	114.8326	71.55403	91.0274	85.73876
MUM1L1	95.66343	90.95507	45.79245	106.2917	45.84734	54.19804	48.52503	151.1541	262.5676
LEAP2	70.29604	992.7763	138.7169	451.5801	510.5374	720.5676	385.7903	294.6538	57.7905
RGL2	354.6416	118.9725	1258.541	217.5422	1799.692	428.8558	1565.416	726.3811	524.0215
KLK9	33.52653	490.1273	32.85361	33.59035	206.0804	46.76141	37.8446	393.1706	45.77435
SAGE1	38.64307	36.06448	40.56398	55.74696	40.42603	46.8106	33.30122	37.1763	34.91061
SLC36A1	2346.811	1483.466	499.7198	942.023	343.3263	764.8967	611.8057	586.6364	2824.253
METTL14	262.2069	236.9672	142.0368	367.161	88.30801	214.3944	86.93581	147.9035	358.7957
RPL23AP13	54.98385	69.28239	155.4673	91.20652	262.0523	72.90726	189.7817	70.55529	71.88251
YBX1	1819.882	2327.318	2375.984	3462.554	3654.088	2165.005	4152.688	3366.794	4527.494
dJ222E13.2	38.08584	582.2421	228.2318	724.0025	38.14097	774.1898	171.9453	251.3256	2219.996
ZNF34	49.45313	1142.763	38.40377	51.07716	41.02882	37.37224	42.27146	197.7975	52.22022
RBMX	1590.442	3989.829	3468.938	4267.739	2025.89	2970.946	2097.951	3281.547	2356.948
SKAP2	1848.269	1437.666	867.0329	2112.301	467.018	932.8729	700.5375	580.1908	1498.469

TIGIT	228.5628	137.8864	280.1456	175.5327	116.6453	141.2764	139.1481	118.1981	230.9908
TMEM196	43.66708	42.5691	34.04751	45.65278	41.9837	33.96756	43.31074	36.07389	50.39987
MUC16	234.547	133.4018	896.0092	109.5965	71.91265	1966.189	2635.309	757.3173	366.4238
TRIM49	47.39729	56.55522	49.87763	46.13509	33.8684	43.63367	48.84698	47.17365	56.68506
MOBK1B	314.8212	141.1391	226.2766	326.0112	156.0921	160.5585	110.7856	154.0327	446.3656
LOC401252	598.1908	680.2757	395.6469	558.9064	314.6342	299.3205	81.73829	281.568	837.9418
SMEK3P	145.9701	114.9247	337.3595	117.7917	58.07802	76.09825	57.28691	67.17612	155.4657
LOC728643	745.883	572.0914	442.4347	872.2377	177.3215	473.3007	286.3438	483.6366	198.4252
ZMYND10	39.56672	32.84659	34.77961	38.98142	149.8984	33.71054	44.08092	47.03121	48.04489
KRTAP10-9	79.15009	89.37245	54.80624	90.73891	63.25814	64.49849	51.16122	60.27372	91.88103
ACTA1	4806.258	13433	9666.005	5617.041	9259.603	14860.87	23381.91	23905.34	6527.659
SMG5	2540.19	3661.414	4037.773	3241.32	3206.876	2736.815	3292.253	5040.842	2894.657
SEC16A	44.04715	33.27249	230.6858	57.22768	237.7919	104.9127	521.1977	325.5236	32.31791
psiTPTE22	35.22036	33.94961	35.00697	51.44381	35.08558	40.75324	45.64125	36.62849	55.03211
ZNF480	69.66338	272.7914	344.2834	326.8127	452.2248	491.4832	500.9931	560.7682	190.2669
LMNB1	53.66001	665.0728	1732.964	511.025	465.7325	280.1123	358.0569	1037.658	581.3789
SEC24A	189.8985	62.32954	121.5684	165.0594	85.57655	91.16422	88.7617	109.6631	94.30396
NOMO1	162.1595	91.29916	148.2304	477.3054	318.3835	103.9677	122.2187	357.0207	150.3076
TRPC2	38.07712	46.89269	42.85523	50.40678	50.96404	45.8319	46.80667	50.19494	30.19807
F2RL3	97.42957	102.4022	254.1821	386.1389	95.6604	202.3394	155.118	146.3435	357.3372
G6PC3	1624.928	751.3271	592.9949	42.40807	493.7916	48.62333	331.0951	763.8823	48.23341
LRP3	18473.54	15308.59	11688.43	10526.12	9342.139	11786.71	11958.26	9613.884	16188.34
GAGE5	33.92316	33.46572	10204.92	38.49113	87.70966	192.1773	99.60681	169.8912	80.24068
HBA2	24054.82	26107.46	9023.572	22781.5	14036.61	17855.37	14313.68	22775.55	21425.66
ZNF100	1246.186	939.9225	356.4592	149.5245	138.7887	344.7553	288.2811	219.3353	638.6605
NAT12	449.969	342.7271	235.2676	357.3447	234.6003	250.9681	197.8203	161.3328	676.5025
SIPA1L1	603.6661	333.1221	308.6986	358.349	234.9686	188.3753	271.9995	333.4598	583.0904
APIP	144.3865	68.46524	41.85765	69.93938	66.53359	93.41895	60.91402	84.42645	50.99632
LYPLA2P1	381.0086	217.219	121.6188	225.5139	133.3987	180.1571	99.55174	129.6632	470.9297
UQCRB	80.50716	275.7532	115.3898	55.32831	241.4364	441.0855	169.3543	221.8721	48.08566
LOC285359	1527.165	640.5557	596.6168	1135.026	365.9242	508.1862	392.7401	684.6314	1197.634
FOXJ3	46.61584	38.05024	3738.91	2597.543	5979.604	3916.955	5395.274	4075.689	40.71018
SPACA5	39.79787	33.93846	58.65716	37.08012	43.62279	68.07582	39.61937	36.76305	42.1742
CDR2L	10592.26	8781.057	8212.357	15268.22	13268.94	13195.26	13456.84	9628.278	9352.118
FAT2	61.64813	36.77036	117.7789	48.8625	47.21294	455.0466	141.0533	186.5191	32.42055
C11orf1	47.59772	667.5912	447.9946	393.321	1433.316	1537.092	1200.536	780.5026	2509.707
FLJ40453	399.7894	274.662	128.0279	261.0191	95.53342	121.9875	112.6974	132.1416	604.1171
LRRC32	8759.192	6946.389	7453.763	7018.737	6189.94	7409.885	7301.881	5734.306	7686.745
IFNA14	37.81359	51.28368	49.01752	36.93452	39.65653	48.88955	43.50628	39.22269	106.1636
TMEM47	650.6583	537.1656	487.0085	333.1295	818.4741	680.8412	645.9619	539.2742	632.8152
KRT33A	76.45527	49.27755	37.30186	74.7007	51.62669	56.95483	51.73133	50.50575	99.95058
TSSC1	192.0731	103.7071	562.0756	151.545	513.924	93.04793	261.5056	430.9674	1616.192
FAM190B	33.75519	36.41532	40.62119	33.54565	40.62148	36.18429	39.12609	48.69436	38.12969
CYBASC3	187.0453	121.0376	668.4031	916.5295	411.7825	71.54612	404.4499	491.9037	861.3746
LDLRAD3	39.83382	36.09182	406.8434	383.813	75.06079	36.99788	33.00876	35.86588	32.51501
F3	61.89809	102.1245	1165.731	2915.346	729.1354	1250.953	3413.277	622.5457	658.6447
OR4A15	244.8466	193.5176	94.43779	201.3397	80.34425	117.5177	70.84988	102.1597	322.6218

RAB27B	128.0115	104.079	80.71455	109.1844	97.26059	99.24441	84.77806	96.93613	156.4693
OR2L8	38.29469	41.32589	35.47229	48.75693	41.18678	57.8867	36.89824	45.68198	32.25028
TGFB1	63.10292	39.42183	45.27882	57.79395	46.87041	52.35045	50.2484	44.14512	55.2365
LOC441601	77.34077	46.10893	52.80504	51.94733	39.14497	45.62872	38.76986	41.89991	42.33183
OR12D3	120.4841	99.69791	60.68369	103.3753	61.84759	73.37642	57.79235	84.46535	150.7721
PARP11	62.2181	46.43104	56.15879	715.4449	59.89755	43.51584	40.37247	50.24332	39.05194
EXT1	134.0331	254.4637	334.4009	1062.342	359.8818	521.9246	565.3044	229.0994	75.62835
CDKN2AIPN	848.8523	827.291	589.0186	457.652	250.1149	337.9327	305.975	305.4614	1212.83
OR2L1P	45.92996	70.29685	43.46532	53.58992	36.53126	77.83965	50.7522	32.1788	70.73674
RPL13L	38.10786	139.9989	182.2287	46.30003	329.2124	44.14968	75.88084	96.18143	57.83579
PAK3	59.84135	34.99371	35.35185	41.51907	42.91174	40.81933	54.20515	45.03628	46.61924
PEX26	44.83261	628.6036	237.8868	288.9599	141.3675	260.6617	175.7028	544.9482	44.08608
KDELR1	649.5002	550.5385	696.3731	912.9193	626.1271	686.7787	815.7297	1120.653	890.3942
HLA-H	43.34216	38.66675	42.05478	46.05881	41.8739	58.70675	79.68802	40.96095	38.96559
LOC390688	94.66967	102.2162	50.86361	125.9904	54.53807	54.73632	57.63392	66.58715	46.77272
FAM76B	39.50491	52.14972	71.13862	43.31067	57.16802	37.84229	68.23467	33.51892	35.60354
UTP11L	2420.282	3124.973	2927.26	3517.339	1500.155	2900.027	1624.145	3133.872	1588.377
AMY2A	62.62918	59.87156	45.40115	43.23065	35.11448	48.47005	37.23444	51.7616	134.3472
PRED57	79.87637	102.8321	62.84497	102.0836	61.93633	67.88203	53.51221	49.38913	57.96996
ALG1L	9607.268	21914.93	5934.091	14209.76	16126.31	13380.21	10068.42	7138.443	11895.89
TMEM194	49.89145	54.9408	52.10526	49.36314	39.73396	38.81851	36.87255	55.28719	70.15764
PDE3B	78.85482	461.5825	238.6174	49.48407	49.98005	42.7012	118.923	37.94527	65.41782
WDR70	407.8028	469.8659	254.9665	55.89349	753.0978	50.23914	978.4167	531.7827	57.62336
VISA	290.4143	137.5552	403.4393	264.5269	688.3381	558.074	1294.891	1181.887	39.01568
FLJ45964	61.23281	88.2198	45.71855	55.60551	50.71323	39.40052	41.81799	44.40323	101.0828
CLPS	36.96147	46.66481	39.09995	48.83859	42.0567	35.37611	37.01179	52.43091	48.51766
KIR3DL1	102.0468	139.3548	70.46606	116.5172	61.67889	182.3584	55.24821	61.40217	61.21391
DPY30	437.0685	1448.934	1186.241	267.5191	632.9375	206.9169	539.3325	454.667	581.0776
ZNF718	61.47554	91.62963	50.73897	99.50517	51.96805	67.2361	48.37693	58.59546	199.254
ATAD3B	41.29803	1699.901	664.8356	1066.793	1289.791	804.1135	841.9161	757.3135	2935.957
SNORD107	33.06478	33.3298	40.54769	40.43079	47.39581	36.65664	42.42095	38.62305	36.05177
SPANXE	38.51393	32.70768	39.82948	45.44812	40.00598	44.24046	37.42976	38.0241	80.79985
CASP12	68.93259	35.2239	45.03298	50.40099	47.04178	43.21613	39.39003	54.27859	35.9821
TDGF1	93.45999	42.49102	62.32078	76.86343	50.72921	42.38444	53.11014	68.57817	91.34288
CLDN4	186.1288	163.9023	85.93987	107.1037	75.28307	152.567	73.56358	149.2122	424.6843
DEFB103A	15942.85	18491.57	12707.66	11305.13	19357.28	7091.402	10260.83	11056.5	11925.23
ARHGEF10	1228.629	197.3981	1736.762	1408.106	2586.271	1170.152	2137.123	1603.114	329.4792
RAB6C	37.97475	59.18333	33.01779	52.28747	41.87407	48.88363	35.65913	46.711	139.7624
UGT3A1	57.3107	100.0553	52.20464	56.73126	58.34158	55.54137	45.82301	48.14515	50.6617
LRRC43	80.79713	55.49742	54.04202	81.45185	55.43879	63.6408	49.58691	95.64144	61.10845
POL3S	856.3368	33.98112	528.8665	39.30837	604.3911	965.9966	566.5508	149.336	32.52165
GPX2	455.8005	257.5015	826.8317	269.2679	2203.117	353.0774	533.4326	2117.411	476.2909
LOC91561	21919.89	21959.89	22375.88	24437.4	23792.46	22821.96	20539.15	24692.3	23053.15
SMA4	47.73103	62.94808	221.1751	525.2231	124.5689	69.73369	188.978	159.9441	79.30494
DULLARD	982.8801	125.3153	406.5058	159.242	339.9039	104.3871	407.4041	531.479	260.0789
SGSH	63.01141	124.1227	58.79955	69.94575	85.73794	46.15901	110.2122	117.3645	66.52234
FAM76A	663.922	594.2128	411.9547	913.6359	285.9055	585.7155	404.1324	319.0064	658.3877

ALKBH3	42.00333	46.69339	879.4481	48.27623	481.4691	45.15592	457.2828	922.9349	46.64532
FLG	7899.296	3270.48	4058.074	2320.973	8060.742	4877.762	1406.75	1176.813	1357.169
CXorf15	51.39455	33.73103	1074.882	77.96858	1237.248	415.7531	634.9142	51.25196	42.86781
ATP13A1	17852.34	18351.76	14769.88	15732.58	13572.25	15640.64	13290.97	13023.93	20201.44
C14orf85	1009.052	1578.261	1015.49	813.3962	701.6074	541.4576	537.7609	726.7562	1420.656
DNAH12L	65.33683	35.23134	37.39223	41.25649	37.2471	37.5867	41.51396	37.98617	37.31538
FCGR3B	506.5281	272.5014	131.955	309.6755	126.5957	218.7756	119.8316	145.0579	662.9952
FTHL11	62.72116	64.7447	43.62968	85.6547	54.81106	59.12715	45.91684	58.88566	87.20425
OR7E91P	40.12148	1413.933	1661.952	66.56034	1805.605	3363.58	1002.571	1212.645	2492.46
C6orf26	91.75001	729.5496	68.24098	152.4552	66.81934	93.92542	58.384	59.24487	150.3171
LOC728758	79.31556	43.40912	134.1902	69.20152	103.7871	264.0246	211.3638	153.248	60.18445
RAB38	69.35039	126.0744	2089.107	1598.342	4221.907	2060.707	3977.188	2787.382	103.9094
SNORD36A	768.8852	710.8237	345.0674	750.499	252.7083	357.4691	219.394	347.0438	698.4115
RAPGEF3	728.077	1468.108	423.3738	1511.246	322.5919	466.3708	768.6598	588.2875	2342.899
CHDH	40.90096	44.69519	37.05594	40.29856	42.23488	34.38368	39.90093	50.31961	61.87026
DEPDC4	114.0988	63.41867	66.72096	126.8115	48.454	73.5607	65.0922	150.9458	132.052
LOC152667	48.72143	45.30797	56.27163	44.30384	37.61794	44.12289	41.45411	35.5545	55.97626
HSF2BP	65.25246	72.95959	52.11817	1512.891	53.69312	542.6458	198.918	65.20385	153.2235
NR2C2AP	4200.522	3525.107	4001.527	3853.028	3761.794	4420.724	3807.427	3183.519	2188.727
TAL2	136.7528	145.999	58.83911	139.2643	70.00955	74.64315	69.5348	61.30746	177.0392
GPR42	39.24491	89.42057	46.86057	36.20956	42.18402	62.67953	49.63975	60.12599	45.59173
C16orf69	2844.311	3743.115	3619.58	3642.687	3021.426	4182.758	3272.807	2096.397	3046.148
MTE	51.6024	51.92594	50.10164	39.29527	58.94471	49.8561	43.04544	50.3955	73.03711
PABPC1	11627.38	10165.6	11418.83	14490.32	11542.39	11309.54	9980.455	7332.618	11202.27
CNOT6	424.8705	1348.943	951.012	782.0253	797.9956	1281.656	1034.502	1672.307	956.5519
A2ML1	4119.994	1629.861	6056.902	1505.835	9201.298	3232.043	4244.985	4088.242	2980.167
ISCA2	2977.149	2909.056	2073.647	2146.514	1024.937	2422.425	1823.689	1930.345	3435.5
OR5M10	57.65938	37.21988	35.02985	42.24625	33.40449	41.13365	36.33485	37.50271	35.92751
PPY2	55.56049	50.98792	42.87043	42.79365	56.65553	53.41282	42.97837	59.99105	61.17404
FABP3	2696.993	1365.43	784.5004	866.3152	614.0703	1892.824	1851.072	3181.643	276.8165
C16orf78	54.58565	112.5588	51.76221	51.97991	42.39247	38.10833	48.64297	91.83376	102.5928
ZNF7	36.09246	32.77971	36.03957	43.48312	387.828	171.0557	283.5158	199.9025	147.5641
LIN9	1800.044	377.9184	796.3795	1072.306	379.993	229.6146	173.3552	408.6256	45.99232
GOLT1A	65.5472	46.02447	35.92132	65.22832	43.59991	35.28045	34.07706	55.70457	55.76979
LOC728621	34.67581	37.86064	38.6345	48.73905	36.20978	34.99044	34.51246	33.87393	39.29418
DVL3	212.3666	157.7206	373.4576	104.6767	141.2379	399.1969	84.25235	160.6325	317.2825
LOC401010	197.2427	1066.197	78.87513	132.2384	58.07047	99.25552	130.7151	231.2888	520.7964
ALOX12P2	39.26526	37.41207	39.61388	49.53254	201.7695	671.1064	121.5901	70.08706	53.53556
GPAT2	58.62117	34.30813	37.95623	35.54087	33.7425	34.87469	39.12182	36.08564	42.53754
CTRB1	168.4397	108.447	79.2119	128.2773	65.22687	104.384	53.08141	43.72409	326.23
HEPACAM	37.5747	43.79192	36.11282	36.12144	61.28691	39.59602	39.19754	34.93634	34.55005
BAGE4	58.76699	45.26532	43.02872	72.96281	37.34345	41.89315	48.57585	40.11012	77.22294
TESSP5	393.3767	206.6883	109.0993	241.9024	99.70669	159.027	92.20674	135.6344	452.9739
DKFZp686I	59.50115	621.8152	56.31683	74.4554	36.89374	460.359	194.5621	46.66193	51.53753
C1orf99	152.8411	63.08236	48.68522	67.1146	58.65593	63.54	45.93254	49.63736	71.49633
TFAMP1	532.8619	625.339	202.1892	361.8687	273.6916	608.6964	183.8958	303.5388	4931.72
C9orf68	45.79558	58.51758	40.07552	39.85744	49.32853	39.13482	47.87507	47.15538	50.92131

TP73L	39.72892	39.66248	990.0307	2384.519	2173.821	1250.54	884.3389	56.95784	49.92044
CASD1	185.4427	127.5274	66.04314	126.9194	139.4215	91.56246	88.94019	171.9353	63.0599
ZKSCAN1	703.9197	191.5982	43.97378	346.3909	77.37366	63.34086	55.15266	37.20967	36.24003
SHISA5	2391.097	2827.299	3113.482	2911.448	2110.775	404.4017	3255.23	1057.345	1965.859
LRFN5	85.19657	48.79447	200.8893	90.60664	55.98328	50.84676	46.20365	49.47404	151.9974
UNCX	46.38895	36.51558	35.09998	30.04921	35.88592	35.68116	35.55827	31.95108	42.94704
OR1S1	36.83747	46.42353	35.98697	43.5883	36.39909	49.3742	38.89359	32.39554	67.66141
LOC439985	34.71215	422.3252	150.4777	57.29218	103.2513	243.5141	32.50794	44.46018	34.88866
LIMCH1	1748.887	4013.395	2214.484	2392.864	3188.773	3234.208	3644.486	4464.746	1301.53
GPSM2	50.85433	97.04258	409.4673	781.766	444.8147	69.53732	570.8156	588.0572	37.92428
RPS3A	690.4399	306.0553	450.283	554.1358	489.7964	362.8907	387.931	259.3212	684.2862
DKFZp686D	64.94482	47.68449	59.81133	39.94123	49.00453	37.86287	57.7601	39.30455	179.7927
OR2M3	53.84095	47.44776	41.20196	41.88947	48.4519	43.70702	41.74379	50.41833	33.94651
CCDC57	40.43439	52.28213	37.35609	65.38542	38.85588	37.15303	41.90213	35.31517	45.47234
ZBTB12	50.03094	39.90554	37.49955	39.15402	138.6951	51.51888	41.74091	34.56593	58.60512
FLJ42258	135.5625	97.51535	72.63594	62.44964	50.34963	53.95197	56.4637	48.4435	178.3882
FAM108A1	2117.406	1494.459	43.90911	44.76044	137.732	391.7531	394.12	319.1379	1572.55
CRIPAK	59.27217	646.2043	2197.63	696.2739	1424.218	1302.55	1316.151	847.7244	44.22635
FAM24B	47.87865	63.42838	393.4063	82.53889	328.9126	371.844	461.5505	348.7413	48.51024
OR6W1P	60.55949	53.35968	34.33651	37.9623	46.10027	49.30614	40.03341	45.2412	49.08049
TPM4	454.8941	66.85091	114.1639	328.3723	90.33154	47.55162	78.51514	90.25719	707.6508
CAMK1	438.0137	232.4151	233.0122	245.7849	195.7586	152.6815	135.5667	95.46306	538.6114
LOC641522	437.3817	254.5932	132.1662	244.1498	231.8822	235.0992	174.0433	177.882	579.2673
C15orf59	162.2943	120.6132	515.261	95.36105	392.7207	68.48642	369.1389	84.60437	2683.369
RANBP9	442.3491	245.4939	104.4991	197.5329	99.69607	147.8151	90.18003	115.5045	573.0779
SPAG5	162.9348	389.3883	120.2511	333.4576	109.9397	179.0588	150.2397	135.5056	258.2739
RPL18A	35559.81	34696.97	33651.85	36295.7	30775.39	32717.4	32500.02	30819.77	39328.77
SERPINF1	19354.85	17703.82	17746.47	13725.86	13863.72	16908	15316.93	18952.42	20556.38
MALAT1	109.2161	482.271	45.51253	69.98139	58.16181	97.96842	54.00517	283.4504	765.8859
CLEC14A	641.779	746.1435	464.4341	485.9377	739.8633	687.4838	578.0651	562.7039	1059.362
LOC401398	175.3679	97.37369	66.50501	172.9399	66.5307	103.5976	67.21346	80.75307	82.30025
SLC25A43	303.3547	51.50797	131.5955	286.5424	185.139	189.987	99.44571	56.74635	54.18706
GRTP1	37.87907	51.4899	44.49495	44.68398	52.40572	38.48903	43.31756	55.025	61.6817
NENF	11048.19	12220.91	12477.1	13548.52	14405.39	13406.09	15244.15	16575.44	12440.29
RNF4	36.98227	47.55086	47.00955	36.39662	51.73292	54.68196	62.76541	52.46821	50.36295
RPL35	10455.31	9595.332	5969.696	8555.802	4509.234	6272.552	5234.593	8606.871	12066.33
BLNK	81.80027	199.933	246.2378	43.88279	110.551	125.2735	113.5469	130.3776	40.07099
DLEU2L	39.00047	260.069	105.2855	61.84449	61.23243	43.28759	84.05288	115.1005	36.85107
ADAM6	208.7927	57.87554	69.96995	91.52441	59.30725	110.3506	60.29994	65.55444	87.86876
FAR1	3309.811	5363.633	6214.809	4134.527	5112.561	2655.833	2981.588	4955.818	2989.837
SPIB	57.21668	197.2652	230.2185	120.2703	66.6538	55.70026	101.6684	286.8959	1140.737
TTTY14	143.8466	107.2637	80.38677	105.4245	72.85181	111.294	66.55538	138.5304	1532.35
CLDN7	34.1344	36.59825	188.1207	34.89614	719.0059	33.86369	233.9708	34.99774	45.3168
ZNF148	10499.76	10917.14	8806.295	11251.72	9536.868	9444.682	9374.433	10185.94	10075.57
RNF138P1	43.29766	36.61613	127.4567	41.19677	101.6326	38.94643	70.69966	38.17416	36.23203
CPSF2	1051.425	1892.513	3300.017	2046.825	2072.311	2500.544	3188.542	2797.259	1076.258
LOC441251	251.8324	174.6828	90.46423	168.3712	76.93192	127.0657	63.91214	82.57719	383.0094

GLB1L2	39.11841	56.95887	45.16783	88.1768	38.21571	37.69712	42.7109	53.20998	54.86116
HIST2H2AA	1415.305	2381.085	1318.87	2554.083	962.903	2247.202	1801.043	2349.873	3158.388
CTBS	617.0942	460.6403	672.1653	1012.114	625.064	634.3915	755.7946	619.6462	355.2006
MGC11929	45.71767	47.67322	58.76706	36.74542	60.02379	42.52204	40.78409	78.32681	52.72881
GUCY2C	56.47775	45.36487	322.2667	46.32616	1770.864	40.15811	447.8258	33.26749	56.01019
GOLGA6B	39.17426	56.97344	217.1882	48.74377	41.79562	42.80258	76.9365	31.78331	42.95887
CCR5	69.41658	55.80489	56.66898	68.45233	55.91229	74.07177	46.66656	54.36143	87.83401
LOC134145	1170.642	1338.052	1035.106	608.7383	834.935	1449.201	1196.24	1048.429	1055.52
POM121	40.616	863.2498	549.4096	675.2866	448.7179	468.0269	333.475	209.7131	49.83463
TMEM173	16194.66	16442.17	14014.27	15552.27	10800.56	12547	12462.05	12977.69	14046.15
NACAP1	39.279	37.2058	51.2083	44.75224	51.60941	39.39944	42.62629	37.82721	40.3511
DEF6	7695.438	8262.811	7822.41	5994.641	5084.338	7714	5369.398	7847.106	9267.916
AKR7A3	133.4843	379.3008	210.0554	406.7448	325.1088	232.3341	456.4192	338.8575	66.48442
TTTY16	41.16716	33.56135	40.15088	35.66489	36.8847	39.81553	41.62483	33.80471	41.89343
C15orf60	99.24819	80.83087	63.74374	49.28718	44.50276	48.96028	46.74638	84.50001	48.21659
RBMY2FP	239.1879	215.2213	147.732	261.3988	145.4914	197.7916	122.5399	197.7815	262.1939
FAM122C	100.3479	68.71275	53.93996	82.49151	109.977	69.38249	74.45796	39.06975	37.20053
FAM153C	43.68258	66.66064	42.3762	38.58521	46.42844	46.47918	52.90605	40.97065	64.15749
LOC441120	40.31218	36.10327	38.11839	32.30394	46.01059	36.83948	39.02719	55.35002	45.85394
CRIM1	985.6723	1360.786	623.0412	2744.029	566.7939	783.0877	733.8741	786.765	934.9471
GPR153	352.9289	424.4818	246.0309	1010.535	96.95061	594.276	207.0423	201.0145	1335.815
SFRS16	1119.018	627.4226	470.6667	667.0271	283.7115	375.7592	475.1346	546.5237	1203.613
PRB3	201.5735	136.2712	66.23025	203.2408	69.91198	112.0912	129.5358	553.2592	322.945
FAHD2B	165.8571	278.6039	381.7375	69.95718	489.8832	91.97214	703.91	560.721	158.1764
FABP5	566.5551	587.1037	1785.866	1178.942	3650.708	919.1902	2745.309	3949.807	653.9741
TDH	40.89326	48.29551	46.63944	41.29436	45.50711	30.93917	31.09867	39.92859	51.90622
ZNF510	36.26232	39.0267	42.41293	37.76728	35.17945	42.83167	35.98581	39.82766	35.06673
LOC440348	35.28915	45.46465	105.084	277.6151	55.78397	41.3708	58.22996	35.2167	38.65948
NBPF15	609.6511	349.3599	200.1127	455.9212	187.0999	227.2357	238.9906	208.5913	759.7429
MGC10997	1100.826	966.1085	393.6418	1212.751	416.2592	755.8551	429.7213	492.8616	998.5809
PNRC2	302.9559	214.4161	132.167	193.739	179.3588	184.6051	163.9306	113.5696	362.0388
SERPINB10	236.0217	109.5765	91.05293	138.3503	84.76331	119.3724	84.39945	101.4573	295.8735
MAN2A1	641.3509	1214.138	3003.792	1856.248	2644.538	2397.911	3230.074	2381.614	46.40418
QSER1	2588.028	2529.758	1023.551	2947.071	653.5807	1099.578	470.4024	1150.783	2760.121
BP75	47.72365	37.79272	51.09127	36.15742	37.52152	62.61442	59.31325	40.90899	48.95927
EPR1	95.51557	99.06891	64.81472	76.45004	48.87073	68.22234	50.97374	67.0107	60.85865
OR2T35	56.93466	61.47695	50.19453	70.79952	43.50861	55.09293	48.01778	38.1966	44.99101
CHAC2	39.90912	46.05587	195.8974	41.74261	139.874	37.20871	95.35316	72.82662	40.69171
MPPED2	44.22021	45.21367	62.40782	39.13597	37.37163	44.63716	31.7394	157.2154	32.91497
ZNF749	130.1998	91.73089	85.93791	219.2603	70.13667	68.87513	61.68714	102.1483	93.74638
RASL11B	106.9223	435.8142	500.0843	44.1016	1146.816	1439.051	1478.311	819.4331	44.59115
C20orf107	149.9453	87.10783	56.50162	48.6444	56.1377	58.55793	50.37093	51.19854	62.32375
ANKRD13C	883.145	40.06428	679.5717	1271.571	626.9037	346.8325	871.0148	609.6699	104.9097
H19	1576.511	2039.182	1257.362	2289.873	6382.063	824.8159	3620.514	4823.458	5599.771
TUBA1A	8257.22	5196.885	3645.364	7601.365	4819.366	3815.71	4215.999	3337.699	7904.551
AKIRIN2	50.21991	37.65122	2877.651	1108.995	4001.535	2491.858	3546.473	3247.227	52.18512
ADCY4	3999.045	2935.935	2192.562	649.552	6332.854	1942.708	5220.442	3247.399	2455.951

SFRP1	97.65604	61.75875	213.5189	806.8431	152.4769	40.15592	34.67544	194.6936	61.79869
RTP3	39.03522	44.52097	35.77404	68.61521	124.7275	51.66265	177.9536	35.85772	35.52051
TMEM40	47.51615	40.33025	293.1853	399.7948	39.69164	38.74356	319.6432	62.34007	168.7043
RASSF10	34.20891	45.0635	239.8667	33.25248	40.84815	56.38243	150.6032	31.8993	39.53609
VPS25	81.66399	212.4825	419.3265	485.8964	226.1006	506.3094	418.4655	462.295	1158.498
TTTY10	297.6666	194.1649	106.7349	232.855	92.52315	152.4798	87.97124	170.3745	322.0112
FOXG1	102.7018	73.81574	46.82867	47.20251	59.77486	62.46209	46.60339	43.44387	49.67838
FLJ45256	8268.933	7714.79	5038.964	6741.786	2622.24	3760.687	2923.038	3433.091	8798.869
C15orf24	104.9411	377.0936	832.145	104.7718	1137.625	626.9345	1788.207	704.8661	243.077
FLJ41841	35.92389	35.6242	95.32266	39.15928	38.00924	161.5347	33.27262	47.58147	41.82347
NRN1	164.3579	111.5123	103.4439	156.4458	93.75246	134.6278	74.71463	119.4226	209.4273
KCTD18	4670.6	2349.902	718.3332	1366.759	196.6348	918.6046	324.0667	731.0146	3231.927
ZFP36L2	47.3765	1207.627	2290.489	2588.868	1366.676	1156.998	1416.089	1146.022	1233.175
RNPC3	1106.928	1027.866	1972.662	1212.516	1650.322	2811.551	1760.052	2147.27	818.2027
CBWD5	468.5224	2430.709	1363.358	3049.557	918.8012	1230.621	736.87	837.8111	607.4933
TMEM64	92.36306	75.86859	37.15502	39.98342	35.35842	35.40586	55.3318	39.78051	39.42844
FAM75A2	78.25026	46.26401	40.19417	37.15385	41.59008	32.18244	46.06706	37.71841	34.15508
NUB1	54.55794	37.10809	39.9533	34.18725	43.7099	56.46758	45.99855	42.96787	47.49237
FLJ45966	261.5076	127.9498	73.37154	114.0858	59.28392	90.8417	61.44285	89.90248	440.8011
HLA-C	350.8885	339.7435	12788.52	7334.655	43.32971	137.4745	32.14972	5138.536	4594.709
TNP1	240.0259	138.4046	82.65402	166.2017	90.43593	128.7868	77.93239	103.6436	409.3784
FLJ22795	9620.978	7670.327	5512.243	6672.136	5054.314	4659.423	4934.053	4194.25	11221.46
SERPINB2	119.0881	134.6645	199.7373	742.2118	364.8624	304.2659	429.2871	565.6118	66.55865
ZNF821	8044.093	8077.304	4599.886	7795.288	4910.244	6269.05	3632.326	2115.279	7135.26
GABARAPL	1112.376	52.86766	66.86584	84.31288	511.2403	64.70991	576.6738	608.9884	83.88714
NOL12	338.7777	694.8187	2471.031	1500.305	3272.019	3707.461	1947.134	1786.418	46.0054
ANP32C	37.02491	37.35846	140.1508	33.58002	34.0531	35.04804	39.18154	33.3273	45.9962
FAM103A1	8256.233	8243.961	5261.303	8063.626	2599.137	5858.251	4584.813	6016.442	7873.813
HMGN1	506.2693	473.8324	96.81628	309.356	586.7178	37.67324	338.4235	404.0117	40.74058
LDHAL6B	51.02808	68.83588	61.28867	31.68956	50.14382	68.67498	41.71614	51.05477	35.66718
PFN1	15945.96	13755.49	14540.52	16756.11	13123.13	15742.18	15702.21	13553.67	12683.77
PSMA6	6453.912	10825.12	19470.81	11582.31	17477.97	13794.77	16242.26	17974.34	7883.104
CBWD1	6483.586	7067.785	5628.589	5731.317	5497.323	6195.429	6053.949	6272.869	2469.331
MTMR15	127.3511	798.1414	200.0947	40.06562	53.68917	596.3792	563.038	188.094	58.76801
SSTR2	105.774	449.1568	56.19003	82.78371	55.6908	51.8577	54.00448	57.14313	110.1189
BAT5	121.2263	860.425	193.1221	110.9066	641.1719	816.0169	450.1406	56.02621	2403.378
PIP5K2A	1073.891	57.28834	648.845	36.65216	41.61842	50.50877	113.9413	37.18406	1558.426
MGC7208C	458.5789	242.6387	151.3938	301.6734	105.9783	226.913	154.5951	229.2919	557.0035
STK38	1948.364	1752.149	997.2026	39.1531	1100.976	850.2097	1532.881	1287.923	51.2662
DTYMK	108.4237	63.05272	51.40469	58.67139	39.48515	57.41059	45.41265	39.59795	45.10354
CGB	333.1738	219.001	99.23779	195.3731	91.25159	119.1605	73.1828	98.88954	384.3781
KIAA0090	8528.552	8167.727	5068.943	8003.106	3372.993	6663.257	5229.417	7016.821	8093.514
FAM50B	677.3957	505.6116	333.4404	616.7164	236.397	51.96835	235.5358	401.518	51.88123
WNT7B	109.2847	71.99093	45.90003	66.35529	168.0972	119.104	147.9575	444.9295	101.4875
MAGEA3	38.51889	111.8885	139.704	31.87355	36.66484	34.16365	30.90728	36.90421	42.54028
TBC1D3B	150.5864	127.2781	70.7427	129.186	371.279	84.85266	196.5087	89.61895	143.4294
ARRDC5	4528.501	4739.057	3256.122	2195.997	240.5276	2404.142	1629.302	1441.645	4102.242

FAM9B	60.51403	60.77975	59.00129	64.86024	56.95842	43.92009	56.33515	56.50657	87.10384
FVT1	61.30573	961.0917	273.1359	1026.059	1038.13	487.3786	495.7291	509.978	68.15668
UPRT	1134.207	581.7132	663.4976	980.4288	457.2061	422.4979	442.6836	613.5783	1394.513
PSD4	4982.251	6971.829	5269.612	6184.36	5838.838	5555.104	4158.551	3201.784	4062.262
CEP76	55.03944	57.37688	34.82955	39.59731	43.00669	55.09194	55.51604	43.2254	61.60559
PHACS	1719.242	51.00022	37.6762	35.10624	154.1006	57.27897	91.01175	246.5746	2236.234
ZNF330	624.2831	704.0237	985.0452	734.2655	1371.789	1180.854	841.8903	1044.139	1015.593
COX11P	35.32087	56.31833	47.87523	43.48838	43.73579	48.5556	36.45941	62.3078	59.77464
COX6B1	115.1669	1630.252	1104.695	1482.176	521.2617	1155.065	566.6195	921.2575	365.792
C14orf50	48.30304	88.13344	64.53402	102.1813	51.61825	64.70348	52.50129	42.01027	131.8186
ZNF681	525.1746	39.14549	69.79208	52.04823	97.43448	96.50319	71.51592	50.3299	34.47021
PIF1	78.02981	65.44318	44.18712	367.6256	229.3448	67.32088	117.0658	172.5639	102.6816
C1QTNF9B	116.4881	39.71949	150.967	46.19817	57.69524	70.7997	46.57442	45.81579	57.43622
ZNF652	7874.893	11400.28	12328.17	8521.962	9802.213	6489.898	8259.558	11073.03	9894.665
HPR	350.1563	204.59	113.0085	233.5053	98.89056	134.2418	87.23036	71.42222	207.4036
NBPF10	8394.156	9894.687	14065.12	10308.43	14447.69	11079.45	15714.82	12988.25	7628.645
EIF2AK1	7316.745	5601.181	3089.137	5991.673	2700.123	5961.366	3668.639	2324.047	6861.881
DFNB59	88.87448	87.20984	58.7901	90.98558	67.26804	69.74946	51.03601	70.2047	119.7879
ACOT6	58.91422	66.76328	35.4608	61.03756	51.98856	36.70636	47.74491	42.2818	46.96198
PGGT1B	341.9481	439.2775	316.8319	499.1586	381.4498	746.8092	202.2017	371.4062	249.4522
PRY	151.8631	49.43408	41.2468	57.97165	45.57756	61.87025	50.39169	49.56831	40.14909
IMP4	11492.49	10251.25	9186.739	10766.51	8064.443	8953.38	8272.662	10686.83	11750.86
SNORD48	4376.857	3174.995	702.6789	1405.586	654.6932	1562.813	665.2838	800.6175	2662.914
LRCH4	12081.14	11205.98	8148.486	8685.403	5524.783	7213.381	5232.615	6403.156	12607.98
CCNA1	90.56132	67.32046	43.51234	46.37474	213.2157	33.18626	48.30556	40.17556	66.03316
OR2A42	920.4107	48.99269	39.1619	46.28645	35.25843	37.61491	38.94471	36.46535	37.22942
STUB1	749.9794	288.9302	42.60137	44.18253	248.3233	310.0051	80.0061	44.46546	54.82698
BLOC1S1	426.625	233.9555	941.9702	256.4335	1019.911	775.5551	943.1851	1489.323	571.4587
GBF1	276.2541	335.4881	165.7956	153.3841	203.6343	259.1564	162.1196	149.5047	333.5629
C14orf19	54.77529	37.82071	33.05041	34.6835	38.27739	34.8341	37.24737	43.98271	57.66889
OR8H2	92.23546	45.51364	36.67782	55.49346	41.3657	75.44026	41.17824	61.96513	73.1553
FKSG44	217.7506	134.4387	67.91225	83.08487	272.6075	54.51832	53.19285	311.9061	678.0861
KIAA1683	1024.557	808.8546	353.6848	303.5218	398.1661	596.3683	287.1498	376.4517	1188.603
ZNF430	7417.405	9852.387	8280.24	6401.097	5191.953	5028.976	5415.633	6658.848	9166.526
SHOC2	341.4016	167.3255	94.36768	199.9253	129.066	236.6148	103.1513	140.1836	346.9758
SH3GLB2	17443.74	21493.72	22227.17	17182.43	23974.39	24079.25	24168.98	21535.2	19216.12
NACA2	342.5526	165.005	95.2944	203.8904	88.74736	148.996	69.63608	72.9995	331.1611
C10orf28	665.3533	368.9215	235.1535	496.5989	235.4139	307.5985	218.8496	235.0087	876.5912
IL1F9	1517.02	3465.323	830.8845	144.8011	5945.708	926.0147	1430.951	5826.794	6567.711
PXDNL	38.29663	68.35942	54.77361	74.43867	52.39914	58.55285	77.29366	357.9035	50.49763
TP53TG3	892.3293	488.3083	784.7776	2731.921	1247.824	1012.113	856.9274	997.0217	51.23881
OR13C9	273.2541	147.4641	94.16505	138.0559	81.77877	125.8402	73.28336	77.08582	266.8255
KRTAP5-5	36.91346	36.36174	29.40376	39.64705	36.27108	34.46437	32.53533	40.94343	34.2093
PDCD1LG2	48.88926	709.4471	32.10015	34.69982	33.35014	43.55135	37.19058	49.64387	32.81707
LOC44137€	70.56686	40.0648	36.22851	54.81128	36.98042	38.19447	41.40873	59.33456	69.6524
C10orf114	157.9017	81.0688	241.261	116.2731	193.4541	126.0142	182.9286	137.7678	89.90649
NKX6-3	169.3228	90.23563	73.3834	187.8827	71.34319	128.5584	68.20045	112.5142	382.7966

SBDSP	202.1396	83.33248	58.64776	123.8871	132.1682	47.77744	130.5209	162.8965	35.95036
STXBP2	804.958	531.0903	322.8627	42.68002	371.4225	319.1668	383.8639	391.0962	34.80243
LOC49230З	43.29357	48.27036	39.45903	57.24206	58.82049	41.98735	137.2291	35.55988	35.0975
HLA-DRB4	124.3527	128.7517	696.7743	126.0894	282.2254	594.1689	1299.555	62.42625	90.73798
DUX4	90.10471	106.4089	66.73451	80.43523	58.99014	72.727	51.77273	46.71013	165.4365
GABRB1	36.35932	53.48074	53.03664	35.90488	161.8727	32.16299	72.01507	37.04821	964.1958
C4orf15	75.7314	40.99772	40.06962	535.6822	45.55521	57.39054	46.34229	35.68893	75.92348
SNORD36C	3316.913	2985.608	1526.908	1716.654	730.2447	1755.246	561.6838	1546.585	3971.843
TACSTD1	789.9465	1182.38	1641.419	1788.687	2386.663	585.3492	1676.224	1279.991	2130.178
RPL24	2882.485	2690.345	793.5203	3445.865	755.4616	1022.493	703.1589	540.0596	1698.448
IL1RAPL1	43.36253	853.0339	199.124	47.43045	494.5807	345.1693	260.732	277.261	38.06254
CCL22	60.63828	347.7656	209.7416	45.23774	247.7029	33.6127	255.51	837.2021	81.99791
TMIGD2	50.92831	50.07302	41.92791	39.80749	33.29344	161.9551	41.44749	36.03193	39.92468
OR5K4	33.89369	34.48183	37.41468	37.78661	34.38068	37.55851	32.88805	36.00201	33.32869
SPERT	253.7451	86.73158	54.08378	113.0636	49.29662	80.47886	72.48173	62.17203	405.9401
FAM40B	81.87698	65.0437	185.6675	66.01033	41.02934	60.95794	39.05476	38.56258	37.57372
SPDEF	52.36856	41.22001	37.0768	41.69616	43.44795	33.10584	289.5576	94.01405	54.15188
C6orf111	2867.26	2940.21	1389.69	2390.702	1204.228	2144.775	959.7999	982.0666	4998.782
PHTF2	45.47948	34.67658	32.70765	381.2033	61.08243	49.00601	97.48824	38.69616	35.87653
C12orf49	38.53352	997.5186	685.3186	1199.574	42.88658	940.2523	698.5187	1144.443	62.0195
TRIP10	116.9281	1119.744	600.3098	59.72025	522.7584	999.3417	775.7831	579.5217	45.1606
GLYATL2	37.80822	63.34073	45.94659	43.20941	40.12974	36.74895	42.43071	31.32221	71.75281
GPR81	44.87862	36.99091	54.92763	52.98299	33.46101	38.61269	40.11119	36.12988	46.8381
WIPF3	166.7598	203.6742	98.75624	206.764	93.19351	149.6054	111.4718	113.7422	43.98258
FLJ13611	104.6745	102.3949	150.0957	126.1102	295.3004	139.2433	235.3496	218.4964	67.59035
NEK1	877.5917	1228.424	1690.997	432.9187	994.8762	1666.699	997.4625	1398.002	50.2867
NMU	523.9913	320.5392	156.6741	33.26778	272.4571	199.3801	73.45596	140.1086	759.4818
DYSFIP1	527.0292	73.17158	112.3325	366.8989	41.04408	44.30032	64.16557	181.74	55.1038
ZNF597	43.99224	71.67849	41.11583	39.03983	42.52347	603.0923	170.1116	62.15449	43.49771
FLJ40113	55.08319	935.9394	292.4999	39.92728	50.68062	294.5309	132.1323	36.78992	36.96864
RKHD1	111.1324	159.9541	62.9157	199.6551	51.22593	52.62468	69.95573	60.70859	87.94034
FLJ36166	53.54813	46.10153	35.20333	41.87821	69.58886	34.69873	36.2454	36.08787	46.8325
AHR	1709.117	2091.216	604.1802	720.8496	546.465	1111.717	780.2676	706.3426	723.2977
UGT2B11	48.26327	75.47234	48.12971	68.68762	44.83088	36.35493	43.98709	49.42643	47.42905
KHDC1	35.69219	45.82298	44.88928	67.79605	159.4598	41.74076	109.2661	42.86769	50.64197
CCDC110	51.6919	57.19562	129.1823	78.99208	41.54148	41.54828	41.14637	134.6642	52.03644
FAM120A	10048.64	11695.74	15591.36	12663.34	14891.21	15914.9	15279.56	13625.05	8860.713
LOC38895	99.04112	163.4405	122.1471	226.6786	96.47097	136.6727	81.13333	119.473	626.1263
PGA3	58.63444	48.41743	53.84801	37.53165	54.00821	60.12441	41.14104	37.61744	71.90617
LYZL2	35.98063	34.53063	37.24162	54.2272	38.56446	50.03038	35.04515	45.88034	38.11155
TTTY11	229.0662	112.8721	74.9288	128.0765	88.71277	82.08484	62.83329	56.2132	239.6121
TULP1	40.51606	48.14167	65.34927	64.67683	51.02119	52.03291	46.34102	62.46831	98.01148
KRT7	67.68791	115.3155	50.70918	89.15102	68.39137	90.90744	458.4534	58.74935	101.4981
CGB5	44.00913	50.10224	62.77434	70.32944	161.8408	38.17714	318.6315	41.74387	49.48287
RAET1E	128.1767	180.7168	86.14584	157.7407	78.76987	135.9384	76.02903	98.04404	392.2675
KLHL12	76.77032	60.29301	92.91695	88.58141	61.73874	52.39354	105.651	54.90246	52.72874
AICDA	70.49869	106.3735	53.44238	107.9017	59.20783	79.89044	46.19348	72.80286	112.3041

UBE2F	13283.98	12108	15452.69	14853.92	15239.87	15263.28	13795.31	16149.82	9768.033
TTTY5	70.7495	71.68913	60.74253	71.13738	57.71947	54.05033	46.62401	82.36804	51.28894
HTR7P	84.93166	69.37238	55.89119	62.94596	67.63451	82.89968	86.08556	52.81974	116.6787
RGPD6	1114.52	45.00502	52.50496	38.9748	67.58347	56.4357	118.0341	64.10865	45.0906
DEFA3	36.95061	41.37985	41.78675	76.51812	38.01859	34.91052	45.32696	40.82414	42.85536
HIST1H4B	1378.85	1078.594	317.589	58.65775	258.8557	31.92936	84.37679	380.8369	52.08517
HAPLN4	128.7536	48.84227	50.16452	75.75734	75.90144	53.87719	55.72095	93.112	122.9466
HLA-A29.1	8047.738	7917.194	2327.411	3126.491	921.0429	4882.572	1373.875	1824.995	5090.731
SNORD34	2897.777	1666.576	581.7795	1151.983	146.1015	438.1671	148.2991	295.5795	2420.789
ITLN1	50.46985	40.72782	51.30618	57.26305	38.17453	50.37667	49.15017	65.46696	69.96188
PIK3C2A	67.97543	412.6793	376.3364	85.49314	262.3971	49.69172	103.7022	129.2274	1046.487
NOTUM	359.8529	183.4672	113.5996	181.6764	135.5704	173.641	93.36094	114.1022	390.0254
IPO5	75.73835	53.30763	35.10329	62.73422	154.6135	58.92461	121.897	68.03336	117.1296
HPGD	66.72117	58.33252	58.27483	97.76611	284.9424	62.25963	56.44201	56.69977	71.57891
XRCC6	2102.122	354.2123	1751.202	1533.184	3266.598	1473.7	3215.248	1759.427	51.06162
CCNYL1	80.86991	1094.862	1315.064	51.28114	1885.91	1247.416	1094.632	949.5095	37.04533
ANGPTL5	63.35969	37.96629	35.05066	37.23849	37.83578	39.06325	36.95552	37.49585	40.95508
FBXO22OS	75.7439	65.65298	54.06237	40.54353	54.011	44.50337	39.03173	54.27655	35.78988
VCX-C	51.91755	36.6636	54.80359	52.14985	122.1454	49.87377	79.15412	65.74518	43.77891
KIR2DS1	113.8416	45.19521	58.02004	39.22672	40.74518	53.68378	35.11481	35.41608	49.01348
RPS4X	3272.447	2693.439	2681.359	3266.204	1746.21	2465.848	1370.185	1325.593	2526.631
ENY2	2378.365	1732.885	1992.038	2509.453	2377.491	2213.294	3220.049	3093.018	1201.242
BBS12	41.80175	51.91589	42.65206	79.24905	89.66552	45.41223	178.0735	147.7947	55.60906
OR2T12	35.7439	34.85862	38.76741	43.20206	34.16491	35.29854	33.03796	49.2841	47.71753
CALR3	36.84752	94.13917	41.66791	55.07724	56.45791	62.44339	36.0707	50.66475	93.09614
FAM151A	157.403	102.1861	77.80074	346.5203	87.0547	126.4574	64.23358	71.31564	225.443
TBC1D3C	45.72344	34.19635	39.24714	38.31415	48.70476	43.09802	126.3981	36.55861	53.39953
MR1	1323.381	1504.508	608.2902	740.9884	875.1916	1345.422	621.2313	668.8802	1053.003
ASPHD2	74.59585	91.28197	54.04499	55.51637	90.79573	95.1272	52.20643	42.76273	259.3661
NACA	712.9984	788.2619	1017.031	1253.356	1178.015	653.7844	945.8053	613.8232	1450.604
NIPSNAP3A	161.1654	125.4706	50.53008	150.1052	37.848	84.40237	48.59145	44.26023	257.0918
ZNF391	55.84041	39.77258	36.27219	38.04185	37.40393	40.80889	34.37439	63.9518	32.89539
TM4SF5	39.47557	34.65504	34.79025	40.68236	49.68279	37.81165	35.46838	44.17521	44.93624
TRMT5	52.28751	63.54089	39.78384	48.2617	32.82078	32.36655	57.86349	38.11589	41.55313
FLJ42133	45.56869	55.83537	66.1151	67.23537	53.28657	64.50418	51.9567	79.74056	65.82118
C11orf76	34.76692	34.04642	35.15136	31.7153	39.86828	31.70577	34.29051	40.55504	68.30406
ASPN	593.7386	255.8406	446.0986	295.434	1100.053	1641.526	792.7403	373.6957	447.718
HSFX1	1581.625	1550.013	760.7333	947.6506	417.1627	1055.239	422.2054	215.0255	1535.428
C15orf56	36.00314	51.23237	33.29947	54.81079	42.1298	36.74832	39.95877	33.22115	46.13493
EPOR	947.8695	447.2484	315.3501	67.04331	490.8946	402.5417	491.9931	328.4016	65.96561
SPATA8	93.62302	39.39391	46.11701	50.49364	45.04934	43.93758	47.51123	43.75648	81.09702
GSTA2	59.15791	38.53769	76.17712	43.5969	38.30352	48.89486	35.09464	59.63074	47.5352
BAGE2	40.16319	50.73248	52.82128	41.26569	41.97275	53.88135	50.3931	36.18308	91.53918
FAM47B	50.58179	65.58749	60.41491	41.74535	39.0725	65.59677	39.64597	32.87231	34.65675
TNIP2	995.9862	262.8755	100.2512	597.5143	215.5341	148.7226	150.458	158.6329	829.0417
JOSD2	68.95165	732.3242	415.9746	864.8527	341.3652	211.0485	249.5661	372.6758	2245.598
TNNI2	9708.768	14154.09	8815.31	10400.03	6240.706	10671.18	10274.58	16013.78	13056.29

REG4	36.73841	63.66709	35.15485	30.70173	40.41779	43.326	37.32024	37.72616	39.17683
ITGAV	1683.795	1709.929	2097.297	3743.263	1093.032	2325.19	2214.595	1871.407	2277.364
SCN9A	49.43114	366.6596	196.2878	124.6358	666.3307	98.10355	421.1348	155.9585	170.8703
PGBD4	51.94268	42.43414	40.29812	55.11094	45.22434	63.15952	44.24178	250.875	48.90807
SEMG2	61.37107	44.53349	55.29922	44.26069	52.85641	48.5575	58.86649	64.87527	50.39078
TPSAB1	1423.397	2285.388	1507.49	47.32419	357.3931	603.4498	658.1122	524.4125	1585.62
CIDECP	54.19715	296.4713	43.07997	38.34641	46.05152	61.38292	51.8555	42.3821	35.11485
PMCH	38.73234	45.24942	54.68739	309.1353	34.61588	38.1697	37.68919	55.29044	32.22662
VCY	252.6838	166.366	77.18772	82.403	55.54092	83.68325	62.10443	61.49773	95.69687
RASD2	12260.88	14009.19	10118.66	12591.33	7216.688	13660.43	9356.514	6893.554	12890.91
PTPLB	13811.49	12361.07	9078.419	13386.72	8392.382	11715.02	7293.388	11534	13773.58
PCDHB18	54.09168	497.0615	377.3467	40.78943	209.6736	127.259	40.2482	149.976	71.96404
LOC389286	9903.076	8694.58	4538.96	7001.646	2074.126	3158.668	3391.096	3517.858	11622.57
LAMP3	376.8312	557.6803	737.7374	545.7083	149.0314	561.2968	344.5068	557.3518	524.3779
SNX10	54.61761	378.692	216.6419	41.05666	114.5213	38.22623	357.9656	73.74667	38.6428
C21orf45	345.3083	225.4242	174.053	280.0594	111.1798	196.8393	144.299	159.0898	287.4859
PFTK1	137.3571	74.40425	70.26434	92.14178	41.25742	77.52785	59.18264	52.45415	174.3666
CXCL5	56.5034	41.90005	46.29802	41.79463	84.31113	50.34721	64.09637	46.4522	35.91016
MT1P2	64.41316	37.51026	41.99594	50.29875	51.10949	36.02117	38.52757	47.73673	47.74231
SLC1A6	74.89075	65.8031	39.19775	44.02207	59.7379	81.74344	64.28241	50.40887	89.16222
FAM55A	41.31667	35.55854	55.07438	67.73101	36.08462	53.29768	41.8086	38.39202	35.36112
SLC6A1	34.32735	51.73432	106.9724	33.26387	53.96419	183.4456	56.32813	62.0946	68.04044
LOC441257	49.01717	57.98606	37.19326	38.30444	42.91019	73.23519	35.16196	41.67186	61.14345
ZNF650	109.902	110.1516	37.80565	175.3149	76.34593	51.19069	61.73106	82.60052	156.8703
CGB2	39.34291	33.48989	35.55004	39.67566	37.25483	49.35954	31.71486	35.32254	40.72533
SPATA22	48.13605	44.83721	81.06042	41.89161	41.96107	52.7694	50.16509	51.6808	65.63269
NP	2383.156	3739.474	5018.687	3186.64	6161.46	4227.211	5860.42	5695.733	47.29515
NUDT15	39.70656	468.7023	809.4578	1504.661	2362.488	1963.429	1876.753	1359.073	1252.866
SUMO1P3	73.14349	52.42349	48.04278	74.26579	58.01794	67.86543	60.76976	64.09937	65.19275
TMEM183B	555.2482	362.0258	237.1214	421.4694	178.7209	277.6274	210.8056	241.6115	737.6939
FAM19A2	56.23429	36.79093	40.62219	58.80435	39.5466	41.26726	35.36614	38.35914	40.31998
LPPR4	458.725	51.53272	119.5378	46.40363	311.1374	243.3698	209.0945	132.4892	35.76015
TRIM74	48.77116	61.58086	48.42217	58.52973	35.81768	47.57541	109.0901	49.08161	38.28017
EXOC3L	51.33011	37.52941	33.92555	59.61155	40.64944	55.02002	43.14737	46.20031	79.34322
RAB8B	1349.075	2358.481	3767.046	3110.533	3426.307	3737.344	3393.39	2916.474	1175.535
FLJ10916	9069.641	8548.033	5743.904	7944.125	7342.903	5936.265	4875.596	5576.222	8851.721
GPI	11333.82	10198.85	6341.626	11790.21	6459.981	6619.993	5477.383	7763.729	11945.85
FOXD4	338.2753	196.5291	102.8216	235.9311	358.0302	156.3691	143.2283	113.9882	477.8808
MT1E	4303.48	3489.614	5269.288	5325.396	3584.891	5104.127	6255.444	4371.667	1967.891
TBC1D3	86.96667	83.81345	47.87664	50.33242	48.53751	59.56169	40.64478	33.74918	50.21429
DEFB114	85.70325	50.2895	51.24237	90.4978	53.21526	51.23599	46.84785	62.87922	59.02244
KRTAP12-1	47.90036	48.89651	135.3167	41.43494	42.5604	50.83273	40.16302	52.14746	45.9543
FTHL3	2366.492	1142.246	1541.892	1324.697	718.049	846.2932	3820.604	1385.152	1609.898
C19orf40	62.47719	52.84612	35.59807	57.81531	36.44806	37.29353	40.46431	44.1099	72.94489
ZNF14	492.8535	287.8396	151.2859	400.6632	155.9152	263.0959	173.2604	128.2844	638.0522
MYO9A	705.3144	125.4647	76.86053	176.9087	253.3598	301.3992	186.7814	147.4563	209.817
RTP4	138.8644	46.68421	63.4911	98.04041	68.20695	83.27558	58.76191	118.8186	122.1628

C9orf36	34.96534	47.6318	42.19191	49.68473	35.8581	39.59158	35.46121	40.10553	36.6466
ZNF133	161.142	114.5718	106.073	132.4913	1077.691	474.1264	395.096	588.7104	248.3688
RBMY1A3P	53.49912	38.89567	47.32121	61.467	59.45162	66.58083	38.97245	66.73372	53.6606
DNAJC2	682.1361	889.613	323.7366	1476.479	205.1822	335.0721	172.9708	431.9749	977.414
ELOVL5	32.69354	372.0705	124.8788	32.64078	237.3788	178.8655	192.6481	120.5871	32.88203
MMS19L	2786.846	1796.023	1991.893	2682.136	1541.202	1581.346	1521.153	1934.368	2706.45
HNRNPA3P	505.6325	276.4785	183.0351	365.6978	174.3062	255.972	177.8623	218.4938	710.3826
FAM155A	44.35395	33.03064	46.52114	32.82541	40.91698	33.40872	43.11836	56.31016	41.11681
C21orf129	41.54577	48.4757	40.37832	47.61593	336.8734	46.23395	184.1827	324.2913	44.50395
CD300LG	240.8615	129.5165	98.9237	190.0146	108.9931	134.3428	85.9658	105.8257	243.2342
XPO7	226.5875	153.6437	83.4008	124.0073	95.27254	138.1373	72.1629	117.8438	460.9442
C2orf83	151.1532	63.34265	52.21091	68.9101	47.28104	56.27139	43.46525	73.24436	56.32046
SFRS4	104.2266	499.9053	2465.63	277.2151	3403.583	2116.014	3112.562	1154.856	61.35768
TDRD1	145.5575	174.7645	87.93031	109.6542	52.85233	62.33554	57.50084	64.76257	54.17284
KCNS3	63.65755	115.141	142.2799	37.34283	200.2579	51.12357	135.8732	64.09031	45.20475
DAZ1	32.24847	35.80357	38.70857	36.44944	34.61636	38.05236	36.92263	44.53667	54.55207
TMEM11	45.17202	282.8034	243.7216	312.0798	285.2924	42.41504	168.9677	159.96	46.65235
ZNF767	7662.973	6840.772	4971.566	5076.106	4298.634	5640.234	3498.449	5819.136	7553.621
CYP2A13	309.2375	131.5076	49.5178	281.9845	41.65549	87.11809	56.2506	75.87303	501.1791
RSL24D1	5011.02	5951.625	6166.23	6663.299	6102.307	7067.347	7108.632	6084.339	2174.929
VDAC1	873.4685	285.708	292.3807	809.6362	218.6409	460.4132	310.1287	511.8357	634.7459
NUDT8	319.8509	182.7068	105.7685	194.7387	104.4731	162.7247	81.73315	106.8908	572.7249
NDUFA11	330.3891	187.473	99.12082	370.8293	84.39613	88.36525	76.70161	103.8281	433.2374
KIR2DS3	34.12458	39.49234	40.22093	40.01287	42.63693	37.4853	35.15777	43.12655	38.70924
ZNF550	118.1312	135.8138	74.67443	1169.565	70.08366	114.268	159.7224	67.57101	187.9799
ZNF826	85.20594	97.74892	88.50184	58.48991	55.27862	64.98924	58.00315	49.17444	166.3615
HNRPR	1129.267	965.0524	1173.235	1770.837	1025.6	783.0799	1003.141	973.2729	641.1923
ITGB2	10028.56	10520.85	13207.95	9051.955	9068.996	13124.38	10797.93	13480.23	9045.267
FCGR1A	47.09019	37.23812	487.3251	45.87862	254.3066	41.29708	350.6971	537.0922	48.75169
C20orf72	846.647	3745.356	4005.587	4823.735	3300.917	3959.04	3867.652	3413.542	2810.197
COX6B2	7393.875	3812.341	4460.627	11672.7	1594.276	4383.363	5823.207	1815.86	8437.811
LOC285735	82.03342	45.18884	45.79425	46.93113	50.77595	35.61918	36.66415	59.66238	45.05722
KIAA0895L	33.35814	42.74618	43.36296	38.82177	36.14348	46.25895	118.5442	50.81316	41.70792
TRPV5	185.5435	185.9983	81.60917	100.1316	59.74116	110.097	53.32542	70.14206	186.4075
SEPHS1	1391.812	2747.332	4000.596	1167.778	4290.077	5283.627	3531.827	3194.396	3119.278
ZNF69	288.5482	138.9562	84.97349	185.6204	60.39888	100.4047	61.85978	76.66105	173.5955
PELI3	198.9747	2385.258	681.7521	2987.788	870.4809	1154.59	646.9555	1005.238	3397.543
CUL5	874.4375	1944.108	1935.574	1359.971	1259.675	1903.249	1124.137	2301.56	1095.061
LOC200030	1960.266	965.087	378.5154	1399.48	463.7749	214.7685	583.4598	214.5629	3278.763
DEFB104B	68.25751	68.20866	44.07594	59.19181	45.83219	63.79132	48.65044	53.47099	69.03617
TTTY6B	70.91888	56.93136	53.16365	75.3262	49.3267	42.72671	37.44159	49.19637	42.41284
TBL3	36.24695	37.59646	33.69403	32.80539	118.7252	39.20536	89.03053	40.60137	930.7597
ZFATAS	33.32319	38.17854	33.78686	30.70893	38.85464	34.36995	33.73126	35.54938	35.8988
FLJ43806	59.45446	42.06364	54.89904	77.20435	53.80811	73.45195	43.63403	204.2165	152.7941
TOR2A	681.3286	574.2711	199.9973	554.8608	308.6165	482.0765	294.911	363.3167	881.1082
KCNJ9	35.68385	37.34266	33.22282	36.17567	50.51068	49.82439	40.25264	36.28473	36.8772
RPS19BP1	38.33326	38.76756	35.77104	37.9216	325.7214	59.62515	241.9721	276.5652	70.4752

ELA3A	52.13524	57.29142	64.29645	40.00983	42.65956	52.31176	42.75253	41.58155	51.06796
ZNF131	479.4634	579.7408	210.6465	471.287	223.2932	191.6841	239.7358	129.1001	771.6758
ZNF43	58.05149	75.96702	127.5463	94.1897	41.30696	42.52898	72.92489	41.04223	49.1036
KRT86	35.95503	42.05877	159.138	38.08576	33.40328	48.06433	146.5954	239.588	41.94314
NAT11	35.27583	33.45004	34.9676	35.7169	33.25597	33.4333	33.30566	40.12474	69.26237
CYP4F2	388.885	567.6064	87.14423	115.6292	197.0459	85.13511	74.89052	222.8013	373.2624
DMRTC2	125.5026	47.74427	36.91579	55.00468	48.08038	34.74541	35.34906	37.33667	64.40247
TUBB8	48.98564	43.64633	44.12041	60.32583	37.73768	39.20889	45.70135	62.57225	74.32299
NDUFAB1	8965.856	12160.56	16896.34	12471.67	21376.81	16479.04	20060.29	18700.24	9646.754
CDRT15P	75.92651	47.33758	61.2747	55.44348	55.10619	63.24988	48.33505	68.16248	174.8842
LRRTM1	37.53864	40.81209	68.10087	60.59121	48.53516	61.53014	37.56867	42.97651	147.1465
LOXL4	8058.844	6832.385	4550.29	4118.3	7020.596	9873.445	3452.398	8082.236	8251.399
PYGO1	45.34469	46.73606	52.45898	46.23417	37.43098	38.89389	37.87741	35.189	56.39672
TATDN1	1158.005	774.3306	721.1985	1047.85	593.5824	1018.498	927.9279	550.5005	665.4182
C4A	810.9194	295.6821	116.2276	384.9649	79.41133	85.93719	62.7124	99.84657	423.5561
SNHG3	5567.034	4578.512	6203.167	6373.73	8814.322	8194.841	8401.983	9788.942	4060.668
PRELID1	65.41161	119.8209	139.1805	85.63831	145.7378	56.17323	186.1396	165.7257	87.32357
C9orf61	40.0733	559.9205	704.7559	34.32156	2160.339	659.5087	736.629	761.7698	45.00504
C16orf93	6688.173	6888.474	7944.109	7365.456	5761.971	6602.078	6036.259	6281.077	7635.676
HCRT	1193.901	734.4823	289.2003	250.9492	136.4989	418.2427	131.6311	339.864	893.8463
FLJ20184	90.47475	63.90576	58.86344	51.33855	68.4346	80.83993	60.04668	63.76222	66.41611
FLJ45974	76.39629	49.60679	55.4881	94.23768	52.24924	44.97422	41.2079	56.87571	48.34455
OR1G1	49.57022	66.84155	47.54906	142.566	48.62702	69.21968	55.69279	58.03376	50.50204
SLC18A3	36.07436	36.33164	36.7028	32.17991	32.98756	45.03223	33.05772	45.29399	40.53894
C12orf24	73.04301	50.22087	302.005	55.86174	332.5372	299.0818	320.1618	310.2107	99.92214
KRTAP5-6	37.52777	98.15579	36.35984	39.90248	38.12305	68.97115	35.00378	35.16787	49.78647
LOC72960	22969.76	25707.71	23722.43	21275.44	21535.2	18754.52	23164.76	21375.52	24795.35
NAT13	279.3151	259.1718	249.3726	355.0714	347.6581	344.6316	173.1824	275.1116	1015.891
TMCO6	663.883	114.5117	241.8384	493.6781	567.8341	669.6942	410.1907	327.8856	876.8375
MTPN	6695.631	5558.315	6924.083	5153.872	5985.995	6598.401	5580.55	7807.126	5151.83
FAM168A	50.99666	50.16864	50.94044	92.25915	54.36691	52.13673	49.78624	49.12061	57.00147
MEPCE	453.9602	688.671	837.4131	532.738	815.2112	780.1999	684.0319	1402.113	907.9574
RPS26P11	33.60687	528.1085	34.88224	32.96722	131.0798	31.38243	114.566	42.19988	45.51435
FLJ21511	43.17707	60.96134	46.46012	36.58357	36.36607	38.49477	72.56	47.68237	35.77545
GTF2IRD2B	3557.363	4690.796	2374.964	2629.848	1024.669	1303.037	844.1299	990.8069	5968.689
VPRBP	248.9285	273.5052	180.4544	184.3053	111.6269	104.4586	85.47129	80.38091	223.5371
NAPB	34.87458	43.19512	401.2872	40.43922	665.9443	562.7927	625.0906	572.6992	35.02235
TPI1	3532.408	2850.446	1908.357	2968.731	2176.915	1665.172	1758.846	2823.096	3731.438
LOC64994	106.5394	125.8708	175.3133	194.1577	114.9114	144.519	155.0269	162.698	39.34354
SPACA5B	60.20871	31.42147	42.7651	52.21325	35.38244	61.89355	42.64328	41.24072	86.20148
SPC24	4899.193	4167.625	8821.157	9619.215	5027.348	5150.135	7877.778	13541.29	8688.467
BCL2L13	36.23773	57.78425	234.0916	47.81831	274.7888	49.46606	328.8222	338.0292	48.19844
ZNF611	42.34544	323.29	186.3893	35.68013	39.3933	37.55017	107.2379	116.8426	36.76088
MAGEA1	127.0989	167.0221	568.4274	167.3123	68.25357	94.21418	71.05502	93.02485	221.402
SYT14	66.05596	81.58628	56.98949	67.23523	57.29775	57.09296	166.2142	154.2818	133.7886
PA2G4P4	114.3569	149.8936	49.98212	129.9717	72.1454	116.2475	58.91113	84.95034	347.8054
BEX2	40.3731	32.74072	394.9561	91.86327	1059.884	53.9008	675.5762	162.3517	63.30184

MTRF1	468.6065	210.6572	215.7983	347.5426	240.4286	337.1256	278.3977	238.9681	58.44611
HCN2	42.16026	37.13255	39.38029	58.77892	38.60999	35.07625	41.66919	35.55377	41.55396
C2orf32	69.20786	39.44538	67.69138	49.55506	48.79171	61.279	41.51241	39.69965	63.34927
ICT1	2329.089	557.2879	52.92453	869.1104	490.5731	430.7848	411.5596	821.5372	91.01948
FRG2	33.30762	41.78348	42.72223	41.51364	33.17088	48.64429	36.24552	50.17451	63.75322
PINX1	231.8831	150.6345	85.83916	179.3752	126.0296	507.2803	147.8971	101.9243	421.3644
BIRC2	9839.996	8787.347	10349.5	19836.91	9129.941	11534.29	16513.95	7193.877	7446.88
DDX1	309.7631	359.2086	229.8206	198.2534	1734.346	518.6694	1566.716	687.7422	387.9683
ALDH1L2	154.2354	424.1491	79.22514	341.5113	149.2581	83.21899	150.5072	94.53107	77.71106
THAP4	52.66196	38.55014	33.7344	34.90239	33.30989	568.6802	33.46139	34.5598	36.05374
RBM7	33.20168	38.05733	51.87511	36.86208	55.84195	90.56429	52.08343	41.04246	46.88916
SCARB1	134.0145	123.9476	309.8245	449.6633	533.3958	372.0997	429.233	508.2641	193.9486
ARMET	10998.25	11343.43	11221.24	9071.582	11586.25	9569.375	10593.44	10427.63	12642.87
C10orf91	49.08835	52.3774	49.76317	279.8905	40.2709	186.3889	80.32658	55.38591	127.29
LIME1	14926.14	15999.6	8782.982	10778.31	8406.34	11689.88	7937.59	8566.647	17518.25
PHYHD1	52.0885	40.17971	56.34509	58.64994	51.79612	41.71673	110.1411	51.17075	88.84549
TTC12	1318.563	35.42595	208.9352	1206.387	38.19054	45.16422	199.3391	99.77764	60.31428
COX7B	281.2547	253.1655	268.5984	347.6534	537.5807	270.1406	384.7342	532.7137	304.9259
ANXA1	5050.822	3229.039	10587.8	4173.897	17942.98	6524.707	11823.79	8269.816	3513.041
FLJ16793	164.2209	159.4354	98.55204	164.1613	94.87808	89.9571	76.03551	85.33371	343.5685
C3orf52	134.4883	47.37083	107.0605	381.8849	201.3055	60.66848	246.922	423.6564	45.08213
NOLC1	40.76377	300.2983	649.2725	69.07285	643.7919	357.0929	885.8273	502.1176	41.55174
FAM154B	35.79375	61.55077	57.82481	34.56897	46.42648	174.0682	47.12843	44.59919	56.15177
LOC33924(	96.48835	404.2078	166.9995	63.38326	231.7516	253.5017	329.2765	610.8853	135.7852
C14orf39	37.58757	60.51329	50.93412	82.6638	43.20977	204.033	55.18	52.38544	56.21365
ZNF461	47.85197	78.5872	47.25061	47.4213	41.61699	38.68018	41.87983	50.24087	139.514
LRTOMT	37.60849	1752.346	2071.775	1502.855	2594.528	1745.646	2300.547	1637.319	45.45927
C16orf11	102.8671	68.50535	68.40648	76.66432	59.60213	100.6895	60.76249	49.34917	171.4461
LOC65386(	49.111	81.15343	36.86541	48.44996	40.86916	38.1595	34.00891	38.55913	74.83138
C17orf77	38.19409	37.06104	201.4339	35.83524	35.12722	52.73352	34.1659	49.32676	34.26963
PGAP1	325.4379	218.1954	115.0982	219.4629	108.1311	113.0999	121.3137	45.74538	463.7698
FAM159A	43.61692	49.71835	39.40145	43.53071	40.2052	36.13328	116.5974	38.52063	58.78439
BRSK1	10877.38	13173.9	7256.042	7275.417	8780.461	9546.839	6193.012	7707.035	13835.3
DHRS4	88.9397	795.6481	1857.162	63.9194	2458.58	1206.836	1484.442	3820.499	107.1712
PFKFB3	45.9507	56.93248	701.2173	1660.616	316.343	318.3653	511.5999	484.316	44.29111
MCART2	71.73312	51.55877	51.8918	51.01867	43.37245	51.34044	50.3111	46.85037	38.42296
DGCR6	39.35254	50.13022	189.8955	51.60666	117.4269	37.21713	57.25794	85.07652	38.66586
NCOR1	2818.894	2913.862	3627.831	3292.151	2368.451	1602.585	1915.542	3993.949	3544.864
RPP21	4537.837	5379.813	6121.132	5065.337	4999.444	6452.005	6488.871	5988.273	5182.575
ZNF485	251.6252	114.6706	98.73116	141.2587	73.39286	83.45991	54.19809	83.25027	377.4766
IFNA7	58.14608	63.56408	62.04755	91.30344	71.80282	75.69283	46.94296	69.51537	52.2215
TSPY1	34.4917	33.82443	29.35011	44.00737	31.11945	36.35618	32.68871	39.49681	37.03432
C14orf131	325.8481	1616.611	2915.794	1004.024	1433.216	144.4462	2922.394	1375.379	357.131
FLJ10213	67.25536	1262.234	2799.633	1954.696	2705.462	2698.664	2569.953	1685.504	1515.202
FOXE1	71.75021	1161.721	778.8173	1301.85	3865.508	3204.317	1974.472	1739.221	39.05539
ANXA8L2	2069.38	153.1854	1338.11	5065.406	972.1182	896.4492	2318.108	2205.842	144.3738
LOC93349	704.9141	1433.533	771.3799	1527.09	741.7141	1129.452	1178.959	775.2809	65.47098

C1orf201	331.0982	198.3403	110.6146	207.1872	89.0963	171.2269	97.57637	112.2688	398.2793
CSRNP2	1103.595	1387.69	2497.551	1296.282	3354.138	2391.035	2835.844	967.6969	48.12463
COX17	36.37708	35.14277	712.368	37.20502	767.8826	41.43195	288.1951	1285.881	40.67791
NOC3L	3929.242	4754.271	3683.959	4956.822	2582.88	4636.21	3663.559	3215.915	4363.145
ARL16	3333.145	4642.42	2280.316	4102.147	1554.516	1695.33	2120.93	2062.012	5615.378
PSG2	58.15416	40.73065	33.81066	51.47213	35.92198	46.10301	37.17058	41.66308	49.00452
TXNRD3IT1	52.67976	52.11917	62.575	47.2762	82.31545	67.10728	41.63992	52.60341	191.0087
ATG12	14649.13	13822.31	11921.99	12470.06	8740.761	9903.356	8076.96	9485.92	13896.28
IL17F	89.24561	97.13254	52.97234	83.54418	54.54045	45.80118	49.06379	46.82779	150.3287
CYR61	75.99284	79.67213	198.8759	535.7537	782.4146	305.3769	463.5292	56.79911	145.3702
XPOT	235.9094	131.9168	204.3951	626.8216	257.2066	100.2359	174.478	95.61994	89.38076
OR10H5	68.06034	55.48795	42.73901	54.75475	46.42502	39.07186	40.80762	55.5626	178.2671
FOXD4L2	37.91539	50.74604	39.60971	34.72004	50.71582	62.67639	38.62908	36.50687	77.31083
HIST1H2AH	3999.073	3104.13	1189.737	4798.403	578.4958	1491.444	696.7231	1070.673	3367.432
CICK0721Q	593.2913	354.0876	306.9074	565.3278	135.679	221.8536	259.9684	188.5652	787.0245
GLS	43.85504	1180.084	1316.988	624.3818	2010.562	1805.5	1738.207	1430.028	1008.111
GDF15	89.66491	84.92962	66.86148	108.407	59.38063	68.14038	104.7532	106.0472	247.2174
IGFALS	252.6473	167.1639	94.09261	140.0568	85.29845	134.9013	71.28259	67.44967	194.0522
CTGLF1	168.5794	151.8171	73.95683	121.3563	64.83321	104.3658	53.28788	79.79344	132.9707
HDGFL1	68.24459	105.025	35.05756	65.623	56.15957	80.32494	52.46862	61.43542	78.07191
C15orf38	5702.459	3653.719	6227.265	4749.369	3701.419	5862.358	6125.622	4973.055	3778.863
MRPL20	31.7683	41.36089	33.9058	38.26058	36.2002	36.42059	33.06584	38.50985	39.19707
SHQ1	55.39349	43.30078	61.96764	354.6954	77.9107	197.0047	114.1638	75.71262	1163.826
FAM122B	68.18951	45.88509	146.2329	37.95279	143.1668	100.6938	135.7206	111.5845	69.73071
HRCT1	33.00227	49.82047	49.23989	49.60819	163.5354	54.57158	236.4225	432.913	223.341
CYLC1	33.93457	47.07971	43.98468	44.51369	34.87708	35.97017	33.24079	37.19867	38.50335
SNORA10	18912.96	15943.1	7142.042	7042.143	4375.327	7932.966	6214.796	5265.739	20803.68
FLJ38482	53.52245	65.29636	54.59885	46.49644	462.4176	56.58238	163.4522	58.44186	62.11039
FCF1	41.28749	1911.061	120.7551	55.14688	50.90504	51.41549	149.4639	134.6165	53.10845
RPL36AL	833.1847	1299.888	1160.955	1444.044	737.9516	665.5074	594.3429	971.8546	937.5554
MRPS35	96.91095	284.8397	54.35983	329.8318	76.48701	65.11044	77.49726	40.76892	90.21644
VAMP8	4351.571	3540.882	3654.12	3762.369	2845.049	3151.657	3188.659	3977.113	3761.288
TTC23L	188.2542	132.9864	62.99407	131.2367	79.61464	87.7896	62.0911	90.79137	383.3026
C1orf91	217.9009	96.55851	49.05618	35.67535	46.65002	342.5052	82.2383	53.78446	46.29008
ZNF83	666.1211	659.0977	1097.328	394.4993	461.3481	395.2258	613.2883	260.9913	768.187
GGT3P	151.4123	104.5775	67.5703	254.0669	79.78064	112.7969	65.69657	74.00494	216.3553
XAGE2B	302.1815	166.6288	86.49161	200.0813	88.85744	143.4211	79.44006	90.69743	457.7396
FGD6	41.32759	53.49617	35.10357	34.923	41.10256	37.33206	55.00286	38.38223	38.98977
LRTM2	85.45014	59.74279	70.44579	120.5914	72.57349	111.2366	53.70681	68.25722	50.9144
CRYBG3	46.28591	75.6736	183.3213	46.92624	186.7984	46.52709	44.29849	53.60813	85.85299
KIR3DL2	131.5349	121.6697	87.58674	63.07307	50.0858	740.3914	55.03501	64.66893	188.3008
PPID	642.0051	1964.972	3510.994	546.7283	4194.32	1950.666	2537.057	4574.25	5798.734
KRTAP5-1	172.5828	145.5713	79.14701	161.8601	146.7505	115.1329	67.71815	100.9555	278.33
DUX3	93.67327	35.39386	174.3169	53.81673	49.71225	37.76823	40.71045	38.07173	38.79416
SLC30A4	5965.618	5264.398	3602.596	3753.606	4217.988	3056.831	2475.376	3008.162	5341.79
CYP2B7P1	41.19693	55.26824	38.71326	42.41242	42.50025	36.74554	133.7979	47.23548	96.64268
FAM21A	604.8389	349.847	307.9538	50.8843	249.6288	34.99847	322.5104	112.3024	53.8563

RPS4Y2	141.0882	55.68364	60.1695	71.09013	53.57336	65.81563	52.9892	50.25877	49.61992
POLA1	218.6603	75.54675	116.4264	148.6784	126.8802	175.147	116.0486	97.88459	322.4925
TUSC4	748.7171	1270.523	1950.168	600.6657	2087.266	1591.756	1977.594	1105.351	102.5624
RPL37	232.2133	102.5281	95.12907	251.9906	84.77895	54.76049	73.84931	87.59294	395.7819
CHCHD2	2269.439	2961.367	3612.235	3493.981	3137.202	3400.608	3034.169	4163.662	2603.418
LOC440396	470.9692	1231.557	418.148	220.633	262.8926	334.5308	272.0946	555.1963	315.6103
ALX3	80.7284	154.8741	54.0473	128.3475	49.15624	64.51107	56.80333	81.34541	99.67056
OR8J1	48.384	36.05719	54.55978	41.78204	34.95305	57.08048	34.88869	60.42932	38.33403
SPRR2D	756.9333	485.6809	3108.92	699.8183	8279.589	873.6487	2493.668	2527.547	2361.671
SRP19	1489.754	2452.235	3386.365	2220.386	3716.439	2176.88	3203.351	4552.396	3868.891
CARD8	66.11511	59.60744	611.2429	53.00099	172.1623	48.56565	309.7453	225.3556	66.12218
TTC19	622.4803	549.2797	202.4043	443.7397	266.6917	39.84993	238.1854	389.8509	674.671
UNK	37.21613	40.3877	45.47799	38.80901	38.05485	50.33887	105.0906	61.83539	35.60625
PRB2	134.3983	81.35992	77.64979	92.53484	76.81643	90.68781	66.89295	94.29547	215.5531
EIF3M	158.9142	121.1942	512.2502	1246.637	398.1819	495.5088	299.3777	327.6418	338.5713
GPR15	37.84979	46.66253	48.17863	50.64687	40.01455	55.27288	41.71341	162.8139	37.48893
C3orf1	768.4697	1371.583	1769.577	1732.214	1601.44	2571.707	1765.642	2282.212	36.72705
LOC220429	282.1605	88.97443	90.91758	108.7327	71.13538	95.70927	60.97136	74.66073	152.65
DEFA1	40.30134	35.94077	32.14865	36.42007	37.51753	35.30305	36.95132	34.0373	35.65401
MPND	101.4268	67.67516	216.1157	101.8225	69.29843	75.16768	184.1266	63.93732	238.2623
MGC29506	268.8998	2382.247	3397.068	219.6548	3370.25	2228.537	2404.346	1315.916	1912.758
C14orf143	112.6274	49.45192	195.0996	73.47579	126.173	84.51305	186.0597	104.5947	126.8354
TRIM16L	746.4308	2077.343	995.8381	1042.88	889.6449	1034.88	850.9496	893.2532	1061.997
OR51F1	84.50531	59.65446	38.69914	51.36366	34.77621	38.05152	40.14051	39.71221	51.75666
HRK	51.40471	36.25735	33.52212	35.7206	242.4736	42.51413	571.5131	248.9705	32.418
RAB42	93.87045	59.11393	78.64671	83.44458	51.52072	59.07842	57.3284	52.60173	138.148
LOC442245	108.9946	48.35018	62.65066	102.5563	61.12066	102.9129	50.14901	67.54011	156.2797
CLEC4G	145.3802	1626.24	1947.724	141.7801	686.84	1177.324	1581.001	742.1086	2801.549
IFNA13	67.36096	53.84864	47.00541	38.26493	39.67322	59.89209	46.65769	40.50102	48.72206
ABCB6	1210.06	2861.049	3677.119	2774.198	4173.476	2994.824	3623.813	4863.95	1230.324
RBMY1J	62.57085	62.40788	54.33051	59.41924	41.69119	43.04594	42.57008	62.42912	63.93704
OR2M1P	244.5326	105.2402	71.8149	82.43757	54.18217	99.79768	58.33789	85.3086	139.3848
ADAM29	86.19816	54.49011	39.42976	82.9243	56.36536	60.6909	46.31967	51.50926	175.3386
TMEM128	36.96159	42.84983	219.4492	345.9673	396.3132	245.3426	302.9801	37.92377	37.91009
STT3B	47.39368	39.18486	144.2746	423.1714	168.5252	40.50174	128.3367	118.3755	36.20122
PLVAP	2489.411	539.1791	923.6379	1978.852	864.6384	56.7024	545.7828	731.3235	2967.088
AMAC1L2	50.5494	46.92145	35.31301	54.10921	51.54407	34.74151	40.0515	52.72683	40.89565
GMCL1	1238.755	40.46684	480.2359	42.29767	451.3373	363.887	752.3904	293.9003	1888.387
LOC153328	215.0712	103.6319	81.59669	107.5601	84.44716	81.49413	63.70431	61.22239	410.7205
FLJ46309	2654.651	4285.987	1926.362	2035.889	1774.082	1914.849	1144.228	1450.252	4487.778
FLJ40244	34.86527	33.1202	32.43815	39.14729	33.92842	39.63096	36.59552	33.76049	33.10229
MRGPRE	174.4788	164.0478	79.54811	129.6588	80.74046	125.1353	74.57099	66.80195	147.0546
PGRMC2	15838.95	15180.72	11081.39	16716.67	12352.71	16171.63	11932.14	6580.299	15383.13
PDE6A	158.6456	174.4291	69.08785	146.2354	74.9437	105.6679	60.397	62.73157	247.58
GAGE4	129.5199	126.7176	92.45394	116.1158	91.22981	132.6715	65.80931	75.59991	305.3745
SUMO1P1	49.16349	43.03546	34.98475	42.49182	42.42831	47.74108	38.1962	35.60243	44.16083
C19orf55	62.1502	41.11591	37.97079	42.90052	46.32037	54.51306	112.4666	37.49514	35.10459

C1QTNF4	40.77516	33.56785	50.36909	40.45605	33.84134	45.48887	39.35831	39.95007	56.91095
CACNB3	8897.987	5525.193	8843.556	8756.869	9421.791	9688.105	8061.731	8264.356	6033.84
PANK3	329.1344	176.0036	190.4427	226.8831	111.524	139.7259	86.3682	203.5821	540.6175
PPARGC1B	67.61456	787.3936	76.00517	72.6802	64.39523	65.642	69.28976	66.59212	60.75151
KRTAP21-1	141.6207	67.87804	68.86353	135.8218	57.85263	60.90742	43.50718	61.18385	124.6269
SPATA4	396.2934	232.9242	114.5604	251.6252	100.772	160.5004	83.66704	147.5405	596.4274
RHOBTB2	806.7196	83.04724	178.8157	93.97881	129.2703	268.4147	162.0467	162.7731	369.7533
INGX	159.8073	88.60913	61.11018	117.1912	60.07759	67.69303	64.48601	53.90251	73.20009
C12orf11	121.9927	1024.022	364.0668	341.8064	451.7396	503.9931	478.052	514.7646	95.26245
TPPP2	172.7465	65.41821	52.50957	59.30196	57.49446	68.43807	48.73936	59.74358	39.42373
NUP93	56.16858	52.05104	880.0306	689.1712	1590.136	462.6864	2103.753	753.591	129.6037
C18orf32	496.8707	447.0389	375.3099	514.3108	285.8289	333.8967	310.237	289.438	745.4251
OR5K3	125.6626	46.98037	50.1083	68.14528	50.49286	80.56776	51.20888	41.45013	94.45844
AGBL4	76.14392	987.3824	97.74246	41.76581	184.3364	43.13583	33.54604	35.2239	233.3832
ZFYVE21	236.9089	196.8493	1706.922	1076.729	3300.814	2617.914	2536.034	2420.625	192.0731
OSR1	8706.888	8652.28	3913.54	2892.737	5987.858	4259.713	3317.217	2884.52	5601.397
EIF4EBP3	158.2946	79.98158	64.70591	177.804	43.99502	74.53067	49.41652	40.39485	78.87658
DEFB122	76.80782	125.2671	65.38987	61.91269	59.94041	60.79364	60.21155	58.06726	107.1792
RGS2	4780.865	7062.479	5701.353	6812.066	7200.939	6774.574	7003.304	5445.086	8120.622
ZNF627	5884.096	4802.104	3309.32	5220.645	2467.625	5221.307	3993.18	1143.212	4194.844
SPRR2C	1248.662	60.38195	3064.868	53.44869	7499.797	1538.275	1293.225	3448.786	1294.382
TTTY9B	49.45722	40.97829	34.37914	42.11992	33.06488	34.34394	36.21828	38.47611	55.87931
MSX2P1	35.48507	43.33982	39.05544	68.42395	39.37917	39.15089	45.53291	42.00475	39.19466
CTNNA2	196.5975	123.6302	89.87399	105.4133	120.7233	126.6342	67.0472	79.82979	358.5331
TSPY3	103.6455	85.27811	38.4741	45.86646	76.39141	57.88633	53.77938	66.08804	73.85625
HADHB	44.87826	1474.77	333.1237	1430.283	603.8868	57.82243	494.8736	410.7722	51.67996
KRTAP5-8	45.27271	39.84644	48.46047	38.68146	43.64047	43.68943	44.27917	40.46716	78.64207
CXorf12	197.5483	213.8711	380.471	239.352	400.9106	246.738	457.009	465.4189	193.6191
HGD	63.96975	72.8502	46.84892	54.17183	52.20757	68.73198	46.14068	51.29166	64.57784
SPANXC	70.7076	35.07335	35.59573	52.90777	38.06952	33.64954	35.7361	55.27514	31.08843
KATNA1	972.5383	48.02828	266.9097	39.50681	161.8502	48.20893	143.7766	35.84349	40.52172
MYEOV	39.39382	44.29391	137.1569	61.24497	48.70346	50.91945	65.87975	223.5204	50.05248
MCART1	38.12448	46.52411	903.8754	891.8636	493.1122	1429.584	480.6259	579.1279	38.27656
ARRDC3	166.9672	600.5555	864.3076	102.2349	85.62855	145.2589	143.4331	230.7621	67.55929
RFPL1S	374.1512	189.3173	106.7578	204.4198	121.1549	196.2413	96.97717	136.0373	536.8582
FAM22G	42.13261	36.35079	42.42662	40.87593	40.10934	40.00352	46.58986	41.9592	119.1334
FDX1	406.3644	216.0913	182.0141	245.8061	112.9036	189.7241	140.3095	206.4827	116.4193
OR5L2	50.28201	33.94458	47.8689	38.5881	35.84124	44.93781	35.07627	40.29384	53.16394
C7orf52	64.79057	51.96617	49.24379	62.3549	45.79916	39.24157	39.25227	40.9311	85.37466
INPP4B	52.85675	141.9309	814.9176	785.8102	413.2396	1022.576	727.3942	731.6739	80.20591
LOC40783[illegible]	201.998	98.03882	75.49576	147.1779	62.97095	100.7919	136.0115	81.54964	477.9268
TTR	70.17113	72.90695	56.08202	102.6665	52.9062	83.70046	54.88278	50.04908	49.39851
SAP30BP	220.4219	187.3284	77.01871	189.9387	455.2884	96.97717	338.6267	394.5348	268.91
ANAPC7	41.91822	47.94431	452.1022	35.69852	110.6746	47.16391	111.9272	564.0402	85.32257
C19orf20	38.11199	33.95612	36.92074	35.30445	32.1845	40.81686	33.79118	35.54766	76.25487
NPDC1	125.9277	41.38731	45.22596	48.72432	41.8921	42.15979	46.69678	58.37378	46.12454
FBXL22	32.8349	38.20484	31.78534	27.19098	34.77896	36.77831	33.89713	40.549	45.97284

VIPR1	24002.54	25396.93	17894.98	24835.98	30063.08	29698.84	29392.66	21089.37	22721.46
ZNF28	46.17678	76.62934	237.5434	57.51543	51.07461	69.64632	160.7523	57.58849	56.80196
LOC38869‹	35.22849	39.81438	112.1568	32.11295	106.1734	43.30502	64.37759	33.12328	49.91183
SCARNA15	102.1742	193.4086	74.68	64.82698	63.0549	69.81447	70.87979	52.59513	161.034
ANKS4B	91.06628	93.74784	60.55737	97.33079	60.6558	59.07578	53.93548	71.7555	106.4159
SLITRK4	118.7883	138.0898	75.31596	119.5472	137.0287	74.35706	68.58834	85.80914	297.4084
OPN1MW	58.9846	35.04907	48.35236	37.40396	36.64779	36.64455	35.61171	50.87164	47.63228
OR7E37P	65.88265	1172.455	685.0155	80.98117	42.98404	40.2674	161.8134	48.68598	38.84966
REV3L	1377.12	1528.78	354.5665	1528.521	419.9941	46.04866	487.9849	330.9693	34.09148
GJA10	36.63641	92.45638	44.08618	49.86058	47.42971	66.59204	44.90185	59.04742	42.31564
H2AFX	255.8146	118.3095	90.87663	171.2046	93.76434	127.0949	62.35843	60.00367	169.7981
NIT2	429.2408	237.572	157.9825	269.9271	316.9646	213.3177	265.1282	179.8843	290.065
LOC59510‹	75.7177	32.97891	161.4256	31.95248	321.802	47.06596	151.4027	36.82897	46.35228
SNHG5	65.58311	392.2004	298.8788	999.8979	399.8351	52.79122	455.6094	626.58	81.68835
OR4F3	73.67629	81.70613	59.97943	72.53223	46.51353	70.35708	39.24334	51.65483	124.8745
OR4F5	75.33618	52.29065	56.90547	57.72758	48.53889	56.48027	40.81596	58.93211	52.31048
HSPB7	41.00827	2819.418	905.2116	1251.287	754.1608	599.5955	2055.752	2168.824	3020.735
ZNF367	96.90958	109.2037	157.8935	101.439	61.90845	78.70624	132.8238	61.24529	196.2935
SLC4A7	1298.451	868.9131	1152.729	1064.207	808.3398	1122.418	880.2409	627.5945	745.7651
ZDHHC3	726.087	74.73811	316.343	67.83264	132.4127	61.2487	60.86662	68.53553	194.5924
ADAM18	50.67382	55.99606	35.79278	46.89638	34.21529	43.08204	31.94383	36.30154	49.5547
SLC37A2	43.48487	34.90367	222.2331	1420.436	138.159	304.4521	76.77115	322.3664	1764.439
C17orf61	4820.818	4270.932	5866.142	7811.162	5125.912	5728.668	5961.591	4842.414	4129.374
GSTM2	11505.33	7098.224	2611.188	2477.929	3479.38	2815.789	3281.259	1643.002	9918.585
CYTL1	34.05323	37.74388	36.59854	35.89137	144.8362	43.28288	37.14783	174.3269	45.83839
SLC19A2	855.6396	512.0626	339.9758	472.6598	230.7155	359.3276	218.6948	208.4444	964.8727
FSTL1	42.47802	47.55824	39.76941	38.51525	733.912	35.87617	429.9724	137.3089	37.19004
KRT40	42.26854	41.64402	39.5152	65.31124	38.61224	34.34302	36.83272	46.22459	43.21675
IL31	42.91848	36.12204	37.38783	44.58071	36.39082	36.47998	35.31198	34.14083	33.19436
TTLL2	48.07918	38.92693	40.66056	42.83771	41.83749	54.73559	40.58204	37.15358	52.98115
N4BP1	126.3323	929.6712	61.86238	947.0724	7446.13	3249.141	6664.464	164.3937	2626.593
IFNA4	73.46423	39.24421	35.16829	53.18428	46.97908	35.39529	43.09042	41.55134	43.00579
LBX1	37.82615	33.59503	33.71819	51.14002	51.84337	55.5527	34.16828	35.54845	80.15261
RBMY2EP	72.2632	74.04253	68.45966	63.49246	44.52556	45.95056	45.50687	60.2307	105.536
UBLCP1	2212.594	2814.229	3453.705	2852.133	3200.507	4233.794	3345.758	3178.693	138.7065
ELA2A	42.15299	86.38781	45.34596	64.64529	46.03798	43.94045	46.24675	67.24668	78.01308
C10orf37	45.14763	72.29757	67.29448	50.84311	57.41116	67.83227	51.89509	55.57507	111.9865
PCYT2	173.897	90.08481	66.20058	127.2249	86.32404	115.0594	48.57507	74.12149	147.9076
BUB1	37.93545	33.7963	121.0956	330.1418	142.0164	210.994	197.5109	127.4276	44.32113
TMPRSS12	391.1342	214.8886	102.3309	225.4007	107.453	175.1743	88.62074	119.6743	473.2056
FPR3	3395.63	1870.831	2085.681	1762.152	477.993	1465.746	1178.298	1159.477	1805.484
GRIA1	150.1202	89.40202	73.79179	140.6198	74.01391	79.70049	58.46554	89.9372	151.1147
ECEL1	74.36977	49.38259	58.51836	75.86626	75.41278	62.5056	54.98795	62.14128	54.70145
OR2A9P	36.98513	52.1521	34.52438	32.65095	100.2716	221.2454	78.01623	43.26724	66.48906
MYL5	48.76821	1509.648	990.7972	62.7725	1093.417	1587.767	1237.673	2212.314	37.27144
VENTXP7	120.0994	82.672	66.71777	86.18952	57.23148	48.2277	58.45725	72.84912	51.5709
MRP63	48.5741	690.0434	576.9111	108.1818	392.9124	251.0864	293.5188	641.4835	54.92668

SMAD7	276.2545	88.05429	74.89582	521.0947	74.70129	133.2763	99.54706	68.41657	344.598
MRC1	66.44327	45.85342	35.54242	41.28144	34.34448	41.97859	37.07146	36.53454	68.62252
HLA-A	7624.331	8462.886	11892.31	6246.612	6278.112	8014.094	13904.49	11751.66	5396.401
GAGE2C	64.91724	40.71204	229.8519	38.43997	41.37532	58.39825	36.59256	39.75952	49.44165
LACE1	45.93924	621.2436	297.9648	437.8669	283.2764	392.0576	239.6589	231.9682	671.9382
PPP1R2P9	70.1268	44.66522	376.1078	40.34225	41.00077	48.00414	40.16592	51.56487	54.86136
TTTY1	53.36717	61.0883	35.58767	50.88354	47.75645	68.38611	49.49847	51.55567	112.4594
TCTEX1D4	114.299	60.63402	50.7925	73.53886	73.76006	39.45259	48.03705	65.34169	59.17783
ST3GAL4	43.39356	282.7523	775.2787	38.16876	591.7645	58.11441	449.8217	398.0571	157.8221
NBPF14	1323.712	1151.1	1201.902	1054.446	854.5264	1237.582	971.1461	745.6746	960.0588
SEPP1	10565.71	10208.37	7496.399	6015.807	4731.344	6132.081	5399.492	7635.627	11657.17
FLVCR2	4857.562	1612.698	2491.737	2440.897	2714.854	1461.983	1395.154	1973.132	4168.487
XRCC2	384.2919	234.6006	139.2991	315.565	107.9387	168.5529	125.7823	129.0181	403.1871
KRT79	1275.93	44.46314	452.2491	64.53644	408.7381	664.2268	519.0826	382.9073	45.28793
SFTPA2	73.68393	53.18193	68.47913	93.46374	56.43757	89.91329	68.34896	66.81973	58.57632
RBMY1F	35.44266	41.41331	37.30887	36.95415	44.65299	57.14285	37.82174	31.39023	38.17693
ZNF699	263.243	210.6907	99.98684	192.1538	88.95422	140.2898	153.5559	77.52309	286.7399
LOC134997	50.15106	56.56487	43.91115	110.2608	37.9815	33.43336	39.10838	39.1274	36.52118
GPN2	14549.19	15458.77	14431.33	16699.03	11509.02	14598.85	16304.03	14688.9	14829.76
TREML4	50.7734	35.44043	47.43645	57.55353	53.08918	41.19618	34.77078	48.59735	39.13085
C6orf66	108.4014	49.86775	58.34708	333.2084	93.1292	63.12329	131.7593	70.49242	57.61925
FAM153B	6710.797	4234.84	2343.927	3078.436	732.7806	1472.789	762.1818	1320.482	5161.293
MUC2	74.91918	121.0088	57.30695	59.26961	57.10532	65.61669	53.77848	39.95763	73.53668
C4orf28	63.34234	79.3493	39.17741	40.44837	32.73566	119.1856	127.4755	676.8094	117.1606
AKNAD1	60.87452	75.26131	170.357	127.5878	145.1526	457.6251	224.3646	388.3643	107.2243
MEIS3P1	201.8286	38.76117	49.24982	46.85079	67.08507	61.8997	60.8351	101.2942	338.1235
SFXN1	866.8093	529.665	258.8416	754.958	48.82908	39.87133	144.9109	150.8011	65.85876
C10orf54	610.6835	1056.751	1247.899	507.7753	926.8257	1177.217	732.3687	1445.192	797.1044
OSMR	1506.624	233.8776	380.8805	276.6454	438.1369	166.9172	720.2929	278.8626	471.4623
OR8U1	52.5834	107.9686	60.5102	103.4522	59.46667	66.13439	56.89481	61.17913	148.5935
MMP26	108.4383	58.36649	37.8871	38.2426	39.62625	40.60192	40.21128	45.29261	38.45606
ATG10	39.37685	37.42126	34.79137	46.00945	32.41877	37.59919	106.0349	35.40019	34.02637
RAET1L	292.2581	169.9388	1110.783	134.0099	81.85676	687.7904	407.7229	1362.547	174.7731
BAGE5	56.86662	92.81017	51.88644	57.76812	59.59118	62.5085	51.65745	213.9165	103.0923
SHROOM4	60.08127	62.74296	40.00536	58.06953	37.20271	54.53966	45.27745	32.77753	99.96543
OR4M2	207.9927	96.82328	63.85273	99.47149	70.07923	80.87596	52.11837	64.60169	243.0372
D2HGDH	83.04929	63.39569	701.4438	107.3022	351.8423	1319.875	459.47	745.0349	217.9511
TLL2	39.27319	35.51774	35.60662	45.25285	38.7729	31.96498	34.85366	33.90503	47.93951
NDST2	64.08475	3236.737	3699.32	1758.247	2888.047	2831.624	3567.291	2980.192	2270.041
CTAG1A	107.9613	96.47636	58.56023	70.15861	75.55582	73.56474	58.56421	61.00047	230.133
UGT1A3	867.3645	147.8926	168.4898	464.3034	93.93245	294.9741	73.59437	98.23882	493.5592
TMEM90B	52.22315	50.09549	47.40034	36.88297	46.39668	66.53313	48.94899	39.75048	79.47078
UGT2B4	129.0632	59.73975	60.33363	51.30693	59.26686	62.01913	47.72227	50.06363	277.284
SLC35F5	808.4299	445.8116	509.9944	827.7777	669.4273	638.1732	994.3467	899.829	120.1731
PRAMEF9	221.5257	112.2922	69.56619	85.39649	61.78907	81.99883	67.87644	66.83733	224.969
JTB	324.0504	199.6754	3360.972	171.8088	4382.347	715.0508	3764.458	3320.599	255.714
KRTAP10-3	79.42429	56.56572	47.96254	46.96431	39.39429	43.76589	50.46818	56.26841	74.04168

BGN	21900.51	15528.42	13548.72	16086.48	14500.07	15423.67	14913.89	13324.48	16066.66
CCNJ	40.16129	39.89756	34.0387	40.69271	37.85408	53.50259	44.3266	37.84557	39.42609
SFTPC	42.21407	48.18439	47.31626	65.13304	42.61976	41.40801	42.1518	33.52002	122.441
WWC2	139.5309	55.35865	142.8348	54.82727	107.6441	260.5339	75.80238	608.5489	97.67592
IFNG	61.89087	47.50028	181	57.14367	41.4543	212.3473	69.7926	49.28499	62.4942
RABAC1	2125.302	36.59653	1427.673	2356.046	1797.474	1777.05	1979.104	2645.034	4260.302
CNOT3	2822.558	1826.666	478.9077	817.8649	448.671	463.7738	361.2273	370.3885	3672.822
DDX26B	117.7759	109.1569	123.3475	104.2318	133.7924	91.60988	125.4362	103.8916	146.3801
LEP	161.8034	175.3668	73.64021	160.2344	60.85283	604.871	67.25536	95.96202	254.3433
RPS17	9726.226	8121.138	6975.353	10119.21	5666.567	6059.724	5315.246	7916.423	10667.82
C4orf16	124.6923	128.7229	107.737	77.08311	61.87336	84.39638	64.0407	121.7843	88.19064
FAM46D	76.89124	93.50695	70.06725	55.6282	59.71934	76.16946	50.95465	64.35565	356.3993
HIST1H3I	120.3216	89.76928	77.28229	226.4327	67.32587	90.39395	63.89164	72.39458	145.3624
OR4D2	76.02959	124.2831	90.73653	117.4295	73.81612	97.51339	67.87064	90.28127	348.1395
DEFB131	44.06641	42.20522	43.81877	51.382	48.4294	56.24143	37.09382	44.54798	43.13138
TMEM164	594.5653	412.7718	297.4989	298.3608	266.4936	259.8459	279.2754	143.0364	317.4485
ARHGAP15	54.26184	63.73484	146.0132	128.8941	162.6768	285.0519	265.5919	101.5715	62.04569
RPLP0P2	540.981	347.6665	95.08659	262.1531	76.97794	61.58905	54.49041	53.99291	1069.46
LASS5	34.98045	32.75982	111.7576	45.56149	116.0435	33.092	170.64	173.3485	43.48306
NPIP	698.854	906.7394	787.8723	772.265	654.821	747.7614	469.8444	505.4713	1501.99
OR6C70	128.0016	43.66665	63.49471	41.87827	58.10412	44.81547	53.20774	46.76688	38.45255
CD52	272.8896	191.244	687.0568	444.3464	84.89894	113.5382	313.0035	127.6952	339.9802
KRTAP9-4	53.64452	55.90677	257.4284	98.76919	642.0903	37.03218	285.025	42.88493	48.56562
P15RS	3167.731	4383.004	5736.689	5335.153	6969.859	5688.583	6241.955	6672.194	2715.579
DMRT1	38.32903	48.4016	40.048	41.76048	51.73985	32.57622	39.24053	42.21395	59.4497
KRTAP1-3	43.9885	68.85795	53.52002	101.968	50.99306	68.49222	52.74541	66.43009	97.69926
ATP4A	257.634	172.8288	85.47683	193.4531	306.1549	118.2632	65.82485	78.39819	468.9262
SNORD56	2495.364	3350.739	1944.791	4199.349	723.425	1808.429	1742.258	2025.574	3734.899
CTGLF3	8795.926	10067.09	9900.633	8712.429	9476.908	12278.37	11002.75	8363.405	8395.086
ODF3B	47.20803	47.59348	261.8539	44.73623	66.33408	47.96871	218.0146	48.9522	885.9011
DERL1	1013.535	97.28825	60.79221	82.42444	147.026	77.56723	113.3181	416.7605	167.1134
PLIN5	1001.874	817.191	448.8678	584.0037	293.4136	385.5273	361.2542	335.7202	1051.081
DNAJC25-G	528.4902	254.1727	107.3973	315.9562	42.66202	157.3341	89.88557	59.9482	564.6688
GDF3	88.72295	42.52321	47.30342	1069.694	53.88965	37.16483	50.47594	51.54336	40.76359
NLGN4Y	36.21289	538.1792	34.87493	37.23173	32.39358	46.14848	211.1397	264.0632	41.64613
CCDC61	118.0145	85.39826	186.0109	98.58478	83.44971	284.0389	66.76047	58.31965	131.2319
MRPS34	44.08653	181.9955	129.7597	508.0414	188.4832	177.7831	168.8	191.7982	118.4264
PRH1	821.6906	475.8472	148.5498	345.1234	96.36932	1106.176	125.7396	203.9522	832.2493
B3GAT3	119.2203	52.66857	36.06836	38.99421	135.6049	32.52628	147.4349	238.6444	38.40244
STYXL1	1609.839	96.66785	67.00179	118.3878	316.2489	64.38113	134.8023	101.7964	113.4046
CDH12P	37.92392	338.7221	42.74532	65.34938	52.14642	67.9772	43.82237	40.5353	49.25873
XLKD1	10927.04	6150.915	3082.072	5612.017	5358.717	3119.503	2595.694	3719.972	10028.27
CDC26	8425.685	8817.929	8709.795	8219.594	7603.098	8348.502	7376.819	9708.152	7879.752
GAS2L3	1047.491	1016.966	2083.327	1145.219	1015.02	551.3542	1401.126	1748.779	148.9841
SPRR2E	25388.79	25756.77	29697.17	13709.51	32714.04	29968.69	27270.66	28247.28	21526.08
SPANXB1	929.6537	697.6941	3009.129	678.3415	65.65414	786.1591	2880.199	130.8181	42.20919
TMEM91	8364.528	5577.381	2689.084	5511.728	1716.283	4625.519	1492.934	4298.427	8223.148

TRAPPC2P1	65.56443	33.46043	45.82613	45.85377	41.33157	42.89304	61.32693	149.007	105.9773
TEX101	51.74827	56.16606	2121.791	36.34295	1605.819	1604.789	1234.136	1200.686	61.51058
MAEL	98.29617	82.79897	67.72331	120.93	76.97205	76.76646	59.95116	64.1815	73.28545
TMC8	188.2194	807.9164	85.90397	201.7409	78.41656	104.8119	68.81318	62.68	233.6354
C21orf24	582.9361	365.8595	271.5963	348.1857	210.7869	275.3505	234.8179	347.9764	811.9858
SLC15A2	34.26927	1268.58	557.7115	511.9285	632.5885	686.7012	401.5573	500.9501	2238.93
ADAMTS7	76.79751	34.06627	33.51343	35.00821	35.5097	40.69682	36.90542	40.94867	43.32206
HMGB1L1	195.7643	80.68549	65.10143	86.33283	180.7299	640.6617	178.2907	78.88618	134.8005
SRP14P1	397.8053	582.2487	132.9382	237.6099	327.9005	535.407	240.3394	473.9787	1363.716
TTTY6	245.2559	153.0802	84.25868	195.1638	70.66564	101.6496	63.42015	71.4313	439.4122
WDR46	32.50837	837.6902	3412.45	1682.411	4833.833	2810.056	4441.841	4931.753	33.5746
TMEM170A	137.8686	262.5673	467.9377	357.1129	350.2164	385.1559	440.432	253.2211	591.434
TMEM39B	67.56516	52.70824	84.50007	44.15032	60.06454	51.43311	48.14217	83.0625	46.29446
ICAM3	204.8228	1020.073	1602.574	171.9982	1484.348	850.4151	1142.965	1929.622	3995.917
GGTA1	121.686	97.64019	79.84195	113.4582	73.58533	84.98934	61.02196	64.3209	403.9919
KIF5C	499.0068	172.1254	163.9696	66.99154	160.487	260.3	120.1685	99.4658	50.74414
KIAA1712	75.6803	293.4003	304.7034	80.75526	542.6887	519.9323	354.2063	427.347	292.6887
SPCS2	586.7236	534.5073	226.01	583.6251	168.9108	330.0157	258.2654	191.6862	862.289
TMED10P	80.0049	47.42078	37.00553	57.4797	42.04198	39.84731	109.8137	137.5356	71.40934
OR1S2	101.5104	52.74329	57.82909	51.91496	38.58735	44.81244	37.13138	54.18155	43.37852
PRO0628	65.92462	767.169	60.21957	48.78349	48.92466	48.59983	48.547	60.28759	64.98035
NDST4	54.52449	52.24727	45.46901	36.35754	49.51043	51.15009	46.53463	43.8521	50.59957
RAB30	381.0895	357.0473	184.8919	225.3469	101.772	120.5887	199.3883	139.0643	551.3337
ARHGEF5L	3537.733	4478.743	4184.554	132.7318	2900.219	2824.413	2637.806	2706.74	2213.604
CCDC42	36.0205	47.6624	45.74391	52.05567	37.65369	41.1722	37.52767	41.83232	34.91967
DEFB107A	160.7067	129.9301	64.30772	157.0561	70.25256	77.80178	70.20694	67.55743	297.9294
MXD1	2967.617	2358.794	2522.026	3220.607	3589.284	1798.969	1647.801	1813.897	4026.008
C4orf36	37.64805	49.10027	35.54617	36.1634	111.7372	35.32044	111.1269	165.7965	61.55941
EPHB4	45.75728	39.51184	32.5197	397.2997	85.59674	32.25548	170.8345	35.92568	35.6067
CCDC127	41.39053	56.95329	47.77914	46.91547	113.9723	48.86767	35.90024	201.2502	37.35999
LRRFIP1	444.717	207.9784	198.9	231.5433	103.1232	139.5062	120.4571	145.0328	205.4122
RNF32	63.92698	36.25149	44.4469	45.95513	40.49875	42.54971	33.96222	63.53518	47.05263
SYNJ2	497.1687	80.52173	215.355	77.2069	187.0759	218.1235	130.6763	156.6076	61.04317
LOC286187	77.82798	44.21343	44.71457	62.3553	36.34109	39.92971	41.62956	37.9014	105.9782
SERPINA6	36.04803	32.93495	40.79901	35.85345	32.46022	37.55547	37.15245	35.60213	34.39579
TUBA3D	74.81879	47.98852	99.72137	73.03929	50.18863	38.07384	43.85764	39.32661	76.34686
MAGOH	37.66323	36.51698	182.3215	38.63895	453.1016	36.00742	331.5121	245.5231	33.03764
ANKRD20A	910.1395	229.9623	1263.937	81.67967	828.2842	353.7289	380.8955	726.2667	115.7777
SLFN13	1604.251	2771.417	2082.24	3286.275	582.7274	2258.182	1183.393	435.7437	4120.025
ARHGAP11	191.539	586.9539	226.1391	1670.063	459.9544	464.5221	1608.355	1150.051	242.0692
CYP2B6	59.33333	50.22263	76.82504	55.59932	40.76252	45.5294	86.25919	49.12649	65.5528
NEFM	186.3808	122.7079	79.63889	156.2507	137.2049	133.5489	92.72661	88.47623	382.4861
GRK1	81.33375	66.97202	61.39855	97.31194	55.60435	45.55841	54.77908	51.31858	88.31055
LYL1	9814.632	8869.7	6290.409	6568.764	3421.646	5956.376	4832.536	5792.17	10679.44
STK32B	2447.906	2083.624	1505.07	674.3434	689.4869	1543.516	1647.122	1602.334	2348.997
PGK1	1398.612	1823.61	2373.337	2830.345	1739.345	2243.314	2148.251	1459.789	771.7035
SHPK	15145.82	17089.67	12996.43	13043.29	14420.88	12814.33	13478.93	15301.92	15591.42

BPY2B	97.20895	81.93262	61.24171	95.50027	67.22341	78.35012	60.04961	79.34987	262.2043
ZNF564	35.9062	434.4024	907.0945	523.5332	557.5784	700.8427	656.3717	284.9621	47.28281
PLA2G4D	656.845	833.6544	68.65137	60.72075	2261.707	331.0859	271.1051	751.6483	1127.6
UBE2S	48.63232	44.56653	34.35973	37.68217	40.12512	60.37048	46.10972	47.34617	173.8254
IAH1	864.5299	223.3362	286.8131	283.7176	933.2499	159.21	468.7978	250.0382	339.316
GLB1L	277.5129	165.8466	100.3694	78.92827	261.8018	652.0033	575.1819	643.9792	305.0503
FABP5L3	136.3124	134.2027	107.1099	106.7332	69.32284	74.70757	67.86044	46.67972	80.92045
ANXA9	58.53045	52.00182	38.92062	40.2023	41.64218	45.41311	58.21904	46.5472	48.143
SREBF2	491.5814	316.343	196.5078	365.5862	226.317	130.3222	171.9818	149.0915	674.829
TMEM126A	75.78609	42.81049	44.60753	55.81278	48.27158	44.20465	401.4857	144.9069	179.2762
TTC21B	466.3722	325.9659	128.2968	588.3897	144.1034	138.5173	198.6835	102.6455	779.5389
RAB40A	39.28729	42.18528	57.40772	31.01715	66.62331	36.08222	44.06868	84.98251	43.2172
RPS2	523.1014	375.426	246.1035	447.2988	200.386	244.0308	203.4159	200.2598	672.4867
ZNF563	432.1245	250.4976	125.9197	246.9126	122.0192	186.2197	109.6236	134.8912	557.6101
LSM1	7467.678	7465.285	8373.658	7137.928	8376.475	7710.166	8083.286	7844.544	7079.465
GSTM1L	42.54548	37.90656	36.43998	32.31017	35.50501	30.44956	35.86685	36.58184	51.01973
EFCBP1	54.96983	50.15023	45.32795	45.31101	35.56185	50.03534	35.13271	37.2265	49.21473
PPM2C	396.3596	290.2641	316.7534	321.9449	398.6467	422.787	496.2377	334.0135	255.3642
CCL3L1	38.38775	67.10677	59.42537	68.31681	52.14414	51.91984	46.66706	62.64968	56.33273
GPR37	95.32716	46.00701	508.6649	82.4444	63.41812	73.94961	136.3701	52.65562	74.33102
KRT6A	30973.11	33218.39	34731.96	33338.2	40423.77	30975.79	32700.93	33782.79	27558.54
OR8J3	134.1233	109.5735	67.17625	92.93005	75.99542	99.56042	57.39131	63.6309	146.6469
RPS15	42.75317	91.12563	167.7339	557.4513	50.16463	57.20826	219.4303	152.0648	41.51868
FAM84A	273.1344	204.9978	126.706	199.4744	132.6691	151.8699	82.42891	126.4374	385.2948
LOC162073	64.2832	59.32998	254.6378	40.57671	361.8569	69.00886	201.0649	54.25885	39.14176
APOBEC3B	52.05417	48.53642	51.30381	43.78164	55.60716	59.12237	44.71658	132.9379	40.80225
ISCA1	69.90768	61.08455	135.9107	67.96247	146.0911	77.20134	188.789	150.7289	95.91966
LOC90586	2546.534	3146.719	577.6774	1131.787	586.5485	896.454	693.721	613.4211	1230.069
SSB	1032.608	451.9716	218.9106	805.0565	184.2351	353.0717	195.2037	292.7739	764.9848
RBP2	211.032	118.5497	85.67559	141.1406	81.53995	96.20097	73.98519	95.16747	304.0901
NEU4	1191.268	6675.736	2067.77	3092.383	759.7198	3982.44	3232.529	4117.874	6563.329
GFPT1	2430.239	1973.064	1249.549	1836.729	1914.436	1537.511	851.8433	1409.923	4717.429
HNRPA1L-2	1101.947	1531.026	1212.29	1692.795	755.7914	977.8267	932.6736	1039.067	1359.215
RPL7L1	10498.24	8650.359	5256.549	7930.705	2587.74	4040.151	3179.932	3945.073	10514.08
C6orf72	69.98061	152.7941	65.16199	155.7682	51.78628	114.8859	48.90457	76.10322	302.6599
CLEC3A	58.3264	94.54413	51.13182	71.19714	59.86961	73.45181	56.38764	40.98652	45.8152
TMCO3	129.6398	57.01488	299.6632	58.05868	316.6634	56.41038	108.2297	52.97124	102.8657
C6orf195	56.09115	367.177	58.2374	50.29557	50.45884	115.4742	51.02858	50.36124	73.38094
NR0B2	45.98207	65.61752	43.88629	68.05537	63.91534	55.21542	53.93715	38.08484	96.0485
GM2A	1666.976	53.1877	671.7855	1022.641	1169.041	74.4959	238.8101	1159.618	88.8116
VDAC2	34.6847	140.1934	113.4425	194.3345	86.77686	128.7216	129.387	111.2028	53.37036
C17orf85	1262.187	1721.508	2228.219	1646.212	1840.894	1911.904	2068.97	2716.698	2104.282
GPR103	105.5272	45.44696	70.61021	44.42248	64.99056	35.68753	62.6454	37.58039	30.90766
[truncated: 593,038 more chars]
